# Viral intra-host evolutionary dynamics revealed via serial passage of Japanese encephalitis virus *in vitro*

**DOI:** 10.1093/ve/veac103

**Published:** 2023-03-28

**Authors:** Bangyao Sun, Ming Ni, Haizhou Liu, Di Liu

**Affiliations:** School of Medical Laboratory, Weifang Medical University, Baotong West Street, Weifang 261053, China; CAS Key Laboratory of Special Pathogens and Biosafety, Wuhan Institute of Virology, Chinese Academy of Sciences, Xiaohongshan 44#, Wuhan 430000, China; Computational Virology Group, Center for Bacteria and Viruses Resources and Bioinformation, Wuhan Institute of Virology, Chinese Academy of Sciences, Xiaohongshan 44#, Wuhan 430000, China; University of Chinese Academy of Sciences, Yuquan Road 19#, Beijing 100049, China; Beijing Institute of Radiation Medicine, Taiping Road 27#, Beijing 100850, China; Computational Virology Group, Center for Bacteria and Viruses Resources and Bioinformation, Wuhan Institute of Virology, Chinese Academy of Sciences, Xiaohongshan 44#, Wuhan 430000, China; CAS Key Laboratory of Special Pathogens and Biosafety, Wuhan Institute of Virology, Chinese Academy of Sciences, Xiaohongshan 44#, Wuhan 430000, China; Computational Virology Group, Center for Bacteria and Viruses Resources and Bioinformation, Wuhan Institute of Virology, Chinese Academy of Sciences, Xiaohongshan 44#, Wuhan 430000, China; University of Chinese Academy of Sciences, Yuquan Road 19#, Beijing 100049, China

**Keywords:** viral intra-host evolution, intra-host single nucleotide variation (iSNV), viral quasispecies, evolutionary dynamics

## Abstract

Analyses of viral inter- and intra-host mutations could better guide the prevention and control of infectious diseases. For a long time, studies on viral evolution have focused on viral inter-host variations. Next-generation sequencing has accelerated the investigations of viral intra-host diversity. However, the theoretical basis and dynamic characteristics of viral intra-host mutations remain unknown. Here, using serial passages of the SA14-14-2 vaccine strain of Japanese encephalitis virus (JEV) as the *in vitro* model, the distribution characteristics of 1,788 detected intra-host single-nucleotide variations (iSNVs) and their mutated frequencies from 477 deep-sequenced samples were analyzed. Our results revealed that in adaptive (baby hamster kidney (BHK)) cells, JEV is under a nearly neutral selection pressure, and both non-synonymous and synonymous mutations represent an S-shaped growth trend over time. A higher positive selection pressure was observed in the nonadaptive (C6/36) cells, and logarithmic growth in non-synonymous iSNVs and linear growth in synonymous iSNVs were observed over time. Moreover, the mutation rates of the NS4B protein and the untranslated region (UTR) of the JEV are significantly different between BHK and C6/36 cells, suggesting that viral selection pressure is regulated by different cellular environments. In addition, no significant difference was detected in the distribution of mutated frequencies of iSNVs between BHK and C6/36 cells.

## Introduction

RNA virus populations are highly diversified as a result of rapid replication, high mutation rates, and multiple mutation patterns (mutation, recombination, reassortment, etc.) as previously reviewed (Domingo, Sheldon, and Perales 2012), causing severe epidemics, pandemics, and huge economic losses in recent years. Investigating the characteristics of viral variation and evolution is crucial for interrupting viral transmission routes and establishing interventions for infectious diseases (Gao 2018; Grubaugh et al. 2019). Viral evolutionary processes are affected by a variety of factors such as mutations, genetic drift, and natural selection (Geoghegan and Holmes 2018). Variations in viral genomes between and within hosts reflect viral evolutionary characteristics at the population and individual levels, as revealed by single-nucleotide polymorphisms (SNPs) and intra-host single-nucleotide variations (iSNVs) (Ni et al. 2016; Renzette et al. 2017), respectively.

In previous studies, viral inter-host diversity and transmission dynamics have been revealed by SNP-based phylogenetic and sequence analyses (Geoghegan and Holmes 2018). With advances in next-generation sequencing (NGS), studies on viral intra-host evolution that can reflect the interactions between viruses and hosts have attracted extensive attention (Holmes et al. 2016). During the coronavirus disease 2019 (COVID-19) pandemic, studies of the within-host diversity of severe acute respiratory syndrome coronavirus 2 (SARS-CoV-2) played crucial roles in the discovery of new variants (Lythgoe et al. 2021), the identification of transmission chains (Du et al. 2020), and elucidating viral pathogenesis (Voloch et al. 2021). In Ebola virus (EBOV) outbreaks in West Africa, iSNV-based analyses of viral within-host evolutionary dynamics revealed the transmission chain of EBOV infection and provided potential drug targets (Gire et al. 2014; Park et al. 2015; Ni et al. 2016). Through the passaging of West Nile virus (Grubaugh et al. 2015) and rabies virus (Bonnaud et al. 2019) in animals in combination with analyses of viral intra-host genetic diversity, the knowledge of natural selection affecting RNA virus populations and viral cross-species transmission has been further improved. Moreover, variations in the polymorphisms of many other viruses within hosts have been elucidated, including the influenza virus (McCrone et al. 2018), imported yellow fever virus (YFV) in China (Chen et al. 2018), and Zika virus in the Americas (Metsky et al. 2017), providing new insights for understanding viral evolution and antiviral therapy. Accordingly, bioinformatics methods and genome amplification approaches for viral intra-host variation analyses have also been developed (Yang et al. 2013; McCrone and Lauring 2016; Ni, Chen, and Liu 2018). However, some basic scientific questions still need to be addressed, such as the features of viral intra-host variation over time and the kinetic characteristics of iSNV emergence, fixation, and disappearance, which could enhance our knowledge of viral intra-host evolution (Jenkins et al. 2002).

In this study, we aimed to investigate the features of viral mutations at a single-nucleotide resolution, which requires a model with a simple background. *In vitro* infection of adaptive (BHK) and nonadaptive (C6/36) cells with the SA14-14-2 vaccine strain of Japanese encephalitis virus (JEV) was selected as the experimental model because of the following advantages: (1) viral passage in cells can provide a relatively continuous and stable environment, suitable for mimicking the viral intra-host evolutionary process (Andino and Domingo 2015); (2) there are few reports on JEV recombination (Twiddy and Holmes 2003; Chuang and Chen 2009; Chiang et al. 2014), and the genome of JEV is a single-stranded RNA molecule (the family *Flaviviridae*) (Mukhopadhyay, Kuhn, and Rossmann 2005). Hence, there is no genome reassortment process, which is appropriate for studying the viral single-nucleotide mutation; (3) in previous reports, the SA14-14-2 vaccine strain was obtained via serial passage in BHK cells (Ni et al. 1994, 1995), indicating that it is adaptive in this cell.

Through viral serial passage *in vitro* and deep sequencing of the viral genome, we identified 1,788 iSNVs from 477 samples and further analyzed the distribution characteristics of these iSNVs and their mutated frequencies. Our findings show that a nearly neutral selection pressure is dominant, and an S-shaped growth trend was observed in both non-synonymous and synonymous mutations over time in the adaptive cells. In the nonadaptive cells, a higher positive selection pressure was observed compared with that in the adaptive cells, with logarithmic growth in non-synonymous iSNVs and linear growth in synonymous iSNVs over time. The mutation rates of the NS4B protein and the UTR of the JEV are significantly different between BHK and C6/36 cells, suggesting that viral selection pressure is unique to the cellular environment. In addition, there is no significant difference in the distribution of mutated frequencies of iSNVs between the BHK and C6/36 cells. Our results facilitate the understanding of viral intra-host dynamics and provide a reference for intra-host evolution of other viruses.

## Results

### Experimental design

**Figure 1.**
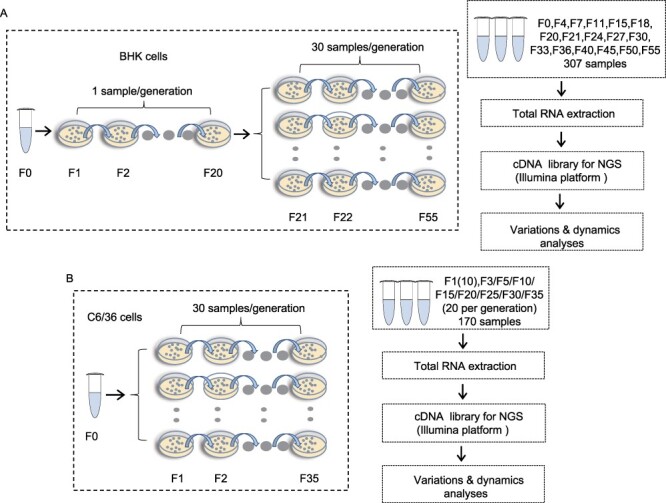
The passage and sequencing strategy of JEV in BHK (A) and C6/36 (B) cells.

We aimed to investigate the characteristics of viral intra-host variation at the population level, which required sufficient variants in the virus population; therefore, accumulating viral mutations by passages is necessary. After two rounds of plaque assays for viral purification, the initial twenty passages of the SA14-14-2 vaccine strain were amplified for viral variation accumulation in one replicate per passage in BHK cells (Fig. 1A). Subsequently, thirty replicates per generation from F21 to F55 were implemented, and 307 samples (every three to five generations) were selected to reveal viral population-genetic characteristics within hosts using high-throughput sequencing combined with viral evolutionary dynamic analyses (Fig. 1A). Next, viral passage in C6/36 cells was performed to compare viral variations in different host environments. A total of thirty-five viral passages were obtained, and 170 samples were selected for sequencing and viral evolutionary dynamic analyses (Fig. 1B).

### iSNVs analysis

This study aimed to investigate the characteristics of viral intra-host variations, and the dynamic process of the occurrence, fixation and disappearance of iSNVs cannot be fully explained if SNPs are ignored. Therefore, both of them were merged as iSNVs together for subsequent analyses after iSNV calling regardless of their difference. A total of 55 iSNVs were identified in the selected six samples from F1 to F20 (Supplementary Tables S1 and S3), among which there were at least twenty-five iSNVs found per sample, except in sample F4 (only three iSNVs, Supplementary Fig. S1A), with a relatively uniform distribution along the viral genome (Supplementary Fig. S1B). Sufficient iSNVs accumulated during the first twenty passages of BHK cells.

**Figure 2.**
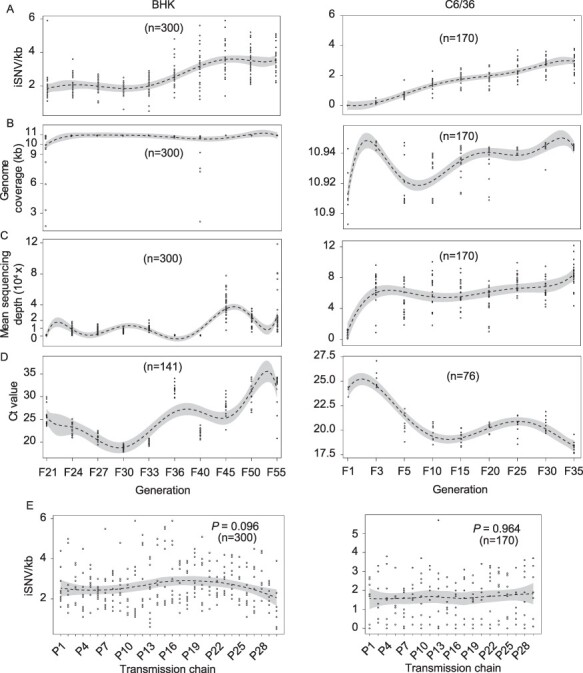
Statistical information of iSNVs in indicated cells. (A–D) The distribution of normalized iSNVs (iSNV/kb, A), genomic coverage (B), genomic mean sequencing depth (C), and cycle threshold (Ct) values of quantitative real-time PCR (D) in BHK (left panel) and C6/36 cells (right panel) in indicated generations by smoothing regression (dashed line) with 95 per cent confidence interval (shaded area). (E) Distribution of normalized iSNVs in thirty transmission chains of BHK cells (left panel) and twenty transmission chains of C6/36 cells (right panel) by smoothing regression (dashed line) with 95 per cent confidence interval (shaded area). Statistical significance was assessed using all pairwise Kruskal–Wallis one-way ANOVA test and *P*-value < 0.01 is considered statistically significant.

In large-population viral passages, 1,369 and 574 iSNVs were detected in the sequenced samples of BHK and C6/36 cells, respectively (including 172 shared iSNVs between BHK and C6/36 cells; Supplementary Tables S1–S4). The number of iSNVs is affected by the genomic coverage, mean sequencing depth, and viral load of the samples, and we tested their association by comparing their respective distribution characteristics over time. Considering that viral genomic coverage varied among our samples, normalized iSNVs (iSNVs/kb, the number of iSNVs/kb) were used instead of the total iSNVs per sample, and the cycle threshold (Ct) values of quantitative real-time polymerase chain reaction (qPCR) were used to measure viral load. Compared to an S-like growth trend and a linear growth trend of iSNVs/kb, however, different characteristics of the genomic coverage, mean sequencing depth, and Ct values of the samples were presented in BHK and C6/36 cells over time (Fig. 2A–D), suggesting that the detected iSNVs were unrelated to NGS data and viral load. In addition, no significant differences in iSNVs/kb were observed among the different transmission chains in BHK and C6/36 cells (Fig. 2E, *P* > 0.01), indicating that there was no cross-contamination or significant infection dose difference between the different transmission chains during viral passages.

### Distribution of iSNVs in BHK cells

**Figure 3.**
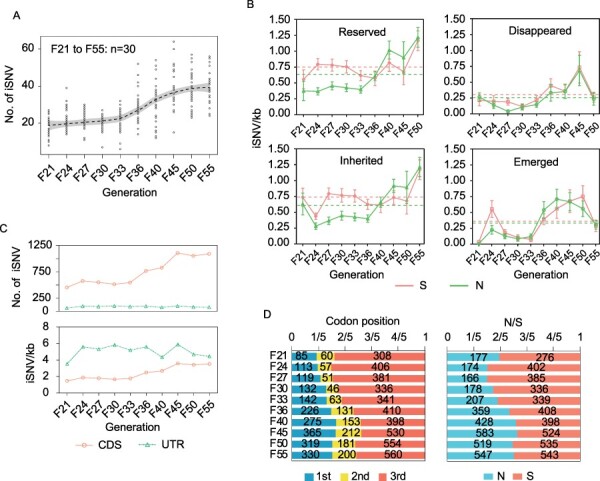
Distribution of iSNVs in BHK cells in indicated generations. (A) Distribution of the total iSNVs by smoothing regression (dashed line) with 95% confidence interval (shaded area). (B) The occurrence and development of total non-synonymous (N) and synonymous (S) iSNVs. The results are presented as the mean ± 95% confidence interval labelled by the error bars. The dashed lines represent the mean in indicated groups. (C) Distribution of total iSNVs (upper panel) and sum of normalized iSNVs (lower panel) of CDS and UTR. (D) Distribution of iSNVs at codon positions (left panel) and non-synonymous (N)/synonymous (S) iSNVs (right panel).


We investigated the distribution characteristics of the iSNVs, including the occurrence and development of iSNVs along the viral genome over time. An S-shaped trend was observed in all iSNV distributions in BHK cells over time (Fig. 3A), which can be roughly estimated as:

(1) $${\rm Y} = {{100} \over {{1 + 10 \ \hat{}\ }^{({{\rm K} - {\rm X}})^{\ast} {\rm HillSlope}}}},$$

where Y is the total number of iSNVs. X is the time of viral passage (96 h per passage), and the other detailed parameters are listed in Table 2. In addition, the number of iSNVs in the UTR did not change significantly, while both non-synonymous and synonymous iSNVs were also in an S-shaped distribution (Supplementary Fig. S2A).

We also used iSNVs/kb to reveal the dynamic process of occurrence and development of non-synonymous and synonymous iSNVs in BHK cells, which contributed to predicting the viral mutation trend. As shown in Equation (4), the reserved iSNVs are determined by the other three. Our results showed that both emerged and disappeared iSNVs exhibited a fluctuation around a constant (Fig. 3B), and they were 0.3354 and 0.2554 in non-synonymous iSNVs and 0.3621 and 0.3036 in synonymous iSNVs, respectively (Table 1). Thus, the reserved iSNVs are up to the inherited ones, which are characterized by an S-shaped trend (Fig. 3B) and can be estimated using Equation (1), with their parameters shown in Table 2.

**Table 1.** The constant parameters of the occurrence and development of iSNVs in indicated cells.

Cells	Objects	*M* ± SD	95 per cent CI
BHK	N-emerged iSNVs	0.3354 ± 0.3536	0.2963–0.3793
	N-disappeared iSNVs	0.2554 ± 0.3396	0.2177–0.2968
	S-emerged iSNVs	0.3621 ± 0.3849	0.3212–0.4078
	S-disappeared iSNVs	0.3036 ± 0.3488	0.2665–0.3487
C6/36	N-emerged iSNVs	0.3861 ± 0.2871	0.3460–0.4275
	N-disappeared iSNVs	0.1490 ± 0.2705	0.1083–0.1910
	S-emerged iSNVs	0.1869 ± 0.2042	0.1567–0.2247
	S-disappeared iSNVs	0.0809 ± 0.1423	0.0600–0.1042

M, mean; SD, standard deviation; CI, confidence interval; N, non-synonymous; and S, synonymous.

**Table 2.** The parameters of Formula 1 of the occurrence and development of iSNVs in BHK cells.

${\mathrm{Y = }}{{{\mathrm{100}}} \over {{\mathrm{1 + 10}}{{\hat{}}^{{\mathrm{(K - X)^{\ast} HillSlope}}}}}}$	*K*		Hillslope	
	*M* ± SE	95 per cent CI	*M* ± SE	95 per cent CI
Total iSNVs	64.06 ± 1.769	60.58–67.54	0.01521 ± 0.001003	0.01323–0.01718
N-reserved iSNVs	143.6 ± 8.121	127.6–159.6	0.02036 ± 0.001616	0.01718–0.02355
N-inherited iSNVs	184.9 ± 16.89	151.7–218.2	0.01508 ± 0.001751	0.01163–0.01852
S-reserved iSNVs	356.1 ± 68.35	221.5–490.6	0.006604 ± 0.001412	0.003823–0.009384
S-inherited iSNVs	428.3 ± 79.84	271.2–585.4	0.005449 ± 0.001117	0.003251–0.007648

*Y*, the number of iSNVs; *X*, the time of passage (96 h per passage); *K*, a constant; HillSlope, the slope of regression curve; *M*, mean; SE, standard error of mean; CI, confidence interval; N, non-synonymous; and S, synonymous.

We then examined iSNV distribution along the viral genome. The results showed that many more iSNVs were observed in the coding sequence (CDS) than in the UTR (Fig. 3C, upper panel), but after normalization by sequence length, the iSNVs/kb in the UTR was more enriched than that in the CDS (Fig. 3C, lower panel). At the same time, iSNVs/kb in the UTR showed an obvious fluctuation, while iSNVs/kb in the CDS increased with an S-shape over time (Fig. 3C, lower panel), which indicates that the viral UTR is under neutral selection. iSNVs at codon positions and the ratio of non-synonymous to synonymous iSNVs (N/S) reflect the selection pressure within the viral population (Ni et al. 2016). In general, iSNVs have a significant preference for the third codon (Fig. 3D, left panel), and the ratio of non-synonymous/synonymous mutations fluctuates from around 0.5 to around 1 over time (Fig. 3D, right panel), suggesting that viral populations are under nearly neutral selection in BHK cells, which is further proven by the fact that the disappeared/reserved ratio of the emerged iSNVs fluctuates around 1 (Supplementary Fig. S3A). We also found that iSNVs at codon positions and the ratio of non-synonymous to synonymous variants varied among viral open reading frames (ORFs) in BHK cells over time (Supplementary Fig. S4A).

### Mutated allele frequency of iSNVs in BHK cells

**Figure 4.**
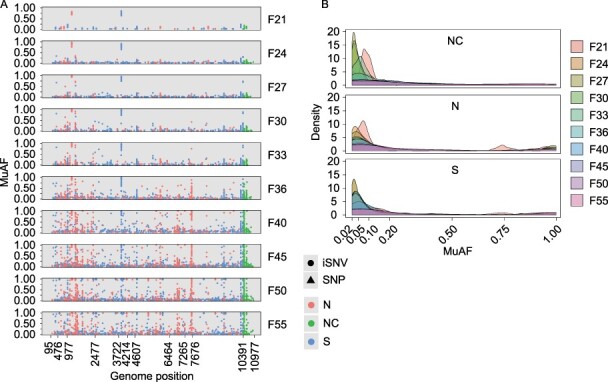
The distribution of MuAFs of iSNVs in BHK cells. (A) The distribution of MuAFs of iSNVs along JEV genome in indicated generations. The figures of X-axis indicate the starting position of each ORF in the genome (N, non-synonymous; S, synonymous; and NC, non-coding). (B) The density distribution of the MuAFs of NC, N, and S iSNVs in indicated generations.

Mutated allele frequency (MuAF) is also an indicator of the selection pressure of the viral population (Li et al. 2010; Acevedo, Brodsky, and Andino 2014). We further examined the distribution of all iSNV MuAFs in the BHK cells. The iSNV MuAFs and their positions along the viral genome are shown in Fig. 4A; iSNVs in the UTR are more enriched than those in the CDS, and the density diagram showed that the proportion of low-frequency non-coding, non-synonymous, and synonymous iSNVs (MuAF < 0.1) gradually declined, while the proportion of high-frequency non-synonymous and synonymous iSNVs increased over time (Fig. 4B). Overall, MuAFs of all iSNVs presented an L-shaped distribution in which the proportion of iSNVs at low frequency was larger than that of high-frequency iSNVs (Fig. 4B), implying that low-frequency iSNVs are likely to be under purifying selection (Chen et al. 2018). We further analyzed the MuAF distributions of the occurrence and development of iSNVs over time, and the violin illustrations showed that larger MuAF distributions and a larger proportion of high-frequency alleles were more represented in inherited and reserved iSNVs than in emerged and disappeared iSNVs (Supplementary Fig. S5A).

### Distribution of iSNVs in C6/36 cells

**Figure 5.**
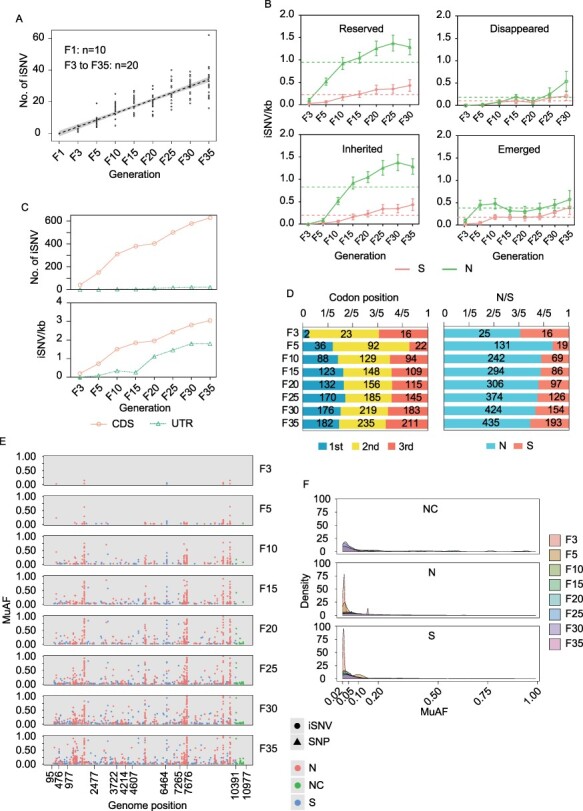
The distribution of iSNVs and their MuAFs in C6/36 cells. (A–D) The d of iSNVs in C6/36 cells in indicated generations and detailed description information is the same as in BHK cells in Fig. 3. (E and F) The distribution of MuAFs of iSNVs in C6/36 cells and detailed description information is the same as in BHK cells in Fig. 4.

Considering that the SA14-14-2 vaccine strain of the JEV has been adaptive in the BHK cells (Ni et al. 1994, 1995), C6/36 cells, the nonadaptive cells of the SA14-14-2 vaccine strain, were used to illustrate and compare the viral variations in different cellular environments by means of serial passage *in vitro* (Fig. 1B), with the NGS data and iSNV information of each sample shown in Supplementary Tables S2 and S4. Compared with the S-shaped distribution of iSNVs in BHK cells, the number of iSNVs in C6/36 cells showed a linear growth over time (Fig. 5A), which can be roughly estimated as:


(2) $${\mathrm{Y}}= {\rm Slope}^{\ast}{\mathrm{X}} + {\mathrm{Yintercept}},$$

using the parameters listed in Table 3. The total number of iSNVs in C6/36 cells increased by 0.9135 per generation (95 per cent confidence interval, 0.8340–0.9930; Table 3). Considering that the viral replication time was 96 h (4 days) per generation, iSNVs accelerated at a rate of 0.2283 variations/day in our study. All samples from Passage F1 were excluded from subsequent analyses because no iSNV was detected in them. In addition, non-synonymous and synonymous iSNVs also increased linearly over time, but the non-coding iSNVs did not change significantly (Supplementary Fig. S2B).

**Table 3.** The parameters of Formula 2 of the occurrence and development of iSNVs in C6/36 cells.

	Slope		*Y* intercept	
*Y* = Slope*X + *Y* intercept	*M* ± SD	95 per cent CI	*M* ± SD	95 per cent CI
Total iSNVs	0.9135 ± 0.04057	0.8340–0.9930	2.643 ± 0.8244	1.027–4.258
S-inherited iSNVs	0.01417 ± 0.001261	0.01170–0.01664	−0.05310 ± 0.02302	−0.09822 to −0.007982
S-reserved iSNVs	0.01488 ± 0.001586	0.01177–0.01799	−5.667e−005 ± 0.02895	−0.05680 to 0.05668

*Y*, the number of iSNVs; *X*, the time of passage (96 h per passage); *K*, a constant; HillSlope, the slope of regression curve; *M*, mean; SE, standard error of mean; CI, confidence interval; N, non-synonymous; and S, synonymous.

Subsequently, we investigated the occurrence and developmental features of viral non-synonymous and synonymous iSNVs. Compared with the reserved and inherited iSNVs in C6/36 cells, both emerged and disappeared iSNVs also exhibited a fluctuation around a constant (Fig. 5B), with values of 0.3861 and 0.1490 in non-synonymous iSNVs and 0.1869 and 0.0809 in synonymous iSNVs, respectively (Table 1). Similarly, the reserved iSNVs are up to the inherited ones. However, the non-synonymous iSNVs were characterized by logarithmic growth over time (Fig. 5B), and the curve fitting formula was as follows:


(3) $$ {\rm Y} = {\rm Slope}^{\ast} {\rm log} {\rm (X)} + {\rm Y} {\mathrm{intercept}},$$

using the parameters listed in Table 4. The number of synonymous iSNVs increased linearly over time (Fig. 5B), and the rates of reserved and inherited iSNVs were 0.01488 and 0.01417 per generation, respectively (Table 3).

**Table 4.** The parameters of Formula 3 of the occurrence and development of iSNVs in C6/36 cells.

	Slope		Y intercept	
*Y* = Slope*log(*X*) + *Y* intercept	*M* ± SD	95 per cent CI	*M* ± SD	95 per cent CI
N-inherited iSNVs	1.477 ± 0.06755	1.343–1.610	−0.8474 ± 0.07698	−0.9996 to −0.6951
N-reserved iSNVs	1.222 ± 0.07562	1.072–1.371	−0.3815 ± 0.08616	−0.5519 to −0.2110

*Y*, the number of iSNVs; *X*, the time of passage (96 h per passage); *K*, a constant; HillSlope, the slope of regression curve. *M*, mean; SE, standard error of mean; CI, confidence interval; N, non-synonymous; and S, synonymous.

Different distribution features of viral iSNVs along the JEV genome were observed in C6/36 cells compared to those in BHK cells. First, the amount of iSNVs in the UTR was less than that in the CDS before and after normalization (Fig. 5C), indicating that iSNVs are more concentrated in the CDS. In addition, iSNVs in the UTR accumulated linearly over time (Fig. 5C, lower panel). Furthermore, iSNVs were distributed equally at each codon position (Fig. 5D, left panel), and non-synonymous mutations were much more common than synonymous mutations over time (Fig. 5D, right panel), suggesting that the virus is subjected to higher positive selection pressure in C6/36 cells than in BHK cells, which is further supported by the fact that most of the emerged iSNVs in C6/36 cells are reserved but does not disappear in the next passage (Supplementary Fig. S3B). Of note, among viral ORFs, varied iSNV distribution features at the codon positions and the ratios of non-synonymous/synonymous over time were observed (Supplementary Fig. S6), which were similar to those in BHK cells.

MuAF distributions of all iSNV sites in C6/36 cells were also examined, and our findings showed that iSNVs in NS4B (genomic positions 7265–7676) were the most enriched region (Fig. 5E). The density diagram showed that non-coding, non-synonymous, and synonymous iSNV MuAFs also featured an L-shaped distribution (Fig. 5F), which is coincident with that in BHK cells, suggesting that low-frequency iSNVs in C6/36 cells are also likely under purifying selection. We also analyzed the MuAFs of the occurrence and development of iSNVs in C6/36 cells, and increasingly larger MuAF distributions with a larger proportion of iSNVs at high frequency were observed in inherited and reserved iSNVs compared with those in emerged and disappeared iSNVs (Supplementary Fig. S5B), which were similar to those in BHK cells.

### iSNVs within viral proteins

**Figure 6.**
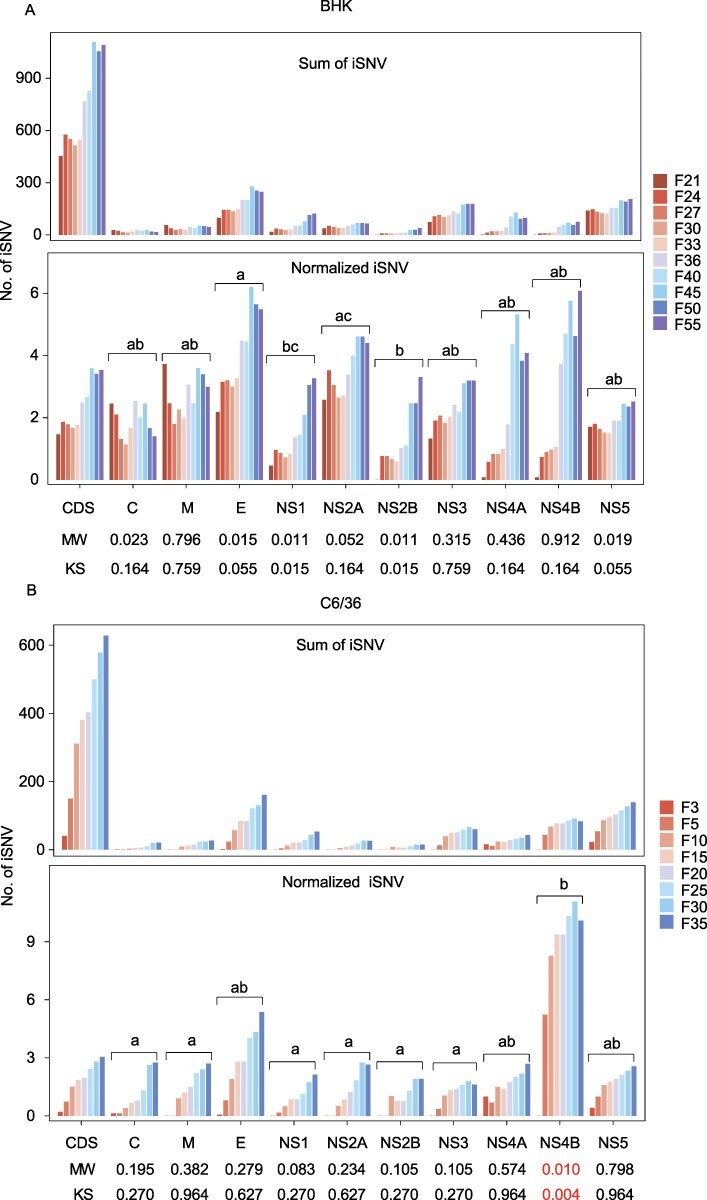
The distribution of total iSNVs (upper panel) and sum of normalized iSNVs (lower panel) in each ORF of JEV in BHK (A) and C6/36 cells (B) in indicated generations. The observed normalized iSNVs for each ORF were compared with that of CDS using Mann–Whitney U test (MW) and Kolmogorov–Smirnov test (KS) with *P* < 0.01. Statistical significance within ORFs was assessed using all pairwise Kruskal–Wallis one-way ANOVA test with *P *< 0.01. The values marked without the same superscript differ significantly.

Identifying viral mutations associated with viral replication, transcriptional regulation, and immune escape is the molecular basis of antiviral drug development (Hildebrandt et al. 2014). We investigated the distribution of iSNVs within ORFs in different cells. Our results showed that iSNVs in all ORFs of JEV accumulate over time before and after normalization (Fig. 6A and B). We compared the normalized iSNVs within each ORF to those in the CDS. No significant difference between ORFs and CDS was found in BHK cells (*P *> 0.01, Fig. 6A, lower panel), but NS4B showed a significant difference compared with CDS in C6/36 cells (*P* < 0.01, Fig. 6B, lower panel). We then examined the differences between the ORFs of both cell lines. In BHK cells, there were significant differences between E and NS1, NS2B, NS2A and NS2B (*P *< 0.01, Fig. 6A, lower panel). In C6/36 cells, significant differences were identified between NS4B and C, M, NS1, NS2A, NS2B, and NS3 *(P* < 0.01, Fig. 6B, lower panel). To sum up, protein E shows the fastest variation rate in BHK cells, while in C6/36 cells, protein NS4B shows the fastest rate. Furthermore, protein NS4B represents the fastest variation rate in both cell lines during viral passages (Fig. 6).

## Discussion

The analysis of viral intra-host variations is of great significance for the identification of the transmission chain in the early stage of an epidemic, the intervention for the epidemics, and antiviral therapy. Illustrating the dynamics of viral intra-host evolution contributes to the understanding of the biological characteristics of viruses (Domingo, Sheldon, and Perales 2012); however, little is known about the viral characteristics of intra-host evolutionary dynamics. In the present study, we preliminarily explored viral evolutionary dynamics within hosts over time using the SA14-14-2 vaccine strain of JEV to infect different cells as models *in vitro*. Our findings showed that the variation characteristics involved in the accumulation of iSNVs, the occurrence and development features of iSNVs, viral selection pressure, and viral genome variations vary between BHK and C6/36 cells (Figs 3, 5, and 6). However, the L-shaped distribution characteristic of iSNV frequencies was found in both cell lines, which has been widely reported in other viruses (Renzette et al. 2017). This may imply that the adaptation of viruses to the environment is determined by viral population diversity as well as immune escape (Andino and Domingo 2015; Parameswaran et al. 2017) instead of MuAFs. In previous reports, viral variation frequencies were regulated by mutation and selection (Acevedo, Brodsky, and Andino 2014), and the influence of iSNV frequencies observed in this study requires further investigation.

Viral evolution is shaped by both stochastic processes (genetic drift, etc.) and determinate factors (selective pressure) (Kouyos, Althaus, and Bonhoeffer 2006; McCrone et al. 2018). Studies have shown that the evolution of the influenza virus within hosts is dominated by a stochastic process (neutral selection) (McCrone et al. 2018), while it is subjected to positive selection at the global scale (Rambaut et al. 2008). Positive selective pressure in viral evolution at the cellular level has also been reported (Illingworth, Fischer, and Mustonen 2014). In this study, JEV evolution was under nearly neutral selection in BHK cells, whereas higher positive selection was observed in C6/36 cells than in BHK cells. Considering that the vaccine strain SA14-14-2 used in this study was adaptive in BHK cells but not in C6/36 cells, the above findings are in line with expectations.

In this study, viral evolutionary strategies varied in different host environments. An S-shaped growth trend in non-synonymous and synonymous iSNVs over time was observed in BHK cells, suggesting likely viral evolutionary processes of random mutations combined with slow variation accumulations under nearly neutral selection. The constant value of emerged non-synonymous iSNVs was slightly lower than that of synonymous iSNVs (0.3354 vs. 0.3621, Table 1), indicating that non-synonymous iSNVs are under purifying selection pressure in BHK cells. The above results suggest that random mutation and purifying selection together shape viral population diversity in BHK cells. Active adaptation to the environment through rapid replication with a high mutation rate is the basis for viral survival (Geoghegan and Holmes 2018). In C6/36 cells, logarithmic growth of non-synonymous iSNVs and linear growth of synonymous iSNVs over time were observed, suggesting that the JEV strain is adapted to C6/36 cells by means of rapid replication and variation accumulation, which can enhance viral fitness (Parameswaran et al. 2017; Li et al. 2022). The main function of the nonstructural protein NS4B, which mutates at the fastest rate in C6/36 cells, is to participate in viral replication and host immune response (Kaufusi et al. 2020). Brackney et al. revealed that the C6/36 cells used in this study lack RNA interference function (Brackney et al. 2010), which is the primary defense of mosquito cells against viral infections. Therefore, the role of NS4B protein in anti-host immunity and promotion of viral replication in this study needs to be further investigated.

Previous studies have shown that the highly conserved secondary structure of the viral UTR participates in viral replication and translation (Fernández-Sanlés et al. 2017). In this study, it was found that the number of iSNVs in the UTR fluctuates in BHK cells over time, and the distribution of iSNVs in the UTR is more concentrated than that in the CDS, suggesting that: (1) the UTR may be involved in the replication and translation of the JEV in BHK cells (Fernández-Sanlés et al. 2017; Ochsenreiter, Hofacker, and Wolfinger 2019); (2) the viral UTR may be under neutral selection, which was also reported in EBOV (Ni et al. 2016) and YFV (Chen et al. 2018). However, a linear growth of iSNVs in the UTR was observed in C6/36 cells, suggesting that natural selection may act on the viral UTR through RNA secondary structures (Scroggs et al. 2019), and variations in the UTR under positive selection pressure were also found in dengue virus (De Borba et al. (2019)). Both results remind us that the selective pressure of the viral UTR is not always neutral but depends on the specific environment. Other studies have shown that mutations in the UTR can also affect viral population structure (Scroggs et al. 2019). Determining whether the viral UTR secondary structure has changed and the effect of viral variations in the UTR on viral population diversity in C6/36 cells requires further investigation.

Viral detrimental variants usually disappear quickly as a result of purifying selection. Our findings showed that 781 of 1,369 iSNVs (57.0 per cent) in BHK cells and 310 of 574 iSNVs (54.0 per cent) in C6/36 cells occurred only once or twice (Supplementary Fig. S7, Supplementary Tables S3, and S4). We also found several mutation sites that were fixed in the viral population, such as C1218T within the *E* gene and C3869T within the *NS2A* gene in BHK cells, and A1911G within the *E* gene and C9688T within the *NS5* gene in C6/36 cells (Figs 4A, 5E, and S7). Except for C3869T, the other three were non-synonymous substitutions. C1218T and C3869T were detected first in sample F18 and were then observed in 278 and 276 of 300 samples, respectively, with an increasingly wider MuAF distribution (Supplementary Fig. S7A and Table S3). A1911G and C9688T were detected first in two and twenty of twenty F3 samples and were subsequently observed in 138 and 127 of 140 samples, respectively (Supplementary Fig. S7B and Table S4). Viral-linked variants have been reported in EBOV with T-to-C substitutions at positions 3008 and 3011 (Ni et al. 2016). Whether these variants (C1218T and C3869T in BHK cells and A1911G and C9688T in C6/36 cells) were linked or accidental could not be identified in this study because of the limitation of the sequencing read length. To date, these four variations have not been found in nature, and determining whether they are adaptive or functional in the viral population requires further investigation.

In this study, we also inferred a few viral population-genetic parameters, which provide important insight into viral intra-host evolution. The reserved iSNVs were determined by the inherited ones, and the numbers of emerged and disappeared iSNVs fluctuated around constant values in both cell lines, indicating that the occurrence and disappearance of viral mutations are regulated by relatively stable factors. The iSNVs that emerged in this study were mainly generated during the viral replication process, which is manipulated by RNA-dependent RNA polymerase (RdRp) and is also affected by purifying selection and viral transmission bottlenecks (Andino and Domingo 2015; McCrone and Lauring 2018). RdRp is considered to be the most conserved protein among flaviviruses during viral evolution (Upadhyay et al. 2017; Collins et al. 2018), although nonstructural genes also contribute to viral genetic diversity in YFV (Collins et al. 2018). The iSNVs that disappeared were mainly affected by the host environment (Holmes et al. 2016), which was stable in our study (Fig. 2E). The difference in the constant values of emerged and disappeared iSNVs between BHK and C6/36 cells may be caused by the different host environments. In our study, the acceleration rate of iSNVs in C6/36 cells was 0.2283 variations/day, and in YFV, it was 0.72 variations/day (Chen et al. 2018). The differences may be caused by different viruses with varied biological characteristics, calculation methods of viral variation rate, and sample sources (cell samples and clinical patient samples, respectively) (Holmes 2013).

In conclusion, in this study, we revealed viral intra-host evolutionary features and the occurrence and development features of iSNVs in different host environments, which improves the understanding of viral evolution and provides a reference for viral intra-host evolution research of other viruses.

## Materials and methods

### Cell culture

The hamster kidney cell line (BHK, ATCC PA-4506) and *Aedes* mosquito cell line (C6/36, ATCC CRL-1660) were used for viral proliferation. BHK cells were cultured in Dulbecco’s modified Eagle’s medium (Sigma) supplemented with 10 per cent fetal bovine serum (FBS, Gibco), 100 units/ml penicillin, l00 μg/ml streptomycin, and 2 mM l-glutamine at 37°C in a 5 per cent CO_2_ incubator. C6/36 cells were maintained in Roswell Park Memorial Institute 1640 medium (RPMI, Hyclone) supplemented with 10 per cent FBS, 100 units/ml penicillin, l00 μg/ml streptomycin, and 2 mM l-glutamine at 28°C in a 5 per cent CO_2_ incubator.

### Viral passage

The SA14-14-2 vaccine strain of JEV used in this study (GenBank accession No. JN604986.1) was kindly donated by Professor Shengbo Cao and Professor Bin Wei. Viral passage was initially implemented in a viral monoclonal manner without interference from other JEV strains; thus, there was no possibility of genome recombination. The parent stocks (F0) were obtained by two rounds of plaque assays (Hong et al. 2015), followed by propagation in BHK cells for 96 h. Confluent monolayer cells in a six-well plate were exposed to 1 ml FBS-free medium fixed with 10 μl viral supernatant (F0) at 37°C for 1 h. Subsequently, the medium was changed, and cells were covered with 4 per cent FBS medium for 96 h for viral replication. After that, the cells (or cellular debris) and viruses were thoroughly mixed, and 10 μl mixtures were used to infect a new six-well plate to complete one passage (Hildebrandt et al. 2014), with viral stocks stored at −80°C. Fifty-five passages (from F1 to F55) were completed in BHK cells, including one replicate per generation from F1 to F20, and thirty parallel replicates per generation from F21 to F55. Thirty-five passages (from F1 to F35) were completed in C6/36 cells, with thirty parallel replicates per generation.

### RNA extraction and NGS

Cellular supernatant (200 μl per sample) was used for RNA extraction using a PureLink™ Viral RNA/DNA Mini Kit (Thermo Fisher Scientific) according to the manufacturer’s instructions. The extracted RNA was subsequently used for NGS library construction using the KAPA RNA HyperPrep Kit (Roche) according to the manufacturer’s instructions. Paired-end reads (2 × 150 bp) were generated using the Illumina HiSeq × Ten platform. The NGS data of the samples are provided in Supplementary Tables S1 (BHK cells) and S2 (C6/36 cells).

### qPCR assays

A total of 50 ng RNA was used to detect the viral load assayed by the Ct values of qPCR with a Luna^®^ Universal One-Step RT-qPCR Kit (New England Biolabs) according to the manufacturer’s instructions. The thermal cycling for qPCR assays was as follows: 10 min at 55°C; 1 min at 95°C; forty cycles (10 s at 95°C and 40 s at 60 °C). The primer sequences were as follows: forward primer, 5ʹ-ATGACTAAAAAACCAGGAGGG-3ʹ and reverse primer, 5ʹ-CTTAGGACATTCGTACGTGATAGTG-3ʹ.

### Calling of iSNVs

The F0 consensus sequence of the JEV was obtained by the *de novo* assembly of NGS data, followed by correction via alignment with the sequence of JEV SA14-14-2 in GenBank (accession No. JN604986.1). The corrected consensus sequence of F0 was the reference for pair-ended read alignment. Quality control and error correction of NGS data and the bioinformatics script of iSNV calling have been described in our previous publications (Ni et al. 2016; Chen et al. 2018), with their accuracy and sensitivity evaluated (Ni, Chen, and Liu 2018). Briefly, the first 10 bp of all reads was removed, followed by the exclusion of the low-quality bases (<Q20) and indels to reduce potential false positive, which requires a minimum read length of 100 bp using Sickle v.1.33 (Joshi and Fass 2011). After error correction using BayesHammer (implemented in SPAdes v3.5.0) (Nikolenko, Korobeynikov, and Alekseyev 2013), clean reads were aligned to the reference using Bowtie2 v2.2.5 (Langmead and Salzberg 2012) with default parameters. Next, ‘mpileup’ files were generated using SAMtools v1.2 (Li et al. 2009) and were subsequently used for iSNV calling via homemade bioinformatic pipelines (http://github.com/generality/iSNV-calling/). The iSNV calling criteria are as follows: (1) samples with more than 1,000 sites, with a sequencing depth ≥80× or a minor allele depth ≥5 were selected; (2) the minor allele frequency cutoff of ≥2 per cent were used to call iSNVs (those variants with the minor allele frequency cutoff of <2 per cent and MuAF of >98 per cent were considered SNPs); (3) depth of the minor allele of ≥5; (4) strand bias of the minor allele less than tenfold. Both iSNVs and SNPs were merged as iSNVs for subsequent analyses after iSNV calling. The iSNV information is provided in Supplementary Tables S1 and S3 (BHK cells) and Supplementary Table S2 and S4 (C6/36 cells).

### Analyses of the occurrence and development of viral iSNVs

In this study, viral iSNVs were divided into the emerged (*de novo* mutation) and inherited (inherited from the previous passage) according to how they occurred and the reserved and disappeared according to how they developed in the next passage. The relationships between the numbers (*N*) of the four aforementioned iSNVs are as follows:


(4) $${{\rm N}_{{\mathrm{Reserved}}}} = {{\rm N}_{{\mathrm{Emerged}}}} + {{\rm N}_{{\mathrm{Inherited}}}} - {{\rm N}_{{\mathrm{Disappeared}}}}$$

Taking the No. 1 transmission chain in BHK cells as an example, the process for determining the occurrence and development of iSNVs is as follows:

(1) First, the iSNVs in the incomplete transmission chain were discarded. For example, if the same variation site was detected from F21 to F36, except for F30, it would be regarded as an incomplete transmission chain (because it is not certain whether this variation disappears in F30 or does not meet the criteria of iSNV calling). All of these iSNVs are provided in Supplementary Tables S5 (BHK cells) and S6 (C6/36 cells).

(2) The development of iSNVs in the last passage was unknown, so they were discarded.

(3) iSNVs in thirty samples of F21 were mixed together to be compared with those of F20 to analyze their occurrence.

## Supplementary Material

veac103_Supp

## Data Availability

The raw sequence data reported in this article have been deposited in the Genome Sequence Archive in National Genomics Data Center, Beijing Institute of Genomics (China National Center for Bioinformation), Chinese Academy of Sciences, under accession No. CRA003716 that is publicly accessible at https://bigd.big.ac.cn/gsa.
