# Correction to: Viral intra-host evolutionary dynamics revealed via serial passage of Japanese encephalitis virus *in vitro*

**DOI:** 10.1093/ve/vead071

**Published:** 2023-11-30

This is a correction to: Bangyao Sun, Ming Ni, Haizhou Liu, Di Liu, Viral intra-host evolutionary dynamics revealed via serial passage of Japanese encephalitis virus *in vitro*, Virus Evolution, Volume 9, Issue 1, 2023, veac103, https://doi.org/10.1093/ve/veac103

In the originally published version of this manuscript, the authors’ affiliation information was incorrect.

The Publisher apologizes for these errors, which have now been corrected.

