# Supplementary material for: Viral intra-host evolutionary dynamics revealed via serial passage of Japanese encephalitis virus in vitro
Source: Virus Evol. 2023 Mar 28;9(1):veac103. doi: 10.1093/ve/veac103 (PMC10185921; doi:10.1093/ve/veac103)
Supplement: veac103_Supp [file veac103_supp.zip › suppl_data/Supplementary_Materials.pdf]

**Supporting information**

Fig S1: Statistical information of iSNVs of F1-F20 in BHK cells. (A) Total iSNVs in indicated generations. (B) Distribution of iSNVs along viral genome in indicated generations.

Fig S2: Distribution of non-coding (NC), non-synonymous (N), and synonymous (S) iSNVs in BHK (A) and C6/36 cells (B) in indicated generations.

Fig S3: Distribution of the disappeared/reserved ratio of the emerged iSNVs in BHK (A) and C6/36 cells (B) over time.

Fig S4: Distribution of iSNVs at codon positions (A) and non-synonymous (N)/synonymous (S) iSNVs (B) in each ORF of indicated generations in BHK cells.

Fig S5: Violin illustration of mutated allele frequencies of occurrence (upper panel) and development (lower panel) of non-synonymous (N) and synonymous (S) iSNVs in BHK (A) and C6/36 cells (B) in marked generations.

Fig S6: Distribution of iSNVs at codon positions (A) and non-synonymous (N)/synonymous (S) iSNVs (B) in each ORF of indicated generations in C6/36 cells.

Fig S7: Distribution of the fixed iSNVs and their mutated allele frequencies (MuAFs) in BHK (A) and C6/36 cells (B) over time. The X axis represents the times of iSNV occurrence, and the Y axis represents the number of corresponding iSNV. Boxes represent the interquartile range (IQR) between 10% and 90% quartiles.

Table S1: Sequencing data in BHK cells

Table S2: Sequencing data in C6/36 cells

Table S3: iSNV information in BHK cells

Table S4: iSNV information in C6/36 cells

Table S5: Discontinuous iSNV sites in the same transmission chain of BHK cells

Table S6: Discontinuous iSNV sites in the same transmission chain of C6/36 cells

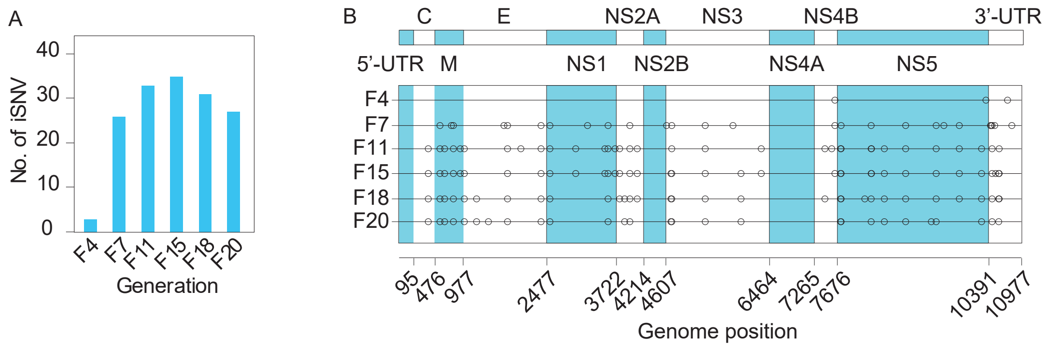


Supplementary Fig1

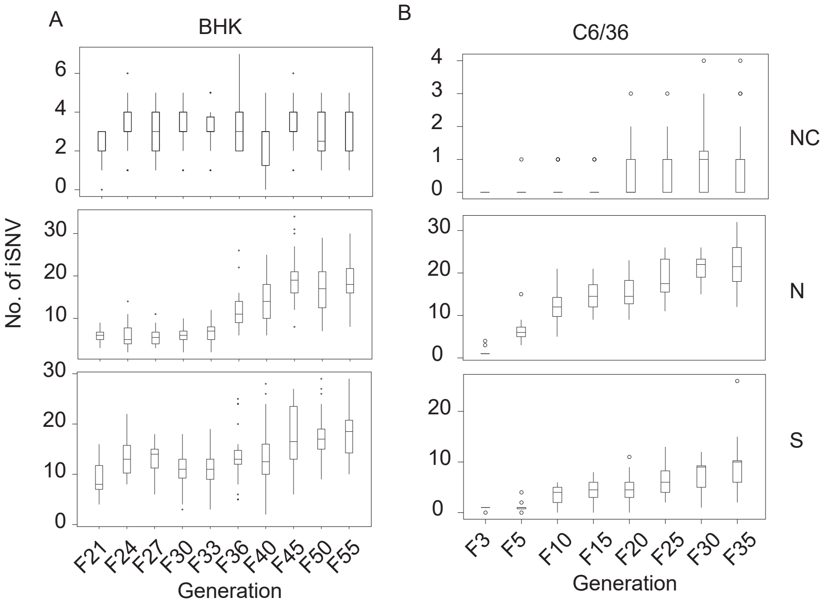


Supplementary Fig2

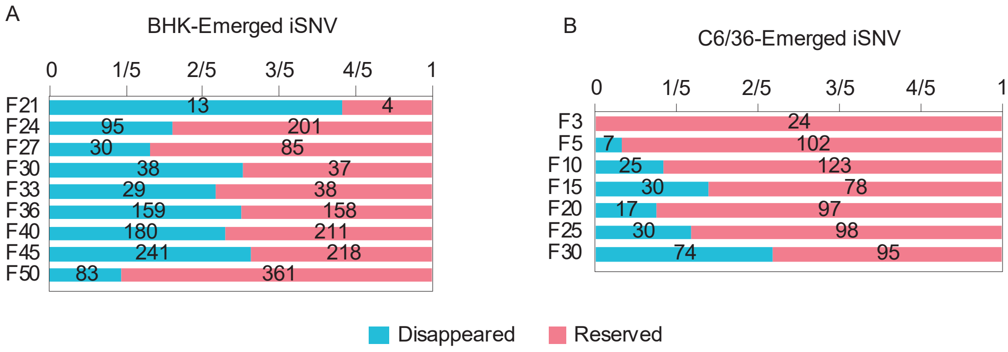


Supplementary Fig3

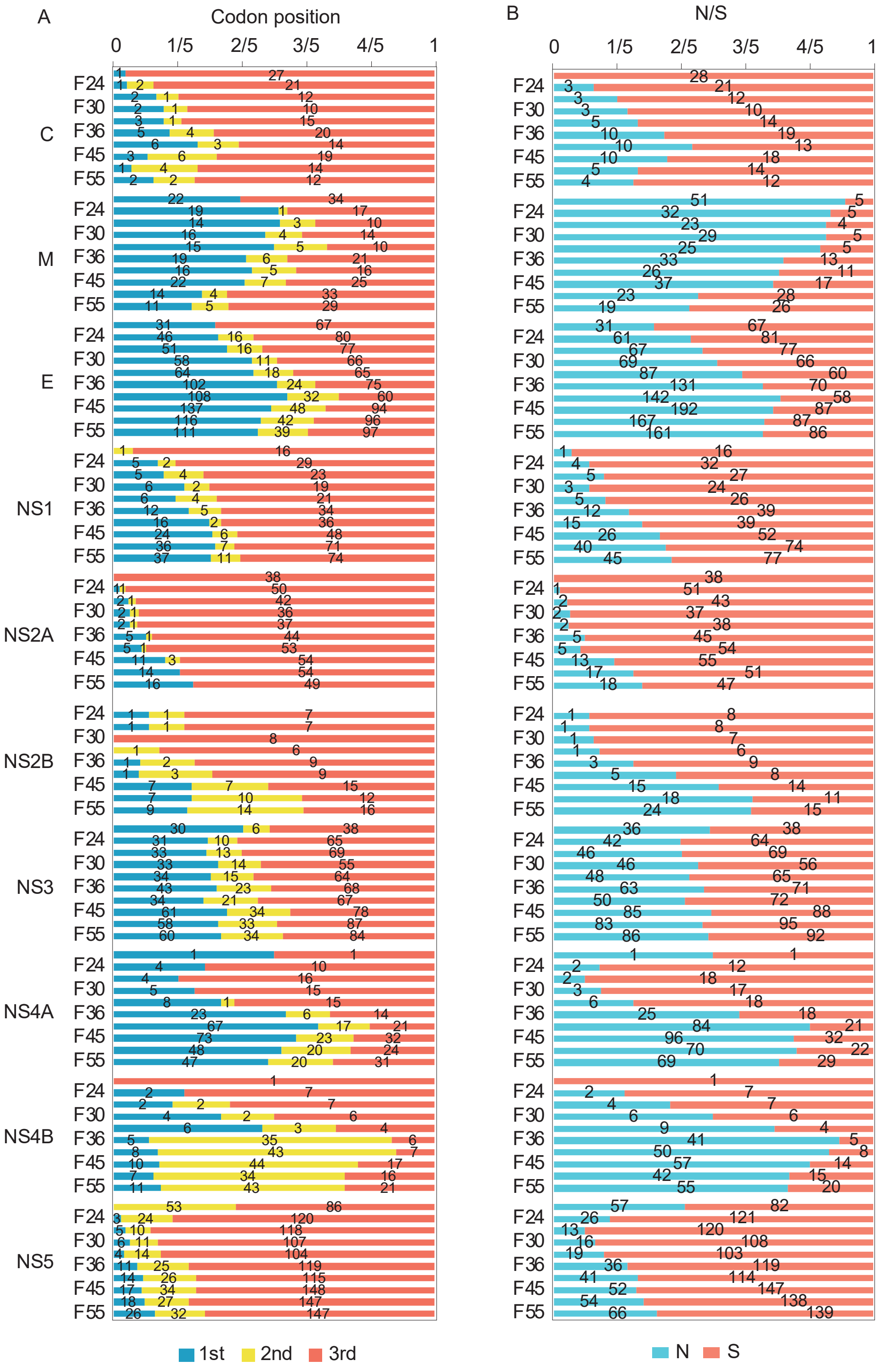


Supplementary Fig4

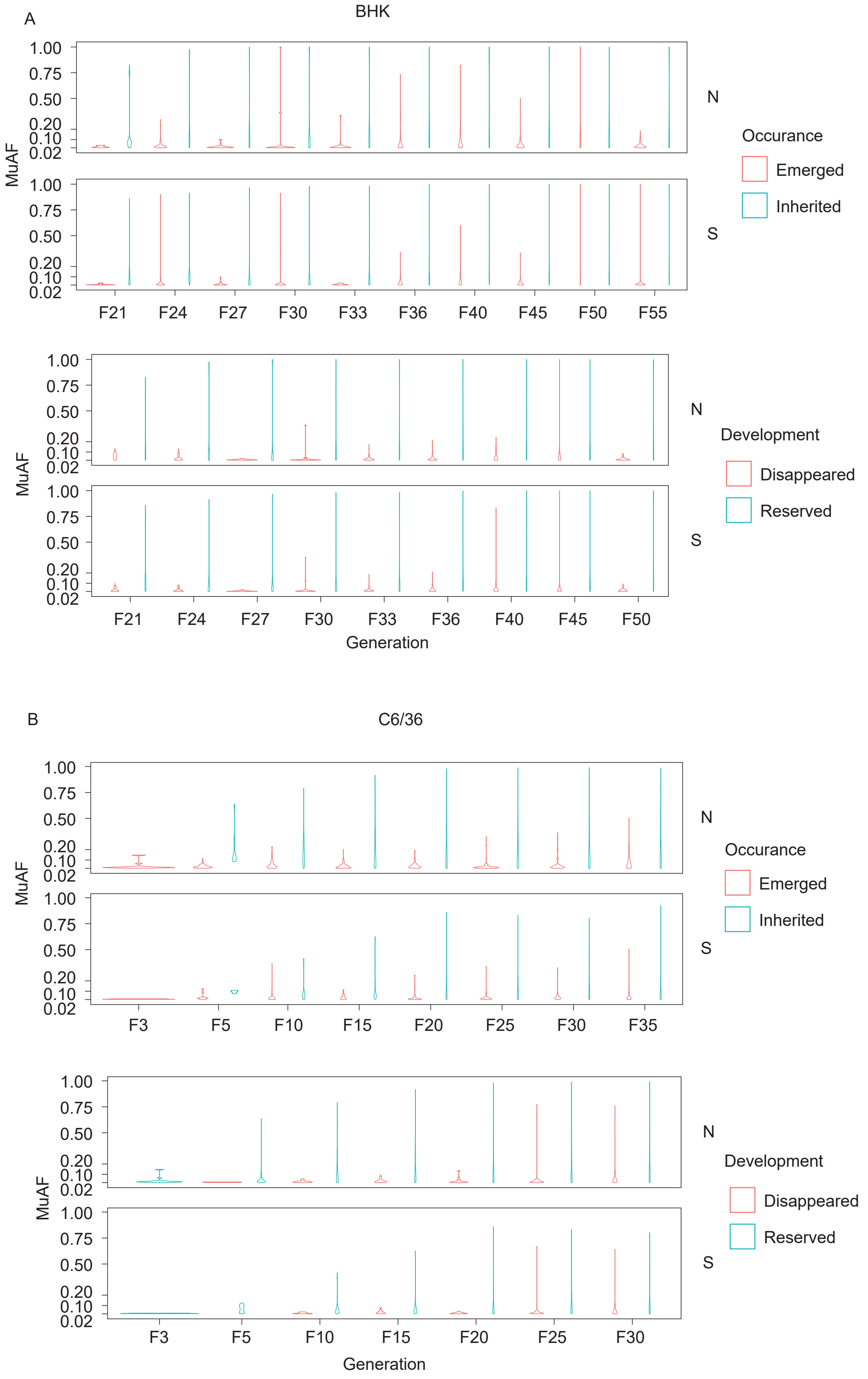


Supplementary Fig5

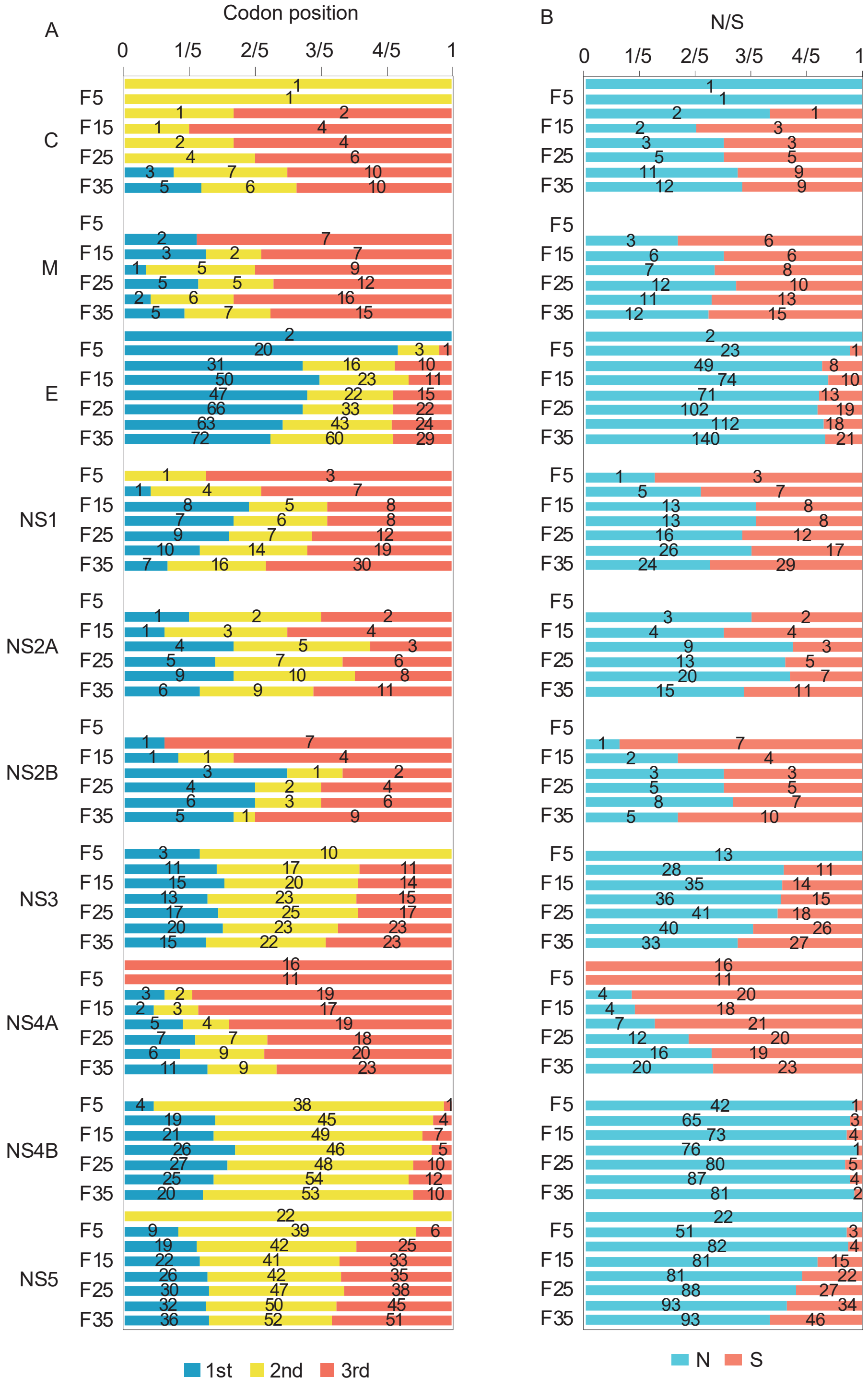


Supplementary Fig6

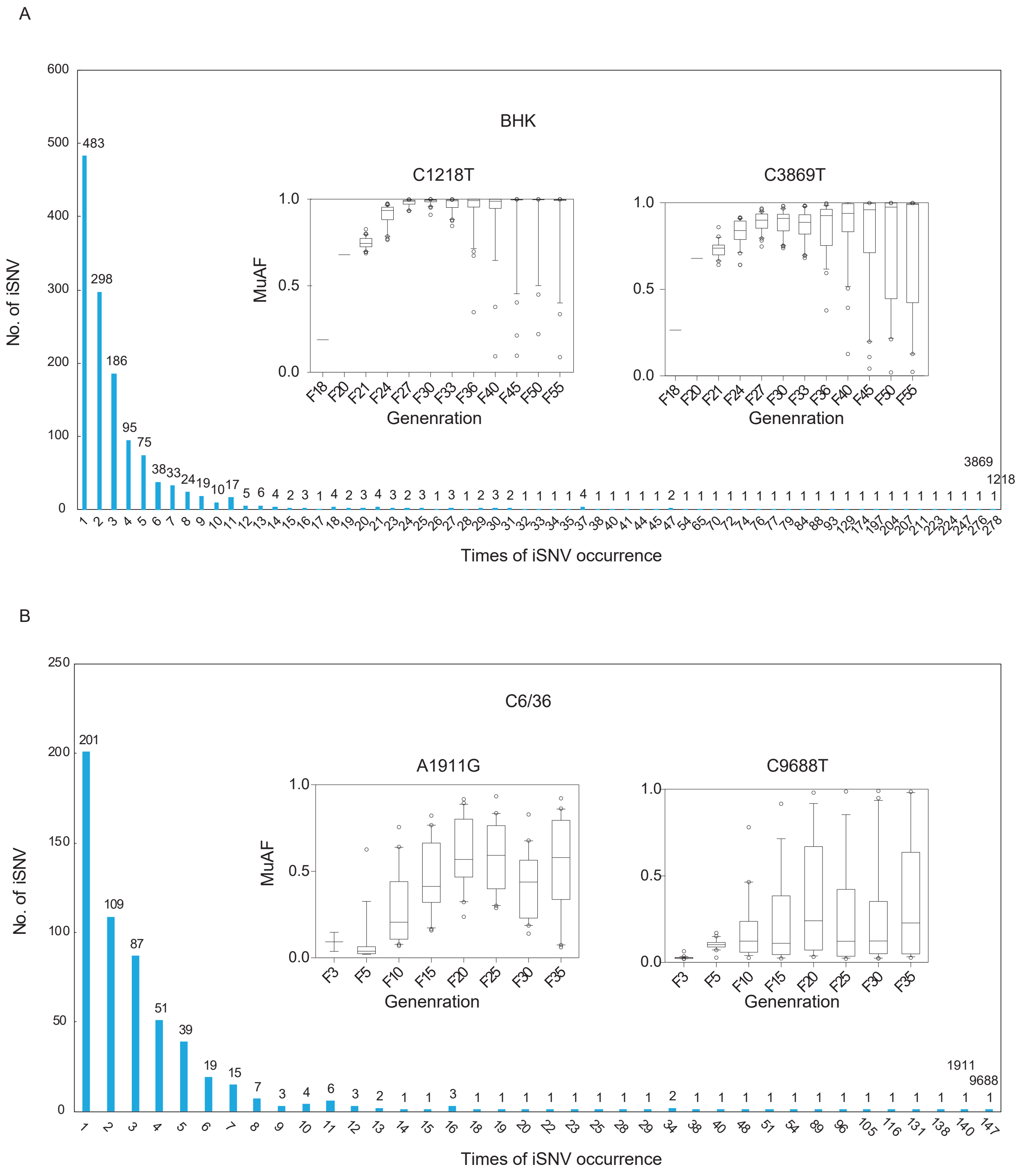


Supplementary Fig7

Table S1: Sequencing data  in BHK cells

| Generation | Sample | Total reads | Percent of JEV | Total JEV reads | Mean site depth | Genome coverage (bp) | iSNV | SNP | Sum |
|---|---|---|---|---|---|---|---|---|---|
| F4 | F4 | 8100107 | 1.50% | 121502 | 1081 | 10888 | 3 | 0 | 3 |
| F7 | F7 | 6057678 | 0.0195 | 118124.721 | 950 | 10879 | 26 | 0 | 26 |
| F11 | F11 | 4554545 | 0.0173 | 78793.6285 | 874 | 10876 | 33 | 0 | 33 |
| F15 | F15 | 4825291 | 0.01875 | 90474.2063 | 1042 | 10886 | 35 | 0 | 35 |
| F18 | F18 | 5610248 | 0.0139 | 77982.4472 | 628 | 10877 | 31 | 0 | 31 |
| F20 | F20 | 5922772 | 0.02245 | 132966.231 | 1248 | 10886 | 27 | 0 | 27 |
| F21 | F21-1 | 4375507 | 0.01715 | 75039.9451 | 920 | 10878 | 25 | 0 | 25 |
| F21 | F21-10 | 5097920 | 0.01085 | 55312.432 | 332 | 10843 | 14 | 0 | 14 |
| F21 | F21-11 | 5209957 | 0.0024 | 12503.8968 | 91 | 1690 | 10 | 0 | 10 |
| F21 | F21-12 | 5467280 | 0.0094 | 51392.432 | 469 | 10844 | 17 | 0 | 17 |
| F21 | F21-13 | 6595226 | 0.0156 | 102885.526 | 861 | 10845 | 22 | 0 | 22 |
| F21 | F21-14 | 7279933 | 0.00305 | 22203.7957 | 107 | 5955 | 11 | 0 | 11 |
| F21 | F21-15 | 5454237 | 0.01885 | 102812.367 | 1098 | 10891 | 20 | 0 | 20 |
| F21 | F21-16 | 4432621 | 0.01505 | 66710.9461 | 822 | 10878 | 26 | 0 | 26 |
| F21 | F21-17 | 8515447 | 0.00405 | 34487.5604 | 183 | 10753 | 14 | 0 | 14 |
| F21 | F21-18 | 5647683 | 0.01685 | 95163.4586 | 919 | 10885 | 21 | 0 | 21 |
| F21 | F21-19 | 5805604 | 0.01435 | 83310.4174 | 663 | 10845 | 23 | 0 | 23 |
| F21 | F21-2 | 4748738 | 0.00495 | 23506.2531 | 151 | 10613 | 14 | 0 | 14 |
| F21 | F21-20 | 8726037 | 0.0026 | 22687.6962 | 125 | 9521 | 13 | 0 | 13 |
| F21 | F21-21 | 5082927 | 0.01085 | 55149.7579 | 356 | 10819 | 16 | 0 | 16 |
| F21 | F21-22 | 6602688 | 0.01575 | 103992.336 | 1004 | 10878 | 19 | 0 | 19 |
| F21 | F21-23 | 6025639 | 0.0021 | 12653.8419 | 94 | 3316 | 8 | 0 | 8 |
| F21 | F21-24 | 5826439 | 0.01965 | 114489.526 | 1074 | 10884 | 22 | 0 | 22 |
| F21 | F21-25 | 4999208 | 0.01365 | 68239.1892 | 484 | 10845 | 22 | 0 | 22 |
| F21 | F21-26 | 5903667 | 0.00355 | 20958.0179 | 123 | 9676 | 11 | 0 | 11 |
| F21 | F21-27 | 6306635 | 0.01995 | 125817.368 | 1226 | 10890 | 27 | 0 | 27 |
| F21 | F21-28 | 5377121 | 0.0144 | 77430.5424 | 667 | 10850 | 21 | 0 | 21 |
| F21 | F21-29 | 7022644 | 0.00235 | 16503.2134 | 107 | 8194 | 11 | 0 | 11 |
| F21 | F21-3 | 4927172 | 0.01525 | 75139.373 | 661 | 10845 | 20 | 0 | 20 |
| F21 | F21-30 | 5057531 | 0.01495 | 75610.0885 | 678 | 10845 | 17 | 0 | 17 |
| F21 | F21-4 | 5062927 | 0.0136 | 68855.8072 | 583 | 10845 | 15 | 0 | 15 |
| F21 | F21-5 | 5133180 | 0.00425 | 21816.015 | 129 | 9938 | 13 | 0 | 13 |
| F21 | F21-6 | 5099140 | 0.0159 | 81076.326 | 590 | 10845 | 16 | 0 | 16 |
| F21 | F21-7 | 5429722 | 0.0133 | 72215.3026 | 468 | 10845 | 17 | 0 | 17 |
| F21 | F21-8 | 5768130 | 0.00525 | 30282.6825 | 156 | 10576 | 15 | 0 | 15 |
| F21 | F21-9 | 5549097 | 0.0191 | 105987.753 | 931 | 10856 | 15 | 0 | 15 |
| F21 | F24-1 | 4552202 | 0.20005 | 910668.01 | 7412 | 10914 | 21 | 0 | 21 |
| F24 | F24-10 | 4352227 | 0.0764 | 332510.143 | 1646 | 10917 | 17 | 0 | 17 |
| F24 | F24-11 | 5682572 | 0.1325 | 752940.79 | 6801 | 10924 | 16 | 0 | 16 |
| F24 | F24-12 | 5918744 | 0.22845 | 1352137.07 | 13843 | 10909 | 18 | 0 | 18 |
| F24 | F24-13 | 4930934 | 0.03975 | 196004.627 | 979 | 10902 | 14 | 0 | 14 |
| F24 | F24-14 | 5612633 | 0.1015 | 569682.25 | 7425 | 10925 | 32 | 0 | 32 |
| F24 | F24-15 | 6198269 | 0.17785 | 1102362.14 | 9681 | 10907 | 19 | 0 | 19 |
| F24 | F24-16 | 6632528 | 0.00685 | 45432.8168 | 235 | 10837 | 22 | 0 | 22 |
| F24 | F24-17 | 6583246 | 0.1028 | 676757.689 | 8200 | 10924 | 20 | 0 | 20 |
| F24 | F24-18 | 5938069 | 0.1535 | 911493.592 | 8773 | 10906 | 18 | 0 | 18 |
| F24 | F24-19 | 5606688 | 0.0241 | 135121.181 | 732 | 10867 | 27 | 0 | 27 |
| F24 | F24-2 | 6106994 | 0.0332 | 202752.201 | 712 | 10888 | 18 | 0 | 18 |
| F24 | F24-20 | 5340553 | 0.0456 | 243529.217 | 2679 | 10903 | 29 | 0 | 29 |
| F24 | F24-21 | 4792732 | 0.1452 | 695904.686 | 6359 | 10905 | 28 | 0 | 28 |
| F24 | F24-22 | 5877819 | 0.0563 | 330921.21 | 1727 | 10895 | 27 | 0 | 27 |
| F24 | F24-23 | 5451230 | 0.01885 | 102755.686 | 1058 | 10899 | 39 | 0 | 39 |
| F24 | F24-24 | 5214302 | 0.0597 | 311293.829 | 2563 | 10895 | 29 | 0 | 29 |
| F24 | F24-25 | 4085303 | 0.1235 | 504534.921 | 4725 | 10910 | 21 | 0 | 21 |
| F24 | F24-26 | 4491954 | 0.0325 | 145988.505 | 639 | 10898 | 20 | 0 | 20 |
| F24 | F24-27 | 4964948 | 0.05205 | 258425.543 | 2085 | 10894 | 17 | 0 | 17 |
| F24 | F24-28 | 4792900 | 0.03995 | 191476.355 | 1445 | 10894 | 30 | 0 | 30 |
| F24 | F24-29 | 5910038 | 0.0129 | 76239.4902 | 355 | 10846 | 30 | 0 | 30 |
| F24 | F24-3 | 5385491 | 0.2283 | 1229507.6 | 9647 | 10908 | 28 | 0 | 28 |
| F24 | F24-30 | 5186260 | 0.0915 | 474542.79 | 3878 | 10899 | 15 | 0 | 15 |
| F24 | F24-4 | 3602175 | 0.13015 | 468823.076 | 5698 | 10911 | 15 | 0 | 15 |
| F24 | F24-5 | 6306847 | 0.01935 | 122037.489 | 595 | 10883 | 24 | 0 | 24 |
| F24 | F24-6 | 6460840 | 0.0686 | 443213.624 | 3915 | 10902 | 23 | 0 | 23 |
| F24 | F24-7 | 4722962 | 0.14425 | 681287.269 | 5067 | 10909 | 16 | 0 | 16 |
| F24 | F24-8 | 5613276 | 0.02645 | 148471.15 | 625 | 10883 | 21 | 0 | 21 |
| F24 | F24-9 | 6456445 | 0.14485 | 935216.058 | 8524 | 10907 | 20 | 0 | 20 |
| F24 | F27-1 | 5001721 | 0.0286 | 143049.221 | 1528 | 10914 | 17 | 1 | 18 |
| F27 | F27-10 | 4495795 | 0.09905 | 445308.495 | 3702 | 10905 | 21 | 1 | 22 |
| F27 | F27-11 | 8735236 | 0.07315 | 638982.513 | 5951 | 10921 | 11 | 1 | 12 |
| F27 | F27-12 | 8502355 | 0.1309 | 1112958.27 | 12226 | 10925 | 22 | 0 | 22 |
| F27 | F27-13 | 3446044 | 0.0898 | 309454.751 | 2854 | 10902 | 10 | 1 | 11 |
| F27 | F27-14 | 8081340 | 0.08545 | 690550.503 | 6417 | 10923 | 23 | 1 | 24 |
| F27 | F27-15 | 9312272 | 0.0956 | 890253.203 | 8726 | 10925 | 20 | 1 | 21 |
| F27 | F27-16 | 4626981 | 0.07395 | 342165.245 | 2717 | 10904 | 24 | 0 | 24 |
| F27 | F27-17 | 7828080 | 0.09815 | 768326.052 | 6890 | 10925 | 21 | 1 | 22 |
| F27 | F27-18 | 8162448 | 0.06935 | 566065.769 | 4434 | 10913 | 21 | 1 | 22 |
| F27 | F27-19 | 6203564 | 0.04475 | 277609.489 | 2229 | 10901 | 25 | 0 | 25 |
| F27 | F27-2 | 8574515 | 0.0746 | 639658.819 | 5863 | 10920 | 16 | 1 | 17 |

Table S1: Sequencing data in BHK cells (Continued)

| Generation | Sample | Total reads | Percent of JEV | Total JEV reads | Mean site depth | Genome coverage (bp) | iSNV | SNP | Sum |
|---|---|---|---|---|---|---|---|---|---|
| F27 | F27-20 | 7969755 | 0.0671 | 534770.561 | 5280 | 10925 | 19 | 1 | 20 |
| F27 | F27-21 | 8663063 | 0.0832 | 720766.842 | 7549 | 10925 | 25 | 1 | 26 |
| F27 | F27-22 | 6588983 | 0.02715 | 178890.888 | 1881 | 10915 | 22 | 1 | 23 |
| F27 | F27-23 | 9145599 | 0.03745 | 342502.683 | 3042 | 10920 | 24 | 0 | 24 |
| F27 | F27-24 | 8479724 | 0.04815 | 408298.711 | 3840 | 10914 | 27 | 0 | 27 |
| F27 | F27-25 | 5049382 | 0.0395 | 199450.589 | 1799 | 10917 | 18 | 1 | 19 |
| F27 | F27-26 | 9790692 | 0.041 | 401418.372 | 3285 | 10908 | 20 | 1 | 21 |
| F27 | F27-27 | 8812015 | 0.1163 | 1024837.34 | 10255 | 10925 | 20 | 1 | 21 |
| F27 | F27-28 | 6371064 | 0.03415 | 217571.836 | 1873 | 10917 | 30 | 0 | 30 |
| F27 | F27-29 | 8530408 | 0.0987 | 841951.27 | 8723 | 10925 | 28 | 1 | 29 |
| F27 | F27-3 | 9011482 | 0.1201 | 1082278.99 | 10290 | 10925 | 27 | 0 | 27 |
| F27 | F27-30 | 8697033 | 0.0814 | 707938.486 | 6665 | 10914 | 11 | 1 | 12 |
| F27 | F27-4 | 6019262 | 0.01695 | 102026.491 | 896 | 10882 | 12 | 0 | 12 |
| F27 | F27-5 | 8530323 | 0.05565 | 474712.475 | 4790 | 10923 | 28 | 0 | 28 |
| F27 | F27-6 | 7224325 | 0.15155 | 1094846.45 | 10364 | 10925 | 26 | 0 | 26 |
| F27 | F27-7 | 4426851 | 0.0645 | 285531.89 | 2554 | 10905 | 17 | 1 | 18 |
| F27 | F27-8 | 8587711 | 0.06925 | 594698.987 | 5519 | 10913 | 16 | 1 | 17 |
| F27 | F27-9 | 9105341 | 0.0756 | 688363.78 | 5882 | 10920 | 23 | 1 | 24 |
| F27 | F30-1 | 10144602 | 0.03485 | 353539.38 | 3449 | 10906 | 21 | 1 | 22 |
| F30 | F30-10 | 11190081 | 0.0211 | 236110.709 | 1940 | 10902 | 21 | 1 | 22 |
| F30 | F30-11 | 11664035 | 0.0234 | 272938.419 | 2761 | 10904 | 14 | 1 | 15 |
| F30 | F30-12 | 9893927 | 0.02645 | 261694.369 | 2552 | 10904 | 21 | 0 | 21 |
| F30 | F30-13 | 9752932 | 0.05425 | 529096.561 | 4758 | 10915 | 5 | 2 | 7 |
| F30 | F30-14 | 10043276 | 0.03995 | 401228.876 | 3211 | 10909 | 22 | 1 | 23 |
| F30 | F30-15 | 12077472 | 0.0653 | 788658.922 | 6312 | 10911 | 18 | 1 | 19 |
| F30 | F30-16 | 9786359 | 0.03845 | 376285.504 | 2924 | 10906 | 20 | 1 | 21 |
| F30 | F30-17 | 10467129 | 0.02015 | 210912.649 | 1685 | 10903 | 19 | 1 | 20 |
| F30 | F30-18 | 11254255 | 0.0369 | 415282.01 | 3272 | 10908 | 22 | 1 | 23 |
| F30 | F30-19 | 11284444 | 0.03245 | 366180.208 | 2934 | 10906 | 22 | 1 | 23 |
| F30 | F30-2 | 11036256 | 0.0287 | 316740.547 | 2804 | 10902 | 17 | 1 | 18 |
| F30 | F30-20 | 10136127 | 0.0277 | 280770.718 | 2099 | 10899 | 19 | 1 | 20 |
| F30 | F30-21 | 10668527 | 0.05385 | 574500.179 | 5780 | 10911 | 23 | 1 | 24 |
| F30 | F30-22 | 11762476 | 0.0474 | 557541.362 | 4975 | 10909 | 23 | 1 | 24 |
| F30 | F30-23 | 11313385 | 0.03405 | 385220.759 | 3362 | 10908 | 25 | 0 | 25 |
| F30 | F30-24 | 9363180 | 0.05565 | 521060.967 | 5163 | 10910 | 24 | 0 | 24 |
| F30 | F30-25 | 8280263 | 0.0758 | 627643.935 | 7239 | 10911 | 20 | 1 | 21 |
| F30 | F30-26 | 8836588 | 0.0464 | 410017.683 | 4166 | 10908 | 18 | 1 | 19 |
| F30 | F30-27 | 10183387 | 0.0655 | 667011.849 | 6800 | 10910 | 20 | 1 | 21 |
| F30 | F30-28 | 9670929 | 0.03855 | 372814.313 | 3452 | 10907 | 27 | 0 | 27 |
| F30 | F30-29 | 8817094 | 0.04865 | 428951.623 | 4598 | 10908 | 20 | 1 | 21 |
| F30 | F30-3 | 11485209 | 0.03875 | 445051.849 | 3897 | 10907 | 18 | 1 | 19 |
| F30 | F30-30 | 8923736 | 0.05965 | 532300.852 | 4844 | 10878 | 5 | 2 | 7 |
| F30 | F30-4 | 10481061 | 0.0221 | 231631.448 | 2311 | 10905 | 15 | 1 | 16 |
| F30 | F30-5 | 10665799 | 0.02085 | 222381.909 | 1947 | 10906 | 23 | 1 | 24 |
| F30 | F30-6 | 11827603 | 0.02495 | 295098.695 | 2737 | 10906 | 27 | 0 | 27 |
| F30 | F30-7 | 10867586 | 0.03115 | 338525.304 | 4164 | 10907 | 18 | 1 | 19 |
| F30 | F30-8 | 10585351 | 0.0186 | 196887.529 | 1576 | 10901 | 16 | 1 | 17 |
| F30 | F30-9 | 10166793 | 0.0386 | 392438.21 | 4102 | 10906 | 26 | 1 | 27 |
| F30 | F33-1 | 8885034 | 0.1022 | 908050.475 | 9656 | 10910 | 22 | 1 | 23 |
| F33 | F33-10 | 8290826 | 0.1048 | 868878.565 | 9653 | 10911 | 17 | 1 | 18 |
| F33 | F33-11 | 7997012 | 0.1109 | 886868.631 | 9686 | 10911 | 14 | 1 | 15 |
| F33 | F33-12 | 8579230 | 0.0949 | 814168.927 | 7706 | 10911 | 22 | 0 | 22 |
| F33 | F33-13 | 10016656 | 0.08495 | 850914.927 | 11090 | 10912 | 7 | 2 | 9 |
| F33 | F33-14 | 10156941 | 0.0924 | 938501.348 | 11064 | 10916 | 18 | 1 | 19 |
| F33 | F33-15 | 12224777 | 0.12165 | 1487144.12 | 19605 | 10925 | 18 | 1 | 19 |
| F33 | F33-16 | 10653083 | 0.06625 | 705766.749 | 9488 | 10921 | 19 | 1 | 20 |
| F33 | F33-17 | 10362895 | 0.0627 | 649753.517 | 8153 | 10911 | 23 | 1 | 24 |
| F33 | F33-18 | 10789965 | 0.08195 | 884237.632 | 11735 | 10921 | 15 | 1 | 16 |
| F33 | F33-19 | 10039582 | 0.06255 | 627975.854 | 9994 | 10925 | 28 | 0 | 28 |
| F33 | F33-2 | 8293821 | 0.0767 | 636136.071 | 7010 | 10911 | 18 | 1 | 19 |
| F33 | F33-20 | 11273276 | 0.05655 | 637503.758 | 8227 | 10925 | 18 | 1 | 19 |
| F33 | F33-21 | 10666175 | 0.06945 | 740765.854 | 9096 | 10913 | 23 | 1 | 24 |
| F33 | F33-22 | 10189482 | 0.07095 | 722943.748 | 10697 | 10922 | 32 | 0 | 32 |
| F33 | F33-23 | 10031125 | 0.05305 | 532151.181 | 8100 | 10921 | 26 | 0 | 26 |
| F33 | F33-24 | 10014992 | 0.0644 | 644965.485 | 7898 | 10911 | 24 | 0 | 24 |
| F33 | F33-25 | 10806287 | 0.0619 | 668909.165 | 9259 | 10911 | 18 | 1 | 19 |
| F33 | F33-26 | 10112294 | 0.05365 | 542524.573 | 7956 | 10911 | 23 | 1 | 24 |
| F33 | F33-27 | 9901878 | 0.0605 | 599063.619 | 8593 | 10910 | 14 | 1 | 15 |
| F33 | F33-28 | 10604268 | 0.0306 | 324490.601 | 3979 | 10906 | 30 | 0 | 30 |
| F33 | F33-29 | 10136098 | 0.0503 | 509845.729 | 7333 | 10909 | 16 | 2 | 18 |
| F33 | F33-3 | 9124551 | 0.1336 | 1219040.01 | 13511 | 10911 | 19 | 0 | 19 |
| F33 | F33-30 | 9802047 | 0.05645 | 553325.553 | 6212 | 10911 | 4 | 2 | 6 |
| F33 | F33-4 | 8914305 | 0.0893 | 796047.437 | 7878 | 10911 | 16 | 1 | 17 |
| F33 | F33-5 | 11698299 | 8.40% | 982072 | 10524 | 10911 | 30 | 0 | 30 |
| F33 | F33-6 | 8789040 | 10.70% | 940427 | 9695 | 10910 | 28 | 0 | 28 |
| F33 | F33-7 | 8977794 | 10.02% | 899126 | 9285 | 10911 | 21 | 1 | 22 |
| F33 | F33-8 | 9458002 | 7.02% | 663952 | 7688 | 10911 | 26 | 1 | 27 |
| F33 | F33-9 | 9059705 | 10.47% | 948551 | 9688 | 10911 | 24 | 1 | 25 |

Table S1: Sequencing data in BHK cells (Continued)

| Generation | Sample | Total reads | Percent of JEV | Total JEV reads | Mean site depth | Genome coverage (bp) | iSNV | SNP | Sum |
|---|---|---|---|---|---|---|---|---|---|
| F33 | F36-1 | 5151136 | 0.59% | 30134 | 281 | 10836 | 25 | 1 | 26 |
| F36 | F36-10 | 5234788 | 0.80% | 41878 | 409 | 10842 | 20 | 1 | 21 |
| F36 | F36-11 | 5877350 | 1.19% | 69647 | 628 | 10844 | 12 | 2 | 14 |
| F36 | F36-12 | 5374410 | 0.78% | 41920 | 476 | 10842 | 33 | 0 | 33 |
| F36 | F36-13 | 3926800 | 2.37% | 93065 | 696 | 10847 | 25 | 2 | 27 |
| F36 | F36-14 | 5015401 | 0.99% | 49402 | 496 | 10845 | 28 | 1 | 29 |
| F36 | F36-15 | 5129411 | 1.00% | 51038 | 388 | 10842 | 27 | 1 | 28 |
| F36 | F36-16 | 5339402 | 0.74% | 39245 | 292 | 10770 | 31 | 1 | 32 |
| F36 | F36-17 | 3875124 | 0.86% | 33132 | 318 | 10837 | 29 | 1 | 30 |
| F36 | F36-18 | 7736278 | 1.28% | 98638 | 569 | 10842 | 24 | 1 | 25 |
| F36 | F36-19 | 6577954 | 1.28% | 83869 | 792 | 10845 | 40 | 0 | 40 |
| F36 | F36-2 | 5349901 | 0.93% | 49754 | 342 | 10839 | 28 | 1 | 29 |
| F36 | F36-20 | 5342319 | 1.09% | 58231 | 466 | 10841 | 26 | 1 | 27 |
| F36 | F36-21 | 5946213 | 0.81% | 48164 | 421 | 10843 | 27 | 1 | 28 |
| F36 | F36-22 | 7293471 | 0.59% | 42667 | 547 | 10845 | 42 | 0 | 42 |
| F36 | F36-23 | 5978138 | 0.69% | 40950 | 342 | 10843 | 33 | 0 | 33 |
| F36 | F36-24 | 5427569 | 0.92% | 49934 | 472 | 10844 | 46 | 0 | 46 |
| F36 | F36-25 | 5975660 | 0.44% | 25994 | 212 | 10674 | 19 | 1 | 20 |
| F36 | F36-26 | 6276028 | 0.90% | 56484 | 456 | 10844 | 24 | 1 | 25 |
| F36 | F36-27 | 6609772 | 0.37% | 24456 | 307 | 10831 | 12 | 2 | 14 |
| F36 | F36-28 | 6493552 | 0.79% | 51299 | 480 | 10845 | 21 | 0 | 21 |
| F36 | F36-29 | 5272179 | 1.26% | 66429 | 741 | 10855 | 29 | 1 | 30 |
| F36 | F36-3 | 5577582 | 0.69% | 38485 | 376 | 10841 | 30 | 1 | 31 |
| F36 | F36-30 | 6838401 | 0.71% | 48553 | 622 | 10845 | 15 | 2 | 17 |
| F36 | F36-4 | 5262158 | 0.99% | 52095 | 472 | 10844 | 22 | 1 | 23 |
| F36 | F36-5 | 5249242 | 0.60% | 31495 | 320 | 10841 | 32 | 0 | 32 |
| F36 | F36-6 | 5849934 | 0.99% | 57914 | 589 | 10845 | 22 | 0 | 22 |
| F36 | F36-7 | 5431064 | 1.15% | 62457 | 639 | 10844 | 32 | 1 | 33 |
| F36 | F36-8 | 5939290 | 0.96% | 56720 | 513 | 10844 | 34 | 1 | 35 |
| F36 | F36-9 | 4100353 | 0.82% | 33418 | 263 | 10818 | 52 | 0 | 52 |
| F36 | F40-1 | 2451101 | 1.41% | 34561 | 274 | 10837 | 22 | 1 | 23 |
| F40 | F40-10 | 3006759 | 0.78% | 23453 | 197 | 10774 | 7 | 6 | 13 |
| F40 | F40-11 | 4287666 | 0.88% | 37731 | 286 | 10837 | 8 | 5 | 13 |
| F40 | F40-12 | 4362070 | 0.89% | 38822 | 298 | 10840 | 51 | 0 | 51 |
| F40 | F40-13 | 2961899 | 1.06% | 31396 | 337 | 10840 | 27 | 1 | 28 |
| F40 | F40-14 | 5899330 | 0.51% | 29792 | 162 | 10654 | 18 | 0 | 18 |
| F40 | F40-15 | 2715329 | 0.82% | 22266 | 104 | 2149 | 12 | 0 | 12 |
| F40 | F40-16 | 4421939 | 0.76% | 33607 | 211 | 10819 | 37 | 1 | 38 |
| F40 | F40-17 | 3508840 | 0.79% | 27720 | 209 | 10808 | 39 | 1 | 40 |
| F40 | F40-18 | 5711353 | 1.37% | 78246 | 417 | 10845 | 36 | 1 | 37 |
| F40 | F40-19 | 4933934 | 0.80% | 39471 | 233 | 10831 | 29 | 1 | 30 |
| F40 | F40-2 | 8488645 | 0.92% | 78096 | 683 | 10846 | 54 | 0 | 54 |
| F40 | F40-20 | 4689949 | 0.43% | 19932 | 136 | 9172 | 34 | 1 | 35 |
| F40 | F40-21 | 4116017 | 1.01% | 41572 | 263 | 10838 | 35 | 2 | 37 |
| F40 | F40-22 | 4053271 | 0.96% | 38911 | 202 | 10741 | 32 | 1 | 33 |
| F40 | F40-23 | 5001573 | 0.85% | 42513 | 221 | 10814 | 32 | 0 | 32 |
| F40 | F40-24 | 4032417 | 0.51% | 20364 | 126 | 7564 | 29 | 0 | 29 |
| F40 | F40-25 | 4980411 | 0.78% | 38598 | 361 | 10842 | 44 | 0 | 44 |
| F40 | F40-26 | 4741189 | 0.90% | 42434 | 398 | 10843 | 23 | 1 | 24 |
| F40 | F40-27 | 5097317 | 1.00% | 50973 | 418 | 10844 | 48 | 1 | 49 |
| F40 | F40-28 | 3338093 | 1.16% | 38555 | 301 | 10840 | 37 | 0 | 37 |
| F40 | F40-29 | 2852863 | 1.49% | 42508 | 562 | 10845 | 20 | 3 | 23 |
| F40 | F40-3 | 3944963 | 1.02% | 40041 | 265 | 10836 | 27 | 1 | 28 |
| F40 | F40-30 | 2765642 | 0.96% | 26550 | 267 | 10839 | 15 | 0 | 15 |
| F40 | F40-4 | 3116649 | 0.97% | 30231 | 343 | 10839 | 35 | 1 | 36 |
| F40 | F40-5 | 3444342 | 1.01% | 34788 | 434 | 10844 | 29 | 1 | 30 |
| F40 | F40-6 | 6104301 | 0.32% | 19229 | 117 | 7229 | 16 | 0 | 16 |
| F40 | F40-7 | 3633873 | 1.42% | 51601 | 447 | 10839 | 18 | 3 | 21 |
| F40 | F40-8 | 4574756 | 0.83% | 37970 | 285 | 10836 | 22 | 2 | 24 |
| F40 | F40-9 | 3654190 | 1.20% | 43850 | 296 | 10838 | 30 | 2 | 32 |
| F40 | F45-1 | 6545988 | 30.64% | 2005363 | 20704 | 10911 | 45 | 3 | 48 |
| F45 | F45-10 | 6229145 | 25.71% | 1601202 | 15467 | 10911 | 27 | 6 | 33 |
| F45 | F45-11 | 6158412 | 45.56% | 2805465 | 29705 | 10925 | 37 | 2 | 39 |
| F45 | F45-12 | 7168140 | 42.98% | 3080508 | 36440 | 10925 | 52 | 0 | 52 |
| F45 | F45-13 | 6827645 | 32.26% | 2202598 | 23509 | 10924 | 38 | 2 | 40 |
| F45 | F45-14 | 5968570 | 33.13% | 1977089 | 19783 | 10911 | 50 | 1 | 51 |
| F45 | F45-15 | 5445574 | 46.43% | 2528108 | 27788 | 10925 | 59 | 1 | 60 |
| F45 | F45-16 | 5347884 | 45.56% | 2436496 | 26988 | 10925 | 63 | 1 | 64 |
| F45 | F45-17 | 7736070 | 61.23% | 4736409 | 51190 | 10925 | 36 | 2 | 38 |
| F45 | F45-18 | 7300768 | 60.05% | 4383746 | 43462 | 10925 | 35 | 1 | 36 |
| F45 | F45-19 | 6976483 | 53.77% | 3750906 | 41180 | 10925 | 44 | 1 | 45 |
| F45 | F45-2 | 5595742 | 22.98% | 1285622 | 15704 | 10910 | 51 | 1 | 52 |
| F45 | F45-20 | 7526652 | 35.47% | 2669703 | 27552 | 10921 | 39 | 1 | 40 |
| F45 | F45-21 | 7766970 | 59.75% | 4640376 | 48403 | 10925 | 32 | 2 | 34 |
| F45 | F45-22 | 6638816 | 58.34% | 3872753 | 38076 | 10925 | 26 | 1 | 27 |
| F45 | F45-23 | 5376102 | 63.67% | 3422964 | 34249 | 10911 | 36 | 0 | 36 |
| F45 | F45-24 | 6273744 | 63.16% | 3962497 | 40168 | 10925 | 43 | 2 | 45 |
| F45 | F45-25 | 4391324 | 56.57% | 2483952 | 27421 | 10915 | 38 | 1 | 39 |

Table S1: Sequencing data in BHK cells (Continued)

| Generation | Sample | Total reads | Percent of JEV | Total JEV reads | Mean site depth | Genome coverage (bp) | iSNV | SNP | Sum |
|---|---|---|---|---|---|---|---|---|---|
| F45 | F45-26 | 5355716 | 62.20% | 3331255 | 27748 | 10925 | 28 | 2 | 30 |
| F45 | F45-27 | 5322375 | 60.34% | 3211521 | 28080 | 10925 | 32 | 2 | 34 |
| F45 | F45-28 | 6211791 | 58.40% | 3627686 | 33453 | 10921 | 48 | 0 | 48 |
| F45 | F45-29 | 7164896 | 79.61% | 5703974 | 54396 | 10925 | 23 | 3 | 26 |
| F45 | F45-3 | 5329466 | 23.87% | 1271877 | 14225 | 10910 | 32 | 2 | 34 |
| F45 | F45-30 | 6680410 | 71.31% | 4763466 | 42418 | 10925 | 8 | 7 | 15 |
| F45 | F45-4 | 6428189 | 8.36% | 537397 | 1561 | 10925 | 48 | 1 | 49 |
| F45 | F45-5 | 6306931 | 51.58% | 3252800 | 33441 | 10921 | 40 | 0 | 40 |
| F45 | F45-6 | 6213012 | 26.78% | 1663534 | 15632 | 10910 | 33 | 0 | 33 |
| F45 | F45-7 | 5574886 | 42.66% | 2378246 | 25524 | 10918 | 59 | 1 | 60 |
| F45 | F45-8 | 6076229 | 56.08% | 3407245 | 35451 | 10925 | 30 | 2 | 32 |
| F45 | F45-9 | 6405100 | 40.19% | 2573889 | 27285 | 10912 | 28 | 2 | 30 |
| F45 | F50-1 | 4784245 | 13.45% | 643481 | 6098 | 10899 | 16 | 16 | 32 |
| F50 | F50-10 | 4651997 | 18.28% | 850152 | 10628 | 10895 | 14 | 15 | 29 |
| F50 | F50-11 | 4799854 | 33.61% | 1612991 | 15792 | 10925 | 33 | 2 | 35 |
| F50 | F50-12 | 6661858 | 38.49% | 2563816 | 25952 | 10925 | 57 | 0 | 57 |
| F50 | F50-13 | 3277577 | 49.33% | 1616829 | 22879 | 10925 | 43 | 2 | 45 |
| F50 | F50-14 | 4567003 | 45.31% | 2069309 | 17027 | 10922 | 54 | 1 | 55 |
| F50 | F50-15 | 6454107 | 36.25% | 2339614 | 19589 | 10922 | 48 | 1 | 49 |
| F50 | F50-16 | 5129428 | 37.83% | 1940206 | 19968 | 10922 | 52 | 1 | 53 |
| F50 | F50-17 | 5961705 | 31.76% | 1893139 | 16574 | 10925 | 39 | 3 | 42 |
| F50 | F50-18 | 4032732 | 45.92% | 1851831 | 21717 | 10914 | 41 | 1 | 42 |
| F50 | F50-19 | 5443953 | 42.44% | 2310141 | 22711 | 10921 | 48 | 1 | 49 |
| F50 | F50-2 | 5335254 | 16.42% | 875782 | 8531 | 10901 | 11 | 15 | 26 |
| F50 | F50-20 | 5187886 | 35.97% | 1865823 | 16761 | 10911 | 43 | 1 | 44 |
| F50 | F50-21 | 3765254 | 38.57% | 1452258 | 15723 | 10911 | 45 | 2 | 47 |
| F50 | F50-22 | 5317927 | 48.71% | 2590096 | 24516 | 10911 | 17 | 6 | 23 |
| F50 | F50-23 | 4539672 | 41.96% | 1904846 | 18953 | 10925 | 45 | 1 | 46 |
| F50 | F50-24 | 4647617 | 8.00% | 371577 | 3477 | 10906 | 34 | 3 | 37 |
| F50 | F50-25 | 6556229 | 35.27% | 2312054 | 18931 | 10921 | 30 | 3 | 33 |
| F50 | F50-26 | 7351027 | 27.62% | 2029986 | 22849 | 10913 | 37 | 2 | 39 |
| F50 | F50-27 | 4209137 | 39.89% | 1679025 | 14831 | 10922 | 37 | 2 | 39 |
| F50 | F50-28 | 6093332 | 30.47% | 1856334 | 17520 | 10925 | 47 | 0 | 47 |
| F50 | F50-29 | 5262316 | 32.35% | 1702096 | 17198 | 10925 | 29 | 3 | 32 |
| F50 | F50-3 | 3668897 | 21.44% | 786612 | 7399 | 10900 | 38 | 2 | 40 |
| F50 | F50-30 | 4631161 | 38.85% | 1799206 | 18235 | 10925 | 18 | 7 | 25 |
| F50 | F50-4 | 5156327 | 25.33% | 1306098 | 13327 | 10902 | 9 | 15 | 24 |
| F50 | F50-5 | 3463850 | 21.35% | 739359 | 6692 | 10900 | 33 | 1 | 34 |
| F50 | F50-6 | 7120694 | 27.86% | 1983469 | 16402 | 10903 | 8 | 16 | 24 |
| F50 | F50-7 | 3020023 | 30.10% | 908876 | 9212 | 10902 | 9 | 16 | 25 |
| F50 | F50-8 | 4544772 | 29.51% | 1340935 | 10588 | 10902 | 20 | 16 | 36 |
| F50 | F50-9 | 5969769 | 31.66% | 1889730 | 18615 | 10907 | 12 | 15 | 27 |
| F50 | F55-1 | 2792203 | 15.85% | 442564 | 4448 | 10917 | 39 | 0 | 39 |
| F55 | F55-10 | 4922015 | 34.49% | 1697357 | 16949 | 10916 | 22 | 16 | 38 |
| F55 | F55-11 | 2287481 | 25.48% | 582850 | 6524 | 10910 | 35 | 2 | 37 |
| F55 | F55-12 | 2749147 | 34.62% | 951755 | 8258 | 10912 | 51 | 0 | 51 |
| F55 | F55-13 | 19532055 | 30.49% | 5955324 | 71344 | 10925 | 42 | 2 | 44 |
| F55 | F55-14 | 18828408 | 46.14% | 8686486 | 83818 | 10925 | 54 | 1 | 55 |
| F55 | F55-15 | 20246745 | 29.11% | 5892815 | 69509 | 10925 | 52 | 1 | 53 |
| F55 | F55-16 | 18941153 | 28.42% | 5382129 | 61798 | 10925 | 50 | 1 | 51 |
| F55 | F55-17 | 17619966 | 24.77% | 4364466 | 49230 | 10925 | 40 | 3 | 43 |
| F55 | F55-18 | 3383052 | 48.18% | 1629954 | 13343 | 10925 | 42 | 1 | 43 |
| F55 | F55-19 | 2867532 | 44.08% | 1263865 | 13120 | 10924 | 46 | 1 | 47 |
| F55 | F55-2 | 4260585 | 30.86% | 1314817 | 12700 | 10918 | 8 | 16 | 24 |
| F55 | F55-20 | 2919206 | 38.07% | 1111342 | 10611 | 10924 | 55 | 1 | 56 |
| F55 | F55-21 | 2771003 | 40.07% | 1110341 | 10233 | 10907 | 40 | 2 | 42 |
| F55 | F55-22 | 3615020 | 51.87% | 1875111 | 15728 | 10909 | 16 | 9 | 25 |
| F55 | F55-23 | 3369792 | 41.99% | 1414807 | 11686 | 10908 | 33 | 1 | 34 |
| F55 | F55-24 | 2853047 | 22.38% | 638369 | 5756 | 10906 | 28 | 7 | 35 |
| F55 | F55-25 | 3869669 | 44.96% | 1739803 | 15435 | 10909 | 31 | 3 | 34 |
| F55 | F55-26 | 3465484 | 44.00% | 1524640 | 13735 | 10910 | 39 | 2 | 41 |
| F55 | F55-27 | 2851540 | 37.99% | 1083157 | 8719 | 10905 | 36 | 2 | 38 |
| F55 | F55-28 | 3207376 | 39.51% | 1267074 | 9845 | 10906 | 28 | 3 | 31 |
| F55 | F55-29 | 4290764 | 57.55% | 2469335 | 22167 | 10910 | 28 | 3 | 31 |
| F55 | F55-3 | 3585879 | 25.80% | 925157 | 8660 | 10917 | 26 | 15 | 41 |
| F55 | F55-30 | 3612107 | 49.28% | 1779866 | 17500 | 10909 | 35 | 8 | 43 |
| F55 | F55-4 | 2990555 | 29.26% | 874887 | 8327 | 10917 | 13 | 15 | 28 |
| F55 | F55-5 | 4266922 | 25.67% | 1095106 | 8720 | 10917 | 23 | 15 | 38 |
| F55 | F55-6 | 4400463 | 33.30% | 1465134 | 11932 | 10917 | 10 | 15 | 25 |
| F55 | F55-7 | 3164092 | 26.87% | 850033 | 9044 | 10917 | 16 | 16 | 32 |
| F55 | F55-8 | 4006210 | 33.93% | 1359107 | 11802 | 10904 | 27 | 10 | 37 |
| F55 | F55-9 | 1785558 | 20.81% | 371485 | 5743 | 10917 | 20 | 12 | 32 |

Table S2: Sequencing data in C6/36 cells

| Generation | Sample | Total reads | Percent of JEV | Total JEV reads | Mean site depth | Genome coverage (bp) | iSNV | SNP | Sum |
|---|---|---|---|---|---|---|---|---|---|
| F1 | F1-1 | 8594577 | 2.10% | 180056 | 2629 | 10909 | 0 | 0 | 0 |
| F1 | F1-10 | 8521642 | 7.69% | 655314 | 7535 | 10913 | 0 | 0 | 0 |
| F1 | F1-13 | 8237623 | 7.48% | 615762 | 6983 | 10910 | 0 | 0 | 0 |
| F1 | F1-16 | 9347386 | 4.00% | 373895 | 4365 | 10909 | 0 | 0 | 0 |
| F1 | F1-19 | 8805635 | 10.81% | 951889 | 12561 | 10943 | 0 | 0 | 0 |
| F1 | F1-22 | 7788123 | 10.99% | 855915 | 8866 | 10910 | 0 | 0 | 0 |
| F1 | F1-25 | 7407574 | 12.02% | 890020 | 11709 | 10927 | 0 | 0 | 0 |
| F1 | F1-28 | 8148005 | 5.55% | 451807 | 4964 | 10906 | 0 | 0 | 0 |
| F1 | F1-4 | 7195251 | 0.87% | 62239 | 735 | 10893 | 0 | 0 | 0 |
| F1 | F1-7 | 9184354 | 3.86% | 354516 | 4069 | 10906 | 0 | 0 | 0 |
| F3 | F3-1 | 6385018 | 10.07% | 642652 | 7477 | 10942 | 2 | 0 | 2 |
| F3 | F3-10 | 8043852 | 70.40% | 5662470 | 61366 | 10947 | 2 | 0 | 2 |
| F3 | F3-11 | 7480233 | 70.63% | 5282915 | 45496 | 10947 | 2 | 0 | 2 |
| F3 | F3-13 | 8490048 | 71.76% | 6092034 | 56571 | 10947 | 1 | 0 | 1 |
| F3 | F3-14 | 8853539 | 68.00% | 6020407 | 61030 | 10947 | 2 | 0 | 2 |
| F3 | F3-16 | 7268786 | 17.94% | 1304020 | 17188 | 10945 | 2 | 0 | 2 |
| F3 | F3-17 | 8232131 | 65.53% | 5394104 | 53923 | 10947 | 2 | 0 | 2 |
| F3 | F3-19 | 7611783 | 70.58% | 5372016 | 48185 | 10947 | 2 | 0 | 2 |
| F3 | F3-2 | 7177830 | 61.83% | 4438052 | 42079 | 10947 | 3 | 0 | 3 |
| F3 | F3-20 | 6379721 | 58.53% | 3734051 | 36006 | 10947 | 2 | 0 | 2 |
| F3 | F3-22 | 5862336 | 72.60% | 4255763 | 41294 | 10947 | 2 | 0 | 2 |
| F3 | F3-23 | 6470519 | 65.26% | 4222337 | 40108 | 10947 | 2 | 0 | 2 |
| F3 | F3-25 | 6742000 | 68.86% | 4642204 | 56566 | 10947 | 5 | 0 | 5 |
| F3 | F3-26 | 6900817 | 70.56% | 4869216 | 59092 | 10947 | 2 | 0 | 2 |
| F3 | F3-28 | 7980206 | 73.63% | 5875826 | 67005 | 10948 | 2 | 0 | 2 |
| F3 | F3-30 | 9032810 | 78.11% | 7055076 | 84769 | 10947 | 1 | 0 | 1 |
| F3 | F3-4 | 8270434 | 71.92% | 5947683 | 72260 | 10947 | 2 | 0 | 2 |
| F3 | F3-5 | 8318454 | 74.53% | 6199328 | 63463 | 10947 | 2 | 0 | 2 |
| F3 | F3-7 | 7207113 | 69.44% | 5004259 | 44130 | 10947 | 1 | 0 | 1 |
| F3 | F3-8 | 7504910 | 71.25% | 5347248 | 55483 | 10947 | 2 | 0 | 2 |
| F5 | F5-1 | 10441166 | 47.70% | 4980436 | 70635 | 10945 | 19 | 0 | 19 |
| F5 | F5-10 | 9676098 | 29.26% | 2830742 | 15721 | 10908 | 8 | 0 | 8 |
| F5 | F5-11 | 9725399 | 54.09% | 5260468 | 37068 | 10939 | 7 | 0 | 7 |
| F5 | F5-13 | 7359764 | 18.53% | 1363764 | 8471 | 10907 | 10 | 0 | 10 |
| F5 | F5-14 | 6612669 | 32.09% | 2121675 | 14049 | 10910 | 9 | 0 | 9 |
| F5 | F5-16 | 9861294 | 59.08% | 5826052 | 79631 | 10947 | 8 | 0 | 8 |
| F5 | F5-17 | 4840093 | 42.54% | 2058734 | 11846 | 10907 | 5 | 0 | 5 |
| F5 | F5-19 | 9316518 | 36.74% | 3422423 | 26393 | 10912 | 6 | 0 | 6 |
| F5 | F5-2 | 7284036 | 75.80% | 5521299 | 69156 | 10947 | 5 | 0 | 5 |
| F5 | F5-20 | 7081685 | 37.03% | 2621994 | 16869 | 10910 | 6 | 0 | 6 |
| F5 | F5-22 | 9786598 | 34.59% | 3385184 | 20351 | 10910 | 10 | 0 | 10 |
| F5 | F5-23 | 7534652 | 46.48% | 3502106 | 20184 | 10910 | 4 | 0 | 4 |
| F5 | F5-25 | 10237300 | 57.98% | 5935075 | 36135 | 10912 | 7 | 0 | 7 |
| F5 | F5-26 | 11440231 | 49.42% | 5653190 | 26650 | 10912 | 6 | 0 | 6 |
| F5 | F5-28 | 8809332 | 41.39% | 3646183 | 25252 | 10910 | 8 | 0 | 8 |
| F5 | F5-30 | 5311522 | 47.94% | 2546344 | 17566 | 10908 | 5 | 0 | 5 |
| F5 | F5-4 | 10855097 | 34.41% | 3734696 | 19094 | 10910 | 6 | 0 | 6 |
| F5 | F5-5 | 8262143 | 54.19% | 4477255 | 26944 | 10921 | 9 | 0 | 9 |
| F5 | F5-7 | 9656886 | 19.96% | 1927514 | 9604 | 10907 | 7 | 0 | 7 |
| F5 | F5-8 | 10301439 | 22.89% | 2357999 | 14001 | 10909 | 6 | 0 | 6 |
| F10 | F10-1 | 4511715 | 56.46% | 2547089 | 17986 | 10939 | 20 | 0 | 20 |
| F10 | F10-10 | 6686793 | 63.20% | 4226053 | 32668 | 10910 | 14 | 0 | 14 |
| F10 | F10-11 | 6682364 | 37.61% | 2513237 | 17839 | 10907 | 16 | 0 | 16 |
| F10 | F10-13 | 8772278 | 63.71% | 5588818 | 83538 | 10938 | 19 | 0 | 19 |
| F10 | F10-14 | 9578186 | 76.92% | 7367062 | 122842 | 10934 | 12 | 0 | 12 |

Table S2: Sequencing data in C6/36 cells (Continued)

| Generation | Sample | Total reads | Percent of JEV | Total JEV reads | Mean site depth | Genome coverage (bp) | iSNV | SNP | Sum |
|---|---|---|---|---|---|---|---|---|---|
| F10 | F10-16 | 4985398 | 48.70% | 2427640 | 19734 | 10937 | 12 | 0 | 12 |
| F10 | F10-17 | 8322451 | 61.75% | 5138697 | 72519 | 10933 | 17 | 0 | 17 |
| F10 | F10-19 | 8469184 | 62.58% | 5299592 | 62190 | 10930 | 15 | 0 | 15 |
| F10 | F10-2 | 6802054 | 62.12% | 4225436 | 29358 | 10908 | 5 | 0 | 5 |
| F10 | F10-20 | 9379075 | 64.98% | 6094523 | 100005 | 10940 | 16 | 0 | 16 |
| F10 | F10-22 | 7621660 | 72.25% | 5506649 | 72772 | 10931 | 19 | 0 | 19 |
| F10 | F10-23 | 8008047 | 67.59% | 5412239 | 68373 | 10940 | 25 | 0 | 25 |
| F10 | F10-25 | 8783650 | 70.57% | 6198183 | 99768 | 10940 | 19 | 0 | 19 |
| F10 | F10-26 | 8629078 | 70.67% | 6098169 | 89501 | 10939 | 9 | 0 | 9 |
| F10 | F10-28 | 9179776 | 71.10% | 6526362 | 81142 | 10940 | 15 | 0 | 15 |
| F10 | F10-30 | 4006247 | 33.18% | 1329072 | 10170 | 10939 | 18 | 0 | 18 |
| F10 | F10-4 | 8683917 | 51.86% | 4503045 | 38275 | 10910 | 16 | 0 | 16 |
| F10 | F10-5 | 7720558 | 52.71% | 4069506 | 27369 | 10909 | 19 | 0 | 19 |
| F10 | F10-7 | 7740527 | 46.34% | 3586573 | 24064 | 10910 | 9 | 0 | 9 |
| F10 | F10-8 | 7373917 | 47.89% | 3531000 | 27055 | 10908 | 20 | 0 | 20 |
| F15 | F15-1 | 3294774 | 61.86% | 2037982 | 23228 | 10942 | 20 | 0 | 20 |
| F15 | F15-10 | 10698193 | 62.94% | 6732908 | 108730 | 10942 | 19 | 0 | 19 |
| F15 | F15-11 | 8261389 | 60.62% | 5008054 | 66509 | 10934 | 22 | 0 | 22 |
| F15 | F15-13 | 8622600 | 30.41% | 2621702 | 34137 | 10913 | 19 | 0 | 19 |
| F15 | F15-14 | 9295050 | 40.22% | 3738469 | 49906 | 10914 | 12 | 0 | 12 |
| F15 | F15-16 | 3981444 | 64.78% | 2578980 | 20559 | 10928 | 20 | 0 | 20 |
| F15 | F15-17 | 8610486 | 44.90% | 3865678 | 58935 | 10933 | 22 | 0 | 22 |
| F15 | F15-19 | 8121699 | 49.44% | 4015368 | 59278 | 10940 | 26 | 0 | 26 |
| F15 | F15-2 | 9849049 | 45.50% | 4480825 | 70915 | 10933 | 12 | 0 | 12 |
| F15 | F15-20 | 10061469 | 55.90% | 5624361 | 81944 | 10942 | 23 | 0 | 23 |
| F15 | F15-22 | 9579225 | 59.13% | 5663717 | 82255 | 10942 | 27 | 0 | 27 |
| F15 | F15-23 | 9122632 | 41.49% | 3784524 | 44792 | 10928 | 24 | 0 | 24 |
| F15 | F15-25 | 11097701 | 57.08% | 6334568 | 78720 | 10945 | 20 | 0 | 20 |
| F15 | F15-26 | 8101850 | 65.26% | 5286862 | 52633 | 10943 | 12 | 0 | 12 |
| F15 | F15-28 | 9332035 | 51.79% | 4832594 | 34686 | 10944 | 21 | 0 | 21 |
| F15 | F15-30 | 4359905 | 68.97% | 3007026 | 25329 | 10936 | 21 | 0 | 21 |
| F15 | F15-4 | 8123811 | 54.63% | 4437632 | 67704 | 10924 | 23 | 0 | 23 |
| F15 | F15-5 | 9543276 | 56.97% | 5436804 | 78503 | 10924 | 13 | 0 | 13 |
| F15 | F15-7 | 8774991 | 56.18% | 4929790 | 68175 | 10937 | 11 | 0 | 11 |
| F15 | F15-8 | 6705456 | 58.72% | 3937108 | 60305 | 10936 | 16 | 0 | 16 |
| F20 | F20-1 | 4394235 | 24.16% | 1061647 | 14588 | 10911 | 20 | 0 | 20 |
| F20 | F20-10 | 9548117 | 46.32% | 4422688 | 50525 | 10936 | 28 | 0 | 28 |
| F20 | F20-11 | 10880928 | 38.87% | 4229417 | 45402 | 10933 | 24 | 0 | 24 |
| F20 | F20-13 | 9634243 | 42.32% | 4077212 | 42396 | 10941 | 23 | 0 | 23 |
| F20 | F20-14 | 7652417 | 55.42% | 4240970 | 53369 | 10941 | 11 | 0 | 11 |
| F20 | F20-16 | 4090149 | 29.61% | 1210889 | 16024 | 10923 | 19 | 0 | 19 |
| F20 | F20-17 | 8167374 | 38.21% | 3120345 | 29038 | 10939 | 28 | 0 | 28 |
| F20 | F20-19 | 8563137 | 37.58% | 3217599 | 29222 | 10944 | 20 | 0 | 20 |
| F20 | F20-2 | 8542366 | 47.09% | 4022173 | 48778 | 10941 | 14 | 0 | 14 |
| F20 | F20-20 | 9787232 | 43.12% | 4219765 | 38858 | 10944 | 23 | 0 | 23 |
| F20 | F20-22 | 9293485 | 49.58% | 4607245 | 46166 | 10944 | 30 | 0 | 30 |
| F20 | F20-23 | 8264365 | 41.08% | 3395001 | 30905 | 10944 | 21 | 0 | 21 |
| F20 | F20-25 | 8376298 | 36.15% | 3027613 | 29116 | 10944 | 24 | 0 | 24 |
| F20 | F20-26 | 8865698 | 38.60% | 3422159 | 33193 | 10943 | 20 | 0 | 20 |
| F20 | F20-28 | 8049121 | 40.00% | 3219246 | 29989 | 10943 | 30 | 0 | 30 |
| F20 | F20-30 | 4687487 | 15.81% | 741092 | 7121 | 10909 | 17 | 0 | 17 |
| F20 | F20-4 | 9943592 | 55.85% | 5553496 | 64714 | 10933 | 18 | 0 | 18 |
| F20 | F20-5 | 8967320 | 50.93% | 4567056 | 45766 | 10941 | 12 | 0 | 12 |
| F20 | F20-7 | 9365486 | 54.62% | 5115428 | 53135 | 10941 | 16 | 1 | 17 |
| F20 | F20-8 | 9966281 | 49.64% | 4947262 | 55675 | 10942 | 17 | 0 | 17 |

Table S2: Sequencing data in C6/36 cells (Continued)

| Generation | Sample | Total reads | Percent of JEV | Total JEV reads | Mean site depth | Genome coverage (bp) | iSNV | SNP | Sum |
|---|---|---|---|---|---|---|---|---|---|
| F25 | F25-1 | 9584906 | 56.22% | 5388155 | 58549 | 10943 | 25 | 0 | 25 |
| F25 | F25-10 | 9444554 | 49.65% | 4688749 | 47347 | 10943 | 40 | 0 | 40 |
| F25 | F25-11 | 8996744 | 62.82% | 5651755 | 61885 | 10944 | 34 | 0 | 34 |
| F25 | F25-13 | 7927290 | 60.36% | 4784912 | 47353 | 10944 | 29 | 0 | 29 |
| F25 | F25-14 | 7879229 | 58.73% | 4627077 | 39455 | 10943 | 15 | 0 | 15 |
| F25 | F25-16 | 8652182 | 66.54% | 5757162 | 66725 | 10943 | 19 | 0 | 19 |
| F25 | F25-17 | 9499540 | 61.92% | 5881640 | 69813 | 10942 | 30 | 0 | 30 |
| F25 | F25-19 | 8255712 | 75.68% | 6247923 | 72346 | 10940 | 17 | 0 | 17 |
| F25 | F25-2 | 10192619 | 60.10% | 6125254 | 47884 | 10943 | 23 | 0 | 23 |
| F25 | F25-20 | 9179942 | 60.30% | 5535505 | 51126 | 10943 | 21 | 0 | 21 |
| F25 | F25-22 | 9928024 | 73.37% | 7284191 | 79319 | 10943 | 36 | 0 | 36 |
| F25 | F25-23 | 10811836 | 57.29% | 6193560 | 65904 | 10939 | 33 | 0 | 33 |
| F25 | F25-25 | 9334948 | 69.86% | 6520928 | 70928 | 10943 | 24 | 0 | 24 |
| F25 | F25-26 | 7841258 | 62.95% | 4935680 | 51691 | 10942 | 21 | 0 | 21 |
| F25 | F25-28 | 6945662 | 56.45% | 3920479 | 38653 | 10942 | 36 | 0 | 36 |
| F25 | F25-30 | 8105553 | 59.37% | 4812267 | 44930 | 10943 | 30 | 0 | 30 |
| F25 | F25-4 | 8742572 | 64.82% | 5666935 | 53690 | 10943 | 23 | 0 | 23 |
| F25 | F25-5 | 9007511 | 61.83% | 5569344 | 52983 | 10943 | 17 | 0 | 17 |
| F25 | F25-7 | 8348903 | 62.23% | 5195105 | 55846 | 10944 | 20 | 1 | 21 |
| F25 | F25-8 | 9260253 | 44.53% | 4123591 | 38217 | 10943 | 23 | 0 | 23 |
| F30 | F30-1 | 8941372 | 69.20% | 6186982 | 59656 | 10943 | 29 | 0 | 29 |
| F30 | F30-10 | 7012185 | 63.38% | 4444323 | 49651 | 10944 | 32 | 0 | 32 |
| F30 | F30-11 | 7231926 | 60.31% | 4361213 | 36146 | 10944 | 36 | 0 | 36 |
| F30 | F30-13 | 7521203 | 79.65% | 5990262 | 66098 | 10946 | 32 | 0 | 32 |
| F30 | F30-14 | 7655038 | 74.50% | 5702621 | 65094 | 10946 | 30 | 0 | 30 |
| F30 | F30-16 | 9430347 | 63.31% | 5969881 | 53429 | 10944 | 26 | 0 | 26 |
| F30 | F30-17 | 7859764 | 42.47% | 3338042 | 28892 | 10943 | 34 | 0 | 34 |
| F30 | F30-19 | 8263054 | 54.23% | 4480641 | 39831 | 10943 | 31 | 0 | 31 |
| F30 | F30-2 | 9455664 | 73.43% | 6943294 | 66237 | 10945 | 33 | 0 | 33 |
| F30 | F30-20 | 7325856 | 57.38% | 4203210 | 38142 | 10943 | 21 | 0 | 21 |
| F30 | F30-22 | 7074627 | 59.97% | 4242654 | 43075 | 10943 | 32 | 0 | 32 |
| F30 | F30-23 | 8173893 | 62.13% | 5078440 | 52501 | 10945 | 32 | 0 | 32 |
| F30 | F30-25 | 8088607 | 52.06% | 4210929 | 38347 | 10943 | 32 | 0 | 32 |
| F30 | F30-26 | 7442742 | 59.09% | 4397544 | 46409 | 10945 | 21 | 0 | 21 |
| F30 | F30-28 | 8672627 | 42.82% | 3713185 | 33214 | 10944 | 39 | 0 | 39 |
| F30 | F30-30 | 11133047 | 47.27% | 5262591 | 54747 | 10947 | 36 | 0 | 36 |
| F30 | F30-4 | 7354051 | 63.42% | 4663571 | 39436 | 10946 | 37 | 0 | 37 |
| F30 | F30-5 | 7581000 | 66.99% | 5078133 | 57877 | 10929 | 19 | 0 | 19 |
| F30 | F30-7 | 7321014 | 72.67% | 5320181 | 42587 | 10943 | 21 | 1 | 22 |
| F30 | F30-8 | 6598145 | 46.89% | 3093540 | 24297 | 10943 | 24 | 1 | 25 |
| F35 | F35-1 | 7758899 | 85.31% | 6618729 | 67942 | 10944 | 29 | 0 | 29 |
| F35 | F35-10 | 6876282 | 91.96% | 6323085 | 67286 | 10944 | 26 | 0 | 26 |
| F35 | F35-11 | 7292338 | 85.14% | 6208697 | 59663 | 10944 | 22 | 0 | 22 |
| F35 | F35-13 | 6574128 | 91.60% | 6021573 | 56638 | 10944 | 62 | 0 | 62 |
| F35 | F35-14 | 7724358 | 92.24% | 7124948 | 71481 | 10944 | 24 | 0 | 24 |
| F35 | F35-16 | 10763578 | 82.84% | 8916548 | 98306 | 10945 | 22 | 0 | 22 |
| F35 | F35-17 | 10029977 | 83.25% | 8349956 | 78601 | 10945 | 30 | 0 | 30 |
| F35 | F35-19 | 7201097 | 79.54% | 5727753 | 63600 | 10945 | 16 | 0 | 16 |
| F35 | F35-2 | 6234271 | 88.60% | 5523564 | 57045 | 10946 | 36 | 0 | 36 |
| F35 | F35-20 | 6805201 | 65.21% | 4437331 | 45886 | 10944 | 37 | 0 | 37 |
| F35 | F35-22 | 6796191 | 46.88% | 3185715 | 33260 | 10945 | 35 | 0 | 35 |
| F35 | F35-23 | 7907059 | 54.70% | 4324766 | 35942 | 10945 | 35 | 0 | 35 |
| F35 | F35-25 | 7908228 | 82.57% | 6529824 | 71993 | 10945 | 31 | 0 | 31 |
| F35 | F35-26 | 8262178 | 73.42% | 6066091 | 54415 | 10943 | 35 | 0 | 35 |
| F35 | F35-28 | 8486508 | 63.25% | 5367292 | 55854 | 10944 | 33 | 0 | 33 |

Table S2: Sequencing data in C6/36 cells (Continued)

| Generation | Sample | Total reads | Percent of JEV | Total JEV reads | Mean site depth | Genome coverage (bp) | iSNV | SNP | Sum |
|---|---|---|---|---|---|---|---|---|---|
| F35 | F35-30 | 7205698 | 81.77% | 5891739 | 58647 | 10942 | 41 | 0 | 41 |
| F35 | F35-4 | 7634764 | 89.43% | 6827769 | 69327 | 10946 | 42 | 0 | 42 |
| F35 | F35-5 | 8413448 | 90.30% | 7597344 | 70975 | 10944 | 35 | 0 | 35 |
| F35 | F35-7 | 6843977 | 90.66% | 6204750 | 67464 | 10944 | 22 | 1 | 23 |
| F35 | F35-8 | 7013046 | 92.65% | 6497587 | 67029 | 10944 | 35 | 0 | 35 |

Table S3: iSNV information in BHK cells

| Generation | Sample | Genome position | Gene | MuAF | Variation pattern | Type |
|---|---|---|---|---|---|---|
| F4 | F4 | 7633 | NS4B | 0.0301 | A:0;G:0;C:1255;T:39;total:1294 | iSNV |
| F4 | F4 | 10337 | NS5 | 0.0243 | A:0;G:0;C:963;T:24;total:987 | iSNV |
| F4 | F4 | 10724 | 3'-UTR | 0.0206 | A:0;G:0;C:17;T:807;total:824 | iSNV |
| F7 | F7 | 563 | M | 0.0578 | A:49;G:798;C:0;T:0;total:847 | iSNV |
| F7 | F7 | 766 | M | 0.0448 | A:1002;G:0;C:47;T:0;total:1049 | iSNV |
| F7 | F7 | 803 | M | 0.026 | A:0;G:749;C:0;T:20;total:769 | iSNV |
| F7 | F7 | 1708 | E | 0.0338 | A:26;G:742;C:0;T:0;total:768 | iSNV |
| F7 | F7 | 1772 | E | 0.0966 | A:0;G:91;C:0;T:851;total:942 | iSNV |
| F7 | F7 | 2372 | E | 0.129 | A:0;G:0;C:425;T:63;total:488 | iSNV |
| F7 | F7 | 2531 | NS1 | 0.0658 | A:0;G:0;C:51;T:724;total:775 | iSNV |
| F7 | F7 | 3204 | NS1 | 0.0201 | A:1795;G:37;C:0;T:0;total:1832 | iSNV |
| F7 | F7 | 3572 | NS1 | 0.0834 | A:0;G:0;C:593;T:54;total:647 | iSNV |
| F7 | F7 | 3962 | NS2A | 0.0683 | A:0;G:0;C:1118;T:82;total:1200 | iSNV |
| F7 | F7 | 4619 | NS3 | 0.0626 | A:0;G:0;C:79;T:1182;total:1261 | iSNV |
| F7 | F7 | 4712 | NS3 | 0.055 | A:60;G:1029;C:0;T:0;total:1089 | iSNV |
| F7 | F7 | 5311 | NS3 | 0.0577 | A:0;G:0;C:865;T:53;total:918 | iSNV |
| F7 | F7 | 5808 | NS3 | 0.0377 | A:49;G:1250;C:0;T:0;total:1299 | iSNV |
| F7 | F7 | 7633 | NS4B | 0.0817 | A:0;G:0;C:1089;T:97;total:1186 | iSNV |
| F7 | F7 | 7744 | NS5 | 0.0746 | A:1053;G:85;C:0;T:0;total:1138 | iSNV |
| F7 | F7 | 8282 | NS5 | 0.0706 | A:0;G:1;C:74;T:973;total:1048 | iSNV |
| F7 | F7 | 8900 | NS5 | 0.0869 | A:1123;G:1;C:107;T:0;total:1231 | iSNV |
| F7 | F7 | 9446 | NS5 | 0.0745 | A:1277;G:103;C:0;T:1;total:1381 | iSNV |
| F7 | F7 | 9584 | NS5 | 0.0203 | A:0;G:0;C:674;T:14;total:688 | iSNV |
| F7 | F7 | 9860 | NS5 | 0.0243 | A:0;G:0;C:18;T:721;total:739 | iSNV |
| F7 | F7 | 10419 | 3'-UTR | 0.0321 | A:0;G:0;C:663;T:22;total:685 | iSNV |
| F7 | F7 | 10428 | 3'-UTR | 0.0291 | A:0;G:0;C:699;T:21;total:720 | iSNV |
| F7 | F7 | 10447 | 3'-UTR | 0.0247 | A:0;G:0;C:630;T:16;total:646 | iSNV |
| F7 | F7 | 10494 | 3'-UTR | 0.0282 | A:689;G:20;C:0;T:0;total:709 | iSNV |
| F7 | F7 | 10801 | 3'-UTR | 0.0224 | A:0;G:0;C:479;T:11;total:490 | iSNV |
| F11 | F11 | 353 | C | 0.0336 | A:1262;G:44;C:0;T:0;total:1306 | iSNV |
| F11 | F11 | 563 | M | 0.3628 | A:295;G:518;C:0;T:0;total:813 | iSNV |
| F11 | F11 | 645 | M | 0.0341 | A:849;G:30;C:0;T:0;total:879 | iSNV |
| F11 | F11 | 803 | M | 0.3164 | A:0;G:486;C:0;T:225;total:711 | iSNV |
| F11 | F11 | 926 | M | 0.0752 | A:0;G:1;C:48;T:589;total:638 | iSNV |
| F11 | F11 | 998 | E | 0.0398 | A:0;G:0;C:675;T:28;total:703 | iSNV |
| F11 | F11 | 1772 | E | 0.3869 | A:0;G:344;C:0;T:545;total:889 | iSNV |
| F11 | F11 | 2011 | E | 0.0362 | A:0;G:0;C:20;T:532;total:552 | iSNV |
| F11 | F11 | 2372 | E | 0.4579 | A:0;G:0;C:245;T:207;total:452 | iSNV |
| F11 | F11 | 2531 | NS1 | 0.3594 | A:0;G:0;C:229;T:408;total:637 | iSNV |
| F11 | F11 | 2993 | NS1 | 0.1371 | A:761;G:121;C:0;T:0;total:882 | iSNV |
| F11 | F11 | 3515 | NS1 | 0.1283 | A:0;G:0;C:428;T:63;total:491 | iSNV |
| F11 | F11 | 3572 | NS1 | 0.3521 | A:0;G:0;C:368;T:200;total:568 | iSNV |
| F11 | F11 | 3698 | NS1 | 0.1015 | A:0;G:513;C:58;T:0;total:571 | iSNV |
| F11 | F11 | 3779 | NS2A | 0.0479 | A:0;G:0;C:37;T:734;total:771 | iSNV |
| F11 | F11 | 3962 | NS2A | 0.3761 | A:0;G:0;C:690;T:416;total:1106 | iSNV |
| F11 | F11 | 4094 | NS2A | 0.1224 | A:638;G:89;C:0;T:0;total:727 | iSNV |
| F11 | F11 | 4712 | NS3 | 0.3614 | A:381;G:673;C:0;T:0;total:1054 | iSNV |
| F11 | F11 | 5311 | NS3 | 0.3284 | A:0;G:0;C:597;T:292;total:889 | iSNV |
| F11 | F11 | 6314 | NS3 | 0.1117 | A:0;G:0;C:62;T:493;total:555 | iSNV |
| F11 | F11 | 7454 | NS4B | 0.0955 | A:445;G:0;C:0;T:47;total:492 | iSNV |
| F11 | F11 | 7576 | NS4B | 0.108 | A:0;G:0;C:685;T:83;total:768 | iSNV |
| F11 | F11 | 7735 | NS5 | 0.1839 | A:177;G:785;C:0;T:0;total:962 | iSNV |
| F11 | F11 | 7744 | NS5 | 0.3535 | A:609;G:333;C:0;T:0;total:942 | iSNV |
| F11 | F11 | 8282 | NS5 | 0.39 | A:0;G:0;C:376;T:588;total:964 | iSNV |
| F11 | F11 | 8288 | NS5 | 0.065 | A:0;G:0;C:63;T:906;total:969 | iSNV |
| F11 | F11 | 8518 | NS5 | 0.2788 | A:649;G:0;C:0;T:251;total:900 | iSNV |
| F11 | F11 | 8900 | NS5 | 0.4122 | A:606;G:0;C:425;T:0;total:1031 | iSNV |
| F11 | F11 | 9446 | NS5 | 0.3923 | A:697;G:450;C:0;T:0;total:1147 | iSNV |
| F11 | F11 | 9860 | NS5 | 0.1143 | A:0;G:0;C:74;T:573;total:647 | iSNV |
| F11 | F11 | 10259 | NS5 | 0.049 | A:776;G:40;C:0;T:0;total:816 | iSNV |
| F11 | F11 | 10447 | 3'-UTR | 0.0428 | A:0;G:0;C:559;T:25;total:584 | iSNV |
| F11 | F11 | 10574 | 3'-UTR | 0.1105 | A:0;G:0;C:539;T:67;total:606 | iSNV |
| F15 | F15 | 353 | C | 0.1128 | A:1218;G:155;C:0;T:0;total:1373 | iSNV |
| F15 | F15 | 563 | M | 0.2369 | A:227;G:731;C:0;T:0;total:958 | iSNV |
| F15 | F15 | 645 | M | 0.077 | A:934;G:78;C:0;T:0;total:1012 | iSNV |
| F15 | F15 | 803 | M | 0.4545 | A:0;G:432;C:0;T:360;total:792 | iSNV |
| F15 | F15 | 926 | M | 0.0429 | A:0;G:0;C:35;T:780;total:815 | iSNV |
| F15 | F15 | 998 | E | 0.1401 | A:0;G:0;C:724;T:118;total:842 | iSNV |
| F15 | F15 | 1772 | E | 0.2609 | A:0;G:287;C:0;T:813;total:1100 | iSNV |
| F15 | F15 | 2372 | E | 0.3368 | A:0;G:0;C:380;T:193;total:573 | iSNV |
| F15 | F15 | 2531 | NS1 | 0.2816 | A:0;G:0;C:225;T:574;total:799 | iSNV |
| F15 | F15 | 2993 | NS1 | 0.0234 | A:915;G:22;C:0;T:0;total:937 | iSNV |
| F15 | F15 | 3515 | NS1 | 0.0232 | A:0;G:0;C:630;T:15;total:645 | iSNV |
| F15 | F15 | 3572 | NS1 | 0.2425 | A:0;G:0;C:559;T:179;total:738 | iSNV |
| F15 | F15 | 3698 | NS1 | 0.0602 | A:0;G:624;C:40;T:0;total:664 | iSNV |
| F15 | F15 | 3779 | NS2A | 0.0403 | A:0;G:0;C:38;T:903;total:941 | iSNV |
| F15 | F15 | 3962 | NS2A | 0.2377 | A:0;G:0;C:1013;T:316;total:1329 | iSNV |
| F15 | F15 | 4094 | NS2A | 0.0847 | A:885;G:82;C:0;T:0;total:967 | iSNV |

| | | | | | | |
|---|---|---|---|---|---|---|
| F15 | F15 | 4697 | NS3 | 0.057 | A:0;G:0;C:68;T:1124;total:1192 | iSNV |
| F15 | F15 | 4712 | NS3 | 0.2573 | A:317;G:915;C:0;T:0;total:1232 | iSNV |
| F15 | F15 | 5311 | NS3 | 0.2652 | A:0;G:0;C:737;T:266;total:1003 | iSNV |
| F15 | F15 | 5952 | NS3 | 0.0678 | A:0;G:0;C:1471;T:107;total:1578 | iSNV |
| F15 | F15 | 6314 | NS3 | 0.067 | A:0;G:0;C:48;T:668;total:716 | iSNV |
| F15 | F15 | 7633 | NS4B | 0.0214 | A:0;G:0;C:1323;T:29;total:1352 | iSNV |
| F15 | F15 | 7735 | NS5 | 0.2532 | A:313;G:923;C:0;T:0;total:1236 | iSNV |
| F15 | F15 | 7744 | NS5 | 0.2696 | A:891;G:329;C:0;T:0;total:1220 | iSNV |
| F15 | F15 | 8282 | NS5 | 0.2936 | A:0;G:0;C:335;T:806;total:1141 | iSNV |
| F15 | F15 | 8288 | NS5 | 0.0381 | A:0;G:0;C:44;T:1110;total:1154 | iSNV |
| F15 | F15 | 8518 | NS5 | 0.4454 | A:590;G:0;C:0;T:474;total:1064 | iSNV |
| F15 | F15 | 8900 | NS5 | 0.2554 | A:965;G:0;C:331;T:0;total:1296 | iSNV |
| F15 | F15 | 9446 | NS5 | 0.2642 | A:1033;G:371;C:0;T:0;total:1404 | iSNV |
| F15 | F15 | 9860 | NS5 | 0.0932 | A:0;G:2;C:80;T:776;total:858 | iSNV |
| F15 | F15 | 10259 | NS5 | 0.1657 | A:891;G:177;C:0;T:0;total:1068 | iSNV |
| F15 | F15 | 10447 | 3'-UTR | 0.112 | A:0;G:0;C:642;T:81;total:723 | iSNV |
| F15 | F15 | 10511 | 3'-UTR | 0.0232 | A:0;G:0;C:19;T:798;total:817 | iSNV |
| F15 | F15 | 10566 | 3'-UTR | 0.0623 | A:0;G:0;C:677;T:45;total:722 | iSNV |
| F15 | F15 | 10574 | 3'-UTR | 0.0796 | A:0;G:0;C:670;T:58;total:728 | iSNV |
| F18 | F18 | 353 | C | 0.0954 | A:692;G:73;C:0;T:0;total:765 | iSNV |
| F18 | F18 | 563 | M | 0.116 | A:71;G:541;C:0;T:0;total:612 | iSNV |
| F18 | F18 | 645 | M | 0.1262 | A:533;G:77;C:0;T:0;total:610 | iSNV |
| F18 | F18 | 803 | M | 0.2665 | A:0;G:377;C:0;T:137;total:514 | iSNV |
| F18 | F18 | 998 | E | 0.3561 | A:0;G:0;C:311;T:172;total:483 | iSNV |
| F18 | F18 | 1218 | E | 0.1886 | A:0;G:0;C:585;T:136;total:721 | iSNV |
| F18 | F18 | 1772 | E | 0.1727 | A:0;G:119;C:1;T:569;total:689 | iSNV |
| F18 | F18 | 2372 | E | 0.1416 | A:0;G:0;C:315;T:52;total:367 | iSNV |
| F18 | F18 | 2531 | NS1 | 0.0956 | A:0;G:0;C:48;T:454;total:502 | iSNV |
| F18 | F18 | 3572 | NS1 | 0.1158 | A:0;G:0;C:458;T:60;total:518 | iSNV |
| F18 | F18 | 3779 | NS2A | 0.0344 | A:0;G:0;C:19;T:532;total:551 | iSNV |
| F18 | F18 | 3869 | NS2A | 0.2647 | A:0;G:0;C:650;T:234;total:884 | iSNV |
| F18 | F18 | 3962 | NS2A | 0.1109 | A:0;G:0;C:785;T:98;total:883 | iSNV |
| F18 | F18 | 4094 | NS2A | 0.0415 | A:576;G:25;C:0;T:0;total:601 | iSNV |
| F18 | F18 | 4697 | NS3 | 0.1323 | A:0;G:0;C:99;T:649;total:748 | iSNV |
| F18 | F18 | 4712 | NS3 | 0.1403 | A:111;G:680;C:0;T:0;total:791 | iSNV |
| F18 | F18 | 5311 | NS3 | 0.1 | A:0;G:0;C:504;T:56;total:560 | iSNV |
| F18 | F18 | 5952 | NS3 | 0.1075 | A:0;G:0;C:722;T:87;total:809 | iSNV |
| F18 | F18 | 7454 | NS4B | 0.0397 | A:387;G:0;C:0;T:16;total:403 | iSNV |
| F18 | F18 | 7735 | NS5 | 0.1831 | A:122;G:544;C:0;T:0;total:666 | iSNV |
| F18 | F18 | 7744 | NS5 | 0.1032 | A:582;G:67;C:0;T:0;total:649 | iSNV |
| F18 | F18 | 8168 | NS5 | 0.0216 | A:0;G:0;C:679;T:15;total:694 | iSNV |
| F18 | F18 | 8282 | NS5 | 0.1279 | A:0;G:0;C:91;T:620;total:711 | iSNV |
| F18 | F18 | 8518 | NS5 | 0.2985 | A:444;G:0;C:0;T:189;total:633 | iSNV |
| F18 | F18 | 8900 | NS5 | 0.1354 | A:498;G:0;C:78;T:0;total:576 | iSNV |
| F18 | F18 | 9446 | NS5 | 0.1284 | A:753;G:111;C:0;T:0;total:864 | iSNV |
| F18 | F18 | 9860 | NS5 | 0.057 | A:0;G:0;C:31;T:512;total:543 | iSNV |
| F18 | F18 | 10259 | NS5 | 0.2203 | A:499;G:141;C:0;T:0;total:640 | iSNV |
| F18 | F18 | 10447 | 3'-UTR | 0.1752 | A:0;G:0;C:306;T:65;total:371 | iSNV |
| F18 | F18 | 10566 | 3'-UTR | 0.1281 | A:0;G:0;C:381;T:56;total:437 | iSNV |
| F18 | F18 | 10574 | 3'-UTR | 0.0463 | A:0;G:0;C:432;T:21;total:453 | iSNV |
| F20 | F20 | 353 | C | 0.0688 | A:1460;G:108;C:0;T:0;total:1568 | iSNV |
| F20 | F20 | 563 | M | 0.0352 | A:41;G:1121;C:0;T:0;total:1162 | iSNV |
| F20 | F20 | 645 | M | 0.0802 | A:1135;G:99;C:0;T:0;total:1234 | iSNV |
| F20 | F20 | 803 | M | 0.1018 | A:0;G:970;C:0;T:110;total:1080 | iSNV |
| F20 | F20 | 998 | E | 0.1708 | A:0;G:0;C:854;T:176;total:1030 | iSNV |
| F20 | F20 | 1218 | E | 0.6796 | A:0;G:0;C:413;T:876;total:1289 | iSNV |
| F20 | F20 | 1430 | E | 0.0252 | A:0;G:0;C:1235;T:32;total:1267 | iSNV |
| F20 | F20 | 1772 | E | 0.0326 | A:0;G:43;C:0;T:1275;total:1318 | iSNV |
| F20 | F20 | 2372 | E | 0.0442 | A:0;G:0;C:712;T:33;total:745 | iSNV |
| F20 | F20 | 2531 | NS1 | 0.0275 | A:0;G:0;C:29;T:1023;total:1052 | iSNV |
| F20 | F20 | 3572 | NS1 | 0.023 | A:0;G:0;C:892;T:21;total:913 | iSNV |
| F20 | F20 | 3869 | NS2A | 0.6783 | A:0;G:0;C:510;T:1075;total:1585 | iSNV |
| F20 | F20 | 3962 | NS2A | 0.0381 | A:0;G:0;C:1512;T:60;total:1572 | iSNV |
| F20 | F20 | 4697 | NS3 | 0.1009 | A:1;G:0;C:156;T:1389;total:1546 | iSNV |
| F20 | F20 | 4712 | NS3 | 0.0432 | A:71;G:1571;C:0;T:0;total:1642 | iSNV |
| F20 | F20 | 5311 | NS3 | 0.0292 | A:0;G:0;C:1161;T:35;total:1196 | iSNV |
| F20 | F20 | 5952 | NS3 | 0.1049 | A:0;G:0;C:1526;T:179;total:1705 | iSNV |
| F20 | F20 | 7735 | NS5 | 0.0658 | A:83;G:1178;C:0;T:0;total:1261 | iSNV |
| F20 | F20 | 7744 | NS5 | 0.049 | A:1162;G:60;C:0;T:0;total:1222 | iSNV |
| F20 | F20 | 8282 | NS5 | 0.036 | A:0;G:0;C:49;T:1310;total:1359 | iSNV |
| F20 | F20 | 8518 | NS5 | 0.1082 | A:1186;G:0;C:0;T:144;total:1330 | iSNV |
| F20 | F20 | 8900 | NS5 | 0.0423 | A:1177;G:0;C:52;T:0;total:1229 | iSNV |
| F20 | F20 | 9359 | NS5 | 0.0342 | A:0;G:0;C:60;T:1692;total:1752 | iSNV |
| F20 | F20 | 9446 | NS5 | 0.0452 | A:1583;G:75;C:1;T:0;total:1659 | iSNV |
| F20 | F20 | 10259 | NS5 | 0.1703 | A:984;G:202;C:0;T:0;total:1186 | iSNV |
| F20 | F20 | 10447 | 3'-UTR | 0.1092 | A:0;G:0;C:685;T:84;total:769 | iSNV |
| F20 | F20 | 10566 | 3'-UTR | 0.1001 | A:1;G:0;C:772;T:86;total:859 | iSNV |
| F21 | F21-1 | 353 | C | 0.0666 | A:1092;G:78;C:0;T:0;total:1170 | iSNV |
| F21 | F21-1 | 563 | M | 0.0392 | A:35;G:857;C:0;T:0;total:892 | iSNV |
| F21 | F21-1 | 645 | M | 0.0665 | A:897;G:64;C:0;T:0;total:961 | iSNV |
| F21 | F21-1 | 803 | M | 0.0783 | A:0;G:835;C:0;T:71;total:906 | iSNV |
| F21 | F21-1 | 998 | E | 0.1692 | A:0;G:0;C:697;T:142;total:839 | iSNV |

| | | | | | | |
|---|---|---|---|---|---|---|
| F21 | F21-1 | 1218 | E | 0.7503 | A:0;G:0;C:257;T:772;total:1029 | iSNV |
| F21 | F21-1 | 1430 | E | 0.0265 | A:0;G:0;C:915;T:25;total:940 | iSNV |
| F21 | F21-1 | 1772 | E | 0.0267 | A:0;G:28;C:4;T:1014;total:1046 | iSNV |
| F21 | F21-1 | 2372 | E | 0.045 | A:0;G:0;C:509;T:24;total:533 | iSNV |
| F21 | F21-1 | 3869 | NS2A | 0.6787 | A:0;G:0;C:386;T:815;total:1201 | iSNV |
| F21 | F21-1 | 3962 | NS2A | 0.0415 | A:0;G:0;C:1130;T:49;total:1179 | iSNV |
| F21 | F21-1 | 4697 | NS3 | 0.1025 | A:0;G:0;C:111;T:971;total:1082 | iSNV |
| F21 | F21-1 | 4712 | NS3 | 0.0258 | A:28;G:1057;C:0;T:0;total:1085 | iSNV |
| F21 | F21-1 | 5311 | NS3 | 0.0225 | A:0;G:0;C:996;T:23;total:1019 | iSNV |
| F21 | F21-1 | 5952 | NS3 | 0.0941 | A:0;G:0;C:1212;T:126;total:1338 | iSNV |
| F21 | F21-1 | 7735 | NS5 | 0.0429 | A:47;G:1047;C:0;T:0;total:1094 | iSNV |
| F21 | F21-1 | 7744 | NS5 | 0.0362 | A:1009;G:38;C:0;T:0;total:1047 | iSNV |
| F21 | F21-1 | 8282 | NS5 | 0.0251 | A:0;G:0;C:25;T:969;total:994 | iSNV |
| F21 | F21-1 | 8518 | NS5 | 0.0716 | A:920;G:0;C:0;T:71;total:991 | iSNV |
| F21 | F21-1 | 8900 | NS5 | 0.0392 | A:881;G:0;C:36;T:0;total:917 | iSNV |
| F21 | F21-1 | 9359 | NS5 | 0.065 | A:0;G:0;C:87;T:1251;total:1338 | iSNV |
| F21 | F21-1 | 9446 | NS5 | 0.0267 | A:1235;G:34;C:0;T:0;total:1269 | iSNV |
| F21 | F21-1 | 10259 | NS5 | 0.1742 | A:749;G:158;C:0;T:0;total:907 | iSNV |
| F21 | F21-1 | 10447 | 3'-UTR | 0.097 | A:0;G:0;C:521;T:56;total:577 | iSNV |
| F21 | F21-1 | 10566 | 3'-UTR | 0.0955 | A:0;G:0;C:596;T:63;total:659 | iSNV |
| F21 | F21-10 | 353 | C | 0.0522 | A:581;G:32;C:0;T:0;total:613 | iSNV |
| F21 | F21-10 | 645 | M | 0.0678 | A:467;G:34;C:0;T:0;total:501 | iSNV |
| F21 | F21-10 | 803 | M | 0.1045 | A:0;G:197;C:0;T:23;total:220 | iSNV |
| F21 | F21-10 | 998 | E | 0.1297 | A:0;G:0;C:208;T:31;total:239 | iSNV |
| F21 | F21-10 | 1218 | E | 0.7433 | A:0;G:0;C:76;T:220;total:296 | iSNV |
| F21 | F21-10 | 1430 | E | 0.0352 | A:0;G:0;C:274;T:10;total:284 | iSNV |
| F21 | F21-10 | 3869 | NS2A | 0.7514 | A:0;G:0;C:95;T:287;total:382 | iSNV |
| F21 | F21-10 | 4697 | NS3 | 0.1202 | A:0;G:0;C:38;T:278;total:316 | iSNV |
| F21 | F21-10 | 5952 | NS3 | 0.0972 | A:0;G:0;C:501;T:54;total:555 | iSNV |
| F21 | F21-10 | 7735 | NS5 | 0.0391 | A:20;G:491;C:0;T:0;total:511 | iSNV |
| F21 | F21-10 | 9359 | NS5 | 0.0628 | A:0;G:0;C:51;T:760;total:811 | iSNV |
| F21 | F21-10 | 10259 | NS5 | 0.1666 | A:395;G:79;C:0;T:0;total:474 | iSNV |
| F21 | F21-10 | 10447 | 3'-UTR | 0.0965 | A:0;G:0;C:234;T:25;total:259 | iSNV |
| F21 | F21-10 | 10566 | 3'-UTR | 0.0783 | A:0;G:1;C:199;T:17;total:217 | iSNV |
| F21 | F21-11 | 645 | M | 0.1044 | A:60;G:7;C:0;T:0;total:67 | iSNV |
| F21 | F21-11 | 803 | M | 0.0943 | A:0;G:48;C:0;T:5;total:53 | iSNV |
| F21 | F21-11 | 998 | E | 0.1379 | A:0;G:0;C:50;T:8;total:58 | iSNV |
| F21 | F21-11 | 1218 | E | 0.6957 | A:0;G:0;C:21;T:48;total:69 | iSNV |
| F21 | F21-11 | 3869 | NS2A | 0.7143 | A:0;G:0;C:24;T:60;total:84 | iSNV |
| F21 | F21-11 | 5952 | NS3 | 0.086 | A:0;G:0;C:85;T:8;total:93 | iSNV |
| F21 | F21-11 | 8518 | NS5 | 0.1428 | A:48;G:0;C:0;T:8;total:56 | iSNV |
| F21 | F21-11 | 9359 | NS5 | 0.0617 | A:0;G:0;C:5;T:76;total:81 | iSNV |
| F21 | F21-11 | 10259 | NS5 | 0.2089 | A:53;G:14;C:0;T:0;total:67 | iSNV |
| F21 | F21-11 | 10566 | 3'-UTR | 0.12 | A:0;G:0;C:44;T:6;total:50 | iSNV |
| F21 | F21-12 | 353 | C | 0.0661 | A:974;G:69;C:0;T:0;total:1043 | iSNV |
| F21 | F21-12 | 645 | M | 0.0524 | A:651;G:36;C:0;T:0;total:687 | iSNV |
| F21 | F21-12 | 803 | M | 0.0673 | A:0;G:263;C:0;T:19;total:282 | iSNV |
| F21 | F21-12 | 998 | E | 0.2264 | A:0;G:0;C:263;T:77;total:340 | iSNV |
| F21 | F21-12 | 1218 | E | 0.7793 | A:0;G:0;C:85;T:300;total:385 | iSNV |
| F21 | F21-12 | 1430 | E | 0.0232 | A:0;G:0;C:336;T:8;total:344 | iSNV |
| F21 | F21-12 | 3869 | NS2A | 0.6834 | A:0;G:0;C:165;T:356;total:521 | iSNV |
| F21 | F21-12 | 4697 | NS3 | 0.0726 | A:0;G:0;C:33;T:421;total:454 | iSNV |
| F21 | F21-12 | 5952 | NS3 | 0.1085 | A:0;G:0;C:706;T:86;total:792 | iSNV |
| F21 | F21-12 | 7735 | NS5 | 0.0452 | A:34;G:717;C:0;T:0;total:751 | iSNV |
| F21 | F21-12 | 8518 | NS5 | 0.0726 | A:319;G:0;C:0;T:25;total:344 | iSNV |
| F21 | F21-12 | 9242 | NS5 | 0.0233 | A:0;G:18;C:0;T:752;total:770 | iSNV |
| F21 | F21-12 | 9359 | NS5 | 0.0678 | A:0;G:0;C:86;T:1181;total:1267 | iSNV |
| F21 | F21-12 | 10259 | NS5 | 0.1944 | A:547;G:132;C:0;T:0;total:679 | iSNV |
| F21 | F21-12 | 10428 | 3'-UTR | 0.024 | A:0;G:0;C:487;T:12;total:499 | iSNV |
| F21 | F21-12 | 10447 | 3'-UTR | 0.0995 | A:0;G:0;C:380;T:42;total:422 | iSNV |
| F21 | F21-12 | 10566 | 3'-UTR | 0.0769 | A:0;G:0;C:288;T:24;total:312 | iSNV |
| F21 | F21-13 | 353 | C | 0.0676 | A:1625;G:118;C:0;T:0;total:1743 | iSNV |
| F21 | F21-13 | 645 | M | 0.0496 | A:1206;G:63;C:0;T:0;total:1269 | iSNV |
| F21 | F21-13 | 803 | M | 0.066 | A:0;G:608;C:0;T:43;total:651 | iSNV |
| F21 | F21-13 | 998 | E | 0.2145 | A:0;G:0;C:509;T:139;total:648 | iSNV |
| F21 | F21-13 | 1218 | E | 0.7391 | A:0;G:0;C:215;T:609;total:824 | iSNV |
| F21 | F21-13 | 1413 | E | 0.0209 | A:842;G:18;C:0;T:0;total:860 | iSNV |
| F21 | F21-13 | 1772 | E | 0.0204 | A:0;G:15;C:0;T:720;total:735 | iSNV |
| F21 | F21-13 | 2372 | E | 0.02 | A:0;G:0;C:294;T:6;total:300 | iSNV |
| F21 | F21-13 | 3869 | NS2A | 0.7557 | A:0;G:0;C:237;T:733;total:970 | iSNV |
| F21 | F21-13 | 3962 | NS2A | 0.021 | A:0;G:0;C:789;T:17;total:806 | iSNV |
| F21 | F21-13 | 4697 | NS3 | 0.106 | A:0;G:0;C:98;T:826;total:924 | iSNV |
| F21 | F21-13 | 5311 | NS3 | 0.0307 | A:0;G:0;C:820;T:26;total:846 | iSNV |
| F21 | F21-13 | 5952 | NS3 | 0.1247 | A:0;G:0;C:1165;T:166;total:1331 | iSNV |
| F21 | F21-13 | 7735 | NS5 | 0.0437 | A:56;G:1225;C:0;T:0;total:1281 | iSNV |
| F21 | F21-13 | 7744 | NS5 | 0.023 | A:1185;G:28;C:0;T:0;total:1213 | iSNV |
| F21 | F21-13 | 9359 | NS5 | 0.0299 | A:0;G:0;C:59;T:1911;total:1970 | iSNV |
| F21 | F21-13 | 9446 | NS5 | 0.0211 | A:1617;G:35;C:0;T:0;total:1652 | iSNV |
| F21 | F21-13 | 10259 | NS5 | 0.1918 | A:1032;G:245;C:0;T:0;total:1277 | iSNV |
| F21 | F21-13 | 10376 | NS5 | 0.02 | A:24;G:1176;C:0;T:0;total:1200 | iSNV |
| F21 | F21-13 | 10419 | 3'-UTR | 0.0291 | A:0;G:1;C:864;T:26;total:891 | iSNV |
| F21 | F21-13 | 10447 | 3'-UTR | 0.1036 | A:0;G:0;C:692;T:80;total:772 | iSNV |

| | | | | | | |
|---|---|---|---|---|---|---|
| F21 | F21-13 | 10566 | 3'-UTR | 0.1015 | A:0;G:0;C:566;T:64;total:630 | iSNV |
| F21 | F21-14 | 353 | C | 0.0654 | A:100;G:7;C:0;T:0;total:107 | iSNV |
| F21 | F21-14 | 803 | M | 0.1136 | A:0;G:39;C:0;T:5;total:44 | iSNV |
| F21 | F21-14 | 998 | E | 0.1666 | A:0;G:0;C:60;T:12;total:72 | iSNV |
| F21 | F21-14 | 1218 | E | 0.744 | A:0;G:0;C:21;T:61;total:82 | iSNV |
| F21 | F21-14 | 4697 | NS3 | 0.1232 | A:0;G:0;C:9;T:64;total:73 | iSNV |
| F21 | F21-14 | 5952 | NS3 | 0.1 | A:0;G:0;C:99;T:11;total:110 | iSNV |
| F21 | F21-14 | 7735 | NS5 | 0.0875 | A:7;G:73;C:0;T:0;total:80 | iSNV |
| F21 | F21-14 | 9359 | NS5 | 0.0833 | A:0;G:0;C:13;T:143;total:156 | iSNV |
| F21 | F21-14 | 10259 | NS5 | 0.1075 | A:83;G:10;C:0;T:0;total:93 | iSNV |
| F21 | F21-14 | 10447 | 3'-UTR | 0.0909 | A:0;G:0;C:60;T:6;total:66 | iSNV |
| F21 | F21-14 | 10566 | 3'-UTR | 0.1081 | A:0;G:0;C:66;T:8;total:74 | iSNV |
| F21 | F21-15 | 353 | C | 0.05 | A:1555;G:82;C:0;T:0;total:1637 | iSNV |
| F21 | F21-15 | 563 | M | 0.0273 | A:28;G:996;C:0;T:0;total:1024 | iSNV |
| F21 | F21-15 | 645 | M | 0.0372 | A:1265;G:49;C:0;T:0;total:1314 | iSNV |
| F21 | F21-15 | 803 | M | 0.0831 | A:0;G:860;C:0;T:78;total:938 | iSNV |
| F21 | F21-15 | 998 | E | 0.1295 | A:0;G:0;C:692;T:103;total:795 | iSNV |
| F21 | F21-15 | 1218 | E | 0.7957 | A:0;G:0;C:233;T:907;total:1140 | iSNV |
| F21 | F21-15 | 2372 | E | 0.0355 | A:0;G:0;C:489;T:18;total:507 | iSNV |
| F21 | F21-15 | 3572 | NS1 | 0.02 | A:0;G:1;C:684;T:14;total:699 | iSNV |
| F21 | F21-15 | 3869 | NS2A | 0.7781 | A:0;G:0;C:273;T:957;total:1230 | iSNV |
| F21 | F21-15 | 3962 | NS2A | 0.028 | A:0;G:0;C:1144;T:33;total:1177 | iSNV |
| F21 | F21-15 | 4697 | NS3 | 0.0721 | A:0;G:0;C:83;T:1068;total:1151 | iSNV |
| F21 | F21-15 | 4712 | NS3 | 0.0257 | A:32;G:1209;C:0;T:0;total:1241 | iSNV |
| F21 | F21-15 | 5952 | NS3 | 0.0644 | A:0;G:0;C:1611;T:111;total:1722 | iSNV |
| F21 | F21-15 | 7735 | NS5 | 0.0599 | A:80;G:1253;C:0;T:1;total:1334 | iSNV |
| F21 | F21-15 | 8518 | NS5 | 0.0691 | A:1171;G:0;C:0;T:87;total:1258 | iSNV |
| F21 | F21-15 | 9359 | NS5 | 0.0396 | A:0;G:0;C:70;T:1696;total:1766 | iSNV |
| F21 | F21-15 | 9491 | NS5 | 0.0283 | A:924;G:27;C:0;T:0;total:951 | iSNV |
| F21 | F21-15 | 10259 | NS5 | 0.1153 | A:1219;G:159;C:0;T:0;total:1378 | iSNV |
| F21 | F21-15 | 10447 | 3'-UTR | 0.0645 | A:0;G:0;C:841;T:58;total:899 | iSNV |
| F21 | F21-15 | 10566 | 3'-UTR | 0.0617 | A:0;G:0;C:623;T:41;total:664 | iSNV |
| F21 | F21-16 | 353 | C | 0.0791 | A:849;G:73;C:0;T:0;total:922 | iSNV |
| F21 | F21-16 | 645 | M | 0.0724 | A:717;G:56;C:0;T:0;total:773 | iSNV |
| F21 | F21-16 | 803 | M | 0.0775 | A:0;G:702;C:0;T:59;total:761 | iSNV |
| F21 | F21-16 | 998 | E | 0.1643 | A:0;G:0;C:671;T:132;total:803 | iSNV |
| F21 | F21-16 | 1218 | E | 0.7536 | A:0;G:0;C:210;T:642;total:852 | iSNV |
| F21 | F21-16 | 1430 | E | 0.0286 | A:0;G:0;C:849;T:25;total:874 | iSNV |
| F21 | F21-16 | 1772 | E | 0.0205 | A:0;G:20;C:0;T:953;total:973 | iSNV |
| F21 | F21-16 | 2372 | E | 0.0243 | A:0;G:0;C:600;T:15;total:615 | iSNV |
| F21 | F21-16 | 2531 | NS1 | 0.027 | A:0;G:0;C:18;T:648;total:666 | iSNV |
| F21 | F21-16 | 3572 | NS1 | 0.0217 | A:0;G:0;C:719;T:16;total:735 | iSNV |
| F21 | F21-16 | 3869 | NS2A | 0.7394 | A:0;G:0;C:250;T:709;total:959 | iSNV |
| F21 | F21-16 | 3962 | NS2A | 0.0308 | A:0;G:0;C:1005;T:32;total:1037 | iSNV |
| F21 | F21-16 | 4697 | NS3 | 0.0807 | A:0;G:0;C:79;T:899;total:978 | iSNV |
| F21 | F21-16 | 4712 | NS3 | 0.0269 | A:26;G:940;C:0;T:0;total:966 | iSNV |
| F21 | F21-16 | 5952 | NS3 | 0.0691 | A:0;G:0;C:1036;T:77;total:1113 | iSNV |
| F21 | F21-16 | 7454 | NS4B | 0.0219 | A:490;G:0;C:0;T:11;total:501 | iSNV |
| F21 | F21-16 | 7735 | NS5 | 0.0536 | A:48;G:847;C:0;T:0;total:895 | iSNV |
| F21 | F21-16 | 7744 | NS5 | 0.032 | A:845;G:28;C:0;T:0;total:873 | iSNV |
| F21 | F21-16 | 8282 | NS5 | 0.0293 | A:0;G:0;C:25;T:826;total:851 | iSNV |
| F21 | F21-16 | 8518 | NS5 | 0.0647 | A:924;G:0;C:0;T:64;total:988 | iSNV |
| F21 | F21-16 | 8900 | NS5 | 0.0245 | A:716;G:0;C:18;T:0;total:734 | iSNV |
| F21 | F21-16 | 9359 | NS5 | 0.0376 | A:0;G:0;C:41;T:1048;total:1089 | iSNV |
| F21 | F21-16 | 9818 | NS5 | 0.0319 | A:0;G:0;C:20;T:606;total:626 | iSNV |
| F21 | F21-16 | 10259 | NS5 | 0.1504 | A:672;G:119;C:0;T:0;total:791 | iSNV |
| F21 | F21-16 | 10447 | 3'-UTR | 0.1013 | A:0;G:0;C:408;T:46;total:454 | iSNV |
| F21 | F21-16 | 10566 | 3'-UTR | 0.1077 | A:0;G:0;C:530;T:64;total:594 | iSNV |
| F21 | F21-17 | 353 | C | 0.0974 | A:278;G:30;C:0;T:0;total:308 | iSNV |
| F21 | F21-17 | 645 | M | 0.0756 | A:232;G:19;C:0;T:0;total:251 | iSNV |
| F21 | F21-17 | 803 | M | 0.1333 | A:0;G:104;C:0;T:16;total:120 | iSNV |
| F21 | F21-17 | 998 | E | 0.1554 | A:0;G:0;C:125;T:23;total:148 | iSNV |
| F21 | F21-17 | 1218 | E | 0.7312 | A:0;G:0;C:50;T:136;total:186 | iSNV |
| F21 | F21-17 | 2372 | E | 0.0645 | A:0;G:0;C:87;T:6;total:93 | iSNV |
| F21 | F21-17 | 3869 | NS2A | 0.6644 | A:0;G:0;C:95;T:188;total:283 | iSNV |
| F21 | F21-17 | 4619 | NS3 | 0.0222 | A:0;G:0;C:6;T:264;total:270 | iSNV |
| F21 | F21-17 | 4697 | NS3 | 0.0738 | A:0;G:0;C:13;T:163;total:176 | iSNV |
| F21 | F21-17 | 5952 | NS3 | 0.0769 | A:0;G:0;C:204;T:17;total:221 | iSNV |
| F21 | F21-17 | 7735 | NS5 | 0.0303 | A:5;G:160;C:0;T:0;total:165 | iSNV |
| F21 | F21-17 | 9359 | NS5 | 0.0687 | A:0;G:0;C:18;T:244;total:262 | iSNV |
| F21 | F21-17 | 10259 | NS5 | 0.1447 | A:195;G:33;C:0;T:0;total:228 | iSNV |
| F21 | F21-17 | 10566 | 3'-UTR | 0.125 | A:0;G:0;C:168;T:24;total:192 | iSNV |
| F21 | F21-18 | 353 | C | 0.0576 | A:1423;G:87;C:0;T:0;total:1510 | iSNV |
| F21 | F21-18 | 563 | M | 0.0278 | A:25;G:874;C:0;T:0;total:899 | iSNV |
| F21 | F21-18 | 645 | M | 0.054 | A:997;G:57;C:0;T:0;total:1054 | iSNV |
| F21 | F21-18 | 803 | M | 0.0774 | A:0;G:727;C:0;T:61;total:788 | iSNV |
| F21 | F21-18 | 998 | E | 0.1614 | A:0;G:0;C:618;T:119;total:737 | iSNV |
| F21 | F21-18 | 1218 | E | 0.7729 | A:0;G:0;C:224;T:762;total:986 | iSNV |
| F21 | F21-18 | 1430 | E | 0.038 | A:0;G:0;C:834;T:33;total:867 | iSNV |
| F21 | F21-18 | 1772 | E | 0.0234 | A:0;G:22;C:0;T:915;total:937 | iSNV |
| F21 | F21-18 | 2372 | E | 0.0216 | A:0;G:0;C:362;T:8;total:370 | iSNV |
| F21 | F21-18 | 3869 | NS2A | 0.6983 | A:0;G:0;C:353;T:817;total:1170 | iSNV |

| | | | | | | |
|---|---|---|---|---|---|---|
| F21 | F21-18 | 4697 | NS3 | 0.1025 | A:0;G:0;C:94;T:823;total:917 | iSNV |
| F21 | F21-18 | 5952 | NS3 | 0.1016 | A:0;G:0;C:1335;T:151;total:1486 | iSNV |
| F21 | F21-18 | 7735 | NS5 | 0.0629 | A:73;G:1081;C:3;T:2;total:1159 | iSNV |
| F21 | F21-18 | 8518 | NS5 | 0.0774 | A:869;G:0;C:0;T:73;total:942 | iSNV |
| F21 | F21-18 | 9359 | NS5 | 0.071 | A:0;G:0;C:110;T:1438;total:1548 | iSNV |
| F21 | F21-18 | 9446 | NS5 | 0.0206 | A:1470;G:31;C:0;T:0;total:1501 | iSNV |
| F21 | F21-18 | 9860 | NS5 | 0.0222 | A:0;G:0;C:15;T:660;total:675 | iSNV |
| F21 | F21-18 | 10259 | NS5 | 0.1724 | A:926;G:193;C:0;T:0;total:1119 | iSNV |
| F21 | F21-18 | 10433 | 3'-UTR | 0.0216 | A:0;G:0;C:16;T:722;total:738 | iSNV |
| F21 | F21-18 | 10447 | 3'-UTR | 0.07 | A:0;G:0;C:637;T:48;total:685 | iSNV |
| F21 | F21-18 | 10566 | 3'-UTR | 0.1057 | A:0;G:0;C:533;T:63;total:596 | iSNV |
| F21 | F21-19 | 353 | C | 0.0947 | A:994;G:104;C:0;T:0;total:1098 | iSNV |
| F21 | F21-19 | 645 | M | 0.1127 | A:771;G:98;C:0;T:0;total:869 | iSNV |
| F21 | F21-19 | 803 | M | 0.0776 | A:0;G:463;C:0;T:39;total:502 | iSNV |
| F21 | F21-19 | 998 | E | 0.2166 | A:0;G:0;C:405;T:112;total:517 | iSNV |
| F21 | F21-19 | 1218 | E | 0.6876 | A:0;G:0;C:209;T:460;total:669 | iSNV |
| F21 | F21-19 | 1430 | E | 0.0296 | A:0;G:0;C:557;T:17;total:574 | iSNV |
| F21 | F21-19 | 1772 | E | 0.0326 | A:0;G:20;C:0;T:593;total:613 | iSNV |
| F21 | F21-19 | 3869 | NS2A | 0.6635 | A:0;G:0;C:279;T:550;total:829 | iSNV |
| F21 | F21-19 | 3962 | NS2A | 0.0221 | A:1;G:0;C:706;T:16;total:723 | iSNV |
| F21 | F21-19 | 4697 | NS3 | 0.0985 | A:0;G:0;C:67;T:613;total:680 | iSNV |
| F21 | F21-19 | 4712 | NS3 | 0.0227 | A:17;G:729;C:0;T:0;total:746 | iSNV |
| F21 | F21-19 | 5952 | NS3 | 0.0773 | A:0;G:0;C:930;T:78;total:1008 | iSNV |
| F21 | F21-19 | 7735 | NS5 | 0.0485 | A:38;G:744;C:0;T:0;total:782 | iSNV |
| F21 | F21-19 | 8168 | NS5 | 0.0335 | A:0;G:0;C:662;T:23;total:685 | iSNV |
| F21 | F21-19 | 8518 | NS5 | 0.0827 | A:621;G:0;C:0;T:56;total:677 | iSNV |
| F21 | F21-19 | 8900 | NS5 | 0.0247 | A:947;G:0;C:24;T:0;total:971 | iSNV |
| F21 | F21-19 | 9359 | NS5 | 0.0217 | A:0;G:0;C:27;T:1214;total:1241 | iSNV |
| F21 | F21-19 | 9446 | NS5 | 0.0219 | A:1071;G:24;C:0;T:0;total:1095 | iSNV |
| F21 | F21-19 | 9491 | NS5 | 0.0272 | A:500;G:14;C:0;T:0;total:514 | iSNV |
| F21 | F21-19 | 10259 | NS5 | 0.2378 | A:657;G:205;C:0;T:0;total:862 | iSNV |
| F21 | F21-19 | 10428 | 3'-UTR | 0.0344 | A:0;G:0;C:588;T:21;total:609 | iSNV |
| F21 | F21-19 | 10447 | 3'-UTR | 0.1651 | A:0;G:0;C:445;T:88;total:533 | iSNV |
| F21 | F21-19 | 10566 | 3'-UTR | 0.0799 | A:0;G:0;C:449;T:39;total:488 | iSNV |
| F21 | F21-2 | 353 | C | 0.0264 | A:184;G:5;C:0;T:0;total:189 | iSNV |
| F21 | F21-2 | 803 | M | 0.0373 | A:0;G:129;C:0;T:5;total:134 | iSNV |
| F21 | F21-2 | 998 | E | 0.1512 | A:0;G:0;C:101;T:18;total:119 | iSNV |
| F21 | F21-2 | 1218 | E | 0.7115 | A:0;G:0;C:43;T:106;total:149 | iSNV |
| F21 | F21-2 | 1430 | E | 0.0391 | A:0;G:0;C:172;T:7;total:179 | iSNV |
| F21 | F21-2 | 4697 | NS3 | 0.109 | A:0;G:0;C:24;T:196;total:220 | iSNV |
| F21 | F21-2 | 4712 | NS3 | 0.0211 | A:5;G:231;C:0;T:0;total:236 | iSNV |
| F21 | F21-2 | 5311 | NS3 | 0.0489 | A:0;G:0;C:136;T:7;total:143 | iSNV |
| F21 | F21-2 | 5952 | NS3 | 0.0815 | A:0;G:0;C:169;T:15;total:184 | iSNV |
| F21 | F21-2 | 8518 | NS5 | 0.0855 | A:139;G:0;C:0;T:13;total:152 | iSNV |
| F21 | F21-2 | 9359 | NS5 | 0.0496 | A:0;G:0;C:8;T:153;total:161 | iSNV |
| F21 | F21-2 | 10259 | NS5 | 0.0941 | A:154;G:16;C:0;T:0;total:170 | iSNV |
| F21 | F21-2 | 10447 | 3'-UTR | 0.0725 | A:0;G:0;C:115;T:9;total:124 | iSNV |
| F21 | F21-2 | 10566 | 3'-UTR | 0.0695 | A:0;G:0;C:107;T:8;total:115 | iSNV |
| F21 | F21-20 | 353 | C | 0.0632 | A:163;G:11;C:0;T:0;total:174 | iSNV |
| F21 | F21-20 | 803 | M | 0.0919 | A:0;G:79;C:0;T:8;total:87 | iSNV |
| F21 | F21-20 | 998 | E | 0.1829 | A:0;G:0;C:67;T:15;total:82 | iSNV |
| F21 | F21-20 | 1218 | E | 0.6973 | A:0;G:0;C:33;T:76;total:109 | iSNV |
| F21 | F21-20 | 3266 | NS1 | 0.0333 | A:145;G:5;C:0;T:0;total:150 | iSNV |
| F21 | F21-20 | 3869 | NS2A | 0.7056 | A:0;G:0;C:48;T:115;total:163 | iSNV |
| F21 | F21-20 | 3962 | NS2A | 0.0365 | A:0;G:0;C:158;T:6;total:164 | iSNV |
| F21 | F21-20 | 4697 | NS3 | 0.125 | A:0;G:0;C:17;T:119;total:136 | iSNV |
| F21 | F21-20 | 5952 | NS3 | 0.1329 | A:0;G:0;C:137;T:21;total:158 | iSNV |
| F21 | F21-20 | 7735 | NS5 | 0.0932 | A:11;G:107;C:0;T:0;total:118 | iSNV |
| F21 | F21-20 | 8518 | NS5 | 0.0786 | A:82;G:0;C:0;T:7;total:89 | iSNV |
| F21 | F21-20 | 10259 | NS5 | 0.18 | A:123;G:27;C:0;T:0;total:150 | iSNV |
| F21 | F21-20 | 10447 | 3'-UTR | 0.1153 | A:0;G:0;C:69;T:9;total:78 | iSNV |
| F21 | F21-21 | 353 | C | 0.0679 | A:398;G:29;C:0;T:0;total:427 | iSNV |
| F21 | F21-21 | 803 | M | 0.0669 | A:0;G:209;C:0;T:15;total:224 | iSNV |
| F21 | F21-21 | 998 | E | 0.1832 | A:0;G:0;C:156;T:35;total:191 | iSNV |
| F21 | F21-21 | 1218 | E | 0.7745 | A:0;G:0;C:83;T:285;total:368 | iSNV |
| F21 | F21-21 | 1430 | E | 0.0271 | A:0;G:0;C:251;T:7;total:258 | iSNV |
| F21 | F21-21 | 2531 | NS1 | 0.0255 | A:0;G:0;C:8;T:305;total:313 | iSNV |
| F21 | F21-21 | 3508 | NS1 | 0.0439 | A:0;G:0;C:174;T:8;total:182 | iSNV |
| F21 | F21-21 | 3869 | NS2A | 0.7666 | A:0;G:0;C:74;T:243;total:317 | iSNV |
| F21 | F21-21 | 4697 | NS3 | 0.0668 | A:0;G:0;C:21;T:293;total:314 | iSNV |
| F21 | F21-21 | 5952 | NS3 | 0.0845 | A:0;G:0;C:487;T:45;total:532 | iSNV |
| F21 | F21-21 | 7735 | NS5 | 0.0389 | A:14;G:345;C:0;T:0;total:359 | iSNV |
| F21 | F21-21 | 9359 | NS5 | 0.0378 | A:0;G:0;C:18;T:458;total:476 | iSNV |
| F21 | F21-21 | 9818 | NS5 | 0.0315 | A:0;G:0;C:9;T:276;total:285 | iSNV |
| F21 | F21-21 | 10259 | NS5 | 0.1876 | A:407;G:94;C:0;T:0;total:501 | iSNV |
| F21 | F21-21 | 10447 | 3'-UTR | 0.0847 | A:0;G:0;C:162;T:15;total:177 | iSNV |
| F21 | F21-21 | 10566 | 3'-UTR | 0.1244 | A:0;G:0;C:183;T:26;total:209 | iSNV |
| F21 | F21-22 | 353 | C | 0.0473 | A:1470;G:73;C:0;T:0;total:1543 | iSNV |
| F21 | F21-22 | 645 | M | 0.046 | A:1140;G:55;C:0;T:0;total:1195 | iSNV |
| F21 | F21-22 | 803 | M | 0.0725 | A:0;G:793;C:0;T:62;total:855 | iSNV |
| F21 | F21-22 | 998 | E | 0.1396 | A:0;G:0;C:690;T:112;total:802 | iSNV |
| F21 | F21-22 | 1218 | E | 0.8012 | A:0;G:0;C:209;T:842;total:1051 | iSNV |

| | | | | | | |
|---|---|---|---|---|---|---|
| F21 | F21-22 | 1430 | E | 0.0282 | A:0;G:0;C:929;T:27;total:956 | iSNV |
| F21 | F21-22 | 1772 | E | 0.0224 | A:0;G:23;C:0;T:1002;total:1025 | iSNV |
| F21 | F21-22 | 2372 | E | 0.0265 | A:0;G:0;C:404;T:11;total:415 | iSNV |
| F21 | F21-22 | 3869 | NS2A | 0.7512 | A:0;G:0;C:312;T:942;total:1254 | iSNV |
| F21 | F21-22 | 4697 | NS3 | 0.0877 | A:0;G:0;C:91;T:946;total:1037 | iSNV |
| F21 | F21-22 | 5150 | NS3 | 0.0246 | A:1028;G:0;C:0;T:26;total:1054 | iSNV |
| F21 | F21-22 | 5952 | NS3 | 0.078 | A:0;G:0;C:1418;T:120;total:1538 | iSNV |
| F21 | F21-22 | 7735 | NS5 | 0.0653 | A:83;G:1188;C:0;T:0;total:1271 | iSNV |
| F21 | F21-22 | 8518 | NS5 | 0.0826 | A:955;G:0;C:0;T:86;total:1041 | iSNV |
| F21 | F21-22 | 9359 | NS5 | 0.0377 | A:0;G:0;C:67;T:1706;total:1773 | iSNV |
| F21 | F21-22 | 9446 | NS5 | 0.026 | A:1570;G:42;C:0;T:0;total:1612 | iSNV |
| F21 | F21-22 | 10259 | NS5 | 0.1344 | A:1101;G:171;C:0;T:0;total:1272 | iSNV |
| F21 | F21-22 | 10447 | 3'-UTR | 0.0862 | A:1;G:0;C:741;T:70;total:812 | iSNV |
| F21 | F21-22 | 10566 | 3'-UTR | 0.0777 | A:0;G:0;C:569;T:48;total:617 | iSNV |
| F21 | F21-23 | 353 | C | 0.098 | A:92;G:10;C:0;T:0;total:102 | iSNV |
| F21 | F21-23 | 803 | M | 0.1276 | A:0;G:41;C:0;T:6;total:47 | iSNV |
| F21 | F21-23 | 998 | E | 0.0961 | A:0;G:0;C:47;T:5;total:52 | iSNV |
| F21 | F21-23 | 1218 | E | 0.72 | A:0;G:0;C:21;T:54;total:75 | iSNV |
| F21 | F21-23 | 3869 | NS2A | 0.8586 | A:0;G:0;C:14;T:85;total:99 | iSNV |
| F21 | F21-23 | 5952 | NS3 | 0.0588 | A:0;G:0;C:96;T:6;total:102 | iSNV |
| F21 | F21-23 | 10259 | NS5 | 0.1139 | A:70;G:9;C:0;T:0;total:79 | iSNV |
| F21 | F21-23 | 10447 | 3'-UTR | 0.1147 | A:0;G:0;C:54;T:7;total:61 | iSNV |
| F21 | F21-24 | 353 | C | 0.0421 | A:1590;G:70;C:0;T:0;total:1660 | iSNV |
| F21 | F21-24 | 645 | M | 0.051 | A:1170;G:63;C:0;T:0;total:1233 | iSNV |
| F21 | F21-24 | 803 | M | 0.0733 | A:0;G:834;C:0;T:66;total:900 | iSNV |
| F21 | F21-24 | 998 | E | 0.1696 | A:0;G:0;C:651;T:133;total:784 | iSNV |
| F21 | F21-24 | 1218 | E | 0.7268 | A:0;G:0;C:285;T:758;total:1043 | iSNV |
| F21 | F21-24 | 1430 | E | 0.0243 | A:0;G:0;C:1040;T:26;total:1066 | iSNV |
| F21 | F21-24 | 2057 | E | 0.0306 | A:0;G:0;C:601;T:19;total:620 | iSNV |
| F21 | F21-24 | 2372 | E | 0.0202 | A:0;G:0;C:436;T:9;total:445 | iSNV |
| F21 | F21-24 | 3257 | NS1 | 0.0256 | A:0;G:0;C:43;T:1635;total:1678 | iSNV |
| F21 | F21-24 | 3353 | NS1 | 0.0221 | A:0;G:0;C:969;T:22;total:991 | iSNV |
| F21 | F21-24 | 3572 | NS1 | 0.021 | A:0;G:0;C:652;T:14;total:666 | iSNV |
| F21 | F21-24 | 3869 | NS2A | 0.7045 | A:0;G:0;C:370;T:882;total:1252 | iSNV |
| F21 | F21-24 | 4697 | NS3 | 0.119 | A:0;G:0;C:129;T:955;total:1084 | iSNV |
| F21 | F21-24 | 5952 | NS3 | 0.1211 | A:0;G:0;C:1444;T:199;total:1643 | iSNV |
| F21 | F21-24 | 6881 | NS4A | 0.0212 | A:0;G:0;C:18;T:829;total:847 | iSNV |
| F21 | F21-24 | 7735 | NS5 | 0.039 | A:55;G:1353;C:0;T:1;total:1409 | iSNV |
| F21 | F21-24 | 8518 | NS5 | 0.076 | A:1021;G:0;C:0;T:84;total:1105 | iSNV |
| F21 | F21-24 | 9359 | NS5 | 0.0459 | A:0;G:0;C:83;T:1723;total:1806 | iSNV |
| F21 | F21-24 | 10259 | NS5 | 0.1866 | A:1133;G:260;C:0;T:0;total:1393 | iSNV |
| F21 | F21-24 | 10428 | 3'-UTR | 0.0218 | A:0;G:0;C:894;T:20;total:914 | iSNV |
| F21 | F21-24 | 10447 | 3'-UTR | 0.0852 | A:0;G:0;C:730;T:68;total:798 | iSNV |
| F21 | F21-24 | 10566 | 3'-UTR | 0.135 | A:0;G:0;C:634;T:99;total:733 | iSNV |
| F21 | F21-25 | 353 | C | 0.0626 | A:703;G:47;C:0;T:0;total:750 | iSNV |
| F21 | F21-25 | 563 | M | 0.0373 | A:15;G:387;C:0;T:0;total:402 | iSNV |
| F21 | F21-25 | 645 | M | 0.045 | A:487;G:23;C:0;T:0;total:510 | iSNV |
| F21 | F21-25 | 803 | M | 0.0757 | A:0;G:354;C:0;T:29;total:383 | iSNV |
| F21 | F21-25 | 998 | E | 0.153 | A:0;G:0;C:321;T:58;total:379 | iSNV |
| F21 | F21-25 | 1218 | E | 0.7098 | A:0;G:0;C:128;T:313;total:441 | iSNV |
| F21 | F21-25 | 3869 | NS2A | 0.7326 | A:0;G:0;C:169;T:463;total:632 | iSNV |
| F21 | F21-25 | 3962 | NS2A | 0.0353 | A:0;G:0;C:519;T:19;total:538 | iSNV |
| F21 | F21-25 | 4187 | NS2A | 0.0402 | A:191;G:8;C:0;T:0;total:199 | iSNV |
| F21 | F21-25 | 4697 | NS3 | 0.0721 | A:0;G:0;C:34;T:437;total:471 | iSNV |
| F21 | F21-25 | 4712 | NS3 | 0.027 | A:14;G:504;C:0;T:0;total:518 | iSNV |
| F21 | F21-25 | 5952 | NS3 | 0.0613 | A:0;G:0;C:688;T:45;total:733 | iSNV |
| F21 | F21-25 | 7735 | NS5 | 0.058 | A:39;G:633;C:0;T:0;total:672 | iSNV |
| F21 | F21-25 | 7744 | NS5 | 0.0229 | A:640;G:15;C:0;T:0;total:655 | iSNV |
| F21 | F21-25 | 8282 | NS5 | 0.023 | A:0;G:0;C:12;T:509;total:521 | iSNV |
| F21 | F21-25 | 8518 | NS5 | 0.0777 | A:522;G:0;C:0;T:44;total:566 | iSNV |
| F21 | F21-25 | 9446 | NS5 | 0.0345 | A:866;G:31;C:0;T:0;total:897 | iSNV |
| F21 | F21-25 | 9491 | NS5 | 0.0288 | A:437;G:13;C:0;T:0;total:450 | iSNV |
| F21 | F21-25 | 10259 | NS5 | 0.1513 | A:583;G:104;C:0;T:0;total:687 | iSNV |
| F21 | F21-25 | 10428 | 3'-UTR | 0.0373 | A:0;G:0;C:464;T:18;total:482 | iSNV |
| F21 | F21-25 | 10447 | 3'-UTR | 0.0722 | A:0;G:0;C:385;T:30;total:415 | iSNV |
| F21 | F21-25 | 10566 | 3'-UTR | 0.0738 | A:0;G:0;C:276;T:22;total:298 | iSNV |
| F21 | F21-26 | 998 | E | 0.2359 | A:0;G:0;C:68;T:21;total:89 | iSNV |
| F21 | F21-26 | 1218 | E | 0.7439 | A:0;G:0;C:31;T:90;total:121 | iSNV |
| F21 | F21-26 | 3869 | NS2A | 0.8049 | A:0;G:0;C:32;T:132;total:164 | iSNV |
| F21 | F21-26 | 4697 | NS3 | 0.0683 | A:0;G:0;C:8;T:109;total:117 | iSNV |
| F21 | F21-26 | 5311 | NS3 | 0.0393 | A:0;G:0;C:122;T:5;total:127 | iSNV |
| F21 | F21-26 | 5952 | NS3 | 0.0677 | A:0;G:0;C:165;T:12;total:177 | iSNV |
| F21 | F21-26 | 7735 | NS5 | 0.0686 | A:7;G:95;C:0;T:0;total:102 | iSNV |
| F21 | F21-26 | 9359 | NS5 | 0.0704 | A:0;G:0;C:10;T:132;total:142 | iSNV |
| F21 | F21-26 | 10259 | NS5 | 0.1526 | A:111;G:20;C:0;T:0;total:131 | iSNV |
| F21 | F21-26 | 10447 | 3'-UTR | 0.1868 | A:0;G:0;C:74;T:17;total:91 | iSNV |
| F21 | F21-26 | 10566 | 3'-UTR | 0.0714 | A:0;G:0;C:91;T:7;total:98 | iSNV |
| F21 | F21-27 | 353 | C | 0.0459 | A:1660;G:80;C:0;T:0;total:1740 | iSNV |
| F21 | F21-27 | 563 | M | 0.05 | A:57;G:1083;C:0;T:0;total:1140 | iSNV |
| F21 | F21-27 | 645 | M | 0.0377 | A:1325;G:52;C:0;T:0;total:1377 | iSNV |
| F21 | F21-27 | 803 | M | 0.0799 | A:1;G:920;C:0;T:80;total:1001 | iSNV |
| F21 | F21-27 | 998 | E | 0.1218 | A:0;G:0;C:843;T:117;total:960 | iSNV |

| | | | | | | |
|---|---|---|---|---|---|---|
| F21 | F21-27 | 1218 | E | 0.7226 | A:0;G:0;C:318;T:828;total:1146 | iSNV |
| F21 | F21-27 | 1430 | E | 0.0293 | A:0;G:1;C:1191;T:36;total:1228 | iSNV |
| F21 | F21-27 | 1772 | E | 0.0703 | A:0;G:89;C:1;T:1176;total:1266 | iSNV |
| F21 | F21-27 | 2372 | E | 0.1111 | A:1;G:0;C:479;T:60;total:540 | iSNV |
| F21 | F21-27 | 2531 | NS1 | 0.0574 | A:0;G:0;C:55;T:903;total:958 | iSNV |
| F21 | F21-27 | 3572 | NS1 | 0.093 | A:0;G:0;C:682;T:70;total:752 | iSNV |
| F21 | F21-27 | 3869 | NS2A | 0.756 | A:0;G:0;C:369;T:1143;total:1512 | iSNV |
| F21 | F21-27 | 3962 | NS2A | 0.0667 | A:2;G:0;C:1341;T:96;total:1439 | iSNV |
| F21 | F21-27 | 4697 | NS3 | 0.0947 | A:0;G:0;C:125;T:1194;total:1319 | iSNV |
| F21 | F21-27 | 4712 | NS3 | 0.0541 | A:76;G:1328;C:0;T:0;total:1404 | iSNV |
| F21 | F21-27 | 5311 | NS3 | 0.0669 | A:0;G:0;C:1198;T:86;total:1284 | iSNV |
| F21 | F21-27 | 5952 | NS3 | 0.0766 | A:0;G:1;C:1781;T:148;total:1930 | iSNV |
| F21 | F21-27 | 7735 | NS5 | 0.0685 | A:99;G:1346;C:0;T:0;total:1445 | iSNV |
| F21 | F21-27 | 7744 | NS5 | 0.0407 | A:1342;G:57;C:0;T:0;total:1399 | iSNV |
| F21 | F21-27 | 8282 | NS5 | 0.0591 | A:0;G:0;C:80;T:1272;total:1352 | iSNV |
| F21 | F21-27 | 8518 | NS5 | 0.0839 | A:1201;G:0;C:0;T:110;total:1311 | iSNV |
| F21 | F21-27 | 8900 | NS5 | 0.0368 | A:1647;G:0;C:63;T:0;total:1710 | iSNV |
| F21 | F21-27 | 9359 | NS5 | 0.0349 | A:0;G:0;C:69;T:1908;total:1977 | iSNV |
| F21 | F21-27 | 9446 | NS5 | 0.0484 | A:1806;G:92;C:0;T:0;total:1898 | iSNV |
| F21 | F21-27 | 10259 | NS5 | 0.1354 | A:1398;G:219;C:0;T:0;total:1617 | iSNV |
| F21 | F21-27 | 10447 | 3'-UTR | 0.0703 | A:0;G:0;C:898;T:68;total:966 | iSNV |
| F21 | F21-27 | 10566 | 3'-UTR | 0.0525 | A:0;G:0;C:758;T:42;total:800 | iSNV |
| F21 | F21-28 | 353 | C | 0.0627 | A:837;G:56;C:0;T:0;total:893 | iSNV |
| F21 | F21-28 | 645 | M | 0.0631 | A:697;G:47;C:0;T:0;total:744 | iSNV |
| F21 | F21-28 | 803 | M | 0.0441 | A:0;G:519;C:0;T:24;total:543 | iSNV |
| F21 | F21-28 | 998 | E | 0.1553 | A:0;G:0;C:446;T:82;total:528 | iSNV |
| F21 | F21-28 | 1218 | E | 0.7266 | A:0;G:0;C:175;T:465;total:640 | iSNV |
| F21 | F21-28 | 1430 | E | 0.0224 | A:0;G:0;C:698;T:16;total:714 | iSNV |
| F21 | F21-28 | 2531 | NS1 | 0.0243 | A:0;G:0;C:14;T:562;total:576 | iSNV |
| F21 | F21-28 | 3572 | NS1 | 0.0207 | A:0;G:0;C:424;T:9;total:433 | iSNV |
| F21 | F21-28 | 3869 | NS2A | 0.7417 | A:0;G:0;C:217;T:623;total:840 | iSNV |
| F21 | F21-28 | 4697 | NS3 | 0.083 | A:0;G:0;C:64;T:707;total:771 | iSNV |
| F21 | F21-28 | 4712 | NS3 | 0.0256 | A:21;G:797;C:0;T:0;total:818 | iSNV |
| F21 | F21-28 | 5311 | NS3 | 0.0202 | A:0;G:0;C:676;T:14;total:690 | iSNV |
| F21 | F21-28 | 5952 | NS3 | 0.093 | A:0;G:0;C:887;T:91;total:978 | iSNV |
| F21 | F21-28 | 7735 | NS5 | 0.0454 | A:37;G:776;C:1;T:0;total:814 | iSNV |
| F21 | F21-28 | 7744 | NS5 | 0.0214 | A:774;G:17;C:0;T:0;total:791 | iSNV |
| F21 | F21-28 | 8518 | NS5 | 0.0562 | A:738;G:0;C:0;T:44;total:782 | iSNV |
| F21 | F21-28 | 8789 | NS5 | 0.0218 | A:626;G:14;C:0;T:0;total:640 | iSNV |
| F21 | F21-28 | 9359 | NS5 | 0.0453 | A:0;G:0;C:48;T:1010;total:1058 | iSNV |
| F21 | F21-28 | 10259 | NS5 | 0.1527 | A:688;G:124;C:0;T:0;total:812 | iSNV |
| F21 | F21-28 | 10447 | 3'-UTR | 0.0741 | A:0;G:0;C:462;T:37;total:499 | iSNV |
| F21 | F21-28 | 10566 | 3'-UTR | 0.0845 | A:0;G:0;C:379;T:35;total:414 | iSNV |
| F21 | F21-29 | 353 | C | 0.0666 | A:112;G:8;C:0;T:0;total:120 | iSNV |
| F21 | F21-29 | 803 | M | 0.0921 | A:0;G:69;C:0;T:7;total:76 | iSNV |
| F21 | F21-29 | 998 | E | 0.1898 | A:0;G:0;C:64;T:15;total:79 | iSNV |
| F21 | F21-29 | 1218 | E | 0.7304 | A:0;G:0;C:24;T:65;total:89 | iSNV |
| F21 | F21-29 | 3869 | NS2A | 0.6819 | A:0;G:0;C:35;T:75;total:110 | iSNV |
| F21 | F21-29 | 4697 | NS3 | 0.0784 | A:0;G:0;C:8;T:94;total:102 | iSNV |
| F21 | F21-29 | 5952 | NS3 | 0.081 | A:0;G:1;C:135;T:12;total:148 | iSNV |
| F21 | F21-29 | 8518 | NS5 | 0.0945 | A:67;G:0;C:0;T:7;total:74 | iSNV |
| F21 | F21-29 | 9359 | NS5 | 0.0538 | A:0;G:0;C:7;T:123;total:130 | iSNV |
| F21 | F21-29 | 10259 | NS5 | 0.2037 | A:86;G:22;C:0;T:0;total:108 | iSNV |
| F21 | F21-29 | 10447 | 3'-UTR | 0.1176 | A:0;G:0;C:75;T:10;total:85 | iSNV |
| F21 | F21-3 | 353 | C | 0.0553 | A:1024;G:60;C:0;T:0;total:1084 | iSNV |
| F21 | F21-3 | 645 | M | 0.0349 | A:801;G:29;C:0;T:0;total:830 | iSNV |
| F21 | F21-3 | 803 | M | 0.1165 | A:0;G:455;C:0;T:60;total:515 | iSNV |
| F21 | F21-3 | 998 | E | 0.1453 | A:0;G:0;C:447;T:76;total:523 | iSNV |
| F21 | F21-3 | 1218 | E | 0.7817 | A:0;G:0;C:143;T:512;total:655 | iSNV |
| F21 | F21-3 | 1772 | E | 0.0224 | A:0;G:14;C:0;T:611;total:625 | iSNV |
| F21 | F21-3 | 2372 | E | 0.0436 | A:0;G:0;C:241;T:11;total:252 | iSNV |
| F21 | F21-3 | 2531 | NS1 | 0.0209 | A:0;G:0;C:10;T:468;total:478 | iSNV |
| F21 | F21-3 | 3869 | NS2A | 0.7325 | A:0;G:0;C:225;T:616;total:841 | iSNV |
| F21 | F21-3 | 4697 | NS3 | 0.1146 | A:0;G:0;C:76;T:587;total:663 | iSNV |
| F21 | F21-3 | 5952 | NS3 | 0.0968 | A:0;G:0;C:942;T:101;total:1043 | iSNV |
| F21 | F21-3 | 6729 | NS4A | 0.0244 | A:0;G:0;C:479;T:12;total:491 | iSNV |
| F21 | F21-3 | 7735 | NS5 | 0.0805 | A:68;G:776;C:0;T:0;total:844 | iSNV |
| F21 | F21-3 | 8518 | NS5 | 0.1104 | A:628;G:0;C:0;T:78;total:706 | iSNV |
| F21 | F21-3 | 9359 | NS5 | 0.048 | A:0;G:0;C:58;T:1148;total:1206 | iSNV |
| F21 | F21-3 | 9446 | NS5 | 0.025 | A:1052;G:27;C:0;T:0;total:1079 | iSNV |
| F21 | F21-3 | 10259 | NS5 | 0.1331 | A:755;G:116;C:0;T:0;total:871 | iSNV |
| F21 | F21-3 | 10428 | 3'-UTR | 0.0296 | A:0;G:0;C:622;T:19;total:641 | iSNV |
| F21 | F21-3 | 10447 | 3'-UTR | 0.0632 | A:0;G:0;C:489;T:33;total:522 | iSNV |
| F21 | F21-3 | 10566 | 3'-UTR | 0.1023 | A:0;G:0;C:386;T:44;total:430 | iSNV |
| F21 | F21-30 | 353 | C | 0.0606 | A:960;G:62;C:0;T:0;total:1022 | iSNV |
| F21 | F21-30 | 645 | M | 0.0542 | A:767;G:44;C:0;T:0;total:811 | iSNV |
| F21 | F21-30 | 803 | M | 0.0722 | A:0;G:539;C:0;T:42;total:581 | iSNV |
| F21 | F21-30 | 998 | E | 0.1517 | A:0;G:0;C:464;T:83;total:547 | iSNV |
| F21 | F21-30 | 1218 | E | 0.7448 | A:0;G:0;C:171;T:499;total:670 | iSNV |
| F21 | F21-30 | 1430 | E | 0.0414 | A:0;G:0;C:671;T:29;total:700 | iSNV |
| F21 | F21-30 | 3869 | NS2A | 0.7022 | A:0;G:0;C:241;T:568;total:809 | iSNV |
| F21 | F21-30 | 4697 | NS3 | 0.1404 | A:0;G:0;C:101;T:618;total:719 | iSNV |

| | | | | | | |
|---|---|---|---|---|---|---|
| F21 | F21-30 | 5952 | NS3 | 0.1234 | A:0;G:0;C:916;T:129;total:1045 | iSNV |
| F21 | F21-30 | 7735 | NS5 | 0.0581 | A:46;G:745;C:0;T:0;total:791 | iSNV |
| F21 | F21-30 | 8168 | NS5 | 0.0204 | A:0;G:0;C:670;T:14;total:684 | iSNV |
| F21 | F21-30 | 8518 | NS5 | 0.0368 | A:705;G:0;C:0;T:27;total:732 | iSNV |
| F21 | F21-30 | 9359 | NS5 | 0.073 | A:0;G:0;C:76;T:964;total:1040 | iSNV |
| F21 | F21-30 | 10259 | NS5 | 0.1931 | A:685;G:164;C:0;T:0;total:849 | iSNV |
| F21 | F21-30 | 10428 | 3'-UTR | 0.0202 | A:0;G:0;C:582;T:12;total:594 | iSNV |
| F21 | F21-30 | 10447 | 3'-UTR | 0.1061 | A:0;G:0;C:463;T:55;total:518 | iSNV |
| F21 | F21-30 | 10566 | 3'-UTR | 0.1113 | A:0;G:0;C:415;T:52;total:467 | iSNV |
| F21 | F21-4 | 353 | C | 0.0482 | A:1045;G:53;C:0;T:0;total:1098 | iSNV |
| F21 | F21-4 | 645 | M | 0.0662 | A:789;G:56;C:0;T:0;total:845 | iSNV |
| F21 | F21-4 | 803 | M | 0.0812 | A:0;G:373;C:0;T:33;total:406 | iSNV |
| F21 | F21-4 | 998 | E | 0.1871 | A:0;G:0;C:330;T:76;total:406 | iSNV |
| F21 | F21-4 | 1218 | E | 0.7975 | A:0;G:0;C:112;T:441;total:553 | iSNV |
| F21 | F21-4 | 1430 | E | 0.0295 | A:0;G:0;C:493;T:15;total:508 | iSNV |
| F21 | F21-4 | 3869 | NS2A | 0.7697 | A:0;G:0;C:173;T:578;total:751 | iSNV |
| F21 | F21-4 | 4697 | NS3 | 0.1049 | A:0;G:0;C:61;T:520;total:581 | iSNV |
| F21 | F21-4 | 5952 | NS3 | 0.1022 | A:0;G:0;C:808;T:92;total:900 | iSNV |
| F21 | F21-4 | 7735 | NS5 | 0.0493 | A:41;G:790;C:0;T:0;total:831 | iSNV |
| F21 | F21-4 | 9359 | NS5 | 0.0521 | A:0;G:0;C:64;T:1164;total:1228 | iSNV |
| F21 | F21-4 | 9818 | NS5 | 0.0343 | A:0;G:0;C:13;T:365;total:378 | iSNV |
| F21 | F21-4 | 10259 | NS5 | 0.1378 | A:688;G:110;C:0;T:0;total:798 | iSNV |
| F21 | F21-4 | 10447 | 3'-UTR | 0.0623 | A:0;G:0;C:436;T:29;total:465 | iSNV |
| F21 | F21-4 | 10566 | 3'-UTR | 0.0879 | A:0;G:0;C:363;T:35;total:398 | iSNV |
| F21 | F21-5 | 353 | C | 0.0705 | A:145;G:11;C:0;T:0;total:156 | iSNV |
| F21 | F21-5 | 645 | M | 0.0578 | A:114;G:7;C:0;T:0;total:121 | iSNV |
| F21 | F21-5 | 803 | M | 0.0795 | A:0;G:81;C:0;T:7;total:88 | iSNV |
| F21 | F21-5 | 998 | E | 0.1553 | A:0;G:0;C:87;T:16;total:103 | iSNV |
| F21 | F21-5 | 1218 | E | 0.8265 | A:0;G:0;C:21;T:100;total:121 | iSNV |
| F21 | F21-5 | 1430 | E | 0.042 | A:0;G:0;C:114;T:5;total:119 | iSNV |
| F21 | F21-5 | 3869 | NS2A | 0.8 | A:0;G:0;C:33;T:132;total:165 | iSNV |
| F21 | F21-5 | 4697 | NS3 | 0.067 | A:0;G:0;C:11;T:153;total:164 | iSNV |
| F21 | F21-5 | 5952 | NS3 | 0.0994 | A:0;G:0;C:154;T:17;total:171 | iSNV |
| F21 | F21-5 | 7735 | NS5 | 0.0714 | A:8;G:104;C:0;T:0;total:112 | iSNV |
| F21 | F21-5 | 8518 | NS5 | 0.0956 | A:104;G:0;C:0;T:11;total:115 | iSNV |
| F21 | F21-5 | 9359 | NS5 | 0.0511 | A:0;G:0;C:9;T:167;total:176 | iSNV |
| F21 | F21-5 | 10259 | NS5 | 0.119 | A:111;G:15;C:0;T:0;total:126 | iSNV |
| F21 | F21-6 | 353 | C | 0.0638 | A:968;G:66;C:0;T:0;total:1034 | iSNV |
| F21 | F21-6 | 645 | M | 0.0413 | A:766;G:33;C:0;T:0;total:799 | iSNV |
| F21 | F21-6 | 803 | M | 0.1142 | A:0;G:341;C:0;T:44;total:385 | iSNV |
| F21 | F21-6 | 998 | E | 0.1327 | A:0;G:0;C:418;T:64;total:482 | iSNV |
| F21 | F21-6 | 1218 | E | 0.7479 | A:0;G:0;C:148;T:439;total:587 | iSNV |
| F21 | F21-6 | 1430 | E | 0.024 | A:0;G:0;C:527;T:13;total:540 | iSNV |
| F21 | F21-6 | 2531 | NS1 | 0.0231 | A:0;G:0;C:12;T:506;total:518 | iSNV |
| F21 | F21-6 | 3869 | NS2A | 0.736 | A:0;G:0;C:193;T:538;total:731 | iSNV |
| F21 | F21-6 | 4697 | NS3 | 0.0756 | A:0;G:0;C:50;T:611;total:661 | iSNV |
| F21 | F21-6 | 5952 | NS3 | 0.071 | A:0;G:0;C:863;T:66;total:929 | iSNV |
| F21 | F21-6 | 7735 | NS5 | 0.0764 | A:61;G:737;C:0;T:0;total:798 | iSNV |
| F21 | F21-6 | 8518 | NS5 | 0.0732 | A:443;G:0;C:0;T:35;total:478 | iSNV |
| F21 | F21-6 | 9359 | NS5 | 0.0583 | A:0;G:0;C:73;T:1179;total:1252 | iSNV |
| F21 | F21-6 | 10259 | NS5 | 0.1226 | A:751;G:105;C:0;T:0;total:856 | iSNV |
| F21 | F21-6 | 10447 | 3'-UTR | 0.0724 | A:0;G:0;C:448;T:35;total:483 | iSNV |
| F21 | F21-6 | 10566 | 3'-UTR | 0.088 | A:0;G:0;C:352;T:34;total:386 | iSNV |
| F21 | F21-7 | 353 | C | 0.042 | A:888;G:39;C:0;T:0;total:927 | iSNV |
| F21 | F21-7 | 645 | M | 0.0632 | A:622;G:42;C:0;T:0;total:664 | iSNV |
| F21 | F21-7 | 803 | M | 0.0704 | A:0;G:277;C:0;T:21;total:298 | iSNV |
| F21 | F21-7 | 998 | E | 0.1706 | A:0;G:0;C:277;T:57;total:334 | iSNV |
| F21 | F21-7 | 1218 | E | 0.7524 | A:0;G:0;C:104;T:316;total:420 | iSNV |
| F21 | F21-7 | 1430 | E | 0.0275 | A:0;G:0;C:389;T:11;total:400 | iSNV |
| F21 | F21-7 | 3257 | NS1 | 0.0203 | A:0;G:0;C:15;T:722;total:737 | iSNV |
| F21 | F21-7 | 3572 | NS1 | 0.0308 | A:0;G:0;C:220;T:7;total:227 | iSNV |
| F21 | F21-7 | 3869 | NS2A | 0.7427 | A:0;G:0;C:132;T:381;total:513 | iSNV |
| F21 | F21-7 | 4697 | NS3 | 0.1024 | A:0;G:0;C:50;T:438;total:488 | iSNV |
| F21 | F21-7 | 5952 | NS3 | 0.1063 | A:0;G:0;C:647;T:77;total:724 | iSNV |
| F21 | F21-7 | 7735 | NS5 | 0.0742 | A:50;G:623;C:0;T:0;total:673 | iSNV |
| F21 | F21-7 | 9359 | NS5 | 0.0278 | A:0;G:0;C:31;T:1083;total:1114 | iSNV |
| F21 | F21-7 | 10259 | NS5 | 0.1529 | A:587;G:106;C:0;T:0;total:693 | iSNV |
| F21 | F21-7 | 10428 | 3'-UTR | 0.0324 | A:0;G:0;C:418;T:14;total:432 | iSNV |
| F21 | F21-7 | 10447 | 3'-UTR | 0.1368 | A:0;G:0;C:309;T:49;total:358 | iSNV |
| F21 | F21-7 | 10566 | 3'-UTR | 0.0866 | A:0;G:0;C:274;T:26;total:300 | iSNV |
| F21 | F21-8 | 353 | C | 0.0648 | A:173;G:12;C:0;T:0;total:185 | iSNV |
| F21 | F21-8 | 803 | M | 0.1151 | A:0;G:123;C:0;T:16;total:139 | iSNV |
| F21 | F21-8 | 998 | E | 0.126 | A:0;G:0;C:104;T:15;total:119 | iSNV |
| F21 | F21-8 | 1218 | E | 0.7715 | A:0;G:0;C:32;T:108;total:140 | iSNV |
| F21 | F21-8 | 3869 | NS2A | 0.6418 | A:0;G:0;C:72;T:129;total:201 | iSNV |
| F21 | F21-8 | 3962 | NS2A | 0.0285 | A:0;G:0;C:204;T:6;total:210 | iSNV |
| F21 | F21-8 | 4697 | NS3 | 0.0804 | A:0;G:0;C:14;T:160;total:174 | iSNV |
| F21 | F21-8 | 5952 | NS3 | 0.0947 | A:0;G:0;C:191;T:20;total:211 | iSNV |
| F21 | F21-8 | 7735 | NS5 | 0.0833 | A:11;G:121;C:0;T:0;total:132 | iSNV |
| F21 | F21-8 | 7744 | NS5 | 0.0468 | A:122;G:6;C:0;T:0;total:128 | iSNV |
| F21 | F21-8 | 8282 | NS5 | 0.0389 | A:0;G:0;C:6;T:148;total:154 | iSNV |
| F21 | F21-8 | 9359 | NS5 | 0.0758 | A:0;G:0;C:16;T:195;total:211 | iSNV |

| | | | | | | |
|---|---|---|---|---|---|---|
| F21 | F21-8 | 10259 | NS5 | 0.139 | A:161;G:26;C:0;T:0;total:187 | iSNV |
| F21 | F21-8 | 10447 | 3'-UTR | 0.0458 | A:0;G:0;C:125;T:6;total:131 | iSNV |
| F21 | F21-8 | 10566 | 3'-UTR | 0.0983 | A:0;G:0;C:110;T:12;total:122 | iSNV |
| F21 | F21-9 | 353 | C | 0.061 | A:1430;G:93;C:0;T:0;total:1523 | iSNV |
| F21 | F21-9 | 645 | M | 0.0435 | A:1143;G:52;C:0;T:0;total:1195 | iSNV |
| F21 | F21-9 | 803 | M | 0.0915 | A:0;G:695;C:0;T:70;total:765 | iSNV |
| F21 | F21-9 | 998 | E | 0.1651 | A:0;G:0;C:546;T:108;total:654 | iSNV |
| F21 | F21-9 | 1218 | E | 0.7457 | A:0;G:0;C:218;T:639;total:857 | iSNV |
| F21 | F21-9 | 1772 | E | 0.02 | A:0;G:17;C:0;T:830;total:847 | iSNV |
| F21 | F21-9 | 3869 | NS2A | 0.7426 | A:0;G:0;C:286;T:825;total:1111 | iSNV |
| F21 | F21-9 | 4697 | NS3 | 0.0804 | A:0;G:0;C:73;T:834;total:907 | iSNV |
| F21 | F21-9 | 5952 | NS3 | 0.0942 | A:0;G:0;C:1422;T:148;total:1570 | iSNV |
| F21 | F21-9 | 7735 | NS5 | 0.0496 | A:61;G:1167;C:0;T:0;total:1228 | iSNV |
| F21 | F21-9 | 8518 | NS5 | 0.0911 | A:838;G:0;C:0;T:84;total:922 | iSNV |
| F21 | F21-9 | 9359 | NS5 | 0.0383 | A:0;G:0;C:67;T:1678;total:1745 | iSNV |
| F21 | F21-9 | 10259 | NS5 | 0.1488 | A:1028;G:180;C:0;T:1;total:1209 | iSNV |
| F21 | F21-9 | 10447 | 3'-UTR | 0.0742 | A:0;G:0;C:661;T:53;total:714 | iSNV |
| F21 | F21-9 | 10566 | 3'-UTR | 0.0801 | A:0;G:0;C:562;T:49;total:611 | iSNV |
| F24 | F24-1 | 645 | M | 0.0261 | A:6960;G:187;C:0;T:0;total:7147 | iSNV |
| F24 | F24-1 | 930 | M | 0.0236 | A:1;G:1;C:6111;T:148;total:6261 | iSNV |
| F24 | F24-1 | 998 | E | 0.1632 | A:2;G:1;C:5174;T:1010;total:6187 | iSNV |
| F24 | F24-1 | 1218 | E | 0.9465 | A:0;G:1;C:371;T:6559;total:6931 | iSNV |
| F24 | F24-1 | 2213 | E | 0.0219 | A:179;G:7981;C:0;T:0;total:8160 | iSNV |
| F24 | F24-1 | 2376 | E | 0.0272 | A:3;G:0;C:5311;T:149;total:5463 | iSNV |
| F24 | F24-1 | 3341 | NS1 | 0.0263 | A:7769;G:210;C:0;T:0;total:7979 | iSNV |
| F24 | F24-1 | 3869 | NS2A | 0.8237 | A:0;G:0;C:1635;T:7637;total:9272 | iSNV |
| F24 | F24-1 | 3926 | NS2A | 0.027 | A:0;G:0;C:9885;T:275;total:10160 | iSNV |
| F24 | F24-1 | 4226 | NS2B | 0.0255 | A:0;G:2;C:109;T:4162;total:4273 | iSNV |
| F24 | F24-1 | 4559 | NS2B | 0.0276 | A:4;G:0;C:8414;T:239;total:8657 | iSNV |
| F24 | F24-1 | 4697 | NS3 | 0.1363 | A:1;G:2;C:1274;T:8068;total:9345 | iSNV |
| F24 | F24-1 | 5952 | NS3 | 0.1332 | A:3;G:0;C:8547;T:1315;total:9865 | iSNV |
| F24 | F24-1 | 7741 | NS5 | 0.0325 | A:7748;G:261;C:3;T:2;total:8014 | iSNV |
| F24 | F24-1 | 8651 | NS5 | 0.0232 | A:5871;G:140;C:1;T:2;total:6014 | iSNV |
| F24 | F24-1 | 9359 | NS5 | 0.1254 | A:0;G:2;C:1231;T:8581;total:9814 | iSNV |
| F24 | F24-1 | 9983 | NS5 | 0.0337 | A:2;G:0;C:5349;T:187;total:5538 | iSNV |
| F24 | F24-1 | 10259 | NS5 | 0.1524 | A:6213;G:1118;C:0;T:1;total:7332 | iSNV |
| F24 | F24-1 | 10447 | 3'-UTR | 0.0282 | A:0;G:0;C:4259;T:124;total:4383 | iSNV |
| F24 | F24-1 | 10560 | 3'-UTR | 0.0216 | A:5193;G:115;C:0;T:0;total:5308 | iSNV |
| F24 | F24-1 | 10566 | 3'-UTR | 0.1359 | A:1;G:0;C:4625;T:728;total:5354 | iSNV |
| F24 | F24-10 | 353 | C | 0.0251 | A:1316;G:34;C:0;T:0;total:1350 | iSNV |
| F24 | F24-10 | 998 | E | 0.085 | A:0;G:0;C:1312;T:122;total:1434 | iSNV |
| F24 | F24-10 | 1218 | E | 0.9412 | A:0;G:0;C:100;T:1599;total:1699 | iSNV |
| F24 | F24-10 | 1283 | E | 0.0208 | A:1;G:0;C:1501;T:32;total:1534 | iSNV |
| F24 | F24-10 | 1430 | E | 0.0646 | A:0;G:0;C:1665;T:115;total:1780 | iSNV |
| F24 | F24-10 | 3869 | NS2A | 0.8858 | A:0;G:0;C:218;T:1690;total:1908 | iSNV |
| F24 | F24-10 | 4001 | NS2A | 0.0217 | A:1;G:2022;C:0;T:45;total:2068 | iSNV |
| F24 | F24-10 | 4697 | NS3 | 0.0659 | A:0;G:0;C:149;T:2110;total:2259 | iSNV |
| F24 | F24-10 | 4745 | NS3 | 0.0275 | A:0;G:1;C:2186;T:62;total:2249 | iSNV |
| F24 | F24-10 | 5952 | NS3 | 0.0631 | A:1;G:0;C:1587;T:107;total:1695 | iSNV |
| F24 | F24-10 | 6206 | NS3 | 0.0713 | A:0;G:0;C:1431;T:110;total:1541 | iSNV |
| F24 | F24-10 | 6398 | NS3 | 0.0256 | A:0;G:1;C:45;T:1706;total:1752 | iSNV |
| F24 | F24-10 | 9152 | NS5 | 0.0265 | A:0;G:1721;C:0;T:47;total:1768 | iSNV |
| F24 | F24-10 | 9359 | NS5 | 0.0529 | A:0;G:1;C:88;T:1573;total:1662 | iSNV |
| F24 | F24-10 | 10259 | NS5 | 0.0769 | A:1858;G:155;C:0;T:0;total:2013 | iSNV |
| F24 | F24-10 | 10447 | 3'-UTR | 0.0388 | A:0;G:0;C:1112;T:45;total:1157 | iSNV |
| F24 | F24-10 | 10566 | 3'-UTR | 0.0489 | A:0;G:0;C:1439;T:74;total:1513 | iSNV |
| F24 | F24-11 | 803 | M | 0.022 | A:1;G:5847;C:0;T:132;total:5980 | iSNV |
| F24 | F24-11 | 998 | E | 0.103 | A:0;G:0;C:5979;T:687;total:6666 | iSNV |
| F24 | F24-11 | 1218 | E | 0.9327 | A:2;G:0;C:473;T:6546;total:7021 | iSNV |
| F24 | F24-11 | 1232 | E | 0.0208 | A:7379;G:157;C:0;T:0;total:7536 | iSNV |
| F24 | F24-11 | 3149 | NS1 | 0.0808 | A:0;G:0;C:611;T:6948;total:7559 | iSNV |
| F24 | F24-11 | 3428 | NS1 | 0.0239 | A:0;G:0;C:6155;T:151;total:6306 | iSNV |
| F24 | F24-11 | 3869 | NS2A | 0.8772 | A:0;G:0;C:819;T:5848;total:6667 | iSNV |
| F24 | F24-11 | 4697 | NS3 | 0.0719 | A:0;G:0;C:591;T:7628;total:8219 | iSNV |
| F24 | F24-11 | 5952 | NS3 | 0.0768 | A:0;G:0;C:7366;T:613;total:7979 | iSNV |
| F24 | F24-11 | 8518 | NS5 | 0.0262 | A:8133;G:1;C:0;T:219;total:8353 | iSNV |
| F24 | F24-11 | 9359 | NS5 | 0.064 | A:0;G:0;C:518;T:7571;total:8089 | iSNV |
| F24 | F24-11 | 9880 | NS5 | 0.0944 | A:670;G:6423;C:0;T:1;total:7094 | iSNV |
| F24 | F24-11 | 10259 | NS5 | 0.1009 | A:7045;G:791;C:0;T:0;total:7836 | iSNV |
| F24 | F24-11 | 10447 | 3'-UTR | 0.0224 | A:0;G:0;C:5760;T:132;total:5892 | iSNV |
| F24 | F24-11 | 10566 | 3'-UTR | 0.0684 | A:0;G:0;C:5562;T:409;total:5971 | iSNV |
| F24 | F24-11 | 10589 | 3'-UTR | 0.0934 | A:0;G:589;C:0;T:5711;total:6300 | iSNV |
| F24 | F24-12 | 353 | C | 0.0847 | A:18245;G:1690;C:8;T:0;total:19943 | iSNV |
| F24 | F24-12 | 645 | M | 0.0778 | A:14224;G:1201;C:0;T:0;total:15425 | iSNV |
| F24 | F24-12 | 998 | E | 0.2246 | A:0;G:1;C:8260;T:2394;total:10655 | iSNV |
| F24 | F24-12 | 1117 | E | 0.0869 | A:10901;G:1039;C:2;T:2;total:11944 | iSNV |
| F24 | F24-12 | 1218 | E | 0.8818 | A:0;G:3;C:1519;T:11326;total:12848 | iSNV |
| F24 | F24-12 | 2277 | E | 0.0723 | A:3;G:2;C:749;T:9604;total:10358 | iSNV |
| F24 | F24-12 | 3869 | NS2A | 0.7728 | A:2;G:3;C:3912;T:13296;total:17213 | iSNV |
| F24 | F24-12 | 3974 | NS2A | 0.025 | A:1;G:0;C:15102;T:388;total:15491 | iSNV |
| F24 | F24-12 | 4697 | NS3 | 0.1235 | A:1;G:2;C:1970;T:13971;total:15944 | iSNV |
| F24 | F24-12 | 5952 | NS3 | 0.1296 | A:0;G:2;C:18674;T:2783;total:21459 | iSNV |

| | | | | | | |
|---|---|---|---|---|---|---|
| F24 | F24-12 | 8198 | NS5 | 0.0298 | A:14130;G:435;C:2;T:0;total:14567 | iSNV |
| F24 | F24-12 | 9242 | NS5 | 0.0204 | A:1;G:458;C:1;T:21943;total:22403 | iSNV |
| F24 | F24-12 | 9359 | NS5 | 0.1296 | A:0;G:2;C:3059;T:20533;total:23594 | iSNV |
| F24 | F24-12 | 10259 | NS5 | 0.2233 | A:13651;G:3928;C:1;T:3;total:17583 | iSNV |
| F24 | F24-12 | 10428 | 3'-UTR | 0.0272 | A:0;G:1;C:12721;T:357;total:13079 | iSNV |
| F24 | F24-12 | 10447 | 3'-UTR | 0.0946 | A:1;G:1;C:10404;T:1088;total:11494 | iSNV |
| F24 | F24-12 | 10566 | 3'-UTR | 0.12 | A:1;G:0;C:7643;T:1043;total:8687 | iSNV |
| F24 | F24-12 | 10589 | 3'-UTR | 0.021 | A:0;G:194;C:0;T:9003;total:9197 | iSNV |
| F24 | F24-13 | 395 | C | 0.3413 | A:654;G:339;C:0;T:0;total:993 | iSNV |
| F24 | F24-13 | 998 | E | 0.0777 | A:0;G:0;C:795;T:67;total:862 | iSNV |
| F24 | F24-13 | 1218 | E | 0.9534 | A:0;G:0;C:45;T:920;total:965 | iSNV |
| F24 | F24-13 | 1413 | E | 0.3462 | A:659;G:349;C:0;T:0;total:1008 | iSNV |
| F24 | F24-13 | 3869 | NS2A | 0.901 | A:0;G:0;C:109;T:991;total:1100 | iSNV |
| F24 | F24-13 | 4697 | NS3 | 0.0668 | A:0;G:0;C:91;T:1270;total:1361 | iSNV |
| F24 | F24-13 | 5952 | NS3 | 0.0564 | A:0;G:0;C:970;T:58;total:1028 | iSNV |
| F24 | F24-13 | 6152 | NS3 | 0.0216 | A:768;G:17;C:0;T:0;total:785 | iSNV |
| F24 | F24-13 | 7304 | NS4B | 0.0216 | A:814;G:0;C:0;T:18;total:832 | iSNV |
| F24 | F24-13 | 7685 | NS5 | 0.0283 | A:0;G:0;C:26;T:892;total:918 | iSNV |
| F24 | F24-13 | 9359 | NS5 | 0.0648 | A:0;G:0;C:63;T:909;total:972 | iSNV |
| F24 | F24-13 | 10259 | NS5 | 0.0773 | A:1074;G:90;C:0;T:0;total:1164 | iSNV |
| F24 | F24-13 | 10376 | NS5 | 0.3088 | A:231;G:517;C:0;T:0;total:748 | iSNV |
| F24 | F24-13 | 10566 | 3'-UTR | 0.0703 | A:0;G:0;C:846;T:64;total:910 | iSNV |
| F24 | F24-14 | 218 | C | 0.0281 | A:7332;G:212;C:0;T:0;total:7544 | iSNV |
| F24 | F24-14 | 340 | C | 0.0397 | A:0;G:0;C:8672;T:359;total:9031 | iSNV |
| F24 | F24-14 | 353 | C | 0.0478 | A:8183;G:411;C:0;T:0;total:8594 | iSNV |
| F24 | F24-14 | 645 | M | 0.0439 | A:7287;G:335;C:1;T:0;total:7623 | iSNV |
| F24 | F24-14 | 854 | M | 0.0246 | A:0;G:0;C:6780;T:171;total:6951 | iSNV |
| F24 | F24-14 | 995 | E | 0.0336 | A:237;G:6797;C:0;T:1;total:7035 | iSNV |
| F24 | F24-14 | 998 | E | 0.1412 | A:0;G:0;C:5787;T:952;total:6739 | iSNV |
| F24 | F24-14 | 1117 | E | 0.072 | A:6886;G:535;C:0;T:0;total:7421 | iSNV |
| F24 | F24-14 | 1218 | E | 0.906 | A:0;G:0;C:728;T:7009;total:7737 | iSNV |
| F24 | F24-14 | 1295 | E | 0.0313 | A:8625;G:0;C:0;T:279;total:8904 | iSNV |
| F24 | F24-14 | 1430 | E | 0.0242 | A:0;G:0;C:8563;T:213;total:8776 | iSNV |
| F24 | F24-14 | 1838 | E | 0.02 | A:1;G:0;C:170;T:8288;total:8459 | iSNV |
| F24 | F24-14 | 2186 | E | 0.0259 | A:191;G:7183;C:0;T:0;total:7374 | iSNV |
| F24 | F24-14 | 3869 | NS2A | 0.8317 | A:0;G:0;C:1244;T:6144;total:7388 | iSNV |
| F24 | F24-14 | 4187 | NS2A | 0.0203 | A:4239;G:88;C:0;T:0;total:4327 | iSNV |
| F24 | F24-14 | 4697 | NS3 | 0.0795 | A:0;G:0;C:710;T:8215;total:8925 | iSNV |
| F24 | F24-14 | 4698 | NS3 | 0.033 | A:2;G:0;C:8613;T:294;total:8909 | iSNV |
| F24 | F24-14 | 5558 | NS3 | 0.0651 | A:534;G:7658;C:0;T:0;total:8192 | iSNV |
| F24 | F24-14 | 5952 | NS3 | 0.0862 | A:1;G:0;C:8151;T:769;total:8921 | iSNV |
| F24 | F24-14 | 6401 | NS3 | 0.0462 | A:0;G:0;C:340;T:7004;total:7344 | iSNV |
| F24 | F24-14 | 6557 | NS4A | 0.0214 | A:6008;G:0;C:0;T:132;total:6140 | iSNV |
| F24 | F24-14 | 6786 | NS4A | 0.0215 | A:0;G:0;C:6400;T:141;total:6541 | iSNV |
| F24 | F24-14 | 7232 | NS4A | 0.0237 | A:0;G:0;C:6120;T:149;total:6269 | iSNV |
| F24 | F24-14 | 9359 | NS5 | 0.0761 | A:0;G:0;C:703;T:8525;total:9228 | iSNV |
| F24 | F24-14 | 9370 | NS5 | 0.0263 | A:0;G:248;C:0;T:9160;total:9408 | iSNV |
| F24 | F24-14 | 9542 | NS5 | 0.0281 | A:0;G:0;C:8106;T:235;total:8341 | iSNV |
| F24 | F24-14 | 9584 | NS5 | 0.0325 | A:1;G:0;C:7697;T:259;total:7957 | iSNV |
| F24 | F24-14 | 10259 | NS5 | 0.1445 | A:7156;G:1209;C:0;T:0;total:8365 | iSNV |
| F24 | F24-14 | 10447 | 3'-UTR | 0.056 | A:1;G:0;C:5811;T:345;total:6157 | iSNV |
| F24 | F24-14 | 10452 | 3'-UTR | 0.0297 | A:5900;G:181;C:0;T:0;total:6081 | iSNV |
| F24 | F24-14 | 10566 | 3'-UTR | 0.0768 | A:2;G:0;C:5765;T:480;total:6247 | iSNV |
| F24 | F24-14 | 10567 | 3'-UTR | 0.0219 | A:3;G:0;C:136;T:6060;total:6199 | iSNV |
| F24 | F24-15 | 197 | C | 0.028 | A:15109;G:436;C:0;T:1;total:15546 | iSNV |
| F24 | F24-15 | 998 | E | 0.0886 | A:0;G:0;C:6711;T:653;total:7364 | iSNV |
| F24 | F24-15 | 1218 | E | 0.967 | A:0;G:0;C:304;T:8898;total:9202 | iSNV |
| F24 | F24-15 | 1430 | E | 0.0251 | A:2;G:0;C:9699;T:250;total:9951 | iSNV |
| F24 | F24-15 | 2465 | E | 0.0234 | A:0;G:1;C:5000;T:120;total:5121 | iSNV |
| F24 | F24-15 | 3869 | NS2A | 0.8938 | A:0;G:0;C:1306;T:10985;total:12291 | iSNV |
| F24 | F24-15 | 4697 | NS3 | 0.0791 | A:0;G:3;C:904;T:10518;total:11425 | iSNV |
| F24 | F24-15 | 5480 | NS3 | 0.0239 | A:0;G:2;C:12566;T:308;total:12876 | iSNV |
| F24 | F24-15 | 5952 | NS3 | 0.0804 | A:1;G:1;C:13595;T:1189;total:14786 | iSNV |
| F24 | F24-15 | 6314 | NS3 | 0.0361 | A:1;G:2;C:236;T:6290;total:6529 | iSNV |
| F24 | F24-15 | 6779 | NS4A | 0.029 | A:8607;G:258;C:1;T:0;total:8866 | iSNV |
| F24 | F24-15 | 6956 | NS4A | 0.0234 | A:1;G:3;C:7395;T:178;total:7577 | iSNV |
| F24 | F24-15 | 7685 | NS5 | 0.0226 | A:2;G:0;C:287;T:12394;total:12683 | iSNV |
| F24 | F24-15 | 8456 | NS5 | 0.0219 | A:13930;G:313;C:0;T:0;total:14243 | iSNV |
| F24 | F24-15 | 9359 | NS5 | 0.0768 | A:0;G:2;C:1228;T:14745;total:15975 | iSNV |
| F24 | F24-15 | 9634 | NS5 | 0.0285 | A:0;G:0;C:175;T:5945;total:6120 | iSNV |
| F24 | F24-15 | 9818 | NS5 | 0.0229 | A:1;G:0;C:157;T:6686;total:6844 | iSNV |
| F24 | F24-15 | 10259 | NS5 | 0.086 | A:10978;G:1034;C:0;T:0;total:12012 | iSNV |
| F24 | F24-15 | 10566 | 3'-UTR | 0.072 | A:0;G:0;C:5655;T:439;total:6094 | iSNV |
| F24 | F24-16 | 353 | C | 0.0621 | A:317;G:21;C:0;T:0;total:338 | iSNV |
| F24 | F24-16 | 645 | M | 0.0354 | A:272;G:10;C:0;T:0;total:282 | iSNV |
| F24 | F24-16 | 998 | E | 0.2242 | A:0;G:0;C:128;T:37;total:165 | iSNV |
| F24 | F24-16 | 1218 | E | 0.8658 | A:0;G:0;C:29;T:187;total:216 | iSNV |
| F24 | F24-16 | 1430 | E | 0.0235 | A:0;G:0;C:207;T:5;total:212 | iSNV |
| F24 | F24-16 | 2218 | E | 0.0414 | A:0;G:0;C:231;T:10;total:241 | iSNV |
| F24 | F24-16 | 3869 | NS2A | 0.7084 | A:0;G:0;C:98;T:238;total:336 | iSNV |
| F24 | F24-16 | 3965 | NS2A | 0.0316 | A:0;G:0;C:306;T:10;total:316 | iSNV |
| F24 | F24-16 | 4049 | NS2A | 0.0358 | A:0;G:0;C:215;T:8;total:223 | iSNV |

| | | | | | | |
|---|---|---|---|---|---|---|
| F24 | F24-16 | 4697 | NS3 | 0.1314 | A:0;G:0;C:33;T:218;total:251 | iSNV |
| F24 | F24-16 | 5952 | NS3 | 0.1319 | A:0;G:0;C:283;T:43;total:326 | iSNV |
| F24 | F24-16 | 7735 | NS5 | 0.042 | A:9;G:205;C:0;T:0;total:214 | iSNV |
| F24 | F24-16 | 9359 | NS5 | 0.1002 | A:0;G:0;C:37;T:332;total:369 | iSNV |
| F24 | F24-16 | 9818 | NS5 | 0.0576 | A:0;G:0;C:9;T:147;total:156 | iSNV |
| F24 | F24-16 | 10259 | NS5 | 0.1711 | A:218;G:45;C:0;T:0;total:263 | iSNV |
| F24 | F24-16 | 10334 | NS5 | 0.0208 | A:0;G:0;C:282;T:6;total:288 | iSNV |
| F24 | F24-16 | 10428 | 3'-UTR | 0.0309 | A:0;G:0;C:219;T:7;total:226 | iSNV |
| F24 | F24-16 | 10447 | 3'-UTR | 0.0634 | A:0;G:0;C:177;T:12;total:189 | iSNV |
| F24 | F24-16 | 10452 | 3'-UTR | 0.0481 | A:178;G:9;C:0;T:0;total:187 | iSNV |
| F24 | F24-16 | 10566 | 3'-UTR | 0.1025 | A:0;G:0;C:175;T:20;total:195 | iSNV |
| F24 | F24-16 | 10578 | 3'-UTR | 0.0304 | A:0;G:0;C:6;T:191;total:197 | iSNV |
| F24 | F24-16 | 10782 | 3'-UTR | 0.0284 | A:0;G:0;C:171;T:5;total:176 | iSNV |
| F24 | F24-17 | 998 | E | 0.1193 | A:1;G:0;C:7018;T:951;total:7970 | iSNV |
| F24 | F24-17 | 1218 | E | 0.9332 | A:0;G:0;C:553;T:7721;total:8274 | iSNV |
| F24 | F24-17 | 1430 | E | 0.031 | A:0;G:0;C:9403;T:301;total:9704 | iSNV |
| F24 | F24-17 | 1586 | E | 0.0226 | A:0;G:0;C:8257;T:191;total:8448 | iSNV |
| F24 | F24-17 | 2376 | E | 0.0482 | A:0;G:0;C:6347;T:322;total:6669 | iSNV |
| F24 | F24-17 | 3869 | NS2A | 0.8404 | A:0;G:0;C:1273;T:6703;total:7976 | iSNV |
| F24 | F24-17 | 4319 | NS2B | 0.0247 | A:188;G:7398;C:0;T:1;total:7587 | iSNV |
| F24 | F24-17 | 4697 | NS3 | 0.1068 | A:1;G:0;C:996;T:8321;total:9318 | iSNV |
| F24 | F24-17 | 4783 | NS3 | 0.02 | A:0;G:0;C:179;T:8758;total:8937 | iSNV |
| F24 | F24-17 | 5546 | NS3 | 0.0216 | A:0;G:0;C:9342;T:207;total:9549 | iSNV |
| F24 | F24-17 | 5952 | NS3 | 0.1103 | A:0;G:0;C:8716;T:1081;total:9797 | iSNV |
| F24 | F24-17 | 6401 | NS3 | 0.0378 | A:0;G:0;C:307;T:7797;total:8104 | iSNV |
| F24 | F24-17 | 6938 | NS4A | 0.025 | A:0;G:0;C:7716;T:198;total:7914 | iSNV |
| F24 | F24-17 | 7151 | NS4A | 0.0442 | A:0;G:0;C:8697;T:403;total:9100 | iSNV |
| F24 | F24-17 | 9359 | NS5 | 0.0922 | A:0;G:0;C:920;T:9053;total:9973 | iSNV |
| F24 | F24-17 | 10259 | NS5 | 0.1313 | A:8040;G:1216;C:0;T:0;total:9256 | iSNV |
| F24 | F24-17 | 10428 | 3'-UTR | 0.0371 | A:0;G:0;C:7369;T:284;total:7653 | iSNV |
| F24 | F24-17 | 10447 | 3'-UTR | 0.0333 | A:0;G:0;C:6686;T:231;total:6917 | iSNV |
| F24 | F24-17 | 10452 | 3'-UTR | 0.0239 | A:6629;G:163;C:0;T:1;total:6793 | iSNV |
| F24 | F24-17 | 10566 | 3'-UTR | 0.0908 | A:0;G:0;C:6531;T:653;total:7184 | iSNV |
| F24 | F24-18 | 998 | E | 0.1508 | A:0;G:1;C:6142;T:1091;total:7234 | iSNV |
| F24 | F24-18 | 1218 | E | 0.9534 | A:1;G:3;C:410;T:8376;total:8790 | iSNV |
| F24 | F24-18 | 1413 | E | 0.0275 | A:9781;G:277;C:0;T:0;total:10058 | iSNV |
| F24 | F24-18 | 1430 | E | 0.0374 | A:0;G:0;C:8847;T:344;total:9191 | iSNV |
| F24 | F24-18 | 1672 | E | 0.0235 | A:1;G:0;C:5937;T:143;total:6081 | iSNV |
| F24 | F24-18 | 3869 | NS2A | 0.8308 | A:1;G:0;C:1808;T:8876;total:10685 | iSNV |
| F24 | F24-18 | 4187 | NS2A | 0.0267 | A:4035;G:111;C:0;T:0;total:4146 | iSNV |
| F24 | F24-18 | 4697 | NS3 | 0.1396 | A:0;G:1;C:1432;T:8824;total:10257 | iSNV |
| F24 | F24-18 | 5952 | NS3 | 0.1392 | A:0;G:0;C:11296;T:1827;total:13123 | iSNV |
| F24 | F24-18 | 7421 | NS4B | 0.0205 | A:5340;G:112;C:0;T:0;total:5452 | iSNV |
| F24 | F24-18 | 8243 | NS5 | 0.0202 | A:4;G:0;C:9984;T:206;total:10194 | iSNV |
| F24 | F24-18 | 9359 | NS5 | 0.1331 | A:0;G:2;C:1768;T:11509;total:13279 | iSNV |
| F24 | F24-18 | 10133 | NS5 | 0.0216 | A:1;G:0;C:8075;T:179;total:8255 | iSNV |
| F24 | F24-18 | 10259 | NS5 | 0.1482 | A:8724;G:1519;C:0;T:1;total:10244 | iSNV |
| F24 | F24-18 | 10376 | NS5 | 0.0247 | A:231;G:9098;C:1;T:2;total:9332 | iSNV |
| F24 | F24-18 | 10447 | 3'-UTR | 0.0393 | A:0;G:0;C:6560;T:269;total:6829 | iSNV |
| F24 | F24-18 | 10566 | 3'-UTR | 0.1266 | A:0;G:0;C:5055;T:733;total:5788 | iSNV |
| F24 | F24-18 | 10590 | 3'-UTR | 0.0237 | A:0;G:0;C:6121;T:149;total:6270 | iSNV |
| F24 | F24-19 | 340 | C | 0.0287 | A:0;G:0;C:911;T:27;total:938 | iSNV |
| F24 | F24-19 | 353 | C | 0.0765 | A:820;G:68;C:0;T:0;total:888 | iSNV |
| F24 | F24-19 | 645 | M | 0.0679 | A:754;G:55;C:0;T:0;total:809 | iSNV |
| F24 | F24-19 | 998 | E | 0.1491 | A:0;G:0;C:502;T:88;total:590 | iSNV |
| F24 | F24-19 | 1218 | E | 0.8886 | A:0;G:0;C:81;T:646;total:727 | iSNV |
| F24 | F24-19 | 1430 | E | 0.0395 | A:0;G:0;C:777;T:32;total:809 | iSNV |
| F24 | F24-19 | 1459 | E | 0.1613 | A:127;G:0;C:659;T:1;total:787 | iSNV |
| F24 | F24-19 | 3278 | NS1 | 0.0404 | A:0;G:0;C:32;T:759;total:791 | iSNV |
| F24 | F24-19 | 3646 | NS1 | 0.1777 | A:0;G:0;C:80;T:370;total:450 | iSNV |
| F24 | F24-19 | 3869 | NS2A | 0.8424 | A:0;G:0;C:137;T:732;total:869 | iSNV |
| F24 | F24-19 | 4442 | NS2B | 0.0229 | A:0;G:0;C:15;T:639;total:654 | iSNV |
| F24 | F24-19 | 4697 | NS3 | 0.0586 | A:0;G:0;C:55;T:882;total:937 | iSNV |
| F24 | F24-19 | 5813 | NS3 | 0.0556 | A:0;G:0;C:832;T:49;total:881 | iSNV |
| F24 | F24-19 | 5927 | NS3 | 0.0555 | A:0;G:0;C:748;T:44;total:792 | iSNV |
| F24 | F24-19 | 5952 | NS3 | 0.0697 | A:0;G:0;C:840;T:63;total:903 | iSNV |
| F24 | F24-19 | 6203 | NS3 | 0.0388 | A:644;G:26;C:0;T:0;total:670 | iSNV |
| F24 | F24-19 | 6413 | NS3 | 0.0455 | A:0;G:0;C:35;T:734;total:769 | iSNV |
| F24 | F24-19 | 9359 | NS5 | 0.0601 | A:0;G:0;C:57;T:891;total:948 | iSNV |
| F24 | F24-19 | 9452 | NS5 | 0.0535 | A:44;G:777;C:0;T:0;total:821 | iSNV |
| F24 | F24-19 | 9584 | NS5 | 0.0239 | A:0;G:0;C:611;T:15;total:626 | iSNV |
| F24 | F24-19 | 9818 | NS5 | 0.0444 | A:0;G:0;C:27;T:581;total:608 | iSNV |
| F24 | F24-19 | 10022 | NS5 | 0.0749 | A:0;G:0;C:52;T:642;total:694 | iSNV |
| F24 | F24-19 | 10259 | NS5 | 0.135 | A:724;G:113;C:0;T:0;total:837 | iSNV |
| F24 | F24-19 | 10358 | NS5 | 0.032 | A:0;G:0;C:635;T:21;total:656 | iSNV |
| F24 | F24-19 | 10447 | 3'-UTR | 0.1001 | A:0;G:0;C:458;T:51;total:509 | iSNV |
| F24 | F24-19 | 10566 | 3'-UTR | 0.0604 | A:0;G:0;C:513;T:33;total:546 | iSNV |
| F24 | F24-19 | 10589 | 3'-UTR | 0.021 | A:0;G:12;C:0;T:559;total:571 | iSNV |
| F24 | F24-2 | 803 | M | 0.0223 | A:0;G:612;C:0;T:14;total:626 | iSNV |
| F24 | F24-2 | 998 | E | 0.0817 | A:0;G:0;C:539;T:48;total:587 | iSNV |
| F24 | F24-2 | 1218 | E | 0.9486 | A:0;G:0;C:38;T:700;total:738 | iSNV |
| F24 | F24-2 | 1413 | E | 0.0356 | A:731;G:27;C:0;T:0;total:758 | iSNV |

| | | | | | | |
|---|---|---|---|---|---|---|
| F24 | F24-2 | 1430 | E | 0.0251 | A:0;G:0;C:736;T:19;total:755 | iSNV |
| F24 | F24-2 | 3693 | NS1 | 0.02 | A:9;G:0;C:0;T:440;total:449 | iSNV |
| F24 | F24-2 | 3869 | NS2A | 0.9019 | A:0;G:0;C:90;T:827;total:917 | iSNV |
| F24 | F24-2 | 4187 | NS2A | 0.0394 | A:414;G:17;C:0;T:0;total:431 | iSNV |
| F24 | F24-2 | 4697 | NS3 | 0.0566 | A:0;G:0;C:51;T:850;total:901 | iSNV |
| F24 | F24-2 | 5729 | NS3 | 0.0211 | A:18;G:834;C:0;T:0;total:852 | iSNV |
| F24 | F24-2 | 5952 | NS3 | 0.0678 | A:0;G:0;C:714;T:52;total:766 | iSNV |
| F24 | F24-2 | 7985 | NS5 | 0.0281 | A:0;G:0;C:655;T:19;total:674 | iSNV |
| F24 | F24-2 | 9350 | NS5 | 0.0926 | A:0;G:0;C:744;T:76;total:820 | iSNV |
| F24 | F24-2 | 9359 | NS5 | 0.0535 | A:0;G:0;C:44;T:777;total:821 | iSNV |
| F24 | F24-2 | 9818 | NS5 | 0.0226 | A:0;G:0;C:13;T:561;total:574 | iSNV |
| F24 | F24-2 | 10259 | NS5 | 0.0618 | A:865;G:57;C:0;T:0;total:922 | iSNV |
| F24 | F24-2 | 10376 | NS5 | 0.0229 | A:14;G:596;C:0;T:0;total:610 | iSNV |
| F24 | F24-2 | 10566 | 3'-UTR | 0.0508 | A:0;G:0;C:616;T:33;total:649 | iSNV |
| F24 | F24-20 | 353 | C | 0.0441 | A:2771;G:128;C:0;T:0;total:2899 | iSNV |
| F24 | F24-20 | 563 | M | 0.0263 | A:69;G:2547;C:0;T:0;total:2616 | iSNV |
| F24 | F24-20 | 645 | M | 0.0421 | A:2565;G:113;C:0;T:0;total:2678 | iSNV |
| F24 | F24-20 | 998 | E | 0.1896 | A:0;G:0;C:2120;T:496;total:2616 | iSNV |
| F24 | F24-20 | 1136 | E | 0.0251 | A:0;G:0;C:68;T:2632;total:2700 | iSNV |
| F24 | F24-20 | 1218 | E | 0.884 | A:0;G:0;C:340;T:2589;total:2929 | iSNV |
| F24 | F24-20 | 1721 | E | 0.0487 | A:2830;G:145;C:0;T:0;total:2975 | iSNV |
| F24 | F24-20 | 1772 | E | 0.0281 | A:0;G:92;C:0;T:3175;total:3267 | iSNV |
| F24 | F24-20 | 2009 | E | 0.0362 | A:0;G:0;C:2231;T:84;total:2315 | iSNV |
| F24 | F24-20 | 2357 | E | 0.0209 | A:2146;G:46;C:0;T:0;total:2192 | iSNV |
| F24 | F24-20 | 2372 | E | 0.0237 | A:0;G:0;C:2182;T:53;total:2235 | iSNV |
| F24 | F24-20 | 2531 | NS1 | 0.0253 | A:0;G:0;C:63;T:2427;total:2490 | iSNV |
| F24 | F24-20 | 3572 | NS1 | 0.0268 | A:0;G:0;C:2214;T:61;total:2275 | iSNV |
| F24 | F24-20 | 3869 | NS2A | 0.7498 | A:0;G:0;C:670;T:2007;total:2677 | iSNV |
| F24 | F24-20 | 3962 | NS2A | 0.0281 | A:0;G:0;C:2865;T:83;total:2948 | iSNV |
| F24 | F24-20 | 4187 | NS2A | 0.0204 | A:1482;G:31;C:0;T:0;total:1513 | iSNV |
| F24 | F24-20 | 4697 | NS3 | 0.1374 | A:0;G:0;C:419;T:2629;total:3048 | iSNV |
| F24 | F24-20 | 4712 | NS3 | 0.0256 | A:78;G:2959;C:0;T:0;total:3037 | iSNV |
| F24 | F24-20 | 5311 | NS3 | 0.0279 | A:0;G:0;C:2361;T:68;total:2429 | iSNV |
| F24 | F24-20 | 5952 | NS3 | 0.1399 | A:0;G:0;C:2668;T:434;total:3102 | iSNV |
| F24 | F24-20 | 8190 | NS5 | 0.0277 | A:0;G:0;C:74;T:2590;total:2664 | iSNV |
| F24 | F24-20 | 9359 | NS5 | 0.1248 | A:0;G:0;C:396;T:2775;total:3171 | iSNV |
| F24 | F24-20 | 9446 | NS5 | 0.0299 | A:3203;G:99;C:0;T:0;total:3302 | iSNV |
| F24 | F24-20 | 10259 | NS5 | 0.1929 | A:2468;G:590;C:0;T:0;total:3058 | iSNV |
| F24 | F24-20 | 10421 | 3'-UTR | 0.0301 | A:2249;G:70;C:0;T:0;total:2319 | iSNV |
| F24 | F24-20 | 10447 | 3'-UTR | 0.0549 | A:0;G:0;C:2063;T:120;total:2183 | iSNV |
| F24 | F24-20 | 10566 | 3'-UTR | 0.1349 | A:0;G:0;C:2109;T:329;total:2438 | iSNV |
| F24 | F24-20 | 10617 | 3'-UTR | 0.0447 | A:2326;G:109;C:1;T:1;total:2437 | iSNV |
| F24 | F24-20 | 10663 | 3'-UTR | 0.0226 | A:0;G:0;C:2802;T:65;total:2867 | iSNV |
| F24 | F24-21 | 897 | M | 0.0357 | A:0;G:1;C:4641;T:172;total:4814 | iSNV |
| F24 | F24-21 | 998 | E | 0.0716 | A:2;G:0;C:4894;T:378;total:5274 | iSNV |
| F24 | F24-21 | 1043 | E | 0.0206 | A:0;G:0;C:5685;T:120;total:5805 | iSNV |
| F24 | F24-21 | 1218 | E | 0.9625 | A:0;G:0;C:234;T:5992;total:6226 | iSNV |
| F24 | F24-21 | 1430 | E | 0.0231 | A:1;G:1;C:6664;T:158;total:6824 | iSNV |
| F24 | F24-21 | 2448 | E | 0.025 | A:0;G:0;C:3887;T:100;total:3987 | iSNV |
| F24 | F24-21 | 3300 | NS1 | 0.0219 | A:0;G:0;C:172;T:7650;total:7822 | iSNV |
| F24 | F24-21 | 3661 | NS1 | 0.0406 | A:0;G:0;C:166;T:3918;total:4084 | iSNV |
| F24 | F24-21 | 3869 | NS2A | 0.9151 | A:1;G:0;C:630;T:6782;total:7413 | iSNV |
| F24 | F24-21 | 4093 | NS2A | 0.0317 | A:5209;G:171;C:0;T:0;total:5380 | iSNV |
| F24 | F24-21 | 4284 | NS2B | 0.0213 | A:0;G:0;C:121;T:5553;total:5674 | iSNV |
| F24 | F24-21 | 4697 | NS3 | 0.0534 | A:0;G:1;C:408;T:7231;total:7640 | iSNV |
| F24 | F24-21 | 5952 | NS3 | 0.0509 | A:0;G:0;C:8521;T:457;total:8978 | iSNV |
| F24 | F24-21 | 6004 | NS3 | 0.0352 | A:2;G:0;C:7804;T:285;total:8091 | iSNV |
| F24 | F24-21 | 6206 | NS3 | 0.0609 | A:0;G:0;C:5097;T:331;total:5428 | iSNV |
| F24 | F24-21 | 6950 | NS4A | 0.0474 | A:1;G:0;C:4734;T:236;total:4971 | iSNV |
| F24 | F24-21 | 7625 | NS4B | 0.0286 | A:0;G:0;C:6941;T:205;total:7146 | iSNV |
| F24 | F24-21 | 7697 | NS5 | 0.036 | A:2;G:7018;C:4;T:263;total:7287 | iSNV |
| F24 | F24-21 | 8194 | NS5 | 0.0323 | A:6724;G:225;C:0;T:1;total:6950 | iSNV |
| F24 | F24-21 | 9065 | NS5 | 0.0317 | A:183;G:5576;C:0;T:2;total:5761 | iSNV |
| F24 | F24-21 | 9359 | NS5 | 0.0444 | A:1;G:1;C:412;T:8851;total:9265 | iSNV |
| F24 | F24-21 | 9860 | NS5 | 0.0205 | A:0;G:2;C:115;T:5485;total:5602 | iSNV |
| F24 | F24-21 | 10259 | NS5 | 0.0668 | A:7010;G:502;C:0;T:0;total:7512 | iSNV |
| F24 | F24-21 | 10358 | NS5 | 0.0273 | A:0;G:0;C:6586;T:185;total:6771 | iSNV |
| F24 | F24-21 | 10447 | 3'-UTR | 0.0322 | A:0;G:0;C:4769;T:159;total:4928 | iSNV |
| F24 | F24-21 | 10566 | 3'-UTR | 0.0533 | A:0;G:1;C:3973;T:224;total:4198 | iSNV |
| F24 | F24-21 | 10578 | 3'-UTR | 0.0346 | A:0;G:1;C:149;T:4147;total:4297 | iSNV |
| F24 | F24-21 | 10766 | 3'-UTR | 0.0289 | A:149;G:4996;C:0;T:1;total:5146 | iSNV |
| F24 | F24-22 | 353 | C | 0.0216 | A:1902;G:42;C:0;T:0;total:1944 | iSNV |
| F24 | F24-22 | 645 | M | 0.0213 | A:1927;G:42;C:0;T:1;total:1970 | iSNV |
| F24 | F24-22 | 998 | E | 0.0944 | A:0;G:1;C:1217;T:127;total:1345 | iSNV |
| F24 | F24-22 | 1218 | E | 0.9574 | A:0;G:0;C:72;T:1617;total:1689 | iSNV |
| F24 | F24-22 | 1397 | E | 0.04 | A:1;G:0;C:1868;T:78;total:1947 | iSNV |
| F24 | F24-22 | 1430 | E | 0.026 | A:0;G:0;C:1718;T:46;total:1764 | iSNV |
| F24 | F24-22 | 1461 | E | 0.1033 | A:0;G:0;C:166;T:1440;total:1606 | iSNV |
| F24 | F24-22 | 1838 | E | 0.025 | A:0;G:0;C:44;T:1709;total:1753 | iSNV |
| F24 | F24-22 | 3137 | NS1 | 0.0323 | A:0;G:0;C:2394;T:80;total:2474 | iSNV |
| F24 | F24-22 | 3401 | NS1 | 0.0231 | A:0;G:0;C:1600;T:38;total:1638 | iSNV |
| F24 | F24-22 | 3671 | NS1 | 0.0492 | A:0;G:1;C:43;T:829;total:873 | iSNV |

| | | | | | | |
|---|---|---|---|---|---|---|
| F24 | F24-22 | 3869 | NS2A | 0.8955 | A:0;G:0;C:232;T:1988;total:2220 | iSNV |
| F24 | F24-22 | 4319 | NS2B | 0.0297 | A:60;G:1955;C:0;T:0;total:2015 | iSNV |
| F24 | F24-22 | 4594 | NS2B | 0.0303 | A:2235;G:70;C:0;T:0;total:2305 | iSNV |
| F24 | F24-22 | 4697 | NS3 | 0.0693 | A:0;G:1;C:146;T:1958;total:2105 | iSNV |
| F24 | F24-22 | 5654 | NS3 | 0.0311 | A:0;G:0;C:2146;T:69;total:2215 | iSNV |
| F24 | F24-22 | 5952 | NS3 | 0.0747 | A:0;G:0;C:2118;T:171;total:2289 | iSNV |
| F24 | F24-22 | 6157 | NS3 | 0.0213 | A:0;G:0;C:1330;T:29;total:1359 | iSNV |
| F24 | F24-22 | 7784 | NS5 | 0.1206 | A:0;G:0;C:1261;T:173;total:1434 | iSNV |
| F24 | F24-22 | 8257 | NS5 | 0.0286 | A:1932;G:57;C:0;T:0;total:1989 | iSNV |
| F24 | F24-22 | 8744 | NS5 | 0.021 | A:0;G:1;C:31;T:1439;total:1471 | iSNV |
| F24 | F24-22 | 9359 | NS5 | 0.0756 | A:0;G:0;C:158;T:1931;total:2089 | iSNV |
| F24 | F24-22 | 10046 | NS5 | 0.021 | A:1625;G:35;C:0;T:0;total:1660 | iSNV |
| F24 | F24-22 | 10259 | NS5 | 0.1009 | A:1834;G:206;C:0;T:0;total:2040 | iSNV |
| F24 | F24-22 | 10447 | 3'-UTR | 0.0347 | A:0;G:0;C:1165;T:42;total:1207 | iSNV |
| F24 | F24-22 | 10566 | 3'-UTR | 0.0744 | A:0;G:0;C:1132;T:91;total:1223 | iSNV |
| F24 | F24-22 | 10589 | 3'-UTR | 0.0248 | A:0;G:32;C:1;T:1256;total:1289 | iSNV |
| F24 | F24-23 | 353 | C | 0.0502 | A:1078;G:57;C:0;T:0;total:1135 | iSNV |
| F24 | F24-23 | 563 | M | 0.0776 | A:80;G:950;C:0;T:0;total:1030 | iSNV |
| F24 | F24-23 | 645 | M | 0.0441 | A:1039;G:48;C:0;T:0;total:1087 | iSNV |
| F24 | F24-23 | 752 | M | 0.0262 | A:1152;G:31;C:0;T:0;total:1183 | iSNV |
| F24 | F24-23 | 803 | M | 0.089 | A:0;G:849;C:0;T:83;total:932 | iSNV |
| F24 | F24-23 | 909 | M | 0.0353 | A:37;G:0;C:1010;T:0;total:1047 | iSNV |
| F24 | F24-23 | 998 | E | 0.1797 | A:0;G:0;C:817;T:179;total:996 | iSNV |
| F24 | F24-23 | 1007 | E | 0.0275 | A:0;G:0;C:1060;T:30;total:1090 | iSNV |
| F24 | F24-23 | 1117 | E | 0.0641 | A:1108;G:76;C:0;T:0;total:1184 | iSNV |
| F24 | F24-23 | 1218 | E | 0.7649 | A:1;G:0;C:270;T:877;total:1148 | iSNV |
| F24 | F24-23 | 1416 | E | 0.1157 | A:1100;G:144;C:0;T:0;total:1244 | iSNV |
| F24 | F24-23 | 1430 | E | 0.0329 | A:0;G:0;C:1231;T:42;total:1273 | iSNV |
| F24 | F24-23 | 1772 | E | 0.0706 | A:0;G:95;C:0;T:1250;total:1345 | iSNV |
| F24 | F24-23 | 2008 | E | 0.0289 | A:28;G:938;C:0;T:0;total:966 | iSNV |
| F24 | F24-23 | 2372 | E | 0.0674 | A:0;G:0;C:830;T:60;total:890 | iSNV |
| F24 | F24-23 | 2531 | NS1 | 0.0784 | A:0;G:0;C:77;T:905;total:982 | iSNV |
| F24 | F24-23 | 2570 | NS1 | 0.04 | A:36;G:864;C:0;T:0;total:900 | iSNV |
| F24 | F24-23 | 3572 | NS1 | 0.0665 | A:0;G:0;C:757;T:54;total:811 | iSNV |
| F24 | F24-23 | 3869 | NS2A | 0.6411 | A:0;G:0;C:355;T:634;total:989 | iSNV |
| F24 | F24-23 | 3962 | NS2A | 0.0828 | A:0;G:0;C:1007;T:91;total:1098 | iSNV |
| F24 | F24-23 | 4697 | NS3 | 0.1546 | A:1;G:0;C:182;T:994;total:1177 | iSNV |
| F24 | F24-23 | 4712 | NS3 | 0.0813 | A:97;G:1096;C:0;T:0;total:1193 | iSNV |
| F24 | F24-23 | 5311 | NS3 | 0.075 | A:0;G:0;C:887;T:72;total:959 | iSNV |
| F24 | F24-23 | 5480 | NS3 | 0.0235 | A:0;G:0;C:1284;T:31;total:1315 | iSNV |
| F24 | F24-23 | 5952 | NS3 | 0.1042 | A:0;G:0;C:1065;T:124;total:1189 | iSNV |
| F24 | F24-23 | 7460 | NS4B | 0.0582 | A:44;G:711;C:0;T:0;total:755 | iSNV |
| F24 | F24-23 | 7527 | NS4B | 0.0251 | A:890;G:23;C:0;T:0;total:913 | iSNV |
| F24 | F24-23 | 7735 | NS5 | 0.0239 | A:21;G:856;C:0;T:0;total:877 | iSNV |
| F24 | F24-23 | 7744 | NS5 | 0.0654 | A:814;G:57;C:0;T:0;total:871 | iSNV |
| F24 | F24-23 | 8282 | NS5 | 0.0738 | A:0;G:0;C:76;T:953;total:1029 | iSNV |
| F24 | F24-23 | 8518 | NS5 | 0.0751 | A:1181;G:0;C:0;T:96;total:1277 | iSNV |
| F24 | F24-23 | 8900 | NS5 | 0.0955 | A:767;G:0;C:81;T:0;total:848 | iSNV |
| F24 | F24-23 | 9359 | NS5 | 0.1081 | A:0;G:0;C:138;T:1138;total:1276 | iSNV |
| F24 | F24-23 | 9446 | NS5 | 0.0747 | A:1237;G:100;C:0;T:0;total:1337 | iSNV |
| F24 | F24-23 | 10013 | NS5 | 0.0385 | A:0;G:0;C:949;T:38;total:987 | iSNV |
| F24 | F24-23 | 10259 | NS5 | 0.1602 | A:1053;G:201;C:0;T:0;total:1254 | iSNV |
| F24 | F24-23 | 10428 | 3'-UTR | 0.1113 | A:0;G:0;C:894;T:112;total:1006 | iSNV |
| F24 | F24-23 | 10447 | 3'-UTR | 0.05 | A:0;G:0;C:874;T:46;total:920 | iSNV |
| F24 | F24-23 | 10566 | 3'-UTR | 0.1221 | A:0;G:0;C:877;T:122;total:999 | iSNV |
| F24 | F24-24 | 353 | C | 0.0529 | A:2792;G:156;C:0;T:0;total:2948 | iSNV |
| F24 | F24-24 | 645 | M | 0.0569 | A:2467;G:149;C:1;T:0;total:2617 | iSNV |
| F24 | F24-24 | 803 | M | 0.1184 | A:0;G:2062;C:0;T:277;total:2339 | iSNV |
| F24 | F24-24 | 998 | E | 0.2284 | A:0;G:0;C:1776;T:526;total:2302 | iSNV |
| F24 | F24-24 | 1013 | E | 0.0301 | A:2280;G:0;C:71;T:0;total:2351 | iSNV |
| F24 | F24-24 | 1117 | E | 0.0455 | A:2449;G:117;C:0;T:0;total:2566 | iSNV |
| F24 | F24-24 | 1218 | E | 0.7712 | A:0;G:1;C:612;T:2061;total:2674 | iSNV |
| F24 | F24-24 | 1430 | E | 0.0384 | A:1;G:0;C:2749;T:110;total:2860 | iSNV |
| F24 | F24-24 | 1440 | E | 0.0205 | A:0;G:0;C:56;T:2673;total:2729 | iSNV |
| F24 | F24-24 | 1892 | E | 0.0267 | A:2397;G:66;C:0;T:0;total:2463 | iSNV |
| F24 | F24-24 | 2277 | E | 0.0319 | A:0;G:0;C:76;T:2301;total:2377 | iSNV |
| F24 | F24-24 | 3110 | NS1 | 0.048 | A:2732;G:138;C:0;T:0;total:2870 | iSNV |
| F24 | F24-24 | 3257 | NS1 | 0.0324 | A:0;G:0;C:107;T:3188;total:3295 | iSNV |
| F24 | F24-24 | 3869 | NS2A | 0.6412 | A:0;G:0;C:970;T:1733;total:2703 | iSNV |
| F24 | F24-24 | 4073 | NS2A | 0.0224 | A:0;G:0;C:2261;T:52;total:2313 | iSNV |
| F24 | F24-24 | 4124 | NS2A | 0.0645 | A:1449;G:100;C:0;T:0;total:1549 | iSNV |
| F24 | F24-24 | 4663 | NS3 | 0.0307 | A:0;G:0;C:2865;T:91;total:2956 | iSNV |
| F24 | F24-24 | 4697 | NS3 | 0.1754 | A:0;G:0;C:551;T:2589;total:3140 | iSNV |
| F24 | F24-24 | 5465 | NS3 | 0.0364 | A:3015;G:114;C:0;T:0;total:3129 | iSNV |
| F24 | F24-24 | 5952 | NS3 | 0.1655 | A:0;G:0;C:2717;T:539;total:3256 | iSNV |
| F24 | F24-24 | 7735 | NS5 | 0.0797 | A:204;G:2354;C:0;T:0;total:2558 | iSNV |
| F24 | F24-24 | 8518 | NS5 | 0.1312 | A:2748;G:0;C:0;T:415;total:3163 | iSNV |
| F24 | F24-24 | 8696 | NS5 | 0.02 | A:1;G:0;C:2151;T:44;total:2196 | iSNV |
| F24 | F24-24 | 9359 | NS5 | 0.1451 | A:0;G:0;C:450;T:2651;total:3101 | iSNV |
| F24 | F24-24 | 10259 | NS5 | 0.2375 | A:2137;G:666;C:0;T:1;total:2804 | iSNV |
| F24 | F24-24 | 10419 | 3'-UTR | 0.0213 | A:1;G:43;C:1950;T:24;total:2018 | iSNV |
| F24 | F24-24 | 10447 | 3'-UTR | 0.0809 | A:0;G:1;C:1690;T:149;total:1840 | iSNV |

| | | | | | | |
|---|---|---|---|---|---|---|
| F24 | F24-24 | 10566 | 3'-UTR | 0.162 | A:0;G:0;C:1500;T:290;total:1790 | iSNV |
| F24 | F24-24 | 10579 | 3'-UTR | 0.0532 | A:0;G:0;C:1761;T:99;total:1860 | iSNV |
| F24 | F24-25 | 323 | C | 0.024 | A:122;G:4946;C:0;T:4;total:5072 | iSNV |
| F24 | F24-25 | 443 | C | 0.0367 | A:1;G:4749;C:0;T:181;total:4931 | iSNV |
| F24 | F24-25 | 491 | M | 0.0283 | A:2;G:1;C:159;T:5446;total:5608 | iSNV |
| F24 | F24-25 | 998 | E | 0.1324 | A:1;G:0;C:3425;T:523;total:3949 | iSNV |
| F24 | F24-25 | 1117 | E | 0.0422 | A:4059;G:179;C:0;T:0;total:4238 | iSNV |
| F24 | F24-25 | 1218 | E | 0.9452 | A:0;G:0;C:238;T:4104;total:4342 | iSNV |
| F24 | F24-25 | 2213 | E | 0.0214 | A:115;G:5246;C:0;T:0;total:5361 | iSNV |
| F24 | F24-25 | 2855 | NS1 | 0.033 | A:210;G:6145;C:0;T:1;total:6356 | iSNV |
| F24 | F24-25 | 2948 | NS1 | 0.0229 | A:0;G:0;C:5162;T:121;total:5283 | iSNV |
| F24 | F24-25 | 3356 | NS1 | 0.0382 | A:157;G:3944;C:0;T:0;total:4101 | iSNV |
| F24 | F24-25 | 3869 | NS2A | 0.8532 | A:0;G:0;C:813;T:4722;total:5535 | iSNV |
| F24 | F24-25 | 4697 | NS3 | 0.1034 | A:0;G:0;C:631;T:5471;total:6102 | iSNV |
| F24 | F24-25 | 5558 | NS3 | 0.0403 | A:221;G:5260;C:0;T:1;total:5482 | iSNV |
| F24 | F24-25 | 5952 | NS3 | 0.1002 | A:1;G:0;C:5484;T:611;total:6096 | iSNV |
| F24 | F24-25 | 9359 | NS5 | 0.0868 | A:1;G:1;C:528;T:5546;total:6076 | iSNV |
| F24 | F24-25 | 9401 | NS5 | 0.0227 | A:1;G:0;C:5589;T:130;total:5720 | iSNV |
| F24 | F24-25 | 9818 | NS5 | 0.086 | A:0;G:0;C:349;T:3706;total:4055 | iSNV |
| F24 | F24-25 | 10259 | NS5 | 0.1239 | A:4228;G:598;C:0;T:0;total:4826 | iSNV |
| F24 | F24-25 | 10428 | 3'-UTR | 0.0471 | A:0;G:0;C:3133;T:155;total:3288 | iSNV |
| F24 | F24-25 | 10447 | 3'-UTR | 0.043 | A:0;G:0;C:2954;T:133;total:3087 | iSNV |
| F24 | F24-25 | 10566 | 3'-UTR | 0.1103 | A:1;G:0;C:3248;T:403;total:3652 | iSNV |
| F24 | F24-26 | 803 | M | 0.0427 | A:0;G:582;C:0;T:26;total:608 | iSNV |
| F24 | F24-26 | 998 | E | 0.1518 | A:0;G:0;C:447;T:80;total:527 | iSNV |
| F24 | F24-26 | 1043 | E | 0.0357 | A:0;G:0;C:594;T:22;total:616 | iSNV |
| F24 | F24-26 | 1218 | E | 0.9125 | A:0;G:0;C:54;T:563;total:617 | iSNV |
| F24 | F24-26 | 3257 | NS1 | 0.0252 | A:0;G:0;C:15;T:579;total:594 | iSNV |
| F24 | F24-26 | 3869 | NS2A | 0.8331 | A:1;G:1;C:116;T:577;total:695 | iSNV |
| F24 | F24-26 | 4187 | NS2A | 0.0325 | A:475;G:16;C:0;T:0;total:491 | iSNV |
| F24 | F24-26 | 4697 | NS3 | 0.1202 | A:0;G:0;C:107;T:783;total:890 | iSNV |
| F24 | F24-26 | 5952 | NS3 | 0.1061 | A:0;G:0;C:606;T:72;total:678 | iSNV |
| F24 | F24-26 | 6626 | NS4A | 0.0333 | A:0;G:0;C:522;T:18;total:540 | iSNV |
| F24 | F24-26 | 7172 | NS4A | 0.0231 | A:0;G:0;C:18;T:761;total:779 | iSNV |
| F24 | F24-26 | 7735 | NS5 | 0.0339 | A:14;G:398;C:0;T:0;total:412 | iSNV |
| F24 | F24-26 | 8518 | NS5 | 0.0381 | A:580;G:0;C:0;T:23;total:603 | iSNV |
| F24 | F24-26 | 9359 | NS5 | 0.1174 | A:0;G:0;C:70;T:526;total:596 | iSNV |
| F24 | F24-26 | 10259 | NS5 | 0.1183 | A:693;G:93;C:0;T:0;total:786 | iSNV |
| F24 | F24-26 | 10428 | 3'-UTR | 0.02 | A:0;G:0;C:391;T:8;total:399 | iSNV |
| F24 | F24-26 | 10429 | 3'-UTR | 0.0255 | A:381;G:10;C:0;T:0;total:391 | iSNV |
| F24 | F24-26 | 10447 | 3'-UTR | 0.0365 | A:0;G:0;C:369;T:14;total:383 | iSNV |
| F24 | F24-26 | 10663 | 3'-UTR | 0.0294 | A:0;G:1;C:659;T:20;total:680 | iSNV |
| F24 | F24-26 | 10925 | 3'-UTR | 0.0612 | A:0;G:0;C:6;T:92;total:98 | iSNV |
| F24 | F24-27 | 998 | E | 0.0789 | A:1;G:0;C:1668;T:143;total:1812 | iSNV |
| F24 | F24-27 | 1136 | E | 0.0419 | A:0;G:0;C:84;T:1920;total:2004 | iSNV |
| F24 | F24-27 | 1218 | E | 0.975 | A:0;G:0;C:52;T:2027;total:2079 | iSNV |
| F24 | F24-27 | 2664 | NS1 | 0.033 | A:0;G:0;C:2396;T:82;total:2478 | iSNV |
| F24 | F24-27 | 3869 | NS2A | 0.9101 | A:0;G:0;C:207;T:2095;total:2302 | iSNV |
| F24 | F24-27 | 4697 | NS3 | 0.082 | A:0;G:0;C:201;T:2250;total:2451 | iSNV |
| F24 | F24-27 | 5952 | NS3 | 0.0705 | A:0;G:0;C:2634;T:200;total:2834 | iSNV |
| F24 | F24-27 | 5963 | NS3 | 0.0216 | A:0;G:0;C:60;T:2717;total:2777 | iSNV |
| F24 | F24-27 | 7626 | NS4B | 0.0241 | A:0;G:54;C:0;T:2184;total:2238 | iSNV |
| F24 | F24-27 | 7892 | NS5 | 0.0312 | A:0;G:2570;C:1;T:83;total:2654 | iSNV |
| F24 | F24-27 | 9359 | NS5 | 0.0704 | A:0;G:1;C:190;T:2505;total:2696 | iSNV |
| F24 | F24-27 | 9818 | NS5 | 0.0499 | A:0;G:0;C:98;T:1863;total:1961 | iSNV |
| F24 | F24-27 | 10259 | NS5 | 0.0863 | A:2285;G:216;C:0;T:0;total:2501 | iSNV |
| F24 | F24-27 | 10428 | 3'-UTR | 0.0211 | A:0;G:0;C:1805;T:39;total:1844 | iSNV |
| F24 | F24-27 | 10447 | 3'-UTR | 0.0244 | A:0;G:1;C:1598;T:40;total:1639 | iSNV |
| F24 | F24-27 | 10566 | 3'-UTR | 0.0616 | A:0;G:0;C:1355;T:89;total:1444 | iSNV |
| F24 | F24-27 | 10572 | 3'-UTR | 0.024 | A:1421;G:35;C:0;T:0;total:1456 | iSNV |
| F24 | F24-28 | 353 | C | 0.0685 | A:1617;G:119;C:0;T:0;total:1736 | iSNV |
| F24 | F24-28 | 645 | M | 0.0637 | A:1395;G:95;C:0;T:0;total:1490 | iSNV |
| F24 | F24-28 | 803 | M | 0.1107 | A:0;G:1068;C:0;T:133;total:1201 | iSNV |
| F24 | F24-28 | 869 | M | 0.0218 | A:30;G:0;C:0;T:1345;total:1375 | iSNV |
| F24 | F24-28 | 897 | M | 0.0447 | A:0;G:1;C:1044;T:49;total:1094 | iSNV |
| F24 | F24-28 | 946 | M | 0.0383 | A:0;G:47;C:0;T:1177;total:1224 | iSNV |
| F24 | F24-28 | 998 | E | 0.1469 | A:0;G:0;C:1010;T:174;total:1184 | iSNV |
| F24 | F24-28 | 1117 | E | 0.0332 | A:1310;G:45;C:0;T:0;total:1355 | iSNV |
| F24 | F24-28 | 1218 | E | 0.7856 | A:0;G:1;C:314;T:1149;total:1464 | iSNV |
| F24 | F24-28 | 1430 | E | 0.0227 | A:1;G:0;C:1414;T:33;total:1448 | iSNV |
| F24 | F24-28 | 1478 | E | 0.0638 | A:0;G:0;C:70;T:1026;total:1096 | iSNV |
| F24 | F24-28 | 1817 | E | 0.021 | A:1531;G:33;C:0;T:0;total:1564 | iSNV |
| F24 | F24-28 | 1898 | E | 0.0232 | A:1303;G:31;C:0;T:0;total:1334 | iSNV |
| F24 | F24-28 | 3869 | NS2A | 0.7565 | A:0;G:0;C:456;T:1416;total:1872 | iSNV |
| F24 | F24-28 | 4697 | NS3 | 0.0751 | A:0;G:0;C:136;T:1673;total:1809 | iSNV |
| F24 | F24-28 | 4742 | NS3 | 0.0441 | A:0;G:0;C:80;T:1731;total:1811 | iSNV |
| F24 | F24-28 | 5665 | NS3 | 0.0529 | A:0;G:0;C:1718;T:96;total:1814 | iSNV |
| F24 | F24-28 | 5952 | NS3 | 0.078 | A:0;G:0;C:1808;T:153;total:1961 | iSNV |
| F24 | F24-28 | 6455 | NS3 | 0.0246 | A:1423;G:36;C:0;T:0;total:1459 | iSNV |
| F24 | F24-28 | 7592 | NS4B | 0.0385 | A:0;G:0;C:1371;T:55;total:1426 | iSNV |
| F24 | F24-28 | 7735 | NS5 | 0.0745 | A:118;G:1464;C:0;T:0;total:1582 | iSNV |
| F24 | F24-28 | 8518 | NS5 | 0.1107 | A:1509;G:0;C:0;T:188;total:1697 | iSNV |

| | | | | | | |
|---|---|---|---|---|---|---|
| F24 | F24-28 | 8648 | NS5 | 0.0275 | A:0;G:0;C:1058;T:30;total:1088 | iSNV |
| F24 | F24-28 | 8789 | NS5 | 0.0293 | A:1257;G:38;C:0;T:0;total:1295 | iSNV |
| F24 | F24-28 | 9359 | NS5 | 0.0598 | A:0;G:0;C:111;T:1745;total:1856 | iSNV |
| F24 | F24-28 | 10259 | NS5 | 0.1317 | A:1226;G:186;C:0;T:0;total:1412 | iSNV |
| F24 | F24-28 | 10447 | 3'-UTR | 0.0521 | A:0;G:0;C:891;T:49;total:940 | iSNV |
| F24 | F24-28 | 10452 | 3'-UTR | 0.0478 | A:895;G:45;C:0;T:0;total:940 | iSNV |
| F24 | F24-28 | 10566 | 3'-UTR | 0.0668 | A:0;G:0;C:908;T:65;total:973 | iSNV |
| F24 | F24-28 | 10589 | 3'-UTR | 0.0212 | A:0;G:22;C:0;T:1015;total:1037 | iSNV |
| F24 | F24-29 | 275 | C | 0.0252 | A:0;G:0;C:386;T:10;total:396 | iSNV |
| F24 | F24-29 | 353 | C | 0.0401 | A:359;G:15;C:0;T:0;total:374 | iSNV |
| F24 | F24-29 | 998 | E | 0.0888 | A:0;G:0;C:277;T:27;total:304 | iSNV |
| F24 | F24-29 | 1117 | E | 0.0239 | A:408;G:10;C:0;T:0;total:418 | iSNV |
| F24 | F24-29 | 1218 | E | 0.8824 | A:0;G:0;C:44;T:330;total:374 | iSNV |
| F24 | F24-29 | 1413 | E | 0.0233 | A:377;G:9;C:0;T:0;total:386 | iSNV |
| F24 | F24-29 | 1430 | E | 0.0383 | A:0;G:0;C:376;T:15;total:391 | iSNV |
| F24 | F24-29 | 2362 | E | 0.0336 | A:0;G:230;C:8;T:0;total:238 | iSNV |
| F24 | F24-29 | 2570 | NS1 | 0.0296 | A:10;G:327;C:0;T:0;total:337 | iSNV |
| F24 | F24-29 | 2627 | NS1 | 0.0464 | A:0;G:0;C:390;T:19;total:409 | iSNV |
| F24 | F24-29 | 3023 | NS1 | 0.0552 | A:19;G:325;C:0;T:0;total:344 | iSNV |
| F24 | F24-29 | 3869 | NS2A | 0.8167 | A:0;G:0;C:66;T:294;total:360 | iSNV |
| F24 | F24-29 | 4187 | NS2A | 0.0434 | A:198;G:9;C:0;T:0;total:207 | iSNV |
| F24 | F24-29 | 4577 | NS2B | 0.0219 | A:0;G:10;C:0;T:446;total:456 | iSNV |
| F24 | F24-29 | 4661 | NS3 | 0.0554 | A:0;G:1;C:391;T:23;total:415 | iSNV |
| F24 | F24-29 | 4697 | NS3 | 0.0513 | A:0;G:0;C:23;T:425;total:448 | iSNV |
| F24 | F24-29 | 4889 | NS3 | 0.0276 | A:0;G:0;C:387;T:11;total:398 | iSNV |
| F24 | F24-29 | 5150 | NS3 | 0.0456 | A:293;G:0;C:0;T:14;total:307 | iSNV |
| F24 | F24-29 | 5597 | NS3 | 0.0321 | A:0;G:0;C:14;T:422;total:436 | iSNV |
| F24 | F24-29 | 5952 | NS3 | 0.063 | A:0;G:0;C:401;T:27;total:428 | iSNV |
| F24 | F24-29 | 6753 | NS4A | 0.0225 | A:0;G:260;C:0;T:6;total:266 | iSNV |
| F24 | F24-29 | 7735 | NS5 | 0.021 | A:5;G:233;C:0;T:0;total:238 | iSNV |
| F24 | F24-29 | 9359 | NS5 | 0.0566 | A:0;G:0;C:21;T:350;total:371 | iSNV |
| F24 | F24-29 | 9557 | NS5 | 0.0215 | A:7;G:318;C:0;T:0;total:325 | iSNV |
| F24 | F24-29 | 10259 | NS5 | 0.1033 | A:347;G:40;C:0;T:0;total:387 | iSNV |
| F24 | F24-29 | 10376 | NS5 | 0.0274 | A:9;G:319;C:0;T:0;total:328 | iSNV |
| F24 | F24-29 | 10447 | 3'-UTR | 0.0566 | A:0;G:0;C:250;T:15;total:265 | iSNV |
| F24 | F24-29 | 10566 | 3'-UTR | 0.0638 | A:0;G:0;C:264;T:18;total:282 | iSNV |
| F24 | F24-29 | 10589 | 3'-UTR | 0.0336 | A:0;G:10;C:0;T:287;total:297 | iSNV |
| F24 | F24-29 | 10800 | 3'-UTR | 0.0211 | A:0;G:0;C:231;T:5;total:236 | iSNV |
| F24 | F24-3 | 353 | C | 0.0462 | A:12801;G:621;C:0;T:1;total:13423 | iSNV |
| F24 | F24-3 | 645 | M | 0.0431 | A:10296;G:464;C:0;T:1;total:10761 | iSNV |
| F24 | F24-3 | 803 | M | 0.0336 | A:2;G:7928;C:2;T:276;total:8208 | iSNV |
| F24 | F24-3 | 996 | E | 0.0295 | A:230;G:7546;C:0;T:1;total:7777 | iSNV |
| F24 | F24-3 | 998 | E | 0.1774 | A:2;G:0;C:6357;T:1372;total:7731 | iSNV |
| F24 | F24-3 | 1117 | E | 0.0442 | A:8004;G:371;C:0;T:1;total:8376 | iSNV |
| F24 | F24-3 | 1218 | E | 0.8953 | A:1;G:3;C:971;T:8298;total:9273 | iSNV |
| F24 | F24-3 | 2465 | E | 0.0332 | A:1;G:0;C:5410;T:186;total:5597 | iSNV |
| F24 | F24-3 | 2654 | NS1 | 0.0248 | A:0;G:3;C:327;T:12829;total:13159 | iSNV |
| F24 | F24-3 | 2780 | NS1 | 0.0247 | A:0;G:3;C:363;T:14274;total:14640 | iSNV |
| F24 | F24-3 | 3300 | NS1 | 0.0337 | A:0;G:2;C:421;T:12044;total:12467 | iSNV |
| F24 | F24-3 | 3350 | NS1 | 0.0234 | A:0;G:1;C:242;T:10068;total:10311 | iSNV |
| F24 | F24-3 | 3858 | NS2A | 0.0239 | A:3;G:0;C:11479;T:282;total:11764 | iSNV |
| F24 | F24-3 | 3869 | NS2A | 0.7932 | A:1;G:1;C:2516;T:9647;total:12165 | iSNV |
| F24 | F24-3 | 4049 | NS2A | 0.0281 | A:0;G:0;C:8253;T:239;total:8492 | iSNV |
| F24 | F24-3 | 4697 | NS3 | 0.1189 | A:0;G:2;C:1349;T:9986;total:11337 | iSNV |
| F24 | F24-3 | 5952 | NS3 | 0.1165 | A:1;G:2;C:13065;T:1724;total:14792 | iSNV |
| F24 | F24-3 | 6236 | NS3 | 0.0211 | A:0;G:1;C:6857;T:148;total:7006 | iSNV |
| F24 | F24-3 | 7625 | NS4B | 0.0334 | A:1;G:1;C:12014;T:416;total:12432 | iSNV |
| F24 | F24-3 | 7735 | NS5 | 0.0256 | A:306;G:11629;C:0;T:1;total:11936 | iSNV |
| F24 | F24-3 | 8518 | NS5 | 0.0353 | A:9891;G:1;C:0;T:363;total:10255 | iSNV |
| F24 | F24-3 | 9230 | NS5 | 0.0216 | A:14469;G:4;C:1;T:320;total:14794 | iSNV |
| F24 | F24-3 | 9359 | NS5 | 0.1166 | A:2;G:0;C:1819;T:13779;total:15600 | iSNV |
| F24 | F24-3 | 10259 | NS5 | 0.1659 | A:9744;G:1939;C:0;T:0;total:11683 | iSNV |
| F24 | F24-3 | 10358 | NS5 | 0.0374 | A:2;G:1;C:10422;T:406;total:10831 | iSNV |
| F24 | F24-3 | 10447 | 3'-UTR | 0.0594 | A:0;G:0;C:7039;T:445;total:7484 | iSNV |
| F24 | F24-3 | 10452 | 3'-UTR | 0.0246 | A:7274;G:184;C:0;T:1;total:7459 | iSNV |
| F24 | F24-3 | 10566 | 3'-UTR | 0.1135 | A:2;G:0;C:4961;T:636;total:5599 | iSNV |
| F24 | F24-30 | 998 | E | 0.0913 | A:0;G:1;C:3092;T:311;total:3404 | iSNV |
| F24 | F24-30 | 1218 | E | 0.9473 | A:0;G:0;C:200;T:3591;total:3791 | iSNV |
| F24 | F24-30 | 1413 | E | 0.2953 | A:3132;G:1313;C:1;T:0;total:4446 | iSNV |
| F24 | F24-30 | 1430 | E | 0.0203 | A:0;G:2;C:4143;T:86;total:4231 | iSNV |
| F24 | F24-30 | 3869 | NS2A | 0.8788 | A:0;G:0;C:507;T:3675;total:4182 | iSNV |
| F24 | F24-30 | 4187 | NS2A | 0.022 | A:2124;G:48;C:0;T:0;total:2172 | iSNV |
| F24 | F24-30 | 4697 | NS3 | 0.0841 | A:0;G:0;C:414;T:4508;total:4922 | iSNV |
| F24 | F24-30 | 5952 | NS3 | 0.0859 | A:1;G:0;C:4627;T:435;total:5063 | iSNV |
| F24 | F24-30 | 8394 | NS5 | 0.0893 | A:0;G:0;C:450;T:4585;total:5035 | iSNV |
| F24 | F24-30 | 9359 | NS5 | 0.0698 | A:1;G:1;C:350;T:4656;total:5008 | iSNV |
| F24 | F24-30 | 10259 | NS5 | 0.103 | A:4046;G:465;C:0;T:0;total:4511 | iSNV |
| F24 | F24-30 | 10376 | NS5 | 0.2885 | A:1105;G:2725;C:0;T:0;total:3830 | iSNV |
| F24 | F24-30 | 10447 | 3'-UTR | 0.0245 | A:0;G:0;C:2905;T:73;total:2978 | iSNV |
| F24 | F24-30 | 10566 | 3'-UTR | 0.0784 | A:0;G:0;C:2515;T:214;total:2729 | iSNV |
| F24 | F24-30 | 10589 | 3'-UTR | 0.0204 | A:0;G:60;C:0;T:2879;total:2939 | iSNV |
| F24 | F24-4 | 353 | C | 0.0206 | A:6436;G:136;C:0;T:0;total:6572 | iSNV |

| | | | | | | |
|---|---|---|---|---|---|---|
| F24 | F24-4 | 803 | M | 0.0225 | A:0;G:5082;C:0;T:117;total:5199 | iSNV |
| F24 | F24-4 | 998 | E | 0.1445 | A:1;G:0;C:4129;T:698;total:4828 | iSNV |
| F24 | F24-4 | 1218 | E | 0.9373 | A:0;G:1;C:334;T:4986;total:5321 | iSNV |
| F24 | F24-4 | 1430 | E | 0.0264 | A:1;G:0;C:5968;T:162;total:6131 | iSNV |
| F24 | F24-4 | 2367 | E | 0.0402 | A:3712;G:156;C:1;T:2;total:3871 | iSNV |
| F24 | F24-4 | 3869 | NS2A | 0.8391 | A:0;G:0;C:1129;T:5884;total:7013 | iSNV |
| F24 | F24-4 | 4187 | NS2A | 0.021 | A:3029;G:65;C:0;T:0;total:3094 | iSNV |
| F24 | F24-4 | 4697 | NS3 | 0.1202 | A:0;G:2;C:884;T:6463;total:7349 | iSNV |
| F24 | F24-4 | 5952 | NS3 | 0.1136 | A:0;G:0;C:6864;T:880;total:7744 | iSNV |
| F24 | F24-4 | 6182 | NS3 | 0.0371 | A:0;G:1;C:193;T:4995;total:5189 | iSNV |
| F24 | F24-4 | 9359 | NS5 | 0.1104 | A:0;G:1;C:811;T:6530;total:7342 | iSNV |
| F24 | F24-4 | 10259 | NS5 | 0.1305 | A:4962;G:745;C:0;T:1;total:5708 | iSNV |
| F24 | F24-4 | 10447 | 3'-UTR | 0.0343 | A:0;G:0;C:3484;T:124;total:3608 | iSNV |
| F24 | F24-4 | 10566 | 3'-UTR | 0.1211 | A:0;G:0;C:3694;T:509;total:4203 | iSNV |
| F24 | F24-5 | 353 | C | 0.0306 | A:665;G:21;C:0;T:0;total:686 | iSNV |
| F24 | F24-5 | 399 | C | 0.0327 | A:26;G:0;C:769;T:0;total:795 | iSNV |
| F24 | F24-5 | 645 | M | 0.0349 | A:663;G:24;C:0;T:0;total:687 | iSNV |
| F24 | F24-5 | 803 | M | 0.0386 | A:0;G:523;C:0;T:21;total:544 | iSNV |
| F24 | F24-5 | 897 | M | 0.0264 | A:0;G:0;C:479;T:13;total:492 | iSNV |
| F24 | F24-5 | 998 | E | 0.1172 | A:0;G:0;C:414;T:55;total:469 | iSNV |
| F24 | F24-5 | 1117 | E | 0.0219 | A:580;G:13;C:0;T:0;total:593 | iSNV |
| F24 | F24-5 | 1218 | E | 0.8777 | A:0;G:0;C:69;T:495;total:564 | iSNV |
| F24 | F24-5 | 2543 | NS1 | 0.0231 | A:0;G:0;C:507;T:12;total:519 | iSNV |
| F24 | F24-5 | 2982 | NS1 | 0.0208 | A:14;G:0;C:657;T:0;total:671 | iSNV |
| F24 | F24-5 | 3869 | NS2A | 0.8174 | A:0;G:0;C:141;T:631;total:772 | iSNV |
| F24 | F24-5 | 4697 | NS3 | 0.0704 | A:0;G:0;C:55;T:726;total:781 | iSNV |
| F24 | F24-5 | 5952 | NS3 | 0.0895 | A:0;G:0;C:722;T:71;total:793 | iSNV |
| F24 | F24-5 | 6062 | NS3 | 0.0254 | A:0;G:0;C:612;T:16;total:628 | iSNV |
| F24 | F24-5 | 7182 | NS4A | 0.0274 | A:0;G:0;C:603;T:17;total:620 | iSNV |
| F24 | F24-5 | 7735 | NS5 | 0.0353 | A:17;G:464;C:0;T:0;total:481 | iSNV |
| F24 | F24-5 | 8612 | NS5 | 0.0352 | A:438;G:16;C:0;T:0;total:454 | iSNV |
| F24 | F24-5 | 9359 | NS5 | 0.0784 | A:0;G:0;C:55;T:646;total:701 | iSNV |
| F24 | F24-5 | 9716 | NS5 | 0.0234 | A:0;G:0;C:416;T:10;total:426 | iSNV |
| F24 | F24-5 | 10259 | NS5 | 0.1227 | A:586;G:82;C:0;T:0;total:668 | iSNV |
| F24 | F24-5 | 10428 | 3'-UTR | 0.0244 | A:0;G:0;C:478;T:12;total:490 | iSNV |
| F24 | F24-5 | 10447 | 3'-UTR | 0.0936 | A:0;G:0;C:387;T:40;total:427 | iSNV |
| F24 | F24-5 | 10452 | 3'-UTR | 0.0212 | A:415;G:9;C:0;T:0;total:424 | iSNV |
| F24 | F24-5 | 10566 | 3'-UTR | 0.1006 | A:0;G:0;C:420;T:47;total:467 | iSNV |
| F24 | F24-6 | 353 | C | 0.1023 | A:5206;G:594;C:3;T:0;total:5803 | iSNV |
| F24 | F24-6 | 645 | M | 0.0824 | A:4138;G:372;C:0;T:1;total:4511 | iSNV |
| F24 | F24-6 | 803 | M | 0.0899 | A:1;G:3044;C:0;T:301;total:3346 | iSNV |
| F24 | F24-6 | 998 | E | 0.124 | A:1;G:0;C:2625;T:372;total:2998 | iSNV |
| F24 | F24-6 | 1117 | E | 0.0759 | A:3186;G:262;C:0;T:0;total:3448 | iSNV |
| F24 | F24-6 | 1218 | E | 0.7921 | A:0;G:0;C:798;T:3039;total:3837 | iSNV |
| F24 | F24-6 | 1430 | E | 0.131 | A:1;G:1;C:3413;T:515;total:3930 | iSNV |
| F24 | F24-6 | 3869 | NS2A | 0.7704 | A:0;G:0;C:1116;T:3743;total:4859 | iSNV |
| F24 | F24-6 | 4697 | NS3 | 0.0343 | A:0;G:0;C:160;T:4502;total:4662 | iSNV |
| F24 | F24-6 | 4783 | NS3 | 0.1378 | A:0;G:1;C:704;T:4401;total:5106 | iSNV |
| F24 | F24-6 | 5952 | NS3 | 0.0397 | A:0;G:1;C:5659;T:234;total:5894 | iSNV |
| F24 | F24-6 | 6206 | NS3 | 0.0207 | A:0;G:0;C:2692;T:57;total:2749 | iSNV |
| F24 | F24-6 | 6906 | NS4A | 0.0368 | A:118;G:3082;C:0;T:0;total:3200 | iSNV |
| F24 | F24-6 | 7625 | NS4B | 0.0403 | A:1;G:1;C:4730;T:199;total:4931 | iSNV |
| F24 | F24-6 | 7735 | NS5 | 0.0768 | A:376;G:4516;C:1;T:0;total:4893 | iSNV |
| F24 | F24-6 | 8432 | NS5 | 0.0241 | A:5341;G:0;C:132;T:0;total:5473 | iSNV |
| F24 | F24-6 | 8518 | NS5 | 0.0823 | A:3944;G:1;C:0;T:354;total:4299 | iSNV |
| F24 | F24-6 | 8726 | NS5 | 0.0286 | A:0;G:1;C:111;T:3760;total:3872 | iSNV |
| F24 | F24-6 | 9359 | NS5 | 0.033 | A:0;G:2;C:216;T:6322;total:6540 | iSNV |
| F24 | F24-6 | 10259 | NS5 | 0.1309 | A:4254;G:641;C:0;T:1;total:4896 | iSNV |
| F24 | F24-6 | 10447 | 3'-UTR | 0.1098 | A:2;G:0;C:2801;T:346;total:3149 | iSNV |
| F24 | F24-6 | 10566 | 3'-UTR | 0.0378 | A:1;G:0;C:2438;T:96;total:2535 | iSNV |
| F24 | F24-6 | 10577 | 3'-UTR | 0.026 | A:0;G:0;C:2506;T:67;total:2573 | iSNV |
| F24 | F24-7 | 998 | E | 0.0961 | A:0;G:1;C:3682;T:392;total:4075 | iSNV |
| F24 | F24-7 | 1218 | E | 0.9555 | A:0;G:0;C:216;T:4634;total:4850 | iSNV |
| F24 | F24-7 | 3257 | NS1 | 0.064 | A:0;G:0;C:410;T:5989;total:6399 | iSNV |
| F24 | F24-7 | 3317 | NS1 | 0.0206 | A:2;G:0;C:5888;T:124;total:6014 | iSNV |
| F24 | F24-7 | 3869 | NS2A | 0.8807 | A:0;G:0;C:742;T:5477;total:6219 | iSNV |
| F24 | F24-7 | 4697 | NS3 | 0.0961 | A:0;G:0;C:625;T:5873;total:6498 | iSNV |
| F24 | F24-7 | 5952 | NS3 | 0.0938 | A:1;G:0;C:5867;T:608;total:6476 | iSNV |
| F24 | F24-7 | 6322 | NS3 | 0.021 | A:2;G:0;C:5201;T:112;total:5315 | iSNV |
| F24 | F24-7 | 9359 | NS5 | 0.0753 | A:0;G:0;C:500;T:6136;total:6636 | iSNV |
| F24 | F24-7 | 9370 | NS5 | 0.0492 | A:333;G:1;C:0;T:6422;total:6756 | iSNV |
| F24 | F24-7 | 9690 | NS5 | 0.0287 | A:124;G:4182;C:0;T:0;total:4306 | iSNV |
| F24 | F24-7 | 9818 | NS5 | 0.0353 | A:1;G:1;C:154;T:4200;total:4356 | iSNV |
| F24 | F24-7 | 10259 | NS5 | 0.102 | A:4549;G:517;C:1;T:0;total:5067 | iSNV |
| F24 | F24-7 | 10428 | 3'-UTR | 0.0388 | A:0;G:2;C:3315;T:134;total:3451 | iSNV |
| F24 | F24-7 | 10447 | 3'-UTR | 0.0465 | A:0;G:0;C:3052;T:149;total:3201 | iSNV |
| F24 | F24-7 | 10566 | 3'-UTR | 0.0863 | A:1;G:0;C:3163;T:299;total:3463 | iSNV |
| F24 | F24-8 | 869 | M | 0.2367 | A:156;G:0;C:0;T:503;total:659 | iSNV |
| F24 | F24-8 | 998 | E | 0.0894 | A:0;G:0;C:468;T:46;total:514 | iSNV |
| F24 | F24-8 | 1218 | E | 0.9719 | A:0;G:0;C:18;T:621;total:639 | iSNV |
| F24 | F24-8 | 1430 | E | 0.2453 | A:0;G:0;C:526;T:171;total:697 | iSNV |
| F24 | F24-8 | 1453 | E | 0.0244 | A:0;G:0;C:17;T:679;total:696 | iSNV |

| | | | | | | |
|---|---|---|---|---|---|---|
| F24 | F24-8 | 3131 | NS1 | 0.0704 | A:699;G:53;C:0;T:0;total:752 | iSNV |
| F24 | F24-8 | 3257 | NS1 | 0.0661 | A:0;G:0;C:46;T:649;total:695 | iSNV |
| F24 | F24-8 | 3869 | NS2A | 0.9104 | A:0;G:0;C:69;T:701;total:770 | iSNV |
| F24 | F24-8 | 4187 | NS2A | 0.0206 | A:380;G:8;C:0;T:0;total:388 | iSNV |
| F24 | F24-8 | 4697 | NS3 | 0.0707 | A:0;G:0;C:57;T:749;total:806 | iSNV |
| F24 | F24-8 | 4835 | NS3 | 0.0286 | A:0;G:0;C:678;T:20;total:698 | iSNV |
| F24 | F24-8 | 5665 | NS3 | 0.0227 | A:0;G:0;C:772;T:18;total:790 | iSNV |
| F24 | F24-8 | 5952 | NS3 | 0.0894 | A:0;G:0;C:662;T:65;total:727 | iSNV |
| F24 | F24-8 | 9359 | NS5 | 0.0789 | A:0;G:0;C:56;T:653;total:709 | iSNV |
| F24 | F24-8 | 9818 | NS5 | 0.02 | A:0;G:0;C:11;T:539;total:550 | iSNV |
| F24 | F24-8 | 10259 | NS5 | 0.0823 | A:735;G:66;C:0;T:0;total:801 | iSNV |
| F24 | F24-8 | 10310 | NS5 | 0.0356 | A:0;G:0;C:677;T:25;total:702 | iSNV |
| F24 | F24-8 | 10428 | 3'-UTR | 0.0207 | A:0;G:0;C:518;T:11;total:529 | iSNV |
| F24 | F24-8 | 10447 | 3'-UTR | 0.0216 | A:0;G:0;C:452;T:10;total:462 | iSNV |
| F24 | F24-8 | 10452 | 3'-UTR | 0.0256 | A:456;G:12;C:0;T:0;total:468 | iSNV |
| F24 | F24-8 | 10566 | 3'-UTR | 0.0662 | A:0;G:0;C:465;T:33;total:498 | iSNV |
| F24 | F24-9 | 900 | M | 0.0284 | A:181;G:6189;C:0;T:1;total:6371 | iSNV |
| F24 | F24-9 | 998 | E | 0.0934 | A:0;G:0;C:6219;T:641;total:6860 | iSNV |
| F24 | F24-9 | 1218 | E | 0.9666 | A:0;G:2;C:274;T:7906;total:8182 | iSNV |
| F24 | F24-9 | 1430 | E | 0.0353 | A:1;G:0;C:8872;T:325;total:9198 | iSNV |
| F24 | F24-9 | 2258 | E | 0.0429 | A:0;G:0;C:288;T:6421;total:6709 | iSNV |
| F24 | F24-9 | 2448 | E | 0.0494 | A:0;G:0;C:4843;T:252;total:5095 | iSNV |
| F24 | F24-9 | 3869 | NS2A | 0.9138 | A:0;G:1;C:879;T:9307;total:10187 | iSNV |
| F24 | F24-9 | 4187 | NS2A | 0.0555 | A:3827;G:225;C:0;T:0;total:4052 | iSNV |
| F24 | F24-9 | 4319 | NS2B | 0.0308 | A:272;G:8546;C:1;T:0;total:8819 | iSNV |
| F24 | F24-9 | 4697 | NS3 | 0.0636 | A:0;G:1;C:663;T:9753;total:10417 | iSNV |
| F24 | F24-9 | 5952 | NS3 | 0.066 | A:0;G:0;C:11644;T:824;total:12468 | iSNV |
| F24 | F24-9 | 6440 | NS3 | 0.0474 | A:1;G:0;C:7660;T:382;total:8043 | iSNV |
| F24 | F24-9 | 6938 | NS4A | 0.0334 | A:1;G:0;C:6306;T:218;total:6525 | iSNV |
| F24 | F24-9 | 9359 | NS5 | 0.0535 | A:0;G:1;C:702;T:12405;total:13108 | iSNV |
| F24 | F24-9 | 9533 | NS5 | 0.0253 | A:0;G:2;C:214;T:8233;total:8449 | iSNV |
| F24 | F24-9 | 9728 | NS5 | 0.0282 | A:0;G:0;C:5499;T:160;total:5659 | iSNV |
| F24 | F24-9 | 9860 | NS5 | 0.0549 | A:0;G:3;C:390;T:6698;total:7091 | iSNV |
| F24 | F24-9 | 10259 | NS5 | 0.0738 | A:9465;G:755;C:0;T:0;total:10220 | iSNV |
| F24 | F24-9 | 10447 | 3'-UTR | 0.025 | A:0;G:0;C:6315;T:162;total:6477 | iSNV |
| F24 | F24-9 | 10566 | 3'-UTR | 0.0598 | A:0;G:0;C:5104;T:325;total:5429 | iSNV |
| F27 | F27-1 | 930 | M | 0.046 | A:0;G:0;C:1449;T:70;total:1519 | iSNV |
| F27 | F27-1 | 998 | E | 0.1471 | A:0;G:0;C:1171;T:202;total:1373 | iSNV |
| F27 | F27-1 | 1218 | E | 0.9899 | A:0;G:0;C:14;T:1360;total:1374 | SNP |
| F27 | F27-1 | 2376 | E | 0.0236 | A:0;G:0;C:1323;T:32;total:1355 | iSNV |
| F27 | F27-1 | 3341 | NS1 | 0.0215 | A:1631;G:36;C:0;T:0;total:1667 | iSNV |
| F27 | F27-1 | 3869 | NS2A | 0.8546 | A:0;G:0;C:229;T:1345;total:1574 | iSNV |
| F27 | F27-1 | 3926 | NS2A | 0.0219 | A:0;G:0;C:1428;T:32;total:1460 | iSNV |
| F27 | F27-1 | 4226 | NS2B | 0.0243 | A:0;G:0;C:30;T:1200;total:1230 | iSNV |
| F27 | F27-1 | 4559 | NS2B | 0.0257 | A:0;G:0;C:1625;T:43;total:1668 | iSNV |
| F27 | F27-1 | 4697 | NS3 | 0.1523 | A:0;G:0;C:178;T:990;total:1168 | iSNV |
| F27 | F27-1 | 5952 | NS3 | 0.156 | A:0;G:0;C:1352;T:250;total:1602 | iSNV |
| F27 | F27-1 | 7741 | NS5 | 0.0392 | A:1420;G:58;C:0;T:0;total:1478 | iSNV |
| F27 | F27-1 | 8651 | NS5 | 0.021 | A:1440;G:31;C:0;T:0;total:1471 | iSNV |
| F27 | F27-1 | 9359 | NS5 | 0.1357 | A:0;G:0;C:287;T:1827;total:2114 | iSNV |
| F27 | F27-1 | 9983 | NS5 | 0.0325 | A:0;G:0;C:1456;T:49;total:1505 | iSNV |
| F27 | F27-1 | 10259 | NS5 | 0.1369 | A:1330;G:211;C:0;T:0;total:1541 | iSNV |
| F27 | F27-1 | 10428 | 3'-UTR | 0.0311 | A:0;G:0;C:1306;T:42;total:1348 | iSNV |
| F27 | F27-1 | 10566 | 3'-UTR | 0.1458 | A:0;G:0;C:1212;T:207;total:1419 | iSNV |
| F27 | F27-10 | 998 | E | 0.0641 | A:0;G:0;C:2872;T:197;total:3069 | iSNV |
| F27 | F27-10 | 1218 | E | 0.987 | A:0;G:0;C:44;T:3329;total:3373 | SNP |
| F27 | F27-10 | 1283 | E | 0.0221 | A:0;G:0;C:3849;T:87;total:3936 | iSNV |
| F27 | F27-10 | 1430 | E | 0.0696 | A:0;G:0;C:3673;T:275;total:3948 | iSNV |
| F27 | F27-10 | 2474 | E | 0.0291 | A:73;G:1;C:1;T:2431;total:2506 | iSNV |
| F27 | F27-10 | 3317 | NS1 | 0.0223 | A:0;G:0;C:4367;T:100;total:4467 | iSNV |
| F27 | F27-10 | 3869 | NS2A | 0.9387 | A:0;G:0;C:281;T:4300;total:4581 | iSNV |
| F27 | F27-10 | 4697 | NS3 | 0.0421 | A:0;G:0;C:195;T:4428;total:4623 | iSNV |
| F27 | F27-10 | 4745 | NS3 | 0.028 | A:0;G:0;C:4605;T:133;total:4738 | iSNV |
| F27 | F27-10 | 5952 | NS3 | 0.0512 | A:1;G:0;C:4780;T:258;total:5039 | iSNV |
| F27 | F27-10 | 6152 | NS3 | 0.0221 | A:2873;G:65;C:0;T:0;total:2938 | iSNV |
| F27 | F27-10 | 6157 | NS3 | 0.0251 | A:0;G:0;C:2913;T:75;total:2988 | iSNV |
| F27 | F27-10 | 6206 | NS3 | 0.0826 | A:0;G:0;C:3463;T:312;total:3775 | iSNV |
| F27 | F27-10 | 6322 | NS3 | 0.0237 | A:0;G:0;C:3571;T:87;total:3658 | iSNV |
| F27 | F27-10 | 6398 | NS3 | 0.0276 | A:0;G:0;C:104;T:3663;total:3767 | iSNV |
| F27 | F27-10 | 7496 | NS4B | 0.0204 | A:0;G:2495;C:0;T:52;total:2547 | iSNV |
| F27 | F27-10 | 9152 | NS5 | 0.025 | A:0;G:4972;C:0;T:128;total:5100 | iSNV |
| F27 | F27-10 | 9359 | NS5 | 0.0539 | A:1;G:0;C:271;T:4755;total:5027 | iSNV |
| F27 | F27-10 | 10259 | NS5 | 0.0538 | A:3428;G:195;C:0;T:0;total:3623 | iSNV |
| F27 | F27-10 | 10428 | 3'-UTR | 0.0246 | A:0;G:0;C:2576;T:65;total:2641 | iSNV |
| F27 | F27-10 | 10447 | 3'-UTR | 0.025 | A:0;G:0;C:2415;T:62;total:2477 | iSNV |
| F27 | F27-10 | 10566 | 3'-UTR | 0.0443 | A:0;G:1;C:2757;T:128;total:2886 | iSNV |
| F27 | F27-11 | 998 | E | 0.0863 | A:1;G:0;C:4742;T:448;total:5191 | iSNV |
| F27 | F27-11 | 1218 | E | 0.991 | A:0;G:0;C:53;T:5805;total:5858 | SNP |
| F27 | F27-11 | 3149 | NS1 | 0.077 | A:0;G:0;C:530;T:6349;total:6879 | iSNV |
| F27 | F27-11 | 3428 | NS1 | 0.0227 | A:0;G:0;C:5544;T:129;total:5673 | iSNV |
| F27 | F27-11 | 3869 | NS2A | 0.9079 | A:2;G:0;C:558;T:5496;total:6056 | iSNV |
| F27 | F27-11 | 4697 | NS3 | 0.0773 | A:0;G:0;C:524;T:6251;total:6775 | iSNV |

| | | | | | | |
|---|---|---|---|---|---|---|
| F27 | F27-11 | 5952 | NS3 | 0.0671 | A:0;G:0;C:6738;T:485;total:7223 | iSNV |
| F27 | F27-11 | 9359 | NS5 | 0.0763 | A:5;G:0;C:605;T:7310;total:7920 | iSNV |
| F27 | F27-11 | 9880 | NS5 | 0.1147 | A:711;G:5481;C:0;T:2;total:6194 | iSNV |
| F27 | F27-11 | 10259 | NS5 | 0.0784 | A:6660;G:567;C:0;T:0;total:7227 | iSNV |
| F27 | F27-11 | 10566 | 3'-UTR | 0.0722 | A:0;G:0;C:4674;T:364;total:5038 | iSNV |
| F27 | F27-11 | 10589 | 3'-UTR | 0.117 | A:0;G:615;C:0;T:4639;total:5254 | iSNV |
| F27 | F27-12 | 353 | C | 0.0551 | A:13029;G:761;C:1;T:4;total:13795 | iSNV |
| F27 | F27-12 | 645 | M | 0.0492 | A:11748;G:609;C:0;T:1;total:12358 | iSNV |
| F27 | F27-12 | 998 | E | 0.1917 | A:0;G:0;C:8550;T:2028;total:10578 | iSNV |
| F27 | F27-12 | 1083 | E | 0.0378 | A:10245;G:403;C:0;T:0;total:10648 | iSNV |
| F27 | F27-12 | 1117 | E | 0.0831 | A:10978;G:996;C:0;T:1;total:11975 | iSNV |
| F27 | F27-12 | 1218 | E | 0.9344 | A:0;G:0;C:782;T:11133;total:11915 | iSNV |
| F27 | F27-12 | 2277 | E | 0.0582 | A:0;G:0;C:576;T:9308;total:9884 | iSNV |
| F27 | F27-12 | 3869 | NS2A | 0.794 | A:2;G:0;C:2569;T:9898;total:12469 | iSNV |
| F27 | F27-12 | 3974 | NS2A | 0.025 | A:2;G:0;C:13687;T:352;total:14041 | iSNV |
| F27 | F27-12 | 4697 | NS3 | 0.1339 | A:1;G:0;C:1939;T:12532;total:14472 | iSNV |
| F27 | F27-12 | 5558 | NS3 | 0.0291 | A:421;G:14036;C:1;T:1;total:14459 | iSNV |
| F27 | F27-12 | 5780 | NS3 | 0.0211 | A:0;G:0;C:330;T:15266;total:15596 | iSNV |
| F27 | F27-12 | 5952 | NS3 | 0.1344 | A:0;G:0;C:13024;T:2023;total:15047 | iSNV |
| F27 | F27-12 | 8198 | NS5 | 0.0317 | A:13045;G:428;C:3;T:0;total:13476 | iSNV |
| F27 | F27-12 | 9242 | NS5 | 0.0241 | A:2;G:431;C:2;T:17432;total:17867 | iSNV |
| F27 | F27-12 | 9359 | NS5 | 0.1451 | A:3;G:0;C:2329;T:13713;total:16045 | iSNV |
| F27 | F27-12 | 10259 | NS5 | 0.1907 | A:11938;G:2815;C:0;T:1;total:14754 | iSNV |
| F27 | F27-12 | 10428 | 3'-UTR | 0.0529 | A:0;G:0;C:11838;T:662;total:12500 | iSNV |
| F27 | F27-12 | 10447 | 3'-UTR | 0.069 | A:3;G:0;C:10512;T:780;total:11295 | iSNV |
| F27 | F27-12 | 10566 | 3'-UTR | 0.1295 | A:1;G:0;C:9022;T:1343;total:10366 | iSNV |
| F27 | F27-12 | 10589 | 3'-UTR | 0.0212 | A:1;G:227;C:3;T:10469;total:10700 | iSNV |
| F27 | F27-12 | 10592 | 3'-UTR | 0.0221 | A:1;G:0;C:10675;T:242;total:10918 | iSNV |
| F27 | F27-13 | 395 | C | 0.6824 | A:1213;G:2606;C:0;T:0;total:3819 | iSNV |
| F27 | F27-13 | 998 | E | 0.0225 | A:2;G:0;C:2300;T:53;total:2355 | iSNV |
| F27 | F27-13 | 1218 | E | 0.995 | A:0;G:1;C:12;T:2551;total:2564 | SNP |
| F27 | F27-13 | 1413 | E | 0.7008 | A:905;G:2119;C:0;T:0;total:3024 | iSNV |
| F27 | F27-13 | 3869 | NS2A | 0.9618 | A:0;G:0;C:135;T:3397;total:3532 | iSNV |
| F27 | F27-13 | 4697 | NS3 | 0.0208 | A:1;G:1;C:73;T:3429;total:3504 | iSNV |
| F27 | F27-13 | 5952 | NS3 | 0.0262 | A:0;G:0;C:3781;T:102;total:3883 | iSNV |
| F27 | F27-13 | 9161 | NS5 | 0.0346 | A:0;G:0;C:134;T:3734;total:3868 | iSNV |
| F27 | F27-13 | 10259 | NS5 | 0.0237 | A:2793;G:68;C:0;T:0;total:2861 | iSNV |
| F27 | F27-13 | 10376 | NS5 | 0.7051 | A:1480;G:619;C:0;T:0;total:2099 | iSNV |
| F27 | F27-13 | 10566 | 3'-UTR | 0.0291 | A:0;G:0;C:1934;T:58;total:1992 | iSNV |
| F27 | F27-14 | 854 | M | 0.0286 | A:0;G:0;C:5670;T:167;total:5837 | iSNV |
| F27 | F27-14 | 998 | E | 0.1131 | A:1;G:0;C:4960;T:633;total:5594 | iSNV |
| F27 | F27-14 | 1117 | E | 0.2038 | A:4872;G:1248;C:0;T:1;total:6121 | iSNV |
| F27 | F27-14 | 1218 | E | 0.9812 | A:0;G:0;C:117;T:6097;total:6214 | SNP |
| F27 | F27-14 | 1295 | E | 0.0269 | A:7065;G:1;C:0;T:196;total:7262 | iSNV |
| F27 | F27-14 | 1430 | E | 0.0237 | A:0;G:1;C:7185;T:175;total:7361 | iSNV |
| F27 | F27-14 | 2186 | E | 0.0203 | A:124;G:5974;C:0;T:0;total:6098 | iSNV |
| F27 | F27-14 | 2282 | E | 0.0215 | A:0;G:0;C:5131;T:113;total:5244 | iSNV |
| F27 | F27-14 | 3869 | NS2A | 0.8823 | A:0;G:0;C:718;T:5382;total:6100 | iSNV |
| F27 | F27-14 | 4697 | NS3 | 0.0998 | A:0;G:0;C:777;T:7002;total:7779 | iSNV |
| F27 | F27-14 | 5558 | NS3 | 0.2076 | A:1599;G:6101;C:0;T:0;total:7700 | iSNV |
| F27 | F27-14 | 5952 | NS3 | 0.1011 | A:0;G:0;C:7079;T:797;total:7876 | iSNV |
| F27 | F27-14 | 6401 | NS3 | 0.0387 | A:0;G:0;C:239;T:5934;total:6173 | iSNV |
| F27 | F27-14 | 6557 | NS4A | 0.0245 | A:5731;G:0;C:0;T:144;total:5875 | iSNV |
| F27 | F27-14 | 6786 | NS4A | 0.0265 | A:0;G:0;C:5728;T:156;total:5884 | iSNV |
| F27 | F27-14 | 7232 | NS4A | 0.0234 | A:1;G:0;C:4914;T:118;total:5033 | iSNV |
| F27 | F27-14 | 9359 | NS5 | 0.1021 | A:0;G:6;C:859;T:7547;total:8412 | iSNV |
| F27 | F27-14 | 9370 | NS5 | 0.0533 | A:0;G:453;C:3;T:8030;total:8486 | iSNV |
| F27 | F27-14 | 9542 | NS5 | 0.0218 | A:0;G:0;C:7312;T:163;total:7475 | iSNV |
| F27 | F27-14 | 10259 | NS5 | 0.1039 | A:7057;G:819;C:0;T:0;total:7876 | iSNV |
| F27 | F27-14 | 10428 | 3'-UTR | 0.0271 | A:0;G:0;C:6200;T:173;total:6373 | iSNV |
| F27 | F27-14 | 10452 | 3'-UTR | 0.0288 | A:5451;G:162;C:0;T:1;total:5614 | iSNV |
| F27 | F27-14 | 10566 | 3'-UTR | 0.1008 | A:3;G:1;C:4705;T:528;total:5237 | iSNV |
| F27 | F27-14 | 10567 | 3'-UTR | 0.0209 | A:0;G:0;C:109;T:5103;total:5212 | iSNV |
| F27 | F27-15 | 197 | C | 0.031 | A:10744;G:344;C:0;T:0;total:11088 | iSNV |
| F27 | F27-15 | 998 | E | 0.0887 | A:1;G:0;C:6960;T:678;total:7639 | iSNV |
| F27 | F27-15 | 1218 | E | 0.9959 | A:2;G:1;C:32;T:8426;total:8461 | SNP |
| F27 | F27-15 | 1413 | E | 0.1013 | A:8854;G:999;C:1;T:1;total:9855 | iSNV |
| F27 | F27-15 | 1430 | E | 0.0292 | A:1;G:0;C:9484;T:286;total:9771 | iSNV |
| F27 | F27-15 | 2917 | NS1 | 0.021 | A:10580;G:227;C:1;T:0;total:10808 | iSNV |
| F27 | F27-15 | 3869 | NS2A | 0.9113 | A:0;G:0;C:790;T:8110;total:8900 | iSNV |
| F27 | F27-15 | 4697 | NS3 | 0.0775 | A:0;G:0;C:786;T:9347;total:10133 | iSNV |
| F27 | F27-15 | 5952 | NS3 | 0.0806 | A:1;G:0;C:9656;T:847;total:10504 | iSNV |
| F27 | F27-15 | 6314 | NS3 | 0.0247 | A:0;G:1;C:205;T:8076;total:8282 | iSNV |
| F27 | F27-15 | 6779 | NS4A | 0.0274 | A:7865;G:222;C:0;T:1;total:8088 | iSNV |
| F27 | F27-15 | 6956 | NS4A | 0.0206 | A:2;G:0;C:8381;T:177;total:8560 | iSNV |
| F27 | F27-15 | 7685 | NS5 | 0.0202 | A:0;G:1;C:189;T:9143;total:9333 | iSNV |
| F27 | F27-15 | 8430 | NS5 | 0.0989 | A:0;G:0;C:9417;T:1034;total:10451 | iSNV |
| F27 | F27-15 | 8456 | NS5 | 0.0254 | A:10720;G:280;C:0;T:0;total:11000 | iSNV |
| F27 | F27-15 | 9359 | NS5 | 0.084 | A:0;G:0;C:939;T:10232;total:11171 | iSNV |
| F27 | F27-15 | 9634 | NS5 | 0.0542 | A:0;G:2;C:507;T:8842;total:9351 | iSNV |
| F27 | F27-15 | 9818 | NS5 | 0.0274 | A:0;G:0;C:232;T:8229;total:8461 | iSNV |
| F27 | F27-15 | 10259 | NS5 | 0.0826 | A:9652;G:870;C:0;T:0;total:10522 | iSNV |

| | | | | | | |
|---|---|---|---|---|---|---|
| F27 | F27-15 | 10376 | NS5 | 0.0979 | A:919;G:8457;C:0;T:3;total:9379 | iSNV |
| F27 | F27-15 | 10566 | 3'-UTR | 0.0712 | A:2;G:1;C:6982;T:536;total:7521 | iSNV |
| F27 | F27-16 | 293 | C | 0.0271 | A:2979;G:83;C:0;T:0;total:3062 | iSNV |
| F27 | F27-16 | 469 | C | 0.0322 | A:0;G:0;C:3088;T:103;total:3191 | iSNV |
| F27 | F27-16 | 803 | M | 0.0224 | A:0;G:2305;C:0;T:53;total:2358 | iSNV |
| F27 | F27-16 | 998 | E | 0.1629 | A:0;G:0;C:2014;T:392;total:2406 | iSNV |
| F27 | F27-16 | 1218 | E | 0.9723 | A:0;G:1;C:73;T:2561;total:2635 | iSNV |
| F27 | F27-16 | 1430 | E | 0.0221 | A:0;G:0;C:2826;T:64;total:2890 | iSNV |
| F27 | F27-16 | 2218 | E | 0.0493 | A:0;G:0;C:2621;T:136;total:2757 | iSNV |
| F27 | F27-16 | 3675 | NS1 | 0.0308 | A:68;G:2137;C:0;T:0;total:2205 | iSNV |
| F27 | F27-16 | 3869 | NS2A | 0.8205 | A:0;G:0;C:579;T:2646;total:3225 | iSNV |
| F27 | F27-16 | 4049 | NS2A | 0.0482 | A:0;G:0;C:2428;T:123;total:2551 | iSNV |
| F27 | F27-16 | 4697 | NS3 | 0.1634 | A:0;G:0;C:561;T:2871;total:3432 | iSNV |
| F27 | F27-16 | 4926 | NS3 | 0.0263 | A:0;G:2775;C:0;T:75;total:2850 | iSNV |
| F27 | F27-16 | 5952 | NS3 | 0.1531 | A:0;G:0;C:2937;T:531;total:3468 | iSNV |
| F27 | F27-16 | 6672 | NS4A | 0.0399 | A:0;G:0;C:2092;T:87;total:2179 | iSNV |
| F27 | F27-16 | 7528 | NS4B | 0.0201 | A:0;G:0;C:2238;T:46;total:2284 | iSNV |
| F27 | F27-16 | 8696 | NS5 | 0.0215 | A:0;G:0;C:2135;T:47;total:2182 | iSNV |
| F27 | F27-16 | 9293 | NS5 | 0.03 | A:96;G:3097;C:0;T:1;total:3194 | iSNV |
| F27 | F27-16 | 9359 | NS5 | 0.1531 | A:0;G:2;C:553;T:3057;total:3612 | iSNV |
| F27 | F27-16 | 9818 | NS5 | 0.0605 | A:1;G:0;C:140;T:2173;total:2314 | iSNV |
| F27 | F27-16 | 10259 | NS5 | 0.1395 | A:2287;G:371;C:0;T:0;total:2658 | iSNV |
| F27 | F27-16 | 10428 | 3'-UTR | 0.0451 | A:0;G:0;C:1734;T:82;total:1816 | iSNV |
| F27 | F27-16 | 10447 | 3'-UTR | 0.0266 | A:0;G:0;C:1680;T:46;total:1726 | iSNV |
| F27 | F27-16 | 10566 | 3'-UTR | 0.1443 | A:0;G:0;C:1814;T:306;total:2120 | iSNV |
| F27 | F27-16 | 10782 | 3'-UTR | 0.042 | A:0;G:0;C:2143;T:94;total:2237 | iSNV |
| F27 | F27-17 | 783 | M | 0.0316 | A:224;G:0;C:0;T:6854;total:7078 | iSNV |
| F27 | F27-17 | 998 | E | 0.0898 | A:0;G:0;C:5491;T:542;total:6033 | iSNV |
| F27 | F27-17 | 1218 | E | 0.9896 | A:0;G:0;C:69;T:6517;total:6586 | SNP |
| F27 | F27-17 | 1430 | E | 0.042 | A:0;G:0;C:7612;T:334;total:7946 | iSNV |
| F27 | F27-17 | 1586 | E | 0.0233 | A:0;G:0;C:5763;T:138;total:5901 | iSNV |
| F27 | F27-17 | 2376 | E | 0.0501 | A:0;G:0;C:4902;T:259;total:5161 | iSNV |
| F27 | F27-17 | 3317 | NS1 | 0.0328 | A:0;G:1;C:7452;T:253;total:7706 | iSNV |
| F27 | F27-17 | 3869 | NS2A | 0.8998 | A:0;G:0;C:698;T:6263;total:6961 | iSNV |
| F27 | F27-17 | 4319 | NS2B | 0.0238 | A:148;G:6065;C:0;T:0;total:6213 | iSNV |
| F27 | F27-17 | 4697 | NS3 | 0.0882 | A:2;G:0;C:716;T:7396;total:8114 | iSNV |
| F27 | F27-17 | 4783 | NS3 | 0.0273 | A:0;G:0;C:215;T:7640;total:7855 | iSNV |
| F27 | F27-17 | 5952 | NS3 | 0.0904 | A:0;G:0;C:7712;T:767;total:8479 | iSNV |
| F27 | F27-17 | 6322 | NS3 | 0.0375 | A:3;G:0;C:6282;T:245;total:6530 | iSNV |
| F27 | F27-17 | 6401 | NS3 | 0.037 | A:0;G:0;C:247;T:6420;total:6667 | iSNV |
| F27 | F27-17 | 6938 | NS4A | 0.0252 | A:0;G:0;C:6103;T:158;total:6261 | iSNV |
| F27 | F27-17 | 7109 | NS4A | 0.0258 | A:2;G:0;C:6696;T:178;total:6876 | iSNV |
| F27 | F27-17 | 7151 | NS4A | 0.04 | A:0;G:0;C:6908;T:288;total:7196 | iSNV |
| F27 | F27-17 | 9359 | NS5 | 0.0866 | A:0;G:1;C:789;T:8314;total:9104 | iSNV |
| F27 | F27-17 | 10259 | NS5 | 0.0884 | A:7630;G:741;C:1;T:1;total:8373 | iSNV |
| F27 | F27-17 | 10428 | 3'-UTR | 0.0453 | A:2;G:1;C:6646;T:316;total:6965 | iSNV |
| F27 | F27-17 | 10452 | 3'-UTR | 0.0219 | A:6011;G:135;C:1;T:1;total:6148 | iSNV |
| F27 | F27-17 | 10566 | 3'-UTR | 0.0841 | A:1;G:0;C:5398;T:496;total:5895 | iSNV |
| F27 | F27-18 | 998 | E | 0.155 | A:1;G:0;C:3328;T:611;total:3940 | iSNV |
| F27 | F27-18 | 1218 | E | 0.9947 | A:1;G:0;C:22;T:4246;total:4269 | SNP |
| F27 | F27-18 | 1413 | E | 0.0783 | A:4492;G:382;C:0;T:0;total:4874 | iSNV |
| F27 | F27-18 | 1430 | E | 0.0338 | A:1;G:0;C:4616;T:162;total:4779 | iSNV |
| F27 | F27-18 | 1672 | E | 0.0367 | A:0;G:0;C:3800;T:145;total:3945 | iSNV |
| F27 | F27-18 | 3667 | NS1 | 0.0231 | A:0;G:0;C:82;T:3453;total:3535 | iSNV |
| F27 | F27-18 | 3869 | NS2A | 0.8467 | A:0;G:0;C:677;T:3737;total:4414 | iSNV |
| F27 | F27-18 | 3932 | NS2A | 0.0217 | A:0;G:0;C:111;T:5000;total:5111 | iSNV |
| F27 | F27-18 | 4187 | NS2A | 0.0229 | A:2474;G:58;C:0;T:0;total:2532 | iSNV |
| F27 | F27-18 | 4697 | NS3 | 0.1522 | A:0;G:0;C:784;T:4364;total:5148 | iSNV |
| F27 | F27-18 | 5952 | NS3 | 0.1449 | A:0;G:0;C:4695;T:796;total:5491 | iSNV |
| F27 | F27-18 | 6157 | NS3 | 0.0316 | A:0;G:0;C:3303;T:108;total:3411 | iSNV |
| F27 | F27-18 | 7421 | NS4B | 0.0244 | A:3426;G:86;C:1;T:0;total:3513 | iSNV |
| F27 | F27-18 | 8243 | NS5 | 0.0318 | A:1;G:0;C:4413;T:145;total:4559 | iSNV |
| F27 | F27-18 | 8414 | NS5 | 0.0221 | A:0;G:0;C:5530;T:125;total:5655 | iSNV |
| F27 | F27-18 | 8791 | NS5 | 0.0217 | A:1;G:0;C:3831;T:85;total:3917 | iSNV |
| F27 | F27-18 | 9359 | NS5 | 0.1523 | A:0;G:0;C:897;T:4989;total:5886 | iSNV |
| F27 | F27-18 | 10133 | NS5 | 0.0276 | A:2;G:1;C:4179;T:119;total:4301 | iSNV |
| F27 | F27-18 | 10259 | NS5 | 0.141 | A:4565;G:750;C:1;T:0;total:5316 | iSNV |
| F27 | F27-18 | 10376 | NS5 | 0.0765 | A:360;G:4340;C:0;T:0;total:4700 | iSNV |
| F27 | F27-18 | 10447 | 3'-UTR | 0.0225 | A:0;G:0;C:3735;T:86;total:3821 | iSNV |
| F27 | F27-18 | 10566 | 3'-UTR | 0.137 | A:2;G:0;C:3171;T:504;total:3677 | iSNV |
| F27 | F27-19 | 645 | M | 0.0224 | A:2442;G:56;C:0;T:1;total:2499 | iSNV |
| F27 | F27-19 | 869 | M | 0.0962 | A:198;G:0;C:0;T:1859;total:2057 | iSNV |
| F27 | F27-19 | 998 | E | 0.0693 | A:0;G:0;C:2025;T:151;total:2176 | iSNV |
| F27 | F27-19 | 1218 | E | 0.9794 | A:0;G:0;C:47;T:2225;total:2272 | iSNV |
| F27 | F27-19 | 1430 | E | 0.1272 | A:0;G:0;C:1858;T:271;total:2129 | iSNV |
| F27 | F27-19 | 1459 | E | 0.09 | A:198;G:0;C:2001;T:0;total:2199 | iSNV |
| F27 | F27-19 | 3646 | NS1 | 0.0877 | A:0;G:0;C:177;T:1839;total:2016 | iSNV |
| F27 | F27-19 | 3869 | NS2A | 0.9114 | A:0;G:0;C:192;T:1973;total:2165 | iSNV |
| F27 | F27-19 | 4697 | NS3 | 0.0634 | A:0;G:0;C:117;T:1727;total:1844 | iSNV |
| F27 | F27-19 | 5813 | NS3 | 0.0558 | A:0;G:0;C:2348;T:139;total:2487 | iSNV |
| F27 | F27-19 | 5927 | NS3 | 0.0587 | A:0;G:0;C:2244;T:140;total:2384 | iSNV |
| F27 | F27-19 | 5952 | NS3 | 0.0524 | A:0;G:0;C:2368;T:131;total:2499 | iSNV |

| | | | | | | |
|---|---|---|---|---|---|---|
| F27 | F27-19 | 6413 | NS3 | 0.0566 | A:0;G:0;C:125;T:2082;total:2207 | iSNV |
| F27 | F27-19 | 6938 | NS4A | 0.0266 | A:0;G:2;C:2046;T:56;total:2104 | iSNV |
| F27 | F27-19 | 8897 | NS5 | 0.0769 | A:0;G:0;C:1548;T:129;total:1677 | iSNV |
| F27 | F27-19 | 9359 | NS5 | 0.0532 | A:0;G:0;C:165;T:2936;total:3101 | iSNV |
| F27 | F27-19 | 9452 | NS5 | 0.0716 | A:208;G:2693;C:0;T:0;total:2901 | iSNV |
| F27 | F27-19 | 9818 | NS5 | 0.0847 | A:1;G:0;C:171;T:1846;total:2018 | iSNV |
| F27 | F27-19 | 10022 | NS5 | 0.0873 | A:0;G:1;C:211;T:2204;total:2416 | iSNV |
| F27 | F27-19 | 10181 | NS5 | 0.0203 | A:0;G:0;C:2263;T:47;total:2310 | iSNV |
| F27 | F27-19 | 10259 | NS5 | 0.0805 | A:2386;G:209;C:1;T:0;total:2596 | iSNV |
| F27 | F27-19 | 10358 | NS5 | 0.1252 | A:0;G:0;C:1621;T:232;total:1853 | iSNV |
| F27 | F27-19 | 10428 | 3'-UTR | 0.0248 | A:1;G:0;C:1527;T:39;total:1567 | iSNV |
| F27 | F27-19 | 10447 | 3'-UTR | 0.0243 | A:0;G:0;C:1203;T:30;total:1233 | iSNV |
| F27 | F27-19 | 10566 | 3'-UTR | 0.0683 | A:0;G:0;C:1786;T:131;total:1917 | iSNV |
| F27 | F27-2 | 568 | M | 0.0296 | A:5919;G:3;C:0;T:181;total:6103 | iSNV |
| F27 | F27-2 | 998 | E | 0.0737 | A:0;G:0;C:4812;T:383;total:5195 | iSNV |
| F27 | F27-2 | 1218 | E | 0.9852 | A:0;G:1;C:84;T:5644;total:5729 | SNP |
| F27 | F27-2 | 1413 | E | 0.1665 | A:5621;G:1123;C:0;T:0;total:6744 | iSNV |
| F27 | F27-2 | 1430 | E | 0.036 | A:0;G:0;C:6560;T:245;total:6805 | iSNV |
| F27 | F27-2 | 3869 | NS2A | 0.9319 | A:0;G:0;C:392;T:5364;total:5756 | iSNV |
| F27 | F27-2 | 4697 | NS3 | 0.0606 | A:0;G:0;C:419;T:6492;total:6911 | iSNV |
| F27 | F27-2 | 5952 | NS3 | 0.0641 | A:0;G:0;C:6488;T:445;total:6933 | iSNV |
| F27 | F27-2 | 7820 | NS5 | 0.0201 | A:0;G:0;C:5550;T:114;total:5664 | iSNV |
| F27 | F27-2 | 7985 | NS5 | 0.0326 | A:0;G:0;C:6871;T:232;total:7103 | iSNV |
| F27 | F27-2 | 9350 | NS5 | 0.0752 | A:0;G:0;C:7373;T:600;total:7973 | iSNV |
| F27 | F27-2 | 9359 | NS5 | 0.0572 | A:0;G:0;C:457;T:7530;total:7987 | iSNV |
| F27 | F27-2 | 9818 | NS5 | 0.031 | A:0;G:0;C:179;T:5593;total:5772 | iSNV |
| F27 | F27-2 | 10259 | NS5 | 0.0642 | A:7018;G:482;C:0;T:0;total:7500 | iSNV |
| F27 | F27-2 | 10376 | NS5 | 0.1608 | A:1069;G:5578;C:0;T:0;total:6647 | iSNV |
| F27 | F27-2 | 10428 | 3'-UTR | 0.0203 | A:0;G:0;C:6211;T:129;total:6340 | iSNV |
| F27 | F27-2 | 10566 | 3'-UTR | 0.0556 | A:1;G:0;C:4904;T:289;total:5194 | iSNV |
| F27 | F27-20 | 998 | E | 0.1722 | A:1;G:2;C:3884;T:809;total:4696 | iSNV |
| F27 | F27-20 | 1117 | E | 0.0296 | A:5139;G:157;C:0;T:0;total:5296 | iSNV |
| F27 | F27-20 | 1136 | E | 0.0295 | A:0;G:0;C:156;T:5126;total:5282 | iSNV |
| F27 | F27-20 | 1218 | E | 0.9911 | A:0;G:1;C:44;T:5008;total:5053 | SNP |
| F27 | F27-20 | 1672 | E | 0.0221 | A:1;G:0;C:4807;T:109;total:4917 | iSNV |
| F27 | F27-20 | 1721 | E | 0.0636 | A:4557;G:310;C:0;T:0;total:4867 | iSNV |
| F27 | F27-20 | 2009 | E | 0.0366 | A:0;G:0;C:4096;T:156;total:4252 | iSNV |
| F27 | F27-20 | 2230 | E | 0.0464 | A:0;G:0;C:4717;T:230;total:4947 | iSNV |
| F27 | F27-20 | 2357 | E | 0.0254 | A:3528;G:92;C:0;T:0;total:3620 | iSNV |
| F27 | F27-20 | 3869 | NS2A | 0.824 | A:0;G:0;C:837;T:3918;total:4755 | iSNV |
| F27 | F27-20 | 4697 | NS3 | 0.1518 | A:1;G:0;C:845;T:4718;total:5564 | iSNV |
| F27 | F27-20 | 5952 | NS3 | 0.1553 | A:0;G:0;C:5060;T:931;total:5991 | iSNV |
| F27 | F27-20 | 6786 | NS4A | 0.051 | A:0;G:0;C:4482;T:241;total:4723 | iSNV |
| F27 | F27-20 | 8190 | NS5 | 0.0246 | A:0;G:0;C:142;T:5616;total:5758 | iSNV |
| F27 | F27-20 | 8243 | NS5 | 0.02 | A:2;G:0;C:5459;T:112;total:5573 | iSNV |
| F27 | F27-20 | 9359 | NS5 | 0.1594 | A:0;G:1;C:1153;T:6077;total:7231 | iSNV |
| F27 | F27-20 | 10259 | NS5 | 0.1581 | A:5918;G:1112;C:0;T:0;total:7030 | iSNV |
| F27 | F27-20 | 10421 | 3'-UTR | 0.0243 | A:5736;G:143;C:0;T:0;total:5879 | iSNV |
| F27 | F27-20 | 10566 | 3'-UTR | 0.153 | A:1;G:0;C:4354;T:787;total:5142 | iSNV |
| F27 | F27-20 | 10617 | 3'-UTR | 0.0501 | A:4904;G:259;C:0;T:0;total:5163 | iSNV |
| F27 | F27-21 | 897 | M | 0.0269 | A:0;G:1;C:6291;T:174;total:6466 | iSNV |
| F27 | F27-21 | 998 | E | 0.0501 | A:9;G:0;C:6557;T:347;total:6913 | iSNV |
| F27 | F27-21 | 1043 | E | 0.0226 | A:0;G:0;C:7493;T:174;total:7667 | iSNV |
| F27 | F27-21 | 1218 | E | 0.9967 | A:0;G:0;C:26;T:7717;total:7743 | SNP |
| F27 | F27-21 | 3300 | NS1 | 0.0217 | A:0;G:0;C:169;T:7605;total:7774 | iSNV |
| F27 | F27-21 | 3661 | NS1 | 0.0668 | A:0;G:0;C:415;T:5797;total:6212 | iSNV |
| F27 | F27-21 | 3869 | NS2A | 0.95 | A:0;G:0;C:359;T:6821;total:7180 | iSNV |
| F27 | F27-21 | 4093 | NS2A | 0.0619 | A:5841;G:386;C:0;T:1;total:6228 | iSNV |
| F27 | F27-21 | 4284 | NS2B | 0.0217 | A:1;G:0;C:127;T:5724;total:5852 | iSNV |
| F27 | F27-21 | 4697 | NS3 | 0.0443 | A:0;G:0;C:366;T:7892;total:8258 | iSNV |
| F27 | F27-21 | 5952 | NS3 | 0.0427 | A:1;G:0;C:8606;T:384;total:8991 | iSNV |
| F27 | F27-21 | 5981 | NS3 | 0.0212 | A:1;G:8025;C:174;T:1;total:8201 | iSNV |
| F27 | F27-21 | 6004 | NS3 | 0.0621 | A:0;G:0;C:7557;T:501;total:8058 | iSNV |
| F27 | F27-21 | 6206 | NS3 | 0.0855 | A:0;G:0;C:6339;T:593;total:6932 | iSNV |
| F27 | F27-21 | 6950 | NS4A | 0.0414 | A:2;G:0;C:6728;T:291;total:7021 | iSNV |
| F27 | F27-21 | 7625 | NS4B | 0.026 | A:1;G:0;C:7396;T:198;total:7595 | iSNV |
| F27 | F27-21 | 7697 | NS5 | 0.0361 | A:0;G:7336;C:0;T:275;total:7611 | iSNV |
| F27 | F27-21 | 8194 | NS5 | 0.0716 | A:7673;G:592;C:0;T:0;total:8265 | iSNV |
| F27 | F27-21 | 9065 | NS5 | 0.0692 | A:545;G:7319;C:0;T:1;total:7865 | iSNV |
| F27 | F27-21 | 9359 | NS5 | 0.0477 | A:0;G:0;C:505;T:10069;total:10574 | iSNV |
| F27 | F27-21 | 10259 | NS5 | 0.049 | A:9345;G:482;C:1;T:0;total:9828 | iSNV |
| F27 | F27-21 | 10358 | NS5 | 0.0296 | A:2;G:1;C:8680;T:265;total:8948 | iSNV |
| F27 | F27-21 | 10428 | 3'-UTR | 0.0206 | A:0;G:0;C:8646;T:182;total:8828 | iSNV |
| F27 | F27-21 | 10566 | 3'-UTR | 0.0451 | A:1;G:1;C:7235;T:342;total:7579 | iSNV |
| F27 | F27-21 | 10578 | 3'-UTR | 0.0715 | A:0;G:0;C:536;T:6957;total:7493 | iSNV |
| F27 | F27-21 | 10766 | 3'-UTR | 0.0288 | A:240;G:8086;C:0;T:0;total:8326 | iSNV |
| F27 | F27-22 | 998 | E | 0.0964 | A:0;G:0;C:1545;T:165;total:1710 | iSNV |
| F27 | F27-22 | 1218 | E | 0.9821 | A:0;G:0;C:31;T:1698;total:1729 | SNP |
| F27 | F27-22 | 1397 | E | 0.0357 | A:0;G:0;C:2238;T:83;total:2321 | iSNV |
| F27 | F27-22 | 1461 | E | 0.1025 | A:0;G:0;C:214;T:1873;total:2087 | iSNV |
| F27 | F27-22 | 1712 | E | 0.0258 | A:0;G:1507;C:40;T:1;total:1548 | iSNV |
| F27 | F27-22 | 3137 | NS1 | 0.0336 | A:0;G:0;C:1553;T:54;total:1607 | iSNV |

| | | | | | | |
|---|---|---|---|---|---|---|
| F27 | F27-22 | 3671 | NS1 | 0.0504 | A:0;G:0;C:75;T:1412;total:1487 | iSNV |
| F27 | F27-22 | 3869 | NS2A | 0.8999 | A:0;G:0;C:198;T:1780;total:1978 | iSNV |
| F27 | F27-22 | 4319 | NS2B | 0.0368 | A:74;G:1935;C:0;T:1;total:2010 | iSNV |
| F27 | F27-22 | 4594 | NS2B | 0.0239 | A:1837;G:45;C:0;T:0;total:1882 | iSNV |
| F27 | F27-22 | 4697 | NS3 | 0.0773 | A:0;G:0;C:126;T:1502;total:1628 | iSNV |
| F27 | F27-22 | 4791 | NS3 | 0.0228 | A:0;G:1665;C:39;T:0;total:1704 | iSNV |
| F27 | F27-22 | 5654 | NS3 | 0.0417 | A:0;G:0;C:1791;T:78;total:1869 | iSNV |
| F27 | F27-22 | 5952 | NS3 | 0.0865 | A:0;G:0;C:1751;T:166;total:1917 | iSNV |
| F27 | F27-22 | 6157 | NS3 | 0.0354 | A:0;G:0;C:1495;T:55;total:1550 | iSNV |
| F27 | F27-22 | 6938 | NS4A | 0.0373 | A:0;G:0;C:1675;T:65;total:1740 | iSNV |
| F27 | F27-22 | 7784 | NS5 | 0.1019 | A:0;G:0;C:1981;T:225;total:2206 | iSNV |
| F27 | F27-22 | 9359 | NS5 | 0.0737 | A:0;G:0;C:193;T:2424;total:2617 | iSNV |
| F27 | F27-22 | 9986 | NS5 | 0.0211 | A:1761;G:38;C:0;T:0;total:1799 | iSNV |
| F27 | F27-22 | 10259 | NS5 | 0.085 | A:1636;G:152;C:0;T:0;total:1788 | iSNV |
| F27 | F27-22 | 10428 | 3'-UTR | 0.0238 | A:0;G:0;C:1553;T:38;total:1591 | iSNV |
| F27 | F27-22 | 10447 | 3'-UTR | 0.0291 | A:0;G:0;C:1233;T:37;total:1270 | iSNV |
| F27 | F27-22 | 10566 | 3'-UTR | 0.0661 | A:0;G:0;C:1540;T:109;total:1649 | iSNV |
| F27 | F27-23 | 353 | C | 0.0263 | A:3733;G:101;C:0;T:0;total:3834 | iSNV |
| F27 | F27-23 | 752 | M | 0.0322 | A:3752;G:125;C:0;T:0;total:3877 | iSNV |
| F27 | F27-23 | 803 | M | 0.0209 | A:1;G:2758;C:0;T:59;total:2818 | iSNV |
| F27 | F27-23 | 854 | M | 0.0264 | A:0;G:0;C:3086;T:84;total:3170 | iSNV |
| F27 | F27-23 | 998 | E | 0.1894 | A:0;G:0;C:2332;T:545;total:2877 | iSNV |
| F27 | F27-23 | 1007 | E | 0.0388 | A:0;G:0;C:2942;T:119;total:3061 | iSNV |
| F27 | F27-23 | 1117 | E | 0.0318 | A:2922;G:96;C:0;T:0;total:3018 | iSNV |
| F27 | F27-23 | 1218 | E | 0.9511 | A:0;G:0;C:144;T:2798;total:2942 | iSNV |
| F27 | F27-23 | 1416 | E | 0.0328 | A:3213;G:109;C:0;T:0;total:3322 | iSNV |
| F27 | F27-23 | 1430 | E | 0.0451 | A:0;G:0;C:3190;T:151;total:3341 | iSNV |
| F27 | F27-23 | 2417 | E | 0.0208 | A:0;G:0;C:45;T:2109;total:2154 | iSNV |
| F27 | F27-23 | 2664 | NS1 | 0.0226 | A:1;G:0;C:3019;T:70;total:3090 | iSNV |
| F27 | F27-23 | 3869 | NS2A | 0.782 | A:0;G:0;C:605;T:2169;total:2774 | iSNV |
| F27 | F27-23 | 4697 | NS3 | 0.153 | A:0;G:0;C:508;T:2812;total:3320 | iSNV |
| F27 | F27-23 | 5102 | NS3 | 0.0332 | A:0;G:0;C:2737;T:94;total:2831 | iSNV |
| F27 | F27-23 | 5645 | NS3 | 0.0227 | A:0;G:0;C:2744;T:64;total:2808 | iSNV |
| F27 | F27-23 | 5952 | NS3 | 0.1638 | A:0;G:1;C:2918;T:572;total:3491 | iSNV |
| F27 | F27-23 | 6151 | NS3 | 0.0252 | A:2126;G:0;C:1;T:55;total:2182 | iSNV |
| F27 | F27-23 | 7460 | NS4B | 0.0713 | A:139;G:1810;C:0;T:0;total:1949 | iSNV |
| F27 | F27-23 | 9359 | NS5 | 0.1612 | A:1;G:0;C:676;T:3514;total:4191 | iSNV |
| F27 | F27-23 | 10259 | NS5 | 0.183 | A:3227;G:723;C:0;T:0;total:3950 | iSNV |
| F27 | F27-23 | 10428 | 3'-UTR | 0.0455 | A:1;G:0;C:3144;T:150;total:3295 | iSNV |
| F27 | F27-23 | 10447 | 3'-UTR | 0.0351 | A:0;G:0;C:2851;T:104;total:2955 | iSNV |
| F27 | F27-23 | 10566 | 3'-UTR | 0.1536 | A:1;G:0;C:2550;T:463;total:3014 | iSNV |
| F27 | F27-24 | 353 | C | 0.0314 | A:4745;G:154;C:0;T:0;total:4899 | iSNV |
| F27 | F27-24 | 645 | M | 0.0315 | A:4176;G:136;C:0;T:1;total:4313 | iSNV |
| F27 | F27-24 | 919 | M | 0.0203 | A:3562;G:74;C:0;T:0;total:3636 | iSNV |
| F27 | F27-24 | 998 | E | 0.2261 | A:2;G:0;C:2681;T:784;total:3467 | iSNV |
| F27 | F27-24 | 1013 | E | 0.0434 | A:3393;G:0;C:154;T:0;total:3547 | iSNV |
| F27 | F27-24 | 1117 | E | 0.0384 | A:3597;G:144;C:0;T:0;total:3741 | iSNV |
| F27 | F27-24 | 1218 | E | 0.9328 | A:1;G:0;C:251;T:3480;total:3732 | iSNV |
| F27 | F27-24 | 1413 | E | 0.0373 | A:4075;G:158;C:0;T:0;total:4233 | iSNV |
| F27 | F27-24 | 1430 | E | 0.0414 | A:0;G:0;C:4048;T:175;total:4223 | iSNV |
| F27 | F27-24 | 1892 | E | 0.0387 | A:3328;G:134;C:0;T:0;total:3462 | iSNV |
| F27 | F27-24 | 3110 | NS1 | 0.0675 | A:3824;G:277;C:0;T:0;total:4101 | iSNV |
| F27 | F27-24 | 3257 | NS1 | 0.0526 | A:0;G:0;C:222;T:3994;total:4216 | iSNV |
| F27 | F27-24 | 3869 | NS2A | 0.7472 | A:1;G:0;C:946;T:2795;total:3742 | iSNV |
| F27 | F27-24 | 4073 | NS2A | 0.0205 | A:0;G:0;C:3153;T:66;total:3219 | iSNV |
| F27 | F27-24 | 4637 | NS3 | 0.0201 | A:4287;G:88;C:0;T:0;total:4375 | iSNV |
| F27 | F27-24 | 4663 | NS3 | 0.0202 | A:0;G:1;C:3914;T:81;total:3996 | iSNV |
| F27 | F27-24 | 4697 | NS3 | 0.1958 | A:0;G:0;C:851;T:3494;total:4345 | iSNV |
| F27 | F27-24 | 5465 | NS3 | 0.067 | A:4089;G:294;C:1;T:0;total:4384 | iSNV |
| F27 | F27-24 | 5952 | NS3 | 0.1889 | A:0;G:0;C:3472;T:809;total:4281 | iSNV |
| F27 | F27-24 | 6107 | NS3 | 0.0255 | A:0;G:0;C:3280;T:86;total:3366 | iSNV |
| F27 | F27-24 | 9359 | NS5 | 0.1829 | A:0;G:0;C:975;T:4355;total:5330 | iSNV |
| F27 | F27-24 | 10259 | NS5 | 0.2366 | A:3999;G:1240;C:0;T:1;total:5240 | iSNV |
| F27 | F27-24 | 10419 | 3'-UTR | 0.0208 | A:0;G:88;C:4059;T:66;total:4213 | iSNV |
| F27 | F27-24 | 10428 | 3'-UTR | 0.021 | A:0;G:1;C:4228;T:91;total:4320 | iSNV |
| F27 | F27-24 | 10447 | 3'-UTR | 0.045 | A:0;G:0;C:3713;T:175;total:3888 | iSNV |
| F27 | F27-24 | 10452 | 3'-UTR | 0.0223 | A:3760;G:86;C:1;T:0;total:3847 | iSNV |
| F27 | F27-24 | 10566 | 3'-UTR | 0.1968 | A:0;G:0;C:3032;T:743;total:3775 | iSNV |
| F27 | F27-25 | 323 | C | 0.0232 | A:32;G:1342;C:0;T:0;total:1374 | iSNV |
| F27 | F27-25 | 443 | C | 0.0303 | A:0;G:1563;C:0;T:49;total:1612 | iSNV |
| F27 | F27-25 | 491 | M | 0.0289 | A:0;G:0;C:54;T:1812;total:1866 | iSNV |
| F27 | F27-25 | 998 | E | 0.087 | A:0;G:0;C:1479;T:141;total:1620 | iSNV |
| F27 | F27-25 | 1117 | E | 0.2342 | A:1657;G:507;C:0;T:0;total:2164 | iSNV |
| F27 | F27-25 | 1218 | E | 0.9962 | A:0;G:0;C:7;T:1817;total:1824 | SNP |
| F27 | F27-25 | 2213 | E | 0.0273 | A:54;G:1923;C:0;T:0;total:1977 | iSNV |
| F27 | F27-25 | 2855 | NS1 | 0.2212 | A:545;G:1917;C:1;T:0;total:2463 | iSNV |
| F27 | F27-25 | 2948 | NS1 | 0.0223 | A:0;G:0;C:2272;T:52;total:2324 | iSNV |
| F27 | F27-25 | 3869 | NS2A | 0.8989 | A:0;G:0;C:184;T:1635;total:1819 | iSNV |
| F27 | F27-25 | 4049 | NS2A | 0.0219 | A:0;G:0;C:1248;T:28;total:1276 | iSNV |
| F27 | F27-25 | 4697 | NS3 | 0.0766 | A:0;G:0;C:110;T:1325;total:1435 | iSNV |
| F27 | F27-25 | 5558 | NS3 | 0.2065 | A:367;G:1408;C:1;T:1;total:1777 | iSNV |
| F27 | F27-25 | 5952 | NS3 | 0.0926 | A:0;G:0;C:1635;T:167;total:1802 | iSNV |

| | | | | | | |
|---|---|---|---|---|---|---|
| F27 | F27-25 | 9359 | NS5 | 0.079 | A:0;G:0;C:191;T:2225;total:2416 | iSNV |
| F27 | F27-25 | 9818 | NS5 | 0.0848 | A:0;G:0;C:145;T:1563;total:1708 | iSNV |
| F27 | F27-25 | 10259 | NS5 | 0.0834 | A:1603;G:146;C:0;T:0;total:1749 | iSNV |
| F27 | F27-25 | 10428 | 3'-UTR | 0.0387 | A:0;G:0;C:1339;T:54;total:1393 | iSNV |
| F27 | F27-25 | 10566 | 3'-UTR | 0.1007 | A:0;G:1;C:1356;T:152;total:1509 | iSNV |
| F27 | F27-26 | 998 | E | 0.1178 | A:0;G:0;C:2619;T:350;total:2969 | iSNV |
| F27 | F27-26 | 1043 | E | 0.0381 | A:1;G:0;C:3176;T:126;total:3303 | iSNV |
| F27 | F27-26 | 1218 | E | 0.9905 | A:0;G:0;C:32;T:3315;total:3347 | SNP |
| F27 | F27-26 | 2474 | E | 0.0229 | A:50;G:0;C:0;T:2132;total:2182 | iSNV |
| F27 | F27-26 | 2744 | NS1 | 0.0241 | A:0;G:92;C:0;T:3714;total:3806 | iSNV |
| F27 | F27-26 | 3257 | NS1 | 0.0311 | A:0;G:0;C:118;T:3669;total:3787 | iSNV |
| F27 | F27-26 | 3869 | NS2A | 0.8818 | A:0;G:0;C:383;T:2857;total:3240 | iSNV |
| F27 | F27-26 | 3993 | NS2A | 0.0217 | A:71;G:3192;C:0;T:0;total:3263 | iSNV |
| F27 | F27-26 | 4187 | NS2A | 0.0258 | A:1544;G:41;C:0;T:0;total:1585 | iSNV |
| F27 | F27-26 | 4289 | NS2B | 0.0209 | A:0;G:1;C:53;T:2478;total:2532 | iSNV |
| F27 | F27-26 | 4697 | NS3 | 0.1095 | A:0;G:0;C:385;T:3129;total:3514 | iSNV |
| F27 | F27-26 | 5952 | NS3 | 0.1059 | A:0;G:0;C:3274;T:388;total:3662 | iSNV |
| F27 | F27-26 | 6080 | NS3 | 0.0285 | A:80;G:2726;C:1;T:0;total:2807 | iSNV |
| F27 | F27-26 | 6626 | NS4A | 0.0257 | A:0;G:0;C:2881;T:76;total:2957 | iSNV |
| F27 | F27-26 | 7172 | NS4A | 0.0252 | A:0;G:0;C:69;T:2667;total:2736 | iSNV |
| F27 | F27-26 | 9359 | NS5 | 0.1161 | A:0;G:0;C:540;T:4110;total:4650 | iSNV |
| F27 | F27-26 | 10259 | NS5 | 0.1193 | A:4036;G:547;C:0;T:0;total:4583 | iSNV |
| F27 | F27-26 | 10428 | 3'-UTR | 0.0542 | A:0;G:0;C:3591;T:206;total:3797 | iSNV |
| F27 | F27-26 | 10429 | 3'-UTR | 0.0234 | A:3707;G:89;C:0;T:0;total:3796 | iSNV |
| F27 | F27-26 | 10566 | 3'-UTR | 0.0985 | A:0;G:0;C:3038;T:332;total:3370 | iSNV |
| F27 | F27-26 | 10663 | 3'-UTR | 0.0285 | A:0;G:0;C:3947;T:116;total:4063 | iSNV |
| F27 | F27-27 | 998 | E | 0.0615 | A:1;G:0;C:8462;T:555;total:9018 | iSNV |
| F27 | F27-27 | 1136 | E | 0.0369 | A:1;G:0;C:380;T:9916;total:10297 | iSNV |
| F27 | F27-27 | 1218 | E | 0.9985 | A:1;G:0;C:15;T:10037;total:10053 | SNP |
| F27 | F27-27 | 1413 | E | 0.0238 | A:11815;G:289;C:0;T:0;total:12104 | iSNV |
| F27 | F27-27 | 2664 | NS1 | 0.14 | A:0;G:0;C:9293;T:1513;total:10806 | iSNV |
| F27 | F27-27 | 3869 | NS2A | 0.9386 | A:2;G:0;C:634;T:9677;total:10313 | iSNV |
| F27 | F27-27 | 4049 | NS2A | 0.0211 | A:1;G:0;C:7828;T:169;total:7998 | iSNV |
| F27 | F27-27 | 4697 | NS3 | 0.0577 | A:1;G:2;C:629;T:10266;total:10898 | iSNV |
| F27 | F27-27 | 4970 | NS3 | 0.0205 | A:210;G:9989;C:0;T:0;total:10199 | iSNV |
| F27 | F27-27 | 5952 | NS3 | 0.055 | A:3;G:0;C:10906;T:636;total:11545 | iSNV |
| F27 | F27-27 | 7626 | NS4B | 0.1541 | A:3;G:1561;C:0;T:8562;total:10126 | iSNV |
| F27 | F27-27 | 7892 | NS5 | 0.0222 | A:9;G:11534;C:0;T:263;total:11806 | iSNV |
| F27 | F27-27 | 9359 | NS5 | 0.0571 | A:0;G:4;C:830;T:13690;total:14524 | iSNV |
| F27 | F27-27 | 9491 | NS5 | 0.021 | A:12002;G:258;C:0;T:1;total:12261 | iSNV |
| F27 | F27-27 | 9818 | NS5 | 0.0402 | A:0;G:0;C:427;T:10174;total:10601 | iSNV |
| F27 | F27-27 | 10259 | NS5 | 0.056 | A:13325;G:791;C:0;T:0;total:14116 | iSNV |
| F27 | F27-27 | 10376 | NS5 | 0.0256 | A:331;G:12572;C:0;T:3;total:12906 | iSNV |
| F27 | F27-27 | 10419 | 3'-UTR | 0.0203 | A:2;G:78;C:11621;T:243;total:11944 | iSNV |
| F27 | F27-27 | 10428 | 3'-UTR | 0.0387 | A:1;G:0;C:11726;T:473;total:12200 | iSNV |
| F27 | F27-27 | 10447 | 3'-UTR | 0.0284 | A:0;G:0;C:10573;T:310;total:10883 | iSNV |
| F27 | F27-27 | 10566 | 3'-UTR | 0.0618 | A:0;G:0;C:9818;T:647;total:10465 | iSNV |
| F27 | F27-28 | 353 | C | 0.0306 | A:1390;G:44;C:0;T:0;total:1434 | iSNV |
| F27 | F27-28 | 645 | M | 0.0348 | A:1989;G:72;C:0;T:3;total:2064 | iSNV |
| F27 | F27-28 | 803 | M | 0.0241 | A:0;G:1617;C:0;T:40;total:1657 | iSNV |
| F27 | F27-28 | 869 | M | 0.0721 | A:133;G:0;C:0;T:1710;total:1843 | iSNV |
| F27 | F27-28 | 897 | M | 0.0432 | A:0;G:0;C:1659;T:75;total:1734 | iSNV |
| F27 | F27-28 | 946 | M | 0.0709 | A:0;G:131;C:0;T:1716;total:1847 | iSNV |
| F27 | F27-28 | 998 | E | 0.0901 | A:0;G:0;C:1565;T:155;total:1720 | iSNV |
| F27 | F27-28 | 1117 | E | 0.031 | A:2275;G:73;C:0;T:0;total:2348 | iSNV |
| F27 | F27-28 | 1218 | E | 0.936 | A:1;G:1;C:116;T:1692;total:1810 | iSNV |
| F27 | F27-28 | 1430 | E | 0.0256 | A:0;G:1;C:1935;T:51;total:1987 | iSNV |
| F27 | F27-28 | 1478 | E | 0.063 | A:0;G:0;C:106;T:1575;total:1681 | iSNV |
| F27 | F27-28 | 2213 | E | 0.0379 | A:82;G:2076;C:0;T:0;total:2158 | iSNV |
| F27 | F27-28 | 2879 | NS1 | 0.0227 | A:0;G:0;C:2617;T:61;total:2678 | iSNV |
| F27 | F27-28 | 3869 | NS2A | 0.8629 | A:0;G:0;C:256;T:1611;total:1867 | iSNV |
| F27 | F27-28 | 4427 | NS2B | 0.0923 | A:0;G:0;C:1798;T:183;total:1981 | iSNV |
| F27 | F27-28 | 4697 | NS3 | 0.0616 | A:0;G:0;C:91;T:1385;total:1476 | iSNV |
| F27 | F27-28 | 4742 | NS3 | 0.0477 | A:0;G:0;C:71;T:1417;total:1488 | iSNV |
| F27 | F27-28 | 5654 | NS3 | 0.0249 | A:0;G:0;C:1761;T:45;total:1806 | iSNV |
| F27 | F27-28 | 5665 | NS3 | 0.0668 | A:0;G:0;C:1772;T:127;total:1899 | iSNV |
| F27 | F27-28 | 5952 | NS3 | 0.06 | A:0;G:0;C:1769;T:113;total:1882 | iSNV |
| F27 | F27-28 | 7592 | NS4B | 0.0627 | A:0;G:0;C:1911;T:128;total:2039 | iSNV |
| F27 | F27-28 | 8518 | NS5 | 0.0221 | A:2070;G:1;C:0;T:47;total:2118 | iSNV |
| F27 | F27-28 | 8789 | NS5 | 0.0295 | A:1378;G:42;C:0;T:0;total:1420 | iSNV |
| F27 | F27-28 | 9359 | NS5 | 0.064 | A:0;G:0;C:166;T:2426;total:2592 | iSNV |
| F27 | F27-28 | 9634 | NS5 | 0.0383 | A:0;G:0;C:82;T:2058;total:2140 | iSNV |
| F27 | F27-28 | 10259 | NS5 | 0.0922 | A:1723;G:175;C:0;T:0;total:1898 | iSNV |
| F27 | F27-28 | 10428 | 3'-UTR | 0.0445 | A:0;G:0;C:1329;T:62;total:1391 | iSNV |
| F27 | F27-28 | 10447 | 3'-UTR | 0.0398 | A:0;G:0;C:1108;T:46;total:1154 | iSNV |
| F27 | F27-28 | 10452 | 3'-UTR | 0.0552 | A:1145;G:67;C:0;T:0;total:1212 | iSNV |
| F27 | F27-28 | 10566 | 3'-UTR | 0.0648 | A:0;G:0;C:1443;T:100;total:1543 | iSNV |
| F27 | F27-29 | 998 | E | 0.0519 | A:1;G:0;C:7552;T:414;total:7967 | iSNV |
| F27 | F27-29 | 1218 | E | 0.9903 | A:0;G:0;C:84;T:8488;total:8572 | SNP |
| F27 | F27-29 | 1295 | E | 0.039 | A:9707;G:0;C:1;T:394;total:10102 | iSNV |
| F27 | F27-29 | 1413 | E | 0.1597 | A:8688;G:1652;C:1;T:1;total:10342 | iSNV |
| F27 | F27-29 | 1430 | E | 0.0235 | A:2;G:0;C:10220;T:247;total:10469 | iSNV |

| | | | | | | |
|---|---|---|---|---|---|---|
| F27 | F27-29 | 2333 | E | 0.028 | A:0;G:0;C:5993;T:173;total:6166 | iSNV |
| F27 | F27-29 | 2362 | E | 0.1419 | A:0;G:4763;C:788;T:1;total:5552 | iSNV |
| F27 | F27-29 | 2627 | NS1 | 0.0203 | A:3;G:0;C:8609;T:179;total:8791 | iSNV |
| F27 | F27-29 | 3023 | NS1 | 0.0397 | A:283;G:6843;C:0;T:0;total:7126 | iSNV |
| F27 | F27-29 | 3869 | NS2A | 0.9469 | A:0;G:0;C:443;T:7895;total:8338 | iSNV |
| F27 | F27-29 | 4187 | NS2A | 0.0372 | A:4188;G:162;C:0;T:0;total:4350 | iSNV |
| F27 | F27-29 | 4661 | NS3 | 0.0388 | A:1;G:0;C:8267;T:334;total:8602 | iSNV |
| F27 | F27-29 | 4697 | NS3 | 0.0443 | A:2;G:0;C:403;T:8692;total:9097 | iSNV |
| F27 | F27-29 | 5150 | NS3 | 0.0224 | A:7455;G:1;C:0;T:171;total:7627 | iSNV |
| F27 | F27-29 | 5702 | NS3 | 0.0705 | A:0;G:0;C:9368;T:711;total:10079 | iSNV |
| F27 | F27-29 | 5952 | NS3 | 0.0437 | A:0;G:0;C:9524;T:436;total:9960 | iSNV |
| F27 | F27-29 | 6753 | NS4A | 0.036 | A:1;G:6308;C:0;T:236;total:6545 | iSNV |
| F27 | F27-29 | 6827 | NS4A | 0.0205 | A:8090;G:170;C:0;T:0;total:8260 | iSNV |
| F27 | F27-29 | 7481 | NS4B | 0.0905 | A:0;G:0;C:520;T:5220;total:5740 | iSNV |
| F27 | F27-29 | 7558 | NS4B | 0.0267 | A:1;G:2;C:8324;T:229;total:8556 | iSNV |
| F27 | F27-29 | 9359 | NS5 | 0.0453 | A:0;G:0;C:550;T:11578;total:12128 | iSNV |
| F27 | F27-29 | 9557 | NS5 | 0.0269 | A:282;G:10191;C:0;T:2;total:10475 | iSNV |
| F27 | F27-29 | 9634 | NS5 | 0.0263 | A:0;G:0;C:262;T:9696;total:9958 | iSNV |
| F27 | F27-29 | 10259 | NS5 | 0.0494 | A:11516;G:599;C:0;T:0;total:12115 | iSNV |
| F27 | F27-29 | 10376 | NS5 | 0.16 | A:1730;G:9079;C:0;T:1;total:10810 | iSNV |
| F27 | F27-29 | 10428 | 3'-UTR | 0.0217 | A:1;G:0;C:10108;T:225;total:10334 | iSNV |
| F27 | F27-29 | 10447 | 3'-UTR | 0.0221 | A:1;G:0;C:9010;T:204;total:9215 | iSNV |
| F27 | F27-29 | 10566 | 3'-UTR | 0.0426 | A:4;G:0;C:8558;T:381;total:8943 | iSNV |
| F27 | F27-29 | 10589 | 3'-UTR | 0.0364 | A:0;G:335;C:0;T:8855;total:9190 | iSNV |
| F27 | F27-3 | 353 | C | 0.0218 | A:11479;G:257;C:0;T:1;total:11737 | iSNV |
| F27 | F27-3 | 645 | M | 0.0244 | A:10755;G:270;C:0;T:1;total:11026 | iSNV |
| F27 | F27-3 | 828 | M | 0.0229 | A:9188;G:216;C:0;T:0;total:9404 | iSNV |
| F27 | F27-3 | 996 | E | 0.1305 | A:1227;G:8171;C:0;T:0;total:9398 | iSNV |
| F27 | F27-3 | 998 | E | 0.1428 | A:2;G:0;C:7860;T:1310;total:9172 | iSNV |
| F27 | F27-3 | 1117 | E | 0.024 | A:9758;G:240;C:0;T:2;total:10000 | iSNV |
| F27 | F27-3 | 1218 | E | 0.9697 | A:0;G:0;C:294;T:9402;total:9696 | iSNV |
| F27 | F27-3 | 1430 | E | 0.0229 | A:1;G:0;C:11561;T:272;total:11834 | iSNV |
| F27 | F27-3 | 1508 | E | 0.0237 | A:5;G:0;C:9257;T:225;total:9487 | iSNV |
| F27 | F27-3 | 2465 | E | 0.1604 | A:0;G:1;C:5988;T:1145;total:7134 | iSNV |
| F27 | F27-3 | 2654 | NS1 | 0.0206 | A:0;G:0;C:232;T:11028;total:11260 | iSNV |
| F27 | F27-3 | 2780 | NS1 | 0.0256 | A:2;G:0;C:332;T:12603;total:12937 | iSNV |
| F27 | F27-3 | 3300 | NS1 | 0.0373 | A:0;G:0;C:417;T:10758;total:11175 | iSNV |
| F27 | F27-3 | 3858 | NS2A | 0.1046 | A:2;G:0;C:9011;T:1053;total:10066 | iSNV |
| F27 | F27-3 | 3869 | NS2A | 0.8513 | A:0;G:0;C:1521;T:8703;total:10224 | iSNV |
| F27 | F27-3 | 4049 | NS2A | 0.0217 | A:2;G:0;C:7807;T:174;total:7983 | iSNV |
| F27 | F27-3 | 4697 | NS3 | 0.1135 | A:0;G:0;C:1380;T:10772;total:12152 | iSNV |
| F27 | F27-3 | 5952 | NS3 | 0.1146 | A:2;G:0;C:10635;T:1377;total:12014 | iSNV |
| F27 | F27-3 | 6236 | NS3 | 0.021 | A:0;G:0;C:8983;T:193;total:9176 | iSNV |
| F27 | F27-3 | 7625 | NS4B | 0.0301 | A:1;G:0;C:10165;T:316;total:10482 | iSNV |
| F27 | F27-3 | 9359 | NS5 | 0.1103 | A:0;G:0;C:1523;T:12282;total:13805 | iSNV |
| F27 | F27-3 | 10259 | NS5 | 0.143 | A:11524;G:1924;C:0;T:0;total:13448 | iSNV |
| F27 | F27-3 | 10358 | NS5 | 0.0355 | A:1;G:0;C:11215;T:414;total:11630 | iSNV |
| F27 | F27-3 | 10428 | 3'-UTR | 0.0204 | A:1;G:0;C:10970;T:229;total:11200 | iSNV |
| F27 | F27-3 | 10447 | 3'-UTR | 0.0379 | A:0;G:0;C:9583;T:378;total:9961 | iSNV |
| F27 | F27-3 | 10452 | 3'-UTR | 0.0251 | A:9605;G:248;C:1;T:0;total:9854 | iSNV |
| F27 | F27-3 | 10566 | 3'-UTR | 0.1162 | A:0;G:1;C:8408;T:1106;total:9515 | iSNV |
| F27 | F27-30 | 998 | E | 0.0307 | A:0;G:0;C:5773;T:183;total:5956 | iSNV |
| F27 | F27-30 | 1218 | E | 0.9978 | A:0;G:0;C:15;T:6543;total:6558 | SNP |
| F27 | F27-30 | 1413 | E | 0.7338 | A:2083;G:5740;C:0;T:0;total:7823 | iSNV |
| F27 | F27-30 | 3869 | NS2A | 0.9675 | A:0;G:0;C:218;T:6485;total:6703 | iSNV |
| F27 | F27-30 | 4697 | NS3 | 0.0288 | A:0;G:0;C:212;T:7130;total:7342 | iSNV |
| F27 | F27-30 | 5580 | NS3 | 0.0206 | A:0;G:0;C:6971;T:147;total:7118 | iSNV |
| F27 | F27-30 | 5952 | NS3 | 0.0278 | A:2;G:0;C:7457;T:214;total:7673 | iSNV |
| F27 | F27-30 | 8394 | NS5 | 0.1957 | A:0;G:0;C:1570;T:6452;total:8022 | iSNV |
| F27 | F27-30 | 9359 | NS5 | 0.028 | A:0;G:1;C:268;T:9272;total:9541 | iSNV |
| F27 | F27-30 | 10259 | NS5 | 0.0287 | A:8850;G:262;C:0;T:0;total:9112 | iSNV |
| F27 | F27-30 | 10376 | NS5 | 0.7397 | A:5863;G:2064;C:0;T:0;total:7927 | iSNV |
| F27 | F27-30 | 10566 | 3'-UTR | 0.028 | A:3;G:0;C:6347;T:183;total:6533 | iSNV |
| F27 | F27-4 | 998 | E | 0.1192 | A:0;G:0;C:746;T:101;total:847 | iSNV |
| F27 | F27-4 | 1218 | E | 0.9798 | A:0;G:0;C:18;T:870;total:888 | iSNV |
| F27 | F27-4 | 1430 | E | 0.0385 | A:0;G:0;C:848;T:34;total:882 | iSNV |
| F27 | F27-4 | 2367 | E | 0.106 | A:706;G:84;C:1;T:1;total:792 | iSNV |
| F27 | F27-4 | 3869 | NS2A | 0.8533 | A:0;G:0;C:125;T:727;total:852 | iSNV |
| F27 | F27-4 | 4697 | NS3 | 0.1409 | A:0;G:0;C:97;T:591;total:688 | iSNV |
| F27 | F27-4 | 5952 | NS3 | 0.1206 | A:0;G:0;C:853;T:117;total:970 | iSNV |
| F27 | F27-4 | 6322 | NS3 | 0.0234 | A:0;G:0;C:873;T:21;total:894 | iSNV |
| F27 | F27-4 | 9359 | NS5 | 0.1145 | A:0;G:0;C:135;T:1044;total:1179 | iSNV |
| F27 | F27-4 | 10259 | NS5 | 0.119 | A:910;G:123;C:0;T:0;total:1033 | iSNV |
| F27 | F27-4 | 10452 | 3'-UTR | 0.029 | A:502;G:15;C:0;T:0;total:517 | iSNV |
| F27 | F27-4 | 10566 | 3'-UTR | 0.1128 | A:1;G:0;C:675;T:86;total:762 | iSNV |
| F27 | F27-5 | 294 | C | 0.0228 | A:137;G:5859;C:0;T:1;total:5997 | iSNV |
| F27 | F27-5 | 353 | C | 0.0243 | A:5569;G:139;C:0;T:0;total:5708 | iSNV |
| F27 | F27-5 | 399 | C | 0.0684 | A:416;G:0;C:5660;T:1;total:6077 | iSNV |
| F27 | F27-5 | 645 | M | 0.0257 | A:5137;G:136;C:0;T:0;total:5273 | iSNV |
| F27 | F27-5 | 897 | M | 0.0686 | A:1;G:1;C:3891;T:287;total:4180 | iSNV |
| F27 | F27-5 | 998 | E | 0.1064 | A:0;G:0;C:3852;T:459;total:4311 | iSNV |
| F27 | F27-5 | 1117 | E | 0.0233 | A:4644;G:111;C:0;T:0;total:4755 | iSNV |

| | | | | | | |
|---|---|---|---|---|---|---|
| F27 | F27-5 | 1218 | E | 0.9544 | A:1;G:0;C:216;T:4514;total:4731 | iSNV |
| F27 | F27-5 | 1413 | E | 0.0309 | A:5259;G:168;C:0;T:0;total:5427 | iSNV |
| F27 | F27-5 | 1430 | E | 0.0259 | A:0;G:0;C:5288;T:141;total:5429 | iSNV |
| F27 | F27-5 | 2126 | E | 0.044 | A:2;G:0;C:3900;T:180;total:4082 | iSNV |
| F27 | F27-5 | 2543 | NS1 | 0.0342 | A:0;G:0;C:3862;T:137;total:3999 | iSNV |
| F27 | F27-5 | 3869 | NS2A | 0.8947 | A:0;G:0;C:502;T:4262;total:4764 | iSNV |
| F27 | F27-5 | 4697 | NS3 | 0.0703 | A:0;G:0;C:386;T:5104;total:5490 | iSNV |
| F27 | F27-5 | 4790 | NS3 | 0.0212 | A:5437;G:118;C:0;T:7;total:5562 | iSNV |
| F27 | F27-5 | 5210 | NS3 | 0.0213 | A:0;G:0;C:3808;T:83;total:3891 | iSNV |
| F27 | F27-5 | 5952 | NS3 | 0.0762 | A:0;G:0;C:5259;T:434;total:5693 | iSNV |
| F27 | F27-5 | 6062 | NS3 | 0.0243 | A:0;G:0;C:4778;T:119;total:4897 | iSNV |
| F27 | F27-5 | 8130 | NS5 | 0.021 | A:0;G:0;C:6078;T:131;total:6209 | iSNV |
| F27 | F27-5 | 9341 | NS5 | 0.0201 | A:6333;G:130;C:0;T:0;total:6463 | iSNV |
| F27 | F27-5 | 9359 | NS5 | 0.0774 | A:1;G:0;C:505;T:6012;total:6518 | iSNV |
| F27 | F27-5 | 10259 | NS5 | 0.0948 | A:5743;G:602;C:0;T:3;total:6348 | iSNV |
| F27 | F27-5 | 10376 | NS5 | 0.0353 | A:197;G:5369;C:0;T:0;total:5566 | iSNV |
| F27 | F27-5 | 10407 | 3'-UTR | 0.0327 | A:0;G:0;C:173;T:5106;total:5279 | iSNV |
| F27 | F27-5 | 10428 | 3'-UTR | 0.0279 | A:0;G:0;C:4974;T:143;total:5117 | iSNV |
| F27 | F27-5 | 10447 | 3'-UTR | 0.0461 | A:0;G:0;C:4342;T:210;total:4552 | iSNV |
| F27 | F27-5 | 10452 | 3'-UTR | 0.02 | A:4394;G:90;C:0;T:1;total:4485 | iSNV |
| F27 | F27-5 | 10566 | 3'-UTR | 0.0758 | A:1;G:0;C:4226;T:347;total:4574 | iSNV |
| F27 | F27-6 | 353 | C | 0.0429 | A:11747;G:527;C:0;T:1;total:12275 | iSNV |
| F27 | F27-6 | 645 | M | 0.0455 | A:10549;G:503;C:0;T:0;total:11052 | iSNV |
| F27 | F27-6 | 998 | E | 0.0843 | A:3;G:1;C:8571;T:790;total:9365 | iSNV |
| F27 | F27-6 | 1117 | E | 0.0393 | A:9938;G:407;C:0;T:0;total:10345 | iSNV |
| F27 | F27-6 | 1218 | E | 0.9334 | A:1;G:1;C:678;T:9488;total:10168 | iSNV |
| F27 | F27-6 | 1430 | E | 0.1842 | A:2;G:2;C:9814;T:2218;total:12036 | iSNV |
| F27 | F27-6 | 2367 | E | 0.0346 | A:6774;G:243;C:0;T:1;total:7018 | iSNV |
| F27 | F27-6 | 3368 | NS1 | 0.0207 | A:190;G:8952;C:0;T:0;total:9142 | iSNV |
| F27 | F27-6 | 3869 | NS2A | 0.9023 | A:0;G:0;C:981;T:9050;total:10031 | iSNV |
| F27 | F27-6 | 4697 | NS3 | 0.0403 | A:0;G:0;C:467;T:11119;total:11586 | iSNV |
| F27 | F27-6 | 4783 | NS3 | 0.1826 | A:0;G:0;C:2012;T:9006;total:11018 | iSNV |
| F27 | F27-6 | 5294 | NS3 | 0.0254 | A:9109;G:238;C:0;T:0;total:9347 | iSNV |
| F27 | F27-6 | 5513 | NS3 | 0.0288 | A:0;G:0;C:351;T:11799;total:12150 | iSNV |
| F27 | F27-6 | 5753 | NS3 | 0.0223 | A:264;G:11542;C:0;T:3;total:11809 | iSNV |
| F27 | F27-6 | 5952 | NS3 | 0.0343 | A:1;G:0;C:11766;T:419;total:12186 | iSNV |
| F27 | F27-6 | 6206 | NS3 | 0.0252 | A:0;G:0;C:9617;T:249;total:9866 | iSNV |
| F27 | F27-6 | 7190 | NS4A | 0.0269 | A:2;G:8171;C:0;T:226;total:8399 | iSNV |
| F27 | F27-6 | 8432 | NS5 | 0.0246 | A:11984;G:2;C:303;T:0;total:12289 | iSNV |
| F27 | F27-6 | 8726 | NS5 | 0.0243 | A:0;G:0;C:248;T:9936;total:10184 | iSNV |
| F27 | F27-6 | 9359 | NS5 | 0.0361 | A:0;G:0;C:500;T:13340;total:13840 | iSNV |
| F27 | F27-6 | 9491 | NS5 | 0.0324 | A:12011;G:403;C:0;T:0;total:12414 | iSNV |
| F27 | F27-6 | 10259 | NS5 | 0.0786 | A:12627;G:1078;C:0;T:0;total:13705 | iSNV |
| F27 | F27-6 | 10428 | 3'-UTR | 0.0396 | A:1;G:0;C:11001;T:454;total:11456 | iSNV |
| F27 | F27-6 | 10447 | 3'-UTR | 0.0586 | A:1;G:0;C:9713;T:605;total:10319 | iSNV |
| F27 | F27-6 | 10566 | 3'-UTR | 0.0343 | A:0;G:0;C:9802;T:349;total:10151 | iSNV |
| F27 | F27-6 | 10577 | 3'-UTR | 0.0327 | A:1;G:0;C:9719;T:329;total:10049 | iSNV |
| F27 | F27-7 | 998 | E | 0.0907 | A:0;G:0;C:1985;T:198;total:2183 | iSNV |
| F27 | F27-7 | 1218 | E | 0.9876 | A:0;G:0;C:30;T:2388;total:2418 | SNP |
| F27 | F27-7 | 1413 | E | 0.0677 | A:2588;G:188;C:0;T:0;total:2776 | iSNV |
| F27 | F27-7 | 3257 | NS1 | 0.1038 | A:0;G:0;C:353;T:3047;total:3400 | iSNV |
| F27 | F27-7 | 3317 | NS1 | 0.0406 | A:0;G:0;C:3137;T:133;total:3270 | iSNV |
| F27 | F27-7 | 3869 | NS2A | 0.9182 | A:0;G:0;C:263;T:2951;total:3214 | iSNV |
| F27 | F27-7 | 4697 | NS3 | 0.0676 | A:0;G:0;C:215;T:2964;total:3179 | iSNV |
| F27 | F27-7 | 5952 | NS3 | 0.0777 | A:0;G:0;C:3215;T:271;total:3486 | iSNV |
| F27 | F27-7 | 6322 | NS3 | 0.0393 | A:0;G:0;C:2270;T:93;total:2363 | iSNV |
| F27 | F27-7 | 7721 | NS5 | 0.0211 | A:2683;G:58;C:1;T:0;total:2742 | iSNV |
| F27 | F27-7 | 9359 | NS5 | 0.0628 | A:0;G:0;C:219;T:3267;total:3486 | iSNV |
| F27 | F27-7 | 9370 | NS5 | 0.0888 | A:316;G:1;C:0;T:3240;total:3557 | iSNV |
| F27 | F27-7 | 9690 | NS5 | 0.0611 | A:119;G:1827;C:0;T:0;total:1946 | iSNV |
| F27 | F27-7 | 9818 | NS5 | 0.0202 | A:0;G:0;C:43;T:2078;total:2121 | iSNV |
| F27 | F27-7 | 10259 | NS5 | 0.0818 | A:2266;G:202;C:0;T:0;total:2468 | iSNV |
| F27 | F27-7 | 10376 | NS5 | 0.0824 | A:162;G:1802;C:0;T:0;total:1964 | iSNV |
| F27 | F27-7 | 10428 | 3'-UTR | 0.0359 | A:0;G:0;C:1634;T:61;total:1695 | iSNV |
| F27 | F27-7 | 10566 | 3'-UTR | 0.077 | A:1;G:0;C:1736;T:145;total:1882 | iSNV |
| F27 | F27-8 | 869 | M | 0.5045 | A:2789;G:1;C:0;T:2741;total:5531 | iSNV |
| F27 | F27-8 | 998 | E | 0.0504 | A:3;G:0;C:4684;T:249;total:4936 | iSNV |
| F27 | F27-8 | 1218 | E | 0.9918 | A:0;G:0;C:43;T:5196;total:5239 | SNP |
| F27 | F27-8 | 1430 | E | 0.5367 | A:0;G:1;C:2983;T:3454;total:6438 | iSNV |
| F27 | F27-8 | 3131 | NS1 | 0.1808 | A:5063;G:1118;C:0;T:2;total:6183 | iSNV |
| F27 | F27-8 | 3869 | NS2A | 0.9463 | A:0;G:0;C:318;T:5594;total:5912 | iSNV |
| F27 | F27-8 | 4697 | NS3 | 0.044 | A:0;G:0;C:293;T:6353;total:6646 | iSNV |
| F27 | F27-8 | 5952 | NS3 | 0.0479 | A:1;G:0;C:6295;T:317;total:6613 | iSNV |
| F27 | F27-8 | 7172 | NS4A | 0.024 | A:0;G:0;C:118;T:4782;total:4900 | iSNV |
| F27 | F27-8 | 7509 | NS4B | 0.0333 | A:141;G:4093;C:0;T:0;total:4234 | iSNV |
| F27 | F27-8 | 9359 | NS5 | 0.0433 | A:0;G:0;C:316;T:6969;total:7285 | iSNV |
| F27 | F27-8 | 9818 | NS5 | 0.0211 | A:0;G:0;C:109;T:5037;total:5146 | iSNV |
| F27 | F27-8 | 10259 | NS5 | 0.0515 | A:6313;G:343;C:0;T:1;total:6657 | iSNV |
| F27 | F27-8 | 10428 | 3'-UTR | 0.025 | A:0;G:1;C:5575;T:143;total:5719 | iSNV |
| F27 | F27-8 | 10447 | 3'-UTR | 0.0201 | A:1;G:0;C:4971;T:102;total:5074 | iSNV |
| F27 | F27-8 | 10452 | 3'-UTR | 0.0288 | A:4851;G:144;C:0;T:0;total:4995 | iSNV |
| F27 | F27-8 | 10566 | 3'-UTR | 0.0464 | A:1;G:0;C:4496;T:219;total:4716 | iSNV |

| | | | | | | |
|---|---|---|---|---|---|---|
| F27 | F27-9 | 900 | M | 0.037 | A:175;G:4543;C:0;T:0;total:4718 | iSNV |
| F27 | F27-9 | 998 | E | 0.0661 | A:0;G:0;C:4770;T:338;total:5108 | iSNV |
| F27 | F27-9 | 1218 | E | 0.9976 | A:0;G:0;C:14;T:5703;total:5717 | SNP |
| F27 | F27-9 | 1413 | E | 0.0235 | A:6384;G:154;C:0;T:0;total:6538 | iSNV |
| F27 | F27-9 | 1430 | E | 0.0403 | A:0;G:0;C:6255;T:263;total:6518 | iSNV |
| F27 | F27-9 | 2258 | E | 0.0391 | A:0;G:0;C:178;T:4374;total:4552 | iSNV |
| F27 | F27-9 | 2448 | E | 0.0325 | A:3;G:0;C:4155;T:140;total:4298 | iSNV |
| F27 | F27-9 | 3869 | NS2A | 0.9341 | A:0;G:0;C:420;T:5951;total:6371 | iSNV |
| F27 | F27-9 | 4187 | NS2A | 0.0606 | A:3034;G:196;C:0;T:0;total:3230 | iSNV |
| F27 | F27-9 | 4319 | NS2B | 0.0388 | A:205;G:5075;C:0;T:0;total:5280 | iSNV |
| F27 | F27-9 | 4697 | NS3 | 0.0625 | A:0;G:0;C:455;T:6819;total:7274 | iSNV |
| F27 | F27-9 | 4718 | NS3 | 0.0243 | A:0;G:0;C:7001;T:175;total:7176 | iSNV |
| F27 | F27-9 | 5952 | NS3 | 0.0606 | A:0;G:0;C:6716;T:434;total:7150 | iSNV |
| F27 | F27-9 | 6440 | NS3 | 0.0318 | A:1;G:0;C:5407;T:178;total:5586 | iSNV |
| F27 | F27-9 | 6938 | NS4A | 0.0356 | A:0;G:0;C:5146;T:190;total:5336 | iSNV |
| F27 | F27-9 | 9359 | NS5 | 0.0651 | A:0;G:2;C:500;T:7168;total:7670 | iSNV |
| F27 | F27-9 | 9533 | NS5 | 0.0289 | A:0;G:0;C:198;T:6631;total:6829 | iSNV |
| F27 | F27-9 | 9728 | NS5 | 0.0333 | A:0;G:1;C:5356;T:185;total:5542 | iSNV |
| F27 | F27-9 | 9860 | NS5 | 0.0269 | A:0;G:1;C:161;T:5823;total:5985 | iSNV |
| F27 | F27-9 | 10259 | NS5 | 0.0636 | A:6651;G:452;C:0;T:0;total:7103 | iSNV |
| F27 | F27-9 | 10376 | NS5 | 0.0266 | A:166;G:6067;C:0;T:0;total:6233 | iSNV |
| F27 | F27-9 | 10428 | 3'-UTR | 0.0274 | A:0;G:0;C:5659;T:160;total:5819 | iSNV |
| F27 | F27-9 | 10452 | 3'-UTR | 0.0214 | A:4965;G:109;C:0;T:0;total:5074 | iSNV |
| F27 | F27-9 | 10566 | 3'-UTR | 0.0593 | A:0;G:0;C:4600;T:290;total:4890 | iSNV |
| F30 | F30-1 | 897 | M | 0.02 | A:0;G:4;C:2681;T:55;total:2740 | iSNV |
| F30 | F30-1 | 930 | M | 0.0443 | A:0;G:0;C:2798;T:130;total:2928 | iSNV |
| F30 | F30-1 | 993 | E | 0.0226 | A:2850;G:66;C:0;T:0;total:2916 | iSNV |
| F30 | F30-1 | 998 | E | 0.1764 | A:0;G:0;C:2427;T:520;total:2947 | iSNV |
| F30 | F30-1 | 1218 | E | 0.9983 | A:0;G:0;C:6;T:3405;total:3411 | SNP |
| F30 | F30-1 | 2213 | E | 0.0319 | A:99;G:2999;C:0;T:0;total:3098 | iSNV |
| F30 | F30-1 | 2376 | E | 0.0308 | A:0;G:0;C:2044;T:65;total:2109 | iSNV |
| F30 | F30-1 | 3341 | NS1 | 0.022 | A:3676;G:83;C:0;T:0;total:3759 | iSNV |
| F30 | F30-1 | 3869 | NS2A | 0.8249 | A:0;G:0;C:685;T:3225;total:3910 | iSNV |
| F30 | F30-1 | 3926 | NS2A | 0.0256 | A:0;G:0;C:4331;T:114;total:4445 | iSNV |
| F30 | F30-1 | 4226 | NS2B | 0.0253 | A:0;G:0;C:49;T:1883;total:1932 | iSNV |
| F30 | F30-1 | 4559 | NS2B | 0.0369 | A:0;G:0;C:3651;T:140;total:3791 | iSNV |
| F30 | F30-1 | 4697 | NS3 | 0.1589 | A:0;G:0;C:623;T:3297;total:3920 | iSNV |
| F30 | F30-1 | 5952 | NS3 | 0.1663 | A:0;G:0;C:3529;T:704;total:4233 | iSNV |
| F30 | F30-1 | 7741 | NS5 | 0.0319 | A:3450;G:114;C:0;T:0;total:3564 | iSNV |
| F30 | F30-1 | 8651 | NS5 | 0.0281 | A:2727;G:79;C:0;T:0;total:2806 | iSNV |
| F30 | F30-1 | 9359 | NS5 | 0.1687 | A:0;G:0;C:829;T:4083;total:4912 | iSNV |
| F30 | F30-1 | 9983 | NS5 | 0.0392 | A:1;G:0;C:3011;T:123;total:3135 | iSNV |
| F30 | F30-1 | 10259 | NS5 | 0.1705 | A:3565;G:733;C:0;T:0;total:4298 | iSNV |
| F30 | F30-1 | 10428 | 3'-UTR | 0.0561 | A:0;G:0;C:3645;T:217;total:3862 | iSNV |
| F30 | F30-1 | 10447 | 3'-UTR | 0.022 | A:1;G:0;C:3199;T:72;total:3272 | iSNV |
| F30 | F30-1 | 10566 | 3'-UTR | 0.1788 | A:0;G:0;C:2488;T:542;total:3030 | iSNV |
| F30 | F30-10 | 998 | E | 0.0705 | A:0;G:0;C:1567;T:119;total:1686 | iSNV |
| F30 | F30-10 | 1218 | E | 0.9968 | A:0;G:0;C:6;T:1840;total:1846 | SNP |
| F30 | F30-10 | 1430 | E | 0.0712 | A:0;G:0;C:1993;T:153;total:2146 | iSNV |
| F30 | F30-10 | 1550 | E | 0.0218 | A:36;G:1613;C:0;T:0;total:1649 | iSNV |
| F30 | F30-10 | 2474 | E | 0.0347 | A:43;G:0;C:0;T:1193;total:1236 | iSNV |
| F30 | F30-10 | 3317 | NS1 | 0.0413 | A:0;G:0;C:2089;T:90;total:2179 | iSNV |
| F30 | F30-10 | 3869 | NS2A | 0.93 | A:0;G:0;C:153;T:2031;total:2184 | iSNV |
| F30 | F30-10 | 4697 | NS3 | 0.0541 | A:0;G:0;C:121;T:2113;total:2234 | iSNV |
| F30 | F30-10 | 4745 | NS3 | 0.0229 | A:0;G:0;C:2344;T:55;total:2399 | iSNV |
| F30 | F30-10 | 4783 | NS3 | 0.0221 | A:0;G:0;C:49;T:2166;total:2215 | iSNV |
| F30 | F30-10 | 5952 | NS3 | 0.0644 | A:0;G:0;C:2308;T:159;total:2467 | iSNV |
| F30 | F30-10 | 6152 | NS3 | 0.0236 | A:1361;G:33;C:0;T:0;total:1394 | iSNV |
| F30 | F30-10 | 6157 | NS3 | 0.0224 | A:0;G:0;C:1391;T:32;total:1423 | iSNV |
| F30 | F30-10 | 6206 | NS3 | 0.0819 | A:0;G:0;C:1714;T:153;total:1867 | iSNV |
| F30 | F30-10 | 6322 | NS3 | 0.0306 | A:0;G:0;C:1741;T:55;total:1796 | iSNV |
| F30 | F30-10 | 6900 | NS4A | 0.0233 | A:39;G:1632;C:0;T:0;total:1671 | iSNV |
| F30 | F30-10 | 9359 | NS5 | 0.0696 | A:0;G:0;C:186;T:2485;total:2671 | iSNV |
| F30 | F30-10 | 10259 | NS5 | 0.0541 | A:2286;G:131;C:0;T:0;total:2417 | iSNV |
| F30 | F30-10 | 10428 | 3'-UTR | 0.0415 | A:0;G:0;C:2009;T:87;total:2096 | iSNV |
| F30 | F30-10 | 10447 | 3'-UTR | 0.023 | A:0;G:0;C:1777;T:42;total:1819 | iSNV |
| F30 | F30-10 | 10566 | 3'-UTR | 0.0591 | A:1;G:0;C:1559;T:98;total:1658 | iSNV |
| F30 | F30-10 | 10632 | 3'-UTR | 0.021 | A:39;G:0;C:1;T:1814;total:1854 | iSNV |
| F30 | F30-11 | 998 | E | 0.08 | A:0;G:0;C:2195;T:191;total:2386 | iSNV |
| F30 | F30-11 | 1218 | E | 0.9858 | A:0;G:0;C:38;T:2630;total:2668 | SNP |
| F30 | F30-11 | 1514 | E | 0.0531 | A:2154;G:0;C:121;T:0;total:2275 | iSNV |
| F30 | F30-11 | 1797 | E | 0.0278 | A:86;G:0;C:0;T:2999;total:3085 | iSNV |
| F30 | F30-11 | 3149 | NS1 | 0.0659 | A:0;G:0;C:239;T:3387;total:3626 | iSNV |
| F30 | F30-11 | 3869 | NS2A | 0.9086 | A:0;G:0;C:279;T:2771;total:3050 | iSNV |
| F30 | F30-11 | 4697 | NS3 | 0.0842 | A:0;G:0;C:265;T:2880;total:3145 | iSNV |
| F30 | F30-11 | 5952 | NS3 | 0.0771 | A:1;G:0;C:3217;T:269;total:3487 | iSNV |
| F30 | F30-11 | 9359 | NS5 | 0.0949 | A:0;G:0;C:366;T:3489;total:3855 | iSNV |
| F30 | F30-11 | 9880 | NS5 | 0.1733 | A:476;G:2270;C:0;T:0;total:2746 | iSNV |
| F30 | F30-11 | 10259 | NS5 | 0.0789 | A:3087;G:265;C:0;T:3;total:3355 | iSNV |
| F30 | F30-11 | 10419 | 3'-UTR | 0.0233 | A:0;G:1;C:2800;T:67;total:2868 | iSNV |
| F30 | F30-11 | 10428 | 3'-UTR | 0.0478 | A:0;G:0;C:2804;T:141;total:2945 | iSNV |
| F30 | F30-11 | 10566 | 3'-UTR | 0.0829 | A:0;G:0;C:2332;T:211;total:2543 | iSNV |

| | | | | | | |
|---|---|---|---|---|---|---|
| F30 | F30-11 | 10589 | 3'-UTR | 0.1676 | A:0;G:445;C:0;T:2209;total:2654 | iSNV |
| F30 | F30-12 | 353 | C | 0.0731 | A:2900;G:229;C:0;T:0;total:3129 | iSNV |
| F30 | F30-12 | 645 | M | 0.0907 | A:2376;G:237;C:0;T:0;total:2613 | iSNV |
| F30 | F30-12 | 869 | M | 0.0391 | A:21;G:103;C:0;T:2507;total:2631 | iSNV |
| F30 | F30-12 | 998 | E | 0.2507 | A:0;G:0;C:1700;T:569;total:2269 | iSNV |
| F30 | F30-12 | 1083 | E | 0.078 | A:2067;G:175;C:0;T:0;total:2242 | iSNV |
| F30 | F30-12 | 1117 | E | 0.142 | A:2192;G:363;C:0;T:0;total:2555 | iSNV |
| F30 | F30-12 | 1218 | E | 0.9101 | A:0;G:0;C:220;T:2226;total:2446 | iSNV |
| F30 | F30-12 | 2277 | E | 0.092 | A:0;G:0;C:170;T:1676;total:1846 | iSNV |
| F30 | F30-12 | 3869 | NS2A | 0.7482 | A:0;G:0;C:750;T:2228;total:2978 | iSNV |
| F30 | F30-12 | 4697 | NS3 | 0.1512 | A:0;G:0;C:461;T:2586;total:3047 | iSNV |
| F30 | F30-12 | 5558 | NS3 | 0.059 | A:188;G:2997;C:0;T:0;total:3185 | iSNV |
| F30 | F30-12 | 5952 | NS3 | 0.1577 | A:0;G:0;C:2642;T:495;total:3137 | iSNV |
| F30 | F30-12 | 8198 | NS5 | 0.0304 | A:2768;G:87;C:0;T:0;total:2855 | iSNV |
| F30 | F30-12 | 9359 | NS5 | 0.1616 | A:0;G:0;C:592;T:3071;total:3663 | iSNV |
| F30 | F30-12 | 9491 | NS5 | 0.0314 | A:2494;G:81;C:0;T:0;total:2575 | iSNV |
| F30 | F30-12 | 10259 | NS5 | 0.2377 | A:2430;G:758;C:0;T:0;total:3188 | iSNV |
| F30 | F30-12 | 10428 | 3'-UTR | 0.0727 | A:0;G:0;C:2434;T:191;total:2625 | iSNV |
| F30 | F30-12 | 10447 | 3'-UTR | 0.1163 | A:0;G:0;C:1990;T:262;total:2252 | iSNV |
| F30 | F30-12 | 10566 | 3'-UTR | 0.1537 | A:0;G:0;C:1833;T:333;total:2166 | iSNV |
| F30 | F30-12 | 10589 | 3'-UTR | 0.0217 | A:1;G:49;C:2;T:2206;total:2258 | iSNV |
| F30 | F30-12 | 10592 | 3'-UTR | 0.0223 | A:0;G:0;C:2276;T:52;total:2328 | iSNV |
| F30 | F30-13 | 395 | C | 0.8152 | A:1131;G:4988;C:0;T:0;total:6119 | iSNV |
| F30 | F30-13 | 1218 | E | 0.999 | A:1;G:0;C:4;T:4608;total:4613 | SNP |
| F30 | F30-13 | 1413 | E | 0.8227 | A:966;G:4480;C:0;T:1;total:5447 | iSNV |
| F30 | F30-13 | 3869 | NS2A | 0.982 | A:0;G:0;C:81;T:4400;total:4481 | SNP |
| F30 | F30-13 | 9161 | NS5 | 0.0383 | A:1;G:0;C:251;T:6297;total:6549 | iSNV |
| F30 | F30-13 | 10376 | NS5 | 0.8257 | A:4378;G:925;C:0;T:1;total:5304 | iSNV |
| F30 | F30-13 | 10428 | 3'-UTR | 0.0321 | A:1;G:0;C:4936;T:164;total:5101 | iSNV |
| F30 | F30-14 | 854 | M | 0.0237 | A:0;G:0;C:3046;T:74;total:3120 | iSNV |
| F30 | F30-14 | 998 | E | 0.1375 | A:0;G:0;C:2432;T:388;total:2820 | iSNV |
| F30 | F30-14 | 1117 | E | 0.2901 | A:2307;G:944;C:0;T:2;total:3253 | iSNV |
| F30 | F30-14 | 1218 | E | 0.9915 | A:0;G:0;C:27;T:3142;total:3169 | SNP |
| F30 | F30-14 | 2282 | E | 0.0289 | A:0;G:0;C:2347;T:70;total:2417 | iSNV |
| F30 | F30-14 | 3869 | NS2A | 0.8368 | A:0;G:0;C:507;T:2599;total:3106 | iSNV |
| F30 | F30-14 | 4697 | NS3 | 0.1456 | A:0;G:0;C:518;T:3039;total:3557 | iSNV |
| F30 | F30-14 | 5558 | NS3 | 0.2594 | A:938;G:2677;C:0;T:0;total:3615 | iSNV |
| F30 | F30-14 | 5952 | NS3 | 0.1331 | A:0;G:0;C:3228;T:496;total:3724 | iSNV |
| F30 | F30-14 | 6401 | NS3 | 0.0299 | A:0;G:0;C:86;T:2790;total:2876 | iSNV |
| F30 | F30-14 | 6557 | NS4A | 0.0216 | A:2709;G:0;C:0;T:60;total:2769 | iSNV |
| F30 | F30-14 | 6786 | NS4A | 0.0248 | A:0;G:0;C:2784;T:71;total:2855 | iSNV |
| F30 | F30-14 | 7232 | NS4A | 0.0243 | A:0;G:0;C:2242;T:56;total:2298 | iSNV |
| F30 | F30-14 | 8195 | NS5 | 0.0205 | A:2;G:0;C:3327;T:70;total:3399 | iSNV |
| F30 | F30-14 | 9359 | NS5 | 0.1325 | A:0;G:1;C:554;T:3626;total:4181 | iSNV |
| F30 | F30-14 | 9370 | NS5 | 0.0748 | A:0;G:319;C:2;T:3940;total:4261 | iSNV |
| F30 | F30-14 | 9634 | NS5 | 0.0201 | A:0;G:0;C:71;T:3450;total:3521 | iSNV |
| F30 | F30-14 | 10259 | NS5 | 0.1462 | A:3725;G:638;C:0;T:0;total:4363 | iSNV |
| F30 | F30-14 | 10428 | 3'-UTR | 0.0464 | A:0;G:0;C:3466;T:169;total:3635 | iSNV |
| F30 | F30-14 | 10447 | 3'-UTR | 0.0221 | A:0;G:0;C:3223;T:73;total:3296 | iSNV |
| F30 | F30-14 | 10452 | 3'-UTR | 0.0204 | A:3158;G:66;C:0;T:0;total:3224 | iSNV |
| F30 | F30-14 | 10566 | 3'-UTR | 0.1459 | A:1;G:1;C:2638;T:451;total:3091 | iSNV |
| F30 | F30-14 | 10567 | 3'-UTR | 0.0231 | A:0;G:0;C:71;T:2993;total:3064 | iSNV |
| F30 | F30-15 | 197 | C | 0.0309 | A:8673;G:277;C:0;T:0;total:8950 | iSNV |
| F30 | F30-15 | 869 | M | 0.0235 | A:150;G:1;C:0;T:6222;total:6373 | iSNV |
| F30 | F30-15 | 998 | E | 0.0904 | A:0;G:0;C:5202;T:517;total:5719 | iSNV |
| F30 | F30-15 | 1218 | E | 0.9992 | A:0;G:1;C:4;T:6110;total:6115 | SNP |
| F30 | F30-15 | 1413 | E | 0.362 | A:4678;G:2656;C:0;T:1;total:7335 | iSNV |
| F30 | F30-15 | 1797 | E | 0.0258 | A:0;G:184;C:0;T:6924;total:7108 | iSNV |
| F30 | F30-15 | 3869 | NS2A | 0.9102 | A:0;G:0;C:597;T:6045;total:6642 | iSNV |
| F30 | F30-15 | 4697 | NS3 | 0.0794 | A:0;G:0;C:574;T:6649;total:7223 | iSNV |
| F30 | F30-15 | 5952 | NS3 | 0.0908 | A:3;G:0;C:6842;T:684;total:7529 | iSNV |
| F30 | F30-15 | 6779 | NS4A | 0.0225 | A:5494;G:127;C:0;T:0;total:5621 | iSNV |
| F30 | F30-15 | 7608 | NS4B | 0.0226 | A:5909;G:137;C:0;T:0;total:6046 | iSNV |
| F30 | F30-15 | 8430 | NS5 | 0.3576 | A:0;G:0;C:4791;T:2668;total:7459 | iSNV |
| F30 | F30-15 | 9359 | NS5 | 0.0847 | A:1;G:0;C:702;T:7580;total:8283 | iSNV |
| F30 | F30-15 | 9634 | NS5 | 0.0568 | A:0;G:0;C:377;T:6259;total:6636 | iSNV |
| F30 | F30-15 | 10259 | NS5 | 0.0852 | A:7551;G:704;C:1;T:0;total:8256 | iSNV |
| F30 | F30-15 | 10376 | NS5 | 0.3524 | A:2582;G:4743;C:0;T:1;total:7326 | iSNV |
| F30 | F30-15 | 10428 | 3'-UTR | 0.024 | A:0;G:2;C:6664;T:164;total:6830 | iSNV |
| F30 | F30-15 | 10566 | 3'-UTR | 0.0862 | A:0;G:0;C:5268;T:497;total:5765 | iSNV |
| F30 | F30-15 | 10572 | 3'-UTR | 0.0206 | A:5629;G:119;C:1;T:2;total:5751 | iSNV |
| F30 | F30-16 | 293 | C | 0.0432 | A:3337;G:151;C:0;T:0;total:3488 | iSNV |
| F30 | F30-16 | 469 | C | 0.0545 | A:0;G:0;C:3521;T:203;total:3724 | iSNV |
| F30 | F30-16 | 998 | E | 0.2412 | A:0;G:0;C:2022;T:643;total:2665 | iSNV |
| F30 | F30-16 | 1218 | E | 0.9856 | A:0;G:2;C:40;T:2873;total:2915 | SNP |
| F30 | F30-16 | 1430 | E | 0.0269 | A:0;G:0;C:3606;T:100;total:3706 | iSNV |
| F30 | F30-16 | 2218 | E | 0.0461 | A:0;G:0;C:2665;T:129;total:2794 | iSNV |
| F30 | F30-16 | 3869 | NS2A | 0.7524 | A:0;G:0;C:735;T:2233;total:2968 | iSNV |
| F30 | F30-16 | 3965 | NS2A | 0.0234 | A:3;G:0;C:3202;T:77;total:3282 | iSNV |
| F30 | F30-16 | 4049 | NS2A | 0.0374 | A:0;G:0;C:2237;T:87;total:2324 | iSNV |
| F30 | F30-16 | 4697 | NS3 | 0.2285 | A:1;G:0;C:753;T:2541;total:3295 | iSNV |
| F30 | F30-16 | 4926 | NS3 | 0.023 | A:0;G:2970;C:0;T:70;total:3040 | iSNV |

| | | | | | | |
|---|---|---|---|---|---|---|
| F30 | F30-16 | 5952 | NS3 | 0.2245 | A:0;G:0;C:2479;T:718;total:3197 | iSNV |
| F30 | F30-16 | 6672 | NS4A | 0.0421 | A:0;G:0;C:2113;T:93;total:2206 | iSNV |
| F30 | F30-16 | 7528 | NS4B | 0.0547 | A:0;G:0;C:2330;T:135;total:2465 | iSNV |
| F30 | F30-16 | 9293 | NS5 | 0.0321 | A:112;G:3369;C:0;T:2;total:3483 | iSNV |
| F30 | F30-16 | 9359 | NS5 | 0.2344 | A:0;G:0;C:906;T:2958;total:3864 | iSNV |
| F30 | F30-16 | 9818 | NS5 | 0.0932 | A:0;G:0;C:265;T:2578;total:2843 | iSNV |
| F30 | F30-16 | 10259 | NS5 | 0.211 | A:3065;G:820;C:0;T:0;total:3885 | iSNV |
| F30 | F30-16 | 10428 | 3'-UTR | 0.068 | A:1;G:0;C:3041;T:222;total:3264 | iSNV |
| F30 | F30-16 | 10566 | 3'-UTR | 0.2315 | A:1;G:0;C:2193;T:661;total:2855 | iSNV |
| F30 | F30-16 | 10782 | 3'-UTR | 0.03 | A:0;G:0;C:2868;T:89;total:2957 | iSNV |
| F30 | F30-17 | 783 | M | 0.0268 | A:49;G:0;C:0;T:1777;total:1826 | iSNV |
| F30 | F30-17 | 998 | E | 0.1259 | A:0;G:0;C:1367;T:197;total:1564 | iSNV |
| F30 | F30-17 | 1218 | E | 0.9953 | A:0;G:0;C:8;T:1681;total:1689 | SNP |
| F30 | F30-17 | 1430 | E | 0.0342 | A:0;G:0;C:1890;T:67;total:1957 | iSNV |
| F30 | F30-17 | 2376 | E | 0.0414 | A:0;G:0;C:1064;T:46;total:1110 | iSNV |
| F30 | F30-17 | 3317 | NS1 | 0.0658 | A:0;G:0;C:1716;T:121;total:1837 | iSNV |
| F30 | F30-17 | 3869 | NS2A | 0.8719 | A:0;G:0;C:218;T:1483;total:1701 | iSNV |
| F30 | F30-17 | 4319 | NS2B | 0.0232 | A:32;G:1345;C:0;T:0;total:1377 | iSNV |
| F30 | F30-17 | 4697 | NS3 | 0.1022 | A:0;G:0;C:202;T:1773;total:1975 | iSNV |
| F30 | F30-17 | 4783 | NS3 | 0.0303 | A:0;G:0;C:59;T:1882;total:1941 | iSNV |
| F30 | F30-17 | 5546 | NS3 | 0.0226 | A:0;G:0;C:1945;T:45;total:1990 | iSNV |
| F30 | F30-17 | 5952 | NS3 | 0.0951 | A:0;G:0;C:1702;T:179;total:1881 | iSNV |
| F30 | F30-17 | 6322 | NS3 | 0.0746 | A:0;G:0;C:1451;T:117;total:1568 | iSNV |
| F30 | F30-17 | 6401 | NS3 | 0.036 | A:0;G:0;C:51;T:1362;total:1413 | iSNV |
| F30 | F30-17 | 7109 | NS4A | 0.0447 | A:0;G:0;C:1581;T:74;total:1655 | iSNV |
| F30 | F30-17 | 7151 | NS4A | 0.0431 | A:0;G:0;C:1687;T:76;total:1763 | iSNV |
| F30 | F30-17 | 9359 | NS5 | 0.0993 | A:0;G:0;C:228;T:2067;total:2295 | iSNV |
| F30 | F30-17 | 10259 | NS5 | 0.0971 | A:2026;G:218;C:0;T:0;total:2244 | iSNV |
| F30 | F30-17 | 10428 | 3'-UTR | 0.0624 | A:0;G:0;C:1786;T:119;total:1905 | iSNV |
| F30 | F30-17 | 10566 | 3'-UTR | 0.0919 | A:2;G:1;C:1607;T:163;total:1773 | iSNV |
| F30 | F30-18 | 945 | M | 0.024 | A:3003;G:0;C:0;T:74;total:3077 | iSNV |
| F30 | F30-18 | 998 | E | 0.1806 | A:0;G:0;C:2426;T:535;total:2961 | iSNV |
| F30 | F30-18 | 1218 | E | 0.996 | A:0;G:0;C:13;T:3180;total:3193 | SNP |
| F30 | F30-18 | 1413 | E | 0.2097 | A:3055;G:811;C:0;T:0;total:3866 | iSNV |
| F30 | F30-18 | 1430 | E | 0.0301 | A:2;G:0;C:3699;T:115;total:3816 | iSNV |
| F30 | F30-18 | 3869 | NS2A | 0.8386 | A:0;G:0;C:512;T:2660;total:3172 | iSNV |
| F30 | F30-18 | 3932 | NS2A | 0.0244 | A:0;G:0;C:88;T:3515;total:3603 | iSNV |
| F30 | F30-18 | 4697 | NS3 | 0.1605 | A:2;G:0;C:611;T:3192;total:3805 | iSNV |
| F30 | F30-18 | 5952 | NS3 | 0.1568 | A:0;G:0;C:3102;T:577;total:3679 | iSNV |
| F30 | F30-18 | 6157 | NS3 | 0.0245 | A:0;G:0;C:2465;T:62;total:2527 | iSNV |
| F30 | F30-18 | 7903 | NS5 | 0.0277 | A:3853;G:110;C:0;T:0;total:3963 | iSNV |
| F30 | F30-18 | 8756 | NS5 | 0.0258 | A:2600;G:69;C:0;T:0;total:2669 | iSNV |
| F30 | F30-18 | 8791 | NS5 | 0.0209 | A:3;G:3;C:2753;T:59;total:2818 | iSNV |
| F30 | F30-18 | 9083 | NS5 | 0.0221 | A:3351;G:76;C:0;T:0;total:3427 | iSNV |
| F30 | F30-18 | 9359 | NS5 | 0.1637 | A:0;G:0;C:723;T:3693;total:4416 | iSNV |
| F30 | F30-18 | 10133 | NS5 | 0.0296 | A:0;G:0;C:3211;T:98;total:3309 | iSNV |
| F30 | F30-18 | 10259 | NS5 | 0.156 | A:3719;G:688;C:0;T:2;total:4409 | iSNV |
| F30 | F30-18 | 10376 | NS5 | 0.1982 | A:770;G:3113;C:0;T:1;total:3884 | iSNV |
| F30 | F30-18 | 10428 | 3'-UTR | 0.0343 | A:1;G:0;C:3570;T:127;total:3698 | iSNV |
| F30 | F30-18 | 10447 | 3'-UTR | 0.022 | A:0;G:0;C:3198;T:72;total:3270 | iSNV |
| F30 | F30-18 | 10452 | 3'-UTR | 0.0349 | A:3122;G:113;C:0;T:0;total:3235 | iSNV |
| F30 | F30-18 | 10559 | 3'-UTR | 0.0262 | A:79;G:2936;C:0;T:0;total:3015 | iSNV |
| F30 | F30-18 | 10566 | 3'-UTR | 0.1476 | A:0;G:0;C:2598;T:450;total:3048 | iSNV |
| F30 | F30-19 | 869 | M | 0.2685 | A:818;G:0;C:0;T:2228;total:3046 | iSNV |
| F30 | F30-19 | 998 | E | 0.0796 | A:0;G:0;C:2414;T:209;total:2623 | iSNV |
| F30 | F30-19 | 1218 | E | 0.987 | A:0;G:0;C:37;T:2792;total:2829 | SNP |
| F30 | F30-19 | 1430 | E | 0.3022 | A:0;G:0;C:2359;T:1022;total:3381 | iSNV |
| F30 | F30-19 | 1459 | E | 0.0765 | A:260;G:0;C:3134;T:1;total:3395 | iSNV |
| F30 | F30-19 | 3646 | NS1 | 0.0624 | A:0;G:0;C:126;T:1892;total:2018 | iSNV |
| F30 | F30-19 | 3869 | NS2A | 0.9233 | A:0;G:1;C:230;T:2767;total:2998 | iSNV |
| F30 | F30-19 | 4697 | NS3 | 0.0497 | A:0;G:0;C:171;T:3264;total:3435 | iSNV |
| F30 | F30-19 | 5813 | NS3 | 0.0387 | A:0;G:0;C:3375;T:136;total:3511 | iSNV |
| F30 | F30-19 | 5927 | NS3 | 0.0437 | A:1;G:0;C:3102;T:142;total:3245 | iSNV |
| F30 | F30-19 | 5952 | NS3 | 0.0478 | A:2;G:0;C:3382;T:170;total:3554 | iSNV |
| F30 | F30-19 | 6413 | NS3 | 0.0427 | A:0;G:0;C:108;T:2418;total:2526 | iSNV |
| F30 | F30-19 | 8897 | NS5 | 0.0571 | A:0;G:0;C:2475;T:150;total:2625 | iSNV |
| F30 | F30-19 | 9359 | NS5 | 0.0569 | A:0;G:0;C:226;T:3739;total:3965 | iSNV |
| F30 | F30-19 | 9452 | NS5 | 0.0569 | A:233;G:3859;C:0;T:0;total:4092 | iSNV |
| F30 | F30-19 | 9818 | NS5 | 0.0541 | A:0;G:0;C:154;T:2689;total:2843 | iSNV |
| F30 | F30-19 | 10022 | NS5 | 0.066 | A:0;G:0;C:193;T:2727;total:2920 | iSNV |
| F30 | F30-19 | 10181 | NS5 | 0.0242 | A:0;G:0;C:3223;T:80;total:3303 | iSNV |
| F30 | F30-19 | 10259 | NS5 | 0.0665 | A:3688;G:263;C:0;T:0;total:3951 | iSNV |
| F30 | F30-19 | 10358 | NS5 | 0.3079 | A:0;G:0;C:2292;T:1020;total:3312 | iSNV |
| F30 | F30-19 | 10428 | 3'-UTR | 0.0375 | A:1;G:0;C:3079;T:120;total:3200 | iSNV |
| F30 | F30-19 | 10447 | 3'-UTR | 0.0237 | A:1;G:0;C:2871;T:70;total:2942 | iSNV |
| F30 | F30-19 | 10566 | 3'-UTR | 0.0546 | A:0;G:0;C:2679;T:155;total:2834 | iSNV |
| F30 | F30-2 | 568 | M | 0.0218 | A:2636;G:0;C:0;T:59;total:2695 | iSNV |
| F30 | F30-2 | 998 | E | 0.0553 | A:0;G:0;C:2202;T:129;total:2331 | iSNV |
| F30 | F30-2 | 1218 | E | 0.9921 | A:0;G:0;C:22;T:2733;total:2755 | SNP |
| F30 | F30-2 | 1413 | E | 0.3833 | A:1946;G:1210;C:0;T:0;total:3156 | iSNV |
| F30 | F30-2 | 1430 | E | 0.0405 | A:0;G:0;C:2864;T:121;total:2985 | iSNV |
| F30 | F30-2 | 2274 | E | 0.0344 | A:0;G:2077;C:0;T:74;total:2151 | iSNV |

| | | | | | | |
|---|---|---|---|---|---|---|
| F30 | F30-2 | 3693 | NS1 | 0.0288 | A:57;G:1;C:0;T:1916;total:1974 | iSNV |
| F30 | F30-2 | 3869 | NS2A | 0.9393 | A:0;G:0;C:206;T:3186;total:3392 | iSNV |
| F30 | F30-2 | 4697 | NS3 | 0.0602 | A:0;G:0;C:194;T:3025;total:3219 | iSNV |
| F30 | F30-2 | 5952 | NS3 | 0.0611 | A:0;G:0;C:3503;T:228;total:3731 | iSNV |
| F30 | F30-2 | 9350 | NS5 | 0.0459 | A:0;G:0;C:3695;T:178;total:3873 | iSNV |
| F30 | F30-2 | 9359 | NS5 | 0.0713 | A:1;G:0;C:279;T:3629;total:3909 | iSNV |
| F30 | F30-2 | 9818 | NS5 | 0.024 | A:0;G:0;C:55;T:2235;total:2290 | iSNV |
| F30 | F30-2 | 10259 | NS5 | 0.0595 | A:3191;G:202;C:0;T:0;total:3393 | iSNV |
| F30 | F30-2 | 10376 | NS5 | 0.3761 | A:1180;G:1957;C:0;T:0;total:3137 | iSNV |
| F30 | F30-2 | 10428 | 3'-UTR | 0.0347 | A:0;G:1;C:2836;T:102;total:2939 | iSNV |
| F30 | F30-2 | 10566 | 3'-UTR | 0.0621 | A:0;G:0;C:2220;T:147;total:2367 | iSNV |
| F30 | F30-2 | 10841 | 3'-UTR | 0.0205 | A:0;G:1286;C:0;T:27;total:1313 | iSNV |
| F30 | F30-20 | 998 | E | 0.2071 | A:0;G:0;C:1462;T:382;total:1844 | iSNV |
| F30 | F30-20 | 1117 | E | 0.1221 | A:1840;G:256;C:0;T:0;total:2096 | iSNV |
| F30 | F30-20 | 1218 | E | 0.999 | A:0;G:0;C:2;T:1970;total:1972 | SNP |
| F30 | F30-20 | 1721 | E | 0.076 | A:1848;G:152;C:0;T:0;total:2000 | iSNV |
| F30 | F30-20 | 2009 | E | 0.0286 | A:0;G:0;C:1662;T:49;total:1711 | iSNV |
| F30 | F30-20 | 2230 | E | 0.097 | A:0;G:0;C:1721;T:185;total:1906 | iSNV |
| F30 | F30-20 | 2357 | E | 0.0222 | A:1229;G:28;C:0;T:0;total:1257 | iSNV |
| F30 | F30-20 | 3869 | NS2A | 0.7644 | A:0;G:0;C:488;T:1583;total:2071 | iSNV |
| F30 | F30-20 | 4403 | NS2B | 0.0211 | A:37;G:0;C:2;T:1710;total:1749 | iSNV |
| F30 | F30-20 | 4697 | NS3 | 0.2282 | A:0;G:2;C:556;T:1878;total:2436 | iSNV |
| F30 | F30-20 | 5952 | NS3 | 0.2015 | A:0;G:0;C:1862;T:470;total:2332 | iSNV |
| F30 | F30-20 | 6151 | NS3 | 0.0225 | A:1606;G:37;C:0;T:0;total:1643 | iSNV |
| F30 | F30-20 | 6560 | NS4A | 0.0356 | A:0;G:1679;C:0;T:62;total:1741 | iSNV |
| F30 | F30-20 | 6786 | NS4A | 0.0865 | A:1;G:0;C:1614;T:153;total:1768 | iSNV |
| F30 | F30-20 | 7629 | NS4B | 0.0315 | A:1994;G:0;C:65;T:0;total:2059 | iSNV |
| F30 | F30-20 | 9359 | NS5 | 0.2314 | A:0;G:0;C:651;T:2162;total:2813 | iSNV |
| F30 | F30-20 | 10259 | NS5 | 0.24 | A:2184;G:690;C:0;T:0;total:2874 | iSNV |
| F30 | F30-20 | 10428 | 3'-UTR | 0.0375 | A:0;G:0;C:2308;T:90;total:2398 | iSNV |
| F30 | F30-20 | 10566 | 3'-UTR | 0.2238 | A:0;G:1;C:1549;T:447;total:1997 | iSNV |
| F30 | F30-20 | 10617 | 3'-UTR | 0.0266 | A:2006;G:55;C:0;T:1;total:2062 | iSNV |
| F30 | F30-21 | 897 | M | 0.0328 | A:1;G:0;C:4673;T:159;total:4833 | iSNV |
| F30 | F30-21 | 998 | E | 0.0548 | A:5;G:0;C:4733;T:275;total:5013 | iSNV |
| F30 | F30-21 | 1218 | E | 0.9962 | A:0;G:1;C:21;T:5727;total:5749 | SNP |
| F30 | F30-21 | 3300 | NS1 | 0.0261 | A:0;G:0;C:166;T:6185;total:6351 | iSNV |
| F30 | F30-21 | 3661 | NS1 | 0.1092 | A:0;G:0;C:506;T:4126;total:4632 | iSNV |
| F30 | F30-21 | 3869 | NS2A | 0.9465 | A:0;G:1;C:326;T:5766;total:6093 | iSNV |
| F30 | F30-21 | 4093 | NS2A | 0.109 | A:4353;G:533;C:0;T:0;total:4886 | iSNV |
| F30 | F30-21 | 4697 | NS3 | 0.053 | A:0;G:0;C:361;T:6450;total:6811 | iSNV |
| F30 | F30-21 | 5952 | NS3 | 0.0497 | A:1;G:0;C:6571;T:344;total:6916 | iSNV |
| F30 | F30-21 | 5981 | NS3 | 0.0222 | A:0;G:6163;C:140;T:0;total:6303 | iSNV |
| F30 | F30-21 | 6004 | NS3 | 0.0986 | A:0;G:0;C:5580;T:611;total:6191 | iSNV |
| F30 | F30-21 | 6206 | NS3 | 0.1064 | A:0;G:0;C:4945;T:589;total:5534 | iSNV |
| F30 | F30-21 | 6950 | NS4A | 0.0327 | A:1;G:0;C:5283;T:179;total:5463 | iSNV |
| F30 | F30-21 | 7625 | NS4B | 0.0274 | A:0;G:0;C:6028;T:170;total:6198 | iSNV |
| F30 | F30-21 | 7697 | NS5 | 0.0361 | A:0;G:5726;C:2;T:215;total:5943 | iSNV |
| F30 | F30-21 | 8194 | NS5 | 0.0882 | A:5847;G:566;C:0;T:2;total:6415 | iSNV |
| F30 | F30-21 | 9065 | NS5 | 0.0974 | A:523;G:4843;C:0;T:2;total:5368 | iSNV |
| F30 | F30-21 | 9359 | NS5 | 0.0476 | A:0;G:0;C:366;T:7320;total:7686 | iSNV |
| F30 | F30-21 | 10259 | NS5 | 0.0528 | A:6756;G:377;C:0;T:0;total:7133 | iSNV |
| F30 | F30-21 | 10358 | NS5 | 0.0306 | A:0;G:1;C:6049;T:191;total:6241 | iSNV |
| F30 | F30-21 | 10428 | 3'-UTR | 0.0355 | A:0;G:0;C:5722;T:211;total:5933 | iSNV |
| F30 | F30-21 | 10566 | 3'-UTR | 0.0486 | A:0;G:0;C:5127;T:262;total:5389 | iSNV |
| F30 | F30-21 | 10578 | 3'-UTR | 0.1182 | A:0;G:0;C:629;T:4691;total:5320 | iSNV |
| F30 | F30-21 | 10766 | 3'-UTR | 0.038 | A:237;G:5990;C:0;T:5;total:6232 | iSNV |
| F30 | F30-22 | 897 | M | 0.0243 | A:0;G:0;C:4169;T:104;total:4273 | iSNV |
| F30 | F30-22 | 998 | E | 0.1043 | A:0;G:0;C:3976;T:463;total:4439 | iSNV |
| F30 | F30-22 | 1218 | E | 0.9858 | A:0;G:0;C:70;T:4842;total:4912 | SNP |
| F30 | F30-22 | 1397 | E | 0.0418 | A:1;G:1;C:5967;T:261;total:6230 | iSNV |
| F30 | F30-22 | 1461 | E | 0.1083 | A:0;G:0;C:604;T:4971;total:5575 | iSNV |
| F30 | F30-22 | 1712 | E | 0.0213 | A:0;G:4534;C:99;T:0;total:4633 | iSNV |
| F30 | F30-22 | 3137 | NS1 | 0.0367 | A:0;G:0;C:5711;T:218;total:5929 | iSNV |
| F30 | F30-22 | 3671 | NS1 | 0.0311 | A:9;G:0;C:118;T:3657;total:3784 | iSNV |
| F30 | F30-22 | 3869 | NS2A | 0.895 | A:0;G:0;C:557;T:4746;total:5303 | iSNV |
| F30 | F30-22 | 4319 | NS2B | 0.0273 | A:122;G:4336;C:0;T:0;total:4458 | iSNV |
| F30 | F30-22 | 4697 | NS3 | 0.0835 | A:0;G:1;C:482;T:5287;total:5770 | iSNV |
| F30 | F30-22 | 4791 | NS3 | 0.0367 | A:1;G:5874;C:224;T:1;total:6100 | iSNV |
| F30 | F30-22 | 5654 | NS3 | 0.0394 | A:0;G:0;C:4988;T:205;total:5193 | iSNV |
| F30 | F30-22 | 5952 | NS3 | 0.0871 | A:0;G:0;C:5362;T:512;total:5874 | iSNV |
| F30 | F30-22 | 6157 | NS3 | 0.0362 | A:0;G:0;C:3799;T:143;total:3942 | iSNV |
| F30 | F30-22 | 6938 | NS4A | 0.0238 | A:2;G:0;C:4217;T:103;total:4322 | iSNV |
| F30 | F30-22 | 7784 | NS5 | 0.1141 | A:0;G:0;C:4252;T:548;total:4800 | iSNV |
| F30 | F30-22 | 8744 | NS5 | 0.0249 | A:0;G:0;C:107;T:4184;total:4291 | iSNV |
| F30 | F30-22 | 9359 | NS5 | 0.0917 | A:1;G:0;C:584;T:5783;total:6368 | iSNV |
| F30 | F30-22 | 10046 | NS5 | 0.0254 | A:4441;G:116;C:0;T:0;total:4557 | iSNV |
| F30 | F30-22 | 10259 | NS5 | 0.0981 | A:5735;G:624;C:0;T:0;total:6359 | iSNV |
| F30 | F30-22 | 10428 | 3'-UTR | 0.0446 | A:3;G:0;C:5049;T:236;total:5288 | iSNV |
| F30 | F30-22 | 10447 | 3'-UTR | 0.0548 | A:2;G:0;C:4427;T:257;total:4686 | iSNV |
| F30 | F30-22 | 10566 | 3'-UTR | 0.077 | A:0;G:0;C:4203;T:351;total:4554 | iSNV |
| F30 | F30-23 | 353 | C | 0.0233 | A:3890;G:93;C:0;T:0;total:3983 | iSNV |
| F30 | F30-23 | 645 | M | 0.0255 | A:3427;G:90;C:0;T:0;total:3517 | iSNV |

| | | | | | | |
|---|---|---|---|---|---|---|
| F30 | F30-23 | 752 | M | 0.0406 | A:3801;G:161;C:0;T:0;total:3962 | iSNV |
| F30 | F30-23 | 854 | M | 0.0203 | A:0;G:0;C:3173;T:66;total:3239 | iSNV |
| F30 | F30-23 | 869 | M | 0.0253 | A:86;G:0;C:0;T:3303;total:3389 | iSNV |
| F30 | F30-23 | 998 | E | 0.2077 | A:0;G:0;C:2300;T:603;total:2903 | iSNV |
| F30 | F30-23 | 1007 | E | 0.0386 | A:0;G:0;C:3006;T:121;total:3127 | iSNV |
| F30 | F30-23 | 1117 | E | 0.038 | A:3057;G:121;C:0;T:0;total:3178 | iSNV |
| F30 | F30-23 | 1218 | E | 0.9608 | A:0;G:0;C:129;T:3154;total:3283 | iSNV |
| F30 | F30-23 | 1416 | E | 0.0424 | A:3742;G:166;C:0;T:0;total:3908 | iSNV |
| F30 | F30-23 | 1430 | E | 0.0541 | A:0;G:0;C:3719;T:213;total:3932 | iSNV |
| F30 | F30-23 | 2664 | NS1 | 0.0247 | A:0;G:0;C:3467;T:88;total:3555 | iSNV |
| F30 | F30-23 | 3869 | NS2A | 0.783 | A:0;G:0;C:788;T:2842;total:3630 | iSNV |
| F30 | F30-23 | 4697 | NS3 | 0.173 | A:1;G:0;C:685;T:3272;total:3958 | iSNV |
| F30 | F30-23 | 5102 | NS3 | 0.0405 | A:0;G:0;C:3364;T:142;total:3506 | iSNV |
| F30 | F30-23 | 5952 | NS3 | 0.1753 | A:0;G:0;C:3230;T:687;total:3917 | iSNV |
| F30 | F30-23 | 6151 | NS3 | 0.0227 | A:2444;G:0;C:0;T:57;total:2501 | iSNV |
| F30 | F30-23 | 7460 | NS4B | 0.0695 | A:150;G:2006;C:0;T:0;total:2156 | iSNV |
| F30 | F30-23 | 8495 | NS5 | 0.0217 | A:0;G:1;C:100;T:4502;total:4603 | iSNV |
| F30 | F30-23 | 9359 | NS5 | 0.1925 | A:0;G:0;C:844;T:3540;total:4384 | iSNV |
| F30 | F30-23 | 10259 | NS5 | 0.2075 | A:3268;G:856;C:0;T:0;total:4124 | iSNV |
| F30 | F30-23 | 10428 | 3'-UTR | 0.057 | A:0;G:0;C:3258;T:197;total:3455 | iSNV |
| F30 | F30-23 | 10447 | 3'-UTR | 0.0509 | A:0;G:0;C:2927;T:157;total:3084 | iSNV |
| F30 | F30-23 | 10566 | 3'-UTR | 0.1678 | A:0;G:0;C:2509;T:506;total:3015 | iSNV |
| F30 | F30-23 | 10617 | 3'-UTR | 0.0216 | A:2943;G:65;C:0;T:0;total:3008 | iSNV |
| F30 | F30-24 | 353 | C | 0.0306 | A:5882;G:186;C:0;T:0;total:6068 | iSNV |
| F30 | F30-24 | 645 | M | 0.0224 | A:5175;G:119;C:0;T:0;total:5294 | iSNV |
| F30 | F30-24 | 919 | M | 0.0248 | A:4561;G:116;C:0;T:0;total:4677 | iSNV |
| F30 | F30-24 | 998 | E | 0.2523 | A:0;G:0;C:3371;T:1138;total:4509 | iSNV |
| F30 | F30-24 | 1013 | E | 0.0369 | A:4533;G:0;C:174;T:0;total:4707 | iSNV |
| F30 | F30-24 | 1117 | E | 0.027 | A:4885;G:136;C:0;T:0;total:5021 | iSNV |
| F30 | F30-24 | 1218 | E | 0.9635 | A:0;G:0;C:185;T:4872;total:5057 | iSNV |
| F30 | F30-24 | 1413 | E | 0.1626 | A:5121;G:995;C:1;T:0;total:6117 | iSNV |
| F30 | F30-24 | 1430 | E | 0.0225 | A:1;G:0;C:6037;T:139;total:6177 | iSNV |
| F30 | F30-24 | 1892 | E | 0.0303 | A:4593;G:144;C:0;T:0;total:4737 | iSNV |
| F30 | F30-24 | 3110 | NS1 | 0.0639 | A:5530;G:378;C:0;T:0;total:5908 | iSNV |
| F30 | F30-24 | 3257 | NS1 | 0.034 | A:0;G:0;C:208;T:5892;total:6100 | iSNV |
| F30 | F30-24 | 3869 | NS2A | 0.7381 | A:0;G:0;C:1394;T:3927;total:5321 | iSNV |
| F30 | F30-24 | 4073 | NS2A | 0.0217 | A:0;G:0;C:4316;T:96;total:4412 | iSNV |
| F30 | F30-24 | 4697 | NS3 | 0.2202 | A:0;G:1;C:1364;T:4829;total:6194 | iSNV |
| F30 | F30-24 | 5465 | NS3 | 0.0706 | A:5998;G:456;C:0;T:0;total:6454 | iSNV |
| F30 | F30-24 | 5952 | NS3 | 0.2155 | A:1;G:0;C:4683;T:1287;total:5971 | iSNV |
| F30 | F30-24 | 9359 | NS5 | 0.2309 | A:0;G:1;C:1573;T:5237;total:6811 | iSNV |
| F30 | F30-24 | 10259 | NS5 | 0.2457 | A:4996;G:1628;C:0;T:0;total:6624 | iSNV |
| F30 | F30-24 | 10376 | NS5 | 0.0373 | A:217;G:5585;C:0;T:2;total:5804 | iSNV |
| F30 | F30-24 | 10428 | 3'-UTR | 0.0425 | A:1;G:3;C:5281;T:235;total:5520 | iSNV |
| F30 | F30-24 | 10447 | 3'-UTR | 0.0435 | A:0;G:0;C:4748;T:216;total:4964 | iSNV |
| F30 | F30-24 | 10452 | 3'-UTR | 0.0272 | A:4785;G:134;C:0;T:0;total:4919 | iSNV |
| F30 | F30-24 | 10566 | 3'-UTR | 0.2025 | A:1;G:0;C:3693;T:938;total:4632 | iSNV |
| F30 | F30-25 | 281 | C | 0.0369 | A:0;G:0;C:8423;T:323;total:8746 | iSNV |
| F30 | F30-25 | 443 | C | 0.025 | A:4;G:7757;C:0;T:199;total:7960 | iSNV |
| F30 | F30-25 | 491 | M | 0.0254 | A:1;G:0;C:231;T:8829;total:9061 | iSNV |
| F30 | F30-25 | 897 | M | 0.0424 | A:2;G:2;C:6018;T:267;total:6289 | iSNV |
| F30 | F30-25 | 998 | E | 0.0742 | A:1;G:0;C:6107;T:490;total:6598 | iSNV |
| F30 | F30-25 | 1117 | E | 0.3717 | A:4374;G:2589;C:0;T:1;total:6964 | iSNV |
| F30 | F30-25 | 1218 | E | 0.9969 | A:1;G:0;C:21;T:6926;total:6948 | SNP |
| F30 | F30-25 | 2213 | E | 0.026 | A:179;G:6690;C:0;T:1;total:6870 | iSNV |
| F30 | F30-25 | 2855 | NS1 | 0.3434 | A:3252;G:6218;C:0;T:0;total:9470 | iSNV |
| F30 | F30-25 | 3356 | NS1 | 0.0374 | A:227;G:5830;C:0;T:1;total:6058 | iSNV |
| F30 | F30-25 | 3869 | NS2A | 0.921 | A:1;G:0;C:589;T:6858;total:7448 | iSNV |
| F30 | F30-25 | 4697 | NS3 | 0.0685 | A:0;G:0;C:590;T:8017;total:8607 | iSNV |
| F30 | F30-25 | 5558 | NS3 | 0.35 | A:2864;G:5315;C:3;T:0;total:8182 | iSNV |
| F30 | F30-25 | 5952 | NS3 | 0.0747 | A:0;G:0;C:8120;T:656;total:8776 | iSNV |
| F30 | F30-25 | 7274 | NS4B | 0.0389 | A:0;G:1;C:4683;T:190;total:4874 | iSNV |
| F30 | F30-25 | 8312 | NS5 | 0.0361 | A:293;G:0;C:7804;T:0;total:8097 | iSNV |
| F30 | F30-25 | 9359 | NS5 | 0.0736 | A:0;G:0;C:700;T:8805;total:9505 | iSNV |
| F30 | F30-25 | 9818 | NS5 | 0.0745 | A:1;G:0;C:506;T:6276;total:6783 | iSNV |
| F30 | F30-25 | 10259 | NS5 | 0.0635 | A:8423;G:572;C:0;T:0;total:8995 | iSNV |
| F30 | F30-25 | 10428 | 3'-UTR | 0.0652 | A:0;G:3;C:7072;T:494;total:7569 | iSNV |
| F30 | F30-25 | 10566 | 3'-UTR | 0.0758 | A:1;G:0;C:6186;T:508;total:6695 | iSNV |
| F30 | F30-26 | 998 | E | 0.1279 | A:0;G:0;C:3318;T:487;total:3805 | iSNV |
| F30 | F30-26 | 1043 | E | 0.0399 | A:0;G:0;C:4060;T:169;total:4229 | iSNV |
| F30 | F30-26 | 1218 | E | 0.9928 | A:0;G:0;C:29;T:3957;total:3986 | SNP |
| F30 | F30-26 | 2235 | E | 0.0212 | A:0;G:0;C:79;T:3631;total:3710 | iSNV |
| F30 | F30-26 | 2474 | E | 0.0277 | A:81;G:0;C:0;T:2839;total:2920 | iSNV |
| F30 | F30-26 | 3257 | NS1 | 0.0221 | A:0;G:0;C:107;T:4729;total:4836 | iSNV |
| F30 | F30-26 | 3869 | NS2A | 0.8501 | A:0;G:0;C:625;T:3543;total:4168 | iSNV |
| F30 | F30-26 | 3993 | NS2A | 0.0202 | A:85;G:4108;C:0;T:1;total:4194 | iSNV |
| F30 | F30-26 | 4289 | NS2B | 0.0217 | A:0;G:0;C:74;T:3334;total:3408 | iSNV |
| F30 | F30-26 | 4697 | NS3 | 0.1271 | A:0;G:0;C:633;T:4346;total:4979 | iSNV |
| F30 | F30-26 | 5952 | NS3 | 0.1229 | A:0;G:0;C:4280;T:600;total:4880 | iSNV |
| F30 | F30-26 | 6080 | NS3 | 0.0496 | A:188;G:3598;C:0;T:0;total:3786 | iSNV |
| F30 | F30-26 | 7172 | NS4A | 0.0361 | A:0;G:0;C:135;T:3601;total:3736 | iSNV |
| F30 | F30-26 | 9359 | NS5 | 0.1386 | A:0;G:0;C:759;T:4716;total:5475 | iSNV |

| | | | | | | |
|---|---|---|---|---|---|---|
| F30 | F30-26 | 10259 | NS5 | 0.1333 | A:4517;G:695;C:0;T:0;total:5212 | iSNV |
| F30 | F30-26 | 10428 | 3'-UTR | 0.1 | A:0;G:0;C:3996;T:444;total:4440 | iSNV |
| F30 | F30-26 | 10429 | 3'-UTR | 0.0286 | A:4302;G:127;C:0;T:0;total:4429 | iSNV |
| F30 | F30-26 | 10566 | 3'-UTR | 0.1221 | A:0;G:0;C:3264;T:454;total:3718 | iSNV |
| F30 | F30-26 | 10663 | 3'-UTR | 0.02 | A:0;G:1;C:4145;T:85;total:4231 | iSNV |
| F30 | F30-27 | 941 | M | 0.0306 | A:185;G:0;C:0;T:5853;total:6038 | iSNV |
| F30 | F30-27 | 998 | E | 0.0452 | A:1;G:0;C:5861;T:278;total:6140 | iSNV |
| F30 | F30-27 | 1136 | E | 0.0234 | A:0;G:1;C:159;T:6632;total:6792 | iSNV |
| F30 | F30-27 | 1218 | E | 0.996 | A:2;G:0;C:25;T:6561;total:6588 | SNP |
| F30 | F30-27 | 1413 | E | 0.0527 | A:7535;G:420;C:0;T:0;total:7955 | iSNV |
| F30 | F30-27 | 2664 | NS1 | 0.4125 | A:0;G:0;C:4318;T:3032;total:7350 | iSNV |
| F30 | F30-27 | 3869 | NS2A | 0.9474 | A:0;G:0;C:402;T:7235;total:7637 | iSNV |
| F30 | F30-27 | 4697 | NS3 | 0.0432 | A:0;G:1;C:353;T:7807;total:8161 | iSNV |
| F30 | F30-27 | 5952 | NS3 | 0.0518 | A:2;G:0;C:7739;T:423;total:8164 | iSNV |
| F30 | F30-27 | 6068 | NS3 | 0.0224 | A:156;G:0;C:6772;T:6;total:6934 | iSNV |
| F30 | F30-27 | 6734 | NS4A | 0.0305 | A:0;G:0;C:180;T:5704;total:5884 | iSNV |
| F30 | F30-27 | 7626 | NS4B | 0.4285 | A:4;G:3026;C:2;T:4029;total:7061 | iSNV |
| F30 | F30-27 | 9359 | NS5 | 0.0528 | A:0;G:0;C:481;T:8628;total:9109 | iSNV |
| F30 | F30-27 | 9818 | NS5 | 0.0277 | A:0;G:0;C:176;T:6156;total:6332 | iSNV |
| F30 | F30-27 | 10259 | NS5 | 0.0405 | A:7958;G:336;C:0;T:0;total:8294 | iSNV |
| F30 | F30-27 | 10376 | NS5 | 0.0476 | A:348;G:6952;C:0;T:1;total:7301 | iSNV |
| F30 | F30-27 | 10419 | 3'-UTR | 0.0458 | A:0;G:24;C:6370;T:307;total:6701 | iSNV |
| F30 | F30-27 | 10428 | 3'-UTR | 0.0521 | A:0;G:0;C:6546;T:360;total:6906 | iSNV |
| F30 | F30-27 | 10447 | 3'-UTR | 0.0243 | A:1;G:1;C:6134;T:153;total:6289 | iSNV |
| F30 | F30-27 | 10566 | 3'-UTR | 0.0427 | A:0;G:1;C:5916;T:264;total:6181 | iSNV |
| F30 | F30-27 | 10837 | 3'-UTR | 0.0285 | A:0;G:0;C:115;T:3912;total:4027 | iSNV |
| F30 | F30-28 | 353 | C | 0.0226 | A:3973;G:92;C:0;T:0;total:4065 | iSNV |
| F30 | F30-28 | 645 | M | 0.0242 | A:3461;G:86;C:0;T:0;total:3547 | iSNV |
| F30 | F30-28 | 869 | M | 0.2042 | A:719;G:14;C:0;T:2787;total:3520 | iSNV |
| F30 | F30-28 | 897 | M | 0.0756 | A:0;G:0;C:2663;T:218;total:2881 | iSNV |
| F30 | F30-28 | 946 | M | 0.0738 | A:0;G:230;C:0;T:2885;total:3115 | iSNV |
| F30 | F30-28 | 998 | E | 0.0772 | A:0;G:0;C:2866;T:240;total:3106 | iSNV |
| F30 | F30-28 | 1218 | E | 0.9572 | A:0;G:1;C:140;T:3126;total:3267 | iSNV |
| F30 | F30-28 | 1428 | E | 0.0208 | A:3854;G:82;C:0;T:1;total:3937 | iSNV |
| F30 | F30-28 | 1430 | E | 0.0255 | A:0;G:0;C:3854;T:101;total:3955 | iSNV |
| F30 | F30-28 | 1478 | E | 0.0718 | A:0;G:0;C:240;T:3101;total:3341 | iSNV |
| F30 | F30-28 | 2213 | E | 0.0511 | A:167;G:3095;C:0;T:0;total:3262 | iSNV |
| F30 | F30-28 | 2879 | NS1 | 0.0261 | A:0;G:0;C:4573;T:123;total:4696 | iSNV |
| F30 | F30-28 | 3869 | NS2A | 0.9118 | A:0;G:0;C:323;T:3336;total:3659 | iSNV |
| F30 | F30-28 | 4427 | NS2B | 0.2102 | A:0;G:0;C:2194;T:584;total:2778 | iSNV |
| F30 | F30-28 | 4697 | NS3 | 0.0479 | A:0;G:0;C:194;T:3854;total:4048 | iSNV |
| F30 | F30-28 | 4742 | NS3 | 0.0679 | A:0;G:0;C:289;T:3966;total:4255 | iSNV |
| F30 | F30-28 | 5665 | NS3 | 0.0777 | A:0;G:0;C:3571;T:301;total:3872 | iSNV |
| F30 | F30-28 | 5952 | NS3 | 0.0504 | A:0;G:0;C:3877;T:206;total:4083 | iSNV |
| F30 | F30-28 | 7592 | NS4B | 0.0819 | A:0;G:0;C:2946;T:263;total:3209 | iSNV |
| F30 | F30-28 | 8789 | NS5 | 0.0261 | A:3015;G:81;C:0;T:0;total:3096 | iSNV |
| F30 | F30-28 | 9359 | NS5 | 0.059 | A:0;G:0;C:272;T:4331;total:4603 | iSNV |
| F30 | F30-28 | 9634 | NS5 | 0.0554 | A:0;G:0;C:191;T:3253;total:3444 | iSNV |
| F30 | F30-28 | 10259 | NS5 | 0.0758 | A:4130;G:339;C:0;T:0;total:4469 | iSNV |
| F30 | F30-28 | 10428 | 3'-UTR | 0.0754 | A:1;G:0;C:3455;T:282;total:3738 | iSNV |
| F30 | F30-28 | 10447 | 3'-UTR | 0.0284 | A:0;G:0;C:3243;T:95;total:3338 | iSNV |
| F30 | F30-28 | 10452 | 3'-UTR | 0.0472 | A:3123;G:155;C:1;T:0;total:3279 | iSNV |
| F30 | F30-28 | 10566 | 3'-UTR | 0.0477 | A:2;G:0;C:3090;T:155;total:3247 | iSNV |
| F30 | F30-29 | 998 | E | 0.0383 | A:0;G:0;C:3808;T:152;total:3960 | iSNV |
| F30 | F30-29 | 1218 | E | 0.9955 | A:0;G:0;C:20;T:4345;total:4365 | SNP |
| F30 | F30-29 | 1295 | E | 0.0247 | A:5276;G:1;C:1;T:134;total:5412 | iSNV |
| F30 | F30-29 | 1413 | E | 0.2886 | A:3881;G:1575;C:0;T:0;total:5456 | iSNV |
| F30 | F30-29 | 2362 | E | 0.2627 | A:0;G:2020;C:720;T:0;total:2740 | iSNV |
| F30 | F30-29 | 3023 | NS1 | 0.0224 | A:88;G:3829;C:1;T:0;total:3918 | iSNV |
| F30 | F30-29 | 3869 | NS2A | 0.9671 | A:0;G:0;C:160;T:4695;total:4855 | iSNV |
| F30 | F30-29 | 4697 | NS3 | 0.0296 | A:0;G:0;C:158;T:5173;total:5331 | iSNV |
| F30 | F30-29 | 5702 | NS3 | 0.1292 | A:2;G:0;C:5197;T:772;total:5971 | iSNV |
| F30 | F30-29 | 5952 | NS3 | 0.0318 | A:0;G:0;C:5419;T:178;total:5597 | iSNV |
| F30 | F30-29 | 6753 | NS4A | 0.0306 | A:1;G:3478;C:0;T:110;total:3589 | iSNV |
| F30 | F30-29 | 7481 | NS4B | 0.1527 | A:0;G:0;C:446;T:2474;total:2920 | iSNV |
| F30 | F30-29 | 7558 | NS4B | 0.0402 | A:0;G:0;C:4273;T:179;total:4452 | iSNV |
| F30 | F30-29 | 7967 | NS5 | 0.0282 | A:1;G:0;C:5643;T:164;total:5808 | iSNV |
| F30 | F30-29 | 9359 | NS5 | 0.0378 | A:0;G:0;C:242;T:6154;total:6396 | iSNV |
| F30 | F30-29 | 9634 | NS5 | 0.0326 | A:0;G:0;C:157;T:4656;total:4813 | iSNV |
| F30 | F30-29 | 10259 | NS5 | 0.0324 | A:5405;G:181;C:0;T:0;total:5586 | iSNV |
| F30 | F30-29 | 10376 | NS5 | 0.2797 | A:1428;G:3676;C:0;T:1;total:5105 | iSNV |
| F30 | F30-29 | 10428 | 3'-UTR | 0.0285 | A:0;G:0;C:4837;T:142;total:4979 | iSNV |
| F30 | F30-29 | 10447 | 3'-UTR | 0.0254 | A:0;G:0;C:4366;T:114;total:4480 | iSNV |
| F30 | F30-29 | 10566 | 3'-UTR | 0.0268 | A:0;G:0;C:4131;T:114;total:4245 | iSNV |
| F30 | F30-3 | 828 | M | 0.1914 | A:2623;G:621;C:0;T:0;total:3244 | iSNV |
| F30 | F30-3 | 869 | M | 0.0261 | A:100;G:0;C:0;T:3719;total:3819 | iSNV |
| F30 | F30-3 | 996 | E | 0.4148 | A:1425;G:2008;C:0;T:2;total:3435 | iSNV |
| F30 | F30-3 | 998 | E | 0.1018 | A:1;G:0;C:3094;T:351;total:3446 | iSNV |
| F30 | F30-3 | 1218 | E | 0.9817 | A:0;G:0;C:72;T:3858;total:3930 | SNP |
| F30 | F30-3 | 1430 | E | 0.0359 | A:0;G:0;C:4243;T:158;total:4401 | iSNV |
| F30 | F30-3 | 2465 | E | 0.4522 | A:1;G:0;C:1386;T:1145;total:2532 | iSNV |
| F30 | F30-3 | 3300 | NS1 | 0.0205 | A:0;G:0;C:93;T:4443;total:4536 | iSNV |

| | | | | | | |
|---|---|---|---|---|---|---|
| F30 | F30-3 | 3858 | NS2A | 0.3575 | A:0;G:0;C:2702;T:1504;total:4206 | iSNV |
| F30 | F30-3 | 3869 | NS2A | 0.8936 | A:0;G:1;C:463;T:3885;total:4349 | iSNV |
| F30 | F30-3 | 4697 | NS3 | 0.0902 | A:0;G:0;C:406;T:4091;total:4497 | iSNV |
| F30 | F30-3 | 5952 | NS3 | 0.0874 | A:0;G:0;C:4350;T:417;total:4767 | iSNV |
| F30 | F30-3 | 7625 | NS4B | 0.0291 | A:0;G:0;C:3860;T:116;total:3976 | iSNV |
| F30 | F30-3 | 9359 | NS5 | 0.0933 | A:0;G:0;C:493;T:4787;total:5280 | iSNV |
| F30 | F30-3 | 10259 | NS5 | 0.0945 | A:4376;G:457;C:0;T:1;total:4834 | iSNV |
| F30 | F30-3 | 10358 | NS5 | 0.023 | A:0;G:0;C:4449;T:105;total:4554 | iSNV |
| F30 | F30-3 | 10428 | 3'-UTR | 0.0332 | A:0;G:0;C:4309;T:148;total:4457 | iSNV |
| F30 | F30-3 | 10447 | 3'-UTR | 0.0226 | A:1;G:1;C:3754;T:87;total:3843 | iSNV |
| F30 | F30-3 | 10566 | 3'-UTR | 0.0906 | A:0;G:0;C:3219;T:321;total:3540 | iSNV |
| F30 | F30-30 | 1218 | E | 0.9986 | A:0;G:0;C:8;T:5452;total:5460 | SNP |
| F30 | F30-30 | 1413 | E | 0.9121 | A:563;G:5837;C:0;T:1;total:6401 | iSNV |
| F30 | F30-30 | 3869 | NS2A | 0.9813 | A:0;G:0;C:184;T:9609;total:9793 | SNP |
| F30 | F30-30 | 5580 | NS3 | 0.0234 | A:1;G:0;C:6466;T:155;total:6622 | iSNV |
| F30 | F30-30 | 8394 | NS5 | 0.2759 | A:0;G:1;C:1728;T:4533;total:6262 | iSNV |
| F30 | F30-30 | 10376 | NS5 | 0.8861 | A:4719;G:607;C:0;T:0;total:5326 | iSNV |
| F30 | F30-30 | 10428 | 3'-UTR | 0.0254 | A:0;G:0;C:4167;T:109;total:4276 | iSNV |
| F30 | F30-4 | 998 | E | 0.1218 | A:0;G:0;C:1715;T:238;total:1953 | iSNV |
| F30 | F30-4 | 1218 | E | 0.9959 | A:1;G:0;C:9;T:2428;total:2438 | SNP |
| F30 | F30-4 | 1430 | E | 0.0314 | A:0;G:0;C:2555;T:83;total:2638 | iSNV |
| F30 | F30-4 | 2367 | E | 0.2041 | A:1173;G:302;C:4;T:0;total:1479 | iSNV |
| F30 | F30-4 | 3869 | NS2A | 0.8586 | A:0;G:0;C:342;T:2076;total:2418 | iSNV |
| F30 | F30-4 | 4697 | NS3 | 0.1322 | A:1;G:0;C:339;T:2224;total:2564 | iSNV |
| F30 | F30-4 | 5952 | NS3 | 0.1276 | A:0;G:0;C:2317;T:339;total:2656 | iSNV |
| F30 | F30-4 | 6182 | NS3 | 0.0278 | A:0;G:0;C:53;T:1853;total:1906 | iSNV |
| F30 | F30-4 | 6322 | NS3 | 0.0477 | A:0;G:0;C:1874;T:94;total:1968 | iSNV |
| F30 | F30-4 | 7097 | NS4A | 0.024 | A:54;G:2194;C:0;T:0;total:2248 | iSNV |
| F30 | F30-4 | 9359 | NS5 | 0.1261 | A:0;G:0;C:402;T:2784;total:3186 | iSNV |
| F30 | F30-4 | 9369 | NS5 | 0.0288 | A:3135;G:93;C:1;T:0;total:3229 | iSNV |
| F30 | F30-4 | 10259 | NS5 | 0.1214 | A:2627;G:363;C:0;T:0;total:2990 | iSNV |
| F30 | F30-4 | 10428 | 3'-UTR | 0.0248 | A:0;G:0;C:2510;T:64;total:2574 | iSNV |
| F30 | F30-4 | 10447 | 3'-UTR | 0.027 | A:0;G:0;C:2157;T:60;total:2217 | iSNV |
| F30 | F30-4 | 10566 | 3'-UTR | 0.1398 | A:0;G:0;C:1777;T:289;total:2066 | iSNV |
| F30 | F30-5 | 294 | C | 0.0208 | A:53;G:2484;C:0;T:0;total:2537 | iSNV |
| F30 | F30-5 | 399 | C | 0.2131 | A:609;G:0;C:2244;T:4;total:2857 | iSNV |
| F30 | F30-5 | 897 | M | 0.204 | A:0;G:0;C:1147;T:294;total:1441 | iSNV |
| F30 | F30-5 | 998 | E | 0.0975 | A:0;G:0;C:1481;T:160;total:1641 | iSNV |
| F30 | F30-5 | 1218 | E | 0.9836 | A:0;G:0;C:32;T:1915;total:1947 | SNP |
| F30 | F30-5 | 1413 | E | 0.0479 | A:2202;G:111;C:0;T:0;total:2313 | iSNV |
| F30 | F30-5 | 2126 | E | 0.1402 | A:0;G:0;C:1312;T:214;total:1526 | iSNV |
| F30 | F30-5 | 2543 | NS1 | 0.0584 | A:0;G:0;C:1433;T:89;total:1522 | iSNV |
| F30 | F30-5 | 3110 | NS1 | 0.0201 | A:2290;G:47;C:0;T:0;total:2337 | iSNV |
| F30 | F30-5 | 3869 | NS2A | 0.8987 | A:0;G:0;C:211;T:1870;total:2081 | iSNV |
| F30 | F30-5 | 4697 | NS3 | 0.0699 | A:3;G:0;C:166;T:2205;total:2374 | iSNV |
| F30 | F30-5 | 4790 | NS3 | 0.037 | A:2338;G:90;C:0;T:0;total:2428 | iSNV |
| F30 | F30-5 | 5952 | NS3 | 0.066 | A:0;G:0;C:2264;T:160;total:2424 | iSNV |
| F30 | F30-5 | 6062 | NS3 | 0.023 | A:0;G:0;C:1953;T:46;total:1999 | iSNV |
| F30 | F30-5 | 6938 | NS4A | 0.0224 | A:0;G:0;C:1527;T:35;total:1562 | iSNV |
| F30 | F30-5 | 8130 | NS5 | 0.0293 | A:1;G:0;C:2480;T:75;total:2556 | iSNV |
| F30 | F30-5 | 9359 | NS5 | 0.0857 | A:2;G:0;C:238;T:2534;total:2774 | iSNV |
| F30 | F30-5 | 10259 | NS5 | 0.0842 | A:2251;G:207;C:0;T:0;total:2458 | iSNV |
| F30 | F30-5 | 10376 | NS5 | 0.0517 | A:118;G:2163;C:0;T:0;total:2281 | iSNV |
| F30 | F30-5 | 10407 | 3'-UTR | 0.0471 | A:0;G:0;C:102;T:2062;total:2164 | iSNV |
| F30 | F30-5 | 10428 | 3'-UTR | 0.0451 | A:0;G:0;C:2029;T:96;total:2125 | iSNV |
| F30 | F30-5 | 10447 | 3'-UTR | 0.0343 | A:0;G:0;C:1771;T:63;total:1834 | iSNV |
| F30 | F30-5 | 10452 | 3'-UTR | 0.0254 | A:1801;G:47;C:0;T:2;total:1850 | iSNV |
| F30 | F30-5 | 10566 | 3'-UTR | 0.0724 | A:0;G:0;C:1676;T:131;total:1807 | iSNV |
| F30 | F30-6 | 353 | C | 0.0271 | A:3262;G:91;C:2;T:1;total:3356 | iSNV |
| F30 | F30-6 | 645 | M | 0.0342 | A:2764;G:98;C:0;T:0;total:2862 | iSNV |
| F30 | F30-6 | 658 | M | 0.022 | A:2744;G:62;C:0;T:0;total:2806 | iSNV |
| F30 | F30-6 | 752 | M | 0.0252 | A:3131;G:81;C:0;T:0;total:3212 | iSNV |
| F30 | F30-6 | 998 | E | 0.0724 | A:0;G:0;C:2087;T:163;total:2250 | iSNV |
| F30 | F30-6 | 1117 | E | 0.0423 | A:2534;G:112;C:0;T:0;total:2646 | iSNV |
| F30 | F30-6 | 1218 | E | 0.9509 | A:0;G:0;C:133;T:2573;total:2706 | iSNV |
| F30 | F30-6 | 1430 | E | 0.165 | A:0;G:0;C:2479;T:490;total:2969 | iSNV |
| F30 | F30-6 | 2181 | E | 0.0219 | A:2495;G:56;C:0;T:0;total:2551 | iSNV |
| F30 | F30-6 | 2367 | E | 0.1133 | A:1353;G:173;C:0;T:0;total:1526 | iSNV |
| F30 | F30-6 | 3869 | NS2A | 0.9218 | A:0;G:0;C:238;T:2803;total:3041 | iSNV |
| F30 | F30-6 | 4697 | NS3 | 0.0297 | A:0;G:0;C:93;T:3036;total:3129 | iSNV |
| F30 | F30-6 | 4783 | NS3 | 0.1709 | A:0;G:0;C:554;T:2687;total:3241 | iSNV |
| F30 | F30-6 | 5102 | NS3 | 0.0261 | A:0;G:0;C:2611;T:70;total:2681 | iSNV |
| F30 | F30-6 | 5513 | NS3 | 0.034 | A:0;G:0;C:120;T:3403;total:3523 | iSNV |
| F30 | F30-6 | 5952 | NS3 | 0.0323 | A:0;G:0;C:3256;T:109;total:3365 | iSNV |
| F30 | F30-6 | 7190 | NS4A | 0.0326 | A:0;G:2102;C:0;T:71;total:2173 | iSNV |
| F30 | F30-6 | 8432 | NS5 | 0.0205 | A:3290;G:0;C:69;T:0;total:3359 | iSNV |
| F30 | F30-6 | 9359 | NS5 | 0.0373 | A:0;G:0;C:151;T:3896;total:4047 | iSNV |
| F30 | F30-6 | 9491 | NS5 | 0.0553 | A:2920;G:171;C:0;T:0;total:3091 | iSNV |
| F30 | F30-6 | 9821 | NS5 | 0.0209 | A:0;G:0;C:53;T:2478;total:2531 | iSNV |
| F30 | F30-6 | 10071 | NS5 | 0.021 | A:49;G:0;C:0;T:2283;total:2332 | iSNV |
| F30 | F30-6 | 10259 | NS5 | 0.068 | A:3272;G:239;C:0;T:0;total:3511 | iSNV |
| F30 | F30-6 | 10428 | 3'-UTR | 0.0712 | A:0;G:0;C:2789;T:214;total:3003 | iSNV |

| | | | | | | |
|---|---|---|---|---|---|---|
| F30 | F30-6 | 10447 | 3'-UTR | 0.0524 | A:3;G:0;C:2456;T:136;total:2595 | iSNV |
| F30 | F30-6 | 10566 | 3'-UTR | 0.0395 | A:2;G:0;C:2279;T:94;total:2375 | iSNV |
| F30 | F30-6 | 10577 | 3'-UTR | 0.0361 | A:0;G:0;C:2317;T:87;total:2404 | iSNV |
| F30 | F30-7 | 998 | E | 0.0831 | A:0;G:0;C:3365;T:305;total:3670 | iSNV |
| F30 | F30-7 | 1218 | E | 0.9935 | A:0;G:0;C:26;T:3919;total:3945 | SNP |
| F30 | F30-7 | 1413 | E | 0.1483 | A:3905;G:680;C:0;T:0;total:4585 | iSNV |
| F30 | F30-7 | 1907 | E | 0.0364 | A:0;G:0;C:3568;T:135;total:3703 | iSNV |
| F30 | F30-7 | 3257 | NS1 | 0.124 | A:0;G:0;C:608;T:4293;total:4901 | iSNV |
| F30 | F30-7 | 3317 | NS1 | 0.0987 | A:1;G:0;C:4243;T:465;total:4709 | iSNV |
| F30 | F30-7 | 3869 | NS2A | 0.9138 | A:0;G:0;C:380;T:4025;total:4405 | iSNV |
| F30 | F30-7 | 4697 | NS3 | 0.0599 | A:0;G:0;C:274;T:4294;total:4568 | iSNV |
| F30 | F30-7 | 5952 | NS3 | 0.0666 | A:0;G:0;C:4806;T:343;total:5149 | iSNV |
| F30 | F30-7 | 6322 | NS3 | 0.1202 | A:0;G:0;C:3145;T:430;total:3575 | iSNV |
| F30 | F30-7 | 9086 | NS5 | 0.0261 | A:0;G:0;C:122;T:4548;total:4670 | iSNV |
| F30 | F30-7 | 9359 | NS5 | 0.0769 | A:0;G:2;C:460;T:5514;total:5976 | iSNV |
| F30 | F30-7 | 9370 | NS5 | 0.1074 | A:651;G:0;C:0;T:5407;total:6058 | iSNV |
| F30 | F30-7 | 9690 | NS5 | 0.1508 | A:603;G:3395;C:0;T:0;total:3998 | iSNV |
| F30 | F30-7 | 10259 | NS5 | 0.0675 | A:4997;G:362;C:0;T:0;total:5359 | iSNV |
| F30 | F30-7 | 10376 | NS5 | 0.165 | A:809;G:4092;C:0;T:0;total:4901 | iSNV |
| F30 | F30-7 | 10428 | 3'-UTR | 0.0696 | A:0;G:0;C:4208;T:315;total:4523 | iSNV |
| F30 | F30-7 | 10447 | 3'-UTR | 0.0201 | A:1;G:0;C:3945;T:81;total:4027 | iSNV |
| F30 | F30-7 | 10566 | 3'-UTR | 0.0724 | A:0;G:0;C:3548;T:277;total:3825 | iSNV |
| F30 | F30-8 | 869 | M | 0.6484 | A:966;G:0;C:0;T:524;total:1490 | iSNV |
| F30 | F30-8 | 998 | E | 0.0387 | A:0;G:0;C:1266;T:51;total:1317 | iSNV |
| F30 | F30-8 | 1218 | E | 1 | A:0;G:0;C:0;T:1479;total:1479 | SNP |
| F30 | F30-8 | 1430 | E | 0.6814 | A:0;G:0;C:558;T:1193;total:1751 | iSNV |
| F30 | F30-8 | 3131 | NS1 | 0.2174 | A:1407;G:391;C:0;T:0;total:1798 | iSNV |
| F30 | F30-8 | 3869 | NS2A | 0.9495 | A:0;G:0;C:89;T:1671;total:1760 | iSNV |
| F30 | F30-8 | 4697 | NS3 | 0.0316 | A:0;G:0;C:56;T:1711;total:1767 | iSNV |
| F30 | F30-8 | 4896 | NS3 | 0.0341 | A:64;G:1809;C:0;T:1;total:1874 | iSNV |
| F30 | F30-8 | 5952 | NS3 | 0.0269 | A:0;G:0;C:1844;T:51;total:1895 | iSNV |
| F30 | F30-8 | 7172 | NS4A | 0.0741 | A:0;G:0;C:101;T:1261;total:1362 | iSNV |
| F30 | F30-8 | 7509 | NS4B | 0.0604 | A:71;G:1103;C:0;T:0;total:1174 | iSNV |
| F30 | F30-8 | 9359 | NS5 | 0.0339 | A:0;G:0;C:76;T:2164;total:2240 | iSNV |
| F30 | F30-8 | 9818 | NS5 | 0.0209 | A:0;G:0;C:30;T:1403;total:1433 | iSNV |
| F30 | F30-8 | 10259 | NS5 | 0.0314 | A:1939;G:63;C:0;T:0;total:2002 | iSNV |
| F30 | F30-8 | 10428 | 3'-UTR | 0.033 | A:0;G:1;C:1698;T:58;total:1757 | iSNV |
| F30 | F30-8 | 10447 | 3'-UTR | 0.0229 | A:0;G:0;C:1530;T:36;total:1566 | iSNV |
| F30 | F30-8 | 10566 | 3'-UTR | 0.0372 | A:1;G:0;C:1370;T:53;total:1424 | iSNV |
| F30 | F30-9 | 869 | M | 0.0254 | A:103;G:4;C:1;T:3938;total:4046 | iSNV |
| F30 | F30-9 | 900 | M | 0.0367 | A:121;G:3167;C:0;T:1;total:3289 | iSNV |
| F30 | F30-9 | 998 | E | 0.0728 | A:0;G:0;C:3296;T:259;total:3555 | iSNV |
| F30 | F30-9 | 1218 | E | 0.9985 | A:0;G:0;C:6;T:3932;total:3938 | SNP |
| F30 | F30-9 | 1413 | E | 0.0839 | A:4385;G:402;C:0;T:0;total:4787 | iSNV |
| F30 | F30-9 | 1430 | E | 0.0343 | A:0;G:0;C:4504;T:160;total:4664 | iSNV |
| F30 | F30-9 | 2258 | E | 0.0464 | A:0;G:0;C:135;T:2771;total:2906 | iSNV |
| F30 | F30-9 | 2448 | E | 0.024 | A:0;G:0;C:2596;T:64;total:2660 | iSNV |
| F30 | F30-9 | 2664 | NS1 | 0.0224 | A:0;G:0;C:4481;T:103;total:4584 | iSNV |
| F30 | F30-9 | 2942 | NS1 | 0.0247 | A:0;G:0;C:4602;T:117;total:4719 | iSNV |
| F30 | F30-9 | 3869 | NS2A | 0.9318 | A:0;G:0;C:316;T:4311;total:4627 | iSNV |
| F30 | F30-9 | 4187 | NS2A | 0.0529 | A:1895;G:106;C:0;T:2;total:2003 | iSNV |
| F30 | F30-9 | 4319 | NS2B | 0.0332 | A:125;G:3629;C:0;T:0;total:3754 | iSNV |
| F30 | F30-9 | 4697 | NS3 | 0.0639 | A:0;G:0;C:306;T:4480;total:4786 | iSNV |
| F30 | F30-9 | 5952 | NS3 | 0.0699 | A:0;G:0;C:4785;T:360;total:5145 | iSNV |
| F30 | F30-9 | 6093 | NS3 | 0.0238 | A:0;G:0;C:91;T:3730;total:3821 | iSNV |
| F30 | F30-9 | 6440 | NS3 | 0.0242 | A:0;G:0;C:3457;T:86;total:3543 | iSNV |
| F30 | F30-9 | 6938 | NS4A | 0.034 | A:1;G:0;C:3460;T:122;total:3583 | iSNV |
| F30 | F30-9 | 9359 | NS5 | 0.0721 | A:0;G:0;C:407;T:5236;total:5643 | iSNV |
| F30 | F30-9 | 9533 | NS5 | 0.0254 | A:0;G:0;C:112;T:4287;total:4399 | iSNV |
| F30 | F30-9 | 9728 | NS5 | 0.0291 | A:0;G:0;C:3234;T:97;total:3331 | iSNV |
| F30 | F30-9 | 9860 | NS5 | 0.0259 | A:0;G:0;C:104;T:3899;total:4003 | iSNV |
| F30 | F30-9 | 10259 | NS5 | 0.0726 | A:4763;G:373;C:0;T:0;total:5136 | iSNV |
| F30 | F30-9 | 10376 | NS5 | 0.0887 | A:431;G:4428;C:0;T:0;total:4859 | iSNV |
| F30 | F30-9 | 10428 | 3'-UTR | 0.0407 | A:0;G:0;C:4239;T:180;total:4419 | iSNV |
| F30 | F30-9 | 10452 | 3'-UTR | 0.0204 | A:3834;G:80;C:0;T:0;total:3914 | iSNV |
| F30 | F30-9 | 10566 | 3'-UTR | 0.0607 | A:0;G:0;C:3386;T:219;total:3605 | iSNV |
| F33 | F33-1 | 930 | M | 0.0221 | A:0;G:1;C:8225;T:186;total:8412 | iSNV |
| F33 | F33-1 | 997 | E | 0.0203 | A:1;G:8744;C:0;T:182;total:8927 | iSNV |
| F33 | F33-1 | 998 | E | 0.1753 | A:0;G:0;C:7075;T:1504;total:8579 | iSNV |
| F33 | F33-1 | 1218 | E | 0.9972 | A:0;G:0;C:25;T:8855;total:8880 | SNP |
| F33 | F33-1 | 1428 | E | 0.0224 | A:11282;G:259;C:1;T:0;total:11542 | iSNV |
| F33 | F33-1 | 2213 | E | 0.0241 | A:206;G:8326;C:0;T:1;total:8533 | iSNV |
| F33 | F33-1 | 2376 | E | 0.0234 | A:1;G:0;C:5881;T:141;total:6023 | iSNV |
| F33 | F33-1 | 3869 | NS2A | 0.8268 | A:0;G:0;C:1854;T:8846;total:10700 | iSNV |
| F33 | F33-1 | 3926 | NS2A | 0.0234 | A:1;G:0;C:11547;T:277;total:11825 | iSNV |
| F33 | F33-1 | 4226 | NS2B | 0.031 | A:0;G:0;C:156;T:4866;total:5022 | iSNV |
| F33 | F33-1 | 4559 | NS2B | 0.0499 | A:2;G:0;C:10094;T:531;total:10627 | iSNV |
| F33 | F33-1 | 4697 | NS3 | 0.164 | A:1;G:0;C:1879;T:9575;total:11455 | iSNV |
| F33 | F33-1 | 5952 | NS3 | 0.1684 | A:2;G:0;C:10085;T:2044;total:12131 | iSNV |
| F33 | F33-1 | 6753 | NS4A | 0.0213 | A:4;G:7159;C:1;T:156;total:7320 | iSNV |
| F33 | F33-1 | 7741 | NS5 | 0.0333 | A:9342;G:322;C:0;T:0;total:9664 | iSNV |
| F33 | F33-1 | 8651 | NS5 | 0.0288 | A:7875;G:234;C:0;T:1;total:8110 | iSNV |

| | | | | | | |
|---|---|---|---|---|---|---|
| F33 | F33-1 | 9359 | NS5 | 0.1805 | A:0;G:0;C:2585;T:11730;total:14315 | iSNV |
| F33 | F33-1 | 9818 | NS5 | 0.0227 | A:0;G:0;C:200;T:8586;total:8786 | iSNV |
| F33 | F33-1 | 9983 | NS5 | 0.0379 | A:0;G:0;C:8029;T:317;total:8346 | iSNV |
| F33 | F33-1 | 10259 | NS5 | 0.1803 | A:9926;G:2185;C:0;T:1;total:12112 | iSNV |
| F33 | F33-1 | 10428 | 3'-UTR | 0.097 | A:0;G:3;C:9463;T:1017;total:10483 | iSNV |
| F33 | F33-1 | 10447 | 3'-UTR | 0.0279 | A:2;G:0;C:9060;T:261;total:9323 | iSNV |
| F33 | F33-1 | 10566 | 3'-UTR | 0.1553 | A:2;G:1;C:7087;T:1304;total:8394 | iSNV |
| F33 | F33-10 | 998 | E | 0.0624 | A:3;G:0;C:7545;T:503;total:8051 | iSNV |
| F33 | F33-10 | 1218 | E | 0.993 | A:0;G:1;C:65;T:9277;total:9343 | SNP |
| F33 | F33-10 | 1430 | E | 0.0724 | A:0;G:1;C:10402;T:812;total:11215 | iSNV |
| F33 | F33-10 | 2474 | E | 0.0449 | A:303;G:1;C:2;T:6430;total:6736 | iSNV |
| F33 | F33-10 | 3317 | NS1 | 0.0701 | A:6;G:0;C:11153;T:842;total:12001 | iSNV |
| F33 | F33-10 | 3869 | NS2A | 0.9308 | A:0;G:0;C:730;T:9815;total:10545 | iSNV |
| F33 | F33-10 | 4697 | NS3 | 0.0559 | A:1;G:1;C:642;T:10827;total:11471 | iSNV |
| F33 | F33-10 | 4745 | NS3 | 0.021 | A:0;G:0;C:12130;T:261;total:12391 | iSNV |
| F33 | F33-10 | 4783 | NS3 | 0.0278 | A:0;G:1;C:314;T:10963;total:11278 | iSNV |
| F33 | F33-10 | 5952 | NS3 | 0.0586 | A:0;G:0;C:11450;T:714;total:12164 | iSNV |
| F33 | F33-10 | 6206 | NS3 | 0.0699 | A:1;G:0;C:8775;T:660;total:9436 | iSNV |
| F33 | F33-10 | 6322 | NS3 | 0.0702 | A:0;G:0;C:7690;T:581;total:8271 | iSNV |
| F33 | F33-10 | 6398 | NS3 | 0.0247 | A:0;G:0;C:221;T:8692;total:8913 | iSNV |
| F33 | F33-10 | 9359 | NS5 | 0.0627 | A:0;G:0;C:849;T:12687;total:13536 | iSNV |
| F33 | F33-10 | 10259 | NS5 | 0.0597 | A:10384;G:660;C:0;T:0;total:11044 | iSNV |
| F33 | F33-10 | 10428 | 3'-UTR | 0.0766 | A:1;G:1;C:9156;T:760;total:9918 | iSNV |
| F33 | F33-10 | 10447 | 3'-UTR | 0.0296 | A:2;G:0;C:8689;T:266;total:8957 | iSNV |
| F33 | F33-10 | 10566 | 3'-UTR | 0.053 | A:0;G:0;C:7565;T:424;total:7989 | iSNV |
| F33 | F33-11 | 998 | E | 0.077 | A:1;G:0;C:7745;T:647;total:8393 | iSNV |
| F33 | F33-11 | 1218 | E | 0.9905 | A:0;G:0;C:91;T:9390;total:9481 | SNP |
| F33 | F33-11 | 1514 | E | 0.1183 | A:7691;G:0;C:1034;T:9;total:8734 | iSNV |
| F33 | F33-11 | 1797 | E | 0.102 | A:1082;G:58;C:3;T:9462;total:10605 | iSNV |
| F33 | F33-11 | 3149 | NS1 | 0.0489 | A:0;G:0;C:605;T:11767;total:12372 | iSNV |
| F33 | F33-11 | 3869 | NS2A | 0.9266 | A:0;G:0;C:771;T:9724;total:10495 | iSNV |
| F33 | F33-11 | 4697 | NS3 | 0.0661 | A:0;G:2;C:762;T:10751;total:11515 | iSNV |
| F33 | F33-11 | 5952 | NS3 | 0.0579 | A:1;G:0;C:11356;T:698;total:12055 | iSNV |
| F33 | F33-11 | 9359 | NS5 | 0.0679 | A:1;G:2;C:901;T:12349;total:13253 | iSNV |
| F33 | F33-11 | 9880 | NS5 | 0.219 | A:2090;G:7452;C:0;T:1;total:9543 | iSNV |
| F33 | F33-11 | 10259 | NS5 | 0.0716 | A:10403;G:803;C:0;T:1;total:11207 | iSNV |
| F33 | F33-11 | 10419 | 3'-UTR | 0.0236 | A:1;G:1;C:9217;T:223;total:9442 | iSNV |
| F33 | F33-11 | 10428 | 3'-UTR | 0.0534 | A:0;G:2;C:9138;T:516;total:9656 | iSNV |
| F33 | F33-11 | 10566 | 3'-UTR | 0.0624 | A:0;G:0;C:7411;T:494;total:7905 | iSNV |
| F33 | F33-11 | 10589 | 3'-UTR | 0.2196 | A:0;G:1813;C:4;T:6437;total:8254 | iSNV |
| F33 | F33-12 | 353 | C | 0.1407 | A:7643;G:1253;C:2;T:3;total:8901 | iSNV |
| F33 | F33-12 | 645 | M | 0.1368 | A:6669;G:1058;C:0;T:2;total:7729 | iSNV |
| F33 | F33-12 | 869 | M | 0.0546 | A:54;G:412;C:0;T:7074;total:7540 | iSNV |
| F33 | F33-12 | 998 | E | 0.3161 | A:2;G:0;C:4512;T:2087;total:6601 | iSNV |
| F33 | F33-12 | 1083 | E | 0.0789 | A:6035;G:517;C:0;T:0;total:6552 | iSNV |
| F33 | F33-12 | 1117 | E | 0.2202 | A:5773;G:1631;C:0;T:0;total:7404 | iSNV |
| F33 | F33-12 | 1218 | E | 0.8458 | A:0;G:0;C:1130;T:6195;total:7325 | iSNV |
| F33 | F33-12 | 2277 | E | 0.1262 | A:1;G:0;C:740;T:5122;total:5863 | iSNV |
| F33 | F33-12 | 3869 | NS2A | 0.6814 | A:0;G:0;C:2590;T:5537;total:8127 | iSNV |
| F33 | F33-12 | 4697 | NS3 | 0.1545 | A:0;G:0;C:1407;T:7699;total:9106 | iSNV |
| F33 | F33-12 | 5558 | NS3 | 0.0691 | A:651;G:8764;C:2;T:0;total:9417 | iSNV |
| F33 | F33-12 | 5747 | NS3 | 0.0232 | A:223;G:9383;C:0;T:0;total:9606 | iSNV |
| F33 | F33-12 | 5780 | NS3 | 0.0244 | A:0;G:0;C:247;T:9867;total:10114 | iSNV |
| F33 | F33-12 | 5952 | NS3 | 0.1637 | A:2;G:0;C:7899;T:1547;total:9448 | iSNV |
| F33 | F33-12 | 7509 | NS4B | 0.0229 | A:135;G:5745;C:0;T:0;total:5880 | iSNV |
| F33 | F33-12 | 8090 | NS5 | 0.0281 | A:261;G:9014;C:0;T:2;total:9277 | iSNV |
| F33 | F33-12 | 9359 | NS5 | 0.1623 | A:0;G:0;C:1679;T:8661;total:10340 | iSNV |
| F33 | F33-12 | 9491 | NS5 | 0.0322 | A:8129;G:271;C:0;T:2;total:8402 | iSNV |
| F33 | F33-12 | 10259 | NS5 | 0.3073 | A:6160;G:2735;C:0;T:3;total:8898 | iSNV |
| F33 | F33-12 | 10428 | 3'-UTR | 0.1149 | A:0;G:0;C:6997;T:909;total:7906 | iSNV |
| F33 | F33-12 | 10447 | 3'-UTR | 0.1723 | A:0;G:0;C:5812;T:1210;total:7022 | iSNV |
| F33 | F33-12 | 10566 | 3'-UTR | 0.1553 | A:3;G:0;C:5385;T:991;total:6379 | iSNV |
| F33 | F33-13 | 395 | C | 0.8218 | A:2501;G:11529;C:0;T:2;total:14032 | iSNV |
| F33 | F33-13 | 998 | E | 0.0205 | A:1;G:0;C:9438;T:198;total:9637 | iSNV |
| F33 | F33-13 | 1218 | E | 0.9969 | A:1;G:1;C:32;T:10596;total:10630 | SNP |
| F33 | F33-13 | 1413 | E | 0.822 | A:2319;G:10701;C:0;T:1;total:13021 | iSNV |
| F33 | F33-13 | 3869 | NS2A | 0.9809 | A:0;G:1;C:224;T:11538;total:11763 | SNP |
| F33 | F33-13 | 9161 | NS5 | 0.0485 | A:0;G:0;C:706;T:13830;total:14536 | iSNV |
| F33 | F33-13 | 10259 | NS5 | 0.0206 | A:12258;G:259;C:0;T:0;total:12517 | iSNV |
| F33 | F33-13 | 10376 | NS5 | 0.823 | A:9472;G:2040;C:1;T:6;total:11519 | iSNV |
| F33 | F33-13 | 10428 | 3'-UTR | 0.0643 | A:0;G:0;C:10318;T:710;total:11028 | iSNV |
| F33 | F33-14 | 506 | M | 0.0202 | A:0;G:1;C:268;T:12996;total:13265 | iSNV |
| F33 | F33-14 | 998 | E | 0.1954 | A:0;G:0;C:7679;T:1865;total:9544 | iSNV |
| F33 | F33-14 | 1117 | E | 0.3305 | A:7062;G:3487;C:0;T:0;total:10549 | iSNV |
| F33 | F33-14 | 1218 | E | 0.9903 | A:0;G:0;C:103;T:10434;total:10537 | SNP |
| F33 | F33-14 | 2282 | E | 0.03 | A:1;G:0;C:8518;T:264;total:8783 | iSNV |
| F33 | F33-14 | 2369 | E | 0.0218 | A:157;G:7022;C:0;T:21;total:7200 | iSNV |
| F33 | F33-14 | 3869 | NS2A | 0.7953 | A:0;G:0;C:2349;T:9122;total:11471 | iSNV |
| F33 | F33-14 | 4697 | NS3 | 0.1882 | A:0;G:0;C:2488;T:10727;total:13215 | iSNV |
| F33 | F33-14 | 5558 | NS3 | 0.3081 | A:4013;G:9008;C:0;T:1;total:13022 | iSNV |
| F33 | F33-14 | 5952 | NS3 | 0.1844 | A:0;G:0;C:11005;T:2489;total:13494 | iSNV |
| F33 | F33-14 | 6401 | NS3 | 0.0266 | A:0;G:0;C:275;T:10046;total:10321 | iSNV |

| | | | | | | |
|---|---|---|---|---|---|---|
| F33 | F33-14 | 6786 | NS4A | 0.0203 | A:1;G:0;C:9997;T:208;total:10206 | iSNV |
| F33 | F33-14 | 8195 | NS5 | 0.0265 | A:9;G:1;C:11973;T:327;total:12310 | iSNV |
| F33 | F33-14 | 9359 | NS5 | 0.1939 | A:1;G:0;C:2928;T:12168;total:15097 | iSNV |
| F33 | F33-14 | 9370 | NS5 | 0.1347 | A:4;G:2077;C:0;T:13329;total:15410 | iSNV |
| F33 | F33-14 | 10259 | NS5 | 0.1873 | A:10129;G:2336;C:0;T:1;total:12466 | iSNV |
| F33 | F33-14 | 10428 | 3'-UTR | 0.059 | A:0;G:0;C:10381;T:651;total:11032 | iSNV |
| F33 | F33-14 | 10447 | 3'-UTR | 0.0271 | A:4;G:0;C:9719;T:271;total:9994 | iSNV |
| F33 | F33-14 | 10566 | 3'-UTR | 0.1727 | A:0;G:0;C:7586;T:1584;total:9170 | iSNV |
| F33 | F33-15 | 998 | E | 0.1957 | A:2;G:0;C:13876;T:3378;total:17256 | iSNV |
| F33 | F33-15 | 1117 | E | 0.3365 | A:12424;G:6303;C:1;T:2;total:18730 | iSNV |
| F33 | F33-15 | 1218 | E | 0.9895 | A:1;G:1;C:194;T:18318;total:18514 | SNP |
| F33 | F33-15 | 2282 | E | 0.0354 | A:0;G:1;C:14092;T:518;total:14611 | iSNV |
| F33 | F33-15 | 2369 | E | 0.0271 | A:342;G:12178;C:0;T:54;total:12574 | iSNV |
| F33 | F33-15 | 3869 | NS2A | 0.7913 | A:1;G:0;C:4031;T:15276;total:19308 | iSNV |
| F33 | F33-15 | 4697 | NS3 | 0.1813 | A:0;G:1;C:4077;T:18406;total:22484 | iSNV |
| F33 | F33-15 | 5558 | NS3 | 0.3328 | A:7258;G:14546;C:1;T:1;total:21806 | iSNV |
| F33 | F33-15 | 5952 | NS3 | 0.1863 | A:3;G:0;C:19903;T:4558;total:24464 | iSNV |
| F33 | F33-15 | 6401 | NS3 | 0.0237 | A:0;G:1;C:430;T:17664;total:18095 | iSNV |
| F33 | F33-15 | 8195 | NS5 | 0.0246 | A:1;G:2;C:21440;T:542;total:21985 | iSNV |
| F33 | F33-15 | 9359 | NS5 | 0.1869 | A:3;G:20;C:4974;T:21613;total:26610 | iSNV |
| F33 | F33-15 | 9370 | NS5 | 0.1296 | A:5;G:3490;C:12;T:23419;total:26926 | iSNV |
| F33 | F33-15 | 9634 | NS5 | 0.0229 | A:0;G:2;C:531;T:22560;total:23093 | iSNV |
| F33 | F33-15 | 10259 | NS5 | 0.1894 | A:18668;G:4365;C:0;T:2;total:23035 | iSNV |
| F33 | F33-15 | 10428 | 3'-UTR | 0.0609 | A:4;G:4;C:19821;T:1287;total:21116 | iSNV |
| F33 | F33-15 | 10447 | 3'-UTR | 0.026 | A:5;G:0;C:18098;T:484;total:18587 | iSNV |
| F33 | F33-15 | 10566 | 3'-UTR | 0.1775 | A:2;G:0;C:14479;T:3127;total:17608 | iSNV |
| F33 | F33-15 | 10567 | 3'-UTR | 0.0203 | A:0;G:0;C:356;T:17179;total:17535 | iSNV |
| F33 | F33-16 | 293 | C | 0.0379 | A:10990;G:433;C:0;T:0;total:11423 | iSNV |
| F33 | F33-16 | 469 | C | 0.064 | A:0;G:0;C:10895;T:745;total:11640 | iSNV |
| F33 | F33-16 | 998 | E | 0.2976 | A:1;G:0;C:6343;T:2688;total:9032 | iSNV |
| F33 | F33-16 | 1218 | E | 0.9897 | A:0;G:0;C:97;T:9305;total:9402 | SNP |
| F33 | F33-16 | 3652 | NS1 | 0.0349 | A:0;G:5;C:225;T:6215;total:6445 | iSNV |
| F33 | F33-16 | 3869 | NS2A | 0.6968 | A:1;G:0;C:2825;T:6489;total:9315 | iSNV |
| F33 | F33-16 | 3965 | NS2A | 0.0309 | A:1;G:1;C:9370;T:299;total:9671 | iSNV |
| F33 | F33-16 | 4049 | NS2A | 0.0354 | A:0;G:0;C:6717;T:247;total:6964 | iSNV |
| F33 | F33-16 | 4697 | NS3 | 0.2735 | A:0;G:0;C:2908;T:7721;total:10629 | iSNV |
| F33 | F33-16 | 4926 | NS3 | 0.0309 | A:2;G:9750;C:0;T:311;total:10063 | iSNV |
| F33 | F33-16 | 5352 | NS3 | 0.0223 | A:192;G:2;C:8379;T:1;total:8574 | iSNV |
| F33 | F33-16 | 5952 | NS3 | 0.2814 | A:0;G:0;C:8261;T:3235;total:11496 | iSNV |
| F33 | F33-16 | 6672 | NS4A | 0.031 | A:0;G:0;C:7768;T:249;total:8017 | iSNV |
| F33 | F33-16 | 7528 | NS4B | 0.1062 | A:0;G:0;C:6813;T:810;total:7623 | iSNV |
| F33 | F33-16 | 9293 | NS5 | 0.0465 | A:553;G:11329;C:2;T:2;total:11886 | iSNV |
| F33 | F33-16 | 9359 | NS5 | 0.2796 | A:1;G:0;C:3685;T:9493;total:13179 | iSNV |
| F33 | F33-16 | 9818 | NS5 | 0.0973 | A:0;G:0;C:937;T:8689;total:9626 | iSNV |
| F33 | F33-16 | 10259 | NS5 | 0.2833 | A:8644;G:3417;C:0;T:0;total:12061 | iSNV |
| F33 | F33-16 | 10428 | 3'-UTR | 0.1035 | A:0;G:2;C:9331;T:1078;total:10411 | iSNV |
| F33 | F33-16 | 10566 | 3'-UTR | 0.2671 | A:4;G:0;C:6245;T:2278;total:8527 | iSNV |
| F33 | F33-17 | 444 | C | 0.0209 | A:9078;G:0;C:0;T:194;total:9272 | iSNV |
| F33 | F33-17 | 907 | M | 0.0205 | A:0;G:1;C:149;T:7111;total:7261 | iSNV |
| F33 | F33-17 | 998 | E | 0.101 | A:1;G:1;C:6784;T:763;total:7549 | iSNV |
| F33 | F33-17 | 1218 | E | 0.993 | A:0;G:0;C:56;T:7905;total:7961 | SNP |
| F33 | F33-17 | 1430 | E | 0.0351 | A:0;G:0;C:9121;T:332;total:9453 | iSNV |
| F33 | F33-17 | 2376 | E | 0.0415 | A:2;G:0;C:4868;T:211;total:5081 | iSNV |
| F33 | F33-17 | 3317 | NS1 | 0.1357 | A:0;G:2;C:7978;T:1253;total:9233 | iSNV |
| F33 | F33-17 | 3401 | NS1 | 0.0233 | A:3;G:0;C:7767;T:186;total:7956 | iSNV |
| F33 | F33-17 | 3869 | NS2A | 0.8936 | A:0;G:2;C:882;T:7399;total:8283 | iSNV |
| F33 | F33-17 | 4697 | NS3 | 0.1037 | A:0;G:0;C:953;T:8230;total:9183 | iSNV |
| F33 | F33-17 | 4783 | NS3 | 0.029 | A:0;G:0;C:264;T:8813;total:9077 | iSNV |
| F33 | F33-17 | 5093 | NS3 | 0.0211 | A:1;G:0;C:7805;T:169;total:7975 | iSNV |
| F33 | F33-17 | 5546 | NS3 | 0.0216 | A:0;G:0;C:9413;T:208;total:9621 | iSNV |
| F33 | F33-17 | 5952 | NS3 | 0.101 | A:0;G:0;C:9034;T:1015;total:10049 | iSNV |
| F33 | F33-17 | 6322 | NS3 | 0.1558 | A:0;G:0;C:6056;T:1118;total:7174 | iSNV |
| F33 | F33-17 | 6401 | NS3 | 0.0281 | A:0;G:0;C:209;T:7203;total:7412 | iSNV |
| F33 | F33-17 | 6938 | NS4A | 0.021 | A:4;G:0;C:6778;T:146;total:6928 | iSNV |
| F33 | F33-17 | 7109 | NS4A | 0.1072 | A:1;G:0;C:6386;T:767;total:7154 | iSNV |
| F33 | F33-17 | 7151 | NS4A | 0.037 | A:0;G:0;C:7571;T:291;total:7862 | iSNV |
| F33 | F33-17 | 9359 | NS5 | 0.0992 | A:0;G:2;C:1136;T:10312;total:11450 | iSNV |
| F33 | F33-17 | 10046 | NS5 | 0.0221 | A:7619;G:173;C:1;T:1;total:7794 | iSNV |
| F33 | F33-17 | 10259 | NS5 | 0.1091 | A:8956;G:1097;C:0;T:0;total:10053 | iSNV |
| F33 | F33-17 | 10428 | 3'-UTR | 0.1016 | A:1;G:2;C:8255;T:934;total:9192 | iSNV |
| F33 | F33-17 | 10566 | 3'-UTR | 0.0945 | A:1;G:0;C:6819;T:712;total:7532 | iSNV |
| F33 | F33-18 | 998 | E | 0.0918 | A:1;G:0;C:9841;T:995;total:10837 | iSNV |
| F33 | F33-18 | 1218 | E | 0.9973 | A:0;G:0;C:32;T:11439;total:11471 | SNP |
| F33 | F33-18 | 1413 | E | 0.4371 | A:7635;G:5932;C:2;T:1;total:13570 | iSNV |
| F33 | F33-18 | 1797 | E | 0.059 | A:1;G:773;C:0;T:12320;total:13094 | iSNV |
| F33 | F33-18 | 3869 | NS2A | 0.9063 | A:0;G:0;C:1097;T:10605;total:11702 | iSNV |
| F33 | F33-18 | 4697 | NS3 | 0.0794 | A:0;G:0;C:1042;T:12071;total:13113 | iSNV |
| F33 | F33-18 | 5952 | NS3 | 0.0894 | A:4;G:0;C:13170;T:1294;total:14468 | iSNV |
| F33 | F33-18 | 7608 | NS4B | 0.0315 | A:10183;G:332;C:0;T:0;total:10515 | iSNV |
| F33 | F33-18 | 8430 | NS5 | 0.3237 | A:3;G:1;C:9994;T:4786;total:14784 | iSNV |
| F33 | F33-18 | 9359 | NS5 | 0.087 | A:0;G:1;C:1479;T:15519;total:16999 | iSNV |
| F33 | F33-18 | 9634 | NS5 | 0.0247 | A:0;G:0;C:319;T:12545;total:12864 | iSNV |

| | | | | | | |
|---|---|---|---|---|---|---|
| F33 | F33-18 | 10259 | NS5 | 0.0886 | A:13550;G:1318;C:0;T:0;total:14868 | iSNV |
| F33 | F33-18 | 10376 | NS5 | 0.4232 | A:6144;G:8370;C:0;T:1;total:14515 | iSNV |
| F33 | F33-18 | 10428 | 3'-UTR | 0.043 | A:0;G:0;C:12919;T:581;total:13500 | iSNV |
| F33 | F33-18 | 10566 | 3'-UTR | 0.0801 | A:0;G:0;C:10066;T:877;total:10943 | iSNV |
| F33 | F33-18 | 10572 | 3'-UTR | 0.0221 | A:10740;G:243;C:0;T:0;total:10983 | iSNV |
| F33 | F33-19 | 353 | C | 0.0334 | A:11832;G:409;C:0;T:1;total:12242 | iSNV |
| F33 | F33-19 | 645 | M | 0.0332 | A:10523;G:362;C:0;T:0;total:10885 | iSNV |
| F33 | F33-19 | 869 | M | 0.2125 | A:2257;G:0;C:0;T:8364;total:10621 | iSNV |
| F33 | F33-19 | 998 | E | 0.1099 | A:0;G:0;C:8513;T:1052;total:9565 | iSNV |
| F33 | F33-19 | 1117 | E | 0.0366 | A:9752;G:371;C:0;T:0;total:10123 | iSNV |
| F33 | F33-19 | 1218 | E | 0.9522 | A:0;G:0;C:471;T:9365;total:9836 | iSNV |
| F33 | F33-19 | 1430 | E | 0.2376 | A:0;G:1;C:8809;T:2746;total:11556 | iSNV |
| F33 | F33-19 | 1459 | E | 0.0406 | A:469;G:1;C:11057;T:16;total:11543 | iSNV |
| F33 | F33-19 | 3278 | NS1 | 0.0324 | A:4;G:0;C:328;T:9780;total:10112 | iSNV |
| F33 | F33-19 | 3646 | NS1 | 0.0401 | A:0;G:1;C:292;T:6976;total:7269 | iSNV |
| F33 | F33-19 | 3869 | NS2A | 0.8797 | A:0;G:0;C:1180;T:8628;total:9808 | iSNV |
| F33 | F33-19 | 4697 | NS3 | 0.0694 | A:1;G:1;C:760;T:10188;total:10950 | iSNV |
| F33 | F33-19 | 5813 | NS3 | 0.0456 | A:0;G:0;C:11152;T:533;total:11685 | iSNV |
| F33 | F33-19 | 5927 | NS3 | 0.0487 | A:0;G:0;C:10881;T:558;total:11439 | iSNV |
| F33 | F33-19 | 5952 | NS3 | 0.0727 | A:0;G:0;C:11477;T:900;total:12377 | iSNV |
| F33 | F33-19 | 6203 | NS3 | 0.0344 | A:9296;G:332;C:0;T:0;total:9628 | iSNV |
| F33 | F33-19 | 6413 | NS3 | 0.0495 | A:0;G:0;C:447;T:8575;total:9022 | iSNV |
| F33 | F33-19 | 8897 | NS5 | 0.083 | A:1;G:0;C:8367;T:758;total:9126 | iSNV |
| F33 | F33-19 | 9359 | NS5 | 0.0746 | A:1;G:2;C:1013;T:12563;total:13579 | iSNV |
| F33 | F33-19 | 9452 | NS5 | 0.0646 | A:899;G:13001;C:0;T:1;total:13901 | iSNV |
| F33 | F33-19 | 9818 | NS5 | 0.0622 | A:0;G:2;C:637;T:9592;total:10231 | iSNV |
| F33 | F33-19 | 10022 | NS5 | 0.0703 | A:0;G:0;C:727;T:9602;total:10329 | iSNV |
| F33 | F33-19 | 10181 | NS5 | 0.0263 | A:1;G:0;C:10604;T:287;total:10892 | iSNV |
| F33 | F33-19 | 10259 | NS5 | 0.1128 | A:10939;G:1391;C:0;T:0;total:12330 | iSNV |
| F33 | F33-19 | 10358 | NS5 | 0.2276 | A:0;G:1;C:9226;T:2720;total:11947 | iSNV |
| F33 | F33-19 | 10428 | 3'-UTR | 0.06 | A:1;G:0;C:10848;T:693;total:11542 | iSNV |
| F33 | F33-19 | 10447 | 3'-UTR | 0.0588 | A:1;G:1;C:9544;T:597;total:10143 | iSNV |
| F33 | F33-19 | 10566 | 3'-UTR | 0.0737 | A:1;G:0;C:9139;T:728;total:9868 | iSNV |
| F33 | F33-2 | 434 | C | 0.0285 | A:230;G:7827;C:0;T:1;total:8058 | iSNV |
| F33 | F33-2 | 568 | M | 0.0284 | A:6965;G:1;C:0;T:204;total:7170 | iSNV |
| F33 | F33-2 | 998 | E | 0.0707 | A:2;G:0;C:5906;T:450;total:6358 | iSNV |
| F33 | F33-2 | 1218 | E | 0.9869 | A:0;G:0;C:87;T:6546;total:6633 | SNP |
| F33 | F33-2 | 1413 | E | 0.454 | A:4447;G:3698;C:0;T:0;total:8145 | iSNV |
| F33 | F33-2 | 1430 | E | 0.0588 | A:0;G:0;C:7430;T:465;total:7895 | iSNV |
| F33 | F33-2 | 2274 | E | 0.0382 | A:0;G:5023;C:0;T:200;total:5223 | iSNV |
| F33 | F33-2 | 3669 | NS1 | 0.0232 | A:0;G:0;C:4490;T:107;total:4597 | iSNV |
| F33 | F33-2 | 3869 | NS2A | 0.9306 | A:0;G:0;C:498;T:6673;total:7171 | iSNV |
| F33 | F33-2 | 4697 | NS3 | 0.0618 | A:0;G:0;C:526;T:7977;total:8503 | iSNV |
| F33 | F33-2 | 5952 | NS3 | 0.0507 | A:0;G:0;C:8399;T:449;total:8848 | iSNV |
| F33 | F33-2 | 9350 | NS5 | 0.0461 | A:1;G:0;C:9686;T:469;total:10156 | iSNV |
| F33 | F33-2 | 9359 | NS5 | 0.0608 | A:1;G:0;C:617;T:9518;total:10136 | iSNV |
| F33 | F33-2 | 9818 | NS5 | 0.0224 | A:0;G:0;C:151;T:6582;total:6733 | iSNV |
| F33 | F33-2 | 10259 | NS5 | 0.0628 | A:8130;G:545;C:0;T:1;total:8676 | iSNV |
| F33 | F33-2 | 10376 | NS5 | 0.4302 | A:3425;G:4536;C:0;T:0;total:7961 | iSNV |
| F33 | F33-2 | 10428 | 3'-UTR | 0.0608 | A:0;G:0;C:7165;T:464;total:7629 | iSNV |
| F33 | F33-2 | 10447 | 3'-UTR | 0.0237 | A:4;G:3;C:6603;T:161;total:6771 | iSNV |
| F33 | F33-2 | 10566 | 3'-UTR | 0.0545 | A:3;G:1;C:5546;T:320;total:5870 | iSNV |
| F33 | F33-20 | 998 | E | 0.2752 | A:0;G:0;C:5694;T:2162;total:7856 | iSNV |
| F33 | F33-20 | 1117 | E | 0.2289 | A:6499;G:1930;C:0;T:1;total:8430 | iSNV |
| F33 | F33-20 | 1218 | E | 0.9958 | A:0;G:1;C:32;T:7790;total:7823 | SNP |
| F33 | F33-20 | 1721 | E | 0.1199 | A:6686;G:911;C:0;T:0;total:7597 | iSNV |
| F33 | F33-20 | 2230 | E | 0.1087 | A:0;G:1;C:6598;T:805;total:7404 | iSNV |
| F33 | F33-20 | 2369 | E | 0.0317 | A:162;G:4942;C:0;T:0;total:5104 | iSNV |
| F33 | F33-20 | 3869 | NS2A | 0.7194 | A:0;G:0;C:2175;T:5575;total:7750 | iSNV |
| F33 | F33-20 | 3929 | NS2A | 0.0313 | A:1;G:0;C:8298;T:269;total:8568 | iSNV |
| F33 | F33-20 | 4697 | NS3 | 0.2599 | A:1;G:0;C:2329;T:6630;total:8960 | iSNV |
| F33 | F33-20 | 5952 | NS3 | 0.2566 | A:0;G:0;C:7475;T:2581;total:10056 | iSNV |
| F33 | F33-20 | 6151 | NS3 | 0.0205 | A:6095;G:128;C:1;T:0;total:6224 | iSNV |
| F33 | F33-20 | 6560 | NS4A | 0.0511 | A:0;G:6579;C:0;T:355;total:6934 | iSNV |
| F33 | F33-20 | 6786 | NS4A | 0.113 | A:1;G:0;C:6426;T:819;total:7246 | iSNV |
| F33 | F33-20 | 7629 | NS4B | 0.047 | A:7452;G:0;C:368;T:0;total:7820 | iSNV |
| F33 | F33-20 | 9359 | NS5 | 0.2548 | A:0;G:3;C:3011;T:8801;total:11815 | iSNV |
| F33 | F33-20 | 10259 | NS5 | 0.2578 | A:7853;G:2729;C:0;T:0;total:10582 | iSNV |
| F33 | F33-20 | 10428 | 3'-UTR | 0.0521 | A:0;G:0;C:8987;T:494;total:9481 | iSNV |
| F33 | F33-20 | 10566 | 3'-UTR | 0.2589 | A:2;G:0;C:5785;T:2022;total:7809 | iSNV |
| F33 | F33-20 | 10617 | 3'-UTR | 0.0211 | A:7771;G:168;C:0;T:0;total:7939 | iSNV |
| F33 | F33-21 | 998 | E | 0.0494 | A:2;G:0;C:8012;T:417;total:8431 | iSNV |
| F33 | F33-21 | 1043 | E | 0.0223 | A:0;G:1;C:9008;T:206;total:9215 | iSNV |
| F33 | F33-21 | 1218 | E | 0.9921 | A:1;G:0;C:70;T:8828;total:8899 | SNP |
| F33 | F33-21 | 3300 | NS1 | 0.0262 | A:0;G:3;C:266;T:9849;total:10118 | iSNV |
| F33 | F33-21 | 3661 | NS1 | 0.1709 | A:0;G:0;C:1081;T:5244;total:6325 | iSNV |
| F33 | F33-21 | 3869 | NS2A | 0.9465 | A:0;G:0;C:489;T:8639;total:9128 | iSNV |
| F33 | F33-21 | 4093 | NS2A | 0.1505 | A:5907;G:1047;C:0;T:1;total:6955 | iSNV |
| F33 | F33-21 | 4697 | NS3 | 0.0461 | A:0;G:1;C:470;T:9718;total:10189 | iSNV |
| F33 | F33-21 | 5952 | NS3 | 0.0463 | A:0;G:0;C:10725;T:521;total:11246 | iSNV |
| F33 | F33-21 | 6004 | NS3 | 0.1478 | A:2;G:0;C:8738;T:1516;total:10256 | iSNV |
| F33 | F33-21 | 6206 | NS3 | 0.1067 | A:2;G:0;C:7852;T:939;total:8793 | iSNV |

| | | | | | | |
|---|---|---|---|---|---|---|
| F33 | F33-21 | 6950 | NS4A | 0.028 | A:1;G:0;C:7731;T:223;total:7955 | iSNV |
| F33 | F33-21 | 7509 | NS4B | 0.0228 | A:142;G:6063;C:0;T:1;total:6206 | iSNV |
| F33 | F33-21 | 7625 | NS4B | 0.0297 | A:0;G:0;C:8158;T:250;total:8408 | iSNV |
| F33 | F33-21 | 7697 | NS5 | 0.0303 | A:7;G:8696;C:0;T:272;total:8975 | iSNV |
| F33 | F33-21 | 8194 | NS5 | 0.1024 | A:9264;G:1058;C:0;T:0;total:10322 | iSNV |
| F33 | F33-21 | 9065 | NS5 | 0.1422 | A:1377;G:8298;C:1;T:2;total:9678 | iSNV |
| F33 | F33-21 | 9359 | NS5 | 0.0524 | A:0;G:0;C:695;T:12561;total:13256 | iSNV |
| F33 | F33-21 | 10259 | NS5 | 0.0505 | A:10821;G:576;C:0;T:0;total:11397 | iSNV |
| F33 | F33-21 | 10358 | NS5 | 0.0325 | A:1;G:1;C:10557;T:355;total:10914 | iSNV |
| F33 | F33-21 | 10428 | 3'-UTR | 0.0486 | A:3;G:0;C:10244;T:524;total:10771 | iSNV |
| F33 | F33-21 | 10447 | 3'-UTR | 0.0221 | A:5;G:0;C:9229;T:209;total:9443 | iSNV |
| F33 | F33-21 | 10566 | 3'-UTR | 0.0425 | A:0;G:1;C:8215;T:365;total:8581 | iSNV |
| F33 | F33-21 | 10578 | 3'-UTR | 0.1673 | A:0;G:0;C:1396;T:6948;total:8344 | iSNV |
| F33 | F33-22 | 353 | C | 0.0429 | A:12225;G:549;C:0;T:0;total:12774 | iSNV |
| F33 | F33-22 | 645 | M | 0.0429 | A:10480;G:470;C:0;T:0;total:10950 | iSNV |
| F33 | F33-22 | 998 | E | 0.1501 | A:0;G:0;C:8422;T:1488;total:9910 | iSNV |
| F33 | F33-22 | 1117 | E | 0.0778 | A:9897;G:836;C:0;T:0;total:10733 | iSNV |
| F33 | F33-22 | 1218 | E | 0.9426 | A:0;G:0;C:607;T:9953;total:10560 | iSNV |
| F33 | F33-22 | 1397 | E | 0.0355 | A:2;G:0;C:13428;T:495;total:13925 | iSNV |
| F33 | F33-22 | 1461 | E | 0.0275 | A:1;G:0;C:350;T:12336;total:12687 | iSNV |
| F33 | F33-22 | 3011 | NS1 | 0.0339 | A:369;G:10484;C:0;T:1;total:10854 | iSNV |
| F33 | F33-22 | 3137 | NS1 | 0.0463 | A:1;G:1;C:11273;T:548;total:11823 | iSNV |
| F33 | F33-22 | 3401 | NS1 | 0.0285 | A:0;G:0;C:10117;T:297;total:10414 | iSNV |
| F33 | F33-22 | 3671 | NS1 | 0.0339 | A:0;G:0;C:246;T:6993;total:7239 | iSNV |
| F33 | F33-22 | 3869 | NS2A | 0.8418 | A:0;G:0;C:1633;T:8689;total:10322 | iSNV |
| F33 | F33-22 | 4319 | NS2B | 0.0224 | A:218;G:9475;C:0;T:1;total:9694 | iSNV |
| F33 | F33-22 | 4697 | NS3 | 0.1039 | A:0;G:0;C:1226;T:10563;total:11789 | iSNV |
| F33 | F33-22 | 4791 | NS3 | 0.0735 | A:3;G:11403;C:905;T:0;total:12311 | iSNV |
| F33 | F33-22 | 5646 | NS3 | 0.0243 | A:10982;G:0;C:274;T:0;total:11256 | iSNV |
| F33 | F33-22 | 5654 | NS3 | 0.0477 | A:0;G:1;C:11117;T:557;total:11675 | iSNV |
| F33 | F33-22 | 5939 | NS3 | 0.0263 | A:6;G:0;C:12650;T:342;total:12998 | iSNV |
| F33 | F33-22 | 5952 | NS3 | 0.0931 | A:0;G:0;C:11876;T:1220;total:13096 | iSNV |
| F33 | F33-22 | 6157 | NS3 | 0.0402 | A:0;G:0;C:7827;T:328;total:8155 | iSNV |
| F33 | F33-22 | 6938 | NS4A | 0.0249 | A:1;G:0;C:8885;T:227;total:9113 | iSNV |
| F33 | F33-22 | 7784 | NS5 | 0.0255 | A:0;G:1;C:11072;T:290;total:11363 | iSNV |
| F33 | F33-22 | 8744 | NS5 | 0.0283 | A:0;G:0;C:272;T:9320;total:9592 | iSNV |
| F33 | F33-22 | 8954 | NS5 | 0.0239 | A:5;G:12406;C:0;T:304;total:12715 | iSNV |
| F33 | F33-22 | 9284 | NS5 | 0.0394 | A:0;G:0;C:605;T:14720;total:15325 | iSNV |
| F33 | F33-22 | 9359 | NS5 | 0.1049 | A:3;G:2;C:1662;T:14168;total:15835 | iSNV |
| F33 | F33-22 | 9634 | NS5 | 0.0214 | A:0;G:0;C:265;T:12083;total:12348 | iSNV |
| F33 | F33-22 | 10046 | NS5 | 0.0293 | A:9813;G:297;C:0;T:1;total:10111 | iSNV |
| F33 | F33-22 | 10259 | NS5 | 0.1424 | A:11261;G:1871;C:0;T:0;total:13132 | iSNV |
| F33 | F33-22 | 10428 | 3'-UTR | 0.082 | A:0;G:2;C:11842;T:1058;total:12902 | iSNV |
| F33 | F33-22 | 10447 | 3'-UTR | 0.0763 | A:5;G:0;C:10255;T:848;total:11108 | iSNV |
| F33 | F33-22 | 10566 | 3'-UTR | 0.0942 | A:0;G:1;C:8989;T:935;total:9925 | iSNV |
| F33 | F33-23 | 353 | C | 0.0876 | A:8839;G:849;C:0;T:0;total:9688 | iSNV |
| F33 | F33-23 | 645 | M | 0.0772 | A:7857;G:658;C:0;T:0;total:8515 | iSNV |
| F33 | F33-23 | 752 | M | 0.0415 | A:9468;G:410;C:0;T:1;total:9879 | iSNV |
| F33 | F33-23 | 803 | M | 0.027 | A:5;G:7472;C:0;T:208;total:7685 | iSNV |
| F33 | F33-23 | 998 | E | 0.2786 | A:0;G:0;C:5439;T:2101;total:7540 | iSNV |
| F33 | F33-23 | 1007 | E | 0.0457 | A:2;G:3;C:7626;T:366;total:7997 | iSNV |
| F33 | F33-23 | 1117 | E | 0.1199 | A:7030;G:958;C:0;T:0;total:7988 | iSNV |
| F33 | F33-23 | 1218 | E | 0.8755 | A:1;G:1;C:1024;T:7195;total:8221 | iSNV |
| F33 | F33-23 | 1430 | E | 0.046 | A:0;G:0;C:9068;T:438;total:9506 | iSNV |
| F33 | F33-23 | 1512 | E | 0.0387 | A:7178;G:289;C:0;T:0;total:7467 | iSNV |
| F33 | F33-23 | 2664 | NS1 | 0.0209 | A:4;G:0;C:8492;T:182;total:8678 | iSNV |
| F33 | F33-23 | 3869 | NS2A | 0.7015 | A:0;G:0;C:2380;T:5593;total:7973 | iSNV |
| F33 | F33-23 | 4697 | NS3 | 0.1797 | A:0;G:0;C:1562;T:7126;total:8688 | iSNV |
| F33 | F33-23 | 5102 | NS3 | 0.0505 | A:0;G:0;C:7927;T:422;total:8349 | iSNV |
| F33 | F33-23 | 5693 | NS3 | 0.0203 | A:0;G:0;C:201;T:9670;total:9871 | iSNV |
| F33 | F33-23 | 5952 | NS3 | 0.1847 | A:0;G:0;C:7916;T:1794;total:9710 | iSNV |
| F33 | F33-23 | 6378 | NS3 | 0.0329 | A:6713;G:0;C:0;T:229;total:6942 | iSNV |
| F33 | F33-23 | 6471 | NS4A | 0.0208 | A:150;G:7046;C:1;T:0;total:7197 | iSNV |
| F33 | F33-23 | 7460 | NS4B | 0.0621 | A:324;G:4888;C:0;T:0;total:5212 | iSNV |
| F33 | F33-23 | 7735 | NS5 | 0.0218 | A:181;G:8110;C:0;T:2;total:8293 | iSNV |
| F33 | F33-23 | 8518 | NS5 | 0.0224 | A:9784;G:3;C:0;T:225;total:10012 | iSNV |
| F33 | F33-23 | 9359 | NS5 | 0.1917 | A:1;G:1;C:2145;T:9041;total:11188 | iSNV |
| F33 | F33-23 | 10259 | NS5 | 0.2638 | A:7347;G:2633;C:0;T:0;total:9980 | iSNV |
| F33 | F33-23 | 10428 | 3'-UTR | 0.0582 | A:0;G:1;C:8711;T:539;total:9251 | iSNV |
| F33 | F33-23 | 10447 | 3'-UTR | 0.1069 | A:1;G:0;C:7275;T:871;total:8147 | iSNV |
| F33 | F33-23 | 10566 | 3'-UTR | 0.1765 | A:1;G:0;C:6364;T:1365;total:7730 | iSNV |
| F33 | F33-24 | 353 | C | 0.0699 | A:8926;G:671;C:0;T:1;total:9598 | iSNV |
| F33 | F33-24 | 645 | M | 0.0609 | A:7473;G:485;C:0;T:0;total:7958 | iSNV |
| F33 | F33-24 | 919 | M | 0.0335 | A:7136;G:248;C:0;T:0;total:7384 | iSNV |
| F33 | F33-24 | 998 | E | 0.2873 | A:0;G:0;C:5190;T:2093;total:7283 | iSNV |
| F33 | F33-24 | 1013 | E | 0.0463 | A:7100;G:0;C:345;T:0;total:7445 | iSNV |
| F33 | F33-24 | 1117 | E | 0.0781 | A:7145;G:606;C:0;T:1;total:7752 | iSNV |
| F33 | F33-24 | 1218 | E | 0.9196 | A:0;G:0;C:623;T:7123;total:7746 | iSNV |
| F33 | F33-24 | 1413 | E | 0.1973 | A:7381;G:1815;C:0;T:0;total:9196 | iSNV |
| F33 | F33-24 | 1892 | E | 0.0343 | A:6669;G:237;C:0;T:1;total:6907 | iSNV |
| F33 | F33-24 | 2277 | E | 0.0369 | A:0;G:0;C:221;T:5756;total:5977 | iSNV |
| F33 | F33-24 | 3110 | NS1 | 0.0657 | A:8168;G:575;C:0;T:0;total:8743 | iSNV |

| | | | | | | |
|---|---|---|---|---|---|---|
| F33 | F33-24 | 3257 | NS1 | 0.0288 | A:0;G:0;C:273;T:9199;total:9472 | iSNV |
| F33 | F33-24 | 3869 | NS2A | 0.6949 | A:0;G:0;C:2492;T:5675;total:8167 | iSNV |
| F33 | F33-24 | 4663 | NS3 | 0.0406 | A:0;G:1;C:8073;T:342;total:8416 | iSNV |
| F33 | F33-24 | 4697 | NS3 | 0.2245 | A:0;G:1;C:1994;T:6886;total:8881 | iSNV |
| F33 | F33-24 | 5465 | NS3 | 0.0747 | A:8748;G:707;C:0;T:1;total:9456 | iSNV |
| F33 | F33-24 | 5952 | NS3 | 0.201 | A:0;G:0;C:7737;T:1947;total:9684 | iSNV |
| F33 | F33-24 | 9359 | NS5 | 0.2317 | A:0;G:0;C:2687;T:8908;total:11595 | iSNV |
| F33 | F33-24 | 10259 | NS5 | 0.28 | A:6937;G:2698;C:0;T:0;total:9635 | iSNV |
| F33 | F33-24 | 10376 | NS5 | 0.049 | A:473;G:9166;C:0;T:0;total:9639 | iSNV |
| F33 | F33-24 | 10428 | 3'-UTR | 0.0753 | A:1;G:1;C:8235;T:671;total:8908 | iSNV |
| F33 | F33-24 | 10447 | 3'-UTR | 0.0829 | A:5;G:0;C:6958;T:630;total:7593 | iSNV |
| F33 | F33-24 | 10452 | 3'-UTR | 0.0224 | A:7397;G:170;C:0;T:0;total:7567 | iSNV |
| F33 | F33-24 | 10566 | 3'-UTR | 0.2082 | A:0;G:0;C:5517;T:1451;total:6968 | iSNV |
| F33 | F33-25 | 443 | C | 0.0384 | A:0;G:10157;C:0;T:406;total:10563 | iSNV |
| F33 | F33-25 | 491 | M | 0.0229 | A:1;G:0;C:277;T:11797;total:12075 | iSNV |
| F33 | F33-25 | 998 | E | 0.0766 | A:1;G:0;C:7748;T:643;total:8392 | iSNV |
| F33 | F33-25 | 1117 | E | 0.4326 | A:5162;G:3937;C:0;T:0;total:9099 | iSNV |
| F33 | F33-25 | 1218 | E | 0.9982 | A:2;G:0;C:15;T:9008;total:9025 | SNP |
| F33 | F33-25 | 2213 | E | 0.0228 | A:196;G:8371;C:0;T:0;total:8567 | iSNV |
| F33 | F33-25 | 2369 | E | 0.0248 | A:142;G:5467;C:0;T:97;total:5706 | iSNV |
| F33 | F33-25 | 2855 | NS1 | 0.4233 | A:5081;G:6919;C:0;T:1;total:12001 | iSNV |
| F33 | F33-25 | 3356 | NS1 | 0.0397 | A:325;G:7861;C:0;T:0;total:8186 | iSNV |
| F33 | F33-25 | 3869 | NS2A | 0.9245 | A:1;G:0;C:706;T:8635;total:9342 | iSNV |
| F33 | F33-25 | 4697 | NS3 | 0.075 | A:0;G:0;C:768;T:9469;total:10237 | iSNV |
| F33 | F33-25 | 5558 | NS3 | 0.4311 | A:4384;G:5784;C:0;T:0;total:10168 | iSNV |
| F33 | F33-25 | 5952 | NS3 | 0.0711 | A:1;G:0;C:10694;T:819;total:11514 | iSNV |
| F33 | F33-25 | 6431 | NS3 | 0.031 | A:0;G:0;C:7979;T:256;total:8235 | iSNV |
| F33 | F33-25 | 9359 | NS5 | 0.0768 | A:0;G:1;C:1010;T:12126;total:13137 | iSNV |
| F33 | F33-25 | 9818 | NS5 | 0.0601 | A:0;G:0;C:564;T:8809;total:9373 | iSNV |
| F33 | F33-25 | 10259 | NS5 | 0.0728 | A:10874;G:855;C:0;T:0;total:11729 | iSNV |
| F33 | F33-25 | 10428 | 3'-UTR | 0.0938 | A:1;G:0;C:9388;T:972;total:10361 | iSNV |
| F33 | F33-25 | 10566 | 3'-UTR | 0.0671 | A:2;G:0;C:8047;T:579;total:8628 | iSNV |
| F33 | F33-26 | 470 | C | 0.0222 | A:9679;G:0;C:0;T:220;total:9899 | iSNV |
| F33 | F33-26 | 998 | E | 0.1425 | A:1;G:0;C:6146;T:1022;total:7169 | iSNV |
| F33 | F33-26 | 1043 | E | 0.0366 | A:0;G:0;C:7676;T:292;total:7968 | iSNV |
| F33 | F33-26 | 1218 | E | 0.9953 | A:0;G:0;C:37;T:7804;total:7841 | SNP |
| F33 | F33-26 | 1263 | E | 0.0201 | A:0;G:0;C:166;T:8069;total:8235 | iSNV |
| F33 | F33-26 | 1428 | E | 0.0341 | A:8606;G:304;C:0;T:0;total:8910 | iSNV |
| F33 | F33-26 | 2235 | E | 0.0273 | A:0;G:1;C:186;T:6618;total:6805 | iSNV |
| F33 | F33-26 | 2474 | E | 0.0318 | A:184;G:3;C:0;T:5588;total:5775 | iSNV |
| F33 | F33-26 | 3257 | NS1 | 0.0342 | A:0;G:0;C:318;T:8972;total:9290 | iSNV |
| F33 | F33-26 | 3869 | NS2A | 0.8673 | A:0;G:0;C:1037;T:6776;total:7813 | iSNV |
| F33 | F33-26 | 3993 | NS2A | 0.0255 | A:206;G:7853;C:1;T:0;total:8060 | iSNV |
| F33 | F33-26 | 4289 | NS2B | 0.0215 | A:0;G:0;C:143;T:6495;total:6638 | iSNV |
| F33 | F33-26 | 4697 | NS3 | 0.1366 | A:1;G:0;C:1198;T:7569;total:8768 | iSNV |
| F33 | F33-26 | 5952 | NS3 | 0.1225 | A:0;G:0;C:8414;T:1175;total:9589 | iSNV |
| F33 | F33-26 | 6080 | NS3 | 0.0694 | A:498;G:6671;C:0;T:0;total:7169 | iSNV |
| F33 | F33-26 | 6626 | NS4A | 0.0243 | A:0;G:0;C:6805;T:170;total:6975 | iSNV |
| F33 | F33-26 | 6806 | NS4A | 0.0222 | A:0;G:7115;C:0;T:162;total:7277 | iSNV |
| F33 | F33-26 | 7172 | NS4A | 0.0291 | A:0;G:0;C:198;T:6606;total:6804 | iSNV |
| F33 | F33-26 | 7244 | NS4A | 0.025 | A:0;G:0;C:136;T:5299;total:5435 | iSNV |
| F33 | F33-26 | 9359 | NS5 | 0.1307 | A:0;G:0;C:1479;T:9834;total:11313 | iSNV |
| F33 | F33-26 | 10259 | NS5 | 0.135 | A:8674;G:1355;C:0;T:1;total:10030 | iSNV |
| F33 | F33-26 | 10428 | 3'-UTR | 0.1803 | A:0;G:1;C:7344;T:1616;total:8961 | iSNV |
| F33 | F33-26 | 10429 | 3'-UTR | 0.0301 | A:8676;G:270;C:0;T:0;total:8946 | iSNV |
| F33 | F33-26 | 10566 | 3'-UTR | 0.132 | A:0;G:0;C:6578;T:1001;total:7579 | iSNV |
| F33 | F33-27 | 941 | M | 0.0283 | A:204;G:0;C:0;T:6988;total:7192 | iSNV |
| F33 | F33-27 | 998 | E | 0.0236 | A:1;G:1;C:7213;T:175;total:7390 | iSNV |
| F33 | F33-27 | 1218 | E | 0.9975 | A:0;G:0;C:21;T:8192;total:8213 | SNP |
| F33 | F33-27 | 1413 | E | 0.0335 | A:9344;G:324;C:0;T:0;total:9668 | iSNV |
| F33 | F33-27 | 2274 | E | 0.0779 | A:0;G:5335;C:0;T:451;total:5786 | iSNV |
| F33 | F33-27 | 2664 | NS1 | 0.7281 | A:9;G:0;C:2468;T:6597;total:9074 | iSNV |
| F33 | F33-27 | 3869 | NS2A | 0.9792 | A:0;G:0;C:199;T:9346;total:9545 | iSNV |
| F33 | F33-27 | 4697 | NS3 | 0.0206 | A:0;G:0;C:186;T:8811;total:8997 | iSNV |
| F33 | F33-27 | 6068 | NS3 | 0.0362 | A:316;G:0;C:8389;T:4;total:8709 | iSNV |
| F33 | F33-27 | 7626 | NS4B | 0.7596 | A:5;G:6601;C:0;T:2091;total:8697 | iSNV |
| F33 | F33-27 | 8765 | NS5 | 0.0242 | A:206;G:8281;C:0;T:1;total:8488 | iSNV |
| F33 | F33-27 | 10376 | NS5 | 0.0328 | A:383;G:11264;C:0;T:2;total:11649 | iSNV |
| F33 | F33-27 | 10419 | 3'-UTR | 0.0697 | A:0;G:4;C:9616;T:721;total:10341 | iSNV |
| F33 | F33-27 | 10428 | 3'-UTR | 0.0893 | A:0;G:0;C:9553;T:937;total:10490 | iSNV |
| F33 | F33-27 | 10447 | 3'-UTR | 0.0211 | A:3;G:1;C:8638;T:187;total:8829 | iSNV |
| F33 | F33-28 | 230 | C | 0.0322 | A:0;G:3;C:159;T:4773;total:4935 | iSNV |
| F33 | F33-28 | 353 | C | 0.0592 | A:5064;G:319;C:0;T:0;total:5383 | iSNV |
| F33 | F33-28 | 645 | M | 0.0665 | A:4178;G:298;C:0;T:0;total:4476 | iSNV |
| F33 | F33-28 | 869 | M | 0.1335 | A:502;G:0;C:0;T:3258;total:3760 | iSNV |
| F33 | F33-28 | 897 | M | 0.0343 | A:0;G:0;C:2926;T:104;total:3030 | iSNV |
| F33 | F33-28 | 946 | M | 0.0692 | A:0;G:239;C:0;T:3211;total:3450 | iSNV |
| F33 | F33-28 | 998 | E | 0.1436 | A:0;G:0;C:3029;T:508;total:3537 | iSNV |
| F33 | F33-28 | 1117 | E | 0.0799 | A:3489;G:303;C:0;T:0;total:3792 | iSNV |
| F33 | F33-28 | 1218 | E | 0.9001 | A:0;G:0;C:373;T:3360;total:3733 | iSNV |
| F33 | F33-28 | 1428 | E | 0.0358 | A:3791;G:141;C:0;T:0;total:3932 | iSNV |
| F33 | F33-28 | 1478 | E | 0.0655 | A:0;G:0;C:203;T:2893;total:3096 | iSNV |

| | | | | | | |
|---|---|---|---|---|---|---|
| F33 | F33-28 | 2213 | E | 0.0731 | A:252;G:3194;C:0;T:0;total:3446 | iSNV |
| F33 | F33-28 | 2879 | NS1 | 0.0355 | A:0;G:0;C:5608;T:207;total:5815 | iSNV |
| F33 | F33-28 | 3869 | NS2A | 0.8514 | A:0;G:0;C:711;T:4073;total:4784 | iSNV |
| F33 | F33-28 | 4427 | NS2B | 0.1203 | A:0;G:0;C:2718;T:372;total:3090 | iSNV |
| F33 | F33-28 | 4697 | NS3 | 0.0598 | A:0;G:0;C:247;T:3883;total:4130 | iSNV |
| F33 | F33-28 | 4742 | NS3 | 0.0413 | A:0;G:0;C:188;T:4364;total:4552 | iSNV |
| F33 | F33-28 | 5654 | NS3 | 0.0539 | A:1;G:1;C:4419;T:252;total:4673 | iSNV |
| F33 | F33-28 | 5665 | NS3 | 0.0586 | A:0;G:0;C:4705;T:293;total:4998 | iSNV |
| F33 | F33-28 | 5705 | NS3 | 0.0227 | A:4984;G:116;C:0;T:0;total:5100 | iSNV |
| F33 | F33-28 | 5952 | NS3 | 0.0618 | A:0;G:0;C:4886;T:322;total:5208 | iSNV |
| F33 | F33-28 | 7592 | NS4B | 0.0638 | A:0;G:0;C:3283;T:224;total:3507 | iSNV |
| F33 | F33-28 | 8789 | NS5 | 0.0222 | A:4214;G:96;C:0;T:0;total:4310 | iSNV |
| F33 | F33-28 | 9359 | NS5 | 0.0621 | A:0;G:0;C:406;T:6128;total:6534 | iSNV |
| F33 | F33-28 | 9634 | NS5 | 0.0842 | A:0;G:0;C:274;T:2980;total:3254 | iSNV |
| F33 | F33-28 | 10259 | NS5 | 0.1379 | A:4498;G:720;C:0;T:1;total:5219 | iSNV |
| F33 | F33-28 | 10428 | 3'-UTR | 0.098 | A:0;G:1;C:4104;T:446;total:4551 | iSNV |
| F33 | F33-28 | 10447 | 3'-UTR | 0.0867 | A:0;G:0;C:3443;T:327;total:3770 | iSNV |
| F33 | F33-28 | 10452 | 3'-UTR | 0.065 | A:3494;G:243;C:0;T:1;total:3738 | iSNV |
| F33 | F33-28 | 10566 | 3'-UTR | 0.0543 | A:1;G:0;C:3167;T:182;total:3350 | iSNV |
| F33 | F33-29 | 996 | E | 0.0301 | A:200;G:6440;C:0;T:0;total:6640 | iSNV |
| F33 | F33-29 | 998 | E | 0.0255 | A:0;G:0;C:6401;T:168;total:6569 | iSNV |
| F33 | F33-29 | 1218 | E | 0.9921 | A:0;G:0;C:57;T:7137;total:7194 | SNP |
| F33 | F33-29 | 1413 | E | 0.1787 | A:7023;G:1529;C:0;T:1;total:8553 | iSNV |
| F33 | F33-29 | 1514 | E | 0.0239 | A:5756;G:0;C:141;T:0;total:5897 | iSNV |
| F33 | F33-29 | 2362 | E | 0.5172 | A:0;G:1956;C:2093;T:2;total:4051 | iSNV |
| F33 | F33-29 | 3869 | NS2A | 0.9802 | A:0;G:0;C:153;T:7551;total:7704 | SNP |
| F33 | F33-29 | 5702 | NS3 | 0.2508 | A:0;G:0;C:7385;T:2473;total:9858 | iSNV |
| F33 | F33-29 | 6753 | NS4A | 0.0341 | A:8;G:5445;C:0;T:193;total:5646 | iSNV |
| F33 | F33-29 | 7481 | NS4B | 0.2685 | A:0;G:0;C:1230;T:3350;total:4580 | iSNV |
| F33 | F33-29 | 7558 | NS4B | 0.0217 | A:0;G:0;C:6806;T:151;total:6957 | iSNV |
| F33 | F33-29 | 7967 | NS5 | 0.026 | A:2;G:0;C:9831;T:263;total:10096 | iSNV |
| F33 | F33-29 | 9359 | NS5 | 0.0205 | A:0;G:0;C:231;T:10987;total:11218 | iSNV |
| F33 | F33-29 | 9634 | NS5 | 0.0297 | A:0;G:0;C:207;T:6761;total:6968 | iSNV |
| F33 | F33-29 | 10259 | NS5 | 0.0233 | A:8976;G:215;C:0;T:0;total:9191 | iSNV |
| F33 | F33-29 | 10376 | NS5 | 0.1689 | A:1535;G:7551;C:0;T:0;total:9086 | iSNV |
| F33 | F33-29 | 10428 | 3'-UTR | 0.0557 | A:0;G:0;C:7911;T:467;total:8378 | iSNV |
| F33 | F33-29 | 10447 | 3'-UTR | 0.0288 | A:0;G:0;C:6875;T:204;total:7079 | iSNV |
| F33 | F33-3 | 353 | C | 0.0223 | A:16024;G:367;C:0;T:1;total:16392 | iSNV |
| F33 | F33-3 | 645 | M | 0.0201 | A:13486;G:277;C:0;T:0;total:13763 | iSNV |
| F33 | F33-3 | 828 | M | 0.2512 | A:8711;G:2923;C:0;T:1;total:11635 | iSNV |
| F33 | F33-3 | 996 | E | 0.5583 | A:6581;G:5209;C:0;T:1;total:11791 | iSNV |
| F33 | F33-3 | 998 | E | 0.0914 | A:0;G:0;C:10898;T:1097;total:11995 | iSNV |
| F33 | F33-3 | 1117 | E | 0.0247 | A:12437;G:316;C:0;T:0;total:12753 | iSNV |
| F33 | F33-3 | 1218 | E | 0.9738 | A:0;G:0;C:335;T:12408;total:12743 | iSNV |
| F33 | F33-3 | 1430 | E | 0.0201 | A:0;G:1;C:15528;T:320;total:15849 | iSNV |
| F33 | F33-3 | 2465 | E | 0.5925 | A:1;G:0;C:3379;T:4911;total:8291 | iSNV |
| F33 | F33-3 | 3858 | NS2A | 0.4598 | A:0;G:0;C:7475;T:6365;total:13840 | iSNV |
| F33 | F33-3 | 3869 | NS2A | 0.9075 | A:1;G:0;C:1332;T:13054;total:14387 | iSNV |
| F33 | F33-3 | 4697 | NS3 | 0.0657 | A:0;G:0;C:1027;T:14584;total:15611 | iSNV |
| F33 | F33-3 | 5952 | NS3 | 0.0628 | A:0;G:0;C:15457;T:1037;total:16494 | iSNV |
| F33 | F33-3 | 7585 | NS4B | 0.0205 | A:1;G:11271;C:236;T:1;total:11509 | iSNV |
| F33 | F33-3 | 9359 | NS5 | 0.0663 | A:3;G:0;C:1304;T:18333;total:19640 | iSNV |
| F33 | F33-3 | 10259 | NS5 | 0.0849 | A:15128;G:1404;C:0;T:2;total:16534 | iSNV |
| F33 | F33-3 | 10428 | 3'-UTR | 0.061 | A:0;G:2;C:14475;T:942;total:15419 | iSNV |
| F33 | F33-3 | 10447 | 3'-UTR | 0.0467 | A:3;G:0;C:13282;T:652;total:13937 | iSNV |
| F33 | F33-3 | 10566 | 3'-UTR | 0.0645 | A:0;G:1;C:11468;T:792;total:12261 | iSNV |
| F33 | F33-30 | 1218 | E | 0.9959 | A:0;G:0;C:24;T:5782;total:5806 | SNP |
| F33 | F33-30 | 1413 | E | 0.9201 | A:554;G:6374;C:0;T:0;total:6928 | iSNV |
| F33 | F33-30 | 3869 | NS2A | 0.9838 | A:0;G:0;C:105;T:6373;total:6478 | SNP |
| F33 | F33-30 | 8394 | NS5 | 0.2719 | A:0;G:1;C:2203;T:5896;total:8100 | iSNV |
| F33 | F33-30 | 10376 | NS5 | 0.9099 | A:6688;G:663;C:0;T:0;total:7351 | iSNV |
| F33 | F33-30 | 10428 | 3'-UTR | 0.0451 | A:0;G:0;C:6559;T:310;total:6869 | iSNV |
| F33 | F33-4 | 998 | E | 0.1202 | A:0;G:0;C:6373;T:871;total:7244 | iSNV |
| F33 | F33-4 | 1218 | E | 0.9966 | A:0;G:0;C:27;T:7795;total:7822 | SNP |
| F33 | F33-4 | 1430 | E | 0.0264 | A:0;G:0;C:9143;T:248;total:9391 | iSNV |
| F33 | F33-4 | 2367 | E | 0.3262 | A:3340;G:1632;C:31;T:0;total:5003 | iSNV |
| F33 | F33-4 | 3573 | NS1 | 0.0205 | A:2;G:0;C:5858;T:123;total:5983 | iSNV |
| F33 | F33-4 | 3869 | NS2A | 0.8791 | A:0;G:0;C:958;T:6961;total:7919 | iSNV |
| F33 | F33-4 | 4187 | NS2A | 0.0213 | A:3668;G:80;C:0;T:0;total:3748 | iSNV |
| F33 | F33-4 | 4697 | NS3 | 0.1128 | A:0;G:0;C:1027;T:8075;total:9102 | iSNV |
| F33 | F33-4 | 5952 | NS3 | 0.1121 | A:2;G:0;C:8302;T:1049;total:9353 | iSNV |
| F33 | F33-4 | 6182 | NS3 | 0.025 | A:0;G:0;C:169;T:6591;total:6760 | iSNV |
| F33 | F33-4 | 6322 | NS3 | 0.0809 | A:0;G:0;C:6449;T:568;total:7017 | iSNV |
| F33 | F33-4 | 7097 | NS4A | 0.039 | A:306;G:7524;C:0;T:0;total:7830 | iSNV |
| F33 | F33-4 | 9359 | NS5 | 0.1117 | A:0;G:0;C:1284;T:10208;total:11492 | iSNV |
| F33 | F33-4 | 10259 | NS5 | 0.1131 | A:8672;G:1106;C:0;T:0;total:9778 | iSNV |
| F33 | F33-4 | 10428 | 3'-UTR | 0.0505 | A:0;G:0;C:8552;T:455;total:9007 | iSNV |
| F33 | F33-4 | 10447 | 3'-UTR | 0.0233 | A:2;G:0;C:7956;T:190;total:8148 | iSNV |
| F33 | F33-4 | 10566 | 3'-UTR | 0.1058 | A:0;G:0;C:6707;T:794;total:7501 | iSNV |
| F33 | F33-5 | 294 | C | 0.0332 | A:441;G:12830;C:0;T:3;total:13274 | iSNV |
| F33 | F33-5 | 353 | C | 0.0365 | A:12614;G:478;C:0;T:1;total:13093 | iSNV |
| F33 | F33-5 | 399 | C | 0.1562 | A:2298;G:2;C:12404;T:4;total:14708 | iSNV |

| | | | | | | |
|---|---|---|---|---|---|---|
| F33 | F33-5 | 645 | M | 0.0346 | A:10633;G:382;C:1;T:0;total:11016 | iSNV |
| F33 | F33-5 | 897 | M | 0.1493 | A:2;G:0;C:7985;T:1402;total:9389 | iSNV |
| F33 | F33-5 | 954 | M | 0.039 | A:0;G:0;C:9918;T:403;total:10321 | iSNV |
| F33 | F33-5 | 998 | E | 0.1268 | A:2;G:0;C:8481;T:1232;total:9715 | iSNV |
| F33 | F33-5 | 1083 | E | 0.0227 | A:8898;G:207;C:0;T:2;total:9107 | iSNV |
| F33 | F33-5 | 1117 | E | 0.0475 | A:9688;G:484;C:0;T:0;total:10172 | iSNV |
| F33 | F33-5 | 1218 | E | 0.9508 | A:1;G:1;C:486;T:9385;total:9873 | iSNV |
| F33 | F33-5 | 1413 | E | 0.0681 | A:11754;G:860;C:1;T:1;total:12616 | iSNV |
| F33 | F33-5 | 2126 | E | 0.1107 | A:0;G:0;C:7125;T:887;total:8012 | iSNV |
| F33 | F33-5 | 2543 | NS1 | 0.0701 | A:3;G:0;C:7598;T:573;total:8174 | iSNV |
| F33 | F33-5 | 3110 | NS1 | 0.0213 | A:11976;G:261;C:0;T:0;total:12237 | iSNV |
| F33 | F33-5 | 3869 | NS2A | 0.878 | A:2;G:1;C:1346;T:9681;total:11030 | iSNV |
| F33 | F33-5 | 4697 | NS3 | 0.079 | A:0;G:0;C:956;T:11144;total:12100 | iSNV |
| F33 | F33-5 | 4790 | NS3 | 0.0561 | A:11590;G:690;C:1;T:0;total:12281 | iSNV |
| F33 | F33-5 | 5952 | NS3 | 0.0795 | A:0;G:0;C:11948;T:1033;total:12981 | iSNV |
| F33 | F33-5 | 6753 | NS4A | 0.0336 | A:2;G:7559;C:0;T:263;total:7824 | iSNV |
| F33 | F33-5 | 7182 | NS4A | 0.0311 | A:3;G:0;C:8106;T:261;total:8370 | iSNV |
| F33 | F33-5 | 8130 | NS5 | 0.0331 | A:1;G:0;C:13758;T:472;total:14231 | iSNV |
| F33 | F33-5 | 9341 | NS5 | 0.0203 | A:14691;G:305;C:0;T:0;total:14996 | iSNV |
| F33 | F33-5 | 9359 | NS5 | 0.0852 | A:2;G:1;C:1293;T:13874;total:15170 | iSNV |
| F33 | F33-5 | 10259 | NS5 | 0.1176 | A:11889;G:1585;C:0;T:1;total:13475 | iSNV |
| F33 | F33-5 | 10376 | NS5 | 0.0658 | A:825;G:11706;C:0;T:0;total:12531 | iSNV |
| F33 | F33-5 | 10407 | 3'-UTR | 0.0615 | A:0;G:1;C:739;T:11264;total:12004 | iSNV |
| F33 | F33-5 | 10428 | 3'-UTR | 0.0878 | A:1;G:0;C:10916;T:1052;total:11969 | iSNV |
| F33 | F33-5 | 10447 | 3'-UTR | 0.059 | A:1;G:2;C:10024;T:629;total:10656 | iSNV |
| F33 | F33-5 | 10452 | 3'-UTR | 0.0225 | A:10361;G:239;C:0;T:16;total:10616 | iSNV |
| F33 | F33-5 | 10566 | 3'-UTR | 0.0814 | A:4;G:1;C:9017;T:800;total:9822 | iSNV |
| F33 | F33-6 | 353 | C | 0.1016 | A:11555;G:1307;C:0;T:1;total:12863 | iSNV |
| F33 | F33-6 | 645 | M | 0.0989 | A:9869;G:1084;C:0;T:1;total:10954 | iSNV |
| F33 | F33-6 | 658 | M | 0.053 | A:10332;G:579;C:1;T:2;total:10914 | iSNV |
| F33 | F33-6 | 752 | M | 0.0217 | A:12257;G:273;C:1;T:0;total:12531 | iSNV |
| F33 | F33-6 | 998 | E | 0.1405 | A:1;G:0;C:7744;T:1267;total:9012 | iSNV |
| F33 | F33-6 | 1117 | E | 0.1122 | A:8648;G:1094;C:0;T:1;total:9743 | iSNV |
| F33 | F33-6 | 1218 | E | 0.8816 | A:0;G:0;C:1115;T:8300;total:9415 | iSNV |
| F33 | F33-6 | 1430 | E | 0.1518 | A:0;G:0;C:9760;T:1747;total:11507 | iSNV |
| F33 | F33-6 | 2367 | E | 0.1717 | A:4195;G:871;C:0;T:6;total:5072 | iSNV |
| F33 | F33-6 | 3869 | NS2A | 0.871 | A:0;G:1;C:1286;T:8681;total:9968 | iSNV |
| F33 | F33-6 | 4697 | NS3 | 0.026 | A:0;G:0;C:291;T:10861;total:11152 | iSNV |
| F33 | F33-6 | 4783 | NS3 | 0.1503 | A:1;G:0;C:1624;T:9180;total:10805 | iSNV |
| F33 | F33-6 | 4974 | NS3 | 0.0439 | A:449;G:9762;C:0;T:0;total:10211 | iSNV |
| F33 | F33-6 | 5102 | NS3 | 0.0271 | A:0;G:0;C:9398;T:262;total:9660 | iSNV |
| F33 | F33-6 | 5513 | NS3 | 0.0306 | A:1;G:0;C:395;T:12496;total:12892 | iSNV |
| F33 | F33-6 | 5952 | NS3 | 0.0269 | A:0;G:0;C:11716;T:324;total:12040 | iSNV |
| F33 | F33-6 | 6061 | NS3 | 0.0411 | A:9098;G:390;C:0;T:0;total:9488 | iSNV |
| F33 | F33-6 | 7060 | NS4A | 0.0436 | A:0;G:1;C:342;T:7491;total:7834 | iSNV |
| F33 | F33-6 | 7190 | NS4A | 0.0294 | A:1;G:6283;C:0;T:191;total:6475 | iSNV |
| F33 | F33-6 | 9359 | NS5 | 0.0302 | A:0;G:1;C:440;T:14114;total:14555 | iSNV |
| F33 | F33-6 | 9491 | NS5 | 0.0913 | A:9326;G:938;C:1;T:0;total:10265 | iSNV |
| F33 | F33-6 | 9785 | NS5 | 0.0278 | A:255;G:1;C:3;T:8882;total:9141 | iSNV |
| F33 | F33-6 | 10259 | NS5 | 0.1236 | A:11271;G:1591;C:1;T:1;total:12864 | iSNV |
| F33 | F33-6 | 10419 | 3'-UTR | 0.0603 | A:3;G:2;C:10933;T:702;total:11640 | iSNV |
| F33 | F33-6 | 10428 | 3'-UTR | 0.1359 | A:2;G:0;C:10249;T:1613;total:11864 | iSNV |
| F33 | F33-6 | 10447 | 3'-UTR | 0.1186 | A:3;G:9;C:9244;T:1246;total:10502 | iSNV |
| F33 | F33-6 | 10566 | 3'-UTR | 0.0273 | A:0;G:0;C:8794;T:247;total:9041 | iSNV |
| F33 | F33-6 | 10577 | 3'-UTR | 0.0237 | A:2;G:0;C:8754;T:213;total:8969 | iSNV |
| F33 | F33-7 | 998 | E | 0.06 | A:1;G:0;C:7298;T:466;total:7765 | iSNV |
| F33 | F33-7 | 1218 | E | 0.9901 | A:0;G:0;C:87;T:8637;total:8724 | SNP |
| F33 | F33-7 | 1413 | E | 0.1609 | A:8994;G:1725;C:0;T:0;total:10719 | iSNV |
| F33 | F33-7 | 1428 | E | 0.02 | A:10171;G:208;C:0;T:0;total:10379 | iSNV |
| F33 | F33-7 | 1907 | E | 0.0312 | A:1;G:0;C:7498;T:242;total:7741 | iSNV |
| F33 | F33-7 | 3257 | NS1 | 0.1219 | A:0;G:0;C:1453;T:10459;total:11912 | iSNV |
| F33 | F33-7 | 3317 | NS1 | 0.2198 | A:5;G:0;C:9088;T:2563;total:11656 | iSNV |
| F33 | F33-7 | 3869 | NS2A | 0.9279 | A:1;G:0;C:727;T:9350;total:10078 | iSNV |
| F33 | F33-7 | 4294 | NS2B | 0.0219 | A:7519;G:169;C:0;T:0;total:7688 | iSNV |
| F33 | F33-7 | 4697 | NS3 | 0.0643 | A:2;G:0;C:707;T:10276;total:10985 | iSNV |
| F33 | F33-7 | 5952 | NS3 | 0.0628 | A:1;G:0;C:11586;T:777;total:12364 | iSNV |
| F33 | F33-7 | 6322 | NS3 | 0.2083 | A:0;G:0;C:6026;T:1586;total:7612 | iSNV |
| F33 | F33-7 | 9086 | NS5 | 0.0266 | A:0;G:0;C:269;T:9807;total:10076 | iSNV |
| F33 | F33-7 | 9359 | NS5 | 0.0613 | A:0;G:0;C:825;T:12629;total:13454 | iSNV |
| F33 | F33-7 | 9370 | NS5 | 0.1089 | A:1476;G:6;C:4;T:12062;total:13548 | iSNV |
| F33 | F33-7 | 9491 | NS5 | 0.0205 | A:9724;G:204;C:0;T:1;total:9929 | iSNV |
| F33 | F33-7 | 9690 | NS5 | 0.2699 | A:2222;G:6008;C:0;T:0;total:8230 | iSNV |
| F33 | F33-7 | 10259 | NS5 | 0.0664 | A:10227;G:728;C:0;T:1;total:10956 | iSNV |
| F33 | F33-7 | 10376 | NS5 | 0.1502 | A:1606;G:9085;C:0;T:1;total:10692 | iSNV |
| F33 | F33-7 | 10428 | 3'-UTR | 0.106 | A:0;G:4;C:8764;T:1040;total:9808 | iSNV |
| F33 | F33-7 | 10447 | 3'-UTR | 0.0246 | A:0;G:0;C:8421;T:213;total:8634 | iSNV |
| F33 | F33-7 | 10566 | 3'-UTR | 0.0525 | A:2;G:0;C:7187;T:399;total:7588 | iSNV |
| F33 | F33-8 | 869 | M | 0.3814 | A:2792;G:3;C:0;T:4525;total:7320 | iSNV |
| F33 | F33-8 | 998 | E | 0.0512 | A:0;G:1;C:6202;T:335;total:6538 | iSNV |
| F33 | F33-8 | 1218 | E | 0.9972 | A:2;G:0;C:19;T:7226;total:7247 | SNP |
| F33 | F33-8 | 1428 | E | 0.0241 | A:8672;G:215;C:2;T:1;total:8890 | iSNV |
| F33 | F33-8 | 1430 | E | 0.402 | A:1;G:0;C:5253;T:3533;total:8787 | iSNV |

| | | | | | | |
|---|---|---|---|---|---|---|
| F33 | F33-8 | 1453 | E | 0.0371 | A:0;G:0;C:325;T:8419;total:8744 | iSNV |
| F33 | F33-8 | 1512 | E | 0.0272 | A:6195;G:175;C:45;T:0;total:6415 | iSNV |
| F33 | F33-8 | 3131 | NS1 | 0.136 | A:8515;G:1341;C:0;T:0;total:9856 | iSNV |
| F33 | F33-8 | 3625 | NS1 | 0.0287 | A:166;G:5603;C:0;T:0;total:5769 | iSNV |
| F33 | F33-8 | 3869 | NS2A | 0.9588 | A:0;G:0;C:350;T:8135;total:8485 | iSNV |
| F33 | F33-8 | 4187 | NS2A | 0.0274 | A:3643;G:103;C:0;T:0;total:3746 | iSNV |
| F33 | F33-8 | 4697 | NS3 | 0.0447 | A:0;G:0;C:402;T:8574;total:8976 | iSNV |
| F33 | F33-8 | 4835 | NS3 | 0.0305 | A:2;G:0;C:9352;T:295;total:9649 | iSNV |
| F33 | F33-8 | 4896 | NS3 | 0.0386 | A:371;G:9221;C:0;T:0;total:9592 | iSNV |
| F33 | F33-8 | 5353 | NS3 | 0.0435 | A:7189;G:327;C:0;T:1;total:7517 | iSNV |
| F33 | F33-8 | 5665 | NS3 | 0.0371 | A:0;G:0;C:9230;T:356;total:9586 | iSNV |
| F33 | F33-8 | 5952 | NS3 | 0.0398 | A:0;G:1;C:9373;T:389;total:9763 | iSNV |
| F33 | F33-8 | 6314 | NS3 | 0.033 | A:0;G:0;C:218;T:6383;total:6601 | iSNV |
| F33 | F33-8 | 7172 | NS4A | 0.1856 | A:0;G:0;C:1230;T:5394;total:6624 | iSNV |
| F33 | F33-8 | 7509 | NS4B | 0.1899 | A:1108;G:4726;C:0;T:0;total:5834 | iSNV |
| F33 | F33-8 | 9359 | NS5 | 0.0429 | A:0;G:0;C:462;T:10302;total:10764 | iSNV |
| F33 | F33-8 | 9818 | NS5 | 0.053 | A:0;G:0;C:350;T:6250;total:6600 | iSNV |
| F33 | F33-8 | 10259 | NS5 | 0.0416 | A:8600;G:374;C:0;T:0;total:8974 | iSNV |
| F33 | F33-8 | 10428 | 3'-UTR | 0.0654 | A:1;G:1;C:7251;T:508;total:7761 | iSNV |
| F33 | F33-8 | 10447 | 3'-UTR | 0.0624 | A:2;G:0;C:6440;T:429;total:6871 | iSNV |
| F33 | F33-8 | 10452 | 3'-UTR | 0.0461 | A:6524;G:316;C:0;T:1;total:6841 | iSNV |
| F33 | F33-8 | 10566 | 3'-UTR | 0.0392 | A:1;G:1;C:5940;T:243;total:6185 | iSNV |
| F33 | F33-9 | 900 | M | 0.035 | A:274;G:7533;C:0;T:2;total:7809 | iSNV |
| F33 | F33-9 | 998 | E | 0.0694 | A:1;G:0;C:7793;T:582;total:8376 | iSNV |
| F33 | F33-9 | 1218 | E | 0.9992 | A:0;G:1;C:7;T:9405;total:9413 | SNP |
| F33 | F33-9 | 1413 | E | 0.118 | A:10211;G:1367;C:0;T:1;total:11579 | iSNV |
| F33 | F33-9 | 1430 | E | 0.0309 | A:0;G:0;C:11080;T:354;total:11434 | iSNV |
| F33 | F33-9 | 2258 | E | 0.0399 | A:0;G:0;C:279;T:6697;total:6976 | iSNV |
| F33 | F33-9 | 2354 | E | 0.0268 | A:0;G:0;C:5301;T:146;total:5447 | iSNV |
| F33 | F33-9 | 2664 | NS1 | 0.0255 | A:0;G:0;C:10775;T:283;total:11058 | iSNV |
| F33 | F33-9 | 2942 | NS1 | 0.0222 | A:6;G:0;C:11350;T:258;total:11614 | iSNV |
| F33 | F33-9 | 3869 | NS2A | 0.9239 | A:1;G:0;C:811;T:9833;total:10645 | iSNV |
| F33 | F33-9 | 4187 | NS2A | 0.0553 | A:4571;G:268;C:0;T:0;total:4839 | iSNV |
| F33 | F33-9 | 4319 | NS2B | 0.0278 | A:251;G:8754;C:0;T:0;total:9005 | iSNV |
| F33 | F33-9 | 4697 | NS3 | 0.0713 | A:0;G:1;C:802;T:10437;total:11240 | iSNV |
| F33 | F33-9 | 5952 | NS3 | 0.0602 | A:0;G:0;C:11373;T:729;total:12102 | iSNV |
| F33 | F33-9 | 6093 | NS3 | 0.0228 | A:0;G:1;C:214;T:9167;total:9382 | iSNV |
| F33 | F33-9 | 6938 | NS4A | 0.0283 | A:0;G:0;C:8575;T:250;total:8825 | iSNV |
| F33 | F33-9 | 7187 | NS4A | 0.0248 | A:1;G:0;C:8047;T:205;total:8253 | iSNV |
| F33 | F33-9 | 9359 | NS5 | 0.0659 | A:0;G:0;C:877;T:12419;total:13296 | iSNV |
| F33 | F33-9 | 9533 | NS5 | 0.0274 | A:0;G:0;C:284;T:10045;total:10329 | iSNV |
| F33 | F33-9 | 9607 | NS5 | 0.0261 | A:9185;G:13;C:247;T:1;total:9446 | iSNV |
| F33 | F33-9 | 9728 | NS5 | 0.0252 | A:0;G:1;C:8206;T:213;total:8420 | iSNV |
| F33 | F33-9 | 10259 | NS5 | 0.0616 | A:10392;G:683;C:0;T:1;total:11076 | iSNV |
| F33 | F33-9 | 10376 | NS5 | 0.1078 | A:1104;G:9133;C:0;T:3;total:10240 | iSNV |
| F33 | F33-9 | 10428 | 3'-UTR | 0.0753 | A:1;G:0;C:8798;T:717;total:9516 | iSNV |
| F33 | F33-9 | 10566 | 3'-UTR | 0.0694 | A:0;G:0;C:7314;T:546;total:7860 | iSNV |
| F36 | F36-1 | 876 | M | 0.0229 | A:0;G:0;C:6;T:256;total:262 | iSNV |
| F36 | F36-1 | 926 | M | 0.178 | A:0;G:0;C:39;T:180;total:219 | iSNV |
| F36 | F36-1 | 998 | E | 0.1479 | A:0;G:0;C:190;T:33;total:223 | iSNV |
| F36 | F36-1 | 1218 | E | 1 | A:0;G:0;C:0;T:236;total:236 | SNP |
| F36 | F36-1 | 1428 | E | 0.0991 | A:209;G:23;C:0;T:0;total:232 | iSNV |
| F36 | F36-1 | 2200 | E | 0.0291 | A:0;G:0;C:233;T:7;total:240 | iSNV |
| F36 | F36-1 | 2376 | E | 0.2596 | A:0;G:0;C:77;T:27;total:104 | iSNV |
| F36 | F36-1 | 3353 | NS1 | 0.0222 | A:0;G:0;C:264;T:6;total:270 | iSNV |
| F36 | F36-1 | 3869 | NS2A | 0.8852 | A:0;G:0;C:41;T:316;total:357 | iSNV |
| F36 | F36-1 | 4559 | NS2B | 0.069 | A:0;G:0;C:256;T:19;total:275 | iSNV |
| F36 | F36-1 | 4697 | NS3 | 0.1402 | A:0;G:0;C:39;T:239;total:278 | iSNV |
| F36 | F36-1 | 5252 | NS3 | 0.0226 | A:5;G:216;C:0;T:0;total:221 | iSNV |
| F36 | F36-1 | 5276 | NS3 | 0.0577 | A:212;G:13;C:0;T:0;total:225 | iSNV |
| F36 | F36-1 | 5867 | NS3 | 0.0269 | A:0;G:397;C:0;T:11;total:408 | iSNV |
| F36 | F36-1 | 5952 | NS3 | 0.132 | A:0;G:0;C:368;T:56;total:424 | iSNV |
| F36 | F36-1 | 6753 | NS4A | 0.0291 | A:0;G:200;C:0;T:6;total:206 | iSNV |
| F36 | F36-1 | 7626 | NS4B | 0.0307 | A:11;G:0;C:0;T:347;total:358 | iSNV |
| F36 | F36-1 | 7633 | NS4B | 0.3852 | A:0;G:0;C:233;T:146;total:379 | iSNV |
| F36 | F36-1 | 7673 | NS4B | 0.0213 | A:412;G:9;C:0;T:0;total:421 | iSNV |
| F36 | F36-1 | 9359 | NS5 | 0.1247 | A:0;G:0;C:71;T:498;total:569 | iSNV |
| F36 | F36-1 | 9983 | NS5 | 0.0494 | A:0;G:0;C:173;T:9;total:182 | iSNV |
| F36 | F36-1 | 10259 | NS5 | 0.1439 | A:339;G:57;C:0;T:0;total:396 | iSNV |
| F36 | F36-1 | 10419 | 3'-UTR | 0.0231 | A:0;G:0;C:295;T:7;total:302 | iSNV |
| F36 | F36-1 | 10428 | 3'-UTR | 0.1862 | A:0;G:0;C:249;T:57;total:306 | iSNV |
| F36 | F36-1 | 10447 | 3'-UTR | 0.0228 | A:0;G:0;C:257;T:6;total:263 | iSNV |
| F36 | F36-1 | 10566 | 3'-UTR | 0.1564 | A:0;G:0;C:151;T:28;total:179 | iSNV |
| F36 | F36-10 | 442 | C | 0.0521 | A:0;G:0;C:564;T:31;total:595 | iSNV |
| F36 | F36-10 | 1218 | E | 1 | A:0;G:0;C:0;T:380;total:380 | SNP |
| F36 | F36-10 | 1283 | E | 0.0348 | A:0;G:0;C:443;T:16;total:459 | iSNV |
| F36 | F36-10 | 1551 | E | 0.0898 | A:0;G:23;C:233;T:0;total:256 | iSNV |
| F36 | F36-10 | 3317 | NS1 | 0.0537 | A:0;G:0;C:511;T:29;total:540 | iSNV |
| F36 | F36-10 | 3869 | NS2A | 0.9595 | A:0;G:0;C:22;T:521;total:543 | iSNV |
| F36 | F36-10 | 4697 | NS3 | 0.0229 | A:0;G:0;C:10;T:425;total:435 | iSNV |
| F36 | F36-10 | 5256 | NS3 | 0.4649 | A:0;G:0;C:183;T:159;total:342 | iSNV |
| F36 | F36-10 | 5546 | NS3 | 0.0652 | A:0;G:0;C:487;T:34;total:521 | iSNV |

| | | | | | | |
|---|---|---|---|---|---|---|
| F36 | F36-10 | 5736 | NS3 | 0.5033 | A:312;G:0;C:308;T:0;total:620 | iSNV |
| F36 | F36-10 | 5944 | NS3 | 0.0267 | A:583;G:16;C:0;T:0;total:599 | iSNV |
| F36 | F36-10 | 5952 | NS3 | 0.028 | A:0;G:0;C:590;T:17;total:607 | iSNV |
| F36 | F36-10 | 6206 | NS3 | 0.0326 | A:0;G:0;C:267;T:9;total:276 | iSNV |
| F36 | F36-10 | 6322 | NS3 | 0.0557 | A:0;G:0;C:220;T:13;total:233 | iSNV |
| F36 | F36-10 | 6900 | NS4A | 0.6131 | A:198;G:125;C:0;T:0;total:323 | iSNV |
| F36 | F36-10 | 7264 | NS4A | 0.4451 | A:0;G:0;C:86;T:69;total:155 | iSNV |
| F36 | F36-10 | 7633 | NS4B | 0.1639 | A:0;G:0;C:459;T:90;total:549 | iSNV |
| F36 | F36-10 | 9359 | NS5 | 0.0236 | A:0;G:0;C:18;T:744;total:762 | iSNV |
| F36 | F36-10 | 10259 | NS5 | 0.0289 | A:570;G:17;C:0;T:0;total:587 | iSNV |
| F36 | F36-10 | 10428 | 3'-UTR | 0.7143 | A:0;G:0;C:116;T:290;total:406 | iSNV |
| F36 | F36-10 | 10566 | 3'-UTR | 0.0286 | A:0;G:0;C:271;T:8;total:279 | iSNV |
| F36 | F36-11 | 998 | E | 0.0262 | A:0;G:0;C:445;T:12;total:457 | iSNV |
| F36 | F36-11 | 1218 | E | 1 | A:0;G:0;C:0;T:556;total:556 | SNP |
| F36 | F36-11 | 1514 | E | 0.0459 | A:436;G:0;C:21;T:0;total:457 | iSNV |
| F36 | F36-11 | 1797 | E | 0.8182 | A:495;G:18;C:0;T:114;total:627 | iSNV |
| F36 | F36-11 | 2618 | NS1 | 0.0785 | A:0;G:0;C:716;T:61;total:777 | iSNV |
| F36 | F36-11 | 3495 | NS1 | 0.0463 | A:0;G:0;C:288;T:14;total:302 | iSNV |
| F36 | F36-11 | 3869 | NS2A | 0.9841 | A:0;G:0;C:14;T:864;total:878 | SNP |
| F36 | F36-11 | 6509 | NS4A | 0.0224 | A:0;G:0;C:13;T:566;total:579 | iSNV |
| F36 | F36-11 | 7633 | NS4B | 0.7353 | A:0;G:0;C:225;T:625;total:850 | iSNV |
| F36 | F36-11 | 8659 | NS5 | 0.0224 | A:479;G:11;C:0;T:0;total:490 | iSNV |
| F36 | F36-11 | 9359 | NS5 | 0.02 | A:0;G:0;C:23;T:1122;total:1145 | iSNV |
| F36 | F36-11 | 9880 | NS5 | 0.7543 | A:307;G:100;C:0;T:0;total:407 | iSNV |
| F36 | F36-11 | 10428 | 3'-UTR | 0.0378 | A:0;G:0;C:610;T:24;total:634 | iSNV |
| F36 | F36-11 | 10589 | 3'-UTR | 0.7807 | A:0;G:363;C:0;T:102;total:465 | iSNV |
| F36 | F36-12 | 332 | C | 0.0507 | A:748;G:40;C:0;T:0;total:788 | iSNV |
| F36 | F36-12 | 353 | C | 0.2314 | A:591;G:178;C:0;T:0;total:769 | iSNV |
| F36 | F36-12 | 645 | M | 0.2685 | A:354;G:130;C:0;T:0;total:484 | iSNV |
| F36 | F36-12 | 869 | M | 0.1183 | A:0;G:60;C:0;T:447;total:507 | iSNV |
| F36 | F36-12 | 998 | E | 0.4117 | A:0;G:0;C:230;T:161;total:391 | iSNV |
| F36 | F36-12 | 1117 | E | 0.3176 | A:290;G:135;C:0;T:0;total:425 | iSNV |
| F36 | F36-12 | 1218 | E | 0.6733 | A:0;G:0;C:148;T:305;total:453 | iSNV |
| F36 | F36-12 | 1337 | E | 0.0662 | A:0;G:0;C:479;T:34;total:513 | iSNV |
| F36 | F36-12 | 2078 | E | 0.0352 | A:0;G:0;C:274;T:10;total:284 | iSNV |
| F36 | F36-12 | 2277 | E | 0.2641 | A:0;G:0;C:98;T:273;total:371 | iSNV |
| F36 | F36-12 | 3869 | NS2A | 0.6626 | A:0;G:1;C:221;T:433;total:655 | iSNV |
| F36 | F36-12 | 4697 | NS3 | 0.0842 | A:0;G:0;C:45;T:489;total:534 | iSNV |
| F36 | F36-12 | 4974 | NS3 | 0.02 | A:10;G:490;C:0;T:0;total:500 | iSNV |
| F36 | F36-12 | 5558 | NS3 | 0.0328 | A:17;G:501;C:0;T:0;total:518 | iSNV |
| F36 | F36-12 | 5747 | NS3 | 0.0482 | A:32;G:631;C:0;T:0;total:663 | iSNV |
| F36 | F36-12 | 5780 | NS3 | 0.081 | A:0;G:0;C:58;T:658;total:716 | iSNV |
| F36 | F36-12 | 5952 | NS3 | 0.0752 | A:0;G:0;C:688;T:56;total:744 | iSNV |
| F36 | F36-12 | 7176 | NS4A | 0.0509 | A:205;G:11;C:0;T:0;total:216 | iSNV |
| F36 | F36-12 | 7178 | NS4A | 0.0504 | A:0;G:0;C:207;T:11;total:218 | iSNV |
| F36 | F36-12 | 7561 | NS4B | 0.0523 | A:0;G:1;C:25;T:452;total:478 | iSNV |
| F36 | F36-12 | 7626 | NS4B | 0.0251 | A:0;G:15;C:0;T:582;total:597 | iSNV |
| F36 | F36-12 | 7633 | NS4B | 0.3217 | A:0;G:0;C:411;T:195;total:606 | iSNV |
| F36 | F36-12 | 8090 | NS5 | 0.0377 | A:21;G:535;C:0;T:0;total:556 | iSNV |
| F36 | F36-12 | 9359 | NS5 | 0.1121 | A:0;G:0;C:92;T:728;total:820 | iSNV |
| F36 | F36-12 | 9491 | NS5 | 0.0705 | A:382;G:29;C:0;T:0;total:411 | iSNV |
| F36 | F36-12 | 10259 | NS5 | 0.3599 | A:376;G:212;C:0;T:1;total:589 | iSNV |
| F36 | F36-12 | 10428 | 3'-UTR | 0.2452 | A:0;G:0;C:360;T:117;total:477 | iSNV |
| F36 | F36-12 | 10447 | 3'-UTR | 0.3029 | A:0;G:0;C:306;T:133;total:439 | iSNV |
| F36 | F36-12 | 10452 | 3'-UTR | 0.0205 | A:430;G:0;C:0;T:9;total:439 | iSNV |
| F36 | F36-12 | 10566 | 3'-UTR | 0.0911 | A:0;G:0;C:299;T:30;total:329 | iSNV |
| F36 | F36-12 | 10589 | 3'-UTR | 0.0284 | A:0;G:10;C:0;T:341;total:351 | iSNV |
| F36 | F36-12 | 10592 | 3'-UTR | 0.0273 | A:0;G:0;C:355;T:10;total:365 | iSNV |
| F36 | F36-12 | 10782 | 3'-UTR | 0.0298 | A:0;G:0;C:325;T:10;total:335 | iSNV |
| F36 | F36-13 | 395 | C | 0.736 | A:452;G:1259;C:0;T:1;total:1712 | iSNV |
| F36 | F36-13 | 530 | M | 0.1468 | A:0;G:0;C:761;T:131;total:892 | iSNV |
| F36 | F36-13 | 854 | M | 0.0431 | A:0;G:0;C:576;T:26;total:602 | iSNV |
| F36 | F36-13 | 978 | E | 0.0232 | A:0;G:0;C:12;T:504;total:516 | iSNV |
| F36 | F36-13 | 998 | E | 0.0202 | A:0;G:0;C:483;T:10;total:493 | iSNV |
| F36 | F36-13 | 1218 | E | 0.9892 | A:0;G:0;C:7;T:637;total:644 | SNP |
| F36 | F36-13 | 1413 | E | 0.7749 | A:165;G:568;C:0;T:0;total:733 | iSNV |
| F36 | F36-13 | 1708 | E | 0.1293 | A:67;G:451;C:0;T:0;total:518 | iSNV |
| F36 | F36-13 | 1892 | E | 0.1061 | A:362;G:0;C:0;T:43;total:405 | iSNV |
| F36 | F36-13 | 3869 | NS2A | 0.9886 | A:0;G:0;C:12;T:1032;total:1044 | SNP |
| F36 | F36-13 | 4052 | NS2A | 0.1017 | A:0;G:0;C:521;T:59;total:580 | iSNV |
| F36 | F36-13 | 5600 | NS3 | 0.025 | A:0;G:0;C:819;T:21;total:840 | iSNV |
| F36 | F36-13 | 6967 | NS4A | 0.0265 | A:14;G:513;C:0;T:0;total:527 | iSNV |
| F36 | F36-13 | 6970 | NS4A | 0.1477 | A:444;G:0;C:77;T:0;total:521 | iSNV |
| F36 | F36-13 | 6981 | NS4A | 0.0615 | A:0;G:0;C:32;T:488;total:520 | iSNV |
| F36 | F36-13 | 7011 | NS4A | 0.0649 | A:0;G:0;C:504;T:35;total:539 | iSNV |
| F36 | F36-13 | 7146 | NS4A | 0.0306 | A:0;G:0;C:11;T:348;total:359 | iSNV |
| F36 | F36-13 | 7176 | NS4A | 0.1004 | A:187;G:21;C:0;T:1;total:209 | iSNV |
| F36 | F36-13 | 7527 | NS4B | 0.1167 | A:613;G:81;C:0;T:0;total:694 | iSNV |
| F36 | F36-13 | 7627 | NS4B | 0.1095 | A:0;G:106;C:862;T:0;total:968 | iSNV |
| F36 | F36-13 | 7633 | NS4B | 0.385 | A:0;G:0;C:599;T:375;total:974 | iSNV |
| F36 | F36-13 | 9161 | NS5 | 0.0263 | A:0;G:1;C:24;T:885;total:910 | iSNV |
| F36 | F36-13 | 10259 | NS5 | 0.0224 | A:697;G:16;C:0;T:0;total:713 | iSNV |

| | | | | | | |
|---|---|---|---|---|---|---|
| F36 | F36-13 | 10330 | NS5 | 0.0309 | A:0;G:0;C:20;T:627;total:647 | iSNV |
| F36 | F36-13 | 10376 | NS5 | 0.7305 | A:399;G:148;C:0;T:2;total:549 | iSNV |
| F36 | F36-13 | 10428 | 3'-UTR | 0.3101 | A:0;G:0;C:367;T:165;total:532 | iSNV |
| F36 | F36-13 | 10588 | 3'-UTR | 0.0421 | A:0;G:659;C:29;T:0;total:688 | iSNV |
| F36 | F36-14 | 506 | M | 0.0553 | A:0;G:0;C:42;T:717;total:759 | iSNV |
| F36 | F36-14 | 836 | M | 0.0294 | A:0;G:0;C:13;T:428;total:441 | iSNV |
| F36 | F36-14 | 889 | M | 0.0205 | A:0;G:0;C:382;T:8;total:390 | iSNV |
| F36 | F36-14 | 998 | E | 0.4185 | A:0;G:0;C:207;T:149;total:356 | iSNV |
| F36 | F36-14 | 1057 | E | 0.0442 | A:12;G:259;C:0;T:0;total:271 | iSNV |
| F36 | F36-14 | 1117 | E | 0.316 | A:277;G:128;C:0;T:0;total:405 | iSNV |
| F36 | F36-14 | 1218 | E | 0.9812 | A:0;G:0;C:8;T:417;total:425 | SNP |
| F36 | F36-14 | 1512 | E | 0.1517 | A:313;G:56;C:0;T:0;total:369 | iSNV |
| F36 | F36-14 | 2274 | E | 0.0479 | A:0;G:417;C:0;T:21;total:438 | iSNV |
| F36 | F36-14 | 2489 | NS1 | 0.0348 | A:0;G:0;C:9;T:249;total:258 | iSNV |
| F36 | F36-14 | 2664 | NS1 | 0.043 | A:0;G:0;C:645;T:29;total:674 | iSNV |
| F36 | F36-14 | 3869 | NS2A | 0.6485 | A:0;G:0;C:231;T:426;total:657 | iSNV |
| F36 | F36-14 | 4124 | NS2A | 0.0671 | A:236;G:17;C:0;T:0;total:253 | iSNV |
| F36 | F36-14 | 4187 | NS2A | 0.0392 | A:196;G:8;C:0;T:0;total:204 | iSNV |
| F36 | F36-14 | 4697 | NS3 | 0.3977 | A:0;G:0;C:210;T:318;total:528 | iSNV |
| F36 | F36-14 | 5516 | NS3 | 0.0334 | A:0;G:0;C:606;T:21;total:627 | iSNV |
| F36 | F36-14 | 5558 | NS3 | 0.2636 | A:145;G:405;C:0;T:0;total:550 | iSNV |
| F36 | F36-14 | 5952 | NS3 | 0.3576 | A:0;G:0;C:476;T:265;total:741 | iSNV |
| F36 | F36-14 | 7561 | NS4B | 0.0574 | A:0;G:0;C:31;T:509;total:540 | iSNV |
| F36 | F36-14 | 7633 | NS4B | 0.275 | A:0;G:0;C:485;T:184;total:669 | iSNV |
| F36 | F36-14 | 8683 | NS5 | 0.0316 | A:0;G:0;C:429;T:14;total:443 | iSNV |
| F36 | F36-14 | 9359 | NS5 | 0.4393 | A:0;G:0;C:413;T:527;total:940 | iSNV |
| F36 | F36-14 | 9370 | NS5 | 0.3499 | A:0;G:330;C:0;T:613;total:943 | iSNV |
| F36 | F36-14 | 10259 | NS5 | 0.4056 | A:378;G:258;C:0;T:0;total:636 | iSNV |
| F36 | F36-14 | 10355 | NS5 | 0.0241 | A:647;G:16;C:0;T:0;total:663 | iSNV |
| F36 | F36-14 | 10428 | 3'-UTR | 0.1185 | A:0;G:0;C:409;T:55;total:464 | iSNV |
| F36 | F36-14 | 10435 | 3'-UTR | 0.0302 | A:417;G:13;C:0;T:0;total:430 | iSNV |
| F36 | F36-14 | 10447 | 3'-UTR | 0.0313 | A:0;G:0;C:371;T:12;total:383 | iSNV |
| F36 | F36-14 | 10566 | 3'-UTR | 0.3971 | A:0;G:0;C:167;T:110;total:277 | iSNV |
| F36 | F36-15 | 506 | M | 0.1743 | A:0;G:0;C:102;T:483;total:585 | iSNV |
| F36 | F36-15 | 569 | M | 0.0298 | A:11;G:358;C:0;T:0;total:369 | iSNV |
| F36 | F36-15 | 998 | E | 0.4912 | A:0;G:0;C:146;T:141;total:287 | iSNV |
| F36 | F36-15 | 1117 | E | 0.2172 | A:226;G:63;C:0;T:1;total:290 | iSNV |
| F36 | F36-15 | 1218 | E | 0.9971 | A:0;G:0;C:1;T:337;total:338 | SNP |
| F36 | F36-15 | 1508 | E | 0.052 | A:0;G:0;C:255;T:14;total:269 | iSNV |
| F36 | F36-15 | 1512 | E | 0.104 | A:241;G:28;C:0;T:0;total:269 | iSNV |
| F36 | F36-15 | 2274 | E | 0.1089 | A:0;G:227;C:2;T:28;total:257 | iSNV |
| F36 | F36-15 | 2282 | E | 0.0275 | A:0;G:0;C:247;T:7;total:254 | iSNV |
| F36 | F36-15 | 2664 | NS1 | 0.0437 | A:0;G:0;C:524;T:24;total:548 | iSNV |
| F36 | F36-15 | 2705 | NS1 | 0.1046 | A:0;G:0;C:522;T:61;total:583 | iSNV |
| F36 | F36-15 | 3869 | NS2A | 0.5944 | A:0;G:0;C:187;T:274;total:461 | iSNV |
| F36 | F36-15 | 4124 | NS2A | 0.0409 | A:211;G:9;C:0;T:0;total:220 | iSNV |
| F36 | F36-15 | 4697 | NS3 | 0.4227 | A:0;G:0;C:175;T:239;total:414 | iSNV |
| F36 | F36-15 | 4974 | NS3 | 0.0267 | A:11;G:400;C:0;T:0;total:411 | iSNV |
| F36 | F36-15 | 5558 | NS3 | 0.2152 | A:110;G:401;C:0;T:0;total:511 | iSNV |
| F36 | F36-15 | 5952 | NS3 | 0.4176 | A:0;G:0;C:343;T:246;total:589 | iSNV |
| F36 | F36-15 | 6089 | NS3 | 0.0207 | A:0;G:0;C:331;T:7;total:338 | iSNV |
| F36 | F36-15 | 7561 | NS4B | 0.0203 | A:0;G:0;C:8;T:385;total:393 | iSNV |
| F36 | F36-15 | 7633 | NS4B | 0.3702 | A:0;G:0;C:301;T:177;total:478 | iSNV |
| F36 | F36-15 | 8195 | NS5 | 0.0271 | A:0;G:0;C:359;T:10;total:369 | iSNV |
| F36 | F36-15 | 9359 | NS5 | 0.418 | A:0;G:0;C:306;T:426;total:732 | iSNV |
| F36 | F36-15 | 9370 | NS5 | 0.2684 | A:0;G:196;C:0;T:534;total:730 | iSNV |
| F36 | F36-15 | 9634 | NS5 | 0.0226 | A:0;G:0;C:5;T:216;total:221 | iSNV |
| F36 | F36-15 | 10259 | NS5 | 0.4086 | A:288;G:199;C:0;T:0;total:487 | iSNV |
| F36 | F36-15 | 10428 | 3'-UTR | 0.1645 | A:0;G:0;C:335;T:66;total:401 | iSNV |
| F36 | F36-15 | 10447 | 3'-UTR | 0.0415 | A:0;G:0;C:346;T:15;total:361 | iSNV |
| F36 | F36-15 | 10566 | 3'-UTR | 0.3657 | A:0;G:0;C:163;T:94;total:257 | iSNV |
| F36 | F36-16 | 293 | C | 0.0253 | A:423;G:11;C:0;T:0;total:434 | iSNV |
| F36 | F36-16 | 469 | C | 0.0637 | A:0;G:0;C:499;T:34;total:533 | iSNV |
| F36 | F36-16 | 803 | M | 0.0377 | A:0;G:153;C:0;T:6;total:159 | iSNV |
| F36 | F36-16 | 998 | E | 0.2336 | A:0;G:0;C:164;T:50;total:214 | iSNV |
| F36 | F36-16 | 1218 | E | 1 | A:0;G:0;C:0;T:232;total:232 | SNP |
| F36 | F36-16 | 1428 | E | 0.0447 | A:235;G:11;C:0;T:0;total:246 | iSNV |
| F36 | F36-16 | 1447 | E | 0.0255 | A:6;G:0;C:229;T:0;total:235 | iSNV |
| F36 | F36-16 | 1672 | E | 0.0571 | A:0;G:0;C:165;T:10;total:175 | iSNV |
| F36 | F36-16 | 2274 | E | 0.0409 | A:0;G:164;C:0;T:7;total:171 | iSNV |
| F36 | F36-16 | 3396 | NS1 | 0.055 | A:0;G:13;C:0;T:223;total:236 | iSNV |
| F36 | F36-16 | 3869 | NS2A | 0.741 | A:0;G:0;C:93;T:266;total:359 | iSNV |
| F36 | F36-16 | 3965 | NS2A | 0.0763 | A:0;G:0;C:230;T:19;total:249 | iSNV |
| F36 | F36-16 | 4697 | NS3 | 0.2338 | A:0;G:0;C:65;T:213;total:278 | iSNV |
| F36 | F36-16 | 5312 | NS3 | 0.0641 | A:19;G:0;C:0;T:277;total:296 | iSNV |
| F36 | F36-16 | 5736 | NS3 | 0.0473 | A:19;G:0;C:382;T:0;total:401 | iSNV |
| F36 | F36-16 | 5753 | NS3 | 0.0377 | A:14;G:357;C:0;T:0;total:371 | iSNV |
| F36 | F36-16 | 5952 | NS3 | 0.1856 | A:0;G:0;C:351;T:80;total:431 | iSNV |
| F36 | F36-16 | 6122 | NS3 | 0.0323 | A:8;G:239;C:0;T:0;total:247 | iSNV |
| F36 | F36-16 | 6900 | NS4A | 0.028 | A:6;G:207;C:0;T:1;total:214 | iSNV |
| F36 | F36-16 | 7528 | NS4B | 0.0732 | A:0;G:0;C:253;T:20;total:273 | iSNV |
| F36 | F36-16 | 7633 | NS4B | 0.2804 | A:0;G:0;C:295;T:115;total:410 | iSNV |

| | | | | | | |
|---|---|---|---|---|---|---|
| F36 | F36-16 | 7735 | NS5 | 0.0402 | A:16;G:382;C:0;T:0;total:398 | iSNV |
| F36 | F36-16 | 8396 | NS5 | 0.0234 | A:499;G:12;C:0;T:0;total:511 | iSNV |
| F36 | F36-16 | 8693 | NS5 | 0.0272 | A:0;G:0;C:250;T:7;total:257 | iSNV |
| F36 | F36-16 | 9359 | NS5 | 0.2688 | A:0;G:0;C:164;T:446;total:610 | iSNV |
| F36 | F36-16 | 9818 | NS5 | 0.1009 | A:0;G:0;C:21;T:187;total:208 | iSNV |
| F36 | F36-16 | 10259 | NS5 | 0.2247 | A:345;G:100;C:0;T:0;total:445 | iSNV |
| F36 | F36-16 | 10351 | NS5 | 0.0255 | A:12;G:458;C:0;T:0;total:470 | iSNV |
| F36 | F36-16 | 10368 | NS5 | 0.0386 | A:18;G:0;C:0;T:448;total:466 | iSNV |
| F36 | F36-16 | 10428 | 3'-UTR | 0.3187 | A:0;G:0;C:203;T:95;total:298 | iSNV |
| F36 | F36-16 | 10566 | 3'-UTR | 0.2548 | A:0;G:0;C:155;T:53;total:208 | iSNV |
| F36 | F36-16 | 10718 | 3'-UTR | 0.064 | A:0;G:0;C:190;T:13;total:203 | iSNV |
| F36 | F36-17 | 291 | C | 0.0416 | A:19;G:0;C:0;T:437;total:456 | iSNV |
| F36 | F36-17 | 444 | C | 0.023 | A:508;G:0;C:0;T:12;total:520 | iSNV |
| F36 | F36-17 | 645 | M | 0.0556 | A:390;G:23;C:0;T:0;total:413 | iSNV |
| F36 | F36-17 | 998 | E | 0.0737 | A:0;G:0;C:226;T:18;total:244 | iSNV |
| F36 | F36-17 | 1218 | E | 0.9885 | A:0;G:0;C:3;T:256;total:259 | SNP |
| F36 | F36-17 | 1413 | E | 0.0582 | A:356;G:22;C:0;T:0;total:378 | iSNV |
| F36 | F36-17 | 1428 | E | 0.0537 | A:317;G:18;C:0;T:0;total:335 | iSNV |
| F36 | F36-17 | 1512 | E | 0.0561 | A:179;G:11;C:6;T:0;total:196 | iSNV |
| F36 | F36-17 | 1797 | E | 0.0247 | A:8;G:1;C:1;T:313;total:323 | iSNV |
| F36 | F36-17 | 2960 | NS1 | 0.0351 | A:0;G:0;C:439;T:16;total:455 | iSNV |
| F36 | F36-17 | 3317 | NS1 | 0.2385 | A:0;G:0;C:316;T:99;total:415 | iSNV |
| F36 | F36-17 | 3341 | NS1 | 0.0506 | A:356;G:19;C:0;T:0;total:375 | iSNV |
| F36 | F36-17 | 3869 | NS2A | 0.9262 | A:0;G:0;C:31;T:389;total:420 | iSNV |
| F36 | F36-17 | 4391 | NS2B | 0.0915 | A:0;G:0;C:258;T:26;total:284 | iSNV |
| F36 | F36-17 | 4697 | NS3 | 0.0555 | A:0;G:0;C:16;T:272;total:288 | iSNV |
| F36 | F36-17 | 4974 | NS3 | 0.0302 | A:10;G:321;C:0;T:0;total:331 | iSNV |
| F36 | F36-17 | 5093 | NS3 | 0.0376 | A:0;G:0;C:307;T:12;total:319 | iSNV |
| F36 | F36-17 | 5607 | NS3 | 0.0203 | A:0;G:0;C:386;T:8;total:394 | iSNV |
| F36 | F36-17 | 5952 | NS3 | 0.0696 | A:0;G:0;C:401;T:30;total:431 | iSNV |
| F36 | F36-17 | 6322 | NS3 | 0.1592 | A:0;G:0;C:132;T:25;total:157 | iSNV |
| F36 | F36-17 | 6753 | NS4A | 0.049 | A:1;G:251;C:0;T:13;total:265 | iSNV |
| F36 | F36-17 | 7109 | NS4A | 0.0625 | A:0;G:0;C:165;T:11;total:176 | iSNV |
| F36 | F36-17 | 7633 | NS4B | 0.3043 | A:0;G:0;C:288;T:126;total:414 | iSNV |
| F36 | F36-17 | 8430 | NS5 | 0.03 | A:0;G:0;C:419;T:13;total:432 | iSNV |
| F36 | F36-17 | 9359 | NS5 | 0.0945 | A:0;G:0;C:56;T:536;total:592 | iSNV |
| F36 | F36-17 | 10259 | NS5 | 0.0759 | A:426;G:35;C:0;T:0;total:461 | iSNV |
| F36 | F36-17 | 10364 | NS5 | 0.055 | A:0;G:0;C:446;T:26;total:472 | iSNV |
| F36 | F36-17 | 10376 | NS5 | 0.0349 | A:16;G:442;C:0;T:0;total:458 | iSNV |
| F36 | F36-17 | 10428 | 3'-UTR | 0.1977 | A:0;G:0;C:284;T:70;total:354 | iSNV |
| F36 | F36-17 | 10566 | 3'-UTR | 0.0707 | A:0;G:0;C:197;T:15;total:212 | iSNV |
| F36 | F36-18 | 948 | M | 0.1018 | A:0;G:0;C:335;T:38;total:373 | iSNV |
| F36 | F36-18 | 998 | E | 0.0769 | A:0;G:0;C:336;T:28;total:364 | iSNV |
| F36 | F36-18 | 1218 | E | 1 | A:0;G:0;C:0;T:543;total:543 | SNP |
| F36 | F36-18 | 1413 | E | 0.6561 | A:195;G:372;C:0;T:0;total:567 | iSNV |
| F36 | F36-18 | 1512 | E | 0.445 | A:202;G:0;C:162;T:0;total:364 | iSNV |
| F36 | F36-18 | 1797 | E | 0.234 | A:0;G:150;C:0;T:491;total:641 | iSNV |
| F36 | F36-18 | 3746 | NS2A | 0.0536 | A:247;G:14;C:0;T:0;total:261 | iSNV |
| F36 | F36-18 | 3869 | NS2A | 0.9637 | A:0;G:0;C:24;T:636;total:660 | iSNV |
| F36 | F36-18 | 4091 | NS2A | 0.3048 | A:114;G:260;C:0;T:0;total:374 | iSNV |
| F36 | F36-18 | 4697 | NS3 | 0.0627 | A:0;G:0;C:36;T:538;total:574 | iSNV |
| F36 | F36-18 | 4989 | NS3 | 0.0216 | A:0;G:0;C:677;T:15;total:692 | iSNV |
| F36 | F36-18 | 5072 | NS3 | 0.0735 | A:30;G:378;C:0;T:0;total:408 | iSNV |
| F36 | F36-18 | 5327 | NS3 | 0.063 | A:401;G:0;C:0;T:27;total:428 | iSNV |
| F36 | F36-18 | 5952 | NS3 | 0.0742 | A:0;G:0;C:798;T:64;total:862 | iSNV |
| F36 | F36-18 | 6969 | NS4A | 0.0317 | A:397;G:13;C:0;T:0;total:410 | iSNV |
| F36 | F36-18 | 7633 | NS4B | 0.5923 | A:0;G:0;C:307;T:446;total:753 | iSNV |
| F36 | F36-18 | 8430 | NS5 | 0.6009 | A:0;G:0;C:289;T:435;total:724 | iSNV |
| F36 | F36-18 | 9359 | NS5 | 0.0764 | A:0;G:0;C:69;T:833;total:902 | iSNV |
| F36 | F36-18 | 9764 | NS5 | 0.2178 | A:0;G:0;C:341;T:95;total:436 | iSNV |
| F36 | F36-18 | 10259 | NS5 | 0.0712 | A:730;G:56;C:0;T:0;total:786 | iSNV |
| F36 | F36-18 | 10295 | NS5 | 0.0525 | A:0;G:0;C:721;T:40;total:761 | iSNV |
| F36 | F36-18 | 10376 | NS5 | 0.5787 | A:309;G:225;C:0;T:0;total:534 | iSNV |
| F36 | F36-18 | 10428 | 3'-UTR | 0.0944 | A:0;G:0;C:374;T:39;total:413 | iSNV |
| F36 | F36-18 | 10447 | 3'-UTR | 0.0271 | A:0;G:0;C:394;T:11;total:405 | iSNV |
| F36 | F36-18 | 10566 | 3'-UTR | 0.0854 | A:0;G:0;C:364;T:34;total:398 | iSNV |
| F36 | F36-19 | 353 | C | 0.0377 | A:1222;G:48;C:0;T:0;total:1270 | iSNV |
| F36 | F36-19 | 515 | M | 0.0737 | A:0;G:0;C:1018;T:81;total:1099 | iSNV |
| F36 | F36-19 | 645 | M | 0.0448 | A:873;G:41;C:0;T:0;total:914 | iSNV |
| F36 | F36-19 | 869 | M | 0.2002 | A:151;G:0;C:0;T:603;total:754 | iSNV |
| F36 | F36-19 | 998 | E | 0.1194 | A:0;G:0;C:560;T:76;total:636 | iSNV |
| F36 | F36-19 | 1218 | E | 0.9589 | A:1;G:0;C:28;T:652;total:681 | iSNV |
| F36 | F36-19 | 1382 | E | 0.1628 | A:0;G:0;C:699;T:136;total:835 | iSNV |
| F36 | F36-19 | 1413 | E | 0.0309 | A:845;G:27;C:0;T:0;total:872 | iSNV |
| F36 | F36-19 | 1428 | E | 0.0244 | A:799;G:20;C:0;T:0;total:819 | iSNV |
| F36 | F36-19 | 1430 | E | 0.3846 | A:0;G:0;C:496;T:310;total:806 | iSNV |
| F36 | F36-19 | 1797 | E | 0.1052 | A:80;G:0;C:0;T:680;total:760 | iSNV |
| F36 | F36-19 | 1911 | E | 0.0305 | A:349;G:11;C:0;T:0;total:360 | iSNV |
| F36 | F36-19 | 3278 | NS1 | 0.0302 | A:0;G:0;C:29;T:931;total:960 | iSNV |
| F36 | F36-19 | 3869 | NS2A | 0.8777 | A:0;G:0;C:123;T:882;total:1005 | iSNV |
| F36 | F36-19 | 4122 | NS2A | 0.0562 | A:0;G:386;C:0;T:23;total:409 | iSNV |
| F36 | F36-19 | 4538 | NS2B | 0.1095 | A:0;G:0;C:699;T:86;total:785 | iSNV |

| | | | | | | |
|---|---|---|---|---|---|---|
| F36 | F36-19 | 4697 | NS3 | 0.0883 | A:0;G:0;C:73;T:753;total:826 | iSNV |
| F36 | F36-19 | 5313 | NS3 | 0.0244 | A:0;G:0;C:18;T:719;total:737 | iSNV |
| F36 | F36-19 | 5813 | NS3 | 0.0242 | A:0;G:0;C:1248;T:31;total:1279 | iSNV |
| F36 | F36-19 | 5927 | NS3 | 0.0244 | A:0;G:0;C:1077;T:27;total:1104 | iSNV |
| F36 | F36-19 | 5952 | NS3 | 0.0757 | A:0;G:0;C:1134;T:93;total:1227 | iSNV |
| F36 | F36-19 | 6203 | NS3 | 0.0311 | A:590;G:19;C:0;T:0;total:609 | iSNV |
| F36 | F36-19 | 6413 | NS3 | 0.072 | A:0;G:0;C:49;T:631;total:680 | iSNV |
| F36 | F36-19 | 7421 | NS4B | 0.0266 | A:438;G:12;C:0;T:0;total:450 | iSNV |
| F36 | F36-19 | 7633 | NS4B | 0.504 | A:0;G:0;C:506;T:514;total:1020 | iSNV |
| F36 | F36-19 | 8702 | NS5 | 0.0601 | A:0;G:0;C:719;T:46;total:765 | iSNV |
| F36 | F36-19 | 8897 | NS5 | 0.0355 | A:0;G:0;C:1085;T:40;total:1125 | iSNV |
| F36 | F36-19 | 9356 | NS5 | 0.0301 | A:0;G:0;C:1381;T:43;total:1424 | iSNV |
| F36 | F36-19 | 9359 | NS5 | 0.0809 | A:0;G:0;C:115;T:1306;total:1421 | iSNV |
| F36 | F36-19 | 9452 | NS5 | 0.0322 | A:41;G:1230;C:0;T:0;total:1271 | iSNV |
| F36 | F36-19 | 9557 | NS5 | 0.0222 | A:15;G:660;C:0;T:0;total:675 | iSNV |
| F36 | F36-19 | 9818 | NS5 | 0.0837 | A:0;G:0;C:46;T:503;total:549 | iSNV |
| F36 | F36-19 | 10022 | NS5 | 0.0413 | A:0;G:0;C:27;T:626;total:653 | iSNV |
| F36 | F36-19 | 10259 | NS5 | 0.1079 | A:917;G:111;C:0;T:0;total:1028 | iSNV |
| F36 | F36-19 | 10358 | NS5 | 0.212 | A:0;G:0;C:810;T:218;total:1028 | iSNV |
| F36 | F36-19 | 10419 | 3'-UTR | 0.0231 | A:0;G:0;C:802;T:19;total:821 | iSNV |
| F36 | F36-19 | 10428 | 3'-UTR | 0.2042 | A:0;G:0;C:643;T:165;total:808 | iSNV |
| F36 | F36-19 | 10447 | 3'-UTR | 0.0725 | A:0;G:0;C:665;T:52;total:717 | iSNV |
| F36 | F36-19 | 10566 | 3'-UTR | 0.0669 | A:0;G:0;C:502;T:36;total:538 | iSNV |
| F36 | F36-19 | 10707 | 3'-UTR | 0.0441 | A:520;G:24;C:0;T:0;total:544 | iSNV |
| F36 | F36-2 | 434 | C | 0.2443 | A:109;G:337;C:0;T:0;total:446 | iSNV |
| F36 | F36-2 | 568 | M | 0.0257 | A:303;G:0;C:0;T:8;total:311 | iSNV |
| F36 | F36-2 | 762 | M | 0.0222 | A:10;G:439;C:0;T:0;total:449 | iSNV |
| F36 | F36-2 | 998 | E | 0.0909 | A:0;G:0;C:230;T:23;total:253 | iSNV |
| F36 | F36-2 | 1166 | E | 0.0865 | A:0;G:0;C:27;T:285;total:312 | iSNV |
| F36 | F36-2 | 1218 | E | 1 | A:0;G:0;C:0;T:281;total:281 | SNP |
| F36 | F36-2 | 1413 | E | 0.4258 | A:213;G:158;C:0;T:0;total:371 | iSNV |
| F36 | F36-2 | 1430 | E | 0.2643 | A:0;G:0;C:256;T:92;total:348 | iSNV |
| F36 | F36-2 | 2274 | E | 0.2304 | A:0;G:187;C:0;T:56;total:243 | iSNV |
| F36 | F36-2 | 2712 | NS1 | 0.0201 | A:0;G:0;C:486;T:10;total:496 | iSNV |
| F36 | F36-2 | 3737 | NS2A | 0.0489 | A:0;G:0;C:175;T:9;total:184 | iSNV |
| F36 | F36-2 | 3869 | NS2A | 0.938 | A:0;G:0;C:26;T:393;total:419 | iSNV |
| F36 | F36-2 | 4160 | NS2A | 0.0675 | A:0;G:0;C:138;T:10;total:148 | iSNV |
| F36 | F36-2 | 4697 | NS3 | 0.0418 | A:0;G:0;C:16;T:366;total:382 | iSNV |
| F36 | F36-2 | 4889 | NS3 | 0.0241 | A:0;G:0;C:485;T:12;total:497 | iSNV |
| F36 | F36-2 | 5911 | NS3 | 0.0219 | A:0;G:0;C:10;T:446;total:456 | iSNV |
| F36 | F36-2 | 5952 | NS3 | 0.0674 | A:0;G:0;C:484;T:35;total:519 | iSNV |
| F36 | F36-2 | 6969 | NS4A | 0.1111 | A:192;G:24;C:0;T:0;total:216 | iSNV |
| F36 | F36-2 | 6996 | NS4A | 0.109 | A:23;G:0;C:0;T:188;total:211 | iSNV |
| F36 | F36-2 | 7633 | NS4B | 0.352 | A:0;G:0;C:265;T:144;total:409 | iSNV |
| F36 | F36-2 | 7963 | NS5 | 0.0824 | A:512;G:46;C:0;T:0;total:558 | iSNV |
| F36 | F36-2 | 8249 | NS5 | 0.0524 | A:0;G:0;C:19;T:343;total:362 | iSNV |
| F36 | F36-2 | 9350 | NS5 | 0.0302 | A:0;G:0;C:545;T:17;total:562 | iSNV |
| F36 | F36-2 | 9359 | NS5 | 0.0661 | A:0;G:0;C:37;T:522;total:559 | iSNV |
| F36 | F36-2 | 10259 | NS5 | 0.0769 | A:360;G:30;C:0;T:0;total:390 | iSNV |
| F36 | F36-2 | 10376 | NS5 | 0.4018 | A:133;G:198;C:0;T:0;total:331 | iSNV |
| F36 | F36-2 | 10428 | 3'-UTR | 0.2335 | A:0;G:0;C:233;T:71;total:304 | iSNV |
| F36 | F36-2 | 10447 | 3'-UTR | 0.0269 | A:0;G:0;C:253;T:7;total:260 | iSNV |
| F36 | F36-2 | 10566 | 3'-UTR | 0.0604 | A:0;G:0;C:202;T:13;total:215 | iSNV |
| F36 | F36-20 | 456 | C | 0.0229 | A:16;G:681;C:0;T:0;total:697 | iSNV |
| F36 | F36-20 | 998 | E | 0.3952 | A:0;G:0;C:205;T:134;total:339 | iSNV |
| F36 | F36-20 | 1117 | E | 0.4716 | A:205;G:183;C:0;T:0;total:388 | iSNV |
| F36 | F36-20 | 1218 | E | 1 | A:0;G:0;C:0;T:404;total:404 | SNP |
| F36 | F36-20 | 1382 | E | 0.0262 | A:0;G:0;C:483;T:13;total:496 | iSNV |
| F36 | F36-20 | 1428 | E | 0.0358 | A:484;G:18;C:0;T:0;total:502 | iSNV |
| F36 | F36-20 | 1593 | E | 0.0324 | A:9;G:268;C:0;T:0;total:277 | iSNV |
| F36 | F36-20 | 1721 | E | 0.252 | A:279;G:94;C:0;T:0;total:373 | iSNV |
| F36 | F36-20 | 2078 | E | 0.0303 | A:0;G:0;C:288;T:9;total:297 | iSNV |
| F36 | F36-20 | 2230 | E | 0.0957 | A:0;G:0;C:387;T:41;total:428 | iSNV |
| F36 | F36-20 | 2274 | E | 0.0357 | A:0;G:351;C:0;T:13;total:364 | iSNV |
| F36 | F36-20 | 2369 | E | 0.104 | A:23;G:198;C:0;T:0;total:221 | iSNV |
| F36 | F36-20 | 3869 | NS2A | 0.6981 | A:0;G:0;C:183;T:423;total:606 | iSNV |
| F36 | F36-20 | 3929 | NS2A | 0.1475 | A:0;G:0;C:543;T:94;total:637 | iSNV |
| F36 | F36-20 | 4319 | NS2B | 0.0239 | A:12;G:490;C:0;T:0;total:502 | iSNV |
| F36 | F36-20 | 4697 | NS3 | 0.3195 | A:0;G:0;C:170;T:362;total:532 | iSNV |
| F36 | F36-20 | 5365 | NS3 | 0.0249 | A:391;G:10;C:0;T:0;total:401 | iSNV |
| F36 | F36-20 | 5602 | NS3 | 0.023 | A:0;G:0;C:552;T:13;total:565 | iSNV |
| F36 | F36-20 | 5952 | NS3 | 0.3147 | A:0;G:0;C:468;T:215;total:683 | iSNV |
| F36 | F36-20 | 6786 | NS4A | 0.0788 | A:0;G:0;C:397;T:34;total:431 | iSNV |
| F36 | F36-20 | 6938 | NS4A | 0.0341 | A:0;G:0;C:311;T:11;total:322 | iSNV |
| F36 | F36-20 | 7268 | NS4B | 0.0243 | A:240;G:0;C:6;T:0;total:246 | iSNV |
| F36 | F36-20 | 7633 | NS4B | 0.3083 | A:0;G:0;C:406;T:181;total:587 | iSNV |
| F36 | F36-20 | 9359 | NS5 | 0.3062 | A:0;G:0;C:249;T:564;total:813 | iSNV |
| F36 | F36-20 | 10259 | NS5 | 0.3099 | A:423;G:190;C:0;T:0;total:613 | iSNV |
| F36 | F36-20 | 10428 | 3'-UTR | 0.1174 | A:0;G:0;C:421;T:56;total:477 | iSNV |
| F36 | F36-20 | 10566 | 3'-UTR | 0.2905 | A:1;G:0;C:231;T:95;total:327 | iSNV |
| F36 | F36-21 | 221 | C | 0.0684 | A:35;G:476;C:0;T:0;total:511 | iSNV |
| F36 | F36-21 | 897 | M | 0.0356 | A:0;G:0;C:325;T:12;total:337 | iSNV |

| | | | | | | |
|---|---|---|---|---|---|---|
| F36 | F36-21 | 998 | E | 0.0449 | A:0;G:0;C:340;T:16;total:356 | iSNV |
| F36 | F36-21 | 1218 | E | 1 | A:0;G:0;C:0;T:363;total:363 | SNP |
| F36 | F36-21 | 1428 | E | 0.1597 | A:305;G:58;C:0;T:0;total:363 | iSNV |
| F36 | F36-21 | 1708 | E | 0.0341 | A:10;G:283;C:0;T:0;total:293 | iSNV |
| F36 | F36-21 | 1797 | E | 0.051 | A:0;G:22;C:0;T:409;total:431 | iSNV |
| F36 | F36-21 | 3146 | NS1 | 0.0391 | A:0;G:0;C:760;T:31;total:791 | iSNV |
| F36 | F36-21 | 3317 | NS1 | 0.0239 | A:0;G:0;C:490;T:12;total:502 | iSNV |
| F36 | F36-21 | 3661 | NS1 | 0.2176 | A:0;G:0;C:42;T:151;total:193 | iSNV |
| F36 | F36-21 | 3869 | NS2A | 0.94 | A:0;G:0;C:29;T:454;total:483 | iSNV |
| F36 | F36-21 | 4093 | NS2A | 0.1702 | A:268;G:55;C:0;T:0;total:323 | iSNV |
| F36 | F36-21 | 4559 | NS2B | 0.1162 | A:0;G:0;C:380;T:50;total:430 | iSNV |
| F36 | F36-21 | 5276 | NS3 | 0.0285 | A:409;G:12;C:0;T:0;total:421 | iSNV |
| F36 | F36-21 | 5952 | NS3 | 0.0293 | A:0;G:0;C:662;T:20;total:682 | iSNV |
| F36 | F36-21 | 6004 | NS3 | 0.1419 | A:0;G:0;C:520;T:86;total:606 | iSNV |
| F36 | F36-21 | 6206 | NS3 | 0.0569 | A:0;G:0;C:265;T:16;total:281 | iSNV |
| F36 | F36-21 | 7049 | NS4A | 0.0231 | A:7;G:296;C:0;T:0;total:303 | iSNV |
| F36 | F36-21 | 7633 | NS4B | 0.2822 | A:0;G:0;C:506;T:199;total:705 | iSNV |
| F36 | F36-21 | 8194 | NS5 | 0.0458 | A:416;G:20;C:0;T:0;total:436 | iSNV |
| F36 | F36-21 | 9359 | NS5 | 0.0287 | A:0;G:0;C:24;T:810;total:834 | iSNV |
| F36 | F36-21 | 10259 | NS5 | 0.0354 | A:544;G:20;C:0;T:0;total:564 | iSNV |
| F36 | F36-21 | 10358 | NS5 | 0.0532 | A:0;G:0;C:516;T:29;total:545 | iSNV |
| F36 | F36-21 | 10428 | 3'-UTR | 0.1448 | A:0;G:0;C:307;T:52;total:359 | iSNV |
| F36 | F36-21 | 10447 | 3'-UTR | 0.0264 | A:0;G:0;C:294;T:8;total:302 | iSNV |
| F36 | F36-21 | 10451 | 3'-UTR | 0.0548 | A:0;G:0;C:293;T:17;total:310 | iSNV |
| F36 | F36-21 | 10513 | 3'-UTR | 0.0255 | A:0;G:0;C:8;T:305;total:313 | iSNV |
| F36 | F36-21 | 10578 | 3'-UTR | 0.2128 | A:0;G:0;C:53;T:196;total:249 | iSNV |
| F36 | F36-22 | 221 | C | 0.0245 | A:19;G:756;C:0;T:0;total:775 | iSNV |
| F36 | F36-22 | 353 | C | 0.1024 | A:876;G:100;C:0;T:0;total:976 | iSNV |
| F36 | F36-22 | 645 | M | 0.0827 | A:676;G:61;C:0;T:0;total:737 | iSNV |
| F36 | F36-22 | 941 | M | 0.0293 | A:13;G:0;C:0;T:430;total:443 | iSNV |
| F36 | F36-22 | 998 | E | 0.1802 | A:0;G:0;C:341;T:75;total:416 | iSNV |
| F36 | F36-22 | 1004 | E | 0.0657 | A:0;G:0;C:28;T:398;total:426 | iSNV |
| F36 | F36-22 | 1218 | E | 0.882 | A:0;G:0;C:62;T:463;total:525 | iSNV |
| F36 | F36-22 | 1397 | E | 0.0389 | A:0;G:0;C:568;T:23;total:591 | iSNV |
| F36 | F36-22 | 1428 | E | 0.1121 | A:467;G:59;C:0;T:0;total:526 | iSNV |
| F36 | F36-22 | 1461 | E | 0.0637 | A:0;G:0;C:25;T:367;total:392 | iSNV |
| F36 | F36-22 | 2451 | E | 0.0434 | A:10;G:220;C:0;T:0;total:230 | iSNV |
| F36 | F36-22 | 3011 | NS1 | 0.0228 | A:13;G:557;C:0;T:0;total:570 | iSNV |
| F36 | F36-22 | 3137 | NS1 | 0.0556 | A:0;G:0;C:900;T:53;total:953 | iSNV |
| F36 | F36-22 | 3572 | NS1 | 0.0522 | A:0;G:0;C:272;T:15;total:287 | iSNV |
| F36 | F36-22 | 3671 | NS1 | 0.0526 | A:0;G:0;C:12;T:216;total:228 | iSNV |
| F36 | F36-22 | 3869 | NS2A | 0.7992 | A:0;G:0;C:134;T:533;total:667 | iSNV |
| F36 | F36-22 | 4319 | NS2B | 0.0684 | A:39;G:531;C:0;T:0;total:570 | iSNV |
| F36 | F36-22 | 4468 | NS2B | 0.0409 | A:15;G:0;C:0;T:351;total:366 | iSNV |
| F36 | F36-22 | 4559 | NS2B | 0.0381 | A:0;G:0;C:529;T:21;total:550 | iSNV |
| F36 | F36-22 | 4697 | NS3 | 0.0904 | A:0;G:0;C:52;T:523;total:575 | iSNV |
| F36 | F36-22 | 5353 | NS3 | 0.0207 | A:614;G:13;C:0;T:0;total:627 | iSNV |
| F36 | F36-22 | 5362 | NS3 | 0.0225 | A:563;G:13;C:0;T:0;total:576 | iSNV |
| F36 | F36-22 | 5646 | NS3 | 0.0975 | A:592;G:0;C:64;T:0;total:656 | iSNV |
| F36 | F36-22 | 5654 | NS3 | 0.0439 | A:0;G:0;C:653;T:30;total:683 | iSNV |
| F36 | F36-22 | 5737 | NS3 | 0.0532 | A:694;G:39;C:0;T:0;total:733 | iSNV |
| F36 | F36-22 | 5952 | NS3 | 0.0764 | A:0;G:0;C:761;T:63;total:824 | iSNV |
| F36 | F36-22 | 6714 | NS4A | 0.0351 | A:14;G:384;C:0;T:0;total:398 | iSNV |
| F36 | F36-22 | 6938 | NS4A | 0.0352 | A:0;G:0;C:410;T:15;total:425 | iSNV |
| F36 | F36-22 | 7116 | NS4A | 0.0422 | A:0;G:0;C:340;T:15;total:355 | iSNV |
| F36 | F36-22 | 7633 | NS4B | 0.2668 | A:0;G:0;C:577;T:210;total:787 | iSNV |
| F36 | F36-22 | 7784 | NS5 | 0.0418 | A:0;G:0;C:664;T:29;total:693 | iSNV |
| F36 | F36-22 | 7901 | NS5 | 0.0241 | A:849;G:21;C:0;T:0;total:870 | iSNV |
| F36 | F36-22 | 8456 | NS5 | 0.0493 | A:674;G:0;C:35;T:0;total:709 | iSNV |
| F36 | F36-22 | 8744 | NS5 | 0.028 | A:0;G:0;C:15;T:520;total:535 | iSNV |
| F36 | F36-22 | 9245 | NS5 | 0.0481 | A:0;G:0;C:790;T:40;total:830 | iSNV |
| F36 | F36-22 | 9359 | NS5 | 0.1215 | A:0;G:0;C:128;T:925;total:1053 | iSNV |
| F36 | F36-22 | 10046 | NS5 | 0.0206 | A:474;G:10;C:0;T:0;total:484 | iSNV |
| F36 | F36-22 | 10259 | NS5 | 0.2274 | A:574;G:169;C:0;T:0;total:743 | iSNV |
| F36 | F36-22 | 10428 | 3'-UTR | 0.2067 | A:0;G:0;C:422;T:110;total:532 | iSNV |
| F36 | F36-22 | 10447 | 3'-UTR | 0.1398 | A:0;G:0;C:412;T:67;total:479 | iSNV |
| F36 | F36-22 | 10451 | 3'-UTR | 0.0481 | A:0;G:0;C:474;T:24;total:498 | iSNV |
| F36 | F36-22 | 10566 | 3'-UTR | 0.0975 | A:0;G:0;C:370;T:40;total:410 | iSNV |
| F36 | F36-23 | 353 | C | 0.2344 | A:480;G:147;C:0;T:0;total:627 | iSNV |
| F36 | F36-23 | 645 | M | 0.2009 | A:322;G:81;C:0;T:0;total:403 | iSNV |
| F36 | F36-23 | 752 | M | 0.024 | A:405;G:10;C:0;T:0;total:415 | iSNV |
| F36 | F36-23 | 803 | M | 0.0947 | A:0;G:191;C:0;T:20;total:211 | iSNV |
| F36 | F36-23 | 998 | E | 0.3774 | A:0;G:0;C:188;T:114;total:302 | iSNV |
| F36 | F36-23 | 1007 | E | 0.0737 | A:0;G:0;C:289;T:23;total:312 | iSNV |
| F36 | F36-23 | 1083 | E | 0.0226 | A:216;G:5;C:0;T:0;total:221 | iSNV |
| F36 | F36-23 | 1117 | E | 0.2619 | A:200;G:71;C:0;T:0;total:271 | iSNV |
| F36 | F36-23 | 1218 | E | 0.6979 | A:0;G:0;C:84;T:194;total:278 | iSNV |
| F36 | F36-23 | 1416 | E | 0.0349 | A:331;G:12;C:0;T:0;total:343 | iSNV |
| F36 | F36-23 | 1512 | E | 0.1528 | A:194;G:35;C:0;T:0;total:229 | iSNV |
| F36 | F36-23 | 1745 | E | 0.0607 | A:17;G:263;C:0;T:0;total:280 | iSNV |
| F36 | F36-23 | 2808 | NS1 | 0.0523 | A:0;G:0;C:23;T:416;total:439 | iSNV |
| F36 | F36-23 | 3257 | NS1 | 0.0359 | A:0;G:0;C:19;T:509;total:528 | iSNV |

| | | | | | | |
|---|---|---|---|---|---|---|
| F36 | F36-23 | 3869 | NS2A | 0.617 | A:0;G:0;C:172;T:277;total:449 | iSNV |
| F36 | F36-23 | 4517 | NS2B | 0.0394 | A:0;G:0;C:390;T:16;total:406 | iSNV |
| F36 | F36-23 | 4697 | NS3 | 0.1374 | A:0;G:0;C:51;T:320;total:371 | iSNV |
| F36 | F36-23 | 5826 | NS3 | 0.1614 | A:431;G:83;C:0;T:0;total:514 | iSNV |
| F36 | F36-23 | 5952 | NS3 | 0.1224 | A:0;G:0;C:466;T:65;total:531 | iSNV |
| F36 | F36-23 | 6419 | NS3 | 0.0205 | A:0;G:0;C:6;T:286;total:292 | iSNV |
| F36 | F36-23 | 6471 | NS4A | 0.138 | A:37;G:231;C:0;T:0;total:268 | iSNV |
| F36 | F36-23 | 6900 | NS4A | 0.1349 | A:0;G:217;C:1;T:34;total:252 | iSNV |
| F36 | F36-23 | 7633 | NS4B | 0.4243 | A:0;G:1;C:288;T:213;total:502 | iSNV |
| F36 | F36-23 | 7735 | NS5 | 0.0677 | A:32;G:438;C:2;T:0;total:472 | iSNV |
| F36 | F36-23 | 8518 | NS5 | 0.0526 | A:306;G:0;C:0;T:17;total:323 | iSNV |
| F36 | F36-23 | 9359 | NS5 | 0.1454 | A:0;G:0;C:103;T:605;total:708 | iSNV |
| F36 | F36-23 | 9416 | NS5 | 0.0203 | A:0;G:0;C:627;T:13;total:640 | iSNV |
| F36 | F36-23 | 10259 | NS5 | 0.3359 | A:344;G:174;C:0;T:0;total:518 | iSNV |
| F36 | F36-23 | 10428 | 3'-UTR | 0.3032 | A:0;G:0;C:216;T:94;total:310 | iSNV |
| F36 | F36-23 | 10447 | 3'-UTR | 0.2172 | A:0;G:0;C:227;T:63;total:290 | iSNV |
| F36 | F36-23 | 10451 | 3'-UTR | 0.0263 | A:0;G:0;C:296;T:8;total:304 | iSNV |
| F36 | F36-23 | 10566 | 3'-UTR | 0.1115 | A:0;G:0;C:231;T:29;total:260 | iSNV |
| F36 | F36-23 | 10804 | 3'-UTR | 0.0975 | A:0;G:0;C:185;T:20;total:205 | iSNV |
| F36 | F36-24 | 353 | C | 0.0726 | A:804;G:63;C:0;T:0;total:867 | iSNV |
| F36 | F36-24 | 385 | C | 0.0224 | A:958;G:22;C:0;T:0;total:980 | iSNV |
| F36 | F36-24 | 645 | M | 0.0642 | A:510;G:35;C:0;T:0;total:545 | iSNV |
| F36 | F36-24 | 658 | M | 0.0349 | A:525;G:19;C:0;T:0;total:544 | iSNV |
| F36 | F36-24 | 716 | M | 0.0372 | A:0;G:595;C:0;T:23;total:618 | iSNV |
| F36 | F36-24 | 998 | E | 0.2515 | A:0;G:0;C:235;T:79;total:314 | iSNV |
| F36 | F36-24 | 1117 | E | 0.0856 | A:331;G:31;C:0;T:0;total:362 | iSNV |
| F36 | F36-24 | 1218 | E | 0.92 | A:0;G:0;C:33;T:379;total:412 | iSNV |
| F36 | F36-24 | 1413 | E | 0.131 | A:398;G:60;C:0;T:0;total:458 | iSNV |
| F36 | F36-24 | 1805 | E | 0.0333 | A:0;G:0;C:464;T:16;total:480 | iSNV |
| F36 | F36-24 | 2076 | E | 0.195 | A:194;G:47;C:0;T:0;total:241 | iSNV |
| F36 | F36-24 | 2234 | E | 0.023 | A:10;G:423;C:0;T:0;total:433 | iSNV |
| F36 | F36-24 | 2277 | E | 0.055 | A:0;G:0;C:22;T:378;total:400 | iSNV |
| F36 | F36-24 | 2369 | E | 0.0462 | A:8;G:165;C:0;T:0;total:173 | iSNV |
| F36 | F36-24 | 2390 | E | 0.0357 | A:7;G:189;C:0;T:0;total:196 | iSNV |
| F36 | F36-24 | 2890 | NS1 | 0.2089 | A:144;G:0;C:545;T:0;total:689 | iSNV |
| F36 | F36-24 | 3110 | NS1 | 0.0483 | A:709;G:36;C:0;T:0;total:745 | iSNV |
| F36 | F36-24 | 3338 | NS1 | 0.0429 | A:0;G:0;C:23;T:513;total:536 | iSNV |
| F36 | F36-24 | 3669 | NS1 | 0.2017 | A:0;G:0;C:182;T:46;total:228 | iSNV |
| F36 | F36-24 | 3866 | NS2A | 0.0283 | A:0;G:0;C:17;T:582;total:599 | iSNV |
| F36 | F36-24 | 3869 | NS2A | 0.7652 | A:0;G:0;C:140;T:456;total:596 | iSNV |
| F36 | F36-24 | 4070 | NS2A | 0.0216 | A:0;G:0;C:8;T:361;total:369 | iSNV |
| F36 | F36-24 | 4663 | NS3 | 0.0525 | A:0;G:0;C:379;T:21;total:400 | iSNV |
| F36 | F36-24 | 4697 | NS3 | 0.1926 | A:0;G:0;C:94;T:394;total:488 | iSNV |
| F36 | F36-24 | 5465 | NS3 | 0.0819 | A:717;G:64;C:0;T:0;total:781 | iSNV |
| F36 | F36-24 | 5737 | NS3 | 0.1857 | A:513;G:117;C:0;T:0;total:630 | iSNV |
| F36 | F36-24 | 5952 | NS3 | 0.1476 | A:0;G:0;C:606;T:105;total:711 | iSNV |
| F36 | F36-24 | 6419 | NS3 | 0.0267 | A:0;G:0;C:11;T:400;total:411 | iSNV |
| F36 | F36-24 | 6717 | NS4A | 0.0213 | A:0;G:0;C:367;T:8;total:375 | iSNV |
| F36 | F36-24 | 7256 | NS4A | 0.0331 | A:8;G:233;C:0;T:0;total:241 | iSNV |
| F36 | F36-24 | 7633 | NS4B | 0.3455 | A:0;G:0;C:445;T:235;total:680 | iSNV |
| F36 | F36-24 | 8130 | NS5 | 0.0279 | A:0;G:0;C:625;T:18;total:643 | iSNV |
| F36 | F36-24 | 8449 | NS5 | 0.027 | A:0;G:0;C:17;T:612;total:629 | iSNV |
| F36 | F36-24 | 9233 | NS5 | 0.1306 | A:0;G:0;C:612;T:92;total:704 | iSNV |
| F36 | F36-24 | 9284 | NS5 | 0.0239 | A:0;G:0;C:17;T:692;total:709 | iSNV |
| F36 | F36-24 | 9359 | NS5 | 0.1866 | A:0;G:0;C:160;T:697;total:857 | iSNV |
| F36 | F36-24 | 9880 | NS5 | 0.0342 | A:12;G:338;C:0;T:0;total:350 | iSNV |
| F36 | F36-24 | 9902 | NS5 | 0.0263 | A:8;G:296;C:0;T:0;total:304 | iSNV |
| F36 | F36-24 | 9922 | NS5 | 0.0553 | A:0;G:0;C:307;T:18;total:325 | iSNV |
| F36 | F36-24 | 10253 | NS5 | 0.0434 | A:551;G:0;C:0;T:25;total:576 | iSNV |
| F36 | F36-24 | 10259 | NS5 | 0.2871 | A:422;G:170;C:0;T:0;total:592 | iSNV |
| F36 | F36-24 | 10376 | NS5 | 0.0707 | A:42;G:552;C:0;T:0;total:594 | iSNV |
| F36 | F36-24 | 10419 | 3'-UTR | 0.0341 | A:0;G:0;C:424;T:15;total:439 | iSNV |
| F36 | F36-24 | 10428 | 3'-UTR | 0.2098 | A:0;G:0;C:354;T:94;total:448 | iSNV |
| F36 | F36-24 | 10447 | 3'-UTR | 0.0673 | A:0;G:0;C:360;T:26;total:386 | iSNV |
| F36 | F36-24 | 10566 | 3'-UTR | 0.1692 | A:0;G:0;C:265;T:54;total:319 | iSNV |
| F36 | F36-25 | 443 | C | 0.0677 | A:0;G:275;C:0;T:20;total:295 | iSNV |
| F36 | F36-25 | 491 | M | 0.0284 | A:0;G:0;C:8;T:273;total:281 | iSNV |
| F36 | F36-25 | 998 | E | 0.1055 | A:0;G:0;C:144;T:17;total:161 | iSNV |
| F36 | F36-25 | 1218 | E | 1 | A:0;G:0;C:0;T:198;total:198 | SNP |
| F36 | F36-25 | 1804 | E | 0.0758 | A:16;G:195;C:0;T:0;total:211 | iSNV |
| F36 | F36-25 | 2828 | NS1 | 0.0375 | A:282;G:11;C:0;T:0;total:293 | iSNV |
| F36 | F36-25 | 2855 | NS1 | 0.2288 | A:81;G:273;C:0;T:0;total:354 | iSNV |
| F36 | F36-25 | 3356 | NS1 | 0.0646 | A:13;G:188;C:0;T:0;total:201 | iSNV |
| F36 | F36-25 | 3869 | NS2A | 0.8652 | A:0;G:0;C:36;T:231;total:267 | iSNV |
| F36 | F36-25 | 4646 | NS3 | 0.0336 | A:0;G:0;C:287;T:10;total:297 | iSNV |
| F36 | F36-25 | 5558 | NS3 | 0.2367 | A:67;G:216;C:0;T:0;total:283 | iSNV |
| F36 | F36-25 | 5952 | NS3 | 0.0597 | A:0;G:0;C:252;T:16;total:268 | iSNV |
| F36 | F36-25 | 5968 | NS3 | 0.0811 | A:22;G:249;C:0;T:0;total:271 | iSNV |
| F36 | F36-25 | 6970 | NS4A | 0.0526 | A:162;G:0;C:9;T:0;total:171 | iSNV |
| F36 | F36-25 | 7633 | NS4B | 0.3355 | A:0;G:0;C:204;T:103;total:307 | iSNV |
| F36 | F36-25 | 9359 | NS5 | 0.0854 | A:0;G:0;C:33;T:353;total:386 | iSNV |
| F36 | F36-25 | 9818 | NS5 | 0.0705 | A:0;G:0;C:11;T:145;total:156 | iSNV |

| | | | | | | |
|---|---|---|---|---|---|---|
| F36 | F36-25 | 10259 | NS5 | 0.0735 | A:252;G:20;C:0;T:0;total:272 | iSNV |
| F36 | F36-25 | 10428 | 3'-UTR | 0.3227 | A:0;G:0;C:149;T:71;total:220 | iSNV |
| F36 | F36-25 | 10566 | 3'-UTR | 0.0592 | A:0;G:0;C:127;T:8;total:135 | iSNV |
| F36 | F36-26 | 470 | C | 0.0234 | A:708;G:0;C:0;T:17;total:725 | iSNV |
| F36 | F36-26 | 694 | M | 0.1168 | A:544;G:72;C:0;T:0;total:616 | iSNV |
| F36 | F36-26 | 998 | E | 0.063 | A:0;G:0;C:327;T:22;total:349 | iSNV |
| F36 | F36-26 | 1218 | E | 0.9979 | A:0;G:0;C:1;T:470;total:471 | SNP |
| F36 | F36-26 | 1262 | E | 0.0581 | A:29;G:0;C:470;T:0;total:499 | iSNV |
| F36 | F36-26 | 1263 | E | 0.078 | A:0;G:0;C:39;T:461;total:500 | iSNV |
| F36 | F36-26 | 1428 | E | 0.4114 | A:236;G:165;C:0;T:0;total:401 | iSNV |
| F36 | F36-26 | 1514 | E | 0.0206 | A:284;G:0;C:6;T:0;total:290 | iSNV |
| F36 | F36-26 | 1844 | E | 0.0204 | A:8;G:383;C:0;T:0;total:391 | iSNV |
| F36 | F36-26 | 1862 | E | 0.0285 | A:0;G:0;C:10;T:340;total:350 | iSNV |
| F36 | F36-26 | 2235 | E | 0.0439 | A:0;G:0;C:16;T:348;total:364 | iSNV |
| F36 | F36-26 | 2474 | E | 0.0411 | A:10;G:0;C:0;T:233;total:243 | iSNV |
| F36 | F36-26 | 3869 | NS2A | 0.9187 | A:0;G:0;C:48;T:542;total:590 | iSNV |
| F36 | F36-26 | 4697 | NS3 | 0.0619 | A:0;G:0;C:28;T:424;total:452 | iSNV |
| F36 | F36-26 | 5952 | NS3 | 0.0665 | A:0;G:0;C:645;T:46;total:691 | iSNV |
| F36 | F36-26 | 6080 | NS3 | 0.0755 | A:26;G:318;C:0;T:0;total:344 | iSNV |
| F36 | F36-26 | 6806 | NS4A | 0.0364 | A:0;G:396;C:0;T:15;total:411 | iSNV |
| F36 | F36-26 | 7633 | NS4B | 0.2763 | A:0;G:0;C:453;T:173;total:626 | iSNV |
| F36 | F36-26 | 8660 | NS5 | 0.0368 | A:13;G:340;C:0;T:0;total:353 | iSNV |
| F36 | F36-26 | 9359 | NS5 | 0.0681 | A:0;G:0;C:59;T:807;total:866 | iSNV |
| F36 | F36-26 | 9491 | NS5 | 0.0297 | A:326;G:10;C:0;T:0;total:336 | iSNV |
| F36 | F36-26 | 10259 | NS5 | 0.0798 | A:588;G:51;C:0;T:0;total:639 | iSNV |
| F36 | F36-26 | 10428 | 3'-UTR | 0.5647 | A:0;G:0;C:175;T:227;total:402 | iSNV |
| F36 | F36-26 | 10447 | 3'-UTR | 0.0687 | A:0;G:0;C:352;T:26;total:378 | iSNV |
| F36 | F36-26 | 10566 | 3'-UTR | 0.0611 | A:0;G:0;C:307;T:20;total:327 | iSNV |
| F36 | F36-27 | 1218 | E | 1 | A:0;G:0;C:0;T:250;total:250 | SNP |
| F36 | F36-27 | 1413 | E | 0.0239 | A:285;G:7;C:0;T:0;total:292 | iSNV |
| F36 | F36-27 | 2274 | E | 0.3173 | A:0;G:141;C:1;T:66;total:208 | iSNV |
| F36 | F36-27 | 2664 | NS1 | 0.7898 | A:0;G:0;C:94;T:353;total:447 | iSNV |
| F36 | F36-27 | 3869 | NS2A | 0.9973 | A:0;G:0;C:1;T:362;total:363 | SNP |
| F36 | F36-27 | 4974 | NS3 | 0.0284 | A:8;G:273;C:0;T:0;total:281 | iSNV |
| F36 | F36-27 | 6734 | NS4A | 0.038 | A:0;G:0;C:8;T:202;total:210 | iSNV |
| F36 | F36-27 | 7626 | NS4B | 0.8821 | A:0;G:404;C:0;T:54;total:458 | iSNV |
| F36 | F36-27 | 7633 | NS4B | 0.0805 | A:0;G:0;C:434;T:38;total:472 | iSNV |
| F36 | F36-27 | 8765 | NS5 | 0.0259 | A:10;G:375;C:0;T:0;total:385 | iSNV |
| F36 | F36-27 | 10376 | NS5 | 0.0242 | A:11;G:442;C:0;T:0;total:453 | iSNV |
| F36 | F36-27 | 10419 | 3'-UTR | 0.0447 | A:0;G:0;C:299;T:14;total:313 | iSNV |
| F36 | F36-27 | 10428 | 3'-UTR | 0.1892 | A:0;G:0;C:257;T:60;total:317 | iSNV |
| F36 | F36-27 | 10447 | 3'-UTR | 0.0295 | A:0;G:0;C:263;T:8;total:271 | iSNV |
| F36 | F36-28 | 230 | C | 0.715 | A:0;G:0;C:489;T:195;total:684 | iSNV |
| F36 | F36-28 | 353 | C | 0.0517 | A:770;G:42;C:0;T:0;total:812 | iSNV |
| F36 | F36-28 | 645 | M | 0.0524 | A:506;G:28;C:0;T:0;total:534 | iSNV |
| F36 | F36-28 | 998 | E | 0.0888 | A:0;G:0;C:318;T:31;total:349 | iSNV |
| F36 | F36-28 | 1044 | E | 0.2576 | A:0;G:0;C:109;T:314;total:423 | iSNV |
| F36 | F36-28 | 1218 | E | 0.9461 | A:0;G:0;C:25;T:438;total:463 | iSNV |
| F36 | F36-28 | 1428 | E | 0.7656 | A:94;G:307;C:0;T:0;total:401 | iSNV |
| F36 | F36-28 | 1512 | E | 0.0411 | A:303;G:13;C:0;T:0;total:316 | iSNV |
| F36 | F36-28 | 1595 | E | 0.0669 | A:18;G:251;C:0;T:0;total:269 | iSNV |
| F36 | F36-28 | 1928 | E | 0.0211 | A:0;G:0;C:5;T:231;total:236 | iSNV |
| F36 | F36-28 | 2213 | E | 0.0212 | A:10;G:460;C:0;T:0;total:470 | iSNV |
| F36 | F36-28 | 3099 | NS1 | 0.0201 | A:16;G:780;C:0;T:0;total:796 | iSNV |
| F36 | F36-28 | 3869 | NS2A | 0.9346 | A:0;G:0;C:45;T:643;total:688 | iSNV |
| F36 | F36-28 | 4348 | NS2B | 0.0324 | A:0;G:0;C:17;T:507;total:524 | iSNV |
| F36 | F36-28 | 4697 | NS3 | 0.0235 | A:0;G:0;C:13;T:538;total:551 | iSNV |
| F36 | F36-28 | 5654 | NS3 | 0.0535 | A:0;G:0;C:548;T:31;total:579 | iSNV |
| F36 | F36-28 | 5705 | NS3 | 0.0454 | A:588;G:28;C:0;T:0;total:616 | iSNV |
| F36 | F36-28 | 7633 | NS4B | 0.7187 | A:0;G:0;C:193;T:493;total:686 | iSNV |
| F36 | F36-28 | 10259 | NS5 | 0.0583 | A:581;G:36;C:0;T:0;total:617 | iSNV |
| F36 | F36-28 | 10428 | 3'-UTR | 0.0938 | A:0;G:0;C:367;T:38;total:405 | iSNV |
| F36 | F36-28 | 10447 | 3'-UTR | 0.0489 | A:0;G:1;C:329;T:17;total:347 | iSNV |
| F36 | F36-29 | 353 | C | 0.0239 | A:1141;G:28;C:0;T:0;total:1169 | iSNV |
| F36 | F36-29 | 645 | M | 0.0221 | A:796;G:18;C:0;T:0;total:814 | iSNV |
| F36 | F36-29 | 996 | E | 0.0272 | A:17;G:608;C:0;T:0;total:625 | iSNV |
| F36 | F36-29 | 998 | E | 0.0393 | A:0;G:0;C:610;T:25;total:635 | iSNV |
| F36 | F36-29 | 1117 | E | 0.0225 | A:650;G:15;C:0;T:1;total:666 | iSNV |
| F36 | F36-29 | 1218 | E | 0.9829 | A:0;G:0;C:13;T:743;total:756 | SNP |
| F36 | F36-29 | 1413 | E | 0.1003 | A:744;G:83;C:0;T:0;total:827 | iSNV |
| F36 | F36-29 | 1447 | E | 0.1026 | A:71;G:0;C:621;T:0;total:692 | iSNV |
| F36 | F36-29 | 1453 | E | 0.0206 | A:14;G:0;C:1;T:664;total:679 | iSNV |
| F36 | F36-29 | 1514 | E | 0.0266 | A:511;G:0;C:14;T:0;total:525 | iSNV |
| F36 | F36-29 | 2230 | E | 0.02 | A:0;G:0;C:587;T:12;total:599 | iSNV |
| F36 | F36-29 | 2362 | E | 0.6443 | A:0;G:127;C:230;T:0;total:357 | iSNV |
| F36 | F36-29 | 2723 | NS1 | 0.0441 | A:0;G:0;C:46;T:995;total:1041 | iSNV |
| F36 | F36-29 | 3454 | NS1 | 0.0262 | A:371;G:0;C:10;T:0;total:381 | iSNV |
| F36 | F36-29 | 3869 | NS2A | 0.9691 | A:0;G:0;C:29;T:907;total:936 | iSNV |
| F36 | F36-29 | 3959 | NS2A | 0.1196 | A:0;G:0;C:736;T:100;total:836 | iSNV |
| F36 | F36-29 | 5150 | NS3 | 0.0453 | A:757;G:1;C:0;T:36;total:794 | iSNV |
| F36 | F36-29 | 5702 | NS3 | 0.1987 | A:0;G:0;C:867;T:215;total:1082 | iSNV |
| F36 | F36-29 | 7481 | NS4B | 0.2183 | A:0;G:0;C:100;T:358;total:458 | iSNV |

| | | | | | | |
|---|---|---|---|---|---|---|
| F36 | F36-29 | 7633 | NS4B | 0.4674 | A:0;G:0;C:524;T:460;total:984 | iSNV |
| F36 | F36-29 | 7967 | NS5 | 0.0377 | A:0;G:0;C:1146;T:45;total:1191 | iSNV |
| F36 | F36-29 | 8567 | NS5 | 0.0255 | A:0;G:0;C:725;T:19;total:744 | iSNV |
| F36 | F36-29 | 8844 | NS5 | 0.0205 | A:15;G:715;C:0;T:0;total:730 | iSNV |
| F36 | F36-29 | 9634 | NS5 | 0.0299 | A:0;G:0;C:13;T:421;total:434 | iSNV |
| F36 | F36-29 | 9659 | NS5 | 0.025 | A:0;G:0;C:389;T:10;total:399 | iSNV |
| F36 | F36-29 | 10259 | NS5 | 0.0269 | A:938;G:26;C:0;T:0;total:964 | iSNV |
| F36 | F36-29 | 10376 | NS5 | 0.082 | A:72;G:806;C:0;T:0;total:878 | iSNV |
| F36 | F36-29 | 10419 | 3'-UTR | 0.0304 | A:0;G:0;C:637;T:20;total:657 | iSNV |
| F36 | F36-29 | 10428 | 3'-UTR | 0.2349 | A:0;G:0;C:495;T:152;total:647 | iSNV |
| F36 | F36-29 | 10447 | 3'-UTR | 0.062 | A:0;G:0;C:544;T:36;total:580 | iSNV |
| F36 | F36-3 | 353 | C | 0.0276 | A:598;G:17;C:0;T:0;total:615 | iSNV |
| F36 | F36-3 | 451 | C | 0.0215 | A:0;G:0;C:545;T:12;total:557 | iSNV |
| F36 | F36-3 | 828 | M | 0.2386 | A:252;G:79;C:0;T:0;total:331 | iSNV |
| F36 | F36-3 | 996 | E | 0.3684 | A:98;G:168;C:0;T:0;total:266 | iSNV |
| F36 | F36-3 | 998 | E | 0.0492 | A:0;G:0;C:270;T:14;total:284 | iSNV |
| F36 | F36-3 | 1218 | E | 0.9802 | A:0;G:0;C:6;T:296;total:302 | SNP |
| F36 | F36-3 | 1263 | E | 0.0567 | A:0;G:0;C:18;T:299;total:317 | iSNV |
| F36 | F36-3 | 1463 | E | 0.0246 | A:0;G:0;C:237;T:6;total:243 | iSNV |
| F36 | F36-3 | 1508 | E | 0.2334 | A:0;G:0;C:197;T:60;total:257 | iSNV |
| F36 | F36-3 | 1512 | E | 0.0236 | A:248;G:6;C:0;T:0;total:254 | iSNV |
| F36 | F36-3 | 1797 | E | 0.0357 | A:0;G:13;C:0;T:351;total:364 | iSNV |
| F36 | F36-3 | 2465 | E | 0.3926 | A:0;G:0;C:116;T:75;total:191 | iSNV |
| F36 | F36-3 | 2780 | NS1 | 0.2665 | A:1;G:0;C:169;T:464;total:634 | iSNV |
| F36 | F36-3 | 3452 | NS1 | 0.0279 | A:174;G:5;C:0;T:0;total:179 | iSNV |
| F36 | F36-3 | 3652 | NS1 | 0.0414 | A:0;G:0;C:7;T:162;total:169 | iSNV |
| F36 | F36-3 | 3858 | NS2A | 0.3606 | A:0;G:0;C:312;T:176;total:488 | iSNV |
| F36 | F36-3 | 3869 | NS2A | 0.9443 | A:0;G:0;C:28;T:474;total:502 | iSNV |
| F36 | F36-3 | 5306 | NS3 | 0.0385 | A:324;G:13;C:0;T:0;total:337 | iSNV |
| F36 | F36-3 | 5415 | NS3 | 0.0207 | A:0;G:2;C:469;T:10;total:481 | iSNV |
| F36 | F36-3 | 5952 | NS3 | 0.0263 | A:0;G:0;C:555;T:15;total:570 | iSNV |
| F36 | F36-3 | 6967 | NS4A | 0.2022 | A:54;G:213;C:0;T:0;total:267 | iSNV |
| F36 | F36-3 | 6971 | NS4A | 0.0588 | A:0;G:256;C:16;T:0;total:272 | iSNV |
| F36 | F36-3 | 6980 | NS4A | 0.0291 | A:266;G:0;C:0;T:8;total:274 | iSNV |
| F36 | F36-3 | 7595 | NS4B | 0.2925 | A:0;G:122;C:0;T:295;total:417 | iSNV |
| F36 | F36-3 | 7633 | NS4B | 0.1773 | A:0;G:0;C:422;T:91;total:513 | iSNV |
| F36 | F36-3 | 9233 | NS5 | 0.0322 | A:0;G:0;C:570;T:19;total:589 | iSNV |
| F36 | F36-3 | 10151 | NS5 | 0.0339 | A:313;G:11;C:0;T:0;total:324 | iSNV |
| F36 | F36-3 | 10259 | NS5 | 0.0412 | A:488;G:21;C:0;T:0;total:509 | iSNV |
| F36 | F36-3 | 10358 | NS5 | 0.0334 | A:0;G:0;C:463;T:16;total:479 | iSNV |
| F36 | F36-3 | 10428 | 3'-UTR | 0.2219 | A:0;G:0;C:291;T:83;total:374 | iSNV |
| F36 | F36-3 | 10447 | 3'-UTR | 0.033 | A:0;G:0;C:322;T:11;total:333 | iSNV |
| F36 | F36-30 | 719 | M | 0.0851 | A:0;G:0;C:859;T:80;total:939 | iSNV |
| F36 | F36-30 | 800 | M | 0.0313 | A:433;G:0;C:0;T:14;total:447 | iSNV |
| F36 | F36-30 | 1218 | E | 0.9901 | A:0;G:0;C:6;T:600;total:606 | SNP |
| F36 | F36-30 | 1413 | E | 0.9101 | A:57;G:577;C:0;T:0;total:634 | iSNV |
| F36 | F36-30 | 1428 | E | 0.3556 | A:317;G:175;C:0;T:0;total:492 | iSNV |
| F36 | F36-30 | 1512 | E | 0.0211 | A:370;G:8;C:0;T:0;total:378 | iSNV |
| F36 | F36-30 | 3191 | NS1 | 0.0509 | A:1118;G:60;C:0;T:0;total:1178 | iSNV |
| F36 | F36-30 | 3869 | NS2A | 0.9845 | A:0;G:0;C:12;T:760;total:772 | SNP |
| F36 | F36-30 | 4005 | NS2A | 0.3392 | A:0;G:0;C:337;T:173;total:510 | iSNV |
| F36 | F36-30 | 4404 | NS2B | 0.0612 | A:383;G:25;C:0;T:0;total:408 | iSNV |
| F36 | F36-30 | 5736 | NS3 | 0.0326 | A:29;G:0;C:860;T:0;total:889 | iSNV |
| F36 | F36-30 | 7633 | NS4B | 0.5316 | A:0;G:0;C:393;T:446;total:839 | iSNV |
| F36 | F36-30 | 8394 | NS5 | 0.2119 | A:0;G:0;C:209;T:777;total:986 | iSNV |
| F36 | F36-30 | 10376 | NS5 | 0.9216 | A:658;G:56;C:0;T:0;total:714 | iSNV |
| F36 | F36-30 | 10419 | 3'-UTR | 0.0282 | A:1;G:0;C:549;T:16;total:566 | iSNV |
| F36 | F36-30 | 10428 | 3'-UTR | 0.2954 | A:0;G:0;C:391;T:164;total:555 | iSNV |
| F36 | F36-30 | 10447 | 3'-UTR | 0.0603 | A:0;G:0;C:467;T:30;total:497 | iSNV |
| F36 | F36-4 | 998 | E | 0.0906 | A:0;G:0;C:341;T:34;total:375 | iSNV |
| F36 | F36-4 | 1218 | E | 1 | A:0;G:0;C:0;T:412;total:412 | SNP |
| F36 | F36-4 | 1263 | E | 0.0881 | A:0;G:0;C:38;T:393;total:431 | iSNV |
| F36 | F36-4 | 1388 | E | 0.0227 | A:0;G:0;C:472;T:11;total:483 | iSNV |
| F36 | F36-4 | 1512 | E | 0.0487 | A:293;G:15;C:0;T:0;total:308 | iSNV |
| F36 | F36-4 | 2367 | E | 0.6234 | A:84;G:139;C:0;T:0;total:223 | iSNV |
| F36 | F36-4 | 3573 | NS1 | 0.0313 | A:0;G:0;C:247;T:8;total:255 | iSNV |
| F36 | F36-4 | 3869 | NS2A | 0.9294 | A:0;G:0;C:45;T:592;total:637 | iSNV |
| F36 | F36-4 | 3993 | NS2A | 0.0319 | A:1;G:454;C:15;T:0;total:470 | iSNV |
| F36 | F36-4 | 4280 | NS2B | 0.0245 | A:0;G:0;C:398;T:10;total:408 | iSNV |
| F36 | F36-4 | 4697 | NS3 | 0.1092 | A:0;G:0;C:51;T:416;total:467 | iSNV |
| F36 | F36-4 | 5952 | NS3 | 0.0847 | A:0;G:0;C:648;T:60;total:708 | iSNV |
| F36 | F36-4 | 6322 | NS3 | 0.0782 | A:0;G:0;C:271;T:23;total:294 | iSNV |
| F36 | F36-4 | 6969 | NS4A | 0.1033 | A:269;G:0;C:31;T:0;total:300 | iSNV |
| F36 | F36-4 | 7010 | NS4A | 0.0431 | A:288;G:0;C:0;T:13;total:301 | iSNV |
| F36 | F36-4 | 7097 | NS4A | 0.0281 | A:8;G:276;C:0;T:0;total:284 | iSNV |
| F36 | F36-4 | 7633 | NS4B | 0.278 | A:0;G:0;C:444;T:171;total:615 | iSNV |
| F36 | F36-4 | 8639 | NS5 | 0.1259 | A:0;G:0;C:49;T:340;total:389 | iSNV |
| F36 | F36-4 | 9359 | NS5 | 0.0929 | A:0;G:0;C:74;T:722;total:796 | iSNV |
| F36 | F36-4 | 10259 | NS5 | 0.0833 | A:605;G:55;C:0;T:0;total:660 | iSNV |
| F36 | F36-4 | 10428 | 3'-UTR | 0.1903 | A:0;G:0;C:370;T:87;total:457 | iSNV |
| F36 | F36-4 | 10447 | 3'-UTR | 0.029 | A:0;G:0;C:401;T:12;total:413 | iSNV |
| F36 | F36-4 | 10566 | 3'-UTR | 0.0957 | A:0;G:0;C:274;T:29;total:303 | iSNV |

| | | | | | | |
|---|---|---|---|---|---|---|
| F36 | F36-5 | 294 | C | 0.044 | A:22;G:478;C:0;T:0;total:500 | iSNV |
| F36 | F36-5 | 353 | C | 0.0378 | A:560;G:22;C:0;T:0;total:582 | iSNV |
| F36 | F36-5 | 399 | C | 0.0889 | A:62;G:0;C:635;T:0;total:697 | iSNV |
| F36 | F36-5 | 645 | M | 0.0403 | A:428;G:18;C:0;T:0;total:446 | iSNV |
| F36 | F36-5 | 897 | M | 0.1195 | A:0;G:0;C:162;T:22;total:184 | iSNV |
| F36 | F36-5 | 943 | M | 0.0234 | A:0;G:0;C:208;T:5;total:213 | iSNV |
| F36 | F36-5 | 954 | M | 0.0315 | A:0;G:0;C:215;T:7;total:222 | iSNV |
| F36 | F36-5 | 998 | E | 0.1105 | A:0;G:0;C:193;T:24;total:217 | iSNV |
| F36 | F36-5 | 1117 | E | 0.0322 | A:240;G:8;C:0;T:0;total:248 | iSNV |
| F36 | F36-5 | 1218 | E | 0.9574 | A:0;G:0;C:11;T:247;total:258 | iSNV |
| F36 | F36-5 | 1413 | E | 0.0673 | A:291;G:21;C:0;T:0;total:312 | iSNV |
| F36 | F36-5 | 1428 | E | 0.02 | A:293;G:6;C:0;T:0;total:299 | iSNV |
| F36 | F36-5 | 2076 | E | 0.1256 | A:174;G:25;C:0;T:0;total:199 | iSNV |
| F36 | F36-5 | 2126 | E | 0.0603 | A:0;G:0;C:187;T:12;total:199 | iSNV |
| F36 | F36-5 | 2543 | NS1 | 0.0955 | A:0;G:0;C:246;T:26;total:272 | iSNV |
| F36 | F36-5 | 3317 | NS1 | 0.0272 | A:0;G:0;C:428;T:12;total:440 | iSNV |
| F36 | F36-5 | 4664 | NS3 | 0.0206 | A:0;G:0;C:6;T:285;total:291 | iSNV |
| F36 | F36-5 | 4697 | NS3 | 0.0376 | A:0;G:1;C:13;T:331;total:345 | iSNV |
| F36 | F36-5 | 4790 | NS3 | 0.0501 | A:417;G:22;C:0;T:0;total:439 | iSNV |
| F36 | F36-5 | 5150 | NS3 | 0.032 | A:302;G:0;C:0;T:10;total:312 | iSNV |
| F36 | F36-5 | 5952 | NS3 | 0.0329 | A:0;G:0;C:469;T:16;total:485 | iSNV |
| F36 | F36-5 | 6753 | NS4A | 0.0393 | A:0;G:220;C:0;T:9;total:229 | iSNV |
| F36 | F36-5 | 6900 | NS4A | 0.0296 | A:7;G:229;C:0;T:0;total:236 | iSNV |
| F36 | F36-5 | 7633 | NS4B | 0.3695 | A:0;G:0;C:244;T:143;total:387 | iSNV |
| F36 | F36-5 | 9341 | NS5 | 0.0267 | A:618;G:17;C:0;T:0;total:635 | iSNV |
| F36 | F36-5 | 9359 | NS5 | 0.042 | A:0;G:0;C:27;T:615;total:642 | iSNV |
| F36 | F36-5 | 10259 | NS5 | 0.0852 | A:397;G:37;C:0;T:0;total:434 | iSNV |
| F36 | F36-5 | 10376 | NS5 | 0.0529 | A:19;G:340;C:0;T:0;total:359 | iSNV |
| F36 | F36-5 | 10407 | 3'-UTR | 0.0298 | A:0;G:0;C:8;T:260;total:268 | iSNV |
| F36 | F36-5 | 10428 | 3'-UTR | 0.2014 | A:0;G:0;C:218;T:55;total:273 | iSNV |
| F36 | F36-5 | 10447 | 3'-UTR | 0.0702 | A:0;G:0;C:225;T:17;total:242 | iSNV |
| F36 | F36-5 | 10566 | 3'-UTR | 0.037 | A:0;G:0;C:208;T:8;total:216 | iSNV |
| F36 | F36-6 | 353 | C | 0.6034 | A:355;G:540;C:0;T:0;total:895 | iSNV |
| F36 | F36-6 | 645 | M | 0.6068 | A:234;G:361;C:0;T:0;total:595 | iSNV |
| F36 | F36-6 | 658 | M | 0.1349 | A:532;G:83;C:0;T:0;total:615 | iSNV |
| F36 | F36-6 | 998 | E | 0.6319 | A:0;G:0;C:169;T:290;total:459 | iSNV |
| F36 | F36-6 | 1115 | E | 0.0234 | A:0;G:13;C:0;T:542;total:555 | iSNV |
| F36 | F36-6 | 1117 | E | 0.6441 | A:199;G:360;C:0;T:0;total:559 | iSNV |
| F36 | F36-6 | 1218 | E | 0.3476 | A:0;G:0;C:396;T:211;total:607 | iSNV |
| F36 | F36-6 | 1430 | E | 0.0202 | A:0;G:0;C:630;T:13;total:643 | iSNV |
| F36 | F36-6 | 3869 | NS2A | 0.3783 | A:0;G:0;C:437;T:266;total:703 | iSNV |
| F36 | F36-6 | 4783 | NS3 | 0.0277 | A:0;G:0;C:20;T:702;total:722 | iSNV |
| F36 | F36-6 | 4974 | NS3 | 0.5944 | A:397;G:271;C:0;T:0;total:668 | iSNV |
| F36 | F36-6 | 6061 | NS3 | 0.5908 | A:282;G:407;C:0;T:0;total:689 | iSNV |
| F36 | F36-6 | 6316 | NS3 | 0.0301 | A:0;G:0;C:354;T:11;total:365 | iSNV |
| F36 | F36-6 | 7060 | NS4A | 0.6396 | A:0;G:0;C:236;T:133;total:369 | iSNV |
| F36 | F36-6 | 7633 | NS4B | 0.588 | A:0;G:0;C:293;T:418;total:711 | iSNV |
| F36 | F36-6 | 9308 | NS5 | 0.021 | A:0;G:0;C:15;T:696;total:711 | iSNV |
| F36 | F36-6 | 9491 | NS5 | 0.1958 | A:427;G:104;C:0;T:0;total:531 | iSNV |
| F36 | F36-6 | 9830 | NS5 | 0.0294 | A:0;G:0;C:13;T:429;total:442 | iSNV |
| F36 | F36-6 | 10259 | NS5 | 0.6527 | A:264;G:496;C:0;T:0;total:760 | iSNV |
| F36 | F36-6 | 10419 | 3'-UTR | 0.6535 | A:0;G:0;C:184;T:347;total:531 | iSNV |
| F36 | F36-6 | 10428 | 3'-UTR | 0.1754 | A:0;G:0;C:437;T:93;total:530 | iSNV |
| F36 | F36-6 | 10447 | 3'-UTR | 0.6652 | A:0;G:0;C:152;T:302;total:454 | iSNV |
| F36 | F36-7 | 998 | E | 0.0423 | A:0;G:0;C:475;T:21;total:496 | iSNV |
| F36 | F36-7 | 1218 | E | 0.993 | A:0;G:0;C:4;T:565;total:569 | SNP |
| F36 | F36-7 | 1400 | E | 0.0364 | A:0;G:0;C:25;T:661;total:686 | iSNV |
| F36 | F36-7 | 1413 | E | 0.1443 | A:587;G:99;C:0;T:0;total:686 | iSNV |
| F36 | F36-7 | 1416 | E | 0.0264 | A:663;G:18;C:0;T:0;total:681 | iSNV |
| F36 | F36-7 | 1428 | E | 0.0524 | A:578;G:32;C:0;T:0;total:610 | iSNV |
| F36 | F36-7 | 1430 | E | 0.0818 | A:0;G:0;C:561;T:50;total:611 | iSNV |
| F36 | F36-7 | 2067 | E | 0.0423 | A:0;G:0;C:15;T:339;total:354 | iSNV |
| F36 | F36-7 | 2275 | E | 0.0247 | A:0;G:0;C:11;T:434;total:445 | iSNV |
| F36 | F36-7 | 3257 | NS1 | 0.0739 | A:0;G:0;C:74;T:927;total:1001 | iSNV |
| F36 | F36-7 | 3317 | NS1 | 0.4086 | A:0;G:0;C:505;T:349;total:854 | iSNV |
| F36 | F36-7 | 3401 | NS1 | 0.0243 | A:0;G:0;C:601;T:15;total:616 | iSNV |
| F36 | F36-7 | 3869 | NS2A | 0.9606 | A:0;G:0;C:31;T:755;total:786 | iSNV |
| F36 | F36-7 | 3906 | NS2A | 0.0361 | A:31;G:824;C:2;T:0;total:857 | iSNV |
| F36 | F36-7 | 4697 | NS3 | 0.0406 | A:0;G:0;C:31;T:731;total:762 | iSNV |
| F36 | F36-7 | 5736 | NS3 | 0.0213 | A:20;G:0;C:918;T:0;total:938 | iSNV |
| F36 | F36-7 | 5952 | NS3 | 0.032 | A:1;G:0;C:906;T:30;total:937 | iSNV |
| F36 | F36-7 | 6233 | NS3 | 0.0322 | A:0;G:0;C:390;T:13;total:403 | iSNV |
| F36 | F36-7 | 6322 | NS3 | 0.3905 | A:0;G:0;C:206;T:132;total:338 | iSNV |
| F36 | F36-7 | 6513 | NS4A | 0.0225 | A:650;G:15;C:0;T:0;total:665 | iSNV |
| F36 | F36-7 | 7633 | NS4B | 0.3213 | A:0;G:0;C:585;T:277;total:862 | iSNV |
| F36 | F36-7 | 7976 | NS5 | 0.0506 | A:0;G:0;C:862;T:46;total:908 | iSNV |
| F36 | F36-7 | 8130 | NS5 | 0.0697 | A:0;G:0;C:760;T:57;total:817 | iSNV |
| F36 | F36-7 | 9359 | NS5 | 0.0373 | A:0;G:0;C:42;T:1081;total:1123 | iSNV |
| F36 | F36-7 | 9370 | NS5 | 0.0814 | A:92;G:0;C:0;T:1037;total:1129 | iSNV |
| F36 | F36-7 | 9491 | NS5 | 0.0203 | A:577;G:12;C:0;T:0;total:589 | iSNV |
| F36 | F36-7 | 9592 | NS5 | 0.0311 | A:436;G:14;C:0;T:0;total:450 | iSNV |
| F36 | F36-7 | 9690 | NS5 | 0.4461 | A:145;G:180;C:0;T:0;total:325 | iSNV |

| | | | | | | |
|---|---|---|---|---|---|---|
| F36 | F36-7 | 10259 | NS5 | 0.0565 | A:768;G:46;C:0;T:0;total:814 | iSNV |
| F36 | F36-7 | 10376 | NS5 | 0.1208 | A:99;G:720;C:0;T:0;total:819 | iSNV |
| F36 | F36-7 | 10428 | 3'-UTR | 0.3578 | A:0;G:0;C:402;T:224;total:626 | iSNV |
| F36 | F36-7 | 10451 | 3'-UTR | 0.0817 | A:0;G:0;C:539;T:48;total:587 | iSNV |
| F36 | F36-7 | 10590 | 3'-UTR | 0.0221 | A:0;G:0;C:486;T:11;total:497 | iSNV |
| F36 | F36-8 | 287 | C | 0.0202 | A:13;G:630;C:0;T:0;total:643 | iSNV |
| F36 | F36-8 | 869 | M | 0.3303 | A:147;G:0;C:0;T:298;total:445 | iSNV |
| F36 | F36-8 | 939 | M | 0.0202 | A:0;G:0;C:8;T:387;total:395 | iSNV |
| F36 | F36-8 | 998 | E | 0.0318 | A:0;G:0;C:365;T:12;total:377 | iSNV |
| F36 | F36-8 | 1218 | E | 1 | A:0;G:0;C:0;T:453;total:453 | SNP |
| F36 | F36-8 | 1428 | E | 0.1422 | A:410;G:68;C:0;T:0;total:478 | iSNV |
| F36 | F36-8 | 1430 | E | 0.3312 | A:0;G:0;C:321;T:159;total:480 | iSNV |
| F36 | F36-8 | 1453 | E | 0.0745 | A:0;G:0;C:34;T:422;total:456 | iSNV |
| F36 | F36-8 | 1512 | E | 0.0901 | A:333;G:33;C:0;T:0;total:366 | iSNV |
| F36 | F36-8 | 2230 | E | 0.0438 | A:0;G:0;C:436;T:20;total:456 | iSNV |
| F36 | F36-8 | 2282 | E | 0.0288 | A:0;G:0;C:370;T:11;total:381 | iSNV |
| F36 | F36-8 | 3131 | NS1 | 0.1259 | A:722;G:104;C:0;T:0;total:826 | iSNV |
| F36 | F36-8 | 3869 | NS2A | 0.9713 | A:0;G:0;C:18;T:609;total:627 | iSNV |
| F36 | F36-8 | 4187 | NS2A | 0.1256 | A:167;G:24;C:0;T:0;total:191 | iSNV |
| F36 | F36-8 | 4697 | NS3 | 0.0371 | A:0;G:0;C:21;T:545;total:566 | iSNV |
| F36 | F36-8 | 4896 | NS3 | 0.043 | A:32;G:711;C:0;T:0;total:743 | iSNV |
| F36 | F36-8 | 5353 | NS3 | 0.1695 | A:436;G:89;C:0;T:0;total:525 | iSNV |
| F36 | F36-8 | 5456 | NS3 | 0.0907 | A:0;G:0;C:661;T:66;total:727 | iSNV |
| F36 | F36-8 | 5665 | NS3 | 0.1018 | A:0;G:0;C:670;T:76;total:746 | iSNV |
| F36 | F36-8 | 5952 | NS3 | 0.0289 | A:0;G:0;C:738;T:22;total:760 | iSNV |
| F36 | F36-8 | 6314 | NS3 | 0.1756 | A:0;G:0;C:49;T:230;total:279 | iSNV |
| F36 | F36-8 | 7334 | NS4B | 0.022 | A:266;G:6;C:0;T:0;total:272 | iSNV |
| F36 | F36-8 | 7509 | NS4B | 0.0233 | A:8;G:335;C:0;T:0;total:343 | iSNV |
| F36 | F36-8 | 7633 | NS4B | 0.3642 | A:0;G:0;C:452;T:259;total:711 | iSNV |
| F36 | F36-8 | 8257 | NS5 | 0.0265 | A:514;G:14;C:0;T:0;total:528 | iSNV |
| F36 | F36-8 | 8357 | NS5 | 0.084 | A:0;G:0;C:697;T:64;total:761 | iSNV |
| F36 | F36-8 | 9359 | NS5 | 0.031 | A:0;G:0;C:29;T:905;total:934 | iSNV |
| F36 | F36-8 | 9570 | NS5 | 0.024 | A:363;G:9;C:2;T:0;total:374 | iSNV |
| F36 | F36-8 | 9818 | NS5 | 0.1891 | A:0;G:0;C:63;T:270;total:333 | iSNV |
| F36 | F36-8 | 10259 | NS5 | 0.0318 | A:699;G:23;C:0;T:0;total:722 | iSNV |
| F36 | F36-8 | 10428 | 3'-UTR | 0.2255 | A:0;G:0;C:364;T:106;total:470 | iSNV |
| F36 | F36-8 | 10444 | 3'-UTR | 0.0207 | A:424;G:9;C:0;T:0;total:433 | iSNV |
| F36 | F36-8 | 10447 | 3'-UTR | 0.1261 | A:0;G:0;C:388;T:56;total:444 | iSNV |
| F36 | F36-8 | 10452 | 3'-UTR | 0.0268 | A:435;G:12;C:0;T:0;total:447 | iSNV |
| F36 | F36-8 | 10566 | 3'-UTR | 0.0202 | A:0;G:0;C:339;T:7;total:346 | iSNV |
| F36 | F36-9 | 498 | M | 0.023 | A:0;G:381;C:0;T:9;total:390 | iSNV |
| F36 | F36-9 | 563 | M | 0.1028 | A:25;G:218;C:0;T:0;total:243 | iSNV |
| F36 | F36-9 | 650 | M | 0.021 | A:0;G:325;C:0;T:7;total:332 | iSNV |
| F36 | F36-9 | 998 | E | 0.0256 | A:1;G:0;C:189;T:5;total:195 | iSNV |
| F36 | F36-9 | 1218 | E | 0.8733 | A:0;G:0;C:27;T:186;total:213 | iSNV |
| F36 | F36-9 | 1413 | E | 0.1741 | A:256;G:54;C:0;T:0;total:310 | iSNV |
| F36 | F36-9 | 1430 | E | 0.0303 | A:0;G:0;C:256;T:8;total:264 | iSNV |
| F36 | F36-9 | 1512 | E | 0.035 | A:193;G:7;C:0;T:0;total:200 | iSNV |
| F36 | F36-9 | 1772 | E | 0.1363 | A:0;G:36;C:0;T:228;total:264 | iSNV |
| F36 | F36-9 | 1911 | E | 0.046 | A:145;G:7;C:0;T:0;total:152 | iSNV |
| F36 | F36-9 | 2258 | E | 0.0252 | A:0;G:0;C:5;T:193;total:198 | iSNV |
| F36 | F36-9 | 2282 | E | 0.0297 | A:0;G:0;C:196;T:6;total:202 | iSNV |
| F36 | F36-9 | 2372 | E | 0.2098 | A:0;G:0;C:64;T:17;total:81 | iSNV |
| F36 | F36-9 | 2531 | NS1 | 0.073 | A:0;G:0;C:16;T:203;total:219 | iSNV |
| F36 | F36-9 | 2664 | NS1 | 0.0214 | A:0;G:0;C:365;T:8;total:373 | iSNV |
| F36 | F36-9 | 2692 | NS1 | 0.026 | A:0;G:337;C:0;T:9;total:346 | iSNV |
| F36 | F36-9 | 2852 | NS1 | 0.0503 | A:0;G:0;C:453;T:24;total:477 | iSNV |
| F36 | F36-9 | 3018 | NS1 | 0.0228 | A:0;G:257;C:0;T:6;total:263 | iSNV |
| F36 | F36-9 | 3572 | NS1 | 0.1136 | A:0;G:0;C:117;T:15;total:132 | iSNV |
| F36 | F36-9 | 3869 | NS2A | 0.8989 | A:0;G:0;C:35;T:311;total:346 | iSNV |
| F36 | F36-9 | 3962 | NS2A | 0.0896 | A:0;G:0;C:254;T:25;total:279 | iSNV |
| F36 | F36-9 | 4697 | NS3 | 0.0416 | A:0;G:0;C:12;T:276;total:288 | iSNV |
| F36 | F36-9 | 4712 | NS3 | 0.0915 | A:27;G:268;C:0;T:0;total:295 | iSNV |
| F36 | F36-9 | 4813 | NS3 | 0.0211 | A:0;G:371;C:0;T:8;total:379 | iSNV |
| F36 | F36-9 | 4974 | NS3 | 0.0437 | A:12;G:262;C:0;T:0;total:274 | iSNV |
| F36 | F36-9 | 4977 | NS3 | 0.0224 | A:0;G:261;C:0;T:6;total:267 | iSNV |
| F36 | F36-9 | 5037 | NS3 | 0.0211 | A:0;G:231;C:0;T:5;total:236 | iSNV |
| F36 | F36-9 | 5311 | NS3 | 0.0655 | A:0;G:0;C:228;T:16;total:244 | iSNV |
| F36 | F36-9 | 5849 | NS3 | 0.0207 | A:0;G:377;C:0;T:8;total:385 | iSNV |
| F36 | F36-9 | 5921 | NS3 | 0.0481 | A:316;G:16;C:0;T:0;total:332 | iSNV |
| F36 | F36-9 | 5952 | NS3 | 0.0392 | A:0;G:0;C:367;T:15;total:382 | iSNV |
| F36 | F36-9 | 6034 | NS3 | 0.0218 | A:0;G:269;C:0;T:6;total:275 | iSNV |
| F36 | F36-9 | 6641 | NS4A | 0.0672 | A:0;G:0;C:111;T:8;total:119 | iSNV |
| F36 | F36-9 | 6921 | NS4A | 0.0273 | A:0;G:178;C:0;T:5;total:183 | iSNV |
| F36 | F36-9 | 7633 | NS4B | 0.3413 | A:0;G:0;C:220;T:114;total:334 | iSNV |
| F36 | F36-9 | 7744 | NS5 | 0.0709 | A:288;G:22;C:0;T:0;total:310 | iSNV |
| F36 | F36-9 | 8282 | NS5 | 0.0651 | A:0;G:0;C:17;T:244;total:261 | iSNV |
| F36 | F36-9 | 8400 | NS5 | 0.022 | A:0;G:399;C:0;T:9;total:408 | iSNV |
| F36 | F36-9 | 8677 | NS5 | 0.0271 | A:0;G:215;C:0;T:6;total:221 | iSNV |
| F36 | F36-9 | 8842 | NS5 | 0.02 | A:0;G:342;C:0;T:7;total:349 | iSNV |
| F36 | F36-9 | 8900 | NS5 | 0.0228 | A:427;G:0;C:10;T:0;total:437 | iSNV |
| F36 | F36-9 | 8938 | NS5 | 0.0207 | A:0;G:471;C:0;T:10;total:481 | iSNV |

| | | | | | | |
|---|---|---|---|---|---|---|
| F36 | F36-9 | 9011 | NS5 | 0.0203 | A:6;G:289;C:0;T:0;total:295 | iSNV |
| F36 | F36-9 | 9150 | NS5 | 0.0223 | A:0;G:306;C:0;T:7;total:313 | iSNV |
| F36 | F36-9 | 9227 | NS5 | 0.022 | A:0;G:311;C:0;T:7;total:318 | iSNV |
| F36 | F36-9 | 9359 | NS5 | 0.0251 | A:0;G:0;C:12;T:466;total:478 | iSNV |
| F36 | F36-9 | 9446 | NS5 | 0.061 | A:431;G:28;C:0;T:0;total:459 | iSNV |
| F36 | F36-9 | 10069 | NS5 | 0.0316 | A:0;G:0;C:214;T:7;total:221 | iSNV |
| F36 | F36-9 | 10259 | NS5 | 0.0395 | A:364;G:15;C:0;T:0;total:379 | iSNV |
| F36 | F36-9 | 10376 | NS5 | 0.181 | A:61;G:275;C:0;T:1;total:337 | iSNV |
| F36 | F36-9 | 10428 | 3'-UTR | 0.1515 | A:1;G:0;C:223;T:40;total:264 | iSNV |
| F36 | F36-9 | 10566 | 3'-UTR | 0.0847 | A:1;G:0;C:161;T:15;total:177 | iSNV |
| F40 | F40-1 | 221 | C | 0.3232 | A:96;G:201;C:0;T:0;total:297 | iSNV |
| F40 | F40-1 | 789 | M | 0.023 | A:0;G:212;C:0;T:5;total:217 | iSNV |
| F40 | F40-1 | 876 | M | 0.0508 | A:0;G:0;C:9;T:168;total:177 | iSNV |
| F40 | F40-1 | 897 | M | 0.2794 | A:0;G:0;C:98;T:38;total:136 | iSNV |
| F40 | F40-1 | 926 | M | 0.0994 | A:0;G:0;C:17;T:154;total:171 | iSNV |
| F40 | F40-1 | 1218 | E | 0.9915 | A:0;G:0;C:2;T:233;total:235 | SNP |
| F40 | F40-1 | 1428 | E | 0.5892 | A:76;G:109;C:0;T:0;total:185 | iSNV |
| F40 | F40-1 | 1772 | E | 0.0224 | A:0;G:5;C:0;T:218;total:223 | iSNV |
| F40 | F40-1 | 3434 | NS1 | 0.13 | A:32;G:214;C:0;T:0;total:246 | iSNV |
| F40 | F40-1 | 3869 | NS2A | 0.9638 | A:0;G:0;C:16;T:425;total:441 | iSNV |
| F40 | F40-1 | 4559 | NS2B | 0.2808 | A:0;G:0;C:251;T:98;total:349 | iSNV |
| F40 | F40-1 | 5276 | NS3 | 0.3587 | A:168;G:94;C:0;T:0;total:262 | iSNV |
| F40 | F40-1 | 5952 | NS3 | 0.0368 | A:0;G:0;C:418;T:16;total:434 | iSNV |
| F40 | F40-1 | 6970 | NS4A | 0.1235 | A:227;G:0;C:32;T:0;total:259 | iSNV |
| F40 | F40-1 | 7127 | NS4A | 0.044 | A:0;G:0;C:10;T:217;total:227 | iSNV |
| F40 | F40-1 | 7536 | NS4B | 0.116 | A:0;G:0;C:29;T:221;total:250 | iSNV |
| F40 | F40-1 | 7626 | NS4B | 0.0298 | A:9;G:0;C:0;T:293;total:302 | iSNV |
| F40 | F40-1 | 7633 | NS4B | 0.9546 | A:0;G:0;C:14;T:294;total:308 | iSNV |
| F40 | F40-1 | 9359 | NS5 | 0.0262 | A:0;G:0;C:15;T:556;total:571 | iSNV |
| F40 | F40-1 | 9983 | NS5 | 0.0413 | A:0;G:0;C:232;T:10;total:242 | iSNV |
| F40 | F40-1 | 10428 | 3'-UTR | 0.1715 | A:0;G:0;C:140;T:29;total:169 | iSNV |
| F40 | F40-1 | 10451 | 3'-UTR | 0.3566 | A:0;G:0;C:92;T:51;total:143 | iSNV |
| F40 | F40-1 | 10668 | 3'-UTR | 0.1272 | A:0;G:21;C:144;T:0;total:165 | iSNV |
| F40 | F40-10 | 1218 | E | 0.9877 | A:0;G:0;C:2;T:160;total:162 | SNP |
| F40 | F40-10 | 1551 | E | 0.8379 | A:0;G:93;C:18;T:0;total:111 | iSNV |
| F40 | F40-10 | 1680 | E | 0.0446 | A:107;G:5;C:0;T:0;total:112 | iSNV |
| F40 | F40-10 | 3869 | NS2A | 1 | A:0;G:0;C:0;T:292;total:292 | SNP |
| F40 | F40-10 | 5215 | NS3 | 0.2956 | A:131;G:55;C:0;T:0;total:186 | iSNV |
| F40 | F40-10 | 5736 | NS3 | 0.9868 | A:299;G:0;C:4;T:0;total:303 | SNP |
| F40 | F40-10 | 6900 | NS4A | 1 | A:186;G:0;C:0;T:0;total:186 | SNP |
| F40 | F40-10 | 7264 | NS4A | 1 | A:0;G:0;C:0;T:108;total:108 | SNP |
| F40 | F40-10 | 7633 | NS4B | 0.7382 | A:0;G:0;C:72;T:203;total:275 | iSNV |
| F40 | F40-10 | 7656 | NS4B | 0.1407 | A:39;G:238;C:0;T:0;total:277 | iSNV |
| F40 | F40-10 | 7673 | NS4B | 0.0208 | A:282;G:6;C:0;T:0;total:288 | iSNV |
| F40 | F40-10 | 9001 | NS5 | 0.0824 | A:178;G:16;C:0;T:0;total:194 | iSNV |
| F40 | F40-10 | 10428 | 3'-UTR | 1 | A:0;G:0;C:0;T:111;total:111 | SNP |
| F40 | F40-11 | 1218 | E | 1 | A:0;G:0;C:0;T:288;total:288 | SNP |
| F40 | F40-11 | 1431 | E | 0.6246 | A:0;G:0;C:101;T:168;total:269 | iSNV |
| F40 | F40-11 | 1797 | E | 1 | A:257;G:0;C:0;T:0;total:257 | SNP |
| F40 | F40-11 | 3869 | NS2A | 1 | A:0;G:0;C:0;T:403;total:403 | SNP |
| F40 | F40-11 | 3956 | NS2A | 0.0408 | A:19;G:446;C:0;T:0;total:465 | iSNV |
| F40 | F40-11 | 5039 | NS3 | 0.026 | A:299;G:8;C:0;T:0;total:307 | iSNV |
| F40 | F40-11 | 6714 | NS4A | 0.0368 | A:9;G:235;C:0;T:0;total:244 | iSNV |
| F40 | F40-11 | 6867 | NS4A | 0.6623 | A:149;G:76;C:0;T:0;total:225 | iSNV |
| F40 | F40-11 | 7633 | NS4B | 0.965 | A:0;G:0;C:13;T:358;total:371 | iSNV |
| F40 | F40-11 | 8948 | NS5 | 0.6005 | A:242;G:161;C:0;T:0;total:403 | iSNV |
| F40 | F40-11 | 9880 | NS5 | 1 | A:207;G:0;C:0;T:0;total:207 | SNP |
| F40 | F40-11 | 10265 | NS5 | 0.0418 | A:0;G:0;C:252;T:11;total:263 | iSNV |
| F40 | F40-11 | 10589 | 3'-UTR | 1 | A:0;G:191;C:0;T:0;total:191 | SNP |
| F40 | F40-12 | 264 | C | 0.0281 | A:518;G:15;C:0;T:0;total:533 | iSNV |
| F40 | F40-12 | 353 | C | 0.2648 | A:347;G:125;C:0;T:0;total:472 | iSNV |
| F40 | F40-12 | 645 | M | 0.245 | A:228;G:74;C:0;T:0;total:302 | iSNV |
| F40 | F40-12 | 869 | M | 0.131 | A:0;G:38;C:0;T:252;total:290 | iSNV |
| F40 | F40-12 | 998 | E | 0.4913 | A:0;G:0;C:117;T:113;total:230 | iSNV |
| F40 | F40-12 | 1083 | E | 0.0288 | A:202;G:6;C:0;T:0;total:208 | iSNV |
| F40 | F40-12 | 1117 | E | 0.3417 | A:156;G:81;C:0;T:0;total:237 | iSNV |
| F40 | F40-12 | 1131 | E | 0.0268 | A:0;G:7;C:254;T:0;total:261 | iSNV |
| F40 | F40-12 | 1218 | E | 0.6766 | A:0;G:0;C:87;T:182;total:269 | iSNV |
| F40 | F40-12 | 1337 | E | 0.0305 | A:0;G:0;C:286;T:9;total:295 | iSNV |
| F40 | F40-12 | 1367 | E | 0.0511 | A:0;G:0;C:278;T:15;total:293 | iSNV |
| F40 | F40-12 | 1428 | E | 0.0661 | A:239;G:17;C:0;T:1;total:257 | iSNV |
| F40 | F40-12 | 1453 | E | 0.046 | A:11;G:0;C:0;T:228;total:239 | iSNV |
| F40 | F40-12 | 1911 | E | 0.0677 | A:165;G:12;C:0;T:0;total:177 | iSNV |
| F40 | F40-12 | 2078 | E | 0.1428 | A:0;G:0;C:186;T:31;total:217 | iSNV |
| F40 | F40-12 | 2277 | E | 0.2952 | A:0;G:0;C:62;T:148;total:210 | iSNV |
| F40 | F40-12 | 2369 | E | 0.1703 | A:23;G:112;C:0;T:0;total:135 | iSNV |
| F40 | F40-12 | 2744 | NS1 | 0.0347 | A:0;G:15;C:0;T:417;total:432 | iSNV |
| F40 | F40-12 | 3257 | NS1 | 0.0404 | A:0;G:0;C:19;T:451;total:470 | iSNV |
| F40 | F40-12 | 3869 | NS2A | 0.6166 | A:0;G:0;C:158;T:254;total:412 | iSNV |
| F40 | F40-12 | 4068 | NS2A | 0.0357 | A:270;G:0;C:0;T:10;total:280 | iSNV |
| F40 | F40-12 | 4098 | NS2A | 0.0238 | A:205;G:5;C:0;T:0;total:210 | iSNV |
| F40 | F40-12 | 4394 | NS2B | 0.0319 | A:0;G:0;C:273;T:9;total:282 | iSNV |

| | | | | | | |
|---|---|---|---|---|---|---|
| F40 | F40-12 | 4613 | NS3 | 0.0217 | A:0;G:0;C:405;T:9;total:414 | iSNV |
| F40 | F40-12 | 4697 | NS3 | 0.1183 | A:0;G:0;C:40;T:298;total:338 | iSNV |
| F40 | F40-12 | 4974 | NS3 | 0.0207 | A:6;G:283;C:0;T:0;total:289 | iSNV |
| F40 | F40-12 | 5333 | NS3 | 0.0344 | A:252;G:9;C:0;T:0;total:261 | iSNV |
| F40 | F40-12 | 5336 | NS3 | 0.0267 | A:0;G:0;C:7;T:255;total:262 | iSNV |
| F40 | F40-12 | 5558 | NS3 | 0.0266 | A:11;G:401;C:0;T:0;total:412 | iSNV |
| F40 | F40-12 | 5705 | NS3 | 0.0404 | A:403;G:17;C:0;T:0;total:420 | iSNV |
| F40 | F40-12 | 5780 | NS3 | 0.2025 | A:0;G:0;C:94;T:370;total:464 | iSNV |
| F40 | F40-12 | 5952 | NS3 | 0.1366 | A:0;G:0;C:360;T:57;total:417 | iSNV |
| F40 | F40-12 | 6098 | NS3 | 0.0242 | A:0;G:0;C:241;T:6;total:247 | iSNV |
| F40 | F40-12 | 6714 | NS4A | 0.0217 | A:5;G:225;C:0;T:0;total:230 | iSNV |
| F40 | F40-12 | 6867 | NS4A | 0.23 | A:49;G:164;C:0;T:0;total:213 | iSNV |
| F40 | F40-12 | 6934 | NS4A | 0.0365 | A:237;G:9;C:0;T:0;total:246 | iSNV |
| F40 | F40-12 | 6969 | NS4A | 0.0344 | A:224;G:8;C:0;T:0;total:232 | iSNV |
| F40 | F40-12 | 7561 | NS4B | 0.06 | A:0;G:0;C:18;T:282;total:300 | iSNV |
| F40 | F40-12 | 7633 | NS4B | 0.8527 | A:0;G:0;C:51;T:295;total:346 | iSNV |
| F40 | F40-12 | 7634 | NS4B | 0.1457 | A:0;G:51;C:0;T:299;total:350 | iSNV |
| F40 | F40-12 | 7656 | NS4B | 0.0324 | A:12;G:358;C:0;T:0;total:370 | iSNV |
| F40 | F40-12 | 9290 | NS5 | 0.0383 | A:0;G:0;C:376;T:15;total:391 | iSNV |
| F40 | F40-12 | 9359 | NS5 | 0.1885 | A:0;G:0;C:89;T:383;total:472 | iSNV |
| F40 | F40-12 | 9491 | NS5 | 0.228 | A:193;G:57;C:0;T:0;total:250 | iSNV |
| F40 | F40-12 | 9728 | NS5 | 0.1304 | A:0;G:0;C:160;T:24;total:184 | iSNV |
| F40 | F40-12 | 9839 | NS5 | 0.1735 | A:0;G:0;C:42;T:200;total:242 | iSNV |
| F40 | F40-12 | 10259 | NS5 | 0.4515 | A:164;G:135;C:0;T:0;total:299 | iSNV |
| F40 | F40-12 | 10419 | 3'-UTR | 0.0324 | A:0;G:0;C:209;T:7;total:216 | iSNV |
| F40 | F40-12 | 10428 | 3'-UTR | 0.5095 | A:0;G:0;C:104;T:108;total:212 | iSNV |
| F40 | F40-12 | 10447 | 3'-UTR | 0.2647 | A:0;G:0;C:150;T:54;total:204 | iSNV |
| F40 | F40-12 | 10566 | 3'-UTR | 0.198 | A:0;G:0;C:162;T:40;total:202 | iSNV |
| F40 | F40-13 | 395 | C | 0.9296 | A:44;G:581;C:0;T:0;total:625 | iSNV |
| F40 | F40-13 | 530 | M | 0.5069 | A:0;G:0;C:179;T:184;total:363 | iSNV |
| F40 | F40-13 | 1218 | E | 0.9737 | A:0;G:0;C:8;T:296;total:304 | iSNV |
| F40 | F40-13 | 1413 | E | 0.9152 | A:28;G:302;C:0;T:0;total:330 | iSNV |
| F40 | F40-13 | 1512 | E | 0.0386 | A:199;G:8;C:0;T:0;total:207 | iSNV |
| F40 | F40-13 | 1708 | E | 0.4014 | A:110;G:164;C:0;T:0;total:274 | iSNV |
| F40 | F40-13 | 1772 | E | 0.0235 | A:0;G:7;C:0;T:290;total:297 | iSNV |
| F40 | F40-13 | 1911 | E | 0.2408 | A:104;G:33;C:0;T:0;total:137 | iSNV |
| F40 | F40-13 | 2282 | E | 0.0506 | A:0;G:0;C:206;T:11;total:217 | iSNV |
| F40 | F40-13 | 2372 | E | 0.0451 | A:0;G:0;C:127;T:6;total:133 | iSNV |
| F40 | F40-13 | 3869 | NS2A | 1 | A:0;G:0;C:0;T:489;total:489 | SNP |
| F40 | F40-13 | 4052 | NS2A | 0.1604 | A:0;G:0;C:225;T:43;total:268 | iSNV |
| F40 | F40-13 | 5372 | NS3 | 0.1005 | A:0;G:0;C:38;T:340;total:378 | iSNV |
| F40 | F40-13 | 5490 | NS3 | 0.1198 | A:382;G:52;C:0;T:0;total:434 | iSNV |
| F40 | F40-13 | 6107 | NS3 | 0.0223 | A:0;G:0;C:263;T:6;total:269 | iSNV |
| F40 | F40-13 | 6781 | NS4A | 0.0429 | A:0;G:290;C:13;T:0;total:303 | iSNV |
| F40 | F40-13 | 6867 | NS4A | 0.0729 | A:21;G:267;C:0;T:0;total:288 | iSNV |
| F40 | F40-13 | 6900 | NS4A | 0.0334 | A:0;G:289;C:0;T:10;total:299 | iSNV |
| F40 | F40-13 | 6970 | NS4A | 0.558 | A:145;G:0;C:183;T:0;total:328 | iSNV |
| F40 | F40-13 | 6981 | NS4A | 0.04 | A:0;G:0;C:13;T:312;total:325 | iSNV |
| F40 | F40-13 | 7011 | NS4A | 0.0439 | A:0;G:0;C:326;T:15;total:341 | iSNV |
| F40 | F40-13 | 7176 | NS4A | 0.1739 | A:152;G:32;C:0;T:0;total:184 | iSNV |
| F40 | F40-13 | 7633 | NS4B | 0.8482 | A:0;G:0;C:60;T:335;total:395 | iSNV |
| F40 | F40-13 | 8080 | NS5 | 0.0265 | A:551;G:15;C:0;T:0;total:566 | iSNV |
| F40 | F40-13 | 9881 | NS5 | 0.0833 | A:209;G:19;C:0;T:0;total:228 | iSNV |
| F40 | F40-13 | 10376 | NS5 | 0.9534 | A:286;G:14;C:0;T:0;total:300 | iSNV |
| F40 | F40-13 | 10428 | 3'-UTR | 0.4549 | A:0;G:0;C:139;T:116;total:255 | iSNV |
| F40 | F40-13 | 10577 | 3'-UTR | 0.0931 | A:0;G:0;C:263;T:27;total:290 | iSNV |
| F40 | F40-14 | 998 | E | 0.961 | A:0;G:0;C:5;T:123;total:128 | iSNV |
| F40 | F40-14 | 1218 | E | 0.9667 | A:0;G:0;C:5;T:145;total:150 | iSNV |
| F40 | F40-14 | 1512 | E | 0.055 | A:103;G:6;C:0;T:0;total:109 | iSNV |
| F40 | F40-14 | 3869 | NS2A | 0.8731 | A:0;G:0;C:24;T:165;total:189 | iSNV |
| F40 | F40-14 | 3962 | NS2A | 0.0299 | A:0;G:0;C:162;T:5;total:167 | iSNV |
| F40 | F40-14 | 4697 | NS3 | 0.9193 | A:0;G:0;C:148;T:13;total:161 | iSNV |
| F40 | F40-14 | 4712 | NS3 | 0.0391 | A:7;G:172;C:0;T:0;total:179 | iSNV |
| F40 | F40-14 | 5952 | NS3 | 0.924 | A:0;G:0;C:19;T:231;total:250 | iSNV |
| F40 | F40-14 | 6523 | NS4A | 0.0495 | A:0;G:0;C:115;T:6;total:121 | iSNV |
| F40 | F40-14 | 6867 | NS4A | 0.8264 | A:119;G:25;C:0;T:0;total:144 | iSNV |
| F40 | F40-14 | 7561 | NS4B | 0.8637 | A:0;G:0;C:152;T:24;total:176 | iSNV |
| F40 | F40-14 | 7633 | NS4B | 0.0804 | A:0;G:0;C:183;T:16;total:199 | iSNV |
| F40 | F40-14 | 8282 | NS5 | 0.0392 | A:0;G:0;C:6;T:147;total:153 | iSNV |
| F40 | F40-14 | 9359 | NS5 | 0.9392 | A:0;G:0;C:247;T:16;total:263 | iSNV |
| F40 | F40-14 | 9370 | NS5 | 0.9385 | A:0;G:244;C:0;T:16;total:260 | iSNV |
| F40 | F40-14 | 9446 | NS5 | 0.0491 | A:232;G:12;C:0;T:0;total:244 | iSNV |
| F40 | F40-14 | 10259 | NS5 | 0.9392 | A:9;G:139;C:0;T:0;total:148 | iSNV |
| F40 | F40-14 | 10435 | 3'-UTR | 0.8462 | A:16;G:88;C:0;T:0;total:104 | iSNV |
| F40 | F40-15 | 1218 | E | 0.8847 | A:0;G:0;C:6;T:46;total:52 | iSNV |
| F40 | F40-15 | 1428 | E | 0.1 | A:45;G:5;C:0;T:0;total:50 | iSNV |
| F40 | F40-15 | 2906 | NS1 | 0.1265 | A:0;G:0;C:69;T:10;total:79 | iSNV |
| F40 | F40-15 | 3177 | NS1 | 0.1233 | A:19;G:135;C:0;T:0;total:154 | iSNV |
| F40 | F40-15 | 3869 | NS2A | 0.8154 | A:0;G:0;C:12;T:53;total:65 | iSNV |
| F40 | F40-15 | 3965 | NS2A | 0.1296 | A:0;G:0;C:47;T:7;total:54 | iSNV |
| F40 | F40-15 | 6867 | NS4A | 0.1956 | A:9;G:37;C:0;T:0;total:46 | iSNV |
| F40 | F40-15 | 7167 | NS4A | 0.1428 | A:7;G:42;C:0;T:0;total:49 | iSNV |

| | | | | | | |
|---|---|---|---|---|---|---|
| F40 | F40-15 | 7633 | NS4B | 0.7946 | A:0;G:0;C:15;T:58;total:73 | iSNV |
| F40 | F40-15 | 8282 | NS5 | 0.1333 | A:0;G:0;C:12;T:78;total:90 | iSNV |
| F40 | F40-15 | 8693 | NS5 | 0.1136 | A:0;G:0;C:39;T:5;total:44 | iSNV |
| F40 | F40-15 | 9359 | NS5 | 0.0854 | A:0;G:0;C:10;T:107;total:117 | iSNV |
| F40 | F40-16 | 469 | C | 0.0243 | A:0;G:0;C:280;T:7;total:287 | iSNV |
| F40 | F40-16 | 998 | E | 0.0731 | A:0;G:0;C:152;T:12;total:164 | iSNV |
| F40 | F40-16 | 1116 | E | 0.0673 | A:180;G:13;C:0;T:0;total:193 | iSNV |
| F40 | F40-16 | 1218 | E | 1 | A:0;G:0;C:0;T:201;total:201 | SNP |
| F40 | F40-16 | 2076 | E | 0.1078 | A:91;G:11;C:0;T:0;total:102 | iSNV |
| F40 | F40-16 | 2274 | E | 0.0416 | A:0;G:115;C:0;T:5;total:120 | iSNV |
| F40 | F40-16 | 2362 | E | 0.4473 | A:0;G:42;C:0;T:34;total:76 | iSNV |
| F40 | F40-16 | 2906 | NS1 | 0.093 | A:0;G:0;C:234;T:24;total:258 | iSNV |
| F40 | F40-16 | 3161 | NS1 | 0.0213 | A:321;G:7;C:0;T:0;total:328 | iSNV |
| F40 | F40-16 | 3177 | NS1 | 0.1044 | A:40;G:343;C:0;T:0;total:383 | iSNV |
| F40 | F40-16 | 3396 | NS1 | 0.0434 | A:0;G:11;C:0;T:242;total:253 | iSNV |
| F40 | F40-16 | 3869 | NS2A | 0.884 | A:0;G:0;C:34;T:259;total:293 | iSNV |
| F40 | F40-16 | 3965 | NS2A | 0.1165 | A:0;G:0;C:235;T:31;total:266 | iSNV |
| F40 | F40-16 | 4136 | NS2A | 0.0902 | A:121;G:12;C:0;T:0;total:133 | iSNV |
| F40 | F40-16 | 4697 | NS3 | 0.0769 | A:0;G:0;C:17;T:204;total:221 | iSNV |
| F40 | F40-16 | 5312 | NS3 | 0.0514 | A:11;G:0;C:0;T:203;total:214 | iSNV |
| F40 | F40-16 | 5448 | NS3 | 0.0383 | A:0;G:0;C:326;T:13;total:339 | iSNV |
| F40 | F40-16 | 5736 | NS3 | 0.0339 | A:11;G:0;C:313;T:0;total:324 | iSNV |
| F40 | F40-16 | 5952 | NS3 | 0.0915 | A:0;G:0;C:248;T:25;total:273 | iSNV |
| F40 | F40-16 | 6287 | NS3 | 0.1066 | A:0;G:0;C:134;T:16;total:150 | iSNV |
| F40 | F40-16 | 6810 | NS4A | 0.0289 | A:0;G:201;C:0;T:6;total:207 | iSNV |
| F40 | F40-16 | 6849 | NS4A | 0.1575 | A:139;G:26;C:0;T:0;total:165 | iSNV |
| F40 | F40-16 | 6867 | NS4A | 0.1676 | A:28;G:139;C:0;T:0;total:167 | iSNV |
| F40 | F40-16 | 7167 | NS4A | 0.139 | A:26;G:161;C:0;T:0;total:187 | iSNV |
| F40 | F40-16 | 7633 | NS4B | 0.843 | A:0;G:0;C:41;T:220;total:261 | iSNV |
| F40 | F40-16 | 7735 | NS5 | 0.0436 | A:11;G:241;C:0;T:0;total:252 | iSNV |
| F40 | F40-16 | 8431 | NS5 | 0.1046 | A:29;G:0;C:248;T:0;total:277 | iSNV |
| F40 | F40-16 | 8444 | NS5 | 0.0404 | A:285;G:12;C:0;T:0;total:297 | iSNV |
| F40 | F40-16 | 8518 | NS5 | 0.0427 | A:224;G:0;C:0;T:10;total:234 | iSNV |
| F40 | F40-16 | 9293 | NS5 | 0.0476 | A:13;G:260;C:0;T:0;total:273 | iSNV |
| F40 | F40-16 | 9359 | NS5 | 0.0614 | A:0;G:0;C:21;T:321;total:342 | iSNV |
| F40 | F40-16 | 9818 | NS5 | 0.0328 | A:0;G:0;C:5;T:147;total:152 | iSNV |
| F40 | F40-16 | 10127 | NS5 | 0.0306 | A:0;G:0;C:158;T:5;total:163 | iSNV |
| F40 | F40-16 | 10259 | NS5 | 0.1306 | A:193;G:29;C:0;T:0;total:222 | iSNV |
| F40 | F40-16 | 10368 | NS5 | 0.1018 | A:22;G:0;C:0;T:194;total:216 | iSNV |
| F40 | F40-16 | 10428 | 3'-UTR | 0.3812 | A:1;G:0;C:98;T:61;total:160 | iSNV |
| F40 | F40-16 | 10666 | 3'-UTR | 0.0386 | A:174;G:7;C:0;T:0;total:181 | iSNV |
| F40 | F40-16 | 10718 | 3'-UTR | 0.0732 | A:0;G:0;C:177;T:14;total:191 | iSNV |
| F40 | F40-17 | 291 | C | 0.0312 | A:7;G:0;C:0;T:217;total:224 | iSNV |
| F40 | F40-17 | 645 | M | 0.3652 | A:139;G:80;C:0;T:0;total:219 | iSNV |
| F40 | F40-17 | 998 | E | 0.0287 | A:0;G:0;C:169;T:5;total:174 | iSNV |
| F40 | F40-17 | 1057 | E | 0.0549 | A:10;G:172;C:0;T:0;total:182 | iSNV |
| F40 | F40-17 | 1218 | E | 1 | A:0;G:0;C:0;T:210;total:210 | SNP |
| F40 | F40-17 | 1413 | E | 0.2028 | A:165;G:42;C:0;T:0;total:207 | iSNV |
| F40 | F40-17 | 1428 | E | 0.1319 | A:171;G:26;C:0;T:0;total:197 | iSNV |
| F40 | F40-17 | 1447 | E | 0.4222 | A:76;G:0;C:104;T:0;total:180 | iSNV |
| F40 | F40-17 | 1512 | E | 0.2056 | A:112;G:0;C:29;T:0;total:141 | iSNV |
| F40 | F40-17 | 1797 | E | 0.0432 | A:0;G:10;C:0;T:221;total:231 | iSNV |
| F40 | F40-17 | 2070 | E | 0.4285 | A:72;G:54;C:0;T:0;total:126 | iSNV |
| F40 | F40-17 | 2231 | E | 0.0379 | A:152;G:6;C:0;T:0;total:158 | iSNV |
| F40 | F40-17 | 2376 | E | 0.4337 | A:0;G:0;C:47;T:36;total:83 | iSNV |
| F40 | F40-17 | 2960 | NS1 | 0.1336 | A:0;G:0;C:201;T:31;total:232 | iSNV |
| F40 | F40-17 | 3002 | NS1 | 0.1847 | A:0;G:0;C:150;T:34;total:184 | iSNV |
| F40 | F40-17 | 3317 | NS1 | 0.0905 | A:0;G:0;C:241;T:24;total:265 | iSNV |
| F40 | F40-17 | 3341 | NS1 | 0.0303 | A:224;G:7;C:0;T:0;total:231 | iSNV |
| F40 | F40-17 | 3869 | NS2A | 0.9701 | A:0;G:0;C:9;T:292;total:301 | iSNV |
| F40 | F40-17 | 4091 | NS2A | 0.1368 | A:26;G:164;C:0;T:0;total:190 | iSNV |
| F40 | F40-17 | 4982 | NS3 | 0.0209 | A:5;G:0;C:0;T:234;total:239 | iSNV |
| F40 | F40-17 | 5060 | NS3 | 0.052 | A:0;G:0;C:182;T:10;total:192 | iSNV |
| F40 | F40-17 | 5327 | NS3 | 0.2653 | A:144;G:0;C:0;T:52;total:196 | iSNV |
| F40 | F40-17 | 5415 | NS3 | 0.1355 | A:0;G:0;C:236;T:37;total:273 | iSNV |
| F40 | F40-17 | 5518 | NS3 | 0.1006 | A:259;G:29;C:0;T:0;total:288 | iSNV |
| F40 | F40-17 | 5575 | NS3 | 0.0526 | A:0;G:0;C:234;T:13;total:247 | iSNV |
| F40 | F40-17 | 5705 | NS3 | 0.0607 | A:263;G:17;C:0;T:0;total:280 | iSNV |
| F40 | F40-17 | 6714 | NS4A | 0.0517 | A:9;G:165;C:0;T:0;total:174 | iSNV |
| F40 | F40-17 | 6867 | NS4A | 0.0335 | A:6;G:173;C:0;T:0;total:179 | iSNV |
| F40 | F40-17 | 6970 | NS4A | 0.2087 | A:144;G:0;C:38;T:0;total:182 | iSNV |
| F40 | F40-17 | 7109 | NS4A | 0.0255 | A:0;G:0;C:191;T:5;total:196 | iSNV |
| F40 | F40-17 | 7151 | NS4A | 0.417 | A:0;G:0;C:116;T:83;total:199 | iSNV |
| F40 | F40-17 | 7167 | NS4A | 0.0702 | A:13;G:172;C:0;T:0;total:185 | iSNV |
| F40 | F40-17 | 7633 | NS4B | 0.951 | A:0;G:0;C:13;T:252;total:265 | iSNV |
| F40 | F40-17 | 7657 | NS4B | 0.0245 | A:278;G:7;C:0;T:0;total:285 | iSNV |
| F40 | F40-17 | 8430 | NS5 | 0.2154 | A:0;G:0;C:244;T:67;total:311 | iSNV |
| F40 | F40-17 | 8648 | NS5 | 0.0547 | A:0;G:0;C:138;T:8;total:146 | iSNV |
| F40 | F40-17 | 9698 | NS5 | 0.0564 | A:7;G:117;C:0;T:0;total:124 | iSNV |
| F40 | F40-17 | 10364 | NS5 | 0.4444 | A:0;G:0;C:120;T:96;total:216 | iSNV |
| F40 | F40-17 | 10376 | NS5 | 0.198 | A:41;G:166;C:0;T:0;total:207 | iSNV |
| F40 | F40-17 | 10428 | 3'-UTR | 0.127 | A:0;G:0;C:158;T:23;total:181 | iSNV |

| | | | | | | |
|---|---|---|---|---|---|---|
| F40 | F40-18 | 780 | M | 0.0218 | A:358;G:8;C:0;T:0;total:366 | iSNV |
| F40 | F40-18 | 781 | M | 0.0215 | A:0;G:364;C:0;T:8;total:372 | iSNV |
| F40 | F40-18 | 948 | M | 0.1554 | A:0;G:0;C:239;T:44;total:283 | iSNV |
| F40 | F40-18 | 998 | E | 0.1792 | A:0;G:0;C:229;T:50;total:279 | iSNV |
| F40 | F40-18 | 1218 | E | 0.9912 | A:0;G:0;C:3;T:337;total:340 | SNP |
| F40 | F40-18 | 1413 | E | 0.7724 | A:97;G:329;C:0;T:0;total:426 | iSNV |
| F40 | F40-18 | 1512 | E | 0.7154 | A:78;G:0;C:196;T:0;total:274 | iSNV |
| F40 | F40-18 | 1562 | E | 0.1516 | A:235;G:42;C:0;T:0;total:277 | iSNV |
| F40 | F40-18 | 1797 | E | 0.2258 | A:0;G:82;C:0;T:281;total:363 | iSNV |
| F40 | F40-18 | 2181 | E | 0.0753 | A:319;G:26;C:0;T:0;total:345 | iSNV |
| F40 | F40-18 | 3660 | NS1 | 0.0215 | A:5;G:227;C:0;T:0;total:232 | iSNV |
| F40 | F40-18 | 3693 | NS1 | 0.0342 | A:0;G:0;C:9;T:254;total:263 | iSNV |
| F40 | F40-18 | 3746 | NS2A | 0.1673 | A:204;G:41;C:0;T:0;total:245 | iSNV |
| F40 | F40-18 | 3869 | NS2A | 0.8258 | A:0;G:0;C:103;T:488;total:591 | iSNV |
| F40 | F40-18 | 4091 | NS2A | 0.5647 | A:179;G:138;C:0;T:0;total:317 | iSNV |
| F40 | F40-18 | 4697 | NS3 | 0.1406 | A:0;G:0;C:72;T:440;total:512 | iSNV |
| F40 | F40-18 | 5072 | NS3 | 0.103 | A:40;G:348;C:0;T:0;total:388 | iSNV |
| F40 | F40-18 | 5327 | NS3 | 0.087 | A:346;G:0;C:0;T:33;total:379 | iSNV |
| F40 | F40-18 | 5952 | NS3 | 0.152 | A:0;G:0;C:530;T:95;total:625 | iSNV |
| F40 | F40-18 | 6692 | NS4A | 0.0408 | A:0;G:0;C:11;T:258;total:269 | iSNV |
| F40 | F40-18 | 6740 | NS4A | 0.0958 | A:0;G:0;C:302;T:32;total:334 | iSNV |
| F40 | F40-18 | 6867 | NS4A | 0.1955 | A:62;G:255;C:0;T:0;total:317 | iSNV |
| F40 | F40-18 | 6900 | NS4A | 0.0433 | A:15;G:331;C:0;T:0;total:346 | iSNV |
| F40 | F40-18 | 6969 | NS4A | 0.0445 | A:365;G:17;C:0;T:0;total:382 | iSNV |
| F40 | F40-18 | 7561 | NS4B | 0.077 | A:0;G:0;C:34;T:407;total:441 | iSNV |
| F40 | F40-18 | 7633 | NS4B | 0.8708 | A:0;G:0;C:65;T:438;total:503 | iSNV |
| F40 | F40-18 | 7859 | NS5 | 0.0211 | A:510;G:11;C:0;T:0;total:521 | iSNV |
| F40 | F40-18 | 8266 | NS5 | 0.027 | A:0;G:0;C:13;T:468;total:481 | iSNV |
| F40 | F40-18 | 8430 | NS5 | 0.7893 | A:0;G:0;C:106;T:397;total:503 | iSNV |
| F40 | F40-18 | 9359 | NS5 | 0.1533 | A:0;G:0;C:102;T:563;total:665 | iSNV |
| F40 | F40-18 | 9764 | NS5 | 0.2621 | A:0;G:0;C:197;T:70;total:267 | iSNV |
| F40 | F40-18 | 10259 | NS5 | 0.1607 | A:355;G:68;C:0;T:0;total:423 | iSNV |
| F40 | F40-18 | 10295 | NS5 | 0.1726 | A:0;G:0;C:369;T:77;total:446 | iSNV |
| F40 | F40-18 | 10376 | NS5 | 0.7648 | A:286;G:88;C:0;T:0;total:374 | iSNV |
| F40 | F40-18 | 10428 | 3'-UTR | 0.2158 | A:0;G:0;C:258;T:71;total:329 | iSNV |
| F40 | F40-18 | 10451 | 3'-UTR | 0.0992 | A:0;G:0;C:254;T:28;total:282 | iSNV |
| F40 | F40-18 | 10566 | 3'-UTR | 0.1541 | A:0;G:0;C:225;T:41;total:266 | iSNV |
| F40 | F40-19 | 869 | M | 0.488 | A:102;G:0;C:0;T:107;total:209 | iSNV |
| F40 | F40-19 | 997 | E | 0.0632 | A:11;G:163;C:0;T:0;total:174 | iSNV |
| F40 | F40-19 | 998 | E | 0.0454 | A:0;G:0;C:168;T:8;total:176 | iSNV |
| F40 | F40-19 | 1218 | E | 1 | A:0;G:0;C:0;T:208;total:208 | SNP |
| F40 | F40-19 | 1382 | E | 0.3843 | A:0;G:0;C:165;T:103;total:268 | iSNV |
| F40 | F40-19 | 1428 | E | 0.0334 | A:231;G:8;C:0;T:0;total:239 | iSNV |
| F40 | F40-19 | 1430 | E | 0.8946 | A:0;G:0;C:25;T:212;total:237 | iSNV |
| F40 | F40-19 | 1797 | E | 0.3454 | A:76;G:0;C:0;T:144;total:220 | iSNV |
| F40 | F40-19 | 1911 | E | 0.0406 | A:118;G:5;C:0;T:0;total:123 | iSNV |
| F40 | F40-19 | 3456 | NS1 | 0.0375 | A:128;G:5;C:0;T:0;total:133 | iSNV |
| F40 | F40-19 | 3869 | NS2A | 0.925 | A:0;G:0;C:24;T:296;total:320 | iSNV |
| F40 | F40-19 | 4034 | NS2A | 0.0738 | A:188;G:15;C:0;T:0;total:203 | iSNV |
| F40 | F40-19 | 4538 | NS2B | 0.4134 | A:0;G:0;C:183;T:129;total:312 | iSNV |
| F40 | F40-19 | 4572 | NS2B | 0.0231 | A:8;G:0;C:0;T:338;total:346 | iSNV |
| F40 | F40-19 | 5313 | NS3 | 0.0476 | A:0;G:0;C:10;T:200;total:210 | iSNV |
| F40 | F40-19 | 5598 | NS3 | 0.0842 | A:23;G:250;C:0;T:0;total:273 | iSNV |
| F40 | F40-19 | 5707 | NS3 | 0.0731 | A:355;G:28;C:0;T:0;total:383 | iSNV |
| F40 | F40-19 | 5952 | NS3 | 0.0539 | A:0;G:0;C:333;T:19;total:352 | iSNV |
| F40 | F40-19 | 6336 | NS3 | 0.0428 | A:134;G:0;C:0;T:6;total:140 | iSNV |
| F40 | F40-19 | 6413 | NS3 | 0.0257 | A:0;G:0;C:6;T:227;total:233 | iSNV |
| F40 | F40-19 | 6714 | NS4A | 0.0507 | A:10;G:187;C:0;T:0;total:197 | iSNV |
| F40 | F40-19 | 6867 | NS4A | 0.3157 | A:54;G:117;C:0;T:0;total:171 | iSNV |
| F40 | F40-19 | 8015 | NS5 | 0.0901 | A:31;G:313;C:0;T:0;total:344 | iSNV |
| F40 | F40-19 | 9359 | NS5 | 0.0393 | A:0;G:0;C:14;T:342;total:356 | iSNV |
| F40 | F40-19 | 10259 | NS5 | 0.0597 | A:236;G:15;C:0;T:0;total:251 | iSNV |
| F40 | F40-19 | 10310 | NS5 | 0.0463 | A:0;G:0;C:247;T:12;total:259 | iSNV |
| F40 | F40-19 | 10358 | NS5 | 0.4917 | A:0;G:0;C:123;T:119;total:242 | iSNV |
| F40 | F40-19 | 10402 | 3'-UTR | 0.1043 | A:0;G:0;C:19;T:163;total:182 | iSNV |
| F40 | F40-19 | 10428 | 3'-UTR | 0.5053 | A:0;G:0;C:94;T:96;total:190 | iSNV |
| F40 | F40-19 | 10566 | 3'-UTR | 0.0419 | A:0;G:0;C:160;T:7;total:167 | iSNV |
| F40 | F40-2 | 167 | C | 0.0247 | A:827;G:21;C:0;T:0;total:848 | iSNV |
| F40 | F40-2 | 434 | C | 0.5124 | A:414;G:394;C:0;T:0;total:808 | iSNV |
| F40 | F40-2 | 563 | M | 0.1329 | A:77;G:502;C:0;T:0;total:579 | iSNV |
| F40 | F40-2 | 998 | E | 0.025 | A:0;G:0;C:505;T:13;total:518 | iSNV |
| F40 | F40-2 | 1166 | E | 0.3789 | A:0;G:0;C:255;T:418;total:673 | iSNV |
| F40 | F40-2 | 1218 | E | 0.819 | A:0;G:0;C:101;T:457;total:558 | iSNV |
| F40 | F40-2 | 1295 | E | 0.2841 | A:446;G:0;C:0;T:177;total:623 | iSNV |
| F40 | F40-2 | 1413 | E | 0.2529 | A:514;G:174;C:0;T:0;total:688 | iSNV |
| F40 | F40-2 | 1430 | E | 0.5208 | A:0;G:0;C:312;T:339;total:651 | iSNV |
| F40 | F40-2 | 1446 | E | 0.0216 | A:14;G:634;C:0;T:0;total:648 | iSNV |
| F40 | F40-2 | 1453 | E | 0.1889 | A:116;G:0;C:0;T:498;total:614 | iSNV |
| F40 | F40-2 | 1512 | E | 0.0229 | A:468;G:11;C:0;T:0;total:479 | iSNV |
| F40 | F40-2 | 1772 | E | 0.1726 | A:0;G:115;C:0;T:551;total:666 | iSNV |
| F40 | F40-2 | 1911 | E | 0.0884 | A:371;G:36;C:0;T:0;total:407 | iSNV |
| F40 | F40-2 | 2274 | E | 0.5199 | A:0;G:194;C:0;T:210;total:404 | iSNV |

| | | | | | | |
|---|---|---|---|---|---|---|
| F40 | F40-2 | 2372 | E | 0.1644 | A:0;G:0;C:254;T:50;total:304 | iSNV |
| F40 | F40-2 | 2531 | NS1 | 0.1644 | A:0;G:0;C:88;T:447;total:535 | iSNV |
| F40 | F40-2 | 2712 | NS1 | 0.0252 | A:0;G:0;C:889;T:23;total:912 | iSNV |
| F40 | F40-2 | 3572 | NS1 | 0.1739 | A:0;G:0;C:456;T:96;total:552 | iSNV |
| F40 | F40-2 | 3737 | NS2A | 0.0481 | A:0;G:0;C:455;T:23;total:478 | iSNV |
| F40 | F40-2 | 3869 | NS2A | 0.8373 | A:0;G:0;C:146;T:751;total:897 | iSNV |
| F40 | F40-2 | 3962 | NS2A | 0.0981 | A:0;G:0;C:717;T:78;total:795 | iSNV |
| F40 | F40-2 | 4058 | NS2A | 0.2532 | A:0;G:0;C:401;T:136;total:537 | iSNV |
| F40 | F40-2 | 4160 | NS2A | 0.08 | A:0;G:0;C:299;T:26;total:325 | iSNV |
| F40 | F40-2 | 4697 | NS3 | 0.0249 | A:0;G:0;C:19;T:743;total:762 | iSNV |
| F40 | F40-2 | 4712 | NS3 | 0.1338 | A:108;G:699;C:0;T:0;total:807 | iSNV |
| F40 | F40-2 | 5311 | NS3 | 0.1258 | A:0;G:0;C:632;T:91;total:723 | iSNV |
| F40 | F40-2 | 5911 | NS3 | 0.0971 | A:0;G:0;C:81;T:753;total:834 | iSNV |
| F40 | F40-2 | 5952 | NS3 | 0.0346 | A:0;G:0;C:892;T:32;total:924 | iSNV |
| F40 | F40-2 | 5987 | NS3 | 0.1371 | A:0;G:0;C:629;T:100;total:729 | iSNV |
| F40 | F40-2 | 6355 | NS3 | 0.1561 | A:0;G:0;C:84;T:454;total:538 | iSNV |
| F40 | F40-2 | 6867 | NS4A | 0.3397 | A:177;G:344;C:0;T:0;total:521 | iSNV |
| F40 | F40-2 | 6969 | NS4A | 0.1384 | A:560;G:90;C:0;T:0;total:650 | iSNV |
| F40 | F40-2 | 6970 | NS4A | 0.148 | A:558;G:0;C:97;T:0;total:655 | iSNV |
| F40 | F40-2 | 6996 | NS4A | 0.1333 | A:86;G:0;C:0;T:559;total:645 | iSNV |
| F40 | F40-2 | 7555 | NS4B | 0.1242 | A:87;G:613;C:0;T:0;total:700 | iSNV |
| F40 | F40-2 | 7633 | NS4B | 0.6195 | A:0;G:1;C:317;T:515;total:833 | iSNV |
| F40 | F40-2 | 7657 | NS4B | 0.0328 | A:824;G:28;C:0;T:0;total:852 | iSNV |
| F40 | F40-2 | 7744 | NS5 | 0.0788 | A:748;G:64;C:0;T:0;total:812 | iSNV |
| F40 | F40-2 | 7963 | NS5 | 0.1147 | A:872;G:113;C:0;T:0;total:985 | iSNV |
| F40 | F40-2 | 8249 | NS5 | 0.0244 | A:0;G:0;C:17;T:678;total:695 | iSNV |
| F40 | F40-2 | 8282 | NS5 | 0.1122 | A:0;G:0;C:77;T:609;total:686 | iSNV |
| F40 | F40-2 | 8570 | NS5 | 0.02 | A:0;G:0;C:16;T:782;total:798 | iSNV |
| F40 | F40-2 | 8900 | NS5 | 0.1011 | A:649;G:0;C:73;T:0;total:722 | iSNV |
| F40 | F40-2 | 9359 | NS5 | 0.0339 | A:0;G:0;C:33;T:938;total:971 | iSNV |
| F40 | F40-2 | 9446 | NS5 | 0.0921 | A:759;G:77;C:0;T:0;total:836 | iSNV |
| F40 | F40-2 | 9585 | NS5 | 0.042 | A:0;G:21;C:0;T:478;total:499 | iSNV |
| F40 | F40-2 | 10069 | NS5 | 0.0295 | A:0;G:0;C:525;T:16;total:541 | iSNV |
| F40 | F40-2 | 10088 | NS5 | 0.0422 | A:23;G:521;C:0;T:0;total:544 | iSNV |
| F40 | F40-2 | 10259 | NS5 | 0.0271 | A:752;G:21;C:0;T:0;total:773 | iSNV |
| F40 | F40-2 | 10376 | NS5 | 0.3458 | A:276;G:522;C:0;T:0;total:798 | iSNV |
| F40 | F40-2 | 10428 | 3'-UTR | 0.247 | A:0;G:2;C:510;T:168;total:680 | iSNV |
| F40 | F40-2 | 10566 | 3'-UTR | 0.0295 | A:0;G:0;C:525;T:16;total:541 | iSNV |
| F40 | F40-2 | 10596 | 3'-UTR | 0.0459 | A:0;G:0;C:26;T:540;total:566 | iSNV |
| F40 | F40-20 | 456 | C | 0.0552 | A:9;G:154;C:0;T:0;total:163 | iSNV |
| F40 | F40-20 | 782 | M | 0.0793 | A:0;G:0;C:10;T:116;total:126 | iSNV |
| F40 | F40-20 | 906 | M | 0.0957 | A:85;G:9;C:0;T:0;total:94 | iSNV |
| F40 | F40-20 | 998 | E | 0.3023 | A:0;G:0;C:60;T:26;total:86 | iSNV |
| F40 | F40-20 | 1117 | E | 0.732 | A:26;G:71;C:0;T:0;total:97 | iSNV |
| F40 | F40-20 | 1218 | E | 1 | A:0;G:0;C:0;T:103;total:103 | SNP |
| F40 | F40-20 | 1382 | E | 0.0797 | A:0;G:0;C:127;T:11;total:138 | iSNV |
| F40 | F40-20 | 1721 | E | 0.3902 | A:50;G:32;C:0;T:0;total:82 | iSNV |
| F40 | F40-20 | 2078 | E | 0.1111 | A:0;G:0;C:64;T:8;total:72 | iSNV |
| F40 | F40-20 | 2274 | E | 0.0853 | A:0;G:75;C:0;T:7;total:82 | iSNV |
| F40 | F40-20 | 2369 | E | 0.5193 | A:27;G:25;C:0;T:0;total:52 | iSNV |
| F40 | F40-20 | 2531 | NS1 | 0.2531 | A:0;G:0;C:20;T:59;total:79 | iSNV |
| F40 | F40-20 | 2790 | NS1 | 0.0823 | A:0;G:0;C:14;T:156;total:170 | iSNV |
| F40 | F40-20 | 3183 | NS1 | 0.0258 | A:6;G:226;C:0;T:0;total:232 | iSNV |
| F40 | F40-20 | 3869 | NS2A | 0.8307 | A:0;G:0;C:31;T:152;total:183 | iSNV |
| F40 | F40-20 | 3929 | NS2A | 0.4132 | A:0;G:0;C:115;T:81;total:196 | iSNV |
| F40 | F40-20 | 4319 | NS2B | 0.1824 | A:27;G:121;C:0;T:0;total:148 | iSNV |
| F40 | F40-20 | 4697 | NS3 | 0.1503 | A:0;G:0;C:23;T:130;total:153 | iSNV |
| F40 | F40-20 | 5376 | NS3 | 0.1527 | A:122;G:0;C:0;T:22;total:144 | iSNV |
| F40 | F40-20 | 5602 | NS3 | 0.0629 | A:0;G:0;C:119;T:8;total:127 | iSNV |
| F40 | F40-20 | 5952 | NS3 | 0.1856 | A:0;G:0;C:136;T:31;total:167 | iSNV |
| F40 | F40-20 | 6867 | NS4A | 0.1951 | A:24;G:99;C:0;T:0;total:123 | iSNV |
| F40 | F40-20 | 6970 | NS4A | 0.0683 | A:109;G:0;C:8;T:0;total:117 | iSNV |
| F40 | F40-20 | 7004 | NS4A | 0.0431 | A:0;G:0;C:111;T:5;total:116 | iSNV |
| F40 | F40-20 | 7633 | NS4B | 0.9338 | A:0;G:0;C:10;T:141;total:151 | iSNV |
| F40 | F40-20 | 7990 | NS5 | 0.0638 | A:132;G:9;C:0;T:0;total:141 | iSNV |
| F40 | F40-20 | 8048 | NS5 | 0.0359 | A:0;G:0;C:161;T:6;total:167 | iSNV |
| F40 | F40-20 | 8217 | NS5 | 0.0292 | A:166;G:5;C:0;T:0;total:171 | iSNV |
| F40 | F40-20 | 9005 | NS5 | 0.0547 | A:0;G:0;C:138;T:8;total:146 | iSNV |
| F40 | F40-20 | 9317 | NS5 | 0.0434 | A:0;G:0;C:8;T:176;total:184 | iSNV |
| F40 | F40-20 | 9359 | NS5 | 0.1981 | A:0;G:0;C:42;T:170;total:212 | iSNV |
| F40 | F40-20 | 10109 | NS5 | 0.1022 | A:9;G:79;C:0;T:0;total:88 | iSNV |
| F40 | F40-20 | 10259 | NS5 | 0.3142 | A:72;G:33;C:0;T:0;total:105 | iSNV |
| F40 | F40-20 | 10428 | 3'-UTR | 0.2021 | A:0;G:0;C:75;T:19;total:94 | iSNV |
| F40 | F40-20 | 10566 | 3'-UTR | 0.1617 | A:0;G:0;C:57;T:11;total:68 | iSNV |
| F40 | F40-21 | 443 | C | 0.0775 | A:0;G:333;C:0;T:28;total:361 | iSNV |
| F40 | F40-21 | 646 | M | 0.0521 | A:0;G:0;C:291;T:16;total:307 | iSNV |
| F40 | F40-21 | 1218 | E | 0.996 | A:0;G:0;C:1;T:246;total:247 | SNP |
| F40 | F40-21 | 1459 | E | 0.1466 | A:33;G:0;C:192;T:0;total:225 | iSNV |
| F40 | F40-21 | 1708 | E | 0.1111 | A:22;G:176;C:0;T:0;total:198 | iSNV |
| F40 | F40-21 | 1766 | E | 0.027 | A:0;G:0;C:6;T:216;total:222 | iSNV |
| F40 | F40-21 | 1797 | E | 0.2677 | A:25;G:64;C:0;T:150;total:239 | iSNV |
| F40 | F40-21 | 2556 | NS1 | 0.2797 | A:54;G:139;C:0;T:0;total:193 | iSNV |

| | | | | | | |
|---|---|---|---|---|---|---|
| F40 | F40-21 | 2960 | NS1 | 0.1522 | A:0;G:0;C:245;T:44;total:289 | iSNV |
| F40 | F40-21 | 3146 | NS1 | 0.1222 | A:0;G:0;C:402;T:56;total:458 | iSNV |
| F40 | F40-21 | 3176 | NS1 | 0.1329 | A:411;G:63;C:0;T:0;total:474 | iSNV |
| F40 | F40-21 | 3661 | NS1 | 0.0652 | A:0;G:0;C:9;T:129;total:138 | iSNV |
| F40 | F40-21 | 3776 | NS2A | 0.0588 | A:13;G:0;C:0;T:208;total:221 | iSNV |
| F40 | F40-21 | 3869 | NS2A | 0.995 | A:0;G:0;C:2;T:393;total:395 | SNP |
| F40 | F40-21 | 4093 | NS2A | 0.1 | A:198;G:22;C:0;T:0;total:220 | iSNV |
| F40 | F40-21 | 4666 | NS3 | 0.0363 | A:0;G:0;C:212;T:8;total:220 | iSNV |
| F40 | F40-21 | 5723 | NS3 | 0.0439 | A:0;G:0;C:17;T:370;total:387 | iSNV |
| F40 | F40-21 | 6004 | NS3 | 0.3757 | A:0;G:0;C:206;T:124;total:330 | iSNV |
| F40 | F40-21 | 6089 | NS3 | 0.133 | A:0;G:0;C:189;T:29;total:218 | iSNV |
| F40 | F40-21 | 6107 | NS3 | 0.0723 | A:0;G:0;C:218;T:17;total:235 | iSNV |
| F40 | F40-21 | 6206 | NS3 | 0.0693 | A:0;G:0;C:188;T:14;total:202 | iSNV |
| F40 | F40-21 | 6969 | NS4A | 0.0307 | A:221;G:7;C:0;T:0;total:228 | iSNV |
| F40 | F40-21 | 7609 | NS4B | 0.0704 | A:21;G:277;C:0;T:0;total:298 | iSNV |
| F40 | F40-21 | 7633 | NS4B | 0.6 | A:0;G:0;C:124;T:186;total:310 | iSNV |
| F40 | F40-21 | 7644 | NS4B | 0.0462 | A:309;G:0;C:15;T:0;total:324 | iSNV |
| F40 | F40-21 | 7768 | NS5 | 0.023 | A:0;G:0;C:254;T:6;total:260 | iSNV |
| F40 | F40-21 | 8194 | NS5 | 0.0588 | A:240;G:15;C:0;T:0;total:255 | iSNV |
| F40 | F40-21 | 9065 | NS5 | 0.3407 | A:77;G:149;C:0;T:0;total:226 | iSNV |
| F40 | F40-21 | 9542 | NS5 | 0.0357 | A:0;G:0;C:189;T:7;total:196 | iSNV |
| F40 | F40-21 | 10217 | NS5 | 0.0326 | A:0;G:0;C:237;T:8;total:245 | iSNV |
| F40 | F40-21 | 10334 | NS5 | 0.0733 | A:1;G:0;C:277;T:22;total:300 | iSNV |
| F40 | F40-21 | 10358 | NS5 | 0.2359 | A:0;G:0;C:204;T:63;total:267 | iSNV |
| F40 | F40-21 | 10428 | 3'-UTR | 0.3423 | A:0;G:0;C:146;T:76;total:222 | iSNV |
| F40 | F40-21 | 10447 | 3'-UTR | 0.059 | A:0;G:0;C:207;T:13;total:220 | iSNV |
| F40 | F40-21 | 10470 | 3'-UTR | 0.1226 | A:186;G:26;C:0;T:0;total:212 | iSNV |
| F40 | F40-21 | 10513 | 3'-UTR | 0.1404 | A:0;G:0;C:33;T:202;total:235 | iSNV |
| F40 | F40-21 | 10578 | 3'-UTR | 0.4147 | A:0;G:0;C:90;T:127;total:217 | iSNV |
| F40 | F40-22 | 347 | C | 0.0489 | A:0;G:0;C:272;T:14;total:286 | iSNV |
| F40 | F40-22 | 998 | E | 0.5642 | A:0;G:0;C:68;T:88;total:156 | iSNV |
| F40 | F40-22 | 1117 | E | 0.0503 | A:151;G:8;C:0;T:0;total:159 | iSNV |
| F40 | F40-22 | 1218 | E | 0.9902 | A:0;G:0;C:2;T:201;total:203 | SNP |
| F40 | F40-22 | 1382 | E | 0.0638 | A:0;G:0;C:176;T:12;total:188 | iSNV |
| F40 | F40-22 | 1428 | E | 0.4779 | A:83;G:76;C:0;T:0;total:159 | iSNV |
| F40 | F40-22 | 1461 | E | 0.136 | A:0;G:0;C:20;T:127;total:147 | iSNV |
| F40 | F40-22 | 1797 | E | 0.0346 | A:0;G:7;C:0;T:195;total:202 | iSNV |
| F40 | F40-22 | 2274 | E | 0.1379 | A:20;G:125;C:0;T:0;total:145 | iSNV |
| F40 | F40-22 | 2792 | NS1 | 0.04 | A:0;G:0;C:240;T:10;total:250 | iSNV |
| F40 | F40-22 | 3137 | NS1 | 0.0271 | A:0;G:0;C:322;T:9;total:331 | iSNV |
| F40 | F40-22 | 3869 | NS2A | 0.5051 | A:0;G:0;C:148;T:151;total:299 | iSNV |
| F40 | F40-22 | 4319 | NS2B | 0.1 | A:19;G:171;C:0;T:0;total:190 | iSNV |
| F40 | F40-22 | 4468 | NS2B | 0.4268 | A:70;G:0;C:0;T:94;total:164 | iSNV |
| F40 | F40-22 | 4697 | NS3 | 0.5328 | A:0;G:0;C:122;T:107;total:229 | iSNV |
| F40 | F40-22 | 5952 | NS3 | 0.4496 | A:0;G:0;C:153;T:125;total:278 | iSNV |
| F40 | F40-22 | 6010 | NS3 | 0.0313 | A:216;G:7;C:0;T:0;total:223 | iSNV |
| F40 | F40-22 | 6867 | NS4A | 0.078 | A:11;G:130;C:0;T:0;total:141 | iSNV |
| F40 | F40-22 | 7211 | NS4A | 0.0824 | A:8;G:0;C:0;T:89;total:97 | iSNV |
| F40 | F40-22 | 7264 | NS4A | 0.0982 | A:0;G:0;C:101;T:11;total:112 | iSNV |
| F40 | F40-22 | 7633 | NS4B | 0.843 | A:0;G:0;C:38;T:204;total:242 | iSNV |
| F40 | F40-22 | 7644 | NS4B | 0.0237 | A:247;G:0;C:6;T:0;total:253 | iSNV |
| F40 | F40-22 | 7784 | NS5 | 0.1162 | A:1;G:0;C:189;T:25;total:215 | iSNV |
| F40 | F40-22 | 8456 | NS5 | 0.0711 | A:274;G:0;C:21;T:0;total:295 | iSNV |
| F40 | F40-22 | 9041 | NS5 | 0.0966 | A:0;G:1;C:186;T:20;total:207 | iSNV |
| F40 | F40-22 | 9245 | NS5 | 0.3307 | A:0;G:0;C:174;T:86;total:260 | iSNV |
| F40 | F40-22 | 9359 | NS5 | 0.4796 | A:0;G:0;C:165;T:179;total:344 | iSNV |
| F40 | F40-22 | 9634 | NS5 | 0.1171 | A:0;G:0;C:13;T:98;total:111 | iSNV |
| F40 | F40-22 | 10025 | NS5 | 0.0406 | A:8;G:189;C:0;T:0;total:197 | iSNV |
| F40 | F40-22 | 10259 | NS5 | 0.3815 | A:141;G:87;C:0;T:0;total:228 | iSNV |
| F40 | F40-22 | 10428 | 3'-UTR | 0.3312 | A:0;G:0;C:105;T:52;total:157 | iSNV |
| F40 | F40-22 | 10447 | 3'-UTR | 0.0444 | A:0;G:0;C:129;T:6;total:135 | iSNV |
| F40 | F40-22 | 10784 | 3'-UTR | 0.0373 | A:129;G:0;C:5;T:0;total:134 | iSNV |
| F40 | F40-23 | 353 | C | 0.5536 | A:121;G:150;C:0;T:0;total:271 | iSNV |
| F40 | F40-23 | 454 | C | 0.023 | A:0;G:0;C:7;T:297;total:304 | iSNV |
| F40 | F40-23 | 645 | M | 0.4438 | A:109;G:87;C:0;T:0;total:196 | iSNV |
| F40 | F40-23 | 803 | M | 0.0612 | A:0;G:138;C:0;T:9;total:147 | iSNV |
| F40 | F40-23 | 939 | M | 0.0597 | A:0;G:0;C:12;T:189;total:201 | iSNV |
| F40 | F40-23 | 998 | E | 0.4838 | A:0;G:0;C:96;T:90;total:186 | iSNV |
| F40 | F40-23 | 1007 | E | 0.1191 | A:0;G:0;C:170;T:23;total:193 | iSNV |
| F40 | F40-23 | 1117 | E | 0.5858 | A:70;G:99;C:0;T:0;total:169 | iSNV |
| F40 | F40-23 | 1218 | E | 0.3782 | A:0;G:0;C:120;T:73;total:193 | iSNV |
| F40 | F40-23 | 1342 | E | 0.0289 | A:0;G:0;C:235;T:7;total:242 | iSNV |
| F40 | F40-23 | 1512 | E | 0.5447 | A:51;G:61;C:0;T:0;total:112 | iSNV |
| F40 | F40-23 | 1745 | E | 0.0378 | A:7;G:178;C:0;T:0;total:185 | iSNV |
| F40 | F40-23 | 1797 | E | 0.0223 | A:0;G:5;C:1;T:218;total:224 | iSNV |
| F40 | F40-23 | 1911 | E | 0.056 | A:118;G:7;C:0;T:0;total:125 | iSNV |
| F40 | F40-23 | 2303 | E | 0.2384 | A:0;G:0;C:115;T:36;total:151 | iSNV |
| F40 | F40-23 | 3257 | NS1 | 0.0993 | A:0;G:0;C:33;T:299;total:332 | iSNV |
| F40 | F40-23 | 3869 | NS2A | 0.3937 | A:0;G:0;C:194;T:126;total:320 | iSNV |
| F40 | F40-23 | 4697 | NS3 | 0.0533 | A:0;G:0;C:12;T:213;total:225 | iSNV |
| F40 | F40-23 | 5826 | NS3 | 0.3469 | A:224;G:119;C:0;T:0;total:343 | iSNV |
| F40 | F40-23 | 5952 | NS3 | 0.0364 | A:0;G:0;C:344;T:13;total:357 | iSNV |

| | | | | | | |
|---|---|---|---|---|---|---|
| F40 | F40-23 | 6471 | NS4A | 0.041 | A:8;G:187;C:0;T:0;total:195 | iSNV |
| F40 | F40-23 | 6530 | NS4A | 0.0551 | A:137;G:8;C:0;T:0;total:145 | iSNV |
| F40 | F40-23 | 6867 | NS4A | 0.2267 | A:39;G:133;C:0;T:0;total:172 | iSNV |
| F40 | F40-23 | 6900 | NS4A | 0.3157 | A:0;G:117;C:0;T:54;total:171 | iSNV |
| F40 | F40-23 | 7633 | NS4B | 0.9596 | A:0;G:0;C:11;T:261;total:272 | iSNV |
| F40 | F40-23 | 8518 | NS5 | 0.0319 | A:212;G:0;C:0;T:7;total:219 | iSNV |
| F40 | F40-23 | 9359 | NS5 | 0.0443 | A:0;G:0;C:19;T:409;total:428 | iSNV |
| F40 | F40-23 | 10259 | NS5 | 0.5359 | A:97;G:112;C:0;T:0;total:209 | iSNV |
| F40 | F40-23 | 10428 | 3'-UTR | 0.3986 | A:0;G:0;C:92;T:61;total:153 | iSNV |
| F40 | F40-23 | 10447 | 3'-UTR | 0.4172 | A:0;G:0;C:88;T:63;total:151 | iSNV |
| F40 | F40-23 | 10566 | 3'-UTR | 0.0568 | A:0;G:0;C:166;T:10;total:176 | iSNV |
| F40 | F40-23 | 10804 | 3'-UTR | 0.2589 | A:0;G:0;C:103;T:36;total:139 | iSNV |
| F40 | F40-24 | 521 | M | 0.066 | A:0;G:0;C:99;T:7;total:106 | iSNV |
| F40 | F40-24 | 920 | M | 0.1466 | A:0;G:0;C:64;T:11;total:75 | iSNV |
| F40 | F40-24 | 998 | E | 0.2631 | A:0;G:0;C:56;T:20;total:76 | iSNV |
| F40 | F40-24 | 1413 | E | 0.1718 | A:53;G:11;C:0;T:0;total:64 | iSNV |
| F40 | F40-24 | 2076 | E | 0.3898 | A:36;G:23;C:0;T:0;total:59 | iSNV |
| F40 | F40-24 | 2890 | NS1 | 0.3482 | A:39;G:0;C:73;T:0;total:112 | iSNV |
| F40 | F40-24 | 3261 | NS1 | 0.0411 | A:163;G:7;C:0;T:0;total:170 | iSNV |
| F40 | F40-24 | 3669 | NS1 | 0.2931 | A:0;G:0;C:41;T:17;total:58 | iSNV |
| F40 | F40-24 | 3869 | NS2A | 0.8955 | A:0;G:0;C:16;T:137;total:153 | iSNV |
| F40 | F40-24 | 4070 | NS2A | 0.0446 | A:0;G:0;C:5;T:107;total:112 | iSNV |
| F40 | F40-24 | 4697 | NS3 | 0.0797 | A:0;G:0;C:11;T:127;total:138 | iSNV |
| F40 | F40-24 | 5465 | NS3 | 0.056 | A:202;G:12;C:0;T:0;total:214 | iSNV |
| F40 | F40-24 | 5737 | NS3 | 0.2807 | A:123;G:48;C:0;T:0;total:171 | iSNV |
| F40 | F40-24 | 5952 | NS3 | 0.0986 | A:0;G:0;C:137;T:15;total:152 | iSNV |
| F40 | F40-24 | 6867 | NS4A | 0.1162 | A:10;G:76;C:0;T:0;total:86 | iSNV |
| F40 | F40-24 | 6969 | NS4A | 0.2134 | A:70;G:19;C:0;T:0;total:89 | iSNV |
| F40 | F40-24 | 7177 | NS4A | 0.2441 | A:0;G:0;C:65;T:21;total:86 | iSNV |
| F40 | F40-24 | 8130 | NS5 | 0.0677 | A:0;G:0;C:165;T:12;total:177 | iSNV |
| F40 | F40-24 | 9183 | NS5 | 0.0514 | A:7;G:129;C:0;T:0;total:136 | iSNV |
| F40 | F40-24 | 9233 | NS5 | 0.1753 | A:0;G:0;C:127;T:27;total:154 | iSNV |
| F40 | F40-24 | 9284 | NS5 | 0.0714 | A:0;G:0;C:11;T:143;total:154 | iSNV |
| F40 | F40-24 | 9359 | NS5 | 0.0994 | A:0;G:0;C:18;T:163;total:181 | iSNV |
| F40 | F40-24 | 9902 | NS5 | 0.2873 | A:25;G:62;C:0;T:0;total:87 | iSNV |
| F40 | F40-24 | 9922 | NS5 | 0.1276 | A:0;G:0;C:82;T:12;total:94 | iSNV |
| F40 | F40-24 | 10253 | NS5 | 0.2539 | A:94;G:0;C:0;T:32;total:126 | iSNV |
| F40 | F40-24 | 10259 | NS5 | 0.0952 | A:114;G:12;C:0;T:0;total:126 | iSNV |
| F40 | F40-24 | 10376 | NS5 | 0.25 | A:28;G:84;C:0;T:0;total:112 | iSNV |
| F40 | F40-24 | 10428 | 3'-UTR | 0.5826 | A:0;G:0;C:43;T:60;total:103 | iSNV |
| F40 | F40-24 | 10566 | 3'-UTR | 0.0958 | A:0;G:0;C:66;T:7;total:73 | iSNV |
| F40 | F40-25 | 443 | C | 0.0779 | A:0;G:414;C:0;T:35;total:449 | iSNV |
| F40 | F40-25 | 491 | M | 0.0777 | A:0;G:0;C:37;T:439;total:476 | iSNV |
| F40 | F40-25 | 563 | M | 0.0239 | A:7;G:285;C:0;T:0;total:292 | iSNV |
| F40 | F40-25 | 998 | E | 0.0584 | A:0;G:0;C:290;T:18;total:308 | iSNV |
| F40 | F40-25 | 1117 | E | 0.0764 | A:278;G:23;C:0;T:0;total:301 | iSNV |
| F40 | F40-25 | 1428 | E | 0.1764 | A:322;G:69;C:0;T:0;total:391 | iSNV |
| F40 | F40-25 | 1433 | E | 0.1365 | A:0;G:53;C:0;T:335;total:388 | iSNV |
| F40 | F40-25 | 1450 | E | 0.0239 | A:367;G:0;C:9;T:0;total:376 | iSNV |
| F40 | F40-25 | 1772 | E | 0.0263 | A:0;G:9;C:0;T:333;total:342 | iSNV |
| F40 | F40-25 | 1789 | E | 0.0321 | A:11;G:0;C:0;T:331;total:342 | iSNV |
| F40 | F40-25 | 1804 | E | 0.1136 | A:40;G:312;C:0;T:0;total:352 | iSNV |
| F40 | F40-25 | 2372 | E | 0.0465 | A:0;G:0;C:164;T:8;total:172 | iSNV |
| F40 | F40-25 | 2828 | NS1 | 0.0538 | A:422;G:24;C:0;T:0;total:446 | iSNV |
| F40 | F40-25 | 2855 | NS1 | 0.1151 | A:57;G:438;C:0;T:0;total:495 | iSNV |
| F40 | F40-25 | 3356 | NS1 | 0.081 | A:27;G:306;C:0;T:0;total:333 | iSNV |
| F40 | F40-25 | 3573 | NS1 | 0.0766 | A:0;G:0;C:241;T:20;total:261 | iSNV |
| F40 | F40-25 | 3869 | NS2A | 0.9096 | A:0;G:1;C:40;T:401;total:442 | iSNV |
| F40 | F40-25 | 3875 | NS2A | 0.0427 | A:0;G:0;C:448;T:20;total:468 | iSNV |
| F40 | F40-25 | 3962 | NS2A | 0.021 | A:0;G:0;C:466;T:10;total:476 | iSNV |
| F40 | F40-25 | 4646 | NS3 | 0.4376 | A:0;G:0;C:275;T:214;total:489 | iSNV |
| F40 | F40-25 | 4697 | NS3 | 0.0367 | A:0;G:0;C:16;T:419;total:435 | iSNV |
| F40 | F40-25 | 4784 | NS3 | 0.0287 | A:0;G:405;C:12;T:0;total:417 | iSNV |
| F40 | F40-25 | 4898 | NS3 | 0.0253 | A:11;G:423;C:0;T:0;total:434 | iSNV |
| F40 | F40-25 | 5558 | NS3 | 0.0863 | A:43;G:455;C:0;T:0;total:498 | iSNV |
| F40 | F40-25 | 5952 | NS3 | 0.0753 | A:0;G:0;C:503;T:41;total:544 | iSNV |
| F40 | F40-25 | 5968 | NS3 | 0.4507 | A:238;G:290;C:0;T:0;total:528 | iSNV |
| F40 | F40-25 | 6215 | NS3 | 0.0555 | A:18;G:306;C:0;T:0;total:324 | iSNV |
| F40 | F40-25 | 6431 | NS3 | 0.0378 | A:0;G:0;C:356;T:14;total:370 | iSNV |
| F40 | F40-25 | 6818 | NS4A | 0.0668 | A:0;G:0;C:307;T:22;total:329 | iSNV |
| F40 | F40-25 | 6867 | NS4A | 0.0293 | A:8;G:265;C:0;T:0;total:273 | iSNV |
| F40 | F40-25 | 6970 | NS4A | 0.0394 | A:292;G:0;C:12;T:0;total:304 | iSNV |
| F40 | F40-25 | 6995 | NS4A | 0.0318 | A:304;G:0;C:0;T:10;total:314 | iSNV |
| F40 | F40-25 | 7034 | NS4A | 0.1762 | A:0;G:0;C:271;T:58;total:329 | iSNV |
| F40 | F40-25 | 7175 | NS4A | 0.0214 | A:0;G:0;C:6;T:274;total:280 | iSNV |
| F40 | F40-25 | 7256 | NS4A | 0.0315 | A:7;G:215;C:0;T:0;total:222 | iSNV |
| F40 | F40-25 | 7555 | NS4B | 0.0238 | A:9;G:369;C:0;T:0;total:378 | iSNV |
| F40 | F40-25 | 7561 | NS4B | 0.0301 | A:0;G:1;C:12;T:385;total:398 | iSNV |
| F40 | F40-25 | 7633 | NS4B | 0.8872 | A:0;G:0;C:50;T:393;total:443 | iSNV |
| F40 | F40-25 | 9359 | NS5 | 0.0754 | A:0;G:0;C:39;T:478;total:517 | iSNV |
| F40 | F40-25 | 9818 | NS5 | 0.1437 | A:0;G:0;C:46;T:274;total:320 | iSNV |
| F40 | F40-25 | 10259 | NS5 | 0.0739 | A:338;G:27;C:0;T:0;total:365 | iSNV |

| | | | | | | |
|---|---|---|---|---|---|---|
| F40 | F40-25 | 10307 | NS5 | 0.0444 | A:0;G:0;C:16;T:344;total:360 | iSNV |
| F40 | F40-25 | 10428 | 3'-UTR | 0.8125 | A:0;G:0;C:51;T:221;total:272 | iSNV |
| F40 | F40-25 | 10566 | 3'-UTR | 0.0581 | A:0;G:0;C:243;T:15;total:258 | iSNV |
| F40 | F40-26 | 694 | M | 0.626 | A:144;G:241;C:0;T:0;total:385 | iSNV |
| F40 | F40-26 | 1218 | E | 0.9822 | A:0;G:0;C:6;T:331;total:337 | SNP |
| F40 | F40-26 | 1428 | E | 0.8701 | A:43;G:288;C:0;T:0;total:331 | iSNV |
| F40 | F40-26 | 1797 | E | 0.0594 | A:0;G:23;C:0;T:364;total:387 | iSNV |
| F40 | F40-26 | 1911 | E | 0.0341 | A:198;G:7;C:0;T:0;total:205 | iSNV |
| F40 | F40-26 | 2372 | E | 0.0454 | A:0;G:0;C:189;T:9;total:198 | iSNV |
| F40 | F40-26 | 3093 | NS1 | 0.0462 | A:0;G:0;C:22;T:454;total:476 | iSNV |
| F40 | F40-26 | 3536 | NS1 | 0.0841 | A:0;G:0;C:283;T:26;total:309 | iSNV |
| F40 | F40-26 | 3869 | NS2A | 0.9728 | A:0;G:0;C:15;T:535;total:550 | iSNV |
| F40 | F40-26 | 4712 | NS3 | 0.0258 | A:12;G:452;C:0;T:0;total:464 | iSNV |
| F40 | F40-26 | 6080 | NS3 | 0.0771 | A:27;G:323;C:0;T:0;total:350 | iSNV |
| F40 | F40-26 | 6714 | NS4A | 0.0874 | A:30;G:313;C:0;T:0;total:343 | iSNV |
| F40 | F40-26 | 6795 | NS4A | 0.0321 | A:0;G:361;C:0;T:12;total:373 | iSNV |
| F40 | F40-26 | 6867 | NS4A | 0.0571 | A:18;G:297;C:0;T:0;total:315 | iSNV |
| F40 | F40-26 | 6947 | NS4A | 0.1149 | A:0;G:0;C:40;T:308;total:348 | iSNV |
| F40 | F40-26 | 7244 | NS4A | 0.1612 | A:0;G:0;C:40;T:208;total:248 | iSNV |
| F40 | F40-26 | 7561 | NS4B | 0.049 | A:0;G:0;C:20;T:388;total:408 | iSNV |
| F40 | F40-26 | 7633 | NS4B | 0.9187 | A:0;G:0;C:38;T:429;total:467 | iSNV |
| F40 | F40-26 | 7982 | NS5 | 0.0976 | A:462;G:0;C:0;T:50;total:512 | iSNV |
| F40 | F40-26 | 8000 | NS5 | 0.0226 | A:0;G:431;C:0;T:10;total:441 | iSNV |
| F40 | F40-26 | 8918 | NS5 | 0.024 | A:0;G:488;C:0;T:12;total:500 | iSNV |
| F40 | F40-26 | 9899 | NS5 | 0.0423 | A:0;G:0;C:339;T:15;total:354 | iSNV |
| F40 | F40-26 | 10061 | NS5 | 0.0293 | A:298;G:0;C:9;T:0;total:307 | iSNV |
| F40 | F40-26 | 10428 | 3'-UTR | 0.9488 | A:0;G:0;C:12;T:222;total:234 | iSNV |
| F40 | F40-27 | 662 | M | 0.044 | A:304;G:14;C:0;T:0;total:318 | iSNV |
| F40 | F40-27 | 716 | M | 0.0234 | A:0;G:374;C:0;T:9;total:383 | iSNV |
| F40 | F40-27 | 876 | M | 0.0204 | A:0;G:0;C:6;T:288;total:294 | iSNV |
| F40 | F40-27 | 906 | M | 0.05 | A:209;G:11;C:0;T:0;total:220 | iSNV |
| F40 | F40-27 | 937 | M | 0.0371 | A:0;G:0;C:9;T:233;total:242 | iSNV |
| F40 | F40-27 | 1218 | E | 0.9824 | A:0;G:0;C:5;T:279;total:284 | SNP |
| F40 | F40-27 | 1413 | E | 0.028 | A:311;G:9;C:1;T:0;total:321 | iSNV |
| F40 | F40-27 | 1428 | E | 0.0342 | A:282;G:10;C:0;T:0;total:292 | iSNV |
| F40 | F40-27 | 1473 | E | 0.0606 | A:217;G:14;C:0;T:0;total:231 | iSNV |
| F40 | F40-27 | 1512 | E | 0.0535 | A:212;G:12;C:0;T:0;total:224 | iSNV |
| F40 | F40-27 | 1797 | E | 0.1269 | A:0;G:41;C:0;T:282;total:323 | iSNV |
| F40 | F40-27 | 1877 | E | 0.0224 | A:0;G:0;C:218;T:5;total:223 | iSNV |
| F40 | F40-27 | 1911 | E | 0.0604 | A:171;G:11;C:0;T:0;total:182 | iSNV |
| F40 | F40-27 | 2274 | E | 0.4061 | A:0;G:136;C:0;T:93;total:229 | iSNV |
| F40 | F40-27 | 2372 | E | 0.041 | A:0;G:0;C:140;T:6;total:146 | iSNV |
| F40 | F40-27 | 2664 | NS1 | 0.5536 | A:0;G:0;C:175;T:217;total:392 | iSNV |
| F40 | F40-27 | 3089 | NS1 | 0.0574 | A:0;G:0;C:31;T:509;total:540 | iSNV |
| F40 | F40-27 | 3572 | NS1 | 0.0264 | A:0;G:0;C:331;T:9;total:340 | iSNV |
| F40 | F40-27 | 3869 | NS2A | 0.9398 | A:0;G:0;C:35;T:546;total:581 | iSNV |
| F40 | F40-27 | 4697 | NS3 | 0.0276 | A:0;G:0;C:14;T:493;total:507 | iSNV |
| F40 | F40-27 | 4974 | NS3 | 0.026 | A:11;G:412;C:0;T:0;total:423 | iSNV |
| F40 | F40-27 | 5597 | NS3 | 0.0217 | A:11;G:0;C:0;T:495;total:506 | iSNV |
| F40 | F40-27 | 5952 | NS3 | 0.0357 | A:0;G:0;C:539;T:20;total:559 | iSNV |
| F40 | F40-27 | 5996 | NS3 | 0.0451 | A:20;G:0;C:422;T:1;total:443 | iSNV |
| F40 | F40-27 | 5997 | NS3 | 0.0444 | A:430;G:0;C:20;T:0;total:450 | iSNV |
| F40 | F40-27 | 6062 | NS3 | 0.2069 | A:0;G:0;C:318;T:83;total:401 | iSNV |
| F40 | F40-27 | 6063 | NS3 | 0.0417 | A:0;G:0;C:390;T:17;total:407 | iSNV |
| F40 | F40-27 | 6182 | NS3 | 0.0214 | A:0;G:0;C:7;T:320;total:327 | iSNV |
| F40 | F40-27 | 6611 | NS4A | 0.2216 | A:0;G:0;C:86;T:302;total:388 | iSNV |
| F40 | F40-27 | 6714 | NS4A | 0.0656 | A:21;G:299;C:0;T:0;total:320 | iSNV |
| F40 | F40-27 | 6734 | NS4A | 0.2553 | A:0;G:0;C:84;T:245;total:329 | iSNV |
| F40 | F40-27 | 6867 | NS4A | 0.0773 | A:25;G:298;C:0;T:0;total:323 | iSNV |
| F40 | F40-27 | 7609 | NS4B | 0.0311 | A:17;G:529;C:0;T:0;total:546 | iSNV |
| F40 | F40-27 | 7626 | NS4B | 0.6795 | A:0;G:390;C:0;T:184;total:574 | iSNV |
| F40 | F40-27 | 7633 | NS4B | 0.5889 | A:0;G:0;C:236;T:338;total:574 | iSNV |
| F40 | F40-27 | 7697 | NS5 | 0.0406 | A:0;G:543;C:0;T:23;total:566 | iSNV |
| F40 | F40-27 | 8266 | NS5 | 0.0246 | A:14;G:0;C:0;T:554;total:568 | iSNV |
| F40 | F40-27 | 9359 | NS5 | 0.0304 | A:0;G:0;C:22;T:700;total:722 | iSNV |
| F40 | F40-27 | 9370 | NS5 | 0.0221 | A:16;G:0;C:0;T:705;total:721 | iSNV |
| F40 | F40-27 | 9634 | NS5 | 0.0443 | A:0;G:0;C:13;T:280;total:293 | iSNV |
| F40 | F40-27 | 9830 | NS5 | 0.0434 | A:0;G:0;C:13;T:286;total:299 | iSNV |
| F40 | F40-27 | 10259 | NS5 | 0.0349 | A:414;G:15;C:0;T:0;total:429 | iSNV |
| F40 | F40-27 | 10358 | NS5 | 0.2744 | A:0;G:0;C:275;T:104;total:379 | iSNV |
| F40 | F40-27 | 10373 | NS5 | 0.0421 | A:0;G:0;C:386;T:17;total:403 | iSNV |
| F40 | F40-27 | 10376 | NS5 | 0.0382 | A:15;G:377;C:0;T:0;total:392 | iSNV |
| F40 | F40-27 | 10419 | 3'-UTR | 0.0393 | A:0;G:0;C:342;T:14;total:356 | iSNV |
| F40 | F40-27 | 10428 | 3'-UTR | 0.396 | A:0;G:0;C:215;T:141;total:356 | iSNV |
| F40 | F40-27 | 10447 | 3'-UTR | 0.2345 | A:0;G:0;C:248;T:76;total:324 | iSNV |
| F40 | F40-27 | 10566 | 3'-UTR | 0.0371 | A:0;G:0;C:285;T:11;total:296 | iSNV |
| F40 | F40-28 | 230 | C | 0.8074 | A:0;G:0;C:306;T:73;total:379 | iSNV |
| F40 | F40-28 | 353 | C | 0.0313 | A:371;G:12;C:0;T:0;total:383 | iSNV |
| F40 | F40-28 | 645 | M | 0.0646 | A:275;G:19;C:0;T:0;total:294 | iSNV |
| F40 | F40-28 | 998 | E | 0.0615 | A:0;G:0;C:183;T:12;total:195 | iSNV |
| F40 | F40-28 | 1044 | E | 0.507 | A:0;G:0;C:109;T:106;total:215 | iSNV |
| F40 | F40-28 | 1087 | E | 0.0792 | A:186;G:16;C:0;T:0;total:202 | iSNV |

| | | | | | | |
|---|---|---|---|---|---|---|
| F40 | F40-28 | 1117 | E | 0.1012 | A:213;G:24;C:0;T:0;total:237 | iSNV |
| F40 | F40-28 | 1118 | E | 0.0209 | A:0;G:0;C:234;T:5;total:239 | iSNV |
| F40 | F40-28 | 1218 | E | 0.941 | A:0;G:0;C:15;T:239;total:254 | iSNV |
| F40 | F40-28 | 1428 | E | 0.875 | A:31;G:217;C:0;T:0;total:248 | iSNV |
| F40 | F40-28 | 1472 | E | 0.0531 | A:178;G:10;C:0;T:0;total:188 | iSNV |
| F40 | F40-28 | 1512 | E | 0.0764 | A:157;G:13;C:0;T:0;total:170 | iSNV |
| F40 | F40-28 | 1595 | E | 0.1337 | A:21;G:136;C:0;T:0;total:157 | iSNV |
| F40 | F40-28 | 1809 | E | 0.0343 | A:10;G:281;C:0;T:0;total:291 | iSNV |
| F40 | F40-28 | 3380 | NS1 | 0.1338 | A:0;G:0;C:36;T:233;total:269 | iSNV |
| F40 | F40-28 | 3869 | NS2A | 0.9377 | A:0;G:0;C:26;T:391;total:417 | iSNV |
| F40 | F40-28 | 4294 | NS2B | 0.0419 | A:251;G:11;C:0;T:0;total:262 | iSNV |
| F40 | F40-28 | 4348 | NS2B | 0.0204 | A:0;G:0;C:6;T:287;total:293 | iSNV |
| F40 | F40-28 | 5654 | NS3 | 0.0684 | A:0;G:0;C:354;T:26;total:380 | iSNV |
| F40 | F40-28 | 5705 | NS3 | 0.0632 | A:370;G:25;C:0;T:0;total:395 | iSNV |
| F40 | F40-28 | 6714 | NS4A | 0.0854 | A:20;G:214;C:0;T:0;total:234 | iSNV |
| F40 | F40-28 | 6729 | NS4A | 0.0512 | A:12;G:0;C:222;T:0;total:234 | iSNV |
| F40 | F40-28 | 6730 | NS4A | 0.047 | A:0;G:0;C:11;T:223;total:234 | iSNV |
| F40 | F40-28 | 6867 | NS4A | 0.148 | A:37;G:213;C:0;T:0;total:250 | iSNV |
| F40 | F40-28 | 6900 | NS4A | 0.0489 | A:12;G:233;C:0;T:0;total:245 | iSNV |
| F40 | F40-28 | 6970 | NS4A | 0.0916 | A:218;G:0;C:22;T:0;total:240 | iSNV |
| F40 | F40-28 | 7104 | NS4A | 0.0422 | A:12;G:272;C:0;T:0;total:284 | iSNV |
| F40 | F40-28 | 7451 | NS4B | 0.0967 | A:0;G:168;C:18;T:0;total:186 | iSNV |
| F40 | F40-28 | 7633 | NS4B | 0.8196 | A:0;G:0;C:72;T:327;total:399 | iSNV |
| F40 | F40-28 | 7656 | NS4B | 0.1083 | A:44;G:362;C:0;T:0;total:406 | iSNV |
| F40 | F40-28 | 7973 | NS5 | 0.093 | A:380;G:0;C:0;T:39;total:419 | iSNV |
| F40 | F40-28 | 10259 | NS5 | 0.059 | A:303;G:19;C:0;T:0;total:322 | iSNV |
| F40 | F40-28 | 10335 | NS5 | 0.0234 | A:0;G:0;C:7;T:292;total:299 | iSNV |
| F40 | F40-28 | 10428 | 3'-UTR | 0.2324 | A:0;G:0;C:175;T:53;total:228 | iSNV |
| F40 | F40-28 | 10447 | 3'-UTR | 0.0733 | A:0;G:0;C:202;T:16;total:218 | iSNV |
| F40 | F40-28 | 10451 | 3'-UTR | 0.0358 | A:0;G:0;C:215;T:8;total:223 | iSNV |
| F40 | F40-28 | 10452 | 3'-UTR | 0.0867 | A:200;G:19;C:0;T:0;total:219 | iSNV |
| F40 | F40-29 | 332 | C | 0.0332 | A:728;G:25;C:0;T:0;total:753 | iSNV |
| F40 | F40-29 | 352 | C | 0.0247 | A:18;G:710;C:0;T:0;total:728 | iSNV |
| F40 | F40-29 | 1218 | E | 0.986 | A:0;G:0;C:7;T:490;total:497 | SNP |
| F40 | F40-29 | 1447 | E | 0.8784 | A:325;G:0;C:45;T:0;total:370 | iSNV |
| F40 | F40-29 | 2362 | E | 0.9522 | A:0;G:12;C:239;T:0;total:251 | iSNV |
| F40 | F40-29 | 3869 | NS2A | 0.9939 | A:0;G:0;C:5;T:814;total:819 | SNP |
| F40 | F40-29 | 3959 | NS2A | 0.7975 | A:0;G:0;C:141;T:555;total:696 | iSNV |
| F40 | F40-29 | 4829 | NS3 | 0.1072 | A:616;G:74;C:0;T:0;total:690 | iSNV |
| F40 | F40-29 | 4943 | NS3 | 0.0979 | A:442;G:48;C:0;T:0;total:490 | iSNV |
| F40 | F40-29 | 5702 | NS3 | 0.1098 | A:0;G:0;C:689;T:85;total:774 | iSNV |
| F40 | F40-29 | 6714 | NS4A | 0.0548 | A:27;G:465;C:0;T:0;total:492 | iSNV |
| F40 | F40-29 | 6715 | NS4A | 0.0489 | A:0;G:0;C:466;T:24;total:490 | iSNV |
| F40 | F40-29 | 6867 | NS4A | 0.2534 | A:128;G:377;C:0;T:0;total:505 | iSNV |
| F40 | F40-29 | 6900 | NS4A | 0.0278 | A:14;G:487;C:0;T:1;total:502 | iSNV |
| F40 | F40-29 | 7481 | NS4B | 0.0887 | A:0;G:0;C:33;T:339;total:372 | iSNV |
| F40 | F40-29 | 7633 | NS4B | 0.9828 | A:0;G:0;C:12;T:684;total:696 | SNP |
| F40 | F40-29 | 8844 | NS5 | 0.0717 | A:43;G:556;C:0;T:0;total:599 | iSNV |
| F40 | F40-29 | 8966 | NS5 | 0.0736 | A:0;G:0;C:729;T:58;total:787 | iSNV |
| F40 | F40-29 | 9659 | NS5 | 0.072 | A:0;G:0;C:322;T:25;total:347 | iSNV |
| F40 | F40-29 | 10046 | NS5 | 0.02 | A:489;G:10;C:0;T:0;total:499 | iSNV |
| F40 | F40-29 | 10428 | 3'-UTR | 0.833 | A:0;G:0;C:68;T:339;total:407 | iSNV |
| F40 | F40-29 | 10447 | 3'-UTR | 0.0353 | A:0;G:0;C:382;T:14;total:396 | iSNV |
| F40 | F40-29 | 10451 | 3'-UTR | 0.0205 | A:0;G:0;C:382;T:8;total:390 | iSNV |
| F40 | F40-3 | 828 | M | 0.3612 | A:122;G:69;C:0;T:0;total:191 | iSNV |
| F40 | F40-3 | 996 | E | 0.4193 | A:78;G:108;C:0;T:0;total:186 | iSNV |
| F40 | F40-3 | 1218 | E | 0.9745 | A:0;G:0;C:6;T:229;total:235 | iSNV |
| F40 | F40-3 | 1255 | E | 0.0876 | A:229;G:22;C:0;T:0;total:251 | iSNV |
| F40 | F40-3 | 1263 | E | 0.0431 | A:0;G:0;C:11;T:244;total:255 | iSNV |
| F40 | F40-3 | 1508 | E | 0.1192 | A:0;G:0;C:133;T:18;total:151 | iSNV |
| F40 | F40-3 | 1772 | E | 0.0248 | A:0;G:6;C:0;T:235;total:241 | iSNV |
| F40 | F40-3 | 1797 | E | 0.3083 | A:0;G:74;C:0;T:166;total:240 | iSNV |
| F40 | F40-3 | 1804 | E | 0.0414 | A:10;G:231;C:0;T:0;total:241 | iSNV |
| F40 | F40-3 | 2465 | E | 0.3781 | A:0;G:0;C:74;T:45;total:119 | iSNV |
| F40 | F40-3 | 2780 | NS1 | 0.1116 | A:0;G:0;C:44;T:350;total:394 | iSNV |
| F40 | F40-3 | 3119 | NS1 | 0.0282 | A:11;G:379;C:0;T:0;total:390 | iSNV |
| F40 | F40-3 | 3858 | NS2A | 0.3929 | A:0;G:0;C:224;T:145;total:369 | iSNV |
| F40 | F40-3 | 3869 | NS2A | 1 | A:0;G:0;C:0;T:378;total:378 | SNP |
| F40 | F40-3 | 4037 | NS2A | 0.0292 | A:0;G:0;C:232;T:7;total:239 | iSNV |
| F40 | F40-3 | 4289 | NS2B | 0.0588 | A:14;G:0;C:0;T:224;total:238 | iSNV |
| F40 | F40-3 | 4292 | NS2B | 0.325 | A:78;G:0;C:0;T:162;total:240 | iSNV |
| F40 | F40-3 | 5306 | NS3 | 0.3202 | A:208;G:98;C:0;T:0;total:306 | iSNV |
| F40 | F40-3 | 5736 | NS3 | 0.0267 | A:11;G:0;C:400;T:0;total:411 | iSNV |
| F40 | F40-3 | 5761 | NS3 | 0.0388 | A:396;G:16;C:0;T:0;total:412 | iSNV |
| F40 | F40-3 | 6980 | NS4A | 0.2128 | A:159;G:0;C:0;T:43;total:202 | iSNV |
| F40 | F40-3 | 7585 | NS4B | 0.0957 | A:0;G:236;C:25;T:0;total:261 | iSNV |
| F40 | F40-3 | 7633 | NS4B | 0.6118 | A:0;G:0;C:132;T:208;total:340 | iSNV |
| F40 | F40-3 | 7660 | NS4B | 0.0217 | A:360;G:8;C:0;T:0;total:368 | iSNV |
| F40 | F40-3 | 9233 | NS5 | 0.0298 | A:0;G:0;C:422;T:13;total:435 | iSNV |
| F40 | F40-3 | 9607 | NS5 | 0.0354 | A:136;G:0;C:5;T:0;total:141 | iSNV |
| F40 | F40-3 | 10428 | 3'-UTR | 0.6915 | A:0;G:0;C:58;T:130;total:188 | iSNV |
| F40 | F40-3 | 10447 | 3'-UTR | 0.0287 | A:0;G:0;C:169;T:5;total:174 | iSNV |

| | | | | | | |
|---|---|---|---|---|---|---|
| F40 | F40-30 | 719 | M | 0.1054 | A:0;G:0;C:246;T:29;total:275 | iSNV |
| F40 | F40-30 | 1218 | E | 0.9107 | A:0;G:0;C:21;T:214;total:235 | iSNV |
| F40 | F40-30 | 1413 | E | 0.9438 | A:13;G:218;C:0;T:0;total:231 | iSNV |
| F40 | F40-30 | 1428 | E | 0.8976 | A:17;G:149;C:0;T:0;total:166 | iSNV |
| F40 | F40-30 | 2421 | E | 0.0446 | A:0;G:0;C:5;T:107;total:112 | iSNV |
| F40 | F40-30 | 3869 | NS2A | 0.9502 | A:0;G:0;C:21;T:400;total:421 | iSNV |
| F40 | F40-30 | 3965 | NS2A | 0.044 | A:0;G:0;C:369;T:17;total:386 | iSNV |
| F40 | F40-30 | 4005 | NS2A | 0.8961 | A:0;G:0;C:29;T:250;total:279 | iSNV |
| F40 | F40-30 | 6714 | NS4A | 0.0553 | A:14;G:239;C:0;T:0;total:253 | iSNV |
| F40 | F40-30 | 6867 | NS4A | 0.2863 | A:69;G:172;C:0;T:0;total:241 | iSNV |
| F40 | F40-30 | 7264 | NS4A | 0.1944 | A:0;G:0;C:116;T:28;total:144 | iSNV |
| F40 | F40-30 | 7633 | NS4B | 0.9375 | A:0;G:0;C:21;T:315;total:336 | iSNV |
| F40 | F40-30 | 7944 | NS5 | 0.023 | A:0;G:1;C:10;T:422;total:433 | iSNV |
| F40 | F40-30 | 10376 | NS5 | 0.9508 | A:193;G:10;C:0;T:0;total:203 | iSNV |
| F40 | F40-30 | 10428 | 3'-UTR | 0.7603 | A:0;G:0;C:41;T:130;total:171 | iSNV |
| F40 | F40-4 | 249 | C | 0.0815 | A:462;G:41;C:0;T:0;total:503 | iSNV |
| F40 | F40-4 | 746 | M | 0.0336 | A:0;G:0;C:373;T:13;total:386 | iSNV |
| F40 | F40-4 | 998 | E | 0.16 | A:0;G:0;C:252;T:48;total:300 | iSNV |
| F40 | F40-4 | 1218 | E | 1 | A:0;G:0;C:0;T:303;total:303 | SNP |
| F40 | F40-4 | 1263 | E | 0.1647 | A:0;G:0;C:57;T:289;total:346 | iSNV |
| F40 | F40-4 | 1388 | E | 0.0749 | A:0;G:0;C:358;T:29;total:387 | iSNV |
| F40 | F40-4 | 1433 | E | 0.026 | A:0;G:9;C:0;T:336;total:345 | iSNV |
| F40 | F40-4 | 1453 | E | 0.0353 | A:12;G:0;C:0;T:327;total:339 | iSNV |
| F40 | F40-4 | 1496 | E | 0.0539 | A:0;G:0;C:13;T:228;total:241 | iSNV |
| F40 | F40-4 | 1512 | E | 0.0987 | A:219;G:24;C:0;T:0;total:243 | iSNV |
| F40 | F40-4 | 1513 | E | 0.0569 | A:232;G:0;C:14;T:0;total:246 | iSNV |
| F40 | F40-4 | 1789 | E | 0.0545 | A:18;G:0;C:0;T:312;total:330 | iSNV |
| F40 | F40-4 | 2367 | E | 0.7119 | A:51;G:126;C:0;T:0;total:177 | iSNV |
| F40 | F40-4 | 2834 | NS1 | 0.0659 | A:411;G:29;C:0;T:0;total:440 | iSNV |
| F40 | F40-4 | 3224 | NS1 | 0.0801 | A:528;G:46;C:0;T:0;total:574 | iSNV |
| F40 | F40-4 | 3336 | NS1 | 0.1056 | A:347;G:41;C:0;T:0;total:388 | iSNV |
| F40 | F40-4 | 3869 | NS2A | 0.833 | A:0;G:0;C:81;T:404;total:485 | iSNV |
| F40 | F40-4 | 4280 | NS2B | 0.0808 | A:0;G:0;C:250;T:22;total:272 | iSNV |
| F40 | F40-4 | 4319 | NS2B | 0.0258 | A:8;G:302;C:0;T:0;total:310 | iSNV |
| F40 | F40-4 | 4697 | NS3 | 0.2004 | A:0;G:0;C:84;T:335;total:419 | iSNV |
| F40 | F40-4 | 5736 | NS3 | 0.0269 | A:14;G:0;C:505;T:0;total:519 | iSNV |
| F40 | F40-4 | 5952 | NS3 | 0.1479 | A:0;G:0;C:409;T:71;total:480 | iSNV |
| F40 | F40-4 | 6322 | NS3 | 0.0282 | A:0;G:0;C:241;T:7;total:248 | iSNV |
| F40 | F40-4 | 6471 | NS4A | 0.0743 | A:22;G:274;C:0;T:0;total:296 | iSNV |
| F40 | F40-4 | 6714 | NS4A | 0.024 | A:8;G:324;C:0;T:0;total:332 | iSNV |
| F40 | F40-4 | 6900 | NS4A | 0.0821 | A:24;G:268;C:0;T:0;total:292 | iSNV |
| F40 | F40-4 | 6969 | NS4A | 0.2547 | A:236;G:1;C:81;T:0;total:318 | iSNV |
| F40 | F40-4 | 7139 | NS4A | 0.0286 | A:0;G:0;C:9;T:305;total:314 | iSNV |
| F40 | F40-4 | 7633 | NS4B | 0.7882 | A:0;G:0;C:86;T:320;total:406 | iSNV |
| F40 | F40-4 | 8639 | NS5 | 0.298 | A:0;G:0;C:76;T:179;total:255 | iSNV |
| F40 | F40-4 | 9359 | NS5 | 0.1587 | A:0;G:0;C:87;T:461;total:548 | iSNV |
| F40 | F40-4 | 10259 | NS5 | 0.2016 | A:289;G:73;C:0;T:0;total:362 | iSNV |
| F40 | F40-4 | 10428 | 3'-UTR | 0.288 | A:0;G:0;C:173;T:70;total:243 | iSNV |
| F40 | F40-4 | 10566 | 3'-UTR | 0.2203 | A:0;G:0;C:184;T:52;total:236 | iSNV |
| F40 | F40-4 | 10705 | 3'-UTR | 0.0225 | A:0;G:0;C:260;T:6;total:266 | iSNV |
| F40 | F40-4 | 10718 | 3'-UTR | 0.083 | A:0;G:0;C:254;T:23;total:277 | iSNV |
| F40 | F40-5 | 294 | C | 0.0228 | A:13;G:555;C:0;T:0;total:568 | iSNV |
| F40 | F40-5 | 386 | C | 0.0568 | A:38;G:631;C:0;T:0;total:669 | iSNV |
| F40 | F40-5 | 399 | C | 0.0758 | A:51;G:0;C:621;T:0;total:672 | iSNV |
| F40 | F40-5 | 897 | M | 0.0388 | A:0;G:0;C:297;T:12;total:309 | iSNV |
| F40 | F40-5 | 1218 | E | 1 | A:0;G:0;C:0;T:435;total:435 | SNP |
| F40 | F40-5 | 2076 | E | 0.2546 | A:240;G:82;C:0;T:0;total:322 | iSNV |
| F40 | F40-5 | 2126 | E | 0.0487 | A:0;G:0;C:312;T:16;total:328 | iSNV |
| F40 | F40-5 | 2202 | E | 0.0247 | A:354;G:0;C:0;T:9;total:363 | iSNV |
| F40 | F40-5 | 2362 | E | 0.0669 | A:0;G:195;C:0;T:14;total:209 | iSNV |
| F40 | F40-5 | 3101 | NS1 | 0.0239 | A:0;G:0;C:611;T:15;total:626 | iSNV |
| F40 | F40-5 | 3869 | NS2A | 0.9774 | A:0;G:0;C:13;T:561;total:574 | iSNV |
| F40 | F40-5 | 5187 | NS3 | 0.0298 | A:0;G:0;C:11;T:358;total:369 | iSNV |
| F40 | F40-5 | 6206 | NS3 | 0.1227 | A:0;G:0;C:336;T:47;total:383 | iSNV |
| F40 | F40-5 | 6335 | NS3 | 0.0357 | A:0;G:0;C:297;T:11;total:308 | iSNV |
| F40 | F40-5 | 6336 | NS3 | 0.1909 | A:233;G:0;C:0;T:55;total:288 | iSNV |
| F40 | F40-5 | 6389 | NS3 | 0.0298 | A:13;G:423;C:0;T:0;total:436 | iSNV |
| F40 | F40-5 | 6714 | NS4A | 0.0989 | A:29;G:264;C:0;T:0;total:293 | iSNV |
| F40 | F40-5 | 6753 | NS4A | 0.1017 | A:0;G:256;C:0;T:29;total:285 | iSNV |
| F40 | F40-5 | 6867 | NS4A | 0.1161 | A:41;G:312;C:0;T:0;total:353 | iSNV |
| F40 | F40-5 | 7633 | NS4B | 0.9249 | A:0;G:0;C:35;T:431;total:466 | iSNV |
| F40 | F40-5 | 7670 | NS4B | 0.0209 | A:11;G:514;C:0;T:0;total:525 | iSNV |
| F40 | F40-5 | 7748 | NS5 | 0.0216 | A:0;G:0;C:11;T:498;total:509 | iSNV |
| F40 | F40-5 | 7774 | NS5 | 0.0251 | A:0;G:0;C:11;T:426;total:437 | iSNV |
| F40 | F40-5 | 8036 | NS5 | 0.0224 | A:0;G:0;C:567;T:13;total:580 | iSNV |
| F40 | F40-5 | 8062 | NS5 | 0.0654 | A:39;G:557;C:0;T:0;total:596 | iSNV |
| F40 | F40-5 | 8126 | NS5 | 0.026 | A:0;G:0;C:635;T:17;total:652 | iSNV |
| F40 | F40-5 | 9341 | NS5 | 0.224 | A:523;G:151;C:0;T:0;total:674 | iSNV |
| F40 | F40-5 | 10419 | 3'-UTR | 0.0243 | A:0;G:0;C:280;T:7;total:287 | iSNV |
| F40 | F40-5 | 10428 | 3'-UTR | 0.5606 | A:0;G:0;C:127;T:162;total:289 | iSNV |
| F40 | F40-5 | 10447 | 3'-UTR | 0.026 | A:0;G:0;C:262;T:7;total:269 | iSNV |
| F40 | F40-6 | 353 | C | 0.8054 | A:29;G:120;C:0;T:0;total:149 | iSNV |

| | | | | | | |
|---|---|---|---|---|---|---|
| F40 | F40-6 | 645 | M | 0.8905 | A:8;G:65;C:0;T:0;total:73 | iSNV |
| F40 | F40-6 | 998 | E | 0.7932 | A:0;G:0;C:18;T:69;total:87 | iSNV |
| F40 | F40-6 | 1117 | E | 0.9 | A:10;G:90;C:0;T:0;total:100 | iSNV |
| F40 | F40-6 | 1218 | E | 0.093 | A:0;G:0;C:78;T:8;total:86 | iSNV |
| F40 | F40-6 | 3869 | NS2A | 0.1266 | A:0;G:0;C:131;T:19;total:150 | iSNV |
| F40 | F40-6 | 6005 | NS3 | 0.1681 | A:0;G:0;C:94;T:19;total:113 | iSNV |
| F40 | F40-6 | 6061 | NS3 | 0.7979 | A:19;G:75;C:0;T:0;total:94 | iSNV |
| F40 | F40-6 | 6867 | NS4A | 0.1194 | A:8;G:59;C:0;T:0;total:67 | iSNV |
| F40 | F40-6 | 7060 | NS4A | 0.8706 | A:0;G:0;C:74;T:11;total:85 | iSNV |
| F40 | F40-6 | 8318 | NS5 | 0.1296 | A:0;G:0;C:94;T:14;total:108 | iSNV |
| F40 | F40-6 | 10165 | NS5 | 0.1694 | A:0;G:0;C:10;T:49;total:59 | iSNV |
| F40 | F40-6 | 10259 | NS5 | 0.7579 | A:23;G:72;C:0;T:0;total:95 | iSNV |
| F40 | F40-6 | 10419 | 3'-UTR | 0.8553 | A:0;G:0;C:11;T:65;total:76 | iSNV |
| F40 | F40-6 | 10428 | 3'-UTR | 0.3783 | A:0;G:0;C:46;T:28;total:74 | iSNV |
| F40 | F40-6 | 10447 | 3'-UTR | 0.9081 | A:0;G:0;C:8;T:79;total:87 | iSNV |
| F40 | F40-7 | 1218 | E | 1 | A:0;G:0;C:0;T:461;total:461 | SNP |
| F40 | F40-7 | 1416 | E | 0.8673 | A:47;G:307;C:0;T:0;total:354 | iSNV |
| F40 | F40-7 | 1430 | E | 0.0432 | A:0;G:0;C:310;T:14;total:324 | iSNV |
| F40 | F40-7 | 2067 | E | 0.8978 | A:0;G:0;C:281;T:32;total:313 | iSNV |
| F40 | F40-7 | 2275 | E | 0.043 | A:0;G:0;C:13;T:289;total:302 | iSNV |
| F40 | F40-7 | 3257 | NS1 | 0.036 | A:0;G:0;C:22;T:589;total:611 | iSNV |
| F40 | F40-7 | 3317 | NS1 | 0.903 | A:0;G:0;C:53;T:493;total:546 | iSNV |
| F40 | F40-7 | 3869 | NS2A | 0.9982 | A:0;G:0;C:1;T:553;total:554 | SNP |
| F40 | F40-7 | 6233 | NS3 | 0.8373 | A:0;G:1;C:55;T:282;total:338 | iSNV |
| F40 | F40-7 | 6322 | NS3 | 0.9132 | A:0;G:0;C:27;T:284;total:311 | iSNV |
| F40 | F40-7 | 6714 | NS4A | 0.2616 | A:101;G:285;C:0;T:0;total:386 | iSNV |
| F40 | F40-7 | 6867 | NS4A | 0.0723 | A:27;G:346;C:0;T:0;total:373 | iSNV |
| F40 | F40-7 | 7633 | NS4B | 0.9944 | A:0;G:0;C:3;T:527;total:530 | SNP |
| F40 | F40-7 | 9370 | NS5 | 0.0275 | A:18;G:0;C:0;T:635;total:653 | iSNV |
| F40 | F40-7 | 9592 | NS5 | 0.0266 | A:438;G:12;C:0;T:0;total:450 | iSNV |
| F40 | F40-7 | 9690 | NS5 | 0.9282 | A:310;G:24;C:0;T:0;total:334 | iSNV |
| F40 | F40-7 | 10428 | 3'-UTR | 0.9711 | A:0;G:0;C:7;T:235;total:242 | iSNV |
| F40 | F40-7 | 10451 | 3'-UTR | 0.0389 | A:0;G:1;C:221;T:9;total:231 | iSNV |
| F40 | F40-7 | 10547 | 3'-UTR | 0.0238 | A:287;G:7;C:0;T:0;total:294 | iSNV |
| F40 | F40-7 | 10568 | 3'-UTR | 0.0243 | A:0;G:0;C:280;T:7;total:287 | iSNV |
| F40 | F40-7 | 10647 | 3'-UTR | 0.1798 | A:0;G:0;C:66;T:301;total:367 | iSNV |
| F40 | F40-8 | 869 | M | 0.0316 | A:8;G:0;C:0;T:245;total:253 | iSNV |
| F40 | F40-8 | 1218 | E | 1 | A:0;G:0;C:0;T:282;total:282 | SNP |
| F40 | F40-8 | 1428 | E | 0.1625 | A:237;G:46;C:0;T:0;total:283 | iSNV |
| F40 | F40-8 | 1430 | E | 0.0451 | A:2;G:0;C:273;T:13;total:288 | iSNV |
| F40 | F40-8 | 1453 | E | 0.7225 | A:1;G:0;C:202;T:78;total:281 | iSNV |
| F40 | F40-8 | 1512 | E | 0.6167 | A:69;G:111;C:0;T:0;total:180 | iSNV |
| F40 | F40-8 | 2054 | E | 0.0452 | A:0;G:0;C:190;T:9;total:199 | iSNV |
| F40 | F40-8 | 3869 | NS2A | 0.9895 | A:0;G:0;C:4;T:374;total:378 | SNP |
| F40 | F40-8 | 4187 | NS2A | 0.1428 | A:102;G:17;C:0;T:0;total:119 | iSNV |
| F40 | F40-8 | 5353 | NS3 | 0.1292 | A:256;G:38;C:0;T:0;total:294 | iSNV |
| F40 | F40-8 | 5456 | NS3 | 0.0971 | A:0;G:0;C:353;T:38;total:391 | iSNV |
| F40 | F40-8 | 5665 | NS3 | 0.6614 | A:0;G:0;C:127;T:248;total:375 | iSNV |
| F40 | F40-8 | 5864 | NS3 | 0.0742 | A:28;G:0;C:0;T:349;total:377 | iSNV |
| F40 | F40-8 | 6314 | NS3 | 0.7428 | A:0;G:0;C:179;T:62;total:241 | iSNV |
| F40 | F40-8 | 6867 | NS4A | 0.672 | A:168;G:82;C:0;T:0;total:250 | iSNV |
| F40 | F40-8 | 6969 | NS4A | 0.0201 | A:243;G:5;C:0;T:0;total:248 | iSNV |
| F40 | F40-8 | 7334 | NS4B | 0.0625 | A:150;G:10;C:0;T:0;total:160 | iSNV |
| F40 | F40-8 | 7367 | NS4B | 0.0574 | A:0;G:0;C:164;T:10;total:174 | iSNV |
| F40 | F40-8 | 7633 | NS4B | 0.951 | A:0;G:0;C:15;T:291;total:306 | iSNV |
| F40 | F40-8 | 8357 | NS5 | 0.0521 | A:0;G:0;C:382;T:21;total:403 | iSNV |
| F40 | F40-8 | 8567 | NS5 | 0.0648 | A:0;G:0;C:274;T:19;total:293 | iSNV |
| F40 | F40-8 | 9818 | NS5 | 0.0797 | A:0;G:0;C:15;T:173;total:188 | iSNV |
| F40 | F40-8 | 10428 | 3'-UTR | 0.2428 | A:0;G:0;C:159;T:51;total:210 | iSNV |
| F40 | F40-8 | 10447 | 3'-UTR | 0.6568 | A:0;G:0;C:69;T:132;total:201 | iSNV |
| F40 | F40-9 | 925 | M | 0.0346 | A:0;G:167;C:6;T:0;total:173 | iSNV |
| F40 | F40-9 | 1057 | E | 0.0437 | A:8;G:175;C:0;T:0;total:183 | iSNV |
| F40 | F40-9 | 1218 | E | 1 | A:0;G:0;C:0;T:277;total:277 | SNP |
| F40 | F40-9 | 1413 | E | 0.0798 | A:265;G:23;C:0;T:0;total:288 | iSNV |
| F40 | F40-9 | 1512 | E | 0.4367 | A:89;G:69;C:0;T:0;total:158 | iSNV |
| F40 | F40-9 | 1797 | E | 0.1195 | A:0;G:33;C:0;T:243;total:276 | iSNV |
| F40 | F40-9 | 2852 | NS1 | 0.3598 | A:0;G:0;C:258;T:145;total:403 | iSNV |
| F40 | F40-9 | 3248 | NS1 | 0.0962 | A:49;G:460;C:0;T:0;total:509 | iSNV |
| F40 | F40-9 | 3419 | NS1 | 0.1468 | A:0;G:0;C:244;T:42;total:286 | iSNV |
| F40 | F40-9 | 3869 | NS2A | 1 | A:0;G:0;C:0;T:430;total:430 | SNP |
| F40 | F40-9 | 3906 | NS2A | 0.0211 | A:10;G:463;C:0;T:0;total:473 | iSNV |
| F40 | F40-9 | 4974 | NS3 | 0.4531 | A:121;G:146;C:0;T:0;total:267 | iSNV |
| F40 | F40-9 | 5136 | NS3 | 0.127 | A:0;G:261;C:38;T:0;total:299 | iSNV |
| F40 | F40-9 | 5665 | NS3 | 0.0416 | A:0;G:0;C:414;T:18;total:432 | iSNV |
| F40 | F40-9 | 5921 | NS3 | 0.4226 | A:224;G:164;C:0;T:0;total:388 | iSNV |
| F40 | F40-9 | 6004 | NS3 | 0.0305 | A:0;G:0;C:286;T:9;total:295 | iSNV |
| F40 | F40-9 | 6714 | NS4A | 0.209 | A:46;G:174;C:0;T:0;total:220 | iSNV |
| F40 | F40-9 | 6729 | NS4A | 0.1244 | A:0;G:28;C:197;T:0;total:225 | iSNV |
| F40 | F40-9 | 6938 | NS4A | 0.1359 | A:0;G:0;C:178;T:28;total:206 | iSNV |
| F40 | F40-9 | 7061 | NS4A | 0.0391 | A:0;G:0;C:270;T:11;total:281 | iSNV |
| F40 | F40-9 | 7561 | NS4B | 0.0418 | A:0;G:0;C:12;T:275;total:287 | iSNV |
| F40 | F40-9 | 7609 | NS4B | 0.0909 | A:27;G:270;C:0;T:0;total:297 | iSNV |

| | | | | | | |
|---|---|---|---|---|---|---|
| F40 | F40-9 | 7633 | NS4B | 0.2987 | A:0;G:0;C:223;T:95;total:318 | iSNV |
| F40 | F40-9 | 7657 | NS4B | 0.1702 | A:268;G:55;C:0;T:0;total:323 | iSNV |
| F40 | F40-9 | 8000 | NS5 | 0.0944 | A:36;G:345;C:0;T:0;total:381 | iSNV |
| F40 | F40-9 | 8087 | NS5 | 0.1262 | A:346;G:50;C:0;T:0;total:396 | iSNV |
| F40 | F40-9 | 8103 | NS5 | 0.0327 | A:0;G:0;C:384;T:13;total:397 | iSNV |
| F40 | F40-9 | 8397 | NS5 | 0.0263 | A:11;G:0;C:406;T:0;total:417 | iSNV |
| F40 | F40-9 | 8777 | NS5 | 0.0372 | A:12;G:310;C:0;T:0;total:322 | iSNV |
| F40 | F40-9 | 9633 | NS5 | 0.1183 | A:20;G:0;C:0;T:149;total:169 | iSNV |
| F40 | F40-9 | 10376 | NS5 | 0.0905 | A:23;G:231;C:0;T:0;total:254 | iSNV |
| F40 | F40-9 | 10428 | 3'-UTR | 0.2294 | A:0;G:0;C:178;T:53;total:231 | iSNV |
| F45 | F45-1 | 221 | C | 0.4794 | A:11929;G:12944;C:3;T:5;total:24881 | iSNV |
| F45 | F45-1 | 482 | M | 0.0412 | A:1134;G:26347;C:1;T:3;total:27485 | iSNV |
| F45 | F45-1 | 897 | M | 0.1615 | A:3;G:3;C:13278;T:2559;total:15843 | iSNV |
| F45 | F45-1 | 926 | M | 0.0208 | A:1;G:2;C:352;T:16506;total:16861 | iSNV |
| F45 | F45-1 | 1064 | E | 0.0436 | A:1;G:2;C:15607;T:713;total:16323 | iSNV |
| F45 | F45-1 | 1218 | E | 0.997 | A:2;G:4;C:55;T:19712;total:19773 | SNP |
| F45 | F45-1 | 1428 | E | 0.882 | A:2393;G:17869;C:1;T:0;total:20263 | iSNV |
| F45 | F45-1 | 1565 | E | 0.0202 | A:308;G:14898;C:9;T:0;total:15215 | iSNV |
| F45 | F45-1 | 1889 | E | 0.0374 | A:0;G:0;C:635;T:16333;total:16968 | iSNV |
| F45 | F45-1 | 2157 | E | 0.0275 | A:3;G:1;C:20102;T:570;total:20676 | iSNV |
| F45 | F45-1 | 2376 | E | 0.0223 | A:0;G:1;C:12501;T:286;total:12788 | iSNV |
| F45 | F45-1 | 3434 | NS1 | 0.0555 | A:956;G:16256;C:6;T:0;total:17218 | iSNV |
| F45 | F45-1 | 3579 | NS1 | 0.0359 | A:15891;G:592;C:0;T:0;total:16483 | iSNV |
| F45 | F45-1 | 3822 | NS2A | 0.1443 | A:17867;G:3015;C:1;T:6;total:20889 | iSNV |
| F45 | F45-1 | 3869 | NS2A | 0.9949 | A:5;G:1;C:114;T:23402;total:23522 | SNP |
| F45 | F45-1 | 4268 | NS2B | 0.1205 | A:14532;G:1994;C:5;T:3;total:16534 | iSNV |
| F45 | F45-1 | 4447 | NS2B | 0.0413 | A:15361;G:662;C:0;T:1;total:16024 | iSNV |
| F45 | F45-1 | 4559 | NS2B | 0.6941 | A:1;G:7;C:6757;T:15320;total:22085 | iSNV |
| F45 | F45-1 | 4974 | NS3 | 0.0361 | A:839;G:22367;C:0;T:1;total:23207 | iSNV |
| F45 | F45-1 | 5276 | NS3 | 0.1602 | A:17345;G:3311;C:3;T:3;total:20662 | iSNV |
| F45 | F45-1 | 5617 | NS3 | 0.0257 | A:22551;G:597;C:2;T:4;total:23154 | iSNV |
| F45 | F45-1 | 6572 | NS4A | 0.0339 | A:533;G:5;C:8;T:15150;total:15696 | iSNV |
| F45 | F45-1 | 6714 | NS4A | 0.5026 | A:8615;G:8532;C:2;T:2;total:17151 | iSNV |
| F45 | F45-1 | 6716 | NS4A | 0.0254 | A:1;G:447;C:3;T:17132;total:17583 | iSNV |
| F45 | F45-1 | 6878 | NS4A | 0.0447 | A:1;G:1;C:17978;T:843;total:18823 | iSNV |
| F45 | F45-1 | 6900 | NS4A | 0.0617 | A:1164;G:17686;C:3;T:1;total:18854 | iSNV |
| F45 | F45-1 | 6934 | NS4A | 0.0225 | A:19306;G:445;C:0;T:0;total:19751 | iSNV |
| F45 | F45-1 | 6970 | NS4A | 0.0535 | A:17892;G:3;C:1013;T:1;total:18909 | iSNV |
| F45 | F45-1 | 7127 | NS4A | 0.034 | A:0;G:3;C:639;T:18108;total:18750 | iSNV |
| F45 | F45-1 | 7445 | NS4B | 0.0308 | A:0;G:0;C:11741;T:374;total:12115 | iSNV |
| F45 | F45-1 | 7536 | NS4B | 0.0602 | A:4;G:1;C:1052;T:16417;total:17474 | iSNV |
| F45 | F45-1 | 7626 | NS4B | 0.0314 | A:756;G:3;C:26;T:23221;total:24006 | iSNV |
| F45 | F45-1 | 7633 | NS4B | 0.9932 | A:0;G:12;C:154;T:24086;total:24252 | SNP |
| F45 | F45-1 | 8129 | NS5 | 0.0388 | A:3;G:3;C:26964;T:1089;total:28059 | iSNV |
| F45 | F45-1 | 8217 | NS5 | 0.0426 | A:25307;G:1128;C:2;T:0;total:26437 | iSNV |
| F45 | F45-1 | 8312 | NS5 | 0.0243 | A:1;G:3;C:25355;T:632;total:25991 | iSNV |
| F45 | F45-1 | 8492 | NS5 | 0.0236 | A:1;G:2;C:27003;T:654;total:27660 | iSNV |
| F45 | F45-1 | 8689 | NS5 | 0.0254 | A:0;G:5;C:15445;T:403;total:15853 | iSNV |
| F45 | F45-1 | 8834 | NS5 | 0.0238 | A:1;G:2;C:18648;T:455;total:19106 | iSNV |
| F45 | F45-1 | 9983 | NS5 | 0.0407 | A:3;G:0;C:16485;T:701;total:17189 | iSNV |
| F45 | F45-1 | 10040 | NS5 | 0.023 | A:460;G:19472;C:1;T:0;total:19933 | iSNV |
| F45 | F45-1 | 10112 | NS5 | 0.0208 | A:20510;G:437;C:3;T:2;total:20952 | iSNV |
| F45 | F45-1 | 10349 | NS5 | 0.0387 | A:1;G:2;C:21408;T:864;total:22275 | iSNV |
| F45 | F45-1 | 10419 | 3'-UTR | 0.0333 | A:4;G:4;C:17418;T:601;total:18027 | iSNV |
| F45 | F45-1 | 10428 | 3'-UTR | 0.1522 | A:2;G:1;C:15158;T:2723;total:17884 | iSNV |
| F45 | F45-1 | 10447 | 3'-UTR | 0.0329 | A:1;G:0;C:15452;T:527;total:15980 | iSNV |
| F45 | F45-1 | 10451 | 3'-UTR | 0.4772 | A:1;G:22;C:8358;T:7652;total:16033 | iSNV |
| F45 | F45-1 | 10668 | 3'-UTR | 0.0588 | A:0;G:1049;C:16777;T:6;total:17832 | iSNV |
| F45 | F45-10 | 296 | C | 0.042 | A:785;G:0;C:17869;T:12;total:18666 | iSNV |
| F45 | F45-10 | 1050 | E | 0.045 | A:1;G:0;C:12834;T:606;total:13441 | iSNV |
| F45 | F45-10 | 1145 | E | 0.0217 | A:0;G:1;C:14515;T:323;total:14839 | iSNV |
| F45 | F45-10 | 1218 | E | 0.999 | A:2;G:0;C:14;T:15028;total:15044 | SNP |
| F45 | F45-10 | 1447 | E | 0.0261 | A:464;G:1;C:17285;T:4;total:17754 | iSNV |
| F45 | F45-10 | 1551 | E | 0.6739 | A:2;G:8761;C:4241;T:1;total:13005 | iSNV |
| F45 | F45-10 | 1797 | E | 0.1085 | A:1;G:1935;C:5;T:15892;total:17833 | iSNV |
| F45 | F45-10 | 1964 | E | 0.0253 | A:1;G:0;C:326;T:12557;total:12884 | iSNV |
| F45 | F45-10 | 2157 | E | 0.0241 | A:0;G:1;C:15335;T:380;total:15716 | iSNV |
| F45 | F45-10 | 2362 | E | 0.0943 | A:1;G:8672;C:345;T:939;total:9957 | iSNV |
| F45 | F45-10 | 3869 | NS2A | 0.9984 | A:3;G:7;C:17;T:16720;total:16747 | SNP |
| F45 | F45-10 | 4122 | NS2A | 0.1161 | A:1105;G:8407;C:0;T:0;total:9512 | iSNV |
| F45 | F45-10 | 4128 | NS2A | 0.0215 | A:0;G:0;C:202;T:9174;total:9376 | iSNV |
| F45 | F45-10 | 4130 | NS2A | 0.0257 | A:244;G:9224;C:0;T:4;total:9472 | iSNV |
| F45 | F45-10 | 4535 | NS2B | 0.0229 | A:15123;G:355;C:1;T:2;total:15481 | iSNV |
| F45 | F45-10 | 5215 | NS3 | 0.283 | A:10874;G:4293;C:0;T:2;total:15169 | iSNV |
| F45 | F45-10 | 5256 | NS3 | 0.9748 | A:0;G:6;C:374;T:14450;total:14830 | iSNV |
| F45 | F45-10 | 5444 | NS3 | 0.0551 | A:4;G:4;C:18160;T:1061;total:19229 | iSNV |
| F45 | F45-10 | 5736 | NS3 | 0.9982 | A:19441;G:0;C:32;T:4;total:19477 | SNP |
| F45 | F45-10 | 5810 | NS3 | 0.0299 | A:19435;G:600;C:2;T:1;total:20038 | iSNV |
| F45 | F45-10 | 5944 | NS3 | 0.1118 | A:18142;G:2284;C:2;T:1;total:20429 | iSNV |
| F45 | F45-10 | 6900 | NS4A | 0.9981 | A:14038;G:26;C:1;T:1;total:14066 | SNP |
| F45 | F45-10 | 7264 | NS4A | 0.9979 | A:2;G:2;C:16;T:9332;total:9352 | SNP |
| F45 | F45-10 | 7633 | NS4B | 0.7728 | A:0;G:1;C:3952;T:13437;total:17390 | iSNV |

| | | | | | | |
|---|---|---|---|---|---|---|
| F45 | F45-10 | 7656 | NS4B | 0.0458 | A:814;G:16952;C:0;T:2;total:17768 | iSNV |
| F45 | F45-10 | 7657 | NS4B | 0.025 | A:17295;G:445;C:1;T:0;total:17741 | iSNV |
| F45 | F45-10 | 8266 | NS5 | 0.0349 | A:1;G:5;C:628;T:17321;total:17955 | iSNV |
| F45 | F45-10 | 8785 | NS5 | 0.0632 | A:884;G:0;C:13088;T:7;total:13979 | iSNV |
| F45 | F45-10 | 9001 | NS5 | 0.1137 | A:13932;G:1789;C:3;T:0;total:15724 | iSNV |
| F45 | F45-10 | 10373 | NS5 | 0.0236 | A:2;G:1;C:14797;T:358;total:15158 | iSNV |
| F45 | F45-10 | 10419 | 3'-UTR | 0.0353 | A:1;G:2;C:12245;T:449;total:12697 | iSNV |
| F45 | F45-10 | 10428 | 3'-UTR | 0.9981 | A:0;G:5;C:19;T:12062;total:12086 | SNP |
| F45 | F45-10 | 10785 | 3'-UTR | 0.0249 | A:298;G:0;C:11626;T:2;total:11926 | iSNV |
| F45 | F45-11 | 655 | M | 0.0239 | A:29418;G:8;C:721;T:0;total:30147 | iSNV |
| F45 | F45-11 | 719 | M | 0.0361 | A:1;G:4;C:32969;T:1238;total:34212 | iSNV |
| F45 | F45-11 | 909 | M | 0.0493 | A:1;G:2;C:26347;T:1367;total:27717 | iSNV |
| F45 | F45-11 | 993 | E | 0.0252 | A:26022;G:673;C:0;T:0;total:26695 | iSNV |
| F45 | F45-11 | 1218 | E | 0.9988 | A:0;G:1;C:36;T:28622;total:28659 | SNP |
| F45 | F45-11 | 1431 | E | 0.1153 | A:4;G:7;C:30186;T:3938;total:34135 | iSNV |
| F45 | F45-11 | 1512 | E | 0.0484 | A:28175;G:1436;C:1;T:1;total:29613 | iSNV |
| F45 | F45-11 | 1514 | E | 0.1015 | A:26579;G:11;C:3004;T:1;total:29595 | iSNV |
| F45 | F45-11 | 1535 | E | 0.0366 | A:0;G:4;C:26434;T:1006;total:27444 | iSNV |
| F45 | F45-11 | 1797 | E | 0.8012 | A:21991;G:5887;C:3;T:6922;total:34803 | iSNV |
| F45 | F45-11 | 2435 | E | 0.0326 | A:3;G:0;C:20824;T:703;total:21530 | iSNV |
| F45 | F45-11 | 2450 | E | 0.0289 | A:2;G:0;C:21934;T:653;total:22589 | iSNV |
| F45 | F45-11 | 2567 | NS1 | 0.0349 | A:3;G:8;C:833;T:22960;total:23804 | iSNV |
| F45 | F45-11 | 2805 | NS1 | 0.0423 | A:37529;G:1660;C:6;T:1;total:39196 | iSNV |
| F45 | F45-11 | 3257 | NS1 | 0.0428 | A:0;G:0;C:1538;T:34371;total:35909 | iSNV |
| F45 | F45-11 | 3495 | NS1 | 0.0734 | A:2;G:1;C:19971;T:1584;total:21558 | iSNV |
| F45 | F45-11 | 3869 | NS2A | 0.9988 | A:1;G:5;C:33;T:30403;total:30442 | SNP |
| F45 | F45-11 | 3906 | NS2A | 0.0265 | A:911;G:33382;C:1;T:4;total:34298 | iSNV |
| F45 | F45-11 | 4091 | NS2A | 0.03 | A:786;G:25349;C:0;T:0;total:26135 | iSNV |
| F45 | F45-11 | 4289 | NS2B | 0.028 | A:2;G:733;C:2;T:25408;total:26145 | iSNV |
| F45 | F45-11 | 4447 | NS2B | 0.0295 | A:23658;G:720;C:2;T:0;total:24380 | iSNV |
| F45 | F45-11 | 5207 | NS3 | 0.0719 | A:24545;G:1904;C:2;T:0;total:26451 | iSNV |
| F45 | F45-11 | 5674 | NS3 | 0.0228 | A:1;G:4;C:35624;T:835;total:36464 | iSNV |
| F45 | F45-11 | 5940 | NS3 | 0.0206 | A:773;G:36633;C:2;T:0;total:37408 | iSNV |
| F45 | F45-11 | 6509 | NS4A | 0.0697 | A:0;G:0;C:2232;T:29747;total:31979 | iSNV |
| F45 | F45-11 | 6714 | NS4A | 0.0271 | A:746;G:26717;C:2;T:0;total:27465 | iSNV |
| F45 | F45-11 | 6867 | NS4A | 0.1464 | A:3997;G:23287;C:2;T:2;total:27288 | iSNV |
| F45 | F45-11 | 6900 | NS4A | 0.0372 | A:1018;G:26291;C:4;T:2;total:27315 | iSNV |
| F45 | F45-11 | 7412 | NS4B | 0.0283 | A:1;G:5;C:626;T:21473;total:22105 | iSNV |
| F45 | F45-11 | 7633 | NS4B | 0.904 | A:0;G:5;C:3042;T:28618;total:31665 | iSNV |
| F45 | F45-11 | 8948 | NS5 | 0.1117 | A:3478;G:27639;C:2;T:2;total:31121 | iSNV |
| F45 | F45-11 | 9370 | NS5 | 0.0449 | A:1713;G:6;C:316;T:36090;total:38125 | iSNV |
| F45 | F45-11 | 9880 | NS5 | 0.7217 | A:19341;G:7461;C:1;T:5;total:26808 | iSNV |
| F45 | F45-11 | 9899 | NS5 | 0.0336 | A:1;G:1;C:24458;T:852;total:25312 | iSNV |
| F45 | F45-11 | 10265 | NS5 | 0.0208 | A:3;G:3;C:31457;T:669;total:32132 | iSNV |
| F45 | F45-11 | 10428 | 3'-UTR | 0.2279 | A:1;G:5;C:18713;T:5528;total:24247 | iSNV |
| F45 | F45-11 | 10568 | 3'-UTR | 0.0435 | A:2;G:2;C:21565;T:983;total:22552 | iSNV |
| F45 | F45-11 | 10589 | 3'-UTR | 0.7197 | A:4;G:16930;C:7;T:6600;total:23541 | iSNV |
| F45 | F45-11 | 10860 | 3'-UTR | 0.0318 | A:392;G:0;C:11919;T:5;total:12316 | iSNV |
| F45 | F45-12 | 353 | C | 0.5812 | A:17355;G:24060;C:1;T:23;total:41439 | iSNV |
| F45 | F45-12 | 645 | M | 0.5686 | A:15546;G:20463;C:15;T:5;total:36029 | iSNV |
| F45 | F45-12 | 869 | M | 0.1401 | A:7;G:5443;C:5;T:33395;total:38850 | iSNV |
| F45 | F45-12 | 998 | E | 0.6052 | A:7;G:6;C:12846;T:19673;total:32532 | iSNV |
| F45 | F45-12 | 1083 | E | 0.0329 | A:29055;G:77;C:993;T:2;total:30127 | iSNV |
| F45 | F45-12 | 1117 | E | 0.5875 | A:14239;G:20270;C:4;T:0;total:34513 | iSNV |
| F45 | F45-12 | 1131 | E | 0.3059 | A:3;G:11081;C:25123;T:6;total:36213 | iSNV |
| F45 | F45-12 | 1218 | E | 0.4036 | A:2;G:5;C:22040;T:14926;total:36973 | iSNV |
| F45 | F45-12 | 1428 | E | 0.0881 | A:37260;G:3602;C:1;T:6;total:40869 | iSNV |
| F45 | F45-12 | 1439 | E | 0.0229 | A:0;G:6;C:918;T:39067;total:39991 | iSNV |
| F45 | F45-12 | 1447 | E | 0.1194 | A:4951;G:2;C:36473;T:26;total:41452 | iSNV |
| F45 | F45-12 | 1453 | E | 0.0476 | A:1936;G:5;C:29;T:38631;total:40601 | iSNV |
| F45 | F45-12 | 1512 | E | 0.0634 | A:33439;G:2266;C:4;T:4;total:35713 | iSNV |
| F45 | F45-12 | 1551 | E | 0.0389 | A:6;G:1358;C:33448;T:13;total:34825 | iSNV |
| F45 | F45-12 | 1640 | E | 0.0306 | A:1051;G:2;C:33265;T:13;total:34331 | iSNV |
| F45 | F45-12 | 2078 | E | 0.0219 | A:2;G:4;C:31704;T:711;total:32421 | iSNV |
| F45 | F45-12 | 2277 | E | 0.587 | A:5;G:0;C:18616;T:13103;total:31724 | iSNV |
| F45 | F45-12 | 2362 | E | 0.1165 | A:1;G:22730;C:2999;T:2;total:25732 | iSNV |
| F45 | F45-12 | 2369 | E | 0.1422 | A:3686;G:22230;C:2;T:1;total:25919 | iSNV |
| F45 | F45-12 | 3869 | NS2A | 0.3836 | A:4;G:4;C:23655;T:14730;total:38393 | iSNV |
| F45 | F45-12 | 3959 | NS2A | 0.1266 | A:2;G:4;C:35279;T:5116;total:40401 | iSNV |
| F45 | F45-12 | 4068 | NS2A | 0.0948 | A:28390;G:4;C:3;T:2975;total:31372 | iSNV |
| F45 | F45-12 | 4070 | NS2A | 0.0248 | A:1;G:787;C:7;T:30890;total:31685 | iSNV |
| F45 | F45-12 | 4293 | NS2B | 0.3114 | A:10135;G:22403;C:0;T:5;total:32543 | iSNV |
| F45 | F45-12 | 4697 | NS3 | 0.025 | A:2;G:9;C:1143;T:44448;total:45602 | iSNV |
| F45 | F45-12 | 4974 | NS3 | 0.3154 | A:12725;G:27518;C:96;T:3;total:40342 | iSNV |
| F45 | F45-12 | 5225 | NS3 | 0.0206 | A:33300;G:702;C:5;T:2;total:34009 | iSNV |
| F45 | F45-12 | 5256 | NS3 | 0.0404 | A:3;G:6;C:33478;T:1412;total:34899 | iSNV |
| F45 | F45-12 | 5266 | NS3 | 0.0309 | A:2;G:1;C:33916;T:1084;total:35003 | iSNV |
| F45 | F45-12 | 5537 | NS3 | 0.249 | A:30420;G:10088;C:1;T:2;total:40511 | iSNV |
| F45 | F45-12 | 5736 | NS3 | 0.0456 | A:2137;G:7;C:44675;T:7;total:46826 | iSNV |
| F45 | F45-12 | 5780 | NS3 | 0.1656 | A:1;G:8;C:7821;T:39379;total:47209 | iSNV |
| F45 | F45-12 | 5813 | NS3 | 0.0227 | A:7;G:5;C:43855;T:1019;total:44886 | iSNV |
| F45 | F45-12 | 5952 | NS3 | 0.0245 | A:3;G:3;C:45642;T:1150;total:46798 | iSNV |

| | | | | | | |
|---|---|---|---|---|---|---|
| F45 | F45-12 | 6672 | NS4A | 0.0202 | A:2;G:9;C:31449;T:651;total:32111 | iSNV |
| F45 | F45-12 | 6714 | NS4A | 0.0388 | A:1329;G:32919;C:0;T:4;total:34252 | iSNV |
| F45 | F45-12 | 6867 | NS4A | 0.0307 | A:1049;G:33005;C:3;T:3;total:34060 | iSNV |
| F45 | F45-12 | 6900 | NS4A | 0.0461 | A:1573;G:32489;C:2;T:5;total:34069 | iSNV |
| F45 | F45-12 | 7264 | NS4A | 0.048 | A:2;G:1;C:24728;T:1249;total:25980 | iSNV |
| F45 | F45-12 | 7633 | NS4B | 0.6094 | A:2;G:6;C:14975;T:23353;total:38336 | iSNV |
| F45 | F45-12 | 7634 | NS4B | 0.1396 | A:0;G:5421;C:8;T:33391;total:38820 | iSNV |
| F45 | F45-12 | 8546 | NS5 | 0.0951 | A:4;G:4;C:41001;T:4314;total:45323 | iSNV |
| F45 | F45-12 | 9359 | NS5 | 0.0263 | A:2;G:6;C:1188;T:43970;total:45166 | iSNV |
| F45 | F45-12 | 9491 | NS5 | 0.1634 | A:32468;G:6343;C:2;T:1;total:38814 | iSNV |
| F45 | F45-12 | 9699 | NS5 | 0.3335 | A:3;G:7;C:23014;T:11524;total:34548 | iSNV |
| F45 | F45-12 | 9728 | NS5 | 0.0251 | A:1;G:1;C:33308;T:859;total:34169 | iSNV |
| F45 | F45-12 | 9839 | NS5 | 0.0254 | A:1;G:6;C:866;T:33203;total:34076 | iSNV |
| F45 | F45-12 | 10259 | NS5 | 0.6092 | A:15779;G:24578;C:2;T:7;total:40366 | iSNV |
| F45 | F45-12 | 10347 | NS5 | 0.3112 | A:4;G:5;C:25178;T:11380;total:36567 | iSNV |
| F45 | F45-12 | 10428 | 3'-UTR | 0.5333 | A:4;G:6;C:14371;T:16410;total:30791 | iSNV |
| F45 | F45-12 | 10447 | 3'-UTR | 0.5772 | A:6;G:3;C:12002;T:16373;total:28384 | iSNV |
| F45 | F45-12 | 10566 | 3'-UTR | 0.0264 | A:3;G:4;C:27548;T:750;total:28305 | iSNV |
| F45 | F45-13 | 395 | C | 0.9381 | A:1816;G:27503;C:1;T:1;total:29321 | iSNV |
| F45 | F45-13 | 530 | M | 0.1013 | A:0;G:2;C:22645;T:2555;total:25202 | iSNV |
| F45 | F45-13 | 1113 | E | 0.3359 | A:14195;G:2;C:7186;T:4;total:21387 | iSNV |
| F45 | F45-13 | 1175 | E | 0.0237 | A:1;G:3;C:588;T:24214;total:24806 | iSNV |
| F45 | F45-13 | 1218 | E | 0.9989 | A:1;G:3;C:21;T:21913;total:21938 | SNP |
| F45 | F45-13 | 1413 | E | 0.9384 | A:1646;G:25066;C:3;T:5;total:26720 | iSNV |
| F45 | F45-13 | 1512 | E | 0.2038 | A:17795;G:4556;C:1;T:0;total:22352 | iSNV |
| F45 | F45-13 | 1708 | E | 0.2423 | A:5729;G:17909;C:1;T:2;total:23641 | iSNV |
| F45 | F45-13 | 1769 | E | 0.0243 | A:0;G:3;C:634;T:25355;total:25992 | iSNV |
| F45 | F45-13 | 1796 | E | 0.3331 | A:6;G:18409;C:4;T:9203;total:27622 | iSNV |
| F45 | F45-13 | 1797 | E | 0.0443 | A:6;G:1222;C:3;T:26296;total:27527 | iSNV |
| F45 | F45-13 | 2543 | NS1 | 0.0342 | A:1;G:2;C:19008;T:675;total:19686 | iSNV |
| F45 | F45-13 | 2786 | NS1 | 0.0595 | A:2;G:1;C:30465;T:1928;total:32396 | iSNV |
| F45 | F45-13 | 3482 | NS1 | 0.0203 | A:4;G:4;C:13298;T:276;total:13582 | iSNV |
| F45 | F45-13 | 3507 | NS1 | 0.0201 | A:15671;G:2;C:323;T:3;total:15999 | iSNV |
| F45 | F45-13 | 3869 | NS2A | 0.9979 | A:1;G:6;C:48;T:25120;total:25175 | SNP |
| F45 | F45-13 | 3906 | NS2A | 0.1933 | A:5397;G:22512;C:2;T:3;total:27914 | iSNV |
| F45 | F45-13 | 4052 | NS2A | 0.0912 | A:2;G:0;C:17731;T:1780;total:19513 | iSNV |
| F45 | F45-13 | 4592 | NS2B | 0.0271 | A:26155;G:730;C:1;T:14;total:26900 | iSNV |
| F45 | F45-13 | 4632 | NS3 | 0.042 | A:3;G:1290;C:4;T:29350;total:30647 | iSNV |
| F45 | F45-13 | 4792 | NS3 | 0.0425 | A:29185;G:1297;C:1;T:2;total:30485 | iSNV |
| F45 | F45-13 | 5372 | NS3 | 0.0366 | A:2;G:3;C:796;T:20923;total:21724 | iSNV |
| F45 | F45-13 | 5490 | NS3 | 0.0645 | A:26598;G:1835;C:0;T:2;total:28435 | iSNV |
| F45 | F45-13 | 6867 | NS4A | 0.0369 | A:795;G:20694;C:2;T:0;total:21491 | iSNV |
| F45 | F45-13 | 6900 | NS4A | 0.0769 | A:178;G:19441;C:1;T:1635;total:21255 | iSNV |
| F45 | F45-13 | 6934 | NS4A | 0.0681 | A:20775;G:1520;C:10;T:2;total:22307 | iSNV |
| F45 | F45-13 | 6969 | NS4A | 0.3282 | A:14863;G:3;C:7266;T:2;total:22134 | iSNV |
| F45 | F45-13 | 6970 | NS4A | 0.0964 | A:19937;G:5;C:2129;T:2;total:22073 | iSNV |
| F45 | F45-13 | 7176 | NS4A | 0.0933 | A:15536;G:1600;C:3;T:0;total:17139 | iSNV |
| F45 | F45-13 | 7256 | NS4A | 0.0294 | A:467;G:15406;C:1;T:1;total:15875 | iSNV |
| F45 | F45-13 | 7595 | NS4B | 0.3673 | A:8307;G:3;C:16;T:14288;total:22614 | iSNV |
| F45 | F45-13 | 7633 | NS4B | 0.2749 | A:0;G:4;C:18588;T:7050;total:25642 | iSNV |
| F45 | F45-13 | 7682 | NS5 | 0.0271 | A:699;G:25011;C:5;T:1;total:25716 | iSNV |
| F45 | F45-13 | 8080 | NS5 | 0.0201 | A:25969;G:534;C:0;T:1;total:26504 | iSNV |
| F45 | F45-13 | 9690 | NS5 | 0.0452 | A:934;G:19723;C:5;T:1;total:20663 | iSNV |
| F45 | F45-13 | 9881 | NS5 | 0.0405 | A:21686;G:916;C:1;T:4;total:22607 | iSNV |
| F45 | F45-13 | 10376 | NS5 | 0.934 | A:19394;G:1371;C:1;T:0;total:20766 | iSNV |
| F45 | F45-13 | 10428 | 3'-UTR | 0.8166 | A:2;G:3;C:3418;T:15208;total:18631 | iSNV |
| F45 | F45-13 | 10577 | 3'-UTR | 0.0394 | A:44;G:0;C:17171;T:707;total:17922 | iSNV |
| F45 | F45-13 | 10579 | 3'-UTR | 0.3417 | A:2;G:1;C:11817;T:6136;total:17956 | iSNV |
| F45 | F45-14 | 516 | M | 0.0742 | A:22150;G:1776;C:2;T:0;total:23928 | iSNV |
| F45 | F45-14 | 686 | M | 0.0618 | A:0;G:0;C:20922;T:1380;total:22302 | iSNV |
| F45 | F45-14 | 836 | M | 0.0852 | A:0;G:7;C:1587;T:17032;total:18626 | iSNV |
| F45 | F45-14 | 998 | E | 0.6636 | A:0;G:3;C:5777;T:11388;total:17168 | iSNV |
| F45 | F45-14 | 1057 | E | 0.1127 | A:1712;G:13472;C:3;T:1;total:15188 | iSNV |
| F45 | F45-14 | 1067 | E | 0.1112 | A:0;G:3;C:14850;T:1859;total:16712 | iSNV |
| F45 | F45-14 | 1076 | E | 0.02 | A:0;G:4;C:15794;T:323;total:16121 | iSNV |
| F45 | F45-14 | 1117 | E | 0.2829 | A:12876;G:5082;C:0;T:3;total:17961 | iSNV |
| F45 | F45-14 | 1151 | E | 0.0323 | A:6;G:8;C:603;T:18033;total:18650 | iSNV |
| F45 | F45-14 | 1218 | E | 0.9982 | A:2;G:4;C:29;T:18617;total:18652 | SNP |
| F45 | F45-14 | 1343 | E | 0.0696 | A:3;G:0;C:20979;T:1572;total:22554 | iSNV |
| F45 | F45-14 | 1512 | E | 0.4317 | A:10701;G:8133;C:3;T:0;total:18837 | iSNV |
| F45 | F45-14 | 1682 | E | 0.0207 | A:2;G:3;C:16271;T:345;total:16621 | iSNV |
| F45 | F45-14 | 1769 | E | 0.0599 | A:1289;G:2;C:2;T:20198;total:21491 | iSNV |
| F45 | F45-14 | 2369 | E | 0.2681 | A:3381;G:8929;C:0;T:300;total:12610 | iSNV |
| F45 | F45-14 | 3167 | NS1 | 0.1064 | A:1;G:0;C:22051;T:2628;total:24680 | iSNV |
| F45 | F45-14 | 3242 | NS1 | 0.025 | A:3;G:1;C:660;T:25711;total:26375 | iSNV |
| F45 | F45-14 | 3267 | NS1 | 0.0223 | A:21865;G:501;C:2;T:1;total:22369 | iSNV |
| F45 | F45-14 | 3869 | NS2A | 0.4533 | A:1;G:0;C:11822;T:9804;total:21627 | iSNV |
| F45 | F45-14 | 3950 | NS2A | 0.0559 | A:12;G:3;C:21599;T:1281;total:22895 | iSNV |
| F45 | F45-14 | 3985 | NS2A | 0.0274 | A:2;G:0;C:19953;T:563;total:20518 | iSNV |
| F45 | F45-14 | 4043 | NS2A | 0.0471 | A:2;G:1;C:15805;T:782;total:16590 | iSNV |
| F45 | F45-14 | 4268 | NS2B | 0.0552 | A:14398;G:842;C:2;T:0;total:15242 | iSNV |
| F45 | F45-14 | 4389 | NS2B | 0.0594 | A:1106;G:17498;C:0;T:0;total:18604 | iSNV |

| | | | | | | |
|---|---|---|---|---|---|---|
| F45 | F45-14 | 4697 | NS3 | 0.6538 | A:1;G:3;C:16112;T:8537;total:24653 | iSNV |
| F45 | F45-14 | 4974 | NS3 | 0.0589 | A:1307;G:20863;C:1;T:1;total:22172 | iSNV |
| F45 | F45-14 | 5359 | NS3 | 0.039 | A:18679;G:5;C:759;T:0;total:19443 | iSNV |
| F45 | F45-14 | 5558 | NS3 | 0.288 | A:6621;G:16358;C:3;T:0;total:22982 | iSNV |
| F45 | F45-14 | 5736 | NS3 | 0.0397 | A:1075;G:3;C:25939;T:2;total:27019 | iSNV |
| F45 | F45-14 | 5822 | NS3 | 0.0489 | A:2;G:2;C:1281;T:24909;total:26194 | iSNV |
| F45 | F45-14 | 5952 | NS3 | 0.649 | A:0;G:3;C:8990;T:16615;total:25608 | iSNV |
| F45 | F45-14 | 6010 | NS3 | 0.0504 | A:22412;G:1192;C:4;T:2;total:23610 | iSNV |
| F45 | F45-14 | 6867 | NS4A | 0.184 | A:3265;G:14470;C:1;T:0;total:17736 | iSNV |
| F45 | F45-14 | 7112 | NS4A | 0.0268 | A:15431;G:7;C:427;T:45;total:15910 | iSNV |
| F45 | F45-14 | 7151 | NS4A | 0.0229 | A:0;G:1;C:17506;T:412;total:17919 | iSNV |
| F45 | F45-14 | 7334 | NS4B | 0.0498 | A:12285;G:645;C:1;T:1;total:12932 | iSNV |
| F45 | F45-14 | 7561 | NS4B | 0.1107 | A:3;G:15;C:2116;T:16972;total:19106 | iSNV |
| F45 | F45-14 | 7633 | NS4B | 0.5673 | A:0;G:3;C:9139;T:11978;total:21120 | iSNV |
| F45 | F45-14 | 7667 | NS4B | 0.0231 | A:3;G:0;C:21593;T:511;total:22107 | iSNV |
| F45 | F45-14 | 8201 | NS5 | 0.0226 | A:20268;G:469;C:2;T:1;total:20740 | iSNV |
| F45 | F45-14 | 8398 | NS5 | 0.0204 | A:527;G:2;C:5;T:25251;total:25785 | iSNV |
| F45 | F45-14 | 9068 | NS5 | 0.0652 | A:5;G:6;C:1168;T:16720;total:17899 | iSNV |
| F45 | F45-14 | 9359 | NS5 | 0.6676 | A:1;G:0;C:17942;T:8935;total:26878 | iSNV |
| F45 | F45-14 | 9370 | NS5 | 0.6633 | A:2;G:18102;C:0;T:9193;total:27297 | iSNV |
| F45 | F45-14 | 9880 | NS5 | 0.0263 | A:488;G:18032;C:0;T:1;total:18521 | iSNV |
| F45 | F45-14 | 9923 | NS5 | 0.0515 | A:0;G:1;C:901;T:16583;total:17485 | iSNV |
| F45 | F45-14 | 10259 | NS5 | 0.6566 | A:7646;G:14613;C:1;T:1;total:22261 | iSNV |
| F45 | F45-14 | 10419 | 3'-UTR | 0.0679 | A:2;G:4;C:15630;T:1140;total:16776 | iSNV |
| F45 | F45-14 | 10428 | 3'-UTR | 0.3925 | A:4;G:4;C:10179;T:6583;total:16770 | iSNV |
| F45 | F45-14 | 10435 | 3'-UTR | 0.1116 | A:14813;G:1863;C:3;T:0;total:16679 | iSNV |
| F45 | F45-14 | 10566 | 3'-UTR | 0.6283 | A:2;G:1;C:5172;T:8736;total:13911 | iSNV |
| F45 | F45-15 | 401 | C | 0.0288 | A:28960;G:860;C:4;T:2;total:29826 | iSNV |
| F45 | F45-15 | 469 | C | 0.0242 | A:0;G:0;C:28859;T:718;total:29577 | iSNV |
| F45 | F45-15 | 803 | M | 0.0787 | A:0;G:23197;C:0;T:1982;total:25179 | iSNV |
| F45 | F45-15 | 810 | M | 0.0632 | A:2;G:2;C:23884;T:1614;total:25502 | iSNV |
| F45 | F45-15 | 998 | E | 0.108 | A:0;G:1;C:22547;T:2731;total:25279 | iSNV |
| F45 | F45-15 | 1116 | E | 0.0867 | A:24525;G:2330;C:2;T:0;total:26857 | iSNV |
| F45 | F45-15 | 1218 | E | 0.9987 | A:1;G:2;C:33;T:25828;total:25864 | SNP |
| F45 | F45-15 | 1428 | E | 0.1931 | A:25663;G:6144;C:2;T:1;total:31810 | iSNV |
| F45 | F45-15 | 1447 | E | 0.0405 | A:1358;G:0;C:32143;T:22;total:33523 | iSNV |
| F45 | F45-15 | 1512 | E | 0.1259 | A:25538;G:3682;C:3;T:3;total:29226 | iSNV |
| F45 | F45-15 | 1672 | E | 0.1013 | A:3;G:1;C:24617;T:2778;total:27399 | iSNV |
| F45 | F45-15 | 1797 | E | 0.0467 | A:4;G:1547;C:7;T:31548;total:33106 | iSNV |
| F45 | F45-15 | 1847 | E | 0.0294 | A:31098;G:944;C:1;T:0;total:32043 | iSNV |
| F45 | F45-15 | 2076 | E | 0.1064 | A:21561;G:2570;C:2;T:0;total:24133 | iSNV |
| F45 | F45-15 | 2241 | E | 0.0315 | A:3;G:25856;C:843;T:4;total:26706 | iSNV |
| F45 | F45-15 | 2274 | E | 0.0308 | A:5;G:22720;C:2;T:724;total:23451 | iSNV |
| F45 | F45-15 | 2362 | E | 0.1284 | A:2;G:18642;C:5;T:2748;total:21397 | iSNV |
| F45 | F45-15 | 2493 | NS1 | 0.1694 | A:18706;G:3818;C:2;T:1;total:22527 | iSNV |
| F45 | F45-15 | 2625 | NS1 | 0.0306 | A:2;G:1;C:30036;T:951;total:30990 | iSNV |
| F45 | F45-15 | 3396 | NS1 | 0.0826 | A:1;G:2269;C:5;T:25191;total:27466 | iSNV |
| F45 | F45-15 | 3811 | NS2A | 0.021 | A:26409;G:567;C:9;T:3;total:26988 | iSNV |
| F45 | F45-15 | 3869 | NS2A | 0.7929 | A:1;G:4;C:5680;T:21734;total:27419 | iSNV |
| F45 | F45-15 | 3965 | NS2A | 0.0551 | A:2;G:1;C:27317;T:1594;total:28914 | iSNV |
| F45 | F45-15 | 4136 | NS2A | 0.0224 | A:19614;G:451;C:2;T:2;total:20069 | iSNV |
| F45 | F45-15 | 4233 | NS2B | 0.0801 | A:1;G:1565;C:1;T:17971;total:19538 | iSNV |
| F45 | F45-15 | 4697 | NS3 | 0.1184 | A:2;G:10;C:4024;T:29950;total:33986 | iSNV |
| F45 | F45-15 | 5312 | NS3 | 0.0947 | A:2508;G:3;C:17;T:23928;total:26456 | iSNV |
| F45 | F45-15 | 5448 | NS3 | 0.0359 | A:3;G:0;C:30404;T:1134;total:31541 | iSNV |
| F45 | F45-15 | 5598 | NS3 | 0.0306 | A:899;G:28408;C:2;T:0;total:29309 | iSNV |
| F45 | F45-15 | 5736 | NS3 | 0.1436 | A:4859;G:6;C:28964;T:7;total:33836 | iSNV |
| F45 | F45-15 | 5753 | NS3 | 0.0358 | A:1191;G:32038;C:0;T:3;total:33232 | iSNV |
| F45 | F45-15 | 5952 | NS3 | 0.1192 | A:4;G:2;C:29116;T:3942;total:33064 | iSNV |
| F45 | F45-15 | 5996 | NS3 | 0.1473 | A:1;G:4;C:24766;T:4282;total:29053 | iSNV |
| F45 | F45-15 | 6122 | NS3 | 0.0696 | A:1950;G:26035;C:0;T:5;total:27990 | iSNV |
| F45 | F45-15 | 6710 | NS4A | 0.0207 | A:2;G:2;C:499;T:23595;total:24098 | iSNV |
| F45 | F45-15 | 6714 | NS4A | 0.0726 | A:1802;G:22987;C:0;T:4;total:24793 | iSNV |
| F45 | F45-15 | 6867 | NS4A | 0.0888 | A:2311;G:23693;C:1;T:4;total:26009 | iSNV |
| F45 | F45-15 | 7116 | NS4A | 0.0385 | A:1;G:1;C:24493;T:983;total:25478 | iSNV |
| F45 | F45-15 | 7167 | NS4A | 0.0807 | A:2112;G:24055;C:0;T:4;total:26171 | iSNV |
| F45 | F45-15 | 7283 | NS4B | 0.0381 | A:0;G:2;C:20215;T:802;total:21019 | iSNV |
| F45 | F45-15 | 7528 | NS4B | 0.0351 | A:2;G:5;C:22966;T:836;total:23809 | iSNV |
| F45 | F45-15 | 7633 | NS4B | 0.6844 | A:1;G:2;C:8698;T:18858;total:27559 | iSNV |
| F45 | F45-15 | 7735 | NS5 | 0.0793 | A:1983;G:22994;C:1;T:1;total:24979 | iSNV |
| F45 | F45-15 | 8396 | NS5 | 0.0371 | A:30486;G:1175;C:0;T:0;total:31661 | iSNV |
| F45 | F45-15 | 8518 | NS5 | 0.0763 | A:33566;G:9;C:2;T:2774;total:36351 | iSNV |
| F45 | F45-15 | 8693 | NS5 | 0.03 | A:2;G:2;C:24334;T:755;total:25093 | iSNV |
| F45 | F45-15 | 9245 | NS5 | 0.0388 | A:1;G:2;C:34059;T:1378;total:35440 | iSNV |
| F45 | F45-15 | 9293 | NS5 | 0.1539 | A:4879;G:26802;C:6;T:4;total:31691 | iSNV |
| F45 | F45-15 | 9359 | NS5 | 0.1219 | A:2;G:3;C:4034;T:29043;total:33082 | iSNV |
| F45 | F45-15 | 9370 | NS5 | 0.0838 | A:2825;G:218;C:11;T:30631;total:33685 | iSNV |
| F45 | F45-15 | 9443 | NS5 | 0.0343 | A:33698;G:1199;C:1;T:2;total:34900 | iSNV |
| F45 | F45-15 | 9634 | NS5 | 0.0338 | A:3;G:7;C:1064;T:30381;total:31455 | iSNV |
| F45 | F45-15 | 9818 | NS5 | 0.0579 | A:1;G:4;C:1697;T:27591;total:29293 | iSNV |
| F45 | F45-15 | 10259 | NS5 | 0.1236 | A:27507;G:3882;C:3;T:1;total:31393 | iSNV |
| F45 | F45-15 | 10295 | NS5 | 0.0497 | A:2;G:6;C:29204;T:1531;total:30743 | iSNV |

| | | | | | | |
|---|---|---|---|---|---|---|
| F45 | F45-15 | 10368 | NS5 | 0.0394 | A:976;G:2;C:10;T:23752;total:24740 | iSNV |
| F45 | F45-15 | 10428 | 3'-UTR | 0.4105 | A:2;G:2;C:13282;T:9254;total:22540 | iSNV |
| F45 | F45-15 | 10566 | 3'-UTR | 0.1111 | A:3;G:1;C:19445;T:2433;total:21882 | iSNV |
| F45 | F45-15 | 10571 | 3'-UTR | 0.0304 | A:21340;G:24;C:672;T:1;total:22037 | iSNV |
| F45 | F45-15 | 10718 | 3'-UTR | 0.0903 | A:3;G:2;C:22293;T:2214;total:24512 | iSNV |
| F45 | F45-16 | 178 | C | 0.0294 | A:1030;G:11;C:2;T:33942;total:34985 | iSNV |
| F45 | F45-16 | 469 | C | 0.0232 | A:1;G:4;C:31141;T:740;total:31886 | iSNV |
| F45 | F45-16 | 803 | M | 0.1713 | A:2;G:19277;C:4;T:3988;total:23271 | iSNV |
| F45 | F45-16 | 810 | M | 0.0348 | A:2;G:1;C:23015;T:831;total:23849 | iSNV |
| F45 | F45-16 | 869 | M | 0.02 | A:567;G:2;C:4;T:27666;total:28239 | iSNV |
| F45 | F45-16 | 998 | E | 0.1237 | A:1;G:2;C:20802;T:2938;total:23743 | iSNV |
| F45 | F45-16 | 1116 | E | 0.054 | A:24101;G:1377;C:4;T:1;total:25483 | iSNV |
| F45 | F45-16 | 1218 | E | 0.9989 | A:0;G:6;C:22;T:25041;total:25069 | SNP |
| F45 | F45-16 | 1428 | E | 0.15 | A:26255;G:4634;C:0;T:0;total:30889 | iSNV |
| F45 | F45-16 | 1447 | E | 0.0525 | A:1664;G:8;C:29942;T:79;total:31693 | iSNV |
| F45 | F45-16 | 1512 | E | 0.1771 | A:22364;G:4817;C:2;T:1;total:27184 | iSNV |
| F45 | F45-16 | 1672 | E | 0.1134 | A:2;G:1;C:21348;T:2731;total:24082 | iSNV |
| F45 | F45-16 | 1797 | E | 0.0381 | A:2;G:1233;C:29;T:31039;total:32303 | iSNV |
| F45 | F45-16 | 2076 | E | 0.0834 | A:19396;G:1765;C:1;T:0;total:21162 | iSNV |
| F45 | F45-16 | 2264 | E | 0.0251 | A:0;G:4;C:551;T:21388;total:21943 | iSNV |
| F45 | F45-16 | 2274 | E | 0.0241 | A:2;G:21911;C:1;T:542;total:22456 | iSNV |
| F45 | F45-16 | 2285 | E | 0.0251 | A:5;G:0;C:22278;T:574;total:22857 | iSNV |
| F45 | F45-16 | 2362 | E | 0.1501 | A:1;G:15154;C:3;T:2679;total:17837 | iSNV |
| F45 | F45-16 | 2493 | NS1 | 0.1024 | A:18298;G:2088;C:1;T:2;total:20389 | iSNV |
| F45 | F45-16 | 2625 | NS1 | 0.023 | A:5;G:0;C:29865;T:704;total:30574 | iSNV |
| F45 | F45-16 | 3161 | NS1 | 0.023 | A:30715;G:725;C:1;T:1;total:31442 | iSNV |
| F45 | F45-16 | 3396 | NS1 | 0.168 | A:0;G:4222;C:3;T:20899;total:25124 | iSNV |
| F45 | F45-16 | 3811 | NS2A | 0.0234 | A:24417;G:586;C:2;T:1;total:25006 | iSNV |
| F45 | F45-16 | 3869 | NS2A | 0.6745 | A:1;G:4;C:9379;T:19422;total:28806 | iSNV |
| F45 | F45-16 | 3965 | NS2A | 0.0585 | A:1;G:3;C:28083;T:1748;total:29835 | iSNV |
| F45 | F45-16 | 4136 | NS2A | 0.0272 | A:16300;G:457;C:0;T:0;total:16757 | iSNV |
| F45 | F45-16 | 4233 | NS2B | 0.0361 | A:1;G:594;C:2;T:15840;total:16437 | iSNV |
| F45 | F45-16 | 4697 | NS3 | 0.1384 | A:1;G:5;C:4743;T:29500;total:34249 | iSNV |
| F45 | F45-16 | 4800 | NS3 | 0.0242 | A:3;G:3;C:859;T:34530;total:35395 | iSNV |
| F45 | F45-16 | 5312 | NS3 | 0.0646 | A:1708;G:8;C:18;T:24689;total:26423 | iSNV |
| F45 | F45-16 | 5448 | NS3 | 0.0307 | A:2;G:1;C:32046;T:1016;total:33065 | iSNV |
| F45 | F45-16 | 5736 | NS3 | 0.1184 | A:4248;G:4;C:31610;T:6;total:35868 | iSNV |
| F45 | F45-16 | 5753 | NS3 | 0.0547 | A:1913;G:33021;C:4;T:3;total:34941 | iSNV |
| F45 | F45-16 | 5952 | NS3 | 0.1377 | A:2;G:2;C:30460;T:4865;total:35329 | iSNV |
| F45 | F45-16 | 5996 | NS3 | 0.077 | A:1;G:3;C:27201;T:2270;total:29475 | iSNV |
| F45 | F45-16 | 6122 | NS3 | 0.164 | A:4617;G:23519;C:0;T:8;total:28144 | iSNV |
| F45 | F45-16 | 6710 | NS4A | 0.0227 | A:1;G:2;C:561;T:24084;total:24648 | iSNV |
| F45 | F45-16 | 6714 | NS4A | 0.0552 | A:1394;G:23830;C:2;T:3;total:25229 | iSNV |
| F45 | F45-16 | 6729 | NS4A | 0.0365 | A:922;G:32;C:24219;T:33;total:25206 | iSNV |
| F45 | F45-16 | 6752 | NS4A | 0.0247 | A:569;G:22389;C:1;T:2;total:22961 | iSNV |
| F45 | F45-16 | 6867 | NS4A | 0.1405 | A:3521;G:21517;C:6;T:2;total:25046 | iSNV |
| F45 | F45-16 | 7116 | NS4A | 0.0634 | A:1;G:0;C:22840;T:1548;total:24389 | iSNV |
| F45 | F45-16 | 7167 | NS4A | 0.0867 | A:2152;G:22647;C:1;T:2;total:24802 | iSNV |
| F45 | F45-16 | 7283 | NS4B | 0.0284 | A:1;G:2;C:17927;T:525;total:18455 | iSNV |
| F45 | F45-16 | 7528 | NS4B | 0.0354 | A:1;G:3;C:21818;T:801;total:22623 | iSNV |
| F45 | F45-16 | 7626 | NS4B | 0.0254 | A:704;G:11;C:17;T:26954;total:27686 | iSNV |
| F45 | F45-16 | 7633 | NS4B | 0.6658 | A:4;G:3;C:9320;T:18558;total:27885 | iSNV |
| F45 | F45-16 | 7735 | NS5 | 0.1623 | A:4184;G:21591;C:2;T:0;total:25777 | iSNV |
| F45 | F45-16 | 8518 | NS5 | 0.1641 | A:29617;G:6;C:6;T:5819;total:35448 | iSNV |
| F45 | F45-16 | 8693 | NS5 | 0.0302 | A:1;G:5;C:21795;T:679;total:22480 | iSNV |
| F45 | F45-16 | 8942 | NS5 | 0.0302 | A:1;G:1;C:27429;T:856;total:28287 | iSNV |
| F45 | F45-16 | 9293 | NS5 | 0.1228 | A:3911;G:27920;C:4;T:0;total:31835 | iSNV |
| F45 | F45-16 | 9359 | NS5 | 0.1437 | A:2;G:9;C:5075;T:30227;total:35313 | iSNV |
| F45 | F45-16 | 9370 | NS5 | 0.0365 | A:1319;G:101;C:89;T:34624;total:36133 | iSNV |
| F45 | F45-16 | 9634 | NS5 | 0.0294 | A:4;G:7;C:796;T:26259;total:27066 | iSNV |
| F45 | F45-16 | 9818 | NS5 | 0.0798 | A:2;G:2;C:2022;T:23308;total:25334 | iSNV |
| F45 | F45-16 | 10259 | NS5 | 0.1425 | A:26534;G:4412;C:1;T:1;total:30948 | iSNV |
| F45 | F45-16 | 10295 | NS5 | 0.0379 | A:1;G:6;C:29416;T:1162;total:30585 | iSNV |
| F45 | F45-16 | 10351 | NS5 | 0.032 | A:852;G:25688;C:3;T:3;total:26546 | iSNV |
| F45 | F45-16 | 10368 | NS5 | 0.0497 | A:1238;G:2;C:9;T:23659;total:24908 | iSNV |
| F45 | F45-16 | 10428 | 3'-UTR | 0.4083 | A:5;G:1;C:13087;T:9035;total:22128 | iSNV |
| F45 | F45-16 | 10447 | 3'-UTR | 0.022 | A:0;G:15;C:20678;T:467;total:21160 | iSNV |
| F45 | F45-16 | 10566 | 3'-UTR | 0.1375 | A:4;G:2;C:16641;T:2655;total:19302 | iSNV |
| F45 | F45-16 | 10718 | 3'-UTR | 0.0676 | A:1;G:1;C:21306;T:1545;total:22853 | iSNV |
| F45 | F45-17 | 645 | M | 0.1871 | A:39696;G:9142;C:1;T:0;total:48839 | iSNV |
| F45 | F45-17 | 943 | M | 0.0338 | A:11;G:0;C:46203;T:1619;total:47833 | iSNV |
| F45 | F45-17 | 1057 | E | 0.0776 | A:3479;G:41342;C:2;T:6;total:44829 | iSNV |
| F45 | F45-17 | 1218 | E | 0.9993 | A:1;G:3;C:31;T:47009;total:47044 | SNP |
| F45 | F45-17 | 1296 | E | 0.0245 | A:7;G:5;C:1374;T:54475;total:55861 | iSNV |
| F45 | F45-17 | 1413 | E | 0.4753 | A:32247;G:29224;C:9;T:3;total:61483 | iSNV |
| F45 | F45-17 | 1428 | E | 0.1685 | A:50430;G:10228;C:3;T:4;total:60665 | iSNV |
| F45 | F45-17 | 1447 | E | 0.1828 | A:11374;G:7;C:50813;T:19;total:62213 | iSNV |
| F45 | F45-17 | 1512 | E | 0.4666 | A:27600;G:743;C:24802;T:2;total:53147 | iSNV |
| F45 | F45-17 | 1797 | E | 0.0205 | A:2;G:1263;C:181;T:60023;total:61469 | iSNV |
| F45 | F45-17 | 1976 | E | 0.0621 | A:6;G:21;C:43181;T:2861;total:46069 | iSNV |
| F45 | F45-17 | 2070 | E | 0.1781 | A:32210;G:6985;C:5;T:3;total:39203 | iSNV |
| F45 | F45-17 | 2376 | E | 0.1822 | A:7;G:3;C:31272;T:6970;total:38252 | iSNV |

| | | | | | | |
|---|---|---|---|---|---|---|
| F45 | F45-17 | 2960 | NS1 | 0.1622 | A:2;G:1;C:51631;T:9998;total:61632 | iSNV |
| F45 | F45-17 | 3002 | NS1 | 0.468 | A:2;G:5;C:27786;T:24458;total:52251 | iSNV |
| F45 | F45-17 | 3211 | NS1 | 0.0348 | A:66864;G:12;C:2415;T:4;total:69295 | iSNV |
| F45 | F45-17 | 3317 | NS1 | 0.021 | A:12;G:2;C:57248;T:1231;total:58493 | iSNV |
| F45 | F45-17 | 3869 | NS2A | 0.9985 | A:1;G:2;C:77;T:52562;total:52642 | SNP |
| F45 | F45-17 | 4091 | NS2A | 0.4344 | A:17621;G:22936;C:0;T:1;total:40558 | iSNV |
| F45 | F45-17 | 4233 | NS2B | 0.0239 | A:4;G:764;C:0;T:31067;total:31835 | iSNV |
| F45 | F45-17 | 4447 | NS2B | 0.0734 | A:39227;G:3109;C:2;T:1;total:42339 | iSNV |
| F45 | F45-17 | 5327 | NS3 | 0.4665 | A:23579;G:3;C:6;T:20630;total:44218 | iSNV |
| F45 | F45-17 | 5415 | NS3 | 0.1588 | A:6;G:2;C:46971;T:8870;total:55849 | iSNV |
| F45 | F45-17 | 5518 | NS3 | 0.0882 | A:56857;G:5505;C:8;T:3;total:62373 | iSNV |
| F45 | F45-17 | 5575 | NS3 | 0.0738 | A:4;G:4;C:51824;T:4132;total:55964 | iSNV |
| F45 | F45-17 | 5705 | NS3 | 0.0744 | A:54717;G:4401;C:6;T:4;total:59128 | iSNV |
| F45 | F45-17 | 5718 | NS3 | 0.079 | A:4901;G:57127;C:2;T:5;total:62035 | iSNV |
| F45 | F45-17 | 6867 | NS4A | 0.0268 | A:1214;G:44000;C:5;T:0;total:45219 | iSNV |
| F45 | F45-17 | 6970 | NS4A | 0.1565 | A:43399;G:78;C:8069;T:4;total:51550 | iSNV |
| F45 | F45-17 | 7151 | NS4A | 0.2047 | A:6;G:3;C:42230;T:10876;total:53115 | iSNV |
| F45 | F45-17 | 7167 | NS4A | 0.0719 | A:3734;G:48184;C:6;T:3;total:51927 | iSNV |
| F45 | F45-17 | 7633 | NS4B | 0.953 | A:1;G:3;C:2305;T:46669;total:48978 | iSNV |
| F45 | F45-17 | 8430 | NS5 | 0.4634 | A:8;G:14;C:32373;T:27984;total:60379 | iSNV |
| F45 | F45-17 | 10364 | NS5 | 0.2037 | A:4;G:0;C:40697;T:10417;total:51118 | iSNV |
| F45 | F45-17 | 10376 | NS5 | 0.4671 | A:23736;G:27069;C:0;T:2;total:50807 | iSNV |
| F45 | F45-17 | 10428 | 3'-UTR | 0.2927 | A:10;G:5;C:32384;T:13412;total:45811 | iSNV |
| F45 | F45-17 | 10447 | 3'-UTR | 0.1186 | A:10;G:7;C:37110;T:4998;total:42125 | iSNV |
| F45 | F45-17 | 10665 | 3'-UTR | 0.0217 | A:4;G:1;C:45674;T:1015;total:46694 | iSNV |
| F45 | F45-18 | 948 | M | 0.0264 | A:5;G:2;C:40006;T:1085;total:41098 | iSNV |
| F45 | F45-18 | 998 | E | 0.2506 | A:2;G:1;C:27872;T:9325;total:37200 | iSNV |
| F45 | F45-18 | 1218 | E | 0.9979 | A:0;G:1;C:82;T:39272;total:39355 | SNP |
| F45 | F45-18 | 1413 | E | 0.6743 | A:16187;G:33502;C:6;T:2;total:49697 | iSNV |
| F45 | F45-18 | 1512 | E | 0.6359 | A:14520;G:35;C:25322;T:1;total:39878 | iSNV |
| F45 | F45-18 | 1562 | E | 0.2477 | A:26794;G:8827;C:7;T:3;total:35631 | iSNV |
| F45 | F45-18 | 1797 | E | 0.3082 | A:20;G:15064;C:6;T:33785;total:48875 | iSNV |
| F45 | F45-18 | 2181 | E | 0.2933 | A:30727;G:12761;C:4;T:7;total:43499 | iSNV |
| F45 | F45-18 | 3660 | NS1 | 0.0324 | A:1115;G:33288;C:0;T:4;total:34407 | iSNV |
| F45 | F45-18 | 3746 | NS2A | 0.0271 | A:27650;G:772;C:2;T:0;total:28424 | iSNV |
| F45 | F45-18 | 3869 | NS2A | 0.7494 | A:4;G:2;C:11529;T:34462;total:45997 | iSNV |
| F45 | F45-18 | 4091 | NS2A | 0.5873 | A:18579;G:13060;C:3;T:1;total:31643 | iSNV |
| F45 | F45-18 | 4697 | NS3 | 0.2401 | A:6;G:3;C:13100;T:41433;total:54542 | iSNV |
| F45 | F45-18 | 5072 | NS3 | 0.1107 | A:4562;G:36634;C:6;T:3;total:41205 | iSNV |
| F45 | F45-18 | 5327 | NS3 | 0.0338 | A:37741;G:4;C:1;T:1324;total:39070 | iSNV |
| F45 | F45-18 | 5707 | NS3 | 0.0211 | A:53826;G:1162;C:4;T:1;total:54993 | iSNV |
| F45 | F45-18 | 5952 | NS3 | 0.2385 | A:14;G:3;C:44447;T:13927;total:58391 | iSNV |
| F45 | F45-18 | 6322 | NS3 | 0.0317 | A:6;G:1;C:38416;T:1261;total:39684 | iSNV |
| F45 | F45-18 | 6715 | NS4A | 0.0205 | A:4;G:4;C:41646;T:875;total:42529 | iSNV |
| F45 | F45-18 | 6740 | NS4A | 0.2568 | A:6;G:1;C:30558;T:10562;total:41127 | iSNV |
| F45 | F45-18 | 6867 | NS4A | 0.3339 | A:13347;G:26613;C:0;T:4;total:39964 | iSNV |
| F45 | F45-18 | 6900 | NS4A | 0.0591 | A:2286;G:36344;C:2;T:5;total:38637 | iSNV |
| F45 | F45-18 | 6927 | NS4A | 0.035 | A:5;G:2;C:38081;T:1385;total:39473 | iSNV |
| F45 | F45-18 | 7091 | NS4A | 0.0212 | A:8;G:0;C:39345;T:853;total:40206 | iSNV |
| F45 | F45-18 | 7561 | NS4B | 0.2197 | A:5;G:11;C:9194;T:32634;total:41844 | iSNV |
| F45 | F45-18 | 7633 | NS4B | 0.732 | A:2;G:4;C:12119;T:33081;total:45206 | iSNV |
| F45 | F45-18 | 8430 | NS5 | 0.6679 | A:11;G:19;C:17797;T:35756;total:53583 | iSNV |
| F45 | F45-18 | 9359 | NS5 | 0.2449 | A:1;G:4;C:14376;T:44307;total:58688 | iSNV |
| F45 | F45-18 | 9764 | NS5 | 0.4374 | A:8;G:2;C:23264;T:18101;total:41375 | iSNV |
| F45 | F45-18 | 10259 | NS5 | 0.241 | A:41164;G:13075;C:0;T:1;total:54240 | iSNV |
| F45 | F45-18 | 10295 | NS5 | 0.2423 | A:8;G:7;C:41178;T:13176;total:54369 | iSNV |
| F45 | F45-18 | 10376 | NS5 | 0.6775 | A:30435;G:14493;C:4;T:4;total:44936 | iSNV |
| F45 | F45-18 | 10419 | 3'-UTR | 0.0206 | A:11;G:1;C:38536;T:813;total:39361 | iSNV |
| F45 | F45-18 | 10428 | 3'-UTR | 0.2332 | A:5;G:1;C:30252;T:9206;total:39464 | iSNV |
| F45 | F45-18 | 10451 | 3'-UTR | 0.2653 | A:9;G:3;C:25747;T:9306;total:35065 | iSNV |
| F45 | F45-18 | 10566 | 3'-UTR | 0.2303 | A:3;G:4;C:23047;T:6899;total:29953 | iSNV |
| F45 | F45-19 | 869 | M | 0.2547 | A:11431;G:14;C:18;T:33405;total:44868 | iSNV |
| F45 | F45-19 | 998 | E | 0.4437 | A:3;G:4;C:20181;T:16102;total:36290 | iSNV |
| F45 | F45-19 | 1218 | E | 0.9979 | A:1;G:2;C:83;T:39356;total:39442 | SNP |
| F45 | F45-19 | 1363 | E | 0.0322 | A:6;G:3;C:43758;T:1459;total:45226 | iSNV |
| F45 | F45-19 | 1382 | E | 0.201 | A:10;G:2;C:38798;T:9764;total:48574 | iSNV |
| F45 | F45-19 | 1428 | E | 0.4347 | A:27585;G:21216;C:2;T:2;total:48805 | iSNV |
| F45 | F45-19 | 1430 | E | 0.478 | A:6;G:0;C:25514;T:23376;total:48896 | iSNV |
| F45 | F45-19 | 1459 | E | 0.022 | A:1077;G:1;C:47789;T:21;total:48888 | iSNV |
| F45 | F45-19 | 1568 | E | 0.0206 | A:6;G:1;C:33628;T:710;total:34345 | iSNV |
| F45 | F45-19 | 1797 | E | 0.2032 | A:10001;G:1977;C:14;T:37203;total:49195 | iSNV |
| F45 | F45-19 | 2183 | E | 0.0523 | A:2236;G:40439;C:2;T:3;total:42680 | iSNV |
| F45 | F45-19 | 2318 | E | 0.0365 | A:0;G:3;C:1353;T:35658;total:37014 | iSNV |
| F45 | F45-19 | 3646 | NS1 | 0.0233 | A:1;G:7;C:742;T:31025;total:31775 | iSNV |
| F45 | F45-19 | 3869 | NS2A | 0.5438 | A:8;G:0;C:20368;T:24270;total:44646 | iSNV |
| F45 | F45-19 | 4122 | NS2A | 0.029 | A:2;G:24032;C:1;T:718;total:24753 | iSNV |
| F45 | F45-19 | 4236 | NS2B | 0.0241 | A:1;G:0;C:613;T:24792;total:25406 | iSNV |
| F45 | F45-19 | 4538 | NS2B | 0.2588 | A:11;G:2;C:30372;T:10613;total:40998 | iSNV |
| F45 | F45-19 | 4697 | NS3 | 0.4384 | A:5;G:11;C:21888;T:28022;total:49926 | iSNV |
| F45 | F45-19 | 5261 | NS3 | 0.0253 | A:38485;G:1000;C:6;T:0;total:39491 | iSNV |
| F45 | F45-19 | 5313 | NS3 | 0.4444 | A:5;G:4;C:17219;T:21514;total:38742 | iSNV |
| F45 | F45-19 | 5336 | NS3 | 0.026 | A:0;G:3;C:1036;T:38761;total:39800 | iSNV |

| | | | | | | |
|---|---|---|---|---|---|---|
| F45 | F45-19 | 5549 | NS3 | 0.0212 | A:3;G:1;C:1109;T:51183;total:52296 | iSNV |
| F45 | F45-19 | 5598 | NS3 | 0.1054 | A:4623;G:39230;C:4;T:0;total:43857 | iSNV |
| F45 | F45-19 | 5952 | NS3 | 0.4369 | A:9;G:5;C:30097;T:23367;total:53478 | iSNV |
| F45 | F45-19 | 6321 | NS3 | 0.0267 | A:996;G:36192;C:6;T:3;total:37197 | iSNV |
| F45 | F45-19 | 6336 | NS3 | 0.024 | A:27150;G:0;C:3;T:668;total:27821 | iSNV |
| F45 | F45-19 | 6347 | NS3 | 0.0308 | A:28575;G:909;C:3;T:2;total:29489 | iSNV |
| F45 | F45-19 | 6413 | NS3 | 0.0305 | A:1;G:3;C:1174;T:37211;total:38389 | iSNV |
| F45 | F45-19 | 6867 | NS4A | 0.2247 | A:8400;G:28977;C:0;T:2;total:37379 | iSNV |
| F45 | F45-19 | 6900 | NS4A | 0.0493 | A:1787;G:34400;C:4;T:5;total:36196 | iSNV |
| F45 | F45-19 | 7364 | NS4B | 0.0288 | A:0;G:2;C:883;T:29695;total:30580 | iSNV |
| F45 | F45-19 | 7378 | NS4B | 0.0344 | A:3;G:0;C:30950;T:1105;total:32058 | iSNV |
| F45 | F45-19 | 7585 | NS4B | 0.3928 | A:0;G:22793;C:14750;T:4;total:37547 | iSNV |
| F45 | F45-19 | 7633 | NS4B | 0.5315 | A:2;G:2;C:20147;T:22847;total:42998 | iSNV |
| F45 | F45-19 | 8756 | NS5 | 0.0225 | A:32573;G:752;C:9;T:1;total:33335 | iSNV |
| F45 | F45-19 | 9359 | NS5 | 0.4406 | A:5;G:4;C:24215;T:30735;total:54959 | iSNV |
| F45 | F45-19 | 9818 | NS5 | 0.0302 | A:0;G:1;C:1135;T:36427;total:37563 | iSNV |
| F45 | F45-19 | 10058 | NS5 | 0.0342 | A:12;G:1;C:30850;T:1095;total:31958 | iSNV |
| F45 | F45-19 | 10249 | NS5 | 0.0713 | A:7;G:3;C:45132;T:3467;total:48609 | iSNV |
| F45 | F45-19 | 10259 | NS5 | 0.4358 | A:27781;G:21465;C:1;T:3;total:49250 | iSNV |
| F45 | F45-19 | 10310 | NS5 | 0.0299 | A:7;G:0;C:46646;T:1440;total:48093 | iSNV |
| F45 | F45-19 | 10358 | NS5 | 0.2742 | A:7;G:0;C:30305;T:11452;total:41764 | iSNV |
| F45 | F45-19 | 10428 | 3'-UTR | 0.7537 | A:2;G:6;C:8839;T:27028;total:35875 | iSNV |
| F45 | F45-19 | 10566 | 3'-UTR | 0.4245 | A:5;G:3;C:16573;T:12234;total:28815 | iSNV |
| F45 | F45-19 | 10854 | 3'-UTR | 0.0218 | A:0;G:1;C:407;T:18203;total:18611 | iSNV |
| F45 | F45-2 | 434 | C | 0.6559 | A:12267;G:6438;C:1;T:1;total:18707 | iSNV |
| F45 | F45-2 | 728 | M | 0.1108 | A:1;G:2;C:17644;T:2199;total:19846 | iSNV |
| F45 | F45-2 | 762 | M | 0.0259 | A:517;G:19404;C:2;T:1;total:19924 | iSNV |
| F45 | F45-2 | 768 | M | 0.061 | A:16486;G:1072;C:2;T:0;total:17560 | iSNV |
| F45 | F45-2 | 998 | E | 0.0388 | A:1;G:1;C:12372;T:500;total:12874 | iSNV |
| F45 | F45-2 | 1057 | E | 0.0214 | A:246;G:11204;C:0;T:0;total:11450 | iSNV |
| F45 | F45-2 | 1166 | E | 0.3489 | A:1;G:5;C:6038;T:11257;total:17301 | iSNV |
| F45 | F45-2 | 1218 | E | 0.9993 | A:0;G:4;C:8;T:15048;total:15060 | SNP |
| F45 | F45-2 | 1295 | E | 0.069 | A:15614;G:7;C:3;T:1158;total:16782 | iSNV |
| F45 | F45-2 | 1331 | E | 0.1729 | A:13856;G:2899;C:2;T:2;total:16759 | iSNV |
| F45 | F45-2 | 1413 | E | 0.1701 | A:14678;G:3010;C:1;T:1;total:17690 | iSNV |
| F45 | F45-2 | 1430 | E | 0.5856 | A:0;G:1;C:6655;T:9402;total:16058 | iSNV |
| F45 | F45-2 | 1446 | E | 0.0829 | A:1319;G:14585;C:1;T:1;total:15906 | iSNV |
| F45 | F45-2 | 1447 | E | 0.046 | A:739;G:0;C:15314;T:3;total:16056 | iSNV |
| F45 | F45-2 | 1453 | E | 0.0464 | A:728;G:6;C:3;T:14942;total:15679 | iSNV |
| F45 | F45-2 | 1512 | E | 0.3931 | A:7742;G:5015;C:0;T:0;total:12757 | iSNV |
| F45 | F45-2 | 2006 | E | 0.0666 | A:653;G:9138;C:0;T:0;total:9791 | iSNV |
| F45 | F45-2 | 2274 | E | 0.6583 | A:0;G:4429;C:2;T:8530;total:12961 | iSNV |
| F45 | F45-2 | 2466 | E | 0.0484 | A:9484;G:483;C:3;T:0;total:9970 | iSNV |
| F45 | F45-2 | 2642 | NS1 | 0.0697 | A:1;G:0;C:17938;T:1346;total:19285 | iSNV |
| F45 | F45-2 | 3493 | NS1 | 0.0496 | A:0;G:474;C:0;T:9069;total:9543 | iSNV |
| F45 | F45-2 | 3644 | NS1 | 0.0605 | A:1;G:2;C:612;T:9494;total:10109 | iSNV |
| F45 | F45-2 | 3669 | NS1 | 0.022 | A:2;G:2;C:9025;T:204;total:9233 | iSNV |
| F45 | F45-2 | 3869 | NS2A | 0.96 | A:0;G:0;C:734;T:17610;total:18344 | iSNV |
| F45 | F45-2 | 4058 | NS2A | 0.2621 | A:0;G:0;C:9601;T:3412;total:13013 | iSNV |
| F45 | F45-2 | 4697 | NS3 | 0.0373 | A:1;G:3;C:706;T:18207;total:18917 | iSNV |
| F45 | F45-2 | 4882 | NS3 | 0.0297 | A:1;G:6;C:19566;T:600;total:20173 | iSNV |
| F45 | F45-2 | 4889 | NS3 | 0.0508 | A:3;G:1;C:19345;T:1036;total:20385 | iSNV |
| F45 | F45-2 | 5450 | NS3 | 0.1167 | A:17568;G:3;C:2;T:2323;total:19896 | iSNV |
| F45 | F45-2 | 5952 | NS3 | 0.0352 | A:0;G:1;C:22231;T:812;total:23044 | iSNV |
| F45 | F45-2 | 6063 | NS3 | 0.0217 | A:3;G:0;C:17608;T:392;total:18003 | iSNV |
| F45 | F45-2 | 6644 | NS4A | 0.0225 | A:0;G:2;C:234;T:10138;total:10374 | iSNV |
| F45 | F45-2 | 6798 | NS4A | 0.0216 | A:0;G:0;C:16923;T:375;total:17298 | iSNV |
| F45 | F45-2 | 6867 | NS4A | 0.2977 | A:4194;G:9891;C:1;T:1;total:14087 | iSNV |
| F45 | F45-2 | 6900 | NS4A | 0.0489 | A:683;G:13274;C:0;T:3;total:13960 | iSNV |
| F45 | F45-2 | 7394 | NS4B | 0.0312 | A:11298;G:365;C:0;T:1;total:11664 | iSNV |
| F45 | F45-2 | 7633 | NS4B | 0.6417 | A:0;G:3;C:6814;T:12196;total:19013 | iSNV |
| F45 | F45-2 | 7799 | NS5 | 0.0562 | A:0;G:0;C:15705;T:936;total:16641 | iSNV |
| F45 | F45-2 | 8249 | NS5 | 0.1065 | A:1;G:6;C:1882;T:15768;total:17657 | iSNV |
| F45 | F45-2 | 8355 | NS5 | 0.0608 | A:1445;G:22309;C:1;T:1;total:23756 | iSNV |
| F45 | F45-2 | 9350 | NS5 | 0.0468 | A:2;G:0;C:21392;T:1052;total:22446 | iSNV |
| F45 | F45-2 | 9359 | NS5 | 0.0388 | A:1;G:3;C:873;T:21621;total:22498 | iSNV |
| F45 | F45-2 | 9575 | NS5 | 0.1517 | A:11174;G:1;C:0;T:1999;total:13174 | iSNV |
| F45 | F45-2 | 9989 | NS5 | 0.0397 | A:0;G:4;C:12664;T:524;total:13192 | iSNV |
| F45 | F45-2 | 10088 | NS5 | 0.115 | A:1611;G:12386;C:2;T:0;total:13999 | iSNV |
| F45 | F45-2 | 10259 | NS5 | 0.0358 | A:17969;G:669;C:0;T:0;total:18638 | iSNV |
| F45 | F45-2 | 10376 | NS5 | 0.1833 | A:2958;G:13171;C:2;T:0;total:16131 | iSNV |
| F45 | F45-2 | 10428 | 3'-UTR | 0.4156 | A:1;G:2;C:7453;T:5304;total:12760 | iSNV |
| F45 | F45-2 | 10447 | 3'-UTR | 0.025 | A:1;G:0;C:11428;T:294;total:11723 | iSNV |
| F45 | F45-2 | 10566 | 3'-UTR | 0.0332 | A:2;G:0;C:10683;T:368;total:11053 | iSNV |
| F45 | F45-2 | 10596 | 3'-UTR | 0.0424 | A:1;G:0;C:536;T:12077;total:12614 | iSNV |
| F45 | F45-2 | 10815 | 3'-UTR | 0.0207 | A:9766;G:207;C:0;T:0;total:9973 | iSNV |
| F45 | F45-20 | 456 | C | 0.0302 | A:993;G:31801;C:4;T:6;total:32804 | iSNV |
| F45 | F45-20 | 614 | M | 0.1145 | A:10;G:23722;C:1;T:3069;total:26802 | iSNV |
| F45 | F45-20 | 782 | M | 0.0332 | A:8;G:27;C:922;T:26753;total:27710 | iSNV |
| F45 | F45-20 | 906 | M | 0.1117 | A:21433;G:2698;C:6;T:2;total:24139 | iSNV |
| F45 | F45-20 | 998 | E | 0.0998 | A:5;G:0;C:21846;T:2423;total:24274 | iSNV |
| F45 | F45-20 | 1117 | E | 0.8754 | A:3239;G:22741;C:0;T:2;total:25982 | iSNV |

| | | | | | | |
|---|---|---|---|---|---|---|
| F45 | F45-20 | 1218 | E | 0.9971 | A:1;G:1;C:75;T:25898;total:25975 | SNP |
| F45 | F45-20 | 1348 | E | 0.0423 | A:30958;G:0;C:1368;T:0;total:32326 | iSNV |
| F45 | F45-20 | 1382 | E | 0.0311 | A:11;G:0;C:31909;T:1026;total:32946 | iSNV |
| F45 | F45-20 | 1459 | E | 0.0444 | A:1446;G:0;C:30987;T:65;total:32498 | iSNV |
| F45 | F45-20 | 1512 | E | 0.025 | A:27071;G:696;C:9;T:0;total:27776 | iSNV |
| F45 | F45-20 | 1593 | E | 0.0206 | A:439;G:20823;C:3;T:1;total:21266 | iSNV |
| F45 | F45-20 | 1721 | E | 0.7828 | A:5555;G:20012;C:1;T:1;total:25569 | iSNV |
| F45 | F45-20 | 1797 | E | 0.0224 | A:3;G:752;C:6;T:32695;total:33456 | iSNV |
| F45 | F45-20 | 1909 | E | 0.0593 | A:1625;G:0;C:25732;T:9;total:27366 | iSNV |
| F45 | F45-20 | 2078 | E | 0.0342 | A:0;G:2;C:20833;T:740;total:21575 | iSNV |
| F45 | F45-20 | 2274 | E | 0.0286 | A:2;G:22364;C:2;T:659;total:23027 | iSNV |
| F45 | F45-20 | 2369 | E | 0.7799 | A:13983;G:3948;C:0;T:2;total:17933 | iSNV |
| F45 | F45-20 | 2531 | NS1 | 0.2138 | A:3;G:5;C:4946;T:18170;total:23124 | iSNV |
| F45 | F45-20 | 2660 | NS1 | 0.0221 | A:0;G:1;C:750;T:33140;total:33891 | iSNV |
| F45 | F45-20 | 3564 | NS1 | 0.0298 | A:2;G:0;C:22784;T:700;total:23486 | iSNV |
| F45 | F45-20 | 3869 | NS2A | 0.892 | A:2;G:1;C:3066;T:25314;total:28383 | iSNV |
| F45 | F45-20 | 3929 | NS2A | 0.7849 | A:6;G:2;C:6922;T:25237;total:32167 | iSNV |
| F45 | F45-20 | 4319 | NS2B | 0.0288 | A:768;G:25851;C:0;T:1;total:26620 | iSNV |
| F45 | F45-20 | 4697 | NS3 | 0.0957 | A:0;G:6;C:3234;T:30529;total:33769 | iSNV |
| F45 | F45-20 | 5376 | NS3 | 0.0573 | A:24960;G:11;C:2;T:1518;total:26491 | iSNV |
| F45 | F45-20 | 5441 | NS3 | 0.0594 | A:2;G:1;C:32332;T:2045;total:34380 | iSNV |
| F45 | F45-20 | 5602 | NS3 | 0.0285 | A:7;G:1;C:28580;T:839;total:29427 | iSNV |
| F45 | F45-20 | 5952 | NS3 | 0.0977 | A:11;G:2;C:32947;T:3569;total:36529 | iSNV |
| F45 | F45-20 | 6059 | NS3 | 0.0446 | A:1;G:7;C:1349;T:28850;total:30207 | iSNV |
| F45 | F45-20 | 6867 | NS4A | 0.1082 | A:2634;G:21704;C:2;T:2;total:24342 | iSNV |
| F45 | F45-20 | 6938 | NS4A | 0.0264 | A:14;G:0;C:22939;T:623;total:23576 | iSNV |
| F45 | F45-20 | 7633 | NS4B | 0.9373 | A:0;G:3;C:1748;T:26124;total:27875 | iSNV |
| F45 | F45-20 | 7990 | NS5 | 0.0261 | A:29455;G:790;C:1;T:2;total:30248 | iSNV |
| F45 | F45-20 | 9359 | NS5 | 0.1031 | A:0;G:1;C:3781;T:32868;total:36650 | iSNV |
| F45 | F45-20 | 10109 | NS5 | 0.2256 | A:5789;G:19864;C:0;T:2;total:25655 | iSNV |
| F45 | F45-20 | 10259 | NS5 | 0.099 | A:30101;G:3308;C:1;T:0;total:33410 | iSNV |
| F45 | F45-20 | 10428 | 3'-UTR | 0.3917 | A:3;G:0;C:14968;T:9644;total:24615 | iSNV |
| F45 | F45-20 | 10447 | 3'-UTR | 0.0351 | A:15;G:2;C:21828;T:796;total:22641 | iSNV |
| F45 | F45-20 | 10566 | 3'-UTR | 0.0905 | A:2;G:0;C:18546;T:1847;total:20395 | iSNV |
| F45 | F45-21 | 443 | C | 0.1969 | A:16;G:43986;C:13;T:10794;total:54809 | iSNV |
| F45 | F45-21 | 1031 | E | 0.0234 | A:14;G:2;C:41346;T:992;total:42354 | iSNV |
| F45 | F45-21 | 1218 | E | 0.9977 | A:1;G:1;C:106;T:45330;total:45438 | SNP |
| F45 | F45-21 | 1363 | E | 0.0287 | A:4;G:0;C:51374;T:1521;total:52899 | iSNV |
| F45 | F45-21 | 1459 | E | 0.19 | A:10860;G:4;C:46246;T:30;total:57140 | iSNV |
| F45 | F45-21 | 1797 | E | 0.6917 | A:4019;G:36041;C:2;T:17863;total:57925 | iSNV |
| F45 | F45-21 | 2362 | E | 0.0828 | A:0;G:28788;C:2600;T:12;total:31400 | iSNV |
| F45 | F45-21 | 2393 | E | 0.1705 | A:2;G:1;C:28684;T:5897;total:34584 | iSNV |
| F45 | F45-21 | 2556 | NS1 | 0.6 | A:21281;G:14196;C:3;T:2;total:35482 | iSNV |
| F45 | F45-21 | 2876 | NS1 | 0.0443 | A:0;G:6;C:3251;T:70097;total:73354 | iSNV |
| F45 | F45-21 | 2960 | NS1 | 0.0695 | A:14;G:2;C:55021;T:4117;total:59154 | iSNV |
| F45 | F45-21 | 3176 | NS1 | 0.0663 | A:54863;G:3902;C:8;T:2;total:58775 | iSNV |
| F45 | F45-21 | 3230 | NS1 | 0.0528 | A:63192;G:3529;C:4;T:1;total:66726 | iSNV |
| F45 | F45-21 | 3869 | NS2A | 0.995 | A:2;G:1;C:252;T:50203;total:50458 | SNP |
| F45 | F45-21 | 5428 | NS3 | 0.0264 | A:56737;G:1540;C:3;T:1;total:58281 | iSNV |
| F45 | F45-21 | 6004 | NS3 | 0.1462 | A:11;G:2;C:48248;T:8266;total:56527 | iSNV |
| F45 | F45-21 | 6089 | NS3 | 0.1966 | A:16;G:2;C:38011;T:9308;total:47337 | iSNV |
| F45 | F45-21 | 6107 | NS3 | 0.281 | A:5;G:4;C:36419;T:14241;total:50669 | iSNV |
| F45 | F45-21 | 7561 | NS4B | 0.0491 | A:3;G:23;C:2230;T:43107;total:45363 | iSNV |
| F45 | F45-21 | 7633 | NS4B | 0.3663 | A:6;G:2;C:30675;T:17737;total:48420 | iSNV |
| F45 | F45-21 | 7644 | NS4B | 0.2301 | A:36800;G:6;C:11004;T:2;total:47812 | iSNV |
| F45 | F45-21 | 7732 | NS5 | 0.0222 | A:991;G:5;C:5;T:43546;total:44547 | iSNV |
| F45 | F45-21 | 8237 | NS5 | 0.0315 | A:13;G:1;C:52864;T:1724;total:54602 | iSNV |
| F45 | F45-21 | 8618 | NS5 | 0.0463 | A:2300;G:47314;C:3;T:17;total:49634 | iSNV |
| F45 | F45-21 | 8738 | NS5 | 0.0439 | A:41634;G:1913;C:4;T:0;total:43551 | iSNV |
| F45 | F45-21 | 8909 | NS5 | 0.0238 | A:3;G:6;C:1086;T:44472;total:45567 | iSNV |
| F45 | F45-21 | 9065 | NS5 | 0.1509 | A:6702;G:37686;C:4;T:6;total:44398 | iSNV |
| F45 | F45-21 | 10334 | NS5 | 0.1091 | A:4;G:1;C:50612;T:6199;total:56816 | iSNV |
| F45 | F45-21 | 10358 | NS5 | 0.5956 | A:5;G:1;C:20019;T:29474;total:49499 | iSNV |
| F45 | F45-21 | 10428 | 3'-UTR | 0.5586 | A:5;G:3;C:19441;T:24586;total:44035 | iSNV |
| F45 | F45-21 | 10447 | 3'-UTR | 0.0451 | A:8;G:1;C:39368;T:1862;total:41239 | iSNV |
| F45 | F45-21 | 10470 | 3'-UTR | 0.1972 | A:30452;G:7483;C:3;T:0;total:37938 | iSNV |
| F45 | F45-21 | 10513 | 3'-UTR | 0.2043 | A:5;G:4;C:8265;T:32173;total:40447 | iSNV |
| F45 | F45-21 | 10578 | 3'-UTR | 0.1503 | A:1;G:6;C:5455;T:30828;total:36290 | iSNV |
| F45 | F45-22 | 347 | C | 0.1843 | A:5;G:3;C:34763;T:7859;total:42630 | iSNV |
| F45 | F45-22 | 998 | E | 0.9603 | A:604;G:6;C:1326;T:31453;total:33389 | iSNV |
| F45 | F45-22 | 1193 | E | 0.0212 | A:2;G:904;C:5;T:41597;total:42508 | iSNV |
| F45 | F45-22 | 1218 | E | 0.9985 | A:0;G:0;C:58;T:36571;total:36629 | SNP |
| F45 | F45-22 | 1428 | E | 0.9075 | A:4164;G:40806;C:3;T:7;total:44980 | iSNV |
| F45 | F45-22 | 1797 | E | 0.051 | A:3;G:2352;C:6;T:43742;total:46103 | iSNV |
| F45 | F45-22 | 3869 | NS2A | 0.0421 | A:12;G:0;C:39595;T:1742;total:41349 | iSNV |
| F45 | F45-22 | 4468 | NS2B | 0.9095 | A:26429;G:4;C:3;T:2632;total:29068 | iSNV |
| F45 | F45-22 | 4697 | NS3 | 0.9582 | A:9;G:0;C:44279;T:1936;total:46224 | iSNV |
| F45 | F45-22 | 5366 | NS3 | 0.0523 | A:34290;G:5;C:1893;T:2;total:36190 | iSNV |
| F45 | F45-22 | 5952 | NS3 | 0.956 | A:1;G:4;C:2093;T:45446;total:47544 | iSNV |
| F45 | F45-22 | 6277 | NS3 | 0.0542 | A:33655;G:1929;C:2;T:1;total:35587 | iSNV |
| F45 | F45-22 | 6867 | NS4A | 0.1896 | A:6379;G:27249;C:3;T:2;total:33633 | iSNV |
| F45 | F45-22 | 7211 | NS4A | 0.1937 | A:5339;G:2;C:4;T:22218;total:27563 | iSNV |

| | | | | | | |
|---|---|---|---|---|---|---|
| F45 | F45-22 | 7264 | NS4A | 0.0272 | A:8;G:1;C:24591;T:688;total:25288 | iSNV |
| F45 | F45-22 | 7633 | NS4B | 0.8773 | A:1;G:3;C:4485;T:32062;total:36551 | iSNV |
| F45 | F45-22 | 7644 | NS4B | 0.0537 | A:35455;G:6;C:2013;T:0;total:37474 | iSNV |
| F45 | F45-22 | 7879 | NS5 | 0.0468 | A:41755;G:2053;C:6;T:2;total:43816 | iSNV |
| F45 | F45-22 | 9245 | NS5 | 0.903 | A:5;G:8;C:4742;T:44110;total:48865 | iSNV |
| F45 | F45-22 | 9359 | NS5 | 0.9527 | A:4;G:4;C:49670;T:2470;total:52148 | iSNV |
| F45 | F45-22 | 9634 | NS5 | 0.0206 | A:2;G:3;C:759;T:36062;total:36826 | iSNV |
| F45 | F45-22 | 9983 | NS5 | 0.0495 | A:6;G:5;C:29210;T:1523;total:30744 | iSNV |
| F45 | F45-22 | 10259 | NS5 | 0.9575 | A:1948;G:43802;C:0;T:9;total:45759 | iSNV |
| F45 | F45-22 | 10428 | 3'-UTR | 0.1411 | A:9;G:1;C:30854;T:5073;total:35937 | iSNV |
| F45 | F45-22 | 10447 | 3'-UTR | 0.0392 | A:5;G:3;C:31373;T:1282;total:32663 | iSNV |
| F45 | F45-22 | 10566 | 3'-UTR | 0.9552 | A:3;G:5;C:1136;T:24208;total:25352 | iSNV |
| F45 | F45-22 | 10784 | 3'-UTR | 0.0489 | A:25556;G:3;C:1315;T:5;total:26879 | iSNV |
| F45 | F45-23 | 353 | C | 0.7361 | A:11687;G:32576;C:3;T:3;total:44269 | iSNV |
| F45 | F45-23 | 454 | C | 0.0294 | A:0;G:2;C:1239;T:40760;total:42001 | iSNV |
| F45 | F45-23 | 645 | M | 0.7161 | A:9600;G:24201;C:6;T:0;total:33807 | iSNV |
| F45 | F45-23 | 803 | M | 0.0365 | A:5;G:29807;C:1;T:1130;total:30943 | iSNV |
| F45 | F45-23 | 998 | E | 0.7665 | A:0;G:5;C:6392;T:20973;total:27370 | iSNV |
| F45 | F45-23 | 1007 | E | 0.0213 | A:2;G:3;C:29093;T:634;total:29732 | iSNV |
| F45 | F45-23 | 1117 | E | 0.7742 | A:7092;G:24305;C:3;T:3;total:31403 | iSNV |
| F45 | F45-23 | 1218 | E | 0.2129 | A:9;G:1;C:27553;T:7459;total:35022 | iSNV |
| F45 | F45-23 | 1512 | E | 0.7442 | A:6701;G:19480;C:10;T:5;total:26196 | iSNV |
| F45 | F45-23 | 1709 | E | 0.0231 | A:603;G:25483;C:0;T:1;total:26087 | iSNV |
| F45 | F45-23 | 1797 | E | 0.0898 | A:0;G:3191;C:37;T:32273;total:35501 | iSNV |
| F45 | F45-23 | 2303 | E | 0.3814 | A:1;G:0;C:18412;T:11356;total:29769 | iSNV |
| F45 | F45-23 | 2808 | NS1 | 0.0283 | A:5;G:64;C:1327;T:45381;total:46777 | iSNV |
| F45 | F45-23 | 3257 | NS1 | 0.0247 | A:0;G:1;C:1159;T:45590;total:46750 | iSNV |
| F45 | F45-23 | 3869 | NS2A | 0.1977 | A:8;G:3;C:33559;T:8277;total:41847 | iSNV |
| F45 | F45-23 | 3974 | NS2A | 0.0257 | A:1;G:0;C:38077;T:1008;total:39086 | iSNV |
| F45 | F45-23 | 4697 | NS3 | 0.0265 | A:0;G:5;C:1072;T:39225;total:40302 | iSNV |
| F45 | F45-23 | 4974 | NS3 | 0.0217 | A:785;G:35296;C:2;T:2;total:36085 | iSNV |
| F45 | F45-23 | 5826 | NS3 | 0.1474 | A:42699;G:7386;C:2;T:1;total:50088 | iSNV |
| F45 | F45-23 | 5952 | NS3 | 0.0292 | A:4;G:2;C:48229;T:1455;total:49690 | iSNV |
| F45 | F45-23 | 6471 | NS4A | 0.0295 | A:936;G:30733;C:1;T:3;total:31673 | iSNV |
| F45 | F45-23 | 6530 | NS4A | 0.0229 | A:24003;G:563;C:3;T:0;total:24569 | iSNV |
| F45 | F45-23 | 6867 | NS4A | 0.4012 | A:11990;G:17893;C:1;T:1;total:29885 | iSNV |
| F45 | F45-23 | 6900 | NS4A | 0.1515 | A:26;G:24559;C:1;T:4392;total:28978 | iSNV |
| F45 | F45-23 | 7182 | NS4A | 0.0209 | A:434;G:0;C:20318;T:4;total:20756 | iSNV |
| F45 | F45-23 | 7633 | NS4B | 0.9473 | A:2;G:3;C:2332;T:41862;total:44199 | iSNV |
| F45 | F45-23 | 7735 | NS5 | 0.0314 | A:1270;G:39131;C:1;T:2;total:40404 | iSNV |
| F45 | F45-23 | 8518 | NS5 | 0.0312 | A:36638;G:6;C:3;T:1181;total:37828 | iSNV |
| F45 | F45-23 | 9359 | NS5 | 0.03 | A:1;G:5;C:1599;T:51540;total:53145 | iSNV |
| F45 | F45-23 | 9722 | NS5 | 0.0212 | A:1;G:0;C:21509;T:466;total:21976 | iSNV |
| F45 | F45-23 | 10259 | NS5 | 0.7652 | A:10223;G:33306;C:0;T:7;total:43536 | iSNV |
| F45 | F45-23 | 10419 | 3'-UTR | 0.0219 | A:5;G:0;C:32615;T:732;total:33352 | iSNV |
| F45 | F45-23 | 10428 | 3'-UTR | 0.3423 | A:5;G:2;C:20975;T:10924;total:31906 | iSNV |
| F45 | F45-23 | 10447 | 3'-UTR | 0.744 | A:6;G:0;C:6784;T:19700;total:26490 | iSNV |
| F45 | F45-23 | 10566 | 3'-UTR | 0.0291 | A:7;G:0;C:21717;T:653;total:22377 | iSNV |
| F45 | F45-23 | 10804 | 3'-UTR | 0.1264 | A:0;G:0;C:17911;T:2592;total:20503 | iSNV |
| F45 | F45-24 | 521 | M | 0.0837 | A:2;G:1;C:40499;T:3703;total:44205 | iSNV |
| F45 | F45-24 | 658 | M | 0.0641 | A:37711;G:2584;C:2;T:0;total:40297 | iSNV |
| F45 | F45-24 | 898 | M | 0.1188 | A:1;G:3;C:4218;T:31273;total:35495 | iSNV |
| F45 | F45-24 | 919 | M | 0.0872 | A:34052;G:3257;C:3;T:1;total:37313 | iSNV |
| F45 | F45-24 | 920 | M | 0.0616 | A:6;G:0;C:35095;T:2305;total:37406 | iSNV |
| F45 | F45-24 | 998 | E | 0.0752 | A:3;G:3;C:34097;T:2777;total:36880 | iSNV |
| F45 | F45-24 | 1013 | E | 0.0792 | A:34561;G:6;C:2975;T:3;total:37545 | iSNV |
| F45 | F45-24 | 1218 | E | 0.9972 | A:0;G:3;C:107;T:38775;total:38885 | SNP |
| F45 | F45-24 | 1413 | E | 0.075 | A:42584;G:3456;C:3;T:0;total:46043 | iSNV |
| F45 | F45-24 | 1892 | E | 0.0753 | A:38036;G:3101;C:2;T:3;total:41142 | iSNV |
| F45 | F45-24 | 2076 | E | 0.7486 | A:8821;G:26257;C:0;T:1;total:35079 | iSNV |
| F45 | F45-24 | 2369 | E | 0.0721 | A:2340;G:30094;C:0;T:5;total:32439 | iSNV |
| F45 | F45-24 | 2771 | NS1 | 0.0717 | A:4;G:0;C:45395;T:3511;total:48910 | iSNV |
| F45 | F45-24 | 2870 | NS1 | 0.1786 | A:14;G:1;C:43553;T:9474;total:53042 | iSNV |
| F45 | F45-24 | 2890 | NS1 | 0.7432 | A:38125;G:7;C:13178;T:3;total:51313 | iSNV |
| F45 | F45-24 | 3261 | NS1 | 0.182 | A:35995;G:8013;C:3;T:0;total:44011 | iSNV |
| F45 | F45-24 | 3284 | NS1 | 0.0203 | A:0;G:5;C:789;T:38016;total:38810 | iSNV |
| F45 | F45-24 | 3608 | NS1 | 0.027 | A:3;G:3;C:39892;T:1110;total:41008 | iSNV |
| F45 | F45-24 | 3669 | NS1 | 0.7517 | A:9;G:6;C:8493;T:25688;total:34196 | iSNV |
| F45 | F45-24 | 3869 | NS2A | 0.9193 | A:2;G:1;C:3103;T:35329;total:38435 | iSNV |
| F45 | F45-24 | 4070 | NS2A | 0.0801 | A:0;G:4;C:2759;T:31667;total:34430 | iSNV |
| F45 | F45-24 | 4286 | NS2B | 0.1971 | A:7049;G:28698;C:4;T:11;total:35762 | iSNV |
| F45 | F45-24 | 4697 | NS3 | 0.0712 | A:4;G:5;C:3444;T:44889;total:48342 | iSNV |
| F45 | F45-24 | 5172 | NS3 | 0.1888 | A:25585;G:3;C:3;T:5957;total:31548 | iSNV |
| F45 | F45-24 | 5195 | NS3 | 0.0661 | A:2;G:1;C:2362;T:33321;total:35686 | iSNV |
| F45 | F45-24 | 5465 | NS3 | 0.069 | A:45648;G:3385;C:7;T:3;total:49043 | iSNV |
| F45 | F45-24 | 5737 | NS3 | 0.7452 | A:11584;G:33859;C:1;T:5;total:45449 | iSNV |
| F45 | F45-24 | 5952 | NS3 | 0.0719 | A:8;G:0;C:43890;T:3401;total:47299 | iSNV |
| F45 | F45-24 | 6714 | NS4A | 0.0431 | A:1586;G:35197;C:3;T:5;total:36791 | iSNV |
| F45 | F45-24 | 6867 | NS4A | 0.095 | A:3587;G:34145;C:2;T:3;total:37737 | iSNV |
| F45 | F45-24 | 6971 | NS4A | 0.1194 | A:7;G:31926;C:4818;T:3598;total:40349 | iSNV |
| F45 | F45-24 | 7193 | NS4A | 0.1955 | A:6;G:6;C:6544;T:26917;total:33473 | iSNV |
| F45 | F45-24 | 7264 | NS4A | 0.0904 | A:4;G:0;C:29451;T:2928;total:32383 | iSNV |

| | | | | | | |
|---|---|---|---|---|---|---|
| F45 | F45-24 | 7633 | NS4B | 0.9874 | A:0;G:2;C:511;T:40055;total:40568 | SNP |
| F45 | F45-24 | 9183 | NS5 | 0.0742 | A:3480;G:43380;C:2;T:8;total:46870 | iSNV |
| F45 | F45-24 | 9233 | NS5 | 0.583 | A:4;G:3;C:20377;T:28474;total:48858 | iSNV |
| F45 | F45-24 | 9359 | NS5 | 0.072 | A:0;G:12;C:3419;T:44006;total:47437 | iSNV |
| F45 | F45-24 | 9902 | NS5 | 0.0588 | A:2245;G:35908;C:1;T:4;total:38158 | iSNV |
| F45 | F45-24 | 9922 | NS5 | 0.0709 | A:5;G:2;C:36353;T:2776;total:39136 | iSNV |
| F45 | F45-24 | 10253 | NS5 | 0.064 | A:44351;G:7;C:1;T:3038;total:47397 | iSNV |
| F45 | F45-24 | 10259 | NS5 | 0.0761 | A:44196;G:3644;C:2;T:1;total:47843 | iSNV |
| F45 | F45-24 | 10376 | NS5 | 0.0644 | A:2432;G:35304;C:1;T:3;total:37740 | iSNV |
| F45 | F45-24 | 10428 | 3'-UTR | 0.7168 | A:4;G:5;C:9292;T:23509;total:32810 | iSNV |
| F45 | F45-24 | 10447 | 3'-UTR | 0.0769 | A:7;G:2;C:28406;T:2368;total:30783 | iSNV |
| F45 | F45-24 | 10566 | 3'-UTR | 0.0671 | A:14;G:1;C:30631;T:2207;total:32853 | iSNV |
| F45 | F45-25 | 443 | C | 0.0323 | A:8;G:30045;C:0;T:1004;total:31057 | iSNV |
| F45 | F45-25 | 491 | M | 0.0218 | A:18;G:2;C:728;T:32622;total:33370 | iSNV |
| F45 | F45-25 | 815 | M | 0.0408 | A:0;G:4;C:985;T:23111;total:24100 | iSNV |
| F45 | F45-25 | 824 | M | 0.1566 | A:20259;G:3763;C:1;T:0;total:24023 | iSNV |
| F45 | F45-25 | 998 | E | 0.033 | A:2;G:0;C:22923;T:783;total:23708 | iSNV |
| F45 | F45-25 | 1083 | E | 0.0335 | A:21281;G:738;C:3;T:2;total:22024 | iSNV |
| F45 | F45-25 | 1117 | E | 0.1344 | A:22489;G:3493;C:0;T:1;total:25983 | iSNV |
| F45 | F45-25 | 1218 | E | 0.9987 | A:1;G:3;C:33;T:26557;total:26594 | SNP |
| F45 | F45-25 | 1428 | E | 0.3351 | A:20257;G:10212;C:2;T:1;total:30472 | iSNV |
| F45 | F45-25 | 1797 | E | 0.3416 | A:3;G:10756;C:8;T:20719;total:31486 | iSNV |
| F45 | F45-25 | 1804 | E | 0.0234 | A:763;G:31718;C:0;T:3;total:32484 | iSNV |
| F45 | F45-25 | 2369 | E | 0.0328 | A:590;G:17339;C:0;T:41;total:17970 | iSNV |
| F45 | F45-25 | 2828 | NS1 | 0.0282 | A:36304;G:1057;C:3;T:7;total:37371 | iSNV |
| F45 | F45-25 | 2855 | NS1 | 0.1375 | A:5623;G:35245;C:1;T:4;total:40873 | iSNV |
| F45 | F45-25 | 3356 | NS1 | 0.0351 | A:784;G:21503;C:2;T:1;total:22290 | iSNV |
| F45 | F45-25 | 3869 | NS2A | 0.9633 | A:1;G:4;C:1091;T:28599;total:29695 | iSNV |
| F45 | F45-25 | 3875 | NS2A | 0.0225 | A:7;G:0;C:30392;T:702;total:31101 | iSNV |
| F45 | F45-25 | 4469 | NS2B | 0.0343 | A:0;G:1;C:685;T:19251;total:19937 | iSNV |
| F45 | F45-25 | 4646 | NS3 | 0.2993 | A:5;G:2;C:25117;T:10732;total:35856 | iSNV |
| F45 | F45-25 | 4697 | NS3 | 0.0365 | A:3;G:9;C:1249;T:32951;total:34212 | iSNV |
| F45 | F45-25 | 5558 | NS3 | 0.1372 | A:4441;G:27907;C:1;T:0;total:32349 | iSNV |
| F45 | F45-25 | 5736 | NS3 | 0.0227 | A:843;G:1;C:36261;T:4;total:37109 | iSNV |
| F45 | F45-25 | 5952 | NS3 | 0.0311 | A:3;G:2;C:35486;T:1142;total:36633 | iSNV |
| F45 | F45-25 | 5968 | NS3 | 0.3395 | A:12256;G:23832;C:1;T:2;total:36091 | iSNV |
| F45 | F45-25 | 6431 | NS3 | 0.0314 | A:5;G:0;C:25082;T:815;total:25902 | iSNV |
| F45 | F45-25 | 6867 | NS4A | 0.0225 | A:547;G:23735;C:0;T:2;total:24284 | iSNV |
| F45 | F45-25 | 6900 | NS4A | 0.0217 | A:525;G:23559;C:6;T:4;total:24094 | iSNV |
| F45 | F45-25 | 6970 | NS4A | 0.0489 | A:23528;G:23;C:1212;T:1;total:24764 | iSNV |
| F45 | F45-25 | 7034 | NS4A | 0.0203 | A:1;G:0;C:24297;T:506;total:24804 | iSNV |
| F45 | F45-25 | 7561 | NS4B | 0.0323 | A:1;G:12;C:882;T:26384;total:27279 | iSNV |
| F45 | F45-25 | 7633 | NS4B | 0.5235 | A:3;G:1;C:14772;T:16223;total:30999 | iSNV |
| F45 | F45-25 | 8675 | NS5 | 0.0241 | A:2;G:5;C:506;T:20398;total:20911 | iSNV |
| F45 | F45-25 | 9080 | NS5 | 0.0419 | A:24804;G:1086;C:1;T:2;total:25893 | iSNV |
| F45 | F45-25 | 9359 | NS5 | 0.0364 | A:0;G:3;C:1341;T:35447;total:36791 | iSNV |
| F45 | F45-25 | 9818 | NS5 | 0.0239 | A:0;G:0;C:581;T:23641;total:24222 | iSNV |
| F45 | F45-25 | 10259 | NS5 | 0.0349 | A:31689;G:1149;C:2;T:0;total:32840 | iSNV |
| F45 | F45-25 | 10428 | 3'-UTR | 0.9249 | A:2;G:6;C:1710;T:21026;total:22744 | iSNV |
| F45 | F45-25 | 10566 | 3'-UTR | 0.034 | A:5;G:2;C:18442;T:650;total:19099 | iSNV |
| F45 | F45-25 | 10632 | 3'-UTR | 0.023 | A:13;G:8;C:499;T:21114;total:21634 | iSNV |
| F45 | F45-26 | 168 | C | 0.0528 | A:0;G:2;C:1980;T:35478;total:37460 | iSNV |
| F45 | F45-26 | 470 | C | 0.0556 | A:28732;G:24;C:2;T:1695;total:30453 | iSNV |
| F45 | F45-26 | 694 | M | 0.4158 | A:16640;G:11846;C:1;T:1;total:28488 | iSNV |
| F45 | F45-26 | 1218 | E | 0.9988 | A:1;G:1;C:29;T:25589;total:25620 | SNP |
| F45 | F45-26 | 1263 | E | 0.057 | A:0;G:6;C:1488;T:24607;total:26101 | iSNV |
| F45 | F45-26 | 1428 | E | 0.8645 | A:4048;G:25810;C:3;T:1;total:29862 | iSNV |
| F45 | F45-26 | 1514 | E | 0.0583 | A:25629;G:4;C:1589;T:1;total:27223 | iSNV |
| F45 | F45-26 | 1797 | E | 0.0645 | A:227;G:2021;C:20;T:29063;total:31331 | iSNV |
| F45 | F45-26 | 3093 | NS1 | 0.0228 | A:2;G:1;C:654;T:27992;total:28649 | iSNV |
| F45 | F45-26 | 3536 | NS1 | 0.2068 | A:3;G:1;C:18734;T:4886;total:23624 | iSNV |
| F45 | F45-26 | 3869 | NS2A | 0.9979 | A:0;G:2;C:55;T:26884;total:26941 | SNP |
| F45 | F45-26 | 4922 | NS3 | 0.0534 | A:4;G:1;C:30888;T:1743;total:32636 | iSNV |
| F45 | F45-26 | 5616 | NS3 | 0.0339 | A:1064;G:2;C:30282;T:8;total:31356 | iSNV |
| F45 | F45-26 | 6080 | NS3 | 0.0672 | A:1885;G:26121;C:0;T:3;total:28009 | iSNV |
| F45 | F45-26 | 6423 | NS3 | 0.0345 | A:883;G:6;C:24634;T:5;total:25528 | iSNV |
| F45 | F45-26 | 6714 | NS4A | 0.2795 | A:7244;G:18657;C:5;T:3;total:25909 | iSNV |
| F45 | F45-26 | 6770 | NS4A | 0.0303 | A:6;G:2;C:26439;T:829;total:27276 | iSNV |
| F45 | F45-26 | 6806 | NS4A | 0.0546 | A:3;G:28979;C:3;T:1677;total:30662 | iSNV |
| F45 | F45-26 | 6947 | NS4A | 0.2174 | A:3;G:4;C:5941;T:21376;total:27324 | iSNV |
| F45 | F45-26 | 6970 | NS4A | 0.0355 | A:26888;G:1;C:992;T:0;total:27881 | iSNV |
| F45 | F45-26 | 7244 | NS4A | 0.3964 | A:3;G:0;C:8525;T:12973;total:21501 | iSNV |
| F45 | F45-26 | 7561 | NS4B | 0.1076 | A:0;G:15;C:2859;T:23673;total:26547 | iSNV |
| F45 | F45-26 | 7633 | NS4B | 0.8577 | A:1;G:1;C:4098;T:24691;total:28791 | iSNV |
| F45 | F45-26 | 7982 | NS5 | 0.058 | A:30681;G:226;C:6;T:1905;total:32818 | iSNV |
| F45 | F45-26 | 8666 | NS5 | 0.0531 | A:1412;G:25173;C:1;T:3;total:26589 | iSNV |
| F45 | F45-26 | 8840 | NS5 | 0.0217 | A:23794;G:529;C:2;T:0;total:24325 | iSNV |
| F45 | F45-26 | 9443 | NS5 | 0.082 | A:33524;G:2998;C:2;T:4;total:36528 | iSNV |
| F45 | F45-26 | 9899 | NS5 | 0.1118 | A:3;G:0;C:23312;T:2937;total:26252 | iSNV |
| F45 | F45-26 | 10373 | NS5 | 0.0288 | A:8;G:0;C:26284;T:780;total:27072 | iSNV |
| F45 | F45-26 | 10428 | 3'-UTR | 0.962 | A:2;G:0;C:848;T:21465;total:22315 | iSNV |
| F45 | F45-27 | 662 | M | 0.1272 | A:25552;G:3726;C:2;T:1;total:29281 | iSNV |

| | | | | | | |
|---|---|---|---|---|---|---|
| F45 | F45-27 | 716 | M | 0.1343 | A:5;G:28497;C:8;T:4425;total:32935 | iSNV |
| F45 | F45-27 | 906 | M | 0.0864 | A:21839;G:2068;C:4;T:2;total:23913 | iSNV |
| F45 | F45-27 | 1046 | E | 0.0203 | A:538;G:25909;C:0;T:3;total:26450 | iSNV |
| F45 | F45-27 | 1083 | E | 0.0325 | A:22184;G:746;C:2;T:1;total:22933 | iSNV |
| F45 | F45-27 | 1109 | E | 0.0333 | A:3;G:0;C:25135;T:866;total:26004 | iSNV |
| F45 | F45-27 | 1218 | E | 0.9976 | A:2;G:2;C:62;T:26599;total:26665 | SNP |
| F45 | F45-27 | 1428 | E | 0.0346 | A:30538;G:1096;C:3;T:0;total:31637 | iSNV |
| F45 | F45-27 | 1512 | E | 0.3111 | A:16682;G:8386;C:1879;T:1;total:26948 | iSNV |
| F45 | F45-27 | 1797 | E | 0.2544 | A:3;G:8265;C:993;T:23222;total:32483 | iSNV |
| F45 | F45-27 | 2274 | E | 0.2512 | A:2;G:18280;C:6;T:6136;total:24424 | iSNV |
| F45 | F45-27 | 2531 | NS1 | 0.0689 | A:1631;G:2;C:28;T:22004;total:23665 | iSNV |
| F45 | F45-27 | 2664 | NS1 | 0.6177 | A:2;G:4;C:12135;T:19598;total:31739 | iSNV |
| F45 | F45-27 | 3089 | NS1 | 0.0307 | A:1;G:14;C:899;T:28314;total:29228 | iSNV |
| F45 | F45-27 | 3181 | NS1 | 0.0231 | A:33637;G:798;C:2;T:0;total:34437 | iSNV |
| F45 | F45-27 | 3869 | NS2A | 0.9919 | A:2;G:3;C:222;T:27626;total:27853 | SNP |
| F45 | F45-27 | 4974 | NS3 | 0.0714 | A:2187;G:28413;C:0;T:5;total:30605 | iSNV |
| F45 | F45-27 | 5617 | NS3 | 0.0309 | A:29601;G:947;C:1;T:0;total:30549 | iSNV |
| F45 | F45-27 | 6063 | NS3 | 0.0785 | A:6;G:0;C:29513;T:2515;total:32034 | iSNV |
| F45 | F45-27 | 6182 | NS3 | 0.0841 | A:2;G:4;C:2099;T:22843;total:24948 | iSNV |
| F45 | F45-27 | 6714 | NS4A | 0.0371 | A:997;G:25819;C:1;T:3;total:26820 | iSNV |
| F45 | F45-27 | 6867 | NS4A | 0.0386 | A:991;G:24662;C:0;T:1;total:25654 | iSNV |
| F45 | F45-27 | 6957 | NS4A | 0.0759 | A:24237;G:1993;C:3;T:0;total:26233 | iSNV |
| F45 | F45-27 | 7626 | NS4B | 0.652 | A:16;G:20160;C:7;T:10777;total:30960 | iSNV |
| F45 | F45-27 | 7633 | NS4B | 0.1361 | A:6;G:2;C:26995;T:4255;total:31258 | iSNV |
| F45 | F45-27 | 7697 | NS5 | 0.0772 | A:5;G:26948;C:2;T:2257;total:29212 | iSNV |
| F45 | F45-27 | 7835 | NS5 | 0.0221 | A:1;G:3;C:575;T:25403;total:25982 | iSNV |
| F45 | F45-27 | 8756 | NS5 | 0.0404 | A:22571;G:952;C:0;T:1;total:23524 | iSNV |
| F45 | F45-27 | 9728 | NS5 | 0.0255 | A:7;G:1;C:24588;T:646;total:25242 | iSNV |
| F45 | F45-27 | 9830 | NS5 | 0.0886 | A:3;G:2;C:2234;T:22951;total:25190 | iSNV |
| F45 | F45-27 | 10373 | NS5 | 0.0271 | A:13;G:1;C:27679;T:774;total:28467 | iSNV |
| F45 | F45-27 | 10428 | 3'-UTR | 0.4297 | A:2;G:3;C:13744;T:10360;total:24109 | iSNV |
| F45 | F45-27 | 10447 | 3'-UTR | 0.0332 | A:26;G:1;C:20952;T:722;total:21701 | iSNV |
| F45 | F45-27 | 10904 | 3'-UTR | 0.0214 | A:0;G:0;C:3741;T:82;total:3823 | iSNV |
| F45 | F45-28 | 230 | C | 0.8801 | A:5;G:3;C:34889;T:4757;total:39654 | iSNV |
| F45 | F45-28 | 353 | C | 0.0808 | A:33719;G:2968;C:2;T:1;total:36690 | iSNV |
| F45 | F45-28 | 645 | M | 0.0747 | A:30801;G:2488;C:1;T:2;total:33292 | iSNV |
| F45 | F45-28 | 734 | M | 0.0373 | A:1440;G:2;C:5;T:37142;total:38589 | iSNV |
| F45 | F45-28 | 828 | M | 0.042 | A:27056;G:1187;C:3;T:5;total:28251 | iSNV |
| F45 | F45-28 | 998 | E | 0.0801 | A:7;G:0;C:24869;T:2167;total:27043 | iSNV |
| F45 | F45-28 | 1044 | E | 0.2719 | A:4;G:7;C:7893;T:21121;total:29025 | iSNV |
| F45 | F45-28 | 1087 | E | 0.0799 | A:23821;G:2070;C:4;T:1;total:25896 | iSNV |
| F45 | F45-28 | 1117 | E | 0.0827 | A:28078;G:2534;C:0;T:2;total:30614 | iSNV |
| F45 | F45-28 | 1218 | E | 0.9069 | A:2;G:2;C:2947;T:28679;total:31630 | iSNV |
| F45 | F45-28 | 1428 | E | 0.9118 | A:3157;G:32618;C:3;T:2;total:35780 | iSNV |
| F45 | F45-28 | 1472 | E | 0.0224 | A:30476;G:700;C:4;T:0;total:31180 | iSNV |
| F45 | F45-28 | 1512 | E | 0.0765 | A:28936;G:2399;C:3;T:1;total:31339 | iSNV |
| F45 | F45-28 | 1595 | E | 0.1149 | A:2909;G:22388;C:2;T:4;total:25303 | iSNV |
| F45 | F45-28 | 1910 | E | 0.0411 | A:1173;G:27332;C:0;T:2;total:28507 | iSNV |
| F45 | F45-28 | 3099 | NS1 | 0.0641 | A:2481;G:36203;C:1;T:4;total:38689 | iSNV |
| F45 | F45-28 | 3380 | NS1 | 0.0208 | A:1;G:0;C:584;T:27450;total:28035 | iSNV |
| F45 | F45-28 | 3719 | NS1 | 0.0279 | A:711;G:2;C:18;T:24682;total:25413 | iSNV |
| F45 | F45-28 | 3869 | NS2A | 0.9171 | A:3;G:5;C:2921;T:32276;total:35205 | iSNV |
| F45 | F45-28 | 4348 | NS2B | 0.0557 | A:1;G:9;C:1872;T:31680;total:33562 | iSNV |
| F45 | F45-28 | 4361 | NS2B | 0.0221 | A:32334;G:731;C:3;T:0;total:33068 | iSNV |
| F45 | F45-28 | 4447 | NS2B | 0.0204 | A:24784;G:517;C:1;T:0;total:25302 | iSNV |
| F45 | F45-28 | 4632 | NS3 | 0.0409 | A:2;G:1814;C:6;T:42426;total:44248 | iSNV |
| F45 | F45-28 | 5043 | NS3 | 0.0506 | A:32185;G:1716;C:1;T:1;total:33903 | iSNV |
| F45 | F45-28 | 5353 | NS3 | 0.0219 | A:35033;G:785;C:2;T:3;total:35823 | iSNV |
| F45 | F45-28 | 5654 | NS3 | 0.0745 | A:5;G:1;C:35757;T:2879;total:38642 | iSNV |
| F45 | F45-28 | 5705 | NS3 | 0.0757 | A:38625;G:3166;C:1;T:1;total:41793 | iSNV |
| F45 | F45-28 | 6714 | NS4A | 0.1199 | A:3824;G:28050;C:1;T:2;total:31877 | iSNV |
| F45 | F45-28 | 6729 | NS4A | 0.0749 | A:2411;G:2;C:29738;T:4;total:32155 | iSNV |
| F45 | F45-28 | 6730 | NS4A | 0.0757 | A:0;G:2;C:2429;T:29621;total:32052 | iSNV |
| F45 | F45-28 | 6737 | NS4A | 0.0493 | A:3;G:0;C:30212;T:1570;total:31785 | iSNV |
| F45 | F45-28 | 6867 | NS4A | 0.1125 | A:3428;G:27031;C:1;T:1;total:30461 | iSNV |
| F45 | F45-28 | 6900 | NS4A | 0.0372 | A:1115;G:28805;C:23;T:18;total:29961 | iSNV |
| F45 | F45-28 | 6970 | NS4A | 0.2437 | A:23651;G:68;C:7646;T:3;total:31368 | iSNV |
| F45 | F45-28 | 7104 | NS4A | 0.1994 | A:5945;G:23861;C:2;T:2;total:29810 | iSNV |
| F45 | F45-28 | 7264 | NS4A | 0.0304 | A:6;G:0;C:23708;T:746;total:24460 | iSNV |
| F45 | F45-28 | 7451 | NS4B | 0.0628 | A:3;G:19097;C:1281;T:1;total:20382 | iSNV |
| F45 | F45-28 | 7626 | NS4B | 0.0223 | A:834;G:13;C:26;T:36511;total:37384 | iSNV |
| F45 | F45-28 | 7633 | NS4B | 0.8886 | A:3;G:1;C:4201;T:33495;total:37700 | iSNV |
| F45 | F45-28 | 7656 | NS4B | 0.0222 | A:861;G:37858;C:1;T:6;total:38726 | iSNV |
| F45 | F45-28 | 7973 | NS5 | 0.0228 | A:39582;G:7;C:4;T:924;total:40517 | iSNV |
| F45 | F45-28 | 8909 | NS5 | 0.024 | A:29;G:2;C:765;T:31018;total:31814 | iSNV |
| F45 | F45-28 | 10259 | NS5 | 0.0779 | A:36791;G:3109;C:0;T:1;total:39901 | iSNV |
| F45 | F45-28 | 10335 | NS5 | 0.0669 | A:2;G:2;C:2609;T:36330;total:38943 | iSNV |
| F45 | F45-28 | 10428 | 3'-UTR | 0.3518 | A:5;G:5;C:18735;T:10176;total:28921 | iSNV |
| F45 | F45-28 | 10447 | 3'-UTR | 0.0882 | A:8;G:2;C:23688;T:2293;total:25991 | iSNV |
| F45 | F45-28 | 10451 | 3'-UTR | 0.0431 | A:4;G:3;C:24614;T:1109;total:25730 | iSNV |
| F45 | F45-28 | 10452 | 3'-UTR | 0.0235 | A:24965;G:601;C:0;T:2;total:25568 | iSNV |
| F45 | F45-29 | 332 | C | 0.076 | A:53522;G:4403;C:5;T:2;total:57932 | iSNV |

| | | | | | | |
|---|---|---|---|---|---|---|
| F45 | F45-29 | 1218 | E | 0.9984 | A:5;G:6;C:72;T:50549;total:50632 | SNP |
| F45 | F45-29 | 1296 | E | 0.0528 | A:2;G:5;C:3077;T:55166;total:58250 | iSNV |
| F45 | F45-29 | 1447 | E | 0.8051 | A:48565;G:6;C:11764;T:3;total:60338 | iSNV |
| F45 | F45-29 | 1914 | E | 0.0302 | A:48339;G:1508;C:1;T:2;total:49850 | iSNV |
| F45 | F45-29 | 2362 | E | 0.9818 | A:9;G:646;C:35995;T:14;total:36664 | SNP |
| F45 | F45-29 | 3438 | NS1 | 0.0229 | A:6;G:0;C:49941;T:1175;total:51122 | iSNV |
| F45 | F45-29 | 3645 | NS1 | 0.0264 | A:1;G:9;C:1343;T:49415;total:50768 | iSNV |
| F45 | F45-29 | 3869 | NS2A | 0.9984 | A:0;G:4;C:80;T:52101;total:52185 | SNP |
| F45 | F45-29 | 3959 | NS2A | 0.7916 | A:1;G:4;C:11648;T:44237;total:55890 | iSNV |
| F45 | F45-29 | 4829 | NS3 | 0.0296 | A:60068;G:1838;C:5;T:2;total:61913 | iSNV |
| F45 | F45-29 | 4943 | NS3 | 0.0304 | A:55779;G:1751;C:2;T:0;total:57532 | iSNV |
| F45 | F45-29 | 5702 | NS3 | 0.0577 | A:18;G:1;C:64094;T:3929;total:68042 | iSNV |
| F45 | F45-29 | 6714 | NS4A | 0.1032 | A:5360;G:46539;C:4;T:3;total:51906 | iSNV |
| F45 | F45-29 | 6715 | NS4A | 0.0683 | A:14;G:4;C:48611;T:3565;total:52194 | iSNV |
| F45 | F45-29 | 6867 | NS4A | 0.3541 | A:18462;G:33659;C:2;T:3;total:52126 | iSNV |
| F45 | F45-29 | 6900 | NS4A | 0.03 | A:1521;G:49041;C:6;T:2;total:50570 | iSNV |
| F45 | F45-29 | 7481 | NS4B | 0.0758 | A:41;G:1;C:2728;T:33193;total:35963 | iSNV |
| F45 | F45-29 | 7627 | NS4B | 0.0269 | A:34;G:1522;C:54864;T:12;total:56432 | iSNV |
| F45 | F45-29 | 7633 | NS4B | 0.9641 | A:1;G:10;C:2007;T:53865;total:55883 | iSNV |
| F45 | F45-29 | 8844 | NS5 | 0.0352 | A:1641;G:44926;C:1;T:2;total:46570 | iSNV |
| F45 | F45-29 | 8966 | NS5 | 0.0295 | A:19;G:4;C:51168;T:1561;total:52752 | iSNV |
| F45 | F45-29 | 9659 | NS5 | 0.0335 | A:23;G:4;C:52141;T:1809;total:53977 | iSNV |
| F45 | F45-29 | 10428 | 3'-UTR | 0.9337 | A:1;G:6;C:2871;T:40399;total:43277 | iSNV |
| F45 | F45-29 | 10447 | 3'-UTR | 0.0212 | A:8;G:4;C:40755;T:885;total:41652 | iSNV |
| F45 | F45-29 | 10513 | 3'-UTR | 0.0217 | A:1;G:6;C:934;T:41986;total:42927 | iSNV |
| F45 | F45-3 | 374 | C | 0.0397 | A:1;G:1;C:19007;T:786;total:19795 | iSNV |
| F45 | F45-3 | 828 | M | 0.407 | A:7326;G:5031;C:1;T:3;total:12361 | iSNV |
| F45 | F45-3 | 996 | E | 0.4261 | A:4867;G:6552;C:0;T:1;total:11420 | iSNV |
| F45 | F45-3 | 1218 | E | 0.9964 | A:0;G:5;C:45;T:13632;total:13682 | SNP |
| F45 | F45-3 | 1255 | E | 0.1504 | A:12449;G:2205;C:1;T:2;total:14657 | iSNV |
| F45 | F45-3 | 1263 | E | 0.0619 | A:0;G:3;C:910;T:13766;total:14679 | iSNV |
| F45 | F45-3 | 1463 | E | 0.0332 | A:1;G:2;C:11846;T:408;total:12257 | iSNV |
| F45 | F45-3 | 1508 | E | 0.047 | A:7;G:0;C:11754;T:581;total:12342 | iSNV |
| F45 | F45-3 | 1797 | E | 0.4659 | A:3;G:7328;C:0;T:8395;total:15726 | iSNV |
| F45 | F45-3 | 1804 | E | 0.0591 | A:954;G:15180;C:0;T:0;total:16134 | iSNV |
| F45 | F45-3 | 2144 | E | 0.0374 | A:0;G:450;C:11566;T:4;total:12020 | iSNV |
| F45 | F45-3 | 2282 | E | 0.0253 | A:0;G:0;C:11826;T:307;total:12133 | iSNV |
| F45 | F45-3 | 2465 | E | 0.4537 | A:2;G:1;C:4973;T:4134;total:9110 | iSNV |
| F45 | F45-3 | 2780 | NS1 | 0.0639 | A:1;G:7;C:1315;T:19233;total:20556 | iSNV |
| F45 | F45-3 | 3452 | NS1 | 0.0569 | A:8578;G:518;C:0;T:0;total:9096 | iSNV |
| F45 | F45-3 | 3858 | NS2A | 0.4261 | A:3;G:0;C:9079;T:6744;total:15826 | iSNV |
| F45 | F45-3 | 3869 | NS2A | 0.9959 | A:0;G:1;C:68;T:16370;total:16439 | SNP |
| F45 | F45-3 | 4289 | NS2B | 0.0882 | A:1130;G:1;C:38;T:11642;total:12811 | iSNV |
| F45 | F45-3 | 4292 | NS2B | 0.426 | A:5491;G:1;C:2;T:7393;total:12887 | iSNV |
| F45 | F45-3 | 5306 | NS3 | 0.3893 | A:9041;G:5767;C:2;T:1;total:14811 | iSNV |
| F45 | F45-3 | 5616 | NS3 | 0.0207 | A:341;G:0;C:16067;T:11;total:16419 | iSNV |
| F45 | F45-3 | 5736 | NS3 | 0.1665 | A:3399;G:5;C:16999;T:2;total:20405 | iSNV |
| F45 | F45-3 | 6967 | NS4A | 0.062 | A:785;G:11863;C:0;T:4;total:12652 | iSNV |
| F45 | F45-3 | 6971 | NS4A | 0.0529 | A:0;G:11952;C:668;T:0;total:12620 | iSNV |
| F45 | F45-3 | 6980 | NS4A | 0.2994 | A:8779;G:5;C:6;T:3758;total:12548 | iSNV |
| F45 | F45-3 | 7264 | NS4A | 0.0241 | A:1;G:0;C:8705;T:215;total:8921 | iSNV |
| F45 | F45-3 | 7585 | NS4B | 0.0719 | A:1;G:12716;C:986;T:2;total:13705 | iSNV |
| F45 | F45-3 | 7595 | NS4B | 0.0737 | A:2;G:1065;C:1;T:13375;total:14443 | iSNV |
| F45 | F45-3 | 7633 | NS4B | 0.4963 | A:0;G:0;C:8260;T:8139;total:16399 | iSNV |
| F45 | F45-3 | 7768 | NS5 | 0.0819 | A:1;G:1;C:12911;T:1153;total:14066 | iSNV |
| F45 | F45-3 | 8363 | NS5 | 0.0214 | A:2;G:19646;C:2;T:431;total:20081 | iSNV |
| F45 | F45-3 | 9370 | NS5 | 0.0226 | A:478;G:3;C:10;T:20602;total:21093 | iSNV |
| F45 | F45-3 | 10428 | 3'-UTR | 0.7918 | A:3;G:3;C:2409;T:9153;total:11568 | iSNV |
| F45 | F45-3 | 10567 | 3'-UTR | 0.0205 | A:0;G:4;C:202;T:9630;total:9836 | iSNV |
| F45 | F45-30 | 719 | M | 0.0797 | A:4;G:3;C:43237;T:3749;total:46993 | iSNV |
| F45 | F45-30 | 1218 | E | 0.9964 | A:0;G:2;C:145;T:39901;total:40048 | SNP |
| F45 | F45-30 | 1413 | E | 0.9902 | A:452;G:46937;C:10;T:3;total:47402 | SNP |
| F45 | F45-30 | 1428 | E | 0.9894 | A:477;G:44855;C:3;T:4;total:45339 | SNP |
| F45 | F45-30 | 3869 | NS2A | 0.995 | A:0;G:4;C:195;T:39351;total:39550 | SNP |
| F45 | F45-30 | 3965 | NS2A | 0.0317 | A:7;G:0;C:41165;T:1349;total:42521 | iSNV |
| F45 | F45-30 | 4005 | NS2A | 0.9888 | A:0;G:3;C:425;T:37496;total:37924 | SNP |
| F45 | F45-30 | 6336 | NS3 | 0.0207 | A:31220;G:6;C:7;T:662;total:31895 | iSNV |
| F45 | F45-30 | 6714 | NS4A | 0.108 | A:4260;G:35159;C:2;T:4;total:39425 | iSNV |
| F45 | F45-30 | 6867 | NS4A | 0.1307 | A:5290;G:35166;C:2;T:4;total:40462 | iSNV |
| F45 | F45-30 | 7264 | NS4A | 0.4021 | A:4;G:3;C:19979;T:13445;total:33431 | iSNV |
| F45 | F45-30 | 7633 | NS4B | 0.9904 | A:2;G:4;C:410;T:42719;total:43135 | SNP |
| F45 | F45-30 | 9293 | NS5 | 0.0279 | A:1296;G:45090;C:1;T:6;total:46393 | iSNV |
| F45 | F45-30 | 10376 | NS5 | 0.9882 | A:36537;G:434;C:2;T:2;total:36975 | SNP |
| F45 | F45-30 | 10428 | 3'-UTR | 0.9403 | A:1;G:4;C:1954;T:30768;total:32727 | iSNV |
| F45 | F45-4 | 457 | C | 0.1291 | A:0;G:0;C:192;T:1295;total:1487 | iSNV |
| F45 | F45-4 | 476 | C | 0.0312 | A:0;G:0;C:1579;T:51;total:1630 | iSNV |
| F45 | F45-4 | 998 | E | 0.2091 | A:0;G:0;C:726;T:192;total:918 | iSNV |
| F45 | F45-4 | 1218 | E | 0.9858 | A:0;G:0;C:16;T:1105;total:1121 | SNP |
| F45 | F45-4 | 1263 | E | 0.1327 | A:0;G:0;C:131;T:856;total:987 | iSNV |
| F45 | F45-4 | 1388 | E | 0.0202 | A:0;G:0;C:1017;T:21;total:1038 | iSNV |
| F45 | F45-4 | 1496 | E | 0.0287 | A:0;G:0;C:27;T:911;total:938 | iSNV |
| F45 | F45-4 | 1512 | E | 0.0967 | A:877;G:94;C:1;T:0;total:972 | iSNV |

| | | | | | | |
|---|---|---|---|---|---|---|
| F45 | F45-4 | 1513 | E | 0.1147 | A:864;G:0;C:112;T:0;total:976 | iSNV |
| F45 | F45-4 | 1551 | E | 0.0241 | A:0;G:21;C:846;T:2;total:869 | iSNV |
| F45 | F45-4 | 1797 | E | 0.0278 | A:36;G:8;C:4;T:1244;total:1292 | iSNV |
| F45 | F45-4 | 2111 | E | 0.0278 | A:0;G:0;C:1013;T:29;total:1042 | iSNV |
| F45 | F45-4 | 2282 | E | 0.023 | A:0;G:0;C:552;T:13;total:565 | iSNV |
| F45 | F45-4 | 2367 | E | 0.4912 | A:320;G:309;C:0;T:0;total:629 | iSNV |
| F45 | F45-4 | 2834 | NS1 | 0.0246 | A:1426;G:36;C:0;T:0;total:1462 | iSNV |
| F45 | F45-4 | 2852 | NS1 | 0.0247 | A:0;G:0;C:1538;T:39;total:1577 | iSNV |
| F45 | F45-4 | 3224 | NS1 | 0.045 | A:1209;G:57;C:0;T:0;total:1266 | iSNV |
| F45 | F45-4 | 3260 | NS1 | 0.0333 | A:32;G:927;C:0;T:0;total:959 | iSNV |
| F45 | F45-4 | 3336 | NS1 | 0.0566 | A:833;G:50;C:0;T:0;total:883 | iSNV |
| F45 | F45-4 | 3422 | NS1 | 0.0248 | A:0;G:0;C:24;T:942;total:966 | iSNV |
| F45 | F45-4 | 3896 | NS2A | 0.0732 | A:219;G:2770;C:0;T:1;total:2990 | iSNV |
| F45 | F45-4 | 4073 | NS2A | 0.0201 | A:0;G:0;C:2280;T:47;total:2327 | iSNV |
| F45 | F45-4 | 4280 | NS2B | 0.0205 | A:0;G:0;C:764;T:16;total:780 | iSNV |
| F45 | F45-4 | 4697 | NS3 | 0.1641 | A:0;G:0;C:427;T:2175;total:2602 | iSNV |
| F45 | F45-4 | 4974 | NS3 | 0.0528 | A:39;G:699;C:0;T:0;total:738 | iSNV |
| F45 | F45-4 | 5256 | NS3 | 0.0299 | A:0;G:0;C:1167;T:36;total:1203 | iSNV |
| F45 | F45-4 | 5736 | NS3 | 0.0649 | A:65;G:0;C:936;T:0;total:1001 | iSNV |
| F45 | F45-4 | 5921 | NS3 | 0.068 | A:466;G:34;C:0;T:0;total:500 | iSNV |
| F45 | F45-4 | 5952 | NS3 | 0.125 | A:0;G:0;C:476;T:68;total:544 | iSNV |
| F45 | F45-4 | 6322 | NS3 | 0.0523 | A:0;G:0;C:2063;T:114;total:2177 | iSNV |
| F45 | F45-4 | 6471 | NS4A | 0.0235 | A:33;G:1368;C:0;T:0;total:1401 | iSNV |
| F45 | F45-4 | 6797 | NS4A | 0.0222 | A:0;G:0;C:4576;T:104;total:4680 | iSNV |
| F45 | F45-4 | 6900 | NS4A | 0.1955 | A:333;G:1369;C:1;T:0;total:1703 | iSNV |
| F45 | F45-4 | 6969 | NS4A | 0.222 | A:1489;G:0;C:425;T:0;total:1914 | iSNV |
| F45 | F45-4 | 7264 | NS4A | 0.0416 | A:0;G:0;C:2025;T:88;total:2113 | iSNV |
| F45 | F45-4 | 7561 | NS4B | 0.0566 | A:0;G:0;C:39;T:650;total:689 | iSNV |
| F45 | F45-4 | 7595 | NS4B | 0.0245 | A:7;G:11;C:5;T:425;total:448 | iSNV |
| F45 | F45-4 | 7633 | NS4B | 0.7156 | A:0;G:0;C:132;T:332;total:464 | iSNV |
| F45 | F45-4 | 7724 | NS5 | 0.0245 | A:318;G:8;C:0;T:0;total:326 | iSNV |
| F45 | F45-4 | 8243 | NS5 | 0.025 | A:0;G:0;C:1712;T:44;total:1756 | iSNV |
| F45 | F45-4 | 8639 | NS5 | 0.2222 | A:0;G:0;C:347;T:1214;total:1561 | iSNV |
| F45 | F45-4 | 9341 | NS5 | 0.0344 | A:924;G:33;C:0;T:0;total:957 | iSNV |
| F45 | F45-4 | 9359 | NS5 | 0.1797 | A:0;G:0;C:172;T:785;total:957 | iSNV |
| F45 | F45-4 | 9434 | NS5 | 0.0357 | A:836;G:31;C:0;T:0;total:867 | iSNV |
| F45 | F45-4 | 10259 | NS5 | 0.1914 | A:1318;G:312;C:0;T:0;total:1630 | iSNV |
| F45 | F45-4 | 10428 | 3'-UTR | 0.5022 | A:0;G:0;C:1032;T:1041;total:2073 | iSNV |
| F45 | F45-4 | 10447 | 3'-UTR | 0.02 | A:0;G:0;C:2148;T:44;total:2192 | iSNV |
| F45 | F45-4 | 10566 | 3'-UTR | 0.1763 | A:0;G:0;C:1910;T:409;total:2319 | iSNV |
| F45 | F45-4 | 10705 | 3'-UTR | 0.0213 | A:0;G:1;C:1740;T:38;total:1779 | iSNV |
| F45 | F45-5 | 353 | C | 0.0436 | A:36043;G:1646;C:2;T:2;total:37693 | iSNV |
| F45 | F45-5 | 386 | C | 0.0666 | A:2730;G:38227;C:1;T:6;total:40964 | iSNV |
| F45 | F45-5 | 399 | C | 0.0789 | A:3414;G:7;C:39829;T:13;total:43263 | iSNV |
| F45 | F45-5 | 645 | M | 0.041 | A:31025;G:1328;C:1;T:0;total:32354 | iSNV |
| F45 | F45-5 | 897 | M | 0.0891 | A:1;G:2;C:25559;T:2502;total:28064 | iSNV |
| F45 | F45-5 | 998 | E | 0.047 | A:6;G:3;C:28794;T:1421;total:30224 | iSNV |
| F45 | F45-5 | 1117 | E | 0.046 | A:30386;G:1467;C:0;T:2;total:31855 | iSNV |
| F45 | F45-5 | 1194 | E | 0.0229 | A:855;G:2;C:36341;T:11;total:37209 | iSNV |
| F45 | F45-5 | 1218 | E | 0.9519 | A:1;G:4;C:1579;T:31234;total:32818 | iSNV |
| F45 | F45-5 | 2076 | E | 0.2457 | A:20562;G:6700;C:4;T:0;total:27266 | iSNV |
| F45 | F45-5 | 2126 | E | 0.0884 | A:4;G:1;C:25845;T:2508;total:28358 | iSNV |
| F45 | F45-5 | 2202 | E | 0.0284 | A:34166;G:9;C:0;T:1000;total:35175 | iSNV |
| F45 | F45-5 | 2362 | E | 0.0724 | A:0;G:19698;C:0;T:1539;total:21237 | iSNV |
| F45 | F45-5 | 2393 | E | 0.025 | A:4;G:3;C:23096;T:593;total:23696 | iSNV |
| F45 | F45-5 | 3869 | NS2A | 0.9527 | A:0;G:4;C:1808;T:36407;total:38219 | iSNV |
| F45 | F45-5 | 4974 | NS3 | 0.0678 | A:2539;G:34872;C:1;T:1;total:37413 | iSNV |
| F45 | F45-5 | 5187 | NS3 | 0.022 | A:3;G:5;C:658;T:29108;total:29774 | iSNV |
| F45 | F45-5 | 6061 | NS3 | 0.0356 | A:36435;G:1348;C:2;T:2;total:37787 | iSNV |
| F45 | F45-5 | 6206 | NS3 | 0.0405 | A:0;G:3;C:30656;T:1297;total:31956 | iSNV |
| F45 | F45-5 | 6335 | NS3 | 0.0705 | A:2;G:1;C:24077;T:1828;total:25908 | iSNV |
| F45 | F45-5 | 6336 | NS3 | 0.3982 | A:14058;G:0;C:34;T:9328;total:23420 | iSNV |
| F45 | F45-5 | 6389 | NS3 | 0.0299 | A:949;G:30751;C:5;T:1;total:31706 | iSNV |
| F45 | F45-5 | 6714 | NS4A | 0.0336 | A:1005;G:28825;C:3;T:5;total:29838 | iSNV |
| F45 | F45-5 | 6753 | NS4A | 0.09 | A:11;G:23940;C:0;T:2371;total:26322 | iSNV |
| F45 | F45-5 | 6867 | NS4A | 0.0793 | A:2235;G:25918;C:1;T:0;total:28154 | iSNV |
| F45 | F45-5 | 6900 | NS4A | 0.0248 | A:703;G:27614;C:4;T:18;total:28339 | iSNV |
| F45 | F45-5 | 6971 | NS4A | 0.0254 | A:3;G:30313;C:791;T:24;total:31131 | iSNV |
| F45 | F45-5 | 7060 | NS4A | 0.0364 | A:2;G:6;C:1174;T:31033;total:32215 | iSNV |
| F45 | F45-5 | 7633 | NS4B | 0.9212 | A:2;G:7;C:2936;T:34277;total:37222 | iSNV |
| F45 | F45-5 | 7748 | NS5 | 0.0322 | A:1;G:5;C:1148;T:34423;total:35577 | iSNV |
| F45 | F45-5 | 8062 | NS5 | 0.0736 | A:2781;G:34969;C:1;T:2;total:37753 | iSNV |
| F45 | F45-5 | 8126 | NS5 | 0.0281 | A:2;G:2;C:42188;T:1223;total:43415 | iSNV |
| F45 | F45-5 | 8306 | NS5 | 0.0235 | A:8;G:6;C:38976;T:942;total:39932 | iSNV |
| F45 | F45-5 | 9308 | NS5 | 0.0237 | A:0;G:11;C:886;T:36476;total:37373 | iSNV |
| F45 | F45-5 | 9341 | NS5 | 0.4089 | A:25879;G:17908;C:4;T:3;total:43794 | iSNV |
| F45 | F45-5 | 9830 | NS5 | 0.0274 | A:0;G:4;C:778;T:27555;total:28337 | iSNV |
| F45 | F45-5 | 10259 | NS5 | 0.0446 | A:37384;G:1748;C:1;T:0;total:39133 | iSNV |
| F45 | F45-5 | 10419 | 3'-UTR | 0.0492 | A:0;G:2;C:26851;T:1392;total:28245 | iSNV |
| F45 | F45-5 | 10428 | 3'-UTR | 0.6366 | A:3;G:5;C:10009;T:17519;total:27536 | iSNV |
| F45 | F45-5 | 10447 | 3'-UTR | 0.0632 | A:3;G:3;C:23870;T:1611;total:25487 | iSNV |
| F45 | F45-6 | 353 | C | 0.8927 | A:2046;G:17019;C:1;T:0;total:19066 | iSNV |

| | | | | | | |
|---|---|---|---|---|---|---|
| F45 | F45-6 | 645 | M | 0.883 | A:1824;G:13750;C:0;T:5;total:15579 | iSNV |
| F45 | F45-6 | 998 | E | 0.8911 | A:0;G:0;C:1484;T:12137;total:13621 | iSNV |
| F45 | F45-6 | 1117 | E | 0.8955 | A:1533;G:13128;C:1;T:3;total:14665 | iSNV |
| F45 | F45-6 | 1218 | E | 0.096 | A:0;G:1;C:14893;T:1583;total:16477 | iSNV |
| F45 | F45-6 | 1261 | E | 0.053 | A:860;G:15361;C:3;T:0;total:16224 | iSNV |
| F45 | F45-6 | 1428 | E | 0.0727 | A:15453;G:1212;C:0;T:1;total:16666 | iSNV |
| F45 | F45-6 | 1511 | E | 0.0205 | A:0;G:2;C:13438;T:282;total:13722 | iSNV |
| F45 | F45-6 | 1512 | E | 0.0907 | A:12136;G:1239;C:279;T:0;total:13654 | iSNV |
| F45 | F45-6 | 1513 | E | 0.0477 | A:13076;G:5;C:656;T:0;total:13737 | iSNV |
| F45 | F45-6 | 1708 | E | 0.0767 | A:1104;G:13270;C:0;T:2;total:14376 | iSNV |
| F45 | F45-6 | 1797 | E | 0.0202 | A:1;G:358;C:6;T:17286;total:17651 | iSNV |
| F45 | F45-6 | 2051 | E | 0.0705 | A:3;G:0;C:10807;T:820;total:11630 | iSNV |
| F45 | F45-6 | 3869 | NS2A | 0.1092 | A:2;G:1;C:16142;T:1981;total:18126 | iSNV |
| F45 | F45-6 | 4098 | NS2A | 0.0681 | A:10714;G:784;C:0;T:1;total:11499 | iSNV |
| F45 | F45-6 | 4590 | NS2B | 0.1185 | A:2239;G:3;C:35;T:16609;total:18886 | iSNV |
| F45 | F45-6 | 4974 | NS3 | 0.8895 | A:14895;G:1851;C:2;T:0;total:16748 | iSNV |
| F45 | F45-6 | 5365 | NS3 | 0.1092 | A:12737;G:1563;C:0;T:4;total:14304 | iSNV |
| F45 | F45-6 | 6005 | NS3 | 0.2158 | A:1;G:1;C:14363;T:3954;total:18319 | iSNV |
| F45 | F45-6 | 6061 | NS3 | 0.8917 | A:1852;G:15243;C:0;T:2;total:17097 | iSNV |
| F45 | F45-6 | 6715 | NS4A | 0.0217 | A:3;G:0;C:14080;T:313;total:14396 | iSNV |
| F45 | F45-6 | 6867 | NS4A | 0.186 | A:2513;G:10992;C:2;T:1;total:13508 | iSNV |
| F45 | F45-6 | 7060 | NS4A | 0.8903 | A:0;G:0;C:12056;T:1487;total:13543 | iSNV |
| F45 | F45-6 | 7633 | NS4B | 0.9076 | A:0;G:3;C:1685;T:16541;total:18229 | iSNV |
| F45 | F45-6 | 8318 | NS5 | 0.1037 | A:0;G:3;C:17515;T:2028;total:19546 | iSNV |
| F45 | F45-6 | 9437 | NS5 | 0.056 | A:20938;G:1244;C:4;T:0;total:22186 | iSNV |
| F45 | F45-6 | 9491 | NS5 | 0.0851 | A:14026;G:1305;C:0;T:2;total:15333 | iSNV |
| F45 | F45-6 | 10133 | NS5 | 0.0226 | A:0;G:1;C:14013;T:325;total:14339 | iSNV |
| F45 | F45-6 | 10165 | NS5 | 0.1605 | A:1;G:3;C:2132;T:11147;total:13283 | iSNV |
| F45 | F45-6 | 10259 | NS5 | 0.892 | A:2030;G:16755;C:0;T:0;total:18785 | iSNV |
| F45 | F45-6 | 10419 | 3'-UTR | 0.9041 | A:2;G:5;C:1359;T:12798;total:14164 | iSNV |
| F45 | F45-6 | 10428 | 3'-UTR | 0.5926 | A:2;G:1;C:5563;T:8086;total:13652 | iSNV |
| F45 | F45-6 | 10447 | 3'-UTR | 0.8988 | A:0;G:0;C:1194;T:10595;total:11789 | iSNV |
| F45 | F45-7 | 418 | C | 0.0232 | A:26439;G:630;C:5;T:2;total:27076 | iSNV |
| F45 | F45-7 | 909 | M | 0.2958 | A:0;G:10;C:16393;T:6891;total:23294 | iSNV |
| F45 | F45-7 | 998 | E | 0.0669 | A:3;G:2;C:21728;T:1560;total:23293 | iSNV |
| F45 | F45-7 | 1130 | E | 0.0582 | A:1;G:2;C:23940;T:1480;total:25423 | iSNV |
| F45 | F45-7 | 1218 | E | 0.9992 | A:0;G:4;C:16;T:24733;total:24753 | SNP |
| F45 | F45-7 | 1296 | E | 0.0574 | A:1;G:5;C:1676;T:27496;total:29178 | iSNV |
| F45 | F45-7 | 1416 | E | 0.3204 | A:19813;G:9346;C:3;T:3;total:29165 | iSNV |
| F45 | F45-7 | 1428 | E | 0.0287 | A:27893;G:827;C:2;T:0;total:28722 | iSNV |
| F45 | F45-7 | 1512 | E | 0.3136 | A:17014;G:7778;C:3;T:0;total:24795 | iSNV |
| F45 | F45-7 | 1621 | E | 0.0289 | A:630;G:21127;C:1;T:3;total:21761 | iSNV |
| F45 | F45-7 | 1797 | E | 0.0603 | A:5;G:1818;C:8;T:28279;total:30110 | iSNV |
| F45 | F45-7 | 1889 | E | 0.021 | A:0;G:3;C:522;T:24293;total:24818 | iSNV |
| F45 | F45-7 | 2067 | E | 0.332 | A:0;G:3;C:6984;T:14047;total:21034 | iSNV |
| F45 | F45-7 | 2275 | E | 0.2043 | A:2;G:5;C:4476;T:17423;total:21906 | iSNV |
| F45 | F45-7 | 2774 | NS1 | 0.0246 | A:32728;G:826;C:2;T:3;total:33559 | iSNV |
| F45 | F45-7 | 2951 | NS1 | 0.069 | A:1;G:3;C:28074;T:2081;total:30159 | iSNV |
| F45 | F45-7 | 3257 | NS1 | 0.3155 | A:1;G:4;C:9729;T:21098;total:30832 | iSNV |
| F45 | F45-7 | 3317 | NS1 | 0.5654 | A:1;G:4;C:12286;T:15977;total:28268 | iSNV |
| F45 | F45-7 | 3854 | NS2A | 0.0284 | A:759;G:25943;C:0;T:4;total:26706 | iSNV |
| F45 | F45-7 | 3869 | NS2A | 0.9285 | A:4;G:3;C:1921;T:24903;total:26831 | iSNV |
| F45 | F45-7 | 4122 | NS2A | 0.0362 | A:3;G:15243;C:2;T:573;total:15821 | iSNV |
| F45 | F45-7 | 4403 | NS2B | 0.0249 | A:0;G:6;C:554;T:21670;total:22230 | iSNV |
| F45 | F45-7 | 4447 | NS2B | 0.0211 | A:19722;G:426;C:5;T:2;total:20155 | iSNV |
| F45 | F45-7 | 4697 | NS3 | 0.068 | A:2;G:3;C:2159;T:29577;total:31741 | iSNV |
| F45 | F45-7 | 4720 | NS3 | 0.0491 | A:0;G:9;C:1521;T:29426;total:30956 | iSNV |
| F45 | F45-7 | 4909 | NS3 | 0.0583 | A:27755;G:13;C:2;T:1721;total:29491 | iSNV |
| F45 | F45-7 | 5402 | NS3 | 0.0225 | A:1;G:627;C:3;T:27127;total:27758 | iSNV |
| F45 | F45-7 | 5665 | NS3 | 0.0703 | A:5;G:3;C:27539;T:2084;total:29631 | iSNV |
| F45 | F45-7 | 5777 | NS3 | 0.0276 | A:15;G:5;C:32745;T:933;total:33698 | iSNV |
| F45 | F45-7 | 5952 | NS3 | 0.0647 | A:3;G:2;C:30854;T:2135;total:32994 | iSNV |
| F45 | F45-7 | 6038 | NS3 | 0.0254 | A:762;G:29210;C:0;T:4;total:29976 | iSNV |
| F45 | F45-7 | 6233 | NS3 | 0.335 | A:0;G:1;C:16143;T:8134;total:24278 | iSNV |
| F45 | F45-7 | 6322 | NS3 | 0.567 | A:1;G:0;C:9888;T:12942;total:22831 | iSNV |
| F45 | F45-7 | 6714 | NS4A | 0.0724 | A:1710;G:21679;C:0;T:207;total:23596 | iSNV |
| F45 | F45-7 | 6758 | NS4A | 0.0506 | A:0;G:20407;C:4;T:1089;total:21500 | iSNV |
| F45 | F45-7 | 6867 | NS4A | 0.0726 | A:1686;G:21513;C:3;T:2;total:23204 | iSNV |
| F45 | F45-7 | 6900 | NS4A | 0.0622 | A:1441;G:21693;C:3;T:1;total:23138 | iSNV |
| F45 | F45-7 | 7028 | NS4A | 0.0246 | A:1;G:1;C:24627;T:622;total:25251 | iSNV |
| F45 | F45-7 | 7633 | NS4B | 0.9314 | A:2;G:4;C:1867;T:25340;total:27213 | iSNV |
| F45 | F45-7 | 7773 | NS5 | 0.0231 | A:22566;G:535;C:1;T:1;total:23103 | iSNV |
| F45 | F45-7 | 8534 | NS5 | 0.0619 | A:1;G:3;C:1964;T:29746;total:31714 | iSNV |
| F45 | F45-7 | 8588 | NS5 | 0.0296 | A:1;G:4;C:884;T:28889;total:29778 | iSNV |
| F45 | F45-7 | 8921 | NS5 | 0.0626 | A:0;G:5;C:23253;T:1555;total:24813 | iSNV |
| F45 | F45-7 | 9050 | NS5 | 0.0513 | A:23422;G:1268;C:1;T:2;total:24693 | iSNV |
| F45 | F45-7 | 9359 | NS5 | 0.0686 | A:8;G:3;C:2243;T:30410;total:32664 | iSNV |
| F45 | F45-7 | 9370 | NS5 | 0.3198 | A:10676;G:8;C:10;T:22687;total:33381 | iSNV |
| F45 | F45-7 | 9389 | NS5 | 0.0348 | A:2;G:4;C:1135;T:31422;total:32563 | iSNV |
| F45 | F45-7 | 9592 | NS5 | 0.1955 | A:21334;G:5186;C:2;T:2;total:26524 | iSNV |
| F45 | F45-7 | 9593 | NS5 | 0.0622 | A:4;G:4;C:1621;T:24432;total:26061 | iSNV |
| F45 | F45-7 | 9634 | NS5 | 0.0572 | A:0;G:4;C:1524;T:25070;total:26598 | iSNV |

| | | | | | | |
|---|---|---|---|---|---|---|
| F45 | F45-7 | 9690 | NS5 | 0.6116 | A:14456;G:9183;C:0;T:0;total:23639 | iSNV |
| F45 | F45-7 | 9821 | NS5 | 0.058 | A:3;G:4;C:1346;T:21815;total:23168 | iSNV |
| F45 | F45-7 | 10259 | NS5 | 0.0683 | A:26684;G:1957;C:1;T:1;total:28643 | iSNV |
| F45 | F45-7 | 10335 | NS5 | 0.029 | A:1;G:5;C:788;T:26346;total:27140 | iSNV |
| F45 | F45-7 | 10428 | 3'-UTR | 0.9138 | A:1;G:4;C:1774;T:18784;total:20563 | iSNV |
| F45 | F45-7 | 10451 | 3'-UTR | 0.0717 | A:2;G:5;C:18537;T:1433;total:19977 | iSNV |
| F45 | F45-7 | 10547 | 3'-UTR | 0.1664 | A:17337;G:3463;C:1;T:1;total:20802 | iSNV |
| F45 | F45-7 | 10566 | 3'-UTR | 0.0653 | A:1;G:0;C:18029;T:1261;total:19291 | iSNV |
| F45 | F45-7 | 10568 | 3'-UTR | 0.31 | A:2;G:4;C:13236;T:5950;total:19192 | iSNV |
| F45 | F45-7 | 10647 | 3'-UTR | 0.1396 | A:0;G:3;C:3261;T:20092;total:23356 | iSNV |
| F45 | F45-8 | 869 | M | 0.1022 | A:3674;G:6;C:8;T:32226;total:35914 | iSNV |
| F45 | F45-8 | 939 | M | 0.1022 | A:1;G:3;C:3204;T:28129;total:31337 | iSNV |
| F45 | F45-8 | 1218 | E | 0.9983 | A:2;G:9;C:49;T:33605;total:33665 | SNP |
| F45 | F45-8 | 1428 | E | 0.4023 | A:24213;G:16304;C:2;T:2;total:40521 | iSNV |
| F45 | F45-8 | 1430 | E | 0.1034 | A:1;G:3;C:36711;T:4235;total:40950 | iSNV |
| F45 | F45-8 | 1453 | E | 0.3349 | A:65;G:5;C:13945;T:27617;total:41632 | iSNV |
| F45 | F45-8 | 1512 | E | 0.2871 | A:23832;G:9626;C:66;T:3;total:33527 | iSNV |
| F45 | F45-8 | 1514 | E | 0.0203 | A:33001;G:7;C:687;T:1;total:33696 | iSNV |
| F45 | F45-8 | 1797 | E | 0.0666 | A:2;G:2750;C:21;T:38510;total:41283 | iSNV |
| F45 | F45-8 | 2054 | E | 0.0402 | A:1;G:1;C:28161;T:1181;total:29344 | iSNV |
| F45 | F45-8 | 3131 | NS1 | 0.0711 | A:37191;G:2850;C:2;T:1;total:40044 | iSNV |
| F45 | F45-8 | 3508 | NS1 | 0.0265 | A:4;G:2;C:24164;T:660;total:24830 | iSNV |
| F45 | F45-8 | 3869 | NS2A | 0.9966 | A:1;G:5;C:118;T:36300;total:36424 | SNP |
| F45 | F45-8 | 4187 | NS2A | 0.4041 | A:10998;G:7460;C:0;T:2;total:18460 | iSNV |
| F45 | F45-8 | 4896 | NS3 | 0.0307 | A:1419;G:44643;C:8;T:4;total:46074 | iSNV |
| F45 | F45-8 | 5353 | NS3 | 0.1518 | A:29598;G:5299;C:2;T:5;total:34904 | iSNV |
| F45 | F45-8 | 5456 | NS3 | 0.1264 | A:3;G:3;C:37982;T:5499;total:43487 | iSNV |
| F45 | F45-8 | 5665 | NS3 | 0.2993 | A:4;G:6;C:29292;T:12521;total:41823 | iSNV |
| F45 | F45-8 | 5864 | NS3 | 0.0391 | A:1706;G:12;C:9;T:41794;total:43521 | iSNV |
| F45 | F45-8 | 6151 | NS3 | 0.021 | A:26642;G:573;C:3;T:0;total:27218 | iSNV |
| F45 | F45-8 | 6277 | NS3 | 0.0434 | A:31491;G:1429;C:0;T:1;total:32921 | iSNV |
| F45 | F45-8 | 6314 | NS3 | 0.3047 | A:1;G:5;C:10039;T:22892;total:32937 | iSNV |
| F45 | F45-8 | 6867 | NS4A | 0.3291 | A:10736;G:21873;C:6;T:4;total:32619 | iSNV |
| F45 | F45-8 | 7334 | NS4B | 0.2314 | A:18175;G:5476;C:2;T:5;total:23658 | iSNV |
| F45 | F45-8 | 7367 | NS4B | 0.0954 | A:1;G:2;C:24213;T:2554;total:26770 | iSNV |
| F45 | F45-8 | 7633 | NS4B | 0.91 | A:4;G:12;C:3453;T:34880;total:38349 | iSNV |
| F45 | F45-8 | 8357 | NS5 | 0.1112 | A:6;G:5;C:41910;T:5245;total:47166 | iSNV |
| F45 | F45-8 | 9818 | NS5 | 0.1551 | A:0;G:2;C:4953;T:26970;total:31925 | iSNV |
| F45 | F45-8 | 10253 | NS5 | 0.0672 | A:37757;G:2723;C:0;T:3;total:40483 | iSNV |
| F45 | F45-8 | 10428 | 3'-UTR | 0.5486 | A:3;G:9;C:13677;T:16607;total:30296 | iSNV |
| F45 | F45-8 | 10447 | 3'-UTR | 0.3203 | A:2;G:3;C:18547;T:8744;total:27296 | iSNV |
| F45 | F45-8 | 10904 | 3'-UTR | 0.0204 | A:0;G:0;C:4601;T:96;total:4697 | iSNV |
| F45 | F45-9 | 925 | M | 0.04 | A:3;G:22566;C:941;T:1;total:23511 | iSNV |
| F45 | F45-9 | 951 | M | 0.0207 | A:521;G:2;C:24583;T:15;total:25121 | iSNV |
| F45 | F45-9 | 1196 | E | 0.0215 | A:630;G:28635;C:4;T:4;total:29273 | iSNV |
| F45 | F45-9 | 1218 | E | 0.998 | A:3;G:1;C:50;T:26394;total:26448 | SNP |
| F45 | F45-9 | 1413 | E | 0.05 | A:30148;G:1589;C:2;T:1;total:31740 | iSNV |
| F45 | F45-9 | 1512 | E | 0.7954 | A:4953;G:19247;C:3;T:1;total:24204 | iSNV |
| F45 | F45-9 | 2852 | NS1 | 0.7942 | A:1;G:6;C:8047;T:31030;total:39084 | iSNV |
| F45 | F45-9 | 3284 | NS1 | 0.0397 | A:1;G:4;C:1126;T:27169;total:28300 | iSNV |
| F45 | F45-9 | 3590 | NS1 | 0.0368 | A:3;G:1;C:21539;T:825;total:22368 | iSNV |
| F45 | F45-9 | 3869 | NS2A | 0.9985 | A:0;G:1;C:48;T:30604;total:30653 | SNP |
| F45 | F45-9 | 4886 | NS3 | 0.0552 | A:475;G:3;C:1906;T:32109;total:34493 | iSNV |
| F45 | F45-9 | 4974 | NS3 | 0.8727 | A:26097;G:3808;C:7;T:1;total:29913 | iSNV |
| F45 | F45-9 | 5921 | NS3 | 0.8776 | A:4015;G:28766;C:2;T:0;total:32783 | iSNV |
| F45 | F45-9 | 6004 | NS3 | 0.1525 | A:4;G:1;C:27325;T:4920;total:32250 | iSNV |
| F45 | F45-9 | 6428 | NS3 | 0.0752 | A:1;G:3;C:23968;T:1950;total:25922 | iSNV |
| F45 | F45-9 | 6714 | NS4A | 0.2518 | A:6286;G:18666;C:3;T:1;total:24956 | iSNV |
| F45 | F45-9 | 6867 | NS4A | 0.0225 | A:552;G:23930;C:0;T:1;total:24483 | iSNV |
| F45 | F45-9 | 6900 | NS4A | 0.0591 | A:1446;G:23012;C:1;T:0;total:24459 | iSNV |
| F45 | F45-9 | 7561 | NS4B | 0.1362 | A:2;G:6;C:3759;T:23814;total:27581 | iSNV |
| F45 | F45-9 | 7595 | NS4B | 0.1194 | A:3241;G:12;C:12;T:23874;total:27139 | iSNV |
| F45 | F45-9 | 7633 | NS4B | 0.2603 | A:5;G:2;C:22816;T:8034;total:30857 | iSNV |
| F45 | F45-9 | 7644 | NS4B | 0.1051 | A:27513;G:7;C:3234;T:1;total:30755 | iSNV |
| F45 | F45-9 | 7991 | NS5 | 0.0436 | A:28914;G:1321;C:5;T:0;total:30240 | iSNV |
| F45 | F45-9 | 8397 | NS5 | 0.0591 | A:2243;G:3;C:35650;T:6;total:37902 | iSNV |
| F45 | F45-9 | 9254 | NS5 | 0.0899 | A:35016;G:4;C:3461;T:1;total:38482 | iSNV |
| F45 | F45-9 | 9981 | NS5 | 0.1355 | A:2939;G:1;C:18748;T:2;total:21690 | iSNV |
| F45 | F45-9 | 10355 | NS5 | 0.0207 | A:26213;G:555;C:2;T:0;total:26770 | iSNV |
| F45 | F45-9 | 10376 | NS5 | 0.0489 | A:1292;G:25124;C:3;T:2;total:26421 | iSNV |
| F45 | F45-9 | 10428 | 3'-UTR | 0.3526 | A:4;G:1;C:14706;T:8013;total:22724 | iSNV |
| F45 | F45-9 | 10447 | 3'-UTR | 0.0235 | A:5;G:6;C:20665;T:498;total:21174 | iSNV |
| F50 | F50-1 | 446 | C | 0.0523 | A:3;G:0;C:7854;T:434;total:8291 | iSNV |
| F50 | F50-1 | 563 | M | 0.9822 | A:5621;G:102;C:0;T:0;total:5723 | SNP |
| F50 | F50-1 | 1257 | E | 0.9828 | A:5920;G:1;C:103;T:0;total:6024 | SNP |
| F50 | F50-1 | 1450 | E | 0.0219 | A:6672;G:2;C:0;T:150;total:6824 | iSNV |
| F50 | F50-1 | 1574 | E | 0.0239 | A:4278;G:0;C:105;T:0;total:4383 | iSNV |
| F50 | F50-1 | 1772 | E | 0.9847 | A:1;G:5903;C:0;T:91;total:5995 | SNP |
| F50 | F50-1 | 2060 | E | 0.0224 | A:0;G:0;C:4057;T:93;total:4150 | iSNV |
| F50 | F50-1 | 2076 | E | 0.2786 | A:2982;G:1152;C:0;T:0;total:4134 | iSNV |
| F50 | F50-1 | 2369 | E | 0.0697 | A:271;G:3616;C:0;T:1;total:3888 | iSNV |
| F50 | F50-1 | 2372 | E | 0.9889 | A:0;G:0;C:43;T:3828;total:3871 | SNP |

| | | | | | | |
|---|---|---|---|---|---|---|
| F50 | F50-1 | 2493 | NS1 | 0.985 | A:65;G:4261;C:0;T:0;total:4326 | SNP |
| F50 | F50-1 | 2498 | NS1 | 0.0573 | A:0;G:0;C:4090;T:249;total:4339 | iSNV |
| F50 | F50-1 | 2504 | NS1 | 0.9843 | A:65;G:4071;C:0;T:0;total:4136 | SNP |
| F50 | F50-1 | 2531 | NS1 | 0.9818 | A:3;G:0;C:4518;T:81;total:4602 | SNP |
| F50 | F50-1 | 3572 | NS1 | 0.9803 | A:0;G:0;C:87;T:4307;total:4394 | SNP |
| F50 | F50-1 | 3602 | NS1 | 0.0626 | A:0;G:274;C:4100;T:2;total:4376 | iSNV |
| F50 | F50-1 | 3908 | NS2A | 0.0989 | A:0;G:0;C:6665;T:732;total:7397 | iSNV |
| F50 | F50-1 | 3962 | NS2A | 0.9833 | A:0;G:0;C:112;T:6559;total:6671 | SNP |
| F50 | F50-1 | 4155 | NS2A | 0.9842 | A:2672;G:0;C:0;T:43;total:2715 | SNP |
| F50 | F50-1 | 4712 | NS3 | 0.9836 | A:7474;G:125;C:0;T:0;total:7599 | SNP |
| F50 | F50-1 | 5311 | NS3 | 0.9831 | A:1;G:0;C:86;T:5049;total:5136 | SNP |
| F50 | F50-1 | 5790 | NS3 | 0.0703 | A:1;G:0;C:581;T:7673;total:8255 | iSNV |
| F50 | F50-1 | 5835 | NS3 | 0.1083 | A:901;G:7413;C:0;T:0;total:8314 | iSNV |
| F50 | F50-1 | 6080 | NS3 | 0.9826 | A:5960;G:105;C:0;T:1;total:6066 | SNP |
| F50 | F50-1 | 6425 | NS3 | 0.0238 | A:134;G:5492;C:0;T:0;total:5626 | iSNV |
| F50 | F50-1 | 8130 | NS5 | 0.043 | A:1;G:0;C:7783;T:350;total:8134 | iSNV |
| F50 | F50-1 | 8282 | NS5 | 0.9883 | A:0;G:0;C:6985;T:83;total:7068 | SNP |
| F50 | F50-1 | 8900 | NS5 | 0.9851 | A:87;G:4;C:6124;T:2;total:6217 | SNP |
| F50 | F50-1 | 9008 | NS5 | 0.0393 | A:0;G:0;C:250;T:6102;total:6352 | iSNV |
| F50 | F50-1 | 9446 | NS5 | 0.984 | A:153;G:9415;C:0;T:1;total:9569 | SNP |
| F50 | F50-1 | 10014 | NS5 | 0.0409 | A:5571;G:0;C:2;T:238;total:5811 | iSNV |
| F50 | F50-1 | 10428 | 3'-UTR | 0.0549 | A:2;G:0;C:5965;T:347;total:6314 | iSNV |
| F50 | F50-10 | 518 | M | 0.0247 | A:12;G:0;C:17908;T:455;total:18375 | iSNV |
| F50 | F50-10 | 563 | M | 0.9974 | A:12245;G:26;C:5;T:1;total:12277 | SNP |
| F50 | F50-10 | 1116 | E | 0.3109 | A:5504;G:2484;C:0;T:0;total:7988 | iSNV |
| F50 | F50-10 | 1257 | E | 0.9963 | A:10387;G:0;C:39;T:0;total:10426 | SNP |
| F50 | F50-10 | 1772 | E | 0.9975 | A:0;G:8713;C:0;T:22;total:8735 | SNP |
| F50 | F50-10 | 1799 | E | 0.1418 | A:0;G:0;C:7952;T:1314;total:9266 | iSNV |
| F50 | F50-10 | 2067 | E | 0.0328 | A:0;G:0;C:149;T:4385;total:4534 | iSNV |
| F50 | F50-10 | 2147 | E | 0.045 | A:0;G:7999;C:0;T:377;total:8376 | iSNV |
| F50 | F50-10 | 2362 | E | 0.0527 | A:0;G:4704;C:0;T:262;total:4966 | iSNV |
| F50 | F50-10 | 2372 | E | 0.9979 | A:0;G:0;C:11;T:5047;total:5058 | SNP |
| F50 | F50-10 | 2493 | NS1 | 0.9974 | A:12;G:4529;C:0;T:0;total:4541 | SNP |
| F50 | F50-10 | 2504 | NS1 | 0.9967 | A:14;G:4497;C:0;T:1;total:4512 | SNP |
| F50 | F50-10 | 2531 | NS1 | 0.996 | A:0;G:0;C:5927;T:24;total:5951 | SNP |
| F50 | F50-10 | 3572 | NS1 | 0.9964 | A:0;G:0;C:21;T:5764;total:5785 | SNP |
| F50 | F50-10 | 3875 | NS2A | 0.0512 | A:8;G:0;C:11601;T:627;total:12236 | iSNV |
| F50 | F50-10 | 3962 | NS2A | 0.9969 | A:0;G:2;C:29;T:9946;total:9977 | SNP |
| F50 | F50-10 | 4155 | NS2A | 0.9967 | A:4410;G:1;C:1;T:13;total:4425 | SNP |
| F50 | F50-10 | 4712 | NS3 | 0.9961 | A:10884;G:41;C:1;T:1;total:10927 | SNP |
| F50 | F50-10 | 5311 | NS3 | 0.9935 | A:0;G:1;C:52;T:8047;total:8100 | SNP |
| F50 | F50-10 | 6080 | NS3 | 0.9976 | A:10352;G:24;C:1;T:0;total:10377 | SNP |
| F50 | F50-10 | 7112 | NS4A | 0.0205 | A:4952;G:0;C:2;T:104;total:5058 | iSNV |
| F50 | F50-10 | 8045 | NS5 | 0.0642 | A:943;G:13735;C:0;T:0;total:14678 | iSNV |
| F50 | F50-10 | 8900 | NS5 | 0.9952 | A:77;G:0;C:17315;T:7;total:17399 | SNP |
| F50 | F50-10 | 9008 | NS5 | 0.0315 | A:0;G:0;C:483;T:14819;total:15302 | iSNV |
| F50 | F50-10 | 9370 | NS5 | 0.1611 | A:3574;G:2;C:0;T:18596;total:22172 | iSNV |
| F50 | F50-10 | 9446 | NS5 | 0.9975 | A:44;G:18837;C:0;T:5;total:18886 | SNP |
| F50 | F50-10 | 10069 | NS5 | 0.0272 | A:3;G:0;C:9928;T:278;total:10209 | iSNV |
| F50 | F50-10 | 10388 | NS5 | 0.0567 | A:3;G:0;C:9718;T:585;total:10306 | iSNV |
| F50 | F50-10 | 10428 | 3'-UTR | 0.3049 | A:3;G:0;C:6094;T:2675;total:8772 | iSNV |
| F50 | F50-11 | 719 | M | 0.0369 | A:6;G:2;C:18007;T:692;total:18707 | iSNV |
| F50 | F50-11 | 909 | M | 0.0479 | A:3;G:0;C:13378;T:674;total:14055 | iSNV |
| F50 | F50-11 | 1218 | E | 0.997 | A:0;G:1;C:45;T:15206;total:15252 | SNP |
| F50 | F50-11 | 1431 | E | 0.0967 | A:6;G:1;C:17185;T:1842;total:19034 | iSNV |
| F50 | F50-11 | 1512 | E | 0.0567 | A:15129;G:911;C:0;T:0;total:16040 | iSNV |
| F50 | F50-11 | 1514 | E | 0.1984 | A:12804;G:2;C:3172;T:2;total:15980 | iSNV |
| F50 | F50-11 | 1535 | E | 0.0374 | A:7;G:0;C:14170;T:552;total:14729 | iSNV |
| F50 | F50-11 | 1551 | E | 0.44 | A:2;G:6402;C:8141;T:5;total:14550 | iSNV |
| F50 | F50-11 | 1797 | E | 0.2125 | A:4027;G:1438;C:7;T:13474;total:18946 | iSNV |
| F50 | F50-11 | 2805 | NS1 | 0.0528 | A:20139;G:1124;C:1;T:1;total:21265 | iSNV |
| F50 | F50-11 | 3257 | NS1 | 0.0535 | A:1;G:1;C:1053;T:18609;total:19664 | iSNV |
| F50 | F50-11 | 3261 | NS1 | 0.0265 | A:19259;G:525;C:1;T:0;total:19785 | iSNV |
| F50 | F50-11 | 3697 | NS1 | 0.4122 | A:1;G:3;C:5701;T:4001;total:9706 | iSNV |
| F50 | F50-11 | 3869 | NS2A | 0.9989 | A:0;G:0;C:18;T:15453;total:15471 | SNP |
| F50 | F50-11 | 4233 | NS2B | 0.4666 | A:0;G:4661;C:1;T:5326;total:9988 | iSNV |
| F50 | F50-11 | 4295 | NS2B | 0.0225 | A:14025;G:1;C:0;T:324;total:14350 | iSNV |
| F50 | F50-11 | 4447 | NS2B | 0.1338 | A:10540;G:1630;C:3;T:2;total:12175 | iSNV |
| F50 | F50-11 | 5207 | NS3 | 0.0493 | A:13143;G:682;C:0;T:1;total:13826 | iSNV |
| F50 | F50-11 | 5256 | NS3 | 0.4506 | A:0;G:1;C:8160;T:6695;total:14856 | iSNV |
| F50 | F50-11 | 5736 | NS3 | 0.4372 | A:8882;G:0;C:11430;T:3;total:20315 | iSNV |
| F50 | F50-11 | 6509 | NS4A | 0.0452 | A:1;G:1;C:760;T:16047;total:16809 | iSNV |
| F50 | F50-11 | 6867 | NS4A | 0.1227 | A:1721;G:12299;C:0;T:2;total:14022 | iSNV |
| F50 | F50-11 | 6900 | NS4A | 0.4552 | A:6127;G:7327;C:6;T:0;total:13460 | iSNV |
| F50 | F50-11 | 7264 | NS4A | 0.4299 | A:2;G:0;C:5881;T:4438;total:10321 | iSNV |
| F50 | F50-11 | 7633 | NS4B | 0.4852 | A:0;G:0;C:8000;T:7541;total:15541 | iSNV |
| F50 | F50-11 | 8948 | NS5 | 0.1006 | A:1678;G:14990;C:1;T:0;total:16669 | iSNV |
| F50 | F50-11 | 9342 | NS5 | 0.0531 | A:2;G:1;C:19973;T:1121;total:21097 | iSNV |
| F50 | F50-11 | 9370 | NS5 | 0.0573 | A:1250;G:4;C:69;T:20491;total:21814 | iSNV |
| F50 | F50-11 | 9830 | NS5 | 0.0204 | A:0;G:0;C:300;T:14359;total:14659 | iSNV |
| F50 | F50-11 | 9880 | NS5 | 0.2262 | A:3601;G:12311;C:1;T:0;total:15913 | iSNV |
| F50 | F50-11 | 9899 | NS5 | 0.1436 | A:4;G:0;C:12459;T:2091;total:14554 | iSNV |

| | | | | | | |
|---|---|---|---|---|---|---|
| F50 | F50-11 | 10428 | 3'-UTR | 0.7763 | A:0;G:3;C:3159;T:10955;total:14117 | iSNV |
| F50 | F50-11 | 10568 | 3'-UTR | 0.0479 | A:2;G:0;C:11661;T:587;total:12250 | iSNV |
| F50 | F50-11 | 10589 | 3'-UTR | 0.237 | A:1;G:3092;C:4;T:9946;total:13043 | iSNV |
| F50 | F50-11 | 10860 | 3'-UTR | 0.1296 | A:765;G:0;C:5127;T:8;total:5900 | iSNV |
| F50 | F50-12 | 332 | C | 0.0509 | A:29488;G:1584;C:0;T:0;total:31072 | iSNV |
| F50 | F50-12 | 353 | C | 0.4918 | A:14547;G:14087;C:2;T:6;total:28642 | iSNV |
| F50 | F50-12 | 645 | M | 0.4964 | A:12370;G:12203;C:3;T:3;total:24579 | iSNV |
| F50 | F50-12 | 869 | M | 0.1524 | A:0;G:3914;C:3;T:21759;total:25676 | iSNV |
| F50 | F50-12 | 998 | E | 0.5371 | A:1;G:0;C:9937;T:11528;total:21466 | iSNV |
| F50 | F50-12 | 1083 | E | 0.2313 | A:16230;G:4;C:4888;T:3;total:21125 | iSNV |
| F50 | F50-12 | 1117 | E | 0.5324 | A:11669;G:13282;C:1;T:0;total:24952 | iSNV |
| F50 | F50-12 | 1131 | E | 0.2577 | A:5;G:6752;C:19433;T:4;total:26194 | iSNV |
| F50 | F50-12 | 1218 | E | 0.4493 | A:6;G:1;C:13854;T:11313;total:25174 | iSNV |
| F50 | F50-12 | 1313 | E | 0.0334 | A:9;G:2;C:25054;T:868;total:25933 | iSNV |
| F50 | F50-12 | 1428 | E | 0.073 | A:27887;G:2199;C:0;T:0;total:30086 | iSNV |
| F50 | F50-12 | 1439 | E | 0.0788 | A:0;G:4;C:2318;T:27067;total:29389 | iSNV |
| F50 | F50-12 | 1447 | E | 0.0793 | A:2443;G:3;C:28325;T:11;total:30782 | iSNV |
| F50 | F50-12 | 1512 | E | 0.1129 | A:22326;G:2843;C:4;T:0;total:25173 | iSNV |
| F50 | F50-12 | 1551 | E | 0.3126 | A:2;G:7218;C:15859;T:8;total:23087 | iSNV |
| F50 | F50-12 | 1640 | E | 0.2347 | A:5003;G:3;C:16293;T:11;total:21310 | iSNV |
| F50 | F50-12 | 2261 | E | 0.0863 | A:1;G:0;C:17454;T:1649;total:19104 | iSNV |
| F50 | F50-12 | 2277 | E | 0.529 | A:8;G:1;C:10127;T:9026;total:19162 | iSNV |
| F50 | F50-12 | 2362 | E | 0.0775 | A:1;G:14528;C:1222;T:4;total:15755 | iSNV |
| F50 | F50-12 | 2369 | E | 0.0357 | A:574;G:15497;C:0;T:0;total:16071 | iSNV |
| F50 | F50-12 | 2499 | NS1 | 0.0225 | A:18652;G:431;C:0;T:1;total:19084 | iSNV |
| F50 | F50-12 | 2942 | NS1 | 0.0714 | A:8;G:1;C:28154;T:2168;total:30331 | iSNV |
| F50 | F50-12 | 3719 | NS1 | 0.026 | A:4;G:9;C:486;T:18179;total:18678 | iSNV |
| F50 | F50-12 | 3869 | NS2A | 0.4957 | A:2;G:0;C:13343;T:13122;total:26467 | iSNV |
| F50 | F50-12 | 3959 | NS2A | 0.0804 | A:4;G:0;C:25635;T:2243;total:27882 | iSNV |
| F50 | F50-12 | 4068 | NS2A | 0.0679 | A:19461;G:5;C:0;T:1420;total:20886 | iSNV |
| F50 | F50-12 | 4070 | NS2A | 0.0754 | A:2;G:1591;C:3;T:19495;total:21091 | iSNV |
| F50 | F50-12 | 4293 | NS2B | 0.2399 | A:5564;G:17618;C:0;T:8;total:23190 | iSNV |
| F50 | F50-12 | 4490 | NS2B | 0.0307 | A:0;G:8;C:676;T:21281;total:21965 | iSNV |
| F50 | F50-12 | 4882 | NS3 | 0.0318 | A:3;G:4;C:32935;T:1085;total:34027 | iSNV |
| F50 | F50-12 | 4974 | NS3 | 0.2666 | A:8031;G:22036;C:45;T:6;total:30118 | iSNV |
| F50 | F50-12 | 5256 | NS3 | 0.3023 | A:5;G:1;C:17339;T:7517;total:24862 | iSNV |
| F50 | F50-12 | 5266 | NS3 | 0.0412 | A:2;G:0;C:23968;T:1030;total:25000 | iSNV |
| F50 | F50-12 | 5537 | NS3 | 0.1991 | A:25044;G:6228;C:2;T:2;total:31276 | iSNV |
| F50 | F50-12 | 5736 | NS3 | 0.3117 | A:10582;G:0;C:23358;T:3;total:33943 | iSNV |
| F50 | F50-12 | 5780 | NS3 | 0.0396 | A:0;G:4;C:1391;T:33699;total:35094 | iSNV |
| F50 | F50-12 | 5813 | NS3 | 0.0759 | A:13;G:3;C:30381;T:2497;total:32894 | iSNV |
| F50 | F50-12 | 6714 | NS4A | 0.0262 | A:651;G:24132;C:2;T:0;total:24785 | iSNV |
| F50 | F50-12 | 6900 | NS4A | 0.3196 | A:6994;G:14884;C:1;T:2;total:21881 | iSNV |
| F50 | F50-12 | 6969 | NS4A | 0.0582 | A:23379;G:1448;C:52;T:0;total:24879 | iSNV |
| F50 | F50-12 | 7067 | NS4A | 0.0375 | A:23986;G:937;C:9;T:0;total:24932 | iSNV |
| F50 | F50-12 | 7178 | NS4A | 0.0565 | A:3;G:1;C:18749;T:1124;total:19877 | iSNV |
| F50 | F50-12 | 7264 | NS4A | 0.2932 | A:1;G:1;C:12130;T:5035;total:17167 | iSNV |
| F50 | F50-12 | 7478 | NS4B | 0.0627 | A:2;G:0;C:14908;T:999;total:15909 | iSNV |
| F50 | F50-12 | 7561 | NS4B | 0.0609 | A:2;G:4;C:1450;T:22343;total:23799 | iSNV |
| F50 | F50-12 | 7633 | NS4B | 0.3679 | A:3;G:2;C:16420;T:9561;total:25986 | iSNV |
| F50 | F50-12 | 7634 | NS4B | 0.0343 | A:0;G:904;C:9;T:25401;total:26314 | iSNV |
| F50 | F50-12 | 8546 | NS5 | 0.0733 | A:8;G:1;C:33078;T:2619;total:35706 | iSNV |
| F50 | F50-12 | 8777 | NS5 | 0.026 | A:589;G:22043;C:5;T:3;total:22640 | iSNV |
| F50 | F50-12 | 9023 | NS5 | 0.0589 | A:0;G:1;C:1457;T:23269;total:24727 | iSNV |
| F50 | F50-12 | 9491 | NS5 | 0.0418 | A:30116;G:1315;C:1;T:3;total:31435 | iSNV |
| F50 | F50-12 | 9699 | NS5 | 0.2826 | A:4;G:1;C:19518;T:7693;total:27216 | iSNV |
| F50 | F50-12 | 10259 | NS5 | 0.4903 | A:16564;G:15937;C:1;T:1;total:32503 | iSNV |
| F50 | F50-12 | 10347 | NS5 | 0.2515 | A:7;G:2;C:23132;T:7779;total:30920 | iSNV |
| F50 | F50-12 | 10428 | 3'-UTR | 0.6649 | A:5;G:2;C:8628;T:17111;total:25746 | iSNV |
| F50 | F50-12 | 10447 | 3'-UTR | 0.4931 | A:5;G:0;C:11769;T:11454;total:23228 | iSNV |
| F50 | F50-12 | 10932 | 3'-UTR | 0.024 | A:284;G:0;C:7;T:0;total:291 | iSNV |
| F50 | F50-13 | 395 | C | 0.9412 | A:1559;G:24923;C:2;T:1;total:26485 | iSNV |
| F50 | F50-13 | 530 | M | 0.0416 | A:3;G:1;C:23570;T:1024;total:24598 | iSNV |
| F50 | F50-13 | 1113 | E | 0.4573 | A:12358;G:1;C:10424;T:8;total:22791 | iSNV |
| F50 | F50-13 | 1175 | E | 0.059 | A:3;G:6;C:1505;T:23961;total:25475 | iSNV |
| F50 | F50-13 | 1218 | E | 0.9979 | A:0;G:1;C:46;T:21550;total:21597 | SNP |
| F50 | F50-13 | 1413 | E | 0.9432 | A:1561;G:25881;C:2;T:7;total:27451 | iSNV |
| F50 | F50-13 | 1512 | E | 0.2372 | A:17729;G:5515;C:3;T:0;total:23247 | iSNV |
| F50 | F50-13 | 1563 | E | 0.0248 | A:2;G:0;C:20602;T:525;total:21129 | iSNV |
| F50 | F50-13 | 1708 | E | 0.1413 | A:3329;G:20215;C:0;T:4;total:23548 | iSNV |
| F50 | F50-13 | 1769 | E | 0.0582 | A:0;G:11;C:1491;T:24092;total:25594 | iSNV |
| F50 | F50-13 | 1786 | E | 0.0323 | A:1;G:5;C:866;T:25887;total:26759 | iSNV |
| F50 | F50-13 | 1789 | E | 0.0262 | A:1;G:3;C:710;T:26366;total:27080 | iSNV |
| F50 | F50-13 | 1796 | E | 0.4557 | A:0;G:14844;C:1;T:12433;total:27278 | iSNV |
| F50 | F50-13 | 1797 | E | 0.0636 | A:0;G:1736;C:0;T:25546;total:27282 | iSNV |
| F50 | F50-13 | 2543 | NS1 | 0.05 | A:0;G:1;C:18047;T:951;total:18999 | iSNV |
| F50 | F50-13 | 2786 | NS1 | 0.1033 | A:5;G:6;C:28118;T:3241;total:31370 | iSNV |
| F50 | F50-13 | 3652 | NS1 | 0.0368 | A:1;G:6;C:635;T:16583;total:17225 | iSNV |
| F50 | F50-13 | 3869 | NS2A | 0.9988 | A:2;G:0;C:27;T:23308;total:23337 | SNP |
| F50 | F50-13 | 3906 | NS2A | 0.2298 | A:5916;G:19811;C:7;T:1;total:25735 | iSNV |
| F50 | F50-13 | 4052 | NS2A | 0.038 | A:1;G:0;C:16902;T:668;total:17571 | iSNV |
| F50 | F50-13 | 4592 | NS2B | 0.0394 | A:24885;G:1023;C:1;T:18;total:25927 | iSNV |

| | | | | | | |
|---|---|---|---|---|---|---|
| F50 | F50-13 | 4632 | NS3 | 0.1087 | A:0;G:3202;C:12;T:26241;total:29455 | iSNV |
| F50 | F50-13 | 4792 | NS3 | 0.0611 | A:26158;G:1703;C:5;T:1;total:27867 | iSNV |
| F50 | F50-13 | 5291 | NS3 | 0.0251 | A:22015;G:568;C:0;T:1;total:22584 | iSNV |
| F50 | F50-13 | 5490 | NS3 | 0.0209 | A:28120;G:601;C:0;T:2;total:28723 | iSNV |
| F50 | F50-13 | 6316 | NS3 | 0.0244 | A:0;G:1;C:19394;T:487;total:19882 | iSNV |
| F50 | F50-13 | 6867 | NS4A | 0.0426 | A:822;G:18441;C:0;T:0;total:19263 | iSNV |
| F50 | F50-13 | 6900 | NS4A | 0.0757 | A:147;G:17309;C:1;T:1431;total:18888 | iSNV |
| F50 | F50-13 | 6934 | NS4A | 0.1011 | A:18447;G:2076;C:1;T:0;total:20524 | iSNV |
| F50 | F50-13 | 6969 | NS4A | 0.4441 | A:11673;G:0;C:9328;T:0;total:21001 | iSNV |
| F50 | F50-13 | 6970 | NS4A | 0.0394 | A:20119;G:1;C:826;T:2;total:20948 | iSNV |
| F50 | F50-13 | 7176 | NS4A | 0.0388 | A:18069;G:731;C:6;T:1;total:18807 | iSNV |
| F50 | F50-13 | 7256 | NS4A | 0.045 | A:751;G:15919;C:0;T:4;total:16674 | iSNV |
| F50 | F50-13 | 7595 | NS4B | 0.4658 | A:9368;G:1;C:2;T:10740;total:20111 | iSNV |
| F50 | F50-13 | 7633 | NS4B | 0.123 | A:2;G:0;C:19847;T:2784;total:22633 | iSNV |
| F50 | F50-13 | 7674 | NS4B | 0.0242 | A:22415;G:18;C:557;T:3;total:22993 | iSNV |
| F50 | F50-13 | 7682 | NS5 | 0.0397 | A:889;G:21495;C:0;T:1;total:22385 | iSNV |
| F50 | F50-13 | 7903 | NS5 | 0.0392 | A:26181;G:1069;C:4;T:0;total:27254 | iSNV |
| F50 | F50-13 | 9266 | NS5 | 0.023 | A:687;G:29103;C:0;T:2;total:29792 | iSNV |
| F50 | F50-13 | 9482 | NS5 | 0.0258 | A:743;G:27944;C:2;T:2;total:28691 | iSNV |
| F50 | F50-13 | 9690 | NS5 | 0.0681 | A:1613;G:22039;C:0;T:0;total:23652 | iSNV |
| F50 | F50-13 | 10376 | NS5 | 0.9408 | A:21163;G:1334;C:2;T:1;total:22500 | iSNV |
| F50 | F50-13 | 10400 | 3'-UTR | 0.0371 | A:4;G:780;C:0;T:20204;total:20988 | iSNV |
| F50 | F50-13 | 10428 | 3'-UTR | 0.9058 | A:2;G:1;C:1863;T:17893;total:19759 | iSNV |
| F50 | F50-13 | 10579 | 3'-UTR | 0.4655 | A:6;G:0;C:9707;T:8462;total:18175 | iSNV |
| F50 | F50-14 | 516 | M | 0.332 | A:13616;G:6770;C:1;T:2;total:20389 | iSNV |
| F50 | F50-14 | 686 | M | 0.1634 | A:2;G:0;C:15874;T:3102;total:18978 | iSNV |
| F50 | F50-14 | 794 | M | 0.0508 | A:1;G:0;C:775;T:14450;total:15226 | iSNV |
| F50 | F50-14 | 836 | M | 0.3726 | A:2;G:2;C:6025;T:10138;total:16167 | iSNV |
| F50 | F50-14 | 998 | E | 0.8258 | A:0;G:6;C:2464;T:11670;total:14140 | iSNV |
| F50 | F50-14 | 1057 | E | 0.0999 | A:1349;G:12150;C:0;T:1;total:13500 | iSNV |
| F50 | F50-14 | 1067 | E | 0.0499 | A:4;G:1;C:14487;T:762;total:15254 | iSNV |
| F50 | F50-14 | 1117 | E | 0.1535 | A:14203;G:2576;C:0;T:0;total:16779 | iSNV |
| F50 | F50-14 | 1218 | E | 0.9983 | A:0;G:1;C:27;T:15864;total:15892 | SNP |
| F50 | F50-14 | 1343 | E | 0.1552 | A:6;G:1;C:16196;T:2978;total:19181 | iSNV |
| F50 | F50-14 | 1512 | E | 0.5568 | A:7351;G:9234;C:0;T:0;total:16585 | iSNV |
| F50 | F50-14 | 1669 | E | 0.0511 | A:4;G:1;C:12550;T:677;total:13232 | iSNV |
| F50 | F50-14 | 1769 | E | 0.16 | A:2938;G:2;C:0;T:15418;total:18358 | iSNV |
| F50 | F50-14 | 2369 | E | 0.158 | A:1563;G:8254;C:1;T:69;total:9887 | iSNV |
| F50 | F50-14 | 2481 | NS1 | 0.0344 | A:11423;G:407;C:1;T:0;total:11831 | iSNV |
| F50 | F50-14 | 2972 | NS1 | 0.0236 | A:3;G:0;C:20657;T:500;total:21160 | iSNV |
| F50 | F50-14 | 3167 | NS1 | 0.0455 | A:4;G:0;C:18795;T:897;total:19696 | iSNV |
| F50 | F50-14 | 3386 | NS1 | 0.0577 | A:0;G:2;C:14588;T:894;total:15484 | iSNV |
| F50 | F50-14 | 3536 | NS1 | 0.0323 | A:6;G:2;C:12958;T:433;total:13399 | iSNV |
| F50 | F50-14 | 3731 | NS2A | 0.0523 | A:9084;G:4;C:502;T:0;total:9590 | iSNV |
| F50 | F50-14 | 3869 | NS2A | 0.2696 | A:6;G:0;C:12930;T:4777;total:17713 | iSNV |
| F50 | F50-14 | 3938 | NS2A | 0.0476 | A:18218;G:912;C:2;T:0;total:19132 | iSNV |
| F50 | F50-14 | 3950 | NS2A | 0.1607 | A:8;G:2;C:16208;T:3107;total:19325 | iSNV |
| F50 | F50-14 | 4043 | NS2A | 0.0295 | A:0;G:1;C:12979;T:395;total:13375 | iSNV |
| F50 | F50-14 | 4274 | NS2B | 0.0338 | A:1;G:4;C:434;T:12381;total:12820 | iSNV |
| F50 | F50-14 | 4294 | NS2B | 0.0211 | A:14652;G:317;C:1;T:2;total:14972 | iSNV |
| F50 | F50-14 | 4389 | NS2B | 0.1461 | A:2274;G:13283;C:0;T:1;total:15558 | iSNV |
| F50 | F50-14 | 4697 | NS3 | 0.8188 | A:10;G:3;C:17200;T:3811;total:21024 | iSNV |
| F50 | F50-14 | 4974 | NS3 | 0.1603 | A:3129;G:16383;C:1;T:1;total:19514 | iSNV |
| F50 | F50-14 | 5330 | NS3 | 0.0257 | A:14671;G:388;C:1;T:2;total:15062 | iSNV |
| F50 | F50-14 | 5359 | NS3 | 0.0238 | A:16152;G:3;C:395;T:0;total:16550 | iSNV |
| F50 | F50-14 | 5558 | NS3 | 0.1511 | A:3070;G:17234;C:0;T:6;total:20310 | iSNV |
| F50 | F50-14 | 5736 | NS3 | 0.0403 | A:928;G:0;C:22058;T:8;total:22994 | iSNV |
| F50 | F50-14 | 5822 | NS3 | 0.0339 | A:0;G:1;C:763;T:21724;total:22488 | iSNV |
| F50 | F50-14 | 5864 | NS3 | 0.0826 | A:2;G:8;C:1707;T:18925;total:20642 | iSNV |
| F50 | F50-14 | 5952 | NS3 | 0.8158 | A:0;G:0;C:4024;T:17820;total:21844 | iSNV |
| F50 | F50-14 | 6010 | NS3 | 0.0319 | A:20239;G:669;C:1;T:0;total:20909 | iSNV |
| F50 | F50-14 | 6523 | NS4A | 0.0202 | A:3;G:1;C:15187;T:314;total:15505 | iSNV |
| F50 | F50-14 | 6867 | NS4A | 0.5463 | A:7926;G:6586;C:1;T:1;total:14514 | iSNV |
| F50 | F50-14 | 7334 | NS4B | 0.0427 | A:9798;G:438;C:0;T:0;total:10236 | iSNV |
| F50 | F50-14 | 7528 | NS4B | 0.0369 | A:0;G:0;C:11967;T:459;total:12426 | iSNV |
| F50 | F50-14 | 7561 | NS4B | 0.0984 | A:0;G:5;C:1426;T:13054;total:14485 | iSNV |
| F50 | F50-14 | 7633 | NS4B | 0.7759 | A:1;G:1;C:3710;T:12843;total:16555 | iSNV |
| F50 | F50-14 | 8090 | NS5 | 0.0367 | A:704;G:18422;C:1;T:6;total:19133 | iSNV |
| F50 | F50-14 | 8114 | NS5 | 0.0346 | A:5;G:0;C:19801;T:710;total:20516 | iSNV |
| F50 | F50-14 | 9068 | NS5 | 0.16 | A:1;G:6;C:2526;T:13247;total:15780 | iSNV |
| F50 | F50-14 | 9359 | NS5 | 0.8268 | A:6;G:1;C:20677;T:4335;total:25019 | iSNV |
| F50 | F50-14 | 9370 | NS5 | 0.8254 | A:14;G:20871;C:2;T:4421;total:25308 | iSNV |
| F50 | F50-14 | 9737 | NS5 | 0.026 | A:6;G:1;C:15069;T:403;total:15479 | iSNV |
| F50 | F50-14 | 9923 | NS5 | 0.036 | A:0;G:4;C:596;T:15931;total:16531 | iSNV |
| F50 | F50-14 | 10259 | NS5 | 0.8194 | A:3784;G:17163;C:0;T:3;total:20950 | iSNV |
| F50 | F50-14 | 10419 | 3'-UTR | 0.1622 | A:0;G:4;C:13922;T:2698;total:16624 | iSNV |
| F50 | F50-14 | 10428 | 3'-UTR | 0.4057 | A:5;G:1;C:9912;T:6772;total:16690 | iSNV |
| F50 | F50-14 | 10435 | 3'-UTR | 0.0899 | A:15137;G:1496;C:1;T:0;total:16634 | iSNV |
| F50 | F50-14 | 10566 | 3'-UTR | 0.7968 | A:0;G:2;C:2423;T:9496;total:11921 | iSNV |
| F50 | F50-15 | 401 | C | 0.0268 | A:24177;G:666;C:4;T:1;total:24848 | iSNV |
| F50 | F50-15 | 803 | M | 0.3366 | A:1;G:10232;C:1;T:5193;total:15427 | iSNV |
| F50 | F50-15 | 810 | M | 0.1528 | A:6;G:0;C:13492;T:2436;total:15934 | iSNV |

| | | | | | | |
|---|---|---|---|---|---|---|
| F50 | F50-15 | 943 | M | 0.023 | A:1;G:3;C:16419;T:387;total:16810 | iSNV |
| F50 | F50-15 | 998 | E | 0.1643 | A:2;G:0;C:13497;T:2654;total:16153 | iSNV |
| F50 | F50-15 | 1218 | E | 0.9987 | A:3;G:3;C:19;T:17833;total:17858 | SNP |
| F50 | F50-15 | 1428 | E | 0.2714 | A:16339;G:6088;C:0;T:1;total:22428 | iSNV |
| F50 | F50-15 | 1512 | E | 0.3749 | A:11701;G:7020;C:1;T:1;total:18723 | iSNV |
| F50 | F50-15 | 1672 | E | 0.0358 | A:1;G:1;C:14733;T:548;total:15283 | iSNV |
| F50 | F50-15 | 1797 | E | 0.1303 | A:2;G:2893;C:10;T:19297;total:22202 | iSNV |
| F50 | F50-15 | 1847 | E | 0.0284 | A:20386;G:596;C:0;T:0;total:20982 | iSNV |
| F50 | F50-15 | 2076 | E | 0.0343 | A:14091;G:501;C:0;T:2;total:14594 | iSNV |
| F50 | F50-15 | 2241 | E | 0.0281 | A:0;G:15821;C:459;T:0;total:16280 | iSNV |
| F50 | F50-15 | 2362 | E | 0.035 | A:1;G:10942;C:0;T:398;total:11341 | iSNV |
| F50 | F50-15 | 2493 | NS1 | 0.283 | A:10218;G:4036;C:6;T:1;total:14261 | iSNV |
| F50 | F50-15 | 2625 | NS1 | 0.0649 | A:1;G:0;C:19046;T:1323;total:20370 | iSNV |
| F50 | F50-15 | 3161 | NS1 | 0.0579 | A:20825;G:1281;C:1;T:1;total:22108 | iSNV |
| F50 | F50-15 | 3396 | NS1 | 0.3184 | A:3;G:5788;C:1;T:12384;total:18176 | iSNV |
| F50 | F50-15 | 3455 | NS1 | 0.0997 | A:12340;G:1368;C:0;T:1;total:13709 | iSNV |
| F50 | F50-15 | 3869 | NS2A | 0.4666 | A:3;G:1;C:11028;T:9652;total:20684 | iSNV |
| F50 | F50-15 | 4233 | NS2B | 0.0652 | A:0;G:793;C:1;T:11363;total:12157 | iSNV |
| F50 | F50-15 | 4402 | NS2B | 0.0446 | A:16163;G:4;C:2;T:756;total:16925 | iSNV |
| F50 | F50-15 | 4697 | NS3 | 0.2091 | A:4;G:1;C:5286;T:19986;total:25277 | iSNV |
| F50 | F50-15 | 5312 | NS3 | 0.0287 | A:536;G:3;C:5;T:18069;total:18613 | iSNV |
| F50 | F50-15 | 5598 | NS3 | 0.0277 | A:614;G:21512;C:9;T:0;total:22135 | iSNV |
| F50 | F50-15 | 5736 | NS3 | 0.0577 | A:1554;G:3;C:25338;T:6;total:26901 | iSNV |
| F50 | F50-15 | 5952 | NS3 | 0.2109 | A:5;G:2;C:19670;T:5261;total:24938 | iSNV |
| F50 | F50-15 | 5996 | NS3 | 0.2286 | A:2;G:4;C:16614;T:4927;total:21547 | iSNV |
| F50 | F50-15 | 6122 | NS3 | 0.3025 | A:6239;G:14376;C:0;T:3;total:20618 | iSNV |
| F50 | F50-15 | 6252 | NS3 | 0.0234 | A:0;G:0;C:464;T:19323;total:19787 | iSNV |
| F50 | F50-15 | 6714 | NS4A | 0.0619 | A:1171;G:17733;C:0;T:2;total:18906 | iSNV |
| F50 | F50-15 | 6867 | NS4A | 0.3352 | A:5679;G:11259;C:3;T:1;total:16942 | iSNV |
| F50 | F50-15 | 7167 | NS4A | 0.0454 | A:775;G:16256;C:0;T:3;total:17034 | iSNV |
| F50 | F50-15 | 7528 | NS4B | 0.0709 | A:1;G:2;C:14214;T:1086;total:15303 | iSNV |
| F50 | F50-15 | 7633 | NS4B | 0.6963 | A:2;G:1;C:6072;T:13914;total:19989 | iSNV |
| F50 | F50-15 | 7735 | NS5 | 0.3119 | A:5957;G:13137;C:0;T:2;total:19096 | iSNV |
| F50 | F50-15 | 8518 | NS5 | 0.3043 | A:18644;G:6;C:4;T:8162;total:26816 | iSNV |
| F50 | F50-15 | 9245 | NS5 | 0.0465 | A:2;G:1;C:26189;T:1279;total:27471 | iSNV |
| F50 | F50-15 | 9284 | NS5 | 0.0331 | A:0;G:1;C:864;T:25174;total:26039 | iSNV |
| F50 | F50-15 | 9293 | NS5 | 0.0496 | A:1195;G:22862;C:2;T:1;total:24060 | iSNV |
| F50 | F50-15 | 9359 | NS5 | 0.2111 | A:0;G:0;C:5871;T:21937;total:27808 | iSNV |
| F50 | F50-15 | 9370 | NS5 | 0.1634 | A:4617;G:124;C:3;T:23505;total:28249 | iSNV |
| F50 | F50-15 | 9443 | NS5 | 0.0431 | A:28343;G:1277;C:2;T:1;total:29623 | iSNV |
| F50 | F50-15 | 10259 | NS5 | 0.2125 | A:19429;G:5245;C:1;T:0;total:24675 | iSNV |
| F50 | F50-15 | 10295 | NS5 | 0.1271 | A:5;G:1;C:21971;T:3201;total:25178 | iSNV |
| F50 | F50-15 | 10428 | 3'-UTR | 0.325 | A:3;G:2;C:13318;T:6415;total:19738 | iSNV |
| F50 | F50-15 | 10451 | 3'-UTR | 0.0342 | A:1;G:5;C:17139;T:608;total:17753 | iSNV |
| F50 | F50-15 | 10566 | 3'-UTR | 0.1989 | A:1;G:0;C:11947;T:2967;total:14915 | iSNV |
| F50 | F50-15 | 10718 | 3'-UTR | 0.0323 | A:0;G:0;C:16953;T:566;total:17519 | iSNV |
| F50 | F50-16 | 178 | C | 0.0235 | A:635;G:7;C:6;T:26276;total:26924 | iSNV |
| F50 | F50-16 | 803 | M | 0.4172 | A:4;G:9228;C:0;T:6611;total:15843 | iSNV |
| F50 | F50-16 | 810 | M | 0.1706 | A:2;G:0;C:13575;T:2793;total:16370 | iSNV |
| F50 | F50-16 | 923 | M | 0.025 | A:0;G:1;C:16183;T:415;total:16599 | iSNV |
| F50 | F50-16 | 998 | E | 0.0979 | A:5;G:0;C:14394;T:1564;total:15963 | iSNV |
| F50 | F50-16 | 1218 | E | 0.9985 | A:0;G:2;C:25;T:17614;total:17641 | SNP |
| F50 | F50-16 | 1428 | E | 0.255 | A:16512;G:5653;C:2;T:0;total:22167 | iSNV |
| F50 | F50-16 | 1432 | E | 0.0316 | A:21580;G:3;C:2;T:706;total:22291 | iSNV |
| F50 | F50-16 | 1447 | E | 0.0362 | A:830;G:0;C:22066;T:10;total:22906 | iSNV |
| F50 | F50-16 | 1512 | E | 0.4122 | A:11024;G:7733;C:0;T:1;total:18758 | iSNV |
| F50 | F50-16 | 1672 | E | 0.0617 | A:5;G:0;C:14789;T:973;total:15767 | iSNV |
| F50 | F50-16 | 1797 | E | 0.047 | A:6;G:1068;C:7;T:21607;total:22688 | iSNV |
| F50 | F50-16 | 2076 | E | 0.0349 | A:14484;G:525;C:1;T:1;total:15011 | iSNV |
| F50 | F50-16 | 2264 | E | 0.0281 | A:0;G:0;C:407;T:14026;total:14433 | iSNV |
| F50 | F50-16 | 2362 | E | 0.0332 | A:0;G:11223;C:0;T:386;total:11609 | iSNV |
| F50 | F50-16 | 2493 | NS1 | 0.2392 | A:10836;G:3408;C:1;T:2;total:14247 | iSNV |
| F50 | F50-16 | 2625 | NS1 | 0.0268 | A:3;G:0;C:19600;T:540;total:20143 | iSNV |
| F50 | F50-16 | 3178 | NS1 | 0.0294 | A:22931;G:6;C:1;T:695;total:23633 | iSNV |
| F50 | F50-16 | 3396 | NS1 | 0.3815 | A:1;G:7140;C:6;T:11565;total:18712 | iSNV |
| F50 | F50-16 | 3869 | NS2A | 0.4387 | A:4;G:0;C:11877;T:9287;total:21168 | iSNV |
| F50 | F50-16 | 4233 | NS2B | 0.0229 | A:1;G:281;C:1;T:11953;total:12236 | iSNV |
| F50 | F50-16 | 4697 | NS3 | 0.1713 | A:2;G:4;C:4353;T:21049;total:25408 | iSNV |
| F50 | F50-16 | 5312 | NS3 | 0.0295 | A:574;G:1;C:10;T:18821;total:19406 | iSNV |
| F50 | F50-16 | 5736 | NS3 | 0.0986 | A:2710;G:0;C:24750;T:5;total:27465 | iSNV |
| F50 | F50-16 | 5753 | NS3 | 0.0336 | A:897;G:25759;C:0;T:2;total:26658 | iSNV |
| F50 | F50-16 | 5813 | NS3 | 0.0222 | A:5;G:0;C:25507;T:580;total:26092 | iSNV |
| F50 | F50-16 | 5885 | NS3 | 0.0525 | A:1;G:6;C:1280;T:23066;total:24353 | iSNV |
| F50 | F50-16 | 5952 | NS3 | 0.1767 | A:3;G:1;C:21115;T:4534;total:25653 | iSNV |
| F50 | F50-16 | 5996 | NS3 | 0.1806 | A:2;G:4;C:17889;T:3946;total:21841 | iSNV |
| F50 | F50-16 | 6122 | NS3 | 0.3712 | A:7854;G:13297;C:0;T:4;total:21155 | iSNV |
| F50 | F50-16 | 6714 | NS4A | 0.0397 | A:774;G:18672;C:0;T:2;total:19448 | iSNV |
| F50 | F50-16 | 6729 | NS4A | 0.12 | A:2333;G:1;C:17078;T:17;total:19429 | iSNV |
| F50 | F50-16 | 6867 | NS4A | 0.2544 | A:4497;G:13171;C:0;T:3;total:17671 | iSNV |
| F50 | F50-16 | 7116 | NS4A | 0.0351 | A:2;G:0;C:16735;T:610;total:17347 | iSNV |
| F50 | F50-16 | 7167 | NS4A | 0.0622 | A:1100;G:16561;C:2;T:3;total:17666 | iSNV |
| F50 | F50-16 | 7528 | NS4B | 0.0926 | A:5;G:0;C:14063;T:1437;total:15505 | iSNV |

| | | | | | | |
|---|---|---|---|---|---|---|
| F50 | F50-16 | 7626 | NS4B | 0.0254 | A:505;G:2;C:15;T:19292;total:19814 | iSNV |
| F50 | F50-16 | 7633 | NS4B | 0.6305 | A:1;G:0;C:7409;T:12638;total:20048 | iSNV |
| F50 | F50-16 | 7735 | NS5 | 0.3734 | A:7173;G:12031;C:2;T:0;total:19206 | iSNV |
| F50 | F50-16 | 8336 | NS5 | 0.0207 | A:0;G:3;C:567;T:26721;total:27291 | iSNV |
| F50 | F50-16 | 8518 | NS5 | 0.3659 | A:17172;G:4;C:1;T:9915;total:27092 | iSNV |
| F50 | F50-16 | 8942 | NS5 | 0.0265 | A:3;G:0;C:21412;T:585;total:22000 | iSNV |
| F50 | F50-16 | 9122 | NS5 | 0.0404 | A:21533;G:907;C:3;T:1;total:22444 | iSNV |
| F50 | F50-16 | 9293 | NS5 | 0.0872 | A:2155;G:22547;C:0;T:4;total:24706 | iSNV |
| F50 | F50-16 | 9359 | NS5 | 0.177 | A:1;G:1;C:5090;T:23656;total:28748 | iSNV |
| F50 | F50-16 | 9370 | NS5 | 0.1704 | A:4982;G:26;C:30;T:24194;total:29232 | iSNV |
| F50 | F50-16 | 9818 | NS5 | 0.0381 | A:0;G:1;C:733;T:18473;total:19207 | iSNV |
| F50 | F50-16 | 10259 | NS5 | 0.1786 | A:20733;G:4509;C:2;T:2;total:25246 | iSNV |
| F50 | F50-16 | 10295 | NS5 | 0.0417 | A:5;G:0;C:24216;T:1054;total:25275 | iSNV |
| F50 | F50-16 | 10351 | NS5 | 0.0243 | A:550;G:22046;C:3;T:5;total:22604 | iSNV |
| F50 | F50-16 | 10428 | 3'-UTR | 0.4638 | A:2;G:1;C:10215;T:8841;total:19059 | iSNV |
| F50 | F50-16 | 10566 | 3'-UTR | 0.1607 | A:4;G:1;C:12951;T:2481;total:15437 | iSNV |
| F50 | F50-16 | 10718 | 3'-UTR | 0.0313 | A:2;G:1;C:17294;T:559;total:17856 | iSNV |
| F50 | F50-17 | 645 | M | 0.1184 | A:14026;G:1886;C:3;T:4;total:15919 | iSNV |
| F50 | F50-17 | 1057 | E | 0.1966 | A:2518;G:10287;C:0;T:1;total:12806 | iSNV |
| F50 | F50-17 | 1218 | E | 0.9988 | A:0;G:2;C:17;T:14708;total:14727 | SNP |
| F50 | F50-17 | 1413 | E | 0.0846 | A:16646;G:1540;C:0;T:0;total:18186 | iSNV |
| F50 | F50-17 | 1428 | E | 0.4734 | A:9197;G:8272;C:2;T:1;total:17472 | iSNV |
| F50 | F50-17 | 1447 | E | 0.1169 | A:2084;G:0;C:15723;T:5;total:17812 | iSNV |
| F50 | F50-17 | 1512 | E | 0.081 | A:13501;G:43;C:1194;T:0;total:14738 | iSNV |
| F50 | F50-17 | 1568 | E | 0.0846 | A:0;G:0;C:11072;T:1024;total:12096 | iSNV |
| F50 | F50-17 | 1976 | E | 0.3562 | A:1;G:6;C:7984;T:4423;total:12414 | iSNV |
| F50 | F50-17 | 2070 | E | 0.1258 | A:10345;G:1490;C:1;T:1;total:11837 | iSNV |
| F50 | F50-17 | 2276 | E | 0.0873 | A:1;G:0;C:11418;T:1093;total:12512 | iSNV |
| F50 | F50-17 | 2376 | E | 0.1208 | A:4;G:0;C:9470;T:1302;total:10776 | iSNV |
| F50 | F50-17 | 2960 | NS1 | 0.471 | A:0;G:1;C:9634;T:8580;total:18215 | iSNV |
| F50 | F50-17 | 3002 | NS1 | 0.0811 | A:3;G:0;C:14307;T:1264;total:15574 | iSNV |
| F50 | F50-17 | 3669 | NS1 | 0.0202 | A:4;G:0;C:12779;T:264;total:13047 | iSNV |
| F50 | F50-17 | 3869 | NS2A | 0.9985 | A:0;G:0;C:26;T:16381;total:16407 | SNP |
| F50 | F50-17 | 3878 | NS2A | 0.0204 | A:17566;G:366;C:2;T:0;total:17934 | iSNV |
| F50 | F50-17 | 4091 | NS2A | 0.065 | A:923;G:13264;C:0;T:0;total:14187 | iSNV |
| F50 | F50-17 | 4233 | NS2B | 0.0971 | A:0;G:1018;C:2;T:9460;total:10480 | iSNV |
| F50 | F50-17 | 4447 | NS2B | 0.1857 | A:10361;G:2364;C:2;T:0;total:12727 | iSNV |
| F50 | F50-17 | 4974 | NS3 | 0.0805 | A:1566;G:17881;C:1;T:3;total:19451 | iSNV |
| F50 | F50-17 | 5327 | NS3 | 0.0799 | A:13014;G:2;C:0;T:1131;total:14147 | iSNV |
| F50 | F50-17 | 5415 | NS3 | 0.4198 | A:1;G:1;C:10979;T:7946;total:18927 | iSNV |
| F50 | F50-17 | 5575 | NS3 | 0.1711 | A:4;G:1;C:16002;T:3305;total:19312 | iSNV |
| F50 | F50-17 | 5705 | NS3 | 0.17 | A:16664;G:3415;C:2;T:2;total:20083 | iSNV |
| F50 | F50-17 | 5718 | NS3 | 0.4225 | A:8864;G:12113;C:0;T:2;total:20979 | iSNV |
| F50 | F50-17 | 5765 | NS3 | 0.0239 | A:5;G:1;C:20910;T:513;total:21429 | iSNV |
| F50 | F50-17 | 5918 | NS3 | 0.0827 | A:1562;G:17308;C:0;T:2;total:18872 | iSNV |
| F50 | F50-17 | 6063 | NS3 | 0.0791 | A:5;G:0;C:17699;T:1522;total:19226 | iSNV |
| F50 | F50-17 | 6970 | NS4A | 0.2906 | A:11543;G:4;C:4733;T:6;total:16286 | iSNV |
| F50 | F50-17 | 7151 | NS4A | 0.2261 | A:2;G:2;C:12822;T:3749;total:16575 | iSNV |
| F50 | F50-17 | 7264 | NS4A | 0.1519 | A:2;G:0;C:10342;T:1854;total:12198 | iSNV |
| F50 | F50-17 | 7481 | NS4B | 0.0685 | A:0;G:0;C:684;T:9289;total:9973 | iSNV |
| F50 | F50-17 | 7633 | NS4B | 0.9915 | A:0;G:0;C:152;T:17553;total:17705 | SNP |
| F50 | F50-17 | 8430 | NS5 | 0.0738 | A:2;G:2;C:19817;T:1580;total:21401 | iSNV |
| F50 | F50-17 | 10364 | NS5 | 0.2043 | A:3;G:0;C:14895;T:3826;total:18724 | iSNV |
| F50 | F50-17 | 10376 | NS5 | 0.072 | A:1358;G:17465;C:12;T:5;total:18840 | iSNV |
| F50 | F50-17 | 10420 | 3'-UTR | 0.023 | A:2;G:1;C:380;T:16100;total:16483 | iSNV |
| F50 | F50-17 | 10428 | 3'-UTR | 0.8034 | A:1;G:0;C:3165;T:12932;total:16098 | iSNV |
| F50 | F50-17 | 10447 | 3'-UTR | 0.4508 | A:2;G:0;C:7745;T:6359;total:14106 | iSNV |
| F50 | F50-17 | 10451 | 3'-UTR | 0.022 | A:3;G:304;C:13405;T:87;total:13799 | iSNV |
| F50 | F50-17 | 10805 | 3'-UTR | 0.0253 | A:307;G:11802;C:0;T:1;total:12110 | iSNV |
| F50 | F50-18 | 554 | M | 0.0217 | A:6;G:0;C:20824;T:464;total:21294 | iSNV |
| F50 | F50-18 | 998 | E | 0.3522 | A:2;G:1;C:11167;T:6074;total:17244 | iSNV |
| F50 | F50-18 | 1218 | E | 0.9981 | A:0;G:0;C:37;T:19073;total:19110 | SNP |
| F50 | F50-18 | 1413 | E | 0.584 | A:9964;G:13982;C:1;T:0;total:23947 | iSNV |
| F50 | F50-18 | 1512 | E | 0.5432 | A:7972;G:9;C:9468;T:2;total:17451 | iSNV |
| F50 | F50-18 | 1562 | E | 0.3764 | A:9541;G:5761;C:3;T:0;total:15305 | iSNV |
| F50 | F50-18 | 1797 | E | 0.3797 | A:1;G:8185;C:2;T:13364;total:21552 | iSNV |
| F50 | F50-18 | 1800 | E | 0.0255 | A:1;G:2;C:554;T:21109;total:21666 | iSNV |
| F50 | F50-18 | 2181 | E | 0.3337 | A:12622;G:6323;C:2;T:1;total:18948 | iSNV |
| F50 | F50-18 | 3032 | NS1 | 0.0276 | A:1;G:0;C:18630;T:529;total:19160 | iSNV |
| F50 | F50-18 | 3440 | NS1 | 0.0255 | A:16865;G:442;C:5;T:1;total:17313 | iSNV |
| F50 | F50-18 | 3869 | NS2A | 0.659 | A:4;G:0;C:7925;T:15305;total:23234 | iSNV |
| F50 | F50-18 | 4091 | NS2A | 0.4964 | A:7266;G:7369;C:1;T:1;total:14637 | iSNV |
| F50 | F50-18 | 4447 | NS2B | 0.0306 | A:15352;G:486;C:4;T:1;total:15843 | iSNV |
| F50 | F50-18 | 4458 | NS2B | 0.0371 | A:581;G:15063;C:0;T:4;total:15648 | iSNV |
| F50 | F50-18 | 4697 | NS3 | 0.3186 | A:2;G:1;C:8407;T:17973;total:26383 | iSNV |
| F50 | F50-18 | 5072 | NS3 | 0.1084 | A:2342;G:19256;C:0;T:2;total:21600 | iSNV |
| F50 | F50-18 | 5365 | NS3 | 0.0609 | A:19845;G:1289;C:2;T:1;total:21137 | iSNV |
| F50 | F50-18 | 5707 | NS3 | 0.0549 | A:28526;G:1658;C:2;T:0;total:30186 | iSNV |
| F50 | F50-18 | 5952 | NS3 | 0.3248 | A:8;G:0;C:20461;T:9849;total:30318 | iSNV |
| F50 | F50-18 | 6322 | NS3 | 0.1111 | A:3;G:0;C:15974;T:1998;total:17975 | iSNV |
| F50 | F50-18 | 6715 | NS4A | 0.0242 | A:5;G:1;C:21121;T:525;total:21652 | iSNV |
| F50 | F50-18 | 6740 | NS4A | 0.3001 | A:2;G:0;C:14623;T:6272;total:20897 | iSNV |

| | | | | | | |
|---|---|---|---|---|---|---|
| F50 | F50-18 | 6867 | NS4A | 0.3761 | A:7374;G:12229;C:1;T:0;total:19604 | iSNV |
| F50 | F50-18 | 6900 | NS4A | 0.062 | A:1161;G:17557;C:0;T:3;total:18721 | iSNV |
| F50 | F50-18 | 6927 | NS4A | 0.1281 | A:8;G:1;C:16852;T:2478;total:19339 | iSNV |
| F50 | F50-18 | 7091 | NS4A | 0.0506 | A:4;G:1;C:17300;T:923;total:18228 | iSNV |
| F50 | F50-18 | 7561 | NS4B | 0.3202 | A:1;G:8;C:6259;T:13276;total:19544 | iSNV |
| F50 | F50-18 | 7633 | NS4B | 0.6421 | A:2;G:4;C:8298;T:14877;total:23181 | iSNV |
| F50 | F50-18 | 7657 | NS4B | 0.0221 | A:23608;G:535;C:1;T:0;total:24144 | iSNV |
| F50 | F50-18 | 7658 | NS4B | 0.0249 | A:0;G:2;C:604;T:23568;total:24174 | iSNV |
| F50 | F50-18 | 7836 | NS5 | 0.0749 | A:7;G:1800;C:22192;T:6;total:24005 | iSNV |
| F50 | F50-18 | 8430 | NS5 | 0.5918 | A:6;G:10;C:11562;T:16742;total:28320 | iSNV |
| F50 | F50-18 | 9359 | NS5 | 0.3242 | A:1;G:2;C:10777;T:22453;total:33233 | iSNV |
| F50 | F50-18 | 9764 | NS5 | 0.432 | A:1;G:3;C:11428;T:8698;total:20130 | iSNV |
| F50 | F50-18 | 10259 | NS5 | 0.3252 | A:18277;G:8810;C:0;T:4;total:27091 | iSNV |
| F50 | F50-18 | 10295 | NS5 | 0.3275 | A:5;G:2;C:18578;T:9053;total:27638 | iSNV |
| F50 | F50-18 | 10376 | NS5 | 0.5988 | A:14763;G:9897;C:0;T:3;total:24663 | iSNV |
| F50 | F50-18 | 10419 | 3'-UTR | 0.0242 | A:15;G:1;C:19992;T:497;total:20505 | iSNV |
| F50 | F50-18 | 10428 | 3'-UTR | 0.3835 | A:4;G:0;C:12514;T:7787;total:20305 | iSNV |
| F50 | F50-18 | 10451 | 3'-UTR | 0.3066 | A:4;G:2;C:12007;T:5313;total:17326 | iSNV |
| F50 | F50-18 | 10566 | 3'-UTR | 0.3117 | A:2;G:0;C:10549;T:4779;total:15330 | iSNV |
| F50 | F50-19 | 869 | M | 0.1209 | A:2905;G:1;C:15;T:21090;total:24011 | iSNV |
| F50 | F50-19 | 998 | E | 0.6205 | A:6;G:0;C:7395;T:12085;total:19486 | iSNV |
| F50 | F50-19 | 1050 | E | 0.023 | A:6;G:0;C:18654;T:441;total:19101 | iSNV |
| F50 | F50-19 | 1218 | E | 0.9991 | A:0;G:1;C:20;T:21437;total:21458 | SNP |
| F50 | F50-19 | 1363 | E | 0.0856 | A:3;G:2;C:22032;T:2064;total:24101 | iSNV |
| F50 | F50-19 | 1382 | E | 0.1498 | A:2;G:0;C:21516;T:3792;total:25310 | iSNV |
| F50 | F50-19 | 1428 | E | 0.6106 | A:9521;G:14927;C:1;T:1;total:24450 | iSNV |
| F50 | F50-19 | 1430 | E | 0.2946 | A:7;G:2;C:17312;T:7237;total:24558 | iSNV |
| F50 | F50-19 | 1568 | E | 0.0288 | A:5;G:1;C:16226;T:483;total:16715 | iSNV |
| F50 | F50-19 | 1672 | E | 0.0208 | A:4;G:0;C:16307;T:347;total:16658 | iSNV |
| F50 | F50-19 | 1797 | E | 0.161 | A:4048;G:1230;C:8;T:19856;total:25142 | iSNV |
| F50 | F50-19 | 2183 | E | 0.0568 | A:1209;G:20056;C:4;T:0;total:21269 | iSNV |
| F50 | F50-19 | 2318 | E | 0.0486 | A:0;G:4;C:921;T:18004;total:18929 | iSNV |
| F50 | F50-19 | 3052 | NS1 | 0.0219 | A:1;G:4;C:421;T:18747;total:19173 | iSNV |
| F50 | F50-19 | 3663 | NS1 | 0.0205 | A:3;G:0;C:15794;T:331;total:16128 | iSNV |
| F50 | F50-19 | 3869 | NS2A | 0.371 | A:5;G:0;C:15028;T:8870;total:23903 | iSNV |
| F50 | F50-19 | 4122 | NS2A | 0.0585 | A:2;G:12317;C:0;T:766;total:13085 | iSNV |
| F50 | F50-19 | 4538 | NS2B | 0.1212 | A:1;G:0;C:20101;T:2773;total:22875 | iSNV |
| F50 | F50-19 | 4697 | NS3 | 0.6153 | A:7;G:3;C:16697;T:10450;total:27157 | iSNV |
| F50 | F50-19 | 4974 | NS3 | 0.0685 | A:1833;G:24907;C:1;T:1;total:26742 | iSNV |
| F50 | F50-19 | 5261 | NS3 | 0.0624 | A:19046;G:1269;C:0;T:2;total:20317 | iSNV |
| F50 | F50-19 | 5313 | NS3 | 0.6244 | A:4;G:2;C:12626;T:7601;total:20233 | iSNV |
| F50 | F50-19 | 5336 | NS3 | 0.0578 | A:0;G:1;C:1225;T:19960;total:21186 | iSNV |
| F50 | F50-19 | 5549 | NS3 | 0.0263 | A:1;G:4;C:764;T:28206;total:28975 | iSNV |
| F50 | F50-19 | 5598 | NS3 | 0.1333 | A:3416;G:22184;C:5;T:3;total:25608 | iSNV |
| F50 | F50-19 | 5952 | NS3 | 0.6167 | A:2;G:2;C:11554;T:18581;total:30139 | iSNV |
| F50 | F50-19 | 6321 | NS3 | 0.0289 | A:584;G:19577;C:2;T:1;total:20164 | iSNV |
| F50 | F50-19 | 6347 | NS3 | 0.0234 | A:16088;G:387;C:5;T:1;total:16481 | iSNV |
| F50 | F50-19 | 6413 | NS3 | 0.0641 | A:0;G:3;C:1420;T:20724;total:22147 | iSNV |
| F50 | F50-19 | 6779 | NS4A | 0.0218 | A:22131;G:494;C:1;T:3;total:22629 | iSNV |
| F50 | F50-19 | 6867 | NS4A | 0.1135 | A:2264;G:17670;C:0;T:4;total:19938 | iSNV |
| F50 | F50-19 | 6900 | NS4A | 0.0885 | A:1682;G:17310;C:1;T:1;total:18994 | iSNV |
| F50 | F50-19 | 7364 | NS4B | 0.0217 | A:0;G:0;C:345;T:15517;total:15862 | iSNV |
| F50 | F50-19 | 7378 | NS4B | 0.0441 | A:3;G:0;C:15856;T:733;total:16592 | iSNV |
| F50 | F50-19 | 7585 | NS4B | 0.5423 | A:3;G:9717;C:11507;T:3;total:21230 | iSNV |
| F50 | F50-19 | 7633 | NS4B | 0.3611 | A:5;G:1;C:15648;T:8848;total:24502 | iSNV |
| F50 | F50-19 | 8756 | NS5 | 0.0339 | A:18498;G:651;C:0;T:0;total:19149 | iSNV |
| F50 | F50-19 | 9290 | NS5 | 0.0246 | A:3;G:0;C:27876;T:706;total:28585 | iSNV |
| F50 | F50-19 | 9359 | NS5 | 0.614 | A:5;G:2;C:20644;T:12986;total:33637 | iSNV |
| F50 | F50-19 | 9688 | NS5 | 0.054 | A:7;G:0;C:18323;T:1048;total:19378 | iSNV |
| F50 | F50-19 | 9818 | NS5 | 0.0582 | A:0;G:2;C:1226;T:19804;total:21032 | iSNV |
| F50 | F50-19 | 10058 | NS5 | 0.0443 | A:5;G:2;C:19218;T:892;total:20117 | iSNV |
| F50 | F50-19 | 10249 | NS5 | 0.0656 | A:7;G:0;C:25084;T:1762;total:26853 | iSNV |
| F50 | F50-19 | 10259 | NS5 | 0.6057 | A:10780;G:16551;C:0;T:4;total:27335 | iSNV |
| F50 | F50-19 | 10310 | NS5 | 0.0228 | A:9;G:2;C:26983;T:632;total:27626 | iSNV |
| F50 | F50-19 | 10358 | NS5 | 0.1439 | A:8;G:0;C:20664;T:3476;total:24148 | iSNV |
| F50 | F50-19 | 10419 | 3'-UTR | 0.0215 | A:2;G:0;C:20104;T:443;total:20549 | iSNV |
| F50 | F50-19 | 10428 | 3'-UTR | 0.8777 | A:1;G:1;C:2458;T:17628;total:20088 | iSNV |
| F50 | F50-19 | 10566 | 3'-UTR | 0.5956 | A:1;G:4;C:6002;T:8834;total:14841 | iSNV |
| F50 | F50-2 | 446 | C | 0.0217 | A:3;G:0;C:10663;T:237;total:10903 | iSNV |
| F50 | F50-2 | 563 | M | 0.9913 | A:8198;G:72;C:0;T:0;total:8270 | SNP |
| F50 | F50-2 | 1257 | E | 0.9919 | A:8806;G:0;C:72;T:0;total:8878 | SNP |
| F50 | F50-2 | 1447 | E | 0.3533 | A:3429;G:0;C:6272;T:3;total:9704 | iSNV |
| F50 | F50-2 | 1514 | E | 0.0206 | A:7921;G:1;C:168;T:49;total:8139 | iSNV |
| F50 | F50-2 | 1772 | E | 0.9913 | A:2;G:8659;C:4;T:70;total:8735 | SNP |
| F50 | F50-2 | 1911 | E | 0.0201 | A:5920;G:122;C:1;T:3;total:6046 | iSNV |
| F50 | F50-2 | 2372 | E | 0.9788 | A:0;G:1;C:125;T:5745;total:5871 | iSNV |
| F50 | F50-2 | 2493 | NS1 | 0.9937 | A:42;G:6526;C:0;T:0;total:6568 | SNP |
| F50 | F50-2 | 2504 | NS1 | 0.993 | A:43;G:6214;C:0;T:1;total:6258 | SNP |
| F50 | F50-2 | 2531 | NS1 | 0.9918 | A:3;G:0;C:6727;T:53;total:6783 | SNP |
| F50 | F50-2 | 3572 | NS1 | 0.989 | A:1;G:0;C:71;T:6439;total:6511 | SNP |
| F50 | F50-2 | 3962 | NS2A | 0.9913 | A:2;G:0;C:81;T:9401;total:9484 | SNP |
| F50 | F50-2 | 4155 | NS2A | 0.9937 | A:4078;G:1;C:0;T:25;total:4104 | SNP |

| | | | | | | |
|---|---|---|---|---|---|---|
| F50 | F50-2 | 4712 | NS3 | 0.9934 | A:11158;G:74;C:0;T:1;total:11233 | SNP |
| F50 | F50-2 | 5311 | NS3 | 0.9912 | A:0;G:1;C:61;T:6907;total:6969 | SNP |
| F50 | F50-2 | 5537 | NS3 | 0.0468 | A:9862;G:485;C:0;T:0;total:10347 | iSNV |
| F50 | F50-2 | 5574 | NS3 | 0.0201 | A:9181;G:189;C:0;T:0;total:9370 | iSNV |
| F50 | F50-2 | 6080 | NS3 | 0.9925 | A:8069;G:60;C:0;T:1;total:8130 | SNP |
| F50 | F50-2 | 8130 | NS5 | 0.0222 | A:2;G:0;C:10560;T:240;total:10802 | iSNV |
| F50 | F50-2 | 8282 | NS5 | 0.9935 | A:2;G:0;C:9621;T:61;total:9684 | SNP |
| F50 | F50-2 | 8900 | NS5 | 0.9933 | A:52;G:1;C:8002;T:1;total:8056 | SNP |
| F50 | F50-2 | 9008 | NS5 | 0.0629 | A:0;G:1;C:533;T:7937;total:8471 | iSNV |
| F50 | F50-2 | 9446 | NS5 | 0.9925 | A:92;G:12380;C:0;T:2;total:12474 | SNP |
| F50 | F50-2 | 10419 | 3'-UTR | 0.0234 | A:7;G:0;C:8245;T:198;total:8450 | iSNV |
| F50 | F50-2 | 10428 | 3'-UTR | 0.0529 | A:4;G:0;C:8106;T:453;total:8563 | iSNV |
| F50 | F50-20 | 614 | M | 0.1114 | A:2;G:14659;C:1;T:1839;total:16501 | iSNV |
| F50 | F50-20 | 906 | M | 0.1188 | A:10810;G:1459;C:1;T:1;total:12271 | iSNV |
| F50 | F50-20 | 998 | E | 0.0292 | A:2;G:0;C:12849;T:387;total:13238 | iSNV |
| F50 | F50-20 | 1088 | E | 0.027 | A:5;G:2;C:12742;T:355;total:13104 | iSNV |
| F50 | F50-20 | 1117 | E | 0.9487 | A:765;G:14128;C:3;T:2;total:14898 | iSNV |
| F50 | F50-20 | 1218 | E | 0.9974 | A:1;G:3;C:36;T:15001;total:15041 | SNP |
| F50 | F50-20 | 1295 | E | 0.0409 | A:17172;G:14;C:1;T:734;total:17921 | iSNV |
| F50 | F50-20 | 1348 | E | 0.0767 | A:17566;G:1;C:1460;T:0;total:19027 | iSNV |
| F50 | F50-20 | 1459 | E | 0.1407 | A:2572;G:1;C:15683;T:22;total:18278 | iSNV |
| F50 | F50-20 | 1571 | E | 0.0578 | A:1;G:0;C:714;T:11627;total:12342 | iSNV |
| F50 | F50-20 | 1586 | E | 0.0377 | A:3;G:0;C:11726;T:460;total:12189 | iSNV |
| F50 | F50-20 | 1721 | E | 0.9199 | A:1075;G:12343;C:0;T:0;total:13418 | iSNV |
| F50 | F50-20 | 1793 | E | 0.0288 | A:538;G:18117;C:0;T:2;total:18657 | iSNV |
| F50 | F50-20 | 1909 | E | 0.1435 | A:2124;G:3;C:12667;T:2;total:14796 | iSNV |
| F50 | F50-20 | 2369 | E | 0.9185 | A:8964;G:796;C:1;T:1;total:9762 | iSNV |
| F50 | F50-20 | 2531 | NS1 | 0.1961 | A:6;G:1;C:2644;T:10829;total:13480 | iSNV |
| F50 | F50-20 | 3002 | NS1 | 0.0357 | A:1;G:1;C:16687;T:618;total:17307 | iSNV |
| F50 | F50-20 | 3564 | NS1 | 0.0527 | A:2;G:0;C:12696;T:707;total:13405 | iSNV |
| F50 | F50-20 | 3593 | NS1 | 0.0807 | A:1;G:0;C:12918;T:1135;total:14054 | iSNV |
| F50 | F50-20 | 3869 | NS2A | 0.9691 | A:0;G:1;C:537;T:16826;total:17364 | iSNV |
| F50 | F50-20 | 3923 | NS2A | 0.0492 | A:0;G:1;C:986;T:19013;total:20000 | iSNV |
| F50 | F50-20 | 3929 | NS2A | 0.9227 | A:24;G:2;C:1537;T:18313;total:19876 | iSNV |
| F50 | F50-20 | 4043 | NS2A | 0.0758 | A:1;G:1;C:12289;T:1009;total:13300 | iSNV |
| F50 | F50-20 | 4627 | NS3 | 0.036 | A:3;G:1;C:21330;T:798;total:22132 | iSNV |
| F50 | F50-20 | 4697 | NS3 | 0.0265 | A:1;G:2;C:565;T:20690;total:21258 | iSNV |
| F50 | F50-20 | 4940 | NS3 | 0.0433 | A:18241;G:826;C:5;T:0;total:19072 | iSNV |
| F50 | F50-20 | 5358 | NS3 | 0.0409 | A:707;G:16543;C:0;T:1;total:17251 | iSNV |
| F50 | F50-20 | 5376 | NS3 | 0.0399 | A:15594;G:3;C:2;T:649;total:16248 | iSNV |
| F50 | F50-20 | 5441 | NS3 | 0.1353 | A:3;G:0;C:18570;T:2907;total:21480 | iSNV |
| F50 | F50-20 | 5546 | NS3 | 0.02 | A:7;G:0;C:20908;T:429;total:21344 | iSNV |
| F50 | F50-20 | 5952 | NS3 | 0.0246 | A:5;G:0;C:21872;T:553;total:22430 | iSNV |
| F50 | F50-20 | 6059 | NS3 | 0.07 | A:1;G:3;C:1339;T:17770;total:19113 | iSNV |
| F50 | F50-20 | 6867 | NS4A | 0.1058 | A:1496;G:12638;C:0;T:1;total:14135 | iSNV |
| F50 | F50-20 | 6971 | NS4A | 0.0518 | A:10;G:13601;C:745;T:14;total:14370 | iSNV |
| F50 | F50-20 | 7526 | NS4B | 0.03 | A:0;G:2;C:379;T:12245;total:12626 | iSNV |
| F50 | F50-20 | 7633 | NS4B | 0.8892 | A:0;G:1;C:2037;T:16340;total:18378 | iSNV |
| F50 | F50-20 | 9359 | NS5 | 0.0277 | A:0;G:3;C:674;T:23632;total:24309 | iSNV |
| F50 | F50-20 | 9622 | NS5 | 0.0205 | A:314;G:14928;C:0;T:1;total:15243 | iSNV |
| F50 | F50-20 | 10109 | NS5 | 0.1968 | A:3356;G:13691;C:0;T:1;total:17048 | iSNV |
| F50 | F50-20 | 10259 | NS5 | 0.0249 | A:20849;G:533;C:1;T:0;total:21383 | iSNV |
| F50 | F50-20 | 10418 | 3'-UTR | 0.055 | A:14773;G:861;C:2;T:1;total:15637 | iSNV |
| F50 | F50-20 | 10428 | 3'-UTR | 0.6358 | A:1;G:2;C:5598;T:9769;total:15370 | iSNV |
| F50 | F50-20 | 10447 | 3'-UTR | 0.023 | A:6;G:0;C:13210;T:312;total:13528 | iSNV |
| F50 | F50-20 | 10566 | 3'-UTR | 0.0246 | A:2;G:0;C:11422;T:289;total:11713 | iSNV |
| F50 | F50-21 | 443 | C | 0.2107 | A:3;G:15483;C:0;T:4136;total:19622 | iSNV |
| F50 | F50-21 | 815 | M | 0.0221 | A:0;G:1;C:273;T:12027;total:12301 | iSNV |
| F50 | F50-21 | 1031 | E | 0.0335 | A:3;G:2;C:11748;T:408;total:12161 | iSNV |
| F50 | F50-21 | 1218 | E | 0.9985 | A:0;G:0;C:20;T:13132;total:13152 | SNP |
| F50 | F50-21 | 1363 | E | 0.1534 | A:1;G:1;C:13041;T:2365;total:15408 | iSNV |
| F50 | F50-21 | 1459 | E | 0.2097 | A:3242;G:3;C:12210;T:5;total:15460 | iSNV |
| F50 | F50-21 | 1797 | E | 0.7258 | A:199;G:10938;C:0;T:4209;total:15346 | iSNV |
| F50 | F50-21 | 2362 | E | 0.1668 | A:1;G:6454;C:1294;T:6;total:7755 | iSNV |
| F50 | F50-21 | 2393 | E | 0.1972 | A:2;G:0;C:6959;T:1710;total:8671 | iSNV |
| F50 | F50-21 | 2556 | NS1 | 0.7057 | A:7120;G:2971;C:2;T:0;total:10093 | iSNV |
| F50 | F50-21 | 2876 | NS1 | 0.0729 | A:0;G:0;C:1627;T:20681;total:22308 | iSNV |
| F50 | F50-21 | 3230 | NS1 | 0.0533 | A:22573;G:1273;C:1;T:0;total:23847 | iSNV |
| F50 | F50-21 | 3378 | NS1 | 0.0224 | A:13465;G:309;C:0;T:0;total:13774 | iSNV |
| F50 | F50-21 | 3404 | NS1 | 0.0214 | A:0;G:0;C:322;T:14692;total:15014 | iSNV |
| F50 | F50-21 | 3728 | NS2A | 0.0324 | A:1;G:0;C:8884;T:298;total:9183 | iSNV |
| F50 | F50-21 | 3776 | NS2A | 0.0747 | A:862;G:1;C:9;T:10657;total:11529 | iSNV |
| F50 | F50-21 | 3869 | NS2A | 0.9975 | A:0;G:0;C:42;T:16530;total:16572 | SNP |
| F50 | F50-21 | 4070 | NS2A | 0.1323 | A:0;G:1655;C:1;T:10846;total:12502 | iSNV |
| F50 | F50-21 | 4688 | NS3 | 0.0285 | A:8;G:3;C:562;T:19103;total:19676 | iSNV |
| F50 | F50-21 | 5428 | NS3 | 0.0249 | A:19458;G:498;C:2;T:0;total:19958 | iSNV |
| F50 | F50-21 | 6004 | NS3 | 0.0922 | A:2;G:0;C:17829;T:1811;total:19642 | iSNV |
| F50 | F50-21 | 6029 | NS3 | 0.0317 | A:1;G:0;C:18726;T:614;total:19341 | iSNV |
| F50 | F50-21 | 6089 | NS3 | 0.1974 | A:1;G:0;C:12794;T:3148;total:15943 | iSNV |
| F50 | F50-21 | 6107 | NS3 | 0.402 | A:2;G:1;C:10074;T:6775;total:16852 | iSNV |
| F50 | F50-21 | 6336 | NS3 | 0.0557 | A:9537;G:1;C:6;T:563;total:10107 | iSNV |
| F50 | F50-21 | 6969 | NS4A | 0.0315 | A:12773;G:416;C:1;T:0;total:13190 | iSNV |

| | | | | | | |
|---|---|---|---|---|---|---|
| F50 | F50-21 | 7561 | NS4B | 0.0483 | A:0;G:4;C:731;T:14393;total:15128 | iSNV |
| F50 | F50-21 | 7595 | NS4B | 0.034 | A:529;G:0;C:0;T:15004;total:15533 | iSNV |
| F50 | F50-21 | 7633 | NS4B | 0.3042 | A:0;G:1;C:12034;T:5263;total:17298 | iSNV |
| F50 | F50-21 | 7644 | NS4B | 0.3878 | A:10390;G:2;C:6586;T:1;total:16979 | iSNV |
| F50 | F50-21 | 8237 | NS5 | 0.0663 | A:4;G:0;C:17228;T:1225;total:18457 | iSNV |
| F50 | F50-21 | 8618 | NS5 | 0.0481 | A:728;G:14382;C:0;T:1;total:15111 | iSNV |
| F50 | F50-21 | 8738 | NS5 | 0.0706 | A:13988;G:1064;C:1;T:0;total:15053 | iSNV |
| F50 | F50-21 | 8909 | NS5 | 0.0337 | A:0;G:2;C:561;T:16042;total:16605 | iSNV |
| F50 | F50-21 | 9038 | NS5 | 0.0537 | A:2;G:1;C:16504;T:937;total:17444 | iSNV |
| F50 | F50-21 | 9050 | NS5 | 0.1313 | A:13912;G:2103;C:0;T:0;total:16015 | iSNV |
| F50 | F50-21 | 9065 | NS5 | 0.0988 | A:1454;G:13253;C:2;T:2;total:14711 | iSNV |
| F50 | F50-21 | 9370 | NS5 | 0.0216 | A:534;G:3;C:40;T:24057;total:24634 | iSNV |
| F50 | F50-21 | 9542 | NS5 | 0.034 | A:2;G:0;C:17890;T:630;total:18522 | iSNV |
| F50 | F50-21 | 9688 | NS5 | 0.0394 | A:2;G:0;C:12832;T:527;total:13361 | iSNV |
| F50 | F50-21 | 10334 | NS5 | 0.0922 | A:2;G:2;C:19301;T:1961;total:21266 | iSNV |
| F50 | F50-21 | 10358 | NS5 | 0.6366 | A:2;G:0;C:6877;T:12041;total:18920 | iSNV |
| F50 | F50-21 | 10428 | 3'-UTR | 0.8105 | A:0;G:0;C:2987;T:12769;total:15756 | iSNV |
| F50 | F50-21 | 10451 | 3'-UTR | 0.0207 | A:1;G:0;C:14043;T:297;total:14341 | iSNV |
| F50 | F50-21 | 10470 | 3'-UTR | 0.2033 | A:10594;G:2705;C:0;T:1;total:13300 | iSNV |
| F50 | F50-21 | 10513 | 3'-UTR | 0.2104 | A:0;G:2;C:3014;T:11304;total:14320 | iSNV |
| F50 | F50-21 | 10578 | 3'-UTR | 0.0952 | A:0;G:2;C:1149;T:10907;total:12058 | iSNV |
| F50 | F50-22 | 347 | C | 0.4712 | A:7;G:1;C:15986;T:14252;total:30246 | iSNV |
| F50 | F50-22 | 998 | E | 0.9412 | A:1187;G:2;C:186;T:18796;total:20171 | iSNV |
| F50 | F50-22 | 1218 | E | 0.9986 | A:1;G:1;C:32;T:22783;total:22817 | SNP |
| F50 | F50-22 | 1428 | E | 0.9772 | A:604;G:25837;C:2;T:2;total:26445 | iSNV |
| F50 | F50-22 | 4244 | NS2B | 0.0498 | A:14251;G:0;C:3;T:748;total:15002 | iSNV |
| F50 | F50-22 | 4468 | NS2B | 0.9751 | A:17854;G:2;C:3;T:457;total:18316 | iSNV |
| F50 | F50-22 | 4697 | NS3 | 0.9898 | A:18;G:0;C:28475;T:276;total:28769 | SNP |
| F50 | F50-22 | 5366 | NS3 | 0.0845 | A:21257;G:9;C:1964;T:1;total:23231 | iSNV |
| F50 | F50-22 | 5414 | NS3 | 0.0254 | A:27911;G:729;C:3;T:1;total:28644 | iSNV |
| F50 | F50-22 | 5871 | NS3 | 0.0251 | A:30112;G:7;C:778;T:1;total:30898 | iSNV |
| F50 | F50-22 | 5952 | NS3 | 0.9909 | A:1;G:2;C:290;T:31817;total:32110 | SNP |
| F50 | F50-22 | 6867 | NS4A | 0.7799 | A:16111;G:4549;C:4;T:1;total:20665 | iSNV |
| F50 | F50-22 | 7211 | NS4A | 0.0521 | A:826;G:0;C:1;T:15025;total:15852 | iSNV |
| F50 | F50-22 | 7532 | NS4B | 0.0448 | A:0;G:1;C:815;T:17355;total:18171 | iSNV |
| F50 | F50-22 | 7633 | NS4B | 0.9383 | A:1;G:3;C:1583;T:24058;total:25645 | iSNV |
| F50 | F50-22 | 7644 | NS4B | 0.0359 | A:25698;G:1;C:959;T:0;total:26658 | iSNV |
| F50 | F50-22 | 9245 | NS5 | 0.9741 | A:5;G:11;C:929;T:34884;total:35829 | iSNV |
| F50 | F50-22 | 9359 | NS5 | 0.989 | A:11;G:0;C:37634;T:408;total:38053 | SNP |
| F50 | F50-22 | 10259 | NS5 | 0.9906 | A:293;G:31326;C:0;T:7;total:31626 | SNP |
| F50 | F50-22 | 10401 | 3'-UTR | 0.0312 | A:1;G:9;C:813;T:25177;total:26000 | iSNV |
| F50 | F50-22 | 10428 | 3'-UTR | 0.2612 | A:3;G:1;C:18803;T:6652;total:25459 | iSNV |
| F50 | F50-22 | 10447 | 3'-UTR | 0.0372 | A:5;G:0;C:21426;T:829;total:22260 | iSNV |
| F50 | F50-22 | 10566 | 3'-UTR | 0.9897 | A:0;G:1;C:170;T:16293;total:16464 | SNP |
| F50 | F50-23 | 353 | C | 0.6955 | A:6547;G:14948;C:1;T:3;total:21499 | iSNV |
| F50 | F50-23 | 454 | C | 0.03 | A:1;G:2;C:622;T:20088;total:20713 | iSNV |
| F50 | F50-23 | 645 | M | 0.721 | A:5099;G:13171;C:2;T:2;total:18274 | iSNV |
| F50 | F50-23 | 803 | M | 0.03 | A:9;G:15406;C:0;T:478;total:15893 | iSNV |
| F50 | F50-23 | 998 | E | 0.7582 | A:1;G:3;C:3972;T:12450;total:16426 | iSNV |
| F50 | F50-23 | 1057 | E | 0.0409 | A:2;G:15510;C:663;T:3;total:16178 | iSNV |
| F50 | F50-23 | 1117 | E | 0.766 | A:4299;G:14070;C:0;T:1;total:18370 | iSNV |
| F50 | F50-23 | 1218 | E | 0.2204 | A:2;G:0;C:14405;T:4075;total:18482 | iSNV |
| F50 | F50-23 | 1296 | E | 0.029 | A:3;G:2;C:586;T:19579;total:20170 | iSNV |
| F50 | F50-23 | 1512 | E | 0.7243 | A:5415;G:14220;C:0;T:0;total:19635 | iSNV |
| F50 | F50-23 | 1797 | E | 0.1922 | A:0;G:4215;C:14;T:17695;total:21924 | iSNV |
| F50 | F50-23 | 2075 | E | 0.0212 | A:4;G:2;C:14254;T:310;total:14570 | iSNV |
| F50 | F50-23 | 2303 | E | 0.5356 | A:0;G:3;C:7183;T:8278;total:15464 | iSNV |
| F50 | F50-23 | 2408 | E | 0.0207 | A:2;G:4;C:12455;T:264;total:12725 | iSNV |
| F50 | F50-23 | 2460 | E | 0.0222 | A:300;G:13177;C:0;T:4;total:13481 | iSNV |
| F50 | F50-23 | 2808 | NS1 | 0.0284 | A:4;G:12;C:683;T:23326;total:24025 | iSNV |
| F50 | F50-23 | 3869 | NS2A | 0.211 | A:5;G:0;C:15439;T:4132;total:19576 | iSNV |
| F50 | F50-23 | 4103 | NS2A | 0.0253 | A:374;G:14385;C:0;T:4;total:14763 | iSNV |
| F50 | F50-23 | 4468 | NS2B | 0.037 | A:553;G:3;C:1;T:14387;total:14944 | iSNV |
| F50 | F50-23 | 4697 | NS3 | 0.0438 | A:0;G:2;C:989;T:21581;total:22572 | iSNV |
| F50 | F50-23 | 4974 | NS3 | 0.1251 | A:2675;G:18702;C:2;T:1;total:21380 | iSNV |
| F50 | F50-23 | 5339 | NS3 | 0.0293 | A:17079;G:516;C:0;T:1;total:17596 | iSNV |
| F50 | F50-23 | 5826 | NS3 | 0.1956 | A:18006;G:4381;C:1;T:1;total:22389 | iSNV |
| F50 | F50-23 | 5952 | NS3 | 0.0459 | A:7;G:2;C:20863;T:1006;total:21878 | iSNV |
| F50 | F50-23 | 6471 | NS4A | 0.0272 | A:458;G:16370;C:1;T:2;total:16831 | iSNV |
| F50 | F50-23 | 6867 | NS4A | 0.5354 | A:9587;G:8324;C:1;T:2;total:17914 | iSNV |
| F50 | F50-23 | 6900 | NS4A | 0.2026 | A:18;G:13480;C:0;T:3430;total:16928 | iSNV |
| F50 | F50-23 | 7172 | NS4A | 0.0238 | A:1;G:3;C:369;T:15084;total:15457 | iSNV |
| F50 | F50-23 | 7182 | NS4A | 0.0912 | A:1395;G:1;C:13888;T:8;total:15292 | iSNV |
| F50 | F50-23 | 7264 | NS4A | 0.0254 | A:3;G:0;C:13648;T:357;total:14008 | iSNV |
| F50 | F50-23 | 7633 | NS4B | 0.9926 | A:0;G:1;C:135;T:18222;total:18358 | SNP |
| F50 | F50-23 | 7735 | NS5 | 0.0265 | A:438;G:16064;C:3;T:4;total:16509 | iSNV |
| F50 | F50-23 | 8518 | NS5 | 0.03 | A:23819;G:3;C:2;T:738;total:24562 | iSNV |
| F50 | F50-23 | 8720 | NS5 | 0.0256 | A:6;G:0;C:17032;T:448;total:17486 | iSNV |
| F50 | F50-23 | 8844 | NS5 | 0.0318 | A:500;G:15205;C:0;T:1;total:15706 | iSNV |
| F50 | F50-23 | 9359 | NS5 | 0.0479 | A:2;G:1;C:1125;T:22313;total:23441 | iSNV |
| F50 | F50-23 | 9500 | NS5 | 0.0268 | A:3;G:0;C:23225;T:640;total:23868 | iSNV |
| F50 | F50-23 | 9605 | NS5 | 0.0277 | A:21541;G:615;C:2;T:1;total:22159 | iSNV |

| | | | | | | |
|---|---|---|---|---|---|---|
| F50 | F50-23 | 9645 | NS5 | 0.0444 | A:890;G:19118;C:0;T:1;total:20009 | iSNV |
| F50 | F50-23 | 9722 | NS5 | 0.0264 | A:13;G:2;C:20267;T:552;total:20834 | iSNV |
| F50 | F50-23 | 10259 | NS5 | 0.7504 | A:6154;G:18492;C:0;T:3;total:24649 | iSNV |
| F50 | F50-23 | 10419 | 3'-UTR | 0.0208 | A:6;G:2;C:19408;T:413;total:19829 | iSNV |
| F50 | F50-23 | 10428 | 3'-UTR | 0.4581 | A:2;G:2;C:10707;T:9055;total:19766 | iSNV |
| F50 | F50-23 | 10447 | 3'-UTR | 0.7117 | A:4;G:5;C:4999;T:12326;total:17334 | iSNV |
| F50 | F50-23 | 10566 | 3'-UTR | 0.0434 | A:2;G:0;C:15861;T:720;total:16583 | iSNV |
| F50 | F50-23 | 10804 | 3'-UTR | 0.1995 | A:1;G:0;C:11423;T:2848;total:14272 | iSNV |
| F50 | F50-24 | 501 | M | 0.0259 | A:4843;G:0;C:129;T:0;total:4972 | iSNV |
| F50 | F50-24 | 898 | M | 0.3804 | A:1;G:0;C:944;T:1536;total:2481 | iSNV |
| F50 | F50-24 | 1067 | E | 0.0281 | A:0;G:1;C:2833;T:82;total:2916 | iSNV |
| F50 | F50-24 | 1193 | E | 0.0264 | A:1;G:0;C:96;T:3531;total:3628 | iSNV |
| F50 | F50-24 | 1218 | E | 0.9975 | A:0;G:0;C:8;T:3092;total:3100 | SNP |
| F50 | F50-24 | 1512 | E | 0.0296 | A:3096;G:12;C:95;T:0;total:3203 | iSNV |
| F50 | F50-24 | 1598 | E | 0.1124 | A:258;G:2037;C:0;T:0;total:2295 | iSNV |
| F50 | F50-24 | 2076 | E | 0.9609 | A:87;G:2137;C:0;T:0;total:2224 | iSNV |
| F50 | F50-24 | 2771 | NS1 | 0.0396 | A:0;G:0;C:4332;T:179;total:4511 | iSNV |
| F50 | F50-24 | 2870 | NS1 | 0.4106 | A:0;G:0;C:3117;T:2172;total:5289 | iSNV |
| F50 | F50-24 | 2890 | NS1 | 0.9611 | A:4366;G:0;C:177;T:0;total:4543 | iSNV |
| F50 | F50-24 | 3041 | NS1 | 0.0202 | A:0;G:0;C:2757;T:57;total:2814 | iSNV |
| F50 | F50-24 | 3261 | NS1 | 0.1638 | A:3623;G:710;C:0;T:0;total:4333 | iSNV |
| F50 | F50-24 | 3608 | NS1 | 0.2981 | A:0;G:0;C:2199;T:934;total:3133 | iSNV |
| F50 | F50-24 | 3669 | NS1 | 0.9565 | A:0;G:2;C:76;T:1667;total:1745 | iSNV |
| F50 | F50-24 | 3869 | NS2A | 0.9879 | A:0;G:0;C:48;T:3897;total:3945 | SNP |
| F50 | F50-24 | 4070 | NS2A | 0.0452 | A:0;G:0;C:118;T:2491;total:2609 | iSNV |
| F50 | F50-24 | 4286 | NS2B | 0.4466 | A:1377;G:1706;C:0;T:0;total:3083 | iSNV |
| F50 | F50-24 | 4850 | NS3 | 0.03 | A:2;G:142;C:4584;T:1;total:4729 | iSNV |
| F50 | F50-24 | 4896 | NS3 | 0.0875 | A:441;G:4594;C:0;T:0;total:5035 | iSNV |
| F50 | F50-24 | 5172 | NS3 | 0.1819 | A:2058;G:1;C:0;T:458;total:2517 | iSNV |
| F50 | F50-24 | 5195 | NS3 | 0.2003 | A:0;G:0;C:565;T:2255;total:2820 | iSNV |
| F50 | F50-24 | 5737 | NS3 | 0.9594 | A:177;G:4178;C:0;T:0;total:4355 | iSNV |
| F50 | F50-24 | 5835 | NS3 | 0.0223 | A:100;G:4376;C:0;T:1;total:4477 | iSNV |
| F50 | F50-24 | 6649 | NS4A | 0.0266 | A:0;G:0;C:67;T:2451;total:2518 | iSNV |
| F50 | F50-24 | 6867 | NS4A | 0.2644 | A:716;G:1991;C:0;T:0;total:2707 | iSNV |
| F50 | F50-24 | 6900 | NS4A | 0.0233 | A:12;G:2459;C:0;T:59;total:2530 | iSNV |
| F50 | F50-24 | 6971 | NS4A | 0.3714 | A:0;G:1542;C:949;T:64;total:2555 | iSNV |
| F50 | F50-24 | 7182 | NS4A | 0.2101 | A:466;G:0;C:1751;T:0;total:2217 | iSNV |
| F50 | F50-24 | 7193 | NS4A | 0.4719 | A:0;G:0;C:1033;T:1156;total:2189 | iSNV |
| F50 | F50-24 | 7264 | NS4A | 0.0471 | A:0;G:0;C:1819;T:90;total:1909 | iSNV |
| F50 | F50-24 | 7367 | NS4B | 0.0348 | A:0;G:0;C:2074;T:75;total:2149 | iSNV |
| F50 | F50-24 | 7633 | NS4B | 0.9939 | A:0;G:0;C:22;T:3529;total:3551 | SNP |
| F50 | F50-24 | 9183 | NS5 | 0.0398 | A:190;G:4578;C:0;T:0;total:4768 | iSNV |
| F50 | F50-24 | 9233 | NS5 | 0.7193 | A:0;G:1;C:1410;T:3611;total:5022 | iSNV |
| F50 | F50-24 | 10428 | 3'-UTR | 0.881 | A:0;G:1;C:430;T:3181;total:3612 | iSNV |
| F50 | F50-24 | 10447 | 3'-UTR | 0.0711 | A:2;G:0;C:3051;T:234;total:3287 | iSNV |
| F50 | F50-25 | 815 | M | 0.0382 | A:0;G:3;C:608;T:15272;total:15883 | iSNV |
| F50 | F50-25 | 824 | M | 0.2682 | A:11416;G:4186;C:0;T:0;total:15602 | iSNV |
| F50 | F50-25 | 1083 | E | 0.0202 | A:15315;G:317;C:7;T:3;total:15642 | iSNV |
| F50 | F50-25 | 1117 | E | 0.0941 | A:16580;G:1724;C:1;T:0;total:18305 | iSNV |
| F50 | F50-25 | 1218 | E | 0.9998 | A:0;G:0;C:4;T:17455;total:17459 | SNP |
| F50 | F50-25 | 1428 | E | 0.1569 | A:19849;G:3697;C:1;T:4;total:23551 | iSNV |
| F50 | F50-25 | 1551 | E | 0.3023 | A:1;G:5033;C:11604;T:6;total:16644 | iSNV |
| F50 | F50-25 | 1797 | E | 0.4382 | A:4;G:9518;C:7;T:12187;total:21716 | iSNV |
| F50 | F50-25 | 2600 | NS1 | 0.3212 | A:4;G:1;C:12155;T:5755;total:17915 | iSNV |
| F50 | F50-25 | 2855 | NS1 | 0.0959 | A:2779;G:26190;C:1;T:6;total:28976 | iSNV |
| F50 | F50-25 | 3869 | NS2A | 0.9814 | A:1;G:1;C:369;T:19528;total:19899 | SNP |
| F50 | F50-25 | 4385 | NS2B | 0.0696 | A:1;G:0;C:15806;T:1184;total:16991 | iSNV |
| F50 | F50-25 | 4469 | NS2B | 0.1362 | A:0;G:1;C:1894;T:12002;total:13897 | iSNV |
| F50 | F50-25 | 4646 | NS3 | 0.0824 | A:5;G:0;C:22746;T:2045;total:24796 | iSNV |
| F50 | F50-25 | 4974 | NS3 | 0.1173 | A:2537;G:19088;C:0;T:2;total:21627 | iSNV |
| F50 | F50-25 | 5256 | NS3 | 0.3075 | A:2;G:3;C:12087;T:5371;total:17463 | iSNV |
| F50 | F50-25 | 5558 | NS3 | 0.0969 | A:2168;G:20200;C:1;T:2;total:22371 | iSNV |
| F50 | F50-25 | 5707 | NS3 | 0.0442 | A:23804;G:1101;C:3;T:0;total:24908 | iSNV |
| F50 | F50-25 | 5736 | NS3 | 0.3226 | A:8298;G:4;C:17420;T:0;total:25722 | iSNV |
| F50 | F50-25 | 5968 | NS3 | 0.0942 | A:2274;G:21837;C:2;T:2;total:24115 | iSNV |
| F50 | F50-25 | 6867 | NS4A | 0.0327 | A:516;G:15254;C:0;T:2;total:15772 | iSNV |
| F50 | F50-25 | 6900 | NS4A | 0.3254 | A:4893;G:10139;C:1;T:2;total:15035 | iSNV |
| F50 | F50-25 | 6970 | NS4A | 0.0279 | A:15966;G:6;C:460;T:0;total:16432 | iSNV |
| F50 | F50-25 | 7264 | NS4A | 0.2971 | A:3;G:2;C:7858;T:3325;total:11188 | iSNV |
| F50 | F50-25 | 7316 | NS4B | 0.0227 | A:251;G:10768;C:0;T:1;total:11020 | iSNV |
| F50 | F50-25 | 7526 | NS4B | 0.0318 | A:0;G:3;C:454;T:13809;total:14266 | iSNV |
| F50 | F50-25 | 7561 | NS4B | 0.0209 | A:0;G:3;C:358;T:16730;total:17091 | iSNV |
| F50 | F50-25 | 7633 | NS4B | 0.1571 | A:3;G:0;C:16058;T:2994;total:19055 | iSNV |
| F50 | F50-25 | 7656 | NS4B | 0.3092 | A:5773;G:12896;C:0;T:1;total:18670 | iSNV |
| F50 | F50-25 | 8675 | NS5 | 0.0492 | A:0;G:0;C:693;T:13367;total:14060 | iSNV |
| F50 | F50-25 | 9080 | NS5 | 0.0393 | A:17115;G:701;C:1;T:2;total:17819 | iSNV |
| F50 | F50-25 | 10428 | 3'-UTR | 0.987 | A:0;G:0;C:254;T:19139;total:19393 | SNP |
| F50 | F50-25 | 10632 | 3'-UTR | 0.0429 | A:4;G:1;C:680;T:15131;total:15816 | iSNV |
| F50 | F50-26 | 168 | C | 0.0432 | A:0;G:2;C:1591;T:35160;total:36753 | iSNV |
| F50 | F50-26 | 470 | C | 0.0494 | A:31109;G:23;C:7;T:1620;total:32759 | iSNV |
| F50 | F50-26 | 542 | M | 0.0369 | A:0;G:0;C:927;T:24165;total:25092 | iSNV |
| F50 | F50-26 | 648 | M | 0.0245 | A:23319;G:3;C:4;T:587;total:23913 | iSNV |

| | | | | | | |
|---|---|---|---|---|---|---|
| F50 | F50-26 | 694 | M | 0.1474 | A:21707;G:3755;C:1;T:1;total:25464 | iSNV |
| F50 | F50-26 | 1218 | E | 0.999 | A:0;G:0;C:21;T:19639;total:19660 | SNP |
| F50 | F50-26 | 1263 | E | 0.0478 | A:0;G:8;C:989;T:19687;total:20684 | iSNV |
| F50 | F50-26 | 1428 | E | 0.6125 | A:9378;G:14816;C:1;T:1;total:24196 | iSNV |
| F50 | F50-26 | 1514 | E | 0.0497 | A:19154;G:0;C:1003;T:0;total:20157 | iSNV |
| F50 | F50-26 | 1797 | E | 0.306 | A:128;G:7249;C:2;T:16303;total:23682 | iSNV |
| F50 | F50-26 | 3344 | NS1 | 0.062 | A:5;G:23102;C:0;T:1529;total:24636 | iSNV |
| F50 | F50-26 | 3536 | NS1 | 0.1983 | A:4;G:0;C:12969;T:3210;total:16183 | iSNV |
| F50 | F50-26 | 3869 | NS2A | 0.9985 | A:1;G:2;C:35;T:24043;total:24081 | SNP |
| F50 | F50-26 | 4922 | NS3 | 0.0477 | A:8;G:14;C:27177;T:1365;total:28564 | iSNV |
| F50 | F50-26 | 5365 | NS3 | 0.0322 | A:20478;G:682;C:2;T:1;total:21163 | iSNV |
| F50 | F50-26 | 5616 | NS3 | 0.0537 | A:1443;G:0;C:25375;T:8;total:26826 | iSNV |
| F50 | F50-26 | 5781 | NS3 | 0.0416 | A:1326;G:30526;C:0;T:1;total:31853 | iSNV |
| F50 | F50-26 | 6080 | NS3 | 0.0775 | A:1753;G:20850;C:1;T:1;total:22605 | iSNV |
| F50 | F50-26 | 6423 | NS3 | 0.2715 | A:5365;G:0;C:14394;T:1;total:19760 | iSNV |
| F50 | F50-26 | 6714 | NS4A | 0.2669 | A:5584;G:15335;C:1;T:0;total:20920 | iSNV |
| F50 | F50-26 | 6770 | NS4A | 0.0443 | A:13;G:2;C:20672;T:960;total:21647 | iSNV |
| F50 | F50-26 | 6806 | NS4A | 0.0443 | A:0;G:22821;C:0;T:1058;total:23879 | iSNV |
| F50 | F50-26 | 6947 | NS4A | 0.2124 | A:1;G:5;C:3870;T:14342;total:18218 | iSNV |
| F50 | F50-26 | 6970 | NS4A | 0.1144 | A:16200;G:3;C:2094;T:0;total:18297 | iSNV |
| F50 | F50-26 | 7244 | NS4A | 0.4182 | A:0;G:0;C:5039;T:7008;total:12047 | iSNV |
| F50 | F50-26 | 7561 | NS4B | 0.2063 | A:1;G:8;C:4099;T:15760;total:19868 | iSNV |
| F50 | F50-26 | 7633 | NS4B | 0.7818 | A:0;G:4;C:4964;T:17778;total:22746 | iSNV |
| F50 | F50-26 | 7982 | NS5 | 0.073 | A:29602;G:330;C:6;T:2358;total:32296 | iSNV |
| F50 | F50-26 | 8103 | NS5 | 0.0327 | A:6;G:3;C:26998;T:915;total:27922 | iSNV |
| F50 | F50-26 | 8471 | NS5 | 0.0639 | A:28450;G:1943;C:4;T:0;total:30397 | iSNV |
| F50 | F50-26 | 8666 | NS5 | 0.0725 | A:1473;G:18837;C:0;T:5;total:20315 | iSNV |
| F50 | F50-26 | 8840 | NS5 | 0.0376 | A:19536;G:764;C:1;T:0;total:20301 | iSNV |
| F50 | F50-26 | 9443 | NS5 | 0.1777 | A:31097;G:6722;C:1;T:4;total:37824 | iSNV |
| F50 | F50-26 | 9899 | NS5 | 0.1065 | A:2;G:1;C:19128;T:2282;total:21413 | iSNV |
| F50 | F50-26 | 10364 | NS5 | 0.1134 | A:108;G:1;C:26091;T:3352;total:29552 | iSNV |
| F50 | F50-26 | 10373 | NS5 | 0.2017 | A:10;G:1;C:24395;T:6170;total:30576 | iSNV |
| F50 | F50-26 | 10428 | 3'-UTR | 0.8996 | A:0;G:1;C:2582;T:23131;total:25714 | iSNV |
| F50 | F50-26 | 10447 | 3'-UTR | 0.0345 | A:12;G:5;C:22536;T:808;total:23361 | iSNV |
| F50 | F50-26 | 10577 | 3'-UTR | 0.0321 | A:6;G:1;C:18405;T:611;total:19023 | iSNV |
| F50 | F50-27 | 292 | C | 0.0478 | A:3;G:0;C:18383;T:923;total:19309 | iSNV |
| F50 | F50-27 | 662 | M | 0.1274 | A:14710;G:2148;C:2;T:0;total:16860 | iSNV |
| F50 | F50-27 | 716 | M | 0.1126 | A:3;G:16785;C:0;T:2131;total:18919 | iSNV |
| F50 | F50-27 | 906 | M | 0.0917 | A:10724;G:1084;C:5;T:1;total:11814 | iSNV |
| F50 | F50-27 | 1046 | E | 0.0217 | A:300;G:13460;C:0;T:4;total:13764 | iSNV |
| F50 | F50-27 | 1083 | E | 0.0302 | A:12135;G:378;C:1;T:0;total:12514 | iSNV |
| F50 | F50-27 | 1109 | E | 0.0286 | A:2;G:0;C:13962;T:412;total:14376 | iSNV |
| F50 | F50-27 | 1218 | E | 0.9992 | A:0;G:0;C:12;T:13785;total:13797 | SNP |
| F50 | F50-27 | 1512 | E | 0.3612 | A:7004;G:5382;C:2513;T:1;total:14900 | iSNV |
| F50 | F50-27 | 1514 | E | 0.1185 | A:13143;G:16;C:1770;T:0;total:14929 | iSNV |
| F50 | F50-27 | 1709 | E | 0.0464 | A:608;G:12473;C:0;T:1;total:13082 | iSNV |
| F50 | F50-27 | 1797 | E | 0.1226 | A:4;G:2120;C:654;T:14504;total:17282 | iSNV |
| F50 | F50-27 | 2270 | E | 0.1221 | A:9260;G:0;C:1289;T:1;total:10550 | iSNV |
| F50 | F50-27 | 2274 | E | 0.3729 | A:8;G:6648;C:0;T:3958;total:10614 | iSNV |
| F50 | F50-27 | 2277 | E | 0.0413 | A:2;G:1;C:441;T:10230;total:10674 | iSNV |
| F50 | F50-27 | 2531 | NS1 | 0.1675 | A:1949;G:3;C:6;T:9673;total:11631 | iSNV |
| F50 | F50-27 | 2664 | NS1 | 0.7626 | A:0;G:2;C:3668;T:11776;total:15446 | iSNV |
| F50 | F50-27 | 3181 | NS1 | 0.0213 | A:17057;G:372;C:2;T:1;total:17432 | iSNV |
| F50 | F50-27 | 3869 | NS2A | 0.9982 | A:1;G:0;C:27;T:15037;total:15065 | SNP |
| F50 | F50-27 | 4682 | NS3 | 0.1064 | A:0;G:2;C:1826;T:15331;total:17159 | iSNV |
| F50 | F50-27 | 4974 | NS3 | 0.0867 | A:1445;G:15204;C:0;T:0;total:16649 | iSNV |
| F50 | F50-27 | 5617 | NS3 | 0.1191 | A:14305;G:1936;C:3;T:0;total:16244 | iSNV |
| F50 | F50-27 | 5737 | NS3 | 0.0245 | A:18408;G:464;C:1;T:5;total:18878 | iSNV |
| F50 | F50-27 | 6063 | NS3 | 0.0942 | A:4;G:1;C:15011;T:1562;total:16578 | iSNV |
| F50 | F50-27 | 6182 | NS3 | 0.0931 | A:2;G:1;C:1229;T:11963;total:13195 | iSNV |
| F50 | F50-27 | 6867 | NS4A | 0.0278 | A:337;G:11778;C:1;T:1;total:12117 | iSNV |
| F50 | F50-27 | 6957 | NS4A | 0.0862 | A:11425;G:1079;C:0;T:0;total:12504 | iSNV |
| F50 | F50-27 | 7250 | NS4A | 0.0465 | A:8477;G:414;C:0;T:0;total:8891 | iSNV |
| F50 | F50-27 | 7626 | NS4B | 0.786 | A:4;G:11382;C:0;T:3101;total:14487 | iSNV |
| F50 | F50-27 | 7633 | NS4B | 0.1106 | A:0;G:0;C:12897;T:1604;total:14501 | iSNV |
| F50 | F50-27 | 7697 | NS5 | 0.0849 | A:8;G:12659;C:1;T:1176;total:13844 | iSNV |
| F50 | F50-27 | 7835 | NS5 | 0.0583 | A:1;G:0;C:856;T:13802;total:14659 | iSNV |
| F50 | F50-27 | 8756 | NS5 | 0.1144 | A:9961;G:1287;C:0;T:0;total:11248 | iSNV |
| F50 | F50-27 | 9728 | NS5 | 0.0333 | A:2;G:0;C:14139;T:488;total:14629 | iSNV |
| F50 | F50-27 | 9830 | NS5 | 0.0953 | A:0;G:3;C:1407;T:13352;total:14762 | iSNV |
| F50 | F50-27 | 10139 | NS5 | 0.0361 | A:1;G:1;C:560;T:14929;total:15491 | iSNV |
| F50 | F50-27 | 10428 | 3'-UTR | 0.5632 | A:3;G:5;C:7327;T:9436;total:16771 | iSNV |
| F50 | F50-27 | 10447 | 3'-UTR | 0.0269 | A:4;G:0;C:15110;T:418;total:15532 | iSNV |
| F50 | F50-27 | 10904 | 3'-UTR | 0.1016 | A:0;G:0;C:2334;T:264;total:2598 | iSNV |
| F50 | F50-28 | 230 | C | 0.9395 | A:1;G:0;C:22507;T:1451;total:23959 | iSNV |
| F50 | F50-28 | 292 | C | 0.0231 | A:4;G:0;C:22206;T:526;total:22736 | iSNV |
| F50 | F50-28 | 353 | C | 0.0302 | A:21219;G:661;C:0;T:0;total:21880 | iSNV |
| F50 | F50-28 | 645 | M | 0.0282 | A:19521;G:568;C:2;T:1;total:20092 | iSNV |
| F50 | F50-28 | 734 | M | 0.0378 | A:841;G:2;C:1;T:21354;total:22198 | iSNV |
| F50 | F50-28 | 828 | M | 0.04 | A:15061;G:629;C:0;T:1;total:15691 | iSNV |
| F50 | F50-28 | 998 | E | 0.034 | A:2;G:0;C:14089;T:497;total:14588 | iSNV |
| F50 | F50-28 | 1044 | E | 0.0933 | A:2;G:1;C:1475;T:14328;total:15806 | iSNV |

| | | | | | | |
|---|---|---|---|---|---|---|
| F50 | F50-28 | 1087 | E | 0.0334 | A:14237;G:493;C:1;T:1;total:14732 | iSNV |
| F50 | F50-28 | 1117 | E | 0.034 | A:16534;G:583;C:0;T:0;total:17117 | iSNV |
| F50 | F50-28 | 1218 | E | 0.9636 | A:0;G:2;C:600;T:15858;total:16460 | iSNV |
| F50 | F50-28 | 1428 | E | 0.9647 | A:733;G:19981;C:0;T:5;total:20719 | iSNV |
| F50 | F50-28 | 1512 | E | 0.0286 | A:17713;G:522;C:6;T:0;total:18241 | iSNV |
| F50 | F50-28 | 1595 | E | 0.0453 | A:605;G:12739;C:1;T:2;total:13347 | iSNV |
| F50 | F50-28 | 1784 | E | 0.1189 | A:3;G:0;C:17341;T:2341;total:19685 | iSNV |
| F50 | F50-28 | 1910 | E | 0.0889 | A:1551;G:15881;C:0;T:2;total:17434 | iSNV |
| F50 | F50-28 | 2369 | E | 0.1259 | A:1335;G:9260;C:0;T:3;total:10598 | iSNV |
| F50 | F50-28 | 3099 | NS1 | 0.0425 | A:759;G:17085;C:0;T:0;total:17844 | iSNV |
| F50 | F50-28 | 3380 | NS1 | 0.0207 | A:0;G:0;C:333;T:15690;total:16023 | iSNV |
| F50 | F50-28 | 3719 | NS1 | 0.0409 | A:509;G:4;C:28;T:11879;total:12420 | iSNV |
| F50 | F50-28 | 3869 | NS2A | 0.9678 | A:1;G:0;C:567;T:17021;total:17589 | iSNV |
| F50 | F50-28 | 4348 | NS2B | 0.0307 | A:1;G:1;C:508;T:15994;total:16504 | iSNV |
| F50 | F50-28 | 4447 | NS2B | 0.0997 | A:11300;G:1253;C:3;T:1;total:12557 | iSNV |
| F50 | F50-28 | 4632 | NS3 | 0.0552 | A:0;G:1180;C:8;T:20157;total:21345 | iSNV |
| F50 | F50-28 | 5043 | NS3 | 0.0655 | A:16253;G:1141;C:0;T:0;total:17394 | iSNV |
| F50 | F50-28 | 5291 | NS3 | 0.0216 | A:15566;G:344;C:1;T:0;total:15911 | iSNV |
| F50 | F50-28 | 5353 | NS3 | 0.0211 | A:16324;G:353;C:0;T:0;total:16677 | iSNV |
| F50 | F50-28 | 5654 | NS3 | 0.0297 | A:5;G:1;C:18098;T:555;total:18659 | iSNV |
| F50 | F50-28 | 5705 | NS3 | 0.031 | A:19863;G:637;C:1;T:0;total:20501 | iSNV |
| F50 | F50-28 | 5737 | NS3 | 0.1224 | A:18777;G:2621;C:1;T:3;total:21402 | iSNV |
| F50 | F50-28 | 5952 | NS3 | 0.0729 | A:3;G:0;C:19515;T:1535;total:21053 | iSNV |
| F50 | F50-28 | 6472 | NS4A | 0.025 | A:0;G:1;C:399;T:15526;total:15926 | iSNV |
| F50 | F50-28 | 6714 | NS4A | 0.0661 | A:1034;G:14597;C:0;T:1;total:15632 | iSNV |
| F50 | F50-28 | 6729 | NS4A | 0.0283 | A:444;G:0;C:15213;T:9;total:15666 | iSNV |
| F50 | F50-28 | 6730 | NS4A | 0.0284 | A:0;G:0;C:443;T:15153;total:15596 | iSNV |
| F50 | F50-28 | 6737 | NS4A | 0.0588 | A:5;G:0;C:14813;T:926;total:15744 | iSNV |
| F50 | F50-28 | 6867 | NS4A | 0.0266 | A:399;G:14562;C:1;T:0;total:14962 | iSNV |
| F50 | F50-28 | 6970 | NS4A | 0.5686 | A:6903;G:8;C:9082;T:8;total:16001 | iSNV |
| F50 | F50-28 | 7104 | NS4A | 0.5537 | A:7725;G:6231;C:3;T:0;total:13959 | iSNV |
| F50 | F50-28 | 7451 | NS4B | 0.0261 | A:0;G:10016;C:269;T:4;total:10289 | iSNV |
| F50 | F50-28 | 7633 | NS4B | 0.945 | A:1;G:1;C:892;T:15320;total:16214 | iSNV |
| F50 | F50-28 | 8909 | NS5 | 0.1299 | A:0;G:2;C:1915;T:12823;total:14740 | iSNV |
| F50 | F50-28 | 9782 | NS5 | 0.0717 | A:1365;G:17654;C:2;T:5;total:19026 | iSNV |
| F50 | F50-28 | 10259 | NS5 | 0.0341 | A:23597;G:834;C:1;T:1;total:24433 | iSNV |
| F50 | F50-28 | 10335 | NS5 | 0.0671 | A:0;G:2;C:1617;T:22466;total:24085 | iSNV |
| F50 | F50-28 | 10428 | 3'-UTR | 0.5424 | A:1;G:0;C:8679;T:10283;total:18963 | iSNV |
| F50 | F50-28 | 10447 | 3'-UTR | 0.0381 | A:1;G:0;C:16919;T:672;total:17592 | iSNV |
| F50 | F50-29 | 332 | C | 0.0399 | A:22149;G:922;C:1;T:0;total:23072 | iSNV |
| F50 | F50-29 | 1218 | E | 0.9983 | A:1;G:2;C:25;T:16043;total:16071 | SNP |
| F50 | F50-29 | 1296 | E | 0.1609 | A:1;G:1;C:3124;T:16285;total:19411 | iSNV |
| F50 | F50-29 | 1447 | E | 0.7201 | A:15141;G:2;C:5889;T:3;total:21035 | iSNV |
| F50 | F50-29 | 1914 | E | 0.1508 | A:12513;G:2223;C:2;T:0;total:14738 | iSNV |
| F50 | F50-29 | 2362 | E | 0.9841 | A:1;G:144;C:9283;T:5;total:9433 | SNP |
| F50 | F50-29 | 3438 | NS1 | 0.1353 | A:3;G:0;C:13000;T:2036;total:15039 | iSNV |
| F50 | F50-29 | 3645 | NS1 | 0.1459 | A:0;G:1;C:1963;T:11485;total:13449 | iSNV |
| F50 | F50-29 | 3869 | NS2A | 0.999 | A:0;G:0;C:18;T:16822;total:16840 | SNP |
| F50 | F50-29 | 3959 | NS2A | 0.7075 | A:2;G:0;C:5107;T:12348;total:17457 | iSNV |
| F50 | F50-29 | 4076 | NS2A | 0.03 | A:2;G:0;C:13276;T:412;total:13690 | iSNV |
| F50 | F50-29 | 4829 | NS3 | 0.0317 | A:18694;G:613;C:1;T:1;total:19309 | iSNV |
| F50 | F50-29 | 4896 | NS3 | 0.0359 | A:777;G:20788;C:5;T:54;total:21624 | iSNV |
| F50 | F50-29 | 4943 | NS3 | 0.0348 | A:17449;G:631;C:2;T:0;total:18082 | iSNV |
| F50 | F50-29 | 5702 | NS3 | 0.0722 | A:4;G:2;C:20486;T:1596;total:22088 | iSNV |
| F50 | F50-29 | 5736 | NS3 | 0.0424 | A:915;G:0;C:20654;T:4;total:21573 | iSNV |
| F50 | F50-29 | 6557 | NS4A | 0.0397 | A:12995;G:538;C:0;T:0;total:13533 | iSNV |
| F50 | F50-29 | 6714 | NS4A | 0.0663 | A:1045;G:14704;C:3;T:1;total:15753 | iSNV |
| F50 | F50-29 | 6715 | NS4A | 0.0215 | A:4;G:1;C:15524;T:342;total:15871 | iSNV |
| F50 | F50-29 | 6867 | NS4A | 0.4304 | A:6517;G:8623;C:0;T:0;total:15140 | iSNV |
| F50 | F50-29 | 6900 | NS4A | 0.0328 | A:471;G:13872;C:0;T:0;total:14343 | iSNV |
| F50 | F50-29 | 7481 | NS4B | 0.0874 | A:1;G:0;C:936;T:9766;total:10703 | iSNV |
| F50 | F50-29 | 7528 | NS4B | 0.1281 | A:1;G:0;C:11914;T:1751;total:13666 | iSNV |
| F50 | F50-29 | 7627 | NS4B | 0.1619 | A:7;G:2616;C:13530;T:2;total:16155 | iSNV |
| F50 | F50-29 | 7633 | NS4B | 0.8369 | A:0;G:0;C:2628;T:13477;total:16105 | iSNV |
| F50 | F50-29 | 8627 | NS5 | 0.0224 | A:1;G:0;C:17277;T:396;total:17674 | iSNV |
| F50 | F50-29 | 8844 | NS5 | 0.032 | A:445;G:13453;C:0;T:0;total:13898 | iSNV |
| F50 | F50-29 | 8966 | NS5 | 0.0322 | A:3;G:0;C:16277;T:543;total:16823 | iSNV |
| F50 | F50-29 | 9032 | NS5 | 0.0413 | A:1;G:1;C:690;T:15995;total:16687 | iSNV |
| F50 | F50-29 | 9659 | NS5 | 0.033 | A:9;G:1;C:17255;T:591;total:17856 | iSNV |
| F50 | F50-29 | 10428 | 3'-UTR | 0.9525 | A:5;G:2;C:846;T:16928;total:17781 | iSNV |
| F50 | F50-29 | 10513 | 3'-UTR | 0.0435 | A:0;G:3;C:745;T:16374;total:17122 | iSNV |
| F50 | F50-3 | 446 | C | 0.0235 | A:4;G:0;C:10342;T:249;total:10595 | iSNV |
| F50 | F50-3 | 563 | M | 0.9744 | A:6756;G:178;C:1;T:0;total:6935 | iSNV |
| F50 | F50-3 | 978 | E | 0.0547 | A:0;G:0;C:337;T:5822;total:6159 | iSNV |
| F50 | F50-3 | 1257 | E | 0.9753 | A:7160;G:0;C:182;T:0;total:7342 | iSNV |
| F50 | F50-3 | 1428 | E | 0.0521 | A:7684;G:423;C:0;T:0;total:8107 | iSNV |
| F50 | F50-3 | 1512 | E | 0.0951 | A:5742;G:604;C:1;T:0;total:6347 | iSNV |
| F50 | F50-3 | 1772 | E | 0.975 | A:1;G:6859;C:0;T:176;total:7036 | iSNV |
| F50 | F50-3 | 1797 | E | 0.3493 | A:0;G:2619;C:0;T:4878;total:7497 | iSNV |
| F50 | F50-3 | 1799 | E | 0.1388 | A:0;G:0;C:6476;T:1044;total:7520 | iSNV |
| F50 | F50-3 | 2240 | E | 0.067 | A:0;G:0;C:5302;T:381;total:5683 | iSNV |
| F50 | F50-3 | 2372 | E | 0.9758 | A:0;G:1;C:105;T:4224;total:4330 | iSNV |

| | | | | | | |
|---|---|---|---|---|---|---|
| F50 | F50-3 | 2493 | NS1 | 0.9719 | A:140;G:4835;C:1;T:1;total:4977 | iSNV |
| F50 | F50-3 | 2504 | NS1 | 0.9712 | A:139;G:4685;C:0;T:0;total:4824 | iSNV |
| F50 | F50-3 | 2531 | NS1 | 0.9713 | A:1;G:0;C:5302;T:157;total:5460 | iSNV |
| F50 | F50-3 | 2735 | NS1 | 0.0242 | A:0;G:0;C:9390;T:233;total:9623 | iSNV |
| F50 | F50-3 | 3143 | NS1 | 0.022 | A:0;G:207;C:0;T:9179;total:9386 | iSNV |
| F50 | F50-3 | 3355 | NS1 | 0.3575 | A:4103;G:2283;C:0;T:0;total:6386 | iSNV |
| F50 | F50-3 | 3459 | NS1 | 0.0284 | A:130;G:4441;C:0;T:0;total:4571 | iSNV |
| F50 | F50-3 | 3572 | NS1 | 0.9741 | A:0;G:0;C:136;T:5111;total:5247 | iSNV |
| F50 | F50-3 | 3962 | NS2A | 0.9762 | A:0;G:0;C:198;T:8111;total:8309 | iSNV |
| F50 | F50-3 | 4155 | NS2A | 0.9721 | A:3202;G:0;C:0;T:92;total:3294 | iSNV |
| F50 | F50-3 | 4397 | NS2B | 0.06 | A:4;G:0;C:6270;T:401;total:6675 | iSNV |
| F50 | F50-3 | 4663 | NS3 | 0.0608 | A:2;G:0;C:7546;T:489;total:8037 | iSNV |
| F50 | F50-3 | 4712 | NS3 | 0.976 | A:9182;G:226;C:0;T:0;total:9408 | iSNV |
| F50 | F50-3 | 5311 | NS3 | 0.9766 | A:1;G:0;C:143;T:5951;total:6095 | iSNV |
| F50 | F50-3 | 5447 | NS3 | 0.0222 | A:2;G:0;C:9850;T:224;total:10076 | iSNV |
| F50 | F50-3 | 5978 | NS3 | 0.3303 | A:5675;G:2800;C:0;T:1;total:8476 | iSNV |
| F50 | F50-3 | 6080 | NS3 | 0.9807 | A:6839;G:135;C:0;T:0;total:6974 | SNP |
| F50 | F50-3 | 6534 | NS4A | 0.1023 | A:566;G:2;C:0;T:4962;total:5530 | iSNV |
| F50 | F50-3 | 6782 | NS4A | 0.0232 | A:0;G:6817;C:162;T:0;total:6979 | iSNV |
| F50 | F50-3 | 7527 | NS4B | 0.0348 | A:5313;G:192;C:0;T:0;total:5505 | iSNV |
| F50 | F50-3 | 8282 | NS5 | 0.9939 | A:0;G:1;C:8455;T:51;total:8507 | SNP |
| F50 | F50-3 | 8900 | NS5 | 0.9765 | A:185;G:0;C:7676;T:3;total:7864 | iSNV |
| F50 | F50-3 | 9008 | NS5 | 0.0445 | A:0;G:0;C:334;T:7168;total:7502 | iSNV |
| F50 | F50-3 | 9132 | NS5 | 0.0205 | A:4;G:0;C:9247;T:194;total:9445 | iSNV |
| F50 | F50-3 | 9370 | NS5 | 0.1367 | A:1629;G:0;C:1;T:10285;total:11915 | iSNV |
| F50 | F50-3 | 9446 | NS5 | 0.9739 | A:298;G:11085;C:0;T:3;total:11386 | iSNV |
| F50 | F50-3 | 10308 | NS5 | 0.0247 | A:248;G:9777;C:0;T:0;total:10025 | iSNV |
| F50 | F50-3 | 10342 | NS5 | 0.0209 | A:3;G:1;C:9742;T:209;total:9955 | iSNV |
| F50 | F50-3 | 10428 | 3'-UTR | 0.1889 | A:1;G:3;C:6220;T:1450;total:7674 | iSNV |
| F50 | F50-30 | 688 | M | 0.0494 | A:19716;G:1026;C:2;T:1;total:20745 | iSNV |
| F50 | F50-30 | 719 | M | 0.04 | A:4;G:1;C:20767;T:866;total:21638 | iSNV |
| F50 | F50-30 | 1218 | E | 0.9991 | A:0;G:2;C:13;T:16569;total:16584 | SNP |
| F50 | F50-30 | 1413 | E | 0.9867 | A:285;G:21474;C:4;T:2;total:21765 | SNP |
| F50 | F50-30 | 1428 | E | 0.9895 | A:213;G:20109;C:0;T:2;total:20324 | SNP |
| F50 | F50-30 | 2541 | NS1 | 0.03 | A:2;G:0;C:14341;T:445;total:14788 | iSNV |
| F50 | F50-30 | 3869 | NS2A | 0.9983 | A:1;G:3;C:26;T:17276;total:17306 | SNP |
| F50 | F50-30 | 3965 | NS2A | 0.0424 | A:5;G:0;C:17624;T:782;total:18411 | iSNV |
| F50 | F50-30 | 4005 | NS2A | 0.9874 | A:1;G:0;C:197;T:15399;total:15597 | SNP |
| F50 | F50-30 | 4468 | NS2B | 0.0237 | A:319;G:0;C:1;T:13095;total:13415 | iSNV |
| F50 | F50-30 | 5025 | NS3 | 0.0218 | A:4;G:0;C:21981;T:491;total:22476 | iSNV |
| F50 | F50-30 | 5737 | NS3 | 0.0579 | A:20982;G:1291;C:0;T:0;total:22273 | iSNV |
| F50 | F50-30 | 6185 | NS3 | 0.0499 | A:5;G:1;C:16423;T:863;total:17292 | iSNV |
| F50 | F50-30 | 6336 | NS3 | 0.0555 | A:11672;G:3;C:2;T:687;total:12364 | iSNV |
| F50 | F50-30 | 6714 | NS4A | 0.1525 | A:2443;G:13564;C:1;T:2;total:16010 | iSNV |
| F50 | F50-30 | 6867 | NS4A | 0.166 | A:2669;G:13405;C:1;T:0;total:16075 | iSNV |
| F50 | F50-30 | 7264 | NS4A | 0.5054 | A:0;G:0;C:5645;T:5767;total:11412 | iSNV |
| F50 | F50-30 | 7633 | NS4B | 0.9913 | A:0;G:1;C:149;T:17015;total:17165 | SNP |
| F50 | F50-30 | 8023 | NS5 | 0.022 | A:476;G:3;C:3;T:21153;total:21635 | iSNV |
| F50 | F50-30 | 8024 | NS5 | 0.0288 | A:7;G:20995;C:0;T:625;total:21627 | iSNV |
| F50 | F50-30 | 9293 | NS5 | 0.0297 | A:653;G:21267;C:0;T:2;total:21922 | iSNV |
| F50 | F50-30 | 9506 | NS5 | 0.0549 | A:1;G:1;C:1403;T:24113;total:25518 | iSNV |
| F50 | F50-30 | 10376 | NS5 | 0.9883 | A:20899;G:245;C:2;T:1;total:21147 | SNP |
| F50 | F50-30 | 10419 | 3'-UTR | 0.0371 | A:3;G:2;C:18578;T:717;total:19300 | iSNV |
| F50 | F50-30 | 10428 | 3'-UTR | 0.9727 | A:0;G:0;C:515;T:18333;total:18848 | iSNV |
| F50 | F50-4 | 563 | M | 0.9982 | A:12843;G:22;C:1;T:1;total:12867 | SNP |
| F50 | F50-4 | 1257 | E | 0.9989 | A:13636;G:1;C:14;T:1;total:13652 | SNP |
| F50 | F50-4 | 1512 | E | 0.6849 | A:3829;G:8322;C:0;T:0;total:12151 | iSNV |
| F50 | F50-4 | 1772 | E | 0.9986 | A:2;G:13764;C:2;T:16;total:13784 | SNP |
| F50 | F50-4 | 1799 | E | 0.0426 | A:4;G:0;C:14043;T:626;total:14673 | iSNV |
| F50 | F50-4 | 2372 | E | 0.9994 | A:0;G:0;C:6;T:8643;total:8649 | SNP |
| F50 | F50-4 | 2493 | NS1 | 0.9986 | A:13;G:9816;C:0;T:1;total:9830 | SNP |
| F50 | F50-4 | 2504 | NS1 | 0.9989 | A:9;G:9440;C:0;T:2;total:9451 | SNP |
| F50 | F50-4 | 2531 | NS1 | 0.9985 | A:2;G:0;C:10322;T:14;total:10338 | SNP |
| F50 | F50-4 | 2684 | NS1 | 0.064 | A:13816;G:946;C:1;T:0;total:14763 | iSNV |
| F50 | F50-4 | 3572 | NS1 | 0.9993 | A:1;G:0;C:7;T:10440;total:10448 | SNP |
| F50 | F50-4 | 3716 | NS1 | 0.0677 | A:1;G:0;C:7666;T:557;total:8224 | iSNV |
| F50 | F50-4 | 3962 | NS2A | 0.9985 | A:0;G:0;C:23;T:14652;total:14675 | SNP |
| F50 | F50-4 | 4155 | NS2A | 0.9981 | A:6510;G:1;C:2;T:10;total:6523 | SNP |
| F50 | F50-4 | 4397 | NS2B | 0.0316 | A:3;G:0;C:11610;T:379;total:11992 | iSNV |
| F50 | F50-4 | 4712 | NS3 | 0.9989 | A:16687;G:16;C:3;T:0;total:16706 | SNP |
| F50 | F50-4 | 5311 | NS3 | 0.9246 | A:1;G:5;C:845;T:10344;total:11195 | iSNV |
| F50 | F50-4 | 6080 | NS3 | 0.9985 | A:12802;G:18;C:2;T:0;total:12822 | SNP |
| F50 | F50-4 | 7880 | NS5 | 0.0204 | A:16728;G:350;C:1;T:1;total:17080 | iSNV |
| F50 | F50-4 | 8282 | NS5 | 0.998 | A:4;G:0;C:15372;T:28;total:15404 | SNP |
| F50 | F50-4 | 8900 | NS5 | 0.9921 | A:95;G:0;C:12457;T:5;total:12557 | SNP |
| F50 | F50-4 | 9370 | NS5 | 0.042 | A:832;G:1;C:2;T:18953;total:19788 | iSNV |
| F50 | F50-4 | 9446 | NS5 | 0.9991 | A:16;G:19414;C:0;T:3;total:19433 | SNP |
| F50 | F50-4 | 10428 | 3'-UTR | 0.1246 | A:0;G:0;C:11915;T:1697;total:13612 | iSNV |
| F50 | F50-5 | 563 | M | 0.9727 | A:6176;G:174;C:1;T:0;total:6351 | iSNV |
| F50 | F50-5 | 593 | M | 0.0308 | A:1;G:0;C:5994;T:191;total:6186 | iSNV |
| F50 | F50-5 | 897 | M | 0.021 | A:1;G:0;C:5074;T:109;total:5184 | iSNV |
| F50 | F50-5 | 1116 | E | 0.0368 | A:6610;G:253;C:0;T:0;total:6863 | iSNV |

| | | | | | | |
|---|---|---|---|---|---|---|
| F50 | F50-5 | 1257 | E | 0.9786 | A:6436;G:0;C:141;T:0;total:6577 | iSNV |
| F50 | F50-5 | 1363 | E | 0.0425 | A:0;G:0;C:6972;T:310;total:7282 | iSNV |
| F50 | F50-5 | 1389 | E | 0.02 | A:7580;G:155;C:0;T:0;total:7735 | iSNV |
| F50 | F50-5 | 1428 | E | 0.1687 | A:6164;G:1251;C:0;T:0;total:7415 | iSNV |
| F50 | F50-5 | 1473 | E | 0.0374 | A:5990;G:6;C:233;T:0;total:6229 | iSNV |
| F50 | F50-5 | 1512 | E | 0.2331 | A:4571;G:1390;C:0;T:0;total:5961 | iSNV |
| F50 | F50-5 | 1772 | E | 0.9672 | A:3;G:6501;C:0;T:221;total:6725 | iSNV |
| F50 | F50-5 | 1799 | E | 0.0541 | A:0;G:0;C:6782;T:388;total:7170 | iSNV |
| F50 | F50-5 | 2372 | E | 0.9719 | A:0;G:2;C:116;T:4004;total:4122 | iSNV |
| F50 | F50-5 | 2493 | NS1 | 0.9785 | A:104;G:4727;C:0;T:0;total:4831 | iSNV |
| F50 | F50-5 | 2504 | NS1 | 0.9785 | A:102;G:4629;C:0;T:0;total:4731 | iSNV |
| F50 | F50-5 | 2531 | NS1 | 0.9801 | A:0;G:0;C:5150;T:105;total:5255 | SNP |
| F50 | F50-5 | 2628 | NS1 | 0.2145 | A:6117;G:1671;C:0;T:0;total:7788 | iSNV |
| F50 | F50-5 | 3533 | NS1 | 0.0437 | A:0;G:215;C:0;T:4701;total:4916 | iSNV |
| F50 | F50-5 | 3572 | NS1 | 0.973 | A:1;G:0;C:133;T:4785;total:4919 | iSNV |
| F50 | F50-5 | 3869 | NS2A | 0.02 | A:1;G:0;C:7528;T:154;total:7683 | iSNV |
| F50 | F50-5 | 3962 | NS2A | 0.9764 | A:0;G:0;C:173;T:7142;total:7315 | iSNV |
| F50 | F50-5 | 4155 | NS2A | 0.9709 | A:3079;G:15;C:0;T:93;total:3187 | iSNV |
| F50 | F50-5 | 4712 | NS3 | 0.978 | A:8285;G:187;C:1;T:0;total:8473 | iSNV |
| F50 | F50-5 | 5311 | NS3 | 0.9672 | A:0;G:3;C:188;T:5540;total:5731 | iSNV |
| F50 | F50-5 | 6080 | NS3 | 0.9737 | A:5959;G:161;C:1;T:0;total:6121 | iSNV |
| F50 | F50-5 | 6901 | NS4A | 0.0433 | A:1;G:3;C:204;T:4494;total:4702 | iSNV |
| F50 | F50-5 | 7199 | NS4A | 0.0397 | A:0;G:0;C:3933;T:163;total:4096 | iSNV |
| F50 | F50-5 | 8282 | NS5 | 0.9798 | A:0;G:0;C:7384;T:153;total:7537 | iSNV |
| F50 | F50-5 | 8900 | NS5 | 0.98 | A:140;G:0;C:6786;T:69;total:6995 | iSNV |
| F50 | F50-5 | 9008 | NS5 | 0.0374 | A:0;G:2;C:243;T:6245;total:6490 | iSNV |
| F50 | F50-5 | 9370 | NS5 | 0.0581 | A:600;G:2;C:0;T:9720;total:10322 | iSNV |
| F50 | F50-5 | 9446 | NS5 | 0.9779 | A:219;G:9657;C:0;T:0;total:9876 | iSNV |
| F50 | F50-5 | 10428 | 3'-UTR | 0.0953 | A:0;G:4;C:6332;T:668;total:7004 | iSNV |
| F50 | F50-5 | 10642 | 3'-UTR | 0.2026 | A:1;G:0;C:1088;T:4279;total:5368 | iSNV |
| F50 | F50-6 | 563 | M | 0.9975 | A:15367;G:37;C:2;T:1;total:15407 | SNP |
| F50 | F50-6 | 1257 | E | 0.9966 | A:16328;G:2;C:54;T:0;total:16384 | SNP |
| F50 | F50-6 | 1453 | E | 0.1396 | A:2507;G:2;C:3;T:15434;total:17946 | iSNV |
| F50 | F50-6 | 1512 | E | 0.6447 | A:5227;G:9472;C:10;T:0;total:14709 | iSNV |
| F50 | F50-6 | 1772 | E | 0.9966 | A:4;G:17164;C:1;T:55;total:17224 | SNP |
| F50 | F50-6 | 1799 | E | 0.0707 | A:2;G:0;C:17034;T:1297;total:18333 | iSNV |
| F50 | F50-6 | 2372 | E | 0.997 | A:1;G:1;C:32;T:11035;total:11069 | SNP |
| F50 | F50-6 | 2493 | NS1 | 0.9973 | A:31;G:11808;C:0;T:1;total:11840 | SNP |
| F50 | F50-6 | 2504 | NS1 | 0.9978 | A:25;G:11278;C:0;T:0;total:11303 | SNP |
| F50 | F50-6 | 2531 | NS1 | 0.9967 | A:5;G:0;C:12312;T:36;total:12353 | SNP |
| F50 | F50-6 | 3572 | NS1 | 0.9958 | A:3;G:0;C:46;T:11576;total:11625 | SNP |
| F50 | F50-6 | 3962 | NS2A | 0.9966 | A:0;G:0;C:60;T:17474;total:17534 | SNP |
| F50 | F50-6 | 4155 | NS2A | 0.9963 | A:7359;G:3;C:0;T:25;total:7387 | SNP |
| F50 | F50-6 | 4712 | NS3 | 0.9971 | A:20227;G:59;C:0;T:1;total:20287 | SNP |
| F50 | F50-6 | 5311 | NS3 | 0.9916 | A:0;G:4;C:105;T:12846;total:12955 | SNP |
| F50 | F50-6 | 6080 | NS3 | 0.9965 | A:15637;G:55;C:0;T:0;total:15692 | SNP |
| F50 | F50-6 | 8282 | NS5 | 0.9978 | A:2;G:0;C:18248;T:39;total:18289 | SNP |
| F50 | F50-6 | 8369 | NS5 | 0.0416 | A:5;G:2;C:21494;T:935;total:22436 | iSNV |
| F50 | F50-6 | 8891 | NS5 | 0.0207 | A:1;G:1;C:319;T:15029;total:15350 | iSNV |
| F50 | F50-6 | 8900 | NS5 | 0.9972 | A:42;G:0;C:15756;T:3;total:15801 | SNP |
| F50 | F50-6 | 9370 | NS5 | 0.073 | A:1834;G:2;C:12;T:23242;total:25090 | iSNV |
| F50 | F50-6 | 9446 | NS5 | 0.9971 | A:71;G:25164;C:1;T:2;total:25238 | SNP |
| F50 | F50-6 | 10428 | 3'-UTR | 0.0871 | A:4;G:1;C:15296;T:1461;total:16762 | iSNV |
| F50 | F50-6 | 10685 | 3'-UTR | 0.048 | A:0;G:0;C:665;T:13183;total:13848 | iSNV |
| F50 | F50-7 | 563 | M | 0.9937 | A:9013;G:58;C:0;T:0;total:9071 | SNP |
| F50 | F50-7 | 1257 | E | 0.9932 | A:9505;G:1;C:65;T:0;total:9571 | SNP |
| F50 | F50-7 | 1428 | E | 0.0271 | A:11132;G:311;C:2;T:0;total:11445 | iSNV |
| F50 | F50-7 | 1447 | E | 0.7674 | A:7982;G:1;C:2421;T:4;total:10408 | iSNV |
| F50 | F50-7 | 1772 | E | 0.9927 | A:4;G:9849;C:0;T:69;total:9922 | SNP |
| F50 | F50-7 | 2018 | E | 0.045 | A:1;G:0;C:5465;T:258;total:5724 | iSNV |
| F50 | F50-7 | 2372 | E | 0.9943 | A:0;G:1;C:37;T:6588;total:6626 | SNP |
| F50 | F50-7 | 2408 | E | 0.0313 | A:1;G:0;C:5937;T:192;total:6130 | iSNV |
| F50 | F50-7 | 2493 | NS1 | 0.9936 | A:44;G:6935;C:1;T:0;total:6980 | SNP |
| F50 | F50-7 | 2504 | NS1 | 0.9934 | A:44;G:6594;C:0;T:0;total:6638 | SNP |
| F50 | F50-7 | 2531 | NS1 | 0.9908 | A:2;G:2;C:7000;T:61;total:7065 | SNP |
| F50 | F50-7 | 2735 | NS1 | 0.0232 | A:1;G:0;C:10983;T:261;total:11245 | iSNV |
| F50 | F50-7 | 3572 | NS1 | 0.9912 | A:0;G:0;C:60;T:6701;total:6761 | SNP |
| F50 | F50-7 | 3962 | NS2A | 0.9926 | A:0;G:0;C:71;T:9427;total:9498 | SNP |
| F50 | F50-7 | 4155 | NS2A | 0.9928 | A:4253;G:1;C:2;T:28;total:4284 | SNP |
| F50 | F50-7 | 4712 | NS3 | 0.9933 | A:11115;G:76;C:0;T:0;total:11191 | SNP |
| F50 | F50-7 | 5311 | NS3 | 0.9903 | A:0;G:3;C:69;T:7286;total:7358 | SNP |
| F50 | F50-7 | 6080 | NS3 | 0.9929 | A:8782;G:63;C:0;T:0;total:8845 | SNP |
| F50 | F50-7 | 8084 | NS5 | 0.7815 | A:1;G:0;C:7642;T:2138;total:9781 | iSNV |
| F50 | F50-7 | 8282 | NS5 | 0.993 | A:0;G:0;C:9922;T:70;total:9992 | SNP |
| F50 | F50-7 | 8900 | NS5 | 0.992 | A:61;G:1;C:7964;T:3;total:8029 | SNP |
| F50 | F50-7 | 9008 | NS5 | 0.8103 | A:4;G:1;C:7760;T:1818;total:9583 | iSNV |
| F50 | F50-7 | 9446 | NS5 | 0.9928 | A:98;G:13695;C:0;T:2;total:13795 | SNP |
| F50 | F50-7 | 10398 | 3'-UTR | 0.0284 | A:280;G:9573;C:0;T:1;total:9854 | iSNV |
| F50 | F50-7 | 10428 | 3'-UTR | 0.0417 | A:3;G:1;C:9039;T:394;total:9437 | iSNV |
| F50 | F50-8 | 563 | M | 0.9954 | A:10346;G:48;C:0;T:0;total:10394 | SNP |
| F50 | F50-8 | 996 | E | 0.0266 | A:239;G:8743;C:0;T:0;total:8982 | iSNV |
| F50 | F50-8 | 1057 | E | 0.0341 | A:280;G:7919;C:0;T:1;total:8200 | iSNV |

| | | | | | | |
|---|---|---|---|---|---|---|
| F50 | F50-8 | 1116 | E | 0.1036 | A:9301;G:1076;C:1;T:0;total:10378 | iSNV |
| F50 | F50-8 | 1257 | E | 0.9943 | A:10484;G:2;C:59;T:0;total:10545 | SNP |
| F50 | F50-8 | 1428 | E | 0.1392 | A:10352;G:1675;C:1;T:0;total:12028 | iSNV |
| F50 | F50-8 | 1512 | E | 0.2489 | A:7486;G:2481;C:0;T:0;total:9967 | iSNV |
| F50 | F50-8 | 1708 | E | 0.0236 | A:226;G:9315;C:0;T:4;total:9545 | iSNV |
| F50 | F50-8 | 1772 | E | 0.9957 | A:1;G:10968;C:0;T:47;total:11016 | SNP |
| F50 | F50-8 | 1799 | E | 0.0527 | A:0;G:2;C:11189;T:623;total:11814 | iSNV |
| F50 | F50-8 | 1883 | E | 0.0621 | A:1;G:0;C:8860;T:587;total:9448 | iSNV |
| F50 | F50-8 | 2372 | E | 0.9945 | A:7;G:1;C:33;T:7339;total:7380 | SNP |
| F50 | F50-8 | 2493 | NS1 | 0.9925 | A:57;G:7664;C:1;T:0;total:7722 | SNP |
| F50 | F50-8 | 2504 | NS1 | 0.9926 | A:54;G:7325;C:0;T:1;total:7380 | SNP |
| F50 | F50-8 | 2531 | NS1 | 0.9944 | A:1;G:0;C:7865;T:44;total:7910 | SNP |
| F50 | F50-8 | 3290 | NS1 | 0.049 | A:561;G:0;C:1;T:10885;total:11447 | iSNV |
| F50 | F50-8 | 3572 | NS1 | 0.9958 | A:0;G:1;C:34;T:8213;total:8248 | SNP |
| F50 | F50-8 | 3962 | NS2A | 0.9962 | A:0;G:1;C:43;T:11297;total:11341 | SNP |
| F50 | F50-8 | 3983 | NS2A | 0.0208 | A:1;G:0;C:10849;T:231;total:11081 | iSNV |
| F50 | F50-8 | 4155 | NS2A | 0.9936 | A:5047;G:2;C:0;T:31;total:5080 | SNP |
| F50 | F50-8 | 4712 | NS3 | 0.9952 | A:13040;G:61;C:2;T:1;total:13104 | SNP |
| F50 | F50-8 | 5311 | NS3 | 0.9949 | A:0;G:0;C:45;T:8646;total:8691 | SNP |
| F50 | F50-8 | 5835 | NS3 | 0.0633 | A:903;G:13355;C:0;T:2;total:14260 | iSNV |
| F50 | F50-8 | 6057 | NS3 | 0.0203 | A:11827;G:6;C:0;T:246;total:12079 | iSNV |
| F50 | F50-8 | 6080 | NS3 | 0.994 | A:10403;G:62;C:1;T:0;total:10466 | SNP |
| F50 | F50-8 | 7583 | NS4B | 0.2395 | A:0;G:7040;C:1;T:2218;total:9259 | iSNV |
| F50 | F50-8 | 7811 | NS5 | 0.0517 | A:520;G:2;C:1;T:9524;total:10047 | iSNV |
| F50 | F50-8 | 8135 | NS5 | 0.0515 | A:0;G:1;C:693;T:12753;total:13447 | iSNV |
| F50 | F50-8 | 8282 | NS5 | 0.9954 | A:0;G:1;C:11648;T:53;total:11702 | SNP |
| F50 | F50-8 | 8870 | NS5 | 0.0609 | A:569;G:8769;C:0;T:4;total:9342 | iSNV |
| F50 | F50-8 | 8900 | NS5 | 0.9955 | A:43;G:0;C:9660;T:1;total:9704 | SNP |
| F50 | F50-8 | 9008 | NS5 | 0.0339 | A:0;G:1;C:367;T:10456;total:10824 | iSNV |
| F50 | F50-8 | 9370 | NS5 | 0.0553 | A:864;G:3;C:5;T:14744;total:15616 | iSNV |
| F50 | F50-8 | 9446 | NS5 | 0.9965 | A:56;G:15836;C:0;T:0;total:15892 | SNP |
| F50 | F50-8 | 10420 | 3'-UTR | 0.0331 | A:0;G:0;C:355;T:10349;total:10704 | iSNV |
| F50 | F50-8 | 10428 | 3'-UTR | 0.1149 | A:2;G:3;C:9576;T:1244;total:10825 | iSNV |
| F50 | F50-9 | 563 | M | 0.9897 | A:18100;G:189;C:0;T:1;total:18290 | SNP |
| F50 | F50-9 | 1055 | E | 0.2572 | A:10484;G:3633;C:3;T:1;total:14121 | iSNV |
| F50 | F50-9 | 1257 | E | 0.9899 | A:18755;G:0;C:190;T:2;total:18947 | SNP |
| F50 | F50-9 | 1447 | E | 0.071 | A:1567;G:0;C:20500;T:2;total:22069 | iSNV |
| F50 | F50-9 | 1512 | E | 0.6819 | A:5648;G:12106;C:1;T:0;total:17755 | iSNV |
| F50 | F50-9 | 1772 | E | 0.9905 | A:5;G:19653;C:2;T:183;total:19843 | SNP |
| F50 | F50-9 | 2372 | E | 0.9924 | A:1;G:1;C:93;T:12305;total:12400 | SNP |
| F50 | F50-9 | 2493 | NS1 | 0.9909 | A:122;G:13500;C:0;T:3;total:13625 | SNP |
| F50 | F50-9 | 2504 | NS1 | 0.9903 | A:125;G:12847;C:0;T:2;total:12974 | SNP |
| F50 | F50-9 | 2531 | NS1 | 0.9892 | A:1;G:0;C:13820;T:150;total:13971 | SNP |
| F50 | F50-9 | 3399 | NS1 | 0.284 | A:11853;G:4704;C:2;T:0;total:16559 | iSNV |
| F50 | F50-9 | 3572 | NS1 | 0.9897 | A:0;G:1;C:144;T:13823;total:13968 | SNP |
| F50 | F50-9 | 3962 | NS2A | 0.9891 | A:0;G:2;C:216;T:19651;total:19869 | SNP |
| F50 | F50-9 | 4155 | NS2A | 0.9905 | A:8549;G:1;C:2;T:79;total:8631 | SNP |
| F50 | F50-9 | 4595 | NS2B | 0.0703 | A:19944;G:1510;C:5;T:0;total:21459 | iSNV |
| F50 | F50-9 | 4712 | NS3 | 0.9893 | A:23036;G:249;C:2;T:0;total:23287 | SNP |
| F50 | F50-9 | 5057 | NS3 | 0.0243 | A:4;G:0;C:17877;T:446;total:18327 | iSNV |
| F50 | F50-9 | 5311 | NS3 | 0.9791 | A:1;G:3;C:315;T:14733;total:15052 | iSNV |
| F50 | F50-9 | 6080 | NS3 | 0.9898 | A:18112;G:187;C:1;T:0;total:18300 | SNP |
| F50 | F50-9 | 6971 | NS4A | 0.0278 | A:0;G:15124;C:434;T:42;total:15600 | iSNV |
| F50 | F50-9 | 8282 | NS5 | 0.9976 | A:9;G:0;C:20597;T:41;total:20647 | SNP |
| F50 | F50-9 | 8396 | NS5 | 0.3989 | A:14698;G:9757;C:3;T:0;total:24458 | iSNV |
| F50 | F50-9 | 8846 | NS5 | 0.0428 | A:15563;G:696;C:0;T:0;total:16259 | iSNV |
| F50 | F50-9 | 8900 | NS5 | 0.9884 | A:202;G:1;C:17486;T:4;total:17693 | SNP |
| F50 | F50-9 | 9446 | NS5 | 0.9872 | A:369;G:28471;C:1;T:2;total:28843 | SNP |
| F50 | F50-9 | 10010 | NS5 | 0.0822 | A:0;G:0;C:1388;T:15485;total:16873 | iSNV |
| F50 | F50-9 | 10428 | 3'-UTR | 0.0499 | A:2;G:1;C:18443;T:970;total:19416 | iSNV |
| F55 | F55-1 | 446 | C | 0.1181 | A:3;G:5;C:4649;T:624;total:5281 | iSNV |
| F55 | F55-1 | 563 | M | 0.9678 | A:4499;G:150;C:0;T:0;total:4649 | iSNV |
| F55 | F55-1 | 755 | M | 0.0213 | A:0;G:0;C:5178;T:113;total:5291 | iSNV |
| F55 | F55-1 | 1257 | E | 0.9664 | A:4169;G:0;C:145;T:0;total:4314 | iSNV |
| F55 | F55-1 | 1428 | E | 0.0448 | A:5554;G:261;C:0;T:0;total:5815 | iSNV |
| F55 | F55-1 | 1431 | E | 0.057 | A:0;G:0;C:5473;T:331;total:5804 | iSNV |
| F55 | F55-1 | 1772 | E | 0.9715 | A:3;G:4865;C:0;T:143;total:5011 | iSNV |
| F55 | F55-1 | 2060 | E | 0.0245 | A:1;G:0;C:3541;T:89;total:3631 | iSNV |
| F55 | F55-1 | 2076 | E | 0.3574 | A:2311;G:1286;C:1;T:0;total:3598 | iSNV |
| F55 | F55-1 | 2369 | E | 0.1744 | A:615;G:2884;C:26;T:1;total:3526 | iSNV |
| F55 | F55-1 | 2372 | E | 0.972 | A:0;G:0;C:98;T:3398;total:3496 | iSNV |
| F55 | F55-1 | 2493 | NS1 | 0.9685 | A:110;G:3377;C:1;T:0;total:3488 | iSNV |
| F55 | F55-1 | 2498 | NS1 | 0.1284 | A:0;G:0;C:3034;T:447;total:3481 | iSNV |
| F55 | F55-1 | 2504 | NS1 | 0.9667 | A:112;G:3244;C:0;T:0;total:3356 | iSNV |
| F55 | F55-1 | 2531 | NS1 | 0.9679 | A:1;G:0;C:3459;T:115;total:3575 | iSNV |
| F55 | F55-1 | 3257 | NS1 | 0.026 | A:0;G:0;C:134;T:5003;total:5137 | iSNV |
| F55 | F55-1 | 3572 | NS1 | 0.9679 | A:0;G:0;C:118;T:3554;total:3672 | iSNV |
| F55 | F55-1 | 3602 | NS1 | 0.1758 | A:0;G:655;C:3069;T:0;total:3724 | iSNV |
| F55 | F55-1 | 3869 | NS2A | 0.0229 | A:1;G:1;C:4171;T:98;total:4271 | iSNV |
| F55 | F55-1 | 3908 | NS2A | 0.1363 | A:1;G:0;C:3927;T:620;total:4548 | iSNV |
| F55 | F55-1 | 3962 | NS2A | 0.9622 | A:1;G:0;C:157;T:3988;total:4146 | iSNV |
| F55 | F55-1 | 4155 | NS2A | 0.9707 | A:2447;G:0;C:0;T:74;total:2521 | iSNV |

| | | | | | | |
|---|---|---|---|---|---|---|
| F55 | F55-1 | 4233 | NS2B | 0.0529 | A:0;G:144;C:2;T:2576;total:2722 | iSNV |
| F55 | F55-1 | 4712 | NS3 | 0.9571 | A:4721;G:212;C:0;T:0;total:4933 | iSNV |
| F55 | F55-1 | 5311 | NS3 | 0.9636 | A:0;G:3;C:138;T:3641;total:3782 | iSNV |
| F55 | F55-1 | 5790 | NS3 | 0.172 | A:0;G:0;C:881;T:4241;total:5122 | iSNV |
| F55 | F55-1 | 5835 | NS3 | 0.1576 | A:796;G:4252;C:0;T:0;total:5048 | iSNV |
| F55 | F55-1 | 6062 | NS3 | 0.0233 | A:0;G:0;C:4567;T:109;total:4676 | iSNV |
| F55 | F55-1 | 6080 | NS3 | 0.9644 | A:4143;G:153;C:1;T:0;total:4297 | iSNV |
| F55 | F55-1 | 6236 | NS3 | 0.0387 | A:0;G:0;C:4088;T:165;total:4253 | iSNV |
| F55 | F55-1 | 6425 | NS3 | 0.0603 | A:220;G:3423;C:0;T:0;total:3643 | iSNV |
| F55 | F55-1 | 7633 | NS4B | 0.0254 | A:1;G:0;C:3947;T:103;total:4051 | iSNV |
| F55 | F55-1 | 8073 | NS5 | 0.0375 | A:0;G:186;C:3;T:4758;total:4947 | iSNV |
| F55 | F55-1 | 8130 | NS5 | 0.1111 | A:0;G:0;C:4784;T:598;total:5382 | iSNV |
| F55 | F55-1 | 8282 | NS5 | 0.9726 | A:0;G:0;C:4283;T:121;total:4404 | iSNV |
| F55 | F55-1 | 8900 | NS5 | 0.9638 | A:132;G:3;C:3501;T:3;total:3639 | iSNV |
| F55 | F55-1 | 9446 | NS5 | 0.9642 | A:216;G:5803;C:0;T:0;total:6019 | iSNV |
| F55 | F55-1 | 10014 | NS5 | 0.108 | A:3680;G:0;C:0;T:446;total:4126 | iSNV |
| F55 | F55-1 | 10428 | 3'-UTR | 0.0758 | A:0;G:0;C:4291;T:352;total:4643 | iSNV |
| F55 | F55-10 | 395 | C | 0.0201 | A:23979;G:494;C:1;T:1;total:24475 | iSNV |
| F55 | F55-10 | 518 | M | 0.0538 | A:5;G:0;C:22017;T:1254;total:23276 | iSNV |
| F55 | F55-10 | 563 | M | 0.9949 | A:17048;G:87;C:1;T:1;total:17137 | SNP |
| F55 | F55-10 | 951 | M | 0.0217 | A:345;G:0;C:15485;T:5;total:15835 | iSNV |
| F55 | F55-10 | 1116 | E | 0.4867 | A:8248;G:7823;C:0;T:1;total:16072 | iSNV |
| F55 | F55-10 | 1257 | E | 0.9946 | A:16670;G:6;C:86;T:0;total:16762 | SNP |
| F55 | F55-10 | 1772 | E | 0.995 | A:1;G:19016;C:1;T:94;total:19112 | SNP |
| F55 | F55-10 | 1799 | E | 0.5137 | A:0;G:1;C:9846;T:10397;total:20244 | iSNV |
| F55 | F55-10 | 2067 | E | 0.0356 | A:0;G:0;C:444;T:12008;total:12452 | iSNV |
| F55 | F55-10 | 2147 | E | 0.0384 | A:12;G:13928;C:0;T:558;total:14498 | iSNV |
| F55 | F55-10 | 2231 | E | 0.0382 | A:15122;G:4;C:2;T:602;total:15730 | iSNV |
| F55 | F55-10 | 2362 | E | 0.0285 | A:2;G:11222;C:1;T:330;total:11555 | iSNV |
| F55 | F55-10 | 2372 | E | 0.9961 | A:3;G:1;C:43;T:11855;total:11902 | SNP |
| F55 | F55-10 | 2493 | NS1 | 0.9936 | A:74;G:11559;C:0;T:1;total:11634 | SNP |
| F55 | F55-10 | 2504 | NS1 | 0.9933 | A:76;G:11116;C:0;T:0;total:11192 | SNP |
| F55 | F55-10 | 2531 | NS1 | 0.9924 | A:2;G:0;C:12161;T:92;total:12255 | SNP |
| F55 | F55-10 | 2700 | NS1 | 0.0524 | A:0;G:4;C:992;T:17929;total:18925 | iSNV |
| F55 | F55-10 | 3572 | NS1 | 0.9948 | A:0;G:0;C:69;T:13140;total:13209 | SNP |
| F55 | F55-10 | 3704 | NS1 | 0.0374 | A:0;G:0;C:402;T:10321;total:10723 | iSNV |
| F55 | F55-10 | 3875 | NS2A | 0.0413 | A:4;G:0;C:16745;T:723;total:17472 | iSNV |
| F55 | F55-10 | 3962 | NS2A | 0.9939 | A:0;G:2;C:101;T:16741;total:16844 | SNP |
| F55 | F55-10 | 4155 | NS2A | 0.9915 | A:8073;G:1;C:0;T:69;total:8143 | SNP |
| F55 | F55-10 | 4712 | NS3 | 0.9939 | A:19415;G:118;C:3;T:0;total:19536 | SNP |
| F55 | F55-10 | 5311 | NS3 | 0.9909 | A:1;G:17;C:112;T:14069;total:14199 | SNP |
| F55 | F55-10 | 6080 | NS3 | 0.9944 | A:16082;G:91;C:0;T:0;total:16173 | SNP |
| F55 | F55-10 | 6971 | NS4A | 0.127 | A:1;G:12414;C:1808;T:5;total:14228 | iSNV |
| F55 | F55-10 | 7264 | NS4A | 0.0688 | A:3;G:0;C:9461;T:700;total:10164 | iSNV |
| F55 | F55-10 | 7657 | NS4B | 0.055 | A:15208;G:886;C:0;T:0;total:16094 | iSNV |
| F55 | F55-10 | 8045 | NS5 | 0.0971 | A:1889;G:17557;C:0;T:3;total:19449 | iSNV |
| F55 | F55-10 | 8282 | NS5 | 0.9935 | A:6;G:1;C:17611;T:110;total:17728 | SNP |
| F55 | F55-10 | 8844 | NS5 | 0.0247 | A:344;G:13531;C:1;T:0;total:13876 | iSNV |
| F55 | F55-10 | 8900 | NS5 | 0.9915 | A:125;G:0;C:14966;T:4;total:15095 | SNP |
| F55 | F55-10 | 9370 | NS5 | 0.5314 | A:13157;G:4;C:0;T:11609;total:24770 | iSNV |
| F55 | F55-10 | 9446 | NS5 | 0.9946 | A:137;G:26050;C:2;T:3;total:26192 | SNP |
| F55 | F55-10 | 10069 | NS5 | 0.0573 | A:1;G:0;C:12910;T:785;total:13696 | iSNV |
| F55 | F55-10 | 10071 | NS5 | 0.1295 | A:1779;G:1;C:2;T:11952;total:13734 | iSNV |
| F55 | F55-10 | 10388 | NS5 | 0.0265 | A:5;G:0;C:18795;T:513;total:19313 | iSNV |
| F55 | F55-10 | 10428 | 3'-UTR | 0.7362 | A:3;G:3;C:4733;T:13202;total:17941 | iSNV |
| F55 | F55-11 | 909 | M | 0.0241 | A:1;G:0;C:6068;T:150;total:6219 | iSNV |
| F55 | F55-11 | 1218 | E | 0.9927 | A:0;G:0;C:48;T:6506;total:6554 | SNP |
| F55 | F55-11 | 1431 | E | 0.2427 | A:0;G:0;C:5847;T:1874;total:7721 | iSNV |
| F55 | F55-11 | 1512 | E | 0.0278 | A:6173;G:177;C:8;T:0;total:6358 | iSNV |
| F55 | F55-11 | 1514 | E | 0.148 | A:5432;G:0;C:944;T:1;total:6377 | iSNV |
| F55 | F55-11 | 1551 | E | 0.5211 | A:1;G:3081;C:2834;T:1;total:5917 | iSNV |
| F55 | F55-11 | 1797 | E | 0.2583 | A:2003;G:109;C:0;T:5642;total:7754 | iSNV |
| F55 | F55-11 | 2597 | NS1 | 0.0425 | A:5945;G:0;C:0;T:264;total:6209 | iSNV |
| F55 | F55-11 | 2805 | NS1 | 0.025 | A:8262;G:212;C:0;T:0;total:8474 | iSNV |
| F55 | F55-11 | 3257 | NS1 | 0.0258 | A:0;G:1;C:196;T:7391;total:7588 | iSNV |
| F55 | F55-11 | 3261 | NS1 | 0.1712 | A:6124;G:1266;C:1;T:0;total:7391 | iSNV |
| F55 | F55-11 | 3697 | NS1 | 0.495 | A:1;G:3;C:2248;T:2208;total:4460 | iSNV |
| F55 | F55-11 | 3869 | NS2A | 0.9952 | A:1;G:0;C:31;T:6502;total:6534 | SNP |
| F55 | F55-11 | 4233 | NS2B | 0.538 | A:0;G:2077;C:0;T:1784;total:3861 | iSNV |
| F55 | F55-11 | 4295 | NS2B | 0.1662 | A:4735;G:0;C:0;T:944;total:5679 | iSNV |
| F55 | F55-11 | 4447 | NS2B | 0.1327 | A:4201;G:643;C:1;T:0;total:4845 | iSNV |
| F55 | F55-11 | 5256 | NS3 | 0.5177 | A:1;G:2;C:2904;T:3113;total:6020 | iSNV |
| F55 | F55-11 | 5276 | NS3 | 0.0207 | A:5998;G:127;C:0;T:0;total:6125 | iSNV |
| F55 | F55-11 | 5736 | NS3 | 0.5101 | A:4054;G:0;C:3895;T:0;total:7949 | iSNV |
| F55 | F55-11 | 6320 | NS3 | 0.0345 | A:0;G:0;C:193;T:5400;total:5593 | iSNV |
| F55 | F55-11 | 6867 | NS4A | 0.2544 | A:1453;G:4256;C:1;T:0;total:5710 | iSNV |
| F55 | F55-11 | 6900 | NS4A | 0.5391 | A:3045;G:2606;C:0;T:3;total:5654 | iSNV |
| F55 | F55-11 | 6985 | NS4A | 0.0361 | A:215;G:5738;C:0;T:2;total:5955 | iSNV |
| F55 | F55-11 | 7264 | NS4A | 0.5013 | A:1;G:0;C:2050;T:2059;total:4110 | iSNV |
| F55 | F55-11 | 7310 | NS4B | 0.0259 | A:3977;G:106;C:0;T:0;total:4083 | iSNV |
| F55 | F55-11 | 7633 | NS4B | 0.4656 | A:1;G:0;C:3568;T:3110;total:6679 | iSNV |
| F55 | F55-11 | 7657 | NS4B | 0.0934 | A:6079;G:627;C:1;T:0;total:6707 | iSNV |

| | | | | | | |
|---|---|---|---|---|---|---|
| F55 | F55-11 | 7904 | NS5 | 0.0305 | A:7210;G:227;C:0;T:0;total:7437 | iSNV |
| F55 | F55-11 | 8948 | NS5 | 0.2419 | A:1655;G:5185;C:0;T:1;total:6841 | iSNV |
| F55 | F55-11 | 9342 | NS5 | 0.0593 | A:0;G:1;C:8208;T:518;total:8727 | iSNV |
| F55 | F55-11 | 9370 | NS5 | 0.0313 | A:283;G:0;C:4;T:8733;total:9020 | iSNV |
| F55 | F55-11 | 9880 | NS5 | 0.2672 | A:1794;G:4918;C:0;T:0;total:6712 | iSNV |
| F55 | F55-11 | 9899 | NS5 | 0.1362 | A:3;G:0;C:5316;T:839;total:6158 | iSNV |
| F55 | F55-11 | 10428 | 3'-UTR | 0.9224 | A:2;G:1;C:488;T:5795;total:6286 | iSNV |
| F55 | F55-11 | 10568 | 3'-UTR | 0.029 | A:1;G:0;C:5817;T:174;total:5992 | iSNV |
| F55 | F55-11 | 10589 | 3'-UTR | 0.27 | A:0;G:1704;C:0;T:4605;total:6309 | iSNV |
| F55 | F55-11 | 10860 | 3'-UTR | 0.1419 | A:413;G:0;C:2496;T:0;total:2909 | iSNV |
| F55 | F55-12 | 332 | C | 0.0952 | A:11762;G:1238;C:0;T:2;total:13002 | iSNV |
| F55 | F55-12 | 353 | C | 0.5742 | A:5014;G:6759;C:0;T:2;total:11775 | iSNV |
| F55 | F55-12 | 645 | M | 0.6329 | A:2964;G:5108;C:0;T:2;total:8074 | iSNV |
| F55 | F55-12 | 869 | M | 0.1665 | A:2;G:1489;C:0;T:7449;total:8940 | iSNV |
| F55 | F55-12 | 998 | E | 0.645 | A:1;G:2;C:2620;T:4756;total:7379 | iSNV |
| F55 | F55-12 | 1083 | E | 0.1378 | A:5720;G:1;C:915;T:1;total:6637 | iSNV |
| F55 | F55-12 | 1117 | E | 0.6513 | A:2713;G:5064;C:0;T:2;total:7779 | iSNV |
| F55 | F55-12 | 1131 | E | 0.4342 | A:0;G:3538;C:4609;T:0;total:8147 | iSNV |
| F55 | F55-12 | 1218 | E | 0.336 | A:3;G:0;C:5122;T:2594;total:7719 | iSNV |
| F55 | F55-12 | 1428 | E | 0.0313 | A:8869;G:287;C:0;T:0;total:9156 | iSNV |
| F55 | F55-12 | 1439 | E | 0.1325 | A:0;G:3;C:1177;T:7698;total:8878 | iSNV |
| F55 | F55-12 | 1447 | E | 0.097 | A:897;G:0;C:8339;T:2;total:9238 | iSNV |
| F55 | F55-12 | 1453 | E | 0.0315 | A:10;G:3;C:285;T:8749;total:9047 | iSNV |
| F55 | F55-12 | 1512 | E | 0.1567 | A:6344;G:1181;C:10;T:0;total:7535 | iSNV |
| F55 | F55-12 | 1551 | E | 0.2125 | A:5;G:1496;C:5537;T:1;total:7039 | iSNV |
| F55 | F55-12 | 1640 | E | 0.1345 | A:946;G:0;C:6073;T:10;total:7029 | iSNV |
| F55 | F55-12 | 2057 | E | 0.0228 | A:1;G:0;C:6037;T:141;total:6179 | iSNV |
| F55 | F55-12 | 2261 | E | 0.0472 | A:1;G:0;C:6691;T:332;total:7024 | iSNV |
| F55 | F55-12 | 2277 | E | 0.6384 | A:1;G:0;C:4489;T:2544;total:7034 | iSNV |
| F55 | F55-12 | 2362 | E | 0.0862 | A:0;G:4798;C:453;T:2;total:5253 | iSNV |
| F55 | F55-12 | 2942 | NS1 | 0.0386 | A:2;G:0;C:8334;T:335;total:8671 | iSNV |
| F55 | F55-12 | 3869 | NS2A | 0.4232 | A:2;G:0;C:5680;T:4170;total:9852 | iSNV |
| F55 | F55-12 | 3959 | NS2A | 0.0865 | A:0;G:1;C:9483;T:899;total:10383 | iSNV |
| F55 | F55-12 | 4068 | NS2A | 0.0244 | A:7571;G:3;C:0;T:190;total:7764 | iSNV |
| F55 | F55-12 | 4070 | NS2A | 0.1237 | A:0;G:968;C:3;T:6853;total:7824 | iSNV |
| F55 | F55-12 | 4293 | NS2B | 0.3904 | A:3167;G:4945;C:0;T:0;total:8112 | iSNV |
| F55 | F55-12 | 4974 | NS3 | 0.3804 | A:3083;G:5019;C:1;T:1;total:8104 | iSNV |
| F55 | F55-12 | 5256 | NS3 | 0.1966 | A:1;G:0;C:6500;T:1591;total:8092 | iSNV |
| F55 | F55-12 | 5266 | NS3 | 0.0941 | A:1;G:2;C:7380;T:767;total:8150 | iSNV |
| F55 | F55-12 | 5537 | NS3 | 0.3274 | A:6484;G:3158;C:0;T:1;total:9643 | iSNV |
| F55 | F55-12 | 5736 | NS3 | 0.1836 | A:1913;G:0;C:8501;T:1;total:10415 | iSNV |
| F55 | F55-12 | 5813 | NS3 | 0.1223 | A:4;G:0;C:8721;T:1216;total:9941 | iSNV |
| F55 | F55-12 | 6900 | NS4A | 0.1862 | A:1431;G:6247;C:2;T:3;total:7683 | iSNV |
| F55 | F55-12 | 6969 | NS4A | 0.1144 | A:7184;G:929;C:1;T:0;total:8114 | iSNV |
| F55 | F55-12 | 7055 | NS4A | 0.0558 | A:0;G:0;C:7351;T:435;total:7786 | iSNV |
| F55 | F55-12 | 7067 | NS4A | 0.0261 | A:8148;G:219;C:0;T:0;total:8367 | iSNV |
| F55 | F55-12 | 7178 | NS4A | 0.1096 | A:0;G:0;C:5884;T:725;total:6609 | iSNV |
| F55 | F55-12 | 7264 | NS4A | 0.1732 | A:1;G:0;C:4839;T:1014;total:5854 | iSNV |
| F55 | F55-12 | 7478 | NS4B | 0.1197 | A:1;G:0;C:4610;T:627;total:5238 | iSNV |
| F55 | F55-12 | 7561 | NS4B | 0.1137 | A:2;G:3;C:896;T:6977;total:7878 | iSNV |
| F55 | F55-12 | 7633 | NS4B | 0.285 | A:0;G:0;C:5853;T:2334;total:8187 | iSNV |
| F55 | F55-12 | 8546 | NS5 | 0.0223 | A:2;G:0;C:8965;T:205;total:9172 | iSNV |
| F55 | F55-12 | 8603 | NS5 | 0.0417 | A:7393;G:322;C:3;T:0;total:7718 | iSNV |
| F55 | F55-12 | 9023 | NS5 | 0.0976 | A:0;G:1;C:626;T:5784;total:6411 | iSNV |
| F55 | F55-12 | 9185 | NS5 | 0.0247 | A:208;G:8198;C:0;T:0;total:8406 | iSNV |
| F55 | F55-12 | 9245 | NS5 | 0.05 | A:1;G:0;C:8837;T:466;total:9304 | iSNV |
| F55 | F55-12 | 9699 | NS5 | 0.4002 | A:0;G:0;C:4652;T:3104;total:7756 | iSNV |
| F55 | F55-12 | 10259 | NS5 | 0.5696 | A:4608;G:6097;C:0;T:0;total:10705 | iSNV |
| F55 | F55-12 | 10347 | NS5 | 0.3983 | A:2;G:1;C:6228;T:4126;total:10357 | iSNV |
| F55 | F55-12 | 10428 | 3'-UTR | 0.6262 | A:3;G:1;C:3205;T:5365;total:8574 | iSNV |
| F55 | F55-12 | 10447 | 3'-UTR | 0.5829 | A:2;G:0;C:3145;T:4393;total:7540 | iSNV |
| F55 | F55-13 | 395 | C | 0.9556 | A:4829;G:103879;C:4;T:5;total:108717 | iSNV |
| F55 | F55-13 | 530 | M | 0.118 | A:5;G:2;C:75368;T:10091;total:85466 | iSNV |
| F55 | F55-13 | 1113 | E | 0.4411 | A:38445;G:0;C:30359;T:13;total:68817 | iSNV |
| F55 | F55-13 | 1175 | E | 0.1095 | A:2;G:1;C:8726;T:70900;total:79629 | iSNV |
| F55 | F55-13 | 1218 | E | 0.9982 | A:8;G:0;C:116;T:68122;total:68246 | SNP |
| F55 | F55-13 | 1413 | E | 0.9643 | A:3145;G:84890;C:8;T:13;total:88056 | iSNV |
| F55 | F55-13 | 1512 | E | 0.3396 | A:45356;G:23337;C:7;T:1;total:68701 | iSNV |
| F55 | F55-13 | 1563 | E | 0.0589 | A:1;G:1;C:60064;T:3765;total:63831 | iSNV |
| F55 | F55-13 | 1708 | E | 0.0286 | A:2157;G:73178;C:0;T:7;total:75342 | iSNV |
| F55 | F55-13 | 1769 | E | 0.1088 | A:3;G:9;C:9080;T:74313;total:83405 | iSNV |
| F55 | F55-13 | 1786 | E | 0.0674 | A:3;G:4;C:5983;T:82729;total:88719 | iSNV |
| F55 | F55-13 | 1789 | E | 0.0594 | A:4;G:4;C:5322;T:84248;total:89578 | iSNV |
| F55 | F55-13 | 1796 | E | 0.432 | A:4;G:50696;C:87;T:38628;total:89415 | iSNV |
| F55 | F55-13 | 1797 | E | 0.0381 | A:3;G:3422;C:32;T:86264;total:89721 | iSNV |
| F55 | F55-13 | 2367 | E | 0.1112 | A:39504;G:4944;C:0;T:5;total:44453 | iSNV |
| F55 | F55-13 | 2543 | NS1 | 0.0618 | A:1;G:0;C:51481;T:3394;total:54876 | iSNV |
| F55 | F55-13 | 2786 | NS1 | 0.1572 | A:5;G:0;C:78481;T:14645;total:93131 | iSNV |
| F55 | F55-13 | 3049 | NS1 | 0.0257 | A:57209;G:1514;C:9;T:1;total:58733 | iSNV |
| F55 | F55-13 | 3869 | NS2A | 0.9987 | A:1;G:4;C:98;T:78818;total:78921 | SNP |
| F55 | F55-13 | 3906 | NS2A | 0.3368 | A:29463;G:57986;C:9;T:5;total:87463 | iSNV |
| F55 | F55-13 | 4447 | NS2B | 0.0949 | A:48702;G:5108;C:8;T:7;total:53825 | iSNV |

| | | | | | | |
|---|---|---|---|---|---|---|
| F55 | F55-13 | 4592 | NS2B | 0.0258 | A:82069;G:2177;C:3;T:24;total:84273 | iSNV |
| F55 | F55-13 | 4632 | NS3 | 0.1497 | A:13;G:14437;C:23;T:81910;total:96383 | iSNV |
| F55 | F55-13 | 4792 | NS3 | 0.0747 | A:79511;G:6424;C:0;T:3;total:85938 | iSNV |
| F55 | F55-13 | 4896 | NS3 | 0.0345 | A:2939;G:81997;C:4;T:5;total:84945 | iSNV |
| F55 | F55-13 | 5140 | NS3 | 0.023 | A:0;G:0;C:66980;T:1580;total:68560 | iSNV |
| F55 | F55-13 | 5291 | NS3 | 0.0632 | A:62416;G:4217;C:4;T:6;total:66643 | iSNV |
| F55 | F55-13 | 5736 | NS3 | 0.0212 | A:1962;G:0;C:90374;T:11;total:92347 | iSNV |
| F55 | F55-13 | 6900 | NS4A | 0.0213 | A:212;G:57155;C:6;T:1252;total:58625 | iSNV |
| F55 | F55-13 | 6934 | NS4A | 0.1426 | A:55044;G:9161;C:2;T:0;total:64207 | iSNV |
| F55 | F55-13 | 6969 | NS4A | 0.4473 | A:35505;G:7;C:28749;T:6;total:64267 | iSNV |
| F55 | F55-13 | 6970 | NS4A | 0.0989 | A:57987;G:30;C:6371;T:4;total:64392 | iSNV |
| F55 | F55-13 | 7005 | NS4A | 0.0884 | A:59243;G:5762;C:3;T:171;total:65179 | iSNV |
| F55 | F55-13 | 7256 | NS4A | 0.0266 | A:1210;G:44233;C:0;T:4;total:45447 | iSNV |
| F55 | F55-13 | 7595 | NS4B | 0.4383 | A:30647;G:2;C:10;T:39257;total:69916 | iSNV |
| F55 | F55-13 | 7633 | NS4B | 0.1871 | A:2;G:2;C:64723;T:14902;total:79629 | iSNV |
| F55 | F55-13 | 7682 | NS5 | 0.0255 | A:2025;G:77340;C:2;T:5;total:79372 | iSNV |
| F55 | F55-13 | 7903 | NS5 | 0.0443 | A:76846;G:3568;C:1;T:1;total:80416 | iSNV |
| F55 | F55-13 | 9266 | NS5 | 0.0277 | A:2497;G:87588;C:0;T:2;total:90087 | iSNV |
| F55 | F55-13 | 9482 | NS5 | 0.0266 | A:2063;G:75203;C:2;T:8;total:77276 | iSNV |
| F55 | F55-13 | 9690 | NS5 | 0.0837 | A:5137;G:56193;C:6;T:3;total:61339 | iSNV |
| F55 | F55-13 | 10376 | NS5 | 0.9531 | A:77314;G:3812;C:1;T:5;total:81132 | iSNV |
| F55 | F55-13 | 10428 | 3'-UTR | 0.9094 | A:3;G:2;C:6577;T:65996;total:72578 | iSNV |
| F55 | F55-13 | 10579 | 3'-UTR | 0.4396 | A:6;G:4;C:36724;T:28823;total:65557 | iSNV |
| F55 | F55-14 | 516 | M | 0.5589 | A:43798;G:55480;C:5;T:4;total:99287 | iSNV |
| F55 | F55-14 | 638 | M | 0.0346 | A:4;G:3;C:3411;T:95125;total:98543 | iSNV |
| F55 | F55-14 | 686 | M | 0.0822 | A:3;G:2;C:92679;T:8303;total:100987 | iSNV |
| F55 | F55-14 | 794 | M | 0.1135 | A:26;G:12;C:9040;T:70559;total:79637 | iSNV |
| F55 | F55-14 | 836 | M | 0.5874 | A:7;G:0;C:48132;T:33824;total:81963 | iSNV |
| F55 | F55-14 | 998 | E | 0.9406 | A:5;G:2;C:4554;T:71992;total:76553 | iSNV |
| F55 | F55-14 | 1057 | E | 0.0716 | A:5312;G:68854;C:1;T:4;total:74171 | iSNV |
| F55 | F55-14 | 1067 | E | 0.0212 | A:1;G:1;C:78777;T:1707;total:80486 | iSNV |
| F55 | F55-14 | 1117 | E | 0.0412 | A:81900;G:3523;C:0;T:6;total:85429 | iSNV |
| F55 | F55-14 | 1218 | E | 0.998 | A:35;G:5;C:128;T:83787;total:83955 | SNP |
| F55 | F55-14 | 1343 | E | 0.0796 | A:3;G:0;C:91649;T:7936;total:99588 | iSNV |
| F55 | F55-14 | 1512 | E | 0.788 | A:18313;G:68041;C:0;T:5;total:86359 | iSNV |
| F55 | F55-14 | 1769 | E | 0.0784 | A:7944;G:5;C:40;T:93260;total:101249 | iSNV |
| F55 | F55-14 | 2369 | E | 0.0456 | A:2671;G:55790;C:1;T:62;total:58524 | iSNV |
| F55 | F55-14 | 2481 | NS1 | 0.0457 | A:58131;G:2787;C:1;T:0;total:60919 | iSNV |
| F55 | F55-14 | 2972 | NS1 | 0.0301 | A:5;G:1;C:90189;T:2803;total:92998 | iSNV |
| F55 | F55-14 | 3167 | NS1 | 0.0207 | A:3;G:1;C:89538;T:1899;total:91441 | iSNV |
| F55 | F55-14 | 3386 | NS1 | 0.0847 | A:1;G:2;C:65610;T:6076;total:71689 | iSNV |
| F55 | F55-14 | 3536 | NS1 | 0.0433 | A:9;G:0;C:59909;T:2718;total:62636 | iSNV |
| F55 | F55-14 | 3669 | NS1 | 0.0215 | A:14;G:1;C:61614;T:1356;total:62985 | iSNV |
| F55 | F55-14 | 3731 | NS2A | 0.0753 | A:56300;G:15;C:4591;T:3;total:60909 | iSNV |
| F55 | F55-14 | 3869 | NS2A | 0.1268 | A:5;G:0;C:75671;T:10990;total:86666 | iSNV |
| F55 | F55-14 | 3938 | NS2A | 0.0206 | A:89304;G:1885;C:1;T:9;total:91199 | iSNV |
| F55 | F55-14 | 3950 | NS2A | 0.0777 | A:56;G:0;C:85907;T:7243;total:93206 | iSNV |
| F55 | F55-14 | 4043 | NS2A | 0.0411 | A:2;G:5;C:64192;T:2752;total:66951 | iSNV |
| F55 | F55-14 | 4274 | NS2B | 0.0465 | A:1;G:14;C:2763;T:56606;total:59384 | iSNV |
| F55 | F55-14 | 4289 | NS2B | 0.0286 | A:4;G:1934;C:42;T:65454;total:67434 | iSNV |
| F55 | F55-14 | 4294 | NS2B | 0.0368 | A:66229;G:2532;C:1;T:5;total:68767 | iSNV |
| F55 | F55-14 | 4389 | NS2B | 0.0747 | A:5560;G:68838;C:1;T:2;total:74401 | iSNV |
| F55 | F55-14 | 4697 | NS3 | 0.9415 | A:4;G:0;C:94712;T:5894;total:100610 | iSNV |
| F55 | F55-14 | 4974 | NS3 | 0.0815 | A:6954;G:78284;C:0;T:3;total:85241 | iSNV |
| F55 | F55-14 | 5330 | NS3 | 0.0307 | A:66712;G:2120;C:12;T:3;total:68847 | iSNV |
| F55 | F55-14 | 5558 | NS3 | 0.0438 | A:4106;G:89585;C:1;T:21;total:93713 | iSNV |
| F55 | F55-14 | 5616 | NS3 | 0.0296 | A:2492;G:3;C:81497;T:51;total:84043 | iSNV |
| F55 | F55-14 | 5822 | NS3 | 0.0428 | A:5;G:3;C:4168;T:93157;total:97333 | iSNV |
| F55 | F55-14 | 5864 | NS3 | 0.1108 | A:7;G:10;C:10465;T:83911;total:94393 | iSNV |
| F55 | F55-14 | 5952 | NS3 | 0.9411 | A:6;G:0;C:5699;T:90962;total:96667 | iSNV |
| F55 | F55-14 | 6010 | NS3 | 0.0441 | A:89354;G:4126;C:3;T:2;total:93485 | iSNV |
| F55 | F55-14 | 6292 | NS3 | 0.0289 | A:71518;G:2135;C:9;T:2;total:73664 | iSNV |
| F55 | F55-14 | 6867 | NS4A | 0.7609 | A:55393;G:17410;C:0;T:4;total:72807 | iSNV |
| F55 | F55-14 | 7528 | NS4B | 0.0471 | A:3;G:0;C:66454;T:3291;total:69748 | iSNV |
| F55 | F55-14 | 7561 | NS4B | 0.0694 | A:0;G:10;C:5549;T:74312;total:79871 | iSNV |
| F55 | F55-14 | 7633 | NS4B | 0.8513 | A:1;G:4;C:13068;T:74764;total:87837 | iSNV |
| F55 | F55-14 | 8114 | NS5 | 0.0439 | A:3;G:3;C:90182;T:4149;total:94337 | iSNV |
| F55 | F55-14 | 8884 | NS5 | 0.0256 | A:70065;G:1841;C:1;T:3;total:71910 | iSNV |
| F55 | F55-14 | 9068 | NS5 | 0.0828 | A:4;G:6;C:5723;T:63369;total:69102 | iSNV |
| F55 | F55-14 | 9359 | NS5 | 0.9446 | A:11;G:4;C:103929;T:6106;total:110050 | iSNV |
| F55 | F55-14 | 9370 | NS5 | 0.9449 | A:31;G:105298;C:2;T:6147;total:111478 | iSNV |
| F55 | F55-14 | 9737 | NS5 | 0.032 | A:1;G:4;C:78975;T:2614;total:81594 | iSNV |
| F55 | F55-14 | 9923 | NS5 | 0.0433 | A:11;G:3;C:3730;T:82307;total:86051 | iSNV |
| F55 | F55-14 | 10259 | NS5 | 0.9443 | A:5950;G:100786;C:1;T:6;total:106743 | iSNV |
| F55 | F55-14 | 10419 | 3'-UTR | 0.0887 | A:4;G:1;C:76900;T:7487;total:84392 | iSNV |
| F55 | F55-14 | 10428 | 3'-UTR | 0.4187 | A:4;G:5;C:49817;T:35903;total:85729 | iSNV |
| F55 | F55-14 | 10435 | 3'-UTR | 0.0713 | A:76924;G:5915;C:17;T:4;total:82860 | iSNV |
| F55 | F55-14 | 10566 | 3'-UTR | 0.9352 | A:9;G:2;C:4645;T:66995;total:71651 | iSNV |
| F55 | F55-15 | 401 | C | 0.0223 | A:90893;G:2079;C:13;T:3;total:92988 | iSNV |
| F55 | F55-15 | 803 | M | 0.316 | A:3;G:39086;C:2;T:18062;total:57153 | iSNV |
| F55 | F55-15 | 810 | M | 0.2681 | A:8;G:2;C:42775;T:15679;total:58464 | iSNV |
| F55 | F55-15 | 943 | M | 0.0308 | A:6;G:1;C:59743;T:1904;total:61654 | iSNV |

| | | | | | | |
|---|---|---|---|---|---|---|
| F55 | F55-15 | 998 | E | 0.2089 | A:5;G:0;C:47020;T:12421;total:59446 | iSNV |
| F55 | F55-15 | 1218 | E | 0.9992 | A:13;G:0;C:41;T:64003;total:64057 | SNP |
| F55 | F55-15 | 1428 | E | 0.3505 | A:50993;G:27528;C:1;T:11;total:78533 | iSNV |
| F55 | F55-15 | 1512 | E | 0.3335 | A:45366;G:22712;C:2;T:5;total:68085 | iSNV |
| F55 | F55-15 | 1672 | E | 0.0287 | A:12;G:0;C:61920;T:1831;total:63763 | iSNV |
| F55 | F55-15 | 1797 | E | 0.1917 | A:12;G:16118;C:23;T:67924;total:84077 | iSNV |
| F55 | F55-15 | 1847 | E | 0.026 | A:73833;G:1972;C:4;T:5;total:75814 | iSNV |
| F55 | F55-15 | 1914 | E | 0.0205 | A:52451;G:1101;C:0;T:3;total:53555 | iSNV |
| F55 | F55-15 | 2076 | E | 0.0292 | A:51873;G:1562;C:7;T:1;total:53443 | iSNV |
| F55 | F55-15 | 2241 | E | 0.0259 | A:9;G:59646;C:1589;T:5;total:61249 | iSNV |
| F55 | F55-15 | 2493 | NS1 | 0.3716 | A:32427;G:19186;C:4;T:5;total:51622 | iSNV |
| F55 | F55-15 | 2625 | NS1 | 0.061 | A:4;G:0;C:64855;T:4216;total:69075 | iSNV |
| F55 | F55-15 | 3161 | NS1 | 0.0843 | A:68530;G:6315;C:8;T:4;total:74857 | iSNV |
| F55 | F55-15 | 3396 | NS1 | 0.2714 | A:6;G:17221;C:8;T:46217;total:63452 | iSNV |
| F55 | F55-15 | 3455 | NS1 | 0.118 | A:44064;G:5900;C:13;T:16;total:49993 | iSNV |
| F55 | F55-15 | 3869 | NS2A | 0.4223 | A:19;G:0;C:41700;T:30502;total:72221 | iSNV |
| F55 | F55-15 | 4233 | NS2B | 0.0489 | A:2;G:1975;C:6;T:38383;total:40366 | iSNV |
| F55 | F55-15 | 4402 | NS2B | 0.0937 | A:54103;G:4;C:5;T:5596;total:59708 | iSNV |
| F55 | F55-15 | 4697 | NS3 | 0.2797 | A:13;G:5;C:23887;T:61482;total:85387 | iSNV |
| F55 | F55-15 | 4974 | NS3 | 0.0264 | A:30;G:74157;C:2019;T:14;total:76220 | iSNV |
| F55 | F55-15 | 5312 | NS3 | 0.0229 | A:1451;G:31;C:42;T:61761;total:63285 | iSNV |
| F55 | F55-15 | 5598 | NS3 | 0.0257 | A:1789;G:67715;C:31;T:15;total:69550 | iSNV |
| F55 | F55-15 | 5736 | NS3 | 0.0295 | A:2594;G:0;C:85197;T:17;total:87808 | iSNV |
| F55 | F55-15 | 5952 | NS3 | 0.2911 | A:8;G:0;C:58563;T:24052;total:82623 | iSNV |
| F55 | F55-15 | 5996 | NS3 | 0.2926 | A:3;G:3;C:49409;T:20441;total:69856 | iSNV |
| F55 | F55-15 | 6122 | NS3 | 0.2542 | A:17447;G:51159;C:3;T:5;total:68614 | iSNV |
| F55 | F55-15 | 6252 | NS3 | 0.026 | A:0;G:2;C:1737;T:64917;total:66656 | iSNV |
| F55 | F55-15 | 6714 | NS4A | 0.0354 | A:2199;G:59777;C:0;T:7;total:61983 | iSNV |
| F55 | F55-15 | 6867 | NS4A | 0.2744 | A:16668;G:44066;C:0;T:4;total:60738 | iSNV |
| F55 | F55-15 | 7167 | NS4A | 0.0558 | A:3855;G:65107;C:9;T:2;total:68973 | iSNV |
| F55 | F55-15 | 7528 | NS4B | 0.1005 | A:4;G:2;C:53241;T:5954;total:59201 | iSNV |
| F55 | F55-15 | 7561 | NS4B | 0.0225 | A:8;G:11;C:1514;T:65717;total:67250 | iSNV |
| F55 | F55-15 | 7575 | NS4B | 0.0634 | A:59339;G:4022;C:5;T:6;total:63372 | iSNV |
| F55 | F55-15 | 7633 | NS4B | 0.6883 | A:4;G:3;C:23393;T:51631;total:75031 | iSNV |
| F55 | F55-15 | 7727 | NS5 | 0.0201 | A:7;G:2;C:1454;T:70592;total:72055 | iSNV |
| F55 | F55-15 | 7735 | NS5 | 0.2801 | A:18925;G:48612;C:5;T:6;total:67548 | iSNV |
| F55 | F55-15 | 8518 | NS5 | 0.2728 | A:64248;G:12;C:1;T:24117;total:88378 | iSNV |
| F55 | F55-15 | 9245 | NS5 | 0.0287 | A:6;G:2;C:90492;T:2678;total:93178 | iSNV |
| F55 | F55-15 | 9284 | NS5 | 0.0309 | A:5;G:1;C:2756;T:86326;total:89088 | iSNV |
| F55 | F55-15 | 9293 | NS5 | 0.0276 | A:2301;G:80870;C:2;T:6;total:83179 | iSNV |
| F55 | F55-15 | 9359 | NS5 | 0.277 | A:14;G:1;C:25938;T:67683;total:93636 | iSNV |
| F55 | F55-15 | 9370 | NS5 | 0.2743 | A:26094;G:147;C:14;T:68849;total:95104 | iSNV |
| F55 | F55-15 | 9443 | NS5 | 0.0295 | A:95881;G:2924;C:0;T:5;total:98810 | iSNV |
| F55 | F55-15 | 10259 | NS5 | 0.2913 | A:64523;G:26526;C:0;T:4;total:91053 | iSNV |
| F55 | F55-15 | 10295 | NS5 | 0.1819 | A:11;G:3;C:76991;T:17125;total:94130 | iSNV |
| F55 | F55-15 | 10428 | 3'-UTR | 0.3412 | A:19;G:2;C:50017;T:25924;total:75962 | iSNV |
| F55 | F55-15 | 10451 | 3'-UTR | 0.033 | A:20;G:3;C:65191;T:2226;total:67440 | iSNV |
| F55 | F55-15 | 10566 | 3'-UTR | 0.2665 | A:14;G:0;C:46897;T:17046;total:63957 | iSNV |
| F55 | F55-15 | 10718 | 3'-UTR | 0.0256 | A:5;G:0;C:75654;T:1992;total:77651 | iSNV |
| F55 | F55-16 | 738 | M | 0.0292 | A:63059;G:25;C:1903;T:4;total:64991 | iSNV |
| F55 | F55-16 | 803 | M | 0.3479 | A:2;G:31227;C:2;T:16663;total:47894 | iSNV |
| F55 | F55-16 | 810 | M | 0.4016 | A:2;G:0;C:29273;T:19648;total:48923 | iSNV |
| F55 | F55-16 | 923 | M | 0.0223 | A:32;G:0;C:49234;T:1125;total:50391 | iSNV |
| F55 | F55-16 | 998 | E | 0.0433 | A:8;G:1;C:48530;T:2198;total:50737 | iSNV |
| F55 | F55-16 | 1218 | E | 0.9988 | A:20;G:0;C:49;T:54428;total:54497 | SNP |
| F55 | F55-16 | 1244 | E | 0.0226 | A:3;G:1;C:57019;T:1323;total:58346 | iSNV |
| F55 | F55-16 | 1428 | E | 0.4346 | A:36966;G:28426;C:1;T:3;total:65396 | iSNV |
| F55 | F55-16 | 1432 | E | 0.0397 | A:62992;G:9;C:11;T:2605;total:65617 | iSNV |
| F55 | F55-16 | 1447 | E | 0.0219 | A:1470;G:0;C:65515;T:18;total:67003 | iSNV |
| F55 | F55-16 | 1459 | E | 0.047 | A:3011;G:5;C:61002;T:39;total:64057 | iSNV |
| F55 | F55-16 | 1512 | E | 0.3462 | A:36709;G:19448;C:1;T:2;total:56160 | iSNV |
| F55 | F55-16 | 1797 | E | 0.0307 | A:5;G:2248;C:8;T:70805;total:73066 | iSNV |
| F55 | F55-16 | 2264 | E | 0.0261 | A:0;G:2;C:1247;T:46416;total:47665 | iSNV |
| F55 | F55-16 | 2493 | NS1 | 0.4604 | A:23405;G:19981;C:0;T:9;total:43395 | iSNV |
| F55 | F55-16 | 2625 | NS1 | 0.0203 | A:4;G:0;C:60146;T:1250;total:61400 | iSNV |
| F55 | F55-16 | 3178 | NS1 | 0.0377 | A:68989;G:7;C:3;T:2706;total:71705 | iSNV |
| F55 | F55-16 | 3396 | NS1 | 0.3068 | A:7;G:16977;C:6;T:38330;total:55320 | iSNV |
| F55 | F55-16 | 3508 | NS1 | 0.0271 | A:6;G:2;C:40929;T:1141;total:42078 | iSNV |
| F55 | F55-16 | 3869 | NS2A | 0.5316 | A:3;G:4;C:30851;T:34994;total:65852 | iSNV |
| F55 | F55-16 | 4109 | NS2A | 0.0254 | A:40764;G:1064;C:1;T:1;total:41830 | iSNV |
| F55 | F55-16 | 4233 | NS2B | 0.02 | A:4;G:730;C:4;T:35633;total:36371 | iSNV |
| F55 | F55-16 | 4294 | NS2B | 0.0261 | A:51096;G:1372;C:1;T:2;total:52471 | iSNV |
| F55 | F55-16 | 4697 | NS3 | 0.1443 | A:3;G:2;C:11194;T:66363;total:77562 | iSNV |
| F55 | F55-16 | 5736 | NS3 | 0.1068 | A:8852;G:1;C:74009;T:3;total:82865 | iSNV |
| F55 | F55-16 | 5885 | NS3 | 0.1306 | A:1;G:8;C:9454;T:62897;total:72360 | iSNV |
| F55 | F55-16 | 5952 | NS3 | 0.1461 | A:6;G:0;C:65043;T:11133;total:76182 | iSNV |
| F55 | F55-16 | 5996 | NS3 | 0.3612 | A:9;G:1;C:39659;T:22436;total:62105 | iSNV |
| F55 | F55-16 | 6122 | NS3 | 0.2992 | A:18484;G:43284;C:0;T:7;total:61775 | iSNV |
| F55 | F55-16 | 6494 | NS4A | 0.02 | A:3;G:0;C:1311;T:64101;total:65415 | iSNV |
| F55 | F55-16 | 6714 | NS4A | 0.0305 | A:1676;G:53103;C:2;T:3;total:54784 | iSNV |
| F55 | F55-16 | 6729 | NS4A | 0.1353 | A:7358;G:1;C:46925;T:94;total:54378 | iSNV |
| F55 | F55-16 | 6867 | NS4A | 0.1585 | A:8652;G:45925;C:0;T:7;total:54584 | iSNV |

| | | | | | | |
|---|---|---|---|---|---|---|
| F55 | F55-16 | 7116 | NS4A | 0.0236 | A:3;G:0;C:57276;T:1389;total:58668 | iSNV |
| F55 | F55-16 | 7167 | NS4A | 0.0616 | A:3731;G:56802;C:3;T:0;total:60536 | iSNV |
| F55 | F55-16 | 7260 | NS4A | 0.0207 | A:3;G:0;C:40497;T:860;total:41360 | iSNV |
| F55 | F55-16 | 7528 | NS4B | 0.1006 | A:3;G:0;C:48909;T:5471;total:54383 | iSNV |
| F55 | F55-16 | 7626 | NS4B | 0.02 | A:1414;G:10;C:32;T:68977;total:70433 | iSNV |
| F55 | F55-16 | 7633 | NS4B | 0.6679 | A:6;G:3;C:23268;T:46773;total:70050 | iSNV |
| F55 | F55-16 | 7735 | NS5 | 0.299 | A:18886;G:44258;C:4;T:3;total:63151 | iSNV |
| F55 | F55-16 | 8518 | NS5 | 0.298 | A:55746;G:8;C:10;T:23679;total:79443 | iSNV |
| F55 | F55-16 | 9122 | NS5 | 0.0936 | A:59107;G:6109;C:0;T:3;total:65219 | iSNV |
| F55 | F55-16 | 9293 | NS5 | 0.1029 | A:7812;G:68052;C:2;T:5;total:75871 | iSNV |
| F55 | F55-16 | 9342 | NS5 | 0.0441 | A:6;G:7;C:82714;T:3818;total:86545 | iSNV |
| F55 | F55-16 | 9359 | NS5 | 0.1417 | A:6;G:1;C:12486;T:75611;total:88104 | iSNV |
| F55 | F55-16 | 9370 | NS5 | 0.3721 | A:33119;G:22;C:67;T:55781;total:88989 | iSNV |
| F55 | F55-16 | 9818 | NS5 | 0.021 | A:2;G:0;C:1313;T:61159;total:62474 | iSNV |
| F55 | F55-16 | 10259 | NS5 | 0.1465 | A:68968;G:11843;C:0;T:3;total:80814 | iSNV |
| F55 | F55-16 | 10295 | NS5 | 0.0308 | A:8;G:0;C:80392;T:2556;total:82956 | iSNV |
| F55 | F55-16 | 10428 | 3'-UTR | 0.6703 | A:7;G:1;C:21520;T:43729;total:65257 | iSNV |
| F55 | F55-16 | 10566 | 3'-UTR | 0.1392 | A:6;G:1;C:48503;T:7847;total:56357 | iSNV |
| F55 | F55-17 | 281 | C | 0.0286 | A:16;G:0;C:65297;T:1928;total:67241 | iSNV |
| F55 | F55-17 | 645 | M | 0.3831 | A:30071;G:18681;C:4;T:3;total:48759 | iSNV |
| F55 | F55-17 | 761 | M | 0.0287 | A:14;G:55062;C:1633;T:8;total:56717 | iSNV |
| F55 | F55-17 | 1057 | E | 0.2308 | A:9364;G:31201;C:0;T:1;total:40566 | iSNV |
| F55 | F55-17 | 1218 | E | 0.9986 | A:8;G:0;C:56;T:44515;total:44579 | SNP |
| F55 | F55-17 | 1428 | E | 0.2799 | A:40500;G:15752;C:4;T:3;total:56259 | iSNV |
| F55 | F55-17 | 1447 | E | 0.3674 | A:20535;G:4;C:35339;T:6;total:55884 | iSNV |
| F55 | F55-17 | 1568 | E | 0.0634 | A:4;G:0;C:40752;T:2761;total:43517 | iSNV |
| F55 | F55-17 | 1577 | E | 0.0547 | A:12;G:44029;C:1;T:2550;total:46592 | iSNV |
| F55 | F55-17 | 1790 | E | 0.0227 | A:1389;G:59736;C:4;T:1;total:61130 | iSNV |
| F55 | F55-17 | 1976 | E | 0.142 | A:4;G:1;C:33376;T:5526;total:38907 | iSNV |
| F55 | F55-17 | 2070 | E | 0.3776 | A:22848;G:13869;C:1;T:3;total:36721 | iSNV |
| F55 | F55-17 | 2276 | E | 0.0683 | A:6;G:0;C:36258;T:2659;total:38923 | iSNV |
| F55 | F55-17 | 2376 | E | 0.3769 | A:1;G:1;C:21106;T:12773;total:33881 | iSNV |
| F55 | F55-17 | 2960 | NS1 | 0.2815 | A:4;G:1;C:37283;T:14613;total:51901 | iSNV |
| F55 | F55-17 | 3669 | NS1 | 0.0952 | A:2;G:3;C:35226;T:3711;total:38942 | iSNV |
| F55 | F55-17 | 3869 | NS2A | 0.999 | A:2;G:0;C:50;T:48645;total:48697 | SNP |
| F55 | F55-17 | 4233 | NS2B | 0.0949 | A:4;G:2852;C:1;T:27176;total:30033 | iSNV |
| F55 | F55-17 | 4447 | NS2B | 0.3911 | A:23244;G:14935;C:1;T:5;total:38185 | iSNV |
| F55 | F55-17 | 4974 | NS3 | 0.068 | A:3492;G:47795;C:0;T:5;total:51292 | iSNV |
| F55 | F55-17 | 5415 | NS3 | 0.2458 | A:4;G:5;C:38663;T:12610;total:51282 | iSNV |
| F55 | F55-17 | 5567 | NS3 | 0.0334 | A:7;G:1;C:1782;T:51476;total:53266 | iSNV |
| F55 | F55-17 | 5575 | NS3 | 0.2102 | A:2;G:0;C:41551;T:11065;total:52618 | iSNV |
| F55 | F55-17 | 5705 | NS3 | 0.2102 | A:44469;G:11842;C:1;T:5;total:56317 | iSNV |
| F55 | F55-17 | 5718 | NS3 | 0.2559 | A:15059;G:43767;C:3;T:3;total:58832 | iSNV |
| F55 | F55-17 | 5765 | NS3 | 0.027 | A:0;G:0;C:58751;T:1631;total:60382 | iSNV |
| F55 | F55-17 | 5793 | NS3 | 0.0364 | A:10;G:4;C:58183;T:2200;total:60397 | iSNV |
| F55 | F55-17 | 5831 | NS3 | 0.0502 | A:4;G:0;C:56156;T:2973;total:59133 | iSNV |
| F55 | F55-17 | 5918 | NS3 | 0.1224 | A:6544;G:46890;C:2;T:2;total:53438 | iSNV |
| F55 | F55-17 | 6063 | NS3 | 0.0623 | A:3;G:0;C:48764;T:3243;total:52010 | iSNV |
| F55 | F55-17 | 6635 | NS4A | 0.0322 | A:2;G:0;C:1091;T:32781;total:33874 | iSNV |
| F55 | F55-17 | 6970 | NS4A | 0.5049 | A:24131;G:1;C:24581;T:22;total:48735 | iSNV |
| F55 | F55-17 | 7151 | NS4A | 0.4415 | A:1;G:0;C:28200;T:22299;total:50500 | iSNV |
| F55 | F55-17 | 7264 | NS4A | 0.2269 | A:1;G:1;C:26484;T:7776;total:34262 | iSNV |
| F55 | F55-17 | 7413 | NS4B | 0.0293 | A:19;G:0;C:36316;T:1099;total:37434 | iSNV |
| F55 | F55-17 | 7481 | NS4B | 0.0815 | A:2;G:3;C:2580;T:29046;total:31631 | iSNV |
| F55 | F55-17 | 7483 | NS4B | 0.0482 | A:30880;G:1566;C:2;T:0;total:32448 | iSNV |
| F55 | F55-17 | 7633 | NS4B | 0.9988 | A:3;G:2;C:58;T:51049;total:51112 | SNP |
| F55 | F55-17 | 8430 | NS5 | 0.021 | A:1;G:0;C:59435;T:1277;total:60713 | iSNV |
| F55 | F55-17 | 10364 | NS5 | 0.423 | A:1;G:2;C:32586;T:23896;total:56485 | iSNV |
| F55 | F55-17 | 10420 | 3'-UTR | 0.1023 | A:1;G:4;C:5377;T:47165;total:52547 | iSNV |
| F55 | F55-17 | 10428 | 3'-UTR | 0.9293 | A:10;G:0;C:3721;T:48868;total:52599 | iSNV |
| F55 | F55-17 | 10447 | 3'-UTR | 0.2721 | A:2;G:1;C:33780;T:12634;total:46417 | iSNV |
| F55 | F55-18 | 998 | E | 0.4735 | A:3;G:0;C:5985;T:5386;total:11374 | iSNV |
| F55 | F55-18 | 1218 | E | 0.996 | A:0;G:1;C:48;T:12018;total:12067 | SNP |
| F55 | F55-18 | 1330 | E | 0.0247 | A:14337;G:364;C:1;T:1;total:14703 | iSNV |
| F55 | F55-18 | 1413 | E | 0.468 | A:8184;G:7203;C:1;T:2;total:15390 | iSNV |
| F55 | F55-18 | 1512 | E | 0.444 | A:7089;G:18;C:5679;T:2;total:12788 | iSNV |
| F55 | F55-18 | 1562 | E | 0.4861 | A:5952;G:5633;C:1;T:0;total:11586 | iSNV |
| F55 | F55-18 | 1797 | E | 0.5082 | A:32;G:7730;C:0;T:7512;total:15274 | iSNV |
| F55 | F55-18 | 2181 | E | 0.203 | A:10629;G:2708;C:0;T:2;total:13339 | iSNV |
| F55 | F55-18 | 3032 | NS1 | 0.0229 | A:1;G:0;C:10381;T:244;total:10626 | iSNV |
| F55 | F55-18 | 3289 | NS1 | 0.0223 | A:13262;G:303;C:2;T:1;total:13568 | iSNV |
| F55 | F55-18 | 3440 | NS1 | 0.0251 | A:10061;G:260;C:2;T:1;total:10324 | iSNV |
| F55 | F55-18 | 3869 | NS2A | 0.5403 | A:2;G:0;C:6473;T:7605;total:14080 | iSNV |
| F55 | F55-18 | 4091 | NS2A | 0.4281 | A:4440;G:5929;C:0;T:1;total:10370 | iSNV |
| F55 | F55-18 | 4262 | NS2B | 0.0248 | A:5;G:0;C:9435;T:241;total:9681 | iSNV |
| F55 | F55-18 | 4447 | NS2B | 0.0361 | A:10065;G:377;C:0;T:0;total:10442 | iSNV |
| F55 | F55-18 | 4458 | NS2B | 0.1222 | A:1123;G:8060;C:0;T:3;total:9186 | iSNV |
| F55 | F55-18 | 4697 | NS3 | 0.4331 | A:2;G:4;C:7038;T:9203;total:16247 | iSNV |
| F55 | F55-18 | 5072 | NS3 | 0.1828 | A:2252;G:10062;C:1;T:2;total:12317 | iSNV |
| F55 | F55-18 | 5365 | NS3 | 0.2112 | A:9715;G:2602;C:1;T:0;total:12318 | iSNV |
| F55 | F55-18 | 5707 | NS3 | 0.1415 | A:14103;G:2326;C:1;T:0;total:16430 | iSNV |
| F55 | F55-18 | 5952 | NS3 | 0.4362 | A:1;G:0;C:8817;T:6824;total:15642 | iSNV |

| | | | | | | |
|---|---|---|---|---|---|---|
| F55 | F55-18 | 6322 | NS3 | 0.1506 | A:2;G:0;C:9976;T:1770;total:11748 | iSNV |
| F55 | F55-18 | 6740 | NS4A | 0.1773 | A:0;G:2;C:10015;T:2159;total:12176 | iSNV |
| F55 | F55-18 | 6867 | NS4A | 0.2392 | A:2916;G:9269;C:1;T:0;total:12186 | iSNV |
| F55 | F55-18 | 6900 | NS4A | 0.0507 | A:610;G:11405;C:0;T:3;total:12018 | iSNV |
| F55 | F55-18 | 6927 | NS4A | 0.1576 | A:4;G:2;C:10433;T:1953;total:12392 | iSNV |
| F55 | F55-18 | 7091 | NS4A | 0.1373 | A:2;G:0;C:10629;T:1692;total:12323 | iSNV |
| F55 | F55-18 | 7561 | NS4B | 0.4241 | A:1;G:2;C:5464;T:7415;total:12882 | iSNV |
| F55 | F55-18 | 7633 | NS4B | 0.5268 | A:0;G:2;C:6487;T:7219;total:13708 | iSNV |
| F55 | F55-18 | 7658 | NS4B | 0.0227 | A:3;G:1;C:304;T:13036;total:13344 | iSNV |
| F55 | F55-18 | 7836 | NS5 | 0.2278 | A:2;G:2796;C:9469;T:3;total:12270 | iSNV |
| F55 | F55-18 | 8336 | NS5 | 0.0231 | A:1;G:4;C:386;T:16280;total:16671 | iSNV |
| F55 | F55-18 | 8430 | NS5 | 0.4736 | A:1;G:4;C:8222;T:7402;total:15629 | iSNV |
| F55 | F55-18 | 8550 | NS5 | 0.0458 | A:16917;G:5;C:0;T:814;total:17736 | iSNV |
| F55 | F55-18 | 9359 | NS5 | 0.4333 | A:1;G:2;C:7668;T:10023;total:17694 | iSNV |
| F55 | F55-18 | 9764 | NS5 | 0.2657 | A:2;G:0;C:10346;T:3746;total:14094 | iSNV |
| F55 | F55-18 | 10259 | NS5 | 0.4367 | A:9643;G:7478;C:0;T:1;total:17122 | iSNV |
| F55 | F55-18 | 10295 | NS5 | 0.4338 | A:2;G:3;C:9970;T:7643;total:17618 | iSNV |
| F55 | F55-18 | 10376 | NS5 | 0.4994 | A:7340;G:7353;C:0;T:2;total:14695 | iSNV |
| F55 | F55-18 | 10419 | 3'-UTR | 0.0292 | A:17;G:0;C:12621;T:381;total:13019 | iSNV |
| F55 | F55-18 | 10428 | 3'-UTR | 0.664 | A:4;G:1;C:4317;T:8524;total:12846 | iSNV |
| F55 | F55-18 | 10451 | 3'-UTR | 0.1924 | A:3;G:1;C:9326;T:2223;total:11553 | iSNV |
| F55 | F55-18 | 10566 | 3'-UTR | 0.4208 | A:3;G:0;C:6641;T:4827;total:11471 | iSNV |
| F55 | F55-19 | 869 | M | 0.0823 | A:1226;G:2;C:0;T:13664;total:14892 | iSNV |
| F55 | F55-19 | 998 | E | 0.7155 | A:18;G:1;C:3385;T:8494;total:11898 | iSNV |
| F55 | F55-19 | 1218 | E | 0.9959 | A:3;G:5;C:46;T:13043;total:13097 | SNP |
| F55 | F55-19 | 1363 | E | 0.2426 | A:1;G:0;C:11223;T:3597;total:14821 | iSNV |
| F55 | F55-19 | 1382 | E | 0.1003 | A:0;G:1;C:14212;T:1586;total:15799 | iSNV |
| F55 | F55-19 | 1428 | E | 0.7156 | A:4323;G:10875;C:1;T:0;total:15199 | iSNV |
| F55 | F55-19 | 1430 | E | 0.2001 | A:0;G:1;C:12406;T:3104;total:15511 | iSNV |
| F55 | F55-19 | 1568 | E | 0.023 | A:5;G:0;C:10054;T:237;total:10296 | iSNV |
| F55 | F55-19 | 1672 | E | 0.0466 | A:1;G:0;C:9936;T:486;total:10423 | iSNV |
| F55 | F55-19 | 1797 | E | 0.0951 | A:1448;G:857;C:4;T:12906;total:15215 | iSNV |
| F55 | F55-19 | 2183 | E | 0.0575 | A:762;G:12468;C:0;T:0;total:13230 | iSNV |
| F55 | F55-19 | 2318 | E | 0.0525 | A:1;G:0;C:572;T:10308;total:10881 | iSNV |
| F55 | F55-19 | 2467 | E | 0.0225 | A:8784;G:203;C:1;T:1;total:8989 | iSNV |
| F55 | F55-19 | 3052 | NS1 | 0.0462 | A:0;G:1;C:480;T:9888;total:10369 | iSNV |
| F55 | F55-19 | 3663 | NS1 | 0.0295 | A:3;G:0;C:9730;T:296;total:10029 | iSNV |
| F55 | F55-19 | 3821 | NS2A | 0.0212 | A:5;G:0;C:12314;T:267;total:12586 | iSNV |
| F55 | F55-19 | 3869 | NS2A | 0.2772 | A:7;G:0;C:10249;T:3934;total:14190 | iSNV |
| F55 | F55-19 | 4122 | NS2A | 0.0824 | A:1;G:7121;C:0;T:640;total:7762 | iSNV |
| F55 | F55-19 | 4538 | NS2B | 0.0996 | A:4;G:0;C:11538;T:1277;total:12819 | iSNV |
| F55 | F55-19 | 4697 | NS3 | 0.7077 | A:4;G:0;C:11284;T:4664;total:15952 | iSNV |
| F55 | F55-19 | 4896 | NS3 | 0.0218 | A:363;G:16234;C:0;T:0;total:16597 | iSNV |
| F55 | F55-19 | 4974 | NS3 | 0.0731 | A:1031;G:13067;C:0;T:0;total:14098 | iSNV |
| F55 | F55-19 | 5261 | NS3 | 0.0572 | A:10601;G:644;C:1;T:1;total:11247 | iSNV |
| F55 | F55-19 | 5313 | NS3 | 0.7087 | A:1;G:1;C:8296;T:3411;total:11709 | iSNV |
| F55 | F55-19 | 5336 | NS3 | 0.0632 | A:0;G:4;C:760;T:11255;total:12019 | iSNV |
| F55 | F55-19 | 5353 | NS3 | 0.0851 | A:11844;G:1103;C:0;T:0;total:12947 | iSNV |
| F55 | F55-19 | 5598 | NS3 | 0.0955 | A:1302;G:12322;C:6;T:3;total:13633 | iSNV |
| F55 | F55-19 | 5952 | NS3 | 0.7078 | A:0;G:3;C:4657;T:11274;total:15934 | iSNV |
| F55 | F55-19 | 6413 | NS3 | 0.0742 | A:1;G:1;C:892;T:11126;total:12020 | iSNV |
| F55 | F55-19 | 6779 | NS4A | 0.0436 | A:11884;G:543;C:0;T:2;total:12429 | iSNV |
| F55 | F55-19 | 6867 | NS4A | 0.0641 | A:704;G:10269;C:0;T:1;total:10974 | iSNV |
| F55 | F55-19 | 6900 | NS4A | 0.1016 | A:1095;G:9679;C:0;T:1;total:10775 | iSNV |
| F55 | F55-19 | 7378 | NS4B | 0.0506 | A:2;G:0;C:9167;T:489;total:9658 | iSNV |
| F55 | F55-19 | 7585 | NS4B | 0.6271 | A:0;G:4609;C:7749;T:0;total:12358 | iSNV |
| F55 | F55-19 | 7633 | NS4B | 0.2767 | A:0;G:1;C:10218;T:3910;total:14129 | iSNV |
| F55 | F55-19 | 8756 | NS5 | 0.0326 | A:10089;G:340;C:0;T:0;total:10429 | iSNV |
| F55 | F55-19 | 9290 | NS5 | 0.0382 | A:10;G:1;C:14682;T:584;total:15277 | iSNV |
| F55 | F55-19 | 9359 | NS5 | 0.7113 | A:2;G:0;C:13072;T:5307;total:18381 | iSNV |
| F55 | F55-19 | 9688 | NS5 | 0.0773 | A:2;G:0;C:11222;T:941;total:12165 | iSNV |
| F55 | F55-19 | 9818 | NS5 | 0.0684 | A:1;G:0;C:903;T:12297;total:13201 | iSNV |
| F55 | F55-19 | 10058 | NS5 | 0.0476 | A:0;G:0;C:10645;T:533;total:11178 | iSNV |
| F55 | F55-19 | 10217 | NS5 | 0.0299 | A:5;G:0;C:15072;T:465;total:15542 | iSNV |
| F55 | F55-19 | 10249 | NS5 | 0.0644 | A:1;G:1;C:16043;T:1106;total:17151 | iSNV |
| F55 | F55-19 | 10259 | NS5 | 0.7064 | A:5083;G:12223;C:1;T:2;total:17309 | iSNV |
| F55 | F55-19 | 10358 | NS5 | 0.1077 | A:4;G:0;C:14072;T:1700;total:15776 | iSNV |
| F55 | F55-19 | 10428 | 3'-UTR | 0.9567 | A:0;G:2;C:575;T:12675;total:13252 | iSNV |
| F55 | F55-19 | 10566 | 3'-UTR | 0.6919 | A:2;G:1;C:3215;T:7216;total:10434 | iSNV |
| F55 | F55-2 | 563 | M | 0.9876 | A:13528;G:165;C:4;T:1;total:13698 | SNP |
| F55 | F55-2 | 1257 | E | 0.9844 | A:12362;G:2;C:194;T:0;total:12558 | SNP |
| F55 | F55-2 | 1447 | E | 0.8189 | A:13136;G:2;C:2907;T:0;total:16045 | iSNV |
| F55 | F55-2 | 1512 | E | 0.0335 | A:14070;G:488;C:4;T:0;total:14562 | iSNV |
| F55 | F55-2 | 1772 | E | 0.9853 | A:7;G:13999;C:1;T:202;total:14209 | SNP |
| F55 | F55-2 | 2372 | E | 0.9816 | A:1;G:0;C:189;T:10112;total:10302 | SNP |
| F55 | F55-2 | 2493 | NS1 | 0.9873 | A:129;G:10199;C:0;T:3;total:10331 | SNP |
| F55 | F55-2 | 2504 | NS1 | 0.9869 | A:126;G:9658;C:0;T:3;total:9787 | SNP |
| F55 | F55-2 | 2531 | NS1 | 0.9856 | A:1;G:0;C:10046;T:146;total:10193 | SNP |
| F55 | F55-2 | 3572 | NS1 | 0.9872 | A:0;G:3;C:134;T:10490;total:10627 | SNP |
| F55 | F55-2 | 3962 | NS2A | 0.9803 | A:1;G:0;C:232;T:11561;total:11794 | SNP |
| F55 | F55-2 | 4155 | NS2A | 0.9884 | A:7807;G:3;C:1;T:88;total:7899 | SNP |
| F55 | F55-2 | 4712 | NS3 | 0.9827 | A:13295;G:234;C:0;T:1;total:13530 | SNP |

| | | | | | | |
|---|---|---|---|---|---|---|
| F55 | F55-2 | 5283 | NS3 | 0.0328 | A:368;G:0;C:10823;T:1;total:11192 | iSNV |
| F55 | F55-2 | 5311 | NS3 | 0.9846 | A:0;G:5;C:160;T:10520;total:10685 | SNP |
| F55 | F55-2 | 5537 | NS3 | 0.1249 | A:12316;G:1759;C:1;T:4;total:14080 | iSNV |
| F55 | F55-2 | 6080 | NS3 | 0.9854 | A:11855;G:175;C:1;T:0;total:12031 | SNP |
| F55 | F55-2 | 8282 | NS5 | 0.9845 | A:3;G:0;C:12014;T:187;total:12204 | SNP |
| F55 | F55-2 | 8900 | NS5 | 0.9818 | A:182;G:3;C:10078;T:2;total:10265 | SNP |
| F55 | F55-2 | 9446 | NS5 | 0.9856 | A:230;G:16122;C:1;T:5;total:16358 | SNP |
| F55 | F55-2 | 9597 | NS5 | 0.0282 | A:2;G:0;C:15435;T:448;total:15885 | iSNV |
| F55 | F55-2 | 9688 | NS5 | 0.0482 | A:2;G:0;C:14926;T:757;total:15685 | iSNV |
| F55 | F55-2 | 10428 | 3'-UTR | 0.0361 | A:3;G:6;C:12674;T:476;total:13159 | iSNV |
| F55 | F55-2 | 10486 | 3'-UTR | 0.0442 | A:2;G:1;C:487;T:10516;total:11006 | iSNV |
| F55 | F55-20 | 614 | M | 0.1135 | A:1;G:10108;C:0;T:1295;total:11404 | iSNV |
| F55 | F55-20 | 906 | M | 0.1239 | A:7734;G:1094;C:0;T:0;total:8828 | iSNV |
| F55 | F55-20 | 1088 | E | 0.0323 | A:4;G:1;C:8666;T:290;total:8961 | iSNV |
| F55 | F55-20 | 1117 | E | 0.9645 | A:362;G:9816;C:0;T:2;total:10180 | iSNV |
| F55 | F55-20 | 1218 | E | 0.9906 | A:2;G:0;C:94;T:10056;total:10152 | SNP |
| F55 | F55-20 | 1295 | E | 0.0574 | A:10907;G:8;C:2;T:666;total:11583 | iSNV |
| F55 | F55-20 | 1348 | E | 0.0886 | A:11693;G:0;C:1138;T:3;total:12834 | iSNV |
| F55 | F55-20 | 1459 | E | 0.1753 | A:2245;G:0;C:10550;T:10;total:12805 | iSNV |
| F55 | F55-20 | 1571 | E | 0.1159 | A:0;G:1;C:994;T:7577;total:8572 | iSNV |
| F55 | F55-20 | 1586 | E | 0.0606 | A:1;G:0;C:7988;T:516;total:8505 | iSNV |
| F55 | F55-20 | 1721 | E | 0.9539 | A:427;G:8827;C:0;T:0;total:9254 | iSNV |
| F55 | F55-20 | 1793 | E | 0.0354 | A:443;G:12069;C:0;T:1;total:12513 | iSNV |
| F55 | F55-20 | 1909 | E | 0.1549 | A:1567;G:0;C:8544;T:3;total:10114 | iSNV |
| F55 | F55-20 | 2369 | E | 0.9587 | A:6167;G:266;C:0;T:0;total:6433 | iSNV |
| F55 | F55-20 | 2531 | NS1 | 0.2334 | A:1;G:0;C:1981;T:6502;total:8484 | iSNV |
| F55 | F55-20 | 2562 | NS1 | 0.0264 | A:202;G:7421;C:1;T:1;total:7625 | iSNV |
| F55 | F55-20 | 2858 | NS1 | 0.0315 | A:14957;G:488;C:1;T:1;total:15447 | iSNV |
| F55 | F55-20 | 3002 | NS1 | 0.0823 | A:1;G:0;C:9577;T:859;total:10437 | iSNV |
| F55 | F55-20 | 3482 | NS1 | 0.0219 | A:0;G:0;C:6381;T:143;total:6524 | iSNV |
| F55 | F55-20 | 3564 | NS1 | 0.0622 | A:0;G:0;C:8497;T:564;total:9061 | iSNV |
| F55 | F55-20 | 3593 | NS1 | 0.1055 | A:1;G:0;C:8458;T:998;total:9457 | iSNV |
| F55 | F55-20 | 3669 | NS1 | 0.0218 | A:4;G:0;C:7598;T:170;total:7772 | iSNV |
| F55 | F55-20 | 3869 | NS2A | 0.9771 | A:3;G:0;C:238;T:10135;total:10376 | iSNV |
| F55 | F55-20 | 3923 | NS2A | 0.1012 | A:0;G:1;C:1194;T:10603;total:11798 | iSNV |
| F55 | F55-20 | 3929 | NS2A | 0.9464 | A:29;G:1;C:628;T:11057;total:11715 | iSNV |
| F55 | F55-20 | 4043 | NS2A | 0.1047 | A:1;G:0;C:7423;T:869;total:8293 | iSNV |
| F55 | F55-20 | 4319 | NS2B | 0.023 | A:233;G:9853;C:0;T:2;total:10088 | iSNV |
| F55 | F55-20 | 4468 | NS2B | 0.0656 | A:492;G:0;C:0;T:6998;total:7490 | iSNV |
| F55 | F55-20 | 4627 | NS3 | 0.0577 | A:0;G:0;C:12457;T:764;total:13221 | iSNV |
| F55 | F55-20 | 4901 | NS3 | 0.0242 | A:1;G:0;C:12934;T:322;total:13257 | iSNV |
| F55 | F55-20 | 4940 | NS3 | 0.1086 | A:9799;G:1194;C:1;T:0;total:10994 | iSNV |
| F55 | F55-20 | 4974 | NS3 | 0.0273 | A:311;G:11012;C:28;T:1;total:11352 | iSNV |
| F55 | F55-20 | 5358 | NS3 | 0.1122 | A:1152;G:9107;C:1;T:1;total:10261 | iSNV |
| F55 | F55-20 | 5365 | NS3 | 0.0262 | A:9220;G:249;C:0;T:0;total:9469 | iSNV |
| F55 | F55-20 | 5376 | NS3 | 0.0206 | A:9374;G:11;C:0;T:198;total:9583 | iSNV |
| F55 | F55-20 | 5441 | NS3 | 0.1543 | A:1;G:1;C:10656;T:1946;total:12604 | iSNV |
| F55 | F55-20 | 5546 | NS3 | 0.0252 | A:4;G:0;C:12577;T:326;total:12907 | iSNV |
| F55 | F55-20 | 6059 | NS3 | 0.0814 | A:1;G:0;C:921;T:10391;total:11313 | iSNV |
| F55 | F55-20 | 6714 | NS4A | 0.0292 | A:281;G:9333;C:0;T:0;total:9614 | iSNV |
| F55 | F55-20 | 6789 | NS4A | 0.0328 | A:0;G:0;C:9472;T:322;total:9794 | iSNV |
| F55 | F55-20 | 6867 | NS4A | 0.1298 | A:1117;G:7486;C:0;T:1;total:8604 | iSNV |
| F55 | F55-20 | 6971 | NS4A | 0.0922 | A:2;G:8598;C:878;T:39;total:9517 | iSNV |
| F55 | F55-20 | 7001 | NS4A | 0.0205 | A:9065;G:0;C:190;T:0;total:9255 | iSNV |
| F55 | F55-20 | 7526 | NS4B | 0.0695 | A:1;G:0;C:565;T:7553;total:8119 | iSNV |
| F55 | F55-20 | 7585 | NS4B | 0.0341 | A:1;G:9033;C:319;T:0;total:9353 | iSNV |
| F55 | F55-20 | 7633 | NS4B | 0.8658 | A:0;G:0;C:1450;T:9354;total:10804 | iSNV |
| F55 | F55-20 | 7772 | NS5 | 0.0224 | A:9085;G:1;C:2;T:209;total:9297 | iSNV |
| F55 | F55-20 | 7990 | NS5 | 0.0221 | A:11284;G:256;C:2;T:0;total:11542 | iSNV |
| F55 | F55-20 | 9569 | NS5 | 0.0285 | A:1;G:0;C:12391;T:364;total:12756 | iSNV |
| F55 | F55-20 | 9622 | NS5 | 0.0666 | A:771;G:10793;C:0;T:3;total:11567 | iSNV |
| F55 | F55-20 | 10013 | NS5 | 0.0229 | A:3;G:0;C:9497;T:223;total:9723 | iSNV |
| F55 | F55-20 | 10109 | NS5 | 0.2252 | A:2488;G:8559;C:0;T:0;total:11047 | iSNV |
| F55 | F55-20 | 10418 | 3'-UTR | 0.1009 | A:9953;G:1118;C:0;T:1;total:11072 | iSNV |
| F55 | F55-20 | 10428 | 3'-UTR | 0.7719 | A:0;G:2;C:2483;T:8400;total:10885 | iSNV |
| F55 | F55-20 | 10447 | 3'-UTR | 0.0219 | A:5;G:0;C:9668;T:217;total:9890 | iSNV |
| F55 | F55-20 | 10594 | 3'-UTR | 0.0233 | A:233;G:9731;C:0;T:2;total:9966 | iSNV |
| F55 | F55-21 | 443 | C | 0.1525 | A:1;G:11768;C:1;T:2119;total:13889 | iSNV |
| F55 | F55-21 | 1218 | E | 0.9948 | A:1;G:0;C:48;T:9206;total:9255 | SNP |
| F55 | F55-21 | 1363 | E | 0.4127 | A:0;G:0;C:5786;T:4066;total:9852 | iSNV |
| F55 | F55-21 | 1459 | E | 0.1482 | A:1370;G:0;C:7864;T:5;total:9239 | iSNV |
| F55 | F55-21 | 1797 | E | 0.8255 | A:11;G:8273;C:1;T:1752;total:10037 | iSNV |
| F55 | F55-21 | 2362 | E | 0.1198 | A:1;G:4065;C:554;T:1;total:4621 | iSNV |
| F55 | F55-21 | 2393 | E | 0.1348 | A:0;G:0;C:4688;T:731;total:5419 | iSNV |
| F55 | F55-21 | 2556 | NS1 | 0.8205 | A:5190;G:1136;C:0;T:0;total:6326 | iSNV |
| F55 | F55-21 | 2876 | NS1 | 0.1595 | A:0;G:0;C:2466;T:12992;total:15458 | iSNV |
| F55 | F55-21 | 3230 | NS1 | 0.0411 | A:16043;G:688;C:1;T:0;total:16732 | iSNV |
| F55 | F55-21 | 3722 | NS1 | 0.0352 | A:0;G:0;C:234;T:6401;total:6635 | iSNV |
| F55 | F55-21 | 3776 | NS2A | 0.0803 | A:652;G:0;C:4;T:7462;total:8118 | iSNV |
| F55 | F55-21 | 3869 | NS2A | 0.9951 | A:1;G:6;C:55;T:12381;total:12443 | SNP |
| F55 | F55-21 | 4070 | NS2A | 0.3596 | A:0;G:3192;C:2;T:5682;total:8876 | iSNV |
| F55 | F55-21 | 6004 | NS3 | 0.0741 | A:3;G:0;C:12829;T:1027;total:13859 | iSNV |

| | | | | | | | |
|---|---|---|---|---|---|---|---|
| F55 | F55-21 | 6029 | NS3 | 0.0346 | A:2;G:1;C:12517;T:450;total:12970 | iSNV |
| F55 | F55-21 | 6089 | NS3 | 0.1449 | A:0;G:0;C:8359;T:1417;total:9776 | iSNV |
| F55 | F55-21 | 6107 | NS3 | 0.4812 | A:0;G:1;C:5382;T:4994;total:10377 | iSNV |
| F55 | F55-21 | 6336 | NS3 | 0.1437 | A:5318;G:2;C:1;T:893;total:6214 | iSNV |
| F55 | F55-21 | 6967 | NS4A | 0.0203 | A:170;G:8168;C:0;T:0;total:8338 | iSNV |
| F55 | F55-21 | 7561 | NS4B | 0.036 | A:0;G:5;C:399;T:10653;total:11057 | iSNV |
| F55 | F55-21 | 7595 | NS4B | 0.022 | A:251;G:2;C:0;T:11138;total:11391 | iSNV |
| F55 | F55-21 | 7633 | NS4B | 0.23 | A:1;G:1;C:9999;T:2988;total:12989 | iSNV |
| F55 | F55-21 | 7644 | NS4B | 0.4806 | A:6687;G:1;C:6189;T:0;total:12877 | iSNV |
| F55 | F55-21 | 7903 | NS5 | 0.1164 | A:13323;G:1756;C:1;T:0;total:15080 | iSNV |
| F55 | F55-21 | 8073 | NS5 | 0.0591 | A:0;G:727;C:2;T:11562;total:12291 | iSNV |
| F55 | F55-21 | 8237 | NS5 | 0.1379 | A:4;G:0;C:10085;T:1614;total:11703 | iSNV |
| F55 | F55-21 | 8618 | NS5 | 0.0405 | A:382;G:9034;C:0;T:2;total:9418 | iSNV |
| F55 | F55-21 | 8738 | NS5 | 0.1322 | A:8650;G:1318;C:0;T:1;total:9969 | iSNV |
| F55 | F55-21 | 9038 | NS5 | 0.1415 | A:2;G:0;C:10407;T:1716;total:12125 | iSNV |
| F55 | F55-21 | 9050 | NS5 | 0.352 | A:6810;G:3702;C:3;T:1;total:10516 | iSNV |
| F55 | F55-21 | 9065 | NS5 | 0.0717 | A:635;G:8215;C:1;T:2;total:8853 | iSNV |
| F55 | F55-21 | 9370 | NS5 | 0.0268 | A:474;G:4;C:12;T:17176;total:17666 | iSNV |
| F55 | F55-21 | 9542 | NS5 | 0.0201 | A:3;G:0;C:9996;T:206;total:10205 | iSNV |
| F55 | F55-21 | 9688 | NS5 | 0.0676 | A:0;G:0;C:6012;T:436;total:6448 | iSNV |
| F55 | F55-21 | 10334 | NS5 | 0.1544 | A:0;G:0;C:11615;T:2121;total:13736 | iSNV |
| F55 | F55-21 | 10358 | NS5 | 0.7292 | A:0;G:0;C:3402;T:9157;total:12559 | iSNV |
| F55 | F55-21 | 10428 | 3'-UTR | 0.8965 | A:1;G:0;C:955;T:8271;total:9227 | iSNV |
| F55 | F55-21 | 10451 | 3'-UTR | 0.0223 | A:2;G:0;C:7930;T:181;total:8113 | iSNV |
| F55 | F55-21 | 10470 | 3'-UTR | 0.1482 | A:6391;G:1113;C:0;T:4;total:7508 | iSNV |
| F55 | F55-21 | 10513 | 3'-UTR | 0.15 | A:0;G:0;C:1247;T:7061;total:8308 | iSNV |
| F55 | F55-21 | 10578 | 3'-UTR | 0.0743 | A:3;G:0;C:543;T:6753;total:7299 | iSNV |
| F55 | F55-22 | 347 | C | 0.6385 | A:5;G:3;C:8119;T:14331;total:22458 | iSNV |
| F55 | F55-22 | 907 | M | 0.1639 | A:0;G:1;C:2580;T:13151;total:15732 | iSNV |
| F55 | F55-22 | 998 | E | 0.9546 | A:576;G:1;C:61;T:12027;total:12665 | iSNV |
| F55 | F55-22 | 1218 | E | 0.9976 | A:1;G:0;C:37;T:15508;total:15546 | SNP |
| F55 | F55-22 | 1428 | E | 0.9868 | A:195;G:14604;C:0;T:1;total:14800 | SNP |
| F55 | F55-22 | 1471 | E | 0.0368 | A:5;G:0;C:10641;T:407;total:11053 | iSNV |
| F55 | F55-22 | 4244 | NS2B | 0.0482 | A:9675;G:0;C:0;T:490;total:10165 | iSNV |
| F55 | F55-22 | 4468 | NS2B | 0.9898 | A:10007;G:0;C:0;T:104;total:10111 | SNP |
| F55 | F55-22 | 4697 | NS3 | 0.9957 | A:4;G:0;C:16134;T:67;total:16205 | SNP |
| F55 | F55-22 | 5366 | NS3 | 0.0654 | A:15709;G:1;C:1100;T:0;total:16810 | iSNV |
| F55 | F55-22 | 5414 | NS3 | 0.0445 | A:19946;G:931;C:1;T:1;total:20879 | iSNV |
| F55 | F55-22 | 5737 | NS3 | 0.1581 | A:18896;G:3549;C:2;T:0;total:22447 | iSNV |
| F55 | F55-22 | 5871 | NS3 | 0.0492 | A:20922;G:2;C:1085;T:0;total:22009 | iSNV |
| F55 | F55-22 | 5952 | NS3 | 0.9965 | A:1;G:3;C:76;T:22651;total:22731 | SNP |
| F55 | F55-22 | 6867 | NS4A | 0.9415 | A:13048;G:812;C:0;T:0;total:13860 | iSNV |
| F55 | F55-22 | 7532 | NS4B | 0.0398 | A:1;G:0;C:586;T:14121;total:14708 | iSNV |
| F55 | F55-22 | 7633 | NS4B | 0.9702 | A:0;G:0;C:644;T:20962;total:21606 | iSNV |
| F55 | F55-22 | 9245 | NS5 | 0.9875 | A:0;G:5;C:270;T:21642;total:21917 | SNP |
| F55 | F55-22 | 9359 | NS5 | 0.9958 | A:7;G:1;C:29055;T:117;total:29180 | SNP |
| F55 | F55-22 | 9690 | NS5 | 0.0306 | A:263;G:8322;C:3;T:0;total:8588 | iSNV |
| F55 | F55-22 | 10259 | NS5 | 0.9964 | A:71;G:19809;C:0;T:2;total:19882 | SNP |
| F55 | F55-22 | 10401 | 3'-UTR | 0.0295 | A:1;G:6;C:432;T:14190;total:14629 | iSNV |
| F55 | F55-22 | 10428 | 3'-UTR | 0.5615 | A:1;G:0;C:5973;T:7645;total:13619 | iSNV |
| F55 | F55-22 | 10447 | 3'-UTR | 0.0216 | A:1;G:1;C:11447;T:253;total:11702 | iSNV |
| F55 | F55-22 | 10566 | 3'-UTR | 0.9911 | A:0;G:3;C:87;T:9950;total:10040 | SNP |
| F55 | F55-23 | 353 | C | 0.8471 | A:2861;G:15838;C:6;T:3;total:18708 | iSNV |
| F55 | F55-23 | 645 | M | 0.8963 | A:1060;G:9159;C:0;T:1;total:10220 | iSNV |
| F55 | F55-23 | 998 | E | 0.9135 | A:1;G:1;C:665;T:7015;total:7682 | iSNV |
| F55 | F55-23 | 1117 | E | 0.9098 | A:747;G:7526;C:0;T:0;total:8273 | iSNV |
| F55 | F55-23 | 1218 | E | 0.0874 | A:4;G:0;C:8564;T:821;total:9389 | iSNV |
| F55 | F55-23 | 1296 | E | 0.0385 | A:0;G:1;C:406;T:10134;total:10541 | iSNV |
| F55 | F55-23 | 1512 | E | 0.898 | A:626;G:5500;C:11;T:0;total:6137 | iSNV |
| F55 | F55-23 | 1797 | E | 0.0667 | A:0;G:630;C:1;T:8803;total:9434 | iSNV |
| F55 | F55-23 | 2303 | E | 0.7266 | A:0;G:1;C:2282;T:6061;total:8344 | iSNV |
| F55 | F55-23 | 3223 | NS1 | 0.0218 | A:3;G:21272;C:1;T:475;total:21751 | iSNV |
| F55 | F55-23 | 3440 | NS1 | 0.1523 | A:5343;G:960;C:0;T:0;total:6303 | iSNV |
| F55 | F55-23 | 3869 | NS2A | 0.1288 | A:2;G:0;C:12969;T:1919;total:14890 | iSNV |
| F55 | F55-23 | 4468 | NS2B | 0.2285 | A:1773;G:0;C:1;T:5984;total:7758 | iSNV |
| F55 | F55-23 | 4799 | NS3 | 0.0302 | A:15478;G:482;C:0;T:0;total:15960 | iSNV |
| F55 | F55-23 | 4974 | NS3 | 0.1662 | A:2065;G:10354;C:1;T:1;total:12421 | iSNV |
| F55 | F55-23 | 5339 | NS3 | 0.1924 | A:9984;G:2379;C:1;T:0;total:12364 | iSNV |
| F55 | F55-23 | 5678 | NS3 | 0.0215 | A:403;G:5;C:8;T:18287;total:18703 | iSNV |
| F55 | F55-23 | 5718 | NS3 | 0.184 | A:3403;G:15082;C:0;T:0;total:18485 | iSNV |
| F55 | F55-23 | 5826 | NS3 | 0.1193 | A:17270;G:2340;C:0;T:0;total:19610 | iSNV |
| F55 | F55-23 | 6472 | NS4A | 0.023 | A:0;G:0;C:231;T:9798;total:10029 | iSNV |
| F55 | F55-23 | 6867 | NS4A | 0.7118 | A:7292;G:2954;C:3;T:0;total:10249 | iSNV |
| F55 | F55-23 | 6900 | NS4A | 0.1285 | A:20;G:8236;C:0;T:1218;total:9474 | iSNV |
| F55 | F55-23 | 7182 | NS4A | 0.1124 | A:577;G:1;C:4554;T:1;total:5133 | iSNV |
| F55 | F55-23 | 7264 | NS4A | 0.02 | A:0;G:0;C:5861;T:120;total:5981 | iSNV |
| F55 | F55-23 | 7307 | NS4B | 0.042 | A:5907;G:259;C:0;T:0;total:6166 | iSNV |
| F55 | F55-23 | 7633 | NS4B | 0.9984 | A:0;G:2;C:27;T:17766;total:17795 | SNP |
| F55 | F55-23 | 9645 | NS5 | 0.0336 | A:191;G:5488;C:0;T:1;total:5680 | iSNV |
| F55 | F55-23 | 9722 | NS5 | 0.0606 | A:0;G:0;C:6165;T:398;total:6563 | iSNV |
| F55 | F55-23 | 10079 | NS5 | 0.0284 | A:8908;G:261;C:0;T:0;total:9169 | iSNV |
| F55 | F55-23 | 10259 | NS5 | 0.8671 | A:2274;G:14825;C:0;T:1;total:17100 | iSNV |

| | | | | | | |
|---|---|---|---|---|---|---|
| F55 | F55-23 | 10428 | 3'-UTR | 0.402 | A:3;G:1;C:7083;T:4766;total:11853 | iSNV |
| F55 | F55-23 | 10447 | 3'-UTR | 0.8556 | A:9;G:1;C:1419;T:8395;total:9824 | iSNV |
| F55 | F55-23 | 10451 | 3'-UTR | 0.0248 | A:0;G:3;C:9957;T:254;total:10214 | iSNV |
| F55 | F55-23 | 10804 | 3'-UTR | 0.121 | A:0;G:0;C:5857;T:807;total:6664 | iSNV |
| F55 | F55-24 | 340 | C | 0.0503 | A:2;G:0;C:7393;T:392;total:7787 | iSNV |
| F55 | F55-24 | 898 | M | 0.5123 | A:0;G:0;C:2106;T:2005;total:4111 | iSNV |
| F55 | F55-24 | 1067 | E | 0.0205 | A:0;G:0;C:4390;T:92;total:4482 | iSNV |
| F55 | F55-24 | 1193 | E | 0.0665 | A:0;G:0;C:404;T:5664;total:6068 | iSNV |
| F55 | F55-24 | 1218 | E | 0.997 | A:0;G:0;C:17;T:5530;total:5547 | SNP |
| F55 | F55-24 | 1598 | E | 0.152 | A:596;G:3323;C:1;T:0;total:3920 | iSNV |
| F55 | F55-24 | 2076 | E | 0.9913 | A:34;G:3866;C:0;T:0;total:3900 | SNP |
| F55 | F55-24 | 2300 | E | 0.0359 | A:0;G:0;C:184;T:4936;total:5120 | iSNV |
| F55 | F55-24 | 2324 | E | 0.0239 | A:0;G:0;C:4969;T:122;total:5091 | iSNV |
| F55 | F55-24 | 2369 | E | 0.1698 | A:500;G:2444;C:0;T:0;total:2944 | iSNV |
| F55 | F55-24 | 2771 | NS1 | 0.0227 | A:0;G:0;C:7641;T:178;total:7819 | iSNV |
| F55 | F55-24 | 2870 | NS1 | 0.5242 | A:5;G:0;C:4262;T:4689;total:8956 | iSNV |
| F55 | F55-24 | 2890 | NS1 | 0.9895 | A:7904;G:5;C:79;T:0;total:7988 | SNP |
| F55 | F55-24 | 3261 | NS1 | 0.0426 | A:7094;G:316;C:1;T:0;total:7411 | iSNV |
| F55 | F55-24 | 3608 | NS1 | 0.4851 | A:0;G:0;C:2495;T:2351;total:4846 | iSNV |
| F55 | F55-24 | 3669 | NS1 | 0.9907 | A:0;G:1;C:28;T:3084;total:3113 | SNP |
| F55 | F55-24 | 3869 | NS2A | 0.9892 | A:0;G:3;C:73;T:6908;total:6984 | SNP |
| F55 | F55-24 | 4286 | NS2B | 0.5562 | A:2781;G:2219;C:0;T:0;total:5000 | iSNV |
| F55 | F55-24 | 4896 | NS3 | 0.1391 | A:1090;G:6745;C:1;T:0;total:7836 | iSNV |
| F55 | F55-24 | 5172 | NS3 | 0.043 | A:4628;G:0;C:0;T:208;total:4836 | iSNV |
| F55 | F55-24 | 5195 | NS3 | 0.3294 | A:0;G:0;C:1776;T:3614;total:5390 | iSNV |
| F55 | F55-24 | 5358 | NS3 | 0.1558 | A:1031;G:5583;C:0;T:0;total:6614 | iSNV |
| F55 | F55-24 | 5737 | NS3 | 0.9889 | A:82;G:7543;C:0;T:3;total:7628 | SNP |
| F55 | F55-24 | 6867 | NS4A | 0.4384 | A:2022;G:2590;C:0;T:0;total:4612 | iSNV |
| F55 | F55-24 | 6971 | NS4A | 0.5099 | A:1;G:2133;C:2189;T:29;total:4352 | iSNV |
| F55 | F55-24 | 7182 | NS4A | 0.3806 | A:1337;G:0;C:2174;T:1;total:3512 | iSNV |
| F55 | F55-24 | 7193 | NS4A | 0.586 | A:0;G:0;C:2038;T:1440;total:3478 | iSNV |
| F55 | F55-24 | 7264 | NS4A | 0.0282 | A:0;G:0;C:3438;T:100;total:3538 | iSNV |
| F55 | F55-24 | 7633 | NS4B | 0.9963 | A:0;G:0;C:26;T:6867;total:6893 | SNP |
| F55 | F55-24 | 8690 | NS5 | 0.0235 | A:1;G:0;C:4353;T:105;total:4459 | iSNV |
| F55 | F55-24 | 9183 | NS5 | 0.0244 | A:179;G:7129;C:0;T:4;total:7312 | iSNV |
| F55 | F55-24 | 9233 | NS5 | 0.6304 | A:0;G:0;C:2941;T:5015;total:7956 | iSNV |
| F55 | F55-24 | 10373 | NS5 | 0.0306 | A:1;G:0;C:6544;T:207;total:6752 | iSNV |
| F55 | F55-24 | 10428 | 3'-UTR | 0.9636 | A:0;G:0;C:190;T:5017;total:5207 | iSNV |
| F55 | F55-24 | 10447 | 3'-UTR | 0.0435 | A:1;G:0;C:4460;T:203;total:4664 | iSNV |
| F55 | F55-25 | 824 | M | 0.525 | A:6216;G:6864;C:0;T:4;total:13084 | iSNV |
| F55 | F55-25 | 1117 | E | 0.0334 | A:13061;G:452;C:0;T:1;total:13514 | iSNV |
| F55 | F55-25 | 1218 | E | 0.9988 | A:1;G:1;C:17;T:14761;total:14780 | SNP |
| F55 | F55-25 | 1428 | E | 0.0547 | A:15986;G:926;C:3;T:0;total:16915 | iSNV |
| F55 | F55-25 | 1551 | E | 0.3079 | A:0;G:3311;C:7438;T:2;total:10751 | iSNV |
| F55 | F55-25 | 1797 | E | 0.5951 | A:2;G:9366;C:0;T:6375;total:15743 | iSNV |
| F55 | F55-25 | 2088 | E | 0.0256 | A:2;G:0;C:11375;T:300;total:11677 | iSNV |
| F55 | F55-25 | 2493 | NS1 | 0.0243 | A:9212;G:230;C:2;T:0;total:9444 | iSNV |
| F55 | F55-25 | 2600 | NS1 | 0.332 | A:7;G:1;C:10226;T:5087;total:15321 | iSNV |
| F55 | F55-25 | 2855 | NS1 | 0.034 | A:842;G:23850;C:0;T:6;total:24698 | iSNV |
| F55 | F55-25 | 3869 | NS2A | 0.9927 | A:1;G:2;C:136;T:18756;total:18895 | SNP |
| F55 | F55-25 | 4385 | NS2B | 0.074 | A:3;G:1;C:11863;T:949;total:12816 | iSNV |
| F55 | F55-25 | 4469 | NS2B | 0.4086 | A:0;G:0;C:4366;T:6319;total:10685 | iSNV |
| F55 | F55-25 | 4646 | NS3 | 0.0242 | A:5;G:0;C:20256;T:503;total:20764 | iSNV |
| F55 | F55-25 | 4974 | NS3 | 0.382 | A:6174;G:9983;C:2;T:2;total:16161 | iSNV |
| F55 | F55-25 | 5256 | NS3 | 0.3183 | A:3;G:0;C:10906;T:5094;total:16003 | iSNV |
| F55 | F55-25 | 5558 | NS3 | 0.035 | A:620;G:17087;C:1;T:1;total:17709 | iSNV |
| F55 | F55-25 | 5707 | NS3 | 0.0211 | A:21894;G:472;C:0;T:0;total:22366 | iSNV |
| F55 | F55-25 | 5736 | NS3 | 0.3642 | A:8168;G:1;C:14250;T:4;total:22423 | iSNV |
| F55 | F55-25 | 5968 | NS3 | 0.0299 | A:682;G:22071;C:0;T:3;total:22756 | iSNV |
| F55 | F55-25 | 6336 | NS3 | 0.021 | A:8653;G:1;C:0;T:186;total:8840 | iSNV |
| F55 | F55-25 | 6867 | NS4A | 0.035 | A:448;G:12323;C:0;T:1;total:12772 | iSNV |
| F55 | F55-25 | 6900 | NS4A | 0.3305 | A:4266;G:8637;C:1;T:3;total:12907 | iSNV |
| F55 | F55-25 | 6923 | NS4A | 0.0424 | A:546;G:12311;C:0;T:2;total:12859 | iSNV |
| F55 | F55-25 | 7104 | NS4A | 0.0996 | A:1080;G:9755;C:1;T:2;total:10838 | iSNV |
| F55 | F55-25 | 7264 | NS4A | 0.2946 | A:1;G:0;C:6017;T:2514;total:8532 | iSNV |
| F55 | F55-25 | 7316 | NS4B | 0.0591 | A:519;G:8248;C:0;T:3;total:8770 | iSNV |
| F55 | F55-25 | 7526 | NS4B | 0.0495 | A:2;G:0;C:631;T:12089;total:12722 | iSNV |
| F55 | F55-25 | 7633 | NS4B | 0.0661 | A:8;G:0;C:18848;T:1336;total:20192 | iSNV |
| F55 | F55-25 | 7656 | NS4B | 0.3277 | A:6687;G:13713;C:2;T:1;total:20403 | iSNV |
| F55 | F55-25 | 8675 | NS5 | 0.0268 | A:0;G:1;C:309;T:11179;total:11489 | iSNV |
| F55 | F55-25 | 10419 | 3'-UTR | 0.0202 | A:3;G:1;C:14357;T:297;total:14658 | iSNV |
| F55 | F55-25 | 10428 | 3'-UTR | 0.9957 | A:1;G:1;C:58;T:13668;total:13728 | SNP |
| F55 | F55-25 | 10632 | 3'-UTR | 0.0221 | A:9;G:1;C:244;T:10740;total:10994 | iSNV |
| F55 | F55-26 | 168 | C | 0.0462 | A:0;G:1;C:1013;T:20892;total:21906 | iSNV |
| F55 | F55-26 | 470 | C | 0.0498 | A:18346;G:14;C:8;T:963;total:19331 | iSNV |
| F55 | F55-26 | 542 | M | 0.0267 | A:0;G:0;C:369;T:13422;total:13791 | iSNV |
| F55 | F55-26 | 694 | M | 0.0336 | A:15441;G:538;C:1;T:0;total:15980 | iSNV |
| F55 | F55-26 | 1218 | E | 0.9995 | A:0;G:0;C:7;T:12787;total:12794 | SNP |
| F55 | F55-26 | 1263 | E | 0.0445 | A:1;G:2;C:620;T:13302;total:13925 | iSNV |
| F55 | F55-26 | 1428 | E | 0.513 | A:6654;G:7007;C:0;T:2;total:13663 | iSNV |
| F55 | F55-26 | 1514 | E | 0.0438 | A:10074;G:1;C:462;T:0;total:10537 | iSNV |
| F55 | F55-26 | 1797 | E | 0.4095 | A:15;G:5738;C:0;T:8256;total:14009 | iSNV |

| | | | | | | |
|---|---|---|---|---|---|---|
| F55 | F55-26 | 3344 | NS1 | 0.1113 | A:2;G:13039;C:0;T:1634;total:14675 | iSNV |
| F55 | F55-26 | 3536 | NS1 | 0.1156 | A:2;G:0;C:8229;T:1076;total:9307 | iSNV |
| F55 | F55-26 | 3869 | NS2A | 0.9981 | A:0;G:0;C:33;T:16674;total:16707 | SNP |
| F55 | F55-26 | 3935 | NS2A | 0.0421 | A:17462;G:769;C:1;T:0;total:18232 | iSNV |
| F55 | F55-26 | 4922 | NS3 | 0.0455 | A:6;G:1;C:14507;T:693;total:15207 | iSNV |
| F55 | F55-26 | 5365 | NS3 | 0.023 | A:13453;G:318;C:1;T:0;total:13772 | iSNV |
| F55 | F55-26 | 5781 | NS3 | 0.0538 | A:1110;G:19497;C:1;T:1;total:20609 | iSNV |
| F55 | F55-26 | 6080 | NS3 | 0.0833 | A:1078;G:11854;C:0;T:1;total:12933 | iSNV |
| F55 | F55-26 | 6380 | NS3 | 0.0475 | A:2;G:0;C:11269;T:563;total:11834 | iSNV |
| F55 | F55-26 | 6423 | NS3 | 0.3811 | A:4793;G:1;C:7778;T:4;total:12576 | iSNV |
| F55 | F55-26 | 6714 | NS4A | 0.1655 | A:1995;G:10053;C:0;T:2;total:12050 | iSNV |
| F55 | F55-26 | 6770 | NS4A | 0.0394 | A:11;G:0;C:11962;T:492;total:12465 | iSNV |
| F55 | F55-26 | 6806 | NS4A | 0.0465 | A:1;G:13684;C:0;T:668;total:14353 | iSNV |
| F55 | F55-26 | 6867 | NS4A | 0.0406 | A:469;G:11055;C:0;T:1;total:11525 | iSNV |
| F55 | F55-26 | 6947 | NS4A | 0.1228 | A:0;G:2;C:1350;T:9641;total:10993 | iSNV |
| F55 | F55-26 | 6970 | NS4A | 0.2692 | A:8071;G:1;C:2975;T:3;total:11050 | iSNV |
| F55 | F55-26 | 7244 | NS4A | 0.4321 | A:1;G:0;C:3150;T:4138;total:7289 | iSNV |
| F55 | F55-26 | 7421 | NS4B | 0.0345 | A:8904;G:319;C:0;T:0;total:9223 | iSNV |
| F55 | F55-26 | 7561 | NS4B | 0.3203 | A:1;G:6;C:4713;T:9994;total:14714 | iSNV |
| F55 | F55-26 | 7633 | NS4B | 0.6811 | A:1;G:1;C:5545;T:11839;total:17386 | iSNV |
| F55 | F55-26 | 7982 | NS5 | 0.0756 | A:17500;G:219;C:2;T:1451;total:19172 | iSNV |
| F55 | F55-26 | 8103 | NS5 | 0.0237 | A:6;G:2;C:16189;T:394;total:16591 | iSNV |
| F55 | F55-26 | 8471 | NS5 | 0.1017 | A:17133;G:1940;C:1;T:0;total:19074 | iSNV |
| F55 | F55-26 | 8666 | NS5 | 0.0219 | A:256;G:11398;C:0;T:3;total:11657 | iSNV |
| F55 | F55-26 | 8840 | NS5 | 0.0404 | A:12792;G:539;C:1;T:0;total:13332 | iSNV |
| F55 | F55-26 | 9443 | NS5 | 0.3188 | A:14871;G:6962;C:2;T:2;total:21837 | iSNV |
| F55 | F55-26 | 9899 | NS5 | 0.0551 | A:2;G:0;C:9196;T:537;total:9735 | iSNV |
| F55 | F55-26 | 10364 | NS5 | 0.1893 | A:10;G:0;C:12390;T:2897;total:15297 | iSNV |
| F55 | F55-26 | 10373 | NS5 | 0.2978 | A:3;G:1;C:10872;T:4613;total:15489 | iSNV |
| F55 | F55-26 | 10428 | 3'-UTR | 0.9475 | A:1;G:3;C:656;T:11833;total:12493 | iSNV |
| F55 | F55-26 | 10561 | 3'-UTR | 0.0363 | A:9019;G:340;C:1;T:1;total:9361 | iSNV |
| F55 | F55-26 | 10577 | 3'-UTR | 0.0211 | A:1;G:0;C:9738;T:210;total:9949 | iSNV |
| F55 | F55-27 | 292 | C | 0.1433 | A:1;G:0;C:11643;T:1949;total:13593 | iSNV |
| F55 | F55-27 | 662 | M | 0.0875 | A:9914;G:952;C:1;T:1;total:10868 | iSNV |
| F55 | F55-27 | 716 | M | 0.1128 | A:2;G:10616;C:0;T:1350;total:11968 | iSNV |
| F55 | F55-27 | 906 | M | 0.0295 | A:5292;G:161;C:0;T:0;total:5453 | iSNV |
| F55 | F55-27 | 1218 | E | 0.988 | A:1;G:1;C:92;T:7693;total:7787 | SNP |
| F55 | F55-27 | 1298 | E | 0.0268 | A:0;G:259;C:0;T:9383;total:9642 | iSNV |
| F55 | F55-27 | 1512 | E | 0.6236 | A:1828;G:2220;C:808;T:0;total:4856 | iSNV |
| F55 | F55-27 | 1514 | E | 0.2551 | A:3636;G:1;C:1246;T:0;total:4883 | iSNV |
| F55 | F55-27 | 1709 | E | 0.1467 | A:793;G:4611;C:0;T:0;total:5404 | iSNV |
| F55 | F55-27 | 1797 | E | 0.03 | A:0;G:236;C:166;T:7454;total:7856 | iSNV |
| F55 | F55-27 | 2270 | E | 0.2933 | A:5239;G:0;C:2177;T:4;total:7420 | iSNV |
| F55 | F55-27 | 2274 | E | 0.49 | A:7;G:3782;C:0;T:3641;total:7430 | iSNV |
| F55 | F55-27 | 2277 | E | 0.121 | A:1;G:0;C:901;T:6539;total:7441 | iSNV |
| F55 | F55-27 | 2531 | NS1 | 0.1597 | A:928;G:1;C:44;T:4835;total:5808 | iSNV |
| F55 | F55-27 | 2664 | NS1 | 0.9162 | A:0;G:0;C:978;T:10688;total:11666 | iSNV |
| F55 | F55-27 | 3869 | NS2A | 0.9891 | A:1;G:1;C:108;T:9896;total:10006 | SNP |
| F55 | F55-27 | 4682 | NS3 | 0.2724 | A:0;G:0;C:2154;T:5751;total:7905 | iSNV |
| F55 | F55-27 | 4974 | NS3 | 0.0323 | A:290;G:8661;C:1;T:0;total:8952 | iSNV |
| F55 | F55-27 | 5617 | NS3 | 0.1911 | A:8400;G:1986;C:2;T:0;total:10388 | iSNV |
| F55 | F55-27 | 5737 | NS3 | 0.0997 | A:11131;G:1234;C:0;T:0;total:12365 | iSNV |
| F55 | F55-27 | 5936 | NS3 | 0.0351 | A:2;G:0;C:13552;T:494;total:14048 | iSNV |
| F55 | F55-27 | 6063 | NS3 | 0.0347 | A:1;G:0;C:9692;T:349;total:10042 | iSNV |
| F55 | F55-27 | 6182 | NS3 | 0.0335 | A:1;G:1;C:158;T:4543;total:4703 | iSNV |
| F55 | F55-27 | 6867 | NS4A | 0.021 | A:140;G:6521;C:0;T:0;total:6661 | iSNV |
| F55 | F55-27 | 6957 | NS4A | 0.0308 | A:5687;G:181;C:1;T:0;total:5869 | iSNV |
| F55 | F55-27 | 7250 | NS4A | 0.1545 | A:3074;G:562;C:0;T:0;total:3636 | iSNV |
| F55 | F55-27 | 7626 | NS4B | 0.9255 | A:2;G:12327;C:0;T:993;total:13322 | iSNV |
| F55 | F55-27 | 7633 | NS4B | 0.0541 | A:1;G:1;C:12816;T:734;total:13552 | iSNV |
| F55 | F55-27 | 7697 | NS5 | 0.0314 | A:3;G:12640;C:1;T:410;total:13054 | iSNV |
| F55 | F55-27 | 7835 | NS5 | 0.1512 | A:0;G:1;C:1770;T:9935;total:11706 | iSNV |
| F55 | F55-27 | 8756 | NS5 | 0.1897 | A:7596;G:1779;C:0;T:0;total:9375 | iSNV |
| F55 | F55-27 | 9728 | NS5 | 0.0213 | A:0;G:0;C:4438;T:97;total:4535 | iSNV |
| F55 | F55-27 | 9830 | NS5 | 0.0499 | A:0;G:1;C:257;T:4883;total:5141 | iSNV |
| F55 | F55-27 | 10139 | NS5 | 0.0882 | A:2;G:4;C:631;T:6516;total:7153 | iSNV |
| F55 | F55-27 | 10400 | 3'-UTR | 0.074 | A:0;G:3;C:670;T:8379;total:9052 | iSNV |
| F55 | F55-27 | 10428 | 3'-UTR | 0.633 | A:1;G:3;C:3029;T:5220;total:8253 | iSNV |
| F55 | F55-27 | 10447 | 3'-UTR | 0.0215 | A:2;G:0;C:6977;T:154;total:7133 | iSNV |
| F55 | F55-27 | 10904 | 3'-UTR | 0.1935 | A:0;G:0;C:400;T:96;total:496 | iSNV |
| F55 | F55-28 | 230 | C | 0.9417 | A:2;G:0;C:14104;T:874;total:14980 | iSNV |
| F55 | F55-28 | 1218 | E | 0.9925 | A:2;G:0;C:64;T:8723;total:8789 | SNP |
| F55 | F55-28 | 1220 | E | 0.0289 | A:1;G:1;C:8811;T:263;total:9076 | iSNV |
| F55 | F55-28 | 1428 | E | 0.9832 | A:129;G:7544;C:0;T:0;total:7673 | SNP |
| F55 | F55-28 | 1784 | E | 0.1808 | A:1;G:0;C:6820;T:1506;total:8327 | iSNV |
| F55 | F55-28 | 1910 | E | 0.0736 | A:369;G:4640;C:0;T:1;total:5010 | iSNV |
| F55 | F55-28 | 2369 | E | 0.1787 | A:537;G:2467;C:0;T:1;total:3005 | iSNV |
| F55 | F55-28 | 3380 | NS1 | 0.0438 | A:0;G:0;C:341;T:7427;total:7768 | iSNV |
| F55 | F55-28 | 3869 | NS2A | 0.993 | A:0;G:0;C:86;T:12115;total:12201 | SNP |
| F55 | F55-28 | 4447 | NS2B | 0.2256 | A:4353;G:1269;C:1;T:0;total:5623 | iSNV |
| F55 | F55-28 | 5737 | NS3 | 0.1644 | A:11765;G:2315;C:0;T:0;total:14080 | iSNV |
| F55 | F55-28 | 5952 | NS3 | 0.2411 | A:0;G:0;C:11991;T:3810;total:15801 | iSNV |

| | | | | | | |
|---|---|---|---|---|---|---|
| F55 | F55-28 | 6155 | NS3 | 0.0329 | A:169;G:4955;C:0;T:1;total:5125 | iSNV |
| F55 | F55-28 | 6408 | NS3 | 0.0406 | A:341;G:8038;C:1;T:0;total:8380 | iSNV |
| F55 | F55-28 | 6472 | NS4A | 0.1657 | A:0;G:1;C:1263;T:6358;total:7622 | iSNV |
| F55 | F55-28 | 6714 | NS4A | 0.0224 | A:165;G:7193;C:0;T:1;total:7359 | iSNV |
| F55 | F55-28 | 6737 | NS4A | 0.0213 | A:2;G:0;C:7281;T:159;total:7442 | iSNV |
| F55 | F55-28 | 6970 | NS4A | 0.7801 | A:1858;G:1;C:6587;T:1;total:8447 | iSNV |
| F55 | F55-28 | 7104 | NS4A | 0.7234 | A:3387;G:1296;C:2;T:0;total:4685 | iSNV |
| F55 | F55-28 | 7287 | NS4B | 0.0455 | A:203;G:0;C:4253;T:2;total:4458 | iSNV |
| F55 | F55-28 | 7454 | NS4B | 0.0444 | A:4729;G:220;C:2;T:0;total:4951 | iSNV |
| F55 | F55-28 | 7633 | NS4B | 0.9457 | A:0;G:0;C:847;T:14738;total:15585 | iSNV |
| F55 | F55-28 | 7656 | NS4B | 0.045 | A:753;G:15971;C:0;T:3;total:16727 | iSNV |
| F55 | F55-28 | 7973 | NS5 | 0.038 | A:13354;G:3;C:0;T:529;total:13886 | iSNV |
| F55 | F55-28 | 8909 | NS5 | 0.1769 | A:0;G:4;C:3120;T:14513;total:17637 | iSNV |
| F55 | F55-28 | 9080 | NS5 | 0.0993 | A:8375;G:924;C:0;T:1;total:9300 | iSNV |
| F55 | F55-28 | 9545 | NS5 | 0.0275 | A:212;G:0;C:7493;T:0;total:7705 | iSNV |
| F55 | F55-28 | 9782 | NS5 | 0.1578 | A:899;G:4795;C:0;T:0;total:5694 | iSNV |
| F55 | F55-28 | 10335 | NS5 | 0.0223 | A:1;G:3;C:343;T:15028;total:15375 | iSNV |
| F55 | F55-28 | 10428 | 3'-UTR | 0.6976 | A:0;G:1;C:2845;T:6559;total:9405 | iSNV |
| F55 | F55-28 | 10452 | 3'-UTR | 0.0364 | A:7840;G:297;C:1;T:0;total:8138 | iSNV |
| F55 | F55-29 | 1218 | E | 0.999 | A:0;G:2;C:20;T:20887;total:20909 | SNP |
| F55 | F55-29 | 1296 | E | 0.2923 | A:4;G:3;C:7678;T:18581;total:26266 | iSNV |
| F55 | F55-29 | 1363 | E | 0.0447 | A:2;G:1;C:23296;T:1092;total:24391 | iSNV |
| F55 | F55-29 | 1447 | E | 0.6171 | A:13166;G:1;C:8175;T:4;total:21346 | iSNV |
| F55 | F55-29 | 1914 | E | 0.2844 | A:8650;G:3438;C:0;T:0;total:12088 | iSNV |
| F55 | F55-29 | 2362 | E | 0.9935 | A:1;G:65;C:10156;T:1;total:10223 | SNP |
| F55 | F55-29 | 3181 | NS1 | 0.0376 | A:30888;G:1208;C:3;T:1;total:32100 | iSNV |
| F55 | F55-29 | 3438 | NS1 | 0.2414 | A:1;G:1;C:12962;T:4126;total:17090 | iSNV |
| F55 | F55-29 | 3645 | NS1 | 0.2974 | A:3;G:0;C:3747;T:8847;total:12597 | iSNV |
| F55 | F55-29 | 3869 | NS2A | 0.9935 | A:0;G:5;C:170;T:26465;total:26640 | SNP |
| F55 | F55-29 | 3959 | NS2A | 0.619 | A:2;G:1;C:9561;T:15528;total:25092 | iSNV |
| F55 | F55-29 | 4439 | NS2B | 0.022 | A:0;G:16080;C:0;T:362;total:16442 | iSNV |
| F55 | F55-29 | 4829 | NS3 | 0.0366 | A:27905;G:1062;C:3;T:2;total:28972 | iSNV |
| F55 | F55-29 | 4896 | NS3 | 0.0423 | A:1275;G:28802;C:2;T:13;total:30092 | iSNV |
| F55 | F55-29 | 4943 | NS3 | 0.0396 | A:20931;G:865;C:0;T:0;total:21796 | iSNV |
| F55 | F55-29 | 4974 | NS3 | 0.0435 | A:1039;G:22822;C:0;T:2;total:23863 | iSNV |
| F55 | F55-29 | 5702 | NS3 | 0.0506 | A:4;G:2;C:33296;T:1775;total:35077 | iSNV |
| F55 | F55-29 | 6867 | NS4A | 0.3005 | A:5533;G:12875;C:1;T:1;total:18410 | iSNV |
| F55 | F55-29 | 6900 | NS4A | 0.0773 | A:1413;G:16853;C:1;T:1;total:18268 | iSNV |
| F55 | F55-29 | 7481 | NS4B | 0.0574 | A:0;G:3;C:752;T:12335;total:13090 | iSNV |
| F55 | F55-29 | 7528 | NS4B | 0.2779 | A:1;G:1;C:13800;T:5312;total:19114 | iSNV |
| F55 | F55-29 | 7627 | NS4B | 0.3019 | A:14;G:8456;C:19534;T:3;total:28007 | iSNV |
| F55 | F55-29 | 7633 | NS4B | 0.694 | A:0;G:3;C:8590;T:19473;total:28066 | iSNV |
| F55 | F55-29 | 8627 | NS5 | 0.0317 | A:1;G:0;C:19889;T:652;total:20542 | iSNV |
| F55 | F55-29 | 8844 | NS5 | 0.0349 | A:751;G:20707;C:1;T:0;total:21459 | iSNV |
| F55 | F55-29 | 8966 | NS5 | 0.0365 | A:2;G:1;C:29098;T:1103;total:30204 | iSNV |
| F55 | F55-29 | 9032 | NS5 | 0.0206 | A:1;G:0;C:530;T:25119;total:25650 | iSNV |
| F55 | F55-29 | 9659 | NS5 | 0.0306 | A:7;G:0;C:12565;T:398;total:12970 | iSNV |
| F55 | F55-29 | 10407 | 3'-UTR | 0.0352 | A:3;G:4;C:742;T:20324;total:21073 | iSNV |
| F55 | F55-29 | 10428 | 3'-UTR | 0.9567 | A:4;G:7;C:854;T:18849;total:19714 | iSNV |
| F55 | F55-29 | 10513 | 3'-UTR | 0.0226 | A:0;G:1;C:400;T:17270;total:17671 | iSNV |
| F55 | F55-3 | 563 | M | 0.9835 | A:8743;G:147;C:0;T:0;total:8890 | SNP |
| F55 | F55-3 | 617 | M | 0.028 | A:1;G:2;C:8387;T:242;total:8632 | iSNV |
| F55 | F55-3 | 978 | E | 0.1449 | A:0;G:0;C:1142;T:6739;total:7881 | iSNV |
| F55 | F55-3 | 995 | E | 0.0259 | A:200;G:7506;C:0;T:1;total:7707 | iSNV |
| F55 | F55-3 | 1257 | E | 0.9807 | A:7918;G:0;C:156;T:0;total:8074 | SNP |
| F55 | F55-3 | 1428 | E | 0.0397 | A:10688;G:443;C:1;T:6;total:11138 | iSNV |
| F55 | F55-3 | 1442 | E | 0.0216 | A:0;G:1;C:235;T:10610;total:10846 | iSNV |
| F55 | F55-3 | 1512 | E | 0.2416 | A:7353;G:2343;C:0;T:1;total:9697 | iSNV |
| F55 | F55-3 | 1772 | E | 0.9831 | A:1;G:9526;C:0;T:163;total:9690 | SNP |
| F55 | F55-3 | 1797 | E | 0.395 | A:2;G:4112;C:1;T:6295;total:10410 | iSNV |
| F55 | F55-3 | 1799 | E | 0.2679 | A:0;G:0;C:7619;T:2789;total:10408 | iSNV |
| F55 | F55-3 | 2240 | E | 0.0688 | A:1;G:0;C:7049;T:521;total:7571 | iSNV |
| F55 | F55-3 | 2372 | E | 0.9836 | A:2;G:0;C:105;T:6416;total:6523 | SNP |
| F55 | F55-3 | 2493 | NS1 | 0.9842 | A:106;G:6641;C:0;T:1;total:6748 | SNP |
| F55 | F55-3 | 2504 | NS1 | 0.9842 | A:102;G:6381;C:0;T:1;total:6484 | SNP |
| F55 | F55-3 | 2531 | NS1 | 0.9818 | A:5;G:0;C:6778;T:121;total:6904 | SNP |
| F55 | F55-3 | 3179 | NS1 | 0.0257 | A:0;G:1;C:239;T:9046;total:9286 | iSNV |
| F55 | F55-3 | 3355 | NS1 | 0.3619 | A:4987;G:2830;C:0;T:1;total:7818 | iSNV |
| F55 | F55-3 | 3459 | NS1 | 0.0314 | A:224;G:6892;C:0;T:0;total:7116 | iSNV |
| F55 | F55-3 | 3572 | NS1 | 0.9833 | A:0;G:0;C:122;T:7176;total:7298 | SNP |
| F55 | F55-3 | 3962 | NS2A | 0.9806 | A:0;G:0;C:165;T:8333;total:8498 | SNP |
| F55 | F55-3 | 4155 | NS2A | 0.983 | A:4842;G:1;C:1;T:82;total:4926 | SNP |
| F55 | F55-3 | 4397 | NS2B | 0.0493 | A:1;G:0;C:7473;T:388;total:7862 | iSNV |
| F55 | F55-3 | 4663 | NS3 | 0.0485 | A:2;G:0;C:9148;T:467;total:9617 | iSNV |
| F55 | F55-3 | 4712 | NS3 | 0.9837 | A:9752;G:162;C:0;T:0;total:9914 | SNP |
| F55 | F55-3 | 5311 | NS3 | 0.9816 | A:2;G:4;C:131;T:7272;total:7409 | SNP |
| F55 | F55-3 | 5978 | NS3 | 0.3674 | A:5747;G:3338;C:0;T:0;total:9085 | iSNV |
| F55 | F55-3 | 6080 | NS3 | 0.985 | A:8182;G:125;C:0;T:0;total:8307 | SNP |
| F55 | F55-3 | 6534 | NS4A | 0.246 | A:1785;G:0;C:0;T:5471;total:7256 | iSNV |
| F55 | F55-3 | 6782 | NS4A | 0.0339 | A:2;G:7762;C:273;T:1;total:8038 | iSNV |
| F55 | F55-3 | 7527 | NS4B | 0.0399 | A:6602;G:275;C:0;T:0;total:6877 | iSNV |
| F55 | F55-3 | 7616 | NS4B | 0.0689 | A:532;G:7180;C:1;T:0;total:7713 | iSNV |

| | | | | | | |
|---|---|---|---|---|---|---|
| F55 | F55-3 | 7633 | NS4B | 0.0201 | A:0;G:0;C:7828;T:161;total:7989 | iSNV |
| F55 | F55-3 | 7657 | NS4B | 0.0333 | A:7337;G:253;C:1;T:1;total:7592 | iSNV |
| F55 | F55-3 | 8282 | NS5 | 0.9899 | A:1;G:0;C:8648;T:88;total:8737 | SNP |
| F55 | F55-3 | 8900 | NS5 | 0.9785 | A:157;G:0;C:7130;T:4;total:7291 | iSNV |
| F55 | F55-3 | 9132 | NS5 | 0.0742 | A:4;G:1;C:9240;T:741;total:9986 | iSNV |
| F55 | F55-3 | 9370 | NS5 | 0.2833 | A:3299;G:3;C:0;T:8339;total:11641 | iSNV |
| F55 | F55-3 | 9446 | NS5 | 0.9831 | A:205;G:11960;C:0;T:1;total:12166 | SNP |
| F55 | F55-3 | 9688 | NS5 | 0.0271 | A:2;G:0;C:9954;T:278;total:10234 | iSNV |
| F55 | F55-3 | 10428 | 3'-UTR | 0.3527 | A:0;G:2;C:5643;T:3076;total:8721 | iSNV |
| F55 | F55-30 | 688 | M | 0.1584 | A:16781;G:3159;C:0;T:1;total:19941 | iSNV |
| F55 | F55-30 | 1218 | E | 0.9968 | A:1;G:2;C:48;T:15638;total:15689 | SNP |
| F55 | F55-30 | 1373 | E | 0.024 | A:17453;G:19;C:1;T:431;total:17904 | iSNV |
| F55 | F55-30 | 1413 | E | 0.984 | A:299;G:18408;C:0;T:2;total:18709 | SNP |
| F55 | F55-30 | 1428 | E | 0.9846 | A:239;G:15361;C:0;T:2;total:15602 | SNP |
| F55 | F55-30 | 2541 | NS1 | 0.0249 | A:2;G:0;C:12590;T:322;total:12914 | iSNV |
| F55 | F55-30 | 3869 | NS2A | 0.9974 | A:0;G:2;C:53;T:20914;total:20969 | SNP |
| F55 | F55-30 | 3888 | NS2A | 0.0278 | A:6;G:21841;C:1;T:627;total:22475 | iSNV |
| F55 | F55-30 | 3933 | NS2A | 0.0356 | A:21618;G:800;C:3;T:0;total:22421 | iSNV |
| F55 | F55-30 | 3965 | NS2A | 0.0249 | A:3;G:0;C:19786;T:507;total:20296 | iSNV |
| F55 | F55-30 | 4005 | NS2A | 0.9832 | A:1;G:1;C:272;T:16016;total:16290 | SNP |
| F55 | F55-30 | 4402 | NS2B | 0.0283 | A:12519;G:5;C:3;T:365;total:12892 | iSNV |
| F55 | F55-30 | 4447 | NS2B | 0.036 | A:11282;G:422;C:2;T:0;total:11706 | iSNV |
| F55 | F55-30 | 4468 | NS2B | 0.0368 | A:430;G:0;C:0;T:11231;total:11661 | iSNV |
| F55 | F55-30 | 4472 | NS2B | 0.0261 | A:3;G:1;C:12744;T:342;total:13090 | iSNV |
| F55 | F55-30 | 4844 | NS3 | 0.0312 | A:3;G:1;C:22673;T:731;total:23408 | iSNV |
| F55 | F55-30 | 4896 | NS3 | 0.0538 | A:1287;G:22589;C:1;T:4;total:23881 | iSNV |
| F55 | F55-30 | 4974 | NS3 | 0.0407 | A:768;G:18043;C:46;T:0;total:18857 | iSNV |
| F55 | F55-30 | 5737 | NS3 | 0.1645 | A:21467;G:4228;C:1;T:3;total:25699 | iSNV |
| F55 | F55-30 | 6041 | NS3 | 0.0296 | A:0;G:638;C:14;T:20851;total:21503 | iSNV |
| F55 | F55-30 | 6185 | NS3 | 0.132 | A:3;G:0;C:11205;T:1705;total:12913 | iSNV |
| F55 | F55-30 | 6336 | NS3 | 0.0431 | A:10054;G:2;C:1;T:454;total:10511 | iSNV |
| F55 | F55-30 | 6714 | NS4A | 0.0728 | A:1168;G:14867;C:1;T:0;total:16036 | iSNV |
| F55 | F55-30 | 6806 | NS4A | 0.0292 | A:539;G:17861;C:1;T:1;total:18402 | iSNV |
| F55 | F55-30 | 6810 | NS4A | 0.028 | A:0;G:17796;C:0;T:513;total:18309 | iSNV |
| F55 | F55-30 | 6867 | NS4A | 0.2859 | A:4251;G:10612;C:0;T:1;total:14864 | iSNV |
| F55 | F55-30 | 6911 | NS4A | 0.0211 | A:14384;G:311;C:0;T:3;total:14698 | iSNV |
| F55 | F55-30 | 7175 | NS4A | 0.0333 | A:0;G:0;C:320;T:9283;total:9603 | iSNV |
| F55 | F55-30 | 7264 | NS4A | 0.4551 | A:2;G:0;C:5068;T:4235;total:9305 | iSNV |
| F55 | F55-30 | 7430 | NS4B | 0.031 | A:0;G:0;C:8413;T:270;total:8683 | iSNV |
| F55 | F55-30 | 7633 | NS4B | 0.9858 | A:0;G:2;C:311;T:21658;total:21971 | SNP |
| F55 | F55-30 | 7658 | NS4B | 0.0281 | A:1;G:5;C:660;T:22761;total:23427 | iSNV |
| F55 | F55-30 | 7922 | NS5 | 0.0327 | A:3;G:8;C:826;T:24382;total:25219 | iSNV |
| F55 | F55-30 | 8023 | NS5 | 0.0333 | A:740;G:1;C:0;T:21421;total:22162 | iSNV |
| F55 | F55-30 | 8024 | NS5 | 0.0862 | A:0;G:20243;C:2;T:1912;total:22157 | iSNV |
| F55 | F55-30 | 9188 | NS5 | 0.0216 | A:1;G:2;C:497;T:22440;total:22940 | iSNV |
| F55 | F55-30 | 9335 | NS5 | 0.0211 | A:11;G:3;C:598;T:27696;total:28308 | iSNV |
| F55 | F55-30 | 9506 | NS5 | 0.1513 | A:0;G:1;C:2873;T:16108;total:18982 | iSNV |
| F55 | F55-30 | 9565 | NS5 | 0.0382 | A:609;G:15327;C:0;T:3;total:15939 | iSNV |
| F55 | F55-30 | 9645 | NS5 | 0.0605 | A:640;G:9937;C:0;T:0;total:10577 | iSNV |
| F55 | F55-30 | 10376 | NS5 | 0.984 | A:19509;G:314;C:0;T:4;total:19827 | SNP |
| F55 | F55-30 | 10419 | 3'-UTR | 0.0517 | A:4;G:8;C:15998;T:873;total:16883 | iSNV |
| F55 | F55-30 | 10428 | 3'-UTR | 0.984 | A:1;G:1;C:252;T:15556;total:15810 | SNP |
| F55 | F55-4 | 563 | M | 0.9972 | A:8730;G:25;C:0;T:0;total:8755 | SNP |
| F55 | F55-4 | 1117 | E | 0.025 | A:7524;G:193;C:0;T:0;total:7717 | iSNV |
| F55 | F55-4 | 1257 | E | 0.9953 | A:7815;G:1;C:36;T:0;total:7852 | SNP |
| F55 | F55-4 | 1512 | E | 0.8399 | A:1273;G:6675;C:0;T:0;total:7948 | iSNV |
| F55 | F55-4 | 1772 | E | 0.9953 | A:2;G:8329;C:0;T:38;total:8369 | SNP |
| F55 | F55-4 | 1799 | E | 0.0326 | A:0;G:0;C:8825;T:298;total:9123 | iSNV |
| F55 | F55-4 | 1911 | E | 0.0224 | A:6824;G:157;C:0;T:0;total:6981 | iSNV |
| F55 | F55-4 | 2372 | E | 0.9972 | A:1;G:0;C:14;T:5323;total:5338 | SNP |
| F55 | F55-4 | 2493 | NS1 | 0.9963 | A:22;G:5809;C:0;T:0;total:5831 | SNP |
| F55 | F55-4 | 2504 | NS1 | 0.9968 | A:18;G:5538;C:0;T:0;total:5556 | SNP |
| F55 | F55-4 | 2531 | NS1 | 0.9963 | A:2;G:0;C:6074;T:21;total:6097 | SNP |
| F55 | F55-4 | 2684 | NS1 | 0.0635 | A:7518;G:510;C:1;T:0;total:8029 | iSNV |
| F55 | F55-4 | 3572 | NS1 | 0.9958 | A:0;G:0;C:29;T:6855;total:6884 | SNP |
| F55 | F55-4 | 3716 | NS1 | 0.0708 | A:1;G:1;C:5614;T:428;total:6044 | iSNV |
| F55 | F55-4 | 3962 | NS2A | 0.9942 | A:1;G:0;C:50;T:8688;total:8739 | SNP |
| F55 | F55-4 | 4155 | NS2A | 0.996 | A:4470;G:2;C:0;T:16;total:4488 | SNP |
| F55 | F55-4 | 4397 | NS2B | 0.0242 | A:2;G:0;C:7276;T:181;total:7459 | iSNV |
| F55 | F55-4 | 4712 | NS3 | 0.9964 | A:10109;G:35;C:2;T:0;total:10146 | SNP |
| F55 | F55-4 | 5311 | NS3 | 0.9127 | A:1;G:1;C:635;T:6630;total:7267 | iSNV |
| F55 | F55-4 | 6080 | NS3 | 0.9945 | A:7951;G:44;C:0;T:0;total:7995 | SNP |
| F55 | F55-4 | 6971 | NS4A | 0.0859 | A:0;G:6637;C:0;T:624;total:7261 | iSNV |
| F55 | F55-4 | 7626 | NS4B | 0.021 | A:163;G:3;C:3;T:7579;total:7748 | iSNV |
| F55 | F55-4 | 7880 | NS5 | 0.0259 | A:9349;G:249;C:0;T:0;total:9598 | iSNV |
| F55 | F55-4 | 8282 | NS5 | 0.9959 | A:6;G:0;C:8884;T:31;total:8921 | SNP |
| F55 | F55-4 | 8900 | NS5 | 0.9895 | A:77;G:0;C:7414;T:2;total:7493 | SNP |
| F55 | F55-4 | 9370 | NS5 | 0.0303 | A:365;G:1;C:2;T:11673;total:12041 | iSNV |
| F55 | F55-4 | 9446 | NS5 | 0.9959 | A:48;G:12335;C:0;T:4;total:12387 | SNP |
| F55 | F55-4 | 10428 | 3'-UTR | 0.1375 | A:0;G:1;C:8335;T:1330;total:9666 | iSNV |
| F55 | F55-5 | 399 | C | 0.0319 | A:367;G:2;C:11124;T:3;total:11496 | iSNV |
| F55 | F55-5 | 563 | M | 0.9983 | A:9218;G:14;C:2;T:0;total:9234 | SNP |

| | | | | | | |
|---|---|---|---|---|---|---|
| F55 | F55-5 | 1116 | E | 0.0241 | A:8475;G:210;C:0;T:0;total:8685 | iSNV |
| F55 | F55-5 | 1257 | E | 0.9962 | A:8213;G:1;C:30;T:1;total:8245 | SNP |
| F55 | F55-5 | 1347 | E | 0.0229 | A:9900;G:233;C:0;T:0;total:10133 | iSNV |
| F55 | F55-5 | 1363 | E | 0.0516 | A:1;G:0;C:9383;T:511;total:9895 | iSNV |
| F55 | F55-5 | 1428 | E | 0.1219 | A:9648;G:1341;C:2;T:1;total:10992 | iSNV |
| F55 | F55-5 | 1473 | E | 0.0706 | A:9447;G:11;C:719;T:0;total:10177 | iSNV |
| F55 | F55-5 | 1512 | E | 0.5614 | A:4220;G:5400;C:0;T:0;total:9620 | iSNV |
| F55 | F55-5 | 1772 | E | 0.9981 | A:2;G:9798;C:0;T:17;total:9817 | SNP |
| F55 | F55-5 | 1799 | E | 0.0614 | A:0;G:0;C:10030;T:657;total:10687 | iSNV |
| F55 | F55-5 | 2372 | E | 0.9964 | A:0;G:2;C:22;T:6570;total:6594 | SNP |
| F55 | F55-5 | 2493 | NS1 | 0.9959 | A:23;G:6544;C:0;T:4;total:6571 | SNP |
| F55 | F55-5 | 2504 | NS1 | 0.9965 | A:21;G:6243;C:0;T:1;total:6265 | SNP |
| F55 | F55-5 | 2531 | NS1 | 0.9966 | A:3;G:0;C:6605;T:20;total:6628 | SNP |
| F55 | F55-5 | 2628 | NS1 | 0.5533 | A:4115;G:5095;C:0;T:1;total:9211 | iSNV |
| F55 | F55-5 | 3533 | NS1 | 0.0773 | A:0;G:568;C:5;T:6766;total:7339 | iSNV |
| F55 | F55-5 | 3572 | NS1 | 0.9995 | A:0;G:0;C:4;T:7296;total:7300 | SNP |
| F55 | F55-5 | 3962 | NS2A | 0.9963 | A:0;G:0;C:31;T:8320;total:8351 | SNP |
| F55 | F55-5 | 4155 | NS2A | 0.9943 | A:5005;G:8;C:2;T:19;total:5034 | SNP |
| F55 | F55-5 | 4712 | NS3 | 0.9979 | A:9815;G:20;C:1;T:0;total:9836 | SNP |
| F55 | F55-5 | 4720 | NS3 | 0.0848 | A:0;G:2;C:831;T:8966;total:9799 | iSNV |
| F55 | F55-5 | 5311 | NS3 | 0.9929 | A:0;G:7;C:46;T:7359;total:7412 | SNP |
| F55 | F55-5 | 6080 | NS3 | 0.9982 | A:8499;G:16;C:0;T:0;total:8515 | SNP |
| F55 | F55-5 | 6901 | NS4A | 0.0788 | A:1;G:3;C:526;T:6139;total:6669 | iSNV |
| F55 | F55-5 | 6934 | NS4A | 0.0323 | A:7215;G:241;C:0;T:0;total:7456 | iSNV |
| F55 | F55-5 | 6970 | NS4A | 0.084 | A:7092;G:1;C:651;T:3;total:7747 | iSNV |
| F55 | F55-5 | 7199 | NS4A | 0.0751 | A:1;G:0;C:5755;T:468;total:6224 | iSNV |
| F55 | F55-5 | 7561 | NS4B | 0.0724 | A:3;G:0;C:554;T:7093;total:7650 | iSNV |
| F55 | F55-5 | 8282 | NS5 | 0.9968 | A:0;G:0;C:8668;T:28;total:8696 | SNP |
| F55 | F55-5 | 8900 | NS5 | 0.9734 | A:19;G:1;C:7072;T:194;total:7286 | iSNV |
| F55 | F55-5 | 9370 | NS5 | 0.07 | A:807;G:1;C:4;T:10716;total:11528 | iSNV |
| F55 | F55-5 | 9446 | NS5 | 0.9986 | A:14;G:12005;C:1;T:3;total:12023 | SNP |
| F55 | F55-5 | 9533 | NS5 | 0.0286 | A:0;G:0;C:336;T:11372;total:11708 | iSNV |
| F55 | F55-5 | 10046 | NS5 | 0.0276 | A:7569;G:215;C:1;T:0;total:7785 | iSNV |
| F55 | F55-5 | 10428 | 3'-UTR | 0.0858 | A:0;G:1;C:8477;T:796;total:9274 | iSNV |
| F55 | F55-5 | 10447 | 3'-UTR | 0.0214 | A:0;G:8;C:8357;T:183;total:8548 | iSNV |
| F55 | F55-5 | 10642 | 3'-UTR | 0.5406 | A:1;G:0;C:4584;T:3897;total:8482 | iSNV |
| F55 | F55-6 | 563 | M | 0.9967 | A:12018;G:38;C:0;T:2;total:12058 | SNP |
| F55 | F55-6 | 1257 | E | 0.996 | A:11102;G:2;C:40;T:3;total:11147 | SNP |
| F55 | F55-6 | 1453 | E | 0.0362 | A:550;G:1;C:1;T:14602;total:15154 | iSNV |
| F55 | F55-6 | 1512 | E | 0.8099 | A:2388;G:10168;C:2;T:1;total:12559 | iSNV |
| F55 | F55-6 | 1772 | E | 0.9965 | A:2;G:13166;C:0;T:45;total:13213 | SNP |
| F55 | F55-6 | 1799 | E | 0.1357 | A:2;G:2;C:12341;T:1939;total:14284 | iSNV |
| F55 | F55-6 | 2372 | E | 0.9965 | A:3;G:0;C:28;T:8705;total:8736 | SNP |
| F55 | F55-6 | 2493 | NS1 | 0.9961 | A:34;G:8913;C:1;T:0;total:8948 | SNP |
| F55 | F55-6 | 2504 | NS1 | 0.9962 | A:33;G:8527;C:0;T:0;total:8560 | SNP |
| F55 | F55-6 | 2531 | NS1 | 0.9961 | A:1;G:2;C:9128;T:33;total:9164 | SNP |
| F55 | F55-6 | 3572 | NS1 | 0.9957 | A:3;G:1;C:37;T:9478;total:9519 | SNP |
| F55 | F55-6 | 3689 | NS1 | 0.0217 | A:0;G:0;C:173;T:7784;total:7957 | iSNV |
| F55 | F55-6 | 3962 | NS2A | 0.9955 | A:0;G:0;C:55;T:11952;total:12007 | SNP |
| F55 | F55-6 | 4155 | NS2A | 0.996 | A:6129;G:2;C:0;T:23;total:6154 | SNP |
| F55 | F55-6 | 4712 | NS3 | 0.9962 | A:13584;G:50;C:1;T:2;total:13637 | SNP |
| F55 | F55-6 | 5311 | NS3 | 0.9658 | A:1;G:7;C:357;T:10065;total:10430 | iSNV |
| F55 | F55-6 | 6080 | NS3 | 0.9958 | A:11589;G:49;C:0;T:1;total:11639 | SNP |
| F55 | F55-6 | 8282 | NS5 | 0.9965 | A:1;G:0;C:12341;T:43;total:12385 | SNP |
| F55 | F55-6 | 8891 | NS5 | 0.0247 | A:1;G:0;C:256;T:10102;total:10359 | iSNV |
| F55 | F55-6 | 8900 | NS5 | 0.9967 | A:28;G:0;C:10357;T:7;total:10392 | SNP |
| F55 | F55-6 | 9370 | NS5 | 0.1529 | A:2621;G:5;C:3;T:14507;total:17136 | iSNV |
| F55 | F55-6 | 9446 | NS5 | 0.9968 | A:51;G:17439;C:0;T:5;total:17495 | SNP |
| F55 | F55-6 | 10253 | NS5 | 0.0208 | A:15571;G:331;C:3;T:0;total:15905 | iSNV |
| F55 | F55-6 | 10428 | 3'-UTR | 0.1703 | A:1;G:0;C:10288;T:2112;total:12401 | iSNV |
| F55 | F55-6 | 10685 | 3'-UTR | 0.0522 | A:0;G:0;C:577;T:10474;total:11051 | iSNV |
| F55 | F55-7 | 563 | M | 0.9969 | A:9380;G:29;C:0;T:1;total:9410 | SNP |
| F55 | F55-7 | 1257 | E | 0.9944 | A:8622;G:1;C:48;T:0;total:8671 | SNP |
| F55 | F55-7 | 1447 | E | 0.8164 | A:8915;G:2;C:2006;T:0;total:10923 | iSNV |
| F55 | F55-7 | 1512 | E | 0.0244 | A:9612;G:241;C:6;T:0;total:9859 | iSNV |
| F55 | F55-7 | 1772 | E | 0.9967 | A:3;G:10264;C:1;T:31;total:10299 | SNP |
| F55 | F55-7 | 1799 | E | 0.0271 | A:1;G:1;C:10887;T:304;total:11193 | iSNV |
| F55 | F55-7 | 2018 | E | 0.0757 | A:2;G:0;C:6699;T:549;total:7250 | iSNV |
| F55 | F55-7 | 2372 | E | 0.9967 | A:0;G:0;C:23;T:6786;total:6809 | SNP |
| F55 | F55-7 | 2408 | E | 0.0277 | A:0;G:0;C:6105;T:174;total:6279 | iSNV |
| F55 | F55-7 | 2493 | NS1 | 0.9963 | A:25;G:6833;C:0;T:1;total:6859 | SNP |
| F55 | F55-7 | 2504 | NS1 | 0.9962 | A:25;G:6466;C:0;T:0;total:6491 | SNP |
| F55 | F55-7 | 2531 | NS1 | 0.9959 | A:1;G:0;C:6954;T:28;total:6983 | SNP |
| F55 | F55-7 | 2735 | NS1 | 0.0321 | A:2;G:0;C:10322;T:343;total:10667 | iSNV |
| F55 | F55-7 | 3530 | NS1 | 0.0291 | A:0;G:1;C:216;T:7199;total:7416 | iSNV |
| F55 | F55-7 | 3572 | NS1 | 0.9949 | A:0;G:0;C:38;T:7359;total:7397 | SNP |
| F55 | F55-7 | 3962 | NS2A | 0.9948 | A:2;G:0;C:42;T:8305;total:8349 | SNP |
| F55 | F55-7 | 4155 | NS2A | 0.9938 | A:4797;G:2;C:0;T:28;total:4827 | SNP |
| F55 | F55-7 | 4712 | NS3 | 0.9935 | A:10178;G:65;C:2;T:0;total:10245 | SNP |
| F55 | F55-7 | 5311 | NS3 | 0.99 | A:0;G:3;C:74;T:7567;total:7644 | SNP |
| F55 | F55-7 | 6080 | NS3 | 0.9959 | A:8716;G:36;C:0;T:0;total:8752 | SNP |
| F55 | F55-7 | 6969 | NS4A | 0.0234 | A:8011;G:0;C:192;T:0;total:8203 | iSNV |

| | | | | | | |
|---|---|---|---|---|---|---|
| F55 | F55-7 | 7322 | NS4B | 0.0288 | A:3;G:0;C:5618;T:167;total:5788 | iSNV |
| F55 | F55-7 | 7561 | NS4B | 0.0229 | A:0;G:4;C:184;T:7831;total:8019 | iSNV |
| F55 | F55-7 | 8084 | NS5 | 0.8098 | A:3;G:0;C:7744;T:1820;total:9567 | iSNV |
| F55 | F55-7 | 8282 | NS5 | 0.9963 | A:1;G:0;C:9041;T:33;total:9075 | SNP |
| F55 | F55-7 | 8900 | NS5 | 0.9964 | A:24;G:0;C:7552;T:4;total:7580 | SNP |
| F55 | F55-7 | 9008 | NS5 | 0.837 | A:0;G:1;C:8005;T:1560;total:9566 | iSNV |
| F55 | F55-7 | 9370 | NS5 | 0.0276 | A:343;G:2;C:4;T:12062;total:12411 | iSNV |
| F55 | F55-7 | 9392 | NS5 | 0.0254 | A:11558;G:302;C:2;T:3;total:11865 | iSNV |
| F55 | F55-7 | 9446 | NS5 | 0.9964 | A:46;G:12689;C:0;T:1;total:12736 | SNP |
| F55 | F55-7 | 10398 | 3'-UTR | 0.0298 | A:285;G:9269;C:0;T:0;total:9554 | iSNV |
| F55 | F55-7 | 10428 | 3'-UTR | 0.0966 | A:4;G:0;C:8470;T:907;total:9381 | iSNV |
| F55 | F55-8 | 563 | M | 0.9974 | A:11111;G:28;C:2;T:0;total:11141 | SNP |
| F55 | F55-8 | 1116 | E | 0.0402 | A:10956;G:459;C:0;T:1;total:11416 | iSNV |
| F55 | F55-8 | 1257 | E | 0.8604 | A:9335;G:4;C:1516;T:0;total:10855 | iSNV |
| F55 | F55-8 | 1428 | E | 0.0503 | A:13049;G:692;C:0;T:0;total:13741 | iSNV |
| F55 | F55-8 | 1512 | E | 0.6701 | A:3691;G:7493;C:2;T:0;total:11186 | iSNV |
| F55 | F55-8 | 1772 | E | 0.9974 | A:5;G:12222;C:3;T:25;total:12255 | SNP |
| F55 | F55-8 | 1799 | E | 0.0474 | A:3;G:3;C:12589;T:628;total:13223 | iSNV |
| F55 | F55-8 | 1883 | E | 0.0264 | A:2;G:0;C:10444;T:284;total:10730 | iSNV |
| F55 | F55-8 | 1911 | E | 0.0819 | A:8706;G:777;C:1;T:0;total:9484 | iSNV |
| F55 | F55-8 | 2372 | E | 0.998 | A:0;G:2;C:14;T:7675;total:7691 | SNP |
| F55 | F55-8 | 2493 | NS1 | 0.8441 | A:1257;G:6801;C:0;T:0;total:8058 | iSNV |
| F55 | F55-8 | 2504 | NS1 | 0.8365 | A:1268;G:6484;C:0;T:0;total:7752 | iSNV |
| F55 | F55-8 | 2531 | NS1 | 0.9968 | A:1;G:0;C:8521;T:27;total:8549 | SNP |
| F55 | F55-8 | 3290 | NS1 | 0.0241 | A:317;G:1;C:3;T:12813;total:13134 | iSNV |
| F55 | F55-8 | 3481 | NS1 | 0.0268 | A:0;G:176;C:0;T:6387;total:6563 | iSNV |
| F55 | F55-8 | 3572 | NS1 | 0.9978 | A:0;G:2;C:19;T:9275;total:9296 | SNP |
| F55 | F55-8 | 3962 | NS2A | 0.9975 | A:0;G:0;C:32;T:12444;total:12476 | SNP |
| F55 | F55-8 | 4155 | NS2A | 0.8551 | A:5137;G:0;C:2;T:871;total:6010 | iSNV |
| F55 | F55-8 | 4712 | NS3 | 0.9984 | A:14315;G:22;C:0;T:1;total:14338 | SNP |
| F55 | F55-8 | 5311 | NS3 | 0.942 | A:2;G:5;C:586;T:9502;total:10095 | iSNV |
| F55 | F55-8 | 5835 | NS3 | 0.0322 | A:473;G:14172;C:2;T:1;total:14648 | iSNV |
| F55 | F55-8 | 6080 | NS3 | 0.8972 | A:10031;G:1150;C:0;T:0;total:11181 | iSNV |
| F55 | F55-8 | 6746 | NS4A | 0.0686 | A:1;G:8827;C:0;T:651;total:9479 | iSNV |
| F55 | F55-8 | 7343 | NS4B | 0.0707 | A:7547;G:575;C:0;T:0;total:8122 | iSNV |
| F55 | F55-8 | 7583 | NS4B | 0.5891 | A:0;G:4200;C:1;T:6019;total:10220 | iSNV |
| F55 | F55-8 | 7656 | NS4B | 0.0409 | A:87;G:10620;C:457;T:3;total:11167 | iSNV |
| F55 | F55-8 | 7657 | NS4B | 0.0347 | A:10820;G:390;C:1;T:1;total:11212 | iSNV |
| F55 | F55-8 | 7744 | NS5 | 0.0738 | A:9305;G:742;C:1;T:1;total:10049 | iSNV |
| F55 | F55-8 | 7811 | NS5 | 0.0501 | A:544;G:1;C:9;T:10298;total:10852 | iSNV |
| F55 | F55-8 | 8135 | NS5 | 0.0466 | A:0;G:3;C:718;T:14680;total:15401 | iSNV |
| F55 | F55-8 | 8282 | NS5 | 0.9981 | A:2;G:0;C:12537;T:23;total:12562 | SNP |
| F55 | F55-8 | 8870 | NS5 | 0.0271 | A:286;G:10245;C:2;T:0;total:10533 | iSNV |
| F55 | F55-8 | 8900 | NS5 | 0.9943 | A:60;G:2;C:10922;T:1;total:10985 | SNP |
| F55 | F55-8 | 9370 | NS5 | 0.0522 | A:893;G:1;C:4;T:16183;total:17081 | iSNV |
| F55 | F55-8 | 9446 | NS5 | 0.9984 | A:24;G:17190;C:2;T:2;total:17218 | SNP |
| F55 | F55-8 | 9688 | NS5 | 0.0567 | A:3;G:2;C:10975;T:661;total:11641 | iSNV |
| F55 | F55-8 | 10428 | 3'-UTR | 0.0888 | A:1;G:0;C:11607;T:1132;total:12740 | iSNV |
| F55 | F55-9 | 563 | M | 0.9811 | A:5893;G:112;C:0;T:2;total:6007 | SNP |
| F55 | F55-9 | 1055 | E | 0.2092 | A:3894;G:1031;C:1;T:0;total:4926 | iSNV |
| F55 | F55-9 | 1257 | E | 0.9836 | A:5440;G:0;C:91;T:0;total:5531 | SNP |
| F55 | F55-9 | 1447 | E | 0.0291 | A:201;G:0;C:6677;T:8;total:6886 | iSNV |
| F55 | F55-9 | 1512 | E | 0.8941 | A:642;G:5419;C:0;T:1;total:6062 | iSNV |
| F55 | F55-9 | 1772 | E | 0.9836 | A:0;G:5726;C:0;T:96;total:5822 | SNP |
| F55 | F55-9 | 2372 | E | 0.9816 | A:3;G:0;C:72;T:3996;total:4071 | SNP |
| F55 | F55-9 | 2493 | NS1 | 0.9828 | A:72;G:4162;C:0;T:1;total:4235 | SNP |
| F55 | F55-9 | 2504 | NS1 | 0.9816 | A:73;G:3887;C:0;T:0;total:3960 | SNP |
| F55 | F55-9 | 2531 | NS1 | 0.9799 | A:0;G:0;C:4046;T:83;total:4129 | iSNV |
| F55 | F55-9 | 3399 | NS1 | 0.2049 | A:4669;G:1204;C:1;T:0;total:5874 | iSNV |
| F55 | F55-9 | 3572 | NS1 | 0.9836 | A:0;G:0;C:85;T:5068;total:5153 | SNP |
| F55 | F55-9 | 3962 | NS2A | 0.9781 | A:0;G:0;C:121;T:5386;total:5507 | iSNV |
| F55 | F55-9 | 4155 | NS2A | 0.9838 | A:3506;G:0;C:1;T:57;total:3564 | SNP |
| F55 | F55-9 | 4233 | NS2B | 0.0397 | A:0;G:151;C:0;T:3651;total:3802 | iSNV |
| F55 | F55-9 | 4595 | NS2B | 0.0256 | A:6224;G:164;C:0;T:0;total:6388 | iSNV |
| F55 | F55-9 | 4712 | NS3 | 0.9807 | A:6337;G:123;C:1;T:1;total:6462 | SNP |
| F55 | F55-9 | 5311 | NS3 | 0.8605 | A:0;G:0;C:713;T:4398;total:5111 | iSNV |
| F55 | F55-9 | 6080 | NS3 | 0.9803 | A:5573;G:112;C:0;T:0;total:5685 | SNP |
| F55 | F55-9 | 6524 | NS4A | 0.023 | A:113;G:4791;C:0;T:0;total:4904 | iSNV |
| F55 | F55-9 | 6971 | NS4A | 0.0743 | A:0;G:5136;C:416;T:44;total:5596 | iSNV |
| F55 | F55-9 | 7658 | NS4B | 0.038 | A:0;G:0;C:186;T:4702;total:4888 | iSNV |
| F55 | F55-9 | 8282 | NS5 | 0.9853 | A:0;G:0;C:5683;T:85;total:5768 | SNP |
| F55 | F55-9 | 8396 | NS5 | 0.5685 | A:2832;G:3730;C:0;T:1;total:6563 | iSNV |
| F55 | F55-9 | 8846 | NS5 | 0.0989 | A:4460;G:490;C:0;T:0;total:4950 | iSNV |
| F55 | F55-9 | 8900 | NS5 | 0.9801 | A:96;G:3;C:4861;T:0;total:4960 | SNP |
| F55 | F55-9 | 9365 | NS5 | 0.0376 | A:1;G:0;C:7384;T:289;total:7674 | iSNV |
| F55 | F55-9 | 9446 | NS5 | 0.9723 | A:221;G:7736;C:1;T:1;total:7959 | iSNV |
| F55 | F55-9 | 10010 | NS5 | 0.0744 | A:0;G:0;C:403;T:5011;total:5414 | iSNV |
| F55 | F55-9 | 10014 | NS5 | 0.0436 | A:5370;G:0;C:1;T:245;total:5616 | iSNV |
| F55 | F55-9 | 10092 | NS5 | 0.0329 | A:14;G:5390;C:184;T:0;total:5588 | iSNV |
| F55 | F55-9 | 10428 | 3'-UTR | 0.1642 | A:0;G:1;C:5025;T:988;total:6014 | iSNV |

Table S4: iSNV information in C6/36 cells

| Generation | Sample | Genome position | Gene | MuAF | Variation pattern | Type |
|---|---|---|---|---|---|---|
| F3 | F3-1 | 6533 | NS4A | 0.0235 | A:0;G:0;C:140;T:5808;total:5948 | iSNV |
| F3 | F3-1 | 9688 | NS5 | 0.0303 | A:0;G:0;C:8847;T:277;total:9124 | iSNV |
| F3 | F3-10 | 6533 | NS4A | 0.0256 | A:2;G:0;C:1280;T:48567;total:49849 | iSNV |
| F3 | F3-10 | 9688 | NS5 | 0.0259 | A:2;G:1;C:62093;T:1655;total:63751 | iSNV |
| F3 | F3-11 | 6533 | NS4A | 0.0319 | A:2;G:1;C:1147;T:34710;total:35860 | iSNV |
| F3 | F3-11 | 9688 | NS5 | 0.035 | A:1;G:1;C:44257;T:1610;total:45869 | iSNV |
| F3 | F3-13 | 9688 | NS5 | 0.021 | A:0;G:0;C:56337;T:1213;total:57550 | iSNV |
| F3 | F3-14 | 6533 | NS4A | 0.0213 | A:1;G:3;C:1086;T:49687;total:50777 | iSNV |
| F3 | F3-14 | 9688 | NS5 | 0.0213 | A:1;G:0;C:65042;T:1416;total:66459 | iSNV |
| F3 | F3-16 | 6533 | NS4A | 0.0261 | A:2;G:1;C:355;T:13234;total:13592 | iSNV |
| F3 | F3-16 | 9688 | NS5 | 0.0254 | A:3;G:1;C:19587;T:512;total:20103 | iSNV |
| F3 | F3-17 | 6533 | NS4A | 0.0266 | A:1;G:4;C:1212;T:44276;total:45493 | iSNV |
| F3 | F3-17 | 9688 | NS5 | 0.0288 | A:1;G:2;C:57263;T:1700;total:58966 | iSNV |
| F3 | F3-19 | 6533 | NS4A | 0.0233 | A:2;G:2;C:963;T:40211;total:41178 | iSNV |
| F3 | F3-19 | 9688 | NS5 | 0.0248 | A:4;G:0;C:51717;T:1319;total:53040 | iSNV |
| F3 | F3-2 | 1911 | E | 0.1475 | A:32051;G:5547;C:0;T:0;total:37598 | iSNV |
| F3 | F3-2 | 9688 | NS5 | 0.0222 | A:2;G:0;C:43905;T:999;total:44906 | iSNV |
| F3 | F3-2 | 10069 | NS5 | 0.1458 | A:1;G:0;C:36925;T:6305;total:43231 | iSNV |
| F3 | F3-20 | 6533 | NS4A | 0.0231 | A:3;G:1;C:715;T:30165;total:30884 | iSNV |
| F3 | F3-20 | 9688 | NS5 | 0.0292 | A:1;G:0;C:40035;T:1206;total:41242 | iSNV |
| F3 | F3-22 | 6533 | NS4A | 0.0271 | A:3;G:0;C:929;T:33343;total:34275 | iSNV |
| F3 | F3-22 | 9688 | NS5 | 0.0287 | A:2;G:0;C:41119;T:1218;total:42339 | iSNV |
| F3 | F3-23 | 6533 | NS4A | 0.032 | A:1;G:11;C:1130;T:34061;total:35203 | iSNV |
| F3 | F3-23 | 9688 | NS5 | 0.0277 | A:3;G:1;C:43990;T:1257;total:45251 | iSNV |
| F3 | F3-25 | 340 | C | 0.0254 | A:3;G:0;C:62628;T:1634;total:64265 | iSNV |
| F3 | F3-25 | 1911 | E | 0.0381 | A:48385;G:1921;C:0;T:0;total:50306 | iSNV |
| F3 | F3-25 | 6533 | NS4A | 0.0278 | A:0;G:1;C:1316;T:45990;total:47307 | iSNV |
| F3 | F3-25 | 9688 | NS5 | 0.0277 | A:1;G:0;C:56759;T:1620;total:58380 | iSNV |
| F3 | F3-25 | 10069 | NS5 | 0.0345 | A:0;G:0;C:52354;T:1872;total:54226 | iSNV |
| F3 | F3-26 | 6533 | NS4A | 0.0232 | A:3;G:5;C:1190;T:49995;total:51193 | iSNV |
| F3 | F3-26 | 9688 | NS5 | 0.0262 | A:1;G:0;C:61413;T:1656;total:63070 | iSNV |
| F3 | F3-28 | 6533 | NS4A | 0.0214 | A:3;G:0;C:1213;T:55382;total:56598 | iSNV |
| F3 | F3-28 | 9688 | NS5 | 0.0216 | A:2;G:0;C:65237;T:1445;total:66684 | iSNV |
| F3 | F3-30 | 9688 | NS5 | 0.0212 | A:1;G:0;C:87285;T:1899;total:89185 | iSNV |
| F3 | F3-4 | 6533 | NS4A | 0.0584 | A:3;G:12;C:3269;T:52687;total:55971 | iSNV |
| F3 | F3-4 | 9688 | NS5 | 0.065 | A:3;G:1;C:72766;T:5064;total:77834 | iSNV |
| F3 | F3-5 | 6533 | NS4A | 0.0247 | A:3;G:0;C:1218;T:47941;total:49162 | iSNV |
| F3 | F3-5 | 9688 | NS5 | 0.0277 | A:2;G:1;C:65453;T:1865;total:67321 | iSNV |
| F3 | F3-7 | 9688 | NS5 | 0.0291 | A:1;G:0;C:45272;T:1358;total:46631 | iSNV |
| F3 | F3-8 | 6533 | NS4A | 0.028 | A:3;G:2;C:1215;T:42109;total:43329 | iSNV |
| F3 | F3-8 | 9688 | NS5 | 0.0296 | A:1;G:0;C:55606;T:1700;total:57307 | iSNV |
| F5 | F5-1 | 1474 | E | 0.0295 | A:73410;G:11;C:2236;T:0;total:75657 | iSNV |
| F5 | F5-1 | 1803 | E | 0.035 | A:76732;G:2788;C:1;T:6;total:79527 | iSNV |
| F5 | F5-1 | 1911 | E | 0.0616 | A:58985;G:3875;C:3;T:3;total:62866 | iSNV |
| F5 | F5-1 | 2030 | E | 0.0338 | A:5;G:1;C:1794;T:51193;total:52993 | iSNV |
| F5 | F5-1 | 2567 | NS1 | 0.0325 | A:8;G:7;C:1774;T:52783;total:54572 | iSNV |
| F5 | F5-1 | 5311 | NS3 | 0.0292 | A:2;G:36;C:1869;T:61918;total:63825 | iSNV |
| F5 | F5-1 | 5358 | NS3 | 0.0375 | A:7;G:63361;C:2474;T:8;total:65850 | iSNV |
| F5 | F5-1 | 6232 | NS3 | 0.0221 | A:62182;G:1408;C:16;T:2;total:63608 | iSNV |
| F5 | F5-1 | 6533 | NS4A | 0.0771 | A:3;G:13;C:4544;T:54308;total:58868 | iSNV |
| F5 | F5-1 | 7495 | NS4B | 0.0318 | A:0;G:5;C:1599;T:48594;total:50198 | iSNV |
| F5 | F5-1 | 7626 | NS4B | 0.0269 | A:5;G:1936;C:27;T:69810;total:71778 | iSNV |
| F5 | F5-1 | 7657 | NS4B | 0.0257 | A:68887;G:1818;C:5;T:2;total:70712 | iSNV |
| F5 | F5-1 | 9476 | NS5 | 0.0205 | A:4;G:7;C:1761;T:83977;total:85749 | iSNV |
| F5 | F5-1 | 9607 | NS5 | 0.0207 | A:80261;G:18;C:1703;T:3;total:81985 | iSNV |
| F5 | F5-1 | 9688 | NS5 | 0.0906 | A:2;G:1;C:69803;T:6957;total:76763 | iSNV |
| F5 | F5-1 | 9932 | NS5 | 0.0206 | A:1512;G:4;C:29;T:71553;total:73098 | iSNV |
| F5 | F5-1 | 10069 | NS5 | 0.0219 | A:6;G:0;C:62129;T:1395;total:63530 | iSNV |
| F5 | F5-1 | 10071 | NS5 | 0.0556 | A:3551;G:3;C:10;T:60258;total:63822 | iSNV |
| F5 | F5-1 | 10092 | NS5 | 0.04 | A:2667;G:63952;C:0;T:17;total:66636 | iSNV |
| F5 | F5-10 | 1911 | E | 0.0541 | A:14833;G:849;C:0;T:0;total:15682 | iSNV |
| F5 | F5-10 | 5311 | NS3 | 0.0223 | A:1;G:10;C:489;T:21364;total:21864 | iSNV |
| F5 | F5-10 | 5782 | NS3 | 0.0256 | A:10535;G:278;C:7;T:2;total:10822 | iSNV |
| F5 | F5-10 | 7495 | NS4B | 0.0327 | A:0;G:1;C:542;T:15988;total:16531 | iSNV |
| F5 | F5-10 | 7657 | NS4B | 0.0416 | A:17688;G:769;C:25;T:0;total:18482 | iSNV |
| F5 | F5-10 | 8640 | NS5 | 0.0232 | A:0;G:0;C:21413;T:509;total:21922 | iSNV |
| F5 | F5-10 | 9688 | NS5 | 0.1135 | A:0;G:0;C:18128;T:2322;total:20450 | iSNV |
| F5 | F5-10 | 10069 | NS5 | 0.0293 | A:0;G:0;C:20037;T:606;total:20643 | iSNV |
| F5 | F5-11 | 6533 | NS4A | 0.124 | A:0;G:1;C:3029;T:21379;total:24409 | iSNV |
| F5 | F5-11 | 7495 | NS4B | 0.0314 | A:1;G:0;C:1033;T:31842;total:32876 | iSNV |
| F5 | F5-11 | 7656 | NS4B | 0.0237 | A:882;G:36177;C:2;T:2;total:37063 | iSNV |
| F5 | F5-11 | 7657 | NS4B | 0.0273 | A:35897;G:1009;C:24;T:1;total:36931 | iSNV |
| F5 | F5-11 | 9607 | NS5 | 0.0356 | A:30621;G:1;C:1131;T:2;total:31755 | iSNV |
| F5 | F5-11 | 9688 | NS5 | 0.1264 | A:3;G:0;C:38786;T:5615;total:44404 | iSNV |
| F5 | F5-11 | 10071 | NS5 | 0.0471 | A:2146;G:2;C:6;T:43376;total:45530 | iSNV |
| F5 | F5-13 | 1803 | E | 0.0282 | A:12952;G:377;C:0;T:0;total:13329 | iSNV |
| F5 | F5-13 | 1911 | E | 0.0243 | A:7982;G:199;C:0;T:0;total:8181 | iSNV |
| F5 | F5-13 | 5358 | NS3 | 0.0337 | A:0;G:11684;C:408;T:0;total:12092 | iSNV |

| | | | | | | |
|---|---|---|---|---|---|---|
| F5 | F5-13 | 6533 | NS4A | 0.0876 | A:0;G:1;C:468;T:4870;total:5339 | iSNV |
| F5 | F5-13 | 7495 | NS4B | 0.0458 | A:0;G:2;C:361;T:7512;total:7875 | iSNV |
| F5 | F5-13 | 7546 | NS4B | 0.0298 | A:9247;G:285;C:0;T:1;total:9533 | iSNV |
| F5 | F5-13 | 7657 | NS4B | 0.0391 | A:8745;G:356;C:3;T:0;total:9104 | iSNV |
| F5 | F5-13 | 9688 | NS5 | 0.0782 | A:1;G:0;C:9570;T:813;total:10384 | iSNV |
| F5 | F5-13 | 10069 | NS5 | 0.0417 | A:0;G:1;C:10249;T:447;total:10697 | iSNV |
| F5 | F5-13 | 10071 | NS5 | 0.034 | A:367;G:0;C:1;T:10416;total:10784 | iSNV |
| F5 | F5-14 | 1911 | E | 0.0715 | A:12063;G:930;C:0;T:1;total:12994 | iSNV |
| F5 | F5-14 | 5782 | NS3 | 0.0264 | A:10656;G:290;C:6;T:0;total:10952 | iSNV |
| F5 | F5-14 | 5835 | NS3 | 0.0296 | A:345;G:11276;C:0;T:0;total:11621 | iSNV |
| F5 | F5-14 | 6533 | NS4A | 0.076 | A:0;G:0;C:633;T:7695;total:8328 | iSNV |
| F5 | F5-14 | 7495 | NS4B | 0.0345 | A:1;G:2;C:447;T:12473;total:12923 | iSNV |
| F5 | F5-14 | 7657 | NS4B | 0.0468 | A:13976;G:687;C:9;T:1;total:14673 | iSNV |
| F5 | F5-14 | 9688 | NS5 | 0.0718 | A:0;G:0;C:16475;T:1275;total:17750 | iSNV |
| F5 | F5-14 | 10069 | NS5 | 0.0583 | A:0;G:0;C:17741;T:1100;total:18841 | iSNV |
| F5 | F5-14 | 10092 | NS5 | 0.0251 | A:484;G:18768;C:0;T:2;total:19254 | iSNV |
| F5 | F5-16 | 1911 | E | 0.0628 | A:69839;G:4682;C:6;T:3;total:74530 | iSNV |
| F5 | F5-16 | 5311 | NS3 | 0.0398 | A:8;G:26;C:2863;T:68954;total:71851 | iSNV |
| F5 | F5-16 | 6533 | NS4A | 0.0876 | A:7;G:9;C:5866;T:61026;total:66908 | iSNV |
| F5 | F5-16 | 7495 | NS4B | 0.0403 | A:5;G:4;C:2332;T:55421;total:57762 | iSNV |
| F5 | F5-16 | 7657 | NS4B | 0.0317 | A:74208;G:2433;C:37;T:2;total:76680 | iSNV |
| F5 | F5-16 | 9688 | NS5 | 0.1007 | A:12;G:5;C:83295;T:9331;total:92643 | iSNV |
| F5 | F5-16 | 9932 | NS5 | 0.0225 | A:1901;G:8;C:27;T:82487;total:84423 | iSNV |
| F5 | F5-16 | 10069 | NS5 | 0.0531 | A:4;G:1;C:68535;T:3850;total:72390 | iSNV |
| F5 | F5-17 | 1911 | E | 0.0206 | A:12122;G:255;C:0;T:0;total:12377 | iSNV |
| F5 | F5-17 | 7495 | NS4B | 0.037 | A:0;G:1;C:476;T:12360;total:12837 | iSNV |
| F5 | F5-17 | 7657 | NS4B | 0.0252 | A:13791;G:357;C:5;T:0;total:14153 | iSNV |
| F5 | F5-17 | 9688 | NS5 | 0.117 | A:0;G:0;C:14025;T:1859;total:15884 | iSNV |
| F5 | F5-17 | 10069 | NS5 | 0.0331 | A:0;G:0;C:14183;T:486;total:14669 | iSNV |
| F5 | F5-19 | 1911 | E | 0.0257 | A:25355;G:671;C:0;T:0;total:26026 | iSNV |
| F5 | F5-19 | 6533 | NS4A | 0.1017 | A:2;G:0;C:1162;T:10255;total:11419 | iSNV |
| F5 | F5-19 | 7495 | NS4B | 0.0321 | A:0;G:2;C:880;T:26511;total:27393 | iSNV |
| F5 | F5-19 | 7657 | NS4B | 0.0384 | A:29204;G:1170;C:65;T:0;total:30439 | iSNV |
| F5 | F5-19 | 9688 | NS5 | 0.1 | A:0;G:0;C:29615;T:3292;total:32907 | iSNV |
| F5 | F5-19 | 10069 | NS5 | 0.0306 | A:0;G:0;C:30053;T:951;total:31004 | iSNV |
| F5 | F5-2 | 1911 | E | 0.6258 | A:22055;G:36882;C:0;T:2;total:58939 | iSNV |
| F5 | F5-2 | 5311 | NS3 | 0.0456 | A:0;G:13;C:2907;T:60814;total:63734 | iSNV |
| F5 | F5-2 | 6533 | NS4A | 0.0217 | A:2;G:2;C:1290;T:58115;total:59409 | iSNV |
| F5 | F5-2 | 9688 | NS5 | 0.0278 | A:0;G:0;C:67042;T:1921;total:68963 | iSNV |
| F5 | F5-2 | 10069 | NS5 | 0.6358 | A:1;G:7;C:21620;T:37734;total:59362 | iSNV |
| F5 | F5-20 | 1911 | E | 0.0262 | A:16422;G:442;C:1;T:0;total:16865 | iSNV |
| F5 | F5-20 | 6533 | NS4A | 0.1086 | A:0;G:0;C:862;T:7071;total:7933 | iSNV |
| F5 | F5-20 | 7495 | NS4B | 0.0395 | A:2;G:4;C:680;T:16523;total:17209 | iSNV |
| F5 | F5-20 | 7657 | NS4B | 0.037 | A:18452;G:710;C:27;T:0;total:19189 | iSNV |
| F5 | F5-20 | 9688 | NS5 | 0.115 | A:1;G:0;C:18498;T:2405;total:20904 | iSNV |
| F5 | F5-20 | 10069 | NS5 | 0.0347 | A:0;G:0;C:19519;T:703;total:20222 | iSNV |
| F5 | F5-22 | 1384 | E | 0.061 | A:23818;G:1;C:9;T:1550;total:25378 | iSNV |
| F5 | F5-22 | 1911 | E | 0.0247 | A:19456;G:494;C:1;T:0;total:19951 | iSNV |
| F5 | F5-22 | 5311 | NS3 | 0.0753 | A:0;G:6;C:2062;T:25297;total:27365 | iSNV |
| F5 | F5-22 | 5626 | NS3 | 0.0275 | A:0;G:0;C:546;T:19267;total:19813 | iSNV |
| F5 | F5-22 | 6533 | NS4A | 0.1007 | A:1;G:0;C:921;T:8217;total:9139 | iSNV |
| F5 | F5-22 | 7495 | NS4B | 0.0293 | A:5;G:2;C:617;T:20384;total:21008 | iSNV |
| F5 | F5-22 | 7657 | NS4B | 0.0275 | A:22660;G:644;C:36;T:0;total:23340 | iSNV |
| F5 | F5-22 | 9225 | NS5 | 0.0569 | A:896;G:0;C:14835;T:2;total:15733 | iSNV |
| F5 | F5-22 | 9688 | NS5 | 0.1075 | A:1;G:0;C:22320;T:2690;total:25011 | iSNV |
| F5 | F5-22 | 10069 | NS5 | 0.0221 | A:0;G:1;C:23408;T:531;total:23940 | iSNV |
| F5 | F5-23 | 1911 | E | 0.0511 | A:18215;G:983;C:2;T:1;total:19201 | iSNV |
| F5 | F5-23 | 7495 | NS4B | 0.0332 | A:1;G:1;C:647;T:18796;total:19445 | iSNV |
| F5 | F5-23 | 7657 | NS4B | 0.0368 | A:21409;G:819;C:27;T:0;total:22255 | iSNV |
| F5 | F5-23 | 9688 | NS5 | 0.1125 | A:1;G:0;C:21165;T:2684;total:23850 | iSNV |
| F5 | F5-25 | 340 | C | 0.2377 | A:5;G:1;C:26772;T:8353;total:35131 | iSNV |
| F5 | F5-25 | 1911 | E | 0.2927 | A:22492;G:9309;C:1;T:0;total:31802 | iSNV |
| F5 | F5-25 | 2882 | NS1 | 0.0361 | A:62213;G:2333;C:1;T:2;total:64549 | iSNV |
| F5 | F5-25 | 7495 | NS4B | 0.0257 | A:2;G:1;C:877;T:33145;total:34025 | iSNV |
| F5 | F5-25 | 7657 | NS4B | 0.0339 | A:40246;G:1415;C:27;T:0;total:41688 | iSNV |
| F5 | F5-25 | 9688 | NS5 | 0.089 | A:0;G:0;C:36973;T:3614;total:40587 | iSNV |
| F5 | F5-25 | 10069 | NS5 | 0.321 | A:0;G:0;C:28556;T:13505;total:42061 | iSNV |
| F5 | F5-26 | 1911 | E | 0.0954 | A:22754;G:2402;C:0;T:0;total:25156 | iSNV |
| F5 | F5-26 | 6533 | NS4A | 0.1041 | A:0;G:1;C:1555;T:13381;total:14937 | iSNV |
| F5 | F5-26 | 7495 | NS4B | 0.0253 | A:1;G:3;C:628;T:24154;total:24786 | iSNV |
| F5 | F5-26 | 7657 | NS4B | 0.0316 | A:29021;G:950;C:14;T:1;total:29986 | iSNV |
| F5 | F5-26 | 9688 | NS5 | 0.0987 | A:0;G:0;C:27166;T:2977;total:30143 | iSNV |
| F5 | F5-26 | 10069 | NS5 | 0.1171 | A:0;G:3;C:27724;T:3678;total:31405 | iSNV |
| F5 | F5-28 | 1911 | E | 0.0261 | A:22817;G:613;C:1;T:0;total:23431 | iSNV |
| F5 | F5-28 | 7494 | NS4B | 0.0262 | A:4;G:23107;C:0;T:623;total:23734 | iSNV |
| F5 | F5-28 | 7495 | NS4B | 0.0546 | A:2;G:0;C:1345;T:23253;total:24600 | iSNV |
| F5 | F5-28 | 7656 | NS4B | 0.0293 | A:863;G:28522;C:1;T:0;total:29386 | iSNV |
| F5 | F5-28 | 7657 | NS4B | 0.0477 | A:27810;G:1397;C:26;T:1;total:29234 | iSNV |
| F5 | F5-28 | 8987 | NS5 | 0.0234 | A:600;G:0;C:3;T:24976;total:25579 | iSNV |
| F5 | F5-28 | 9688 | NS5 | 0.0844 | A:1;G:1;C:27057;T:2495;total:29554 | iSNV |
| F5 | F5-28 | 10069 | NS5 | 0.0469 | A:0;G:0;C:28026;T:1381;total:29407 | iSNV |
| F5 | F5-30 | 3230 | NS1 | 0.0218 | A:15339;G:342;C:0;T:0;total:15681 | iSNV |

| | | | | | | |
|---|---|---|---|---|---|---|
| F5 | F5-30 | 6533 | NS4A | 0.1244 | A:0;G:0;C:812;T:5711;total:6523 | iSNV |
| F5 | F5-30 | 7495 | NS4B | 0.0289 | A:1;G:1;C:518;T:17381;total:17901 | iSNV |
| F5 | F5-30 | 7657 | NS4B | 0.0576 | A:19751;G:1209;C:16;T:1;total:20977 | iSNV |
| F5 | F5-30 | 9688 | NS5 | 0.0998 | A:1;G:0;C:20214;T:2243;total:22458 | iSNV |
| F5 | F5-4 | 1911 | E | 0.0209 | A:19059;G:408;C:1;T:0;total:19468 | iSNV |
| F5 | F5-4 | 7657 | NS4B | 0.0384 | A:19281;G:771;C:11;T:0;total:20063 | iSNV |
| F5 | F5-4 | 9220 | NS5 | 0.0261 | A:15077;G:3;C:405;T:1;total:15486 | iSNV |
| F5 | F5-4 | 9688 | NS5 | 0.1705 | A:3;G:1;C:20517;T:4220;total:24741 | iSNV |
| F5 | F5-4 | 10069 | NS5 | 0.047 | A:2;G:0;C:23362;T:1154;total:24518 | iSNV |
| F5 | F5-4 | 10092 | NS5 | 0.0266 | A:663;G:24174;C:0;T:6;total:24843 | iSNV |
| F5 | F5-5 | 1911 | E | 0.0527 | A:24195;G:1347;C:3;T:0;total:25545 | iSNV |
| F5 | F5-5 | 5311 | NS3 | 0.0247 | A:0;G:26;C:798;T:31379;total:32203 | iSNV |
| F5 | F5-5 | 7322 | NS4B | 0.0362 | A:1;G:0;C:20529;T:772;total:21302 | iSNV |
| F5 | F5-5 | 7495 | NS4B | 0.0344 | A:2;G:1;C:883;T:24751;total:25637 | iSNV |
| F5 | F5-5 | 7657 | NS4B | 0.039 | A:26540;G:1081;C:32;T:2;total:27655 | iSNV |
| F5 | F5-5 | 9688 | NS5 | 0.1055 | A:1;G:0;C:29810;T:3516;total:33327 | iSNV |
| F5 | F5-5 | 10092 | NS5 | 0.0256 | A:857;G:32588;C:1;T:7;total:33453 | iSNV |
| F5 | F5-5 | 10097 | NS5 | 0.0385 | A:2;G:33173;C:0;T:1329;total:34504 | iSNV |
| F5 | F5-5 | 10797 | 3'-UTR | 0.0387 | A:8;G:16723;C:5;T:675;total:17411 | iSNV |
| F5 | F5-7 | 1911 | E | 0.0206 | A:9286;G:196;C:0;T:0;total:9482 | iSNV |
| F5 | F5-7 | 3139 | NS1 | 0.0583 | A:11899;G:737;C:0;T:1;total:12637 | iSNV |
| F5 | F5-7 | 7495 | NS4B | 0.0872 | A:1;G:0;C:805;T:8418;total:9224 | iSNV |
| F5 | F5-7 | 7657 | NS4B | 0.027 | A:9832;G:274;C:5;T:0;total:10111 | iSNV |
| F5 | F5-7 | 8225 | NS5 | 0.0325 | A:0;G:0;C:310;T:9201;total:9511 | iSNV |
| F5 | F5-7 | 9688 | NS5 | 0.1516 | A:0;G:0;C:10804;T:1931;total:12735 | iSNV |
| F5 | F5-7 | 10069 | NS5 | 0.0574 | A:0;G:0;C:11723;T:715;total:12438 | iSNV |
| F5 | F5-8 | 1786 | E | 0.0228 | A:1;G:5;C:496;T:21199;total:21701 | iSNV |
| F5 | F5-8 | 1911 | E | 0.024 | A:12923;G:319;C:4;T:0;total:13246 | iSNV |
| F5 | F5-8 | 7495 | NS4B | 0.0343 | A:1;G:3;C:497;T:13987;total:14488 | iSNV |
| F5 | F5-8 | 7657 | NS4B | 0.0489 | A:15104;G:777;C:3;T:1;total:15885 | iSNV |
| F5 | F5-8 | 9688 | NS5 | 0.1163 | A:0;G:0;C:15858;T:2088;total:17946 | iSNV |
| F5 | F5-8 | 10069 | NS5 | 0.0329 | A:0;G:1;C:16689;T:568;total:17258 | iSNV |
| F10 | F10-1 | 1417 | E | 0.2246 | A:1;G:2;C:14646;T:4245;total:18894 | iSNV |
| F10 | F10-1 | 1474 | E | 0.0885 | A:14854;G:1;C:1443;T:4;total:16302 | iSNV |
| F10 | F10-1 | 1803 | E | 0.1778 | A:17133;G:3706;C:0;T:1;total:20840 | iSNV |
| F10 | F10-1 | 1911 | E | 0.2462 | A:11487;G:3753;C:2;T:0;total:15242 | iSNV |
| F10 | F10-1 | 2030 | E | 0.0997 | A:1;G:2;C:1414;T:12760;total:14177 | iSNV |
| F10 | F10-1 | 2567 | NS1 | 0.0956 | A:0;G:8;C:1325;T:12524;total:13857 | iSNV |
| F10 | F10-1 | 3993 | NS2A | 0.1574 | A:3038;G:16252;C:1;T:1;total:19292 | iSNV |
| F10 | F10-1 | 5311 | NS3 | 0.2326 | A:0;G:10;C:3950;T:13019;total:16979 | iSNV |
| F10 | F10-1 | 5358 | NS3 | 0.1818 | A:1;G:14461;C:3215;T:0;total:17677 | iSNV |
| F10 | F10-1 | 6533 | NS4A | 0.0234 | A:2;G:6;C:369;T:15377;total:15754 | iSNV |
| F10 | F10-1 | 7495 | NS4B | 0.217 | A:0;G:4;C:2968;T:10701;total:13673 | iSNV |
| F10 | F10-1 | 7526 | NS4B | 0.0567 | A:903;G:2;C:39;T:14980;total:15924 | iSNV |
| F10 | F10-1 | 7626 | NS4B | 0.2341 | A:1;G:4850;C:7;T:15855;total:20713 | iSNV |
| F10 | F10-1 | 7657 | NS4B | 0.0589 | A:19342;G:1211;C:5;T:0;total:20558 | iSNV |
| F10 | F10-1 | 9669 | NS5 | 0.0279 | A:15510;G:446;C:3;T:1;total:15960 | iSNV |
| F10 | F10-1 | 9688 | NS5 | 0.027 | A:0;G:0;C:16316;T:454;total:16770 | iSNV |
| F10 | F10-1 | 9932 | NS5 | 0.2247 | A:3717;G:1;C:7;T:12811;total:16536 | iSNV |
| F10 | F10-1 | 10071 | NS5 | 0.1776 | A:2577;G:0;C:5;T:11923;total:14505 | iSNV |
| F10 | F10-1 | 10086 | NS5 | 0.0895 | A:1352;G:4;C:7;T:13732;total:15095 | iSNV |
| F10 | F10-1 | 10092 | NS5 | 0.0219 | A:341;G:15160;C:1;T:2;total:15504 | iSNV |
| F10 | F10-10 | 1406 | E | 0.0291 | A:1218;G:40571;C:1;T:6;total:41796 | iSNV |
| F10 | F10-10 | 1786 | E | 0.0227 | A:3;G:5;C:1087;T:46725;total:47820 | iSNV |
| F10 | F10-10 | 1911 | E | 0.3142 | A:18295;G:8387;C:10;T:0;total:26692 | iSNV |
| F10 | F10-10 | 3949 | NS2A | 0.0222 | A:1;G:0;C:958;T:42035;total:42994 | iSNV |
| F10 | F10-10 | 4394 | NS2B | 0.0249 | A:0;G:0;C:28654;T:734;total:29388 | iSNV |
| F10 | F10-10 | 5311 | NS3 | 0.0424 | A:1;G:24;C:1572;T:35466;total:37063 | iSNV |
| F10 | F10-10 | 6368 | NS3 | 0.0375 | A:1;G:846;C:14;T:21690;total:22551 | iSNV |
| F10 | F10-10 | 6533 | NS4A | 0.1128 | A:2;G:0;C:2063;T:16217;total:18282 | iSNV |
| F10 | F10-10 | 7495 | NS4B | 0.1496 | A:2;G:2;C:4446;T:25269;total:29719 | iSNV |
| F10 | F10-10 | 7657 | NS4B | 0.2772 | A:25316;G:9716;C:12;T:1;total:35045 | iSNV |
| F10 | F10-10 | 8640 | NS5 | 0.0685 | A:4;G:0;C:40918;T:3013;total:43935 | iSNV |
| F10 | F10-10 | 9688 | NS5 | 0.1436 | A:2;G:0;C:31644;T:5307;total:36953 | iSNV |
| F10 | F10-10 | 9932 | NS5 | 0.0518 | A:1516;G:0;C:6;T:27695;total:29217 | iSNV |
| F10 | F10-10 | 10069 | NS5 | 0.2002 | A:2;G:0;C:31141;T:7799;total:38942 | iSNV |
| F10 | F10-11 | 1421 | E | 0.0208 | A:0;G:1;C:479;T:22518;total:22998 | iSNV |
| F10 | F10-11 | 1803 | E | 0.0403 | A:26705;G:1124;C:0;T:0;total:27829 | iSNV |
| F10 | F10-11 | 1911 | E | 0.1544 | A:13143;G:2401;C:3;T:0;total:15547 | iSNV |
| F10 | F10-11 | 1913 | E | 0.0381 | A:13992;G:1;C:555;T:8;total:14556 | iSNV |
| F10 | F10-11 | 5311 | NS3 | 0.0586 | A:2;G:17;C:1224;T:19624;total:20867 | iSNV |
| F10 | F10-11 | 5358 | NS3 | 0.0414 | A:4;G:23247;C:1006;T:0;total:24257 | iSNV |
| F10 | F10-11 | 6523 | NS4A | 0.1258 | A:0;G:1;C:7771;T:1119;total:8891 | iSNV |
| F10 | F10-11 | 6533 | NS4A | 0.1435 | A:0;G:0;C:1296;T:7733;total:9029 | iSNV |
| F10 | F10-11 | 7495 | NS4B | 0.147 | A:0;G:0;C:2449;T:14208;total:16657 | iSNV |
| F10 | F10-11 | 7656 | NS4B | 0.1145 | A:2272;G:17569;C:0;T:0;total:19841 | iSNV |
| F10 | F10-11 | 7657 | NS4B | 0.1573 | A:16675;G:3113;C:1;T:0;total:19789 | iSNV |
| F10 | F10-11 | 8900 | NS5 | 0.0308 | A:591;G:12;C:18550;T:2;total:19155 | iSNV |
| F10 | F10-11 | 9607 | NS5 | 0.1362 | A:12226;G:1;C:1929;T:1;total:14157 | iSNV |
| F10 | F10-11 | 9688 | NS5 | 0.1439 | A:2;G:0;C:17812;T:2996;total:20810 | iSNV |
| F10 | F10-11 | 9932 | NS5 | 0.086 | A:1361;G:0;C:1;T:14449;total:15811 | iSNV |
| F10 | F10-11 | 10071 | NS5 | 0.1806 | A:3887;G:0;C:0;T:17627;total:21514 | iSNV |

| | | | | | | |
|---|---|---|---|---|---|---|
| F10 | F10-13 | 344 | C | 0.0819 | A:10;G:8655;C:23;T:96966;total:105654 | iSNV |
| F10 | F10-13 | 542 | M | 0.0646 | A:43;G:6880;C:46;T:99373;total:106342 | iSNV |
| F10 | F10-13 | 1435 | E | 0.0302 | A:2606;G:83477;C:0;T:6;total:86089 | iSNV |
| F10 | F10-13 | 1803 | E | 0.3038 | A:78187;G:34128;C:3;T:2;total:112320 | iSNV |
| F10 | F10-13 | 1911 | E | 0.0934 | A:60038;G:6195;C:50;T:2;total:66285 | iSNV |
| F10 | F10-13 | 3926 | NS2A | 0.0645 | A:7;G:5;C:90988;T:6281;total:97281 | iSNV |
| F10 | F10-13 | 4943 | NS3 | 0.1714 | A:60476;G:12516;C:1;T:5;total:72998 | iSNV |
| F10 | F10-13 | 5358 | NS3 | 0.3137 | A:3;G:67055;C:30659;T:6;total:97723 | iSNV |
| F10 | F10-13 | 6533 | NS4A | 0.0393 | A:2;G:3;C:2133;T:52125;total:54263 | iSNV |
| F10 | F10-13 | 7167 | NS4A | 0.0242 | A:1914;G:77092;C:1;T:3;total:79010 | iSNV |
| F10 | F10-13 | 7495 | NS4B | 0.2816 | A:6;G:3;C:19538;T:49816;total:69363 | iSNV |
| F10 | F10-13 | 7546 | NS4B | 0.3275 | A:54305;G:26466;C:18;T:2;total:80791 | iSNV |
| F10 | F10-13 | 7656 | NS4B | 0.0333 | A:2835;G:82199;C:15;T:3;total:85052 | iSNV |
| F10 | F10-13 | 7657 | NS4B | 0.1551 | A:71583;G:13146;C:19;T:3;total:84751 | iSNV |
| F10 | F10-13 | 9688 | NS5 | 0.0587 | A:6;G:2;C:77536;T:4841;total:82385 | iSNV |
| F10 | F10-13 | 9932 | NS5 | 0.0558 | A:4479;G:8;C:24;T:75694;total:80205 | iSNV |
| F10 | F10-13 | 10069 | NS5 | 0.0256 | A:2;G:0;C:93291;T:2456;total:95749 | iSNV |
| F10 | F10-13 | 10071 | NS5 | 0.3043 | A:29093;G:1;C:12;T:66491;total:95597 | iSNV |
| F10 | F10-13 | 10092 | NS5 | 0.2294 | A:22694;G:76215;C:2;T:5;total:98916 | iSNV |
| F10 | F10-14 | 1911 | E | 0.4685 | A:54113;G:47724;C:9;T:1;total:101847 | iSNV |
| F10 | F10-14 | 2541 | NS1 | 0.02 | A:9;G:0;C:111965;T:2293;total:114267 | iSNV |
| F10 | F10-14 | 4394 | NS2B | 0.0247 | A:1;G:2;C:127892;T:3244;total:131139 | iSNV |
| F10 | F10-14 | 5311 | NS3 | 0.0378 | A:12;G:51;C:5327;T:135346;total:140736 | iSNV |
| F10 | F10-14 | 5835 | NS3 | 0.1202 | A:13156;G:96264;C:6;T:2;total:109428 | iSNV |
| F10 | F10-14 | 6533 | NS4A | 0.0336 | A:3;G:7;C:2228;T:63906;total:66144 | iSNV |
| F10 | F10-14 | 7495 | NS4B | 0.0677 | A:10;G:25;C:8092;T:111351;total:119478 | iSNV |
| F10 | F10-14 | 7657 | NS4B | 0.2985 | A:89465;G:38153;C:189;T:6;total:127813 | iSNV |
| F10 | F10-14 | 9688 | NS5 | 0.0403 | A:5;G:2;C:125273;T:5272;total:130552 | iSNV |
| F10 | F10-14 | 9932 | NS5 | 0.0414 | A:4812;G:8;C:44;T:111327;total:116191 | iSNV |
| F10 | F10-14 | 10069 | NS5 | 0.388 | A:3;G:0;C:87175;T:55270;total:142448 | iSNV |
| F10 | F10-14 | 10092 | NS5 | 0.0326 | A:4941;G:146451;C:31;T:10;total:151433 | iSNV |
| F10 | F10-16 | 1911 | E | 0.5911 | A:6540;G:9448;C:3;T:2;total:15993 | iSNV |
| F10 | F10-16 | 3685 | NS1 | 0.2179 | A:15085;G:4204;C:2;T:1;total:19292 | iSNV |
| F10 | F10-16 | 5311 | NS3 | 0.0807 | A:0;G:27;C:1444;T:16422;total:17893 | iSNV |
| F10 | F10-16 | 5312 | NS3 | 0.036 | A:0;G:5;C:641;T:17132;total:17778 | iSNV |
| F10 | F10-16 | 6533 | NS4A | 0.0338 | A:0;G:5;C:582;T:16603;total:17190 | iSNV |
| F10 | F10-16 | 7495 | NS4B | 0.097 | A:0;G:10;C:1380;T:12826;total:14216 | iSNV |
| F10 | F10-16 | 7656 | NS4B | 0.0423 | A:960;G:21700;C:0;T:5;total:22665 | iSNV |
| F10 | F10-16 | 7657 | NS4B | 0.1651 | A:18920;G:3745;C:8;T:1;total:22674 | iSNV |
| F10 | F10-16 | 9688 | NS5 | 0.047 | A:3;G:0;C:16927;T:835;total:17765 | iSNV |
| F10 | F10-16 | 9932 | NS5 | 0.0497 | A:888;G:7;C:3;T:16955;total:17853 | iSNV |
| F10 | F10-16 | 10069 | NS5 | 0.5314 | A:1;G:1;C:6903;T:7825;total:14730 | iSNV |
| F10 | F10-16 | 10808 | 3'-UTR | 0.0773 | A:1229;G:14655;C:1;T:1;total:15886 | iSNV |
| F10 | F10-17 | 1298 | E | 0.0221 | A:1845;G:23;C:16;T:81415;total:83299 | iSNV |
| F10 | F10-17 | 1432 | E | 0.0373 | A:69779;G:9;C:15;T:2710;total:72513 | iSNV |
| F10 | F10-17 | 1786 | E | 0.0302 | A:3;G:9;C:2967;T:95252;total:98231 | iSNV |
| F10 | F10-17 | 1911 | E | 0.1655 | A:51677;G:10253;C:8;T:1;total:61939 | iSNV |
| F10 | F10-17 | 4394 | NS2B | 0.0227 | A:0;G:0;C:75074;T:1745;total:76819 | iSNV |
| F10 | F10-17 | 5311 | NS3 | 0.0365 | A:5;G:56;C:2909;T:76564;total:79534 | iSNV |
| F10 | F10-17 | 5664 | NS3 | 0.0475 | A:79977;G:3990;C:0;T:2;total:83969 | iSNV |
| F10 | F10-17 | 6533 | NS4A | 0.106 | A:5;G:5;C:4822;T:40639;total:45471 | iSNV |
| F10 | F10-17 | 7495 | NS4B | 0.1505 | A:4;G:3;C:9749;T:55001;total:64757 | iSNV |
| F10 | F10-17 | 7543 | NS4B | 0.0252 | A:70994;G:1837;C:2;T:1;total:72834 | iSNV |
| F10 | F10-17 | 7656 | NS4B | 0.1975 | A:14462;G:58744;C:15;T:4;total:73225 | iSNV |
| F10 | F10-17 | 7657 | NS4B | 0.1882 | A:59296;G:13760;C:25;T:1;total:73082 | iSNV |
| F10 | F10-17 | 8656 | NS5 | 0.0301 | A:5;G:2;C:2456;T:79115;total:81578 | iSNV |
| F10 | F10-17 | 8659 | NS5 | 0.0312 | A:75453;G:2434;C:22;T:3;total:77912 | iSNV |
| F10 | F10-17 | 9688 | NS5 | 0.1234 | A:4;G:0;C:63241;T:8910;total:72155 | iSNV |
| F10 | F10-17 | 9932 | NS5 | 0.0866 | A:5977;G:2;C:24;T:62961;total:68964 | iSNV |
| F10 | F10-17 | 10069 | NS5 | 0.0702 | A:1;G:0;C:77539;T:5863;total:83403 | iSNV |
| F10 | F10-19 | 1911 | E | 0.1143 | A:43550;G:5623;C:11;T:0;total:49184 | iSNV |
| F10 | F10-19 | 4394 | NS2B | 0.0222 | A:2;G:0;C:60595;T:1379;total:61976 | iSNV |
| F10 | F10-19 | 4817 | NS3 | 0.0206 | A:7;G:5;C:1410;T:66835;total:68257 | iSNV |
| F10 | F10-19 | 4943 | NS3 | 0.102 | A:47942;G:5448;C:2;T:1;total:53393 | iSNV |
| F10 | F10-19 | 6533 | NS4A | 0.0918 | A:0;G:1;C:3424;T:33864;total:37289 | iSNV |
| F10 | F10-19 | 7495 | NS4B | 0.1394 | A:2;G:1;C:7615;T:46996;total:54614 | iSNV |
| F10 | F10-19 | 7656 | NS4B | 0.0966 | A:6427;G:60067;C:30;T:3;total:66527 | iSNV |
| F10 | F10-19 | 7657 | NS4B | 0.3322 | A:44078;G:22002;C:144;T:2;total:66226 | iSNV |
| F10 | F10-19 | 8659 | NS5 | 0.0263 | A:65223;G:1768;C:8;T:2;total:67001 | iSNV |
| F10 | F10-19 | 9360 | NS5 | 0.1009 | A:8040;G:71608;C:2;T:2;total:79652 | iSNV |
| F10 | F10-19 | 9688 | NS5 | 0.1588 | A:1;G:0;C:51887;T:9800;total:61688 | iSNV |
| F10 | F10-19 | 9932 | NS5 | 0.0484 | A:2763;G:2;C:14;T:54243;total:57022 | iSNV |
| F10 | F10-19 | 10069 | NS5 | 0.0513 | A:0;G:1;C:66229;T:3584;total:69814 | iSNV |
| F10 | F10-19 | 10071 | NS5 | 0.0405 | A:2852;G:0;C:10;T:67399;total:70261 | iSNV |
| F10 | F10-19 | 10217 | NS5 | 0.021 | A:0;G:0;C:79445;T:1707;total:81152 | iSNV |
| F10 | F10-2 | 1911 | E | 0.7553 | A:6066;G:18715;C:0;T:2;total:24783 | iSNV |
| F10 | F10-2 | 5311 | NS3 | 0.0774 | A:1;G:8;C:3095;T:36853;total:39957 | iSNV |
| F10 | F10-2 | 7495 | NS4B | 0.0277 | A:4;G:3;C:819;T:28679;total:29505 | iSNV |
| F10 | F10-2 | 7657 | NS4B | 0.0594 | A:32750;G:2072;C:19;T:3;total:34844 | iSNV |
| F10 | F10-2 | 10069 | NS5 | 0.7919 | A:1;G:0;C:7434;T:28281;total:35716 | iSNV |
| F10 | F10-20 | 1046 | E | 0.1152 | A:0;G:96319;C:12551;T:13;total:108883 | iSNV |
| F10 | F10-20 | 1343 | E | 0.0215 | A:2;G:1;C:109652;T:2412;total:112067 | iSNV |

| | | | | | | |
|---|---|---|---|---|---|---|
| F10 | F10-20 | 1803 | E | 0.0432 | A:119538;G:5409;C:4;T:4;total:124955 | iSNV |
| F10 | F10-20 | 1911 | E | 0.1571 | A:63477;G:11849;C:72;T:0;total:75398 | iSNV |
| F10 | F10-20 | 5311 | NS3 | 0.0242 | A:7;G:75;C:2483;T:99970;total:102535 | iSNV |
| F10 | F10-20 | 5358 | NS3 | 0.0404 | A:47;G:110055;C:4644;T:3;total:114749 | iSNV |
| F10 | F10-20 | 5835 | NS3 | 0.032 | A:3295;G:99476;C:2;T:4;total:102777 | iSNV |
| F10 | F10-20 | 6533 | NS4A | 0.0698 | A:4;G:3;C:4873;T:64847;total:69727 | iSNV |
| F10 | F10-20 | 7495 | NS4B | 0.272 | A:5;G:4;C:22377;T:59853;total:82239 | iSNV |
| F10 | F10-20 | 7656 | NS4B | 0.0222 | A:2286;G:100615;C:61;T:3;total:102965 | iSNV |
| F10 | F10-20 | 7657 | NS4B | 0.3002 | A:71562;G:30792;C:189;T:2;total:102545 | iSNV |
| F10 | F10-20 | 9688 | NS5 | 0.2159 | A:4;G:2;C:78779;T:21695;total:100480 | iSNV |
| F10 | F10-20 | 9932 | NS5 | 0.1393 | A:13708;G:8;C:15;T:84662;total:98393 | iSNV |
| F10 | F10-20 | 10069 | NS5 | 0.0335 | A:1;G:1;C:112394;T:3907;total:116303 | iSNV |
| F10 | F10-20 | 10071 | NS5 | 0.0434 | A:5077;G:0;C:14;T:111792;total:116883 | iSNV |
| F10 | F10-20 | 10115 | NS5 | 0.1128 | A:0;G:3;C:117873;T:15000;total:132876 | iSNV |
| F10 | F10-22 | 584 | M | 0.0206 | A:2;G:0;C:78670;T:1656;total:80328 | iSNV |
| F10 | F10-22 | 1384 | E | 0.2622 | A:57656;G:2;C:38;T:20506;total:78202 | iSNV |
| F10 | F10-22 | 1786 | E | 0.0253 | A:3;G:4;C:2463;T:94539;total:97009 | iSNV |
| F10 | F10-22 | 1812 | E | 0.0343 | A:97268;G:104;C:3460;T:4;total:100836 | iSNV |
| F10 | F10-22 | 1911 | E | 0.0896 | A:56430;G:5563;C:28;T:1;total:62022 | iSNV |
| F10 | F10-22 | 1957 | E | 0.0238 | A:0;G:0;C:65770;T:1604;total:67374 | iSNV |
| F10 | F10-22 | 5311 | NS3 | 0.1261 | A:0;G:56;C:10046;T:69527;total:79629 | iSNV |
| F10 | F10-22 | 5626 | NS3 | 0.0459 | A:0;G:2;C:3432;T:71188;total:74622 | iSNV |
| F10 | F10-22 | 6533 | NS4A | 0.0784 | A:1;G:6;C:3469;T:40722;total:44198 | iSNV |
| F10 | F10-22 | 7495 | NS4B | 0.0814 | A:2;G:8;C:5556;T:62678;total:68244 | iSNV |
| F10 | F10-22 | 7656 | NS4B | 0.0837 | A:6443;G:70497;C:21;T:4;total:76965 | iSNV |
| F10 | F10-22 | 7657 | NS4B | 0.1427 | A:65638;G:10945;C:67;T:6;total:76656 | iSNV |
| F10 | F10-22 | 9225 | NS5 | 0.2579 | A:20632;G:0;C:59328;T:12;total:79972 | iSNV |
| F10 | F10-22 | 9688 | NS5 | 0.0949 | A:2;G:2;C:65596;T:6885;total:72485 | iSNV |
| F10 | F10-22 | 9909 | NS5 | 0.0421 | A:65706;G:2892;C:0;T:2;total:68600 | iSNV |
| F10 | F10-22 | 9932 | NS5 | 0.0572 | A:3971;G:7;C:5;T:65323;total:69306 | iSNV |
| F10 | F10-22 | 9937 | NS5 | 0.0251 | A:69208;G:1785;C:1;T:4;total:70998 | iSNV |
| F10 | F10-22 | 10069 | NS5 | 0.0262 | A:2;G:0;C:83004;T:2240;total:85246 | iSNV |
| F10 | F10-22 | 10401 | 3'-UTR | 0.0259 | A:1891;G:7;C:12;T:70844;total:72754 | iSNV |
| F10 | F10-23 | 497 | M | 0.0261 | A:18;G:98058;C:2631;T:13;total:100720 | iSNV |
| F10 | F10-23 | 1116 | E | 0.021 | A:78019;G:1676;C:1;T:3;total:79699 | iSNV |
| F10 | F10-23 | 1735 | E | 0.0443 | A:5;G:4;C:3678;T:79190;total:82877 | iSNV |
| F10 | F10-23 | 1786 | E | 0.0224 | A:5;G:7;C:1968;T:85640;total:87620 | iSNV |
| F10 | F10-23 | 1803 | E | 0.0258 | A:87215;G:2319;C:3;T:2;total:89539 | iSNV |
| F10 | F10-23 | 1911 | E | 0.2548 | A:38893;G:13303;C:10;T:0;total:52206 | iSNV |
| F10 | F10-23 | 3001 | NS1 | 0.0224 | A:1723;G:75085;C:2;T:2;total:76812 | iSNV |
| F10 | F10-23 | 3368 | NS1 | 0.024 | A:1401;G:56874;C:0;T:9;total:58284 | iSNV |
| F10 | F10-23 | 4718 | NS3 | 0.042 | A:3;G:0;C:75775;T:3323;total:79101 | iSNV |
| F10 | F10-23 | 5311 | NS3 | 0.0206 | A:2;G:35;C:1468;T:69595;total:71100 | iSNV |
| F10 | F10-23 | 5358 | NS3 | 0.0261 | A:5;G:79621;C:2138;T:1;total:81765 | iSNV |
| F10 | F10-23 | 5835 | NS3 | 0.0247 | A:1741;G:68469;C:1;T:5;total:70216 | iSNV |
| F10 | F10-23 | 6533 | NS4A | 0.0931 | A:2;G:5;C:4273;T:41602;total:45882 | iSNV |
| F10 | F10-23 | 7193 | NS4A | 0.0741 | A:0;G:6;C:3822;T:47731;total:51559 | iSNV |
| F10 | F10-23 | 7495 | NS4B | 0.1201 | A:0;G:3;C:6908;T:50566;total:57477 | iSNV |
| F10 | F10-23 | 7543 | NS4B | 0.0313 | A:68154;G:2208;C:1;T:1;total:70364 | iSNV |
| F10 | F10-23 | 7547 | NS4B | 0.0735 | A:56;G:5200;C:26;T:65453;total:70735 | iSNV |
| F10 | F10-23 | 7656 | NS4B | 0.0712 | A:5243;G:68281;C:21;T:1;total:73546 | iSNV |
| F10 | F10-23 | 7657 | NS4B | 0.2824 | A:52548;G:20700;C:47;T:1;total:73296 | iSNV |
| F10 | F10-23 | 8832 | NS5 | 0.0482 | A:4;G:2;C:3819;T:75294;total:79119 | iSNV |
| F10 | F10-23 | 9688 | NS5 | 0.0939 | A:1;G:1;C:58551;T:6072;total:64625 | iSNV |
| F10 | F10-23 | 9932 | NS5 | 0.0647 | A:4145;G:8;C:11;T:59808;total:63972 | iSNV |
| F10 | F10-23 | 10069 | NS5 | 0.0382 | A:2;G:0;C:73883;T:2938;total:76823 | iSNV |
| F10 | F10-23 | 10071 | NS5 | 0.0283 | A:2190;G:0;C:8;T:75031;total:77229 | iSNV |
| F10 | F10-23 | 10092 | NS5 | 0.1074 | A:8480;G:70431;C:0;T:4;total:78915 | iSNV |
| F10 | F10-25 | 340 | C | 0.4443 | A:8;G:3;C:63207;T:50548;total:113766 | iSNV |
| F10 | F10-25 | 656 | M | 0.0323 | A:13;G:5;C:4009;T:119879;total:123906 | iSNV |
| F10 | F10-25 | 1501 | E | 0.0294 | A:0;G:0;C:78865;T:2389;total:81254 | iSNV |
| F10 | F10-25 | 1911 | E | 0.6414 | A:29192;G:52200;C:8;T:5;total:81405 | iSNV |
| F10 | F10-25 | 2141 | E | 0.0271 | A:97172;G:2717;C:0;T:7;total:99896 | iSNV |
| F10 | F10-25 | 2275 | E | 0.0246 | A:2;G:2;C:1891;T:74763;total:76658 | iSNV |
| F10 | F10-25 | 2873 | NS1 | 0.0362 | A:166189;G:6;C:3;T:6260;total:172458 | iSNV |
| F10 | F10-25 | 2882 | NS1 | 0.067 | A:151995;G:10931;C:35;T:4;total:162965 | iSNV |
| F10 | F10-25 | 5311 | NS3 | 0.0526 | A:3;G:63;C:5864;T:105396;total:111326 | iSNV |
| F10 | F10-25 | 5835 | NS3 | 0.0387 | A:3498;G:86718;C:4;T:4;total:90224 | iSNV |
| F10 | F10-25 | 6533 | NS4A | 0.0311 | A:5;G:3;C:2023;T:62840;total:64871 | iSNV |
| F10 | F10-25 | 6861 | NS4A | 0.0455 | A:1;G:0;C:85351;T:4078;total:89430 | iSNV |
| F10 | F10-25 | 7495 | NS4B | 0.0588 | A:4;G:6;C:5429;T:86858;total:92297 | iSNV |
| F10 | F10-25 | 7575 | NS4B | 0.026 | A:87178;G:2334;C:4;T:5;total:89521 | iSNV |
| F10 | F10-25 | 7656 | NS4B | 0.0861 | A:8593;G:91068;C:23;T:5;total:99689 | iSNV |
| F10 | F10-25 | 7657 | NS4B | 0.1667 | A:82713;G:16565;C:35;T:4;total:99317 | iSNV |
| F10 | F10-25 | 9221 | NS5 | 0.0276 | A:105911;G:9;C:40;T:3008;total:108968 | iSNV |
| F10 | F10-25 | 9688 | NS5 | 0.0455 | A:2;G:0;C:95446;T:4550;total:99998 | iSNV |
| F10 | F10-25 | 10069 | NS5 | 0.6077 | A:2;G:0;C:45633;T:70672;total:116307 | iSNV |
| F10 | F10-26 | 1563 | E | 0.022 | A:5;G:0;C:83593;T:1888;total:85486 | iSNV |
| F10 | F10-26 | 1911 | E | 0.6142 | A:28136;G:44779;C:6;T:4;total:72925 | iSNV |
| F10 | F10-26 | 6533 | NS4A | 0.0545 | A:3;G:0;C:2808;T:48702;total:51513 | iSNV |
| F10 | F10-26 | 7179 | NS4A | 0.1417 | A:10274;G:62205;C:1;T:5;total:72485 | iSNV |
| F10 | F10-26 | 7495 | NS4B | 0.0475 | A:3;G:9;C:4243;T:85025;total:89280 | iSNV |

| | | | | | | |
|---|---|---|---|---|---|---|
| F10 | F10-26 | 7656 | NS4B | 0.0506 | A:4835;G:90324;C:213;T:7;total:95379 | iSNV |
| F10 | F10-26 | 7657 | NS4B | 0.1387 | A:81819;G:13187;C:40;T:2;total:95048 | iSNV |
| F10 | F10-26 | 9688 | NS5 | 0.0617 | A:3;G:1;C:90552;T:5959;total:96515 | iSNV |
| F10 | F10-26 | 10069 | NS5 | 0.619 | A:3;G:2;C:39246;T:63737;total:102988 | iSNV |
| F10 | F10-28 | 836 | M | 0.0357 | A:5;G:0;C:2634;T:71085;total:73724 | iSNV |
| F10 | F10-28 | 902 | M | 0.0489 | A:4;G:2;C:65297;T:3360;total:68663 | iSNV |
| F10 | F10-28 | 1911 | E | 0.3525 | A:40808;G:22230;C:10;T:2;total:63050 | iSNV |
| F10 | F10-28 | 4394 | NS2B | 0.0324 | A:4;G:1;C:80490;T:2700;total:83195 | iSNV |
| F10 | F10-28 | 5311 | NS3 | 0.0636 | A:3;G:34;C:5208;T:76629;total:81874 | iSNV |
| F10 | F10-28 | 6533 | NS4A | 0.0562 | A:0;G:5;C:3602;T:60422;total:64029 | iSNV |
| F10 | F10-28 | 7271 | NS4B | 0.0284 | A:3;G:2;C:1582;T:53936;total:55523 | iSNV |
| F10 | F10-28 | 7494 | NS4B | 0.0551 | A:57;G:61371;C:4;T:3583;total:65015 | iSNV |
| F10 | F10-28 | 7495 | NS4B | 0.3072 | A:3;G:4;C:20349;T:45869;total:66225 | iSNV |
| F10 | F10-28 | 7656 | NS4B | 0.1167 | A:9976;G:75373;C:60;T:2;total:85411 | iSNV |
| F10 | F10-28 | 7657 | NS4B | 0.2215 | A:66281;G:18874;C:28;T:2;total:85185 | iSNV |
| F10 | F10-28 | 8987 | NS5 | 0.0457 | A:4039;G:1;C:30;T:84257;total:88327 | iSNV |
| F10 | F10-28 | 9688 | NS5 | 0.0663 | A:4;G:0;C:71287;T:5065;total:76356 | iSNV |
| F10 | F10-28 | 9932 | NS5 | 0.0416 | A:3096;G:2;C:7;T:71141;total:74246 | iSNV |
| F10 | F10-28 | 10069 | NS5 | 0.0804 | A:1;G:0;C:80388;T:7030;total:87419 | iSNV |
| F10 | F10-30 | 785 | M | 0.0383 | A:10240;G:409;C:2;T:3;total:10654 | iSNV |
| F10 | F10-30 | 1461 | E | 0.0407 | A:0;G:2;C:411;T:9676;total:10089 | iSNV |
| F10 | F10-30 | 1911 | E | 0.1631 | A:7463;G:1456;C:5;T:0;total:8924 | iSNV |
| F10 | F10-30 | 2129 | E | 0.3672 | A:6522;G:3786;C:0;T:1;total:10309 | iSNV |
| F10 | F10-30 | 2876 | NS1 | 0.02 | A:0;G:4;C:273;T:13334;total:13611 | iSNV |
| F10 | F10-30 | 3230 | NS1 | 0.3657 | A:8273;G:4772;C:1;T:0;total:13046 | iSNV |
| F10 | F10-30 | 4192 | NS2A | 0.0323 | A:0;G:0;C:190;T:5684;total:5874 | iSNV |
| F10 | F10-30 | 4664 | NS3 | 0.0226 | A:0;G:3;C:280;T:12091;total:12374 | iSNV |
| F10 | F10-30 | 5311 | NS3 | 0.0234 | A:0;G:13;C:229;T:9523;total:9765 | iSNV |
| F10 | F10-30 | 6533 | NS4A | 0.4159 | A:1;G:1;C:3556;T:4992;total:8550 | iSNV |
| F10 | F10-30 | 7495 | NS4B | 0.0878 | A:0;G:1;C:648;T:6729;total:7378 | iSNV |
| F10 | F10-30 | 7543 | NS4B | 0.0456 | A:9074;G:434;C:1;T:1;total:9510 | iSNV |
| F10 | F10-30 | 7656 | NS4B | 0.0374 | A:416;G:10605;C:81;T:0;total:11102 | iSNV |
| F10 | F10-30 | 7657 | NS4B | 0.5399 | A:5111;G:5996;C:1;T:0;total:11108 | iSNV |
| F10 | F10-30 | 8900 | NS5 | 0.0244 | A:210;G:2;C:8386;T:0;total:8598 | iSNV |
| F10 | F10-30 | 9688 | NS5 | 0.4647 | A:0;G:0;C:5217;T:4529;total:9746 | iSNV |
| F10 | F10-30 | 9932 | NS5 | 0.0605 | A:555;G:6;C:0;T:8607;total:9168 | iSNV |
| F10 | F10-30 | 10069 | NS5 | 0.0423 | A:1;G:0;C:7459;T:330;total:7790 | iSNV |
| F10 | F10-4 | 1786 | E | 0.034 | A:6;G:25;C:2062;T:58466;total:60559 | iSNV |
| F10 | F10-4 | 1911 | E | 0.0689 | A:32272;G:2391;C:3;T:2;total:34668 | iSNV |
| F10 | F10-4 | 1914 | E | 0.071 | A:29699;G:2270;C:0;T:1;total:31970 | iSNV |
| F10 | F10-4 | 2477 | E | 0.0264 | A:1;G:756;C:6;T:27832;total:28595 | iSNV |
| F10 | F10-4 | 3761 | NS2A | 0.0304 | A:4;G:0;C:42902;T:1347;total:44253 | iSNV |
| F10 | F10-4 | 4250 | NS2B | 0.0773 | A:34031;G:2854;C:1;T:3;total:36889 | iSNV |
| F10 | F10-4 | 5311 | NS3 | 0.0278 | A:0;G:77;C:1348;T:47050;total:48475 | iSNV |
| F10 | F10-4 | 6533 | NS4A | 0.1769 | A:22;G:1;C:3142;T:14587;total:17752 | iSNV |
| F10 | F10-4 | 7495 | NS4B | 0.0914 | A:2;G:1;C:3468;T:34447;total:37918 | iSNV |
| F10 | F10-4 | 7656 | NS4B | 0.0913 | A:3705;G:36853;C:11;T:0;total:40569 | iSNV |
| F10 | F10-4 | 7657 | NS4B | 0.2823 | A:28952;G:11419;C:65;T:2;total:40438 | iSNV |
| F10 | F10-4 | 9220 | NS5 | 0.0291 | A:31214;G:0;C:938;T:0;total:32152 | iSNV |
| F10 | F10-4 | 9688 | NS5 | 0.2375 | A:0;G:0;C:35708;T:11125;total:46833 | iSNV |
| F10 | F10-4 | 9932 | NS5 | 0.0471 | A:1536;G:1;C:8;T:31026;total:32571 | iSNV |
| F10 | F10-4 | 10069 | NS5 | 0.066 | A:0;G:0;C:43225;T:3056;total:46281 | iSNV |
| F10 | F10-4 | 10092 | NS5 | 0.0423 | A:1993;G:45057;C:0;T:6;total:47056 | iSNV |
| F10 | F10-5 | 933 | M | 0.0518 | A:1522;G:27840;C:1;T:1;total:29364 | iSNV |
| F10 | F10-5 | 1473 | E | 0.0211 | A:22410;G:484;C:11;T:0;total:22905 | iSNV |
| F10 | F10-5 | 1911 | E | 0.3588 | A:14860;G:8326;C:13;T:2;total:23201 | iSNV |
| F10 | F10-5 | 4394 | NS2B | 0.0203 | A:1;G:0;C:24153;T:503;total:24657 | iSNV |
| F10 | F10-5 | 4745 | NS3 | 0.0216 | A:2;G:0;C:28244;T:624;total:28870 | iSNV |
| F10 | F10-5 | 5311 | NS3 | 0.1058 | A:5;G:33;C:3464;T:29209;total:32711 | iSNV |
| F10 | F10-5 | 5543 | NS3 | 0.029 | A:3;G:0;C:36153;T:1083;total:37239 | iSNV |
| F10 | F10-5 | 6523 | NS4A | 0.0413 | A:0;G:1;C:13000;T:561;total:13562 | iSNV |
| F10 | F10-5 | 6533 | NS4A | 0.0633 | A:1;G:4;C:854;T:12625;total:13484 | iSNV |
| F10 | F10-5 | 7322 | NS4B | 0.0202 | A:0;G:0;C:20510;T:423;total:20933 | iSNV |
| F10 | F10-5 | 7495 | NS4B | 0.0693 | A:1;G:1;C:1808;T:24264;total:26074 | iSNV |
| F10 | F10-5 | 7656 | NS4B | 0.0802 | A:2340;G:24774;C:2057;T:1;total:29172 | iSNV |
| F10 | F10-5 | 7657 | NS4B | 0.1926 | A:23460;G:5599;C:11;T:0;total:29070 | iSNV |
| F10 | F10-5 | 8093 | NS5 | 0.0291 | A:0;G:0;C:32381;T:973;total:33354 | iSNV |
| F10 | F10-5 | 9688 | NS5 | 0.2819 | A:3;G:0;C:23054;T:9052;total:32109 | iSNV |
| F10 | F10-5 | 9932 | NS5 | 0.0307 | A:737;G:2;C:5;T:23248;total:23992 | iSNV |
| F10 | F10-5 | 10092 | NS5 | 0.0253 | A:860;G:33044;C:0;T:0;total:33904 | iSNV |
| F10 | F10-5 | 10097 | NS5 | 0.0206 | A:2;G:34116;C:2;T:720;total:34840 | iSNV |
| F10 | F10-5 | 10419 | 3'-UTR | 0.0288 | A:2;G:0;C:21002;T:624;total:21628 | iSNV |
| F10 | F10-7 | 1911 | E | 0.1055 | A:19161;G:2262;C:4;T:2;total:21429 | iSNV |
| F10 | F10-7 | 3139 | NS1 | 0.707 | A:9542;G:23017;C:0;T:1;total:32560 | iSNV |
| F10 | F10-7 | 7495 | NS4B | 0.7334 | A:0;G:0;C:15304;T:5566;total:20870 | iSNV |
| F10 | F10-7 | 7543 | NS4B | 0.025 | A:25002;G:643;C:0;T:0;total:25645 | iSNV |
| F10 | F10-7 | 7656 | NS4B | 0.0232 | A:603;G:25346;C:1;T:4;total:25954 | iSNV |
| F10 | F10-7 | 7657 | NS4B | 0.0383 | A:24895;G:992;C:2;T:0;total:25889 | iSNV |
| F10 | F10-7 | 9688 | NS5 | 0.7811 | A:0;G:0;C:6399;T:22830;total:29229 | iSNV |
| F10 | F10-7 | 9932 | NS5 | 0.0274 | A:582;G:0;C:3;T:20639;total:21224 | iSNV |
| F10 | F10-7 | 10069 | NS5 | 0.0615 | A:0;G:0;C:27351;T:1794;total:29145 | iSNV |
| F10 | F10-8 | 401 | C | 0.0333 | A:38669;G:1336;C:9;T:0;total:40014 | iSNV |

| | | | | | | |
|---|---|---|---|---|---|---|
| F10 | F10-8 | 798 | M | 0.1421 | A:16844;G:1;C:2791;T:0;total:19636 | iSNV |
| F10 | F10-8 | 1348 | E | 0.0351 | A:33692;G:4;C:1226;T:2;total:34924 | iSNV |
| F10 | F10-8 | 1786 | E | 0.0231 | A:1;G:8;C:967;T:40735;total:41711 | iSNV |
| F10 | F10-8 | 1911 | E | 0.0775 | A:22233;G:1873;C:41;T:0;total:24147 | iSNV |
| F10 | F10-8 | 3368 | NS1 | 0.0352 | A:822;G:22511;C:0;T:0;total:23333 | iSNV |
| F10 | F10-8 | 3538 | NS1 | 0.0315 | A:20320;G:6;C:663;T:0;total:20989 | iSNV |
| F10 | F10-8 | 4401 | NS2B | 0.0362 | A:0;G:21407;C:805;T:3;total:22215 | iSNV |
| F10 | F10-8 | 5311 | NS3 | 0.0341 | A:1;G:15;C:1127;T:31868;total:33011 | iSNV |
| F10 | F10-8 | 5528 | NS3 | 0.0345 | A:32423;G:1162;C:0;T:1;total:33586 | iSNV |
| F10 | F10-8 | 5835 | NS3 | 0.0901 | A:2180;G:21997;C:0;T:0;total:24177 | iSNV |
| F10 | F10-8 | 5975 | NS3 | 0.0273 | A:2;G:9;C:520;T:18503;total:19034 | iSNV |
| F10 | F10-8 | 6533 | NS4A | 0.295 | A:2;G:0;C:4309;T:10292;total:14603 | iSNV |
| F10 | F10-8 | 7495 | NS4B | 0.0905 | A:1;G:0;C:2343;T:23534;total:25878 | iSNV |
| F10 | F10-8 | 7656 | NS4B | 0.0827 | A:2276;G:25232;C:4;T:0;total:27512 | iSNV |
| F10 | F10-8 | 7657 | NS4B | 0.5055 | A:13570;G:13866;C:0;T:1;total:27437 | iSNV |
| F10 | F10-8 | 9688 | NS5 | 0.2806 | A:0;G:0;C:22545;T:8796;total:31341 | iSNV |
| F10 | F10-8 | 9932 | NS5 | 0.0548 | A:1310;G:3;C:24;T:22537;total:23874 | iSNV |
| F10 | F10-8 | 10069 | NS5 | 0.0322 | A:1;G:0;C:31343;T:1043;total:32387 | iSNV |
| F10 | F10-8 | 10419 | 3'-UTR | 0.1755 | A:0;G:1;C:17375;T:3699;total:21075 | iSNV |
| F15 | F15-1 | 1116 | E | 0.0316 | A:21830;G:713;C:2;T:3;total:22548 | iSNV |
| F15 | F15-1 | 1417 | E | 0.3172 | A:2;G:2;C:16377;T:7612;total:23993 | iSNV |
| F15 | F15-1 | 1474 | E | 0.0344 | A:20985;G:4;C:748;T:6;total:21743 | iSNV |
| F15 | F15-1 | 1803 | E | 0.2081 | A:21162;G:5564;C:0;T:1;total:26727 | iSNV |
| F15 | F15-1 | 1911 | E | 0.3426 | A:13288;G:6928;C:1;T:2;total:20219 | iSNV |
| F15 | F15-1 | 2030 | E | 0.0412 | A:1;G:5;C:773;T:17949;total:18728 | iSNV |
| F15 | F15-1 | 2567 | NS1 | 0.0394 | A:0;G:6;C:697;T:16980;total:17683 | iSNV |
| F15 | F15-1 | 3528 | NS1 | 0.0294 | A:0;G:0;C:18983;T:577;total:19560 | iSNV |
| F15 | F15-1 | 3993 | NS2A | 0.1803 | A:4384;G:19915;C:6;T:1;total:24306 | iSNV |
| F15 | F15-1 | 5311 | NS3 | 0.3372 | A:0;G:32;C:7741;T:15182;total:22955 | iSNV |
| F15 | F15-1 | 5358 | NS3 | 0.2053 | A:5;G:18769;C:4851;T:2;total:23627 | iSNV |
| F15 | F15-1 | 6707 | NS4A | 0.0264 | A:491;G:8;C:2;T:18054;total:18555 | iSNV |
| F15 | F15-1 | 7495 | NS4B | 0.2713 | A:0;G:4;C:4875;T:13086;total:17965 | iSNV |
| F15 | F15-1 | 7526 | NS4B | 0.0964 | A:2023;G:5;C:33;T:18918;total:20979 | iSNV |
| F15 | F15-1 | 7626 | NS4B | 0.3506 | A:7;G:9188;C:6;T:17001;total:26202 | iSNV |
| F15 | F15-1 | 7657 | NS4B | 0.0242 | A:24425;G:606;C:1;T:0;total:25032 | iSNV |
| F15 | F15-1 | 9932 | NS5 | 0.2946 | A:6439;G:5;C:14;T:15397;total:21855 | iSNV |
| F15 | F15-1 | 10071 | NS5 | 0.2102 | A:3999;G:3;C:2;T:15015;total:19019 | iSNV |
| F15 | F15-1 | 10086 | NS5 | 0.045 | A:891;G:8;C:2;T:18868;total:19769 | iSNV |
| F15 | F15-1 | 10092 | NS5 | 0.0615 | A:1246;G:19003;C:0;T:2;total:20251 | iSNV |
| F15 | F15-10 | 1116 | E | 0.1246 | A:114564;G:16311;C:2;T:5;total:130882 | iSNV |
| F15 | F15-10 | 1296 | E | 0.0216 | A:5;G:2;C:2792;T:125920;total:128719 | iSNV |
| F15 | F15-10 | 1347 | E | 0.0207 | A:123099;G:2603;C:6;T:1;total:125709 | iSNV |
| F15 | F15-10 | 1406 | E | 0.0515 | A:6483;G:119180;C:1;T:11;total:125675 | iSNV |
| F15 | F15-10 | 1474 | E | 0.0261 | A:94173;G:10;C:10;T:2527;total:96720 | iSNV |
| F15 | F15-10 | 1911 | E | 0.4391 | A:50149;G:39292;C:38;T:4;total:89483 | iSNV |
| F15 | F15-10 | 2226 | E | 0.0239 | A:5;G:0;C:101590;T:2496;total:104091 | iSNV |
| F15 | F15-10 | 3399 | NS1 | 0.02 | A:104288;G:2136;C:2;T:1;total:106427 | iSNV |
| F15 | F15-10 | 3949 | NS2A | 0.0401 | A:7;G:13;C:5550;T:132512;total:138082 | iSNV |
| F15 | F15-10 | 5311 | NS3 | 0.0427 | A:2;G:169;C:4915;T:109972;total:115058 | iSNV |
| F15 | F15-10 | 6368 | NS3 | 0.0453 | A:8;G:3803;C:27;T:80008;total:83846 | iSNV |
| F15 | F15-10 | 6533 | NS4A | 0.0528 | A:2;G:6;C:4179;T:74855;total:79042 | iSNV |
| F15 | F15-10 | 7495 | NS4B | 0.1467 | A:10;G:6;C:14080;T:81864;total:95960 | iSNV |
| F15 | F15-10 | 7657 | NS4B | 0.4508 | A:57557;G:47270;C:9;T:2;total:104838 | iSNV |
| F15 | F15-10 | 8640 | NS5 | 0.0254 | A:6;G:0;C:120918;T:3152;total:124076 | iSNV |
| F15 | F15-10 | 9688 | NS5 | 0.0957 | A:3;G:1;C:101481;T:10740;total:112225 | iSNV |
| F15 | F15-10 | 9932 | NS5 | 0.0367 | A:3884;G:7;C:48;T:101788;total:105727 | iSNV |
| F15 | F15-10 | 10069 | NS5 | 0.2891 | A:3;G:3;C:88434;T:35974;total:124414 | iSNV |
| F15 | F15-10 | 10308 | NS5 | 0.0946 | A:13774;G:131801;C:3;T:11;total:145589 | iSNV |
| F15 | F15-11 | 221 | C | 0.0319 | A:1154;G:34971;C:2;T:1;total:36128 | iSNV |
| F15 | F15-11 | 1417 | E | 0.0449 | A:2;G:0;C:71501;T:3362;total:74865 | iSNV |
| F15 | F15-11 | 1421 | E | 0.0552 | A:7;G:3;C:4166;T:71210;total:75386 | iSNV |
| F15 | F15-11 | 1803 | E | 0.0643 | A:83028;G:5708;C:3;T:8;total:88747 | iSNV |
| F15 | F15-11 | 1911 | E | 0.3311 | A:36391;G:18020;C:0;T:4;total:54415 | iSNV |
| F15 | F15-11 | 1913 | E | 0.2182 | A:39772;G:3;C:11120;T:52;total:50947 | iSNV |
| F15 | F15-11 | 3329 | NS1 | 0.0206 | A:60200;G:1268;C:0;T:2;total:61470 | iSNV |
| F15 | F15-11 | 5311 | NS3 | 0.109 | A:3;G:198;C:7860;T:64035;total:72096 | iSNV |
| F15 | F15-11 | 5353 | NS3 | 0.0294 | A:79703;G:2416;C:2;T:1;total:82122 | iSNV |
| F15 | F15-11 | 5358 | NS3 | 0.0536 | A:11;G:78655;C:4457;T:4;total:83127 | iSNV |
| F15 | F15-11 | 6523 | NS4A | 0.3058 | A:1;G:2;C:32498;T:14319;total:46820 | iSNV |
| F15 | F15-11 | 6533 | NS4A | 0.0955 | A:4;G:1;C:4274;T:40464;total:44743 | iSNV |
| F15 | F15-11 | 7495 | NS4B | 0.1083 | A:3;G:1;C:6442;T:52999;total:59445 | iSNV |
| F15 | F15-11 | 7629 | NS4B | 0.0209 | A:72855;G:0;C:1559;T:1;total:74415 | iSNV |
| F15 | F15-11 | 7656 | NS4B | 0.077 | A:5203;G:62282;C:6;T:5;total:67496 | iSNV |
| F15 | F15-11 | 7657 | NS4B | 0.1555 | A:56830;G:10471;C:2;T:2;total:67305 | iSNV |
| F15 | F15-11 | 8165 | NS5 | 0.0264 | A:4;G:0;C:73467;T:1994;total:75465 | iSNV |
| F15 | F15-11 | 8900 | NS5 | 0.0511 | A:3175;G:17;C:58871;T:15;total:62078 | iSNV |
| F15 | F15-11 | 9607 | NS5 | 0.3292 | A:35421;G:1;C:17393;T:9;total:52824 | iSNV |
| F15 | F15-11 | 9688 | NS5 | 0.1109 | A:3;G:0;C:60439;T:7544;total:67986 | iSNV |
| F15 | F15-11 | 9932 | NS5 | 0.043 | A:2817;G:3;C:17;T:62646;total:65483 | iSNV |
| F15 | F15-11 | 10071 | NS5 | 0.3793 | A:29449;G:2;C:8;T:48175;total:77634 | iSNV |
| F15 | F15-13 | 344 | C | 0.1957 | A:7;G:6671;C:5;T:27403;total:34086 | iSNV |
| F15 | F15-13 | 542 | M | 0.0588 | A:7;G:2301;C:13;T:36809;total:39130 | iSNV |

| | | | | | | |
|---|---|---|---|---|---|---|
| F15 | F15-13 | 1116 | E | 0.0396 | A:42638;G:1759;C:0;T:1;total:44398 | iSNV |
| F15 | F15-13 | 1435 | E | 0.0832 | A:2952;G:32489;C:1;T:3;total:35445 | iSNV |
| F15 | F15-13 | 1468 | E | 0.0234 | A:701;G:0;C:29137;T:24;total:29862 | iSNV |
| F15 | F15-13 | 1803 | E | 0.6333 | A:18038;G:31138;C:1;T:1;total:49178 | iSNV |
| F15 | F15-13 | 1911 | E | 0.3198 | A:21337;G:10050;C:31;T:0;total:31418 | iSNV |
| F15 | F15-13 | 2160 | E | 0.0248 | A:0;G:0;C:35039;T:892;total:35931 | iSNV |
| F15 | F15-13 | 3926 | NS2A | 0.0557 | A:8;G:0;C:45706;T:2700;total:48414 | iSNV |
| F15 | F15-13 | 4943 | NS3 | 0.1869 | A:21005;G:4831;C:0;T:1;total:25837 | iSNV |
| F15 | F15-13 | 5358 | NS3 | 0.6514 | A:1;G:16894;C:31550;T:15;total:48460 | iSNV |
| F15 | F15-13 | 5993 | NS3 | 0.0592 | A:18289;G:1152;C:0;T:0;total:19441 | iSNV |
| F15 | F15-13 | 7495 | NS4B | 0.2353 | A:2;G:2;C:8463;T:27490;total:35957 | iSNV |
| F15 | F15-13 | 7546 | NS4B | 0.6669 | A:12598;G:25201;C:10;T:2;total:37811 | iSNV |
| F15 | F15-13 | 7657 | NS4B | 0.1138 | A:32739;G:4209;C:22;T:1;total:36971 | iSNV |
| F15 | F15-13 | 8927 | NS5 | 0.0651 | A:31036;G:2163;C:2;T:3;total:33204 | iSNV |
| F15 | F15-13 | 9353 | NS5 | 0.0397 | A:32780;G:1358;C:15;T:3;total:34156 | iSNV |
| F15 | F15-13 | 10071 | NS5 | 0.6271 | A:25282;G:0;C:4;T:15042;total:40328 | iSNV |
| F15 | F15-13 | 10092 | NS5 | 0.2342 | A:9850;G:32193;C:0;T:2;total:42045 | iSNV |
| F15 | F15-14 | 541 | M | 0.0336 | A:8;G:14;C:1925;T:55245;total:57192 | iSNV |
| F15 | F15-14 | 1116 | E | 0.0267 | A:61404;G:1690;C:0;T:3;total:63097 | iSNV |
| F15 | F15-14 | 1911 | E | 0.6187 | A:15738;G:25523;C:4;T:0;total:41265 | iSNV |
| F15 | F15-14 | 2541 | NS1 | 0.0269 | A:1;G:1;C:43915;T:1218;total:45135 | iSNV |
| F15 | F15-14 | 5311 | NS3 | 0.0525 | A:3;G:34;C:3002;T:54049;total:57088 | iSNV |
| F15 | F15-14 | 5835 | NS3 | 0.2014 | A:8708;G:34508;C:1;T:4;total:43221 | iSNV |
| F15 | F15-14 | 6047 | NS3 | 0.0305 | A:0;G:958;C:11;T:30391;total:31360 | iSNV |
| F15 | F15-14 | 7495 | NS4B | 0.0381 | A:5;G:5;C:1889;T:47626;total:49525 | iSNV |
| F15 | F15-14 | 7656 | NS4B | 0.0252 | A:1291;G:49534;C:219;T:0;total:51044 | iSNV |
| F15 | F15-14 | 7657 | NS4B | 0.5015 | A:25338;G:25423;C:65;T:2;total:50828 | iSNV |
| F15 | F15-14 | 9688 | NS5 | 0.0232 | A:0;G:0;C:51721;T:1231;total:52952 | iSNV |
| F15 | F15-14 | 10069 | NS5 | 0.4646 | A:1;G:1;C:31188;T:27071;total:58261 | iSNV |
| F15 | F15-16 | 1417 | E | 0.0268 | A:1;G:0;C:22420;T:618;total:23039 | iSNV |
| F15 | F15-16 | 1911 | E | 0.6832 | A:4575;G:9862;C:1;T:2;total:14440 | iSNV |
| F15 | F15-16 | 2069 | E | 0.0496 | A:827;G:0;C:15827;T:7;total:16661 | iSNV |
| F15 | F15-16 | 2433 | E | 0.0523 | A:789;G:14276;C:7;T:2;total:15074 | iSNV |
| F15 | F15-16 | 3096 | NS1 | 0.0428 | A:21338;G:12;C:0;T:955;total:22305 | iSNV |
| F15 | F15-16 | 3542 | NS1 | 0.0246 | A:0;G:3;C:415;T:16410;total:16828 | iSNV |
| F15 | F15-16 | 3685 | NS1 | 0.1449 | A:14869;G:2522;C:0;T:3;total:17394 | iSNV |
| F15 | F15-16 | 5311 | NS3 | 0.3941 | A:1;G:39;C:7999;T:12255;total:20294 | iSNV |
| F15 | F15-16 | 5312 | NS3 | 0.3245 | A:0;G:3;C:6539;T:13606;total:20148 | iSNV |
| F15 | F15-16 | 5358 | NS3 | 0.0322 | A:26;G:21059;C:702;T:1;total:21788 | iSNV |
| F15 | F15-16 | 5875 | NS3 | 0.0835 | A:1;G:17;C:2173;T:23825;total:26016 | iSNV |
| F15 | F15-16 | 7495 | NS4B | 0.0595 | A:2;G:10;C:866;T:13671;total:14549 | iSNV |
| F15 | F15-16 | 7656 | NS4B | 0.0413 | A:972;G:22523;C:1;T:2;total:23498 | iSNV |
| F15 | F15-16 | 7657 | NS4B | 0.5118 | A:11471;G:12015;C:7;T:2;total:23495 | iSNV |
| F15 | F15-16 | 7751 | NS5 | 0.0577 | A:1;G:3;C:1075;T:17540;total:18619 | iSNV |
| F15 | F15-16 | 9688 | NS5 | 0.0267 | A:0;G:0;C:16173;T:445;total:16618 | iSNV |
| F15 | F15-16 | 10069 | NS5 | 0.3699 | A:0;G:3;C:9634;T:5658;total:15295 | iSNV |
| F15 | F15-16 | 10071 | NS5 | 0.0349 | A:543;G:1;C:1;T:14975;total:15520 | iSNV |
| F15 | F15-16 | 10109 | NS5 | 0.0267 | A:30;G:18004;C:495;T:1;total:18530 | iSNV |
| F15 | F15-16 | 10808 | 3'-UTR | 0.0837 | A:1342;G:14672;C:4;T:2;total:16020 | iSNV |
| F15 | F15-17 | 287 | C | 0.0424 | A:2181;G:49190;C:2;T:29;total:51402 | iSNV |
| F15 | F15-17 | 1116 | E | 0.048 | A:68575;G:3461;C:0;T:0;total:72036 | iSNV |
| F15 | F15-17 | 1298 | E | 0.1169 | A:7962;G:19;C:11;T:60089;total:68081 | iSNV |
| F15 | F15-17 | 1432 | E | 0.0482 | A:56060;G:23;C:19;T:2843;total:58945 | iSNV |
| F15 | F15-17 | 1911 | E | 0.456 | A:26593;G:22298;C:1;T:2;total:48894 | iSNV |
| F15 | F15-17 | 1963 | E | 0.0207 | A:0;G:0;C:53425;T:1134;total:54559 | iSNV |
| F15 | F15-17 | 5216 | NS3 | 0.0504 | A:53577;G:2847;C:1;T:1;total:56426 | iSNV |
| F15 | F15-17 | 5311 | NS3 | 0.0561 | A:2;G:88;C:3358;T:56349;total:59797 | iSNV |
| F15 | F15-17 | 5343 | NS3 | 0.0233 | A:1;G:2;C:1495;T:62609;total:64107 | iSNV |
| F15 | F15-17 | 5664 | NS3 | 0.0331 | A:63863;G:2192;C:0;T:5;total:66060 | iSNV |
| F15 | F15-17 | 6449 | NS3 | 0.0207 | A:0;G:1;C:1131;T:53360;total:54492 | iSNV |
| F15 | F15-17 | 6533 | NS4A | 0.0389 | A:1;G:7;C:1758;T:43351;total:45117 | iSNV |
| F15 | F15-17 | 7488 | NS4B | 0.0544 | A:46803;G:88;C:0;T:2698;total:49589 | iSNV |
| F15 | F15-17 | 7495 | NS4B | 0.126 | A:3;G:2;C:6374;T:44199;total:50578 | iSNV |
| F15 | F15-17 | 7543 | NS4B | 0.0299 | A:54330;G:1678;C:3;T:1;total:56012 | iSNV |
| F15 | F15-17 | 7656 | NS4B | 0.3886 | A:21257;G:33385;C:51;T:3;total:54696 | iSNV |
| F15 | F15-17 | 7657 | NS4B | 0.1479 | A:46331;G:8052;C:24;T:3;total:54410 | iSNV |
| F15 | F15-17 | 8656 | NS5 | 0.1274 | A:0;G:0;C:7786;T:53320;total:61106 | iSNV |
| F15 | F15-17 | 8659 | NS5 | 0.132 | A:51058;G:7772;C:8;T:1;total:58839 | iSNV |
| F15 | F15-17 | 9688 | NS5 | 0.0493 | A:0;G:0;C:55338;T:2875;total:58213 | iSNV |
| F15 | F15-17 | 9932 | NS5 | 0.0732 | A:4519;G:4;C:13;T:57129;total:61665 | iSNV |
| F15 | F15-17 | 10069 | NS5 | 0.0896 | A:2;G:0;C:62934;T:6198;total:69134 | iSNV |
| F15 | F15-19 | 1116 | E | 0.0448 | A:68334;G:3210;C:0;T:3;total:71547 | iSNV |
| F15 | F15-19 | 1296 | E | 0.0272 | A:4;G:1;C:1955;T:69895;total:71855 | iSNV |
| F15 | F15-19 | 1383 | E | 0.0269 | A:66136;G:1834;C:2;T:3;total:67975 | iSNV |
| F15 | F15-19 | 1389 | E | 0.0209 | A:65194;G:96;C:1399;T:3;total:66692 | iSNV |
| F15 | F15-19 | 1638 | E | 0.0474 | A:2728;G:0;C:54760;T:9;total:57497 | iSNV |
| F15 | F15-19 | 1773 | E | 0.0267 | A:7;G:5;C:1970;T:71563;total:73545 | iSNV |
| F15 | F15-19 | 1911 | E | 0.2961 | A:34106;G:14365;C:28;T:0;total:48499 | iSNV |
| F15 | F15-19 | 2490 | NS1 | 0.029 | A:14;G:47135;C:1;T:1412;total:48562 | iSNV |
| F15 | F15-19 | 3149 | NS1 | 0.0237 | A:4;G:4;C:1835;T:75487;total:77330 | iSNV |
| F15 | F15-19 | 4517 | NS2B | 0.0287 | A:2019;G:0;C:68145;T:25;total:70189 | iSNV |
| F15 | F15-19 | 4943 | NS3 | 0.1354 | A:46080;G:7222;C:1;T:1;total:53304 | iSNV |

| | | | | | | |
|---|---|---|---|---|---|---|
| F15 | F15-19 | 5311 | NS3 | 0.0517 | A:5;G:56;C:3066;T:56089;total:59216 | iSNV |
| F15 | F15-19 | 6533 | NS4A | 0.0595 | A:1;G:1;C:2603;T:41119;total:43724 | iSNV |
| F15 | F15-19 | 7495 | NS4B | 0.1603 | A:4;G:3;C:7864;T:41167;total:49038 | iSNV |
| F15 | F15-19 | 7656 | NS4B | 0.0697 | A:4045;G:53905;C:14;T:5;total:57969 | iSNV |
| F15 | F15-19 | 7657 | NS4B | 0.4248 | A:33213;G:24556;C:27;T:3;total:57799 | iSNV |
| F15 | F15-19 | 8659 | NS5 | 0.031 | A:57812;G:1852;C:12;T:1;total:59677 | iSNV |
| F15 | F15-19 | 8900 | NS5 | 0.0332 | A:1860;G:255;C:53788;T:15;total:55918 | iSNV |
| F15 | F15-19 | 9360 | NS5 | 0.1325 | A:9708;G:63522;C:1;T:2;total:73233 | iSNV |
| F15 | F15-19 | 9688 | NS5 | 0.2427 | A:1;G:0;C:45594;T:14618;total:60213 | iSNV |
| F15 | F15-19 | 9692 | NS5 | 0.0301 | A:1856;G:1;C:59623;T:30;total:61510 | iSNV |
| F15 | F15-19 | 9932 | NS5 | 0.0417 | A:2503;G:18;C:17;T:57383;total:59921 | iSNV |
| F15 | F15-19 | 10069 | NS5 | 0.0551 | A:1;G:0;C:64051;T:3740;total:67792 | iSNV |
| F15 | F15-19 | 10071 | NS5 | 0.0595 | A:4065;G:1;C:4;T:64182;total:68252 | iSNV |
| F15 | F15-19 | 10085 | NS5 | 0.0208 | A:1;G:2;C:66884;T:1427;total:68314 | iSNV |
| F15 | F15-19 | 10097 | NS5 | 0.022 | A:7;G:71060;C:2;T:1603;total:72672 | iSNV |
| F15 | F15-2 | 1911 | E | 0.8216 | A:9907;G:45595;C:6;T:1;total:55509 | iSNV |
| F15 | F15-2 | 2961 | NS1 | 0.0475 | A:106227;G:5304;C:4;T:2;total:111537 | iSNV |
| F15 | F15-2 | 3511 | NS1 | 0.0676 | A:10;G:12;C:3404;T:46915;total:50341 | iSNV |
| F15 | F15-2 | 5311 | NS3 | 0.0921 | A:4;G:54;C:7762;T:76403;total:84223 | iSNV |
| F15 | F15-2 | 7448 | NS4B | 0.0568 | A:65234;G:3933;C:5;T:3;total:69175 | iSNV |
| F15 | F15-2 | 7495 | NS4B | 0.0551 | A:8;G:3;C:3871;T:66367;total:70249 | iSNV |
| F15 | F15-2 | 7575 | NS4B | 0.0222 | A:66079;G:1507;C:2;T:3;total:67591 | iSNV |
| F15 | F15-2 | 7656 | NS4B | 0.0862 | A:6364;G:66797;C:635;T:5;total:73801 | iSNV |
| F15 | F15-2 | 7657 | NS4B | 0.1836 | A:60037;G:13512;C:31;T:0;total:73580 | iSNV |
| F15 | F15-2 | 8194 | NS5 | 0.0216 | A:73939;G:1640;C:6;T:51;total:75636 | iSNV |
| F15 | F15-2 | 9053 | NS5 | 0.0281 | A:42677;G:1236;C:0;T:2;total:43915 | iSNV |
| F15 | F15-2 | 10069 | NS5 | 0.8175 | A:5;G:2;C:14895;T:66711;total:81613 | iSNV |
| F15 | F15-20 | 1046 | E | 0.0772 | A:0;G:78230;C:6550;T:11;total:84791 | iSNV |
| F15 | F15-20 | 1116 | E | 0.0447 | A:84221;G:3949;C:0;T:13;total:88183 | iSNV |
| F15 | F15-20 | 1343 | E | 0.0262 | A:3;G:2;C:87762;T:2364;total:90131 | iSNV |
| F15 | F15-20 | 1390 | E | 0.0283 | A:78159;G:14;C:2285;T:110;total:80568 | iSNV |
| F15 | F15-20 | 1468 | E | 0.0925 | A:6501;G:0;C:63478;T:268;total:70247 | iSNV |
| F15 | F15-20 | 1803 | E | 0.1306 | A:83648;G:12568;C:0;T:0;total:96216 | iSNV |
| F15 | F15-20 | 1911 | E | 0.3474 | A:40443;G:21623;C:169;T:1;total:62236 | iSNV |
| F15 | F15-20 | 3579 | NS1 | 0.0226 | A:65034;G:1509;C:1;T:1;total:66545 | iSNV |
| F15 | F15-20 | 4181 | NS2A | 0.0821 | A:3;G:0;C:48099;T:4306;total:52408 | iSNV |
| F15 | F15-20 | 5311 | NS3 | 0.0379 | A:4;G:139;C:3203;T:81151;total:84497 | iSNV |
| F15 | F15-20 | 5358 | NS3 | 0.1173 | A:28;G:83752;C:11142;T:4;total:94926 | iSNV |
| F15 | F15-20 | 5835 | NS3 | 0.0247 | A:2162;G:85133;C:5;T:2;total:87302 | iSNV |
| F15 | F15-20 | 6533 | NS4A | 0.0446 | A:6;G:9;C:2870;T:61444;total:64329 | iSNV |
| F15 | F15-20 | 7481 | NS4B | 0.0818 | A:11;G:4;C:5435;T:60925;total:66375 | iSNV |
| F15 | F15-20 | 7495 | NS4B | 0.2952 | A:5;G:3;C:20143;T:48061;total:68212 | iSNV |
| F15 | F15-20 | 7543 | NS4B | 0.024 | A:75464;G:1861;C:0;T:1;total:77326 | iSNV |
| F15 | F15-20 | 7657 | NS4B | 0.3885 | A:48581;G:30908;C:54;T:7;total:79550 | iSNV |
| F15 | F15-20 | 8081 | NS5 | 0.0303 | A:103259;G:3236;C:2;T:5;total:106502 | iSNV |
| F15 | F15-20 | 9688 | NS5 | 0.2731 | A:5;G:2;C:60895;T:22891;total:83793 | iSNV |
| F15 | F15-20 | 9932 | NS5 | 0.0875 | A:7489;G:7;C:16;T:78024;total:85536 | iSNV |
| F15 | F15-20 | 10069 | NS5 | 0.0265 | A:3;G:0;C:92362;T:2521;total:94886 | iSNV |
| F15 | F15-20 | 10071 | NS5 | 0.1169 | A:11156;G:1;C:2;T:84228;total:95387 | iSNV |
| F15 | F15-20 | 10115 | NS5 | 0.0702 | A:2;G:1;C:100477;T:7595;total:108075 | iSNV |
| F15 | F15-22 | 584 | M | 0.117 | A:8;G:0;C:80448;T:10665;total:91121 | iSNV |
| F15 | F15-22 | 1384 | E | 0.3205 | A:63940;G:12;C:40;T:30194;total:94186 | iSNV |
| F15 | F15-22 | 1786 | E | 0.1152 | A:17;G:7;C:11925;T:91482;total:103431 | iSNV |
| F15 | F15-22 | 1812 | E | 0.0379 | A:102251;G:318;C:4044;T:8;total:106621 | iSNV |
| F15 | F15-22 | 1911 | E | 0.1662 | A:55094;G:10995;C:56;T:0;total:66145 | iSNV |
| F15 | F15-22 | 1957 | E | 0.028 | A:5;G:0;C:69116;T:1992;total:71113 | iSNV |
| F15 | F15-22 | 2843 | NS1 | 0.1102 | A:6;G:0;C:111750;T:13843;total:125599 | iSNV |
| F15 | F15-22 | 4501 | NS2B | 0.0416 | A:81333;G:3532;C:13;T:2;total:84880 | iSNV |
| F15 | F15-22 | 4640 | NS3 | 0.1078 | A:80434;G:9728;C:5;T:5;total:90172 | iSNV |
| F15 | F15-22 | 5311 | NS3 | 0.125 | A:0;G:96;C:10064;T:70290;total:80450 | iSNV |
| F15 | F15-22 | 5626 | NS3 | 0.0309 | A:3;G:1;C:2665;T:83542;total:86211 | iSNV |
| F15 | F15-22 | 6533 | NS4A | 0.1598 | A:3;G:4;C:9799;T:51481;total:61287 | iSNV |
| F15 | F15-22 | 7448 | NS4B | 0.0212 | A:62165;G:14;C:1347;T:4;total:63530 | iSNV |
| F15 | F15-22 | 7495 | NS4B | 0.0661 | A:7;G:8;C:4470;T:63096;total:67581 | iSNV |
| F15 | F15-22 | 7543 | NS4B | 0.0468 | A:77429;G:3803;C:1;T:0;total:81233 | iSNV |
| F15 | F15-22 | 7656 | NS4B | 0.118 | A:9760;G:72857;C:49;T:7;total:82673 | iSNV |
| F15 | F15-22 | 7657 | NS4B | 0.245 | A:62167;G:20201;C:56;T:3;total:82427 | iSNV |
| F15 | F15-22 | 8064 | NS5 | 0.0446 | A:4722;G:100953;C:2;T:20;total:105697 | iSNV |
| F15 | F15-22 | 8894 | NS5 | 0.0421 | A:3292;G:7;C:74638;T:124;total:78061 | iSNV |
| F15 | F15-22 | 9225 | NS5 | 0.3145 | A:32605;G:2;C:71029;T:23;total:103659 | iSNV |
| F15 | F15-22 | 9688 | NS5 | 0.1821 | A:5;G:0;C:68025;T:15148;total:83178 | iSNV |
| F15 | F15-22 | 9909 | NS5 | 0.0376 | A:79336;G:3108;C:0;T:0;total:82444 | iSNV |
| F15 | F15-22 | 9932 | NS5 | 0.044 | A:3545;G:3;C:29;T:76950;total:80527 | iSNV |
| F15 | F15-22 | 9937 | NS5 | 0.0241 | A:80205;G:1987;C:1;T:2;total:82195 | iSNV |
| F15 | F15-22 | 10014 | NS5 | 0.0434 | A:88203;G:17;C:4009;T:24;total:92253 | iSNV |
| F15 | F15-22 | 10069 | NS5 | 0.0224 | A:4;G:1;C:89522;T:2054;total:91581 | iSNV |
| F15 | F15-22 | 10401 | 3'-UTR | 0.0256 | A:2216;G:15;C:11;T:84194;total:86436 | iSNV |
| F15 | F15-23 | 483 | M | 0.044 | A:8;G:2689;C:10;T:58346;total:61053 | iSNV |
| F15 | F15-23 | 497 | M | 0.0635 | A:25;G:59323;C:4029;T:16;total:63393 | iSNV |
| F15 | F15-23 | 1116 | E | 0.0364 | A:54144;G:2048;C:0;T:1;total:56193 | iSNV |
| F15 | F15-23 | 1417 | E | 0.0545 | A:1;G:2;C:45786;T:2644;total:48433 | iSNV |
| F15 | F15-23 | 1735 | E | 0.0424 | A:4;G:5;C:2302;T:51878;total:54189 | iSNV |

| | | | | | | |
|---|---|---|---|---|---|---|
| F15 | F15-23 | 1803 | E | 0.0271 | A:59406;G:1658;C:0;T:0;total:61064 | iSNV |
| F15 | F15-23 | 1911 | E | 0.6088 | A:14847;G:23091;C:6;T:1;total:37945 | iSNV |
| F15 | F15-23 | 3001 | NS1 | 0.0535 | A:3061;G:54059;C:3;T:5;total:57128 | iSNV |
| F15 | F15-23 | 4718 | NS3 | 0.0425 | A:3;G:0;C:44165;T:1961;total:46129 | iSNV |
| F15 | F15-23 | 5358 | NS3 | 0.0288 | A:13;G:55584;C:1650;T:1;total:57248 | iSNV |
| F15 | F15-23 | 5835 | NS3 | 0.0515 | A:2047;G:37651;C:2;T:5;total:39705 | iSNV |
| F15 | F15-23 | 6533 | NS4A | 0.0293 | A:2;G:0;C:872;T:28843;total:29717 | iSNV |
| F15 | F15-23 | 7193 | NS4A | 0.3783 | A:2;G:0;C:13856;T:22766;total:36624 | iSNV |
| F15 | F15-23 | 7495 | NS4B | 0.0828 | A:3;G:2;C:3562;T:39433;total:43000 | iSNV |
| F15 | F15-23 | 7547 | NS4B | 0.4066 | A:15;G:18589;C:11;T:27100;total:45715 | iSNV |
| F15 | F15-23 | 7656 | NS4B | 0.0462 | A:2068;G:42678;C:3;T:2;total:44751 | iSNV |
| F15 | F15-23 | 7657 | NS4B | 0.2317 | A:34212;G:10323;C:8;T:2;total:44545 | iSNV |
| F15 | F15-23 | 8832 | NS5 | 0.0746 | A:3;G:3;C:3940;T:48817;total:52763 | iSNV |
| F15 | F15-23 | 9688 | NS5 | 0.0317 | A:3;G:1;C:45434;T:1491;total:46929 | iSNV |
| F15 | F15-23 | 9932 | NS5 | 0.0467 | A:2157;G:8;C:17;T:43908;total:46090 | iSNV |
| F15 | F15-23 | 10069 | NS5 | 0.0294 | A:1;G:0;C:53521;T:1626;total:55148 | iSNV |
| F15 | F15-23 | 10071 | NS5 | 0.0271 | A:1509;G:0;C:4;T:54107;total:55620 | iSNV |
| F15 | F15-23 | 10092 | NS5 | 0.4281 | A:23609;G:31525;C:1;T:2;total:55137 | iSNV |
| F15 | F15-23 | 10325 | NS5 | 0.0292 | A:4;G:19;C:1902;T:63057;total:64982 | iSNV |
| F15 | F15-25 | 340 | C | 0.3773 | A:7;G:1;C:52859;T:32045;total:84912 | iSNV |
| F15 | F15-25 | 656 | M | 0.0625 | A:2;G:0;C:4394;T:65904;total:70300 | iSNV |
| F15 | F15-25 | 1501 | E | 0.0683 | A:0;G:0;C:75197;T:5518;total:80715 | iSNV |
| F15 | F15-25 | 1911 | E | 0.6786 | A:22483;G:47436;C:23;T:0;total:69942 | iSNV |
| F15 | F15-25 | 2141 | E | 0.0477 | A:68479;G:3433;C:1;T:7;total:71920 | iSNV |
| F15 | F15-25 | 2275 | E | 0.0381 | A:2;G:4;C:2524;T:63563;total:66093 | iSNV |
| F15 | F15-25 | 2873 | NS1 | 0.0892 | A:102156;G:18;C:10;T:10011;total:112195 | iSNV |
| F15 | F15-25 | 2882 | NS1 | 0.0513 | A:102823;G:5568;C:52;T:12;total:108455 | iSNV |
| F15 | F15-25 | 5181 | NS3 | 0.0205 | A:4;G:0;C:65334;T:1374;total:66712 | iSNV |
| F15 | F15-25 | 5311 | NS3 | 0.1158 | A:4;G:74;C:8321;T:63443;total:71842 | iSNV |
| F15 | F15-25 | 5835 | NS3 | 0.0754 | A:6839;G:83807;C:3;T:1;total:90650 | iSNV |
| F15 | F15-25 | 6861 | NS4A | 0.0769 | A:1;G:0;C:65330;T:5445;total:70776 | iSNV |
| F15 | F15-25 | 7495 | NS4B | 0.0944 | A:4;G:0;C:5627;T:53952;total:59583 | iSNV |
| F15 | F15-25 | 7543 | NS4B | 0.0484 | A:69201;G:3526;C:0;T:0;total:72727 | iSNV |
| F15 | F15-25 | 7575 | NS4B | 0.0698 | A:67428;G:5061;C:1;T:0;total:72490 | iSNV |
| F15 | F15-25 | 7656 | NS4B | 0.1327 | A:9846;G:64277;C:63;T:5;total:74191 | iSNV |
| F15 | F15-25 | 7657 | NS4B | 0.2475 | A:55664;G:18317;C:13;T:3;total:73997 | iSNV |
| F15 | F15-25 | 9221 | NS5 | 0.0509 | A:95284;G:13;C:9;T:5119;total:100425 | iSNV |
| F15 | F15-25 | 9688 | NS5 | 0.042 | A:5;G:1;C:83224;T:3654;total:86884 | iSNV |
| F15 | F15-25 | 10069 | NS5 | 0.5816 | A:2;G:1;C:28453;T:39548;total:68004 | iSNV |
| F15 | F15-26 | 1911 | E | 0.7626 | A:10535;G:33832;C:5;T:1;total:44373 | iSNV |
| F15 | F15-26 | 5311 | NS3 | 0.0225 | A:1;G:74;C:1069;T:46159;total:47303 | iSNV |
| F15 | F15-26 | 6125 | NS3 | 0.023 | A:1265;G:53575;C:0;T:4;total:54844 | iSNV |
| F15 | F15-26 | 6533 | NS4A | 0.0393 | A:1;G:2;C:1841;T:44934;total:46778 | iSNV |
| F15 | F15-26 | 7179 | NS4A | 0.1949 | A:8629;G:35630;C:1;T:1;total:44261 | iSNV |
| F15 | F15-26 | 7495 | NS4B | 0.0534 | A:2;G:3;C:2115;T:37470;total:39590 | iSNV |
| F15 | F15-26 | 7656 | NS4B | 0.0512 | A:2616;G:48262;C:113;T:6;total:50997 | iSNV |
| F15 | F15-26 | 7657 | NS4B | 0.3575 | A:32579;G:18168;C:63;T:1;total:50811 | iSNV |
| F15 | F15-26 | 8091 | NS5 | 0.0208 | A:2;G:3;C:55683;T:1183;total:56871 | iSNV |
| F15 | F15-26 | 9358 | NS5 | 0.201 | A:13483;G:53563;C:4;T:6;total:67056 | iSNV |
| F15 | F15-26 | 9688 | NS5 | 0.0608 | A:3;G:0;C:54090;T:3503;total:57596 | iSNV |
| F15 | F15-26 | 10069 | NS5 | 0.7201 | A:1;G:0;C:12406;T:31908;total:44315 | iSNV |
| F15 | F15-28 | 836 | M | 0.0422 | A:1;G:0;C:1202;T:27274;total:28477 | iSNV |
| F15 | F15-28 | 902 | M | 0.1085 | A:0;G:1;C:26232;T:3194;total:29427 | iSNV |
| F15 | F15-28 | 982 | E | 0.0754 | A:28332;G:1;C:2313;T:3;total:30649 | iSNV |
| F15 | F15-28 | 1389 | E | 0.0245 | A:36898;G:928;C:4;T:0;total:37830 | iSNV |
| F15 | F15-28 | 1911 | E | 0.5601 | A:14060;G:17892;C:8;T:0;total:31960 | iSNV |
| F15 | F15-28 | 4394 | NS2B | 0.0233 | A:3;G:1;C:30161;T:721;total:30886 | iSNV |
| F15 | F15-28 | 5311 | NS3 | 0.1492 | A:1;G:19;C:4624;T:26336;total:30980 | iSNV |
| F15 | F15-28 | 5940 | NS3 | 0.031 | A:1246;G:38890;C:0;T:0;total:40136 | iSNV |
| F15 | F15-28 | 6533 | NS4A | 0.0416 | A:1;G:1;C:1275;T:29369;total:30646 | iSNV |
| F15 | F15-28 | 7265 | NS4A | 0.0713 | A:1816;G:23649;C:0;T:3;total:25468 | iSNV |
| F15 | F15-28 | 7271 | NS4B | 0.0393 | A:1;G:0;C:973;T:23745;total:24719 | iSNV |
| F15 | F15-28 | 7494 | NS4B | 0.0686 | A:21;G:22742;C:1;T:1677;total:24441 | iSNV |
| F15 | F15-28 | 7495 | NS4B | 0.3296 | A:1;G:0;C:8254;T:16781;total:25036 | iSNV |
| F15 | F15-28 | 7656 | NS4B | 0.1079 | A:3527;G:29118;C:19;T:3;total:32667 | iSNV |
| F15 | F15-28 | 7657 | NS4B | 0.253 | A:24291;G:8230;C:3;T:0;total:32524 | iSNV |
| F15 | F15-28 | 8456 | NS5 | 0.0712 | A:42728;G:9;C:1;T:3281;total:46019 | iSNV |
| F15 | F15-28 | 8987 | NS5 | 0.0495 | A:1780;G:0;C:8;T:34123;total:35911 | iSNV |
| F15 | F15-28 | 9688 | NS5 | 0.0744 | A:3;G:0;C:36520;T:2937;total:39460 | iSNV |
| F15 | F15-28 | 9932 | NS5 | 0.0216 | A:758;G:0;C:7;T:34245;total:35010 | iSNV |
| F15 | F15-28 | 10069 | NS5 | 0.0839 | A:0;G:0;C:27341;T:2504;total:29845 | iSNV |
| F15 | F15-28 | 10092 | NS5 | 0.0769 | A:2386;G:28632;C:4;T:3;total:31025 | iSNV |
| F15 | F15-30 | 785 | M | 0.0487 | A:24407;G:1250;C:3;T:5;total:25665 | iSNV |
| F15 | F15-30 | 1116 | E | 0.0248 | A:23817;G:606;C:0;T:2;total:24425 | iSNV |
| F15 | F15-30 | 1390 | E | 0.0234 | A:27427;G:5;C:659;T:8;total:28099 | iSNV |
| F15 | F15-30 | 1417 | E | 0.0254 | A:1;G:1;C:27574;T:719;total:28295 | iSNV |
| F15 | F15-30 | 1461 | E | 0.0528 | A:0;G:4;C:1343;T:24063;total:25410 | iSNV |
| F15 | F15-30 | 1911 | E | 0.3867 | A:12008;G:7576;C:5;T:1;total:19590 | iSNV |
| F15 | F15-30 | 2129 | E | 0.6042 | A:9845;G:15023;C:3;T:2;total:24873 | iSNV |
| F15 | F15-30 | 3230 | NS1 | 0.5718 | A:13889;G:18538;C:6;T:1;total:32434 | iSNV |
| F15 | F15-30 | 4192 | NS2A | 0.0471 | A:0;G:6;C:659;T:13316;total:13981 | iSNV |
| F15 | F15-30 | 4385 | NS2B | 0.0224 | A:3;G:0;C:24764;T:568;total:25335 | iSNV |

| | | | | | | |
|---|---|---|---|---|---|---|
| F15 | F15-30 | 6533 | NS4A | 0.6253 | A:2;G:2;C:13074;T:7838;total:20916 | iSNV |
| F15 | F15-30 | 6644 | NS4A | 0.0334 | A:0;G:5;C:604;T:17442;total:18051 | iSNV |
| F15 | F15-30 | 7373 | NS4B | 0.0223 | A:54;G:21682;C:0;T:496;total:22232 | iSNV |
| F15 | F15-30 | 7495 | NS4B | 0.0669 | A:0;G:13;C:1254;T:17451;total:18718 | iSNV |
| F15 | F15-30 | 7543 | NS4B | 0.0531 | A:23622;G:1326;C:2;T:0;total:24950 | iSNV |
| F15 | F15-30 | 7626 | NS4B | 0.0214 | A:5;G:647;C:11;T:29552;total:30215 | iSNV |
| F15 | F15-30 | 7657 | NS4B | 0.6954 | A:8967;G:20468;C:1;T:2;total:29438 | iSNV |
| F15 | F15-30 | 8900 | NS5 | 0.024 | A:536;G:2;C:21698;T:7;total:22243 | iSNV |
| F15 | F15-30 | 9688 | NS5 | 0.6645 | A:2;G:3;C:7445;T:14740;total:22190 | iSNV |
| F15 | F15-30 | 9932 | NS5 | 0.0624 | A:1412;G:19;C:2;T:21178;total:22611 | iSNV |
| F15 | F15-30 | 10069 | NS5 | 0.0256 | A:0;G:0;C:19565;T:516;total:20081 | iSNV |
| F15 | F15-4 | 1116 | E | 0.0405 | A:80395;G:3395;C:0;T:4;total:83794 | iSNV |
| F15 | F15-4 | 1786 | E | 0.0458 | A:4;G:10;C:4093;T:85193;total:89300 | iSNV |
| F15 | F15-4 | 1911 | E | 0.3215 | A:38044;G:18037;C:10;T:0;total:56091 | iSNV |
| F15 | F15-4 | 1914 | E | 0.0891 | A:48540;G:4749;C:1;T:0;total:53290 | iSNV |
| F15 | F15-4 | 2477 | E | 0.0518 | A:7;G:2753;C:17;T:50340;total:53117 | iSNV |
| F15 | F15-4 | 3761 | NS2A | 0.0425 | A:0;G:3;C:63855;T:2837;total:66695 | iSNV |
| F15 | F15-4 | 3962 | NS2A | 0.0228 | A:9;G:2;C:1563;T:66934;total:68508 | iSNV |
| F15 | F15-4 | 4021 | NS2A | 0.0222 | A:1306;G:57316;C:0;T:6;total:58628 | iSNV |
| F15 | F15-4 | 4250 | NS2B | 0.0906 | A:49358;G:4921;C:0;T:6;total:54285 | iSNV |
| F15 | F15-4 | 5311 | NS3 | 0.041 | A:2;G:164;C:3086;T:71837;total:75089 | iSNV |
| F15 | F15-4 | 6533 | NS4A | 0.2837 | A:66;G:1;C:13217;T:33292;total:46576 | iSNV |
| F15 | F15-4 | 7381 | NS4B | 0.0438 | A:0;G:0;C:63884;T:2933;total:66817 | iSNV |
| F15 | F15-4 | 7495 | NS4B | 0.0631 | A:5;G:2;C:4138;T:61349;total:65494 | iSNV |
| F15 | F15-4 | 7543 | NS4B | 0.0243 | A:69280;G:1727;C:1;T:2;total:71010 | iSNV |
| F15 | F15-4 | 7546 | NS4B | 0.0263 | A:67708;G:1833;C:15;T:5;total:69561 | iSNV |
| F15 | F15-4 | 7656 | NS4B | 0.1066 | A:7248;G:60688;C:37;T:6;total:67979 | iSNV |
| F15 | F15-4 | 7657 | NS4B | 0.5009 | A:33790;G:33870;C:36;T:2;total:67698 | iSNV |
| F15 | F15-4 | 8140 | NS5 | 0.0251 | A:91640;G:243;C:2375;T:4;total:94262 | iSNV |
| F15 | F15-4 | 9220 | NS5 | 0.0298 | A:71560;G:7;C:2205;T:2;total:73774 | iSNV |
| F15 | F15-4 | 9688 | NS5 | 0.3743 | A:0;G:2;C:43804;T:26207;total:70013 | iSNV |
| F15 | F15-4 | 9932 | NS5 | 0.0341 | A:2257;G:4;C:4;T:63861;total:66126 | iSNV |
| F15 | F15-4 | 10069 | NS5 | 0.0659 | A:2;G:0;C:75341;T:5319;total:80662 | iSNV |
| F15 | F15-4 | 10092 | NS5 | 0.0434 | A:3616;G:79618;C:0;T:7;total:83241 | iSNV |
| F15 | F15-5 | 933 | M | 0.04 | A:3155;G:75704;C:5;T:4;total:78868 | iSNV |
| F15 | F15-5 | 1911 | E | 0.7671 | A:15242;G:50150;C:31;T:3;total:65426 | iSNV |
| F15 | F15-5 | 3081 | NS1 | 0.0483 | A:102307;G:5201;C:5;T:1;total:107514 | iSNV |
| F15 | F15-5 | 4853 | NS3 | 0.0245 | A:74653;G:1879;C:0;T:8;total:76540 | iSNV |
| F15 | F15-5 | 5311 | NS3 | 0.3308 | A:2;G:33;C:28375;T:57343;total:85753 | iSNV |
| F15 | F15-5 | 6523 | NS4A | 0.088 | A:0;G:1;C:45604;T:4401;total:50006 | iSNV |
| F15 | F15-5 | 6637 | NS4A | 0.1048 | A:4;G:1;C:6130;T:52323;total:58458 | iSNV |
| F15 | F15-5 | 7495 | NS4B | 0.0278 | A:9;G:9;C:2183;T:76116;total:78317 | iSNV |
| F15 | F15-5 | 7656 | NS4B | 0.3284 | A:4549;G:48749;C:26066;T:5;total:79369 | iSNV |
| F15 | F15-5 | 7657 | NS4B | 0.2656 | A:58159;G:21042;C:2;T:1;total:79204 | iSNV |
| F15 | F15-5 | 8093 | NS5 | 0.0203 | A:0;G:0;C:94863;T:1970;total:96833 | iSNV |
| F15 | F15-5 | 9688 | NS5 | 0.3959 | A:3;G:0;C:51075;T:33488;total:84566 | iSNV |
| F15 | F15-5 | 9887 | NS5 | 0.0356 | A:3;G:3;C:3439;T:93046;total:96491 | iSNV |
| F15 | F15-7 | 697 | M | 0.0233 | A:2;G:0;C:2046;T:85602;total:87650 | iSNV |
| F15 | F15-7 | 1116 | E | 0.0541 | A:73549;G:4214;C:0;T:2;total:77765 | iSNV |
| F15 | F15-7 | 1911 | E | 0.2317 | A:44109;G:13328;C:84;T:1;total:57522 | iSNV |
| F15 | F15-7 | 3139 | NS1 | 0.8675 | A:13063;G:85498;C:2;T:8;total:98571 | iSNV |
| F15 | F15-7 | 5311 | NS3 | 0.0224 | A:4;G:50;C:1633;T:71154;total:72841 | iSNV |
| F15 | F15-7 | 5336 | NS3 | 0.0229 | A:4;G:1;C:1705;T:72627;total:74337 | iSNV |
| F15 | F15-7 | 6533 | NS4A | 0.056 | A:1;G:0;C:2982;T:50191;total:53174 | iSNV |
| F15 | F15-7 | 7495 | NS4B | 0.8646 | A:1;G:0;C:47696;T:7471;total:55168 | iSNV |
| F15 | F15-7 | 7543 | NS4B | 0.0343 | A:62760;G:2231;C:1;T:0;total:64992 | iSNV |
| F15 | F15-7 | 9688 | NS5 | 0.917 | A:4;G:0;C:5634;T:62205;total:67843 | iSNV |
| F15 | F15-7 | 10069 | NS5 | 0.0482 | A:0;G:0;C:73946;T:3745;total:77691 | iSNV |
| F15 | F15-8 | 401 | C | 0.0242 | A:72804;G:1811;C:13;T:12;total:74640 | iSNV |
| F15 | F15-8 | 798 | M | 0.2927 | A:39716;G:21;C:16446;T:1;total:56184 | iSNV |
| F15 | F15-8 | 1348 | E | 0.0263 | A:66669;G:3;C:1806;T:4;total:68482 | iSNV |
| F15 | F15-8 | 1911 | E | 0.1588 | A:40349;G:7935;C:1677;T:1;total:49962 | iSNV |
| F15 | F15-8 | 3538 | NS1 | 0.2039 | A:35959;G:2;C:9211;T:0;total:45172 | iSNV |
| F15 | F15-8 | 4401 | NS2B | 0.2001 | A:1;G:49104;C:12285;T:3;total:61393 | iSNV |
| F15 | F15-8 | 5311 | NS3 | 0.037 | A:0;G:52;C:2351;T:61062;total:63465 | iSNV |
| F15 | F15-8 | 5528 | NS3 | 0.2003 | A:58753;G:14720;C:0;T:5;total:73478 | iSNV |
| F15 | F15-8 | 5835 | NS3 | 0.2521 | A:14564;G:43194;C:1;T:2;total:57761 | iSNV |
| F15 | F15-8 | 6533 | NS4A | 0.4665 | A:2;G:3;C:20560;T:23507;total:44072 | iSNV |
| F15 | F15-8 | 7495 | NS4B | 0.0487 | A:3;G:4;C:2692;T:52471;total:55170 | iSNV |
| F15 | F15-8 | 7656 | NS4B | 0.0403 | A:2385;G:56716;C:10;T:1;total:59112 | iSNV |
| F15 | F15-8 | 7657 | NS4B | 0.8142 | A:10951;G:47971;C:2;T:0;total:58924 | iSNV |
| F15 | F15-8 | 9688 | NS5 | 0.4711 | A:5;G:0;C:32462;T:28930;total:61397 | iSNV |
| F15 | F15-8 | 9932 | NS5 | 0.0293 | A:1731;G:0;C:69;T:57198;total:58998 | iSNV |
| F15 | F15-8 | 10419 | 3'-UTR | 0.4188 | A:2;G:1;C:34284;T:24715;total:59002 | iSNV |
| F20 | F20-1 | 1116 | E | 0.0986 | A:12739;G:1395;C:0;T:0;total:14134 | iSNV |
| F20 | F20-1 | 1417 | E | 0.0338 | A:2;G:0;C:14587;T:511;total:15100 | iSNV |
| F20 | F20-1 | 1482 | E | 0.0384 | A:438;G:10952;C:2;T:1;total:11393 | iSNV |
| F20 | F20-1 | 1803 | E | 0.1877 | A:13105;G:3030;C:0;T:1;total:16136 | iSNV |
| F20 | F20-1 | 1911 | E | 0.6379 | A:3580;G:6284;C:21;T:1;total:9886 | iSNV |
| F20 | F20-1 | 3528 | NS1 | 0.0373 | A:0;G:0;C:10409;T:404;total:10813 | iSNV |
| F20 | F20-1 | 3814 | NS2A | 0.0514 | A:0;G:1;C:787;T:14507;total:15295 | iSNV |
| F20 | F20-1 | 3993 | NS2A | 0.1425 | A:2228;G:13404;C:1;T:0;total:15633 | iSNV |

| | | | | | | |
|---|---|---|---|---|---|---|
| F20 | F20-1 | 5311 | NS3 | 0.7049 | A:1;G:12;C:9672;T:4056;total:13741 | iSNV |
| F20 | F20-1 | 5358 | NS3 | 0.1789 | A:0;G:12411;C:2705;T:3;total:15119 | iSNV |
| F20 | F20-1 | 6707 | NS4A | 0.0386 | A:446;G:5;C:2;T:11091;total:11544 | iSNV |
| F20 | F20-1 | 7495 | NS4B | 0.0526 | A:0;G:3;C:656;T:11812;total:12471 | iSNV |
| F20 | F20-1 | 7526 | NS4B | 0.212 | A:3052;G:4;C:20;T:11319;total:14395 | iSNV |
| F20 | F20-1 | 7626 | NS4B | 0.7207 | A:4;G:12558;C:4;T:4872;total:17438 | iSNV |
| F20 | F20-1 | 7657 | NS4B | 0.0349 | A:16734;G:606;C:9;T:0;total:17349 | iSNV |
| F20 | F20-1 | 9688 | NS5 | 0.0455 | A:1;G:0;C:10895;T:520;total:11416 | iSNV |
| F20 | F20-1 | 9932 | NS5 | 0.0306 | A:389;G:0;C:0;T:12308;total:12697 | iSNV |
| F20 | F20-1 | 10071 | NS5 | 0.1814 | A:2082;G:0;C:1;T:9391;total:11474 | iSNV |
| F20 | F20-1 | 10086 | NS5 | 0.0324 | A:388;G:3;C:1;T:11558;total:11950 | iSNV |
| F20 | F20-1 | 10092 | NS5 | 0.0346 | A:424;G:11825;C:0;T:3;total:12252 | iSNV |
| F20 | F20-10 | 541 | M | 0.0221 | A:1246;G:5;C:28;T:54904;total:56183 | iSNV |
| F20 | F20-10 | 1116 | E | 0.078 | A:44741;G:3788;C:1;T:4;total:48534 | iSNV |
| F20 | F20-10 | 1247 | E | 0.0209 | A:1;G:0;C:1107;T:51700;total:52808 | iSNV |
| F20 | F20-10 | 1296 | E | 0.0305 | A:2;G:0;C:1730;T:54864;total:56596 | iSNV |
| F20 | F20-10 | 1347 | E | 0.0217 | A:56114;G:1250;C:7;T:1;total:57372 | iSNV |
| F20 | F20-10 | 1390 | E | 0.0219 | A:53306;G:6;C:1195;T:6;total:54513 | iSNV |
| F20 | F20-10 | 1406 | E | 0.0718 | A:4241;G:54793;C:3;T:2;total:59039 | iSNV |
| F20 | F20-10 | 1474 | E | 0.0343 | A:47252;G:1;C:11;T:1682;total:48946 | iSNV |
| F20 | F20-10 | 1911 | E | 0.4957 | A:20037;G:19705;C:6;T:1;total:39749 | iSNV |
| F20 | F20-10 | 2226 | E | 0.0348 | A:1;G:0;C:45681;T:1648;total:47330 | iSNV |
| F20 | F20-10 | 2364 | E | 0.0222 | A:1;G:0;C:35116;T:800;total:35917 | iSNV |
| F20 | F20-10 | 3113 | NS1 | 0.066 | A:28;G:3;C:3637;T:51387;total:55055 | iSNV |
| F20 | F20-10 | 3949 | NS2A | 0.0448 | A:2;G:4;C:2703;T:57600;total:60309 | iSNV |
| F20 | F20-10 | 5311 | NS3 | 0.1416 | A:1;G:101;C:6842;T:41350;total:48294 | iSNV |
| F20 | F20-10 | 6368 | NS3 | 0.125 | A:4;G:5463;C:2;T:38223;total:43692 | iSNV |
| F20 | F20-10 | 6533 | NS4A | 0.0648 | A:2;G:2;C:2784;T:40146;total:42934 | iSNV |
| F20 | F20-10 | 7373 | NS4B | 0.1018 | A:38;G:35187;C:3996;T:5;total:39226 | iSNV |
| F20 | F20-10 | 7495 | NS4B | 0.1233 | A:2;G:2;C:4448;T:31620;total:36072 | iSNV |
| F20 | F20-10 | 7656 | NS4B | 0.0415 | A:2204;G:50782;C:86;T:2;total:53074 | iSNV |
| F20 | F20-10 | 7657 | NS4B | 0.5252 | A:25146;G:27807;C:2;T:1;total:52956 | iSNV |
| F20 | F20-10 | 8360 | NS5 | 0.021 | A:7;G:0;C:1303;T:60656;total:61966 | iSNV |
| F20 | F20-10 | 9246 | NS5 | 0.0206 | A:66221;G:1398;C:1;T:0;total:67620 | iSNV |
| F20 | F20-10 | 9688 | NS5 | 0.1251 | A:0;G:0;C:36404;T:5208;total:41612 | iSNV |
| F20 | F20-10 | 9932 | NS5 | 0.0247 | A:1077;G:2;C:23;T:42378;total:43480 | iSNV |
| F20 | F20-10 | 10043 | NS5 | 0.0232 | A:42913;G:1021;C:2;T:2;total:43938 | iSNV |
| F20 | F20-10 | 10069 | NS5 | 0.4376 | A:0;G:1;C:21879;T:17029;total:38909 | iSNV |
| F20 | F20-10 | 10111 | NS5 | 0.1105 | A:41998;G:5220;C:0;T:2;total:47220 | iSNV |
| F20 | F20-10 | 10308 | NS5 | 0.0632 | A:3633;G:53838;C:2;T:1;total:57474 | iSNV |
| F20 | F20-11 | 221 | C | 0.0644 | A:2977;G:43217;C:8;T:2;total:46204 | iSNV |
| F20 | F20-11 | 1417 | E | 0.025 | A:0;G:0;C:50249;T:1290;total:51539 | iSNV |
| F20 | F20-11 | 1803 | E | 0.1189 | A:47031;G:6350;C:0;T:0;total:53381 | iSNV |
| F20 | F20-11 | 1911 | E | 0.3441 | A:20751;G:10891;C:0;T:1;total:31643 | iSNV |
| F20 | F20-11 | 1913 | E | 0.3503 | A:19599;G:4;C:10598;T:47;total:30248 | iSNV |
| F20 | F20-11 | 5311 | NS3 | 0.1798 | A:1;G:135;C:7826;T:35548;total:43510 | iSNV |
| F20 | F20-11 | 5353 | NS3 | 0.0398 | A:45656;G:1895;C:0;T:5;total:47556 | iSNV |
| F20 | F20-11 | 5358 | NS3 | 0.1009 | A:3;G:42384;C:4758;T:0;total:47145 | iSNV |
| F20 | F20-11 | 6523 | NS4A | 0.4288 | A:0;G:0;C:21539;T:16170;total:37709 | iSNV |
| F20 | F20-11 | 6525 | NS4A | 0.0313 | A:1206;G:1;C:37186;T:31;total:38424 | iSNV |
| F20 | F20-11 | 6533 | NS4A | 0.0694 | A:0;G:2;C:2512;T:33638;total:36152 | iSNV |
| F20 | F20-11 | 7495 | NS4B | 0.0971 | A:2;G:1;C:3156;T:29323;total:32482 | iSNV |
| F20 | F20-11 | 7536 | NS4B | 0.0217 | A:861;G:10;C:7;T:38792;total:39670 | iSNV |
| F20 | F20-11 | 7575 | NS4B | 0.0602 | A:39592;G:2537;C:1;T:1;total:42131 | iSNV |
| F20 | F20-11 | 7629 | NS4B | 0.0846 | A:45360;G:0;C:4194;T:7;total:49561 | iSNV |
| F20 | F20-11 | 7656 | NS4B | 0.0955 | A:4578;G:43330;C:11;T:1;total:47920 | iSNV |
| F20 | F20-11 | 7657 | NS4B | 0.0636 | A:44819;G:3046;C:3;T:1;total:47869 | iSNV |
| F20 | F20-11 | 8900 | NS5 | 0.0639 | A:2875;G:6;C:42047;T:12;total:44940 | iSNV |
| F20 | F20-11 | 9170 | NS5 | 0.0561 | A:4;G:3;C:3298;T:55382;total:58687 | iSNV |
| F20 | F20-11 | 9607 | NS5 | 0.4401 | A:19610;G:1;C:15416;T:1;total:35028 | iSNV |
| F20 | F20-11 | 9688 | NS5 | 0.0936 | A:0;G:0;C:30202;T:3122;total:33324 | iSNV |
| F20 | F20-11 | 9995 | NS5 | 0.0233 | A:4;G:0;C:845;T:35296;total:36145 | iSNV |
| F20 | F20-11 | 10069 | NS5 | 0.0331 | A:1;G:0;C:33322;T:1141;total:34464 | iSNV |
| F20 | F20-11 | 10071 | NS5 | 0.5559 | A:19073;G:4;C:0;T:15241;total:34318 | iSNV |
| F20 | F20-13 | 344 | C | 0.2197 | A:1;G:9857;C:11;T:34990;total:44859 | iSNV |
| F20 | F20-13 | 542 | M | 0.068 | A:1;G:3194;C:16;T:43698;total:46909 | iSNV |
| F20 | F20-13 | 1435 | E | 0.0214 | A:992;G:45158;C:0;T:3;total:46153 | iSNV |
| F20 | F20-13 | 1468 | E | 0.0215 | A:912;G:1;C:41296;T:16;total:42225 | iSNV |
| F20 | F20-13 | 1803 | E | 0.4919 | A:26353;G:25523;C:1;T:3;total:51880 | iSNV |
| F20 | F20-13 | 1911 | E | 0.5146 | A:17037;G:18030;C:27;T:0;total:35094 | iSNV |
| F20 | F20-13 | 3156 | NS1 | 0.0229 | A:1146;G:48836;C:0;T:1;total:49983 | iSNV |
| F20 | F20-13 | 3926 | NS2A | 0.0676 | A:6;G:0;C:45549;T:3307;total:48862 | iSNV |
| F20 | F20-13 | 4021 | NS2A | 0.021 | A:840;G:38970;C:2;T:9;total:39821 | iSNV |
| F20 | F20-13 | 4943 | NS3 | 0.4056 | A:26239;G:17916;C:2;T:4;total:44161 | iSNV |
| F20 | F20-13 | 5311 | NS3 | 0.1917 | A:0;G:55;C:7681;T:32328;total:40064 | iSNV |
| F20 | F20-13 | 5358 | NS3 | 0.4974 | A:6;G:20826;C:20630;T:6;total:41468 | iSNV |
| F20 | F20-13 | 5993 | NS3 | 0.1983 | A:34868;G:8630;C:1;T:4;total:43503 | iSNV |
| F20 | F20-13 | 7495 | NS4B | 0.4279 | A:2;G:0;C:12592;T:16832;total:29426 | iSNV |
| F20 | F20-13 | 7546 | NS4B | 0.5288 | A:17751;G:19910;C:4;T:0;total:37665 | iSNV |
| F20 | F20-13 | 7657 | NS4B | 0.226 | A:33649;G:9835;C:28;T:0;total:43512 | iSNV |
| F20 | F20-13 | 8927 | NS5 | 0.2049 | A:33003;G:8509;C:5;T:1;total:41518 | iSNV |
| F20 | F20-13 | 9353 | NS5 | 0.029 | A:51804;G:1552;C:20;T:3;total:53379 | iSNV |

| | | | | | | |
|---|---|---|---|---|---|---|
| F20 | F20-13 | 10071 | NS5 | 0.5024 | A:16785;G:2;C:1;T:16629;total:33417 | iSNV |
| F20 | F20-13 | 10092 | NS5 | 0.4551 | A:15972;G:19115;C:0;T:2;total:35089 | iSNV |
| F20 | F20-13 | 10424 | 3'-UTR | 0.0227 | A:9;G:2;C:897;T:38448;total:39356 | iSNV |
| F20 | F20-13 | 10611 | 3'-UTR | 0.0214 | A:45260;G:994;C:0;T:0;total:46254 | iSNV |
| F20 | F20-13 | 10723 | 3'-UTR | 0.0306 | A:2;G:1;C:46036;T:1458;total:47497 | iSNV |
| F20 | F20-14 | 541 | M | 0.0269 | A:13;G:18;C:1597;T:57536;total:59164 | iSNV |
| F20 | F20-14 | 574 | M | 0.023 | A:1196;G:50666;C:2;T:1;total:51865 | iSNV |
| F20 | F20-14 | 1296 | E | 0.0227 | A:1;G:0;C:1342;T:57664;total:59007 | iSNV |
| F20 | F20-14 | 1911 | E | 0.7755 | A:9518;G:32869;C:6;T:0;total:42393 | iSNV |
| F20 | F20-14 | 4023 | NS2A | 0.0398 | A:1945;G:46840;C:5;T:4;total:48794 | iSNV |
| F20 | F20-14 | 5311 | NS3 | 0.0431 | A:3;G:36;C:2206;T:48910;total:51155 | iSNV |
| F20 | F20-14 | 5835 | NS3 | 0.1868 | A:12943;G:56311;C:4;T:1;total:69259 | iSNV |
| F20 | F20-14 | 7495 | NS4B | 0.0301 | A:2;G:3;C:1172;T:37644;total:38821 | iSNV |
| F20 | F20-14 | 7656 | NS4B | 0.0489 | A:2689;G:51655;C:551;T:2;total:54897 | iSNV |
| F20 | F20-14 | 7657 | NS4B | 0.7837 | A:11830;G:42801;C:44;T:2;total:54677 | iSNV |
| F20 | F20-14 | 10069 | NS5 | 0.6968 | A:1;G:1;C:12656;T:29070;total:41728 | iSNV |
| F20 | F20-16 | 1417 | E | 0.0321 | A:0;G:0;C:16585;T:551;total:17136 | iSNV |
| F20 | F20-16 | 1911 | E | 0.8392 | A:2029;G:10587;C:1;T:0;total:12617 | iSNV |
| F20 | F20-16 | 2069 | E | 0.0597 | A:809;G:0;C:12735;T:0;total:13544 | iSNV |
| F20 | F20-16 | 2433 | E | 0.0566 | A:702;G:11686;C:1;T:2;total:12391 | iSNV |
| F20 | F20-16 | 3096 | NS1 | 0.1361 | A:15672;G:3;C:0;T:2470;total:18145 | iSNV |
| F20 | F20-16 | 3542 | NS1 | 0.0428 | A:4;G:1;C:584;T:13055;total:13644 | iSNV |
| F20 | F20-16 | 3685 | NS1 | 0.1518 | A:13016;G:2331;C:2;T:0;total:15349 | iSNV |
| F20 | F20-16 | 5311 | NS3 | 0.6412 | A:3;G:34;C:9495;T:5334;total:14866 | iSNV |
| F20 | F20-16 | 5312 | NS3 | 0.5718 | A:0;G:1;C:8493;T:6362;total:14856 | iSNV |
| F20 | F20-16 | 5875 | NS3 | 0.1853 | A:3;G:5;C:3625;T:15921;total:19554 | iSNV |
| F20 | F20-16 | 7495 | NS4B | 0.0292 | A:0;G:5;C:345;T:11460;total:11810 | iSNV |
| F20 | F20-16 | 7546 | NS4B | 0.068 | A:13374;G:1042;C:899;T:2;total:15317 | iSNV |
| F20 | F20-16 | 7656 | NS4B | 0.0611 | A:1072;G:16460;C:0;T:0;total:17532 | iSNV |
| F20 | F20-16 | 7657 | NS4B | 0.7748 | A:3942;G:13551;C:2;T:2;total:17497 | iSNV |
| F20 | F20-16 | 7751 | NS5 | 0.0761 | A:2;G:0;C:1085;T:13156;total:14243 | iSNV |
| F20 | F20-16 | 10069 | NS5 | 0.237 | A:0;G:0;C:9471;T:2942;total:12413 | iSNV |
| F20 | F20-16 | 10092 | NS5 | 0.0577 | A:780;G:12727;C:0;T:2;total:13509 | iSNV |
| F20 | F20-16 | 10597 | 3'-UTR | 0.063 | A:1;G:0;C:13878;T:934;total:14813 | iSNV |
| F20 | F20-16 | 10808 | 3'-UTR | 0.0825 | A:1018;G:11309;C:2;T:4;total:12333 | iSNV |
| F20 | F20-17 | 287 | C | 0.0264 | A:826;G:30382;C:5;T:5;total:31218 | iSNV |
| F20 | F20-17 | 1116 | E | 0.0242 | A:25988;G:647;C:0;T:0;total:26635 | iSNV |
| F20 | F20-17 | 1298 | E | 0.4046 | A:12187;G:12;C:5;T:17914;total:30118 | iSNV |
| F20 | F20-17 | 1432 | E | 0.0333 | A:29594;G:4;C:3;T:1021;total:30622 | iSNV |
| F20 | F20-17 | 1453 | E | 0.0378 | A:5;G:1;C:1185;T:30090;total:31281 | iSNV |
| F20 | F20-17 | 1491 | E | 0.0569 | A:24657;G:1490;C:2;T:1;total:26150 | iSNV |
| F20 | F20-17 | 1706 | E | 0.0248 | A:30606;G:781;C:1;T:5;total:31393 | iSNV |
| F20 | F20-17 | 1911 | E | 0.4612 | A:11822;G:10126;C:4;T:0;total:21952 | iSNV |
| F20 | F20-17 | 1963 | E | 0.042 | A:0;G:0;C:22705;T:996;total:23701 | iSNV |
| F20 | F20-17 | 2367 | E | 0.0315 | A:19928;G:650;C:3;T:4;total:20585 | iSNV |
| F20 | F20-17 | 5216 | NS3 | 0.2151 | A:20834;G:5711;C:1;T:0;total:26546 | iSNV |
| F20 | F20-17 | 5311 | NS3 | 0.1155 | A:2;G:23;C:3257;T:24896;total:28178 | iSNV |
| F20 | F20-17 | 5343 | NS3 | 0.0444 | A:4;G:1;C:1256;T:26976;total:28237 | iSNV |
| F20 | F20-17 | 5940 | NS3 | 0.0297 | A:1096;G:35705;C:0;T:2;total:36803 | iSNV |
| F20 | F20-17 | 6449 | NS3 | 0.04 | A:2;G:0;C:1122;T:26918;total:28042 | iSNV |
| F20 | F20-17 | 6533 | NS4A | 0.0228 | A:0;G:3;C:531;T:22739;total:23273 | iSNV |
| F20 | F20-17 | 6978 | NS4A | 0.0807 | A:2187;G:24895;C:2;T:1;total:27085 | iSNV |
| F20 | F20-17 | 7488 | NS4B | 0.2152 | A:15499;G:147;C:0;T:4292;total:19938 | iSNV |
| F20 | F20-17 | 7495 | NS4B | 0.0555 | A:1;G:1;C:1148;T:19519;total:20669 | iSNV |
| F20 | F20-17 | 7656 | NS4B | 0.2709 | A:8383;G:22533;C:22;T:0;total:30938 | iSNV |
| F20 | F20-17 | 7657 | NS4B | 0.2402 | A:23428;G:7429;C:65;T:0;total:30922 | iSNV |
| F20 | F20-17 | 8656 | NS5 | 0.4271 | A:2;G:0;C:12377;T:16594;total:28973 | iSNV |
| F20 | F20-17 | 8659 | NS5 | 0.4349 | A:16048;G:12358;C:5;T:1;total:28412 | iSNV |
| F20 | F20-17 | 9221 | NS5 | 0.0371 | A:37822;G:12;C:1462;T:6;total:39302 | iSNV |
| F20 | F20-17 | 9688 | NS5 | 0.0647 | A:2;G:1;C:22624;T:1567;total:24194 | iSNV |
| F20 | F20-17 | 9932 | NS5 | 0.0327 | A:809;G:0;C:7;T:23883;total:24699 | iSNV |
| F20 | F20-17 | 10069 | NS5 | 0.2221 | A:0;G:0;C:17586;T:5023;total:22609 | iSNV |
| F20 | F20-17 | 10092 | NS5 | 0.0544 | A:1307;G:22601;C:98;T:0;total:24006 | iSNV |
| F20 | F20-19 | 971 | M | 0.0378 | A:0;G:2;C:1050;T:26690;total:27742 | iSNV |
| F20 | F20-19 | 1116 | E | 0.0202 | A:28106;G:580;C:0;T:1;total:28687 | iSNV |
| F20 | F20-19 | 1296 | E | 0.365 | A:1;G:1;C:10985;T:19102;total:30089 | iSNV |
| F20 | F20-19 | 1638 | E | 0.1823 | A:5773;G:1;C:25873;T:4;total:31651 | iSNV |
| F20 | F20-19 | 1911 | E | 0.4871 | A:14087;G:13393;C:13;T:1;total:27494 | iSNV |
| F20 | F20-19 | 4943 | NS3 | 0.4738 | A:15949;G:14369;C:5;T:1;total:30324 | iSNV |
| F20 | F20-19 | 5311 | NS3 | 0.1072 | A:2;G:20;C:2789;T:23182;total:25993 | iSNV |
| F20 | F20-19 | 6533 | NS4A | 0.0273 | A:3;G:0;C:717;T:25470;total:26190 | iSNV |
| F20 | F20-19 | 7495 | NS4B | 0.1103 | A:1;G:1;C:2413;T:19461;total:21876 | iSNV |
| F20 | F20-19 | 7546 | NS4B | 0.1037 | A:23660;G:2740;C:2;T:0;total:26402 | iSNV |
| F20 | F20-19 | 7656 | NS4B | 0.0241 | A:664;G:26780;C:0;T:0;total:27444 | iSNV |
| F20 | F20-19 | 7657 | NS4B | 0.7559 | A:6670;G:20645;C:1;T:1;total:27317 | iSNV |
| F20 | F20-19 | 8786 | NS5 | 0.0253 | A:3;G:0;C:663;T:25448;total:26114 | iSNV |
| F20 | F20-19 | 8900 | NS5 | 0.0346 | A:848;G:274;C:23364;T:8;total:24494 | iSNV |
| F20 | F20-19 | 9360 | NS5 | 0.4678 | A:16319;G:18558;C:0;T:1;total:34878 | iSNV |
| F20 | F20-19 | 9688 | NS5 | 0.6913 | A:4;G:1;C:10261;T:22970;total:33236 | iSNV |
| F20 | F20-19 | 10069 | NS5 | 0.0339 | A:2;G:0;C:24642;T:867;total:25511 | iSNV |
| F20 | F20-19 | 10085 | NS5 | 0.0296 | A:0;G:1;C:25120;T:768;total:25889 | iSNV |
| F20 | F20-19 | 10415 | 3'-UTR | 0.3811 | A:1;G:0;C:10194;T:16553;total:26748 | iSNV |

| | | | | | | |
|---|---|---|---|---|---|---|
| F20 | F20-19 | 10807 | 3'-UTR | 0.0278 | A:24769;G:710;C:5;T:13;total:25497 | iSNV |
| F20 | F20-2 | 1911 | E | 0.8273 | A:6392;G:30603;C:7;T:1;total:37003 | iSNV |
| F20 | F20-2 | 2961 | NS1 | 0.067 | A:54387;G:3910;C:3;T:3;total:58303 | iSNV |
| F20 | F20-2 | 3139 | NS1 | 0.0445 | A:54308;G:2531;C:1;T:1;total:56841 | iSNV |
| F20 | F20-2 | 3464 | NS1 | 0.0353 | A:1;G:0;C:36772;T:1347;total:38120 | iSNV |
| F20 | F20-2 | 3511 | NS1 | 0.2821 | A:11;G:2;C:8999;T:22886;total:31898 | iSNV |
| F20 | F20-2 | 5311 | NS3 | 0.1478 | A:1;G:47;C:6877;T:39584;total:46509 | iSNV |
| F20 | F20-2 | 7448 | NS4B | 0.271 | A:24428;G:9085;C:1;T:7;total:33521 | iSNV |
| F20 | F20-2 | 7495 | NS4B | 0.0924 | A:2;G:0;C:3178;T:31180;total:34360 | iSNV |
| F20 | F20-2 | 7575 | NS4B | 0.0279 | A:43614;G:1254;C:3;T:1;total:44872 | iSNV |
| F20 | F20-2 | 7656 | NS4B | 0.2039 | A:9967;G:37753;C:1138;T:2;total:48860 | iSNV |
| F20 | F20-2 | 7657 | NS4B | 0.436 | A:27463;G:21241;C:5;T:1;total:48710 | iSNV |
| F20 | F20-2 | 9053 | NS5 | 0.0253 | A:47593;G:1236;C:0;T:4;total:48833 | iSNV |
| F20 | F20-2 | 9688 | NS5 | 0.1251 | A:0;G:0;C:36448;T:5212;total:41660 | iSNV |
| F20 | F20-2 | 10069 | NS5 | 0.8273 | A:2;G:1;C:6413;T:30701;total:37117 | iSNV |
| F20 | F20-20 | 1116 | E | 0.0385 | A:36188;G:1453;C:0;T:3;total:37644 | iSNV |
| F20 | F20-20 | 1296 | E | 0.1555 | A:6;G:1;C:6169;T:33490;total:39666 | iSNV |
| F20 | F20-20 | 1733 | E | 0.0477 | A:17;G:1;C:2128;T:42423;total:44569 | iSNV |
| F20 | F20-20 | 1803 | E | 0.1469 | A:41001;G:7065;C:0;T:1;total:48067 | iSNV |
| F20 | F20-20 | 1813 | E | 0.0484 | A:44973;G:11;C:2293;T:6;total:47283 | iSNV |
| F20 | F20-20 | 1911 | E | 0.4197 | A:20643;G:15051;C:161;T:1;total:35856 | iSNV |
| F20 | F20-20 | 3492 | NS1 | 0.0592 | A:26629;G:0;C:1678;T:3;total:28310 | iSNV |
| F20 | F20-20 | 3998 | NS2A | 0.0218 | A:38050;G:852;C:1;T:2;total:38905 | iSNV |
| F20 | F20-20 | 4566 | NS2B | 0.1281 | A:18;G:36547;C:3;T:5375;total:41943 | iSNV |
| F20 | F20-20 | 5311 | NS3 | 0.1067 | A:4;G:110;C:3731;T:31103;total:34948 | iSNV |
| F20 | F20-20 | 5358 | NS3 | 0.1437 | A:4;G:31018;C:5210;T:1;total:36233 | iSNV |
| F20 | F20-20 | 5835 | NS3 | 0.023 | A:1007;G:42584;C:3;T:4;total:43598 | iSNV |
| F20 | F20-20 | 6533 | NS4A | 0.0395 | A:2;G:2;C:1378;T:33468;total:34850 | iSNV |
| F20 | F20-20 | 7495 | NS4B | 0.4052 | A:1;G:1;C:11397;T:16726;total:28125 | iSNV |
| F20 | F20-20 | 7656 | NS4B | 0.188 | A:6767;G:29208;C:1;T:1;total:35977 | iSNV |
| F20 | F20-20 | 7657 | NS4B | 0.1987 | A:28759;G:7134;C:3;T:1;total:35897 | iSNV |
| F20 | F20-20 | 8390 | NS5 | 0.0259 | A:1172;G:43931;C:0;T:2;total:45105 | iSNV |
| F20 | F20-20 | 9688 | NS5 | 0.4074 | A:1;G:1;C:25973;T:17861;total:43836 | iSNV |
| F20 | F20-20 | 9932 | NS5 | 0.0312 | A:1231;G:2;C:9;T:38127;total:39369 | iSNV |
| F20 | F20-20 | 10069 | NS5 | 0.2016 | A:0;G:0;C:26584;T:6713;total:33297 | iSNV |
| F20 | F20-20 | 10071 | NS5 | 0.1454 | A:4866;G:3;C:2;T:28594;total:33465 | iSNV |
| F20 | F20-20 | 10097 | NS5 | 0.0583 | A:1;G:33956;C:12;T:2105;total:36074 | iSNV |
| F20 | F20-20 | 10663 | 3'-UTR | 0.0263 | A:7;G:1225;C:45309;T:34;total:46575 | iSNV |
| F20 | F20-22 | 584 | M | 0.2977 | A:2;G:1;C:32315;T:13705;total:46023 | iSNV |
| F20 | F20-22 | 1298 | E | 0.0593 | A:2892;G:18;C:23;T:45810;total:48743 | iSNV |
| F20 | F20-22 | 1384 | E | 0.2549 | A:38474;G:7;C:14;T:13171;total:51666 | iSNV |
| F20 | F20-22 | 1390 | E | 0.0978 | A:45475;G:4967;C:313;T:3;total:50758 | iSNV |
| F20 | F20-22 | 1786 | E | 0.3011 | A:1;G:0;C:17033;T:39531;total:56565 | iSNV |
| F20 | F20-22 | 1911 | E | 0.2371 | A:33404;G:10415;C:107;T:0;total:43926 | iSNV |
| F20 | F20-22 | 2072 | E | 0.0214 | A:8;G:1;C:870;T:39726;total:40605 | iSNV |
| F20 | F20-22 | 2447 | E | 0.0247 | A:6;G:39526;C:1003;T:10;total:40545 | iSNV |
| F20 | F20-22 | 2843 | NS1 | 0.2901 | A:1;G:2;C:45761;T:18705;total:64469 | iSNV |
| F20 | F20-22 | 4501 | NS2B | 0.0304 | A:41469;G:1301;C:4;T:1;total:42775 | iSNV |
| F20 | F20-22 | 4640 | NS3 | 0.2933 | A:38308;G:15906;C:1;T:1;total:54216 | iSNV |
| F20 | F20-22 | 5210 | NS3 | 0.0633 | A:4;G:0;C:37240;T:2521;total:39765 | iSNV |
| F20 | F20-22 | 5311 | NS3 | 0.1049 | A:1;G:48;C:4340;T:36959;total:41348 | iSNV |
| F20 | F20-22 | 5626 | NS3 | 0.0222 | A:0;G:1;C:993;T:43573;total:44567 | iSNV |
| F20 | F20-22 | 5723 | NS3 | 0.0249 | A:46;G:2;C:1297;T:50724;total:52069 | iSNV |
| F20 | F20-22 | 6041 | NS3 | 0.0201 | A:22;G:1036;C:46;T:50220;total:51324 | iSNV |
| F20 | F20-22 | 6533 | NS4A | 0.3632 | A:1;G:0;C:14750;T:25857;total:40608 | iSNV |
| F20 | F20-22 | 6620 | NS4A | 0.0526 | A:37867;G:2103;C:2;T:2;total:39974 | iSNV |
| F20 | F20-22 | 7264 | NS4A | 0.0422 | A:1;G:0;C:35611;T:1572;total:37184 | iSNV |
| F20 | F20-22 | 7495 | NS4B | 0.0355 | A:1;G:1;C:1240;T:33658;total:34900 | iSNV |
| F20 | F20-22 | 7543 | NS4B | 0.0398 | A:40651;G:1685;C:0;T:0;total:42336 | iSNV |
| F20 | F20-22 | 7656 | NS4B | 0.2478 | A:10720;G:32513;C:11;T:1;total:43245 | iSNV |
| F20 | F20-22 | 7657 | NS4B | 0.4032 | A:25735;G:17403;C:20;T:1;total:43159 | iSNV |
| F20 | F20-22 | 8064 | NS5 | 0.0326 | A:1637;G:48509;C:5;T:6;total:50157 | iSNV |
| F20 | F20-22 | 8894 | NS5 | 0.1531 | A:5839;G:14;C:32124;T:150;total:38127 | iSNV |
| F20 | F20-22 | 9225 | NS5 | 0.2582 | A:14909;G:1;C:42806;T:14;total:57730 | iSNV |
| F20 | F20-22 | 9321 | NS5 | 0.02 | A:2;G:4;C:1096;T:53697;total:54799 | iSNV |
| F20 | F20-22 | 9688 | NS5 | 0.3963 | A:0;G:0;C:30544;T:20055;total:50599 | iSNV |
| F20 | F20-22 | 10014 | NS5 | 0.0546 | A:41858;G:6;C:2419;T:6;total:44289 | iSNV |
| F20 | F20-22 | 10069 | NS5 | 0.0239 | A:0;G:0;C:38711;T:950;total:39661 | iSNV |
| F20 | F20-23 | 1417 | E | 0.023 | A:0;G:0;C:34291;T:810;total:35101 | iSNV |
| F20 | F20-23 | 1803 | E | 0.0251 | A:38167;G:985;C:1;T:0;total:39153 | iSNV |
| F20 | F20-23 | 1911 | E | 0.8106 | A:5299;G:22662;C:3;T:0;total:27964 | iSNV |
| F20 | F20-23 | 3001 | NS1 | 0.0209 | A:569;G:26563;C:9;T:4;total:27145 | iSNV |
| F20 | F20-23 | 5311 | NS3 | 0.0295 | A:1;G:42;C:822;T:26965;total:27830 | iSNV |
| F20 | F20-23 | 5358 | NS3 | 0.0224 | A:0;G:28305;C:651;T:2;total:28958 | iSNV |
| F20 | F20-23 | 5656 | NS3 | 0.0272 | A:29468;G:825;C:7;T:1;total:30301 | iSNV |
| F20 | F20-23 | 5835 | NS3 | 0.0243 | A:844;G:33772;C:2;T:0;total:34618 | iSNV |
| F20 | F20-23 | 7193 | NS4A | 0.725 | A:0;G:0;C:18557;T:7039;total:25596 | iSNV |
| F20 | F20-23 | 7494 | NS4B | 0.0233 | A:536;G:22405;C:0;T:9;total:22950 | iSNV |
| F20 | F20-23 | 7495 | NS4B | 0.0467 | A:0;G:0;C:1111;T:22658;total:23769 | iSNV |
| F20 | F20-23 | 7547 | NS4B | 0.7255 | A:4;G:20526;C:2;T:7771;total:28303 | iSNV |
| F20 | F20-23 | 7656 | NS4B | 0.0201 | A:594;G:28909;C:2;T:0;total:29505 | iSNV |
| F20 | F20-23 | 7657 | NS4B | 0.075 | A:27181;G:2206;C:5;T:1;total:29393 | iSNV |

| | | | | | | |
|---|---|---|---|---|---|---|
| F20 | F20-23 | 8396 | NS5 | 0.0265 | A:33623;G:918;C:1;T:1;total:34543 | iSNV |
| F20 | F20-23 | 9521 | NS5 | 0.0217 | A:2;G:1;C:37396;T:833;total:38232 | iSNV |
| F20 | F20-23 | 10069 | NS5 | 0.026 | A:0;G:0;C:26104;T:699;total:26803 | iSNV |
| F20 | F20-23 | 10071 | NS5 | 0.0238 | A:644;G:1;C:0;T:26396;total:27041 | iSNV |
| F20 | F20-23 | 10092 | NS5 | 0.7955 | A:21660;G:5569;C:0;T:3;total:27232 | iSNV |
| F20 | F20-23 | 10325 | NS5 | 0.0509 | A:1;G:2;C:1868;T:34786;total:36657 | iSNV |
| F20 | F20-23 | 10451 | 3'-UTR | 0.0204 | A:0;G:0;C:26653;T:557;total:27210 | iSNV |
| F20 | F20-25 | 290 | C | 0.023 | A:1;G:4;C:744;T:31533;total:32282 | iSNV |
| F20 | F20-25 | 340 | C | 0.4111 | A:7;G:2;C:18876;T:13185;total:32070 | iSNV |
| F20 | F20-25 | 656 | M | 0.1231 | A:1;G:2;C:3307;T:23533;total:26843 | iSNV |
| F20 | F20-25 | 1911 | E | 0.7527 | A:6610;G:20092;C:18;T:0;total:26720 | iSNV |
| F20 | F20-25 | 2141 | E | 0.1024 | A:23744;G:2710;C:2;T:0;total:26456 | iSNV |
| F20 | F20-25 | 2873 | NS1 | 0.2308 | A:31801;G:7;C:11;T:9549;total:41368 | iSNV |
| F20 | F20-25 | 2882 | NS1 | 0.0324 | A:39106;G:1311;C:0;T:3;total:40420 | iSNV |
| F20 | F20-25 | 4493 | NS2B | 0.0227 | A:0;G:0;C:605;T:26037;total:26642 | iSNV |
| F20 | F20-25 | 5311 | NS3 | 0.3266 | A:0;G:36;C:8476;T:17436;total:25948 | iSNV |
| F20 | F20-25 | 5835 | NS3 | 0.084 | A:2782;G:30311;C:2;T:0;total:33095 | iSNV |
| F20 | F20-25 | 6861 | NS4A | 0.0415 | A:0;G:0;C:24377;T:1058;total:25435 | iSNV |
| F20 | F20-25 | 7223 | NS4A | 0.0321 | A:0;G:1;C:793;T:23858;total:24652 | iSNV |
| F20 | F20-25 | 7488 | NS4B | 0.0252 | A:20822;G:540;C:0;T:7;total:21369 | iSNV |
| F20 | F20-25 | 7495 | NS4B | 0.1743 | A:2;G:0;C:3812;T:18051;total:21865 | iSNV |
| F20 | F20-25 | 7543 | NS4B | 0.0549 | A:25505;G:1482;C:0;T:1;total:26988 | iSNV |
| F20 | F20-25 | 7575 | NS4B | 0.0349 | A:26378;G:954;C:0;T:0;total:27332 | iSNV |
| F20 | F20-25 | 7656 | NS4B | 0.1996 | A:5457;G:21790;C:87;T:0;total:27334 | iSNV |
| F20 | F20-25 | 7657 | NS4B | 0.1765 | A:22471;G:4819;C:3;T:0;total:27293 | iSNV |
| F20 | F20-25 | 8174 | NS5 | 0.027 | A:857;G:30774;C:0;T:2;total:31633 | iSNV |
| F20 | F20-25 | 9221 | NS5 | 0.1011 | A:33247;G:3;C:10;T:3744;total:37004 | iSNV |
| F20 | F20-25 | 9646 | NS5 | 0.0247 | A:29395;G:747;C:0;T:5;total:30147 | iSNV |
| F20 | F20-25 | 9688 | NS5 | 0.0326 | A:0;G:0;C:30830;T:1042;total:31872 | iSNV |
| F20 | F20-25 | 10069 | NS5 | 0.6901 | A:0;G:1;C:7581;T:16878;total:24460 | iSNV |
| F20 | F20-25 | 10092 | NS5 | 0.0307 | A:808;G:25336;C:107;T:1;total:26252 | iSNV |
| F20 | F20-26 | 1058 | E | 0.036 | A:27735;G:1038;C:0;T:2;total:28775 | iSNV |
| F20 | F20-26 | 1296 | E | 0.0264 | A:0;G:0;C:923;T:34038;total:34961 | iSNV |
| F20 | F20-26 | 1911 | E | 0.8935 | A:2975;G:24947;C:0;T:0;total:27922 | iSNV |
| F20 | F20-26 | 4070 | NS2A | 0.0243 | A:1;G:651;C:14;T:26044;total:26710 | iSNV |
| F20 | F20-26 | 4380 | NS2B | 0.0247 | A:746;G:4;C:29380;T:22;total:30152 | iSNV |
| F20 | F20-26 | 5311 | NS3 | 0.0346 | A:0;G:78;C:1024;T:28480;total:29582 | iSNV |
| F20 | F20-26 | 6329 | NS3 | 0.0259 | A:792;G:29747;C:0;T:0;total:30539 | iSNV |
| F20 | F20-26 | 6533 | NS4A | 0.0216 | A:1;G:0;C:650;T:29403;total:30054 | iSNV |
| F20 | F20-26 | 6854 | NS4A | 0.0224 | A:0;G:0;C:700;T:30426;total:31126 | iSNV |
| F20 | F20-26 | 7104 | NS4A | 0.025 | A:792;G:30803;C:1;T:3;total:31599 | iSNV |
| F20 | F20-26 | 7179 | NS4A | 0.1141 | A:3361;G:26077;C:0;T:2;total:29440 | iSNV |
| F20 | F20-26 | 7494 | NS4B | 0.0297 | A:22;G:23518;C:3;T:723;total:24266 | iSNV |
| F20 | F20-26 | 7495 | NS4B | 0.0579 | A:2;G:0;C:1453;T:23616;total:25071 | iSNV |
| F20 | F20-26 | 7656 | NS4B | 0.0323 | A:1017;G:30381;C:23;T:0;total:31421 | iSNV |
| F20 | F20-26 | 7657 | NS4B | 0.7533 | A:7728;G:23535;C:58;T:1;total:31322 | iSNV |
| F20 | F20-26 | 8987 | NS5 | 0.0245 | A:842;G:3;C:2;T:33432;total:34279 | iSNV |
| F20 | F20-26 | 9358 | NS5 | 0.5757 | A:23912;G:17630;C:0;T:2;total:41544 | iSNV |
| F20 | F20-26 | 9688 | NS5 | 0.0401 | A:1;G:1;C:33980;T:1420;total:35402 | iSNV |
| F20 | F20-26 | 10069 | NS5 | 0.8938 | A:0;G:1;C:2993;T:25172;total:28166 | iSNV |
| F20 | F20-26 | 10416 | 3'-UTR | 0.0297 | A:30561;G:936;C:2;T:1;total:31500 | iSNV |
| F20 | F20-28 | 235 | C | 0.063 | A:0;G:0;C:2144;T:31860;total:34004 | iSNV |
| F20 | F20-28 | 650 | M | 0.0298 | A:899;G:29180;C:1;T:0;total:30080 | iSNV |
| F20 | F20-28 | 836 | M | 0.0329 | A:4;G:0;C:832;T:24425;total:25261 | iSNV |
| F20 | F20-28 | 902 | M | 0.0882 | A:0;G:0;C:23863;T:2311;total:26174 | iSNV |
| F20 | F20-28 | 982 | E | 0.2412 | A:21201;G:3;C:6743;T:2;total:27949 | iSNV |
| F20 | F20-28 | 1417 | E | 0.0476 | A:1;G:0;C:32333;T:1617;total:33951 | iSNV |
| F20 | F20-28 | 1450 | E | 0.0352 | A:33730;G:22;C:1235;T:2;total:34989 | iSNV |
| F20 | F20-28 | 1468 | E | 0.0642 | A:2152;G:0;C:31337;T:8;total:33497 | iSNV |
| F20 | F20-28 | 1786 | E | 0.037 | A:1;G:1;C:1377;T:35743;total:37122 | iSNV |
| F20 | F20-28 | 1911 | E | 0.483 | A:14589;G:13643;C:11;T:1;total:28244 | iSNV |
| F20 | F20-28 | 2987 | NS1 | 0.0442 | A:30323;G:1403;C:3;T:0;total:31729 | iSNV |
| F20 | F20-28 | 3768 | NS2A | 0.0223 | A:0;G:6;C:609;T:26637;total:27252 | iSNV |
| F20 | F20-28 | 4177 | NS2A | 0.0244 | A:3;G:0;C:16886;T:424;total:17313 | iSNV |
| F20 | F20-28 | 5311 | NS3 | 0.2561 | A:0;G:36;C:6845;T:19838;total:26719 | iSNV |
| F20 | F20-28 | 6533 | NS4A | 0.0544 | A:2;G:2;C:1477;T:25624;total:27105 | iSNV |
| F20 | F20-28 | 7265 | NS4A | 0.2329 | A:5472;G:18013;C:0;T:0;total:23485 | iSNV |
| F20 | F20-28 | 7494 | NS4B | 0.0278 | A:12;G:21322;C:0;T:612;total:21946 | iSNV |
| F20 | F20-28 | 7495 | NS4B | 0.2968 | A:1;G:0;C:6618;T:15677;total:22296 | iSNV |
| F20 | F20-28 | 7656 | NS4B | 0.0699 | A:2010;G:26665;C:60;T:3;total:28738 | iSNV |
| F20 | F20-28 | 7657 | NS4B | 0.4504 | A:15744;G:12908;C:2;T:1;total:28655 | iSNV |
| F20 | F20-28 | 8205 | NS5 | 0.0311 | A:29366;G:945;C:4;T:1;total:30316 | iSNV |
| F20 | F20-28 | 8410 | NS5 | 0.0413 | A:33110;G:1427;C:1;T:3;total:34541 | iSNV |
| F20 | F20-28 | 8456 | NS5 | 0.2364 | A:28275;G:6;C:7;T:8761;total:37049 | iSNV |
| F20 | F20-28 | 8900 | NS5 | 0.0216 | A:538;G:0;C:24329;T:7;total:24874 | iSNV |
| F20 | F20-28 | 8987 | NS5 | 0.0242 | A:720;G:1;C:12;T:28924;total:29657 | iSNV |
| F20 | F20-28 | 9688 | NS5 | 0.141 | A:2;G:0;C:28162;T:4623;total:32787 | iSNV |
| F20 | F20-28 | 10069 | NS5 | 0.0841 | A:0;G:1;C:24259;T:2230;total:26490 | iSNV |
| F20 | F20-28 | 10071 | NS5 | 0.0302 | A:808;G:0;C:2;T:25938;total:26748 | iSNV |
| F20 | F20-28 | 10092 | NS5 | 0.271 | A:7391;G:19853;C:27;T:0;total:27271 | iSNV |
| F20 | F20-28 | 10095 | NS5 | 0.0415 | A:0;G:27040;C:1171;T:2;total:28213 | iSNV |
| F20 | F20-30 | 697 | M | 0.0239 | A:0;G:4;C:181;T:7375;total:7560 | iSNV |

| | | | | | | |
|---|---|---|---|---|---|---|
| F20 | F20-30 | 785 | M | 0.0419 | A:6941;G:304;C:0;T:0;total:7245 | iSNV |
| F20 | F20-30 | 1116 | E | 0.0282 | A:6566;G:191;C:0;T:0;total:6757 | iSNV |
| F20 | F20-30 | 1461 | E | 0.0442 | A:0;G:1;C:297;T:6415;total:6713 | iSNV |
| F20 | F20-30 | 1911 | E | 0.6252 | A:1946;G:3240;C:5;T:0;total:5191 | iSNV |
| F20 | F20-30 | 2129 | E | 0.8475 | A:1032;G:5731;C:0;T:1;total:6764 | iSNV |
| F20 | F20-30 | 3230 | NS1 | 0.8205 | A:1663;G:7600;C:0;T:1;total:9264 | iSNV |
| F20 | F20-30 | 3633 | NS1 | 0.0369 | A:6698;G:257;C:1;T:0;total:6956 | iSNV |
| F20 | F20-30 | 3975 | NS2A | 0.0395 | A:1;G:318;C:4;T:7712;total:8035 | iSNV |
| F20 | F20-30 | 6533 | NS4A | 0.8591 | A:0;G:0;C:4838;T:794;total:5632 | iSNV |
| F20 | F20-30 | 6644 | NS4A | 0.0554 | A:0;G:0;C:273;T:4651;total:4924 | iSNV |
| F20 | F20-30 | 7373 | NS4B | 0.0214 | A:70;G:5855;C:1;T:130;total:6056 | iSNV |
| F20 | F20-30 | 7495 | NS4B | 0.053 | A:1;G:1;C:272;T:4852;total:5126 | iSNV |
| F20 | F20-30 | 7657 | NS4B | 0.8684 | A:1057;G:6969;C:1;T:1;total:8028 | iSNV |
| F20 | F20-30 | 9688 | NS5 | 0.8919 | A:0;G:0;C:598;T:4933;total:5531 | iSNV |
| F20 | F20-30 | 9932 | NS5 | 0.0435 | A:274;G:0;C:0;T:6013;total:6287 | iSNV |
| F20 | F20-30 | 10086 | NS5 | 0.0401 | A:238;G:1;C:0;T:5686;total:5925 | iSNV |
| F20 | F20-4 | 1786 | E | 0.0563 | A:12;G:3;C:4317;T:72291;total:76623 | iSNV |
| F20 | F20-4 | 1911 | E | 0.6221 | A:18668;G:30710;C:12;T:0;total:49390 | iSNV |
| F20 | F20-4 | 1914 | E | 0.032 | A:47113;G:1558;C:3;T:0;total:48674 | iSNV |
| F20 | F20-4 | 4021 | NS2A | 0.1315 | A:8031;G:52992;C:1;T:10;total:61034 | iSNV |
| F20 | F20-4 | 4250 | NS2B | 0.0331 | A:41447;G:1421;C:1;T:2;total:42871 | iSNV |
| F20 | F20-4 | 5311 | NS3 | 0.0439 | A:2;G:71;C:2816;T:61147;total:64036 | iSNV |
| F20 | F20-4 | 6533 | NS4A | 0.7153 | A:21;G:1;C:36949;T:14718;total:51689 | iSNV |
| F20 | F20-4 | 7495 | NS4B | 0.0259 | A:3;G:4;C:1220;T:45708;total:46935 | iSNV |
| F20 | F20-4 | 7546 | NS4B | 0.1343 | A:52234;G:8107;C:9;T:4;total:60354 | iSNV |
| F20 | F20-4 | 7656 | NS4B | 0.0343 | A:2401;G:67428;C:19;T:3;total:69851 | iSNV |
| F20 | F20-4 | 7657 | NS4B | 0.8365 | A:11392;G:58267;C:3;T:1;total:69663 | iSNV |
| F20 | F20-4 | 8140 | NS5 | 0.0473 | A:80080;G:117;C:3986;T:1;total:84184 | iSNV |
| F20 | F20-4 | 9220 | NS5 | 0.0242 | A:87292;G:4;C:2174;T:2;total:89472 | iSNV |
| F20 | F20-4 | 9688 | NS5 | 0.7831 | A:0;G:1;C:11014;T:39749;total:50764 | iSNV |
| F20 | F20-4 | 9932 | NS5 | 0.0203 | A:1065;G:4;C:9;T:51204;total:52282 | iSNV |
| F20 | F20-4 | 10069 | NS5 | 0.2134 | A:0;G:0;C:38244;T:10377;total:48621 | iSNV |
| F20 | F20-4 | 10092 | NS5 | 0.0721 | A:3758;G:48341;C:5;T:1;total:52105 | iSNV |
| F20 | F20-4 | 10632 | 3'-UTR | 0.2107 | A:13;G:25;C:12786;T:47843;total:60667 | iSNV |
| F20 | F20-5 | 1911 | E | 0.9165 | A:3072;G:33675;C:2;T:1;total:36750 | iSNV |
| F20 | F20-5 | 1946 | E | 0.0299 | A:0;G:0;C:37442;T:1155;total:38597 | iSNV |
| F20 | F20-5 | 3081 | NS1 | 0.1528 | A:39635;G:7151;C:2;T:2;total:46790 | iSNV |
| F20 | F20-5 | 4853 | NS3 | 0.0333 | A:52471;G:1810;C:1;T:9;total:54291 | iSNV |
| F20 | F20-5 | 5311 | NS3 | 0.5807 | A:2;G:23;C:24344;T:17600;total:41969 | iSNV |
| F20 | F20-5 | 6523 | NS4A | 0.0476 | A:0;G:0;C:40216;T:2012;total:42228 | iSNV |
| F20 | F20-5 | 6637 | NS4A | 0.2928 | A:2;G:1;C:9941;T:24003;total:33947 | iSNV |
| F20 | F20-5 | 7656 | NS4B | 0.5891 | A:1864;G:18311;C:24371;T:9;total:44555 | iSNV |
| F20 | F20-5 | 7657 | NS4B | 0.3023 | A:31142;G:13500;C:2;T:1;total:44645 | iSNV |
| F20 | F20-5 | 9688 | NS5 | 0.3405 | A:0;G:0;C:26928;T:13904;total:40832 | iSNV |
| F20 | F20-5 | 9887 | NS5 | 0.0744 | A:0;G:1;C:3221;T:40056;total:43278 | iSNV |
| F20 | F20-5 | 10092 | NS5 | 0.0277 | A:329;G:36417;C:1048;T:2;total:37796 | iSNV |
| F20 | F20-7 | 697 | M | 0.0224 | A:2;G:0;C:1273;T:55317;total:56592 | iSNV |
| F20 | F20-7 | 1116 | E | 0.0502 | A:49166;G:2602;C:0;T:5;total:51773 | iSNV |
| F20 | F20-7 | 1911 | E | 0.5142 | A:21119;G:22208;C:143;T:1;total:43471 | iSNV |
| F20 | F20-7 | 1913 | E | 0.0276 | A:41252;G:3;C:18;T:1175;total:42448 | iSNV |
| F20 | F20-7 | 3139 | NS1 | 0.7843 | A:13507;G:49086;C:0;T:2;total:62595 | iSNV |
| F20 | F20-7 | 5311 | NS3 | 0.144 | A:4;G:67;C:7192;T:42661;total:49924 | iSNV |
| F20 | F20-7 | 5336 | NS3 | 0.0394 | A:2;G:0;C:1957;T:47697;total:49656 | iSNV |
| F20 | F20-7 | 5835 | NS3 | 0.0214 | A:1462;G:66781;C:1;T:1;total:68245 | iSNV |
| F20 | F20-7 | 6533 | NS4A | 0.1879 | A:2;G:3;C:8606;T:37177;total:45788 | iSNV |
| F20 | F20-7 | 7495 | NS4B | 0.7771 | A:0;G:1;C:26335;T:7558;total:33894 | iSNV |
| F20 | F20-7 | 7543 | NS4B | 0.1543 | A:37727;G:6886;C:1;T:0;total:44614 | iSNV |
| F20 | F20-7 | 7657 | NS4B | 0.0437 | A:50741;G:2320;C:0;T:0;total:53061 | iSNV |
| F20 | F20-7 | 8140 | NS5 | 0.0398 | A:65654;G:2722;C:1;T:4;total:68381 | iSNV |
| F20 | F20-7 | 9370 | NS5 | 0.0362 | A:2523;G:17;C:26;T:67113;total:69679 | iSNV |
| F20 | F20-7 | 9688 | NS5 | 0.9807 | A:3;G:0;C:897;T:45501;total:46401 | SNP |
| F20 | F20-7 | 10069 | NS5 | 0.0891 | A:1;G:0;C:37707;T:3690;total:41398 | iSNV |
| F20 | F20-7 | 10379 | NS5 | 0.1324 | A:44194;G:6749;C:3;T:1;total:50947 | iSNV |
| F20 | F20-8 | 494 | M | 0.0224 | A:9;G:1;C:66435;T:1526;total:67971 | iSNV |
| F20 | F20-8 | 798 | M | 0.3153 | A:32248;G:10;C:14861;T:4;total:47123 | iSNV |
| F20 | F20-8 | 1417 | E | 0.0264 | A:0;G:2;C:59989;T:1629;total:61620 | iSNV |
| F20 | F20-8 | 1911 | E | 0.3223 | A:26665;G:15018;C:4899;T:4;total:46586 | iSNV |
| F20 | F20-8 | 3538 | NS1 | 0.2752 | A:31424;G:3;C:11937;T:7;total:43371 | iSNV |
| F20 | F20-8 | 4401 | NS2B | 0.2626 | A:4;G:37743;C:13448;T:6;total:51201 | iSNV |
| F20 | F20-8 | 5311 | NS3 | 0.037 | A:0;G:45;C:1992;T:51763;total:53800 | iSNV |
| F20 | F20-8 | 5528 | NS3 | 0.2701 | A:44322;G:16410;C:0;T:13;total:60745 | iSNV |
| F20 | F20-8 | 5835 | NS3 | 0.3089 | A:22012;G:49236;C:5;T:2;total:71255 | iSNV |
| F20 | F20-8 | 6533 | NS4A | 0.5782 | A:0;G:0;C:27131;T:19799;total:46930 | iSNV |
| F20 | F20-8 | 7495 | NS4B | 0.0438 | A:4;G:0;C:1766;T:38526;total:40296 | iSNV |
| F20 | F20-8 | 7657 | NS4B | 0.9026 | A:5418;G:50158;C:2;T:2;total:55580 | iSNV |
| F20 | F20-8 | 8534 | NS5 | 0.2564 | A:15;G:3;C:19236;T:55752;total:75006 | iSNV |
| F20 | F20-8 | 9688 | NS5 | 0.6026 | A:1;G:0;C:19546;T:29634;total:49181 | iSNV |
| F20 | F20-8 | 9932 | NS5 | 0.02 | A:974;G:2;C:64;T:47425;total:48465 | iSNV |
| F20 | F20-8 | 10419 | 3'-UTR | 0.5649 | A:3;G:2;C:21580;T:28012;total:49597 | iSNV |
| F20 | F20-8 | 10814 | 3'-UTR | 0.0301 | A:0;G:43642;C:3;T:1356;total:45001 | iSNV |
| F25 | F25-1 | 1116 | E | 0.2102 | A:44786;G:11922;C:3;T:2;total:56713 | iSNV |
| F25 | F25-1 | 1296 | E | 0.0609 | A:5;G:0;C:3482;T:53636;total:57123 | iSNV |

| | | | | | | |
|---|---|---|---|---|---|---|
| F25 | F25-1 | 1417 | E | 0.022 | A:1;G:0;C:65611;T:1476;total:67088 | iSNV |
| F25 | F25-1 | 1482 | E | 0.0524 | A:2975;G:53710;C:3;T:6;total:56694 | iSNV |
| F25 | F25-1 | 1803 | E | 0.1394 | A:60604;G:9818;C:1;T:2;total:70425 | iSNV |
| F25 | F25-1 | 1911 | E | 0.5181 | A:23111;G:24765;C:69;T:4;total:47949 | iSNV |
| F25 | F25-1 | 2298 | E | 0.0221 | A:1029;G:45328;C:2;T:5;total:46364 | iSNV |
| F25 | F25-1 | 3528 | NS1 | 0.0442 | A:2;G:4;C:40233;T:1861;total:42100 | iSNV |
| F25 | F25-1 | 3814 | NS2A | 0.0354 | A:11;G:2;C:1870;T:50885;total:52768 | iSNV |
| F25 | F25-1 | 3993 | NS2A | 0.1021 | A:5773;G:50734;C:8;T:3;total:56518 | iSNV |
| F25 | F25-1 | 4233 | NS2B | 0.0317 | A:4;G:1164;C:2;T:35485;total:36655 | iSNV |
| F25 | F25-1 | 5311 | NS3 | 0.7451 | A:1;G:68;C:38638;T:13243;total:51950 | iSNV |
| F25 | F25-1 | 5358 | NS3 | 0.1445 | A:2;G:48132;C:8135;T:4;total:56273 | iSNV |
| F25 | F25-1 | 6707 | NS4A | 0.0439 | A:2067;G:1;C:11;T:44998;total:47077 | iSNV |
| F25 | F25-1 | 7179 | NS4A | 0.0365 | A:1736;G:45689;C:106;T:5;total:47536 | iSNV |
| F25 | F25-1 | 7495 | NS4B | 0.0533 | A:5;G:3;C:2462;T:43665;total:46135 | iSNV |
| F25 | F25-1 | 7526 | NS4B | 0.1745 | A:9246;G:6;C:96;T:43622;total:52970 | iSNV |
| F25 | F25-1 | 7575 | NS4B | 0.0216 | A:55988;G:1239;C:1;T:0;total:57228 | iSNV |
| F25 | F25-1 | 7626 | NS4B | 0.7766 | A:40;G:48141;C:2;T:13864;total:62047 | iSNV |
| F25 | F25-1 | 7657 | NS4B | 0.0384 | A:55575;G:2225;C:2;T:2;total:57804 | iSNV |
| F25 | F25-1 | 9688 | NS5 | 0.1399 | A:0;G:4;C:48876;T:7957;total:56837 | iSNV |
| F25 | F25-1 | 10071 | NS5 | 0.138 | A:7032;G:1;C:10;T:43885;total:50928 | iSNV |
| F25 | F25-1 | 10086 | NS5 | 0.0232 | A:1200;G:0;C:4;T:50335;total:51539 | iSNV |
| F25 | F25-1 | 10092 | NS5 | 0.0609 | A:3187;G:49099;C:1;T:3;total:52290 | iSNV |
| F25 | F25-1 | 10434 | 3'-UTR | 0.022 | A:58395;G:1314;C:5;T:3;total:59717 | iSNV |
| F25 | F25-10 | 400 | C | 0.0258 | A:57719;G:1529;C:0;T:6;total:59254 | iSNV |
| F25 | F25-10 | 820 | M | 0.025 | A:896;G:25;C:34765;T:19;total:35705 | iSNV |
| F25 | F25-10 | 821 | M | 0.0263 | A:24;G:34462;C:933;T:5;total:35424 | iSNV |
| F25 | F25-10 | 1116 | E | 0.044 | A:43163;G:1987;C:0;T:0;total:45150 | iSNV |
| F25 | F25-10 | 1247 | E | 0.1743 | A:0;G:1;C:8073;T:38224;total:46298 | iSNV |
| F25 | F25-10 | 1296 | E | 0.0357 | A:0;G:0;C:1703;T:45971;total:47674 | iSNV |
| F25 | F25-10 | 1342 | E | 0.0262 | A:1;G:1;C:49447;T:1333;total:50782 | iSNV |
| F25 | F25-10 | 1347 | E | 0.0296 | A:48477;G:1481;C:0;T:1;total:49959 | iSNV |
| F25 | F25-10 | 1359 | E | 0.024 | A:49274;G:1216;C:1;T:0;total:50491 | iSNV |
| F25 | F25-10 | 1390 | E | 0.0286 | A:48825;G:12;C:1438;T:3;total:50278 | iSNV |
| F25 | F25-10 | 1406 | E | 0.0499 | A:2730;G:51928;C:3;T:9;total:54670 | iSNV |
| F25 | F25-10 | 1474 | E | 0.0505 | A:47050;G:9;C:32;T:2509;total:49600 | iSNV |
| F25 | F25-10 | 1708 | E | 0.0327 | A:1692;G:49942;C:0;T:4;total:51638 | iSNV |
| F25 | F25-10 | 1911 | E | 0.4315 | A:21192;G:16093;C:6;T:0;total:37291 | iSNV |
| F25 | F25-10 | 2226 | E | 0.0481 | A:2;G:1;C:40333;T:2039;total:42375 | iSNV |
| F25 | F25-10 | 2364 | E | 0.0261 | A:1;G:0;C:32951;T:886;total:33838 | iSNV |
| F25 | F25-10 | 2924 | NS1 | 0.0326 | A:0;G:2;C:1893;T:56083;total:57978 | iSNV |
| F25 | F25-10 | 3113 | NS1 | 0.197 | A:21;G:3;C:9374;T:38166;total:47564 | iSNV |
| F25 | F25-10 | 4021 | NS2A | 0.028 | A:1267;G:43851;C:0;T:5;total:45123 | iSNV |
| F25 | F25-10 | 4985 | NS3 | 0.0286 | A:2;G:4;C:1558;T:52814;total:54378 | iSNV |
| F25 | F25-10 | 5311 | NS3 | 0.2782 | A:0;G:87;C:11456;T:29634;total:41177 | iSNV |
| F25 | F25-10 | 6241 | NS3 | 0.0205 | A:3;G:0;C:1039;T:49397;total:50439 | iSNV |
| F25 | F25-10 | 6368 | NS3 | 0.3311 | A:7;G:13106;C:4;T:26460;total:39577 | iSNV |
| F25 | F25-10 | 6533 | NS4A | 0.025 | A:3;G:2;C:998;T:38769;total:39772 | iSNV |
| F25 | F25-10 | 6707 | NS4A | 0.0249 | A:4;G:2;C:959;T:37469;total:38434 | iSNV |
| F25 | F25-10 | 6922 | NS4A | 0.02 | A:821;G:40147;C:0;T:3;total:40971 | iSNV |
| F25 | F25-10 | 7373 | NS4B | 0.3329 | A:47;G:26238;C:13125;T:8;total:39418 | iSNV |
| F25 | F25-10 | 7495 | NS4B | 0.0878 | A:2;G:1;C:3140;T:32610;total:35753 | iSNV |
| F25 | F25-10 | 7656 | NS4B | 0.1935 | A:8598;G:35778;C:46;T:0;total:44422 | iSNV |
| F25 | F25-10 | 7657 | NS4B | 0.6397 | A:15993;G:28394;C:0;T:1;total:44388 | iSNV |
| F25 | F25-10 | 8140 | NS5 | 0.0328 | A:57270;G:542;C:1966;T:8;total:59786 | iSNV |
| F25 | F25-10 | 8360 | NS5 | 0.0245 | A:3;G:3;C:1480;T:58888;total:60374 | iSNV |
| F25 | F25-10 | 9246 | NS5 | 0.0242 | A:62448;G:1551;C:1;T:0;total:64000 | iSNV |
| F25 | F25-10 | 9688 | NS5 | 0.0552 | A:1;G:0;C:45886;T:2683;total:48570 | iSNV |
| F25 | F25-10 | 10043 | NS5 | 0.03 | A:45992;G:1424;C:0;T:2;total:47418 | iSNV |
| F25 | F25-10 | 10068 | NS5 | 0.0306 | A:41297;G:1304;C:0;T:1;total:42602 | iSNV |
| F25 | F25-10 | 10069 | NS5 | 0.36 | A:0;G:0;C:26922;T:15144;total:42066 | iSNV |
| F25 | F25-10 | 10111 | NS5 | 0.317 | A:33058;G:15351;C:0;T:6;total:48415 | iSNV |
| F25 | F25-10 | 10308 | NS5 | 0.0345 | A:2089;G:58333;C:3;T:1;total:60426 | iSNV |
| F25 | F25-10 | 10493 | 3'-UTR | 0.0276 | A:3;G:1210;C:16;T:42495;total:43724 | iSNV |
| F25 | F25-11 | 221 | C | 0.0873 | A:5845;G:61078;C:3;T:1;total:66927 | iSNV |
| F25 | F25-11 | 1417 | E | 0.027 | A:3;G:0;C:66164;T:1838;total:68005 | iSNV |
| F25 | F25-11 | 1468 | E | 0.0287 | A:1937;G:0;C:65333;T:23;total:67293 | iSNV |
| F25 | F25-11 | 1803 | E | 0.1831 | A:59405;G:13325;C:9;T:3;total:72742 | iSNV |
| F25 | F25-11 | 1911 | E | 0.3606 | A:32458;G:18308;C:1;T:3;total:50770 | iSNV |
| F25 | F25-11 | 1913 | E | 0.4381 | A:27491;G:3;C:21466;T:37;total:48997 | iSNV |
| F25 | F25-11 | 2306 | E | 0.0259 | A:49550;G:1321;C:5;T:1;total:50877 | iSNV |
| F25 | F25-11 | 3242 | NS1 | 0.0299 | A:5;G:3;C:2283;T:73820;total:76111 | iSNV |
| F25 | F25-11 | 3989 | NS2A | 0.0294 | A:1874;G:61701;C:12;T:36;total:63623 | iSNV |
| F25 | F25-11 | 5311 | NS3 | 0.2048 | A:1;G:323;C:10990;T:42328;total:53642 | iSNV |
| F25 | F25-11 | 5353 | NS3 | 0.0317 | A:55833;G:1829;C:1;T:4;total:57667 | iSNV |
| F25 | F25-11 | 5358 | NS3 | 0.1858 | A:3;G:46382;C:10587;T:0;total:56972 | iSNV |
| F25 | F25-11 | 6523 | NS4A | 0.4689 | A:0;G:0;C:28792;T:25430;total:54222 | iSNV |
| F25 | F25-11 | 6533 | NS4A | 0.0232 | A:0;G:3;C:1197;T:50214;total:51414 | iSNV |
| F25 | F25-11 | 7495 | NS4B | 0.0912 | A:5;G:1;C:4195;T:41749;total:45950 | iSNV |
| F25 | F25-11 | 7536 | NS4B | 0.0244 | A:1301;G:3;C:23;T:51891;total:53218 | iSNV |
| F25 | F25-11 | 7557 | NS4B | 0.0258 | A:1544;G:58243;C:0;T:3;total:59790 | iSNV |
| F25 | F25-11 | 7575 | NS4B | 0.0873 | A:50656;G:4852;C:3;T:4;total:55515 | iSNV |
| F25 | F25-11 | 7629 | NS4B | 0.1339 | A:54424;G:7;C:8419;T:4;total:62854 | iSNV |

| | | | | | | |
|---|---|---|---|---|---|---|
| F25 | F25-11 | 7656 | NS4B | 0.144 | A:8344;G:49499;C:86;T:1;total:57930 | iSNV |
| F25 | F25-11 | 7657 | NS4B | 0.0856 | A:52805;G:4949;C:0;T:2;total:57756 | iSNV |
| F25 | F25-11 | 7919 | NS5 | 0.0241 | A:1848;G:74636;C:10;T:5;total:76499 | iSNV |
| F25 | F25-11 | 8398 | NS5 | 0.0551 | A:4081;G:3;C:8;T:69953;total:74045 | iSNV |
| F25 | F25-11 | 8900 | NS5 | 0.0367 | A:2071;G:5;C:54222;T:8;total:56306 | iSNV |
| F25 | F25-11 | 9170 | NS5 | 0.0836 | A:4;G:1;C:6597;T:72282;total:78884 | iSNV |
| F25 | F25-11 | 9512 | NS5 | 0.0222 | A:4;G:1;C:1829;T:80544;total:82378 | iSNV |
| F25 | F25-11 | 9607 | NS5 | 0.4659 | A:38481;G:1;C:33586;T:8;total:72076 | iSNV |
| F25 | F25-11 | 9688 | NS5 | 0.0386 | A:3;G:2;C:67152;T:2700;total:69857 | iSNV |
| F25 | F25-11 | 9995 | NS5 | 0.0796 | A:2;G:2;C:4829;T:55777;total:60610 | iSNV |
| F25 | F25-11 | 10069 | NS5 | 0.0653 | A:1;G:0;C:52593;T:3680;total:56274 | iSNV |
| F25 | F25-11 | 10071 | NS5 | 0.662 | A:36843;G:2;C:3;T:18817;total:55665 | iSNV |
| F25 | F25-11 | 10086 | NS5 | 0.045 | A:2568;G:3;C:3;T:54444;total:57018 | iSNV |
| F25 | F25-11 | 10092 | NS5 | 0.0285 | A:1659;G:56544;C:0;T:2;total:58205 | iSNV |
| F25 | F25-11 | 10409 | 3'-UTR | 0.0294 | A:4;G:62089;C:1;T:1883;total:63977 | iSNV |
| F25 | F25-13 | 245 | C | 0.0412 | A:3;G:4;C:2411;T:56068;total:58486 | iSNV |
| F25 | F25-13 | 344 | C | 0.2275 | A:4;G:12522;C:14;T:42492;total:55032 | iSNV |
| F25 | F25-13 | 542 | M | 0.029 | A:3;G:1583;C:18;T:52968;total:54572 | iSNV |
| F25 | F25-13 | 1296 | E | 0.0283 | A:3;G:1;C:1408;T:48334;total:49746 | iSNV |
| F25 | F25-13 | 1468 | E | 0.0225 | A:1209;G:2;C:52416;T:25;total:53652 | iSNV |
| F25 | F25-13 | 1803 | E | 0.5014 | A:28166;G:28319;C:0;T:1;total:56486 | iSNV |
| F25 | F25-13 | 1911 | E | 0.6883 | A:12423;G:27376;C:51;T:1;total:39851 | iSNV |
| F25 | F25-13 | 2780 | NS1 | 0.0213 | A:6;G:0;C:1376;T:62940;total:64322 | iSNV |
| F25 | F25-13 | 3156 | NS1 | 0.0602 | A:3142;G:49030;C:0;T:3;total:52175 | iSNV |
| F25 | F25-13 | 3675 | NS1 | 0.047 | A:1721;G:34839;C:0;T:6;total:36566 | iSNV |
| F25 | F25-13 | 3926 | NS2A | 0.0275 | A:1;G:0;C:50310;T:1426;total:51737 | iSNV |
| F25 | F25-13 | 4021 | NS2A | 0.0243 | A:1053;G:42192;C:0;T:7;total:43252 | iSNV |
| F25 | F25-13 | 4198 | NS2A | 0.032 | A:25043;G:828;C:0;T:0;total:25871 | iSNV |
| F25 | F25-13 | 4943 | NS3 | 0.454 | A:27780;G:23115;C:4;T:4;total:50903 | iSNV |
| F25 | F25-13 | 5311 | NS3 | 0.369 | A:0;G:86;C:14598;T:24872;total:39556 | iSNV |
| F25 | F25-13 | 5358 | NS3 | 0.5101 | A:5;G:20636;C:21477;T:3;total:42121 | iSNV |
| F25 | F25-13 | 5993 | NS3 | 0.2304 | A:36308;G:10875;C:1;T:5;total:47189 | iSNV |
| F25 | F25-13 | 7229 | NS4A | 0.0366 | A:3;G:0;C:33988;T:1294;total:35285 | iSNV |
| F25 | F25-13 | 7495 | NS4B | 0.4442 | A:0;G:0;C:14628;T:18303;total:32931 | iSNV |
| F25 | F25-13 | 7546 | NS4B | 0.5307 | A:18759;G:21201;C:5;T:0;total:39965 | iSNV |
| F25 | F25-13 | 7657 | NS4B | 0.2586 | A:31873;G:11130;C:19;T:2;total:43024 | iSNV |
| F25 | F25-13 | 8927 | NS5 | 0.2437 | A:34409;G:11097;C:7;T:4;total:45517 | iSNV |
| F25 | F25-13 | 9353 | NS5 | 0.0212 | A:60959;G:1322;C:2;T:3;total:62286 | iSNV |
| F25 | F25-13 | 9688 | NS5 | 0.022 | A:0;G:2;C:51121;T:1153;total:52276 | iSNV |
| F25 | F25-13 | 10071 | NS5 | 0.5117 | A:21289;G:2;C:5;T:20323;total:41619 | iSNV |
| F25 | F25-13 | 10092 | NS5 | 0.4652 | A:19862;G:22823;C:1;T:4;total:42690 | iSNV |
| F25 | F25-13 | 10424 | 3'-UTR | 0.0571 | A:11;G:3;C:2773;T:45745;total:48532 | iSNV |
| F25 | F25-13 | 10611 | 3'-UTR | 0.0534 | A:47099;G:2658;C:0;T:8;total:49765 | iSNV |
| F25 | F25-13 | 10723 | 3'-UTR | 0.0806 | A:2;G:2;C:45698;T:4011;total:49713 | iSNV |
| F25 | F25-14 | 574 | M | 0.023 | A:895;G:37928;C:2;T:0;total:38825 | iSNV |
| F25 | F25-14 | 1296 | E | 0.1085 | A:2;G:0;C:4473;T:36739;total:41214 | iSNV |
| F25 | F25-14 | 1911 | E | 0.8191 | A:5866;G:26548;C:4;T:1;total:32419 | iSNV |
| F25 | F25-14 | 3453 | NS1 | 0.0226 | A:656;G:28323;C:0;T:1;total:28980 | iSNV |
| F25 | F25-14 | 4023 | NS2A | 0.0458 | A:1633;G:33963;C:1;T:1;total:35598 | iSNV |
| F25 | F25-14 | 4394 | NS2B | 0.0235 | A:7;G:0;C:34253;T:828;total:35088 | iSNV |
| F25 | F25-14 | 5311 | NS3 | 0.0587 | A:1;G:63;C:2063;T:32974;total:35101 | iSNV |
| F25 | F25-14 | 5835 | NS3 | 0.132 | A:6014;G:39516;C:0;T:3;total:45533 | iSNV |
| F25 | F25-14 | 7381 | NS4B | 0.0228 | A:0;G:1;C:33152;T:774;total:33927 | iSNV |
| F25 | F25-14 | 7495 | NS4B | 0.0441 | A:0;G:0;C:1293;T:27971;total:29264 | iSNV |
| F25 | F25-14 | 7656 | NS4B | 0.0894 | A:3215;G:32241;C:467;T:1;total:35924 | iSNV |
| F25 | F25-14 | 7657 | NS4B | 0.8093 | A:6832;G:28949;C:28;T:6;total:35815 | iSNV |
| F25 | F25-14 | 9407 | NS5 | 0.0337 | A:2;G:1;C:48230;T:1687;total:49920 | iSNV |
| F25 | F25-14 | 9880 | NS5 | 0.0339 | A:1446;G:41116;C:12;T:1;total:42575 | iSNV |
| F25 | F25-14 | 10069 | NS5 | 0.7579 | A:0;G:0;C:8088;T:25308;total:33396 | iSNV |
| F25 | F25-16 | 1417 | E | 0.0706 | A:2;G:1;C:68601;T:5215;total:73819 | iSNV |
| F25 | F25-16 | 1911 | E | 0.8362 | A:8751;G:44640;C:0;T:2;total:53393 | iSNV |
| F25 | F25-16 | 2069 | E | 0.0315 | A:1719;G:0;C:52819;T:5;total:54543 | iSNV |
| F25 | F25-16 | 2433 | E | 0.0355 | A:1754;G:47557;C:5;T:5;total:49321 | iSNV |
| F25 | F25-16 | 3096 | NS1 | 0.1898 | A:56698;G:31;C:7;T:13295;total:70031 | iSNV |
| F25 | F25-16 | 3542 | NS1 | 0.0285 | A:12;G:3;C:1480;T:50304;total:51799 | iSNV |
| F25 | F25-16 | 3685 | NS1 | 0.1258 | A:49035;G:7062;C:3;T:4;total:56104 | iSNV |
| F25 | F25-16 | 4885 | NS3 | 0.023 | A:76626;G:8;C:1809;T:1;total:78444 | iSNV |
| F25 | F25-16 | 5311 | NS3 | 0.7348 | A:2;G:124;C:44926;T:16264;total:61316 | iSNV |
| F25 | F25-16 | 5312 | NS3 | 0.6993 | A:6;G:0;C:42595;T:18320;total:60921 | iSNV |
| F25 | F25-16 | 5875 | NS3 | 0.2891 | A:5;G:2;C:22855;T:56182;total:79044 | iSNV |
| F25 | F25-16 | 7546 | NS4B | 0.1424 | A:48473;G:8770;C:4323;T:1;total:61567 | iSNV |
| F25 | F25-16 | 7656 | NS4B | 0.0684 | A:4665;G:63492;C:8;T:2;total:68167 | iSNV |
| F25 | F25-16 | 7657 | NS4B | 0.8716 | A:8723;G:59173;C:4;T:3;total:67903 | iSNV |
| F25 | F25-16 | 7751 | NS5 | 0.1125 | A:7;G:1;C:6546;T:51624;total:58178 | iSNV |
| F25 | F25-16 | 10069 | NS5 | 0.1578 | A:1;G:0;C:44484;T:8339;total:52824 | iSNV |
| F25 | F25-16 | 10092 | NS5 | 0.0666 | A:3731;G:52258;C:1;T:9;total:55999 | iSNV |
| F25 | F25-16 | 10597 | 3'-UTR | 0.0626 | A:7;G:1;C:59679;T:3986;total:63673 | iSNV |
| F25 | F25-16 | 10808 | 3'-UTR | 0.0788 | A:3995;G:46670;C:7;T:2;total:50674 | iSNV |
| F25 | F25-17 | 287 | C | 0.0566 | A:4402;G:73342;C:12;T:8;total:77764 | iSNV |
| F25 | F25-17 | 1082 | E | 0.0431 | A:53392;G:8;C:3;T:2407;total:55810 | iSNV |
| F25 | F25-17 | 1116 | E | 0.0283 | A:61117;G:1783;C:5;T:4;total:62909 | iSNV |
| F25 | F25-17 | 1298 | E | 0.4856 | A:33919;G:23;C:15;T:35888;total:69845 | iSNV |

| | | | | | | |
|---|---|---|---|---|---|---|
| F25 | F25-17 | 1432 | E | 0.023 | A:70200;G:11;C:4;T:1653;total:71868 | iSNV |
| F25 | F25-17 | 1453 | E | 0.0374 | A:13;G:3;C:2761;T:70958;total:73735 | iSNV |
| F25 | F25-17 | 1491 | E | 0.0509 | A:58677;G:3149;C:0;T:4;total:61830 | iSNV |
| F25 | F25-17 | 1706 | E | 0.0201 | A:72417;G:1487;C:1;T:3;total:73908 | iSNV |
| F25 | F25-17 | 1911 | E | 0.5716 | A:22220;G:29621;C:24;T:1;total:51866 | iSNV |
| F25 | F25-17 | 1963 | E | 0.0522 | A:0;G:0;C:53315;T:2938;total:56253 | iSNV |
| F25 | F25-17 | 2367 | E | 0.0305 | A:46013;G:1451;C:2;T:3;total:47469 | iSNV |
| F25 | F25-17 | 5216 | NS3 | 0.227 | A:50803;G:14925;C:1;T:2;total:65731 | iSNV |
| F25 | F25-17 | 5311 | NS3 | 0.1925 | A:3;G:65;C:13019;T:54520;total:67607 | iSNV |
| F25 | F25-17 | 5343 | NS3 | 0.0595 | A:5;G:0;C:4051;T:63915;total:67971 | iSNV |
| F25 | F25-17 | 5940 | NS3 | 0.0702 | A:6326;G:83669;C:4;T:4;total:90003 | iSNV |
| F25 | F25-17 | 6449 | NS3 | 0.0474 | A:2;G:0;C:3248;T:65154;total:68404 | iSNV |
| F25 | F25-17 | 6978 | NS4A | 0.0973 | A:6572;G:60894;C:7;T:3;total:67476 | iSNV |
| F25 | F25-17 | 7488 | NS4B | 0.2234 | A:39103;G:190;C:4;T:11310;total:50607 | iSNV |
| F25 | F25-17 | 7495 | NS4B | 0.0467 | A:6;G:7;C:2453;T:50016;total:52482 | iSNV |
| F25 | F25-17 | 7656 | NS4B | 0.3036 | A:22401;G:51288;C:78;T:0;total:73767 | iSNV |
| F25 | F25-17 | 7657 | NS4B | 0.3048 | A:51286;G:22517;C:69;T:1;total:73873 | iSNV |
| F25 | F25-17 | 8656 | NS5 | 0.4834 | A:0;G:2;C:34470;T:36832;total:71304 | iSNV |
| F25 | F25-17 | 8659 | NS5 | 0.4907 | A:35697;G:34402;C:4;T:2;total:70105 | iSNV |
| F25 | F25-17 | 9203 | NS5 | 0.0341 | A:3144;G:89002;C:0;T:3;total:92149 | iSNV |
| F25 | F25-17 | 9221 | NS5 | 0.032 | A:90423;G:37;C:2996;T:6;total:93462 | iSNV |
| F25 | F25-17 | 9688 | NS5 | 0.0579 | A:5;G:0;C:55856;T:3438;total:59299 | iSNV |
| F25 | F25-17 | 9932 | NS5 | 0.0254 | A:1582;G:5;C:18;T:60564;total:62169 | iSNV |
| F25 | F25-17 | 10069 | NS5 | 0.2388 | A:2;G:0;C:41405;T:12993;total:54400 | iSNV |
| F25 | F25-17 | 10092 | NS5 | 0.0399 | A:2312;G:54835;C:779;T:3;total:57929 | iSNV |
| F25 | F25-17 | 10811 | 3'-UTR | 0.0214 | A:1109;G:50489;C:2;T:32;total:51632 | iSNV |
| F25 | F25-19 | 971 | M | 0.0508 | A:1;G:1;C:3438;T:64140;total:67580 | iSNV |
| F25 | F25-19 | 1296 | E | 0.5768 | A:5;G:1;C:42392;T:31120;total:73518 | iSNV |
| F25 | F25-19 | 1638 | E | 0.1841 | A:13419;G:0;C:59458;T:10;total:72887 | iSNV |
| F25 | F25-19 | 1911 | E | 0.6078 | A:22931;G:35496;C:27;T:4;total:58458 | iSNV |
| F25 | F25-19 | 4943 | NS3 | 0.6737 | A:24400;G:50352;C:10;T:5;total:74767 | iSNV |
| F25 | F25-19 | 5311 | NS3 | 0.1023 | A:2;G:89;C:7062;T:61824;total:68977 | iSNV |
| F25 | F25-19 | 7495 | NS4B | 0.0555 | A:1;G:2;C:2958;T:50315;total:53276 | iSNV |
| F25 | F25-19 | 7546 | NS4B | 0.1396 | A:57805;G:9386;C:17;T:3;total:67211 | iSNV |
| F25 | F25-19 | 7656 | NS4B | 0.0307 | A:2307;G:72701;C:23;T:0;total:75031 | iSNV |
| F25 | F25-19 | 7657 | NS4B | 0.9059 | A:7033;G:67683;C:4;T:4;total:74724 | iSNV |
| F25 | F25-19 | 8786 | NS5 | 0.0251 | A:6;G:3;C:1636;T:63292;total:64937 | iSNV |
| F25 | F25-19 | 8900 | NS5 | 0.0273 | A:1755;G:633;C:61680;T:11;total:64079 | iSNV |
| F25 | F25-19 | 9360 | NS5 | 0.6594 | A:61336;G:31694;C:3;T:3;total:93036 | iSNV |
| F25 | F25-19 | 9688 | NS5 | 0.832 | A:1;G:2;C:10608;T:52505;total:63116 | iSNV |
| F25 | F25-19 | 10085 | NS5 | 0.0247 | A:1;G:0;C:57554;T:1458;total:59013 | iSNV |
| F25 | F25-19 | 10415 | 3'-UTR | 0.5921 | A:0;G:2;C:38335;T:26416;total:64753 | iSNV |
| F25 | F25-19 | 10807 | 3'-UTR | 0.0284 | A:51888;G:1523;C:13;T:26;total:53450 | iSNV |
| F25 | F25-2 | 497 | M | 0.0414 | A:26;G:59681;C:6;T:2581;total:62294 | iSNV |
| F25 | F25-2 | 1098 | E | 0.0429 | A:9;G:4;C:1781;T:39659;total:41453 | iSNV |
| F25 | F25-2 | 1347 | E | 0.044 | A:49854;G:2300;C:0;T:6;total:52160 | iSNV |
| F25 | F25-2 | 1804 | E | 0.0431 | A:2549;G:56483;C:2;T:4;total:59038 | iSNV |
| F25 | F25-2 | 1911 | E | 0.7422 | A:10774;G:30993;C:15;T:0;total:41782 | iSNV |
| F25 | F25-2 | 2961 | NS1 | 0.0379 | A:51657;G:2037;C:2;T:4;total:53700 | iSNV |
| F25 | F25-2 | 3139 | NS1 | 0.0461 | A:47480;G:2297;C:2;T:1;total:49780 | iSNV |
| F25 | F25-2 | 3464 | NS1 | 0.0383 | A:1;G:3;C:37692;T:1503;total:39199 | iSNV |
| F25 | F25-2 | 3511 | NS1 | 0.3188 | A:4;G:4;C:10669;T:22786;total:33463 | iSNV |
| F25 | F25-2 | 5243 | NS3 | 0.0365 | A:7;G:2;C:1601;T:42246;total:43856 | iSNV |
| F25 | F25-2 | 5311 | NS3 | 0.252 | A:2;G:90;C:11410;T:33765;total:45267 | iSNV |
| F25 | F25-2 | 6523 | NS4A | 0.0369 | A:0;G:2;C:40377;T:1548;total:41927 | iSNV |
| F25 | F25-2 | 7328 | NS4B | 0.0412 | A:31481;G:1354;C:1;T:1;total:32837 | iSNV |
| F25 | F25-2 | 7448 | NS4B | 0.303 | A:23208;G:10094;C:3;T:5;total:33310 | iSNV |
| F25 | F25-2 | 7495 | NS4B | 0.0773 | A:1;G:0;C:2596;T:30968;total:33565 | iSNV |
| F25 | F25-2 | 7575 | NS4B | 0.0252 | A:40533;G:1048;C:1;T:2;total:41584 | iSNV |
| F25 | F25-2 | 7656 | NS4B | 0.242 | A:10811;G:31712;C:2138;T:1;total:44662 | iSNV |
| F25 | F25-2 | 7657 | NS4B | 0.4966 | A:22439;G:22145;C:7;T:0;total:44591 | iSNV |
| F25 | F25-2 | 8217 | NS5 | 0.0343 | A:54480;G:1937;C:2;T:2;total:56421 | iSNV |
| F25 | F25-2 | 8219 | NS5 | 0.0433 | A:6;G:2;C:2468;T:54469;total:56945 | iSNV |
| F25 | F25-2 | 9053 | NS5 | 0.0247 | A:47507;G:1208;C:1;T:6;total:48722 | iSNV |
| F25 | F25-2 | 9688 | NS5 | 0.2038 | A:0;G:0;C:39423;T:10094;total:49517 | iSNV |
| F25 | F25-2 | 10069 | NS5 | 0.7295 | A:0;G:2;C:10879;T:29330;total:40211 | iSNV |
| F25 | F25-20 | 1116 | E | 0.0639 | A:47063;G:3213;C:0;T:4;total:50280 | iSNV |
| F25 | F25-20 | 1296 | E | 0.328 | A:2;G:0;C:18378;T:37635;total:56015 | iSNV |
| F25 | F25-20 | 1733 | E | 0.053 | A:24;G:4;C:2970;T:53002;total:56000 | iSNV |
| F25 | F25-20 | 1803 | E | 0.1643 | A:51262;G:10085;C:1;T:2;total:61350 | iSNV |
| F25 | F25-20 | 1813 | E | 0.0533 | A:57894;G:16;C:3263;T:2;total:61175 | iSNV |
| F25 | F25-20 | 1911 | E | 0.3883 | A:23877;G:15278;C:189;T:0;total:39344 | iSNV |
| F25 | F25-20 | 2466 | E | 0.0322 | A:41513;G:2;C:1385;T:2;total:42902 | iSNV |
| F25 | F25-20 | 3492 | NS1 | 0.0506 | A:32996;G:8;C:1761;T:4;total:34769 | iSNV |
| F25 | F25-20 | 4566 | NS2B | 0.3076 | A:16;G:40786;C:5;T:18135;total:58942 | iSNV |
| F25 | F25-20 | 5311 | NS3 | 0.1074 | A:1;G:197;C:4731;T:39096;total:44025 | iSNV |
| F25 | F25-20 | 5358 | NS3 | 0.1603 | A:5;G:38546;C:7363;T:4;total:45918 | iSNV |
| F25 | F25-20 | 5835 | NS3 | 0.0249 | A:1632;G:63761;C:4;T:0;total:65397 | iSNV |
| F25 | F25-20 | 6533 | NS4A | 0.0218 | A:2;G:5;C:986;T:44170;total:45163 | iSNV |
| F25 | F25-20 | 7495 | NS4B | 0.2964 | A:4;G:1;C:12342;T:29287;total:41634 | iSNV |
| F25 | F25-20 | 7656 | NS4B | 0.4148 | A:23548;G:33196;C:14;T:2;total:56760 | iSNV |
| F25 | F25-20 | 7657 | NS4B | 0.1663 | A:47386;G:9453;C:1;T:1;total:56841 | iSNV |

| | | | | | | |
|---|---|---|---|---|---|---|
| F25 | F25-20 | 9688 | NS5 | 0.3015 | A:0;G:0;C:32447;T:14011;total:46458 | iSNV |
| F25 | F25-20 | 10069 | NS5 | 0.3811 | A:1;G:1;C:25595;T:15763;total:41360 | iSNV |
| F25 | F25-20 | 10071 | NS5 | 0.1651 | A:6904;G:0;C:0;T:34904;total:41808 | iSNV |
| F25 | F25-20 | 10097 | NS5 | 0.0466 | A:3;G:43598;C:2;T:2136;total:45739 | iSNV |
| F25 | F25-20 | 10663 | 3'-UTR | 0.0257 | A:7;G:1252;C:47293;T:36;total:48588 | iSNV |
| F25 | F25-22 | 503 | M | 0.0274 | A:2732;G:96748;C:5;T:0;total:99485 | iSNV |
| F25 | F25-22 | 584 | M | 0.1969 | A:4;G:1;C:61367;T:15050;total:76422 | iSNV |
| F25 | F25-22 | 1296 | E | 0.0221 | A:9;G:2;C:1906;T:84074;total:85991 | iSNV |
| F25 | F25-22 | 1298 | E | 0.2387 | A:20035;G:111;C:35;T:63740;total:83921 | iSNV |
| F25 | F25-22 | 1384 | E | 0.38 | A:53410;G:12;C:24;T:32770;total:86216 | iSNV |
| F25 | F25-22 | 1390 | E | 0.2568 | A:62440;G:21692;C:337;T:1;total:84470 | iSNV |
| F25 | F25-22 | 1491 | E | 0.0554 | A:69382;G:4076;C:2;T:4;total:73464 | iSNV |
| F25 | F25-22 | 1504 | E | 0.0329 | A:10;G:0;C:2447;T:71894;total:74351 | iSNV |
| F25 | F25-22 | 1735 | E | 0.0217 | A:5;G:9;C:1962;T:88223;total:90199 | iSNV |
| F25 | F25-22 | 1786 | E | 0.193 | A:6;G:3;C:18424;T:77023;total:95456 | iSNV |
| F25 | F25-22 | 1800 | E | 0.0531 | A:5237;G:3;C:21;T:93227;total:98488 | iSNV |
| F25 | F25-22 | 1911 | E | 0.3009 | A:45985;G:19822;C:62;T:2;total:65871 | iSNV |
| F25 | F25-22 | 1912 | E | 0.0296 | A:62794;G:18;C:35;T:1922;total:64769 | iSNV |
| F25 | F25-22 | 2447 | E | 0.022 | A:13;G:59822;C:1351;T:4;total:61190 | iSNV |
| F25 | F25-22 | 2843 | NS1 | 0.1862 | A:1;G:0;C:92195;T:21103;total:113299 | iSNV |
| F25 | F25-22 | 3025 | NS1 | 0.0322 | A:97;G:5;C:2137;T:63927;total:66166 | iSNV |
| F25 | F25-22 | 4501 | NS2B | 0.0365 | A:77780;G:2950;C:9;T:0;total:80739 | iSNV |
| F25 | F25-22 | 4640 | NS3 | 0.1849 | A:86885;G:19718;C:1;T:6;total:106610 | iSNV |
| F25 | F25-22 | 5210 | NS3 | 0.0453 | A:1;G:0;C:67465;T:3208;total:70674 | iSNV |
| F25 | F25-22 | 5311 | NS3 | 0.0456 | A:3;G:60;C:3452;T:72026;total:75541 | iSNV |
| F25 | F25-22 | 5723 | NS3 | 0.0207 | A:41;G:7;C:2292;T:108177;total:110517 | iSNV |
| F25 | F25-22 | 6533 | NS4A | 0.2279 | A:2;G:1;C:14956;T:50652;total:65611 | iSNV |
| F25 | F25-22 | 6620 | NS4A | 0.0367 | A:60005;G:2288;C:5;T:1;total:62299 | iSNV |
| F25 | F25-22 | 6944 | NS4A | 0.027 | A:8;G:75220;C:2090;T:1;total:77319 | iSNV |
| F25 | F25-22 | 7264 | NS4A | 0.0339 | A:1;G:0;C:55283;T:1943;total:57227 | iSNV |
| F25 | F25-22 | 7415 | NS4B | 0.0261 | A:1;G:62517;C:6;T:1680;total:64204 | iSNV |
| F25 | F25-22 | 7495 | NS4B | 0.0488 | A:5;G:3;C:2826;T:54968;total:57802 | iSNV |
| F25 | F25-22 | 7543 | NS4B | 0.0394 | A:70564;G:2895;C:0;T:2;total:73461 | iSNV |
| F25 | F25-22 | 7656 | NS4B | 0.3694 | A:30063;G:51209;C:92;T:1;total:81365 | iSNV |
| F25 | F25-22 | 7657 | NS4B | 0.2879 | A:57884;G:23417;C:13;T:0;total:81314 | iSNV |
| F25 | F25-22 | 8064 | NS5 | 0.0381 | A:3529;G:88947;C:7;T:2;total:92485 | iSNV |
| F25 | F25-22 | 8194 | NS5 | 0.0363 | A:80050;G:3023;C:17;T:11;total:83101 | iSNV |
| F25 | F25-22 | 8894 | NS5 | 0.2678 | A:18220;G:12;C:48803;T:976;total:68011 | iSNV |
| F25 | F25-22 | 9225 | NS5 | 0.3862 | A:39983;G:1;C:63510;T:22;total:103516 | iSNV |
| F25 | F25-22 | 9688 | NS5 | 0.2508 | A:0;G:1;C:52059;T:17433;total:69493 | iSNV |
| F25 | F25-22 | 10014 | NS5 | 0.0448 | A:63100;G:6;C:2963;T:9;total:66078 | iSNV |
| F25 | F25-23 | 483 | M | 0.0485 | A:32;G:4228;C:40;T:82710;total:87010 | iSNV |
| F25 | F25-23 | 540 | M | 0.0207 | A:1488;G:70128;C:1;T:7;total:71624 | iSNV |
| F25 | F25-23 | 603 | M | 0.03 | A:9;G:0;C:1866;T:60178;total:62053 | iSNV |
| F25 | F25-23 | 1116 | E | 0.0233 | A:59413;G:1419;C:0;T:1;total:60833 | iSNV |
| F25 | F25-23 | 1296 | E | 0.0415 | A:3;G:4;C:2980;T:68674;total:71661 | iSNV |
| F25 | F25-23 | 1417 | E | 0.0816 | A:1;G:4;C:65900;T:5858;total:71763 | iSNV |
| F25 | F25-23 | 1803 | E | 0.03 | A:73547;G:2281;C:1;T:2;total:75831 | iSNV |
| F25 | F25-23 | 1911 | E | 0.7416 | A:11640;G:33400;C:2;T:0;total:45042 | iSNV |
| F25 | F25-23 | 2181 | E | 0.0239 | A:60378;G:1484;C:1;T:6;total:61869 | iSNV |
| F25 | F25-23 | 3001 | NS1 | 0.051 | A:3240;G:60251;C:3;T:0;total:63494 | iSNV |
| F25 | F25-23 | 3851 | NS2A | 0.0275 | A:4;G:0;C:2198;T:77582;total:79784 | iSNV |
| F25 | F25-23 | 4736 | NS3 | 0.0344 | A:2977;G:83409;C:2;T:1;total:86389 | iSNV |
| F25 | F25-23 | 5311 | NS3 | 0.0476 | A:1;G:183;C:3161;T:62950;total:66295 | iSNV |
| F25 | F25-23 | 5358 | NS3 | 0.0292 | A:9;G:68236;C:2056;T:5;total:70306 | iSNV |
| F25 | F25-23 | 5656 | NS3 | 0.0241 | A:78650;G:1946;C:4;T:1;total:80601 | iSNV |
| F25 | F25-23 | 5835 | NS3 | 0.0526 | A:4881;G:87806;C:7;T:4;total:92698 | iSNV |
| F25 | F25-23 | 6615 | NS4A | 0.0241 | A:0;G:0;C:53362;T:1318;total:54680 | iSNV |
| F25 | F25-23 | 7193 | NS4A | 0.6677 | A:4;G:1;C:32399;T:16131;total:48535 | iSNV |
| F25 | F25-23 | 7264 | NS4A | 0.026 | A:0;G:0;C:41984;T:1122;total:43106 | iSNV |
| F25 | F25-23 | 7373 | NS4B | 0.0315 | A:1617;G:49591;C:4;T:41;total:51253 | iSNV |
| F25 | F25-23 | 7495 | NS4B | 0.0665 | A:0;G:3;C:3226;T:45220;total:48449 | iSNV |
| F25 | F25-23 | 7547 | NS4B | 0.6641 | A:13;G:40552;C:18;T:20527;total:61110 | iSNV |
| F25 | F25-23 | 7641 | NS4B | 0.0214 | A:0;G:0;C:72846;T:1596;total:74442 | iSNV |
| F25 | F25-23 | 7656 | NS4B | 0.0206 | A:1456;G:69059;C:2;T:1;total:70518 | iSNV |
| F25 | F25-23 | 7657 | NS4B | 0.0639 | A:65862;G:4498;C:24;T:4;total:70388 | iSNV |
| F25 | F25-23 | 7658 | NS4B | 0.0207 | A:3;G:28;C:1460;T:68937;total:70428 | iSNV |
| F25 | F25-23 | 8396 | NS5 | 0.0382 | A:83264;G:3313;C:1;T:1;total:86579 | iSNV |
| F25 | F25-23 | 9521 | NS5 | 0.0362 | A:0;G:0;C:67351;T:2533;total:69884 | iSNV |
| F25 | F25-23 | 9688 | NS5 | 0.0256 | A:0;G:0;C:48464;T:1274;total:49738 | iSNV |
| F25 | F25-23 | 10069 | NS5 | 0.0415 | A:1;G:0;C:48492;T:2103;total:50596 | iSNV |
| F25 | F25-23 | 10071 | NS5 | 0.0297 | A:1522;G:0;C:1;T:49582;total:51105 | iSNV |
| F25 | F25-23 | 10092 | NS5 | 0.7738 | A:40573;G:11863;C:1;T:1;total:52438 | iSNV |
| F25 | F25-23 | 10325 | NS5 | 0.0441 | A:2;G:8;C:3330;T:72055;total:75395 | iSNV |
| F25 | F25-25 | 290 | C | 0.0221 | A:3;G:4;C:1714;T:75734;total:77455 | iSNV |
| F25 | F25-25 | 340 | C | 0.3258 | A:21;G:1;C:54514;T:26357;total:80893 | iSNV |
| F25 | F25-25 | 656 | M | 0.1393 | A:3;G:4;C:8937;T:55173;total:64117 | iSNV |
| F25 | F25-25 | 1130 | E | 0.0223 | A:2;G:0;C:67875;T:1549;total:69426 | iSNV |
| F25 | F25-25 | 1296 | E | 0.0592 | A:3;G:4;C:4456;T:70787;total:75250 | iSNV |
| F25 | F25-25 | 1911 | E | 0.7714 | A:13055;G:43990;C:44;T:0;total:57089 | iSNV |
| F25 | F25-25 | 2083 | E | 0.0312 | A:56392;G:15;C:1822;T:11;total:58240 | iSNV |
| F25 | F25-25 | 2141 | E | 0.0975 | A:59213;G:6400;C:0;T:3;total:65616 | iSNV |

| | | | | | | |
|---|---|---|---|---|---|---|
| F25 | F25-25 | 2873 | NS1 | 0.2069 | A:80684;G:16;C:18;T:21059;total:101777 | iSNV |
| F25 | F25-25 | 2882 | NS1 | 0.0289 | A:94749;G:2821;C:2;T:9;total:97581 | iSNV |
| F25 | F25-25 | 4493 | NS2B | 0.0201 | A:5;G:0;C:1360;T:66257;total:67622 | iSNV |
| F25 | F25-25 | 5311 | NS3 | 0.3066 | A:0;G:201;C:20826;T:46889;total:67916 | iSNV |
| F25 | F25-25 | 5835 | NS3 | 0.0947 | A:8799;G:84080;C:3;T:3;total:92885 | iSNV |
| F25 | F25-25 | 6861 | NS4A | 0.0772 | A:1;G:0;C:55272;T:4627;total:59900 | iSNV |
| F25 | F25-25 | 7495 | NS4B | 0.2099 | A:1;G:2;C:10770;T:40519;total:51292 | iSNV |
| F25 | F25-25 | 7543 | NS4B | 0.0528 | A:61122;G:3408;C:0;T:0;total:64530 | iSNV |
| F25 | F25-25 | 7575 | NS4B | 0.0426 | A:63168;G:2815;C:1;T:2;total:65986 | iSNV |
| F25 | F25-25 | 7656 | NS4B | 0.2405 | A:17339;G:54181;C:567;T:1;total:72088 | iSNV |
| F25 | F25-25 | 7657 | NS4B | 0.2556 | A:53662;G:18434;C:3;T:2;total:72101 | iSNV |
| F25 | F25-25 | 9221 | NS5 | 0.1122 | A:81522;G:28;C:12;T:10309;total:91871 | iSNV |
| F25 | F25-25 | 9646 | NS5 | 0.0596 | A:55052;G:3494;C:5;T:11;total:58562 | iSNV |
| F25 | F25-25 | 9688 | NS5 | 0.0269 | A:2;G:3;C:58739;T:1626;total:60370 | iSNV |
| F25 | F25-25 | 10069 | NS5 | 0.6616 | A:1;G:0;C:18399;T:35969;total:54369 | iSNV |
| F25 | F25-25 | 10092 | NS5 | 0.0742 | A:4416;G:54889;C:177;T:4;total:59486 | iSNV |
| F25 | F25-26 | 398 | C | 0.0232 | A:1494;G:62854;C:4;T:8;total:64360 | iSNV |
| F25 | F25-26 | 1058 | E | 0.0448 | A:39513;G:1856;C:2;T:3;total:41374 | iSNV |
| F25 | F25-26 | 1296 | E | 0.095 | A:6;G:1;C:5092;T:48481;total:53580 | iSNV |
| F25 | F25-26 | 1298 | E | 0.0674 | A:41;G:3556;C:19;T:49133;total:52749 | iSNV |
| F25 | F25-26 | 1911 | E | 0.7966 | A:8337;G:32632;C:0;T:1;total:40970 | iSNV |
| F25 | F25-26 | 4070 | NS2A | 0.1366 | A:3;G:6055;C:9;T:38245;total:44312 | iSNV |
| F25 | F25-26 | 4380 | NS2B | 0.1279 | A:6435;G:4;C:43832;T:33;total:50304 | iSNV |
| F25 | F25-26 | 5311 | NS3 | 0.0365 | A:1;G:370;C:1860;T:48610;total:50841 | iSNV |
| F25 | F25-26 | 5871 | NS3 | 0.0806 | A:57456;G:4;C:5039;T:6;total:62505 | iSNV |
| F25 | F25-26 | 6329 | NS3 | 0.1265 | A:6138;G:42355;C:3;T:2;total:48498 | iSNV |
| F25 | F25-26 | 7104 | NS4A | 0.1382 | A:6450;G:40195;C:3;T:1;total:46649 | iSNV |
| F25 | F25-26 | 7179 | NS4A | 0.0737 | A:3069;G:38530;C:1;T:1;total:41601 | iSNV |
| F25 | F25-26 | 7494 | NS4B | 0.1501 | A:32;G:31383;C:4;T:5549;total:36968 | iSNV |
| F25 | F25-26 | 7495 | NS4B | 0.0585 | A:3;G:0;C:2235;T:35911;total:38149 | iSNV |
| F25 | F25-26 | 7656 | NS4B | 0.0214 | A:1142;G:52110;C:22;T:1;total:53275 | iSNV |
| F25 | F25-26 | 7657 | NS4B | 0.8696 | A:6922;G:46108;C:38;T:2;total:53070 | iSNV |
| F25 | F25-26 | 8987 | NS5 | 0.1276 | A:7496;G:2;C:8;T:51229;total:58735 | iSNV |
| F25 | F25-26 | 9358 | NS5 | 0.5401 | A:36241;G:30863;C:2;T:2;total:67108 | iSNV |
| F25 | F25-26 | 9688 | NS5 | 0.0267 | A:1;G:0;C:43468;T:1196;total:44665 | iSNV |
| F25 | F25-26 | 10069 | NS5 | 0.9343 | A:0;G:1;C:2568;T:36474;total:39043 | iSNV |
| F25 | F25-26 | 10416 | 3'-UTR | 0.0556 | A:43850;G:2582;C:1;T:2;total:46435 | iSNV |
| F25 | F25-28 | 235 | C | 0.1361 | A:0;G:0;C:6101;T:38710;total:44811 | iSNV |
| F25 | F25-28 | 650 | M | 0.0357 | A:1361;G:36750;C:0;T:2;total:38113 | iSNV |
| F25 | F25-28 | 836 | M | 0.0315 | A:1;G:0;C:1064;T:32660;total:33725 | iSNV |
| F25 | F25-28 | 902 | M | 0.0511 | A:1;G:0;C:31441;T:1696;total:33138 | iSNV |
| F25 | F25-28 | 982 | E | 0.2419 | A:25691;G:1;C:8200;T:0;total:33892 | iSNV |
| F25 | F25-28 | 1296 | E | 0.0214 | A:1;G:1;C:884;T:40275;total:41161 | iSNV |
| F25 | F25-28 | 1417 | E | 0.0713 | A:1;G:0;C:41041;T:3153;total:44195 | iSNV |
| F25 | F25-28 | 1450 | E | 0.0349 | A:42564;G:22;C:1542;T:2;total:44130 | iSNV |
| F25 | F25-28 | 1468 | E | 0.155 | A:6305;G:1;C:34336;T:11;total:40653 | iSNV |
| F25 | F25-28 | 1786 | E | 0.0293 | A:5;G:6;C:1404;T:46342;total:47757 | iSNV |
| F25 | F25-28 | 1911 | E | 0.4381 | A:18044;G:14085;C:14;T:2;total:32145 | iSNV |
| F25 | F25-28 | 3768 | NS2A | 0.0738 | A:4;G:1;C:2406;T:30162;total:32573 | iSNV |
| F25 | F25-28 | 4177 | NS2A | 0.091 | A:1;G:0;C:18834;T:1887;total:20722 | iSNV |
| F25 | F25-28 | 4294 | NS2B | 0.0234 | A:33596;G:805;C:0;T:0;total:34401 | iSNV |
| F25 | F25-28 | 5311 | NS3 | 0.3214 | A:0;G:127;C:11429;T:23998;total:35554 | iSNV |
| F25 | F25-28 | 6533 | NS4A | 0.042 | A:2;G:2;C:1466;T:33388;total:34858 | iSNV |
| F25 | F25-28 | 6950 | NS4A | 0.0312 | A:4;G:0;C:35844;T:1155;total:37003 | iSNV |
| F25 | F25-28 | 7245 | NS4A | 0.0285 | A:756;G:0;C:25755;T:1;total:26512 | iSNV |
| F25 | F25-28 | 7265 | NS4A | 0.2531 | A:6671;G:19680;C:0;T:0;total:26351 | iSNV |
| F25 | F25-28 | 7495 | NS4B | 0.2896 | A:1;G:2;C:7509;T:18415;total:25927 | iSNV |
| F25 | F25-28 | 7517 | NS4B | 0.0242 | A:733;G:29450;C:0;T:2;total:30185 | iSNV |
| F25 | F25-28 | 7656 | NS4B | 0.0455 | A:1701;G:35556;C:84;T:1;total:37342 | iSNV |
| F25 | F25-28 | 7657 | NS4B | 0.5802 | A:15621;G:21578;C:1;T:7;total:37207 | iSNV |
| F25 | F25-28 | 8410 | NS5 | 0.0546 | A:46464;G:2688;C:3;T:5;total:49160 | iSNV |
| F25 | F25-28 | 8456 | NS5 | 0.2544 | A:39061;G:6;C:4;T:13335;total:52406 | iSNV |
| F25 | F25-28 | 8656 | NS5 | 0.0226 | A:6;G:0;C:802;T:34598;total:35406 | iSNV |
| F25 | F25-28 | 9032 | NS5 | 0.0227 | A:5;G:0;C:923;T:39707;total:40635 | iSNV |
| F25 | F25-28 | 9605 | NS5 | 0.0206 | A:37232;G:9;C:786;T:1;total:38028 | iSNV |
| F25 | F25-28 | 9688 | NS5 | 0.1042 | A:1;G:0;C:30088;T:3501;total:33590 | iSNV |
| F25 | F25-28 | 9839 | NS5 | 0.0225 | A:4;G:1;C:783;T:34006;total:34794 | iSNV |
| F25 | F25-28 | 10069 | NS5 | 0.0745 | A:1;G:0;C:28790;T:2319;total:31110 | iSNV |
| F25 | F25-28 | 10071 | NS5 | 0.0683 | A:2144;G:0;C:2;T:29240;total:31386 | iSNV |
| F25 | F25-28 | 10079 | NS5 | 0.071 | A:29295;G:2245;C:0;T:43;total:31583 | iSNV |
| F25 | F25-28 | 10092 | NS5 | 0.2979 | A:9761;G:22969;C:26;T:0;total:32756 | iSNV |
| F25 | F25-28 | 10095 | NS5 | 0.0516 | A:0;G:32336;C:1762;T:1;total:34099 | iSNV |
| F25 | F25-28 | 10447 | 3'-UTR | 0.0424 | A:1423;G:0;C:32098;T:21;total:33542 | iSNV |
| F25 | F25-30 | 697 | M | 0.0273 | A:1;G:0;C:1209;T:42917;total:44127 | iSNV |
| F25 | F25-30 | 785 | M | 0.0871 | A:38143;G:3641;C:2;T:1;total:41787 | iSNV |
| F25 | F25-30 | 840 | M | 0.0331 | A:35424;G:1217;C:29;T:1;total:36671 | iSNV |
| F25 | F25-30 | 1116 | E | 0.0361 | A:40287;G:1509;C:0;T:4;total:41800 | iSNV |
| F25 | F25-30 | 1296 | E | 0.0206 | A:0;G:0;C:929;T:44132;total:45061 | iSNV |
| F25 | F25-30 | 1298 | E | 0.0215 | A:69;G:952;C:13;T:43208;total:44242 | iSNV |
| F25 | F25-30 | 1390 | E | 0.0247 | A:44329;G:10;C:1126;T:0;total:45465 | iSNV |
| F25 | F25-30 | 1417 | E | 0.0324 | A:0;G:0;C:46633;T:1566;total:48199 | iSNV |
| F25 | F25-30 | 1461 | E | 0.0886 | A:1;G:1;C:4212;T:43318;total:47532 | iSNV |

| | | | | | | |
|---|---|---|---|---|---|---|
| F25 | F25-30 | 1563 | E | 0.0261 | A:9;G:2;C:39819;T:1068;total:40898 | iSNV |
| F25 | F25-30 | 1911 | E | 0.5767 | A:16391;G:22295;C:26;T:1;total:38713 | iSNV |
| F25 | F25-30 | 2129 | E | 0.832 | A:6917;G:34237;C:0;T:6;total:41160 | iSNV |
| F25 | F25-30 | 2291 | E | 0.0333 | A:35862;G:1237;C:1;T:2;total:37102 | iSNV |
| F25 | F25-30 | 2510 | NS1 | 0.03 | A:32426;G:1004;C:2;T:3;total:33435 | iSNV |
| F25 | F25-30 | 3230 | NS1 | 0.7757 | A:12774;G:44149;C:3;T:0;total:56926 | iSNV |
| F25 | F25-30 | 3633 | NS1 | 0.0354 | A:39975;G:1470;C:0;T:6;total:41451 | iSNV |
| F25 | F25-30 | 3934 | NS2A | 0.0244 | A:0;G:2;C:1228;T:48949;total:50179 | iSNV |
| F25 | F25-30 | 3975 | NS2A | 0.084 | A:18;G:4131;C:13;T:44967;total:49129 | iSNV |
| F25 | F25-30 | 4385 | NS2B | 0.0647 | A:2;G:5;C:42096;T:2917;total:45020 | iSNV |
| F25 | F25-30 | 4974 | NS3 | 0.0289 | A:1465;G:49104;C:0;T:1;total:50570 | iSNV |
| F25 | F25-30 | 5311 | NS3 | 0.064 | A:1;G:135;C:2739;T:39877;total:42752 | iSNV |
| F25 | F25-30 | 6533 | NS4A | 0.7998 | A:1;G:1;C:30169;T:7554;total:37725 | iSNV |
| F25 | F25-30 | 6644 | NS4A | 0.0437 | A:2;G:1;C:1421;T:31090;total:32514 | iSNV |
| F25 | F25-30 | 7373 | NS4B | 0.0688 | A:1108;G:33389;C:19;T:2552;total:37068 | iSNV |
| F25 | F25-30 | 7495 | NS4B | 0.0807 | A:0;G:1;C:2593;T:29511;total:32105 | iSNV |
| F25 | F25-30 | 7657 | NS4B | 0.8559 | A:6244;G:37082;C:4;T:0;total:43330 | iSNV |
| F25 | F25-30 | 9688 | NS5 | 0.8386 | A:4;G:1;C:7420;T:38523;total:45948 | iSNV |
| F25 | F25-30 | 9932 | NS5 | 0.0878 | A:3613;G:13;C:9;T:37515;total:41150 | iSNV |
| F25 | F25-30 | 10086 | NS5 | 0.0912 | A:3437;G:0;C:1;T:34238;total:37676 | iSNV |
| F25 | F25-30 | 10723 | 3'-UTR | 0.0291 | A:7;G:1;C:44125;T:1323;total:45456 | iSNV |
| F25 | F25-4 | 1112 | E | 0.0284 | A:1587;G:54169;C:3;T:3;total:55762 | iSNV |
| F25 | F25-4 | 1474 | E | 0.322 | A:40188;G:3;C:19093;T:8;total:59292 | iSNV |
| F25 | F25-4 | 1679 | E | 0.3393 | A:36041;G:18520;C:2;T:5;total:54568 | iSNV |
| F25 | F25-4 | 1786 | E | 0.0308 | A:11;G:6;C:2078;T:65282;total:67377 | iSNV |
| F25 | F25-4 | 1911 | E | 0.2887 | A:36171;G:14697;C:32;T:0;total:50900 | iSNV |
| F25 | F25-4 | 2037 | E | 0.11 | A:4748;G:6;C:38379;T:24;total:43157 | iSNV |
| F25 | F25-4 | 3428 | NS1 | 0.0931 | A:5;G:0;C:44158;T:4536;total:48699 | iSNV |
| F25 | F25-4 | 3761 | NS2A | 0.1086 | A:0;G:2;C:43870;T:5345;total:49217 | iSNV |
| F25 | F25-4 | 5180 | NS3 | 0.0525 | A:2159;G:38947;C:6;T:2;total:41114 | iSNV |
| F25 | F25-4 | 5311 | NS3 | 0.3422 | A:2;G:235;C:15593;T:29730;total:45560 | iSNV |
| F25 | F25-4 | 5835 | NS3 | 0.0335 | A:2029;G:58516;C:2;T:3;total:60550 | iSNV |
| F25 | F25-4 | 5946 | NS3 | 0.2416 | A:13547;G:8;C:42499;T:17;total:56071 | iSNV |
| F25 | F25-4 | 6533 | NS4A | 0.054 | A:3;G:2;C:2379;T:41605;total:43989 | iSNV |
| F25 | F25-4 | 7381 | NS4B | 0.1054 | A:0;G:2;C:39875;T:4700;total:44577 | iSNV |
| F25 | F25-4 | 7495 | NS4B | 0.2955 | A:2;G:2;C:11196;T:26678;total:37878 | iSNV |
| F25 | F25-4 | 7629 | NS4B | 0.025 | A:54261;G:5;C:1396;T:6;total:55668 | iSNV |
| F25 | F25-4 | 7656 | NS4B | 0.1497 | A:7835;G:44435;C:60;T:2;total:52332 | iSNV |
| F25 | F25-4 | 7657 | NS4B | 0.3037 | A:36292;G:15846;C:26;T:2;total:52166 | iSNV |
| F25 | F25-4 | 9292 | NS5 | 0.0341 | A:6;G:61598;C:2176;T:5;total:63785 | iSNV |
| F25 | F25-4 | 9688 | NS5 | 0.0829 | A:4;G:0;C:51922;T:4697;total:56623 | iSNV |
| F25 | F25-4 | 9932 | NS5 | 0.0316 | A:1726;G:8;C:13;T:52871;total:54618 | iSNV |
| F25 | F25-4 | 10068 | NS5 | 0.0388 | A:46260;G:1868;C:0;T:1;total:48129 | iSNV |
| F25 | F25-4 | 10069 | NS5 | 0.3907 | A:1;G:2;C:29064;T:18643;total:47710 | iSNV |
| F25 | F25-5 | 1296 | E | 0.0824 | A:3;G:0;C:4605;T:51273;total:55881 | iSNV |
| F25 | F25-5 | 1911 | E | 0.9334 | A:2914;G:40788;C:0;T:1;total:43703 | iSNV |
| F25 | F25-5 | 1946 | E | 0.0469 | A:2;G:1;C:42766;T:2109;total:44878 | iSNV |
| F25 | F25-5 | 3081 | NS1 | 0.2706 | A:38281;G:14209;C:3;T:1;total:52494 | iSNV |
| F25 | F25-5 | 4069 | NS2A | 0.0249 | A:4;G:1070;C:28;T:41702;total:42804 | iSNV |
| F25 | F25-5 | 4550 | NS2B | 0.0213 | A:4;G:5;C:1145;T:52564;total:53718 | iSNV |
| F25 | F25-5 | 5311 | NS3 | 0.7895 | A:1;G:38;C:36499;T:9746;total:46284 | iSNV |
| F25 | F25-5 | 6523 | NS4A | 0.0249 | A:0;G:1;C:44952;T:1152;total:46105 | iSNV |
| F25 | F25-5 | 6637 | NS4A | 0.4868 | A:1;G:1;C:16812;T:17719;total:34533 | iSNV |
| F25 | F25-5 | 7495 | NS4B | 0.0225 | A:1;G:1;C:842;T:36511;total:37355 | iSNV |
| F25 | F25-5 | 7543 | NS4B | 0.0286 | A:44311;G:1309;C:0;T:3;total:45623 | iSNV |
| F25 | F25-5 | 7656 | NS4B | 0.7889 | A:1480;G:10127;C:36349;T:11;total:47967 | iSNV |
| F25 | F25-5 | 7657 | NS4B | 0.1502 | A:40858;G:7226;C:0;T:2;total:48086 | iSNV |
| F25 | F25-5 | 8093 | NS5 | 0.0214 | A:3;G:6;C:56615;T:1242;total:57866 | iSNV |
| F25 | F25-5 | 9688 | NS5 | 0.1645 | A:2;G:1;C:43286;T:8526;total:51815 | iSNV |
| F25 | F25-5 | 9887 | NS5 | 0.0914 | A:0;G:1;C:4951;T:49162;total:54114 | iSNV |
| F25 | F25-5 | 10092 | NS5 | 0.1014 | A:400;G:42491;C:4842;T:2;total:47735 | iSNV |
| F25 | F25-7 | 442 | C | 0.0256 | A:2;G:2;C:62648;T:1648;total:64300 | iSNV |
| F25 | F25-7 | 505 | M | 0.0363 | A:7;G:4;C:2614;T:69289;total:71914 | iSNV |
| F25 | F25-7 | 697 | M | 0.0237 | A:4;G:0;C:1406;T:57800;total:59210 | iSNV |
| F25 | F25-7 | 1116 | E | 0.0598 | A:51090;G:3254;C:0;T:1;total:54345 | iSNV |
| F25 | F25-7 | 1911 | E | 0.6573 | A:15497;G:29579;C:138;T:1;total:45215 | iSNV |
| F25 | F25-7 | 1913 | E | 0.0227 | A:43373;G:7;C:18;T:1009;total:44407 | iSNV |
| F25 | F25-7 | 3139 | NS1 | 0.588 | A:25692;G:36649;C:2;T:2;total:62345 | iSNV |
| F25 | F25-7 | 3800 | NS2A | 0.0324 | A:4;G:2;C:1479;T:44029;total:45514 | iSNV |
| F25 | F25-7 | 5311 | NS3 | 0.2849 | A:1;G:111;C:13679;T:34215;total:48006 | iSNV |
| F25 | F25-7 | 5336 | NS3 | 0.0225 | A:3;G:1;C:1044;T:45268;total:46316 | iSNV |
| F25 | F25-7 | 5835 | NS3 | 0.0391 | A:2582;G:63282;C:5;T:3;total:65872 | iSNV |
| F25 | F25-7 | 6533 | NS4A | 0.3867 | A:3;G:4;C:17352;T:27506;total:44865 | iSNV |
| F25 | F25-7 | 7495 | NS4B | 0.5682 | A:2;G:0;C:21379;T:16250;total:37631 | iSNV |
| F25 | F25-7 | 7543 | NS4B | 0.2756 | A:32935;G:12532;C:0;T:0;total:45467 | iSNV |
| F25 | F25-7 | 7657 | NS4B | 0.1347 | A:42909;G:6682;C:0;T:3;total:49594 | iSNV |
| F25 | F25-7 | 8140 | NS5 | 0.1185 | A:59926;G:8059;C:11;T:2;total:67998 | iSNV |
| F25 | F25-7 | 9370 | NS5 | 0.1233 | A:9371;G:7;C:30;T:66558;total:75966 | iSNV |
| F25 | F25-7 | 9384 | NS5 | 0.0238 | A:72440;G:1772;C:3;T:1;total:74216 | iSNV |
| F25 | F25-7 | 9688 | NS5 | 0.988 | A:2;G:0;C:779;T:63874;total:64655 | SNP |
| F25 | F25-7 | 10069 | NS5 | 0.0942 | A:1;G:0;C:44322;T:4610;total:48933 | iSNV |
| F25 | F25-7 | 10379 | NS5 | 0.2527 | A:44478;G:15047;C:2;T:0;total:59527 | iSNV |

| | | | | | | |
|---|---|---|---|---|---|---|
| F25 | F25-8 | 761 | M | 0.0216 | A:861;G:38884;C:0;T:0;total:39745 | iSNV |
| F25 | F25-8 | 798 | M | 0.2011 | A:23729;G:6;C:5977;T:1;total:29713 | iSNV |
| F25 | F25-8 | 1116 | E | 0.0372 | A:34431;G:1333;C:0;T:2;total:35766 | iSNV |
| F25 | F25-8 | 1296 | E | 0.0628 | A:4;G:1;C:2354;T:35122;total:37481 | iSNV |
| F25 | F25-8 | 1417 | E | 0.1316 | A:1;G:2;C:36194;T:5489;total:41686 | iSNV |
| F25 | F25-8 | 1660 | E | 0.0274 | A:5;G:0;C:35027;T:990;total:36022 | iSNV |
| F25 | F25-8 | 1911 | E | 0.3183 | A:17469;G:9480;C:2825;T:0;total:29774 | iSNV |
| F25 | F25-8 | 3538 | NS1 | 0.529 | A:12597;G:2;C:14142;T:1;total:26742 | iSNV |
| F25 | F25-8 | 4023 | NS2A | 0.0228 | A:810;G:34653;C:2;T:4;total:35469 | iSNV |
| F25 | F25-8 | 4401 | NS2B | 0.511 | A:1;G:16121;C:16840;T:4;total:32966 | iSNV |
| F25 | F25-8 | 5311 | NS3 | 0.0267 | A:2;G:40;C:908;T:32978;total:33928 | iSNV |
| F25 | F25-8 | 5528 | NS3 | 0.5182 | A:20999;G:22578;C:2;T:1;total:43580 | iSNV |
| F25 | F25-8 | 5835 | NS3 | 0.1732 | A:7913;G:37746;C:1;T:3;total:45663 | iSNV |
| F25 | F25-8 | 6533 | NS4A | 0.7715 | A:5;G:1;C:23626;T:7002;total:30634 | iSNV |
| F25 | F25-8 | 7495 | NS4B | 0.0205 | A:3;G:0;C:587;T:27991;total:28581 | iSNV |
| F25 | F25-8 | 7657 | NS4B | 0.961 | A:1382;G:34014;C:3;T:0;total:35399 | iSNV |
| F25 | F25-8 | 8140 | NS5 | 0.0599 | A:45173;G:2882;C:0;T:5;total:48060 | iSNV |
| F25 | F25-8 | 8534 | NS5 | 0.1549 | A:6;G:5;C:8704;T:47455;total:56170 | iSNV |
| F25 | F25-8 | 9221 | NS5 | 0.0242 | A:50552;G:21;C:1259;T:7;total:51839 | iSNV |
| F25 | F25-8 | 9688 | NS5 | 0.7851 | A:1;G:0;C:9116;T:33285;total:42402 | iSNV |
| F25 | F25-8 | 10080 | NS5 | 0.0284 | A:33262;G:2;C:974;T:1;total:34239 | iSNV |
| F25 | F25-8 | 10419 | 3'-UTR | 0.764 | A:2;G:5;C:9255;T:29949;total:39211 | iSNV |
| F25 | F25-8 | 10814 | 3'-UTR | 0.0287 | A:0;G:30317;C:0;T:898;total:31215 | iSNV |
| F30 | F30-1 | 398 | C | 0.0742 | A:5352;G:66742;C:12;T:6;total:72112 | iSNV |
| F30 | F30-1 | 1116 | E | 0.2089 | A:43849;G:11586;C:0;T:4;total:55439 | iSNV |
| F30 | F30-1 | 1296 | E | 0.1686 | A:2;G:1;C:9177;T:45225;total:54405 | iSNV |
| F30 | F30-1 | 1298 | E | 0.0258 | A:23;G:1401;C:15;T:52699;total:54138 | iSNV |
| F30 | F30-1 | 1347 | E | 0.0318 | A:57809;G:1900;C:17;T:0;total:59726 | iSNV |
| F30 | F30-1 | 1417 | E | 0.0714 | A:4;G:3;C:61098;T:4701;total:65806 | iSNV |
| F30 | F30-1 | 1482 | E | 0.0372 | A:2201;G:56955;C:5;T:1;total:59162 | iSNV |
| F30 | F30-1 | 1803 | E | 0.1367 | A:60944;G:9656;C:2;T:4;total:70606 | iSNV |
| F30 | F30-1 | 1911 | E | 0.3495 | A:33431;G:18006;C:76;T:1;total:51514 | iSNV |
| F30 | F30-1 | 2298 | E | 0.0298 | A:1345;G:43644;C:2;T:6;total:44997 | iSNV |
| F30 | F30-1 | 3528 | NS1 | 0.0475 | A:1;G:6;C:43980;T:2196;total:46183 | iSNV |
| F30 | F30-1 | 3814 | NS2A | 0.0207 | A:10;G:1;C:1143;T:53878;total:55032 | iSNV |
| F30 | F30-1 | 3993 | NS2A | 0.0953 | A:5536;G:52513;C:4;T:7;total:58060 | iSNV |
| F30 | F30-1 | 4233 | NS2B | 0.0707 | A:2;G:2800;C:4;T:36785;total:39591 | iSNV |
| F30 | F30-1 | 5006 | NS3 | 0.0213 | A:67600;G:15;C:2;T:1477;total:69094 | iSNV |
| F30 | F30-1 | 5311 | NS3 | 0.6955 | A:3;G:370;C:37904;T:16761;total:55038 | iSNV |
| F30 | F30-1 | 5358 | NS3 | 0.1305 | A:6;G:49875;C:7490;T:3;total:57374 | iSNV |
| F30 | F30-1 | 6707 | NS4A | 0.0703 | A:3467;G:8;C:34;T:45790;total:49299 | iSNV |
| F30 | F30-1 | 7179 | NS4A | 0.0605 | A:3098;G:47689;C:386;T:5;total:51178 | iSNV |
| F30 | F30-1 | 7495 | NS4B | 0.0926 | A:1;G:5;C:4279;T:41900;total:46185 | iSNV |
| F30 | F30-1 | 7526 | NS4B | 0.0983 | A:5226;G:0;C:107;T:47811;total:53144 | iSNV |
| F30 | F30-1 | 7575 | NS4B | 0.0237 | A:55609;G:1352;C:1;T:0;total:56962 | iSNV |
| F30 | F30-1 | 7626 | NS4B | 0.7183 | A:8;G:44532;C:6;T:17475;total:62021 | iSNV |
| F30 | F30-1 | 7657 | NS4B | 0.0346 | A:55648;G:2000;C:4;T:1;total:57653 | iSNV |
| F30 | F30-1 | 8091 | NS5 | 0.0259 | A:5;G:1;C:64633;T:1722;total:66361 | iSNV |
| F30 | F30-1 | 9688 | NS5 | 0.1624 | A:0;G:0;C:55387;T:10739;total:66126 | iSNV |
| F30 | F30-1 | 9932 | NS5 | 0.0527 | A:3180;G:6;C:34;T:57033;total:60253 | iSNV |
| F30 | F30-1 | 10071 | NS5 | 0.1282 | A:6868;G:1;C:4;T:46688;total:53561 | iSNV |
| F30 | F30-1 | 10092 | NS5 | 0.1124 | A:6182;G:48784;C:3;T:0;total:54969 | iSNV |
| F30 | F30-10 | 400 | C | 0.039 | A:61566;G:2505;C:4;T:4;total:64079 | iSNV |
| F30 | F30-10 | 820 | M | 0.0491 | A:1777;G:6;C:34358;T:22;total:36163 | iSNV |
| F30 | F30-10 | 821 | M | 0.052 | A:10;G:33953;C:1865;T:5;total:35833 | iSNV |
| F30 | F30-10 | 1247 | E | 0.4614 | A:3;G:2;C:18823;T:21963;total:40791 | iSNV |
| F30 | F30-10 | 1296 | E | 0.0654 | A:3;G:0;C:2820;T:40252;total:43075 | iSNV |
| F30 | F30-10 | 1342 | E | 0.0402 | A:4;G:5;C:44406;T:1864;total:46279 | iSNV |
| F30 | F30-10 | 1347 | E | 0.0575 | A:42864;G:2617;C:0;T:2;total:45483 | iSNV |
| F30 | F30-10 | 1359 | E | 0.0387 | A:44399;G:1788;C:1;T:0;total:46188 | iSNV |
| F30 | F30-10 | 1708 | E | 0.045 | A:1959;G:41484;C:0;T:6;total:43449 | iSNV |
| F30 | F30-10 | 1911 | E | 0.571 | A:15734;G:20934;C:3;T:1;total:36672 | iSNV |
| F30 | F30-10 | 2924 | NS1 | 0.0459 | A:1;G:1;C:2335;T:48468;total:50805 | iSNV |
| F30 | F30-10 | 3113 | NS1 | 0.3789 | A:9;G:2;C:17286;T:28315;total:45612 | iSNV |
| F30 | F30-10 | 4021 | NS2A | 0.0201 | A:930;G:45207;C:0;T:4;total:46141 | iSNV |
| F30 | F30-10 | 4985 | NS3 | 0.0209 | A:2;G:2;C:1203;T:56185;total:57392 | iSNV |
| F30 | F30-10 | 5311 | NS3 | 0.417 | A:2;G:160;C:19984;T:27774;total:47920 | iSNV |
| F30 | F30-10 | 6368 | NS3 | 0.3432 | A:4;G:14666;C:11;T:28052;total:42733 | iSNV |
| F30 | F30-10 | 6533 | NS4A | 0.0227 | A:1;G:3;C:1057;T:45460;total:46521 | iSNV |
| F30 | F30-10 | 6707 | NS4A | 0.0383 | A:2;G:0;C:1648;T:41332;total:42982 | iSNV |
| F30 | F30-10 | 7373 | NS4B | 0.3694 | A:103;G:24427;C:14379;T:16;total:38925 | iSNV |
| F30 | F30-10 | 7486 | NS4B | 0.0256 | A:3;G:3;C:911;T:34624;total:35541 | iSNV |
| F30 | F30-10 | 7495 | NS4B | 0.0456 | A:5;G:2;C:1665;T:34784;total:36456 | iSNV |
| F30 | F30-10 | 7638 | NS4B | 0.0316 | A:50971;G:1664;C:1;T:0;total:52636 | iSNV |
| F30 | F30-10 | 7656 | NS4B | 0.3851 | A:18449;G:28633;C:811;T:2;total:47895 | iSNV |
| F30 | F30-10 | 7657 | NS4B | 0.518 | A:23100;G:24818;C:1;T:2;total:47921 | iSNV |
| F30 | F30-10 | 8140 | NS5 | 0.1676 | A:52288;G:430;C:10619;T:11;total:63348 | iSNV |
| F30 | F30-10 | 8798 | NS5 | 0.021 | A:47729;G:1028;C:8;T:5;total:48770 | iSNV |
| F30 | F30-10 | 9688 | NS5 | 0.0644 | A:0;G:0;C:54911;T:3785;total:58696 | iSNV |
| F30 | F30-10 | 10068 | NS5 | 0.0373 | A:49269;G:1909;C:0;T:1;total:51179 | iSNV |
| F30 | F30-10 | 10069 | NS5 | 0.4087 | A:1;G:0;C:29784;T:20591;total:50376 | iSNV |
| F30 | F30-10 | 10092 | NS5 | 0.0242 | A:1285;G:51763;C:11;T:3;total:53062 | iSNV |

| | | | | | | |
|---|---|---|---|---|---|---|
| F30 | F30-10 | 10111 | NS5 | 0.3451 | A:38190;G:20127;C:1;T:4;total:58322 | iSNV |
| F30 | F30-10 | 10518 | 3'-UTR | 0.0351 | A:3;G:0;C:48538;T:1768;total:50309 | iSNV |
| F30 | F30-11 | 221 | C | 0.0337 | A:1387;G:39754;C:7;T:8;total:41156 | iSNV |
| F30 | F30-11 | 226 | C | 0.0212 | A:4;G:0;C:915;T:42064;total:42983 | iSNV |
| F30 | F30-11 | 455 | C | 0.0555 | A:2294;G:0;C:35;T:38984;total:41313 | iSNV |
| F30 | F30-11 | 1296 | E | 0.0584 | A:2;G:0;C:1826;T:29434;total:31262 | iSNV |
| F30 | F30-11 | 1417 | E | 0.0758 | A:0;G:0;C:33351;T:2738;total:36089 | iSNV |
| F30 | F30-11 | 1468 | E | 0.07 | A:2425;G:0;C:32168;T:19;total:34612 | iSNV |
| F30 | F30-11 | 1803 | E | 0.1078 | A:32627;G:3943;C:0;T:0;total:36570 | iSNV |
| F30 | F30-11 | 1911 | E | 0.1401 | A:23952;G:3905;C:0;T:1;total:27858 | iSNV |
| F30 | F30-11 | 1913 | E | 0.5776 | A:11125;G:1;C:15190;T:17;total:26333 | iSNV |
| F30 | F30-11 | 1934 | E | 0.0434 | A:0;G:3;C:23912;T:1086;total:25001 | iSNV |
| F30 | F30-11 | 2306 | E | 0.0518 | A:24352;G:1333;C:0;T:0;total:25685 | iSNV |
| F30 | F30-11 | 2685 | NS1 | 0.0297 | A:909;G:29679;C:13;T:3;total:30604 | iSNV |
| F30 | F30-11 | 3242 | NS1 | 0.1357 | A:8;G:0;C:5697;T:36261;total:41966 | iSNV |
| F30 | F30-11 | 3989 | NS2A | 0.1503 | A:5595;G:31594;C:7;T:13;total:37209 | iSNV |
| F30 | F30-11 | 5311 | NS3 | 0.2791 | A:0;G:714;C:9661;T:24235;total:34610 | iSNV |
| F30 | F30-11 | 5312 | NS3 | 0.0497 | A:3;G:8;C:1699;T:32413;total:34123 | iSNV |
| F30 | F30-11 | 5358 | NS3 | 0.2197 | A:3;G:27395;C:7718;T:2;total:35118 | iSNV |
| F30 | F30-11 | 6523 | NS4A | 0.5469 | A:0;G:1;C:15016;T:18119;total:33136 | iSNV |
| F30 | F30-11 | 7495 | NS4B | 0.195 | A:3;G:0;C:4758;T:19633;total:24394 | iSNV |
| F30 | F30-11 | 7557 | NS4B | 0.0618 | A:2005;G:30397;C:0;T:6;total:32408 | iSNV |
| F30 | F30-11 | 7575 | NS4B | 0.0374 | A:29860;G:1161;C:2;T:1;total:31024 | iSNV |
| F30 | F30-11 | 7629 | NS4B | 0.2704 | A:25733;G:0;C:9538;T:1;total:35272 | iSNV |
| F30 | F30-11 | 7656 | NS4B | 0.0691 | A:2397;G:31935;C:321;T:4;total:34657 | iSNV |
| F30 | F30-11 | 7657 | NS4B | 0.0865 | A:31624;G:2997;C:1;T:0;total:34622 | iSNV |
| F30 | F30-11 | 7919 | NS5 | 0.0459 | A:2048;G:42518;C:3;T:2;total:44571 | iSNV |
| F30 | F30-11 | 8398 | NS5 | 0.1927 | A:8682;G:1;C:2;T:36369;total:45054 | iSNV |
| F30 | F30-11 | 8900 | NS5 | 0.0298 | A:1061;G:0;C:34502;T:6;total:35569 | iSNV |
| F30 | F30-11 | 9170 | NS5 | 0.0328 | A:10;G:1;C:1605;T:47248;total:48864 | iSNV |
| F30 | F30-11 | 9607 | NS5 | 0.5363 | A:21048;G:1;C:24324;T:9;total:45382 | iSNV |
| F30 | F30-11 | 9688 | NS5 | 0.0288 | A:2;G:0;C:41362;T:1228;total:42592 | iSNV |
| F30 | F30-11 | 9995 | NS5 | 0.214 | A:1;G:0;C:7619;T:27970;total:35590 | iSNV |
| F30 | F30-11 | 10069 | NS5 | 0.031 | A:0;G:1;C:36512;T:1171;total:37684 | iSNV |
| F30 | F30-11 | 10071 | NS5 | 0.7589 | A:28294;G:2;C:2;T:8991;total:37289 | iSNV |
| F30 | F30-11 | 10086 | NS5 | 0.0626 | A:2387;G:2;C:2;T:35734;total:38125 | iSNV |
| F30 | F30-11 | 10092 | NS5 | 0.0534 | A:2087;G:36957;C:4;T:2;total:39050 | iSNV |
| F30 | F30-11 | 10409 | 3'-UTR | 0.1367 | A:7;G:37494;C:3;T:5943;total:43447 | iSNV |
| F30 | F30-13 | 245 | C | 0.0648 | A:10;G:5;C:5455;T:78650;total:84120 | iSNV |
| F30 | F30-13 | 344 | C | 0.3663 | A:6;G:27705;C:35;T:47876;total:75622 | iSNV |
| F30 | F30-13 | 503 | M | 0.0424 | A:3555;G:80105;C:3;T:6;total:83669 | iSNV |
| F30 | F30-13 | 1296 | E | 0.1152 | A:2;G:0;C:6786;T:52101;total:58889 | iSNV |
| F30 | F30-13 | 1468 | E | 0.028 | A:1931;G:3;C:66854;T:28;total:68816 | iSNV |
| F30 | F30-13 | 1619 | E | 0.0485 | A:2698;G:14;C:24;T:52837;total:55573 | iSNV |
| F30 | F30-13 | 1803 | E | 0.5218 | A:34874;G:38047;C:0;T:6;total:72927 | iSNV |
| F30 | F30-13 | 1911 | E | 0.5047 | A:29035;G:29543;C:34;T:1;total:58613 | iSNV |
| F30 | F30-13 | 2780 | NS1 | 0.0201 | A:30;G:4;C:1527;T:74357;total:75918 | iSNV |
| F30 | F30-13 | 3156 | NS1 | 0.0382 | A:2556;G:64261;C:2;T:1;total:66820 | iSNV |
| F30 | F30-13 | 3634 | NS1 | 0.0535 | A:7;G:3;C:3257;T:57501;total:60768 | iSNV |
| F30 | F30-13 | 3675 | NS1 | 0.0671 | A:3966;G:55048;C:2;T:5;total:59021 | iSNV |
| F30 | F30-13 | 3773 | NS2A | 0.0424 | A:16;G:3;C:2659;T:59930;total:62608 | iSNV |
| F30 | F30-13 | 4198 | NS2A | 0.0347 | A:44965;G:1620;C:0;T:1;total:46586 | iSNV |
| F30 | F30-13 | 4943 | NS3 | 0.4515 | A:37531;G:30912;C:9;T:3;total:68455 | iSNV |
| F30 | F30-13 | 5311 | NS3 | 0.324 | A:3;G:389;C:19983;T:41291;total:61666 | iSNV |
| F30 | F30-13 | 5358 | NS3 | 0.5186 | A:1;G:29323;C:31575;T:8;total:60907 | iSNV |
| F30 | F30-13 | 5993 | NS3 | 0.3799 | A:39748;G:24366;C:7;T:9;total:64130 | iSNV |
| F30 | F30-13 | 6568 | NS4A | 0.0417 | A:53119;G:2316;C:4;T:2;total:55441 | iSNV |
| F30 | F30-13 | 7067 | NS4A | 0.0397 | A:66612;G:2758;C:8;T:4;total:69382 | iSNV |
| F30 | F30-13 | 7229 | NS4A | 0.0716 | A:0;G:1;C:50127;T:3869;total:53997 | iSNV |
| F30 | F30-13 | 7495 | NS4B | 0.428 | A:5;G:5;C:19832;T:26484;total:46326 | iSNV |
| F30 | F30-13 | 7546 | NS4B | 0.5349 | A:25839;G:29697;C:7;T:1;total:55544 | iSNV |
| F30 | F30-13 | 7657 | NS4B | 0.4052 | A:36646;G:25014;C:62;T:4;total:61726 | iSNV |
| F30 | F30-13 | 8927 | NS5 | 0.3776 | A:40782;G:24760;C:9;T:8;total:65559 | iSNV |
| F30 | F30-13 | 9224 | NS5 | 0.0409 | A:3446;G:80690;C:6;T:10;total:84152 | iSNV |
| F30 | F30-13 | 9688 | NS5 | 0.0527 | A:3;G:1;C:76262;T:4249;total:80515 | iSNV |
| F30 | F30-13 | 10071 | NS5 | 0.5223 | A:33539;G:3;C:3;T:30683;total:64228 | iSNV |
| F30 | F30-13 | 10092 | NS5 | 0.4451 | A:29580;G:36868;C:2;T:4;total:66454 | iSNV |
| F30 | F30-13 | 10424 | 3'-UTR | 0.0365 | A:11;G:2;C:2527;T:66522;total:69062 | iSNV |
| F30 | F30-13 | 10611 | 3'-UTR | 0.033 | A:73211;G:2504;C:6;T:4;total:75725 | iSNV |
| F30 | F30-13 | 10723 | 3'-UTR | 0.0506 | A:1;G:1;C:73512;T:3925;total:77439 | iSNV |
| F30 | F30-14 | 299 | C | 0.0245 | A:80708;G:14;C:2029;T:5;total:82756 | iSNV |
| F30 | F30-14 | 541 | M | 0.0207 | A:8;G:35;C:1495;T:70560;total:72098 | iSNV |
| F30 | F30-14 | 1296 | E | 0.1731 | A:4;G:1;C:10404;T:49682;total:60091 | iSNV |
| F30 | F30-14 | 1417 | E | 0.0219 | A:0;G:1;C:66602;T:1492;total:68095 | iSNV |
| F30 | F30-14 | 1911 | E | 0.6774 | A:18770;G:39389;C:7;T:2;total:58168 | iSNV |
| F30 | F30-14 | 3299 | NS1 | 0.0302 | A:2038;G:1;C:65247;T:20;total:67306 | iSNV |
| F30 | F30-14 | 3453 | NS1 | 0.0331 | A:1867;G:54432;C:1;T:3;total:56303 | iSNV |
| F30 | F30-14 | 3511 | NS1 | 0.0434 | A:17;G:3;C:2180;T:48001;total:50201 | iSNV |
| F30 | F30-14 | 3530 | NS1 | 0.0202 | A:1157;G:193;C:36;T:55787;total:57173 | iSNV |
| F30 | F30-14 | 3773 | NS2A | 0.0236 | A:21;G:0;C:1468;T:60622;total:62111 | iSNV |
| F30 | F30-14 | 4023 | NS2A | 0.0276 | A:1635;G:57511;C:3;T:4;total:59153 | iSNV |
| F30 | F30-14 | 4336 | NS2B | 0.0282 | A:7;G:0;C:1776;T:60978;total:62761 | iSNV |

| | | | | | | |
|---|---|---|---|---|---|---|
| F30 | F30-14 | 4394 | NS2B | 0.0676 | A:2;G:0;C:56418;T:4092;total:60512 | iSNV |
| F30 | F30-14 | 5311 | NS3 | 0.1161 | A:2;G:580;C:7176;T:54001;total:61759 | iSNV |
| F30 | F30-14 | 5612 | NS3 | 0.0242 | A:6;G:6;C:1708;T:68700;total:70420 | iSNV |
| F30 | F30-14 | 5835 | NS3 | 0.1624 | A:12166;G:62729;C:4;T:5;total:74904 | iSNV |
| F30 | F30-14 | 5987 | NS3 | 0.0222 | A:0;G:2;C:65327;T:1488;total:66817 | iSNV |
| F30 | F30-14 | 6935 | NS4A | 0.029 | A:62314;G:11;C:1865;T:18;total:64208 | iSNV |
| F30 | F30-14 | 6979 | NS4A | 0.0247 | A:4;G:1677;C:66129;T:26;total:67836 | iSNV |
| F30 | F30-14 | 7381 | NS4B | 0.0706 | A:2;G:2;C:51547;T:3920;total:55471 | iSNV |
| F30 | F30-14 | 7495 | NS4B | 0.1002 | A:7;G:2;C:4870;T:43721;total:48600 | iSNV |
| F30 | F30-14 | 7528 | NS4B | 0.0294 | A:1;G:1;C:56231;T:1704;total:57937 | iSNV |
| F30 | F30-14 | 7656 | NS4B | 0.1274 | A:7616;G:51570;C:561;T:3;total:59750 | iSNV |
| F30 | F30-14 | 7657 | NS4B | 0.7399 | A:15485;G:44005;C:29;T:1;total:59520 | iSNV |
| F30 | F30-14 | 8194 | NS5 | 0.0332 | A:66999;G:2309;C:17;T:150;total:69475 | iSNV |
| F30 | F30-14 | 9335 | NS5 | 0.021 | A:6;G:3;C:1722;T:80193;total:81924 | iSNV |
| F30 | F30-14 | 9407 | NS5 | 0.0996 | A:1;G:2;C:72073;T:7974;total:80050 | iSNV |
| F30 | F30-14 | 9688 | NS5 | 0.0414 | A:3;G:3;C:74914;T:3239;total:78159 | iSNV |
| F30 | F30-14 | 9880 | NS5 | 0.098 | A:7169;G:65946;C:11;T:6;total:73132 | iSNV |
| F30 | F30-14 | 10069 | NS5 | 0.5966 | A:3;G:4;C:25083;T:37081;total:62171 | iSNV |
| F30 | F30-16 | 1130 | E | 0.0206 | A:2;G:3;C:52015;T:1096;total:53116 | iSNV |
| F30 | F30-16 | 1296 | E | 0.0405 | A:6;G:1;C:2108;T:49851;total:51966 | iSNV |
| F30 | F30-16 | 1417 | E | 0.1734 | A:2;G:0;C:47814;T:10036;total:57852 | iSNV |
| F30 | F30-16 | 1911 | E | 0.6244 | A:18186;G:30222;C:1;T:1;total:48410 | iSNV |
| F30 | F30-16 | 3096 | NS1 | 0.1355 | A:42513;G:24;C:4;T:6670;total:49211 | iSNV |
| F30 | F30-16 | 3685 | NS1 | 0.0938 | A:42402;G:4390;C:0;T:5;total:46797 | iSNV |
| F30 | F30-16 | 4089 | NS2A | 0.0203 | A:46547;G:969;C:0;T:1;total:47517 | iSNV |
| F30 | F30-16 | 4885 | NS3 | 0.0697 | A:58998;G:18;C:4423;T:1;total:63440 | iSNV |
| F30 | F30-16 | 5311 | NS3 | 0.7313 | A:0;G:554;C:37606;T:14024;total:52184 | iSNV |
| F30 | F30-16 | 5312 | NS3 | 0.713 | A:2;G:3;C:36981;T:14892;total:51878 | iSNV |
| F30 | F30-16 | 5875 | NS3 | 0.243 | A:2;G:1;C:14808;T:46103;total:60914 | iSNV |
| F30 | F30-16 | 7495 | NS4B | 0.0301 | A:1;G:0;C:1208;T:38839;total:40048 | iSNV |
| F30 | F30-16 | 7546 | NS4B | 0.1402 | A:38694;G:6650;C:2084;T:3;total:47431 | iSNV |
| F30 | F30-16 | 7656 | NS4B | 0.048 | A:2478;G:49047;C:8;T:2;total:51535 | iSNV |
| F30 | F30-16 | 7657 | NS4B | 0.8413 | A:8139;G:43107;C:14;T:1;total:51261 | iSNV |
| F30 | F30-16 | 7751 | NS5 | 0.2687 | A:3;G:0;C:11531;T:31380;total:42914 | iSNV |
| F30 | F30-16 | 8073 | NS5 | 0.0262 | A:7;G:1575;C:10;T:58471;total:60063 | iSNV |
| F30 | F30-16 | 8438 | NS5 | 0.0364 | A:1;G:2;C:2426;T:64080;total:66509 | iSNV |
| F30 | F30-16 | 9688 | NS5 | 0.0243 | A:2;G:1;C:56962;T:1422;total:58387 | iSNV |
| F30 | F30-16 | 10069 | NS5 | 0.1078 | A:3;G:0;C:40958;T:4951;total:45912 | iSNV |
| F30 | F30-16 | 10080 | NS5 | 0.0246 | A:45920;G:2;C:1160;T:6;total:47088 | iSNV |
| F30 | F30-16 | 10092 | NS5 | 0.0442 | A:2151;G:46438;C:14;T:2;total:48605 | iSNV |
| F30 | F30-16 | 10097 | NS5 | 0.0205 | A:2;G:49689;C:4;T:1044;total:50739 | iSNV |
| F30 | F30-16 | 10343 | NS5 | 0.0542 | A:1;G:1;C:57632;T:3303;total:60937 | iSNV |
| F30 | F30-16 | 10597 | 3'-UTR | 0.039 | A:2;G:0;C:50578;T:2053;total:52633 | iSNV |
| F30 | F30-16 | 10808 | 3'-UTR | 0.0702 | A:2871;G:37993;C:3;T:3;total:40870 | iSNV |
| F30 | F30-17 | 287 | C | 0.0624 | A:2064;G:30957;C:2;T:2;total:33025 | iSNV |
| F30 | F30-17 | 1082 | E | 0.0514 | A:19771;G:5;C:1;T:1072;total:20849 | iSNV |
| F30 | F30-17 | 1117 | E | 0.0263 | A:22961;G:5;C:621;T:3;total:23590 | iSNV |
| F30 | F30-17 | 1298 | E | 0.4272 | A:10392;G:17;C:2;T:13913;total:24324 | iSNV |
| F30 | F30-17 | 1453 | E | 0.0857 | A:6;G:0;C:2428;T:25875;total:28309 | iSNV |
| F30 | F30-17 | 1786 | E | 0.0282 | A:7;G:1;C:842;T:28981;total:29831 | iSNV |
| F30 | F30-17 | 1911 | E | 0.5226 | A:10789;G:11771;C:36;T:0;total:22596 | iSNV |
| F30 | F30-17 | 1963 | E | 0.0351 | A:2;G:0;C:22782;T:829;total:23613 | iSNV |
| F30 | F30-17 | 2367 | E | 0.0724 | A:18574;G:1452;C:5;T:3;total:20034 | iSNV |
| F30 | F30-17 | 3973 | NS2A | 0.0295 | A:0;G:0;C:32734;T:998;total:33732 | iSNV |
| F30 | F30-17 | 4896 | NS3 | 0.1009 | A:3782;G:33676;C:2;T:4;total:37464 | iSNV |
| F30 | F30-17 | 5216 | NS3 | 0.0655 | A:25828;G:1812;C:0;T:0;total:27640 | iSNV |
| F30 | F30-17 | 5311 | NS3 | 0.2286 | A:0;G:80;C:6568;T:22083;total:28731 | iSNV |
| F30 | F30-17 | 5343 | NS3 | 0.1132 | A:1;G:0;C:3255;T:25490;total:28746 | iSNV |
| F30 | F30-17 | 5747 | NS3 | 0.0872 | A:3291;G:34422;C:2;T:0;total:37715 | iSNV |
| F30 | F30-17 | 5940 | NS3 | 0.1182 | A:4195;G:31278;C:0;T:0;total:35473 | iSNV |
| F30 | F30-17 | 6449 | NS3 | 0.0284 | A:4;G:1;C:868;T:29585;total:30458 | iSNV |
| F30 | F30-17 | 6533 | NS4A | 0.0235 | A:1;G:1;C:630;T:26092;total:26724 | iSNV |
| F30 | F30-17 | 6978 | NS4A | 0.1428 | A:4260;G:25558;C:0;T:3;total:29821 | iSNV |
| F30 | F30-17 | 7488 | NS4B | 0.067 | A:19880;G:42;C:1;T:1431;total:21354 | iSNV |
| F30 | F30-17 | 7495 | NS4B | 0.038 | A:0;G:4;C:830;T:21003;total:21837 | iSNV |
| F30 | F30-17 | 7543 | NS4B | 0.0213 | A:26439;G:577;C:2;T:1;total:27019 | iSNV |
| F30 | F30-17 | 7656 | NS4B | 0.3912 | A:11415;G:17719;C:38;T:0;total:29172 | iSNV |
| F30 | F30-17 | 7657 | NS4B | 0.4476 | A:16110;G:13081;C:30;T:0;total:29221 | iSNV |
| F30 | F30-17 | 8354 | NS5 | 0.0217 | A:2;G:1;C:809;T:36316;total:37128 | iSNV |
| F30 | F30-17 | 8656 | NS5 | 0.3582 | A:0;G:0;C:11164;T:19995;total:31159 | iSNV |
| F30 | F30-17 | 8659 | NS5 | 0.3658 | A:19308;G:11141;C:3;T:2;total:30454 | iSNV |
| F30 | F30-17 | 9203 | NS5 | 0.064 | A:2412;G:35258;C:0;T:2;total:37672 | iSNV |
| F30 | F30-17 | 9221 | NS5 | 0.062 | A:34892;G:13;C:2309;T:1;total:37215 | iSNV |
| F30 | F30-17 | 9596 | NS5 | 0.0889 | A:28475;G:19;C:5;T:2782;total:31281 | iSNV |
| F30 | F30-17 | 9688 | NS5 | 0.0976 | A:1;G:0;C:28564;T:3091;total:31656 | iSNV |
| F30 | F30-17 | 10069 | NS5 | 0.3814 | A:0;G:0;C:15387;T:9490;total:24877 | iSNV |
| F30 | F30-17 | 10092 | NS5 | 0.0316 | A:840;G:25409;C:326;T:1;total:26576 | iSNV |
| F30 | F30-17 | 10811 | 3'-UTR | 0.0783 | A:1853;G:21784;C:4;T:13;total:23654 | iSNV |
| F30 | F30-19 | 226 | C | 0.0405 | A:6;G:0;C:1645;T:38906;total:40557 | iSNV |
| F30 | F30-19 | 308 | C | 0.033 | A:40467;G:1392;C:246;T:7;total:42112 | iSNV |
| F30 | F30-19 | 310 | C | 0.0408 | A:7;G:0;C:1711;T:40207;total:41925 | iSNV |
| F30 | F30-19 | 914 | M | 0.0386 | A:0;G:0;C:32341;T:1301;total:33642 | iSNV |

| | | | | | | |
|---|---|---|---|---|---|---|
| F30 | F30-19 | 971 | M | 0.0929 | A:1;G:3;C:3133;T:30584;total:33721 | iSNV |
| F30 | F30-19 | 1296 | E | 0.4421 | A:2;G:0;C:15821;T:19962;total:35785 | iSNV |
| F30 | F30-19 | 1348 | E | 0.0394 | A:36636;G:1;C:6;T:1504;total:38147 | iSNV |
| F30 | F30-19 | 1638 | E | 0.3135 | A:11977;G:1;C:26215;T:3;total:38196 | iSNV |
| F30 | F30-19 | 1911 | E | 0.4083 | A:19403;G:13400;C:10;T:1;total:32814 | iSNV |
| F30 | F30-19 | 3685 | NS1 | 0.0271 | A:32675;G:14;C:0;T:914;total:33603 | iSNV |
| F30 | F30-19 | 4017 | NS2A | 0.0283 | A:35699;G:76;C:3;T:1045;total:36823 | iSNV |
| F30 | F30-19 | 4173 | NS2A | 0.0347 | A:22246;G:802;C:4;T:0;total:23052 | iSNV |
| F30 | F30-19 | 4394 | NS2B | 0.0257 | A:1008;G:1;C:38085;T:15;total:39109 | iSNV |
| F30 | F30-19 | 4943 | NS3 | 0.4569 | A:23611;G:19868;C:4;T:0;total:43483 | iSNV |
| F30 | F30-19 | 5311 | NS3 | 0.1065 | A:1;G:183;C:4173;T:34817;total:39174 | iSNV |
| F30 | F30-19 | 6533 | NS4A | 0.037 | A:4;G:2;C:1266;T:32943;total:34215 | iSNV |
| F30 | F30-19 | 6943 | NS4A | 0.0516 | A:2129;G:0;C:39056;T:18;total:41203 | iSNV |
| F30 | F30-19 | 7495 | NS4B | 0.0779 | A:1;G:3;C:2250;T:26614;total:28868 | iSNV |
| F30 | F30-19 | 7546 | NS4B | 0.2653 | A:25845;G:9341;C:14;T:1;total:35201 | iSNV |
| F30 | F30-19 | 7557 | NS4B | 0.0345 | A:246;G:36992;C:0;T:1334;total:38572 | iSNV |
| F30 | F30-19 | 7559 | NS4B | 0.0352 | A:1412;G:0;C:38655;T:13;total:40080 | iSNV |
| F30 | F30-19 | 7656 | NS4B | 0.0672 | A:2667;G:36922;C:86;T:2;total:39677 | iSNV |
| F30 | F30-19 | 7657 | NS4B | 0.8092 | A:7549;G:32007;C:3;T:0;total:39559 | iSNV |
| F30 | F30-19 | 8786 | NS5 | 0.0245 | A:3;G:0;C:913;T:36223;total:37139 | iSNV |
| F30 | F30-19 | 9360 | NS5 | 0.4474 | A:22617;G:27931;C:0;T:0;total:50548 | iSNV |
| F30 | F30-19 | 9688 | NS5 | 0.8164 | A:3;G:0;C:7844;T:34864;total:42711 | iSNV |
| F30 | F30-19 | 10097 | NS5 | 0.0389 | A:4;G:35961;C:23;T:1458;total:37446 | iSNV |
| F30 | F30-19 | 10415 | 3'-UTR | 0.4149 | A:2;G:0;C:14546;T:20506;total:35054 | iSNV |
| F30 | F30-19 | 10433 | 3'-UTR | 0.033 | A:1132;G:1;C:14;T:33083;total:34230 | iSNV |
| F30 | F30-19 | 10807 | 3'-UTR | 0.0267 | A:30385;G:836;C:3;T:15;total:31239 | iSNV |
| F30 | F30-19 | 10809 | 3'-UTR | 0.0239 | A:5;G:2;C:30704;T:755;total:31466 | iSNV |
| F30 | F30-2 | 312 | C | 0.0404 | A:3043;G:72263;C:2;T:2;total:75310 | iSNV |
| F30 | F30-2 | 497 | M | 0.0998 | A:30;G:73944;C:8;T:8205;total:82187 | iSNV |
| F30 | F30-2 | 1098 | E | 0.1101 | A:6;G:3;C:6398;T:51691;total:58098 | iSNV |
| F30 | F30-2 | 1127 | E | 0.0312 | A:7;G:1;C:2101;T:65089;total:67198 | iSNV |
| F30 | F30-2 | 1296 | E | 0.032 | A:7;G:0;C:2059;T:62262;total:64328 | iSNV |
| F30 | F30-2 | 1347 | E | 0.1066 | A:61671;G:7361;C:0;T:8;total:69040 | iSNV |
| F30 | F30-2 | 1417 | E | 0.0361 | A:0;G:5;C:72486;T:2718;total:75209 | iSNV |
| F30 | F30-2 | 1804 | E | 0.1054 | A:8706;G:73864;C:1;T:9;total:82580 | iSNV |
| F30 | F30-2 | 1911 | E | 0.5446 | A:29113;G:34787;C:21;T:0;total:63921 | iSNV |
| F30 | F30-2 | 2291 | E | 0.0312 | A:53065;G:1714;C:2;T:1;total:54782 | iSNV |
| F30 | F30-2 | 2624 | NS1 | 0.029 | A:2033;G:67960;C:7;T:3;total:70003 | iSNV |
| F30 | F30-2 | 3139 | NS1 | 0.0642 | A:61537;G:4222;C:0;T:2;total:65761 | iSNV |
| F30 | F30-2 | 3464 | NS1 | 0.0308 | A:2;G:2;C:55370;T:1760;total:57134 | iSNV |
| F30 | F30-2 | 3511 | NS1 | 0.2788 | A:14;G:13;C:14054;T:36321;total:50402 | iSNV |
| F30 | F30-2 | 4069 | NS2A | 0.022 | A:7;G:1202;C:66;T:53268;total:54543 | iSNV |
| F30 | F30-2 | 4314 | NS2B | 0.0279 | A:62159;G:1786;C:1;T:0;total:63946 | iSNV |
| F30 | F30-2 | 4522 | NS2B | 0.0253 | A:0;G:4;C:68661;T:1788;total:70453 | iSNV |
| F30 | F30-2 | 5243 | NS3 | 0.0363 | A:4;G:0;C:2178;T:57816;total:59998 | iSNV |
| F30 | F30-2 | 5311 | NS3 | 0.4194 | A:3;G:337;C:26129;T:35823;total:62292 | iSNV |
| F30 | F30-2 | 6335 | NS3 | 0.0203 | A:1;G:0;C:55991;T:1164;total:57156 | iSNV |
| F30 | F30-2 | 6523 | NS4A | 0.037 | A:0;G:4;C:55975;T:2151;total:58130 | iSNV |
| F30 | F30-2 | 7223 | NS4A | 0.0284 | A:7;G:4;C:1558;T:53283;total:54852 | iSNV |
| F30 | F30-2 | 7328 | NS4B | 0.096 | A:42360;G:4499;C:0;T:3;total:46862 | iSNV |
| F30 | F30-2 | 7448 | NS4B | 0.2637 | A:35266;G:12634;C:1;T:2;total:47903 | iSNV |
| F30 | F30-2 | 7495 | NS4B | 0.0802 | A:0;G:1;C:3851;T:44139;total:47991 | iSNV |
| F30 | F30-2 | 7575 | NS4B | 0.0325 | A:56656;G:1906;C:2;T:4;total:58568 | iSNV |
| F30 | F30-2 | 7656 | NS4B | 0.3277 | A:19564;G:37678;C:2446;T:0;total:59688 | iSNV |
| F30 | F30-2 | 7657 | NS4B | 0.472 | A:31485;G:28152;C:3;T:4;total:59644 | iSNV |
| F30 | F30-2 | 8217 | NS5 | 0.0335 | A:72676;G:2524;C:1;T:1;total:75202 | iSNV |
| F30 | F30-2 | 8219 | NS5 | 0.1031 | A:9;G:4;C:7806;T:67825;total:75644 | iSNV |
| F30 | F30-2 | 8447 | NS5 | 0.0209 | A:9;G:3;C:1781;T:83421;total:85214 | iSNV |
| F30 | F30-2 | 9688 | NS5 | 0.3659 | A:2;G:1;C:48477;T:27985;total:76465 | iSNV |
| F30 | F30-2 | 10069 | NS5 | 0.5289 | A:0;G:2;C:27250;T:30590;total:57842 | iSNV |
| F30 | F30-20 | 1116 | E | 0.0367 | A:30391;G:1159;C:4;T:1;total:31555 | iSNV |
| F30 | F30-20 | 1296 | E | 0.6015 | A:2;G:0;C:19245;T:12753;total:32000 | iSNV |
| F30 | F30-20 | 1347 | E | 0.0229 | A:33745;G:793;C:1;T:2;total:34541 | iSNV |
| F30 | F30-20 | 1417 | E | 0.0358 | A:0;G:2;C:34932;T:1298;total:36232 | iSNV |
| F30 | F30-20 | 1803 | E | 0.1508 | A:36242;G:6437;C:0;T:1;total:42680 | iSNV |
| F30 | F30-20 | 1911 | E | 0.3083 | A:21872;G:9791;C:86;T:1;total:31750 | iSNV |
| F30 | F30-20 | 2466 | E | 0.1462 | A:22930;G:7;C:3929;T:2;total:26868 | iSNV |
| F30 | F30-20 | 3579 | NS1 | 0.0491 | A:32815;G:1696;C:0;T:5;total:34516 | iSNV |
| F30 | F30-20 | 3797 | NS2A | 0.0306 | A:35862;G:3;C:0;T:1134;total:36999 | iSNV |
| F30 | F30-20 | 4566 | NS2B | 0.5117 | A:12;G:21657;C:7;T:22674;total:44350 | iSNV |
| F30 | F30-20 | 5311 | NS3 | 0.0814 | A:1;G:249;C:3059;T:34246;total:37555 | iSNV |
| F30 | F30-20 | 5358 | NS3 | 0.1248 | A:6;G:33439;C:4773;T:3;total:38221 | iSNV |
| F30 | F30-20 | 5835 | NS3 | 0.0406 | A:1913;G:45170;C:6;T:2;total:47091 | iSNV |
| F30 | F30-20 | 7373 | NS4B | 0.0334 | A:1074;G:31070;C:0;T:2;total:32146 | iSNV |
| F30 | F30-20 | 7495 | NS4B | 0.1311 | A:1;G:2;C:3808;T:25229;total:29040 | iSNV |
| F30 | F30-20 | 7656 | NS4B | 0.6166 | A:23272;G:14477;C:7;T:0;total:37756 | iSNV |
| F30 | F30-20 | 7657 | NS4B | 0.2156 | A:29669;G:8156;C:2;T:2;total:37829 | iSNV |
| F30 | F30-20 | 9688 | NS5 | 0.136 | A:0;G:0;C:36234;T:5704;total:41938 | iSNV |
| F30 | F30-20 | 10069 | NS5 | 0.5766 | A:1;G:2;C:13362;T:18190;total:31555 | iSNV |
| F30 | F30-20 | 10071 | NS5 | 0.1372 | A:4385;G:0;C:4;T:27553;total:31942 | iSNV |
| F30 | F30-20 | 10663 | 3'-UTR | 0.0274 | A:7;G:1115;C:39539;T:24;total:40685 | iSNV |
| F30 | F30-22 | 503 | M | 0.1932 | A:9261;G:38646;C:3;T:3;total:47913 | iSNV |

| | | | | | | |
|---|---|---|---|---|---|---|
| F30 | F30-22 | 584 | M | 0.3058 | A:17;G:0;C:24504;T:10802;total:35323 | iSNV |
| F30 | F30-22 | 697 | M | 0.0234 | A:6;G:0;C:913;T:37965;total:38884 | iSNV |
| F30 | F30-22 | 1298 | E | 0.1737 | A:6431;G:22;C:12;T:30542;total:37007 | iSNV |
| F30 | F30-22 | 1384 | E | 0.3823 | A:23400;G:7;C:7;T:14492;total:37906 | iSNV |
| F30 | F30-22 | 1390 | E | 0.3296 | A:24872;G:12251;C:35;T:1;total:37159 | iSNV |
| F30 | F30-22 | 1491 | E | 0.0449 | A:33316;G:1570;C:7;T:3;total:34896 | iSNV |
| F30 | F30-22 | 1504 | E | 0.2165 | A:2;G:2;C:7621;T:27563;total:35188 | iSNV |
| F30 | F30-22 | 1786 | E | 0.3049 | A:5;G:4;C:13551;T:30871;total:44431 | iSNV |
| F30 | F30-22 | 1800 | E | 0.0458 | A:2107;G:2;C:8;T:43869;total:45986 | iSNV |
| F30 | F30-22 | 1911 | E | 0.1878 | A:25391;G:5873;C:0;T:1;total:31265 | iSNV |
| F30 | F30-22 | 1912 | E | 0.1189 | A:26785;G:11;C:8;T:3620;total:30424 | iSNV |
| F30 | F30-22 | 2843 | NS1 | 0.298 | A:4;G:3;C:35460;T:15061;total:50528 | iSNV |
| F30 | F30-22 | 3025 | NS1 | 0.2065 | A:73;G:0;C:6406;T:24536;total:31015 | iSNV |
| F30 | F30-22 | 3368 | NS1 | 0.0546 | A:2101;G:36311;C:1;T:0;total:38413 | iSNV |
| F30 | F30-22 | 4640 | NS3 | 0.2553 | A:45467;G:15593;C:5;T:4;total:61069 | iSNV |
| F30 | F30-22 | 5210 | NS3 | 0.0614 | A:3;G:1;C:36123;T:2365;total:38492 | iSNV |
| F30 | F30-22 | 5311 | NS3 | 0.0236 | A:0;G:91;C:1001;T:41147;total:42239 | iSNV |
| F30 | F30-22 | 6533 | NS4A | 0.2753 | A:1;G:14;C:10751;T:28280;total:39046 | iSNV |
| F30 | F30-22 | 6944 | NS4A | 0.1733 | A:6;G:36947;C:7750;T:5;total:44708 | iSNV |
| F30 | F30-22 | 7264 | NS4A | 0.0604 | A:0;G:0;C:34108;T:2195;total:36303 | iSNV |
| F30 | F30-22 | 7373 | NS4B | 0.0441 | A:1638;G:35259;C:170;T:29;total:37096 | iSNV |
| F30 | F30-22 | 7495 | NS4B | 0.24 | A:2;G:3;C:7681;T:24305;total:31991 | iSNV |
| F30 | F30-22 | 7656 | NS4B | 0.2775 | A:13114;G:34085;C:49;T:1;total:47249 | iSNV |
| F30 | F30-22 | 7657 | NS4B | 0.3729 | A:29683;G:17653;C:1;T:1;total:47338 | iSNV |
| F30 | F30-22 | 8190 | NS5 | 0.0297 | A:7;G:3;C:1445;T:47185;total:48640 | iSNV |
| F30 | F30-22 | 8194 | NS5 | 0.1141 | A:41777;G:5386;C:4;T:6;total:47173 | iSNV |
| F30 | F30-22 | 8894 | NS5 | 0.1814 | A:6802;G:5;C:30464;T:215;total:37486 | iSNV |
| F30 | F30-22 | 9225 | NS5 | 0.3163 | A:18123;G:2;C:39152;T:9;total:57286 | iSNV |
| F30 | F30-22 | 9688 | NS5 | 0.3039 | A:1;G:1;C:30043;T:13119;total:43164 | iSNV |
| F30 | F30-22 | 9932 | NS5 | 0.0779 | A:3203;G:0;C:7;T:37858;total:41068 | iSNV |
| F30 | F30-22 | 10014 | NS5 | 0.0217 | A:39678;G:8;C:883;T:5;total:40574 | iSNV |
| F30 | F30-23 | 503 | M | 0.182 | A:10485;G:47090;C:5;T:0;total:57580 | iSNV |
| F30 | F30-23 | 584 | M | 0.3275 | A:26;G:3;C:29049;T:14165;total:43243 | iSNV |
| F30 | F30-23 | 697 | M | 0.0211 | A:1;G:0;C:982;T:45363;total:46346 | iSNV |
| F30 | F30-23 | 1298 | E | 0.1737 | A:7773;G:27;C:7;T:36917;total:44724 | iSNV |
| F30 | F30-23 | 1384 | E | 0.3646 | A:29229;G:9;C:10;T:16790;total:46038 | iSNV |
| F30 | F30-23 | 1390 | E | 0.3113 | A:31136;G:14094;C:31;T:1;total:45262 | iSNV |
| F30 | F30-23 | 1491 | E | 0.0416 | A:41808;G:1819;C:0;T:6;total:43633 | iSNV |
| F30 | F30-23 | 1504 | E | 0.2052 | A:2;G:0;C:8982;T:34784;total:43768 | iSNV |
| F30 | F30-23 | 1786 | E | 0.3229 | A:8;G:0;C:17241;T:36131;total:53380 | iSNV |
| F30 | F30-23 | 1800 | E | 0.0409 | A:2263;G:3;C:14;T:53000;total:55280 | iSNV |
| F30 | F30-23 | 1911 | E | 0.1871 | A:31877;G:7341;C:9;T:2;total:39229 | iSNV |
| F30 | F30-23 | 1912 | E | 0.1158 | A:33710;G:9;C:20;T:4421;total:38160 | iSNV |
| F30 | F30-23 | 2843 | NS1 | 0.3189 | A:2;G:0;C:41865;T:19607;total:61474 | iSNV |
| F30 | F30-23 | 3025 | NS1 | 0.1959 | A:70;G:2;C:7533;T:30846;total:38451 | iSNV |
| F30 | F30-23 | 3368 | NS1 | 0.0517 | A:2419;G:44310;C:1;T:3;total:46733 | iSNV |
| F30 | F30-23 | 4640 | NS3 | 0.2707 | A:53162;G:19741;C:0;T:2;total:72905 | iSNV |
| F30 | F30-23 | 5210 | NS3 | 0.0672 | A:2;G:0;C:44580;T:3216;total:47798 | iSNV |
| F30 | F30-23 | 5311 | NS3 | 0.0219 | A:1;G:76;C:1141;T:50821;total:52039 | iSNV |
| F30 | F30-23 | 6533 | NS4A | 0.2929 | A:3;G:19;C:14191;T:34233;total:48446 | iSNV |
| F30 | F30-23 | 6944 | NS4A | 0.1656 | A:3;G:47294;C:9389;T:5;total:56691 | iSNV |
| F30 | F30-23 | 7264 | NS4A | 0.0586 | A:0;G:0;C:41603;T:2591;total:44194 | iSNV |
| F30 | F30-23 | 7373 | NS4B | 0.0459 | A:2080;G:42896;C:237;T:46;total:45259 | iSNV |
| F30 | F30-23 | 7495 | NS4B | 0.2295 | A:2;G:5;C:9054;T:30374;total:39435 | iSNV |
| F30 | F30-23 | 7656 | NS4B | 0.2846 | A:15832;G:39709;C:69;T:2;total:55612 | iSNV |
| F30 | F30-23 | 7657 | NS4B | 0.3814 | A:34395;G:21215;C:3;T:2;total:55615 | iSNV |
| F30 | F30-23 | 8190 | NS5 | 0.0302 | A:7;G:1;C:1806;T:57897;total:59711 | iSNV |
| F30 | F30-23 | 8194 | NS5 | 0.1185 | A:50634;G:6813;C:9;T:10;total:57466 | iSNV |
| F30 | F30-23 | 8894 | NS5 | 0.1834 | A:8298;G:12;C:36634;T:295;total:45239 | iSNV |
| F30 | F30-23 | 9225 | NS5 | 0.3004 | A:20719;G:3;C:48214;T:20;total:68956 | iSNV |
| F30 | F30-23 | 9688 | NS5 | 0.3124 | A:2;G:0;C:38333;T:17423;total:55758 | iSNV |
| F30 | F30-23 | 9932 | NS5 | 0.0768 | A:3877;G:1;C:24;T:46575;total:50477 | iSNV |
| F30 | F30-23 | 10014 | NS5 | 0.0219 | A:47790;G:6;C:1074;T:2;total:48872 | iSNV |
| F30 | F30-25 | 340 | C | 0.1636 | A:18;G:0;C:34209;T:6698;total:40925 | iSNV |
| F30 | F30-25 | 399 | C | 0.1169 | A:4910;G:2;C:37077;T:2;total:41991 | iSNV |
| F30 | F30-25 | 656 | M | 0.0982 | A:3;G:0;C:3092;T:28360;total:31455 | iSNV |
| F30 | F30-25 | 1296 | E | 0.1129 | A:1;G:0;C:4160;T:32656;total:36817 | iSNV |
| F30 | F30-25 | 1347 | E | 0.0358 | A:35616;G:1325;C:5;T:3;total:36949 | iSNV |
| F30 | F30-25 | 1465 | E | 0.1997 | A:28064;G:9;C:7009;T:4;total:35086 | iSNV |
| F30 | F30-25 | 1911 | E | 0.5143 | A:15271;G:16156;C:14;T:0;total:31441 | iSNV |
| F30 | F30-25 | 2083 | E | 0.0251 | A:29597;G:10;C:764;T:6;total:30377 | iSNV |
| F30 | F30-25 | 2141 | E | 0.1102 | A:29844;G:3699;C:0;T:2;total:33545 | iSNV |
| F30 | F30-25 | 2558 | NS1 | 0.0399 | A:1;G:1;C:24742;T:1029;total:25773 | iSNV |
| F30 | F30-25 | 2873 | NS1 | 0.1134 | A:44195;G:11;C:6;T:5659;total:49871 | iSNV |
| F30 | F30-25 | 3511 | NS1 | 0.0323 | A:11;G:4;C:948;T:28310;total:29273 | iSNV |
| F30 | F30-25 | 3914 | NS2A | 0.0215 | A:46273;G:1019;C:1;T:0;total:47293 | iSNV |
| F30 | F30-25 | 4233 | NS2B | 0.0215 | A:3;G:600;C:3;T:27270;total:27876 | iSNV |
| F30 | F30-25 | 5025 | NS3 | 0.03 | A:1;G:1;C:49164;T:1524;total:50690 | iSNV |
| F30 | F30-25 | 5181 | NS3 | 0.0368 | A:0;G:0;C:31919;T:1221;total:33140 | iSNV |
| F30 | F30-25 | 5311 | NS3 | 0.2558 | A:0;G:639;C:9389;T:26673;total:36701 | iSNV |
| F30 | F30-25 | 5835 | NS3 | 0.0974 | A:4526;G:41906;C:3;T:0;total:46435 | iSNV |
| F30 | F30-25 | 6024 | NS3 | 0.0321 | A:2;G:0;C:1423;T:42839;total:44264 | iSNV |

| | | | | | | |
|---|---|---|---|---|---|---|
| F30 | F30-25 | 6861 | NS4A | 0.0539 | A:2;G:0;C:33695;T:1922;total:35619 | iSNV |
| F30 | F30-25 | 7373 | NS4B | 0.0339 | A:150;G:30737;C:50;T:1088;total:32025 | iSNV |
| F30 | F30-25 | 7495 | NS4B | 0.2127 | A:3;G:1;C:6091;T:22536;total:28631 | iSNV |
| F30 | F30-25 | 7543 | NS4B | 0.0432 | A:34495;G:1558;C:1;T:0;total:36054 | iSNV |
| F30 | F30-25 | 7575 | NS4B | 0.0379 | A:35849;G:1415;C:0;T:0;total:37264 | iSNV |
| F30 | F30-25 | 7656 | NS4B | 0.2126 | A:8443;G:30885;C:380;T:1;total:39709 | iSNV |
| F30 | F30-25 | 7657 | NS4B | 0.4551 | A:21583;G:18030;C:0;T:1;total:39614 | iSNV |
| F30 | F30-25 | 7913 | NS5 | 0.06 | A:5;G:1;C:2818;T:44092;total:46916 | iSNV |
| F30 | F30-25 | 9221 | NS5 | 0.1526 | A:42331;G:22;C:6;T:7630;total:49989 | iSNV |
| F30 | F30-25 | 9646 | NS5 | 0.0435 | A:36540;G:1663;C:2;T:0;total:38205 | iSNV |
| F30 | F30-25 | 9688 | NS5 | 0.0494 | A:1;G:0;C:37831;T:1970;total:39802 | iSNV |
| F30 | F30-25 | 10069 | NS5 | 0.6052 | A:1;G:0;C:12350;T:18924;total:31275 | iSNV |
| F30 | F30-25 | 10092 | NS5 | 0.0555 | A:1903;G:32197;C:146;T:4;total:34250 | iSNV |
| F30 | F30-26 | 312 | C | 0.0522 | A:2608;G:47338;C:1;T:0;total:49947 | iSNV |
| F30 | F30-26 | 1058 | E | 0.0337 | A:34337;G:1200;C:0;T:2;total:35539 | iSNV |
| F30 | F30-26 | 1296 | E | 0.0658 | A:1;G:1;C:2762;T:39157;total:41921 | iSNV |
| F30 | F30-26 | 1298 | E | 0.3462 | A:25;G:14189;C:11;T:26750;total:40975 | iSNV |
| F30 | F30-26 | 1911 | E | 0.4681 | A:20776;G:18297;C:8;T:2;total:39083 | iSNV |
| F30 | F30-26 | 4070 | NS2A | 0.4113 | A:7;G:16944;C:13;T:24228;total:41192 | iSNV |
| F30 | F30-26 | 4380 | NS2B | 0.4012 | A:18809;G:7;C:28047;T:13;total:46876 | iSNV |
| F30 | F30-26 | 5311 | NS3 | 0.0284 | A:1;G:1182;C:1273;T:42276;total:44732 | iSNV |
| F30 | F30-26 | 5638 | NS3 | 0.0382 | A:1;G:1948;C:48965;T:14;total:50928 | iSNV |
| F30 | F30-26 | 5871 | NS3 | 0.3399 | A:36600;G:3;C:18854;T:3;total:55460 | iSNV |
| F30 | F30-26 | 6329 | NS3 | 0.3948 | A:18059;G:27668;C:3;T:1;total:45731 | iSNV |
| F30 | F30-26 | 7104 | NS4A | 0.4098 | A:19316;G:27809;C:6;T:0;total:47131 | iSNV |
| F30 | F30-26 | 7179 | NS4A | 0.0423 | A:1918;G:43351;C:0;T:1;total:45270 | iSNV |
| F30 | F30-26 | 7494 | NS4B | 0.4604 | A:41;G:20482;C:9;T:17520;total:38052 | iSNV |
| F30 | F30-26 | 7495 | NS4B | 0.034 | A:1;G:3;C:1341;T:38035;total:39380 | iSNV |
| F30 | F30-26 | 7657 | NS4B | 0.9377 | A:2884;G:43310;C:49;T:2;total:46245 | iSNV |
| F30 | F30-26 | 8987 | NS5 | 0.3967 | A:18793;G:8;C:7;T:28561;total:47369 | iSNV |
| F30 | F30-26 | 9358 | NS5 | 0.3034 | A:16602;G:38108;C:1;T:7;total:54718 | iSNV |
| F30 | F30-26 | 9688 | NS5 | 0.0233 | A:2;G:1;C:50223;T:1199;total:51425 | iSNV |
| F30 | F30-26 | 10069 | NS5 | 0.9672 | A:1;G:0;C:1260;T:37118;total:38379 | iSNV |
| F30 | F30-26 | 10416 | 3'-UTR | 0.0294 | A:39599;G:1202;C:2;T:4;total:40807 | iSNV |
| F30 | F30-28 | 235 | C | 0.0899 | A:3;G:2;C:3611;T:36540;total:40156 | iSNV |
| F30 | F30-28 | 307 | C | 0.2431 | A:0;G:2;C:27999;T:8996;total:36997 | iSNV |
| F30 | F30-28 | 323 | C | 0.0483 | A:1632;G:32141;C:1;T:1;total:33775 | iSNV |
| F30 | F30-28 | 650 | M | 0.0288 | A:777;G:26197;C:2;T:0;total:26976 | iSNV |
| F30 | F30-28 | 982 | E | 0.1809 | A:21225;G:3;C:4689;T:2;total:25919 | iSNV |
| F30 | F30-28 | 1296 | E | 0.0273 | A:1;G:0;C:810;T:28826;total:29637 | iSNV |
| F30 | F30-28 | 1417 | E | 0.0507 | A:0;G:1;C:29183;T:1559;total:30743 | iSNV |
| F30 | F30-28 | 1450 | E | 0.0291 | A:30869;G:32;C:929;T:7;total:31837 | iSNV |
| F30 | F30-28 | 1468 | E | 0.1284 | A:3875;G:0;C:26298;T:5;total:30178 | iSNV |
| F30 | F30-28 | 1786 | E | 0.2871 | A:0;G:2;C:9868;T:24495;total:34365 | iSNV |
| F30 | F30-28 | 1911 | E | 0.2134 | A:21409;G:5809;C:0;T:2;total:27220 | iSNV |
| F30 | F30-28 | 2161 | E | 0.0424 | A:27176;G:2;C:1207;T:16;total:28401 | iSNV |
| F30 | F30-28 | 2162 | E | 0.2905 | A:3;G:4;C:8203;T:20018;total:28228 | iSNV |
| F30 | F30-28 | 2437 | E | 0.2755 | A:0;G:0;C:17149;T:6523;total:23672 | iSNV |
| F30 | F30-28 | 3346 | NS1 | 0.0304 | A:36705;G:1152;C:5;T:0;total:37862 | iSNV |
| F30 | F30-28 | 3511 | NS1 | 0.0596 | A:4;G:22;C:1648;T:25936;total:27610 | iSNV |
| F30 | F30-28 | 3768 | NS2A | 0.1338 | A:3;G:5;C:4580;T:29621;total:34209 | iSNV |
| F30 | F30-28 | 4177 | NS2A | 0.1675 | A:0;G:3;C:17742;T:3571;total:21316 | iSNV |
| F30 | F30-28 | 4294 | NS2B | 0.0214 | A:27046;G:593;C:0;T:2;total:27641 | iSNV |
| F30 | F30-28 | 4394 | NS2B | 0.064 | A:2;G:0;C:27156;T:1860;total:29018 | iSNV |
| F30 | F30-28 | 5267 | NS3 | 0.0412 | A:6;G:2;C:1105;T:25705;total:26818 | iSNV |
| F30 | F30-28 | 5311 | NS3 | 0.2527 | A:0;G:171;C:6873;T:20148;total:27192 | iSNV |
| F30 | F30-28 | 6472 | NS4A | 0.0412 | A:0;G:3;C:1243;T:28917;total:30163 | iSNV |
| F30 | F30-28 | 7265 | NS4A | 0.3205 | A:9392;G:19904;C:0;T:0;total:29296 | iSNV |
| F30 | F30-28 | 7271 | NS4B | 0.0661 | A:1;G:0;C:1901;T:26844;total:28746 | iSNV |
| F30 | F30-28 | 7495 | NS4B | 0.0823 | A:0;G:4;C:2261;T:25197;total:27462 | iSNV |
| F30 | F30-28 | 7657 | NS4B | 0.8901 | A:4035;G:32639;C:9;T:1;total:36684 | iSNV |
| F30 | F30-28 | 8410 | NS5 | 0.0333 | A:42438;G:1466;C:0;T:0;total:43904 | iSNV |
| F30 | F30-28 | 8456 | NS5 | 0.317 | A:31754;G:10;C:7;T:14752;total:46523 | iSNV |
| F30 | F30-28 | 9584 | NS5 | 0.2005 | A:0;G:1;C:33281;T:8347;total:41629 | iSNV |
| F30 | F30-28 | 9688 | NS5 | 0.251 | A:1;G:0;C:29784;T:9984;total:39769 | iSNV |
| F30 | F30-28 | 9998 | NS5 | 0.0437 | A:2;G:0;C:30335;T:1389;total:31726 | iSNV |
| F30 | F30-28 | 10069 | NS5 | 0.0264 | A:0;G:0;C:30117;T:817;total:30934 | iSNV |
| F30 | F30-28 | 10071 | NS5 | 0.0455 | A:1418;G:0;C:1;T:29739;total:31158 | iSNV |
| F30 | F30-28 | 10079 | NS5 | 0.1239 | A:27163;G:3854;C:1;T:70;total:31088 | iSNV |
| F30 | F30-28 | 10092 | NS5 | 0.3752 | A:12144;G:20203;C:6;T:8;total:32361 | iSNV |
| F30 | F30-28 | 10095 | NS5 | 0.0337 | A:0;G:32534;C:1137;T:1;total:33672 | iSNV |
| F30 | F30-28 | 10397 | 3'-UTR | 0.043 | A:4;G:4;C:1429;T:31749;total:33186 | iSNV |
| F30 | F30-28 | 10447 | 3'-UTR | 0.025 | A:720;G:1;C:28001;T:11;total:28733 | iSNV |
| F30 | F30-30 | 323 | C | 0.0233 | A:1304;G:54482;C:1;T:1;total:55788 | iSNV |
| F30 | F30-30 | 785 | M | 0.1332 | A:39193;G:6024;C:2;T:0;total:45219 | iSNV |
| F30 | F30-30 | 1181 | E | 0.0666 | A:3;G:3;C:46312;T:3308;total:49626 | iSNV |
| F30 | F30-30 | 1296 | E | 0.1044 | A:2;G:0;C:4882;T:41858;total:46742 | iSNV |
| F30 | F30-30 | 1417 | E | 0.0605 | A:1;G:0;C:45939;T:2959;total:48899 | iSNV |
| F30 | F30-30 | 1461 | E | 0.138 | A:3;G:0;C:6930;T:43262;total:50195 | iSNV |
| F30 | F30-30 | 1563 | E | 0.0474 | A:1;G:2;C:44596;T:2221;total:46820 | iSNV |
| F30 | F30-30 | 1911 | E | 0.3939 | A:27800;G:18078;C:15;T:0;total:45893 | iSNV |
| F30 | F30-30 | 2129 | E | 0.8022 | A:9222;G:37381;C:1;T:9;total:46613 | iSNV |

| | | | | | | |
|---|---|---|---|---|---|---|
| F30 | F30-30 | 2291 | E | 0.0641 | A:39593;G:2716;C:0;T:4;total:42313 | iSNV |
| F30 | F30-30 | 2510 | NS1 | 0.0497 | A:36119;G:1891;C:0;T:3;total:38013 | iSNV |
| F30 | F30-30 | 3001 | NS1 | 0.0256 | A:1182;G:44962;C:3;T:6;total:46153 | iSNV |
| F30 | F30-30 | 3230 | NS1 | 0.6741 | A:22157;G:45821;C:3;T:0;total:67981 | iSNV |
| F30 | F30-30 | 3497 | NS1 | 0.0465 | A:5;G:2047;C:14;T:41878;total:43944 | iSNV |
| F30 | F30-30 | 3633 | NS1 | 0.0626 | A:52657;G:3519;C:0;T:1;total:56177 | iSNV |
| F30 | F30-30 | 3934 | NS2A | 0.0389 | A:1;G:4;C:2337;T:57659;total:60001 | iSNV |
| F30 | F30-30 | 3975 | NS2A | 0.1081 | A:17;G:6654;C:30;T:54825;total:61526 | iSNV |
| F30 | F30-30 | 4060 | NS2A | 0.0483 | A:1;G:2;C:2319;T:45596;total:47918 | iSNV |
| F30 | F30-30 | 4385 | NS2B | 0.159 | A:2;G:3;C:48200;T:9117;total:57322 | iSNV |
| F30 | F30-30 | 4974 | NS3 | 0.0493 | A:3224;G:62081;C:5;T:0;total:65310 | iSNV |
| F30 | F30-30 | 5311 | NS3 | 0.1057 | A:1;G:530;C:5664;T:47374;total:53569 | iSNV |
| F30 | F30-30 | 6533 | NS4A | 0.6942 | A:1;G:0;C:35124;T:15479;total:50604 | iSNV |
| F30 | F30-30 | 7373 | NS4B | 0.1608 | A:2946;G:37898;C:66;T:7843;total:48753 | iSNV |
| F30 | F30-30 | 7378 | NS4B | 0.0443 | A:0;G:0;C:49082;T:2276;total:51358 | iSNV |
| F30 | F30-30 | 7381 | NS4B | 0.0215 | A:0;G:0;C:50172;T:1105;total:51277 | iSNV |
| F30 | F30-30 | 7495 | NS4B | 0.1107 | A:2;G:4;C:4782;T:38387;total:43175 | iSNV |
| F30 | F30-30 | 7546 | NS4B | 0.0548 | A:49002;G:2853;C:110;T:3;total:51968 | iSNV |
| F30 | F30-30 | 7656 | NS4B | 0.0207 | A:157;G:54103;C:1150;T:5;total:55415 | iSNV |
| F30 | F30-30 | 7657 | NS4B | 0.8476 | A:8405;G:46727;C:4;T:0;total:55136 | iSNV |
| F30 | F30-30 | 8372 | NS5 | 0.0461 | A:6;G:5;C:3012;T:62188;total:65211 | iSNV |
| F30 | F30-30 | 9688 | NS5 | 0.7298 | A:2;G:1;C:17865;T:48229;total:66097 | iSNV |
| F30 | F30-30 | 9932 | NS5 | 0.1096 | A:5929;G:0;C:19;T:48120;total:54068 | iSNV |
| F30 | F30-30 | 10069 | NS5 | 0.0447 | A:0;G:0;C:46600;T:2181;total:48781 | iSNV |
| F30 | F30-30 | 10071 | NS5 | 0.0449 | A:2209;G:0;C:0;T:46906;total:49115 | iSNV |
| F30 | F30-30 | 10086 | NS5 | 0.1164 | A:5710;G:1;C:1;T:43315;total:49027 | iSNV |
| F30 | F30-30 | 10723 | 3'-UTR | 0.0474 | A:2;G:5;C:56378;T:2808;total:59193 | iSNV |
| F30 | F30-4 | 806 | M | 0.0325 | A:8;G:2;C:1134;T:33740;total:34884 | iSNV |
| F30 | F30-4 | 1296 | E | 0.0536 | A:5;G:0;C:2066;T:36402;total:38473 | iSNV |
| F30 | F30-4 | 1474 | E | 0.1139 | A:37409;G:6;C:4814;T:2;total:42231 | iSNV |
| F30 | F30-4 | 1679 | E | 0.1277 | A:31594;G:4629;C:0;T:2;total:36225 | iSNV |
| F30 | F30-4 | 1786 | E | 0.0223 | A:7;G:1;C:1032;T:45215;total:46255 | iSNV |
| F30 | F30-4 | 1911 | E | 0.1942 | A:30959;G:7465;C:14;T:0;total:38438 | iSNV |
| F30 | F30-4 | 1913 | E | 0.0416 | A:35974;G:5;C:1562;T:3;total:37544 | iSNV |
| F30 | F30-4 | 2037 | E | 0.0593 | A:1907;G:1;C:30193;T:14;total:32115 | iSNV |
| F30 | F30-4 | 3006 | NS1 | 0.0322 | A:1300;G:39027;C:3;T:4;total:40334 | iSNV |
| F30 | F30-4 | 3137 | NS1 | 0.0401 | A:4;G:1;C:42288;T:1771;total:44064 | iSNV |
| F30 | F30-4 | 3428 | NS1 | 0.4549 | A:7;G:1;C:23523;T:19640;total:43171 | iSNV |
| F30 | F30-4 | 3761 | NS2A | 0.08 | A:2;G:4;C:37779;T:3287;total:41072 | iSNV |
| F30 | F30-4 | 4068 | NS2A | 0.0414 | A:32927;G:1426;C:1;T:8;total:34362 | iSNV |
| F30 | F30-4 | 4232 | NS2B | 0.0463 | A:852;G:17517;C:2;T:0;total:18371 | iSNV |
| F30 | F30-4 | 5180 | NS3 | 0.0255 | A:499;G:19022;C:1;T:3;total:19525 | iSNV |
| F30 | F30-4 | 5311 | NS3 | 0.369 | A:0;G:325;C:8317;T:13896;total:22538 | iSNV |
| F30 | F30-4 | 5835 | NS3 | 0.0229 | A:668;G:28409;C:0;T:0;total:29077 | iSNV |
| F30 | F30-4 | 5946 | NS3 | 0.0982 | A:2666;G:0;C:24461;T:4;total:27131 | iSNV |
| F30 | F30-4 | 6533 | NS4A | 0.0565 | A:1;G:1;C:1997;T:33337;total:35336 | iSNV |
| F30 | F30-4 | 6738 | NS4A | 0.1295 | A:4;G:0;C:4838;T:32504;total:37346 | iSNV |
| F30 | F30-4 | 7007 | NS4A | 0.0282 | A:1168;G:40188;C:2;T:3;total:41361 | iSNV |
| F30 | F30-4 | 7061 | NS4A | 0.0383 | A:1;G:4;C:43093;T:1719;total:44817 | iSNV |
| F30 | F30-4 | 7381 | NS4B | 0.092 | A:2;G:3;C:33441;T:3391;total:36837 | iSNV |
| F30 | F30-4 | 7495 | NS4B | 0.1233 | A:2;G:2;C:3968;T:28186;total:32158 | iSNV |
| F30 | F30-4 | 7656 | NS4B | 0.1286 | A:5464;G:36871;C:142;T:2;total:42479 | iSNV |
| F30 | F30-4 | 7657 | NS4B | 0.6329 | A:15540;G:26768;C:13;T:1;total:42322 | iSNV |
| F30 | F30-4 | 8153 | NS5 | 0.0297 | A:48830;G:1495;C:0;T:1;total:50326 | iSNV |
| F30 | F30-4 | 8780 | NS5 | 0.0402 | A:39271;G:13;C:19;T:1649;total:40952 | iSNV |
| F30 | F30-4 | 8891 | NS5 | 0.0354 | A:1;G:0;C:1404;T:38228;total:39633 | iSNV |
| F30 | F30-4 | 9292 | NS5 | 0.0468 | A:6;G:50086;C:2461;T:2;total:52555 | iSNV |
| F30 | F30-4 | 9688 | NS5 | 0.0831 | A:2;G:1;C:47389;T:4296;total:51688 | iSNV |
| F30 | F30-4 | 9932 | NS5 | 0.0425 | A:1946;G:2;C:17;T:43727;total:45692 | iSNV |
| F30 | F30-4 | 10068 | NS5 | 0.0509 | A:41281;G:2218;C:0;T:2;total:43501 | iSNV |
| F30 | F30-4 | 10069 | NS5 | 0.2037 | A:2;G:0;C:34278;T:8770;total:43050 | iSNV |
| F30 | F30-4 | 10632 | 3'-UTR | 0.0647 | A:27;G:15;C:2880;T:41586;total:44508 | iSNV |
| F30 | F30-4 | 10664 | 3'-UTR | 0.0247 | A:1236;G:48716;C:2;T:2;total:49956 | iSNV |
| F30 | F30-4 | 10712 | 3'-UTR | 0.0341 | A:3;G:1;C:46533;T:1646;total:48183 | iSNV |
| F30 | F30-5 | 539 | M | 0.0266 | A:1941;G:8;C:70799;T:35;total:72783 | iSNV |
| F30 | F30-5 | 1296 | E | 0.16 | A:2;G:0;C:9398;T:49310;total:58710 | iSNV |
| F30 | F30-5 | 1468 | E | 0.0697 | A:3561;G:1;C:47482;T:22;total:51066 | iSNV |
| F30 | F30-5 | 1911 | E | 0.8284 | A:6164;G:29741;C:9;T:1;total:35915 | iSNV |
| F30 | F30-5 | 1946 | E | 0.0627 | A:3;G:0;C:37163;T:2487;total:39653 | iSNV |
| F30 | F30-5 | 3081 | NS1 | 0.2653 | A:46242;G:16707;C:8;T:0;total:62957 | iSNV |
| F30 | F30-5 | 4069 | NS2A | 0.0442 | A:7;G:2169;C:70;T:46746;total:48992 | iSNV |
| F30 | F30-5 | 4550 | NS2B | 0.0399 | A:8;G:5;C:2445;T:58784;total:61242 | iSNV |
| F30 | F30-5 | 5311 | NS3 | 0.8524 | A:1;G:215;C:45586;T:7932;total:53734 | iSNV |
| F30 | F30-5 | 6637 | NS4A | 0.5375 | A:4;G:3;C:23856;T:20535;total:44398 | iSNV |
| F30 | F30-5 | 7373 | NS4B | 0.024 | A:1080;G:43760;C:0;T:9;total:44849 | iSNV |
| F30 | F30-5 | 7495 | NS4B | 0.0679 | A:0;G:0;C:2574;T:35288;total:37862 | iSNV |
| F30 | F30-5 | 7543 | NS4B | 0.0753 | A:45569;G:3714;C:0;T:1;total:49284 | iSNV |
| F30 | F30-5 | 7656 | NS4B | 0.7885 | A:2665;G:13023;C:45856;T:12;total:6155[illegible] | iSNV |
| F30 | F30-5 | 7657 | NS4B | 0.1018 | A:55442;G:6288;C:1;T:3;total:61734 | iSNV |
| F30 | F30-5 | 8093 | NS5 | 0.0637 | A:4;G:2;C:65311;T:4446;total:69763 | iSNV |
| F30 | F30-5 | 9688 | NS5 | 0.1121 | A:0;G:0;C:35425;T:4474;total:39899 | iSNV |
| F30 | F30-5 | 9887 | NS5 | 0.1038 | A:4;G:1;C:6458;T:55719;total:62182 | iSNV |

| | | | | | | |
|---|---|---|---|---|---|---|
| F30 | F30-5 | 10092 | NS5 | 0.1557 | A:1009;G:54156;C:10174;T:4;total:65343 | iSNV |
| F30 | F30-7 | 505 | M | 0.2322 | A:4;G:5;C:12439;T:41122;total:53570 | iSNV |
| F30 | F30-7 | 533 | M | 0.0243 | A:15;G:0;C:1168;T:46845;total:48028 | iSNV |
| F30 | F30-7 | 697 | M | 0.0338 | A:2;G:0;C:1480;T:42249;total:43731 | iSNV |
| F30 | F30-7 | 1116 | E | 0.031 | A:40861;G:1311;C:0;T:5;total:42177 | iSNV |
| F30 | F30-7 | 1296 | E | 0.036 | A:1;G:3;C:1366;T:36574;total:37944 | iSNV |
| F30 | F30-7 | 1417 | E | 0.0293 | A:3;G:2;C:41315;T:1250;total:42570 | iSNV |
| F30 | F30-7 | 1911 | E | 0.6609 | A:10696;G:20767;C:77;T:0;total:31540 | iSNV |
| F30 | F30-7 | 3139 | NS1 | 0.4876 | A:22150;G:21089;C:0;T:5;total:43244 | iSNV |
| F30 | F30-7 | 3800 | NS2A | 0.0337 | A:0;G:1;C:1351;T:38675;total:40027 | iSNV |
| F30 | F30-7 | 4192 | NS2A | 0.0215 | A:1;G:0;C:585;T:26580;total:27166 | iSNV |
| F30 | F30-7 | 5311 | NS3 | 0.2214 | A:1;G:306;C:8931;T:31094;total:40332 | iSNV |
| F30 | F30-7 | 5835 | NS3 | 0.0394 | A:2277;G:55439;C:1;T:1;total:57718 | iSNV |
| F30 | F30-7 | 6533 | NS4A | 0.4775 | A:5;G:0;C:17997;T:19683;total:37685 | iSNV |
| F30 | F30-7 | 7495 | NS4B | 0.4705 | A:1;G:1;C:12480;T:14039;total:26521 | iSNV |
| F30 | F30-7 | 7543 | NS4B | 0.2024 | A:26953;G:6843;C:2;T:2;total:33800 | iSNV |
| F30 | F30-7 | 7657 | NS4B | 0.327 | A:28604;G:13899;C:1;T:0;total:42504 | iSNV |
| F30 | F30-7 | 8140 | NS5 | 0.2979 | A:39936;G:16953;C:2;T:4;total:56895 | iSNV |
| F30 | F30-7 | 9254 | NS5 | 0.0349 | A:52975;G:1919;C:0;T:3;total:54897 | iSNV |
| F30 | F30-7 | 9370 | NS5 | 0.3112 | A:17841;G:5;C:18;T:39452;total:57316 | iSNV |
| F30 | F30-7 | 9688 | NS5 | 0.9911 | A:1;G:0;C:363;T:40374;total:40738 | SNP |
| F30 | F30-7 | 10069 | NS5 | 0.0888 | A:0;G:0;C:37399;T:3647;total:41046 | iSNV |
| F30 | F30-7 | 10379 | NS5 | 0.1688 | A:38719;G:7868;C:1;T:0;total:46588 | iSNV |
| F30 | F30-8 | 504 | M | 0.1004 | A:0;G:2948;C:26382;T:4;total:29334 | iSNV |
| F30 | F30-8 | 761 | M | 0.0377 | A:810;G:20650;C:0;T:1;total:21461 | iSNV |
| F30 | F30-8 | 798 | M | 0.0603 | A:16266;G:2;C:1045;T:0;total:17313 | iSNV |
| F30 | F30-8 | 1241 | E | 0.0223 | A:2;G:0;C:461;T:20205;total:20668 | iSNV |
| F30 | F30-8 | 1296 | E | 0.1 | A:1;G:0;C:1861;T:16745;total:18607 | iSNV |
| F30 | F30-8 | 1417 | E | 0.2135 | A:3;G:0;C:17252;T:4685;total:21940 | iSNV |
| F30 | F30-8 | 1579 | E | 0.1153 | A:1;G:1;C:2167;T:16613;total:18782 | iSNV |
| F30 | F30-8 | 1660 | E | 0.1621 | A:2;G:0;C:15795;T:3058;total:18855 | iSNV |
| F30 | F30-8 | 1911 | E | 0.2806 | A:12981;G:5195;C:332;T:0;total:18508 | iSNV |
| F30 | F30-8 | 3538 | NS1 | 0.6501 | A:7540;G:2;C:14000;T:4;total:21546 | iSNV |
| F30 | F30-8 | 4023 | NS2A | 0.1104 | A:2509;G:20213;C:0;T:0;total:22722 | iSNV |
| F30 | F30-8 | 4401 | NS2B | 0.6324 | A:1;G:8058;C:13853;T:5;total:21917 | iSNV |
| F30 | F30-8 | 4896 | NS3 | 0.0893 | A:2568;G:26168;C:0;T:0;total:28736 | iSNV |
| F30 | F30-8 | 5311 | NS3 | 0.024 | A:0;G:35;C:570;T:23103;total:23708 | iSNV |
| F30 | F30-8 | 5528 | NS3 | 0.6431 | A:9988;G:17983;C:3;T:9;total:27983 | iSNV |
| F30 | F30-8 | 5835 | NS3 | 0.0389 | A:1169;G:28869;C:0;T:0;total:30038 | iSNV |
| F30 | F30-8 | 6533 | NS4A | 0.9422 | A:2;G:0;C:20671;T:1269;total:21942 | iSNV |
| F30 | F30-8 | 7381 | NS4B | 0.0984 | A:0;G:0;C:18490;T:2018;total:20508 | iSNV |
| F30 | F30-8 | 7657 | NS4B | 0.9884 | A:268;G:23061;C:2;T:1;total:23332 | SNP |
| F30 | F30-8 | 8140 | NS5 | 0.2356 | A:24022;G:7407;C:3;T:2;total:31434 | iSNV |
| F30 | F30-8 | 8534 | NS5 | 0.0312 | A:3;G:1;C:1146;T:35478;total:36628 | iSNV |
| F30 | F30-8 | 9221 | NS5 | 0.1121 | A:28919;G:9;C:3654;T:3;total:32585 | iSNV |
| F30 | F30-8 | 9688 | NS5 | 0.9495 | A:3;G:0;C:1605;T:30134;total:31742 | iSNV |
| F30 | F30-8 | 10080 | NS5 | 0.0398 | A:23848;G:1;C:991;T:4;total:24844 | iSNV |
| F30 | F30-8 | 10419 | 3'-UTR | 0.941 | A:1;G:10;C:1532;T:24408;total:25951 | iSNV |
| F35 | F35-1 | 1116 | E | 0.1732 | A:55585;G:11650;C:2;T:3;total:67240 | iSNV |
| F35 | F35-1 | 1286 | E | 0.1601 | A:2;G:1;C:51890;T:9898;total:61791 | iSNV |
| F35 | F35-1 | 1296 | E | 0.2064 | A:7;G:0;C:13325;T:51217;total:64549 | iSNV |
| F35 | F35-1 | 1384 | E | 0.0989 | A:67666;G:10;C:5;T:7436;total:75117 | iSNV |
| F35 | F35-1 | 1390 | E | 0.1053 | A:65892;G:7764;C:10;T:0;total:73666 | iSNV |
| F35 | F35-1 | 1417 | E | 0.1612 | A:3;G:4;C:65883;T:12668;total:78558 | iSNV |
| F35 | F35-1 | 1491 | E | 0.105 | A:66571;G:7811;C:7;T:1;total:74390 | iSNV |
| F35 | F35-1 | 1800 | E | 0.1032 | A:8693;G:19;C:19;T:75466;total:84197 | iSNV |
| F35 | F35-1 | 1911 | E | 0.5683 | A:27566;G:36279;C:5;T:3;total:63853 | iSNV |
| F35 | F35-1 | 2531 | NS1 | 0.3013 | A:3;G:2;C:43125;T:18599;total:61729 | iSNV |
| F35 | F35-1 | 3097 | NS1 | 0.0209 | A:65652;G:22;C:1402;T:1;total:67077 | iSNV |
| F35 | F35-1 | 3428 | NS1 | 0.0668 | A:3;G:1;C:61366;T:4397;total:65767 | iSNV |
| F35 | F35-1 | 4021 | NS2A | 0.3588 | A:23285;G:41584;C:5;T:7;total:64881 | iSNV |
| F35 | F35-1 | 4367 | NS2B | 0.1763 | A:2;G:16;C:11021;T:51461;total:62500 | iSNV |
| F35 | F35-1 | 5311 | NS3 | 0.061 | A:5;G:470;C:3725;T:56848;total:61048 | iSNV |
| F35 | F35-1 | 6533 | NS4A | 0.5713 | A:3;G:0;C:32743;T:24577;total:57323 | iSNV |
| F35 | F35-1 | 6738 | NS4A | 0.0516 | A:14;G:5;C:2947;T:54063;total:57029 | iSNV |
| F35 | F35-1 | 7381 | NS4B | 0.0608 | A:1;G:3;C:53894;T:3493;total:57391 | iSNV |
| F35 | F35-1 | 7495 | NS4B | 0.0261 | A:3;G:3;C:1278;T:47554;total:48838 | iSNV |
| F35 | F35-1 | 7546 | NS4B | 0.3558 | A:36766;G:20329;C:25;T:1;total:57121 | iSNV |
| F35 | F35-1 | 7656 | NS4B | 0.02 | A:1248;G:61062;C:42;T:1;total:62353 | iSNV |
| F35 | F35-1 | 7657 | NS4B | 0.8384 | A:10034;G:52053;C:1;T:1;total:62089 | iSNV |
| F35 | F35-1 | 8194 | NS5 | 0.1006 | A:65034;G:7280;C:12;T:19;total:72345 | iSNV |
| F35 | F35-1 | 8600 | NS5 | 0.0889 | A:2;G:5;C:78008;T:7617;total:85632 | iSNV |
| F35 | F35-1 | 9220 | NS5 | 0.1659 | A:70758;G:6;C:14083;T:1;total:84848 | iSNV |
| F35 | F35-1 | 9225 | NS5 | 0.0952 | A:8093;G:4;C:76865;T:30;total:84992 | iSNV |
| F35 | F35-1 | 9688 | NS5 | 0.597 | A:6;G:1;C:30981;T:45873;total:76861 | iSNV |
| F35 | F35-1 | 10069 | NS5 | 0.2048 | A:0;G:2;C:47353;T:12201;total:59556 | iSNV |
| F35 | F35-1 | 10632 | 3'-UTR | 0.2004 | A:24;G:17;C:12735;T:50761;total:63537 | iSNV |
| F35 | F35-10 | 400 | C | 0.02 | A:75044;G:1532;C:9;T:5;total:76590 | iSNV |
| F35 | F35-10 | 820 | M | 0.02 | A:1165;G:18;C:56800;T:27;total:58010 | iSNV |
| F35 | F35-10 | 821 | M | 0.0213 | A:19;G:56200;C:1229;T:2;total:57450 | iSNV |
| F35 | F35-10 | 1247 | E | 0.4072 | A:4;G:3;C:26229;T:38176;total:64412 | iSNV |
| F35 | F35-10 | 1296 | E | 0.0334 | A:2;G:3;C:2239;T:64637;total:66881 | iSNV |

| | | | | | | |
|---|---|---|---|---|---|---|
| F35 | F35-10 | 1342 | E | 0.2137 | A:2;G:2;C:55722;T:15148;total:70874 | iSNV |
| F35 | F35-10 | 1347 | E | 0.2102 | A:55074;G:14663;C:1;T:3;total:69741 | iSNV |
| F35 | F35-10 | 1359 | E | 0.2002 | A:55990;G:14021;C:2;T:3;total:70016 | iSNV |
| F35 | F35-10 | 1384 | E | 0.2826 | A:51964;G:4;C:12;T:20480;total:72460 | iSNV |
| F35 | F35-10 | 1390 | E | 0.2963 | A:50455;G:21266;C:46;T:2;total:71769 | iSNV |
| F35 | F35-10 | 1491 | E | 0.2906 | A:51358;G:21044;C:2;T:7;total:72411 | iSNV |
| F35 | F35-10 | 1708 | E | 0.2233 | A:17046;G:59277;C:0;T:10;total:76333 | iSNV |
| F35 | F35-10 | 1800 | E | 0.3048 | A:25718;G:2;C:30;T:58623;total:84373 | iSNV |
| F35 | F35-10 | 1911 | E | 0.4124 | A:37173;G:26100;C:1;T:0;total:63274 | iSNV |
| F35 | F35-10 | 2924 | NS1 | 0.2275 | A:2;G:0;C:19345;T:65671;total:85018 | iSNV |
| F35 | F35-10 | 3113 | NS1 | 0.4061 | A:23;G:1;C:26395;T:38567;total:64986 | iSNV |
| F35 | F35-10 | 3350 | NS1 | 0.0217 | A:4;G:0;C:1428;T:64119;total:65551 | iSNV |
| F35 | F35-10 | 3932 | NS2A | 0.0317 | A:0;G:8;C:2309;T:70509;total:72826 | iSNV |
| F35 | F35-10 | 5311 | NS3 | 0.42 | A:1;G:53;C:26102;T:35981;total:62137 | iSNV |
| F35 | F35-10 | 6707 | NS4A | 0.0216 | A:94;G:4;C:1207;T:54533;total:55838 | iSNV |
| F35 | F35-10 | 7656 | NS4B | 0.4149 | A:26512;G:37381;C:0;T:2;total:63895 | iSNV |
| F35 | F35-10 | 7657 | NS4B | 0.2594 | A:47448;G:16629;C:8;T:1;total:64086 | iSNV |
| F35 | F35-10 | 8140 | NS5 | 0.2564 | A:59927;G:1042;C:21026;T:8;total:82003 | iSNV |
| F35 | F35-10 | 9225 | NS5 | 0.2981 | A:24789;G:3;C:58340;T:14;total:83146 | iSNV |
| F35 | F35-10 | 10068 | NS5 | 0.2408 | A:44461;G:14103;C:0;T:3;total:58567 | iSNV |
| F35 | F35-10 | 10069 | NS5 | 0.404 | A:1;G:0;C:34706;T:23528;total:58235 | iSNV |
| F35 | F35-11 | 918 | M | 0.026 | A:53317;G:1428;C:2;T:1;total:54748 | iSNV |
| F35 | F35-11 | 1292 | E | 0.4844 | A:1;G:2;C:28012;T:29812;total:57827 | iSNV |
| F35 | F35-11 | 1417 | E | 0.491 | A:4;G:2;C:35133;T:33904;total:69043 | iSNV |
| F35 | F35-11 | 1468 | E | 0.0289 | A:1968;G:3;C:65868;T:34;total:67873 | iSNV |
| F35 | F35-11 | 1544 | E | 0.4958 | A:1;G:4;C:31467;T:30958;total:62430 | iSNV |
| F35 | F35-11 | 1911 | E | 0.4289 | A:32239;G:24223;C:4;T:1;total:56467 | iSNV |
| F35 | F35-11 | 2306 | E | 0.0252 | A:50866;G:1317;C:3;T:3;total:52189 | iSNV |
| F35 | F35-11 | 5271 | NS3 | 0.3762 | A:0;G:1;C:21123;T:35015;total:56139 | iSNV |
| F35 | F35-11 | 5311 | NS3 | 0.5081 | A:1;G:26;C:27816;T:26957;total:54800 | iSNV |
| F35 | F35-11 | 5705 | NS3 | 0.0252 | A:70447;G:1825;C:1;T:3;total:72276 | iSNV |
| F35 | F35-11 | 6533 | NS4A | 0.5047 | A:2;G:0;C:26386;T:25901;total:52289 | iSNV |
| F35 | F35-11 | 7091 | NS4A | 0.5005 | A:4;G:0;C:28732;T:28784;total:57520 | iSNV |
| F35 | F35-11 | 7373 | NS4B | 0.3799 | A:17419;G:28422;C:0;T:0;total:45841 | iSNV |
| F35 | F35-11 | 7495 | NS4B | 0.0253 | A:3;G:1;C:1063;T:40784;total:41851 | iSNV |
| F35 | F35-11 | 7657 | NS4B | 0.9628 | A:1986;G:51277;C:4;T:1;total:53268 | iSNV |
| F35 | F35-11 | 7850 | NS5 | 0.5074 | A:1;G:1;C:28954;T:29820;total:58776 | iSNV |
| F35 | F35-11 | 7919 | NS5 | 0.0277 | A:1935;G:67877;C:0;T:0;total:69812 | iSNV |
| F35 | F35-11 | 8449 | NS5 | 0.5036 | A:2;G:2;C:37647;T:37123;total:74774 | iSNV |
| F35 | F35-11 | 9224 | NS5 | 0.0391 | A:2873;G:70491;C:6;T:1;total:73371 | iSNV |
| F35 | F35-11 | 9688 | NS5 | 0.5299 | A:2;G:1;C:29412;T:33148;total:62563 | iSNV |
| F35 | F35-11 | 10069 | NS5 | 0.4389 | A:0;G:0;C:27589;T:21589;total:49178 | iSNV |
| F35 | F35-11 | 10092 | NS5 | 0.03 | A:1571;G:50628;C:2;T:4;total:52205 | iSNV |
| F35 | F35-13 | 175 | C | 0.0366 | A:8;G:22;C:2342;T:61513;total:63885 | iSNV |
| F35 | F35-13 | 245 | C | 0.0274 | A:2;G:1;C:1818;T:64307;total:66128 | iSNV |
| F35 | F35-13 | 344 | C | 0.142 | A:3;G:9015;C:6;T:54461;total:63485 | iSNV |
| F35 | F35-13 | 503 | M | 0.0362 | A:2644;G:70232;C:5;T:3;total:72884 | iSNV |
| F35 | F35-13 | 796 | M | 0.0287 | A:47678;G:1410;C:0;T:16;total:49104 | iSNV |
| F35 | F35-13 | 861 | M | 0.0315 | A:53075;G:1727;C:0;T:6;total:54808 | iSNV |
| F35 | F35-13 | 875 | M | 0.0271 | A:2;G:6;C:56654;T:1584;total:58246 | iSNV |
| F35 | F35-13 | 926 | M | 0.1112 | A:4;G:1;C:5499;T:43946;total:49450 | iSNV |
| F35 | F35-13 | 1296 | E | 0.133 | A:3;G:0;C:7707;T:50199;total:57909 | iSNV |
| F35 | F35-13 | 1348 | E | 0.1785 | A:50649;G:11011;C:2;T:0;total:61662 | iSNV |
| F35 | F35-13 | 1354 | E | 0.0276 | A:2;G:3;C:1668;T:58638;total:60311 | iSNV |
| F35 | F35-13 | 1450 | E | 0.0347 | A:65188;G:7;C:2349;T:2;total:67546 | iSNV |
| F35 | F35-13 | 1468 | E | 0.0253 | A:1635;G:5;C:62743;T:21;total:64404 | iSNV |
| F35 | F35-13 | 1619 | E | 0.0367 | A:1829;G:15;C:6;T:47913;total:49763 | iSNV |
| F35 | F35-13 | 1803 | E | 0.2187 | A:54836;G:15351;C:0;T:3;total:70190 | iSNV |
| F35 | F35-13 | 1911 | E | 0.8364 | A:7955;G:40656;C:2;T:3;total:48616 | iSNV |
| F35 | F35-13 | 2181 | E | 0.0308 | A:54698;G:1742;C:1;T:5;total:56446 | iSNV |
| F35 | F35-13 | 2467 | E | 0.0702 | A:42448;G:3208;C:2;T:1;total:45659 | iSNV |
| F35 | F35-13 | 2636 | NS1 | 0.0282 | A:4;G:2;C:62624;T:1823;total:64453 | iSNV |
| F35 | F35-13 | 2780 | NS1 | 0.0382 | A:17;G:1;C:3057;T:76942;total:80017 | iSNV |
| F35 | F35-13 | 3156 | NS1 | 0.0229 | A:1425;G:60749;C:0;T:3;total:62177 | iSNV |
| F35 | F35-13 | 3458 | NS1 | 0.0394 | A:6;G:0;C:1721;T:41912;total:43639 | iSNV |
| F35 | F35-13 | 3488 | NS1 | 0.1891 | A:7567;G:2;C:7;T:32439;total:40015 | iSNV |
| F35 | F35-13 | 3634 | NS1 | 0.0504 | A:6;G:4;C:2320;T:43620;total:45950 | iSNV |
| F35 | F35-13 | 3652 | NS1 | 0.153 | A:3;G:0;C:6724;T:37205;total:43932 | iSNV |
| F35 | F35-13 | 3675 | NS1 | 0.0281 | A:1173;G:40561;C:1;T:1;total:41736 | iSNV |
| F35 | F35-13 | 3773 | NS2A | 0.0372 | A:1;G:0;C:1753;T:45335;total:47089 | iSNV |
| F35 | F35-13 | 3963 | NS2A | 0.0353 | A:1;G:4;C:2116;T:57708;total:59829 | iSNV |
| F35 | F35-13 | 4198 | NS2A | 0.0805 | A:31341;G:2745;C:2;T:0;total:34088 | iSNV |
| F35 | F35-13 | 4343 | NS2B | 0.0291 | A:1;G:3;C:54347;T:1632;total:55983 | iSNV |
| F35 | F35-13 | 4470 | NS2B | 0.0211 | A:3;G:3;C:49605;T:1072;total:50683 | iSNV |
| F35 | F35-13 | 4766 | NS3 | 0.0229 | A:11;G:6;C:1475;T:62647;total:64139 | iSNV |
| F35 | F35-13 | 4943 | NS3 | 0.763 | A:14226;G:45781;C:1;T:6;total:60014 | iSNV |
| F35 | F35-13 | 5271 | NS3 | 0.0216 | A:1;G:2;C:1149;T:51809;total:52961 | iSNV |
| F35 | F35-13 | 5311 | NS3 | 0.776 | A:1;G:2143;C:36910;T:11277;total:50331 | iSNV |
| F35 | F35-13 | 5358 | NS3 | 0.2209 | A:0;G:42538;C:12066;T:5;total:54609 | iSNV |
| F35 | F35-13 | 5747 | NS3 | 0.0338 | A:2440;G:69665;C:0;T:1;total:72106 | iSNV |
| F35 | F35-13 | 5993 | NS3 | 0.1527 | A:48869;G:8811;C:3;T:1;total:57684 | iSNV |
| F35 | F35-13 | 6227 | NS3 | 0.035 | A:2106;G:57954;C:1;T:3;total:60064 | iSNV |

| | | | | | | |
|---|---|---|---|---|---|---|
| F35 | F35-13 | 6568 | NS4A | 0.0344 | A:46959;G:1674;C:4;T:1;total:48638 | iSNV |
| F35 | F35-13 | 6624 | NS4A | 0.0233 | A:49670;G:6;C:1190;T:3;total:50869 | iSNV |
| F35 | F35-13 | 7067 | NS4A | 0.0491 | A:53642;G:2776;C:4;T:1;total:56423 | iSNV |
| F35 | F35-13 | 7229 | NS4A | 0.0789 | A:0;G:2;C:37979;T:3257;total:41238 | iSNV |
| F35 | F35-13 | 7495 | NS4B | 0.736 | A:2;G:1;C:26269;T:9424;total:35696 | iSNV |
| F35 | F35-13 | 7546 | NS4B | 0.2469 | A:33366;G:10945;C:5;T:3;total:44319 | iSNV |
| F35 | F35-13 | 7657 | NS4B | 0.159 | A:43546;G:8235;C:1;T:1;total:51783 | iSNV |
| F35 | F35-13 | 7658 | NS4B | 0.0319 | A:1;G:0;C:1653;T:50136;total:51790 | iSNV |
| F35 | F35-13 | 7952 | NS5 | 0.0317 | A:70091;G:8;C:8;T:2296;total:72403 | iSNV |
| F35 | F35-13 | 8105 | NS5 | 0.0294 | A:1883;G:62018;C:3;T:2;total:63906 | iSNV |
| F35 | F35-13 | 8115 | NS5 | 0.0487 | A:63861;G:3270;C:1;T:1;total:67133 | iSNV |
| F35 | F35-13 | 8927 | NS5 | 0.1578 | A:47083;G:8827;C:7;T:1;total:55918 | iSNV |
| F35 | F35-13 | 9026 | NS5 | 0.0202 | A:2;G:3;C:56239;T:1162;total:57406 | iSNV |
| F35 | F35-13 | 9224 | NS5 | 0.0349 | A:2489;G:68619;C:2;T:8;total:71118 | iSNV |
| F35 | F35-13 | 9587 | NS5 | 0.0272 | A:1716;G:61211;C:1;T:4;total:62932 | iSNV |
| F35 | F35-13 | 9688 | NS5 | 0.228 | A:4;G:3;C:42998;T:12708;total:55713 | iSNV |
| F35 | F35-13 | 10071 | NS5 | 0.2294 | A:10966;G:1;C:3;T:36830;total:47800 | iSNV |
| F35 | F35-13 | 10092 | NS5 | 0.7594 | A:36965;G:11716;C:1;T:2;total:48684 | iSNV |
| F35 | F35-13 | 10235 | NS5 | 0.0277 | A:1;G:0;C:61751;T:1764;total:63516 | iSNV |
| F35 | F35-13 | 10424 | 3'-UTR | 0.0233 | A:9;G:5;C:1273;T:53294;total:54581 | iSNV |
| F35 | F35-13 | 10611 | 3'-UTR | 0.0228 | A:50248;G:1175;C:0;T:0;total:51423 | iSNV |
| F35 | F35-13 | 10723 | 3'-UTR | 0.0333 | A:2;G:1;C:51689;T:1785;total:53477 | iSNV |
| F35 | F35-13 | 10794 | 3'-UTR | 0.0212 | A:1;G:1;C:41920;T:910;total:42832 | iSNV |
| F35 | F35-14 | 299 | C | 0.0836 | A:74166;G:10;C:6771;T:16;total:80963 | iSNV |
| F35 | F35-14 | 1296 | E | 0.4392 | A:1;G:0;C:31043;T:39621;total:70665 | iSNV |
| F35 | F35-14 | 1384 | E | 0.0587 | A:73629;G:49;C:8;T:4603;total:78289 | iSNV |
| F35 | F35-14 | 1390 | E | 0.0625 | A:70749;G:4723;C:9;T:1;total:75482 | iSNV |
| F35 | F35-14 | 1417 | E | 0.0396 | A:5;G:2;C:76663;T:3165;total:79835 | iSNV |
| F35 | F35-14 | 1491 | E | 0.0608 | A:66179;G:4290;C:7;T:5;total:70481 | iSNV |
| F35 | F35-14 | 1800 | E | 0.0616 | A:5398;G:0;C:26;T:82085;total:87509 | iSNV |
| F35 | F35-14 | 1911 | E | 0.8159 | A:11089;G:49118;C:7;T:0;total:60214 | iSNV |
| F35 | F35-14 | 3530 | NS1 | 0.0796 | A:4534;G:403;C:24;T:51932;total:56893 | iSNV |
| F35 | F35-14 | 3773 | NS2A | 0.0264 | A:63;G:0;C:1808;T:66462;total:68333 | iSNV |
| F35 | F35-14 | 4394 | NS2B | 0.0486 | A:6;G:3;C:65762;T:3360;total:69131 | iSNV |
| F35 | F35-14 | 5311 | NS3 | 0.2098 | A:2;G:1176;C:13998;T:51529;total:66705 | iSNV |
| F35 | F35-14 | 5612 | NS3 | 0.0708 | A:3;G:5;C:5544;T:72712;total:78264 | iSNV |
| F35 | F35-14 | 5835 | NS3 | 0.067 | A:5818;G:81001;C:2;T:2;total:86823 | iSNV |
| F35 | F35-14 | 5987 | NS3 | 0.023 | A:5;G:0;C:72821;T:1721;total:74547 | iSNV |
| F35 | F35-14 | 6979 | NS4A | 0.0631 | A:21;G:4722;C:69977;T:24;total:74744 | iSNV |
| F35 | F35-14 | 7381 | NS4B | 0.0561 | A:3;G:0;C:58480;T:3478;total:61961 | iSNV |
| F35 | F35-14 | 7546 | NS4B | 0.0209 | A:66739;G:1430;C:41;T:2;total:68212 | iSNV |
| F35 | F35-14 | 7656 | NS4B | 0.1213 | A:8854;G:63475;C:628;T:5;total:72962 | iSNV |
| F35 | F35-14 | 7657 | NS4B | 0.7879 | A:15432;G:57283;C:8;T:7;total:72730 | iSNV |
| F35 | F35-14 | 7724 | NS5 | 0.064 | A:61029;G:4174;C:5;T:3;total:65211 | iSNV |
| F35 | F35-14 | 9225 | NS5 | 0.0599 | A:5309;G:0;C:83209;T:26;total:88544 | iSNV |
| F35 | F35-14 | 9688 | NS5 | 0.037 | A:1;G:0;C:67326;T:2587;total:69914 | iSNV |
| F35 | F35-14 | 10069 | NS5 | 0.7835 | A:1;G:1;C:12669;T:45838;total:58509 | iSNV |
| F35 | F35-16 | 1296 | E | 0.1211 | A:2;G:1;C:12417;T:90102;total:102522 | iSNV |
| F35 | F35-16 | 1298 | E | 0.0339 | A:333;G:3436;C:87;T:97355;total:101211 | iSNV |
| F35 | F35-16 | 1417 | E | 0.2441 | A:3;G:1;C:81772;T:26408;total:108184 | iSNV |
| F35 | F35-16 | 1911 | E | 0.7039 | A:26407;G:62759;C:5;T:0;total:89171 | iSNV |
| F35 | F35-16 | 3096 | NS1 | 0.1463 | A:86749;G:54;C:14;T:14884;total:101701 | iSNV |
| F35 | F35-16 | 3452 | NS1 | 0.0262 | A:74469;G:2007;C:3;T:4;total:76483 | iSNV |
| F35 | F35-16 | 3453 | NS1 | 0.0284 | A:2184;G:74581;C:4;T:2;total:76771 | iSNV |
| F35 | F35-16 | 3685 | NS1 | 0.0937 | A:79508;G:8228;C:2;T:33;total:87771 | iSNV |
| F35 | F35-16 | 4355 | NS2B | 0.0311 | A:97247;G:3126;C:3;T:3;total:100379 | iSNV |
| F35 | F35-16 | 4885 | NS3 | 0.0927 | A:106622;G:17;C:10897;T:3;total:117539 | iSNV |
| F35 | F35-16 | 5311 | NS3 | 0.8621 | A:2;G:288;C:74236;T:11930;total:86456 | iSNV |
| F35 | F35-16 | 5312 | NS3 | 0.8552 | A:5;G:3;C:73636;T:12478;total:86122 | iSNV |
| F35 | F35-16 | 5875 | NS3 | 0.2629 | A:6;G:0;C:28132;T:78843;total:106981 | iSNV |
| F35 | F35-16 | 7381 | NS4B | 0.0245 | A:1;G:0;C:83032;T:2089;total:85122 | iSNV |
| F35 | F35-16 | 7546 | NS4B | 0.3197 | A:59923;G:28897;C:1538;T:5;total:90363 | iSNV |
| F35 | F35-16 | 7657 | NS4B | 0.9485 | A:5064;G:93242;C:3;T:4;total:98313 | iSNV |
| F35 | F35-16 | 7751 | NS5 | 0.2792 | A:5;G:0;C:22473;T:57997;total:80475 | iSNV |
| F35 | F35-16 | 8438 | NS5 | 0.0487 | A:2;G:3;C:5802;T:113235;total:119042 | iSNV |
| F35 | F35-16 | 9688 | NS5 | 0.0334 | A:4;G:1;C:97119;T:3357;total:100481 | iSNV |
| F35 | F35-16 | 10069 | NS5 | 0.1066 | A:0;G:3;C:70715;T:8446;total:79164 | iSNV |
| F35 | F35-16 | 10343 | NS5 | 0.0787 | A:6;G:0;C:95695;T:8183;total:103884 | iSNV |
| F35 | F35-16 | 10808 | 3'-UTR | 0.0738 | A:5363;G:67274;C:8;T:18;total:72663 | iSNV |
| F35 | F35-17 | 287 | C | 0.0427 | A:3751;G:83881;C:14;T:7;total:87653 | iSNV |
| F35 | F35-17 | 1082 | E | 0.0259 | A:63338;G:11;C:0;T:1688;total:65037 | iSNV |
| F35 | F35-17 | 1298 | E | 0.5747 | A:43773;G:90;C:13;T:32475;total:76351 | iSNV |
| F35 | F35-17 | 1758 | E | 0.0301 | A:2441;G:78555;C:6;T:4;total:81006 | iSNV |
| F35 | F35-17 | 1911 | E | 0.8623 | A:9138;G:57051;C:129;T:2;total:66320 | iSNV |
| F35 | F35-17 | 1963 | E | 0.0823 | A:1;G:2;C:64871;T:5818;total:70692 | iSNV |
| F35 | F35-17 | 3161 | NS1 | 0.0321 | A:88157;G:2931;C:0;T:7;total:91095 | iSNV |
| F35 | F35-17 | 3368 | NS1 | 0.0329 | A:189;G:65684;C:6;T:2248;total:68127 | iSNV |
| F35 | F35-17 | 3675 | NS1 | 0.0666 | A:4458;G:62391;C:4;T:12;total:66865 | iSNV |
| F35 | F35-17 | 3973 | NS2A | 0.0648 | A:4;G:3;C:81997;T:5684;total:87688 | iSNV |
| F35 | F35-17 | 4896 | NS3 | 0.0506 | A:5053;G:94604;C:8;T:5;total:99670 | iSNV |
| F35 | F35-17 | 5216 | NS3 | 0.1614 | A:59149;G:11388;C:2;T:2;total:70541 | iSNV |
| F35 | F35-17 | 5311 | NS3 | 0.3874 | A:3;G:410;C:27981;T:43825;total:72219 | iSNV |

| | | | | | | |
|---|---|---|---|---|---|---|
| F35 | F35-17 | 5343 | NS3 | 0.1318 | A:5;G:2;C:9850;T:64859;total:74716 | iSNV |
| F35 | F35-17 | 5747 | NS3 | 0.0454 | A:4452;G:93565;C:1;T:3;total:98021 | iSNV |
| F35 | F35-17 | 6449 | NS3 | 0.0785 | A:1;G:3;C:6085;T:71413;total:77502 | iSNV |
| F35 | F35-17 | 6978 | NS4A | 0.0839 | A:6583;G:71808;C:1;T:5;total:78397 | iSNV |
| F35 | F35-17 | 7488 | NS4B | 0.1575 | A:48671;G:57;C:1;T:9114;total:57843 | iSNV |
| F35 | F35-17 | 7495 | NS4B | 0.0202 | A:1;G:3;C:1219;T:58967;total:60190 | iSNV |
| F35 | F35-17 | 7656 | NS4B | 0.4534 | A:36120;G:43498;C:26;T:4;total:79648 | iSNV |
| F35 | F35-17 | 7657 | NS4B | 0.309 | A:54975;G:24591;C:5;T:1;total:79572 | iSNV |
| F35 | F35-17 | 8656 | NS5 | 0.573 | A:3;G:1;C:44169;T:32924;total:77097 | iSNV |
| F35 | F35-17 | 8659 | NS5 | 0.5776 | A:32214;G:44032;C:6;T:4;total:76256 | iSNV |
| F35 | F35-17 | 9203 | NS5 | 0.1863 | A:18498;G:80757;C:3;T:9;total:99267 | iSNV |
| F35 | F35-17 | 9584 | NS5 | 0.0616 | A:2;G:2;C:81226;T:5337;total:86567 | iSNV |
| F35 | F35-17 | 9596 | NS5 | 0.0472 | A:76831;G:48;C:8;T:3809;total:80696 | iSNV |
| F35 | F35-17 | 9688 | NS5 | 0.0276 | A:1;G:4;C:77701;T:2207;total:79913 | iSNV |
| F35 | F35-17 | 10069 | NS5 | 0.3648 | A:0;G:0;C:40100;T:23036;total:63136 | iSNV |
| F35 | F35-17 | 10092 | NS5 | 0.0255 | A:1617;G:64410;C:1732;T:4;total:67763 | iSNV |
| F35 | F35-17 | 10811 | 3'-UTR | 0.207 | A:12517;G:47919;C:10;T:11;total:60457 | iSNV |
| F35 | F35-19 | 1296 | E | 0.9232 | A:0;G:2;C:55962;T:4659;total:60623 | iSNV |
| F35 | F35-19 | 1638 | E | 0.0396 | A:2599;G:0;C:62987;T:5;total:65591 | iSNV |
| F35 | F35-19 | 1911 | E | 0.9217 | A:4122;G:48473;C:25;T:1;total:52621 | iSNV |
| F35 | F35-19 | 4173 | NS2A | 0.1915 | A:27733;G:6572;C:3;T:1;total:34309 | iSNV |
| F35 | F35-19 | 4943 | NS3 | 0.9249 | A:5214;G:64183;C:11;T:1;total:69409 | iSNV |
| F35 | F35-19 | 5311 | NS3 | 0.2933 | A:2;G:6927;C:16368;T:32505;total:55802 | iSNV |
| F35 | F35-19 | 6268 | NS3 | 0.0232 | A:66108;G:9;C:1574;T:3;total:67694 | iSNV |
| F35 | F35-19 | 6933 | NS4A | 0.0205 | A:59779;G:3;C:1252;T:0;total:61034 | iSNV |
| F35 | F35-19 | 6943 | NS4A | 0.3778 | A:23795;G:0;C:39164;T:11;total:62970 | iSNV |
| F35 | F35-19 | 7546 | NS4B | 0.0441 | A:55356;G:2559;C:10;T:1;total:57926 | iSNV |
| F35 | F35-19 | 7657 | NS4B | 0.9715 | A:1815;G:61665;C:2;T:6;total:63488 | iSNV |
| F35 | F35-19 | 9246 | NS5 | 0.1401 | A:70995;G:11570;C:3;T:3;total:82571 | iSNV |
| F35 | F35-19 | 9360 | NS5 | 0.9204 | A:72502;G:6277;C:4;T:3;total:78786 | iSNV |
| F35 | F35-19 | 9688 | NS5 | 0.9798 | A:2;G:1;C:1307;T:63137;total:64447 | iSNV |
| F35 | F35-19 | 10013 | NS5 | 0.0263 | A:2;G:0;C:53325;T:1446;total:54773 | iSNV |
| F35 | F35-19 | 10415 | 3'-UTR | 0.9148 | A:1;G:0;C:49606;T:4623;total:54230 | iSNV |
| F35 | F35-2 | 505 | M | 0.0302 | A:2;G:25;C:2094;T:67104;total:69225 | iSNV |
| F35 | F35-2 | 1127 | E | 0.025 | A:3;G:2;C:1483;T:57665;total:59153 | iSNV |
| F35 | F35-2 | 1296 | E | 0.4269 | A:1;G:2;C:22764;T:30550;total:53317 | iSNV |
| F35 | F35-2 | 1384 | E | 0.1035 | A:54295;G:10;C:4;T:6273;total:60582 | iSNV |
| F35 | F35-2 | 1390 | E | 0.1099 | A:52423;G:6478;C:9;T:0;total:58910 | iSNV |
| F35 | F35-2 | 1417 | E | 0.4423 | A:9;G:4;C:35166;T:27904;total:63083 | iSNV |
| F35 | F35-2 | 1491 | E | 0.093 | A:55795;G:5728;C:5;T:2;total:61530 | iSNV |
| F35 | F35-2 | 1800 | E | 0.0958 | A:6909;G:2;C:26;T:65162;total:72099 | iSNV |
| F35 | F35-2 | 1911 | E | 0.3357 | A:37969;G:19223;C:54;T:1;total:57247 | iSNV |
| F35 | F35-2 | 2291 | E | 0.0258 | A:47015;G:1246;C:1;T:3;total:48265 | iSNV |
| F35 | F35-2 | 2531 | NS1 | 0.0804 | A:9;G:0;C:48444;T:4240;total:52693 | iSNV |
| F35 | F35-2 | 2624 | NS1 | 0.0253 | A:1569;G:60200;C:5;T:14;total:61788 | iSNV |
| F35 | F35-2 | 3139 | NS1 | 0.0331 | A:57663;G:1976;C:5;T:2;total:59646 | iSNV |
| F35 | F35-2 | 3428 | NS1 | 0.0268 | A:0;G:1;C:53748;T:1485;total:55234 | iSNV |
| F35 | F35-2 | 3464 | NS1 | 0.0217 | A:0;G:1;C:47936;T:1066;total:49003 | iSNV |
| F35 | F35-2 | 3511 | NS1 | 0.0686 | A:11;G:4;C:3009;T:40784;total:43808 | iSNV |
| F35 | F35-2 | 4021 | NS2A | 0.0954 | A:5119;G:48514;C:3;T:7;total:53643 | iSNV |
| F35 | F35-2 | 4314 | NS2B | 0.0249 | A:54610;G:1397;C:0;T:2;total:56009 | iSNV |
| F35 | F35-2 | 4988 | NS3 | 0.0282 | A:2;G:0;C:1835;T:63037;total:64874 | iSNV |
| F35 | F35-2 | 5311 | NS3 | 0.5237 | A:2;G:286;C:26689;T:24543;total:51520 | iSNV |
| F35 | F35-2 | 6533 | NS4A | 0.1613 | A:1;G:2;C:7983;T:41475;total:49461 | iSNV |
| F35 | F35-2 | 6738 | NS4A | 0.0229 | A:9;G:0;C:1115;T:47481;total:48605 | iSNV |
| F35 | F35-2 | 7381 | NS4B | 0.0206 | A:2;G:5;C:48073;T:1013;total:49093 | iSNV |
| F35 | F35-2 | 7448 | NS4B | 0.0589 | A:40700;G:2550;C:1;T:9;total:43260 | iSNV |
| F35 | F35-2 | 7495 | NS4B | 0.0492 | A:3;G:2;C:2127;T:41043;total:43175 | iSNV |
| F35 | F35-2 | 7546 | NS4B | 0.096 | A:45968;G:4886;C:19;T:2;total:50875 | iSNV |
| F35 | F35-2 | 7575 | NS4B | 0.0246 | A:50737;G:1281;C:6;T:6;total:52030 | iSNV |
| F35 | F35-2 | 7656 | NS4B | 0.4893 | A:25700;G:26199;C:623;T:1;total:52523 | iSNV |
| F35 | F35-2 | 7657 | NS4B | 0.3145 | A:35975;G:16509;C:4;T:2;total:52490 | iSNV |
| F35 | F35-2 | 8140 | NS5 | 0.0288 | A:68251;G:2027;C:84;T:2;total:70364 | iSNV |
| F35 | F35-2 | 9225 | NS5 | 0.1014 | A:7099;G:1;C:62861;T:25;total:69986 | iSNV |
| F35 | F35-2 | 9370 | NS5 | 0.0296 | A:2129;G:51;C:17;T:69490;total:71687 | iSNV |
| F35 | F35-2 | 9688 | NS5 | 0.6758 | A:3;G:2;C:21238;T:44247;total:65490 | iSNV |
| F35 | F35-2 | 9926 | NS5 | 0.3848 | A:34671;G:21695;C:4;T:6;total:56376 | iSNV |
| F35 | F35-2 | 10069 | NS5 | 0.1984 | A:0;G:2;C:39564;T:9795;total:49361 | iSNV |
| F35 | F35-2 | 10632 | 3'-UTR | 0.0542 | A:22;G:17;C:2879;T:50104;total:53022 | iSNV |
| F35 | F35-20 | 503 | M | 0.2501 | A:13885;G:41617;C:5;T:2;total:55509 | iSNV |
| F35 | F35-20 | 929 | M | 0.2452 | A:29142;G:9472;C:1;T:0;total:38615 | iSNV |
| F35 | F35-20 | 1296 | E | 0.5662 | A:3;G:1;C:24677;T:18912;total:43593 | iSNV |
| F35 | F35-20 | 1347 | E | 0.035 | A:45772;G:1664;C:2;T:1;total:47439 | iSNV |
| F35 | F35-20 | 1373 | E | 0.0224 | A:45604;G:1048;C:0;T:3;total:46655 | iSNV |
| F35 | F35-20 | 1384 | E | 0.2365 | A:36582;G:9;C:10;T:11342;total:47943 | iSNV |
| F35 | F35-20 | 1390 | E | 0.2477 | A:35282;G:11645;C:71;T:1;total:46999 | iSNV |
| F35 | F35-20 | 1417 | E | 0.154 | A:3;G:2;C:41731;T:7603;total:49339 | iSNV |
| F35 | F35-20 | 1432 | E | 0.0217 | A:47200;G:55;C:1051;T:2;total:48308 | iSNV |
| F35 | F35-20 | 1504 | E | 0.2404 | A:5;G:3;C:10881;T:34362;total:45251 | iSNV |
| F35 | F35-20 | 1803 | E | 0.0667 | A:56502;G:4043;C:1;T:1;total:60547 | iSNV |
| F35 | F35-20 | 1911 | E | 0.3696 | A:26383;G:15492;C:33;T:0;total:41908 | iSNV |
| F35 | F35-20 | 2232 | E | 0.0405 | A:16;G:40235;C:0;T:1700;total:41951 | iSNV |

| | | | | | | |
|---|---|---|---|---|---|---|
| F35 | F35-20 | 2466 | E | 0.0439 | A:33711;G:8;C:1550;T:1;total:35270 | iSNV |
| F35 | F35-20 | 3025 | NS1 | 0.2435 | A:6;G:2;C:9271;T:28781;total:38060 | iSNV |
| F35 | F35-20 | 3661 | NS1 | 0.0368 | A:2;G:1;C:1409;T:36853;total:38265 | iSNV |
| F35 | F35-20 | 3797 | NS2A | 0.0248 | A:40330;G:5;C:2;T:1029;total:41366 | iSNV |
| F35 | F35-20 | 4566 | NS2B | 0.5935 | A:9;G:23277;C:13;T:33956;total:57255 | iSNV |
| F35 | F35-20 | 5210 | NS3 | 0.0301 | A:3;G:0;C:37405;T:1161;total:38569 | iSNV |
| F35 | F35-20 | 5311 | NS3 | 0.0609 | A:0;G:473;C:2570;T:39105;total:42148 | iSNV |
| F35 | F35-20 | 5358 | NS3 | 0.0719 | A:1;G:41671;C:3232;T:2;total:44906 | iSNV |
| F35 | F35-20 | 5602 | NS3 | 0.0431 | A:3;G:0;C:46488;T:2099;total:48590 | iSNV |
| F35 | F35-20 | 5835 | NS3 | 0.0273 | A:1486;G:52828;C:5;T:0;total:54319 | iSNV |
| F35 | F35-20 | 6568 | NS4A | 0.039 | A:37025;G:1504;C:0;T:1;total:38530 | iSNV |
| F35 | F35-20 | 6637 | NS4A | 0.0291 | A:2;G:2;C:1048;T:34853;total:35905 | iSNV |
| F35 | F35-20 | 6944 | NS4A | 0.2416 | A:2;G:34401;C:10965;T:0;total:45368 | iSNV |
| F35 | F35-20 | 7373 | NS4B | 0.0413 | A:1485;G:34409;C:0;T:1;total:35895 | iSNV |
| F35 | F35-20 | 7495 | NS4B | 0.2753 | A:5;G:4;C:8829;T:23231;total:32069 | iSNV |
| F35 | F35-20 | 7656 | NS4B | 0.6647 | A:29273;G:14781;C:17;T:0;total:44071 | iSNV |
| F35 | F35-20 | 7657 | NS4B | 0.0276 | A:42875;G:1220;C:2;T:1;total:44098 | iSNV |
| F35 | F35-20 | 9025 | NS5 | 0.0253 | A:45046;G:1171;C:0;T:1;total:46218 | iSNV |
| F35 | F35-20 | 9146 | NS5 | 0.0297 | A:1;G:26;C:1547;T:50365;total:51939 | iSNV |
| F35 | F35-20 | 9225 | NS5 | 0.2481 | A:14819;G:0;C:44879;T:16;total:59714 | iSNV |
| F35 | F35-20 | 9585 | NS5 | 0.2344 | A:5;G:21;C:11471;T:37438;total:48935 | iSNV |
| F35 | F35-20 | 9688 | NS5 | 0.0582 | A:5;G:3;C:42618;T:2637;total:45263 | iSNV |
| F35 | F35-20 | 10069 | NS5 | 0.5996 | A:0;G:1;C:14198;T:21253;total:35452 | iSNV |
| F35 | F35-20 | 10071 | NS5 | 0.0711 | A:2554;G:2;C:2;T:33337;total:35895 | iSNV |
| F35 | F35-22 | 306 | C | 0.0531 | A:1648;G:29339;C:0;T:4;total:30991 | iSNV |
| F35 | F35-22 | 503 | M | 0.4062 | A:11260;G:16458;C:2;T:0;total:27720 | iSNV |
| F35 | F35-22 | 584 | M | 0.1364 | A:13;G:0;C:15381;T:2433;total:17827 | iSNV |
| F35 | F35-22 | 697 | M | 0.0217 | A:3;G:0;C:377;T:16914;total:17294 | iSNV |
| F35 | F35-22 | 1298 | E | 0.0608 | A:933;G:10;C:1;T:14383;total:15327 | iSNV |
| F35 | F35-22 | 1384 | E | 0.8137 | A:2901;G:2;C:10;T:12653;total:15566 | iSNV |
| F35 | F35-22 | 1390 | E | 0.8022 | A:3119;G:12642;C:3;T:0;total:15764 | iSNV |
| F35 | F35-22 | 1491 | E | 0.1168 | A:14061;G:1860;C:0;T:0;total:15921 | iSNV |
| F35 | F35-22 | 1504 | E | 0.6234 | A:4;G:0;C:9801;T:5925;total:15730 | iSNV |
| F35 | F35-22 | 1786 | E | 0.0879 | A:0;G:0;C:1727;T:17908;total:19635 | iSNV |
| F35 | F35-22 | 1800 | E | 0.1059 | A:2141;G:0;C:6;T:18064;total:20211 | iSNV |
| F35 | F35-22 | 1911 | E | 0.0618 | A:14013;G:924;C:0;T:1;total:14938 | iSNV |
| F35 | F35-22 | 1912 | E | 0.0397 | A:13953;G:2;C:1;T:577;total:14533 | iSNV |
| F35 | F35-22 | 2843 | NS1 | 0.1001 | A:0;G:0;C:19969;T:2223;total:22192 | iSNV |
| F35 | F35-22 | 3025 | NS1 | 0.4736 | A:5;G:1;C:7834;T:8699;total:16539 | iSNV |
| F35 | F35-22 | 3368 | NS1 | 0.2537 | A:8493;G:24974;C:2;T:2;total:33471 | iSNV |
| F35 | F35-22 | 3800 | NS2A | 0.0305 | A:1;G:5;C:1100;T:34934;total:36040 | iSNV |
| F35 | F35-22 | 4640 | NS3 | 0.0874 | A:46092;G:4418;C:1;T:2;total:50513 | iSNV |
| F35 | F35-22 | 5210 | NS3 | 0.0273 | A:0;G:0;C:33417;T:938;total:34355 | iSNV |
| F35 | F35-22 | 6533 | NS4A | 0.1139 | A:1;G:11;C:4062;T:31561;total:35635 | iSNV |
| F35 | F35-22 | 6620 | NS4A | 0.0228 | A:34420;G:806;C:1;T:1;total:35228 | iSNV |
| F35 | F35-22 | 6944 | NS4A | 0.2423 | A:5;G:31000;C:9921;T:8;total:40934 | iSNV |
| F35 | F35-22 | 7264 | NS4A | 0.026 | A:0;G:0;C:30857;T:824;total:31681 | iSNV |
| F35 | F35-22 | 7373 | NS4B | 0.27 | A:8609;G:23225;C:32;T:16;total:31882 | iSNV |
| F35 | F35-22 | 7495 | NS4B | 0.3876 | A:0;G:2;C:10721;T:16933;total:27656 | iSNV |
| F35 | F35-22 | 7656 | NS4B | 0.3557 | A:13226;G:23922;C:34;T:0;total:37182 | iSNV |
| F35 | F35-22 | 7657 | NS4B | 0.1633 | A:31144;G:6082;C:0;T:0;total:37226 | iSNV |
| F35 | F35-22 | 8190 | NS5 | 0.0376 | A:5;G:1;C:1584;T:40536;total:42126 | iSNV |
| F35 | F35-22 | 8194 | NS5 | 0.0511 | A:38316;G:2065;C:4;T:1;total:40386 | iSNV |
| F35 | F35-22 | 8894 | NS5 | 0.0629 | A:2067;G:19;C:30675;T:57;total:32818 | iSNV |
| F35 | F35-22 | 9225 | NS5 | 0.3282 | A:15454;G:2;C:31612;T:12;total:47080 | iSNV |
| F35 | F35-22 | 9657 | NS5 | 0.0334 | A:36742;G:1271;C:0;T:0;total:38013 | iSNV |
| F35 | F35-22 | 9688 | NS5 | 0.1254 | A:0;G:2;C:35350;T:5073;total:40425 | iSNV |
| F35 | F35-22 | 9783 | NS5 | 0.0926 | A:2;G:0;C:36488;T:3724;total:40214 | iSNV |
| F35 | F35-22 | 9932 | NS5 | 0.1414 | A:5208;G:2;C:1;T:31610;total:36821 | iSNV |
| F35 | F35-23 | 306 | C | 0.0584 | A:2056;G:33143;C:2;T:1;total:35202 | iSNV |
| F35 | F35-23 | 430 | C | 0.0217 | A:4;G:2;C:703;T:31553;total:32262 | iSNV |
| F35 | F35-23 | 503 | M | 0.4604 | A:15854;G:18575;C:3;T:0;total:34432 | iSNV |
| F35 | F35-23 | 584 | M | 0.0903 | A:14;G:0;C:22456;T:2231;total:24701 | iSNV |
| F35 | F35-23 | 697 | M | 0.035 | A:4;G:0;C:900;T:24741;total:25645 | iSNV |
| F35 | F35-23 | 1298 | E | 0.064 | A:1540;G:14;C:6;T:22480;total:24040 | iSNV |
| F35 | F35-23 | 1384 | E | 0.8094 | A:4558;G:1;C:10;T:19344;total:23913 | iSNV |
| F35 | F35-23 | 1390 | E | 0.8029 | A:4783;G:19474;C:4;T:2;total:24263 | iSNV |
| F35 | F35-23 | 1491 | E | 0.1476 | A:20184;G:3498;C:0;T:5;total:23687 | iSNV |
| F35 | F35-23 | 1504 | E | 0.5793 | A:1;G:0;C:13630;T:9903;total:23534 | iSNV |
| F35 | F35-23 | 1786 | E | 0.0649 | A:6;G:3;C:1892;T:27247;total:29148 | iSNV |
| F35 | F35-23 | 1800 | E | 0.1414 | A:4232;G:0;C:10;T:25674;total:29916 | iSNV |
| F35 | F35-23 | 1911 | E | 0.0738 | A:20754;G:1654;C:0;T:0;total:22408 | iSNV |
| F35 | F35-23 | 1912 | E | 0.0356 | A:21050;G:8;C:3;T:779;total:21840 | iSNV |
| F35 | F35-23 | 2843 | NS1 | 0.0753 | A:1;G:0;C:30965;T:2522;total:33488 | iSNV |
| F35 | F35-23 | 3025 | NS1 | 0.5014 | A:0;G:0;C:11237;T:11177;total:22414 | iSNV |
| F35 | F35-23 | 3368 | NS1 | 0.1728 | A:5978;G:28596;C:0;T:1;total:34575 | iSNV |
| F35 | F35-23 | 3800 | NS2A | 0.0409 | A:3;G:2;C:1463;T:34288;total:35756 | iSNV |
| F35 | F35-23 | 4640 | NS3 | 0.083 | A:48400;G:4387;C:6;T:0;total:52793 | iSNV |
| F35 | F35-23 | 5210 | NS3 | 0.0237 | A:0;G:1;C:33865;T:823;total:34689 | iSNV |
| F35 | F35-23 | 6533 | NS4A | 0.1012 | A:4;G:8;C:3578;T:31762;total:35352 | iSNV |
| F35 | F35-23 | 6944 | NS4A | 0.333 | A:3;G:26887;C:13431;T:3;total:40324 | iSNV |
| F35 | F35-23 | 7264 | NS4A | 0.0238 | A:0;G:0;C:30577;T:746;total:31323 | iSNV |

| | | | | | | |
|---|---|---|---|---|---|---|
| F35 | F35-23 | 7373 | NS4B | 0.1913 | A:6131;G:25835;C:25;T:42;total:32033 | iSNV |
| F35 | F35-23 | 7495 | NS4B | 0.4199 | A:1;G:2;C:11784;T:16275;total:28062 | iSNV |
| F35 | F35-23 | 7656 | NS4B | 0.2631 | A:10312;G:28839;C:36;T:1;total:39188 | iSNV |
| F35 | F35-23 | 7657 | NS4B | 0.1694 | A:32536;G:6641;C:1;T:2;total:39180 | iSNV |
| F35 | F35-23 | 8190 | NS5 | 0.0591 | A:5;G:3;C:2487;T:39540;total:42035 | iSNV |
| F35 | F35-23 | 8194 | NS5 | 0.0856 | A:37127;G:3479;C:5;T:4;total:40615 | iSNV |
| F35 | F35-23 | 8894 | NS5 | 0.0611 | A:2081;G:36;C:31862;T:76;total:34055 | iSNV |
| F35 | F35-23 | 9225 | NS5 | 0.4927 | A:24238;G:2;C:24932;T:17;total:49189 | iSNV |
| F35 | F35-23 | 9657 | NS5 | 0.0415 | A:37236;G:1615;C:5;T:0;total:38856 | iSNV |
| F35 | F35-23 | 9688 | NS5 | 0.1135 | A:0;G:0;C:36556;T:4681;total:41237 | iSNV |
| F35 | F35-23 | 9783 | NS5 | 0.0552 | A:1;G:0;C:39004;T:2282;total:41287 | iSNV |
| F35 | F35-23 | 9932 | NS5 | 0.0854 | A:3161;G:1;C:6;T:33835;total:37003 | iSNV |
| F35 | F35-25 | 290 | C | 0.0296 | A:4;G:4;C:2318;T:75814;total:78140 | iSNV |
| F35 | F35-25 | 340 | C | 0.2015 | A:13;G:0;C:63131;T:15935;total:79079 | iSNV |
| F35 | F35-25 | 399 | C | 0.2147 | A:17493;G:7;C:63931;T:18;total:81449 | iSNV |
| F35 | F35-25 | 656 | M | 0.0859 | A:13;G:0;C:5643;T:60002;total:65658 | iSNV |
| F35 | F35-25 | 1296 | E | 0.2192 | A:5;G:4;C:15892;T:56587;total:72488 | iSNV |
| F35 | F35-25 | 1465 | E | 0.2687 | A:53036;G:8;C:19498;T:7;total:72549 | iSNV |
| F35 | F35-25 | 1911 | E | 0.5905 | A:26596;G:38337;C:1;T:0;total:64934 | iSNV |
| F35 | F35-25 | 2083 | E | 0.0553 | A:57251;G:13;C:3356;T:19;total:60639 | iSNV |
| F35 | F35-25 | 2141 | E | 0.0887 | A:61298;G:5969;C:1;T:4;total:67272 | iSNV |
| F35 | F35-25 | 2558 | NS1 | 0.0301 | A:9;G:0;C:51541;T:1605;total:53155 | iSNV |
| F35 | F35-25 | 2873 | NS1 | 0.1744 | A:82050;G:44;C:21;T:17349;total:99464 | iSNV |
| F35 | F35-25 | 3773 | NS2A | 0.027 | A:13;G:3;C:1788;T:64256;total:66060 | iSNV |
| F35 | F35-25 | 4233 | NS2B | 0.0224 | A:7;G:1133;C:6;T:49256;total:50402 | iSNV |
| F35 | F35-25 | 4493 | NS2B | 0.0278 | A:20;G:0;C:2010;T:70163;total:72193 | iSNV |
| F35 | F35-25 | 5025 | NS3 | 0.0269 | A:2;G:2;C:89311;T:2470;total:91785 | iSNV |
| F35 | F35-25 | 5311 | NS3 | 0.2682 | A:2;G:1558;C:17806;T:47024;total:66390 | iSNV |
| F35 | F35-25 | 5835 | NS3 | 0.0714 | A:6116;G:79504;C:4;T:2;total:85626 | iSNV |
| F35 | F35-25 | 6324 | NS3 | 0.0261 | A:70020;G:10;C:42;T:1880;total:71952 | iSNV |
| F35 | F35-25 | 6861 | NS4A | 0.0283 | A:2;G:0;C:62654;T:1830;total:64486 | iSNV |
| F35 | F35-25 | 7373 | NS4B | 0.0277 | A:1605;G:55828;C:25;T:303;total:57761 | iSNV |
| F35 | F35-25 | 7495 | NS4B | 0.1862 | A:3;G:4;C:9657;T:42172;total:51836 | iSNV |
| F35 | F35-25 | 7543 | NS4B | 0.0576 | A:60498;G:3700;C:0;T:0;total:64198 | iSNV |
| F35 | F35-25 | 7575 | NS4B | 0.0569 | A:62617;G:3782;C:4;T:0;total:66403 | iSNV |
| F35 | F35-25 | 7656 | NS4B | 0.1532 | A:10838;G:58956;C:937;T:3;total:70734 | iSNV |
| F35 | F35-25 | 7657 | NS4B | 0.5226 | A:33682;G:36856;C:2;T:2;total:70542 | iSNV |
| F35 | F35-25 | 8045 | NS5 | 0.0332 | A:2768;G:80581;C:2;T:9;total:83360 | iSNV |
| F35 | F35-25 | 9221 | NS5 | 0.0924 | A:83348;G:30;C:26;T:8495;total:91899 | iSNV |
| F35 | F35-25 | 9646 | NS5 | 0.0234 | A:67393;G:1619;C:4;T:9;total:69025 | iSNV |
| F35 | F35-25 | 10069 | NS5 | 0.7838 | A:2;G:1;C:12166;T:44078;total:56247 | iSNV |
| F35 | F35-25 | 10080 | NS5 | 0.0226 | A:57331;G:3;C:1331;T:2;total:58667 | iSNV |
| F35 | F35-25 | 10092 | NS5 | 0.0296 | A:1825;G:59804;C:23;T:3;total:61655 | iSNV |
| F35 | F35-26 | 312 | C | 0.0548 | A:3267;G:56287;C:3;T:3;total:59560 | iSNV |
| F35 | F35-26 | 323 | C | 0.0251 | A:1449;G:56065;C:2;T:7;total:57523 | iSNV |
| F35 | F35-26 | 338 | C | 0.0215 | A:7;G:4;C:1300;T:59131;total:60442 | iSNV |
| F35 | F35-26 | 398 | C | 0.0271 | A:1810;G:64918;C:3;T:7;total:66738 | iSNV |
| F35 | F35-26 | 399 | C | 0.0336 | A:2234;G:2;C:64179;T:10;total:66425 | iSNV |
| F35 | F35-26 | 1058 | E | 0.0499 | A:40863;G:2147;C:0;T:3;total:43013 | iSNV |
| F35 | F35-26 | 1296 | E | 0.1753 | A:1;G:6;C:9479;T:44583;total:54069 | iSNV |
| F35 | F35-26 | 1298 | E | 0.2244 | A:99;G:11928;C:34;T:41077;total:53138 | iSNV |
| F35 | F35-26 | 1417 | E | 0.0614 | A:2;G:1;C:54845;T:3593;total:58441 | iSNV |
| F35 | F35-26 | 1450 | E | 0.0335 | A:56543;G:1965;C:9;T:3;total:58520 | iSNV |
| F35 | F35-26 | 1489 | E | 0.0474 | A:3;G:3;C:49354;T:2458;total:51818 | iSNV |
| F35 | F35-26 | 1911 | E | 0.7399 | A:11279;G:32057;C:19;T:1;total:43356 | iSNV |
| F35 | F35-26 | 4070 | NS2A | 0.2494 | A:2;G:10953;C:403;T:32548;total:43906 | iSNV |
| F35 | F35-26 | 4380 | NS2B | 0.2357 | A:12617;G:4;C:40866;T:23;total:53510 | iSNV |
| F35 | F35-26 | 4712 | NS3 | 0.0301 | A:68747;G:2139;C:0;T:3;total:70889 | iSNV |
| F35 | F35-26 | 5311 | NS3 | 0.1182 | A:2;G:5673;C:2303;T:40007;total:47985 | iSNV |
| F35 | F35-26 | 5871 | NS3 | 0.2447 | A:49052;G:4;C:15903;T:9;total:64968 | iSNV |
| F35 | F35-26 | 6329 | NS3 | 0.2298 | A:11932;G:39980;C:2;T:3;total:51917 | iSNV |
| F35 | F35-26 | 6628 | NS4A | 0.0338 | A:1;G:1;C:45767;T:1604;total:47373 | iSNV |
| F35 | F35-26 | 7104 | NS4A | 0.2472 | A:11841;G:36050;C:5;T:0;total:47896 | iSNV |
| F35 | F35-26 | 7179 | NS4A | 0.0784 | A:3457;G:40582;C:1;T:3;total:44043 | iSNV |
| F35 | F35-26 | 7199 | NS4A | 0.0592 | A:0;G:0;C:40310;T:2540;total:42850 | iSNV |
| F35 | F35-26 | 7373 | NS4B | 0.0338 | A:1501;G:42784;C:0;T:10;total:44295 | iSNV |
| F35 | F35-26 | 7494 | NS4B | 0.2733 | A:21;G:28143;C:2;T:10597;total:38763 | iSNV |
| F35 | F35-26 | 7495 | NS4B | 0.0621 | A:2;G:1;C:2501;T:37749;total:40253 | iSNV |
| F35 | F35-26 | 7657 | NS4B | 0.8818 | A:6584;G:49092;C:6;T:0;total:55682 | iSNV |
| F35 | F35-26 | 7718 | NS5 | 0.0256 | A:50002;G:1315;C:1;T:3;total:51321 | iSNV |
| F35 | F35-26 | 8012 | NS5 | 0.0283 | A:59060;G:1722;C:5;T:0;total:60787 | iSNV |
| F35 | F35-26 | 8987 | NS5 | 0.2325 | A:13925;G:10;C:20;T:45927;total:59882 | iSNV |
| F35 | F35-26 | 9358 | NS5 | 0.5152 | A:35706;G:33616;C:4;T:4;total:69330 | iSNV |
| F35 | F35-26 | 9722 | NS5 | 0.0407 | A:2;G:0;C:49688;T:2110;total:51800 | iSNV |
| F35 | F35-26 | 10069 | NS5 | 0.9573 | A:0;G:4;C:1809;T:40512;total:42325 | iSNV |
| F35 | F35-26 | 10416 | 3'-UTR | 0.0724 | A:45326;G:3541;C:0;T:5;total:48872 | iSNV |
| F35 | F35-26 | 10470 | 3'-UTR | 0.0204 | A:38377;G:802;C:1;T:1;total:39181 | iSNV |
| F35 | F35-26 | 10812 | 3'-UTR | 0.0205 | A:837;G:7;C:64;T:39752;total:40660 | iSNV |
| F35 | F35-28 | 235 | C | 0.5995 | A:1;G:0;C:39862;T:26636;total:66499 | iSNV |
| F35 | F35-28 | 307 | C | 0.1164 | A:4;G:0;C:57125;T:7533;total:64662 | iSNV |
| F35 | F35-28 | 323 | C | 0.0206 | A:1267;G:60217;C:1;T:2;total:61487 | iSNV |
| F35 | F35-28 | 483 | M | 0.0857 | A:18;G:5705;C:27;T:60771;total:66521 | iSNV |

| | | | | | | |
|---|---|---|---|---|---|---|
| F35 | F35-28 | 951 | M | 0.0388 | A:0;G:1;C:44452;T:1796;total:46249 | iSNV |
| F35 | F35-28 | 982 | E | 0.7088 | A:13057;G:0;C:31768;T:11;total:44836 | iSNV |
| F35 | F35-28 | 1296 | E | 0.4178 | A:1;G:0;C:21525;T:29993;total:51519 | iSNV |
| F35 | F35-28 | 1468 | E | 0.7308 | A:39732;G:4;C:14641;T:5;total:54382 | iSNV |
| F35 | F35-28 | 1786 | E | 0.0875 | A:3;G:0;C:5638;T:58740;total:64381 | iSNV |
| F35 | F35-28 | 1911 | E | 0.0816 | A:43304;G:3851;C:2;T:0;total:47157 | iSNV |
| F35 | F35-28 | 2162 | E | 0.0894 | A:9;G:3;C:4632;T:47154;total:51798 | iSNV |
| F35 | F35-28 | 2276 | E | 0.3987 | A:1;G:0;C:25595;T:16974;total:42570 | iSNV |
| F35 | F35-28 | 2437 | E | 0.0843 | A:1;G:1;C:35697;T:3290;total:38989 | iSNV |
| F35 | F35-28 | 3511 | NS1 | 0.0414 | A:9;G:44;C:1772;T:40942;total:42767 | iSNV |
| F35 | F35-28 | 3768 | NS2A | 0.0399 | A:1;G:3;C:2018;T:48536;total:50558 | iSNV |
| F35 | F35-28 | 3776 | NS2A | 0.0398 | A:5;G:13;C:2047;T:49287;total:51352 | iSNV |
| F35 | F35-28 | 4177 | NS2A | 0.0493 | A:0;G:1;C:29010;T:1505;total:30516 | iSNV |
| F35 | F35-28 | 4394 | NS2B | 0.0307 | A:0;G:2;C:49979;T:1586;total:51567 | iSNV |
| F35 | F35-28 | 5311 | NS3 | 0.0985 | A:0;G:863;C:4791;T:42948;total:48602 | iSNV |
| F35 | F35-28 | 6854 | NS4A | 0.0224 | A:0;G:1;C:1218;T:53072;total:54291 | iSNV |
| F35 | F35-28 | 6884 | NS4A | 0.0207 | A:1;G:1;C:51354;T:1089;total:52445 | iSNV |
| F35 | F35-28 | 7265 | NS4A | 0.6867 | A:28041;G:12799;C:0;T:3;total:40843 | iSNV |
| F35 | F35-28 | 7495 | NS4B | 0.048 | A:3;G:0;C:2060;T:40784;total:42847 | iSNV |
| F35 | F35-28 | 7657 | NS4B | 0.9273 | A:4287;G:54608;C:4;T:0;total:58899 | iSNV |
| F35 | F35-28 | 7988 | NS5 | 0.0209 | A:10;G:68352;C:6;T:1460;total:69828 | iSNV |
| F35 | F35-28 | 8456 | NS5 | 0.6868 | A:23497;G:11;C:2;T:51493;total:75003 | iSNV |
| F35 | F35-28 | 9584 | NS5 | 0.0984 | A:0;G:1;C:56873;T:6212;total:63086 | iSNV |
| F35 | F35-28 | 9688 | NS5 | 0.1274 | A:3;G:0;C:49764;T:7267;total:57034 | iSNV |
| F35 | F35-28 | 9998 | NS5 | 0.0214 | A:0;G:0;C:46268;T:1016;total:47284 | iSNV |
| F35 | F35-28 | 10071 | NS5 | 0.0295 | A:1389;G:0;C:2;T:45634;total:47025 | iSNV |
| F35 | F35-28 | 10079 | NS5 | 0.0388 | A:44176;G:1835;C:2;T:1213;total:47226 | iSNV |
| F35 | F35-28 | 10092 | NS5 | 0.7141 | A:34759;G:13920;C:1;T:1;total:48681 | iSNV |
| F35 | F35-28 | 10397 | 3'-UTR | 0.0207 | A:1;G:5;C:1160;T:54708;total:55874 | iSNV |
| F35 | F35-30 | 785 | M | 0.0287 | A:55796;G:1649;C:1;T:4;total:57450 | iSNV |
| F35 | F35-30 | 1181 | E | 0.2542 | A:4;G:0;C:48502;T:16535;total:65041 | iSNV |
| F35 | F35-30 | 1296 | E | 0.054 | A:5;G:1;C:3368;T:58955;total:62329 | iSNV |
| F35 | F35-30 | 1298 | E | 0.0606 | A:19;G:3692;C:9;T:57136;total:60856 | iSNV |
| F35 | F35-30 | 1417 | E | 0.1348 | A:1;G:0;C:56948;T:8878;total:65827 | iSNV |
| F35 | F35-30 | 1461 | E | 0.0301 | A:3;G:0;C:1915;T:61507;total:63425 | iSNV |
| F35 | F35-30 | 1563 | E | 0.0477 | A:5;G:0;C:48813;T:2446;total:51264 | iSNV |
| F35 | F35-30 | 1911 | E | 0.7131 | A:13054;G:32437;C:4;T:2;total:45497 | iSNV |
| F35 | F35-30 | 2129 | E | 0.8557 | A:7475;G:44299;C:5;T:1;total:51780 | iSNV |
| F35 | F35-30 | 2291 | E | 0.0268 | A:49408;G:1363;C:0;T:4;total:50775 | iSNV |
| F35 | F35-30 | 2510 | NS1 | 0.0482 | A:41262;G:2091;C:0;T:5;total:43358 | iSNV |
| F35 | F35-30 | 3001 | NS1 | 0.0774 | A:4206;G:50057;C:8;T:2;total:54273 | iSNV |
| F35 | F35-30 | 3230 | NS1 | 0.8279 | A:13599;G:65410;C:3;T:0;total:79012 | iSNV |
| F35 | F35-30 | 3425 | NS1 | 0.0713 | A:1;G:2;C:50127;T:3849;total:53979 | iSNV |
| F35 | F35-30 | 3633 | NS1 | 0.2163 | A:36890;G:10188;C:3;T:5;total:47086 | iSNV |
| F35 | F35-30 | 3934 | NS2A | 0.0477 | A:5;G:4;C:3332;T:66481;total:69822 | iSNV |
| F35 | F35-30 | 3975 | NS2A | 0.0301 | A:7;G:1934;C:28;T:62283;total:64252 | iSNV |
| F35 | F35-30 | 4060 | NS2A | 0.2186 | A:1;G:2;C:9918;T:35437;total:45358 | iSNV |
| F35 | F35-30 | 4068 | NS2A | 0.0727 | A:43055;G:19;C:0;T:3380;total:46454 | iSNV |
| F35 | F35-30 | 4315 | NS2B | 0.0395 | A:2;G:0;C:2372;T:57592;total:59966 | iSNV |
| F35 | F35-30 | 4974 | NS3 | 0.2056 | A:13671;G:52818;C:2;T:2;total:66493 | iSNV |
| F35 | F35-30 | 5311 | NS3 | 0.3951 | A:2;G:179;C:20846;T:31726;total:52753 | iSNV |
| F35 | F35-30 | 5918 | NS3 | 0.0769 | A:5163;G:61891;C:0;T:1;total:67055 | iSNV |
| F35 | F35-30 | 6533 | NS4A | 0.8591 | A:0;G:0;C:39292;T:6449;total:45741 | iSNV |
| F35 | F35-30 | 7373 | NS4B | 0.0252 | A:1151;G:43511;C:0;T:912;total:45574 | iSNV |
| F35 | F35-30 | 7378 | NS4B | 0.2255 | A:0;G:0;C:36741;T:10699;total:47440 | iSNV |
| F35 | F35-30 | 7495 | NS4B | 0.0644 | A:4;G:3;C:2693;T:39090;total:41790 | iSNV |
| F35 | F35-30 | 7546 | NS4B | 0.2509 | A:39111;G:13105;C:9;T:2;total:52227 | iSNV |
| F35 | F35-30 | 7626 | NS4B | 0.0286 | A:3;G:1817;C:15;T:61560;total:63395 | iSNV |
| F35 | F35-30 | 7656 | NS4B | 0.0733 | A:24;G:54867;C:4344;T:3;total:59238 | iSNV |
| F35 | F35-30 | 7657 | NS4B | 0.8606 | A:8247;G:50885;C:3;T:6;total:59141 | iSNV |
| F35 | F35-30 | 7661 | NS4B | 0.0404 | A:2431;G:57734;C:5;T:3;total:60173 | iSNV |
| F35 | F35-30 | 8372 | NS5 | 0.2384 | A:1;G:3;C:17596;T:56191;total:73791 | iSNV |
| F35 | F35-30 | 8888 | NS5 | 0.0269 | A:51176;G:1417;C:0;T:1;total:52594 | iSNV |
| F35 | F35-30 | 8906 | NS5 | 0.0601 | A:1;G:4;C:3267;T:51011;total:54283 | iSNV |
| F35 | F35-30 | 9688 | NS5 | 0.949 | A:1;G:0;C:2675;T:49762;total:52438 | iSNV |
| F35 | F35-30 | 9932 | NS5 | 0.0312 | A:1614;G:0;C:34;T:50062;total:51710 | iSNV |
| F35 | F35-30 | 10069 | NS5 | 0.0215 | A:0;G:0;C:46805;T:1031;total:47836 | iSNV |
| F35 | F35-30 | 10071 | NS5 | 0.0324 | A:1563;G:1;C:2;T:46664;total:48230 | iSNV |
| F35 | F35-30 | 10086 | NS5 | 0.0309 | A:1530;G:1;C:4;T:47933;total:49468 | iSNV |
| F35 | F35-30 | 10723 | 3'-UTR | 0.0222 | A:4;G:0;C:51440;T:1171;total:52615 | iSNV |
| F35 | F35-4 | 806 | M | 0.1653 | A:3;G:9;C:8996;T:45394;total:54402 | iSNV |
| F35 | F35-4 | 1116 | E | 0.0475 | A:60022;G:2995;C:3;T:1;total:63021 | iSNV |
| F35 | F35-4 | 1296 | E | 0.3723 | A:5;G:1;C:22264;T:37526;total:59796 | iSNV |
| F35 | F35-4 | 1384 | E | 0.1821 | A:56034;G:26;C:8;T:12487;total:68555 | iSNV |
| F35 | F35-4 | 1390 | E | 0.1915 | A:54585;G:12946;C:60;T:5;total:67596 | iSNV |
| F35 | F35-4 | 1417 | E | 0.2323 | A:8;G:1;C:55319;T:16751;total:72079 | iSNV |
| F35 | F35-4 | 1474 | E | 0.0706 | A:65642;G:8;C:4995;T:33;total:70678 | iSNV |
| F35 | F35-4 | 1491 | E | 0.1856 | A:55701;G:12702;C:4;T:1;total:68408 | iSNV |
| F35 | F35-4 | 1679 | E | 0.0744 | A:59171;G:4759;C:1;T:2;total:63933 | iSNV |
| F35 | F35-4 | 1800 | E | 0.1841 | A:14711;G:3;C:24;T:65139;total:79877 | iSNV |
| F35 | F35-4 | 1911 | E | 0.3428 | A:41185;G:21513;C:48;T:6;total:62752 | iSNV |
| F35 | F35-4 | 1913 | E | 0.0328 | A:59546;G:16;C:2021;T:6;total:61589 | iSNV |

| | | | | | | |
|---|---|---|---|---|---|---|
| F35 | F35-4 | 2037 | E | 0.0576 | A:2945;G:2;C:48102;T:25;total:51074 | iSNV |
| F35 | F35-4 | 3184 | NS1 | 0.0314 | A:14;G:12;C:2630;T:80894;total:83550 | iSNV |
| F35 | F35-4 | 3428 | NS1 | 0.1876 | A:1;G:1;C:57520;T:13285;total:70807 | iSNV |
| F35 | F35-4 | 3761 | NS2A | 0.0278 | A:4;G:3;C:68075;T:1952;total:70034 | iSNV |
| F35 | F35-4 | 4068 | NS2A | 0.0288 | A:57380;G:1707;C:3;T:14;total:59104 | iSNV |
| F35 | F35-4 | 4367 | NS2B | 0.032 | A:3;G:26;C:1914;T:57773;total:59716 | iSNV |
| F35 | F35-4 | 5311 | NS3 | 0.4003 | A:4;G:272;C:23145;T:34387;total:57808 | iSNV |
| F35 | F35-4 | 6533 | NS4A | 0.034 | A:2;G:3;C:2090;T:59222;total:61317 | iSNV |
| F35 | F35-4 | 6738 | NS4A | 0.1175 | A:17;G:2;C:7419;T:55653;total:63091 | iSNV |
| F35 | F35-4 | 7007 | NS4A | 0.0302 | A:2220;G:71102;C:1;T:2;total:73325 | iSNV |
| F35 | F35-4 | 7061 | NS4A | 0.0312 | A:3;G:1;C:73936;T:2382;total:76322 | iSNV |
| F35 | F35-4 | 7381 | NS4B | 0.0313 | A:2;G:0;C:60016;T:1944;total:61962 | iSNV |
| F35 | F35-4 | 7495 | NS4B | 0.0966 | A:3;G:4;C:5254;T:49116;total:54377 | iSNV |
| F35 | F35-4 | 7626 | NS4B | 0.0777 | A:39;G:5728;C:14;T:67865;total:73646 | iSNV |
| F35 | F35-4 | 7656 | NS4B | 0.1732 | A:11718;G:55548;C:348;T:3;total:67617 | iSNV |
| F35 | F35-4 | 7657 | NS4B | 0.4275 | A:38548;G:28806;C:9;T:7;total:67370 | iSNV |
| F35 | F35-4 | 8153 | NS5 | 0.0315 | A:79971;G:2603;C:3;T:3;total:82580 | iSNV |
| F35 | F35-4 | 8194 | NS5 | 0.137 | A:66766;G:10611;C:12;T:16;total:77405 | iSNV |
| F35 | F35-4 | 8891 | NS5 | 0.0278 | A:5;G:0;C:1784;T:62183;total:63972 | iSNV |
| F35 | F35-4 | 9225 | NS5 | 0.1574 | A:14315;G:3;C:76556;T:25;total:90899 | iSNV |
| F35 | F35-4 | 9292 | NS5 | 0.1414 | A:7;G:73364;C:12084;T:2;total:85457 | iSNV |
| F35 | F35-4 | 9688 | NS5 | 0.242 | A:4;G:1;C:62435;T:19942;total:82382 | iSNV |
| F35 | F35-4 | 9926 | NS5 | 0.1157 | A:65819;G:8626;C:6;T:56;total:74507 | iSNV |
| F35 | F35-4 | 9932 | NS5 | 0.0337 | A:2450;G:8;C:38;T:69991;total:72487 | iSNV |
| F35 | F35-4 | 10068 | NS5 | 0.1497 | A:55614;G:9796;C:0;T:3;total:65413 | iSNV |
| F35 | F35-4 | 10069 | NS5 | 0.132 | A:2;G:1;C:56501;T:8595;total:65099 | iSNV |
| F35 | F35-4 | 10261 | NS5 | 0.1178 | A:2;G:1;C:75401;T:10072;total:85476 | iSNV |
| F35 | F35-4 | 10632 | 3'-UTR | 0.1099 | A:34;G:19;C:7731;T:62544;total:70328 | iSNV |
| F35 | F35-4 | 10664 | 3'-UTR | 0.1306 | A:10260;G:68266;C:6;T:1;total:78533 | iSNV |
| F35 | F35-4 | 10712 | 3'-UTR | 0.0275 | A:2;G:1;C:72849;T:2065;total:74917 | iSNV |
| F35 | F35-5 | 539 | M | 0.116 | A:9336;G:1;C:71085;T:26;total:80448 | iSNV |
| F35 | F35-5 | 1163 | E | 0.0262 | A:2062;G:3;C:42;T:76508;total:78615 | iSNV |
| F35 | F35-5 | 1296 | E | 0.3096 | A:4;G:0;C:21564;T:48076;total:69644 | iSNV |
| F35 | F35-5 | 1450 | E | 0.0335 | A:82189;G:2856;C:29;T:4;total:85078 | iSNV |
| F35 | F35-5 | 1468 | E | 0.1839 | A:14973;G:3;C:66384;T:27;total:81387 | iSNV |
| F35 | F35-5 | 1491 | E | 0.0209 | A:75850;G:1625;C:2;T:4;total:77481 | iSNV |
| F35 | F35-5 | 1800 | E | 0.02 | A:1803;G:5;C:35;T:87987;total:89830 | iSNV |
| F35 | F35-5 | 1911 | E | 0.7749 | A:15224;G:52362;C:19;T:1;total:67606 | iSNV |
| F35 | F35-5 | 1946 | E | 0.0524 | A:1;G:6;C:64151;T:3551;total:67709 | iSNV |
| F35 | F35-5 | 2200 | E | 0.0447 | A:4;G:1;C:67113;T:3147;total:70265 | iSNV |
| F35 | F35-5 | 3077 | NS1 | 0.0264 | A:5;G:5;C:67491;T:1835;total:69336 | iSNV |
| F35 | F35-5 | 3081 | NS1 | 0.2128 | A:54879;G:14844;C:1;T:3;total:69727 | iSNV |
| F35 | F35-5 | 3125 | NS1 | 0.0294 | A:1;G:2;C:71153;T:2157;total:73313 | iSNV |
| F35 | F35-5 | 3634 | NS1 | 0.0379 | A:10;G:7;C:2410;T:61095;total:63522 | iSNV |
| F35 | F35-5 | 3692 | NS1 | 0.0344 | A:2;G:0;C:1977;T:55405;total:57384 | iSNV |
| F35 | F35-5 | 3910 | NS2A | 0.0271 | A:76381;G:2129;C:1;T:8;total:78519 | iSNV |
| F35 | F35-5 | 4034 | NS2A | 0.0381 | A:58054;G:2300;C:2;T:6;total:60362 | iSNV |
| F35 | F35-5 | 4069 | NS2A | 0.0453 | A:31;G:2663;C:145;T:55937;total:58776 | iSNV |
| F35 | F35-5 | 4550 | NS2B | 0.0382 | A:14;G:11;C:2778;T:69835;total:72638 | iSNV |
| F35 | F35-5 | 5311 | NS3 | 0.9383 | A:1;G:103;C:59867;T:3948;total:63919 | iSNV |
| F35 | F35-5 | 5348 | NS3 | 0.0241 | A:69404;G:1716;C:36;T:4;total:71160 | iSNV |
| F35 | F35-5 | 6637 | NS4A | 0.618 | A:2;G:0;C:32000;T:19788;total:51790 | iSNV |
| F35 | F35-5 | 7131 | NS4A | 0.0791 | A:16;G:3;C:5485;T:63835;total:69339 | iSNV |
| F35 | F35-5 | 7495 | NS4B | 0.1569 | A:3;G:1;C:7761;T:41687;total:49452 | iSNV |
| F35 | F35-5 | 7543 | NS4B | 0.1588 | A:49097;G:9273;C:1;T:2;total:58373 | iSNV |
| F35 | F35-5 | 7656 | NS4B | 0.7573 | A:310;G:15066;C:46675;T:12;total:62063 | iSNV |
| F35 | F35-5 | 7657 | NS4B | 0.0314 | A:60249;G:1957;C:2;T:1;total:62209 | iSNV |
| F35 | F35-5 | 8093 | NS5 | 0.17 | A:5;G:8;C:62704;T:12854;total:75571 | iSNV |
| F35 | F35-5 | 8688 | NS5 | 0.0277 | A:34;G:62723;C:12;T:1792;total:64561 | iSNV |
| F35 | F35-5 | 9302 | NS5 | 0.0357 | A:31;G:6;C:2667;T:71823;total:74527 | iSNV |
| F35 | F35-5 | 9688 | NS5 | 0.0408 | A:1;G:1;C:72071;T:3072;total:75145 | iSNV |
| F35 | F35-5 | 9887 | NS5 | 0.1642 | A:3;G:3;C:11774;T:59905;total:71685 | iSNV |
| F35 | F35-5 | 10092 | NS5 | 0.2398 | A:917;G:46815;C:15063;T:5;total:62800 | iSNV |
| F35 | F35-5 | 10420 | 3'-UTR | 0.0341 | A:15;G:1;C:2257;T:63872;total:66145 | iSNV |
| F35 | F35-5 | 10811 | 3'-UTR | 0.0272 | A:1487;G:52977;C:1;T:21;total:54486 | iSNV |
| F35 | F35-7 | 505 | M | 0.5405 | A:4;G:1;C:45329;T:38552;total:83886 | iSNV |
| F35 | F35-7 | 697 | M | 0.0891 | A:11;G:0;C:6483;T:66207;total:72701 | iSNV |
| F35 | F35-7 | 1116 | E | 0.0712 | A:60528;G:4643;C:2;T:4;total:65177 | iSNV |
| F35 | F35-7 | 1296 | E | 0.0498 | A:2;G:0;C:3368;T:64179;total:67549 | iSNV |
| F35 | F35-7 | 1417 | E | 0.0908 | A:2;G:0;C:71655;T:7157;total:78814 | iSNV |
| F35 | F35-7 | 1496 | E | 0.0216 | A:3;G:0;C:1631;T:73566;total:75200 | iSNV |
| F35 | F35-7 | 1911 | E | 0.8011 | A:11996;G:48295;C:18;T:2;total:60311 | iSNV |
| F35 | F35-7 | 3139 | NS1 | 0.2312 | A:54151;G:16294;C:4;T:2;total:70451 | iSNV |
| F35 | F35-7 | 5311 | NS3 | 0.1813 | A:0;G:1558;C:11430;T:50023;total:63011 | iSNV |
| F35 | F35-7 | 5490 | NS3 | 0.0236 | A:79899;G:1932;C:11;T:3;total:81845 | iSNV |
| F35 | F35-7 | 5835 | NS3 | 0.0211 | A:1802;G:83202;C:7;T:7;total:85018 | iSNV |
| F35 | F35-7 | 6533 | NS4A | 0.7334 | A:3;G:2;C:41378;T:15047;total:56430 | iSNV |
| F35 | F35-7 | 7495 | NS4B | 0.216 | A:2;G:2;C:9884;T:35854;total:45742 | iSNV |
| F35 | F35-7 | 7543 | NS4B | 0.1531 | A:45813;G:8285;C:4;T:0;total:54102 | iSNV |
| F35 | F35-7 | 7657 | NS4B | 0.6001 | A:23751;G:35625;C:3;T:4;total:59383 | iSNV |
| F35 | F35-7 | 8140 | NS5 | 0.5766 | A:34239;G:46608;C:9;T:3;total:80859 | iSNV |
| F35 | F35-7 | 9254 | NS5 | 0.0816 | A:79160;G:7037;C:0;T:3;total:86200 | iSNV |

| | | | | | | |
|---|---|---|---|---|---|---|
| F35 | F35-7 | 9370 | NS5 | 0.5886 | A:52786;G:30;C:15;T:36934;total:89765 | iSNV |
| F35 | F35-7 | 9603 | NS5 | 0.0281 | A:76230;G:2207;C:0;T:2;total:78439 | iSNV |
| F35 | F35-7 | 9688 | NS5 | 0.986 | A:7;G:0;C:1026;T:72505;total:73538 | SNP |
| F35 | F35-7 | 10069 | NS5 | 0.0333 | A:0;G:4;C:54492;T:1882;total:56378 | iSNV |
| F35 | F35-7 | 10379 | NS5 | 0.1463 | A:57498;G:9856;C:4;T:1;total:67359 | iSNV |
| F35 | F35-7 | 10811 | 3'-UTR | 0.0606 | A:2956;G:45790;C:2;T:2;total:48750 | iSNV |
| F35 | F35-8 | 398 | C | 0.0272 | A:2135;G:76093;C:5;T:8;total:78241 | iSNV |
| F35 | F35-8 | 603 | M | 0.0224 | A:5;G:3;C:1573;T:68526;total:70107 | iSNV |
| F35 | F35-8 | 806 | M | 0.0899 | A:1;G:9;C:5103;T:51643;total:56756 | iSNV |
| F35 | F35-8 | 1116 | E | 0.0925 | A:61186;G:6244;C:1;T:2;total:67433 | iSNV |
| F35 | F35-8 | 1296 | E | 0.5049 | A:4;G:4;C:32418;T:31798;total:64224 | iSNV |
| F35 | F35-8 | 1384 | E | 0.2007 | A:58789;G:6;C:7;T:14771;total:73573 | iSNV |
| F35 | F35-8 | 1390 | E | 0.2116 | A:56802;G:15262;C:33;T:3;total:72100 | iSNV |
| F35 | F35-8 | 1417 | E | 0.4761 | A:4;G:0;C:40116;T:36464;total:76584 | iSNV |
| F35 | F35-8 | 1474 | E | 0.0894 | A:67319;G:2;C:6616;T:16;total:73953 | iSNV |
| F35 | F35-8 | 1491 | E | 0.1979 | A:57072;G:14086;C:4;T:2;total:71164 | iSNV |
| F35 | F35-8 | 1679 | E | 0.0958 | A:62123;G:6583;C:1;T:3;total:68710 | iSNV |
| F35 | F35-8 | 1800 | E | 0.1974 | A:17019;G:3;C:13;T:69138;total:86173 | iSNV |
| F35 | F35-8 | 1911 | E | 0.1822 | A:53257;G:11936;C:317;T:0;total:65510 | iSNV |
| F35 | F35-8 | 1948 | E | 0.0295 | A:9;G:4;C:1891;T:61982;total:63886 | iSNV |
| F35 | F35-8 | 2037 | E | 0.0873 | A:4711;G:6;C:49200;T:14;total:53931 | iSNV |
| F35 | F35-8 | 3184 | NS1 | 0.0761 | A:10;G:3;C:5774;T:70050;total:75837 | iSNV |
| F35 | F35-8 | 4367 | NS2B | 0.0714 | A:1;G:20;C:4756;T:61788;total:66565 | iSNV |
| F35 | F35-8 | 5311 | NS3 | 0.5413 | A:2;G:140;C:32337;T:27523;total:60002 | iSNV |
| F35 | F35-8 | 6990 | NS4A | 0.0292 | A:61767;G:1860;C:1;T:2;total:63630 | iSNV |
| F35 | F35-8 | 7061 | NS4A | 0.0331 | A:2;G:3;C:65691;T:2250;total:67946 | iSNV |
| F35 | F35-8 | 7381 | NS4B | 0.0216 | A:2;G:0;C:54642;T:1208;total:55852 | iSNV |
| F35 | F35-8 | 7495 | NS4B | 0.0728 | A:5;G:2;C:3613;T:45955;total:49575 | iSNV |
| F35 | F35-8 | 7626 | NS4B | 0.1383 | A:30;G:9483;C:23;T:58988;total:68524 | iSNV |
| F35 | F35-8 | 7656 | NS4B | 0.3353 | A:21092;G:41557;C:248;T:4;total:62901 | iSNV |
| F35 | F35-8 | 7657 | NS4B | 0.2026 | A:50085;G:12730;C:1;T:5;total:62821 | iSNV |
| F35 | F35-8 | 8194 | NS5 | 0.163 | A:57137;G:11136;C:2;T:8;total:68283 | iSNV |
| F35 | F35-8 | 9225 | NS5 | 0.2035 | A:16695;G:1;C:65314;T:7;total:82017 | iSNV |
| F35 | F35-8 | 9292 | NS5 | 0.0911 | A:5;G:69505;C:6968;T:1;total:76479 | iSNV |
| F35 | F35-8 | 9688 | NS5 | 0.3837 | A:1;G:1;C:43411;T:27035;total:70448 | iSNV |
| F35 | F35-8 | 9926 | NS5 | 0.2993 | A:44907;G:19229;C:3;T:104;total:64243 | iSNV |
| F35 | F35-8 | 9932 | NS5 | 0.0262 | A:1646;G:46;C:8;T:60927;total:62627 | iSNV |
| F35 | F35-8 | 10068 | NS5 | 0.0955 | A:51965;G:5489;C:0;T:5;total:57459 | iSNV |
| F35 | F35-8 | 10069 | NS5 | 0.1304 | A:0;G:3;C:49679;T:7456;total:57138 | iSNV |
| F35 | F35-8 | 10261 | NS5 | 0.146 | A:1;G:2;C:64877;T:11100;total:75980 | iSNV |
| F35 | F35-8 | 10664 | 3'-UTR | 0.0898 | A:6087;G:61661;C:6;T:4;total:67758 | iSNV |

Table S5: Discontinuous iSNV sites in the same transmission chain of BHK cells

| Type | Sample | Generation | Transmisson chain | Position | Gene | Mutation | MuAF |
|---|---|---|---|---|---|---|---|
| iSNV | F21-1 | F21 | P1 | 563 | M | S | 0.0392 |
| SNP | F50-1 | F50 | P1 | 563 | M | S | 0.9822 |
| iSNV | F55-1 | F55 | P1 | 563 | M | S | 0.9678 |
| iSNV | F30-1 | F30 | P1 | 897 | M | N | 0.02 |
| iSNV | F40-1 | F40 | P1 | 897 | M | N | 0.2794 |
| iSNV | F45-1 | F45 | P1 | 897 | M | N | 0.1615 |
| iSNV | F21-1 | F21 | P1 | 1772 | E | S | 0.0267 |
| iSNV | F40-1 | F40 | P1 | 1772 | E | S | 0.0224 |
| SNP | F50-1 | F50 | P1 | 1772 | E | S | 0.9847 |
| iSNV | F55-1 | F55 | P1 | 1772 | E | S | 0.9715 |
| iSNV | F24-1 | F24 | P1 | 2213 | E | S | 0.0219 |
| iSNV | F30-1 | F30 | P1 | 2213 | E | S | 0.0319 |
| iSNV | F33-1 | F33 | P1 | 2213 | E | S | 0.0241 |
| iSNV | F21-1 | F21 | P1 | 2372 | E | S | 0.045 |
| SNP | F50-1 | F50 | P1 | 2372 | E | S | 0.9889 |
| iSNV | F55-1 | F55 | P1 | 2372 | E | S | 0.972 |
| iSNV | F24-1 | F24 | P1 | 2376 | E | S | 0.0272 |
| iSNV | F27-1 | F27 | P1 | 2376 | E | S | 0.0236 |
| iSNV | F30-1 | F30 | P1 | 2376 | E | S | 0.0308 |
| iSNV | F33-1 | F33 | P1 | 2376 | E | S | 0.0234 |
| iSNV | F36-1 | F36 | P1 | 2376 | E | S | 0.2596 |
| iSNV | F45-1 | F45 | P1 | 2376 | E | S | 0.0223 |
| iSNV | F21-1 | F21 | P1 | 3869 | NS2A | S | 0.6787 |
| iSNV | F24-1 | F24 | P1 | 3869 | NS2A | S | 0.8237 |
| iSNV | F27-1 | F27 | P1 | 3869 | NS2A | S | 0.8546 |
| iSNV | F30-1 | F30 | P1 | 3869 | NS2A | S | 0.8249 |
| iSNV | F33-1 | F33 | P1 | 3869 | NS2A | S | 0.8268 |
| iSNV | F36-1 | F36 | P1 | 3869 | NS2A | S | 0.8852 |
| iSNV | F40-1 | F40 | P1 | 3869 | NS2A | S | 0.9638 |
| SNP | F45-1 | F45 | P1 | 3869 | NS2A | S | 0.9949 |
| iSNV | F55-1 | F55 | P1 | 3869 | NS2A | S | 0.0229 |
| iSNV | F21-1 | F21 | P1 | 3962 | NS2A | S | 0.0415 |
| SNP | F50-1 | F50 | P1 | 3962 | NS2A | S | 0.9833 |
| iSNV | F55-1 | F55 | P1 | 3962 | NS2A | S | 0.9622 |
| iSNV | F21-1 | F21 | P1 | 4712 | NS3 | S | 0.0258 |
| SNP | F50-1 | F50 | P1 | 4712 | NS3 | S | 0.9836 |
| iSNV | F55-1 | F55 | P1 | 4712 | NS3 | S | 0.9571 |
| iSNV | F21-1 | F21 | P1 | 5311 | NS3 | N | 0.0225 |
| SNP | F50-1 | F50 | P1 | 5311 | NS3 | N | 0.9831 |
| iSNV | F55-1 | F55 | P1 | 5311 | NS3 | N | 0.9636 |
| iSNV | F36-1 | F36 | P1 | 7633 | NS4B | N | 0.3852 |
| iSNV | F40-1 | F40 | P1 | 7633 | NS4B | N | 0.9546 |
| SNP | F45-1 | F45 | P1 | 7633 | NS4B | N | 0.9932 |
| iSNV | F55-1 | F55 | P1 | 7633 | NS4B | N | 0.0254 |
| iSNV | F21-1 | F21 | P1 | 8282 | NS5 | S | 0.0251 |
| SNP | F50-1 | F50 | P1 | 8282 | NS5 | S | 0.9883 |
| iSNV | F55-1 | F55 | P1 | 8282 | NS5 | S | 0.9726 |
| iSNV | F21-1 | F21 | P1 | 8900 | NS5 | N | 0.0392 |
| SNP | F50-1 | F50 | P1 | 8900 | NS5 | N | 0.9851 |
| iSNV | F55-1 | F55 | P1 | 8900 | NS5 | N | 0.9638 |
| iSNV | F21-1 | F21 | P1 | 9446 | NS5 | S | 0.0267 |
| SNP | F50-1 | F50 | P1 | 9446 | NS5 | S | 0.984 |

Table S5: Discontinuous iSNV sites in the same transmission chain of BHK cells (Continued)

| Type | Sample | Generation | Transmission chain | Position | Gene | Mutation | MuAF |
|---|---|---|---|---|---|---|---|
| iSNV | F55-1 | F55 | P1 | 9446 | NS5 | S | 0.9642 |
| iSNV | F36-1 | F36 | P1 | 10419 | 3'-UTR | NC | 0.0231 |
| iSNV | F45-1 | F45 | P1 | 10419 | 3'-UTR | NC | 0.0333 |
| iSNV | F21-1 | F21 | P1 | 10447 | 3'-UTR | NC | 0.097 |
| iSNV | F24-1 | F24 | P1 | 10447 | 3'-UTR | NC | 0.0282 |
| iSNV | F30-1 | F30 | P1 | 10447 | 3'-UTR | NC | 0.022 |
| iSNV | F33-1 | F33 | P1 | 10447 | 3'-UTR | NC | 0.0279 |
| iSNV | F36-1 | F36 | P1 | 10447 | 3'-UTR | NC | 0.0228 |
| iSNV | F45-1 | F45 | P1 | 10447 | 3'-UTR | NC | 0.0329 |
| iSNV | F24-10 | F24 | P10 | 1283 | E | S | 0.0208 |
| iSNV | F27-10 | F27 | P10 | 1283 | E | S | 0.0221 |
| iSNV | F36-10 | F36 | P10 | 1283 | E | S | 0.0348 |
| iSNV | F36-10 | F36 | P10 | 5256 | NS3 | S | 0.4649 |
| iSNV | F45-10 | F45 | P10 | 5256 | NS3 | S | 0.9748 |
| iSNV | F36-10 | F36 | P10 | 5944 | NS3 | N | 0.0267 |
| iSNV | F45-10 | F45 | P10 | 5944 | NS3 | N | 0.1118 |
| iSNV | F24-10 | F24 | P10 | 6398 | NS3 | S | 0.0256 |
| iSNV | F27-10 | F27 | P10 | 6398 | NS3 | S | 0.0276 |
| iSNV | F33-10 | F33 | P10 | 6398 | NS3 | S | 0.0247 |
| iSNV | F36-10 | F36 | P10 | 7264 | NS4A | N | 0.4451 |
| SNP | F40-10 | F40 | P10 | 7264 | NS4A | N | 1 |
| SNP | F45-10 | F45 | P10 | 7264 | NS4A | N | 0.9979 |
| iSNV | F55-10 | F55 | P10 | 7264 | NS4A | N | 0.0688 |
| iSNV | F45-10 | F45 | P10 | 7657 | NS4B | N | 0.025 |
| iSNV | F55-10 | F55 | P10 | 7657 | NS4B | N | 0.055 |
| iSNV | F30-11 | F30 | P11 | 1514 | E | N | 0.0531 |
| iSNV | F33-11 | F33 | P11 | 1514 | E | N | 0.1183 |
| iSNV | F36-11 | F36 | P11 | 1514 | E | N | 0.0459 |
| iSNV | F45-11 | F45 | P11 | 1514 | E | N | 0.1015 |
| iSNV | F50-11 | F50 | P11 | 1514 | E | N | 0.1984 |
| iSNV | F55-11 | F55 | P11 | 1514 | E | N | 0.148 |
| iSNV | F36-11 | F36 | P11 | 3495 | NS1 | N | 0.0463 |
| iSNV | F45-11 | F45 | P11 | 3495 | NS1 | N | 0.0734 |
| iSNV | F36-11 | F36 | P11 | 6509 | NS4A | S | 0.0224 |
| iSNV | F45-11 | F45 | P11 | 6509 | NS4A | S | 0.0697 |
| iSNV | F50-11 | F50 | P11 | 6509 | NS4A | S | 0.0452 |
| iSNV | F30-11 | F30 | P11 | 10428 | 3'-UTR | NC | 0.0478 |
| iSNV | F33-11 | F33 | P11 | 10428 | 3'-UTR | NC | 0.0534 |
| iSNV | F36-11 | F36 | P11 | 10428 | 3'-UTR | NC | 0.0378 |
| iSNV | F45-11 | F45 | P11 | 10428 | 3'-UTR | NC | 0.2279 |
| iSNV | F50-11 | F50 | P11 | 10428 | 3'-UTR | NC | 0.7763 |
| iSNV | F55-11 | F55 | P11 | 10428 | 3'-UTR | NC | 0.9224 |
| iSNV | F36-12 | F36 | P12 | 332 | C | S | 0.0507 |
| iSNV | F50-12 | F50 | P12 | 332 | C | S | 0.0509 |
| iSNV | F55-12 | F55 | P12 | 332 | C | S | 0.0952 |
| iSNV | F27-12 | F27 | P12 | 1083 | E | N | 0.0378 |
| iSNV | F30-12 | F30 | P12 | 1083 | E | N | 0.078 |
| iSNV | F33-12 | F33 | P12 | 1083 | E | N | 0.0789 |
| iSNV | F40-12 | F40 | P12 | 1083 | E | N | 0.0288 |
| iSNV | F45-12 | F45 | P12 | 1083 | E | N | 0.0329 |
| iSNV | F50-12 | F50 | P12 | 1083 | E | N | 0.2313 |
| iSNV | F55-12 | F55 | P12 | 1083 | E | N | 0.1378 |
| iSNV | F40-12 | F40 | P12 | 1453 | E | N | 0.046 |

Table S5: Discontinuous iSNV sites in the same transmission chain of BHK cells (Continued)

| Type | Sample | Generation | Transmission chain | Position | Gene | Mutation | MuAF |
|---|---|---|---|---|---|---|---|
| iSNV | F45-12 | F45 | P12 | 1453 | E | N | 0.0476 |
| iSNV | F55-12 | F55 | P12 | 1453 | E | N | 0.0315 |
| iSNV | F40-12 | F40 | P12 | 6969 | NS4A | N | 0.0344 |
| iSNV | F50-12 | F50 | P12 | 6969 | NS4A | N | 0.0582 |
| iSNV | F55-12 | F55 | P12 | 6969 | NS4A | N | 0.1144 |
| iSNV | F36-12 | F36 | P12 | 7178 | NS4A | S | 0.0504 |
| iSNV | F50-12 | F50 | P12 | 7178 | NS4A | S | 0.0565 |
| iSNV | F55-12 | F55 | P12 | 7178 | NS4A | S | 0.1096 |
| iSNV | F36-12 | F36 | P12 | 7561 | NS4B | N | 0.0523 |
| iSNV | F40-12 | F40 | P12 | 7561 | NS4B | N | 0.06 |
| iSNV | F50-12 | F50 | P12 | 7561 | NS4B | N | 0.0609 |
| iSNV | F55-12 | F55 | P12 | 7561 | NS4B | N | 0.1137 |
| iSNV | F27-12 | F27 | P12 | 10592 | 3'-UTR | NC | 0.0221 |
| iSNV | F30-12 | F30 | P12 | 10592 | 3'-UTR | NC | 0.0223 |
| iSNV | F36-12 | F36 | P12 | 10592 | 3'-UTR | NC | 0.0273 |
| iSNV | F21-13 | F21 | P13 | 998 | E | S | 0.2145 |
| iSNV | F24-13 | F24 | P13 | 998 | E | S | 0.0777 |
| iSNV | F27-13 | F27 | P13 | 998 | E | S | 0.0225 |
| iSNV | F33-13 | F33 | P13 | 998 | E | S | 0.0205 |
| iSNV | F36-13 | F36 | P13 | 998 | E | S | 0.0202 |
| iSNV | F21-13 | F21 | P13 | 1772 | E | S | 0.0204 |
| iSNV | F40-13 | F40 | P13 | 1772 | E | S | 0.0235 |
| iSNV | F21-13 | F21 | P13 | 2372 | E | S | 0.02 |
| iSNV | F40-13 | F40 | P13 | 2372 | E | S | 0.0451 |
| iSNV | F21-13 | F21 | P13 | 10259 | NS5 | S | 0.1918 |
| iSNV | F24-13 | F24 | P13 | 10259 | NS5 | S | 0.0773 |
| iSNV | F27-13 | F27 | P13 | 10259 | NS5 | S | 0.0237 |
| iSNV | F33-13 | F33 | P13 | 10259 | NS5 | S | 0.0206 |
| iSNV | F36-13 | F36 | P13 | 10259 | NS5 | S | 0.0224 |
| iSNV | F36-14 | F36 | P14 | 836 | M | S | 0.0294 |
| iSNV | F45-14 | F45 | P14 | 836 | M | S | 0.0852 |
| iSNV | F50-14 | F50 | P14 | 836 | M | S | 0.3726 |
| iSNV | F55-14 | F55 | P14 | 836 | M | S | 0.5874 |
| iSNV | F36-14 | F36 | P14 | 1057 | E | N | 0.0442 |
| iSNV | F45-14 | F45 | P14 | 1057 | E | N | 0.1127 |
| iSNV | F50-14 | F50 | P14 | 1057 | E | N | 0.0999 |
| iSNV | F55-14 | F55 | P14 | 1057 | E | N | 0.0716 |
| iSNV | F24-14 | F24 | P14 | 1117 | E | N | 0.072 |
| iSNV | F27-14 | F27 | P14 | 1117 | E | N | 0.2038 |
| iSNV | F30-14 | F30 | P14 | 1117 | E | N | 0.2901 |
| iSNV | F33-14 | F33 | P14 | 1117 | E | N | 0.3305 |
| iSNV | F36-14 | F36 | P14 | 1117 | E | N | 0.316 |
| iSNV | F45-14 | F45 | P14 | 1117 | E | N | 0.2829 |
| iSNV | F50-14 | F50 | P14 | 1117 | E | N | 0.1535 |
| iSNV | F55-14 | F55 | P14 | 1117 | E | N | 0.0412 |
| iSNV | F33-14 | F33 | P14 | 2369 | E | N | 0.0218 |
| iSNV | F45-14 | F45 | P14 | 2369 | E | N | 0.2681 |
| iSNV | F50-14 | F50 | P14 | 2369 | E | N | 0.158 |
| iSNV | F55-14 | F55 | P14 | 2369 | E | N | 0.0456 |
| iSNV | F24-14 | F24 | P14 | 4187 | NS2A | S | 0.0203 |
| iSNV | F36-14 | F36 | P14 | 4187 | NS2A | S | 0.0392 |
| iSNV | F24-14 | F24 | P14 | 5558 | NS3 | S | 0.0651 |
| iSNV | F27-14 | F27 | P14 | 5558 | NS3 | S | 0.2076 |

Table S5: Discontinuous iSNV sites in the same transmission chain of BHK cells (Continued)

| Type | Sample | Generation | Transmission chain | Position | Gene | Mutation | MuAF |
|---|---|---|---|---|---|---|---|
| iSNV | F30-14 | F30 | P14 | 5558 | NS3 | S | 0.2594 |
| iSNV | F33-14 | F33 | P14 | 5558 | NS3 | S | 0.3081 |
| iSNV | F36-14 | F36 | P14 | 5558 | NS3 | S | 0.2636 |
| iSNV | F45-14 | F45 | P14 | 5558 | NS3 | S | 0.288 |
| iSNV | F50-14 | F50 | P14 | 5558 | NS3 | S | 0.1511 |
| iSNV | F55-14 | F55 | P14 | 5558 | NS3 | S | 0.0438 |
| iSNV | F40-14 | F40 | P14 | 6523 | NS4A | N | 0.0495 |
| iSNV | F50-14 | F50 | P14 | 6523 | NS4A | N | 0.0202 |
| iSNV | F27-14 | F27 | P14 | 10428 | 3'-UTR | NC | 0.0271 |
| iSNV | F30-14 | F30 | P14 | 10428 | 3'-UTR | NC | 0.0464 |
| iSNV | F33-14 | F33 | P14 | 10428 | 3'-UTR | NC | 0.059 |
| iSNV | F36-14 | F36 | P14 | 10428 | 3'-UTR | NC | 0.1185 |
| iSNV | F45-14 | F45 | P14 | 10428 | 3'-UTR | NC | 0.3925 |
| iSNV | F50-14 | F50 | P14 | 10428 | 3'-UTR | NC | 0.4057 |
| iSNV | F55-14 | F55 | P14 | 10428 | 3'-UTR | NC | 0.4187 |
| iSNV | F21-14 | F21 | P14 | 10447 | 3'-UTR | NC | 0.0909 |
| iSNV | F24-14 | F24 | P14 | 10447 | 3'-UTR | NC | 0.056 |
| iSNV | F30-14 | F30 | P14 | 10447 | 3'-UTR | NC | 0.0221 |
| iSNV | F33-14 | F33 | P14 | 10447 | 3'-UTR | NC | 0.0271 |
| iSNV | F36-14 | F36 | P14 | 10447 | 3'-UTR | NC | 0.0313 |
| iSNV | F21-14 | F21 | P14 | 10566 | 3'-UTR | NC | 0.1081 |
| iSNV | F24-14 | F24 | P14 | 10566 | 3'-UTR | NC | 0.0768 |
| iSNV | F27-14 | F27 | P14 | 10566 | 3'-UTR | NC | 0.1008 |
| iSNV | F30-14 | F30 | P14 | 10566 | 3'-UTR | NC | 0.1459 |
| iSNV | F33-14 | F33 | P14 | 10566 | 3'-UTR | NC | 0.1727 |
| iSNV | F36-14 | F36 | P14 | 10566 | 3'-UTR | NC | 0.3971 |
| iSNV | F45-14 | F45 | P14 | 10566 | 3'-UTR | NC | 0.6283 |
| iSNV | F50-14 | F50 | P14 | 10566 | 3'-UTR | NC | 0.7968 |
| iSNV | F55-14 | F55 | P14 | 10566 | 3'-UTR | NC | 0.9352 |
| iSNV | F21-15 | F21 | P15 | 803 | M | N | 0.0831 |
| iSNV | F45-15 | F45 | P15 | 803 | M | N | 0.0787 |
| iSNV | F50-15 | F50 | P15 | 803 | M | N | 0.3366 |
| iSNV | F55-15 | F55 | P15 | 803 | M | N | 0.316 |
| iSNV | F21-15 | F21 | P15 | 998 | E | S | 0.1295 |
| iSNV | F24-15 | F24 | P15 | 998 | E | S | 0.0886 |
| iSNV | F27-15 | F27 | P15 | 998 | E | S | 0.0887 |
| iSNV | F30-15 | F30 | P15 | 998 | E | S | 0.0904 |
| iSNV | F33-15 | F33 | P15 | 998 | E | S | 0.1957 |
| iSNV | F36-15 | F36 | P15 | 998 | E | S | 0.4912 |
| iSNV | F45-15 | F45 | P15 | 998 | E | S | 0.108 |
| iSNV | F50-15 | F50 | P15 | 998 | E | S | 0.1643 |
| iSNV | F55-15 | F55 | P15 | 998 | E | S | 0.2089 |
| iSNV | F36-15 | F36 | P15 | 1512 | E | N | 0.104 |
| iSNV | F45-15 | F45 | P15 | 1512 | E | N | 0.1259 |
| iSNV | F50-15 | F50 | P15 | 1512 | E | N | 0.3749 |
| iSNV | F55-15 | F55 | P15 | 1512 | E | N | 0.3335 |
| iSNV | F30-15 | F30 | P15 | 1797 | E | N | 0.0258 |
| iSNV | F45-15 | F45 | P15 | 1797 | E | N | 0.0467 |
| iSNV | F50-15 | F50 | P15 | 1797 | E | N | 0.1303 |
| iSNV | F55-15 | F55 | P15 | 1797 | E | N | 0.1917 |
| iSNV | F36-15 | F36 | P15 | 2274 | E | N | 0.1089 |
| iSNV | F45-15 | F45 | P15 | 2274 | E | N | 0.0308 |
| iSNV | F21-15 | F21 | P15 | 4697 | NS3 | S | 0.0721 |

Table S5: Discontinuous iSNV sites in the same transmission chain of BHK cells (Continued)

| Type | Sample | Generation | Transmission chain | Position | Gene | Mutation | MuAF |
|---|---|---|---|---|---|---|---|
| iSNV | F24-15 | F24 | P15 | 4697 | NS3 | S | 0.0791 |
| iSNV | F27-15 | F27 | P15 | 4697 | NS3 | S | 0.0775 |
| iSNV | F30-15 | F30 | P15 | 4697 | NS3 | S | 0.0794 |
| iSNV | F33-15 | F33 | P15 | 4697 | NS3 | S | 0.1813 |
| iSNV | F36-15 | F36 | P15 | 4697 | NS3 | S | 0.4227 |
| iSNV | F45-15 | F45 | P15 | 4697 | NS3 | S | 0.1184 |
| iSNV | F50-15 | F50 | P15 | 4697 | NS3 | S | 0.2091 |
| iSNV | F55-15 | F55 | P15 | 4697 | NS3 | S | 0.2797 |
| iSNV | F36-15 | F36 | P15 | 4974 | NS3 | N | 0.0267 |
| iSNV | F55-15 | F55 | P15 | 4974 | NS3 | N | 0.0264 |
| iSNV | F33-15 | F33 | P15 | 5558 | NS3 | S | 0.3328 |
| iSNV | F36-15 | F36 | P15 | 5558 | NS3 | S | 0.2152 |
| iSNV | F45-15 | F45 | P15 | 5598 | NS3 | N | 0.0306 |
| iSNV | F50-15 | F50 | P15 | 5598 | NS3 | N | 0.0277 |
| iSNV | F55-15 | F55 | P15 | 5598 | NS3 | N | 0.0257 |
| iSNV | F21-15 | F21 | P15 | 5952 | NS3 | N | 0.0644 |
| iSNV | F24-15 | F24 | P15 | 5952 | NS3 | N | 0.0804 |
| iSNV | F27-15 | F27 | P15 | 5952 | NS3 | N | 0.0806 |
| iSNV | F30-15 | F30 | P15 | 5952 | NS3 | N | 0.0908 |
| iSNV | F33-15 | F33 | P15 | 5952 | NS3 | N | 0.1863 |
| iSNV | F36-15 | F36 | P15 | 5952 | NS3 | N | 0.4176 |
| iSNV | F45-15 | F45 | P15 | 5952 | NS3 | N | 0.1192 |
| iSNV | F50-15 | F50 | P15 | 5952 | NS3 | N | 0.2109 |
| iSNV | F55-15 | F55 | P15 | 5952 | NS3 | N | 0.2911 |
| iSNV | F36-15 | F36 | P15 | 7561 | NS4B | N | 0.0203 |
| iSNV | F55-15 | F55 | P15 | 7561 | NS4B | N | 0.0225 |
| iSNV | F21-15 | F21 | P15 | 7735 | NS5 | N | 0.0599 |
| iSNV | F45-15 | F45 | P15 | 7735 | NS5 | N | 0.0793 |
| iSNV | F50-15 | F50 | P15 | 7735 | NS5 | N | 0.3119 |
| iSNV | F55-15 | F55 | P15 | 7735 | NS5 | N | 0.2801 |
| iSNV | F21-15 | F21 | P15 | 8518 | NS5 | N | 0.0691 |
| iSNV | F45-15 | F45 | P15 | 8518 | NS5 | N | 0.0763 |
| iSNV | F50-15 | F50 | P15 | 8518 | NS5 | N | 0.3043 |
| iSNV | F55-15 | F55 | P15 | 8518 | NS5 | N | 0.2728 |
| iSNV | F33-15 | F33 | P15 | 9370 | NS5 | N | 0.1296 |
| iSNV | F36-15 | F36 | P15 | 9370 | NS5 | N | 0.2684 |
| iSNV | F45-15 | F45 | P15 | 9370 | NS5 | N | 0.0838 |
| iSNV | F50-15 | F50 | P15 | 9370 | NS5 | N | 0.1634 |
| iSNV | F55-15 | F55 | P15 | 9370 | NS5 | N | 0.2743 |
| iSNV | F24-15 | F24 | P15 | 9634 | NS5 | N | 0.0285 |
| iSNV | F27-15 | F27 | P15 | 9634 | NS5 | N | 0.0542 |
| iSNV | F30-15 | F30 | P15 | 9634 | NS5 | N | 0.0568 |
| iSNV | F33-15 | F33 | P15 | 9634 | NS5 | N | 0.0229 |
| iSNV | F36-15 | F36 | P15 | 9634 | NS5 | N | 0.0226 |
| iSNV | F45-15 | F45 | P15 | 9634 | NS5 | N | 0.0338 |
| iSNV | F24-15 | F24 | P15 | 9818 | NS5 | S | 0.0229 |
| iSNV | F27-15 | F27 | P15 | 9818 | NS5 | S | 0.0274 |
| iSNV | F45-15 | F45 | P15 | 9818 | NS5 | S | 0.0579 |
| iSNV | F21-15 | F21 | P15 | 10259 | NS5 | S | 0.1153 |
| iSNV | F24-15 | F24 | P15 | 10259 | NS5 | S | 0.086 |
| iSNV | F27-15 | F27 | P15 | 10259 | NS5 | S | 0.0826 |
| iSNV | F30-15 | F30 | P15 | 10259 | NS5 | S | 0.0852 |
| iSNV | F33-15 | F33 | P15 | 10259 | NS5 | S | 0.1894 |

Table S5: Discontinuous iSNV sites in the same transmission chain of BHK cells (Continued)

| Type | Sample | Generation | Transmission chain | Position | Gene | Mutation | MuAF |
|---|---|---|---|---|---|---|---|
| iSNV | F36-15 | F36 | P15 | 10259 | NS5 | S | 0.4086 |
| iSNV | F45-15 | F45 | P15 | 10259 | NS5 | S | 0.1236 |
| iSNV | F50-15 | F50 | P15 | 10259 | NS5 | S | 0.2125 |
| iSNV | F55-15 | F55 | P15 | 10259 | NS5 | S | 0.2913 |
| iSNV | F30-15 | F30 | P15 | 10428 | 3'-UTR | NC | 0.024 |
| iSNV | F33-15 | F33 | P15 | 10428 | 3'-UTR | NC | 0.0609 |
| iSNV | F36-15 | F36 | P15 | 10428 | 3'-UTR | NC | 0.1645 |
| iSNV | F45-15 | F45 | P15 | 10428 | 3'-UTR | NC | 0.4105 |
| iSNV | F50-15 | F50 | P15 | 10428 | 3'-UTR | NC | 0.325 |
| iSNV | F55-15 | F55 | P15 | 10428 | 3'-UTR | NC | 0.3412 |
| iSNV | F21-15 | F21 | P15 | 10447 | 3'-UTR | NC | 0.0645 |
| iSNV | F33-15 | F33 | P15 | 10447 | 3'-UTR | NC | 0.026 |
| iSNV | F36-15 | F36 | P15 | 10447 | 3'-UTR | NC | 0.0415 |
| iSNV | F21-15 | F21 | P15 | 10566 | 3'-UTR | NC | 0.0617 |
| iSNV | F24-15 | F24 | P15 | 10566 | 3'-UTR | NC | 0.072 |
| iSNV | F27-15 | F27 | P15 | 10566 | 3'-UTR | NC | 0.0712 |
| iSNV | F30-15 | F30 | P15 | 10566 | 3'-UTR | NC | 0.0862 |
| iSNV | F33-15 | F33 | P15 | 10566 | 3'-UTR | NC | 0.1775 |
| iSNV | F36-15 | F36 | P15 | 10566 | 3'-UTR | NC | 0.3657 |
| iSNV | F45-15 | F45 | P15 | 10566 | 3'-UTR | NC | 0.1111 |
| iSNV | F50-15 | F50 | P15 | 10566 | 3'-UTR | NC | 0.1989 |
| iSNV | F55-15 | F55 | P15 | 10566 | 3'-UTR | NC | 0.2665 |
| iSNV | F21-16 | F21 | P16 | 803 | M | N | 0.0775 |
| iSNV | F27-16 | F27 | P16 | 803 | M | N | 0.0224 |
| iSNV | F36-16 | F36 | P16 | 803 | M | N | 0.0377 |
| iSNV | F45-16 | F45 | P16 | 803 | M | N | 0.1713 |
| iSNV | F50-16 | F50 | P16 | 803 | M | N | 0.4172 |
| iSNV | F55-16 | F55 | P16 | 803 | M | N | 0.3479 |
| iSNV | F36-16 | F36 | P16 | 1428 | E | N | 0.0447 |
| iSNV | F45-16 | F45 | P16 | 1428 | E | N | 0.15 |
| iSNV | F50-16 | F50 | P16 | 1428 | E | N | 0.255 |
| iSNV | F55-16 | F55 | P16 | 1428 | E | N | 0.4346 |
| iSNV | F36-16 | F36 | P16 | 1447 | E | N | 0.0255 |
| iSNV | F45-16 | F45 | P16 | 1447 | E | N | 0.0525 |
| iSNV | F50-16 | F50 | P16 | 1447 | E | N | 0.0362 |
| iSNV | F55-16 | F55 | P16 | 1447 | E | N | 0.0219 |
| iSNV | F36-16 | F36 | P16 | 1672 | E | N | 0.0571 |
| iSNV | F45-16 | F45 | P16 | 1672 | E | N | 0.1134 |
| iSNV | F50-16 | F50 | P16 | 1672 | E | N | 0.0617 |
| iSNV | F36-16 | F36 | P16 | 5753 | NS3 | S | 0.0377 |
| iSNV | F45-16 | F45 | P16 | 5753 | NS3 | S | 0.0547 |
| iSNV | F50-16 | F50 | P16 | 5753 | NS3 | S | 0.0336 |
| iSNV | F36-16 | F36 | P16 | 6122 | NS3 | S | 0.0323 |
| iSNV | F45-16 | F45 | P16 | 6122 | NS3 | S | 0.164 |
| iSNV | F50-16 | F50 | P16 | 6122 | NS3 | S | 0.3712 |
| iSNV | F55-16 | F55 | P16 | 6122 | NS3 | S | 0.2992 |
| iSNV | F27-16 | F27 | P16 | 7528 | NS4B | N | 0.0201 |
| iSNV | F30-16 | F30 | P16 | 7528 | NS4B | N | 0.0547 |
| iSNV | F33-16 | F33 | P16 | 7528 | NS4B | N | 0.1062 |
| iSNV | F36-16 | F36 | P16 | 7528 | NS4B | N | 0.0732 |
| iSNV | F45-16 | F45 | P16 | 7528 | NS4B | N | 0.0354 |
| iSNV | F50-16 | F50 | P16 | 7528 | NS4B | N | 0.0926 |
| iSNV | F55-16 | F55 | P16 | 7528 | NS4B | N | 0.1006 |

Table S5: Discontinuous iSNV sites in the same transmission chain of BHK cells (Continued)

| Type | Sample | Generation | Transmission chain | Position | Gene | Mutation | MuAF |
|---|---|---|---|---|---|---|---|
| iSNV | F21-16 | F21 | P16 | 7735 | NS5 | N | 0.0536 |
| iSNV | F24-16 | F24 | P16 | 7735 | NS5 | N | 0.042 |
| iSNV | F36-16 | F36 | P16 | 7735 | NS5 | N | 0.0402 |
| iSNV | F40-16 | F40 | P16 | 7735 | NS5 | N | 0.0436 |
| iSNV | F45-16 | F45 | P16 | 7735 | NS5 | N | 0.1623 |
| iSNV | F50-16 | F50 | P16 | 7735 | NS5 | N | 0.3734 |
| iSNV | F55-16 | F55 | P16 | 7735 | NS5 | N | 0.299 |
| iSNV | F21-16 | F21 | P16 | 8518 | NS5 | N | 0.0647 |
| iSNV | F40-16 | F40 | P16 | 8518 | NS5 | N | 0.0427 |
| iSNV | F45-16 | F45 | P16 | 8518 | NS5 | N | 0.1641 |
| iSNV | F50-16 | F50 | P16 | 8518 | NS5 | N | 0.3659 |
| iSNV | F55-16 | F55 | P16 | 8518 | NS5 | N | 0.298 |
| iSNV | F36-16 | F36 | P16 | 8693 | NS5 | S | 0.0272 |
| iSNV | F45-16 | F45 | P16 | 8693 | NS5 | S | 0.0302 |
| iSNV | F27-16 | F27 | P16 | 9293 | NS5 | S | 0.03 |
| iSNV | F30-16 | F30 | P16 | 9293 | NS5 | S | 0.0321 |
| iSNV | F33-16 | F33 | P16 | 9293 | NS5 | S | 0.0465 |
| iSNV | F40-16 | F40 | P16 | 9293 | NS5 | S | 0.0476 |
| iSNV | F45-16 | F45 | P16 | 9293 | NS5 | S | 0.1228 |
| iSNV | F50-16 | F50 | P16 | 9293 | NS5 | S | 0.0872 |
| iSNV | F55-16 | F55 | P16 | 9293 | NS5 | S | 0.1029 |
| iSNV | F36-16 | F36 | P16 | 10351 | NS5 | N | 0.0255 |
| iSNV | F45-16 | F45 | P16 | 10351 | NS5 | N | 0.032 |
| iSNV | F50-16 | F50 | P16 | 10351 | NS5 | N | 0.0243 |
| iSNV | F21-16 | F21 | P16 | 10447 | 3'-UTR | NC | 0.1013 |
| iSNV | F24-16 | F24 | P16 | 10447 | 3'-UTR | NC | 0.0634 |
| iSNV | F27-16 | F27 | P16 | 10447 | 3'-UTR | NC | 0.0266 |
| iSNV | F45-16 | F45 | P16 | 10447 | 3'-UTR | NC | 0.022 |
| iSNV | F21-16 | F21 | P16 | 10566 | 3'-UTR | NC | 0.1077 |
| iSNV | F24-16 | F24 | P16 | 10566 | 3'-UTR | NC | 0.1025 |
| iSNV | F27-16 | F27 | P16 | 10566 | 3'-UTR | NC | 0.1443 |
| iSNV | F30-16 | F30 | P16 | 10566 | 3'-UTR | NC | 0.2315 |
| iSNV | F33-16 | F33 | P16 | 10566 | 3'-UTR | NC | 0.2671 |
| iSNV | F36-16 | F36 | P16 | 10566 | 3'-UTR | NC | 0.2548 |
| iSNV | F45-16 | F45 | P16 | 10566 | 3'-UTR | NC | 0.1375 |
| iSNV | F50-16 | F50 | P16 | 10566 | 3'-UTR | NC | 0.1607 |
| iSNV | F55-16 | F55 | P16 | 10566 | 3'-UTR | NC | 0.1392 |
| iSNV | F21-17 | F21 | P17 | 645 | M | N | 0.0756 |
| iSNV | F36-17 | F36 | P17 | 645 | M | N | 0.0556 |
| iSNV | F40-17 | F40 | P17 | 645 | M | N | 0.3652 |
| iSNV | F45-17 | F45 | P17 | 645 | M | N | 0.1871 |
| iSNV | F50-17 | F50 | P17 | 645 | M | N | 0.1184 |
| iSNV | F55-17 | F55 | P17 | 645 | M | N | 0.3831 |
| iSNV | F24-17 | F24 | P17 | 2376 | E | S | 0.0482 |
| iSNV | F27-17 | F27 | P17 | 2376 | E | S | 0.0501 |
| iSNV | F30-17 | F30 | P17 | 2376 | E | S | 0.0414 |
| iSNV | F33-17 | F33 | P17 | 2376 | E | S | 0.0415 |
| iSNV | F40-17 | F40 | P17 | 2376 | E | S | 0.4337 |
| iSNV | F45-17 | F45 | P17 | 2376 | E | S | 0.1822 |
| iSNV | F50-17 | F50 | P17 | 2376 | E | S | 0.1208 |
| iSNV | F55-17 | F55 | P17 | 2376 | E | S | 0.3769 |
| iSNV | F24-17 | F24 | P17 | 6938 | NS4A | S | 0.025 |
| iSNV | F27-17 | F27 | P17 | 6938 | NS4A | S | 0.0252 |

Table S5: Discontinuous iSNV sites in the same transmission chain of BHK cells (Continued)

| Type | Sample | Generation | Transmission chain | Position | Gene | Mutation | MuAF |
|---|---|---|---|---|---|---|---|
| iSNV | F33-17 | F33 | P17 | 6938 | NS4A | S | 0.021 |
| iSNV | F24-17 | F24 | P17 | 7151 | NS4A | S | 0.0442 |
| iSNV | F27-17 | F27 | P17 | 7151 | NS4A | S | 0.04 |
| iSNV | F30-17 | F30 | P17 | 7151 | NS4A | S | 0.0431 |
| iSNV | F33-17 | F33 | P17 | 7151 | NS4A | S | 0.037 |
| iSNV | F40-17 | F40 | P17 | 7151 | NS4A | S | 0.417 |
| iSNV | F45-17 | F45 | P17 | 7151 | NS4A | S | 0.2047 |
| iSNV | F50-17 | F50 | P17 | 7151 | NS4A | S | 0.2261 |
| iSNV | F55-17 | F55 | P17 | 7151 | NS4A | S | 0.4415 |
| iSNV | F24-17 | F24 | P17 | 10447 | 3'-UTR | NC | 0.0333 |
| iSNV | F45-17 | F45 | P17 | 10447 | 3'-UTR | NC | 0.1186 |
| iSNV | F50-17 | F50 | P17 | 10447 | 3'-UTR | NC | 0.4508 |
| iSNV | F55-17 | F55 | P17 | 10447 | 3'-UTR | NC | 0.2721 |
| iSNV | F21-18 | F21 | P18 | 10447 | 3'-UTR | NC | 0.07 |
| iSNV | F24-18 | F24 | P18 | 10447 | 3'-UTR | NC | 0.0393 |
| iSNV | F27-18 | F27 | P18 | 10447 | 3'-UTR | NC | 0.0225 |
| iSNV | F30-18 | F30 | P18 | 10447 | 3'-UTR | NC | 0.022 |
| iSNV | F36-18 | F36 | P18 | 10447 | 3'-UTR | NC | 0.0271 |
| iSNV | F21-19 | F21 | P19 | 353 | C | S | 0.0947 |
| iSNV | F24-19 | F24 | P19 | 353 | C | S | 0.0765 |
| iSNV | F33-19 | F33 | P19 | 353 | C | S | 0.0334 |
| iSNV | F36-19 | F36 | P19 | 353 | C | S | 0.0377 |
| iSNV | F21-19 | F21 | P19 | 645 | M | N | 0.1127 |
| iSNV | F24-19 | F24 | P19 | 645 | M | N | 0.0679 |
| iSNV | F27-19 | F27 | P19 | 645 | M | N | 0.0224 |
| iSNV | F33-19 | F33 | P19 | 645 | M | N | 0.0332 |
| iSNV | F36-19 | F36 | P19 | 645 | M | N | 0.0448 |
| iSNV | F24-19 | F24 | P19 | 1459 | E | N | 0.1613 |
| iSNV | F27-19 | F27 | P19 | 1459 | E | N | 0.09 |
| iSNV | F30-19 | F30 | P19 | 1459 | E | N | 0.0765 |
| iSNV | F33-19 | F33 | P19 | 1459 | E | N | 0.0406 |
| iSNV | F45-19 | F45 | P19 | 1459 | E | N | 0.022 |
| iSNV | F24-19 | F24 | P19 | 3278 | NS1 | S | 0.0404 |
| iSNV | F33-19 | F33 | P19 | 3278 | NS1 | S | 0.0324 |
| iSNV | F36-19 | F36 | P19 | 3278 | NS1 | S | 0.0302 |
| iSNV | F24-19 | F24 | P19 | 3646 | NS1 | N | 0.1777 |
| iSNV | F27-19 | F27 | P19 | 3646 | NS1 | N | 0.0877 |
| iSNV | F30-19 | F30 | P19 | 3646 | NS1 | N | 0.0624 |
| iSNV | F33-19 | F33 | P19 | 3646 | NS1 | N | 0.0401 |
| iSNV | F45-19 | F45 | P19 | 3646 | NS1 | N | 0.0233 |
| iSNV | F36-19 | F36 | P19 | 4122 | NS2A | N | 0.0562 |
| iSNV | F45-19 | F45 | P19 | 4122 | NS2A | N | 0.029 |
| iSNV | F50-19 | F50 | P19 | 4122 | NS2A | N | 0.0585 |
| iSNV | F55-19 | F55 | P19 | 4122 | NS2A | N | 0.0824 |
| iSNV | F21-19 | F21 | P19 | 4697 | NS3 | S | 0.0985 |
| iSNV | F24-19 | F24 | P19 | 4697 | NS3 | S | 0.0586 |
| iSNV | F27-19 | F27 | P19 | 4697 | NS3 | S | 0.0634 |
| iSNV | F30-19 | F30 | P19 | 4697 | NS3 | S | 0.0497 |
| iSNV | F33-19 | F33 | P19 | 4697 | NS3 | S | 0.0694 |
| iSNV | F36-19 | F36 | P19 | 4697 | NS3 | S | 0.0883 |
| iSNV | F45-19 | F45 | P19 | 4697 | NS3 | S | 0.4384 |
| iSNV | F50-19 | F50 | P19 | 4697 | NS3 | S | 0.6153 |
| iSNV | F55-19 | F55 | P19 | 4697 | NS3 | S | 0.7077 |

Table S5: Discontinuous iSNV sites in the same transmission chain of BHK cells (Continued)

| Type | Sample | Generation | Transmission chain | Position | Gene | Mutation | MuAF |
|---|---|---|---|---|---|---|---|
| iSNV | F24-19 | F24 | P19 | 6203 | NS3 | S | 0.0388 |
| iSNV | F33-19 | F33 | P19 | 6203 | NS3 | S | 0.0344 |
| iSNV | F36-19 | F36 | P19 | 6203 | NS3 | S | 0.0311 |
| iSNV | F36-19 | F36 | P19 | 7633 | NS4B | N | 0.504 |
| iSNV | F45-19 | F45 | P19 | 7633 | NS4B | N | 0.5315 |
| iSNV | F50-19 | F50 | P19 | 7633 | NS4B | N | 0.3611 |
| iSNV | F55-19 | F55 | P19 | 7633 | NS4B | N | 0.2767 |
| iSNV | F24-19 | F24 | P19 | 9818 | NS5 | S | 0.0444 |
| iSNV | F27-19 | F27 | P19 | 9818 | NS5 | S | 0.0847 |
| iSNV | F30-19 | F30 | P19 | 9818 | NS5 | S | 0.0541 |
| iSNV | F33-19 | F33 | P19 | 9818 | NS5 | S | 0.0622 |
| iSNV | F36-19 | F36 | P19 | 9818 | NS5 | S | 0.0837 |
| iSNV | F45-19 | F45 | P19 | 9818 | NS5 | S | 0.0302 |
| iSNV | F50-19 | F50 | P19 | 9818 | NS5 | S | 0.0582 |
| iSNV | F55-19 | F55 | P19 | 9818 | NS5 | S | 0.0684 |
| iSNV | F36-19 | F36 | P19 | 10419 | 3'-UTR | NC | 0.0231 |
| iSNV | F50-19 | F50 | P19 | 10419 | 3'-UTR | NC | 0.0215 |
| iSNV | F21-19 | F21 | P19 | 10428 | 3'-UTR | NC | 0.0344 |
| iSNV | F27-19 | F27 | P19 | 10428 | 3'-UTR | NC | 0.0248 |
| iSNV | F30-19 | F30 | P19 | 10428 | 3'-UTR | NC | 0.0375 |
| iSNV | F33-19 | F33 | P19 | 10428 | 3'-UTR | NC | 0.06 |
| iSNV | F36-19 | F36 | P19 | 10428 | 3'-UTR | NC | 0.2042 |
| iSNV | F40-19 | F40 | P19 | 10428 | 3'-UTR | NC | 0.5053 |
| iSNV | F45-19 | F45 | P19 | 10428 | 3'-UTR | NC | 0.7537 |
| iSNV | F50-19 | F50 | P19 | 10428 | 3'-UTR | NC | 0.8777 |
| iSNV | F55-19 | F55 | P19 | 10428 | 3'-UTR | NC | 0.9567 |
| iSNV | F40-2 | F40 | P2 | 563 | M | S | 0.1329 |
| SNP | F50-2 | F50 | P2 | 563 | M | S | 0.9913 |
| SNP | F55-2 | F55 | P2 | 563 | M | S | 0.9876 |
| iSNV | F36-2 | F36 | P2 | 762 | M | N | 0.0222 |
| iSNV | F45-2 | F45 | P2 | 762 | M | N | 0.0259 |
| iSNV | F40-2 | F40 | P2 | 1512 | E | N | 0.0229 |
| iSNV | F45-2 | F45 | P2 | 1512 | E | N | 0.3931 |
| iSNV | F55-2 | F55 | P2 | 1512 | E | N | 0.0335 |
| iSNV | F40-2 | F40 | P2 | 1772 | E | S | 0.1726 |
| SNP | F50-2 | F50 | P2 | 1772 | E | S | 0.9913 |
| SNP | F55-2 | F55 | P2 | 1772 | E | S | 0.9853 |
| iSNV | F40-2 | F40 | P2 | 1911 | E | N | 0.0884 |
| iSNV | F50-2 | F50 | P2 | 1911 | E | N | 0.0201 |
| iSNV | F40-2 | F40 | P2 | 2372 | E | S | 0.1644 |
| iSNV | F50-2 | F50 | P2 | 2372 | E | S | 0.9788 |
| SNP | F55-2 | F55 | P2 | 2372 | E | S | 0.9816 |
| iSNV | F40-2 | F40 | P2 | 2531 | NS1 | S | 0.1644 |
| SNP | F50-2 | F50 | P2 | 2531 | NS1 | S | 0.9918 |
| SNP | F55-2 | F55 | P2 | 2531 | NS1 | S | 0.9856 |
| iSNV | F40-2 | F40 | P2 | 3572 | NS1 | S | 0.1739 |
| SNP | F50-2 | F50 | P2 | 3572 | NS1 | S | 0.989 |
| SNP | F55-2 | F55 | P2 | 3572 | NS1 | S | 0.9872 |
| iSNV | F33-2 | F33 | P2 | 3669 | NS1 | N | 0.0232 |
| iSNV | F45-2 | F45 | P2 | 3669 | NS1 | N | 0.022 |
| iSNV | F24-2 | F24 | P2 | 3693 | NS1 | N | 0.02 |
| iSNV | F30-2 | F30 | P2 | 3693 | NS1 | N | 0.0288 |
| iSNV | F40-2 | F40 | P2 | 3962 | NS2A | S | 0.0981 |

Table S5: Discontinuous iSNV sites in the same transmission chain of BHK cells (Continued)

| Type | Sample | Generation | Transmission chain | Position | Gene | Mutation | MuAF |
|---|---|---|---|---|---|---|---|
| SNP | F50-2 | F50 | P2 | 3962 | NS2A | S | 0.9913 |
| SNP | F55-2 | F55 | P2 | 3962 | NS2A | S | 0.9803 |
| iSNV | F21-2 | F21 | P2 | 4712 | NS3 | S | 0.0211 |
| iSNV | F40-2 | F40 | P2 | 4712 | NS3 | S | 0.1338 |
| SNP | F50-2 | F50 | P2 | 4712 | NS3 | S | 0.9934 |
| SNP | F55-2 | F55 | P2 | 4712 | NS3 | S | 0.9827 |
| iSNV | F36-2 | F36 | P2 | 4889 | NS3 | S | 0.0241 |
| iSNV | F45-2 | F45 | P2 | 4889 | NS3 | S | 0.0508 |
| iSNV | F21-2 | F21 | P2 | 5311 | NS3 | N | 0.0489 |
| iSNV | F40-2 | F40 | P2 | 5311 | NS3 | N | 0.1258 |
| SNP | F50-2 | F50 | P2 | 5311 | NS3 | N | 0.9912 |
| SNP | F55-2 | F55 | P2 | 5311 | NS3 | N | 0.9846 |
| iSNV | F40-2 | F40 | P2 | 8282 | NS5 | S | 0.1122 |
| SNP | F50-2 | F50 | P2 | 8282 | NS5 | S | 0.9935 |
| SNP | F55-2 | F55 | P2 | 8282 | NS5 | S | 0.9845 |
| iSNV | F40-2 | F40 | P2 | 8900 | NS5 | N | 0.1011 |
| SNP | F50-2 | F50 | P2 | 8900 | NS5 | N | 0.9933 |
| SNP | F55-2 | F55 | P2 | 8900 | NS5 | N | 0.9818 |
| iSNV | F24-2 | F24 | P2 | 9350 | NS5 | S | 0.0926 |
| iSNV | F27-2 | F27 | P2 | 9350 | NS5 | S | 0.0752 |
| iSNV | F30-2 | F30 | P2 | 9350 | NS5 | S | 0.0459 |
| iSNV | F33-2 | F33 | P2 | 9350 | NS5 | S | 0.0461 |
| iSNV | F36-2 | F36 | P2 | 9350 | NS5 | S | 0.0302 |
| iSNV | F45-2 | F45 | P2 | 9350 | NS5 | S | 0.0468 |
| iSNV | F40-2 | F40 | P2 | 9446 | NS5 | S | 0.0921 |
| SNP | F50-2 | F50 | P2 | 9446 | NS5 | S | 0.9925 |
| SNP | F55-2 | F55 | P2 | 9446 | NS5 | S | 0.9856 |
| iSNV | F21-2 | F21 | P2 | 10447 | 3'-UTR | NC | 0.0725 |
| iSNV | F33-2 | F33 | P2 | 10447 | 3'-UTR | NC | 0.0237 |
| iSNV | F36-2 | F36 | P2 | 10447 | 3'-UTR | NC | 0.0269 |
| iSNV | F45-2 | F45 | P2 | 10447 | 3'-UTR | NC | 0.025 |
| iSNV | F36-20 | F36 | P20 | 1593 | E | N | 0.0324 |
| iSNV | F45-20 | F45 | P20 | 1593 | E | N | 0.0206 |
| iSNV | F24-20 | F24 | P20 | 2531 | NS1 | S | 0.0253 |
| iSNV | F40-20 | F40 | P20 | 2531 | NS1 | S | 0.2531 |
| iSNV | F45-20 | F45 | P20 | 2531 | NS1 | S | 0.2138 |
| iSNV | F50-20 | F50 | P20 | 2531 | NS1 | S | 0.1961 |
| iSNV | F55-20 | F55 | P20 | 2531 | NS1 | S | 0.2334 |
| iSNV | F36-20 | F36 | P20 | 4319 | NS2B | S | 0.0239 |
| iSNV | F40-20 | F40 | P20 | 4319 | NS2B | S | 0.1824 |
| iSNV | F45-20 | F45 | P20 | 4319 | NS2B | S | 0.0288 |
| iSNV | F55-20 | F55 | P20 | 4319 | NS2B | S | 0.023 |
| iSNV | F36-20 | F36 | P20 | 5365 | NS3 | N | 0.0249 |
| iSNV | F55-20 | F55 | P20 | 5365 | NS3 | N | 0.0262 |
| iSNV | F36-20 | F36 | P20 | 6938 | NS4A | S | 0.0341 |
| iSNV | F45-20 | F45 | P20 | 6938 | NS4A | S | 0.0264 |
| iSNV | F21-20 | F21 | P20 | 10447 | 3'-UTR | NC | 0.1153 |
| iSNV | F24-20 | F24 | P20 | 10447 | 3'-UTR | NC | 0.0549 |
| iSNV | F45-20 | F45 | P20 | 10447 | 3'-UTR | NC | 0.0351 |
| iSNV | F50-20 | F50 | P20 | 10447 | 3'-UTR | NC | 0.023 |
| iSNV | F55-20 | F55 | P20 | 10447 | 3'-UTR | NC | 0.0219 |
| iSNV | F24-21 | F24 | P21 | 897 | M | N | 0.0357 |
| iSNV | F27-21 | F27 | P21 | 897 | M | N | 0.0269 |

Table S5: Discontinuous iSNV sites in the same transmission chain of BHK cells (Continued)

| Type | Sample | Generation | Transmission chain | Position | Gene | Mutation | MuAF |
|---|---|---|---|---|---|---|---|
| iSNV | F30-21 | F30 | P21 | 897 | M | N | 0.0328 |
| iSNV | F36-21 | F36 | P21 | 897 | M | N | 0.0356 |
| iSNV | F24-21 | F24 | P21 | 1043 | E | S | 0.0206 |
| iSNV | F27-21 | F27 | P21 | 1043 | E | S | 0.0226 |
| iSNV | F33-21 | F33 | P21 | 1043 | E | S | 0.0223 |
| iSNV | F40-21 | F40 | P21 | 3776 | NS2A | S | 0.0588 |
| iSNV | F50-21 | F50 | P21 | 3776 | NS2A | S | 0.0747 |
| iSNV | F55-21 | F55 | P21 | 3776 | NS2A | S | 0.0803 |
| iSNV | F40-21 | F40 | P21 | 6969 | NS4A | N | 0.0307 |
| iSNV | F50-21 | F50 | P21 | 6969 | NS4A | N | 0.0315 |
| iSNV | F24-21 | F24 | P21 | 9065 | NS5 | S | 0.0317 |
| iSNV | F27-21 | F27 | P21 | 9065 | NS5 | S | 0.0692 |
| iSNV | F30-21 | F30 | P21 | 9065 | NS5 | S | 0.0974 |
| iSNV | F33-21 | F33 | P21 | 9065 | NS5 | S | 0.1422 |
| iSNV | F40-21 | F40 | P21 | 9065 | NS5 | S | 0.3407 |
| iSNV | F45-21 | F45 | P21 | 9065 | NS5 | S | 0.1509 |
| iSNV | F50-21 | F50 | P21 | 9065 | NS5 | S | 0.0988 |
| iSNV | F55-21 | F55 | P21 | 9065 | NS5 | S | 0.0717 |
| iSNV | F40-21 | F40 | P21 | 9542 | NS5 | S | 0.0357 |
| iSNV | F50-21 | F50 | P21 | 9542 | NS5 | S | 0.034 |
| iSNV | F55-21 | F55 | P21 | 9542 | NS5 | S | 0.0201 |
| iSNV | F21-21 | F21 | P21 | 10447 | 3'-UTR | NC | 0.0847 |
| iSNV | F24-21 | F24 | P21 | 10447 | 3'-UTR | NC | 0.0322 |
| iSNV | F33-21 | F33 | P21 | 10447 | 3'-UTR | NC | 0.0221 |
| iSNV | F36-21 | F36 | P21 | 10447 | 3'-UTR | NC | 0.0264 |
| iSNV | F40-21 | F40 | P21 | 10447 | 3'-UTR | NC | 0.059 |
| iSNV | F45-21 | F45 | P21 | 10447 | 3'-UTR | NC | 0.0451 |
| iSNV | F36-21 | F36 | P21 | 10451 | 3'-UTR | NC | 0.0548 |
| iSNV | F50-21 | F50 | P21 | 10451 | 3'-UTR | NC | 0.0207 |
| iSNV | F55-21 | F55 | P21 | 10451 | 3'-UTR | NC | 0.0223 |
| iSNV | F21-22 | F21 | P22 | 353 | C | S | 0.0473 |
| iSNV | F24-22 | F24 | P22 | 353 | C | S | 0.0216 |
| iSNV | F33-22 | F33 | P22 | 353 | C | S | 0.0429 |
| iSNV | F36-22 | F36 | P22 | 353 | C | S | 0.1024 |
| iSNV | F21-22 | F21 | P22 | 645 | M | N | 0.046 |
| iSNV | F24-22 | F24 | P22 | 645 | M | N | 0.0213 |
| iSNV | F33-22 | F33 | P22 | 645 | M | N | 0.0429 |
| iSNV | F36-22 | F36 | P22 | 645 | M | N | 0.0827 |
| iSNV | F33-22 | F33 | P22 | 1117 | E | N | 0.0778 |
| iSNV | F40-22 | F40 | P22 | 1117 | E | N | 0.0503 |
| iSNV | F24-22 | F24 | P22 | 3401 | NS1 | S | 0.0231 |
| iSNV | F33-22 | F33 | P22 | 3401 | NS1 | S | 0.0285 |
| iSNV | F36-22 | F36 | P22 | 5737 | NS3 | N | 0.0532 |
| iSNV | F55-22 | F55 | P22 | 5737 | NS3 | N | 0.1581 |
| iSNV | F24-22 | F24 | P22 | 8744 | NS5 | S | 0.021 |
| iSNV | F30-22 | F30 | P22 | 8744 | NS5 | S | 0.0249 |
| iSNV | F33-22 | F33 | P22 | 8744 | NS5 | S | 0.0283 |
| iSNV | F36-22 | F36 | P22 | 8744 | NS5 | S | 0.028 |
| iSNV | F33-22 | F33 | P22 | 9634 | NS5 | N | 0.0214 |
| iSNV | F40-22 | F40 | P22 | 9634 | NS5 | N | 0.1171 |
| iSNV | F45-22 | F45 | P22 | 9634 | NS5 | N | 0.0206 |
| iSNV | F24-22 | F24 | P22 | 10046 | NS5 | S | 0.021 |
| iSNV | F30-22 | F30 | P22 | 10046 | NS5 | S | 0.0254 |

Table S5: Discontinuous iSNV sites in the same transmission chain of BHK cells (Continued)

| Type | Sample | Generation | Transmission chain | Position | Gene | Mutation | MuAF |
|---|---|---|---|---|---|---|---|
| iSNV | F33-22 | F33 | P22 | 10046 | NS5 | S | 0.0293 |
| iSNV | F36-22 | F36 | P22 | 10046 | NS5 | S | 0.0206 |
| iSNV | F21-22 | F21 | P22 | 10566 | 3'-UTR | NC | 0.0777 |
| iSNV | F24-22 | F24 | P22 | 10566 | 3'-UTR | NC | 0.0744 |
| iSNV | F27-22 | F27 | P22 | 10566 | 3'-UTR | NC | 0.0661 |
| iSNV | F30-22 | F30 | P22 | 10566 | 3'-UTR | NC | 0.077 |
| iSNV | F33-22 | F33 | P22 | 10566 | 3'-UTR | NC | 0.0942 |
| iSNV | F36-22 | F36 | P22 | 10566 | 3'-UTR | NC | 0.0975 |
| iSNV | F45-22 | F45 | P22 | 10566 | 3'-UTR | NC | 0.9552 |
| SNP | F50-22 | F50 | P22 | 10566 | 3'-UTR | NC | 0.9897 |
| SNP | F55-22 | F55 | P22 | 10566 | 3'-UTR | NC | 0.9911 |
| iSNV | F24-23 | F24 | P23 | 645 | M | N | 0.0441 |
| iSNV | F30-23 | F30 | P23 | 645 | M | N | 0.0255 |
| iSNV | F33-23 | F33 | P23 | 645 | M | N | 0.0772 |
| iSNV | F36-23 | F36 | P23 | 645 | M | N | 0.2009 |
| iSNV | F40-23 | F40 | P23 | 645 | M | N | 0.4438 |
| iSNV | F45-23 | F45 | P23 | 645 | M | N | 0.7161 |
| iSNV | F50-23 | F50 | P23 | 645 | M | N | 0.721 |
| iSNV | F55-23 | F55 | P23 | 645 | M | N | 0.8963 |
| iSNV | F21-23 | F21 | P23 | 803 | M | N | 0.1276 |
| iSNV | F24-23 | F24 | P23 | 803 | M | N | 0.089 |
| iSNV | F27-23 | F27 | P23 | 803 | M | N | 0.0209 |
| iSNV | F33-23 | F33 | P23 | 803 | M | N | 0.027 |
| iSNV | F36-23 | F36 | P23 | 803 | M | N | 0.0947 |
| iSNV | F40-23 | F40 | P23 | 803 | M | N | 0.0612 |
| iSNV | F45-23 | F45 | P23 | 803 | M | N | 0.0365 |
| iSNV | F50-23 | F50 | P23 | 803 | M | N | 0.03 |
| iSNV | F36-23 | F36 | P23 | 2808 | NS1 | N | 0.0523 |
| iSNV | F45-23 | F45 | P23 | 2808 | NS1 | N | 0.0283 |
| iSNV | F50-23 | F50 | P23 | 2808 | NS1 | N | 0.0284 |
| iSNV | F24-23 | F24 | P23 | 7735 | NS5 | N | 0.0239 |
| iSNV | F33-23 | F33 | P23 | 7735 | NS5 | N | 0.0218 |
| iSNV | F36-23 | F36 | P23 | 7735 | NS5 | N | 0.0677 |
| iSNV | F45-23 | F45 | P23 | 7735 | NS5 | N | 0.0314 |
| iSNV | F50-23 | F50 | P23 | 7735 | NS5 | N | 0.0265 |
| iSNV | F24-23 | F24 | P23 | 8518 | NS5 | N | 0.0751 |
| iSNV | F33-23 | F33 | P23 | 8518 | NS5 | N | 0.0224 |
| iSNV | F36-23 | F36 | P23 | 8518 | NS5 | N | 0.0526 |
| iSNV | F40-23 | F40 | P23 | 8518 | NS5 | N | 0.0319 |
| iSNV | F45-23 | F45 | P23 | 8518 | NS5 | N | 0.0312 |
| iSNV | F50-23 | F50 | P23 | 8518 | NS5 | N | 0.03 |
| iSNV | F36-23 | F36 | P23 | 10451 | 3'-UTR | NC | 0.0263 |
| iSNV | F55-23 | F55 | P23 | 10451 | 3'-UTR | NC | 0.0248 |
| iSNV | F36-24 | F36 | P24 | 658 | M | N | 0.0349 |
| iSNV | F45-24 | F45 | P24 | 658 | M | N | 0.0641 |
| iSNV | F27-24 | F27 | P24 | 919 | M | N | 0.0203 |
| iSNV | F30-24 | F30 | P24 | 919 | M | N | 0.0248 |
| iSNV | F33-24 | F33 | P24 | 919 | M | N | 0.0335 |
| iSNV | F45-24 | F45 | P24 | 919 | M | N | 0.0872 |
| iSNV | F24-24 | F24 | P24 | 1013 | E | S | 0.0301 |
| iSNV | F27-24 | F27 | P24 | 1013 | E | S | 0.0434 |
| iSNV | F30-24 | F30 | P24 | 1013 | E | S | 0.0369 |
| iSNV | F33-24 | F33 | P24 | 1013 | E | S | 0.0463 |

Table S5: Discontinuous iSNV sites in the same transmission chain of BHK cells (Continued)

| Type | Sample | Generation | Transmission chain | Position | Gene | Mutation | MuAF |
|---|---|---|---|---|---|---|---|
| iSNV | F45-24 | F45 | P24 | 1013 | E | S | 0.0792 |
| iSNV | F21-24 | F21 | P24 | 1218 | E | N | 0.7268 |
| iSNV | F24-24 | F24 | P24 | 1218 | E | N | 0.7712 |
| iSNV | F27-24 | F27 | P24 | 1218 | E | N | 0.9328 |
| iSNV | F30-24 | F30 | P24 | 1218 | E | N | 0.9635 |
| iSNV | F33-24 | F33 | P24 | 1218 | E | N | 0.9196 |
| iSNV | F36-24 | F36 | P24 | 1218 | E | N | 0.92 |
| SNP | F45-24 | F45 | P24 | 1218 | E | N | 0.9972 |
| SNP | F50-24 | F50 | P24 | 1218 | E | N | 0.9975 |
| SNP | F55-24 | F55 | P24 | 1218 | E | N | 0.997 |
| iSNV | F24-24 | F24 | P24 | 1892 | E | S | 0.0267 |
| iSNV | F27-24 | F27 | P24 | 1892 | E | S | 0.0387 |
| iSNV | F30-24 | F30 | P24 | 1892 | E | S | 0.0303 |
| iSNV | F33-24 | F33 | P24 | 1892 | E | S | 0.0343 |
| iSNV | F45-24 | F45 | P24 | 1892 | E | S | 0.0753 |
| iSNV | F24-24 | F24 | P24 | 2277 | E | N | 0.0319 |
| iSNV | F33-24 | F33 | P24 | 2277 | E | N | 0.0369 |
| iSNV | F36-24 | F36 | P24 | 2277 | E | N | 0.055 |
| iSNV | F36-24 | F36 | P24 | 2369 | E | N | 0.0462 |
| iSNV | F45-24 | F45 | P24 | 2369 | E | N | 0.0721 |
| iSNV | F55-24 | F55 | P24 | 2369 | E | N | 0.1698 |
| iSNV | F24-24 | F24 | P24 | 4663 | NS3 | N | 0.0307 |
| iSNV | F27-24 | F27 | P24 | 4663 | NS3 | N | 0.0202 |
| iSNV | F33-24 | F33 | P24 | 4663 | NS3 | N | 0.0406 |
| iSNV | F36-24 | F36 | P24 | 4663 | NS3 | N | 0.0525 |
| iSNV | F36-24 | F36 | P24 | 7633 | NS4B | N | 0.3455 |
| SNP | F45-24 | F45 | P24 | 7633 | NS4B | N | 0.9874 |
| SNP | F50-24 | F50 | P24 | 7633 | NS4B | N | 0.9939 |
| SNP | F55-24 | F55 | P24 | 7633 | NS4B | N | 0.9963 |
| iSNV | F24-24 | F24 | P24 | 10419 | 3'-UTR | NC | 0.0213 |
| iSNV | F27-24 | F27 | P24 | 10419 | 3'-UTR | NC | 0.0208 |
| iSNV | F36-24 | F36 | P24 | 10419 | 3'-UTR | NC | 0.0341 |
| iSNV | F21-24 | F21 | P24 | 10428 | 3'-UTR | NC | 0.0218 |
| iSNV | F27-24 | F27 | P24 | 10428 | 3'-UTR | NC | 0.021 |
| iSNV | F30-24 | F30 | P24 | 10428 | 3'-UTR | NC | 0.0425 |
| iSNV | F33-24 | F33 | P24 | 10428 | 3'-UTR | NC | 0.0753 |
| iSNV | F36-24 | F36 | P24 | 10428 | 3'-UTR | NC | 0.2098 |
| iSNV | F40-24 | F40 | P24 | 10428 | 3'-UTR | NC | 0.5826 |
| iSNV | F45-24 | F45 | P24 | 10428 | 3'-UTR | NC | 0.7168 |
| iSNV | F50-24 | F50 | P24 | 10428 | 3'-UTR | NC | 0.881 |
| iSNV | F55-24 | F55 | P24 | 10428 | 3'-UTR | NC | 0.9636 |
| iSNV | F21-24 | F21 | P24 | 10447 | 3'-UTR | NC | 0.0852 |
| iSNV | F24-24 | F24 | P24 | 10447 | 3'-UTR | NC | 0.0809 |
| iSNV | F27-24 | F27 | P24 | 10447 | 3'-UTR | NC | 0.045 |
| iSNV | F30-24 | F30 | P24 | 10447 | 3'-UTR | NC | 0.0435 |
| iSNV | F33-24 | F33 | P24 | 10447 | 3'-UTR | NC | 0.0829 |
| iSNV | F36-24 | F36 | P24 | 10447 | 3'-UTR | NC | 0.0673 |
| iSNV | F45-24 | F45 | P24 | 10447 | 3'-UTR | NC | 0.0769 |
| iSNV | F50-24 | F50 | P24 | 10447 | 3'-UTR | NC | 0.0711 |
| iSNV | F55-24 | F55 | P24 | 10447 | 3'-UTR | NC | 0.0435 |
| iSNV | F21-25 | F21 | P25 | 563 | M | S | 0.0373 |
| iSNV | F40-25 | F40 | P25 | 563 | M | S | 0.0239 |
| iSNV | F24-25 | F24 | P25 | 1117 | E | N | 0.0422 |

Table S5: Discontinuous iSNV sites in the same transmission chain of BHK cells (Continued)

| Type | Sample | Generation | Transmission chain | Position | Gene | Mutation | MuAF |
|---|---|---|---|---|---|---|---|
| iSNV | F27-25 | F27 | P25 | 1117 | E | N | 0.2342 |
| iSNV | F30-25 | F30 | P25 | 1117 | E | N | 0.3717 |
| iSNV | F33-25 | F33 | P25 | 1117 | E | N | 0.4326 |
| iSNV | F40-25 | F40 | P25 | 1117 | E | N | 0.0764 |
| iSNV | F45-25 | F45 | P25 | 1117 | E | N | 0.1344 |
| iSNV | F50-25 | F50 | P25 | 1117 | E | N | 0.0941 |
| iSNV | F55-25 | F55 | P25 | 1117 | E | N | 0.0334 |
| iSNV | F21-25 | F21 | P25 | 1218 | E | N | 0.7098 |
| iSNV | F24-25 | F24 | P25 | 1218 | E | N | 0.9452 |
| SNP | F27-25 | F27 | P25 | 1218 | E | N | 0.9962 |
| SNP | F30-25 | F30 | P25 | 1218 | E | N | 0.9969 |
| SNP | F33-25 | F33 | P25 | 1218 | E | N | 0.9982 |
| SNP | F36-25 | F36 | P25 | 1218 | E | N | 1 |
| SNP | F45-25 | F45 | P25 | 1218 | E | N | 0.9987 |
| SNP | F50-25 | F50 | P25 | 1218 | E | N | 0.9998 |
| SNP | F55-25 | F55 | P25 | 1218 | E | N | 0.9988 |
| iSNV | F33-25 | F33 | P25 | 2369 | E | N | 0.0248 |
| iSNV | F45-25 | F45 | P25 | 2369 | E | N | 0.0328 |
| iSNV | F21-25 | F21 | P25 | 3962 | NS2A | S | 0.0353 |
| iSNV | F40-25 | F40 | P25 | 3962 | NS2A | S | 0.021 |
| iSNV | F21-25 | F21 | P25 | 4697 | NS3 | S | 0.0721 |
| iSNV | F24-25 | F24 | P25 | 4697 | NS3 | S | 0.1034 |
| iSNV | F27-25 | F27 | P25 | 4697 | NS3 | S | 0.0766 |
| iSNV | F30-25 | F30 | P25 | 4697 | NS3 | S | 0.0685 |
| iSNV | F33-25 | F33 | P25 | 4697 | NS3 | S | 0.075 |
| iSNV | F40-25 | F40 | P25 | 4697 | NS3 | S | 0.0367 |
| iSNV | F45-25 | F45 | P25 | 4697 | NS3 | S | 0.0365 |
| iSNV | F50-25 | F50 | P25 | 4974 | NS3 | N | 0.1173 |
| iSNV | F55-25 | F55 | P25 | 4974 | NS3 | N | 0.382 |
| iSNV | F33-25 | F33 | P25 | 6431 | NS3 | S | 0.031 |
| iSNV | F40-25 | F40 | P25 | 6431 | NS3 | S | 0.0378 |
| iSNV | F45-25 | F45 | P25 | 6431 | NS3 | S | 0.0314 |
| iSNV | F33-26 | F33 | P26 | 470 | C | N | 0.0222 |
| iSNV | F36-26 | F36 | P26 | 470 | C | N | 0.0234 |
| iSNV | F45-26 | F45 | P26 | 470 | C | N | 0.0556 |
| iSNV | F50-26 | F50 | P26 | 470 | C | N | 0.0494 |
| iSNV | F55-26 | F55 | P26 | 470 | C | N | 0.0498 |
| iSNV | F33-26 | F33 | P26 | 1263 | E | N | 0.0201 |
| iSNV | F36-26 | F36 | P26 | 1263 | E | N | 0.078 |
| iSNV | F45-26 | F45 | P26 | 1263 | E | N | 0.057 |
| iSNV | F50-26 | F50 | P26 | 1263 | E | N | 0.0478 |
| iSNV | F55-26 | F55 | P26 | 1263 | E | N | 0.0445 |
| iSNV | F36-26 | F36 | P26 | 1514 | E | N | 0.0206 |
| iSNV | F45-26 | F45 | P26 | 1514 | E | N | 0.0583 |
| iSNV | F50-26 | F50 | P26 | 1514 | E | N | 0.0497 |
| iSNV | F55-26 | F55 | P26 | 1514 | E | N | 0.0438 |
| iSNV | F24-26 | F24 | P26 | 6626 | NS4A | S | 0.0333 |
| iSNV | F27-26 | F27 | P26 | 6626 | NS4A | S | 0.0257 |
| iSNV | F33-26 | F33 | P26 | 6626 | NS4A | S | 0.0243 |
| iSNV | F33-26 | F33 | P26 | 6806 | NS4A | S | 0.0222 |
| iSNV | F36-26 | F36 | P26 | 6806 | NS4A | S | 0.0364 |
| iSNV | F45-26 | F45 | P26 | 6806 | NS4A | S | 0.0546 |
| iSNV | F50-26 | F50 | P26 | 6806 | NS4A | S | 0.0443 |

Table S5: Discontinuous iSNV sites in the same transmission chain of BHK cells (Continued)

| Type | Sample | Generation | Transmission chain | Position | Gene | Mutation | MuAF |
|---|---|---|---|---|---|---|---|
| iSNV | F55-26 | F55 | P26 | 6806 | NS4A | S | 0.0465 |
| iSNV | F40-26 | F40 | P26 | 6867 | NS4A | N | 0.0571 |
| iSNV | F55-26 | F55 | P26 | 6867 | NS4A | N | 0.0406 |
| iSNV | F33-26 | F33 | P26 | 7244 | NS4A | S | 0.025 |
| iSNV | F40-26 | F40 | P26 | 7244 | NS4A | S | 0.1612 |
| iSNV | F45-26 | F45 | P26 | 7244 | NS4A | S | 0.3964 |
| iSNV | F50-26 | F50 | P26 | 7244 | NS4A | S | 0.4182 |
| iSNV | F55-26 | F55 | P26 | 7244 | NS4A | S | 0.4321 |
| iSNV | F21-26 | F21 | P26 | 10447 | 3'-UTR | NC | 0.1868 |
| iSNV | F24-26 | F24 | P26 | 10447 | 3'-UTR | NC | 0.0365 |
| iSNV | F36-26 | F36 | P26 | 10447 | 3'-UTR | NC | 0.0687 |
| iSNV | F50-26 | F50 | P26 | 10447 | 3'-UTR | NC | 0.0345 |
| iSNV | F21-26 | F21 | P26 | 10566 | 3'-UTR | NC | 0.0714 |
| iSNV | F27-26 | F27 | P26 | 10566 | 3'-UTR | NC | 0.0985 |
| iSNV | F30-26 | F30 | P26 | 10566 | 3'-UTR | NC | 0.1221 |
| iSNV | F33-26 | F33 | P26 | 10566 | 3'-UTR | NC | 0.132 |
| iSNV | F36-26 | F36 | P26 | 10566 | 3'-UTR | NC | 0.0611 |
| iSNV | F21-27 | F21 | P27 | 2372 | E | S | 0.1111 |
| iSNV | F40-27 | F40 | P27 | 2372 | E | S | 0.041 |
| iSNV | F21-27 | F21 | P27 | 2531 | NS1 | S | 0.0574 |
| iSNV | F45-27 | F45 | P27 | 2531 | NS1 | S | 0.0689 |
| iSNV | F50-27 | F50 | P27 | 2531 | NS1 | S | 0.1675 |
| iSNV | F55-27 | F55 | P27 | 2531 | NS1 | S | 0.1597 |
| iSNV | F21-27 | F21 | P27 | 3572 | NS1 | S | 0.093 |
| iSNV | F40-27 | F40 | P27 | 3572 | NS1 | S | 0.0264 |
| iSNV | F21-27 | F21 | P27 | 5952 | NS3 | N | 0.0766 |
| iSNV | F24-27 | F24 | P27 | 5952 | NS3 | N | 0.0705 |
| iSNV | F27-27 | F27 | P27 | 5952 | NS3 | N | 0.055 |
| iSNV | F30-27 | F30 | P27 | 5952 | NS3 | N | 0.0518 |
| iSNV | F40-27 | F40 | P27 | 5952 | NS3 | N | 0.0357 |
| iSNV | F21-27 | F21 | P27 | 9359 | NS5 | S | 0.0349 |
| iSNV | F24-27 | F24 | P27 | 9359 | NS5 | S | 0.0704 |
| iSNV | F27-27 | F27 | P27 | 9359 | NS5 | S | 0.0571 |
| iSNV | F30-27 | F30 | P27 | 9359 | NS5 | S | 0.0528 |
| iSNV | F40-27 | F40 | P27 | 9359 | NS5 | S | 0.0304 |
| iSNV | F21-27 | F21 | P27 | 10259 | NS5 | S | 0.1354 |
| iSNV | F24-27 | F24 | P27 | 10259 | NS5 | S | 0.0863 |
| iSNV | F27-27 | F27 | P27 | 10259 | NS5 | S | 0.056 |
| iSNV | F30-27 | F30 | P27 | 10259 | NS5 | S | 0.0405 |
| iSNV | F40-27 | F40 | P27 | 10259 | NS5 | S | 0.0349 |
| iSNV | F21-27 | F21 | P27 | 10566 | 3'-UTR | NC | 0.0525 |
| iSNV | F24-27 | F24 | P27 | 10566 | 3'-UTR | NC | 0.0616 |
| iSNV | F27-27 | F27 | P27 | 10566 | 3'-UTR | NC | 0.0618 |
| iSNV | F30-27 | F30 | P27 | 10566 | 3'-UTR | NC | 0.0427 |
| iSNV | F40-27 | F40 | P27 | 10566 | 3'-UTR | NC | 0.0371 |
| iSNV | F24-28 | F24 | P28 | 1117 | E | N | 0.0332 |
| iSNV | F27-28 | F27 | P28 | 1117 | E | N | 0.031 |
| iSNV | F33-28 | F33 | P28 | 1117 | E | N | 0.0799 |
| iSNV | F40-28 | F40 | P28 | 1117 | E | N | 0.1012 |
| iSNV | F45-28 | F45 | P28 | 1117 | E | N | 0.0827 |
| iSNV | F50-28 | F50 | P28 | 1117 | E | N | 0.034 |
| iSNV | F36-28 | F36 | P28 | 3099 | NS1 | N | 0.0201 |
| iSNV | F45-28 | F45 | P28 | 3099 | NS1 | N | 0.0641 |

Table S5: Discontinuous iSNV sites in the same transmission chain of BHK cells (Continued)

| Type | Sample | Generation | Transmission chain | Position | Gene | Mutation | MuAF |
|---|---|---|---|---|---|---|---|
| iSNV | F50-28 | F50 | P28 | 3099 | NS1 | N | 0.0425 |
| iSNV | F27-28 | F27 | P28 | 5654 | NS3 | S | 0.0249 |
| iSNV | F33-28 | F33 | P28 | 5654 | NS3 | S | 0.0539 |
| iSNV | F36-28 | F36 | P28 | 5654 | NS3 | S | 0.0535 |
| iSNV | F40-28 | F40 | P28 | 5654 | NS3 | S | 0.0684 |
| iSNV | F45-28 | F45 | P28 | 5654 | NS3 | S | 0.0745 |
| iSNV | F50-28 | F50 | P28 | 5654 | NS3 | S | 0.0297 |
| iSNV | F21-28 | F21 | P28 | 5952 | NS3 | N | 0.093 |
| iSNV | F24-28 | F24 | P28 | 5952 | NS3 | N | 0.078 |
| iSNV | F27-28 | F27 | P28 | 5952 | NS3 | N | 0.06 |
| iSNV | F30-28 | F30 | P28 | 5952 | NS3 | N | 0.0504 |
| iSNV | F33-28 | F33 | P28 | 5952 | NS3 | N | 0.0618 |
| iSNV | F50-28 | F50 | P28 | 5952 | NS3 | N | 0.0729 |
| iSNV | F55-28 | F55 | P28 | 5952 | NS3 | N | 0.2411 |
| iSNV | F40-28 | F40 | P28 | 7656 | NS4B | N | 0.1083 |
| iSNV | F45-28 | F45 | P28 | 7656 | NS4B | N | 0.0222 |
| iSNV | F55-28 | F55 | P28 | 7656 | NS4B | N | 0.045 |
| iSNV | F40-28 | F40 | P28 | 7973 | NS5 | N | 0.093 |
| iSNV | F45-28 | F45 | P28 | 7973 | NS5 | N | 0.0228 |
| iSNV | F55-28 | F55 | P28 | 7973 | NS5 | N | 0.038 |
| iSNV | F24-28 | F24 | P28 | 10452 | 3'-UTR | NC | 0.0478 |
| iSNV | F27-28 | F27 | P28 | 10452 | 3'-UTR | NC | 0.0552 |
| iSNV | F30-28 | F30 | P28 | 10452 | 3'-UTR | NC | 0.0472 |
| iSNV | F33-28 | F33 | P28 | 10452 | 3'-UTR | NC | 0.065 |
| iSNV | F40-28 | F40 | P28 | 10452 | 3'-UTR | NC | 0.0867 |
| iSNV | F45-28 | F45 | P28 | 10452 | 3'-UTR | NC | 0.0235 |
| iSNV | F55-28 | F55 | P28 | 10452 | 3'-UTR | NC | 0.0364 |
| iSNV | F21-29 | F21 | P29 | 353 | C | S | 0.0666 |
| iSNV | F24-29 | F24 | P29 | 353 | C | S | 0.0401 |
| iSNV | F36-29 | F36 | P29 | 353 | C | S | 0.0239 |
| iSNV | F24-29 | F24 | P29 | 1117 | E | N | 0.0239 |
| iSNV | F36-29 | F36 | P29 | 1117 | E | N | 0.0225 |
| iSNV | F24-29 | F24 | P29 | 5150 | NS3 | S | 0.0456 |
| iSNV | F27-29 | F27 | P29 | 5150 | NS3 | S | 0.0224 |
| iSNV | F36-29 | F36 | P29 | 5150 | NS3 | S | 0.0453 |
| iSNV | F21-3 | F21 | P3 | 353 | C | S | 0.0553 |
| iSNV | F24-3 | F24 | P3 | 353 | C | S | 0.0462 |
| iSNV | F27-3 | F27 | P3 | 353 | C | S | 0.0218 |
| iSNV | F33-3 | F33 | P3 | 353 | C | S | 0.0223 |
| iSNV | F36-3 | F36 | P3 | 353 | C | S | 0.0276 |
| iSNV | F21-3 | F21 | P3 | 645 | M | N | 0.0349 |
| iSNV | F24-3 | F24 | P3 | 645 | M | N | 0.0431 |
| iSNV | F27-3 | F27 | P3 | 645 | M | N | 0.0244 |
| iSNV | F33-3 | F33 | P3 | 645 | M | N | 0.0201 |
| iSNV | F24-3 | F24 | P3 | 1117 | E | N | 0.0442 |
| iSNV | F27-3 | F27 | P3 | 1117 | E | N | 0.024 |
| iSNV | F33-3 | F33 | P3 | 1117 | E | N | 0.0247 |
| iSNV | F36-3 | F36 | P3 | 1463 | E | S | 0.0246 |
| iSNV | F45-3 | F45 | P3 | 1463 | E | S | 0.0332 |
| iSNV | F27-3 | F27 | P3 | 1508 | E | S | 0.0237 |
| iSNV | F36-3 | F36 | P3 | 1508 | E | S | 0.2334 |
| iSNV | F40-3 | F40 | P3 | 1508 | E | S | 0.1192 |
| iSNV | F45-3 | F45 | P3 | 1508 | E | S | 0.047 |

Table S5: Discontinuous iSNV sites in the same transmission chain of BHK cells (Continued)

| Type | Sample | Generation | Transmission chain | Position | Gene | Mutation | MuAF |
|---|---|---|---|---|---|---|---|
| iSNV | F36-3 | F36 | P3 | 1512 | E | N | 0.0236 |
| iSNV | F50-3 | F50 | P3 | 1512 | E | N | 0.0951 |
| iSNV | F55-3 | F55 | P3 | 1512 | E | N | 0.2416 |
| iSNV | F21-3 | F21 | P3 | 1772 | E | S | 0.0224 |
| iSNV | F40-3 | F40 | P3 | 1772 | E | S | 0.0248 |
| iSNV | F50-3 | F50 | P3 | 1772 | E | S | 0.975 |
| SNP | F55-3 | F55 | P3 | 1772 | E | S | 0.9831 |
| iSNV | F21-3 | F21 | P3 | 2372 | E | S | 0.0436 |
| iSNV | F50-3 | F50 | P3 | 2372 | E | S | 0.9758 |
| SNP | F55-3 | F55 | P3 | 2372 | E | S | 0.9836 |
| iSNV | F21-3 | F21 | P3 | 2531 | NS1 | S | 0.0209 |
| iSNV | F50-3 | F50 | P3 | 2531 | NS1 | S | 0.9713 |
| SNP | F55-3 | F55 | P3 | 2531 | NS1 | S | 0.9818 |
| iSNV | F24-3 | F24 | P3 | 2780 | NS1 | S | 0.0247 |
| iSNV | F27-3 | F27 | P3 | 2780 | NS1 | S | 0.0256 |
| iSNV | F36-3 | F36 | P3 | 2780 | NS1 | S | 0.2665 |
| iSNV | F40-3 | F40 | P3 | 2780 | NS1 | S | 0.1116 |
| iSNV | F45-3 | F45 | P3 | 2780 | NS1 | S | 0.0639 |
| iSNV | F36-3 | F36 | P3 | 3452 | NS1 | S | 0.0279 |
| iSNV | F45-3 | F45 | P3 | 3452 | NS1 | S | 0.0569 |
| iSNV | F36-3 | F36 | P3 | 6967 | NS4A | N | 0.2022 |
| iSNV | F45-3 | F45 | P3 | 6967 | NS4A | N | 0.062 |
| iSNV | F36-3 | F36 | P3 | 6971 | NS4A | N | 0.0588 |
| iSNV | F45-3 | F45 | P3 | 6971 | NS4A | N | 0.0529 |
| iSNV | F36-3 | F36 | P3 | 7595 | NS4B | N | 0.2925 |
| iSNV | F45-3 | F45 | P3 | 7595 | NS4B | N | 0.0737 |
| iSNV | F36-3 | F36 | P3 | 7633 | NS4B | N | 0.1773 |
| iSNV | F40-3 | F40 | P3 | 7633 | NS4B | N | 0.6118 |
| iSNV | F45-3 | F45 | P3 | 7633 | NS4B | N | 0.4963 |
| iSNV | F55-3 | F55 | P3 | 7633 | NS4B | N | 0.0201 |
| iSNV | F21-3 | F21 | P3 | 9446 | NS5 | S | 0.025 |
| iSNV | F50-3 | F50 | P3 | 9446 | NS5 | S | 0.9739 |
| SNP | F55-3 | F55 | P3 | 9446 | NS5 | S | 0.9831 |
| iSNV | F21-3 | F21 | P3 | 10428 | 3'-UTR | NC | 0.0296 |
| iSNV | F27-3 | F27 | P3 | 10428 | 3'-UTR | NC | 0.0204 |
| iSNV | F30-3 | F30 | P3 | 10428 | 3'-UTR | NC | 0.0332 |
| iSNV | F33-3 | F33 | P3 | 10428 | 3'-UTR | NC | 0.061 |
| iSNV | F36-3 | F36 | P3 | 10428 | 3'-UTR | NC | 0.2219 |
| iSNV | F40-3 | F40 | P3 | 10428 | 3'-UTR | NC | 0.6915 |
| iSNV | F45-3 | F45 | P3 | 10428 | 3'-UTR | NC | 0.7918 |
| iSNV | F50-3 | F50 | P3 | 10428 | 3'-UTR | NC | 0.1889 |
| iSNV | F55-3 | F55 | P3 | 10428 | 3'-UTR | NC | 0.3527 |
| iSNV | F36-30 | F36 | P30 | 10419 | 3'-UTR | NC | 0.0282 |
| iSNV | F50-30 | F50 | P30 | 10419 | 3'-UTR | NC | 0.0371 |
| iSNV | F55-30 | F55 | P30 | 10419 | 3'-UTR | NC | 0.0517 |
| iSNV | F21-30 | F21 | P30 | 10428 | 3'-UTR | NC | 0.0202 |
| iSNV | F30-30 | F30 | P30 | 10428 | 3'-UTR | NC | 0.0254 |
| iSNV | F33-30 | F33 | P30 | 10428 | 3'-UTR | NC | 0.0451 |
| iSNV | F36-30 | F36 | P30 | 10428 | 3'-UTR | NC | 0.2954 |
| iSNV | F40-30 | F40 | P30 | 10428 | 3'-UTR | NC | 0.7603 |
| iSNV | F45-30 | F45 | P30 | 10428 | 3'-UTR | NC | 0.9403 |
| iSNV | F50-30 | F50 | P30 | 10428 | 3'-UTR | NC | 0.9727 |
| SNP | F55-30 | F55 | P30 | 10428 | 3'-UTR | NC | 0.984 |

Table S5: Discontinuous iSNV sites in the same transmission chain of BHK cells (Continued)

| Type | Sample | Generation | Transmission chain | Position | Gene | Mutation | MuAF |
|---|---|---|---|---|---|---|---|
| iSNV | F21-30 | F21 | P30 | 10447 | 3'-UTR | NC | 0.1061 |
| iSNV | F24-30 | F24 | P30 | 10447 | 3'-UTR | NC | 0.0245 |
| iSNV | F36-30 | F36 | P30 | 10447 | 3'-UTR | NC | 0.0603 |
| iSNV | F24-4 | F24 | P4 | 4187 | NS2A | S | 0.021 |
| iSNV | F33-4 | F33 | P4 | 4187 | NS2A | S | 0.0213 |
| iSNV | F24-4 | F24 | P4 | 6182 | NS3 | S | 0.0371 |
| iSNV | F30-4 | F30 | P4 | 6182 | NS3 | S | 0.0278 |
| iSNV | F33-4 | F33 | P4 | 6182 | NS3 | S | 0.025 |
| iSNV | F21-4 | F21 | P4 | 10447 | 3'-UTR | NC | 0.0623 |
| iSNV | F24-4 | F24 | P4 | 10447 | 3'-UTR | NC | 0.0343 |
| iSNV | F30-4 | F30 | P4 | 10447 | 3'-UTR | NC | 0.027 |
| iSNV | F33-4 | F33 | P4 | 10447 | 3'-UTR | NC | 0.0233 |
| iSNV | F36-4 | F36 | P4 | 10447 | 3'-UTR | NC | 0.029 |
| iSNV | F45-4 | F45 | P4 | 10447 | 3'-UTR | NC | 0.02 |
| iSNV | F21-5 | F21 | P5 | 353 | C | S | 0.0705 |
| iSNV | F24-5 | F24 | P5 | 353 | C | S | 0.0306 |
| iSNV | F27-5 | F27 | P5 | 353 | C | S | 0.0243 |
| iSNV | F33-5 | F33 | P5 | 353 | C | S | 0.0365 |
| iSNV | F36-5 | F36 | P5 | 353 | C | S | 0.0378 |
| iSNV | F45-5 | F45 | P5 | 353 | C | S | 0.0436 |
| iSNV | F24-5 | F24 | P5 | 399 | C | N | 0.0327 |
| iSNV | F27-5 | F27 | P5 | 399 | C | N | 0.0684 |
| iSNV | F30-5 | F30 | P5 | 399 | C | N | 0.2131 |
| iSNV | F33-5 | F33 | P5 | 399 | C | N | 0.1562 |
| iSNV | F36-5 | F36 | P5 | 399 | C | N | 0.0889 |
| iSNV | F40-5 | F40 | P5 | 399 | C | N | 0.0758 |
| iSNV | F45-5 | F45 | P5 | 399 | C | N | 0.0789 |
| iSNV | F55-5 | F55 | P5 | 399 | C | N | 0.0319 |
| iSNV | F21-5 | F21 | P5 | 645 | M | N | 0.0578 |
| iSNV | F24-5 | F24 | P5 | 645 | M | N | 0.0349 |
| iSNV | F27-5 | F27 | P5 | 645 | M | N | 0.0257 |
| iSNV | F33-5 | F33 | P5 | 645 | M | N | 0.0346 |
| iSNV | F36-5 | F36 | P5 | 645 | M | N | 0.0403 |
| iSNV | F45-5 | F45 | P5 | 645 | M | N | 0.041 |
| iSNV | F21-5 | F21 | P5 | 998 | E | S | 0.1553 |
| iSNV | F24-5 | F24 | P5 | 998 | E | S | 0.1172 |
| iSNV | F27-5 | F27 | P5 | 998 | E | S | 0.1064 |
| iSNV | F30-5 | F30 | P5 | 998 | E | S | 0.0975 |
| iSNV | F33-5 | F33 | P5 | 998 | E | S | 0.1268 |
| iSNV | F36-5 | F36 | P5 | 998 | E | S | 0.1105 |
| iSNV | F45-5 | F45 | P5 | 998 | E | S | 0.047 |
| iSNV | F24-5 | F24 | P5 | 1117 | E | N | 0.0219 |
| iSNV | F27-5 | F27 | P5 | 1117 | E | N | 0.0233 |
| iSNV | F33-5 | F33 | P5 | 1117 | E | N | 0.0475 |
| iSNV | F36-5 | F36 | P5 | 1117 | E | N | 0.0322 |
| iSNV | F45-5 | F45 | P5 | 1117 | E | N | 0.046 |
| iSNV | F36-5 | F36 | P5 | 1428 | E | N | 0.02 |
| iSNV | F50-5 | F50 | P5 | 1428 | E | N | 0.1687 |
| iSNV | F55-5 | F55 | P5 | 1428 | E | N | 0.1219 |
| iSNV | F21-5 | F21 | P5 | 1430 | E | N | 0.042 |
| iSNV | F27-5 | F27 | P5 | 1430 | E | N | 0.0259 |
| iSNV | F21-5 | F21 | P5 | 3869 | NS2A | S | 0.8 |
| iSNV | F24-5 | F24 | P5 | 3869 | NS2A | S | 0.8174 |

Table S5: Discontinuous iSNV sites in the same transmission chain of BHK cells (Continued)

| Type | Sample | Generation | Transmission chain | Position | Gene | Mutation | MuAF |
|---|---|---|---|---|---|---|---|
| iSNV | F27-5 | F27 | P5 | 3869 | NS2A | S | 0.8947 |
| iSNV | F30-5 | F30 | P5 | 3869 | NS2A | S | 0.8987 |
| iSNV | F33-5 | F33 | P5 | 3869 | NS2A | S | 0.878 |
| iSNV | F40-5 | F40 | P5 | 3869 | NS2A | S | 0.9774 |
| iSNV | F45-5 | F45 | P5 | 3869 | NS2A | S | 0.9527 |
| iSNV | F50-5 | F50 | P5 | 3869 | NS2A | S | 0.02 |
| iSNV | F36-5 | F36 | P5 | 6900 | NS4A | N | 0.0296 |
| iSNV | F45-5 | F45 | P5 | 6900 | NS4A | N | 0.0248 |
| iSNV | F24-5 | F24 | P5 | 7182 | NS4A | S | 0.0274 |
| iSNV | F33-5 | F33 | P5 | 7182 | NS4A | S | 0.0311 |
| iSNV | F27-5 | F27 | P5 | 9341 | NS5 | S | 0.0201 |
| iSNV | F33-5 | F33 | P5 | 9341 | NS5 | S | 0.0203 |
| iSNV | F36-5 | F36 | P5 | 9341 | NS5 | S | 0.0267 |
| iSNV | F40-5 | F40 | P5 | 9341 | NS5 | S | 0.224 |
| iSNV | F45-5 | F45 | P5 | 9341 | NS5 | S | 0.4089 |
| iSNV | F21-5 | F21 | P5 | 10259 | NS5 | S | 0.119 |
| iSNV | F24-5 | F24 | P5 | 10259 | NS5 | S | 0.1227 |
| iSNV | F27-5 | F27 | P5 | 10259 | NS5 | S | 0.0948 |
| iSNV | F30-5 | F30 | P5 | 10259 | NS5 | S | 0.0842 |
| iSNV | F33-5 | F33 | P5 | 10259 | NS5 | S | 0.1176 |
| iSNV | F36-5 | F36 | P5 | 10259 | NS5 | S | 0.0852 |
| iSNV | F45-5 | F45 | P5 | 10259 | NS5 | S | 0.0446 |
| iSNV | F24-5 | F24 | P5 | 10447 | 3'-UTR | NC | 0.0936 |
| iSNV | F27-5 | F27 | P5 | 10447 | 3'-UTR | NC | 0.0461 |
| iSNV | F30-5 | F30 | P5 | 10447 | 3'-UTR | NC | 0.0343 |
| iSNV | F33-5 | F33 | P5 | 10447 | 3'-UTR | NC | 0.059 |
| iSNV | F36-5 | F36 | P5 | 10447 | 3'-UTR | NC | 0.0702 |
| iSNV | F40-5 | F40 | P5 | 10447 | 3'-UTR | NC | 0.026 |
| iSNV | F45-5 | F45 | P5 | 10447 | 3'-UTR | NC | 0.0632 |
| iSNV | F55-5 | F55 | P5 | 10447 | 3'-UTR | NC | 0.0214 |
| iSNV | F21-6 | F21 | P6 | 2531 | NS1 | S | 0.0231 |
| SNP | F50-6 | F50 | P6 | 2531 | NS1 | S | 0.9967 |
| SNP | F55-6 | F55 | P6 | 2531 | NS1 | S | 0.9961 |
| iSNV | F33-6 | F33 | P6 | 4974 | NS3 | N | 0.0439 |
| iSNV | F36-6 | F36 | P6 | 4974 | NS3 | N | 0.5944 |
| iSNV | F45-6 | F45 | P6 | 4974 | NS3 | N | 0.8895 |
| iSNV | F36-6 | F36 | P6 | 7633 | NS4B | N | 0.588 |
| iSNV | F45-6 | F45 | P6 | 7633 | NS4B | N | 0.9076 |
| iSNV | F27-6 | F27 | P6 | 9491 | NS5 | S | 0.0324 |
| iSNV | F30-6 | F30 | P6 | 9491 | NS5 | S | 0.0553 |
| iSNV | F33-6 | F33 | P6 | 9491 | NS5 | S | 0.0913 |
| iSNV | F36-6 | F36 | P6 | 9491 | NS5 | S | 0.1958 |
| iSNV | F45-6 | F45 | P6 | 9491 | NS5 | S | 0.0851 |
| iSNV | F21-7 | F21 | P7 | 998 | E | S | 0.1706 |
| iSNV | F24-7 | F24 | P7 | 998 | E | S | 0.0961 |
| iSNV | F27-7 | F27 | P7 | 998 | E | S | 0.0907 |
| iSNV | F30-7 | F30 | P7 | 998 | E | S | 0.0831 |
| iSNV | F33-7 | F33 | P7 | 998 | E | S | 0.06 |
| iSNV | F36-7 | F36 | P7 | 998 | E | S | 0.0423 |
| iSNV | F45-7 | F45 | P7 | 998 | E | S | 0.0669 |
| iSNV | F33-7 | F33 | P7 | 1428 | E | N | 0.02 |
| iSNV | F36-7 | F36 | P7 | 1428 | E | N | 0.0524 |
| iSNV | F45-7 | F45 | P7 | 1428 | E | N | 0.0287 |

Table S5: Discontinuous iSNV sites in the same transmission chain of BHK cells (Continued)

| Type | Sample | Generation | Transmission chain | Position | Gene | Mutation | MuAF |
|---|---|---|---|---|---|---|---|
| iSNV | F50-7 | F50 | P7 | 1428 | E | N | 0.0271 |
| iSNV | F21-7 | F21 | P7 | 1430 | E | N | 0.0275 |
| iSNV | F36-7 | F36 | P7 | 1430 | E | N | 0.0818 |
| iSNV | F40-7 | F40 | P7 | 1430 | E | N | 0.0432 |
| iSNV | F45-7 | F45 | P7 | 1512 | E | N | 0.3136 |
| iSNV | F55-7 | F55 | P7 | 1512 | E | N | 0.0244 |
| iSNV | F21-7 | F21 | P7 | 3572 | NS1 | S | 0.0308 |
| SNP | F50-7 | F50 | P7 | 3572 | NS1 | S | 0.9912 |
| SNP | F55-7 | F55 | P7 | 3572 | NS1 | S | 0.9949 |
| iSNV | F21-7 | F21 | P7 | 4697 | NS3 | S | 0.1024 |
| iSNV | F24-7 | F24 | P7 | 4697 | NS3 | S | 0.0961 |
| iSNV | F27-7 | F27 | P7 | 4697 | NS3 | S | 0.0676 |
| iSNV | F30-7 | F30 | P7 | 4697 | NS3 | S | 0.0599 |
| iSNV | F33-7 | F33 | P7 | 4697 | NS3 | S | 0.0643 |
| iSNV | F36-7 | F36 | P7 | 4697 | NS3 | S | 0.0406 |
| iSNV | F45-7 | F45 | P7 | 4697 | NS3 | S | 0.068 |
| iSNV | F21-7 | F21 | P7 | 5952 | NS3 | N | 0.1063 |
| iSNV | F24-7 | F24 | P7 | 5952 | NS3 | N | 0.0938 |
| iSNV | F27-7 | F27 | P7 | 5952 | NS3 | N | 0.0777 |
| iSNV | F30-7 | F30 | P7 | 5952 | NS3 | N | 0.0666 |
| iSNV | F33-7 | F33 | P7 | 5952 | NS3 | N | 0.0628 |
| iSNV | F36-7 | F36 | P7 | 5952 | NS3 | N | 0.032 |
| iSNV | F45-7 | F45 | P7 | 5952 | NS3 | N | 0.0647 |
| iSNV | F21-7 | F21 | P7 | 9359 | NS5 | S | 0.0278 |
| iSNV | F24-7 | F24 | P7 | 9359 | NS5 | S | 0.0753 |
| iSNV | F27-7 | F27 | P7 | 9359 | NS5 | S | 0.0628 |
| iSNV | F30-7 | F30 | P7 | 9359 | NS5 | S | 0.0769 |
| iSNV | F33-7 | F33 | P7 | 9359 | NS5 | S | 0.0613 |
| iSNV | F36-7 | F36 | P7 | 9359 | NS5 | S | 0.0373 |
| iSNV | F45-7 | F45 | P7 | 9359 | NS5 | S | 0.0686 |
| iSNV | F24-7 | F24 | P7 | 9370 | NS5 | N | 0.0492 |
| iSNV | F27-7 | F27 | P7 | 9370 | NS5 | N | 0.0888 |
| iSNV | F30-7 | F30 | P7 | 9370 | NS5 | N | 0.1074 |
| iSNV | F33-7 | F33 | P7 | 9370 | NS5 | N | 0.1089 |
| iSNV | F36-7 | F36 | P7 | 9370 | NS5 | N | 0.0814 |
| iSNV | F40-7 | F40 | P7 | 9370 | NS5 | N | 0.0275 |
| iSNV | F45-7 | F45 | P7 | 9370 | NS5 | N | 0.3198 |
| iSNV | F55-7 | F55 | P7 | 9370 | NS5 | N | 0.0276 |
| iSNV | F21-7 | F21 | P7 | 10259 | NS5 | S | 0.1529 |
| iSNV | F24-7 | F24 | P7 | 10259 | NS5 | S | 0.102 |
| iSNV | F27-7 | F27 | P7 | 10259 | NS5 | S | 0.0818 |
| iSNV | F30-7 | F30 | P7 | 10259 | NS5 | S | 0.0675 |
| iSNV | F33-7 | F33 | P7 | 10259 | NS5 | S | 0.0664 |
| iSNV | F36-7 | F36 | P7 | 10259 | NS5 | S | 0.0565 |
| iSNV | F45-7 | F45 | P7 | 10259 | NS5 | S | 0.0683 |
| iSNV | F21-7 | F21 | P7 | 10566 | 3'-UTR | NC | 0.0866 |
| iSNV | F24-7 | F24 | P7 | 10566 | 3'-UTR | NC | 0.0863 |
| iSNV | F27-7 | F27 | P7 | 10566 | 3'-UTR | NC | 0.077 |
| iSNV | F30-7 | F30 | P7 | 10566 | 3'-UTR | NC | 0.0724 |
| iSNV | F33-7 | F33 | P7 | 10566 | 3'-UTR | NC | 0.0525 |
| iSNV | F45-7 | F45 | P7 | 10566 | 3'-UTR | NC | 0.0653 |
| iSNV | F36-8 | F36 | P8 | 939 | M | N | 0.0202 |
| iSNV | F45-8 | F45 | P8 | 939 | M | N | 0.1022 |

Table S5: Discontinuous iSNV sites in the same transmission chain of BHK cells (Continued)

| Type | Sample | Generation | Transmission chain | Position | Gene | Mutation | MuAF |
|---|---|---|---|---|---|---|---|
| iSNV | F24-8 | F24 | P8 | 1453 | E | N | 0.0244 |
| iSNV | F33-8 | F33 | P8 | 1453 | E | N | 0.0371 |
| iSNV | F36-8 | F36 | P8 | 1453 | E | N | 0.0745 |
| iSNV | F40-8 | F40 | P8 | 1453 | E | N | 0.7225 |
| iSNV | F45-8 | F45 | P8 | 1453 | E | N | 0.3349 |
| iSNV | F24-8 | F24 | P8 | 3131 | NS1 | S | 0.0704 |
| iSNV | F27-8 | F27 | P8 | 3131 | NS1 | S | 0.1808 |
| iSNV | F30-8 | F30 | P8 | 3131 | NS1 | S | 0.2174 |
| iSNV | F33-8 | F33 | P8 | 3131 | NS1 | S | 0.136 |
| iSNV | F36-8 | F36 | P8 | 3131 | NS1 | S | 0.1259 |
| iSNV | F45-8 | F45 | P8 | 3131 | NS1 | S | 0.0711 |
| iSNV | F21-8 | F21 | P8 | 3962 | NS2A | S | 0.0285 |
| SNP | F50-8 | F50 | P8 | 3962 | NS2A | S | 0.9962 |
| SNP | F55-8 | F55 | P8 | 3962 | NS2A | S | 0.9975 |
| iSNV | F24-8 | F24 | P8 | 4187 | NS2A | S | 0.0206 |
| iSNV | F33-8 | F33 | P8 | 4187 | NS2A | S | 0.0274 |
| iSNV | F36-8 | F36 | P8 | 4187 | NS2A | S | 0.1256 |
| iSNV | F40-8 | F40 | P8 | 4187 | NS2A | S | 0.1428 |
| iSNV | F45-8 | F45 | P8 | 4187 | NS2A | S | 0.4041 |
| iSNV | F24-8 | F24 | P8 | 4835 | NS3 | S | 0.0286 |
| iSNV | F33-8 | F33 | P8 | 4835 | NS3 | S | 0.0305 |
| iSNV | F30-8 | F30 | P8 | 4896 | NS3 | N | 0.0341 |
| iSNV | F33-8 | F33 | P8 | 4896 | NS3 | N | 0.0386 |
| iSNV | F36-8 | F36 | P8 | 4896 | NS3 | N | 0.043 |
| iSNV | F45-8 | F45 | P8 | 4896 | NS3 | N | 0.0307 |
| iSNV | F24-8 | F24 | P8 | 5665 | NS3 | N | 0.0227 |
| iSNV | F33-8 | F33 | P8 | 5665 | NS3 | N | 0.0371 |
| iSNV | F36-8 | F36 | P8 | 5665 | NS3 | N | 0.1018 |
| iSNV | F40-8 | F40 | P8 | 5665 | NS3 | N | 0.6614 |
| iSNV | F45-8 | F45 | P8 | 5665 | NS3 | N | 0.2993 |
| iSNV | F21-8 | F21 | P8 | 7744 | NS5 | N | 0.0468 |
| iSNV | F55-8 | F55 | P8 | 7744 | NS5 | N | 0.0738 |
| iSNV | F21-8 | F21 | P8 | 8282 | NS5 | S | 0.0389 |
| SNP | F50-8 | F50 | P8 | 8282 | NS5 | S | 0.9954 |
| SNP | F55-8 | F55 | P8 | 8282 | NS5 | S | 0.9981 |
| iSNV | F24-8 | F24 | P8 | 10452 | 3'-UTR | NC | 0.0256 |
| iSNV | F27-8 | F27 | P8 | 10452 | 3'-UTR | NC | 0.0288 |
| iSNV | F33-8 | F33 | P8 | 10452 | 3'-UTR | NC | 0.0461 |
| iSNV | F36-8 | F36 | P8 | 10452 | 3'-UTR | NC | 0.0268 |
| iSNV | F36-9 | F36 | P9 | 563 | M | S | 0.1028 |
| SNP | F50-9 | F50 | P9 | 563 | M | S | 0.9897 |
| SNP | F55-9 | F55 | P9 | 563 | M | S | 0.9811 |
| iSNV | F21-9 | F21 | P9 | 1772 | E | S | 0.02 |
| iSNV | F36-9 | F36 | P9 | 1772 | E | S | 0.1363 |
| SNP | F50-9 | F50 | P9 | 1772 | E | S | 0.9905 |
| SNP | F55-9 | F55 | P9 | 1772 | E | S | 0.9836 |
| iSNV | F36-9 | F36 | P9 | 2372 | E | S | 0.2098 |
| SNP | F50-9 | F50 | P9 | 2372 | E | S | 0.9924 |
| SNP | F55-9 | F55 | P9 | 2372 | E | S | 0.9816 |
| iSNV | F36-9 | F36 | P9 | 2531 | NS1 | S | 0.073 |
| SNP | F50-9 | F50 | P9 | 2531 | NS1 | S | 0.9892 |
| iSNV | F55-9 | F55 | P9 | 2531 | NS1 | S | 0.9799 |
| iSNV | F36-9 | F36 | P9 | 3572 | NS1 | S | 0.1136 |

Table S5: Discontinuous iSNV sites in the same transmission chain of BHK cells (Continued)

| Type | Sample | Generation | Transmission chain | Position | Gene | Mutation | MuAF |
|---|---|---|---|---|---|---|---|
| SNP | F50-9 | F50 | P9 | 3572 | NS1 | S | 0.9897 |
| SNP | F55-9 | F55 | P9 | 3572 | NS1 | S | 0.9836 |
| iSNV | F36-9 | F36 | P9 | 3962 | NS2A | S | 0.0896 |
| SNP | F50-9 | F50 | P9 | 3962 | NS2A | S | 0.9891 |
| iSNV | F55-9 | F55 | P9 | 3962 | NS2A | S | 0.9781 |
| iSNV | F36-9 | F36 | P9 | 4712 | NS3 | S | 0.0915 |
| SNP | F50-9 | F50 | P9 | 4712 | NS3 | S | 0.9893 |
| SNP | F55-9 | F55 | P9 | 4712 | NS3 | S | 0.9807 |
| iSNV | F36-9 | F36 | P9 | 5311 | NS3 | N | 0.0655 |
| iSNV | F50-9 | F50 | P9 | 5311 | NS3 | N | 0.9791 |
| iSNV | F55-9 | F55 | P9 | 5311 | NS3 | N | 0.8605 |
| iSNV | F24-9 | F24 | P9 | 6938 | NS4A | S | 0.0334 |
| iSNV | F27-9 | F27 | P9 | 6938 | NS4A | S | 0.0356 |
| iSNV | F30-9 | F30 | P9 | 6938 | NS4A | S | 0.034 |
| iSNV | F33-9 | F33 | P9 | 6938 | NS4A | S | 0.0283 |
| iSNV | F40-9 | F40 | P9 | 6938 | NS4A | S | 0.1359 |
| iSNV | F36-9 | F36 | P9 | 8282 | NS5 | S | 0.0651 |
| SNP | F50-9 | F50 | P9 | 8282 | NS5 | S | 0.9976 |
| SNP | F55-9 | F55 | P9 | 8282 | NS5 | S | 0.9853 |
| iSNV | F36-9 | F36 | P9 | 8900 | NS5 | N | 0.0228 |
| SNP | F50-9 | F50 | P9 | 8900 | NS5 | N | 0.9884 |
| SNP | F55-9 | F55 | P9 | 8900 | NS5 | N | 0.9801 |
| iSNV | F36-9 | F36 | P9 | 9446 | NS5 | S | 0.061 |
| SNP | F50-9 | F50 | P9 | 9446 | NS5 | S | 0.9872 |
| iSNV | F55-9 | F55 | P9 | 9446 | NS5 | S | 0.9723 |
| iSNV | F21-9 | F21 | P9 | 10447 | 3'-UTR | NC | 0.0742 |
| iSNV | F24-9 | F24 | P9 | 10447 | 3'-UTR | NC | 0.025 |
| iSNV | F45-9 | F45 | P9 | 10447 | 3'-UTR | NC | 0.0235 |

NC, noncoding. N, non-synonymous. S, synonymous. MuAF, mutated allele frequency.

Table S6: Discontinuous iSNV sites in the same transmission chain of C6/36 cells

| Type | Sample | Generation | Transmission chain | Position | Gene | Mutation | MuAF |
|---|---|---|---|---|---|---|---|
| iSNV | F3-1 | F3 | P1 | 6533 | NS4A | S | 0.0235 |
| iSNV | F5-1 | F5 | P1 | 6533 | NS4A | S | 0.0771 |
| iSNV | F10-1 | F10 | P1 | 6533 | NS4A | S | 0.0234 |
| iSNV | F35-1 | F35 | P1 | 6533 | NS4A | S | 0.5713 |
| iSNV | F3-1 | F3 | P1 | 9688 | NS5 | N | 0.0303 |
| iSNV | F5-1 | F5 | P1 | 9688 | NS5 | N | 0.0906 |
| iSNV | F10-1 | F10 | P1 | 9688 | NS5 | N | 0.027 |
| iSNV | F20-1 | F20 | P1 | 9688 | NS5 | N | 0.0455 |
| iSNV | F25-1 | F25 | P1 | 9688 | NS5 | N | 0.1399 |
| iSNV | F30-1 | F30 | P1 | 9688 | NS5 | N | 0.1624 |
| iSNV | F35-1 | F35 | P1 | 9688 | NS5 | N | 0.597 |
| iSNV | F5-1 | F5 | P1 | 9932 | NS5 | N | 0.0206 |
| iSNV | F10-1 | F10 | P1 | 9932 | NS5 | N | 0.2247 |
| iSNV | F15-1 | F15 | P1 | 9932 | NS5 | N | 0.2946 |
| iSNV | F20-1 | F20 | P1 | 9932 | NS5 | N | 0.0306 |
| iSNV | F30-1 | F30 | P1 | 9932 | NS5 | N | 0.0527 |
| iSNV | F5-1 | F5 | P1 | 10069 | NS5 | N | 0.0219 |
| iSNV | F35-1 | F35 | P1 | 10069 | NS5 | N | 0.2048 |
| iSNV | F20-10 | F20 | P10 | 1390 | E | N | 0.0219 |
| iSNV | F25-10 | F25 | P10 | 1390 | E | N | 0.0286 |
| iSNV | F35-10 | F35 | P10 | 1390 | E | N | 0.2963 |
| iSNV | F3-10 | F3 | P10 | 6533 | NS4A | S | 0.0256 |
| iSNV | F10-10 | F10 | P10 | 6533 | NS4A | S | 0.1128 |
| iSNV | F15-10 | F15 | P10 | 6533 | NS4A | S | 0.0528 |
| iSNV | F20-10 | F20 | P10 | 6533 | NS4A | S | 0.0648 |
| iSNV | F25-10 | F25 | P10 | 6533 | NS4A | S | 0.025 |
| iSNV | F30-10 | F30 | P10 | 6533 | NS4A | S | 0.0227 |
| iSNV | F3-11 | F3 | P11 | 6533 | NS4A | S | 0.0319 |
| iSNV | F5-11 | F5 | P11 | 6533 | NS4A | S | 0.124 |
| iSNV | F10-11 | F10 | P11 | 6533 | NS4A | S | 0.1435 |
| iSNV | F15-11 | F15 | P11 | 6533 | NS4A | S | 0.0955 |
| iSNV | F20-11 | F20 | P11 | 6533 | NS4A | S | 0.0694 |
| iSNV | F25-11 | F25 | P11 | 6533 | NS4A | S | 0.0232 |
| iSNV | F35-11 | F35 | P11 | 6533 | NS4A | S | 0.5047 |
| iSNV | F3-13 | F3 | P13 | 9688 | NS5 | N | 0.021 |
| iSNV | F5-13 | F5 | P13 | 9688 | NS5 | N | 0.0782 |
| iSNV | F10-13 | F10 | P13 | 9688 | NS5 | N | 0.0587 |
| iSNV | F25-13 | F25 | P13 | 9688 | NS5 | N | 0.022 |
| iSNV | F30-13 | F30 | P13 | 9688 | NS5 | N | 0.0527 |
| iSNV | F35-13 | F35 | P13 | 9688 | NS5 | N | 0.228 |
| iSNV | F10-14 | F10 | P14 | 4394 | NS2B | S | 0.0247 |
| iSNV | F25-14 | F25 | P14 | 4394 | NS2B | S | 0.0235 |
| iSNV | F30-14 | F30 | P14 | 4394 | NS2B | S | 0.0676 |
| iSNV | F35-14 | F35 | P14 | 4394 | NS2B | S | 0.0486 |
| iSNV | F3-14 | F3 | P14 | 9688 | NS5 | N | 0.0213 |
| iSNV | F5-14 | F5 | P14 | 9688 | NS5 | N | 0.0718 |
| iSNV | F10-14 | F10 | P14 | 9688 | NS5 | N | 0.0403 |
| iSNV | F15-14 | F15 | P14 | 9688 | NS5 | N | 0.0232 |
| iSNV | F30-14 | F30 | P14 | 9688 | NS5 | N | 0.0414 |
| iSNV | F35-14 | F35 | P14 | 9688 | NS5 | N | 0.037 |
| iSNV | F5-16 | F5 | P16 | 7495 | NS4B | N | 0.0403 |
| iSNV | F10-16 | F10 | P16 | 7495 | NS4B | N | 0.097 |
| iSNV | F15-16 | F15 | P16 | 7495 | NS4B | N | 0.0595 |

Table S6: Discontinuous iSNV sites in the same transmission chain of C6/36 cells (Continued)

| Type | Sample | Generation | Transmission chain | Position | Gene | Mutation | MuAF |
|---|---|---|---|---|---|---|---|
| iSNV | F20-16 | F20 | P16 | 7495 | NS4B | N | 0.0292 |
| iSNV | F30-16 | F30 | P16 | 7495 | NS4B | N | 0.0301 |
| iSNV | F3-16 | F3 | P16 | 9688 | NS5 | N | 0.0254 |
| iSNV | F5-16 | F5 | P16 | 9688 | NS5 | N | 0.1007 |
| iSNV | F10-16 | F10 | P16 | 9688 | NS5 | N | 0.047 |
| iSNV | F15-16 | F15 | P16 | 9688 | NS5 | N | 0.0267 |
| iSNV | F30-16 | F30 | P16 | 9688 | NS5 | N | 0.0243 |
| iSNV | F35-16 | F35 | P16 | 9688 | NS5 | N | 0.0334 |
| iSNV | F10-17 | F10 | P17 | 1786 | E | N | 0.0302 |
| iSNV | F30-17 | F30 | P17 | 1786 | E | N | 0.0282 |
| iSNV | F3-17 | F3 | P17 | 6533 | NS4A | S | 0.0266 |
| iSNV | F10-17 | F10 | P17 | 6533 | NS4A | S | 0.106 |
| iSNV | F15-17 | F15 | P17 | 6533 | NS4A | S | 0.0389 |
| iSNV | F20-17 | F20 | P17 | 6533 | NS4A | S | 0.0228 |
| iSNV | F30-17 | F30 | P17 | 6533 | NS4A | S | 0.0235 |
| iSNV | F10-17 | F10 | P17 | 7543 | NS4B | N | 0.0252 |
| iSNV | F15-17 | F15 | P17 | 7543 | NS4B | N | 0.0299 |
| iSNV | F30-17 | F30 | P17 | 7543 | NS4B | N | 0.0213 |
| iSNV | F10-19 | F10 | P19 | 4394 | NS2B | S | 0.0222 |
| iSNV | F30-19 | F30 | P19 | 4394 | NS2B | S | 0.0257 |
| iSNV | F3-19 | F3 | P19 | 6533 | NS4A | S | 0.0233 |
| iSNV | F5-19 | F5 | P19 | 6533 | NS4A | S | 0.1017 |
| iSNV | F10-19 | F10 | P19 | 6533 | NS4A | S | 0.0918 |
| iSNV | F15-19 | F15 | P19 | 6533 | NS4A | S | 0.0595 |
| iSNV | F20-19 | F20 | P19 | 6533 | NS4A | S | 0.0273 |
| iSNV | F30-19 | F30 | P19 | 6533 | NS4A | S | 0.037 |
| iSNV | F15-19 | F15 | P19 | 10097 | NS5 | N | 0.022 |
| iSNV | F30-19 | F30 | P19 | 10097 | NS5 | N | 0.0389 |
| iSNV | F5-2 | F5 | P2 | 6533 | NS4A | S | 0.0217 |
| iSNV | F35-2 | F35 | P2 | 6533 | NS4A | S | 0.1613 |
| iSNV | F3-2 | F3 | P2 | 9688 | NS5 | N | 0.0222 |
| iSNV | F5-2 | F5 | P2 | 9688 | NS5 | N | 0.0278 |
| iSNV | F20-2 | F20 | P2 | 9688 | NS5 | N | 0.1251 |
| iSNV | F25-2 | F25 | P2 | 9688 | NS5 | N | 0.2038 |
| iSNV | F30-2 | F30 | P2 | 9688 | NS5 | N | 0.3659 |
| iSNV | F35-2 | F35 | P2 | 9688 | NS5 | N | 0.6758 |
| iSNV | F15-20 | F15 | P20 | 1390 | E | N | 0.0283 |
| iSNV | F35-20 | F35 | P20 | 1390 | E | N | 0.2477 |
| iSNV | F15-20 | F15 | P20 | 3579 | NS1 | N | 0.0226 |
| iSNV | F30-20 | F30 | P20 | 3579 | NS1 | N | 0.0491 |
| iSNV | F10-22 | F10 | P22 | 9932 | NS5 | N | 0.0572 |
| iSNV | F15-22 | F15 | P22 | 9932 | NS5 | N | 0.044 |
| iSNV | F30-22 | F30 | P22 | 9932 | NS5 | N | 0.0779 |
| iSNV | F35-22 | F35 | P22 | 9932 | NS5 | N | 0.1414 |
| iSNV | F15-23 | F15 | P23 | 483 | M | N | 0.044 |
| iSNV | F25-23 | F25 | P23 | 483 | M | N | 0.0485 |
| iSNV | F10-23 | F10 | P23 | 1116 | E | N | 0.021 |
| iSNV | F15-23 | F15 | P23 | 1116 | E | N | 0.0364 |
| iSNV | F25-23 | F25 | P23 | 1116 | E | N | 0.0233 |
| iSNV | F10-23 | F10 | P23 | 1786 | E | N | 0.0224 |
| iSNV | F30-23 | F30 | P23 | 1786 | E | N | 0.3229 |
| iSNV | F35-23 | F35 | P23 | 1786 | E | N | 0.0649 |
| iSNV | F10-23 | F10 | P23 | 3368 | NS1 | S | 0.024 |

Table S6: Discontinuous iSNV sites in the same transmission chain of C6/36 cells (Continued)

| Type | Sample | Generation | Transmission chain | Position | Gene | Mutation | MuAF |
|---|---|---|---|---|---|---|---|
| iSNV | F30-23 | F30 | P23 | 3368 | NS1 | S | 0.0517 |
| iSNV | F35-23 | F35 | P23 | 3368 | NS1 | S | 0.1728 |
| iSNV | F10-23 | F10 | P23 | 5311 | NS3 | N | 0.0206 |
| iSNV | F20-23 | F20 | P23 | 5311 | NS3 | N | 0.0295 |
| iSNV | F25-23 | F25 | P23 | 5311 | NS3 | N | 0.0476 |
| iSNV | F30-23 | F30 | P23 | 5311 | NS3 | N | 0.0219 |
| iSNV | F3-23 | F3 | P23 | 6533 | NS4A | S | 0.032 |
| iSNV | F10-23 | F10 | P23 | 6533 | NS4A | S | 0.0931 |
| iSNV | F15-23 | F15 | P23 | 6533 | NS4A | S | 0.0293 |
| iSNV | F30-23 | F30 | P23 | 6533 | NS4A | S | 0.2929 |
| iSNV | F35-23 | F35 | P23 | 6533 | NS4A | S | 0.1012 |
| iSNV | F3-23 | F3 | P23 | 9688 | NS5 | N | 0.0277 |
| iSNV | F5-23 | F5 | P23 | 9688 | NS5 | N | 0.1125 |
| iSNV | F10-23 | F10 | P23 | 9688 | NS5 | N | 0.0939 |
| iSNV | F15-23 | F15 | P23 | 9688 | NS5 | N | 0.0317 |
| iSNV | F25-23 | F25 | P23 | 9688 | NS5 | N | 0.0256 |
| iSNV | F30-23 | F30 | P23 | 9688 | NS5 | N | 0.3124 |
| iSNV | F35-23 | F35 | P23 | 9688 | NS5 | N | 0.1135 |
| iSNV | F10-23 | F10 | P23 | 9932 | NS5 | N | 0.0647 |
| iSNV | F15-23 | F15 | P23 | 9932 | NS5 | N | 0.0467 |
| iSNV | F30-23 | F30 | P23 | 9932 | NS5 | N | 0.0768 |
| iSNV | F35-23 | F35 | P23 | 9932 | NS5 | N | 0.0854 |
| iSNV | F20-25 | F20 | P25 | 4493 | NS2B | S | 0.0227 |
| iSNV | F25-25 | F25 | P25 | 4493 | NS2B | S | 0.0201 |
| iSNV | F35-25 | F35 | P25 | 4493 | NS2B | S | 0.0278 |
| iSNV | F15-25 | F15 | P25 | 5181 | NS3 | S | 0.0205 |
| iSNV | F30-25 | F30 | P25 | 5181 | NS3 | S | 0.0368 |
| iSNV | F3-25 | F3 | P25 | 6533 | NS4A | S | 0.0278 |
| iSNV | F10-25 | F10 | P25 | 6533 | NS4A | S | 0.0311 |
| iSNV | F25-26 | F25 | P26 | 398 | C | S | 0.0232 |
| iSNV | F35-26 | F35 | P26 | 398 | C | S | 0.0271 |
| iSNV | F10-28 | F10 | P28 | 4394 | NS2B | S | 0.0324 |
| iSNV | F15-28 | F15 | P28 | 4394 | NS2B | S | 0.0233 |
| iSNV | F30-28 | F30 | P28 | 4394 | NS2B | S | 0.064 |
| iSNV | F35-28 | F35 | P28 | 4394 | NS2B | S | 0.0307 |
| iSNV | F3-28 | F3 | P28 | 6533 | NS4A | S | 0.0214 |
| iSNV | F10-28 | F10 | P28 | 6533 | NS4A | S | 0.0562 |
| iSNV | F15-28 | F15 | P28 | 6533 | NS4A | S | 0.0416 |
| iSNV | F20-28 | F20 | P28 | 6533 | NS4A | S | 0.0544 |
| iSNV | F25-28 | F25 | P28 | 6533 | NS4A | S | 0.042 |
| iSNV | F25-30 | F25 | P30 | 1298 | E | N | 0.0215 |
| iSNV | F35-30 | F35 | P30 | 1298 | E | N | 0.0606 |
| iSNV | F15-30 | F15 | P30 | 1390 | E | N | 0.0234 |
| iSNV | F25-30 | F25 | P30 | 1390 | E | N | 0.0247 |
| iSNV | F15-30 | F15 | P30 | 1417 | E | N | 0.0254 |
| iSNV | F25-30 | F25 | P30 | 1417 | E | N | 0.0324 |
| iSNV | F30-30 | F30 | P30 | 1417 | E | N | 0.0605 |
| iSNV | F35-30 | F35 | P30 | 1417 | E | N | 0.1348 |
| iSNV | F15-30 | F15 | P30 | 4385 | NS2B | S | 0.0224 |
| iSNV | F25-30 | F25 | P30 | 4385 | NS2B | S | 0.0647 |
| iSNV | F30-30 | F30 | P30 | 4385 | NS2B | S | 0.159 |
| iSNV | F30 | F10 | P30 | 5311 | NS3 | N | 0.0234 |
| iSNV | F25-30 | F25 | P30 | 5311 | NS3 | N | 0.064 |

Table S6: Discontinuous iSNV sites in the same transmission chain of C6/36 cells (Continued)

| Type | Sample | Generation | Transmission chain | Position | Gene | Mutation | MuAF |
|---|---|---|---|---|---|---|---|
| iSNV | F30-30 | F30 | P30 | 5311 | NS3 | N | 0.1057 |
| iSNV | F35-30 | F35 | P30 | 5311 | NS3 | N | 0.3951 |
| iSNV | F15-30 | F15 | P30 | 7626 | NS4B | N | 0.0214 |
| iSNV | F35-30 | F35 | P30 | 7626 | NS4B | N | 0.0286 |
| iSNV | F30 | F10 | P30 | 7656 | NS4B | N | 0.0374 |
| iSNV | F30-30 | F30 | P30 | 7656 | NS4B | N | 0.0207 |
| iSNV | F35-30 | F35 | P30 | 7656 | NS4B | N | 0.0733 |
| iSNV | F30 | F10 | P30 | 10069 | NS5 | N | 0.0423 |
| iSNV | F15-30 | F15 | P30 | 10069 | NS5 | N | 0.0256 |
| iSNV | F30-30 | F30 | P30 | 10069 | NS5 | N | 0.0447 |
| iSNV | F35-30 | F35 | P30 | 10069 | NS5 | N | 0.0215 |
| iSNV | F15-4 | F15 | P4 | 1116 | E | N | 0.0405 |
| iSNV | F35-4 | F35 | P4 | 1116 | E | N | 0.0475 |
| iSNV | F10-4 | F10 | P4 | 3761 | NS2A | S | 0.0304 |
| iSNV | F15-4 | F15 | P4 | 3761 | NS2A | S | 0.0425 |
| iSNV | F25-4 | F25 | P4 | 3761 | NS2A | S | 0.1086 |
| iSNV | F30-4 | F30 | P4 | 3761 | NS2A | S | 0.08 |
| iSNV | F35-4 | F35 | P4 | 3761 | NS2A | S | 0.0278 |
| iSNV | F3-4 | F3 | P4 | 6533 | NS4A | S | 0.0584 |
| iSNV | F10-4 | F10 | P4 | 6533 | NS4A | S | 0.1769 |
| iSNV | F15-4 | F15 | P4 | 6533 | NS4A | S | 0.2837 |
| iSNV | F20-4 | F20 | P4 | 6533 | NS4A | S | 0.7153 |
| iSNV | F25-4 | F25 | P4 | 6533 | NS4A | S | 0.054 |
| iSNV | F30-4 | F30 | P4 | 6533 | NS4A | S | 0.0565 |
| iSNV | F35-4 | F35 | P4 | 6533 | NS4A | S | 0.034 |
| iSNV | F15-4 | F15 | P4 | 7381 | NS4B | N | 0.0438 |
| iSNV | F25-4 | F25 | P4 | 7381 | NS4B | N | 0.1054 |
| iSNV | F30-4 | F30 | P4 | 7381 | NS4B | N | 0.092 |
| iSNV | F35-4 | F35 | P4 | 7381 | NS4B | N | 0.0313 |
| iSNV | F20-4 | F20 | P4 | 10632 | 3'-UTR | NC | 0.2107 |
| iSNV | F30-4 | F30 | P4 | 10632 | 3'-UTR | NC | 0.0647 |
| iSNV | F35-4 | F35 | P4 | 10632 | 3'-UTR | NC | 0.1099 |
| iSNV | F3-5 | F3 | P5 | 6533 | NS4A | S | 0.0247 |
| iSNV | F5 | F10 | P5 | 6533 | NS4A | S | 0.0633 |
| iSNV | F5-5 | F5 | P5 | 7495 | NS4B | N | 0.0344 |
| iSNV | F5 | F10 | P5 | 7495 | NS4B | N | 0.0693 |
| iSNV | F15-5 | F15 | P5 | 7495 | NS4B | N | 0.0278 |
| iSNV | F25-5 | F25 | P5 | 7495 | NS4B | N | 0.0225 |
| iSNV | F30-5 | F30 | P5 | 7495 | NS4B | N | 0.0679 |
| iSNV | F35-5 | F35 | P5 | 7495 | NS4B | N | 0.1569 |
| iSNV | F5 | F10 | P5 | 8093 | NS5 | S | 0.0291 |
| iSNV | F15-5 | F15 | P5 | 8093 | NS5 | S | 0.0203 |
| iSNV | F25-5 | F25 | P5 | 8093 | NS5 | S | 0.0214 |
| iSNV | F30-5 | F30 | P5 | 8093 | NS5 | S | 0.0637 |
| iSNV | F35-5 | F35 | P5 | 8093 | NS5 | S | 0.17 |
| iSNV | F5-5 | F5 | P5 | 10092 | NS5 | N | 0.0256 |
| iSNV | F5 | F10 | P5 | 10092 | NS5 | N | 0.0253 |
| iSNV | F20-5 | F20 | P5 | 10092 | NS5 | N | 0.0277 |
| iSNV | F25-5 | F25 | P5 | 10092 | NS5 | N | 0.1014 |
| iSNV | F30-5 | F30 | P5 | 10092 | NS5 | N | 0.1557 |
| iSNV | F35-5 | F35 | P5 | 10092 | NS5 | N | 0.2398 |
| iSNV | F5-7 | F5 | P7 | 7657 | NS4B | N | 0.027 |
| iSNV | F10-7 | F10 | P7 | 7657 | NS4B | N | 0.0383 |

Table S6: Discontinuous iSNV sites in the same transmission chain of C6/36 cells (Continued)

| Type | Sample | Generation | Transmission chain | Position | Gene | Mutation | MuAF |
|---|---|---|---|---|---|---|---|
| iSNV | F20-7 | F20 | P7 | 7657 | NS4B | N | 0.0437 |
| iSNV | F25-7 | F25 | P7 | 7657 | NS4B | N | 0.1347 |
| iSNV | F30-7 | F30 | P7 | 7657 | NS4B | N | 0.327 |
| iSNV | F35-7 | F35 | P7 | 7657 | NS4B | N | 0.6001 |
| iSNV | F25-8 | F25 | P8 | 1116 | E | N | 0.0372 |
| iSNV | F35-8 | F35 | P8 | 1116 | E | N | 0.0925 |
| iSNV | F3-8 | F3 | P8 | 6533 | NS4A | S | 0.028 |
| iSNV | F10-8 | F10 | P8 | 6533 | NS4A | S | 0.295 |
| iSNV | F15-8 | F15 | P8 | 6533 | NS4A | S | 0.4665 |
| iSNV | F20-8 | F20 | P8 | 6533 | NS4A | S | 0.5782 |
| iSNV | F25-8 | F25 | P8 | 6533 | NS4A | S | 0.7715 |
| iSNV | F30-8 | F30 | P8 | 6533 | NS4A | S | 0.9422 |
| iSNV | F5-8 | F5 | P8 | 7495 | NS4B | N | 0.0343 |
| iSNV | F10-8 | F10 | P8 | 7495 | NS4B | N | 0.0905 |
| iSNV | F15-8 | F15 | P8 | 7495 | NS4B | N | 0.0487 |
| iSNV | F20-8 | F20 | P8 | 7495 | NS4B | N | 0.0438 |
| iSNV | F25-8 | F25 | P8 | 7495 | NS4B | N | 0.0205 |
| iSNV | F35-8 | F35 | P8 | 7495 | NS4B | N | 0.0728 |
| iSNV | F10-8 | F10 | P8 | 7656 | NS4B | N | 0.0827 |
| iSNV | F15-8 | F15 | P8 | 7656 | NS4B | N | 0.0403 |
| iSNV | F35-8 | F35 | P8 | 7656 | NS4B | N | 0.3353 |
| iSNV | F10-8 | F10 | P8 | 9932 | NS5 | N | 0.0548 |
| iSNV | F15-8 | F15 | P8 | 9932 | NS5 | N | 0.0293 |
| iSNV | F20-8 | F20 | P8 | 9932 | NS5 | N | 0.02 |
| iSNV | F35-8 | F35 | P8 | 9932 | NS5 | N | 0.0262 |
| iSNV | F5-8 | F5 | P8 | 10069 | NS5 | N | 0.0329 |
| iSNV | F10-8 | F10 | P8 | 10069 | NS5 | N | 0.0322 |
| iSNV | F35-8 | F35 | P8 | 10069 | NS5 | N | 0.1304 |

NC, noncoding. N, non-synonymous. S, synonymous. MuAF, mutated allele frequency.
